# Cervical Cancer Screening Activity at an Accredited Centre in Romania, 2018–2025: COVID-19 Disruption, Recovery and the Transition to Primary HPV Testing

**DOI:** 10.3390/healthcare14172850

**Published:** 2026-09-04

**Authors:** Laura Maghiar, Francesca Paiusan, Raul Chioibas, Andreea-Adriana Neamțu, Ciprian-Nicușor Solomon, Mariana Ganea, Diana Constanța Pelea, Paul Andrei Țenț, Lucia Georgeta Daina, Alon Vigdorovits, Ovidiu Laurean Pop, Teodor-Andrei Maghiar

**Affiliations:** 1 Department of Psycho-Neurosciences and Rehabilitation, Faculty of Medicine and Pharmacy, University of Oradea, Universității Str., No. 1, 410087 Oradea, Romania; laura.maghiar@uoradea.ro (L.M.); lucidaina@gmail.com (L.G.D.); 2 Department of Dermatovenerology, Clinical County Emergency Hospital Bihor, Gheorghe Doja Str., No. 65, 410169 Oradea, Romania; 3 Doctoral School of Biomedical Sciences, University of Oradea, Universității Str., No. 1, 410087 Oradea, Romania; francesca.paiusan@icloud.com (F.P.); alonvigdorovits@gmail.com (A.V.); drovipop@yahoo.com (O.L.P.); 4 Department of Surgery I, Faculty of Medicine, “Victor Babeș” University of Medicine and Pharmacy Timișoara, Eftimie Murgu Square, No. 2, 300041 Timișoara, Romania; 5 CBS Medcom Hospital, Popa Șapcă Str., No. 12, 300047 Timișoara, Romania; 6 Department of Toxicology, Faculty of Pharmacy, “Victor Babeș” University of Medicine and Pharmacy Timișoara, Eftimie Murgu Square, No. 2, 300041 Timișoara, Romania; 7 Research Centre for Pharmaco-Toxicological Evaluation, Faculty of Pharmacy, “Victor Babeș” University of Medicine and Pharmacy Timișoara, Eftimie Murgu Square, No. 2, 300041 Timișoara, Romania; 8 Department of Pathology, Clinical County Emergency Hospital of Arad, Andrenyi Karoly Str., No. 2–4, 310037 Arad, Romania; 9 Department of Pathology, “Pius Brînzeu” Clinical County Emergency Hospital Timișoara, Liviu Rebreanu Boulevard, No. 156, 300723 Timișoara, Romania; 10 1st Clinic of Obstetrics and Gynecology, “Pius Brînzeu” Clinical County Emergency Hospital Timișoara, Liviu Rebreanu Boulevard, No. 156, 300723 Timișoara, Romania; dr.solomonciprian@protonmail.com; 11 Faculty of Medicine and Pharmacy, University of Oradea, Universității Str., No. 1, 410087 Oradea, Romania; mganea@uoradea.ro (M.G.); teodormaghiar@yahoo.com (T.-A.M.); 12 Preclinic Department, Faculty of Medicine and Pharmacy, University of Oradea, Universității Str., No. 1, 410087 Oradea, Romania; diana_pelea@uoradea.ro; 13 Department of Oral and Maxillo-Facial Surgery, Faculty of Medicine and Pharmacy, University of Oradea, Universității Str., No. 1, 410087 Oradea, Romania; tent_andrei@yahoo.com; 14 Department of Anatomic Pathology, Clinical County Emergency Hospital Bihor, Gheorghe Doja Str., No. 65, 410169 Oradea, Romania; 15 Department of Morphological Disciplines, Faculty of Medicine and Pharmacy, University of Oradea, Universității Str., No. 1, 410087 Oradea, Romania; 16 Department of Surgery, Pelican Hospital, Corneliu Coposu Str., No. 2, 410450 Oradea, Romania; 17 Department of Surgical Disciplines, Faculty of Medicine and Pharmacy, University of Oradea, Universității Str., No. 1, 410087 Oradea, Romania

**Keywords:** cervical cancer, cervical cytology, HPV testing, cancer screening, coverage, COVID-19, Romania, health policy, secondary prevention, health services research

## Abstract

Background: Romania has one of the highest cervical cancer incidence and mortality rates in the European Union (EU) and one of the lowest levels of screening participation. We examined eight years of screening activity at an accredited centre in Bihor County, north-western Romania, encompassing the pre-pandemic period (2018–2019), the COVID-19 pandemic (2020–2021), the post-pandemic recovery (2022–2023) and the transition from primary cytology to primary human papillomavirus (HPV) DNA screening (2024–2025). Methods: This retrospective, single-centre study included 17,807 de-identified screening-episode records collected between 2018 and 2025 at Pelican Clinical Hospital, Oradea. Cytology was the primary screening test in the cytology era (2018–2023), whereas high-risk HPV (HR-HPV) DNA testing (cobas HPV Test, Roche, Pleasanton, CA, USA) with reflex cytology was used in the HPV-primary era (2024–2025). Screening activity was expressed relative to the provider’s registered administrative catchment of 65,978 women. Because quarterly counts were markedly overdispersed, changes in screening volume were analysed using negative-binomial incidence-rate ratios (IRRs) and a segmented negative-binomial interrupted time-series; cytological positivity was evaluated using age-adjusted logistic regression and the Cochran–Armitage trend test. Results: The number of screening episodes recorded over eight years corresponded to 27.0% (95% confidence interval [CI] 26.6–27.3) of the registered catchment, while annual throughput never exceeded 5.2%. Mean monthly screening volume declined from 264.5 tests in the pre-pandemic period (2018–2019) to 88.6 during the pandemic (2020–2021; IRR 0.335, 95% CI 0.177–0.634; 66.5% reduction; *p* < 0.001), corresponding to an estimated deficit of approximately 3700 tests; the interrupted time-series estimated an immediate 86% reduction at lockdown onset. Activity recovered to 61% of the pre-pandemic rate during the post-pandemic recovery (2022–2023; IRR 0.614, 95% CI 0.482–0.783) and to 90% in the HPV-primary era (2024–2025; IRR 0.898, 95% CI 0.733–1.100), coinciding with the transition to primary HPV screening. All primary HPV tests produced valid results, compared with an annual unsatisfactory-sample rate of 2.9–4.2% during primary cytology. Cytological positivity remained stable during 2018–2023, and the cytological high-grade squamous intraepithelial lesion (HSIL) detection rate per 1000 screened did not change significantly during the pandemic, although absolute HSIL detections declined with screening volume. In 2024–2025, HR-HPV positivity was 22.8% (1301/5700; 95% CI 21.8–23.9), and 47.9% of HPV-positive women had abnormal reflex cytology. Conclusions: The pandemic substantially reduced screening activity at a provider already operating with low throughput relative to its registered catchment. Activity subsequently returned to near pre-pandemic levels during the period coinciding with the introduction of primary HPV screening, which also improved primary-sample adequacy. However, the absence of persistent patient linkage and of follow-up data from colposcopy, histology and treatment precluded estimation of population-based coverage, screening intervals and detection of histologically confirmed cervical intraepithelial neoplasia grade 3 or worse (CIN3+). The findings support population-based invitation and recall, clearly defined age-specific screening and follow-up algorithms, self-sampling, targeted catch-up after pandemic-related delays and longitudinal evaluation using histologically confirmed CIN3+ as the principal clinical endpoint.

## 1. Introduction

Cervical cancer is one of the most preventable malignancies. Almost all cases are caused by persistent infection with oncogenic human papillomavirus (HPV) types, and invasive cancer usually develops slowly over many years through pre-cancerous lesions that can be detected by screening and treated before progression [1,2]. HPV vaccination can prevent the infections responsible for most cervical cancers, while screening identifies HPV infections and cervical lesions requiring surveillance or treatment [1]. Despite these opportunities for prevention, cervical cancer remains a major global health problem: it is the fifth most commonly diagnosed cancer among women worldwide, with approximately 604,000 new cases and 280,000 deaths in 2024, and it remains the leading cause of cancer death among women in 26 countries, reflecting substantial geographical inequalities in access to vaccination, screening and treatment [2,3].

In 2020, the World Health Organization (WHO) launched its global strategy to eliminate cervical cancer as a public-health problem, articulating the 90–70–90 targets (90% of girls fully vaccinated against HPV by age 15, 70% of women screened with a high-performance test by ages 35 and 45 and 90% of women with cervical pre-cancer or invasive cancer receiving appropriate treatment) and an elimination threshold of fewer than four cases per 100,000 women-years [1]. The WHO Regional Office for Europe subsequently translated this global strategy into a regional roadmap (2022–2030) that commits Member States to HPV-based screening and organised call-and-recall as the operational route to elimination [4]. Progress rests on two pillars: HPV vaccination as primary prevention, and organised screening with timely treatment of detected lesions as secondary prevention [1].

These commitments are reinforced at the level of European Union (EU) health policy. Europe’s Beating Cancer Plan (2021) established an EU-supported cancer-screening scheme with the goal that 90% of the eligible population be offered cervical screening by 2025 [5], and the Council of the EU’s 2022 Recommendation on cancer screening, which updated the 2003 Recommendation, endorsed testing for HPV as the preferred primary tool for women aged 30–65 at intervals of five years or more, tailored to vaccination history [6]. Romania has begun to align with this framework: Law 293/2022 and the National Plan for the Prevention and Control of Cancer 2023–2030 set out, for the first time, measurable national objectives for organised screening and HPV-based testing [7]. Whether these policy commitments are being realised on the ground, particularly in the country’s high-burden, historically under-served regions, is an empirical question that regional programme data are well placed to answer.

Romania carries one of the heaviest cervical cancer burdens in the EU [8,9]. GLOBOCAN 2020 estimates placed its age-standardised incidence at roughly 22.6 and mortality at 10.8 per 100,000 women [3]; the most recent (2022) estimates report an age-standardised incidence of 21.7 and a mortality of 9.3 per 100,000, roughly twice the European average for incidence (10.6) and about two-and-a-half times that for mortality (3.9), accounting for 1793 deaths nationally [8]. Cervical cancer is the second most common cancer among Romanian women aged 15–44 [10]. Romania’s organised programme, launched in 2012, offers free Papanicolaou testing to insured women aged 25–64 referred by a programme-registered general practitioner, with free treatment of detected pre-cancer [11]. Within this programme, family physicians play a gatekeeping and mobilisation role: they identify and inform eligible women, issue the screening referral and are intended to act as the main point of recall, although in practice their involvement has remained heterogeneous and largely opportunistic [11,12]. In practice, it has operated opportunistically rather than as a true population-based programme: there is no fully functioning centralised invitation-and-recall system, national participation has stagnated below 20% [9], survey data place screening within the recommended interval at roughly half the EU average [13] and HPV-vaccination coverage has been the lowest in the EU [10,12]. The first regional pilot, in north-eastern Romania, reported a participation rate of only 16.9% over its first four years [14]. The policy landscape has, however, shifted substantially in recent years. From December 2023, HPV vaccination was fully reimbursed for girls and boys aged 11–18 years and 50% compensated for women aged 19–45 years, on prescription from any physician in a contractual relationship with the National Health Insurance House (Government Decision 781/2023 and Order 3987/1156/2023) [15,16], a change accompanied by intensified public communication around HPV-related disease. From 1 October 2025, following the entry into force of Law 100/2025, full reimbursement has been extended to girls and boys aged 11–26 years, with 50% compensation maintained for women aged 27–45 years [17]. In June 2024, the Ministry of Health approved a national cervical cancer screening methodology (Order 3735/2024) that formalises HPV-based primary testing with reflex cytology triage as the national screening standard [18], and a recent policy analysis has mapped the remaining steps needed to translate Romania’s elimination commitments into practice [19]. These developments frame both the transition to HPV-primary screening observed at this centre from 2024 and the interpretation of recent participation trends.

The COVID-19 pandemic disrupted cancer screening worldwide as non-urgent services were suspended; a meta-analysis estimated that the proportion of women screened fell by roughly half during the first pandemic year [20]. Modelling studies project measurable excess cervical cancer incidence from even temporary disruption, concentrated among under-screened women [21,22,23], with parallel evidence of cancer deaths attributable to diagnostic delay [24]. Most of this evidence, however, derives from well-organised, high-coverage programmes in high-income settings; the trajectory of disruption, and crucially of recovery, in a chronically under-performing, high-burden Central and Eastern European programme is far less documented, and the concurrent transition to HPV-based primary screening in such a setting has not been described.

Using eight years of individual-level programme data from an accredited screening centre in Bihor County, we aimed to (i) describe screening coverage relative to the registered target population and the cytological and HPV yield over 2018–2025; (ii) quantify the magnitude of the COVID-19-related disruption and the extent of subsequent recovery, including under HPV-primary screening; and (iii) situate these findings within Romania’s screening system and the European and Romanian policy frameworks for cervical cancer elimination.

## 2. Materials and Methods

### 2.1. Study Design and Setting

This was a retrospective, observational analysis of individual-level, routinely collected programme records. The setting was the screening laboratory of Pelican Clinical Hospital, Oradea, an accredited provider within the national cervical cancer screening programme serving Bihor County, north-western Romania. The coverage denominator used throughout is the registered target population of 65,978 women recorded in the programme returns; this represents the women enumerated for the programme at this provider, not an independently validated census of all age-eligible (25–64) women resident in the county. For external context, the 2021 national census recorded 283,400 resident women in Bihor County overall [25], of whom those aged 25–64 form a subset; the registered figure therefore reflects a single provider’s catchment, and coverage is expressed relative to this registered denominator (see Section 4.6 Strengths and Limitations). “Registered” women are those enumerated in the provider’s official programme returns as the eligible female population (aged 25–64 years) assigned to this provider for screening purposes; this administrative figure serves solely as the denominator for throughput calculations, and registered women who did not attend do not otherwise appear in the dataset. As an accredited provider within the national programme, the centre operates a programme-contracted sample-collection and laboratory service: women attend either on referral from programme-registered family physicians and gynaecologists or on their own initiative, samples are processed in the hospital’s accredited laboratory and results are returned to the referring physician, who is responsible for communicating them and organising follow-up; the centre does not itself operate a population-based invitation or recall system.

### 2.2. Data Source and Study Population

**Figure 1:**
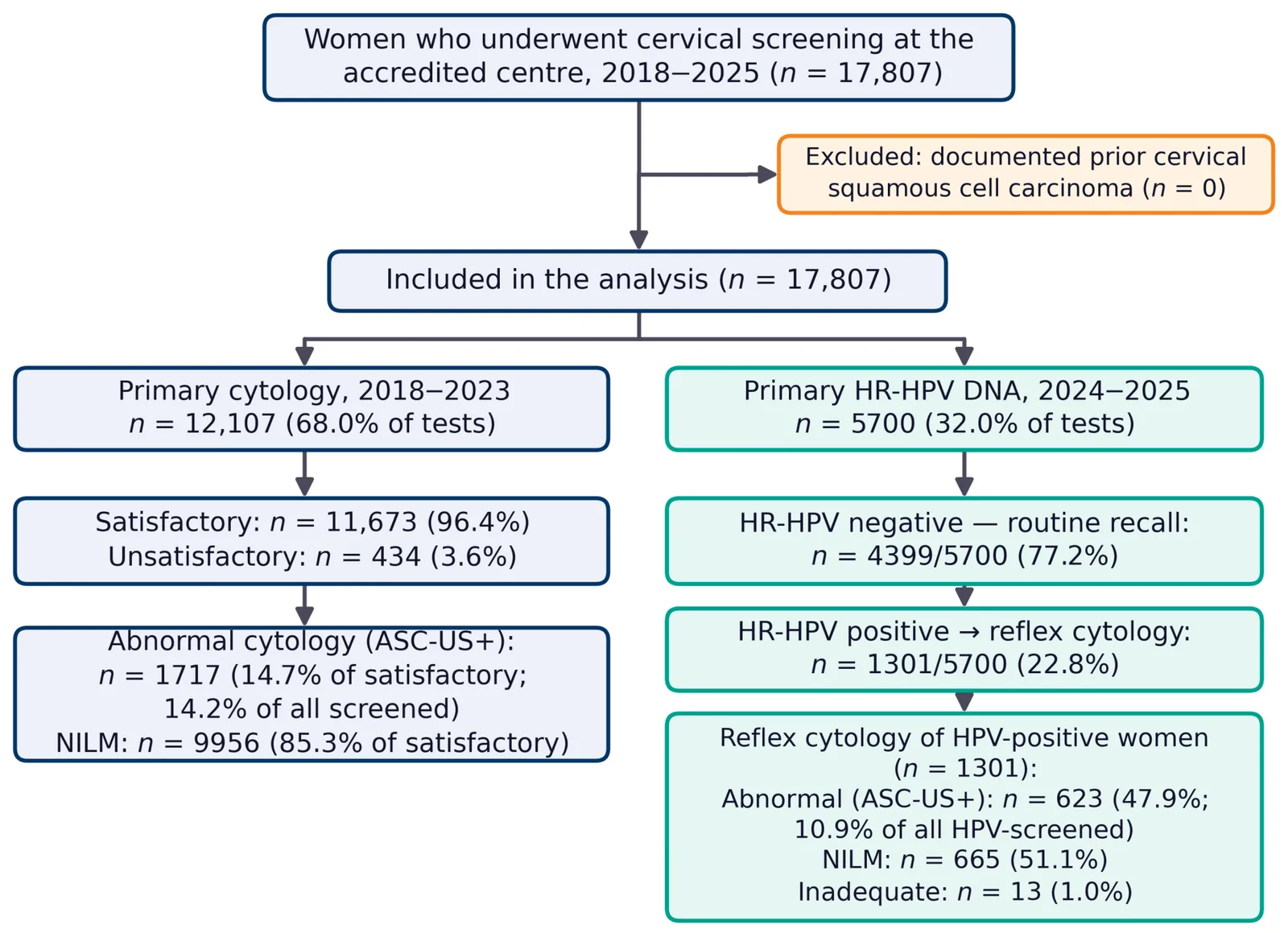
Flow of screening episodes. All screening episodes recorded at the accredited centre during 2018–2025 were eligible, the only exclusion being a documented prior diagnosis of cervical squamous cell carcinoma (*n* = 0). The analytic sample is shown by primary screening modality (cytology, 2018–2023, *n* = 12,107; HR-HPV DNA with reflex cytology, 2024–2025, *n* = 5700), with numbers and percentages for sample adequacy and results in each pathway; among HR-HPV-positive women, the distribution of abnormal, normal and inadequate reflex cytology is shown. Percentages use the denominators indicated in each box. ASC-US+, atypical squamous cells of undetermined significance or worse; NILM, negative for intraepithelial lesion or malignancy.

The analysis used a de-identified, individual-level dataset of 17,807 screening-episode records for women screened between 1 January 2018 and 31 December 2025, extracted from the centre’s laboratory information system and reconciled against the official quarterly screening-programme returns (Anexa 8 of the Romanian National Cervical Cancer Screening Programme) signed by the unit’s medical manager and the programme coordinator. Each record contained the year and calendar quarter of the test (the programme returns being quarterly, Anexa 8, calendar month was not an independently recorded field), the primary screening test performed, the woman’s five-year age band and age in years, the high-risk HPV (HR-HPV) result (where applicable), sample adequacy, the cytological result and its Bethesda category and grade and any concurrent infective finding. No directly identifying information was used. All women who underwent cervical screening at the centre during the study period were eligible for inclusion; the sole exclusion criterion was a documented previous diagnosis of cervical squamous cell carcinoma. No eligible woman was identified for exclusion on this basis, yielding a final analytic sample of 17,807 screening episodes (Figure 1).

### 2.3. Screening Modality and Programme Eras

The observation window spans four programme eras defined a priori by calendar period and screening modality: the pre-pandemic period (2018–2019; cytology), the COVID-19 pandemic (2020–2021, with 2021 data covering January–September; cytology), the post-pandemic recovery (2022–2023; cytology) and the HPV-primary era (2024–2025), in which HR-HPV DNA testing replaced cytology as the primary test, with cytology performed only as a reflex triage of HPV-positive women. Of the 17,807 tests, 12,107 (68.0%) were primary cytology and 5700 (32.0%) were primary HR-HPV DNA tests.

### 2.4. Screening Algorithms and Management Pathways

Throughout the cytology era (2018–2023), screening followed the norms of the Romanian national cervical cancer screening programme: asymptomatic women aged 25–64 years were eligible for a free Papanicolaou test upon referral by a programme-registered family physician or gynaecologist, with routine rescreening recommended after a negative (negative for intraepithelial lesion or malignancy, NILM) result at the programme-defined interval and free treatment of detected pre-cancer [11]. Abnormal cytology was managed according to the national programme norms and prevailing European guidance: women with atypical squamous cells of undetermined significance (ASC-US) or low-grade squamous intraepithelial lesion (LSIL) were recommended for repeat cytology at 6–12 months or HPV triage testing (available on an opportunistic basis), with colposcopy reserved for persistent or progressive abnormalities, whereas women with atypical squamous cells, which cannot exclude a high-grade lesion (ASC-H), high-grade squamous intraepithelial lesion (HSIL), or atypical glandular cells (AGCs), were referred directly for colposcopy and biopsy. After a normal colposcopic examination and/or negative biopsy, women were advised to resume routine screening following a period of cytological surveillance. Because diagnostic work-up and treatment took place in referral gynaecology services outside the screening laboratory, these downstream steps are not recorded in the analysed dataset (see Section 4.6 Strengths and Limitations).

From January 2024, the centre adopted HR-HPV DNA testing as the primary screening test with reflex cytology triage, and the algorithm was subsequently formalised nationally by the June 2024 screening methodology (Order 3735/2024) [18]: asymptomatic women aged 25–64 years undergo primary HPV testing; HPV-negative women are recalled at five years (three years for women under 30); HPV-positive women undergo reflex liquid-based cytology from the same specimen; those with abnormal reflex cytology (ASC-US or worse, including AGC) are referred for colposcopy and biopsy; and those with normal reflex cytology (NILM) enter 12-month HPV-based surveillance, with colposcopy indicated for persistent HPV positivity at retesting. After normal colposcopy and/or negative biopsy, women return to HPV-based surveillance before resuming the routine interval. Because only the pooled HR-HPV result was recorded in the programme returns, genotype-specific management (for example, direct colposcopy referral of HPV16/18-positive women) was not applied, and all HPV-positive women followed the same reflex-cytology triage pathway. As for the cytology era, adherence to the recommended intervals and the outcomes of colposcopy, biopsy and surveillance are not captured in the dataset.

### 2.5. Laboratory Methods

Cervical specimens were processed as liquid-based cytology. Slides were stained by the Papanicolaou (Pap) method and reported according to the 2014 Bethesda System for reporting cervical cytology. In the HPV-primary era (2024–2025), primary screening was performed with the cobas HPV Test (Roche Molecular Systems, Pleasanton, CA, USA), a real-time polymerase-chain-reaction assay that detects 14 HR-HPV types, with separate detection of HPV16 and HPV18 and a pooled result for the other 12 high-risk types (31, 33, 35, 39, 45, 51, 52, 56, 58, 59, 66 and 68), and includes a β-globin internal control for specimen adequacy; the assay is clinically validated for primary cervical cancer screening [26]. Testing was performed on the cobas 4800 system (Roche Molecular Systems) using cervical specimens collected in ThinPrep PreservCyt solution (Hologic, Marlborough, MA, USA), the liquid-based collection medium validated for use with this assay, according to the manufacturer’s instructions; results were called automatically by the system software at the assay’s validated cycle-threshold cut-offs, with no laboratory-modified thresholds, and a test was considered valid when the β-globin internal control was satisfied. Although the assay reports HPV16 and HPV18 separately, only the overall (pooled) HR-HPV result was recorded in the programme returns analysed here. Liquid-based cytology was performed only as reflex triage of HR-HPV-positive samples.

### 2.6. Variables and Definitions

For each woman, we recorded the screening test performed, its adequacy (satisfactory vs. unsatisfactory/inadequate) and the outcome. Coverage was defined as tests performed divided by the registered target population (65,978); in the absence of a persistent personal identifier, this measure represents provider-level screening throughput relative to the registered catchment rather than population-based coverage (see Section 4.6 Strengths and Limitations). For cytology, cytological positivity was defined as smears positive for any epithelial abnormality divided by satisfactory smears; positives were classified into Bethesda subcategories (ASC-US, LSIL, ASC-H, HSIL and AGC), and the high-grade (HSIL) detection rate was expressed per 1000 women screened. For the HPV-primary era, HR-HPV positivity was defined as HR-HPV-positive tests divided by valid HPV tests, and the reflex-cytology distribution among HPV-positive women was tabulated separately. Screening volume was available by calendar quarter and by eight five-year age strata (25–29 through 60–64). Data for 2021 covered the first three quarters (nine months) and are flagged; comparisons involving 2021 use either the unadjusted nine-month count or a ×12/9 annualised estimate, as stated.

### 2.7. Statistical Analysis

Proportions are presented with two-sided Wilson 95% CIs. Because the programme returns were compiled by calendar quarter, all count-based inference was conducted on the 30 quarterly test counts with recorded activity across 2018 Q1–2025 Q4; the single zero-activity quarter (2020 Q2, Romania’s hard-lockdown quarter) and the uncovered 2021 Q4 were not available as valid count observations and were therefore excluded from the fitted series (the period-contrast IRRs nevertheless incorporate the zero-activity quarter through the period totals), with the immediate effect of the lockdown instead captured by the level-change term at the April 2020 change point of the interrupted time-series. Quarterly counts were markedly overdispersed relative to the Poisson distribution (Pearson χ^2^/degrees of freedom = 69.9), so all count-based inference used negative-binomial models, whose fit was far superior to Poisson (Akaike information criterion 523 vs. 2225); the negative-binomial dispersion parameter (α) was estimated by the method of moments and used throughout, and the pandemic estimate was additionally checked with a quasi-Poisson (scaled) model. Changes in screening throughput were quantified as negative-binomial IRRs on quarterly counts: the primary comparison contrasted the pandemic period (2020 plus 2021 Q1–Q3) with the pre-pandemic period (2018–2019), and two further comparisons contrasted the post-pandemic recovery (2022–2023) and the HPV-primary era (2024–2025) with the pre-pandemic baseline. We estimated the absolute pandemic deficit as the difference between the tests expected had the pre-pandemic rate continued and those observed. The robustness of the pandemic IRR was examined in pre-specified sensitivity analyses (an alternative baseline of 2019 only; a complete-year comparison of 2019 versus 2020; and the quasi-Poisson model). To characterise the full temporal trajectory, we fitted a segmented negative-binomial interrupted time series to the quarterly counts (2018 Q1–2025 Q4), with a linear time term and level- and slope-change terms at three pre-specified change points (the April 2020 lockdown, the 2022 recovery and the 2024 transition to HPV-primary screening), and derived a counterfactual by extrapolating the pre-pandemic trend. Cytological positivity over the six cytology years (2018–2023) was modelled with logistic regression on individual results, reporting the odds ratio (OR) of a positive result per calendar year both crude and adjusted for five-year age band, complemented by the Cochran–Armitage trend test; pre-pandemic versus pandemic positivity was compared with Fisher’s exact test. The time trend in the HSIL detection rate was tested with a Poisson log-linear model with the number screened as offset. As a descriptive comparison of test-based detection across eras, the proportion of women with ASC-US or worse per woman screened was contrasted between the cytology-based and HPV-based periods using a risk ratio (RR) with a normal-approximation 95% CI and the χ^2^ test. Analyses were performed in Python 3.12 (pandas 2.2, SciPy 1.13, statsmodels 0.14); the significance threshold was α = 0.05.

### 2.8. Ethics

This study used de-identified, individual-level programme-extracted data. Ethical approval was obtained from the Ethics Committee of Pelican Clinical Hospital, Oradea (approval no. 2688, dated 29 December 2021). On admission to the hospital, all patients had signed the institutional consent authorising the further processing of their data for teaching and research purposes; because the present analysis used only retrospective, de-identified records, no additional study-specific consent was required. This study was conducted in accordance with the Declaration of Helsinki.

### 2.9. Reporting and Data Integrity

This study is reported in accordance with the STROBE (Strengthening the Reporting of Observational Studies in Epidemiology) statement [27] and the RECORD (REporting of studies Conducted using Observational Routinely collected Data) extension for routinely collected health data [28]; a completed checklist is provided as Supplementary Material. Two documented data-quality points are noted: age band was not recorded for 130 women screened in 2018 (0.7% of the dataset), so age-stratified denominators are 130 lower than the corresponding test totals for that year; and, in the HPV-primary era, reflex cytology was performed only for HPV-positive women, so cytological positivity is reported for the cytology years and HR-HPV positivity for the HPV years, with the two not directly comparable.

## 3. Results

### 3.1. Eight Years of Screening: Cumulative Screening Activity and Yield

Between 2018 and 2025, 17,807 screening episodes were recorded at the centre, corresponding to 27.0% (95% CI 26.6–27.3) of the 65,978 registered women; because episodes could not be linked to individual women, this figure represents cumulative provider-level throughput relative to the registered catchment rather than population-based coverage. Across the six cytology years (2018–2023), 1717 of 11,673 satisfactory smears were abnormal (positivity 14.7% of satisfactory smears, 95% CI 14.1–15.4; equivalently 14.2% of all 12,107 tests); adding the 623 abnormal reflex-cytology results from HPV-positive women in 2024–2025 gives 2340 abnormal cytology results over the full period. Annual throughput relative to the registered population never exceeded 5.2% (2019) in any year and fell as low as 1.10% in the partial year 2021 (Table 1, Figure S1).

### 3.2. Screening Volume: Collapse, Recovery and HPV-Primary Scale-Up

**Figure 2:**
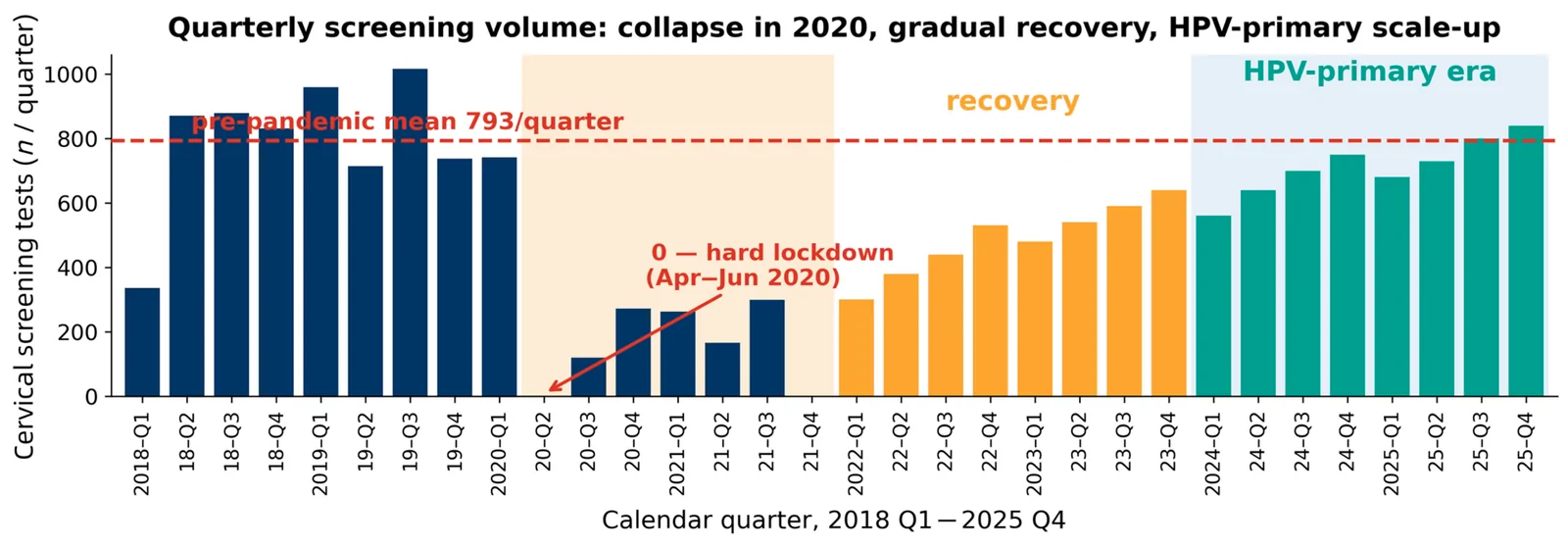
Quarterly cervical screening volume, 2018 Q1–2025 Q4. Navy bars, cytology (pre-pandemic and pandemic); orange bars, cytology during the post-pandemic recovery (shaded band = pandemic); teal bars, HR-HPV primary era. The dashed line marks the pre-pandemic quarterly mean. Screening fell to zero during the second-quarter 2020 lockdown and recovered progressively thereafter.

Quarterly returns across 2018 Q1–2025 Q4 (30 quarters with recorded activity; no tests during the 2020 Q2 lockdown quarter, and 2021 covered Q1–Q3 only) show a clear four-phase trajectory (Figure 2). After a stable pre-pandemic average of 793 tests per quarter, activity fell to zero in the second quarter of 2020, the period of Romania’s hard national lockdown (April–June 2020), recovered only partially through 2021, then rose steadily during 2022–2023 and again under HPV-primary screening in 2024–2025, when the final quarters approached pre-pandemic levels. Annual volume fell from a 2019 peak of 3429 tests to 1132 in 2020 and 728 in the first three quarters of 2021 then climbed to 1650 (2022), 2250 (2023), 2650 (2024) and 3050 (2025) (Table 1).

### 3.3. Collapse of Screening Activity During the COVID-19 Pandemic

**Figure 3:**
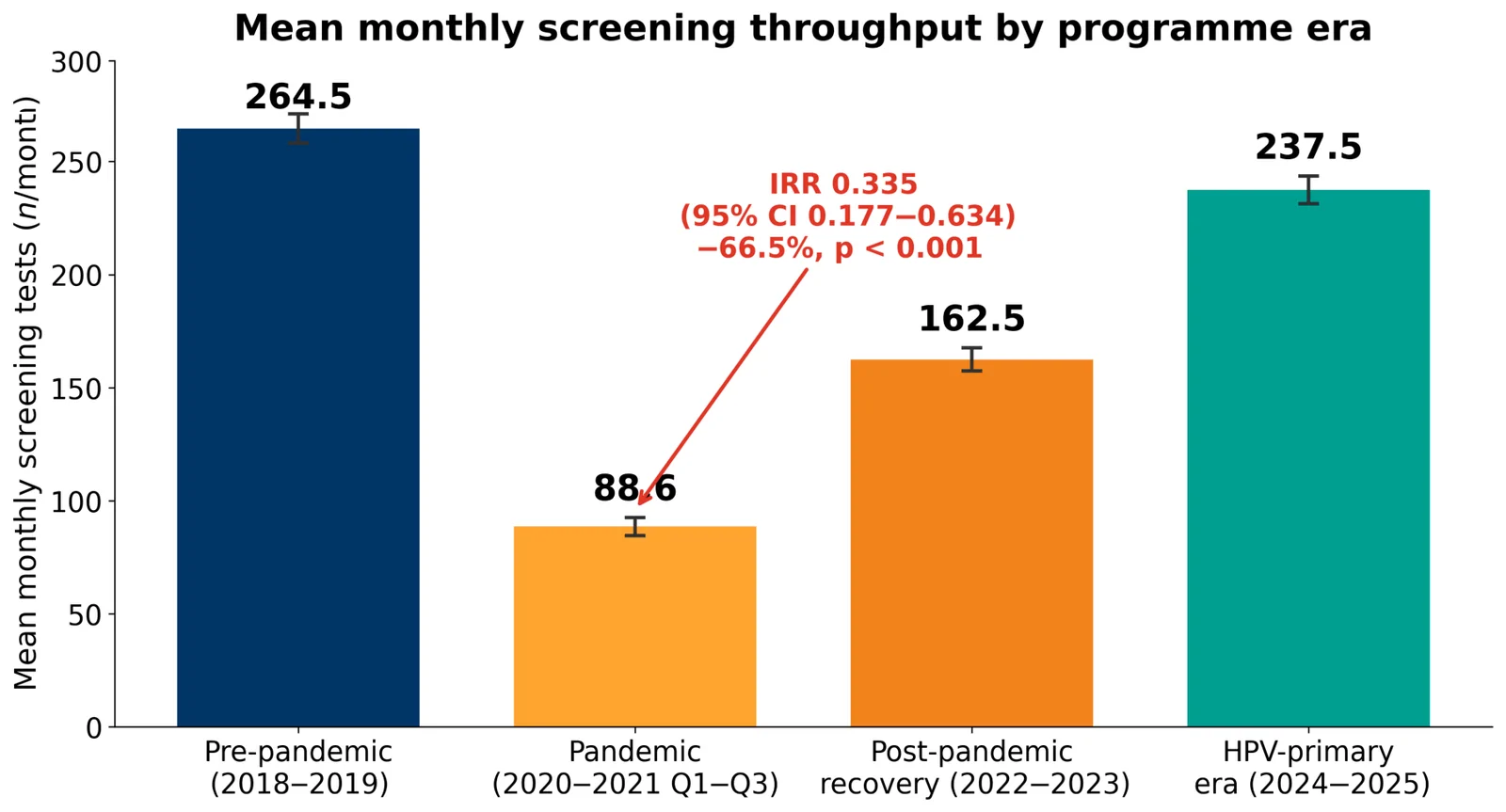
Mean monthly screening throughput by programme era, with the negative-binomial incidence-rate ratio (estimated on quarterly counts) for the pandemic-versus-pre-pandemic contrast. Error bars are approximate 95% intervals on the monthly rate.

Mean monthly throughput fell from 264.5 tests/month before the pandemic to 88.6 during it, a negative-binomial IRR of 0.335 (95% CI 0.177–0.634), a 66.5% reduction (*p* < 0.001; Figure 3, Table S1). Because the quarterly counts were markedly overdispersed (Pearson χ^2^/df = 69.9), this interval is appropriately wide; the estimate was unchanged under a quasi-Poisson model (0.335, 95% CI 0.188–0.597) and robust across pre-specified sensitivity analyses (point estimate 0.31–0.34 throughout; the interval excluded 1.0 in the primary, 2019-baseline and quasi-Poisson analyses and marginally included it in the 2019-versus-2020 complete-year comparison; Figure S2, Table S2). In absolute terms, over the same 21-month pandemic window (all of 2020 plus January–September 2021), approximately 5550 tests would have been expected had the pre-pandemic rate held constant, against 1860 observed, an estimated deficit of about 3700 tests (a 66.5% shortfall). Extrapolating the modest rising pre-pandemic trend instead raises the expected count to roughly 8300 and the deficit to about 6400 tests over the same window (Figure S3); we take the constant-rate figure (~3700) as the primary, more conservative estimate.

The segmented negative-binomial interrupted time-series, fitted to the quarterly counts, confirmed and refined this picture (Figure S3). Against a modestly rising pre-pandemic baseline (+3.4% per quarter, 95% CI −4.1 to +11.6%), screening volume dropped abruptly at the April 2020 lockdown (an immediate level-change ratio of 0.14, 95% CI 0.09–0.24, i.e., an approximately 86% instantaneous fall) and then climbed steadily through the recovery and into the HPV-primary era, when the last quarters of 2025 met the pre-pandemic counterfactual.

### 3.4. Recovery and the Transition to HPV-Primary Screening

Screening activity recovered progressively after 2021. In 2022–2023, the mean monthly rate reached 162.5 tests/month, 61.4% of the pre-pandemic rate (negative-binomial IRR 0.614, 95% CI 0.482–0.783; *p* < 0.001), still well below baseline but a clear rebound from the pandemic trough. Under HPV-primary screening in 2024–2025, the rate rose further to 237.5 tests/month, 89.8% of the pre-pandemic level (IRR 0.898, 95% CI 0.733–1.100; *p* = 0.30), statistically indistinguishable from the pre-pandemic baseline, with the last two quarters of 2025 matching pre-pandemic throughput (Figure 2). The modality change also markedly improved sample adequacy: the primary HR-HPV DNA test returned a valid result in every sample (0% invalid), against a 2.9–4.2% unsatisfactory-smear rate in the cytology years, while reflex cytology among HPV-positive women was inadequate in 1.0% (Table 1).

**Figure 4:**
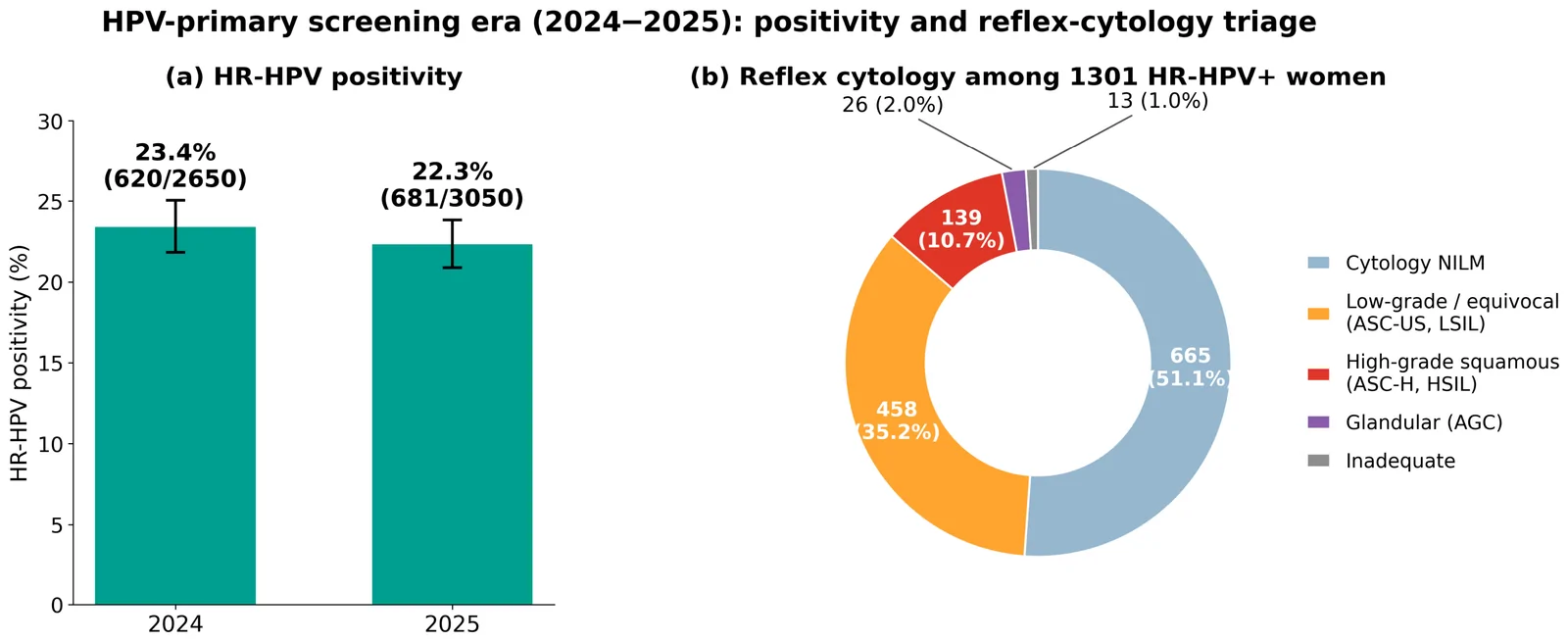
HPV-primary screening era (2024–2025). (**a**) HR-HPV positivity by year (23.4%, 620/2650 in 2024; 22.3%, 681/3050 in 2025), with 95% Wilson confidence intervals. (**b**) Distribution of reflex cytology among the 1301 HR-HPV-positive women, with counts and percentages; categories are arranged clockwise from the top: NILM (*n* = 665, 51.1%), low-grade/equivocal (ASC-US/LSIL; *n* = 458, 35.2%), high-grade squamous (ASC-H/HSIL; *n* = 139, 10.7%), glandular (AGC; *n* = 26, 2.0%) and inadequate (*n* = 13, 1.0%). NILM denotes a normal reflex smear in an HPV-positive woman.

In 2024–2025, HR-HPV DNA was positive in 1301 of 5700 tests, an overall positivity of 22.8% (95% CI 21.8–23.9), consistent between years (23.4% in 2024, 22.3% in 2025; Figure 4a). Reflex cytology of the 1301 HPV-positive women was normal (NILM) in 665 (51.1%), abnormal in 623 (47.9%; 458 low-grade or equivocal [ASC-US/LSIL], 139 high-grade squamous [ASC-H/HSIL, of which 101 definite HSIL] and 26 glandular [AGC]) and inadequate in the remaining 13 (1.0%) (Figure 4b).

Considered per woman screened, the detection of any cytological abnormality (ASC-US or worse) was lower under HPV-primary screening than under primary cytology: 623/5700 (10.9%) versus 1717/12,107 (14.2%), an absolute difference of 3.3 percentage points and a relative reduction of approximately 23% (RR 0.77, 95% CI 0.71–0.84; *p* < 0.001). This comparison should be interpreted cautiously: cytology was performed for all women during the cytology-based period but only for HPV-positive women during the HPV-based period, and the two periods may differ in the age structure, screening history and underlying risk of the women attending (see Section 4.6 Strengths and Limitations).

Applying a standard HPV-primary triage algorithm to these test results, under which HPV-positive women with any abnormal reflex cytology (ASC-US or worse, including atypical glandular cells) would be referred for immediate colposcopy, while those with a normal reflex smear (NILM) would enter 12-month HPV-based surveillance, projects that, of the 1301 HPV-positive women, 623 (47.9%) would be referred for immediate colposcopy and 665 (51.1%) to surveillance, with 13 (1.0%) requiring a repeat test for an inadequate reflex smear. This corresponds to a projected immediate-colposcopy referral rate of 10.9% of all women screened by HPV (623/5700), of whom 139 (2.4% of those screened) had high-grade squamous cytology that would warrant prioritised referral. These figures are analytical projections obtained by applying the triage rule to the observed test results; they are not observed colposcopy or histology outcomes, which were not available in this dataset and would need to be confirmed against actual referral and biopsy data.

### 3.5. Cytological Yield and Lesion Grade

Cytological positivity per satisfactory smear was stable across the cytology years (2018: 14.7%; 2019: 14.3%; 2020: 15.5%; 2021: 13.6%; 2022: 15.5%; 2023: 14.7%; Figure S4). In logistic regression, the odds of a positive smear did not change with calendar year (OR 1.01, 95% CI 0.98–1.03, *p* = 0.69); this was unchanged after adjustment for five-year age band (adjusted OR 1.01, 95% CI 0.98–1.03), and the Cochran–Armitage test concurred (Z = 0.40, *p* = 0.69), giving no evidence of a time trend once the age mix of attenders is taken into account. Overall positivity did not differ between the pre-pandemic and pandemic periods (14.5% vs. 14.7%; Fisher *p* = 0.82), giving no evidence that pandemic attenders were systematically higher- or lower-risk overall.

Across the full period, the 2340 abnormal cytology results were predominantly low-grade: ASC-US accounted for 1009 (43.1%) and LSIL for 633 (27.1%), so 1642 (70.2%) were low-grade or equivocal; high-grade squamous cytology comprised 158 ASC-H (6.8%) and 423 definite HSIL (18.1%), alongside 117 AGC (5.0%) (Table 2). No invasive carcinoma was recorded in the categorised data.

**Figure 5:**
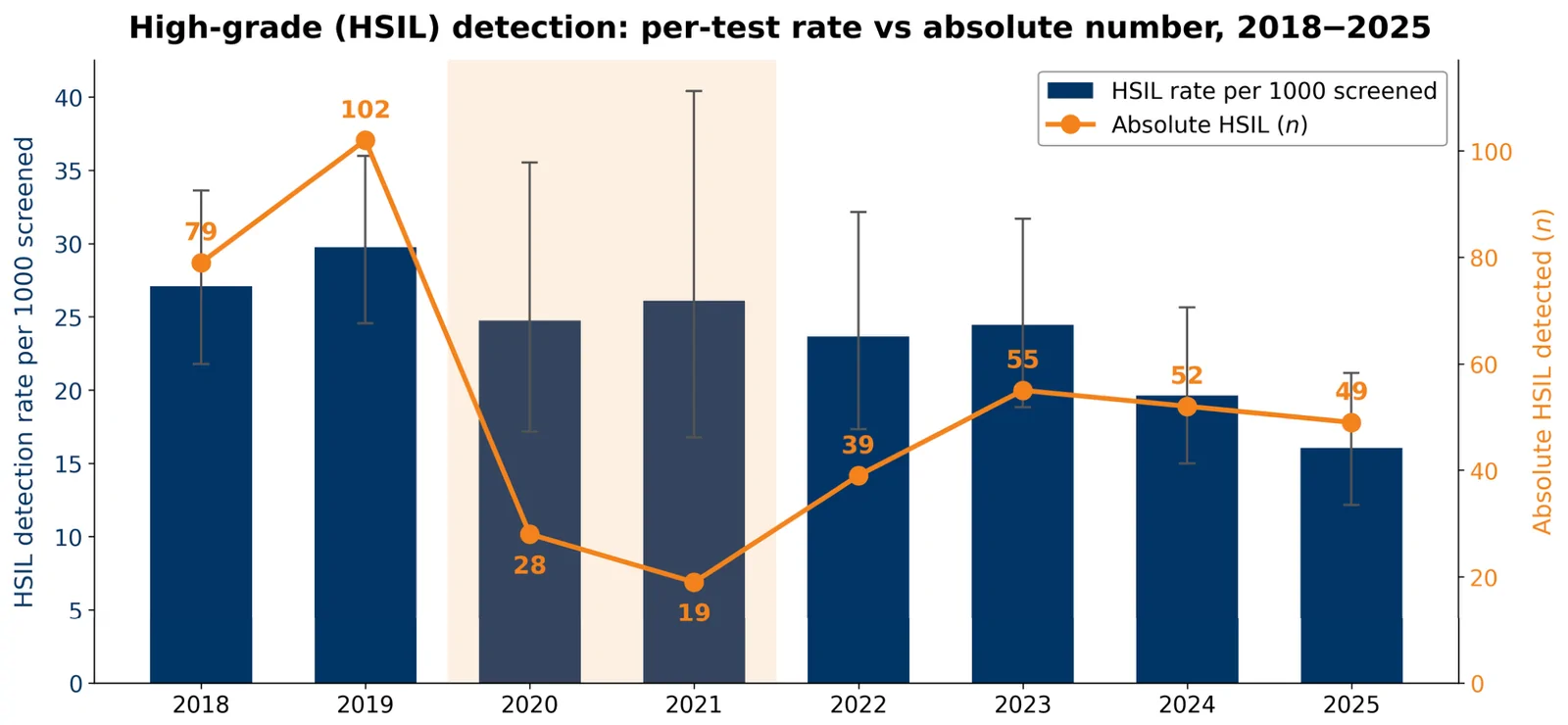
High-grade (HSIL) detection, 2018–2025: detection rate per 1000 women screened (bars, left axis, with 95% Wilson CIs) and absolute number of HSIL detected (line, right axis). Shaded band = pandemic period.

High-grade cytological findings (HSIL) showed a different pattern (Figure 5). The absolute number of HSIL detected fell during the pandemic (from 102 in 2019 to 28 in 2020 and 19 in the first three quarters of 2021), but the HSIL detection rate per 1000 women screened remained broadly stable (27.1, 29.7, 24.7 and 26.1 per 1000 in 2018–2021, with no significant time trend; Poisson rate ratio 0.98 per year, 95% CI 0.85–1.13, *p* = 0.78) and stayed in a similar range during the recovery (23.6 and 24.4 per 1000 in 2022–2023) before easing to 19.6 and 16.1 per 1000 in the HPV-primary years, when HSIL was detected through reflex cytology of HPV-positive women. The stable per-test detection rate despite falling absolute detections indicates that the reduced high-grade yield during the pandemic reflected fewer women screened rather than any loss of test performance; the principal harm is therefore likely to lie in high-grade lesions left undetected because women were not screened. Because high-grade lesions in the 2024–2025 HPV-primary years were identified only through reflex cytology of HPV-positive women, the per-1000 detection rates for those two years are not directly comparable with the cytology-era rates and should be read with that modality change in mind.

### 3.6. Sample Adequacy, Age Distribution and Concurrent Infections

The unsatisfactory-sample rate ranged from 2.9% to 4.2% across the cytology years and fell to zero under HR-HPV DNA testing, which returned a valid result in every sample; reflex cytology among HPV-positive women was inadequate in 1.0% (Table 1). Screening remained concentrated in older women throughout: three-fifths of tests (60.3%) were in women aged 40–64 and only 39.7% in the youngest eligible groups (25–39), with the 40–44 band the single largest stratum (3577 women, 20.2%) (Table S3). Encouragingly, participation by women aged 25–39 rose markedly in the HPV-primary era (from 32–34% of attenders before the pandemic to a larger share in 2024–2025), though the overall age profile still skews older than the ages at which incident oncogenic-HPV infection peaks. Concurrent infective findings were noted in 707 women: Candida (*n* = 343), *Trichomonas vaginalis* (*n* = 178), Gardnerella/bacterial vaginosis (*n* = 123) and other organisms (*n* = 63).

## 4. Discussion

### 4.1. Principal Findings

Three findings stand out from these eight years of programme data. First, even in its best years, the programme reached only about 4–5% of its registered target population annually, and roughly 27% cumulatively, a chronic coverage deficit consistent with Romania’s standing among the EU’s lowest-screening, highest-burden countries [9,13]. Second, the COVID-19 pandemic produced a two-thirds collapse in throughput (IRR 0.335; −66.5%), superimposed on that already inadequate baseline, with screening reaching zero during the 2020 lockdown. Third, and newly demonstrable with the extended data, activity recovered progressively thereafter, reaching about 90% of pre-pandemic volume under HPV-primary screening in 2024–2025, which simultaneously eliminated unsatisfactory primary samples: the HR-HPV DNA test returned a valid result in every sample, against 2.9–4.2% unsatisfactory smears under cytology. The pandemic did not create the coverage gap; it deepened a pre-existing one that the subsequent recovery, coinciding with the transition to HPV-primary screening, has begun, but not yet finished, to close.

### 4.2. Coverage in the European and Romanian Policy Context

Our coverage figures fall far short of the benchmarks now enshrined in European policy. Europe’s Beating Cancer Plan set the goal that 90% of the eligible population be offered cervical screening by 2025 [5], and the 2022 EU Council Recommendation frames HPV testing as the preferred primary modality for women aged 30–65 [6]; the WHO European roadmap similarly makes organised, HPV-based screening the operational core of elimination [4]. An annual coverage of 4–5%, and roughly 27% cumulatively, sits at the opposite end of this spectrum from the >70% achieved by organised, invitation-based programmes in the Netherlands, Finland and the United Kingdom [9] and below the roughly half-of-EU-average within-interval screening recorded for Romania [13]. The structural explanation is well rehearsed: opportunistic delivery without centralised invitation and recall, fragmented administration, limited provider engagement and rural access barriers [11,12]. Romania’s Law 293/2022 and National Cancer Plan 2023–2030 now provide, for the first time, a legislative basis and measurable objectives for organised, HPV-based screening [7]; the challenge is implementation. Cervical screening in Romania is delivered through a network of regionally accredited providers, of which this centre is one; individual accredited providers, however active, cannot collectively substitute for a population-based call-and-recall system in the absence of centralised invitation and recall. Our denominator captures only this centre’s registered catchment, so true county-wide coverage, summing all providers and opportunistic testing, may be somewhat higher, but the direction of the finding is unambiguous. Encouragingly, the reforms adopted since 2023, namely the expansion of reimbursed HPV vaccination from December 2023 [15,16], extended further from 1 October 2025 [17], and the June 2024 national screening methodology mandating HPV-based primary testing with reflex cytology triage [18], directly target the structural weaknesses identified here, and recent policy analyses outline the implementation steps still required [19].

### 4.3. The Pandemic Collapse and the Shape of Recovery

The magnitude of disruption we observed (−66.5%; IRR 0.335) sits at the upper end of the international range. A meta-analysis of early-pandemic data found the pooled proportion of women screened fell by roughly half [20], so the two-thirds collapse here is steeper than the typical organised-programme experience. Many well-organised Western programmes suffered steep but time-limited suspensions followed by rapid, invitation-driven catch-up [22]; modelling by Castanon and colleagues [21] and the multi-country analyses by Smith and colleagues [22] indicate that even temporary disruptions, if not recovered, translate into measurable excess cervical cancer cases concentrated among under-screened women [23]. The disruption was not confined to screening: across Romania, the pandemic strained the wider cancer-care continuum, with a three-year national study documenting substantial reductions and delays in colorectal cancer surgery over the same period [29], indicating that the screening losses reported here compounded broader interruptions to cancer diagnosis and treatment. The extended data let us characterise recovery directly: activity returned to about three-fifths of baseline in 2022–2023 and to about nine-tenths under HPV-primary screening in 2024–2025. Because the transition to HPV-primary testing coincided with the later stages of post-pandemic recovery, and with the December 2023 expansion of reimbursed HPV vaccination [15,16], the heightened public attention to HPV-related disease that accompanied it and the June 2024 national screening methodology [18], the 2024–2025 increase in volume cannot be attributed to the modality change alone. This recovery is encouraging but incomplete: roughly 3700 tests were never performed during 2020–2021, drawn from a population already heavily under-screened, and a volume rebound does not by itself recover the lesions missed in the interim. The reassuring absence of a positivity shift between the pre-pandemic and pandemic periods should not be misread as benign; it indicates that the collapse was one of volume rather than case-mix, so the harm lies in lesions not detected at all, underscoring the need for active catch-up of the missed cohorts rather than a simple return to steady-state throughput.

### 4.4. Cytological and HPV Yield, Lesion Grade and Age

Cytological positivity (~14–16% per satisfactory smear) is high relative to organised Western programmes, where positivity is typically a few percent [9], reflecting both the opportunistic, partly symptom- or risk-driven nature of attendance and Romania’s high background oncogenic-HPV prevalence. In the national survey reporting regional estimates, high-risk HPV prevalence was 19.2% in the northern grouping that includes Bihor and 23.0% in the western region [30]; comparably elevated positivity has been documented in primary HPV-based screening in north-western Romania [31]. Our own HPV-primary data are concordant and, if anything, at the upper end of this range: HR-HPV positivity was 22.8% in 2024–2025, closely matching the 23.0% reported for the western region, and about half of HPV-positive women had abnormal reflex cytology, with roughly one in nine showing high-grade squamous changes. Cytological positivity was stable over 2018–2023 (no significant time trend, unchanged after age adjustment), consistent with a stable attender risk profile across the cytology era rather than any secular change in disease. Importantly, the Bethesda breakdown shows that abnormal cytology is not equivalent to cancer averted: most positives were low-grade or equivocal (70.2% ASC-US/LSIL), with definite high-grade squamous (HSIL) lesions accounting for 18.1%; the programme’s preventive value lies in detecting and treating high-grade lesions, which requires colposcopy and histological follow-up not captured here. Throughout this article, HSIL refers to a cytological result: without linkage to histology, it cannot be equated with histologically confirmed cervical intraepithelial neoplasia grade 3 or worse (CIN3+), and the reported rates therefore describe screening-test outcomes rather than confirmed precancerous lesions. Adjunctive molecular biomarkers may help narrow this triage gap: p16/Ki-67 dual staining (CINtec PLUS) has been evaluated in Romania as an alternative method for detecting high-grade cervical lesions and improving the specificity of cytology-based screening [32]. More broadly, definitive diagnosis and management of the lesions identified at screening rest on accurate histopathological examination, the decisive step in confirming or excluding malignancy across diverse tumours and unusual presentations [33,34,35], supported, where relevant, by correlative imaging and cross-sectional radiology to delineate disease extent [36,37].

The high-grade findings reinforce the interpretation of the pandemic. The HSIL rate per 1000 women screened stayed broadly stable across the pandemic while absolute detections fell with volume, indicating that the drop in high-grade yield was driven by fewer women screened rather than any change in per-test detection. The age profile remained skewed towards women aged 40–64, though the greater participation of younger women (25–39) in the HPV-primary era is a welcome shift, since these are the ages at which incident oncogenic-HPV infection is most common and at which screening and vaccination carry the greatest preventive value.

When comparing the two screening strategies, an important distinction must also be drawn between baseline and cumulative detection. Primary HPV screening with immediate colposcopy restricted to HPV-positive women with ASC-US or worse may detect fewer high-grade lesions at the baseline round than cytology-based screening, in which all women with ASC-US or worse are referred irrespective of HPV status, including the small subgroup with HPV-negative HSIL and underlying CIN3+. Baseline detection alone, however, does not reflect the overall effectiveness of HPV-based screening: additional CIN3+ lesions are detected through surveillance of HPV-positive women with normal reflex cytology, whose CIN3+ risk is substantially higher than that of HPV-negative women, and a negative HPV test provides greater and longer-lasting reassurance against CIN3+ at the next screening round than normal cytology alone [6,26]. The lower per-woman detection of cytological abnormalities that we observed under HPV-primary screening (10.9% versus 14.2%) should be read in this light. A valid comparison of the two strategies therefore requires complete longitudinal follow-up, including of HPV-positive women with normal reflex cytology, with histologically confirmed CIN3+ as the endpoint, rather than reliance on baseline cytological abnormalities.

### 4.5. Implications for Practice and Policy

These findings reinforce the direction of Romania’s recent reforms and align this centre’s experience with European policy. The observed transition to HR-HPV primary testing, which restored volume to near pre-pandemic levels and eliminated unsatisfactory primary samples (the HR-HPV DNA test was valid in every sample), is exactly the modality shift endorsed by the 2022 EU Council Recommendation [6] and Romania’s National Cancer Plan 2023–2030 [7], and it is the operational core of the WHO European elimination roadmap [4] and Europe’s Beating Cancer Plan [5]. Realising the benefit at population scale, however, requires the missing infrastructure: a functioning invitation-and-recall system anchored to the registered population, active recruitment by family physicians, prioritised catch-up of the cohorts missed in 2020–2021 and targeted outreach to rural and under-served communities. Equity deserves particular emphasis: national data show that high-risk HPV prevalence and cervical cancer burden fall unevenly across Romania’s regions and ethnic subpopulations, including Roma communities [30], and although our dataset records only age and cannot itself examine residence or ethnicity, the pronounced skew of attendance towards older women is consistent with rural and marginalised populations being under-reached, so these groups should be an explicit priority for invitation, self-sampling and outreach. Self-sampling, shown to be acceptable to Romanian women [38], offers a concrete lever to reach non-attenders and could be paired with HPV-primary testing to extend coverage rapidly. For service planning, the HPV-primary test results imply a tangible triage workload (roughly one in nine women screened by HPV would be referred for colposcopy and about one in forty would have high-grade cytology warranting prioritised referral) that programmes adopting HPV-based screening will need to resource, though these are projections from test results that require confirmation against actual colposcopy data. Beyond primary screening and triage, there is growing interest in low-cost, routinely available biomarkers to refine risk stratification and prioritise follow-up in oncology more broadly, for example, pre-operative inflammatory indices such as the neutrophil-to-lymphocyte and platelet-to-lymphocyte ratios, whose prognostic value has been demonstrated in other cancers [39]; whether such adjuncts can help prioritise colposcopy referral or catch-up in resource-constrained cervical programmes is an open question that merits evaluation. Equally, the value of early detection is ultimately realised only through the treatment pillar of the elimination strategy, so parallel efforts to reduce the morbidity of oncological surgery, for example, evidence syntheses of perioperative techniques such as closed-incision negative-pressure wound therapy in complex cancer operations [40], complement, rather than compete with, investment in screening. Finally, the value of screening is fully realised only within a patient-centred care pathway that spans detection, treatment and recovery. On the demand side, documented barriers to timely care-seeking among Romanian patients illustrate the attitudinal and access obstacles that invitation and outreach must overcome [41]. On the diagnostic side, non-invasive circulating markers such as blood and plasma microRNAs are being explored to refine risk stratification in colorectal and other cancers [42]. And on the survivorship side, multi-disciplinary rehabilitation and psycho-oncological support that protect functional recovery and quality of life after cancer treatment [43,44,45] complement, rather than compete with, investment in early detection.

### 4.6. Strengths and Limitations

The principal strengths of this study are the eight-year observation period, which encompasses pre-pandemic activity, the COVID-19 disruption, the post-pandemic recovery and the transition to primary HPV screening, and the use of routinely collected, individual-level screening records reconciled with official, coordinator-signed programme returns. This study provides rare empirical evidence on cervical screening activity in a high-burden, historically underserved Central and Eastern European setting. The statistical analysis accounts for the substantial overdispersion of the quarterly counts through negative-binomial models, a segmented interrupted time-series and pre-specified sensitivity analyses, and reporting was guided by the STROBE statement and the RECORD extension for routinely collected health data [27,28].

Several limitations require consideration. First, this was a single-centre study, and the denominator of 65,978 women represents the provider’s administrative catchment rather than an independently validated population of all screening-eligible women in Bihor County; against the 2021 census count of 283,400 resident women in the county [25], of whom those aged 25–64 form a subset, coverage of the full age-eligible population would be substantially lower. Furthermore, the records lacked a persistent personal identifier across screening episodes, so women screened in more than one year may have been counted more than once. The reported percentages are consequently better interpreted as provider-level screening throughput relative to the registered catchment than as population-based coverage. This study cannot determine the proportion of unique women screened within the recommended interval, previous screening history, re-attendance, adherence to recommended intervals after a negative test or the extent of overscreening and direct comparison with coverage estimates from organised programmes, which generally measure the proportion of eligible women screened within a specified multi-year interval, should be made cautiously.

Second, the transition from cytology-based to HPV-based screening coincided with the later stages of post-pandemic recovery and with substantial national policy changes, so the increase in screening volume during 2024–2025 cannot be attributed solely to the change in screening modality. The two periods may also differ in the age, previous screening history and underlying risk of the women attending; cytological positivity during primary cytology and abnormal reflex cytology among HPV-positive women represent different testing pathways and are not directly comparable without adjustment for these differences. Detailed information on the screening indication, adherence to the recommended intervals and age-specific algorithms and previous test results was unavailable. In addition, although the cobas HPV assay reports HPV16 and HPV18 separately, only the pooled HR-HPV result was recorded in the programme returns, so genotype-specific positivity and genotype-guided management could not be examined.

Third, the dataset was not linked to colposcopy, biopsy, histological diagnosis, treatment, or cancer-incidence outcomes. Cytological HSIL was therefore used as an indicator of a significant screening finding but cannot be considered equivalent to histologically confirmed CIN3+. This study cannot estimate CIN3+ detection, sensitivity, specificity, positive predictive value, treatment rates, or the downstream effect of the pandemic-related reduction in screening, and it cannot compare the clinical effectiveness of the two screening strategies because follow-up outcomes were unavailable, particularly for HPV-positive women with normal reflex cytology, who entered surveillance. A valid comparison of cytology-based and HPV-based screening would require longitudinal follow-up through repeat testing, colposcopy and histology, with CIN3+ as the principal clinical endpoint.

Finally, data for 2021 covered only the first three quarters and required annualisation for some comparisons, and age band was missing for 130 screening episodes in 2018. These limitations, together with the observational before-and-after design, mean that the findings should be interpreted as a descriptive analysis of screening activity and test outcomes at one provider rather than as a causal evaluation of the comparative effectiveness of cytology-based and HPV-based screening.

## 5. Conclusions

At this accredited centre in a Romanian region with a high cervical cancer burden, screening activity over eight years corresponded to approximately 27% of the registered target population. Because repeated screening episodes could not be linked to individual women and the denominator represented a single provider’s administrative catchment, this figure should not be interpreted as population-based screening coverage. Screening activity declined by approximately two-thirds during the COVID-19 pandemic and subsequently recovered to nearly the pre-pandemic level during the period coinciding with the transition to primary HPV screening, which also produced valid results in all samples, compared with an unsatisfactory-sample rate of 2.9–4.2% during cytology-based screening.

These findings describe screening activity and test outcomes but do not establish the comparative clinical effectiveness of cytology-based and HPV-based screening. The absence of longitudinal linkage to colposcopy, histology and treatment means that cytological HSIL cannot be considered equivalent to CIN3+, and the detection of confirmed precancerous lesions could not be evaluated. Future assessments should include complete follow-up of all screening outcomes, particularly of HPV-positive women with normal reflex cytology, and use histologically confirmed CIN3+ as the principal endpoint. Set against Europe’s Beating Cancer Plan [5], the 2022 EU cancer-screening Recommendation [6], the WHO European elimination roadmap [4] and Romania’s National Cancer Plan 2023–2030 and 2024 national screening methodology [7,18], the findings nevertheless highlight the need for population-based invitation and recall, clearly defined age-specific screening and follow-up algorithms, appropriate intervals after negative HPV tests, self-sampling for non-attenders and targeted catch-up of women whose screening was delayed during the pandemic.

## Figures and Tables

**Table 1:** Annual screening activity, coverage and primary-test positivity, Pelican Clinical Hospital, Bihor County, 2018–2025.

Year	Primary Test	Tests Performed (*n*)	Unsatisfactory *n* (%)	Activity/Catchment ^a^ (%)	Abnormal Cytology/HR-HPV+ (*n*)	Positivity/HR-HPV+ ^b^ (%)
2018	Cytology	2918	97 (3.3)	4.42	415	14.7
2019	Cytology	3429	106 (3.1)	5.20	476	14.3
2020	Cytology	1132	48 (4.2)	1.72	168	15.5
2021 ^c^	Cytology	728	21 (2.9)	1.10	96	13.6
2022	Cytology	1650	69 (4.2)	2.50	245	15.5
2023	Cytology	2250	93 (4.1)	3.41	317	14.7
2024	HR-HPV DNA	2650	0 (0.0)	4.02	620	23.4
2025	HR-HPV DNA	3050	0 (0.0)	4.62	681	22.3
**2018–2025**	**—**	**17,807**	**—**	**26.99**	**—**	**—**

^a^ Screening activity relative to the registered catchment (tests performed/registered target population, 65,978); this reflects provider-level throughput, not population-based coverage. ^b^ For cytology years, positivity = abnormal/satisfactory smears; for HPV-primary years (2024–2025), the final column is HR-HPV positivity (HR-HPV+/valid HPV tests) and the preceding column reports HR-HPV+ counts. ^c^ 2021 covers Q1–Q3 only (nine months). In 2024–2025, the primary HR-HPV DNA test returned a valid result in every sample (0% unsatisfactory); reflex cytology among the 1301 HR-HPV-positive women was inadequate in 13 cases (3 in 2024, 10 in 2025), i.e., 1.0% of HPV-positive women.

**Table 2:** Distribution of abnormal cytology by Bethesda category, 2018–2025.

Year	ASC-US	LSIL	ASC-H	HSIL	AGC	Abnormal (*d*)
2018	179	105	28	79	24	415
2019	195	125	40	102	14	476
2020	71	53	9	28	7	168
2021 ^a^	40	21	7	19	9	96
2022	102	74	17	39	13	245
2023	127	92	19	55	24	317
2024 ^b^	159	68	19	52	13	311
2025 ^b^	136	95	19	49	13	312
**Total**	**1009**	**633**	**158**	**423**	**117**	**2340**

ASC-US, atypical squamous cells of undetermined significance; LSIL, low-grade squamous intraepithelial lesion; ASC-H, atypical squamous cells—cannot exclude HSIL; HSIL, high-grade squamous intraepithelial lesion; AGC, atypical glandular cells. ^a^ 2021 covers Q1–Q3. ^b^ For 2024–2025, the counts are reflex cytology among HR-HPV-positive women.

## Data Availability

The de-identified individual-level data analysed in this study are available as Supplementary Material Table S5.

## Supplementary Materials

The following supporting information can be downloaded at: https://www.mdpi.com/article/10.3390/healthcare14172850/s1, Figure S1: Annual screening volume (bars, left axis) and coverage of the registered target population (line, right axis), 2018–2025; Figure S2: Robustness of the pandemic-related reduction in screening volume; Figure S3: Segmented negative-binomial interrupted time-series of quarterly screening volume, 2018–2025; Figure S4: Cytological positivity (positive/satisfactory smears) with 95% Wilson confidence intervals, 2018–2023, with the Cochran–Armitage trend statistic (Z = 0.40, *p* = 0.69); Table S1: Summary of inferential analyses; Table S2: Sensitivity of the screening-volume incidence-rate ratio to the choice of baseline period and the dispersion model; Table S3: Distribution of screened women by age group and programme era, 2018–2025; Table S4: Completed STROBE/RECORD reporting checklist; Table S5: Raw data.
