# Supplementary material for: Cervical Cancer Screening Activity at an Accredited Centre in Romania, 2018–2025: COVID-19 Disruption, Recovery and the Transition to Primary HPV Testing
Source: Healthcare (Basel). 2026 Sep 4;14(17):2850. doi: 10.3390/healthcare14172850 (PMC13566146; doi:10.3390/healthcare14172850)
Supplement: Supplementary file 1 [file healthcare-14-02850-s001.zip › healthcare-4482925-supplementary.pdf]

Supplementary Materials

Cervical Cancer Screening Activity at an Accredited Centre in Romania, 2018–2025: COVID-19 Disruption, Recovery and the Transition to Primary HPV Testing

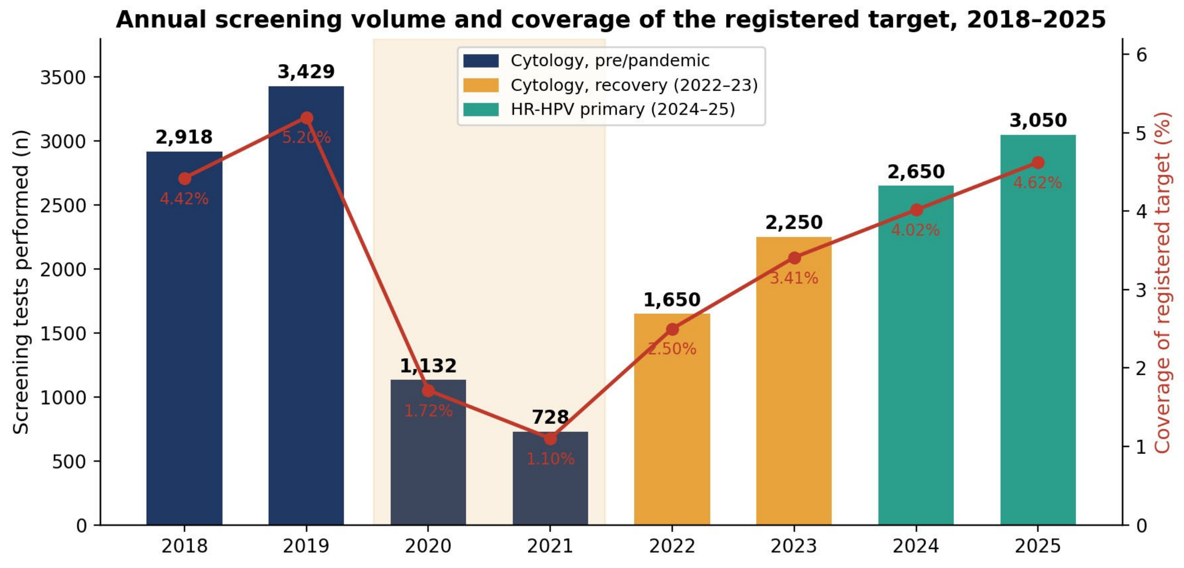

Figure S1. Annual screening volume (bars, left axis) and coverage of the registered target population (line, right axis), 2018–2025. Navy, cytology (pre/pandemic); orange, cytology recovery; teal, HR-HPV primary. The shaded band marks the COVID-19 period.

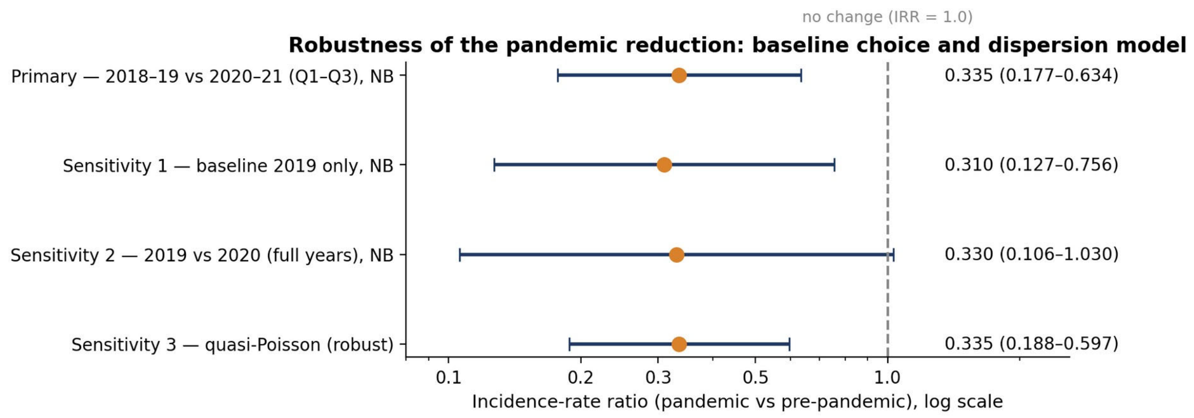

Figure S2. Robustness of the pandemic-related reduction in screening volume. Negative-binomial incidence-rate ratios (pandemic vs. pre-pandemic) for the primary analysis, two alternative baselines and a quasi-Poisson dispersion check, with 95% confidence intervals on a logarithmic scale; the dashed line marks no change (IRR = 1.0).

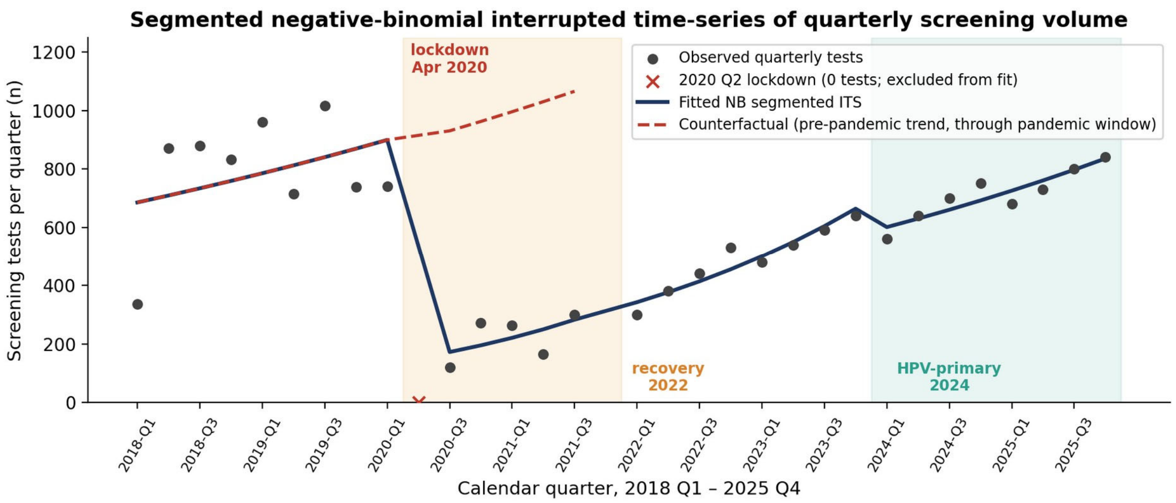

Figure S3. Segmented negative-binomial interrupted time-series of quarterly screening volume, 2018–2025. Points are observed quarterly test counts; the solid line is the fitted model with level and slope changes at the April 2020 lockdown, the 2022 recovery and the 2024 transition to HPV-primary screening; the dashed line is the counterfactual extrapolating the pre-pandemic trend through the pandemic window. The zero-activity lockdown quarter (2020 Q2) is shown for reference but was not available as a valid count observation for the fitted series.

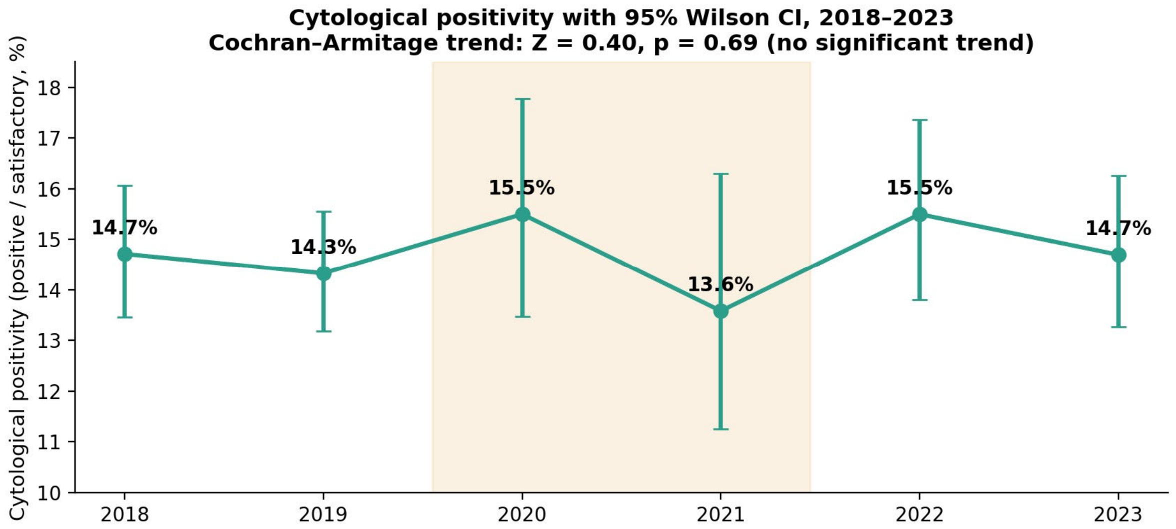

Figure S4. Cytological positivity (positive / satisfactory smears) with 95% Wilson confidence intervals, 2018–2023, with the Cochran–Armitage trend statistic ($Z = 0.40$, $p = 0.69$).

Table S1. Summary of inferential analyses. Count-based comparisons use negative-binomial models fitted to the 30 quarterly counts, which were overdispersed (Pearson χ^2/df = 69.9; negative-binomial vs. Poisson AIC 523 vs. 2225). IRR, incidence-rate ratio; ITS, interrupted time-series; OR, odds ratio; n.s., not significant.

Comparison	Model / statistic	Estimate (95% CI)	p-value
Volume: pandemic (2020–2021) vs. pre-pandemic	Negative-binomial IRR	0.335 (0.177–0.634)	<0.001
Volume: pandemic — dispersion check	Quasi-Poisson IRR	0.335 (0.188–0.597)	<0.001

Comparison	Model / statistic	Estimate (95% CI)	p-value
Volume: immediate drop at lockdown onset	NB ITS level change	0.14 (0.09–0.24)	<0.001
Volume: recovery (2022–2023) vs. pre-pandemic	Negative-binomial IRR	0.614 (0.482–0.783)	<0.001
Volume: HPV era (2024–2025) vs. pre-pandemic	Negative-binomial IRR	0.898 (0.733–1.100)	0.30 (n.s.)
Positivity trend, 2018–2023 (per year)	Logistic OR, crude	1.01 (0.98–1.03)	0.69
Positivity trend, 2018–2023 (per year)	Logistic OR, age-adjusted	1.01 (0.98–1.03)	0.71
Positivity: pandemic vs. pre-pandemic	Fisher exact	OR 0.98 (n.s.)	0.82
HSIL detection-rate trend, 2018–2021	Poisson rate ratio/yr	0.98 (0.85–1.13)	0.78
HR-HPV positivity, 2024–2025	Proportion	22.8% (21.8–23.9)	—

Table S2. Sensitivity of the screening-volume incidence-rate ratio to the choice of baseline period and the dispersion model. NB, negative-binomial. "Reduction" is (1 − IRR) × 100.

Specification	IRR (95% CI)	Reduction
Primary—2018–2019 vs. 2020–2021 (Q1–Q3), NB	0.335 (0.177–0.634)	−66.5%
Sensitivity 1—baseline 2019 only, NB	0.310 (0.127–0.756)	−69.0%
Sensitivity 2—2019 vs. 2020 (complete years), NB	0.330 (0.106–1.030)	−67.0%
Sensitivity 3—quasi-Poisson (robust to distribution)	0.335 (0.188–0.597)	−66.5%

Table S3. Distribution of screened women by age group and programme era, 2018–2025. Percentages are of the 17,677 women with a recorded age group. [a]Age band was not recorded for 130 women screened in 2018, so the pre-pandemic column totals 6,217 against 6,347 tests performed in 2018–2019.

Age group (years)	Pre-pandemic (2018–2019)	Pandemic (2020–2021)	Recovery (2022–2023)	HPV-primary (2024–2025)	Total (%)
25–29	398	108	374	715	1,595 (9.0)
30–34	769	201	569	943	2,482 (14.0)
35–39	982	284	647	1,026	2,939 (16.6)
40–44	1,323	391	780	1,083	3,577 (20.2)
45–49	917	355	523	711	2,506 (14.2)
50–54	763	239	406	512	1,920 (10.9)
55–59	601	200	350	426	1,577 (8.9)
60–64	464	82	251	284	1,081 (6.1)
**Total**	6,217[a]	1,860	3,900	5,700	17,677

Table S4. Completed STROBE/RECORD reporting checklist. Items follow the RECORD statement (Benchimol et al., PLoS Med 2015;12(10):e1001885), an extension of the STROBE statement for observational studies (von Elm et al., Lancet 2007;370:1453–1457) to studies conducted using routinely collected health data. Section symbols (§) refer to numbered sections of the revised manuscript. "Not applicable" entries reflect that the study used a single-source, de-identified extract without inter-database linkage or code-based case selection.

Item No.	STROBE / RECORD item	Reported in manuscript
**Title and Abstract**		
1	STROBE: (a) Indicate the study's design with a commonly used term in the title or the abstract. (b) Provide in the abstract an informative and balanced summary of what was done and what was found.	Title; Abstract (structured Background / Methods / Results / Conclusions).
RECORD 1.1	The type of data used should be specified in the title or abstract. When possible, the name of the databases used should be included.	Abstract (Methods)—"de-identified screening-episode records"; database named in §2.2 (centre laboratory information system; quarterly programme returns, Anexa 8).
RECORD 1.2	If applicable, the geographic region and timeframe within which the study took place should be reported in the title or abstract.	Title and Abstract—Bihor County, north-western Romania; 2018–2025.
RECORD 1.3	If linkage between databases was conducted for the study, this should be clearly stated in the title or abstract.	Not applicable—no inter-database linkage; a single-source extract was reconciled against aggregate programme returns (§2.2).
**Introduction**		
2	STROBE: Explain the scientific background and rationale for the investigation being reported.	§1 Introduction.
3	STROBE: State specific objectives, including any pre-specified hypotheses.	§1 Introduction (final paragraph, aims i–iii); Abstract (Background).
**Methods**		
4	STROBE: Study design. Present key elements of study design early in the paper.	§2.1 Study Design and Setting; Abstract (Methods).
5	STROBE: Setting. Describe the setting, locations and relevant dates, including periods of recruitment, exposure, follow-up and data collection.	§2.1; §2.2 (1 January 2018–31 December 2025); §2.3 (four programme eras); §2.4 Screening Algorithms and Management Pathways.
6	STROBE: Participants. (a) Give the eligibility criteria and the sources and methods of selection of participants.	§2.2—all women screened at the centre were eligible; the sole exclusion was a documented prior cervical squamous cell carcinoma. Flow of screening episodes in Figure 1.
RECORD 6.1	The methods of study population selection (such as codes or algorithms used to identify subjects) should be listed in detail. If this is not possible, an explanation should be provided.	§2.2—dataset extracted from the centre's laboratory information system and reconciled against the signed quarterly returns (Anexa 8); all screened women were included, with no diagnostic codes or algorithms applied.
RECORD 6.2	Any validation studies of the codes or algorithms used to select the population should be referenced. If validation was conducted for this study and not	Not applicable—no codes or algorithms were used to select the population

Item No.	STROBE / RECORD item	Reported in manuscript
	published elsewhere, detailed methods and results should be provided.	(direct extraction of all screened women; §2.2).
RECORD 6.3	If the study involved linkage of databases, consider use of a flow diagram or other graphical display to demonstrate the data linkage process, including the number of individuals with linked data at each stage.	Not applicable—no database linkage. Flow of screening episodes is shown in Figure 1.
7	STROBE: Variables. Clearly define all outcomes, exposures, predictors, potential confounders and effect modifiers. Give diagnostic criteria, if applicable.	§2.6 Variables and Definitions; §2.5 Laboratory Methods (2014 Bethesda System for cytology; cobas HPV Test); §2.4 Screening Algorithms and Management Pathways.
RECORD 7.1	A complete list of codes and algorithms used to classify exposures, outcomes, confounders and effect modifiers should be provided. If these cannot be reported, an explanation should be provided.	§2.5–2.6—cytology classified by the 2014 Bethesda System; outcomes (screening activity relative to the registered catchment, cytological positivity, HR-HPV positivity, HSIL detection rate) defined explicitly. No administrative code lists were used.
8	STROBE: Data sources/measurement. For each variable of interest, give sources of data and details of methods of assessment (measurement). Describe comparability of assessment methods if there is more than one group.	§2.2 (data source); §2.5 (liquid-based cytology, Papanicolaou staining, cobas HR-HPV DNA testing); §2.6.
9	STROBE: Bias. Describe any efforts to address potential sources of bias.	§2.7 (negative-binomial models for overdispersion; pre-specified sensitivity analyses; age-adjusted logistic regression); §2.9 (data-integrity points); §4.6 (Limitations).
10	STROBE: Study size. Explain how the study size was arrived at.	§2.2—the analytic sample comprised all 17,807 screening episodes recorded during 2018–2025 (full census of attenders); no formal sample-size calculation.
11	STROBE: Quantitative variables. Explain how quantitative variables were handled in the analyses. If applicable, describe which groupings were chosen and why.	§2.6 (five-year age bands 25–29 to 60–64; calendar quarters); §2.7 (quarterly counts; 2021 annualised ×12/9).
12	STROBE: Statistical methods. (a) Describe all statistical methods, including those used to control for confounding; (b) methods to examine subgroups and interactions; (c) how missing data were addressed; (d) analytical approach; (e) sensitivity analyses.	§2.7 Statistical Analysis.
RECORD 12.1	Authors should describe the extent to which the investigators had access to the database population used to create the study population.	§2.2—investigators had access to the full de-identified centre dataset, reconciled with the coordinator-signed programme returns.
RECORD 12.2	Authors should provide information on the data cleaning methods used in the study.	§2.9 Reporting and Data Integrity (documented data-quality points; age band missing for 130 women in 2018); §2.7.

Item No.	STROBE / RECORD item	Reported in manuscript
RECORD 12.3	State whether the study included person-level, institutional-level, or other data linkage across two or more databases. The methods of linkage and methods of linkage quality evaluation should be provided.	Not applicable—no cross-database linkage; institution-level reconciliation of the extract against aggregate quarterly returns only (§2.2).
**Results**		
13	STROBE: Participants. (a) Report the numbers of individuals at each stage of the study; (b) give reasons for non-participation at each stage; (c) consider use of a flow diagram.	§3.1; Figure 1 (flow of screening episodes).
RECORD 13.1	Describe in detail the selection of the persons included in the study (i.e., study population selection) including filtering based on data quality, data availability and linkage. The selection of included persons can be described in the text and/or by means of the study flow diagram.	§2.2; §2.9; Figure 1.
14	STROBE: Descriptive data. (a) Give characteristics of study participants and information on exposures and potential confounders; (b) indicate the number of participants with missing data for each variable of interest.	§3.1, §3.6; Table 1; Table S3 (age distribution). Missing age band (130 women) noted in §2.9.
15	STROBE: Outcome data. Report numbers of outcome events or summary measures.	§3.1–3.6; Tables 1–2; Figures 2–5, Figures S1 and S4.
16	STROBE: Main results. (a) Give unadjusted and, where applicable, confounder-adjusted estimates and their precision (e.g., 95% CI); (b) report category boundaries when continuous variables were categorised; (c) translate estimates of relative risk into absolute risk where meaningful.	§3.2–3.5 (IRRs, ITS with 95% CI); age-adjusted OR in §3.5; per-woman risk ratio (RR 0.77) in §3.4; absolute pandemic deficit (~3,700 tests) in §3.3; Tables 1–2; Tables S1–S2.
17	STROBE: Other analyses. Report other analyses done—e.g., analyses of subgroups and interactions, and sensitivity analyses.	§3.3 (pre-specified sensitivity analyses of the IRR); age-adjusted positivity §3.5; Table S2; Figure S2.
**Discussion**		
18	STROBE: Key results. Summarise key results with reference to study objectives.	§4.1 Principal Findings.
19	STROBE: Limitations. Discuss limitations of the study, taking into account sources of potential bias or imprecision. Discuss both direction and magnitude of any potential bias.	§4.6 Strengths and Limitations.
RECORD 19.1	Discuss the implications of using data that were not created or collected to answer the specific research question(s). Include discussion of misclassification bias, unmeasured confounding, missing data, and changing eligibility over time, as they pertain to the study being reported.	§4.6—records represent screening episodes rather than uniquely identified women; no linkage to histology, treatment, or cancer-incidence outcomes; missing age band; registered-denominator caveats; non-comparability of cytological vs. HR-HPV positivity across the modality change; genotype-specific results not recorded.
20	STROBE: Interpretation. Give a cautious overall interpretation of results considering objectives, limitations, multiplicity of analyses, results from similar studies and other relevant evidence.	§4.2–4.5; §5 Conclusions.

Item No.	STROBE / RECORD item	Reported in manuscript
21	STROBE: Generalisability. Discuss the generalisability (external validity) of the study results.	§4.2 and §4.6 (monocentric design; single-provider registered-catchment denominator); §5.
**Other Information**		
22	STROBE: Funding. Give the source of funding and the role of the funders for the present study and, if applicable, for the original study on which the present article is based.	Funding statement ("This research received no external funding"); Acknowledgments.
RECORD 22.1	Authors should provide information on how to access any supplemental information such as the study protocol, raw data, or programming code.	Data Availability Statement and Supplementary Materials—de-identified individual-level raw data provided in Table S5; analysis software specified in §2.7 (Python 3.12, pandas 2.2, SciPy 1.13, statsmodels 0.14).

Table S5. Raw data. De-identified individual-level dataset of all 17,807 screening episodes analysed in this study (pseudonymous record ID; year, quarter and month of testing; programme period; primary screening test; five-year age band and age in years; HR-HPV result, where applicable; sample adequacy; cytological result with Bethesda category and grade; and concurrent infective findings). Owing to its size, Table S5 is provided as a separate supplementary file.
