# Supplementary material for: Cervical Cancer Screening Activity at an Accredited Centre in Romania, 2018–2025: COVID-19 Disruption, Recovery and the Transition to Primary HPV Testing
Source: Healthcare (Basel). 2026 Sep 4;14(17):2850. doi: 10.3390/healthcare14172850 (PMC13566146; doi:10.3390/healthcare14172850)
Supplement: Supplementary file 1 [file healthcare-14-02850-s001.zip › healthcare-4482925-supplementary Table S5.pdf]

Table S5. Raw data.

patient_id	year	quarter	month	period	primary_test	age_group	age_years	hpv_result	adequacy	result	bethesda	grade	infection
P00001	2018	2018-Q1	2018-01	Pre-pandemic	Cytology (Pap)	50-54	53	Not performed	Satisfactory	NILM	NILM	Negative (NILM)	
P00002	2018	2018-Q1	2018-01	Pre-pandemic	Cytology (Pap)	40-44	43	Not performed	Satisfactory	NILM	NILM	Negative (NILM)	
P00003	2018	2018-Q1	2018-01	Pre-pandemic	Cytology (Pap)	50-54	51	Not performed	Satisfactory	NILM	NILM	Negative (NILM)	
P00004	2018	2018-Q1	2018-01	Pre-pandemic	Cytology (Pap)	55-59	59	Not performed	Satisfactory	NILM	NILM	Negative (NILM)	
P00005	2018	2018-Q1	2018-01	Pre-pandemic	Cytology (Pap)	35-39	36	Not performed	Satisfactory	Positive	LSIL	Low-grade / equivocal squamous	
P00006	2018	2018-Q1	2018-01	Pre-pandemic	Cytology (Pap)	40-44	41	Not performed	Satisfactory	Positive	LSIL	Low-grade / equivocal squamous	
P00007	2018	2018-Q1	2018-01	Pre-pandemic	Cytology (Pap)	45-49	46	Not performed	Satisfactory	NILM	NILM	Negative (NILM)	
P00008	2018	2018-Q1	2018-01	Pre-pandemic	Cytology (Pap)	50-54	51	Not performed	Satisfactory	NILM	NILM	Negative (NILM)	
P00009	2018	2018-Q1	2018-01	Pre-pandemic	Cytology (Pap)	40-44	40	Not performed	Satisfactory	NILM	NILM	Negative (NILM)	
P00010	2018	2018-Q1	2018-01	Pre-pandemic	Cytology (Pap)	60-64	62	Not performed	Satisfactory	Positive	HSIL	High-grade squamous	
P00011	2018	2018-Q1	2018-01	Pre-pandemic	Cytology (Pap)	25-29	28	Not performed	Satisfactory	Positive	ASC-US	Low-grade / equivocal squamous	
P00012	2018	2018-Q1	2018-01	Pre-pandemic	Cytology (Pap)	30-34	34	Not performed	Satisfactory	Positive	LSIL	Low-grade / equivocal squamous	
P00013	2018	2018-Q1	2018-01	Pre-pandemic	Cytology (Pap)	40-44	44	Not performed	Satisfactory	NILM	NILM	Negative (NILM)	
P00014	2018	2018-Q1	2018-01	Pre-pandemic	Cytology (Pap)	40-44	40	Not performed	Satisfactory	NILM	NILM	Negative (NILM)	
P00015	2018	2018-Q1	2018-01	Pre-pandemic	Cytology (Pap)	Not recorded		Not performed	Satisfactory	Positive	ASC-US	Low-grade / equivocal squamous	
P00016	2018	2018-Q1	2018-01	Pre-pandemic	Cytology (Pap)	35-39	39	Not performed	Satisfactory	NILM	NILM	Negative (NILM)	
P00017	2018	2018-Q1	2018-01	Pre-pandemic	Cytology (Pap)	40-44	43	Not performed	Satisfactory	NILM	NILM	Negative (NILM)	
P00018	2018	2018-Q1	2018-01	Pre-pandemic	Cytology (Pap)	30-34	31	Not performed	Unsatisfactory	Unsatisfactory	Unsatisfactory	Unsatisfactory	
P00019	2018	2018-Q1	2018-01	Pre-pandemic	Cytology (Pap)	25-29	28	Not performed	Satisfactory	Positive	HSIL	High-grade squamous	
P00020	2018	2018-Q1	2018-01	Pre-pandemic	Cytology (Pap)	50-54	54	Not performed	Satisfactory	NILM	NILM	Negative (NILM)	
P00021	2018	2018-Q1	2018-01	Pre-pandemic	Cytology (Pap)	40-44	44	Not performed	Satisfactory	NILM	NILM	Negative (NILM)	
P00022	2018	2018-Q1	2018-01	Pre-pandemic	Cytology (Pap)	50-54	51	Not performed	Satisfactory	NILM	NILM	Negative (NILM)	
P00023	2018	2018-Q1	2018-01	Pre-pandemic	Cytology (Pap)	25-29	27	Not performed	Satisfactory	NILM	NILM	Negative (NILM)	
P00024	2018	2018-Q1	2018-01	Pre-pandemic	Cytology (Pap)	35-39	38	Not performed	Satisfactory	NILM	NILM	Negative (NILM)	
P00025	2018	2018-Q1	2018-01	Pre-pandemic	Cytology (Pap)	45-49	46	Not performed	Satisfactory	NILM	NILM	Negative (NILM)	
P00026	2018	2018-Q1	2018-01	Pre-pandemic	Cytology (Pap)	60-64	61	Not performed	Satisfactory	Positive	HSIL	High-grade squamous	
P00027	2018	2018-Q1	2018-01	Pre-pandemic	Cytology (Pap)	50-54	53	Not performed	Satisfactory	NILM	NILM	Negative (NILM)	
P00028	2018	2018-Q1	2018-01	Pre-pandemic	Cytology (Pap)	40-44	41	Not performed	Satisfactory	NILM	NILM	Negative (NILM)	
P00029	2018	2018-Q1	2018-01	Pre-pandemic	Cytology (Pap)	30-34	34	Not performed	Satisfactory	NILM	NILM	Negative (NILM)	
P00030	2018	2018-Q1	2018-01	Pre-pandemic	Cytology (Pap)	50-54	52	Not performed	Satisfactory	Positive	LSIL	Low-grade / equivocal squamous	
P00031	2018	2018-Q1	2018-01	Pre-pandemic	Cytology (Pap)	45-49	45	Not performed	Satisfactory	Positive	HSIL	High-grade squamous	
P00032	2018	2018-Q1	2018-01	Pre-pandemic	Cytology (Pap)	35-39	39	Not performed	Satisfactory	NILM	NILM	Negative (NILM)	
P00033	2018	2018-Q1	2018-01	Pre-pandemic	Cytology (Pap)	45-49	45	Not performed	Satisfactory	Positive	LSIL	Low-grade / equivocal squamous	
P00034	2018	2018-Q1	2018-01	Pre-pandemic	Cytology (Pap)	35-39	38	Not performed	Satisfactory	NILM	NILM	Negative (NILM)	Candida
P00035	2018	2018-Q1	2018-01	Pre-pandemic	Cytology (Pap)	50-54	52	Not performed	Unsatisfactory	Unsatisfactory	Unsatisfactory	Unsatisfactory	
P00036	2018	2018-Q1	2018-01	Pre-pandemic	Cytology (Pap)	30-34	33	Not performed	Satisfactory	NILM	NILM	Negative (NILM)	
P00037	2018	2018-Q1	2018-01	Pre-pandemic	Cytology (Pap)	30-34	30	Not performed	Satisfactory	NILM	NILM	Negative (NILM)	
P00038	2018	2018-Q1	2018-01	Pre-pandemic	Cytology (Pap)	40-44	42	Not performed	Satisfactory	NILM	NILM	Negative (NILM)	
P00039	2018	2018-Q1	2018-01	Pre-pandemic	Cytology (Pap)	45-49	49	Not performed	Satisfactory	NILM	NILM	Negative (NILM)	
P00040	2018	2018-Q1	2018-01	Pre-pandemic	Cytology (Pap)	30-34	30	Not performed	Satisfactory	Positive	LSIL	Low-grade / equivocal squamous	
P00041	2018	2018-Q1	2018-01	Pre-pandemic	Cytology (Pap)	30-34	33	Not performed	Satisfactory	NILM	NILM	Negative (NILM)	
P00042	2018	2018-Q1	2018-01	Pre-pandemic	Cytology (Pap)	35-39	36	Not performed	Satisfactory	NILM	NILM	Negative (NILM)	
P00043	2018	2018-Q1	2018-01	Pre-pandemic	Cytology (Pap)	Not recorded		Not performed	Satisfactory	NILM	NILM	Negative (NILM)	Candida
P00044	2018	2018-Q1	2018-01	Pre-pandemic	Cytology (Pap)	50-54	52	Not performed	Satisfactory	NILM	NILM	Negative (NILM)	
P00045	2018	2018-Q1	2018-01	Pre-pandemic	Cytology (Pap)	45-49	49	Not performed	Satisfactory	NILM	NILM	Negative (NILM)	
P00046	2018	2018-Q1	2018-01	Pre-pandemic	Cytology (Pap)	50-54	50	Not performed	Satisfactory	NILM	NILM	Negative (NILM)	
P00047	2018	2018-Q1	2018-01	Pre-pandemic	Cytology (Pap)	50-54	53	Not performed	Satisfactory	NILM	NILM	Negative (NILM)	
P00048	2018	2018-Q1	2018-01	Pre-pandemic	Cytology (Pap)	50-54	54	Not performed	Satisfactory	NILM	NILM	Negative (NILM)	
P00049	2018	2018-Q1	2018-01	Pre-pandemic	Cytology (Pap)	55-59	56	Not performed	Satisfactory	NILM	NILM	Negative (NILM)	
P00050	2018	2018-Q1	2018-01	Pre-pandemic	Cytology (Pap)	30-34	34	Not performed	Satisfactory	NILM	NILM	Negative (NILM)	
P00051	2018	2018-Q1	2018-01	Pre-pandemic	Cytology (Pap)	60-64	63	Not performed	Satisfactory	NILM	NILM	Negative (NILM)	
P00052	2018	2018-Q1	2018-01	Pre-pandemic	Cytology (Pap)	50-54	50	Not performed	Satisfactory	NILM	NILM	Negative (NILM)	
P00053	2018	2018-Q1	2018-01	Pre-pandemic	Cytology (Pap)	25-29	27	Not performed	Satisfactory	NILM	NILM	Negative (NILM)	Trichomonas
P00054	2018	2018-Q1	2018-01	Pre-pandemic	Cytology (Pap)	25-29	26	Not performed	Satisfactory	Positive	LSIL	Low-grade / equivocal squamous	
P00055	2018	2018-Q1	2018-01	Pre-pandemic	Cytology (Pap)	55-59	56	Not performed	Satisfactory	Positive	LSIL	Low-grade / equivocal squamous	
P00056	2018	2018-Q1	2018-01	Pre-pandemic	Cytology (Pap)	35-39	36	Not performed	Unsatisfactory	Unsatisfactory	Unsatisfactory	Unsatisfactory	
P00057	2018	2018-Q1	2018-01	Pre-pandemic	Cytology (Pap)	45-49	48	Not performed	Satisfactory	NILM	NILM	Negative (NILM)	
P00058	2018	2018-Q1	2018-01	Pre-pandemic	Cytology (Pap)	40-44	44	Not performed	Satisfactory	NILM	NILM	Negative (NILM)	
P00059	2018	2018-Q1	2018-01	Pre-pandemic	Cytology (Pap)	30-34	31	Not performed	Satisfactory	NILM	NILM	Negative (NILM)	
P00060	2018	2018-Q1	2018-01	Pre-pandemic	Cytology (Pap)	55-59	59	Not performed	Satisfactory	NILM	NILM	Negative (NILM)	
P00061	2018	2018-Q1	2018-01	Pre-pandemic	Cytology (Pap)	60-64	61	Not performed	Satisfactory	Positive	ASC-US	Low-grade / equivocal squamous	
P00062	2018	2018-Q1	2018-01	Pre-pandemic	Cytology (Pap)	40-44	40	Not performed	Satisfactory	NILM	NILM	Negative (NILM)	
P00063	2018	2018-Q1	2018-01	Pre-pandemic	Cytology (Pap)	50-54	50	Not performed	Satisfactory	NILM	NILM	Negative (NILM)	Candida
P00064	2018	2018-Q1	2018-01	Pre-pandemic	Cytology (Pap)	35-39	35	Not performed	Satisfactory	NILM	NILM	Negative (NILM)	
P00065	2018	2018-Q1	2018-01	Pre-pandemic	Cytology (Pap)	35-39	36	Not performed	Satisfactory	Positive	LSIL	Low-grade / equivocal squamous	
P00066	2018	2018-Q1	2018-01	Pre-pandemic	Cytology (Pap)	30-34	33	Not performed	Satisfactory	NILM	NILM	Negative (NILM)	Trichomonas
P00067	2018	2018-Q1	2018-01	Pre-pandemic	Cytology (Pap)	35-39	38	Not performed	Satisfactory	NILM	NILM	Negative (NILM)	
P00068	2018	2018-Q1	2018-01	Pre-pandemic	Cytology (Pap)	50-54	50	Not performed	Satisfactory	NILM	NILM	Negative (NILM)	
P00069	2018	2018-Q1	2018-01	Pre-pandemic	Cytology (Pap)	40-44	43	Not performed	Satisfactory	NILM	NILM	Negative (NILM)	
P00070	2018	2018-Q1	2018-01	Pre-pandemic	Cytology (Pap)	30-34	30	Not performed	Satisfactory	NILM	NILM	Negative (NILM)	
P00071	2018	2018-Q1	2018-01	Pre-pandemic	Cytology (Pap)	45-49	46	Not performed	Satisfactory	NILM	NILM	Negative (NILM)	
P00072	2018	2018-Q1	2018-01	Pre-pandemic	Cytology (Pap)	60-64	62	Not performed	Satisfactory	NILM	NILM	Negative (NILM)	
P00073	2018	2018-Q1	2018-01	Pre-pandemic	Cytology (Pap)	40-44	44	Not performed	Satisfactory	Positive	LSIL	Low-grade / equivocal squamous	
P00074	2018	2018-Q1	2018-01	Pre-pandemic	Cytology (Pap)	45-49	45	Not performed	Satisfactory	Positive	ASC-US	Low-grade / equivocal squamous	Gardnerella
P00075	2018	2018-Q1	2018-01	Pre-pandemic	Cytology (Pap)	60-64	63	Not performed	Satisfactory	NILM	NILM	Negative (NILM)	
P00076	2018	2018-Q1	2018-01	Pre-pandemic	Cytology (Pap)	Not recorded		Not performed	Satisfactory	NILM	NILM	Negative (NILM)	
P00077	2018	2018-Q1	2018-01	Pre-pandemic	Cytology (Pap)	45-49	47	Not performed	Satisfactory	NILM	NILM	Negative (NILM)	Other
P00078	2018	2018-Q1	2018-01	Pre-pandemic	Cytology (Pap)	50-54	51	Not performed	Satisfactory	NILM	NILM	Negative (NILM)	

patient_id	year	quarter	month	period	primary_test	age_group	age_years	hpv_result	adequacy	result	bethesda	grade	infection
P00079	2018	2018-Q1	2018-01	Pre-pandemic	Cytology (Pap)	40-44	44	Not performed	Satisfactory	NILM	NILM	Negative (NILM)	
P00080	2018	2018-Q1	2018-01	Pre-pandemic	Cytology (Pap)	55-59	58	Not performed	Satisfactory	NILM	NILM	Negative (NILM)	
P00081	2018	2018-Q1	2018-01	Pre-pandemic	Cytology (Pap)	50-54	51	Not performed	Satisfactory	Positive	AGC	Glandular	Trichomonas
P00082	2018	2018-Q1	2018-01	Pre-pandemic	Cytology (Pap)	55-59	55	Not performed	Satisfactory	NILM	NILM	Negative (NILM)	
P00083	2018	2018-Q1	2018-01	Pre-pandemic	Cytology (Pap)	50-54	51	Not performed	Satisfactory	NILM	NILM	Negative (NILM)	Trichomonas
P00084	2018	2018-Q1	2018-01	Pre-pandemic	Cytology (Pap)	35-39	38	Not performed	Satisfactory	NILM	NILM	Negative (NILM)	
P00085	2018	2018-Q1	2018-01	Pre-pandemic	Cytology (Pap)	35-39	37	Not performed	Satisfactory	NILM	NILM	Negative (NILM)	
P00086	2018	2018-Q1	2018-01	Pre-pandemic	Cytology (Pap)	45-49	49	Not performed	Satisfactory	NILM	NILM	Negative (NILM)	
P00087	2018	2018-Q1	2018-01	Pre-pandemic	Cytology (Pap)	30-34	33	Not performed	Satisfactory	NILM	NILM	Negative (NILM)	
P00088	2018	2018-Q1	2018-01	Pre-pandemic	Cytology (Pap)	45-49	46	Not performed	Satisfactory	NILM	NILM	Negative (NILM)	
P00089	2018	2018-Q1	2018-01	Pre-pandemic	Cytology (Pap)	30-34	32	Not performed	Satisfactory	Positive	LSIL	Low-grade / equivocal squamous	
P00090	2018	2018-Q1	2018-01	Pre-pandemic	Cytology (Pap)	35-39	38	Not performed	Satisfactory	NILM	NILM	Negative (NILM)	
P00091	2018	2018-Q1	2018-01	Pre-pandemic	Cytology (Pap)	25-29	25	Not performed	Satisfactory	NILM	NILM	Negative (NILM)	
P00092	2018	2018-Q1	2018-01	Pre-pandemic	Cytology (Pap)	25-29	26	Not performed	Satisfactory	NILM	NILM	Negative (NILM)	
P00093	2018	2018-Q1	2018-01	Pre-pandemic	Cytology (Pap)	55-59	59	Not performed	Satisfactory	NILM	NILM	Negative (NILM)	
P00094	2018	2018-Q1	2018-01	Pre-pandemic	Cytology (Pap)	Not recorded		Not performed	Satisfactory	Positive	ASC-US	Low-grade / equivocal squamous	
P00095	2018	2018-Q1	2018-01	Pre-pandemic	Cytology (Pap)	40-44	41	Not performed	Satisfactory	NILM	NILM	Negative (NILM)	
P00096	2018	2018-Q1	2018-01	Pre-pandemic	Cytology (Pap)	30-34	34	Not performed	Satisfactory	NILM	NILM	Negative (NILM)	
P00097	2018	2018-Q1	2018-01	Pre-pandemic	Cytology (Pap)	35-39	38	Not performed	Unsatisfactory	Unsatisfactory	Unsatisfactory	Unsatisfactory	
P00098	2018	2018-Q1	2018-01	Pre-pandemic	Cytology (Pap)	40-44	40	Not performed	Satisfactory	NILM	NILM	Negative (NILM)	
P00099	2018	2018-Q1	2018-01	Pre-pandemic	Cytology (Pap)	40-44	40	Not performed	Satisfactory	Positive	LSIL	Low-grade / equivocal squamous	
P00100	2018	2018-Q1	2018-01	Pre-pandemic	Cytology (Pap)	55-59	55	Not performed	Satisfactory	NILM	NILM	Negative (NILM)	
P00101	2018	2018-Q1	2018-01	Pre-pandemic	Cytology (Pap)	50-54	51	Not performed	Satisfactory	NILM	NILM	Negative (NILM)	
P00102	2018	2018-Q1	2018-01	Pre-pandemic	Cytology (Pap)	45-49	47	Not performed	Satisfactory	NILM	NILM	Negative (NILM)	
P00103	2018	2018-Q1	2018-01	Pre-pandemic	Cytology (Pap)	50-54	51	Not performed	Satisfactory	NILM	NILM	Negative (NILM)	
P00104	2018	2018-Q1	2018-01	Pre-pandemic	Cytology (Pap)	30-34	31	Not performed	Satisfactory	NILM	NILM	Negative (NILM)	
P00105	2018	2018-Q1	2018-01	Pre-pandemic	Cytology (Pap)	40-44	43	Not performed	Satisfactory	NILM	NILM	Negative (NILM)	
P00106	2018	2018-Q1	2018-01	Pre-pandemic	Cytology (Pap)	55-59	56	Not performed	Satisfactory	Positive	ASC-US	Low-grade / equivocal squamous	
P00107	2018	2018-Q1	2018-01	Pre-pandemic	Cytology (Pap)	45-49	46	Not performed	Satisfactory	NILM	NILM	Negative (NILM)	
P00108	2018	2018-Q1	2018-01	Pre-pandemic	Cytology (Pap)	60-64	64	Not performed	Satisfactory	NILM	NILM	Negative (NILM)	
P00109	2018	2018-Q1	2018-01	Pre-pandemic	Cytology (Pap)	30-34	32	Not performed	Satisfactory	NILM	NILM	Negative (NILM)	
P00110	2018	2018-Q1	2018-01	Pre-pandemic	Cytology (Pap)	60-64	63	Not performed	Satisfactory	NILM	NILM	Negative (NILM)	
P00111	2018	2018-Q1	2018-01	Pre-pandemic	Cytology (Pap)	25-29	26	Not performed	Satisfactory	NILM	NILM	Negative (NILM)	
P00112	2018	2018-Q1	2018-01	Pre-pandemic	Cytology (Pap)	35-39	37	Not performed	Satisfactory	NILM	NILM	Negative (NILM)	
P00113	2018	2018-Q1	2018-02	Pre-pandemic	Cytology (Pap)	40-44	40	Not performed	Satisfactory	NILM	NILM	Negative (NILM)	
P00114	2018	2018-Q1	2018-02	Pre-pandemic	Cytology (Pap)	35-39	37	Not performed	Satisfactory	NILM	NILM	Negative (NILM)	
P00115	2018	2018-Q1	2018-02	Pre-pandemic	Cytology (Pap)	25-29	26	Not performed	Unsatisfactory	Unsatisfactory	Unsatisfactory	Unsatisfactory	
P00116	2018	2018-Q1	2018-02	Pre-pandemic	Cytology (Pap)	50-54	50	Not performed	Satisfactory	NILM	NILM	Negative (NILM)	
P00117	2018	2018-Q1	2018-02	Pre-pandemic	Cytology (Pap)	60-64	64	Not performed	Satisfactory	NILM	NILM	Negative (NILM)	
P00118	2018	2018-Q1	2018-02	Pre-pandemic	Cytology (Pap)	Not recorded		Not performed	Satisfactory	Positive	HSIL	High-grade squamous	
P00119	2018	2018-Q1	2018-02	Pre-pandemic	Cytology (Pap)	40-44	42	Not performed	Satisfactory	NILM	NILM	Negative (NILM)	
P00120	2018	2018-Q1	2018-02	Pre-pandemic	Cytology (Pap)	50-54	50	Not performed	Satisfactory	NILM	NILM	Negative (NILM)	
P00121	2018	2018-Q1	2018-02	Pre-pandemic	Cytology (Pap)	35-39	39	Not performed	Satisfactory	NILM	NILM	Negative (NILM)	
P00122	2018	2018-Q1	2018-02	Pre-pandemic	Cytology (Pap)	45-49	45	Not performed	Satisfactory	Positive	LSIL	Low-grade / equivocal squamous	
P00123	2018	2018-Q1	2018-02	Pre-pandemic	Cytology (Pap)	35-39	37	Not performed	Satisfactory	NILM	NILM	Negative (NILM)	
P00124	2018	2018-Q1	2018-02	Pre-pandemic	Cytology (Pap)	45-49	46	Not performed	Satisfactory	NILM	NILM	Negative (NILM)	
P00125	2018	2018-Q1	2018-02	Pre-pandemic	Cytology (Pap)	30-34	33	Not performed	Satisfactory	NILM	NILM	Negative (NILM)	
P00126	2018	2018-Q1	2018-02	Pre-pandemic	Cytology (Pap)	35-39	38	Not performed	Satisfactory	NILM	NILM	Negative (NILM)	
P00127	2018	2018-Q1	2018-02	Pre-pandemic	Cytology (Pap)	40-44	42	Not performed	Satisfactory	Positive	LSIL	Low-grade / equivocal squamous	
P00128	2018	2018-Q1	2018-02	Pre-pandemic	Cytology (Pap)	25-29	28	Not performed	Satisfactory	NILM	NILM	Negative (NILM)	
P00129	2018	2018-Q1	2018-02	Pre-pandemic	Cytology (Pap)	55-59	58	Not performed	Satisfactory	NILM	NILM	Negative (NILM)	
P00130	2018	2018-Q1	2018-02	Pre-pandemic	Cytology (Pap)	45-49	48	Not performed	Satisfactory	NILM	NILM	Negative (NILM)	
P00131	2018	2018-Q1	2018-02	Pre-pandemic	Cytology (Pap)	45-49	47	Not performed	Satisfactory	NILM	NILM	Negative (NILM)	Candida
P00132	2018	2018-Q1	2018-02	Pre-pandemic	Cytology (Pap)	55-59	59	Not performed	Satisfactory	NILM	NILM	Negative (NILM)	
P00133	2018	2018-Q1	2018-02	Pre-pandemic	Cytology (Pap)	40-44	40	Not performed	Satisfactory	NILM	NILM	Negative (NILM)	
P00134	2018	2018-Q1	2018-02	Pre-pandemic	Cytology (Pap)	30-34	31	Not performed	Satisfactory	NILM	NILM	Negative (NILM)	
P00135	2018	2018-Q1	2018-02	Pre-pandemic	Cytology (Pap)	40-44	40	Not performed	Satisfactory	NILM	NILM	Negative (NILM)	
P00136	2018	2018-Q1	2018-02	Pre-pandemic	Cytology (Pap)	60-64	62	Not performed	Satisfactory	NILM	NILM	Negative (NILM)	
P00137	2018	2018-Q1	2018-02	Pre-pandemic	Cytology (Pap)	40-44	43	Not performed	Satisfactory	Positive	ASC-US	Low-grade / equivocal squamous	
P00138	2018	2018-Q1	2018-02	Pre-pandemic	Cytology (Pap)	35-39	38	Not performed	Satisfactory	NILM	NILM	Negative (NILM)	
P00139	2018	2018-Q1	2018-02	Pre-pandemic	Cytology (Pap)	40-44	41	Not performed	Satisfactory	NILM	NILM	Negative (NILM)	
P00140	2018	2018-Q1	2018-02	Pre-pandemic	Cytology (Pap)	35-39	37	Not performed	Satisfactory	NILM	NILM	Negative (NILM)	
P00141	2018	2018-Q1	2018-02	Pre-pandemic	Cytology (Pap)	30-34	31	Not performed	Satisfactory	NILM	NILM	Negative (NILM)	
P00142	2018	2018-Q1	2018-02	Pre-pandemic	Cytology (Pap)	25-29	27	Not performed	Satisfactory	NILM	NILM	Negative (NILM)	
P00143	2018	2018-Q1	2018-02	Pre-pandemic	Cytology (Pap)	40-44	40	Not performed	Satisfactory	NILM	NILM	Negative (NILM)	
P00144	2018	2018-Q1	2018-02	Pre-pandemic	Cytology (Pap)	30-34	32	Not performed	Satisfactory	Positive	LSIL	Low-grade / equivocal squamous	
P00145	2018	2018-Q1	2018-02	Pre-pandemic	Cytology (Pap)	30-34	31	Not performed	Satisfactory	Positive	ASC-US	Low-grade / equivocal squamous	
P00146	2018	2018-Q1	2018-02	Pre-pandemic	Cytology (Pap)	30-34	31	Not performed	Satisfactory	NILM	NILM	Negative (NILM)	
P00147	2018	2018-Q1	2018-02	Pre-pandemic	Cytology (Pap)	50-54	53	Not performed	Satisfactory	NILM	NILM	Negative (NILM)	
P00148	2018	2018-Q1	2018-02	Pre-pandemic	Cytology (Pap)	40-44	42	Not performed	Satisfactory	NILM	NILM	Negative (NILM)	
P00149	2018	2018-Q1	2018-02	Pre-pandemic	Cytology (Pap)	40-44	43	Not performed	Satisfactory	Positive	ASC-US	Low-grade / equivocal squamous	
P00150	2018	2018-Q1	2018-02	Pre-pandemic	Cytology (Pap)	35-39	35	Not performed	Satisfactory	NILM	NILM	Negative (NILM)	
P00151	2018	2018-Q1	2018-02	Pre-pandemic	Cytology (Pap)	55-59	58	Not performed	Satisfactory	NILM	NILM	Negative (NILM)	
P00152	2018	2018-Q1	2018-02	Pre-pandemic	Cytology (Pap)	40-44	43	Not performed	Satisfactory	NILM	NILM	Negative (NILM)	
P00153	2018	2018-Q1	2018-02	Pre-pandemic	Cytology (Pap)	50-54	52	Not performed	Satisfactory	NILM	NILM	Negative (NILM)	
P00154	2018	2018-Q1	2018-02	Pre-pandemic	Cytology (Pap)	35-39	35	Not performed	Satisfactory	NILM	NILM	Negative (NILM)	
P00155	2018	2018-Q1	2018-02	Pre-pandemic	Cytology (Pap)	40-44	44	Not performed	Satisfactory	NILM	NILM	Negative (NILM)	
P00156	2018	2018-Q1	2018-02	Pre-pandemic	Cytology (Pap)	40-44	42	Not performed	Satisfactory	NILM	NILM	Negative (NILM)	
P00157	2018	2018-Q1	2018-02	Pre-pandemic	Cytology (Pap)	60-64	62	Not performed	Satisfactory	NILM	NILM	Negative (NILM)	
P00158	2018	2018-Q1	2018-02	Pre-pandemic	Cytology (Pap)	55-59	58	Not performed	Satisfactory	NILM	NILM	Negative (NILM)	

patient_id	year	quarter	month	period	primary_test	age_group	age_years	hpv_result	adequacy	result	bethesda	grade	infection
P00159	2018	2018-Q1	2018-02	Pre-pandemic	Cytology (Pap)	50-54	54	Not performed	Satisfactory	NILM	NILM	Negative (NILM)	
P00160	2018	2018-Q1	2018-02	Pre-pandemic	Cytology (Pap)	50-54	51	Not performed	Satisfactory	Positive	ASC-US	Low-grade / equivocal squamous	
P00161	2018	2018-Q1	2018-02	Pre-pandemic	Cytology (Pap)	40-44	42	Not performed	Satisfactory	NILM	NILM	Negative (NILM)	
P00162	2018	2018-Q1	2018-02	Pre-pandemic	Cytology (Pap)	35-39	38	Not performed	Satisfactory	NILM	NILM	Negative (NILM)	
P00163	2018	2018-Q1	2018-02	Pre-pandemic	Cytology (Pap)	55-59	57	Not performed	Satisfactory	NILM	NILM	Negative (NILM)	
P00164	2018	2018-Q1	2018-02	Pre-pandemic	Cytology (Pap)	35-39	38	Not performed	Satisfactory	NILM	NILM	Negative (NILM)	
P00165	2018	2018-Q1	2018-02	Pre-pandemic	Cytology (Pap)	Not recorded		Not performed	Satisfactory	NILM	NILM	Negative (NILM)	
P00166	2018	2018-Q1	2018-02	Pre-pandemic	Cytology (Pap)	40-44	44	Not performed	Satisfactory	NILM	NILM	Negative (NILM)	
P00167	2018	2018-Q1	2018-02	Pre-pandemic	Cytology (Pap)	25-29	26	Not performed	Satisfactory	NILM	NILM	Negative (NILM)	
P00168	2018	2018-Q1	2018-02	Pre-pandemic	Cytology (Pap)	40-44	42	Not performed	Satisfactory	NILM	NILM	Negative (NILM)	
P00169	2018	2018-Q1	2018-02	Pre-pandemic	Cytology (Pap)	45-49	48	Not performed	Satisfactory	NILM	NILM	Negative (NILM)	
P00170	2018	2018-Q1	2018-02	Pre-pandemic	Cytology (Pap)	30-34	34	Not performed	Satisfactory	NILM	NILM	Negative (NILM)	
P00171	2018	2018-Q1	2018-02	Pre-pandemic	Cytology (Pap)	35-39	39	Not performed	Satisfactory	NILM	NILM	Negative (NILM)	
P00172	2018	2018-Q1	2018-02	Pre-pandemic	Cytology (Pap)	35-39	38	Not performed	Satisfactory	NILM	NILM	Negative (NILM)	
P00173	2018	2018-Q1	2018-02	Pre-pandemic	Cytology (Pap)	25-29	26	Not performed	Satisfactory	NILM	NILM	Negative (NILM)	Trichomonas
P00174	2018	2018-Q1	2018-02	Pre-pandemic	Cytology (Pap)	40-44	43	Not performed	Satisfactory	NILM	NILM	Negative (NILM)	
P00175	2018	2018-Q1	2018-02	Pre-pandemic	Cytology (Pap)	40-44	42	Not performed	Satisfactory	NILM	NILM	Negative (NILM)	
P00176	2018	2018-Q1	2018-02	Pre-pandemic	Cytology (Pap)	40-44	41	Not performed	Satisfactory	Positive	ASC-US	Low-grade / equivocal squamous	
P00177	2018	2018-Q1	2018-02	Pre-pandemic	Cytology (Pap)	35-39	37	Not performed	Satisfactory	NILM	NILM	Negative (NILM)	
P00178	2018	2018-Q1	2018-02	Pre-pandemic	Cytology (Pap)	30-34	30	Not performed	Satisfactory	NILM	NILM	Negative (NILM)	
P00179	2018	2018-Q1	2018-02	Pre-pandemic	Cytology (Pap)	30-34	32	Not performed	Satisfactory	NILM	NILM	Negative (NILM)	
P00180	2018	2018-Q1	2018-02	Pre-pandemic	Cytology (Pap)	35-39	37	Not performed	Satisfactory	NILM	NILM	Negative (NILM)	
P00181	2018	2018-Q1	2018-02	Pre-pandemic	Cytology (Pap)	60-64	61	Not performed	Satisfactory	NILM	NILM	Negative (NILM)	
P00182	2018	2018-Q1	2018-02	Pre-pandemic	Cytology (Pap)	25-29	26	Not performed	Unsatisfactory	Unsatisfactory	Unsatisfactory	Unsatisfactory	
P00183	2018	2018-Q1	2018-02	Pre-pandemic	Cytology (Pap)	40-44	42	Not performed	Satisfactory	NILM	NILM	Negative (NILM)	
P00184	2018	2018-Q1	2018-02	Pre-pandemic	Cytology (Pap)	40-44	43	Not performed	Satisfactory	NILM	NILM	Negative (NILM)	
P00185	2018	2018-Q1	2018-02	Pre-pandemic	Cytology (Pap)	50-54	51	Not performed	Satisfactory	NILM	NILM	Negative (NILM)	
P00186	2018	2018-Q1	2018-02	Pre-pandemic	Cytology (Pap)	45-49	48	Not performed	Satisfactory	Positive	ASC-US	Low-grade / equivocal squamous	
P00187	2018	2018-Q1	2018-02	Pre-pandemic	Cytology (Pap)	30-34	32	Not performed	Satisfactory	NILM	NILM	Negative (NILM)	
P00188	2018	2018-Q1	2018-02	Pre-pandemic	Cytology (Pap)	35-39	38	Not performed	Satisfactory	NILM	NILM	Negative (NILM)	
P00189	2018	2018-Q1	2018-02	Pre-pandemic	Cytology (Pap)	35-39	35	Not performed	Satisfactory	NILM	NILM	Negative (NILM)	
P00190	2018	2018-Q1	2018-02	Pre-pandemic	Cytology (Pap)	50-54	50	Not performed	Satisfactory	NILM	NILM	Negative (NILM)	
P00191	2018	2018-Q1	2018-02	Pre-pandemic	Cytology (Pap)	35-39	39	Not performed	Satisfactory	Positive	ASC-US	Low-grade / equivocal squamous	
P00192	2018	2018-Q1	2018-02	Pre-pandemic	Cytology (Pap)	35-39	39	Not performed	Satisfactory	NILM	NILM	Negative (NILM)	
P00193	2018	2018-Q1	2018-02	Pre-pandemic	Cytology (Pap)	60-64	61	Not performed	Satisfactory	NILM	NILM	Negative (NILM)	
P00194	2018	2018-Q1	2018-02	Pre-pandemic	Cytology (Pap)	35-39	35	Not performed	Satisfactory	NILM	NILM	Negative (NILM)	
P00195	2018	2018-Q1	2018-02	Pre-pandemic	Cytology (Pap)	35-39	39	Not performed	Satisfactory	NILM	NILM	Negative (NILM)	
P00196	2018	2018-Q1	2018-02	Pre-pandemic	Cytology (Pap)	35-39	37	Not performed	Satisfactory	NILM	NILM	Negative (NILM)	
P00197	2018	2018-Q1	2018-02	Pre-pandemic	Cytology (Pap)	40-44	41	Not performed	Satisfactory	Positive	AGC	Glandular	
P00198	2018	2018-Q1	2018-02	Pre-pandemic	Cytology (Pap)	35-39	39	Not performed	Satisfactory	NILM	NILM	Negative (NILM)	
P00199	2018	2018-Q1	2018-02	Pre-pandemic	Cytology (Pap)	50-54	53	Not performed	Satisfactory	NILM	NILM	Negative (NILM)	Candida
P00200	2018	2018-Q1	2018-02	Pre-pandemic	Cytology (Pap)	40-44	41	Not performed	Unsatisfactory	Unsatisfactory	Unsatisfactory	Unsatisfactory	
P00201	2018	2018-Q1	2018-02	Pre-pandemic	Cytology (Pap)	30-34	32	Not performed	Satisfactory	NILM	NILM	Negative (NILM)	
P00202	2018	2018-Q1	2018-02	Pre-pandemic	Cytology (Pap)	45-49	48	Not performed	Satisfactory	Positive	LSIL	Low-grade / equivocal squamous	
P00203	2018	2018-Q1	2018-02	Pre-pandemic	Cytology (Pap)	40-44	43	Not performed	Satisfactory	NILM	NILM	Negative (NILM)	
P00204	2018	2018-Q1	2018-02	Pre-pandemic	Cytology (Pap)	50-54	54	Not performed	Satisfactory	NILM	NILM	Negative (NILM)	
P00205	2018	2018-Q1	2018-02	Pre-pandemic	Cytology (Pap)	50-54	54	Not performed	Satisfactory	NILM	NILM	Negative (NILM)	
P00206	2018	2018-Q1	2018-02	Pre-pandemic	Cytology (Pap)	60-64	64	Not performed	Satisfactory	NILM	NILM	Negative (NILM)	
P00207	2018	2018-Q1	2018-02	Pre-pandemic	Cytology (Pap)	30-34	34	Not performed	Satisfactory	NILM	NILM	Negative (NILM)	
P00208	2018	2018-Q1	2018-02	Pre-pandemic	Cytology (Pap)	45-49	47	Not performed	Satisfactory	NILM	NILM	Negative (NILM)	
P00209	2018	2018-Q1	2018-02	Pre-pandemic	Cytology (Pap)	50-54	51	Not performed	Satisfactory	Positive	ASC-US	Low-grade / equivocal squamous	
P00210	2018	2018-Q1	2018-02	Pre-pandemic	Cytology (Pap)	40-44	40	Not performed	Satisfactory	NILM	NILM	Negative (NILM)	
P00211	2018	2018-Q1	2018-02	Pre-pandemic	Cytology (Pap)	60-64	60	Not performed	Satisfactory	NILM	NILM	Negative (NILM)	
P00212	2018	2018-Q1	2018-02	Pre-pandemic	Cytology (Pap)	50-54	52	Not performed	Satisfactory	NILM	NILM	Negative (NILM)	
P00213	2018	2018-Q1	2018-02	Pre-pandemic	Cytology (Pap)	40-44	40	Not performed	Satisfactory	NILM	NILM	Negative (NILM)	
P00214	2018	2018-Q1	2018-02	Pre-pandemic	Cytology (Pap)	45-49	49	Not performed	Satisfactory	Positive	HSIL	High-grade squamous	
P00215	2018	2018-Q1	2018-02	Pre-pandemic	Cytology (Pap)	25-29	25	Not performed	Satisfactory	NILM	NILM	Negative (NILM)	
P00216	2018	2018-Q1	2018-02	Pre-pandemic	Cytology (Pap)	40-44	40	Not performed	Satisfactory	NILM	NILM	Negative (NILM)	
P00217	2018	2018-Q1	2018-02	Pre-pandemic	Cytology (Pap)	40-44	43	Not performed	Satisfactory	NILM	NILM	Negative (NILM)	
P00218	2018	2018-Q1	2018-02	Pre-pandemic	Cytology (Pap)	25-29	27	Not performed	Satisfactory	NILM	NILM	Negative (NILM)	
P00219	2018	2018-Q1	2018-02	Pre-pandemic	Cytology (Pap)	40-44	42	Not performed	Satisfactory	NILM	NILM	Negative (NILM)	
P00220	2018	2018-Q1	2018-02	Pre-pandemic	Cytology (Pap)	55-59	59	Not performed	Unsatisfactory	Unsatisfactory	Unsatisfactory	Unsatisfactory	
P00221	2018	2018-Q1	2018-02	Pre-pandemic	Cytology (Pap)	60-64	64	Not performed	Satisfactory	NILM	NILM	Negative (NILM)	
P00222	2018	2018-Q1	2018-02	Pre-pandemic	Cytology (Pap)	35-39	39	Not performed	Satisfactory	NILM	NILM	Negative (NILM)	Candida
P00223	2018	2018-Q1	2018-02	Pre-pandemic	Cytology (Pap)	40-44	42	Not performed	Satisfactory	Positive	ASC-US	Low-grade / equivocal squamous	
P00224	2018	2018-Q1	2018-02	Pre-pandemic	Cytology (Pap)	35-39	36	Not performed	Satisfactory	NILM	NILM	Negative (NILM)	
P00225	2018	2018-Q1	2018-03	Pre-pandemic	Cytology (Pap)	35-39	35	Not performed	Satisfactory	NILM	NILM	Negative (NILM)	
P00226	2018	2018-Q1	2018-03	Pre-pandemic	Cytology (Pap)	60-64	63	Not performed	Satisfactory	NILM	NILM	Negative (NILM)	
P00227	2018	2018-Q1	2018-03	Pre-pandemic	Cytology (Pap)	45-49	48	Not performed	Satisfactory	NILM	NILM	Negative (NILM)	
P00228	2018	2018-Q1	2018-03	Pre-pandemic	Cytology (Pap)	35-39	36	Not performed	Satisfactory	NILM	NILM	Negative (NILM)	
P00229	2018	2018-Q1	2018-03	Pre-pandemic	Cytology (Pap)	35-39	37	Not performed	Satisfactory	Positive	ASC-US	Low-grade / equivocal squamous	
P00230	2018	2018-Q1	2018-03	Pre-pandemic	Cytology (Pap)	55-59	58	Not performed	Satisfactory	NILM	NILM	Negative (NILM)	
P00231	2018	2018-Q1	2018-03	Pre-pandemic	Cytology (Pap)	45-49	47	Not performed	Satisfactory	Positive	LSIL	Low-grade / equivocal squamous	
P00232	2018	2018-Q1	2018-03	Pre-pandemic	Cytology (Pap)	35-39	36	Not performed	Satisfactory	NILM	NILM	Negative (NILM)	
P00233	2018	2018-Q1	2018-03	Pre-pandemic	Cytology (Pap)	Not recorded		Not performed	Satisfactory	NILM	NILM	Negative (NILM)	
P00234	2018	2018-Q1	2018-03	Pre-pandemic	Cytology (Pap)	25-29	26	Not performed	Satisfactory	NILM	NILM	Negative (NILM)	
P00235	2018	2018-Q1	2018-03	Pre-pandemic	Cytology (Pap)	30-34	33	Not performed	Unsatisfactory	Unsatisfactory	Unsatisfactory	Unsatisfactory	
P00236	2018	2018-Q1	2018-03	Pre-pandemic	Cytology (Pap)	35-39	38	Not performed	Satisfactory	NILM	NILM	Negative (NILM)	
P00237	2018	2018-Q1	2018-03	Pre-pandemic	Cytology (Pap)	55-59	58	Not performed	Satisfactory	Positive	LSIL	Low-grade / equivocal squamous	
P00238	2018	2018-Q1	2018-03	Pre-pandemic	Cytology (Pap)	Not recorded		Not performed	Satisfactory	Positive	ASC-US	Low-grade / equivocal squamous	

patient_id	year	quarter	month	period	primary_test	age_group	age_years	hpv_result	adequacy	result	bethesda	grade	infection
P00239	2018	2018-Q1	2018-03	Pre-pandemic	Cytology (Pap)	Not recorded		Not performed	Satisfactory	NILM	NILM	Negative (NILM)	
P00240	2018	2018-Q1	2018-03	Pre-pandemic	Cytology (Pap)	25-29	29	Not performed	Satisfactory	NILM	NILM	Negative (NILM)	
P00241	2018	2018-Q1	2018-03	Pre-pandemic	Cytology (Pap)	30-34	31	Not performed	Satisfactory	NILM	NILM	Negative (NILM)	
P00242	2018	2018-Q1	2018-03	Pre-pandemic	Cytology (Pap)	40-44	42	Not performed	Satisfactory	NILM	NILM	Negative (NILM)	
P00243	2018	2018-Q1	2018-03	Pre-pandemic	Cytology (Pap)	35-39	37	Not performed	Satisfactory	NILM	NILM	Negative (NILM)	
P00244	2018	2018-Q1	2018-03	Pre-pandemic	Cytology (Pap)	55-59	56	Not performed	Satisfactory	NILM	NILM	Negative (NILM)	
P00245	2018	2018-Q1	2018-03	Pre-pandemic	Cytology (Pap)	30-34	32	Not performed	Satisfactory	NILM	NILM	Negative (NILM)	
P00246	2018	2018-Q1	2018-03	Pre-pandemic	Cytology (Pap)	30-34	33	Not performed	Satisfactory	NILM	NILM	Negative (NILM)	
P00247	2018	2018-Q1	2018-03	Pre-pandemic	Cytology (Pap)	40-44	44	Not performed	Satisfactory	NILM	NILM	Negative (NILM)	
P00248	2018	2018-Q1	2018-03	Pre-pandemic	Cytology (Pap)	35-39	38	Not performed	Satisfactory	NILM	NILM	Negative (NILM)	
P00249	2018	2018-Q1	2018-03	Pre-pandemic	Cytology (Pap)	40-44	41	Not performed	Satisfactory	NILM	NILM	Negative (NILM)	
P00250	2018	2018-Q1	2018-03	Pre-pandemic	Cytology (Pap)	45-49	48	Not performed	Satisfactory	NILM	NILM	Negative (NILM)	
P00251	2018	2018-Q1	2018-03	Pre-pandemic	Cytology (Pap)	40-44	44	Not performed	Satisfactory	Positive	AGC	Glandular	
P00252	2018	2018-Q1	2018-03	Pre-pandemic	Cytology (Pap)	40-44	43	Not performed	Satisfactory	Positive	HSIL	High-grade squamous	
P00253	2018	2018-Q1	2018-03	Pre-pandemic	Cytology (Pap)	50-54	51	Not performed	Satisfactory	NILM	NILM	Negative (NILM)	
P00254	2018	2018-Q1	2018-03	Pre-pandemic	Cytology (Pap)	30-34	30	Not performed	Satisfactory	Positive	LSIL	Low-grade / equivocal squamous	
P00255	2018	2018-Q1	2018-03	Pre-pandemic	Cytology (Pap)	40-44	44	Not performed	Satisfactory	NILM	NILM	Negative (NILM)	
P00256	2018	2018-Q1	2018-03	Pre-pandemic	Cytology (Pap)	30-34	31	Not performed	Satisfactory	NILM	NILM	Negative (NILM)	
P00257	2018	2018-Q1	2018-03	Pre-pandemic	Cytology (Pap)	35-39	38	Not performed	Satisfactory	NILM	NILM	Negative (NILM)	
P00258	2018	2018-Q1	2018-03	Pre-pandemic	Cytology (Pap)	45-49	49	Not performed	Satisfactory	NILM	NILM	Negative (NILM)	Trichomonas
P00259	2018	2018-Q1	2018-03	Pre-pandemic	Cytology (Pap)	50-54	50	Not performed	Satisfactory	Positive	ASC-US	Low-grade / equivocal squamous	Candida
P00260	2018	2018-Q1	2018-03	Pre-pandemic	Cytology (Pap)	25-29	25	Not performed	Satisfactory	NILM	NILM	Negative (NILM)	
P00261	2018	2018-Q1	2018-03	Pre-pandemic	Cytology (Pap)	40-44	42	Not performed	Satisfactory	NILM	NILM	Negative (NILM)	
P00262	2018	2018-Q1	2018-03	Pre-pandemic	Cytology (Pap)	35-39	38	Not performed	Satisfactory	Positive	ASC-US	Low-grade / equivocal squamous	
P00263	2018	2018-Q1	2018-03	Pre-pandemic	Cytology (Pap)	50-54	50	Not performed	Unsatisfactory	Unsatisfactory	Unsatisfactory	Unsatisfactory	
P00264	2018	2018-Q1	2018-03	Pre-pandemic	Cytology (Pap)	45-49	47	Not performed	Satisfactory	NILM	NILM	Negative (NILM)	
P00265	2018	2018-Q1	2018-03	Pre-pandemic	Cytology (Pap)	25-29	25	Not performed	Satisfactory	NILM	NILM	Negative (NILM)	
P00266	2018	2018-Q1	2018-03	Pre-pandemic	Cytology (Pap)	35-39	39	Not performed	Satisfactory	NILM	NILM	Negative (NILM)	
P00267	2018	2018-Q1	2018-03	Pre-pandemic	Cytology (Pap)	40-44	44	Not performed	Satisfactory	NILM	NILM	Negative (NILM)	
P00268	2018	2018-Q1	2018-03	Pre-pandemic	Cytology (Pap)	45-49	47	Not performed	Satisfactory	NILM	NILM	Negative (NILM)	
P00269	2018	2018-Q1	2018-03	Pre-pandemic	Cytology (Pap)	25-29	25	Not performed	Satisfactory	NILM	NILM	Negative (NILM)	
P00270	2018	2018-Q1	2018-03	Pre-pandemic	Cytology (Pap)	55-59	59	Not performed	Unsatisfactory	Unsatisfactory	Unsatisfactory	Unsatisfactory	
P00271	2018	2018-Q1	2018-03	Pre-pandemic	Cytology (Pap)	30-34	32	Not performed	Satisfactory	NILM	NILM	Negative (NILM)	
P00272	2018	2018-Q1	2018-03	Pre-pandemic	Cytology (Pap)	45-49	48	Not performed	Satisfactory	NILM	NILM	Negative (NILM)	
P00273	2018	2018-Q1	2018-03	Pre-pandemic	Cytology (Pap)	Not recorded		Not performed	Satisfactory	NILM	NILM	Negative (NILM)	
P00274	2018	2018-Q1	2018-03	Pre-pandemic	Cytology (Pap)	35-39	37	Not performed	Satisfactory	NILM	NILM	Negative (NILM)	
P00275	2018	2018-Q1	2018-03	Pre-pandemic	Cytology (Pap)	45-49	46	Not performed	Satisfactory	NILM	NILM	Negative (NILM)	
P00276	2018	2018-Q1	2018-03	Pre-pandemic	Cytology (Pap)	35-39	36	Not performed	Satisfactory	NILM	NILM	Negative (NILM)	
P00277	2018	2018-Q1	2018-03	Pre-pandemic	Cytology (Pap)	40-44	44	Not performed	Satisfactory	NILM	NILM	Negative (NILM)	
P00278	2018	2018-Q1	2018-03	Pre-pandemic	Cytology (Pap)	45-49	45	Not performed	Satisfactory	NILM	NILM	Negative (NILM)	
P00279	2018	2018-Q1	2018-03	Pre-pandemic	Cytology (Pap)	30-34	33	Not performed	Satisfactory	NILM	NILM	Negative (NILM)	
P00280	2018	2018-Q1	2018-03	Pre-pandemic	Cytology (Pap)	30-34	30	Not performed	Satisfactory	Positive	ASC-US	Low-grade / equivocal squamous	
P00281	2018	2018-Q1	2018-03	Pre-pandemic	Cytology (Pap)	45-49	45	Not performed	Satisfactory	NILM	NILM	Negative (NILM)	
P00282	2018	2018-Q1	2018-03	Pre-pandemic	Cytology (Pap)	30-34	32	Not performed	Satisfactory	NILM	NILM	Negative (NILM)	
P00283	2018	2018-Q1	2018-03	Pre-pandemic	Cytology (Pap)	40-44	44	Not performed	Satisfactory	NILM	NILM	Negative (NILM)	
P00284	2018	2018-Q1	2018-03	Pre-pandemic	Cytology (Pap)	40-44	41	Not performed	Satisfactory	NILM	NILM	Negative (NILM)	
P00285	2018	2018-Q1	2018-03	Pre-pandemic	Cytology (Pap)	40-44	44	Not performed	Satisfactory	NILM	NILM	Negative (NILM)	
P00286	2018	2018-Q1	2018-03	Pre-pandemic	Cytology (Pap)	25-29	27	Not performed	Satisfactory	NILM	NILM	Negative (NILM)	
P00287	2018	2018-Q1	2018-03	Pre-pandemic	Cytology (Pap)	40-44	41	Not performed	Satisfactory	Positive	LSIL	Low-grade / equivocal squamous	
P00288	2018	2018-Q1	2018-03	Pre-pandemic	Cytology (Pap)	30-34	33	Not performed	Satisfactory	NILM	NILM	Negative (NILM)	
P00289	2018	2018-Q1	2018-03	Pre-pandemic	Cytology (Pap)	45-49	45	Not performed	Unsatisfactory	Unsatisfactory	Unsatisfactory	Unsatisfactory	
P00290	2018	2018-Q1	2018-03	Pre-pandemic	Cytology (Pap)	40-44	43	Not performed	Satisfactory	NILM	NILM	Negative (NILM)	Trichomonas
P00291	2018	2018-Q1	2018-03	Pre-pandemic	Cytology (Pap)	35-39	36	Not performed	Satisfactory	NILM	NILM	Negative (NILM)	
P00292	2018	2018-Q1	2018-03	Pre-pandemic	Cytology (Pap)	50-54	51	Not performed	Unsatisfactory	Unsatisfactory	Unsatisfactory	Unsatisfactory	
P00293	2018	2018-Q1	2018-03	Pre-pandemic	Cytology (Pap)	30-34	30	Not performed	Satisfactory	NILM	NILM	Negative (NILM)	
P00294	2018	2018-Q1	2018-03	Pre-pandemic	Cytology (Pap)	35-39	38	Not performed	Satisfactory	NILM	NILM	Negative (NILM)	
P00295	2018	2018-Q1	2018-03	Pre-pandemic	Cytology (Pap)	40-44	41	Not performed	Satisfactory	NILM	NILM	Negative (NILM)	
P00296	2018	2018-Q1	2018-03	Pre-pandemic	Cytology (Pap)	40-44	42	Not performed	Satisfactory	NILM	NILM	Negative (NILM)	
P00297	2018	2018-Q1	2018-03	Pre-pandemic	Cytology (Pap)	45-49	47	Not performed	Satisfactory	NILM	NILM	Negative (NILM)	
P00298	2018	2018-Q1	2018-03	Pre-pandemic	Cytology (Pap)	50-54	52	Not performed	Satisfactory	Positive	ASC-US	Low-grade / equivocal squamous	
P00299	2018	2018-Q1	2018-03	Pre-pandemic	Cytology (Pap)	45-49	45	Not performed	Satisfactory	Positive	LSIL	Low-grade / equivocal squamous	
P00300	2018	2018-Q1	2018-03	Pre-pandemic	Cytology (Pap)	30-34	34	Not performed	Satisfactory	NILM	NILM	Negative (NILM)	
P00301	2018	2018-Q1	2018-03	Pre-pandemic	Cytology (Pap)	45-49	46	Not performed	Satisfactory	NILM	NILM	Negative (NILM)	
P00302	2018	2018-Q1	2018-03	Pre-pandemic	Cytology (Pap)	40-44	44	Not performed	Satisfactory	NILM	NILM	Negative (NILM)	
P00303	2018	2018-Q1	2018-03	Pre-pandemic	Cytology (Pap)	Not recorded		Not performed	Satisfactory	NILM	NILM	Negative (NILM)	
P00304	2018	2018-Q1	2018-03	Pre-pandemic	Cytology (Pap)	55-59	56	Not performed	Satisfactory	NILM	NILM	Negative (NILM)	
P00305	2018	2018-Q1	2018-03	Pre-pandemic	Cytology (Pap)	35-39	37	Not performed	Satisfactory	Positive	LSIL	Low-grade / equivocal squamous	Candida
P00306	2018	2018-Q1	2018-03	Pre-pandemic	Cytology (Pap)	45-49	49	Not performed	Satisfactory	NILM	NILM	Negative (NILM)	
P00307	2018	2018-Q1	2018-03	Pre-pandemic	Cytology (Pap)	Not recorded		Not performed	Satisfactory	NILM	NILM	Negative (NILM)	
P00308	2018	2018-Q1	2018-03	Pre-pandemic	Cytology (Pap)	35-39	35	Not performed	Satisfactory	NILM	NILM	Negative (NILM)	
P00309	2018	2018-Q1	2018-03	Pre-pandemic	Cytology (Pap)	50-54	50	Not performed	Satisfactory	NILM	NILM	Negative (NILM)	
P00310	2018	2018-Q1	2018-03	Pre-pandemic	Cytology (Pap)	50-54	51	Not performed	Satisfactory	NILM	NILM	Negative (NILM)	
P00311	2018	2018-Q1	2018-03	Pre-pandemic	Cytology (Pap)	40-44	41	Not performed	Satisfactory	Positive	LSIL	Low-grade / equivocal squamous	
P00312	2018	2018-Q1	2018-03	Pre-pandemic	Cytology (Pap)	45-49	46	Not performed	Satisfactory	NILM	NILM	Negative (NILM)	
P00313	2018	2018-Q1	2018-03	Pre-pandemic	Cytology (Pap)	55-59	59	Not performed	Satisfactory	NILM	NILM	Negative (NILM)	
P00314	2018	2018-Q1	2018-03	Pre-pandemic	Cytology (Pap)	Not recorded		Not performed	Satisfactory	NILM	NILM	Negative (NILM)	
P00315	2018	2018-Q1	2018-03	Pre-pandemic	Cytology (Pap)	40-44	40	Not performed	Satisfactory	NILM	NILM	Negative (NILM)	
P00316	2018	2018-Q1	2018-03	Pre-pandemic	Cytology (Pap)	35-39	35	Not performed	Satisfactory	NILM	NILM	Negative (NILM)	
P00317	2018	2018-Q1	2018-03	Pre-pandemic	Cytology (Pap)	40-44	44	Not performed	Satisfactory	NILM	NILM	Negative (NILM)	
P00318	2018	2018-Q1	2018-03	Pre-pandemic	Cytology (Pap)	55-59	58	Not performed	Satisfactory	NILM	NILM	Negative (NILM)	

patient_id	year	quarter	month	period	primary_test	age_group	age_years	hpv_result	adequacy	result	bethesda	grade	infection
P00319	2018	2018-Q1	2018-03	Pre-pandemic	Cytology (Pap)	35-39	36	Not performed	Satisfactory	NILM	NILM	Negative (NILM)	
P00320	2018	2018-Q1	2018-03	Pre-pandemic	Cytology (Pap)	45-49	47	Not performed	Satisfactory	NILM	NILM	Negative (NILM)	
P00321	2018	2018-Q1	2018-03	Pre-pandemic	Cytology (Pap)	50-54	51	Not performed	Satisfactory	NILM	NILM	Negative (NILM)	
P00322	2018	2018-Q1	2018-03	Pre-pandemic	Cytology (Pap)	25-29	29	Not performed	Satisfactory	NILM	NILM	Negative (NILM)	
P00323	2018	2018-Q1	2018-03	Pre-pandemic	Cytology (Pap)	30-34	34	Not performed	Satisfactory	NILM	NILM	Negative (NILM)	
P00324	2018	2018-Q1	2018-03	Pre-pandemic	Cytology (Pap)	40-44	44	Not performed	Satisfactory	NILM	NILM	Negative (NILM)	
P00325	2018	2018-Q1	2018-03	Pre-pandemic	Cytology (Pap)	35-39	36	Not performed	Satisfactory	NILM	NILM	Negative (NILM)	
P00326	2018	2018-Q1	2018-03	Pre-pandemic	Cytology (Pap)	55-59	57	Not performed	Satisfactory	NILM	NILM	Negative (NILM)	
P00327	2018	2018-Q1	2018-03	Pre-pandemic	Cytology (Pap)	40-44	40	Not performed	Satisfactory	NILM	NILM	Negative (NILM)	
P00328	2018	2018-Q1	2018-03	Pre-pandemic	Cytology (Pap)	30-34	32	Not performed	Satisfactory	Positive	LSIL	Low-grade / equivocal squamous	
P00329	2018	2018-Q1	2018-03	Pre-pandemic	Cytology (Pap)	40-44	43	Not performed	Satisfactory	NILM	NILM	Negative (NILM)	
P00330	2018	2018-Q1	2018-03	Pre-pandemic	Cytology (Pap)	45-49	46	Not performed	Satisfactory	NILM	NILM	Negative (NILM)	
P00331	2018	2018-Q1	2018-03	Pre-pandemic	Cytology (Pap)	50-54	52	Not performed	Satisfactory	NILM	NILM	Negative (NILM)	
P00332	2018	2018-Q1	2018-03	Pre-pandemic	Cytology (Pap)	40-44	41	Not performed	Satisfactory	Positive	LSIL	Low-grade / equivocal squamous	
P00333	2018	2018-Q1	2018-03	Pre-pandemic	Cytology (Pap)	45-49	45	Not performed	Satisfactory	Positive	ASC-H	High-grade squamous	
P00334	2018	2018-Q1	2018-03	Pre-pandemic	Cytology (Pap)	25-29	26	Not performed	Satisfactory	NILM	NILM	Negative (NILM)	
P00335	2018	2018-Q1	2018-03	Pre-pandemic	Cytology (Pap)	60-64	63	Not performed	Satisfactory	NILM	NILM	Negative (NILM)	
P00336	2018	2018-Q1	2018-03	Pre-pandemic	Cytology (Pap)	35-39	39	Not performed	Satisfactory	NILM	NILM	Negative (NILM)	Candida
P00337	2018	2018-Q2	2018-04	Pre-pandemic	Cytology (Pap)	35-39	35	Not performed	Satisfactory	NILM	NILM	Negative (NILM)	
P00338	2018	2018-Q2	2018-04	Pre-pandemic	Cytology (Pap)	50-54	51	Not performed	Satisfactory	NILM	NILM	Negative (NILM)	
P00339	2018	2018-Q2	2018-04	Pre-pandemic	Cytology (Pap)	35-39	38	Not performed	Satisfactory	NILM	NILM	Negative (NILM)	
P00340	2018	2018-Q2	2018-04	Pre-pandemic	Cytology (Pap)	55-59	55	Not performed	Satisfactory	NILM	NILM	Negative (NILM)	
P00341	2018	2018-Q2	2018-04	Pre-pandemic	Cytology (Pap)	45-49	46	Not performed	Satisfactory	Positive	ASC-US	Low-grade / equivocal squamous	
P00342	2018	2018-Q2	2018-04	Pre-pandemic	Cytology (Pap)	30-34	34	Not performed	Satisfactory	NILM	NILM	Negative (NILM)	
P00343	2018	2018-Q2	2018-04	Pre-pandemic	Cytology (Pap)	35-39	38	Not performed	Satisfactory	NILM	NILM	Negative (NILM)	
P00344	2018	2018-Q2	2018-04	Pre-pandemic	Cytology (Pap)	35-39	39	Not performed	Satisfactory	NILM	NILM	Negative (NILM)	
P00345	2018	2018-Q2	2018-04	Pre-pandemic	Cytology (Pap)	40-44	40	Not performed	Satisfactory	NILM	NILM	Negative (NILM)	
P00346	2018	2018-Q2	2018-04	Pre-pandemic	Cytology (Pap)	40-44	41	Not performed	Satisfactory	NILM	NILM	Negative (NILM)	
P00347	2018	2018-Q2	2018-04	Pre-pandemic	Cytology (Pap)	25-29	26	Not performed	Satisfactory	NILM	NILM	Negative (NILM)	
P00348	2018	2018-Q2	2018-04	Pre-pandemic	Cytology (Pap)	45-49	48	Not performed	Satisfactory	NILM	NILM	Negative (NILM)	
P00349	2018	2018-Q2	2018-04	Pre-pandemic	Cytology (Pap)	40-44	42	Not performed	Satisfactory	NILM	NILM	Negative (NILM)	
P00350	2018	2018-Q2	2018-04	Pre-pandemic	Cytology (Pap)	45-49	47	Not performed	Satisfactory	NILM	NILM	Negative (NILM)	
P00351	2018	2018-Q2	2018-04	Pre-pandemic	Cytology (Pap)	35-39	37	Not performed	Satisfactory	NILM	NILM	Negative (NILM)	
P00352	2018	2018-Q2	2018-04	Pre-pandemic	Cytology (Pap)	35-39	39	Not performed	Satisfactory	NILM	NILM	Negative (NILM)	
P00353	2018	2018-Q2	2018-04	Pre-pandemic	Cytology (Pap)	60-64	60	Not performed	Satisfactory	NILM	NILM	Negative (NILM)	
P00354	2018	2018-Q2	2018-04	Pre-pandemic	Cytology (Pap)	50-54	54	Not performed	Satisfactory	NILM	NILM	Negative (NILM)	
P00355	2018	2018-Q2	2018-04	Pre-pandemic	Cytology (Pap)	25-29	28	Not performed	Satisfactory	NILM	NILM	Negative (NILM)	
P00356	2018	2018-Q2	2018-04	Pre-pandemic	Cytology (Pap)	45-49	45	Not performed	Satisfactory	NILM	NILM	Negative (NILM)	
P00357	2018	2018-Q2	2018-04	Pre-pandemic	Cytology (Pap)	50-54	52	Not performed	Satisfactory	NILM	NILM	Negative (NILM)	
P00358	2018	2018-Q2	2018-04	Pre-pandemic	Cytology (Pap)	35-39	36	Not performed	Satisfactory	NILM	NILM	Negative (NILM)	
P00359	2018	2018-Q2	2018-04	Pre-pandemic	Cytology (Pap)	30-34	31	Not performed	Satisfactory	NILM	NILM	Negative (NILM)	
P00360	2018	2018-Q2	2018-04	Pre-pandemic	Cytology (Pap)	35-39	35	Not performed	Satisfactory	NILM	NILM	Negative (NILM)	
P00361	2018	2018-Q2	2018-04	Pre-pandemic	Cytology (Pap)	60-64	62	Not performed	Satisfactory	NILM	NILM	Negative (NILM)	
P00362	2018	2018-Q2	2018-04	Pre-pandemic	Cytology (Pap)	25-29	29	Not performed	Satisfactory	NILM	NILM	Negative (NILM)	
P00363	2018	2018-Q2	2018-04	Pre-pandemic	Cytology (Pap)	30-34	32	Not performed	Satisfactory	NILM	NILM	Negative (NILM)	
P00364	2018	2018-Q2	2018-04	Pre-pandemic	Cytology (Pap)	40-44	42	Not performed	Satisfactory	NILM	NILM	Negative (NILM)	
P00365	2018	2018-Q2	2018-04	Pre-pandemic	Cytology (Pap)	Not recorded		Not performed	Satisfactory	NILM	NILM	Negative (NILM)	
P00366	2018	2018-Q2	2018-04	Pre-pandemic	Cytology (Pap)	45-49	45	Not performed	Satisfactory	NILM	NILM	Negative (NILM)	
P00367	2018	2018-Q2	2018-04	Pre-pandemic	Cytology (Pap)	45-49	48	Not performed	Satisfactory	Positive	ASC-H	High-grade squamous	Candida
P00368	2018	2018-Q2	2018-04	Pre-pandemic	Cytology (Pap)	40-44	43	Not performed	Satisfactory	NILM	NILM	Negative (NILM)	
P00369	2018	2018-Q2	2018-04	Pre-pandemic	Cytology (Pap)	60-64	60	Not performed	Satisfactory	NILM	NILM	Negative (NILM)	
P00370	2018	2018-Q2	2018-04	Pre-pandemic	Cytology (Pap)	40-44	40	Not performed	Satisfactory	NILM	NILM	Negative (NILM)	
P00371	2018	2018-Q2	2018-04	Pre-pandemic	Cytology (Pap)	35-39	37	Not performed	Satisfactory	NILM	NILM	Negative (NILM)	
P00372	2018	2018-Q2	2018-04	Pre-pandemic	Cytology (Pap)	35-39	36	Not performed	Satisfactory	NILM	NILM	Negative (NILM)	
P00373	2018	2018-Q2	2018-04	Pre-pandemic	Cytology (Pap)	45-49	49	Not performed	Satisfactory	Positive	HSIL	High-grade squamous	
P00374	2018	2018-Q2	2018-04	Pre-pandemic	Cytology (Pap)	50-54	52	Not performed	Satisfactory	NILM	NILM	Negative (NILM)	
P00375	2018	2018-Q2	2018-04	Pre-pandemic	Cytology (Pap)	60-64	62	Not performed	Unsatisfactory	Unsatisfactory	Unsatisfactory	Unsatisfactory	
P00376	2018	2018-Q2	2018-04	Pre-pandemic	Cytology (Pap)	30-34	32	Not performed	Satisfactory	Positive	LSIL	Low-grade / equivocal squamous	
P00377	2018	2018-Q2	2018-04	Pre-pandemic	Cytology (Pap)	60-64	60	Not performed	Satisfactory	NILM	NILM	Negative (NILM)	
P00378	2018	2018-Q2	2018-04	Pre-pandemic	Cytology (Pap)	35-39	38	Not performed	Satisfactory	Positive	LSIL	Low-grade / equivocal squamous	
P00379	2018	2018-Q2	2018-04	Pre-pandemic	Cytology (Pap)	45-49	47	Not performed	Satisfactory	NILM	NILM	Negative (NILM)	
P00380	2018	2018-Q2	2018-04	Pre-pandemic	Cytology (Pap)	25-29	29	Not performed	Satisfactory	NILM	NILM	Negative (NILM)	
P00381	2018	2018-Q2	2018-04	Pre-pandemic	Cytology (Pap)	55-59	56	Not performed	Satisfactory	NILM	NILM	Negative (NILM)	
P00382	2018	2018-Q2	2018-04	Pre-pandemic	Cytology (Pap)	55-59	59	Not performed	Satisfactory	NILM	NILM	Negative (NILM)	
P00383	2018	2018-Q2	2018-04	Pre-pandemic	Cytology (Pap)	50-54	51	Not performed	Unsatisfactory	Unsatisfactory	Unsatisfactory	Unsatisfactory	
P00384	2018	2018-Q2	2018-04	Pre-pandemic	Cytology (Pap)	40-44	40	Not performed	Satisfactory	NILM	NILM	Negative (NILM)	
P00385	2018	2018-Q2	2018-04	Pre-pandemic	Cytology (Pap)	40-44	42	Not performed	Satisfactory	NILM	NILM	Negative (NILM)	
P00386	2018	2018-Q2	2018-04	Pre-pandemic	Cytology (Pap)	30-34	33	Not performed	Satisfactory	Positive	ASC-US	Low-grade / equivocal squamous	
P00387	2018	2018-Q2	2018-04	Pre-pandemic	Cytology (Pap)	50-54	53	Not performed	Satisfactory	NILM	NILM	Negative (NILM)	Other
P00388	2018	2018-Q2	2018-04	Pre-pandemic	Cytology (Pap)	45-49	49	Not performed	Satisfactory	Positive	HSIL	High-grade squamous	Trichomonas
P00389	2018	2018-Q2	2018-04	Pre-pandemic	Cytology (Pap)	60-64	61	Not performed	Satisfactory	NILM	NILM	Negative (NILM)	
P00390	2018	2018-Q2	2018-04	Pre-pandemic	Cytology (Pap)	60-64	61	Not performed	Satisfactory	Positive	HSIL	High-grade squamous	
P00391	2018	2018-Q2	2018-04	Pre-pandemic	Cytology (Pap)	30-34	34	Not performed	Satisfactory	NILM	NILM	Negative (NILM)	
P00392	2018	2018-Q2	2018-04	Pre-pandemic	Cytology (Pap)	40-44	43	Not performed	Satisfactory	NILM	NILM	Negative (NILM)	
P00393	2018	2018-Q2	2018-04	Pre-pandemic	Cytology (Pap)	40-44	40	Not performed	Satisfactory	NILM	NILM	Negative (NILM)	
P00394	2018	2018-Q2	2018-04	Pre-pandemic	Cytology (Pap)	35-39	36	Not performed	Satisfactory	NILM	NILM	Negative (NILM)	
P00395	2018	2018-Q2	2018-04	Pre-pandemic	Cytology (Pap)	40-44	41	Not performed	Satisfactory	NILM	NILM	Negative (NILM)	
P00396	2018	2018-Q2	2018-04	Pre-pandemic	Cytology (Pap)	Not recorded		Not performed	Satisfactory	NILM	NILM	Negative (NILM)	
P00397	2018	2018-Q2	2018-04	Pre-pandemic	Cytology (Pap)	Not recorded		Not performed	Satisfactory	NILM	NILM	Negative (NILM)	
P00398	2018	2018-Q2	2018-04	Pre-pandemic	Cytology (Pap)	25-29	27	Not performed	Satisfactory	NILM	NILM	Negative (NILM)	

patient_id	year	quarter	month	period	primary_test	age_group	age_years	hpv_result	adequacy	result	bethesda	grade	infection
P00399	2018	2018-Q2	2018-04	Pre-pandemic	Cytology (Pap)	55-59	56	Not performed	Satisfactory	Positive	ASC-US	Low-grade / equivocal squamous	
P00400	2018	2018-Q2	2018-04	Pre-pandemic	Cytology (Pap)	30-34	34	Not performed	Satisfactory	NILM	NILM	Negative (NILM)	
P00401	2018	2018-Q2	2018-04	Pre-pandemic	Cytology (Pap)	35-39	35	Not performed	Satisfactory	NILM	NILM	Negative (NILM)	
P00402	2018	2018-Q2	2018-04	Pre-pandemic	Cytology (Pap)	50-54	53	Not performed	Satisfactory	NILM	NILM	Negative (NILM)	
P00403	2018	2018-Q2	2018-04	Pre-pandemic	Cytology (Pap)	30-34	30	Not performed	Satisfactory	NILM	NILM	Negative (NILM)	
P00404	2018	2018-Q2	2018-04	Pre-pandemic	Cytology (Pap)	55-59	59	Not performed	Satisfactory	NILM	NILM	Negative (NILM)	
P00405	2018	2018-Q2	2018-04	Pre-pandemic	Cytology (Pap)	60-64	60	Not performed	Satisfactory	NILM	NILM	Negative (NILM)	
P00406	2018	2018-Q2	2018-04	Pre-pandemic	Cytology (Pap)	60-64	61	Not performed	Satisfactory	NILM	NILM	Negative (NILM)	
P00407	2018	2018-Q2	2018-04	Pre-pandemic	Cytology (Pap)	25-29	26	Not performed	Satisfactory	NILM	NILM	Negative (NILM)	Trichomonas
P00408	2018	2018-Q2	2018-04	Pre-pandemic	Cytology (Pap)	40-44	41	Not performed	Satisfactory	NILM	NILM	Negative (NILM)	
P00409	2018	2018-Q2	2018-04	Pre-pandemic	Cytology (Pap)	45-49	47	Not performed	Satisfactory	NILM	NILM	Negative (NILM)	
P00410	2018	2018-Q2	2018-04	Pre-pandemic	Cytology (Pap)	30-34	30	Not performed	Satisfactory	NILM	NILM	Negative (NILM)	
P00411	2018	2018-Q2	2018-04	Pre-pandemic	Cytology (Pap)	40-44	42	Not performed	Satisfactory	NILM	NILM	Negative (NILM)	
P00412	2018	2018-Q2	2018-04	Pre-pandemic	Cytology (Pap)	40-44	41	Not performed	Satisfactory	NILM	NILM	Negative (NILM)	
P00413	2018	2018-Q2	2018-04	Pre-pandemic	Cytology (Pap)	50-54	54	Not performed	Satisfactory	NILM	NILM	Negative (NILM)	
P00414	2018	2018-Q2	2018-04	Pre-pandemic	Cytology (Pap)	55-59	58	Not performed	Unsatisfactory	Unsatisfactory	Unsatisfactory	Unsatisfactory	
P00415	2018	2018-Q2	2018-04	Pre-pandemic	Cytology (Pap)	35-39	36	Not performed	Satisfactory	NILM	NILM	Negative (NILM)	
P00416	2018	2018-Q2	2018-04	Pre-pandemic	Cytology (Pap)	30-34	30	Not performed	Satisfactory	NILM	NILM	Negative (NILM)	
P00417	2018	2018-Q2	2018-04	Pre-pandemic	Cytology (Pap)	40-44	40	Not performed	Satisfactory	NILM	NILM	Negative (NILM)	
P00418	2018	2018-Q2	2018-04	Pre-pandemic	Cytology (Pap)	35-39	39	Not performed	Satisfactory	NILM	NILM	Negative (NILM)	
P00419	2018	2018-Q2	2018-04	Pre-pandemic	Cytology (Pap)	50-54	53	Not performed	Satisfactory	NILM	NILM	Negative (NILM)	Trichomonas
P00420	2018	2018-Q2	2018-04	Pre-pandemic	Cytology (Pap)	45-49	48	Not performed	Satisfactory	NILM	NILM	Negative (NILM)	
P00421	2018	2018-Q2	2018-04	Pre-pandemic	Cytology (Pap)	35-39	35	Not performed	Satisfactory	NILM	NILM	Negative (NILM)	
P00422	2018	2018-Q2	2018-04	Pre-pandemic	Cytology (Pap)	40-44	41	Not performed	Satisfactory	NILM	NILM	Negative (NILM)	
P00423	2018	2018-Q2	2018-04	Pre-pandemic	Cytology (Pap)	60-64	64	Not performed	Satisfactory	NILM	NILM	Negative (NILM)	
P00424	2018	2018-Q2	2018-04	Pre-pandemic	Cytology (Pap)	55-59	56	Not performed	Satisfactory	NILM	NILM	Negative (NILM)	
P00425	2018	2018-Q2	2018-04	Pre-pandemic	Cytology (Pap)	60-64	62	Not performed	Satisfactory	NILM	NILM	Negative (NILM)	
P00426	2018	2018-Q2	2018-04	Pre-pandemic	Cytology (Pap)	50-54	53	Not performed	Satisfactory	NILM	NILM	Negative (NILM)	
P00427	2018	2018-Q2	2018-04	Pre-pandemic	Cytology (Pap)	25-29	26	Not performed	Satisfactory	Positive	ASC-US	Low-grade / equivocal squamous	
P00428	2018	2018-Q2	2018-04	Pre-pandemic	Cytology (Pap)	40-44	43	Not performed	Satisfactory	Positive	HSIL	High-grade squamous	
P00429	2018	2018-Q2	2018-04	Pre-pandemic	Cytology (Pap)	55-59	59	Not performed	Satisfactory	Positive	LSIL	Low-grade / equivocal squamous	
P00430	2018	2018-Q2	2018-04	Pre-pandemic	Cytology (Pap)	35-39	35	Not performed	Satisfactory	NILM	NILM	Negative (NILM)	
P00431	2018	2018-Q2	2018-04	Pre-pandemic	Cytology (Pap)	35-39	37	Not performed	Satisfactory	Positive	ASC-H	High-grade squamous	
P00432	2018	2018-Q2	2018-04	Pre-pandemic	Cytology (Pap)	55-59	59	Not performed	Satisfactory	NILM	NILM	Negative (NILM)	
P00433	2018	2018-Q2	2018-04	Pre-pandemic	Cytology (Pap)	45-49	46	Not performed	Satisfactory	NILM	NILM	Negative (NILM)	
P00434	2018	2018-Q2	2018-04	Pre-pandemic	Cytology (Pap)	45-49	46	Not performed	Satisfactory	NILM	NILM	Negative (NILM)	
P00435	2018	2018-Q2	2018-04	Pre-pandemic	Cytology (Pap)	45-49	49	Not performed	Satisfactory	NILM	NILM	Negative (NILM)	
P00436	2018	2018-Q2	2018-04	Pre-pandemic	Cytology (Pap)	40-44	43	Not performed	Satisfactory	NILM	NILM	Negative (NILM)	
P00437	2018	2018-Q2	2018-04	Pre-pandemic	Cytology (Pap)	30-34	31	Not performed	Satisfactory	NILM	NILM	Negative (NILM)	
P00438	2018	2018-Q2	2018-04	Pre-pandemic	Cytology (Pap)	30-34	30	Not performed	Satisfactory	NILM	NILM	Negative (NILM)	
P00439	2018	2018-Q2	2018-04	Pre-pandemic	Cytology (Pap)	35-39	38	Not performed	Satisfactory	NILM	NILM	Negative (NILM)	
P00440	2018	2018-Q2	2018-04	Pre-pandemic	Cytology (Pap)	60-64	64	Not performed	Satisfactory	Positive	ASC-US	Low-grade / equivocal squamous	
P00441	2018	2018-Q2	2018-04	Pre-pandemic	Cytology (Pap)	30-34	31	Not performed	Satisfactory	NILM	NILM	Negative (NILM)	
P00442	2018	2018-Q2	2018-04	Pre-pandemic	Cytology (Pap)	60-64	61	Not performed	Satisfactory	NILM	NILM	Negative (NILM)	
P00443	2018	2018-Q2	2018-04	Pre-pandemic	Cytology (Pap)	50-54	51	Not performed	Satisfactory	NILM	NILM	Negative (NILM)	
P00444	2018	2018-Q2	2018-04	Pre-pandemic	Cytology (Pap)	Not recorded		Not performed	Satisfactory	NILM	NILM	Negative (NILM)	
P00445	2018	2018-Q2	2018-04	Pre-pandemic	Cytology (Pap)	40-44	43	Not performed	Satisfactory	NILM	NILM	Negative (NILM)	
P00446	2018	2018-Q2	2018-04	Pre-pandemic	Cytology (Pap)	50-54	51	Not performed	Satisfactory	Positive	AGC	Glandular	
P00447	2018	2018-Q2	2018-04	Pre-pandemic	Cytology (Pap)	60-64	61	Not performed	Satisfactory	NILM	NILM	Negative (NILM)	
P00448	2018	2018-Q2	2018-04	Pre-pandemic	Cytology (Pap)	25-29	27	Not performed	Satisfactory	Positive	HSIL	High-grade squamous	
P00449	2018	2018-Q2	2018-04	Pre-pandemic	Cytology (Pap)	40-44	41	Not performed	Satisfactory	NILM	NILM	Negative (NILM)	
P00450	2018	2018-Q2	2018-04	Pre-pandemic	Cytology (Pap)	35-39	39	Not performed	Satisfactory	NILM	NILM	Negative (NILM)	Other
P00451	2018	2018-Q2	2018-04	Pre-pandemic	Cytology (Pap)	30-34	34	Not performed	Satisfactory	Positive	HSIL	High-grade squamous	
P00452	2018	2018-Q2	2018-04	Pre-pandemic	Cytology (Pap)	50-54	54	Not performed	Satisfactory	Positive	ASC-US	Low-grade / equivocal squamous	
P00453	2018	2018-Q2	2018-04	Pre-pandemic	Cytology (Pap)	40-44	40	Not performed	Satisfactory	NILM	NILM	Negative (NILM)	
P00454	2018	2018-Q2	2018-04	Pre-pandemic	Cytology (Pap)	40-44	41	Not performed	Satisfactory	Positive	HSIL	High-grade squamous	
P00455	2018	2018-Q2	2018-04	Pre-pandemic	Cytology (Pap)	35-39	36	Not performed	Satisfactory	NILM	NILM	Negative (NILM)	Gardnerella
P00456	2018	2018-Q2	2018-04	Pre-pandemic	Cytology (Pap)	45-49	49	Not performed	Satisfactory	NILM	NILM	Negative (NILM)	
P00457	2018	2018-Q2	2018-04	Pre-pandemic	Cytology (Pap)	Not recorded		Not performed	Satisfactory	Positive	ASC-H	High-grade squamous	
P00458	2018	2018-Q2	2018-04	Pre-pandemic	Cytology (Pap)	30-34	31	Not performed	Satisfactory	NILM	NILM	Negative (NILM)	
P00459	2018	2018-Q2	2018-04	Pre-pandemic	Cytology (Pap)	55-59	58	Not performed	Satisfactory	NILM	NILM	Negative (NILM)	
P00460	2018	2018-Q2	2018-04	Pre-pandemic	Cytology (Pap)	30-34	33	Not performed	Satisfactory	NILM	NILM	Negative (NILM)	
P00461	2018	2018-Q2	2018-04	Pre-pandemic	Cytology (Pap)	55-59	59	Not performed	Satisfactory	NILM	NILM	Negative (NILM)	
P00462	2018	2018-Q2	2018-04	Pre-pandemic	Cytology (Pap)	25-29	29	Not performed	Satisfactory	NILM	NILM	Negative (NILM)	
P00463	2018	2018-Q2	2018-04	Pre-pandemic	Cytology (Pap)	40-44	40	Not performed	Unsatisfactory	Unsatisfactory	Unsatisfactory	Unsatisfactory	
P00464	2018	2018-Q2	2018-04	Pre-pandemic	Cytology (Pap)	Not recorded		Not performed	Satisfactory	NILM	NILM	Negative (NILM)	
P00465	2018	2018-Q2	2018-04	Pre-pandemic	Cytology (Pap)	30-34	31	Not performed	Satisfactory	NILM	NILM	Negative (NILM)	
P00466	2018	2018-Q2	2018-04	Pre-pandemic	Cytology (Pap)	25-29	27	Not performed	Satisfactory	NILM	NILM	Negative (NILM)	
P00467	2018	2018-Q2	2018-04	Pre-pandemic	Cytology (Pap)	Not recorded		Not performed	Satisfactory	NILM	NILM	Negative (NILM)	
P00468	2018	2018-Q2	2018-04	Pre-pandemic	Cytology (Pap)	50-54	52	Not performed	Satisfactory	NILM	NILM	Negative (NILM)	
P00469	2018	2018-Q2	2018-04	Pre-pandemic	Cytology (Pap)	60-64	64	Not performed	Satisfactory	NILM	NILM	Negative (NILM)	
P00470	2018	2018-Q2	2018-04	Pre-pandemic	Cytology (Pap)	35-39	37	Not performed	Satisfactory	NILM	NILM	Negative (NILM)	
P00471	2018	2018-Q2	2018-04	Pre-pandemic	Cytology (Pap)	55-59	58	Not performed	Satisfactory	NILM	NILM	Negative (NILM)	
P00472	2018	2018-Q2	2018-04	Pre-pandemic	Cytology (Pap)	40-44	41	Not performed	Satisfactory	NILM	NILM	Negative (NILM)	
P00473	2018	2018-Q2	2018-04	Pre-pandemic	Cytology (Pap)	40-44	40	Not performed	Unsatisfactory	Unsatisfactory	Unsatisfactory	Unsatisfactory	
P00474	2018	2018-Q2	2018-04	Pre-pandemic	Cytology (Pap)	55-59	55	Not performed	Satisfactory	NILM	NILM	Negative (NILM)	
P00475	2018	2018-Q2	2018-04	Pre-pandemic	Cytology (Pap)	40-44	40	Not performed	Satisfactory	NILM	NILM	Negative (NILM)	
P00476	2018	2018-Q2	2018-04	Pre-pandemic	Cytology (Pap)	40-44	43	Not performed	Satisfactory	NILM	NILM	Negative (NILM)	
P00477	2018	2018-Q2	2018-04	Pre-pandemic	Cytology (Pap)	40-44	40	Not performed	Satisfactory	NILM	NILM	Negative (NILM)	
P00478	2018	2018-Q2	2018-04	Pre-pandemic	Cytology (Pap)	40-44	42	Not performed	Satisfactory	NILM	NILM	Negative (NILM)	

patient_id	year	quarter	month	period	primary_test	age_group	age_years	hpv_result	adequacy	result	bethesda	grade	infection
P00479	2018	2018-Q2	2018-04	Pre-pandemic	Cytology (Pap)	60-64	61	Not performed	Satisfactory	NILM	NILM	Negative (NILM)	Trichomonas
P00480	2018	2018-Q2	2018-04	Pre-pandemic	Cytology (Pap)	60-64	60	Not performed	Satisfactory	Positive	ASC-H	High-grade squamous	
P00481	2018	2018-Q2	2018-04	Pre-pandemic	Cytology (Pap)	50-54	52	Not performed	Satisfactory	NILM	NILM	Negative (NILM)	
P00482	2018	2018-Q2	2018-04	Pre-pandemic	Cytology (Pap)	40-44	41	Not performed	Satisfactory	Positive	ASC-US	Low-grade / equivocal squamous	
P00483	2018	2018-Q2	2018-04	Pre-pandemic	Cytology (Pap)	60-64	62	Not performed	Satisfactory	NILM	NILM	Negative (NILM)	
P00484	2018	2018-Q2	2018-04	Pre-pandemic	Cytology (Pap)	30-34	32	Not performed	Satisfactory	NILM	NILM	Negative (NILM)	
P00485	2018	2018-Q2	2018-04	Pre-pandemic	Cytology (Pap)	50-54	50	Not performed	Satisfactory	NILM	NILM	Negative (NILM)	
P00486	2018	2018-Q2	2018-04	Pre-pandemic	Cytology (Pap)	50-54	54	Not performed	Satisfactory	NILM	NILM	Negative (NILM)	
P00487	2018	2018-Q2	2018-04	Pre-pandemic	Cytology (Pap)	40-44	44	Not performed	Satisfactory	NILM	NILM	Negative (NILM)	
P00488	2018	2018-Q2	2018-04	Pre-pandemic	Cytology (Pap)	55-59	57	Not performed	Satisfactory	NILM	NILM	Negative (NILM)	
P00489	2018	2018-Q2	2018-04	Pre-pandemic	Cytology (Pap)	55-59	56	Not performed	Satisfactory	NILM	NILM	Negative (NILM)	
P00490	2018	2018-Q2	2018-04	Pre-pandemic	Cytology (Pap)	25-29	27	Not performed	Satisfactory	NILM	NILM	Negative (NILM)	
P00491	2018	2018-Q2	2018-04	Pre-pandemic	Cytology (Pap)	35-39	35	Not performed	Satisfactory	NILM	NILM	Negative (NILM)	
P00492	2018	2018-Q2	2018-04	Pre-pandemic	Cytology (Pap)	55-59	56	Not performed	Satisfactory	NILM	NILM	Negative (NILM)	
P00493	2018	2018-Q2	2018-04	Pre-pandemic	Cytology (Pap)	45-49	47	Not performed	Satisfactory	NILM	NILM	Negative (NILM)	
P00494	2018	2018-Q2	2018-04	Pre-pandemic	Cytology (Pap)	40-44	43	Not performed	Satisfactory	NILM	NILM	Negative (NILM)	
P00495	2018	2018-Q2	2018-04	Pre-pandemic	Cytology (Pap)	60-64	61	Not performed	Satisfactory	NILM	NILM	Negative (NILM)	
P00496	2018	2018-Q2	2018-04	Pre-pandemic	Cytology (Pap)	60-64	62	Not performed	Satisfactory	NILM	NILM	Negative (NILM)	
P00497	2018	2018-Q2	2018-04	Pre-pandemic	Cytology (Pap)	30-34	34	Not performed	Satisfactory	NILM	NILM	Negative (NILM)	
P00498	2018	2018-Q2	2018-04	Pre-pandemic	Cytology (Pap)	45-49	47	Not performed	Unsatisfactory	Unsatisfactory	Unsatisfactory	Unsatisfactory	
P00499	2018	2018-Q2	2018-04	Pre-pandemic	Cytology (Pap)	60-64	61	Not performed	Satisfactory	Positive	LSIL	Low-grade / equivocal squamous	
P00500	2018	2018-Q2	2018-04	Pre-pandemic	Cytology (Pap)	25-29	28	Not performed	Satisfactory	NILM	NILM	Negative (NILM)	
P00501	2018	2018-Q2	2018-04	Pre-pandemic	Cytology (Pap)	60-64	60	Not performed	Satisfactory	NILM	NILM	Negative (NILM)	Trichomonas
P00502	2018	2018-Q2	2018-04	Pre-pandemic	Cytology (Pap)	55-59	55	Not performed	Satisfactory	NILM	NILM	Negative (NILM)	
P00503	2018	2018-Q2	2018-04	Pre-pandemic	Cytology (Pap)	40-44	42	Not performed	Satisfactory	NILM	NILM	Negative (NILM)	
P00504	2018	2018-Q2	2018-04	Pre-pandemic	Cytology (Pap)	40-44	41	Not performed	Satisfactory	Positive	LSIL	Low-grade / equivocal squamous	
P00505	2018	2018-Q2	2018-04	Pre-pandemic	Cytology (Pap)	40-44	41	Not performed	Unsatisfactory	Unsatisfactory	Unsatisfactory	Unsatisfactory	Candida
P00506	2018	2018-Q2	2018-04	Pre-pandemic	Cytology (Pap)	45-49	49	Not performed	Satisfactory	Positive	ASC-US	Low-grade / equivocal squamous	
P00507	2018	2018-Q2	2018-04	Pre-pandemic	Cytology (Pap)	30-34	30	Not performed	Satisfactory	NILM	NILM	Negative (NILM)	
P00508	2018	2018-Q2	2018-04	Pre-pandemic	Cytology (Pap)	35-39	38	Not performed	Satisfactory	NILM	NILM	Negative (NILM)	
P00509	2018	2018-Q2	2018-04	Pre-pandemic	Cytology (Pap)	30-34	30	Not performed	Satisfactory	NILM	NILM	Negative (NILM)	
P00510	2018	2018-Q2	2018-04	Pre-pandemic	Cytology (Pap)	55-59	57	Not performed	Satisfactory	Positive	ASC-US	Low-grade / equivocal squamous	
P00511	2018	2018-Q2	2018-04	Pre-pandemic	Cytology (Pap)	30-34	32	Not performed	Satisfactory	NILM	NILM	Negative (NILM)	
P00512	2018	2018-Q2	2018-04	Pre-pandemic	Cytology (Pap)	60-64	63	Not performed	Satisfactory	NILM	NILM	Negative (NILM)	
P00513	2018	2018-Q2	2018-04	Pre-pandemic	Cytology (Pap)	60-64	61	Not performed	Satisfactory	NILM	NILM	Negative (NILM)	
P00514	2018	2018-Q2	2018-04	Pre-pandemic	Cytology (Pap)	40-44	44	Not performed	Satisfactory	NILM	NILM	Negative (NILM)	
P00515	2018	2018-Q2	2018-04	Pre-pandemic	Cytology (Pap)	35-39	36	Not performed	Satisfactory	NILM	NILM	Negative (NILM)	
P00516	2018	2018-Q2	2018-04	Pre-pandemic	Cytology (Pap)	30-34	30	Not performed	Satisfactory	NILM	NILM	Negative (NILM)	
P00517	2018	2018-Q2	2018-04	Pre-pandemic	Cytology (Pap)	50-54	50	Not performed	Satisfactory	NILM	NILM	Negative (NILM)	
P00518	2018	2018-Q2	2018-04	Pre-pandemic	Cytology (Pap)	40-44	40	Not performed	Satisfactory	NILM	NILM	Negative (NILM)	
P00519	2018	2018-Q2	2018-04	Pre-pandemic	Cytology (Pap)	40-44	41	Not performed	Satisfactory	NILM	NILM	Negative (NILM)	
P00520	2018	2018-Q2	2018-04	Pre-pandemic	Cytology (Pap)	40-44	41	Not performed	Satisfactory	NILM	NILM	Negative (NILM)	
P00521	2018	2018-Q2	2018-04	Pre-pandemic	Cytology (Pap)	Not recorded		Not performed	Satisfactory	NILM	NILM	Negative (NILM)	
P00522	2018	2018-Q2	2018-04	Pre-pandemic	Cytology (Pap)	60-64	61	Not performed	Satisfactory	NILM	NILM	Negative (NILM)	
P00523	2018	2018-Q2	2018-04	Pre-pandemic	Cytology (Pap)	50-54	52	Not performed	Satisfactory	Positive	LSIL	Low-grade / equivocal squamous	Candida
P00524	2018	2018-Q2	2018-04	Pre-pandemic	Cytology (Pap)	60-64	64	Not performed	Satisfactory	NILM	NILM	Negative (NILM)	
P00525	2018	2018-Q2	2018-04	Pre-pandemic	Cytology (Pap)	45-49	47	Not performed	Satisfactory	NILM	NILM	Negative (NILM)	
P00526	2018	2018-Q2	2018-04	Pre-pandemic	Cytology (Pap)	50-54	50	Not performed	Satisfactory	Positive	LSIL	Low-grade / equivocal squamous	
P00527	2018	2018-Q2	2018-04	Pre-pandemic	Cytology (Pap)	30-34	30	Not performed	Satisfactory	NILM	NILM	Negative (NILM)	
P00528	2018	2018-Q2	2018-04	Pre-pandemic	Cytology (Pap)	25-29	28	Not performed	Satisfactory	NILM	NILM	Negative (NILM)	
P00529	2018	2018-Q2	2018-04	Pre-pandemic	Cytology (Pap)	25-29	25	Not performed	Satisfactory	NILM	NILM	Negative (NILM)	
P00530	2018	2018-Q2	2018-04	Pre-pandemic	Cytology (Pap)	45-49	48	Not performed	Satisfactory	NILM	NILM	Negative (NILM)	
P00531	2018	2018-Q2	2018-04	Pre-pandemic	Cytology (Pap)	55-59	55	Not performed	Satisfactory	NILM	NILM	Negative (NILM)	
P00532	2018	2018-Q2	2018-04	Pre-pandemic	Cytology (Pap)	55-59	57	Not performed	Satisfactory	NILM	NILM	Negative (NILM)	
P00533	2018	2018-Q2	2018-04	Pre-pandemic	Cytology (Pap)	35-39	38	Not performed	Satisfactory	NILM	NILM	Negative (NILM)	
P00534	2018	2018-Q2	2018-04	Pre-pandemic	Cytology (Pap)	25-29	28	Not performed	Satisfactory	NILM	NILM	Negative (NILM)	
P00535	2018	2018-Q2	2018-04	Pre-pandemic	Cytology (Pap)	35-39	39	Not performed	Satisfactory	NILM	NILM	Negative (NILM)	
P00536	2018	2018-Q2	2018-04	Pre-pandemic	Cytology (Pap)	45-49	48	Not performed	Satisfactory	NILM	NILM	Negative (NILM)	
P00537	2018	2018-Q2	2018-04	Pre-pandemic	Cytology (Pap)	40-44	44	Not performed	Satisfactory	NILM	NILM	Negative (NILM)	
P00538	2018	2018-Q2	2018-04	Pre-pandemic	Cytology (Pap)	35-39	37	Not performed	Satisfactory	NILM	NILM	Negative (NILM)	
P00539	2018	2018-Q2	2018-04	Pre-pandemic	Cytology (Pap)	35-39	37	Not performed	Satisfactory	NILM	NILM	Negative (NILM)	
P00540	2018	2018-Q2	2018-04	Pre-pandemic	Cytology (Pap)	35-39	37	Not performed	Satisfactory	Positive	ASC-US	Low-grade / equivocal squamous	
P00541	2018	2018-Q2	2018-04	Pre-pandemic	Cytology (Pap)	35-39	35	Not performed	Satisfactory	NILM	NILM	Negative (NILM)	
P00542	2018	2018-Q2	2018-04	Pre-pandemic	Cytology (Pap)	55-59	57	Not performed	Satisfactory	NILM	NILM	Negative (NILM)	
P00543	2018	2018-Q2	2018-04	Pre-pandemic	Cytology (Pap)	60-64	64	Not performed	Satisfactory	Positive	LSIL	Low-grade / equivocal squamous	
P00544	2018	2018-Q2	2018-04	Pre-pandemic	Cytology (Pap)	35-39	36	Not performed	Satisfactory	NILM	NILM	Negative (NILM)	
P00545	2018	2018-Q2	2018-04	Pre-pandemic	Cytology (Pap)	30-34	30	Not performed	Satisfactory	NILM	NILM	Negative (NILM)	
P00546	2018	2018-Q2	2018-04	Pre-pandemic	Cytology (Pap)	Not recorded		Not performed	Satisfactory	NILM	NILM	Negative (NILM)	
P00547	2018	2018-Q2	2018-04	Pre-pandemic	Cytology (Pap)	55-59	55	Not performed	Satisfactory	NILM	NILM	Negative (NILM)	
P00548	2018	2018-Q2	2018-04	Pre-pandemic	Cytology (Pap)	30-34	31	Not performed	Satisfactory	Positive	ASC-US	Low-grade / equivocal squamous	
P00549	2018	2018-Q2	2018-04	Pre-pandemic	Cytology (Pap)	55-59	57	Not performed	Satisfactory	NILM	NILM	Negative (NILM)	
P00550	2018	2018-Q2	2018-04	Pre-pandemic	Cytology (Pap)	25-29	27	Not performed	Satisfactory	NILM	NILM	Negative (NILM)	
P00551	2018	2018-Q2	2018-04	Pre-pandemic	Cytology (Pap)	25-29	29	Not performed	Satisfactory	Positive	ASC-US	Low-grade / equivocal squamous	
P00552	2018	2018-Q2	2018-04	Pre-pandemic	Cytology (Pap)	60-64	60	Not performed	Satisfactory	NILM	NILM	Negative (NILM)	
P00553	2018	2018-Q2	2018-04	Pre-pandemic	Cytology (Pap)	40-44	42	Not performed	Satisfactory	NILM	NILM	Negative (NILM)	
P00554	2018	2018-Q2	2018-04	Pre-pandemic	Cytology (Pap)	45-49	47	Not performed	Satisfactory	NILM	NILM	Negative (NILM)	
P00555	2018	2018-Q2	2018-04	Pre-pandemic	Cytology (Pap)	35-39	39	Not performed	Satisfactory	NILM	NILM	Negative (NILM)	
P00556	2018	2018-Q2	2018-04	Pre-pandemic	Cytology (Pap)	55-59	59	Not performed	Satisfactory	NILM	NILM	Negative (NILM)	
P00557	2018	2018-Q2	2018-04	Pre-pandemic	Cytology (Pap)	45-49	46	Not performed	Satisfactory	NILM	NILM	Negative (NILM)	
P00558	2018	2018-Q2	2018-04	Pre-pandemic	Cytology (Pap)	45-49	46	Not performed	Satisfactory	NILM	NILM	Negative (NILM)	

patient_id	year	quarter	month	period	primary_test	age_group	age_years	hpv_result	adequacy	result	bethesda	grade	infection
P00559	2018	2018-Q2	2018-04	Pre-pandemic	Cytology (Pap)	35-39	38	Not performed	Satisfactory	NILM	NILM	Negative (NILM)	
P00560	2018	2018-Q2	2018-04	Pre-pandemic	Cytology (Pap)	35-39	38	Not performed	Satisfactory	NILM	NILM	Negative (NILM)	
P00561	2018	2018-Q2	2018-04	Pre-pandemic	Cytology (Pap)	30-34	34	Not performed	Satisfactory	NILM	NILM	Negative (NILM)	
P00562	2018	2018-Q2	2018-04	Pre-pandemic	Cytology (Pap)	35-39	35	Not performed	Satisfactory	NILM	NILM	Negative (NILM)	
P00563	2018	2018-Q2	2018-04	Pre-pandemic	Cytology (Pap)	45-49	49	Not performed	Satisfactory	NILM	NILM	Negative (NILM)	
P00564	2018	2018-Q2	2018-04	Pre-pandemic	Cytology (Pap)	45-49	47	Not performed	Satisfactory	NILM	NILM	Negative (NILM)	
P00565	2018	2018-Q2	2018-04	Pre-pandemic	Cytology (Pap)	Not recorded		Not performed	Satisfactory	NILM	NILM	Negative (NILM)	
P00566	2018	2018-Q2	2018-04	Pre-pandemic	Cytology (Pap)	55-59	55	Not performed	Satisfactory	NILM	NILM	Negative (NILM)	
P00567	2018	2018-Q2	2018-04	Pre-pandemic	Cytology (Pap)	55-59	58	Not performed	Satisfactory	NILM	NILM	Negative (NILM)	Trichomonas
P00568	2018	2018-Q2	2018-04	Pre-pandemic	Cytology (Pap)	50-54	50	Not performed	Satisfactory	NILM	NILM	Negative (NILM)	
P00569	2018	2018-Q2	2018-04	Pre-pandemic	Cytology (Pap)	40-44	40	Not performed	Satisfactory	NILM	NILM	Negative (NILM)	
P00570	2018	2018-Q2	2018-04	Pre-pandemic	Cytology (Pap)	35-39	37	Not performed	Satisfactory	NILM	NILM	Negative (NILM)	
P00571	2018	2018-Q2	2018-04	Pre-pandemic	Cytology (Pap)	35-39	38	Not performed	Satisfactory	NILM	NILM	Negative (NILM)	Gardnerella
P00572	2018	2018-Q2	2018-04	Pre-pandemic	Cytology (Pap)	60-64	63	Not performed	Satisfactory	NILM	NILM	Negative (NILM)	
P00573	2018	2018-Q2	2018-04	Pre-pandemic	Cytology (Pap)	35-39	38	Not performed	Satisfactory	Positive	ASC-US	Low-grade / equivocal squamous	Candida
P00574	2018	2018-Q2	2018-04	Pre-pandemic	Cytology (Pap)	40-44	42	Not performed	Satisfactory	NILM	NILM	Negative (NILM)	
P00575	2018	2018-Q2	2018-04	Pre-pandemic	Cytology (Pap)	35-39	39	Not performed	Satisfactory	NILM	NILM	Negative (NILM)	
P00576	2018	2018-Q2	2018-04	Pre-pandemic	Cytology (Pap)	40-44	41	Not performed	Satisfactory	Positive	ASC-US	Low-grade / equivocal squamous	
P00577	2018	2018-Q2	2018-04	Pre-pandemic	Cytology (Pap)	25-29	29	Not performed	Satisfactory	NILM	NILM	Negative (NILM)	
P00578	2018	2018-Q2	2018-04	Pre-pandemic	Cytology (Pap)	25-29	29	Not performed	Satisfactory	NILM	NILM	Negative (NILM)	
P00579	2018	2018-Q2	2018-04	Pre-pandemic	Cytology (Pap)	40-44	40	Not performed	Satisfactory	NILM	NILM	Negative (NILM)	
P00580	2018	2018-Q2	2018-04	Pre-pandemic	Cytology (Pap)	35-39	37	Not performed	Satisfactory	NILM	NILM	Negative (NILM)	
P00581	2018	2018-Q2	2018-04	Pre-pandemic	Cytology (Pap)	40-44	43	Not performed	Satisfactory	NILM	NILM	Negative (NILM)	
P00582	2018	2018-Q2	2018-04	Pre-pandemic	Cytology (Pap)	35-39	36	Not performed	Satisfactory	NILM	NILM	Negative (NILM)	
P00583	2018	2018-Q2	2018-04	Pre-pandemic	Cytology (Pap)	40-44	40	Not performed	Satisfactory	NILM	NILM	Negative (NILM)	
P00584	2018	2018-Q2	2018-04	Pre-pandemic	Cytology (Pap)	60-64	64	Not performed	Satisfactory	NILM	NILM	Negative (NILM)	
P00585	2018	2018-Q2	2018-04	Pre-pandemic	Cytology (Pap)	50-54	52	Not performed	Satisfactory	NILM	NILM	Negative (NILM)	
P00586	2018	2018-Q2	2018-04	Pre-pandemic	Cytology (Pap)	Not recorded		Not performed	Satisfactory	NILM	NILM	Negative (NILM)	
P00587	2018	2018-Q2	2018-04	Pre-pandemic	Cytology (Pap)	45-49	48	Not performed	Satisfactory	NILM	NILM	Negative (NILM)	
P00588	2018	2018-Q2	2018-04	Pre-pandemic	Cytology (Pap)	30-34	33	Not performed	Satisfactory	NILM	NILM	Negative (NILM)	
P00589	2018	2018-Q2	2018-04	Pre-pandemic	Cytology (Pap)	45-49	47	Not performed	Satisfactory	NILM	NILM	Negative (NILM)	
P00590	2018	2018-Q2	2018-04	Pre-pandemic	Cytology (Pap)	60-64	64	Not performed	Satisfactory	NILM	NILM	Negative (NILM)	
P00591	2018	2018-Q2	2018-04	Pre-pandemic	Cytology (Pap)	25-29	25	Not performed	Satisfactory	NILM	NILM	Negative (NILM)	
P00592	2018	2018-Q2	2018-04	Pre-pandemic	Cytology (Pap)	25-29	25	Not performed	Satisfactory	Positive	HSIL	High-grade squamous	
P00593	2018	2018-Q2	2018-04	Pre-pandemic	Cytology (Pap)	25-29	25	Not performed	Satisfactory	NILM	NILM	Negative (NILM)	
P00594	2018	2018-Q2	2018-04	Pre-pandemic	Cytology (Pap)	25-29	29	Not performed	Satisfactory	NILM	NILM	Negative (NILM)	
P00595	2018	2018-Q2	2018-04	Pre-pandemic	Cytology (Pap)	55-59	55	Not performed	Satisfactory	NILM	NILM	Negative (NILM)	
P00596	2018	2018-Q2	2018-04	Pre-pandemic	Cytology (Pap)	40-44	41	Not performed	Satisfactory	NILM	NILM	Negative (NILM)	
P00597	2018	2018-Q2	2018-04	Pre-pandemic	Cytology (Pap)	45-49	48	Not performed	Satisfactory	NILM	NILM	Negative (NILM)	Gardnerella
P00598	2018	2018-Q2	2018-04	Pre-pandemic	Cytology (Pap)	30-34	31	Not performed	Satisfactory	NILM	NILM	Negative (NILM)	
P00599	2018	2018-Q2	2018-04	Pre-pandemic	Cytology (Pap)	35-39	38	Not performed	Satisfactory	Positive	ASC-US	Low-grade / equivocal squamous	
P00600	2018	2018-Q2	2018-04	Pre-pandemic	Cytology (Pap)	25-29	29	Not performed	Satisfactory	NILM	NILM	Negative (NILM)	
P00601	2018	2018-Q2	2018-04	Pre-pandemic	Cytology (Pap)	35-39	39	Not performed	Satisfactory	NILM	NILM	Negative (NILM)	
P00602	2018	2018-Q2	2018-04	Pre-pandemic	Cytology (Pap)	45-49	45	Not performed	Satisfactory	Positive	ASC-US	Low-grade / equivocal squamous	
P00603	2018	2018-Q2	2018-04	Pre-pandemic	Cytology (Pap)	35-39	35	Not performed	Unsatisfactory	Unsatisfactory	Unsatisfactory	Unsatisfactory	
P00604	2018	2018-Q2	2018-04	Pre-pandemic	Cytology (Pap)	40-44	40	Not performed	Unsatisfactory	Unsatisfactory	Unsatisfactory	Unsatisfactory	
P00605	2018	2018-Q2	2018-04	Pre-pandemic	Cytology (Pap)	35-39	37	Not performed	Satisfactory	NILM	NILM	Negative (NILM)	
P00606	2018	2018-Q2	2018-04	Pre-pandemic	Cytology (Pap)	50-54	51	Not performed	Unsatisfactory	Unsatisfactory	Unsatisfactory	Unsatisfactory	
P00607	2018	2018-Q2	2018-04	Pre-pandemic	Cytology (Pap)	40-44	41	Not performed	Satisfactory	NILM	NILM	Negative (NILM)	
P00608	2018	2018-Q2	2018-04	Pre-pandemic	Cytology (Pap)	45-49	45	Not performed	Satisfactory	NILM	NILM	Negative (NILM)	
P00609	2018	2018-Q2	2018-04	Pre-pandemic	Cytology (Pap)	35-39	35	Not performed	Satisfactory	NILM	NILM	Negative (NILM)	
P00610	2018	2018-Q2	2018-04	Pre-pandemic	Cytology (Pap)	60-64	63	Not performed	Satisfactory	Positive	ASC-US	Low-grade / equivocal squamous	
P00611	2018	2018-Q2	2018-04	Pre-pandemic	Cytology (Pap)	40-44	42	Not performed	Satisfactory	NILM	NILM	Negative (NILM)	
P00612	2018	2018-Q2	2018-04	Pre-pandemic	Cytology (Pap)	25-29	26	Not performed	Satisfactory	NILM	NILM	Negative (NILM)	
P00613	2018	2018-Q2	2018-04	Pre-pandemic	Cytology (Pap)	30-34	31	Not performed	Satisfactory	NILM	NILM	Negative (NILM)	
P00614	2018	2018-Q2	2018-04	Pre-pandemic	Cytology (Pap)	40-44	42	Not performed	Satisfactory	NILM	NILM	Negative (NILM)	
P00615	2018	2018-Q2	2018-04	Pre-pandemic	Cytology (Pap)	45-49	46	Not performed	Satisfactory	NILM	NILM	Negative (NILM)	
P00616	2018	2018-Q2	2018-04	Pre-pandemic	Cytology (Pap)	40-44	43	Not performed	Satisfactory	NILM	NILM	Negative (NILM)	
P00617	2018	2018-Q2	2018-04	Pre-pandemic	Cytology (Pap)	Not recorded		Not performed	Satisfactory	NILM	NILM	Negative (NILM)	
P00618	2018	2018-Q2	2018-04	Pre-pandemic	Cytology (Pap)	40-44	40	Not performed	Satisfactory	NILM	NILM	Negative (NILM)	
P00619	2018	2018-Q2	2018-04	Pre-pandemic	Cytology (Pap)	45-49	47	Not performed	Satisfactory	NILM	NILM	Negative (NILM)	
P00620	2018	2018-Q2	2018-04	Pre-pandemic	Cytology (Pap)	45-49	46	Not performed	Satisfactory	NILM	NILM	Negative (NILM)	
P00621	2018	2018-Q2	2018-04	Pre-pandemic	Cytology (Pap)	40-44	43	Not performed	Satisfactory	NILM	NILM	Negative (NILM)	
P00622	2018	2018-Q2	2018-04	Pre-pandemic	Cytology (Pap)	30-34	33	Not performed	Satisfactory	NILM	NILM	Negative (NILM)	
P00623	2018	2018-Q2	2018-04	Pre-pandemic	Cytology (Pap)	40-44	40	Not performed	Satisfactory	Positive	ASC-US	Low-grade / equivocal squamous	
P00624	2018	2018-Q2	2018-04	Pre-pandemic	Cytology (Pap)	50-54	50	Not performed	Satisfactory	NILM	NILM	Negative (NILM)	
P00625	2018	2018-Q2	2018-04	Pre-pandemic	Cytology (Pap)	50-54	52	Not performed	Unsatisfactory	Unsatisfactory	Unsatisfactory	Unsatisfactory	
P00626	2018	2018-Q2	2018-04	Pre-pandemic	Cytology (Pap)	40-44	44	Not performed	Unsatisfactory	Unsatisfactory	Unsatisfactory	Unsatisfactory	
P00627	2018	2018-Q2	2018-04	Pre-pandemic	Cytology (Pap)	40-44	42	Not performed	Satisfactory	NILM	NILM	Negative (NILM)	
P00628	2018	2018-Q2	2018-05	Pre-pandemic	Cytology (Pap)	50-54	52	Not performed	Unsatisfactory	Unsatisfactory	Unsatisfactory	Unsatisfactory	
P00629	2018	2018-Q2	2018-05	Pre-pandemic	Cytology (Pap)	35-39	36	Not performed	Satisfactory	NILM	NILM	Negative (NILM)	
P00630	2018	2018-Q2	2018-05	Pre-pandemic	Cytology (Pap)	25-29	27	Not performed	Satisfactory	NILM	NILM	Negative (NILM)	
P00631	2018	2018-Q2	2018-05	Pre-pandemic	Cytology (Pap)	40-44	40	Not performed	Satisfactory	NILM	NILM	Negative (NILM)	Candida
P00632	2018	2018-Q2	2018-05	Pre-pandemic	Cytology (Pap)	25-29	25	Not performed	Satisfactory	NILM	NILM	Negative (NILM)	
P00633	2018	2018-Q2	2018-05	Pre-pandemic	Cytology (Pap)	40-44	44	Not performed	Satisfactory	NILM	NILM	Negative (NILM)	
P00634	2018	2018-Q2	2018-05	Pre-pandemic	Cytology (Pap)	45-49	45	Not performed	Satisfactory	Positive	LSIL	Low-grade / equivocal squamous	
P00635	2018	2018-Q2	2018-05	Pre-pandemic	Cytology (Pap)	35-39	37	Not performed	Satisfactory	NILM	NILM	Negative (NILM)	
P00636	2018	2018-Q2	2018-05	Pre-pandemic	Cytology (Pap)	45-49	48	Not performed	Satisfactory	NILM	NILM	Negative (NILM)	
P00637	2018	2018-Q2	2018-05	Pre-pandemic	Cytology (Pap)	55-59	58	Not performed	Satisfactory	NILM	NILM	Negative (NILM)	
P00638	2018	2018-Q2	2018-05	Pre-pandemic	Cytology (Pap)	55-59	56	Not performed	Satisfactory	NILM	NILM	Negative (NILM)	

patient_id	year	quarter	month	period	primary_test	age_group	age_years	hpv_result	adequacy	result	bethesda	grade	infection
P00639	2018	2018-Q2	2018-05	Pre-pandemic	Cytology (Pap)	50-54	52	Not performed	Satisfactory	NILM	NILM	Negative (NILM)	
P00640	2018	2018-Q2	2018-05	Pre-pandemic	Cytology (Pap)	45-49	49	Not performed	Satisfactory	NILM	NILM	Negative (NILM)	
P00641	2018	2018-Q2	2018-05	Pre-pandemic	Cytology (Pap)	30-34	33	Not performed	Satisfactory	Positive	LSIL	Low-grade / equivocal squamous	
P00642	2018	2018-Q2	2018-05	Pre-pandemic	Cytology (Pap)	45-49	46	Not performed	Satisfactory	NILM	NILM	Negative (NILM)	
P00643	2018	2018-Q2	2018-05	Pre-pandemic	Cytology (Pap)	35-39	39	Not performed	Satisfactory	Positive	ASC-H	High-grade squamous	
P00644	2018	2018-Q2	2018-05	Pre-pandemic	Cytology (Pap)	40-44	42	Not performed	Satisfactory	NILM	NILM	Negative (NILM)	
P00645	2018	2018-Q2	2018-05	Pre-pandemic	Cytology (Pap)	35-39	38	Not performed	Satisfactory	NILM	NILM	Negative (NILM)	
P00646	2018	2018-Q2	2018-05	Pre-pandemic	Cytology (Pap)	30-34	32	Not performed	Satisfactory	NILM	NILM	Negative (NILM)	
P00647	2018	2018-Q2	2018-05	Pre-pandemic	Cytology (Pap)	60-64	64	Not performed	Satisfactory	NILM	NILM	Negative (NILM)	
P00648	2018	2018-Q2	2018-05	Pre-pandemic	Cytology (Pap)	35-39	37	Not performed	Satisfactory	NILM	NILM	Negative (NILM)	
P00649	2018	2018-Q2	2018-05	Pre-pandemic	Cytology (Pap)	40-44	41	Not performed	Satisfactory	Positive	LSIL	Low-grade / equivocal squamous	
P00650	2018	2018-Q2	2018-05	Pre-pandemic	Cytology (Pap)	50-54	53	Not performed	Satisfactory	NILM	NILM	Negative (NILM)	
P00651	2018	2018-Q2	2018-05	Pre-pandemic	Cytology (Pap)	35-39	37	Not performed	Satisfactory	NILM	NILM	Negative (NILM)	
P00652	2018	2018-Q2	2018-05	Pre-pandemic	Cytology (Pap)	30-34	31	Not performed	Satisfactory	NILM	NILM	Negative (NILM)	
P00653	2018	2018-Q2	2018-05	Pre-pandemic	Cytology (Pap)	25-29	26	Not performed	Satisfactory	NILM	NILM	Negative (NILM)	
P00654	2018	2018-Q2	2018-05	Pre-pandemic	Cytology (Pap)	60-64	62	Not performed	Satisfactory	NILM	NILM	Negative (NILM)	
P00655	2018	2018-Q2	2018-05	Pre-pandemic	Cytology (Pap)	25-29	29	Not performed	Satisfactory	NILM	NILM	Negative (NILM)	
P00656	2018	2018-Q2	2018-05	Pre-pandemic	Cytology (Pap)	45-49	46	Not performed	Satisfactory	NILM	NILM	Negative (NILM)	
P00657	2018	2018-Q2	2018-05	Pre-pandemic	Cytology (Pap)	50-54	54	Not performed	Satisfactory	NILM	NILM	Negative (NILM)	
P00658	2018	2018-Q2	2018-05	Pre-pandemic	Cytology (Pap)	40-44	41	Not performed	Satisfactory	NILM	NILM	Negative (NILM)	
P00659	2018	2018-Q2	2018-05	Pre-pandemic	Cytology (Pap)	55-59	59	Not performed	Satisfactory	NILM	NILM	Negative (NILM)	
P00660	2018	2018-Q2	2018-05	Pre-pandemic	Cytology (Pap)	50-54	52	Not performed	Satisfactory	NILM	NILM	Negative (NILM)	
P00661	2018	2018-Q2	2018-05	Pre-pandemic	Cytology (Pap)	50-54	54	Not performed	Satisfactory	Positive	LSIL	Low-grade / equivocal squamous	
P00662	2018	2018-Q2	2018-05	Pre-pandemic	Cytology (Pap)	30-34	34	Not performed	Satisfactory	NILM	NILM	Negative (NILM)	
P00663	2018	2018-Q2	2018-05	Pre-pandemic	Cytology (Pap)	40-44	40	Not performed	Satisfactory	NILM	NILM	Negative (NILM)	
P00664	2018	2018-Q2	2018-05	Pre-pandemic	Cytology (Pap)	45-49	46	Not performed	Satisfactory	NILM	NILM	Negative (NILM)	Candida
P00665	2018	2018-Q2	2018-05	Pre-pandemic	Cytology (Pap)	45-49	49	Not performed	Satisfactory	NILM	NILM	Negative (NILM)	
P00666	2018	2018-Q2	2018-05	Pre-pandemic	Cytology (Pap)	45-49	48	Not performed	Satisfactory	NILM	NILM	Negative (NILM)	
P00667	2018	2018-Q2	2018-05	Pre-pandemic	Cytology (Pap)	40-44	41	Not performed	Satisfactory	NILM	NILM	Negative (NILM)	
P00668	2018	2018-Q2	2018-05	Pre-pandemic	Cytology (Pap)	55-59	59	Not performed	Satisfactory	NILM	NILM	Negative (NILM)	
P00669	2018	2018-Q2	2018-05	Pre-pandemic	Cytology (Pap)	30-34	30	Not performed	Satisfactory	NILM	NILM	Negative (NILM)	
P00670	2018	2018-Q2	2018-05	Pre-pandemic	Cytology (Pap)	40-44	42	Not performed	Satisfactory	NILM	NILM	Negative (NILM)	
P00671	2018	2018-Q2	2018-05	Pre-pandemic	Cytology (Pap)	25-29	28	Not performed	Satisfactory	NILM	NILM	Negative (NILM)	
P00672	2018	2018-Q2	2018-05	Pre-pandemic	Cytology (Pap)	50-54	54	Not performed	Satisfactory	NILM	NILM	Negative (NILM)	
P00673	2018	2018-Q2	2018-05	Pre-pandemic	Cytology (Pap)	40-44	40	Not performed	Satisfactory	NILM	NILM	Negative (NILM)	
P00674	2018	2018-Q2	2018-05	Pre-pandemic	Cytology (Pap)	25-29	25	Not performed	Satisfactory	NILM	NILM	Negative (NILM)	
P00675	2018	2018-Q2	2018-05	Pre-pandemic	Cytology (Pap)	50-54	53	Not performed	Satisfactory	NILM	NILM	Negative (NILM)	
P00676	2018	2018-Q2	2018-05	Pre-pandemic	Cytology (Pap)	40-44	40	Not performed	Satisfactory	NILM	NILM	Negative (NILM)	Candida
P00677	2018	2018-Q2	2018-05	Pre-pandemic	Cytology (Pap)	50-54	53	Not performed	Satisfactory	NILM	NILM	Negative (NILM)	
P00678	2018	2018-Q2	2018-05	Pre-pandemic	Cytology (Pap)	35-39	38	Not performed	Unsatisfactory	Unsatisfactory	Unsatisfactory	Unsatisfactory	
P00679	2018	2018-Q2	2018-05	Pre-pandemic	Cytology (Pap)	Not recorded		Not performed	Satisfactory	NILM	NILM	Negative (NILM)	
P00680	2018	2018-Q2	2018-05	Pre-pandemic	Cytology (Pap)	45-49	45	Not performed	Satisfactory	NILM	NILM	Negative (NILM)	
P00681	2018	2018-Q2	2018-05	Pre-pandemic	Cytology (Pap)	35-39	35	Not performed	Unsatisfactory	Unsatisfactory	Unsatisfactory	Unsatisfactory	
P00682	2018	2018-Q2	2018-05	Pre-pandemic	Cytology (Pap)	30-34	30	Not performed	Satisfactory	NILM	NILM	Negative (NILM)	
P00683	2018	2018-Q2	2018-05	Pre-pandemic	Cytology (Pap)	40-44	41	Not performed	Satisfactory	NILM	NILM	Negative (NILM)	
P00684	2018	2018-Q2	2018-05	Pre-pandemic	Cytology (Pap)	45-49	45	Not performed	Satisfactory	Positive	LSIL	Low-grade / equivocal squamous	
P00685	2018	2018-Q2	2018-05	Pre-pandemic	Cytology (Pap)	50-54	51	Not performed	Satisfactory	Positive	ASC-US	Low-grade / equivocal squamous	
P00686	2018	2018-Q2	2018-05	Pre-pandemic	Cytology (Pap)	45-49	47	Not performed	Satisfactory	NILM	NILM	Negative (NILM)	
P00687	2018	2018-Q2	2018-05	Pre-pandemic	Cytology (Pap)	35-39	36	Not performed	Satisfactory	NILM	NILM	Negative (NILM)	
P00688	2018	2018-Q2	2018-05	Pre-pandemic	Cytology (Pap)	35-39	35	Not performed	Satisfactory	NILM	NILM	Negative (NILM)	
P00689	2018	2018-Q2	2018-05	Pre-pandemic	Cytology (Pap)	40-44	41	Not performed	Unsatisfactory	Unsatisfactory	Unsatisfactory	Unsatisfactory	
P00690	2018	2018-Q2	2018-05	Pre-pandemic	Cytology (Pap)	35-39	37	Not performed	Satisfactory	Positive	ASC-US	Low-grade / equivocal squamous	
P00691	2018	2018-Q2	2018-05	Pre-pandemic	Cytology (Pap)	40-44	42	Not performed	Satisfactory	NILM	NILM	Negative (NILM)	
P00692	2018	2018-Q2	2018-05	Pre-pandemic	Cytology (Pap)	35-39	36	Not performed	Satisfactory	NILM	NILM	Negative (NILM)	Gardnerella
P00693	2018	2018-Q2	2018-05	Pre-pandemic	Cytology (Pap)	30-34	34	Not performed	Satisfactory	NILM	NILM	Negative (NILM)	
P00694	2018	2018-Q2	2018-05	Pre-pandemic	Cytology (Pap)	30-34	31	Not performed	Satisfactory	NILM	NILM	Negative (NILM)	
P00695	2018	2018-Q2	2018-05	Pre-pandemic	Cytology (Pap)	40-44	40	Not performed	Satisfactory	NILM	NILM	Negative (NILM)	
P00696	2018	2018-Q2	2018-05	Pre-pandemic	Cytology (Pap)	60-64	64	Not performed	Unsatisfactory	Unsatisfactory	Unsatisfactory	Unsatisfactory	
P00697	2018	2018-Q2	2018-05	Pre-pandemic	Cytology (Pap)	45-49	45	Not performed	Satisfactory	NILM	NILM	Negative (NILM)	
P00698	2018	2018-Q2	2018-05	Pre-pandemic	Cytology (Pap)	50-54	52	Not performed	Satisfactory	Positive	HSIL	High-grade squamous	
P00699	2018	2018-Q2	2018-05	Pre-pandemic	Cytology (Pap)	40-44	40	Not performed	Satisfactory	Positive	ASC-US	Low-grade / equivocal squamous	
P00700	2018	2018-Q2	2018-05	Pre-pandemic	Cytology (Pap)	35-39	36	Not performed	Satisfactory	Positive	HSIL	High-grade squamous	
P00701	2018	2018-Q2	2018-05	Pre-pandemic	Cytology (Pap)	60-64	62	Not performed	Satisfactory	NILM	NILM	Negative (NILM)	
P00702	2018	2018-Q2	2018-05	Pre-pandemic	Cytology (Pap)	30-34	33	Not performed	Satisfactory	NILM	NILM	Negative (NILM)	
P00703	2018	2018-Q2	2018-05	Pre-pandemic	Cytology (Pap)	25-29	27	Not performed	Satisfactory	NILM	NILM	Negative (NILM)	Candida
P00704	2018	2018-Q2	2018-05	Pre-pandemic	Cytology (Pap)	45-49	49	Not performed	Satisfactory	Positive	HSIL	High-grade squamous	
P00705	2018	2018-Q2	2018-05	Pre-pandemic	Cytology (Pap)	Not recorded		Not performed	Satisfactory	NILM	NILM	Negative (NILM)	
P00706	2018	2018-Q2	2018-05	Pre-pandemic	Cytology (Pap)	Not recorded		Not performed	Satisfactory	NILM	NILM	Negative (NILM)	
P00707	2018	2018-Q2	2018-05	Pre-pandemic	Cytology (Pap)	40-44	40	Not performed	Satisfactory	NILM	NILM	Negative (NILM)	
P00708	2018	2018-Q2	2018-05	Pre-pandemic	Cytology (Pap)	45-49	48	Not performed	Satisfactory	NILM	NILM	Negative (NILM)	
P00709	2018	2018-Q2	2018-05	Pre-pandemic	Cytology (Pap)	55-59	57	Not performed	Satisfactory	Positive	LSIL	Low-grade / equivocal squamous	
P00710	2018	2018-Q2	2018-05	Pre-pandemic	Cytology (Pap)	55-59	58	Not performed	Satisfactory	NILM	NILM	Negative (NILM)	
P00711	2018	2018-Q2	2018-05	Pre-pandemic	Cytology (Pap)	45-49	49	Not performed	Satisfactory	NILM	NILM	Negative (NILM)	
P00712	2018	2018-Q2	2018-05	Pre-pandemic	Cytology (Pap)	35-39	39	Not performed	Satisfactory	NILM	NILM	Negative (NILM)	
P00713	2018	2018-Q2	2018-05	Pre-pandemic	Cytology (Pap)	45-49	47	Not performed	Satisfactory	NILM	NILM	Negative (NILM)	
P00714	2018	2018-Q2	2018-05	Pre-pandemic	Cytology (Pap)	60-64	61	Not performed	Satisfactory	NILM	NILM	Negative (NILM)	
P00715	2018	2018-Q2	2018-05	Pre-pandemic	Cytology (Pap)	25-29	27	Not performed	Satisfactory	NILM	NILM	Negative (NILM)	
P00716	2018	2018-Q2	2018-05	Pre-pandemic	Cytology (Pap)	60-64	61	Not performed	Satisfactory	NILM	NILM	Negative (NILM)	
P00717	2018	2018-Q2	2018-05	Pre-pandemic	Cytology (Pap)	55-59	55	Not performed	Satisfactory	NILM	NILM	Negative (NILM)	
P00718	2018	2018-Q2	2018-05	Pre-pandemic	Cytology (Pap)	25-29	27	Not performed	Satisfactory	NILM	NILM	Negative (NILM)	

patient_id	year	quarter	month	period	primary_test	age_group	age_years	hpv_result	adequacy	result	bethesda	grade	infection
P00719	2018	2018-Q2	2018-05	Pre-pandemic	Cytology (Pap)	50-54	54	Not performed	Satisfactory	NILM	NILM	Negative (NILM)	
P00720	2018	2018-Q2	2018-05	Pre-pandemic	Cytology (Pap)	40-44	41	Not performed	Satisfactory	NILM	NILM	Negative (NILM)	
P00721	2018	2018-Q2	2018-05	Pre-pandemic	Cytology (Pap)	40-44	41	Not performed	Satisfactory	NILM	NILM	Negative (NILM)	
P00722	2018	2018-Q2	2018-05	Pre-pandemic	Cytology (Pap)	60-64	64	Not performed	Satisfactory	Positive	ASC-US	Low-grade / equivocal squamous	
P00723	2018	2018-Q2	2018-05	Pre-pandemic	Cytology (Pap)	40-44	44	Not performed	Satisfactory	NILM	NILM	Negative (NILM)	
P00724	2018	2018-Q2	2018-05	Pre-pandemic	Cytology (Pap)	55-59	58	Not performed	Satisfactory	NILM	NILM	Negative (NILM)	
P00725	2018	2018-Q2	2018-05	Pre-pandemic	Cytology (Pap)	55-59	58	Not performed	Satisfactory	NILM	NILM	Negative (NILM)	
P00726	2018	2018-Q2	2018-05	Pre-pandemic	Cytology (Pap)	60-64	61	Not performed	Unsatisfactory	Unsatisfactory	Unsatisfactory	Unsatisfactory	
P00727	2018	2018-Q2	2018-05	Pre-pandemic	Cytology (Pap)	30-34	34	Not performed	Satisfactory	Positive	ASC-US	Low-grade / equivocal squamous	
P00728	2018	2018-Q2	2018-05	Pre-pandemic	Cytology (Pap)	30-34	32	Not performed	Satisfactory	NILM	NILM	Negative (NILM)	
P00729	2018	2018-Q2	2018-05	Pre-pandemic	Cytology (Pap)	60-64	64	Not performed	Satisfactory	NILM	NILM	Negative (NILM)	
P00730	2018	2018-Q2	2018-05	Pre-pandemic	Cytology (Pap)	35-39	36	Not performed	Satisfactory	NILM	NILM	Negative (NILM)	
P00731	2018	2018-Q2	2018-05	Pre-pandemic	Cytology (Pap)	60-64	64	Not performed	Satisfactory	NILM	NILM	Negative (NILM)	
P00732	2018	2018-Q2	2018-05	Pre-pandemic	Cytology (Pap)	40-44	44	Not performed	Satisfactory	NILM	NILM	Negative (NILM)	
P00733	2018	2018-Q2	2018-05	Pre-pandemic	Cytology (Pap)	50-54	52	Not performed	Satisfactory	NILM	NILM	Negative (NILM)	
P00734	2018	2018-Q2	2018-05	Pre-pandemic	Cytology (Pap)	60-64	60	Not performed	Satisfactory	Positive	ASC-US	Low-grade / equivocal squamous	
P00735	2018	2018-Q2	2018-05	Pre-pandemic	Cytology (Pap)	25-29	25	Not performed	Satisfactory	NILM	NILM	Negative (NILM)	
P00736	2018	2018-Q2	2018-05	Pre-pandemic	Cytology (Pap)	Not recorded		Not performed	Satisfactory	NILM	NILM	Negative (NILM)	
P00737	2018	2018-Q2	2018-05	Pre-pandemic	Cytology (Pap)	55-59	59	Not performed	Satisfactory	NILM	NILM	Negative (NILM)	
P00738	2018	2018-Q2	2018-05	Pre-pandemic	Cytology (Pap)	60-64	64	Not performed	Satisfactory	NILM	NILM	Negative (NILM)	
P00739	2018	2018-Q2	2018-05	Pre-pandemic	Cytology (Pap)	45-49	47	Not performed	Satisfactory	NILM	NILM	Negative (NILM)	
P00740	2018	2018-Q2	2018-05	Pre-pandemic	Cytology (Pap)	40-44	41	Not performed	Satisfactory	NILM	NILM	Negative (NILM)	
P00741	2018	2018-Q2	2018-05	Pre-pandemic	Cytology (Pap)	60-64	61	Not performed	Satisfactory	NILM	NILM	Negative (NILM)	
P00742	2018	2018-Q2	2018-05	Pre-pandemic	Cytology (Pap)	45-49	48	Not performed	Satisfactory	NILM	NILM	Negative (NILM)	
P00743	2018	2018-Q2	2018-05	Pre-pandemic	Cytology (Pap)	45-49	45	Not performed	Satisfactory	NILM	NILM	Negative (NILM)	
P00744	2018	2018-Q2	2018-05	Pre-pandemic	Cytology (Pap)	30-34	30	Not performed	Satisfactory	NILM	NILM	Negative (NILM)	
P00745	2018	2018-Q2	2018-05	Pre-pandemic	Cytology (Pap)	35-39	39	Not performed	Unsatisfactory	Unsatisfactory	Unsatisfactory	Unsatisfactory	
P00746	2018	2018-Q2	2018-05	Pre-pandemic	Cytology (Pap)	30-34	32	Not performed	Satisfactory	NILM	NILM	Negative (NILM)	
P00747	2018	2018-Q2	2018-05	Pre-pandemic	Cytology (Pap)	45-49	49	Not performed	Satisfactory	NILM	NILM	Negative (NILM)	
P00748	2018	2018-Q2	2018-05	Pre-pandemic	Cytology (Pap)	35-39	37	Not performed	Satisfactory	NILM	NILM	Negative (NILM)	
P00749	2018	2018-Q2	2018-05	Pre-pandemic	Cytology (Pap)	45-49	47	Not performed	Satisfactory	NILM	NILM	Negative (NILM)	
P00750	2018	2018-Q2	2018-05	Pre-pandemic	Cytology (Pap)	25-29	26	Not performed	Satisfactory	NILM	NILM	Negative (NILM)	
P00751	2018	2018-Q2	2018-05	Pre-pandemic	Cytology (Pap)	25-29	29	Not performed	Unsatisfactory	Unsatisfactory	Unsatisfactory	Unsatisfactory	
P00752	2018	2018-Q2	2018-05	Pre-pandemic	Cytology (Pap)	40-44	42	Not performed	Satisfactory	NILM	NILM	Negative (NILM)	
P00753	2018	2018-Q2	2018-05	Pre-pandemic	Cytology (Pap)	45-49	45	Not performed	Satisfactory	NILM	NILM	Negative (NILM)	
P00754	2018	2018-Q2	2018-05	Pre-pandemic	Cytology (Pap)	40-44	44	Not performed	Satisfactory	Positive	HSIL	High-grade squamous	
P00755	2018	2018-Q2	2018-05	Pre-pandemic	Cytology (Pap)	35-39	37	Not performed	Satisfactory	NILM	NILM	Negative (NILM)	
P00756	2018	2018-Q2	2018-05	Pre-pandemic	Cytology (Pap)	60-64	60	Not performed	Unsatisfactory	Unsatisfactory	Unsatisfactory	Unsatisfactory	
P00757	2018	2018-Q2	2018-05	Pre-pandemic	Cytology (Pap)	25-29	27	Not performed	Satisfactory	NILM	NILM	Negative (NILM)	
P00758	2018	2018-Q2	2018-05	Pre-pandemic	Cytology (Pap)	30-34	33	Not performed	Satisfactory	NILM	NILM	Negative (NILM)	
P00759	2018	2018-Q2	2018-05	Pre-pandemic	Cytology (Pap)	55-59	56	Not performed	Satisfactory	NILM	NILM	Negative (NILM)	
P00760	2018	2018-Q2	2018-05	Pre-pandemic	Cytology (Pap)	25-29	26	Not performed	Satisfactory	NILM	NILM	Negative (NILM)	
P00761	2018	2018-Q2	2018-05	Pre-pandemic	Cytology (Pap)	30-34	30	Not performed	Satisfactory	NILM	NILM	Negative (NILM)	
P00762	2018	2018-Q2	2018-05	Pre-pandemic	Cytology (Pap)	50-54	51	Not performed	Satisfactory	NILM	NILM	Negative (NILM)	
P00763	2018	2018-Q2	2018-05	Pre-pandemic	Cytology (Pap)	45-49	45	Not performed	Satisfactory	NILM	NILM	Negative (NILM)	
P00764	2018	2018-Q2	2018-05	Pre-pandemic	Cytology (Pap)	35-39	38	Not performed	Satisfactory	NILM	NILM	Negative (NILM)	
P00765	2018	2018-Q2	2018-05	Pre-pandemic	Cytology (Pap)	45-49	48	Not performed	Satisfactory	Positive	AGC	Glandular	
P00766	2018	2018-Q2	2018-05	Pre-pandemic	Cytology (Pap)	40-44	43	Not performed	Satisfactory	NILM	NILM	Negative (NILM)	
P00767	2018	2018-Q2	2018-05	Pre-pandemic	Cytology (Pap)	30-34	34	Not performed	Satisfactory	Positive	ASC-US	Low-grade / equivocal squamous	
P00768	2018	2018-Q2	2018-05	Pre-pandemic	Cytology (Pap)	35-39	39	Not performed	Satisfactory	NILM	NILM	Negative (NILM)	
P00769	2018	2018-Q2	2018-05	Pre-pandemic	Cytology (Pap)	35-39	38	Not performed	Satisfactory	NILM	NILM	Negative (NILM)	
P00770	2018	2018-Q2	2018-05	Pre-pandemic	Cytology (Pap)	45-49	47	Not performed	Satisfactory	NILM	NILM	Negative (NILM)	
P00771	2018	2018-Q2	2018-05	Pre-pandemic	Cytology (Pap)	45-49	49	Not performed	Satisfactory	NILM	NILM	Negative (NILM)	
P00772	2018	2018-Q2	2018-05	Pre-pandemic	Cytology (Pap)	35-39	38	Not performed	Satisfactory	NILM	NILM	Negative (NILM)	
P00773	2018	2018-Q2	2018-05	Pre-pandemic	Cytology (Pap)	60-64	62	Not performed	Satisfactory	NILM	NILM	Negative (NILM)	
P00774	2018	2018-Q2	2018-05	Pre-pandemic	Cytology (Pap)	25-29	25	Not performed	Satisfactory	NILM	NILM	Negative (NILM)	
P00775	2018	2018-Q2	2018-05	Pre-pandemic	Cytology (Pap)	40-44	41	Not performed	Satisfactory	NILM	NILM	Negative (NILM)	
P00776	2018	2018-Q2	2018-05	Pre-pandemic	Cytology (Pap)	30-34	32	Not performed	Satisfactory	NILM	NILM	Negative (NILM)	
P00777	2018	2018-Q2	2018-05	Pre-pandemic	Cytology (Pap)	40-44	41	Not performed	Satisfactory	NILM	NILM	Negative (NILM)	
P00778	2018	2018-Q2	2018-05	Pre-pandemic	Cytology (Pap)	35-39	35	Not performed	Unsatisfactory	Unsatisfactory	Unsatisfactory	Unsatisfactory	
P00779	2018	2018-Q2	2018-05	Pre-pandemic	Cytology (Pap)	25-29	27	Not performed	Satisfactory	Positive	AGC	Glandular	
P00780	2018	2018-Q2	2018-05	Pre-pandemic	Cytology (Pap)	50-54	50	Not performed	Satisfactory	NILM	NILM	Negative (NILM)	
P00781	2018	2018-Q2	2018-05	Pre-pandemic	Cytology (Pap)	35-39	35	Not performed	Satisfactory	NILM	NILM	Negative (NILM)	
P00782	2018	2018-Q2	2018-05	Pre-pandemic	Cytology (Pap)	45-49	47	Not performed	Satisfactory	NILM	NILM	Negative (NILM)	
P00783	2018	2018-Q2	2018-05	Pre-pandemic	Cytology (Pap)	35-39	38	Not performed	Satisfactory	NILM	NILM	Negative (NILM)	
P00784	2018	2018-Q2	2018-05	Pre-pandemic	Cytology (Pap)	45-49	47	Not performed	Satisfactory	NILM	NILM	Negative (NILM)	
P00785	2018	2018-Q2	2018-05	Pre-pandemic	Cytology (Pap)	40-44	44	Not performed	Satisfactory	NILM	NILM	Negative (NILM)	
P00786	2018	2018-Q2	2018-05	Pre-pandemic	Cytology (Pap)	25-29	26	Not performed	Satisfactory	Positive	HSIL	High-grade squamous	
P00787	2018	2018-Q2	2018-05	Pre-pandemic	Cytology (Pap)	35-39	39	Not performed	Satisfactory	NILM	NILM	Negative (NILM)	Candida
P00788	2018	2018-Q2	2018-05	Pre-pandemic	Cytology (Pap)	35-39	35	Not performed	Satisfactory	NILM	NILM	Negative (NILM)	
P00789	2018	2018-Q2	2018-05	Pre-pandemic	Cytology (Pap)	40-44	44	Not performed	Satisfactory	NILM	NILM	Negative (NILM)	
P00790	2018	2018-Q2	2018-05	Pre-pandemic	Cytology (Pap)	40-44	42	Not performed	Satisfactory	Positive	ASC-US	Low-grade / equivocal squamous	
P00791	2018	2018-Q2	2018-05	Pre-pandemic	Cytology (Pap)	45-49	47	Not performed	Satisfactory	NILM	NILM	Negative (NILM)	
P00792	2018	2018-Q2	2018-05	Pre-pandemic	Cytology (Pap)	50-54	53	Not performed	Satisfactory	Positive	HSIL	High-grade squamous	
P00793	2018	2018-Q2	2018-05	Pre-pandemic	Cytology (Pap)	30-34	31	Not performed	Satisfactory	NILM	NILM	Negative (NILM)	
P00794	2018	2018-Q2	2018-05	Pre-pandemic	Cytology (Pap)	35-39	36	Not performed	Satisfactory	NILM	NILM	Negative (NILM)	
P00795	2018	2018-Q2	2018-05	Pre-pandemic	Cytology (Pap)	50-54	51	Not performed	Satisfactory	NILM	NILM	Negative (NILM)	
P00796	2018	2018-Q2	2018-05	Pre-pandemic	Cytology (Pap)	30-34	32	Not performed	Satisfactory	NILM	NILM	Negative (NILM)	
P00797	2018	2018-Q2	2018-05	Pre-pandemic	Cytology (Pap)	60-64	60	Not performed	Satisfactory	Positive	ASC-US	Low-grade / equivocal squamous	
P00798	2018	2018-Q2	2018-05	Pre-pandemic	Cytology (Pap)	55-59	59	Not performed	Satisfactory	NILM	NILM	Negative (NILM)	

patient_id	year	quarter	month	period	primary_test	age_group	age_years	hpv_result	adequacy	result	bethesda	grade	infection
P00799	2018	2018-Q2	2018-05	Pre-pandemic	Cytology (Pap)	55-59	59	Not performed	Satisfactory	NILM	NILM	Negative (NILM)	
P00800	2018	2018-Q2	2018-05	Pre-pandemic	Cytology (Pap)	50-54	54	Not performed	Satisfactory	NILM	NILM	Negative (NILM)	
P00801	2018	2018-Q2	2018-05	Pre-pandemic	Cytology (Pap)	60-64	62	Not performed	Satisfactory	NILM	NILM	Negative (NILM)	
P00802	2018	2018-Q2	2018-05	Pre-pandemic	Cytology (Pap)	60-64	61	Not performed	Satisfactory	NILM	NILM	Negative (NILM)	
P00803	2018	2018-Q2	2018-05	Pre-pandemic	Cytology (Pap)	40-44	41	Not performed	Satisfactory	NILM	NILM	Negative (NILM)	
P00804	2018	2018-Q2	2018-05	Pre-pandemic	Cytology (Pap)	60-64	63	Not performed	Satisfactory	NILM	NILM	Negative (NILM)	
P00805	2018	2018-Q2	2018-05	Pre-pandemic	Cytology (Pap)	25-29	29	Not performed	Satisfactory	NILM	NILM	Negative (NILM)	
P00806	2018	2018-Q2	2018-05	Pre-pandemic	Cytology (Pap)	40-44	44	Not performed	Satisfactory	NILM	NILM	Negative (NILM)	
P00807	2018	2018-Q2	2018-05	Pre-pandemic	Cytology (Pap)	30-34	31	Not performed	Satisfactory	NILM	NILM	Negative (NILM)	
P00808	2018	2018-Q2	2018-05	Pre-pandemic	Cytology (Pap)	40-44	42	Not performed	Satisfactory	NILM	NILM	Negative (NILM)	
P00809	2018	2018-Q2	2018-05	Pre-pandemic	Cytology (Pap)	30-34	32	Not performed	Unsatisfactory	Unsatisfactory	Unsatisfactory	Unsatisfactory	
P00810	2018	2018-Q2	2018-05	Pre-pandemic	Cytology (Pap)	60-64	63	Not performed	Satisfactory	NILM	NILM	Negative (NILM)	Candida
P00811	2018	2018-Q2	2018-05	Pre-pandemic	Cytology (Pap)	25-29	28	Not performed	Satisfactory	Positive	LSIL	Low-grade / equivocal squamous	
P00812	2018	2018-Q2	2018-05	Pre-pandemic	Cytology (Pap)	50-54	50	Not performed	Satisfactory	Positive	LSIL	Low-grade / equivocal squamous	
P00813	2018	2018-Q2	2018-05	Pre-pandemic	Cytology (Pap)	50-54	52	Not performed	Satisfactory	NILM	NILM	Negative (NILM)	
P00814	2018	2018-Q2	2018-05	Pre-pandemic	Cytology (Pap)	35-39	35	Not performed	Satisfactory	NILM	NILM	Negative (NILM)	
P00815	2018	2018-Q2	2018-05	Pre-pandemic	Cytology (Pap)	30-34	33	Not performed	Satisfactory	NILM	NILM	Negative (NILM)	
P00816	2018	2018-Q2	2018-05	Pre-pandemic	Cytology (Pap)	30-34	31	Not performed	Satisfactory	Positive	HSIL	High-grade squamous	
P00817	2018	2018-Q2	2018-05	Pre-pandemic	Cytology (Pap)	30-34	33	Not performed	Satisfactory	NILM	NILM	Negative (NILM)	
P00818	2018	2018-Q2	2018-05	Pre-pandemic	Cytology (Pap)	45-49	45	Not performed	Satisfactory	NILM	NILM	Negative (NILM)	
P00819	2018	2018-Q2	2018-05	Pre-pandemic	Cytology (Pap)	30-34	33	Not performed	Satisfactory	NILM	NILM	Negative (NILM)	
P00820	2018	2018-Q2	2018-05	Pre-pandemic	Cytology (Pap)	45-49	47	Not performed	Satisfactory	NILM	NILM	Negative (NILM)	
P00821	2018	2018-Q2	2018-05	Pre-pandemic	Cytology (Pap)	50-54	51	Not performed	Satisfactory	NILM	NILM	Negative (NILM)	
P00822	2018	2018-Q2	2018-05	Pre-pandemic	Cytology (Pap)	30-34	34	Not performed	Satisfactory	NILM	NILM	Negative (NILM)	
P00823	2018	2018-Q2	2018-05	Pre-pandemic	Cytology (Pap)	45-49	45	Not performed	Satisfactory	NILM	NILM	Negative (NILM)	
P00824	2018	2018-Q2	2018-05	Pre-pandemic	Cytology (Pap)	45-49	46	Not performed	Satisfactory	NILM	NILM	Negative (NILM)	
P00825	2018	2018-Q2	2018-05	Pre-pandemic	Cytology (Pap)	35-39	38	Not performed	Satisfactory	NILM	NILM	Negative (NILM)	
P00826	2018	2018-Q2	2018-05	Pre-pandemic	Cytology (Pap)	60-64	60	Not performed	Satisfactory	Positive	ASC-US	Low-grade / equivocal squamous	
P00827	2018	2018-Q2	2018-05	Pre-pandemic	Cytology (Pap)	50-54	52	Not performed	Satisfactory	Positive	ASC-US	Low-grade / equivocal squamous	
P00828	2018	2018-Q2	2018-05	Pre-pandemic	Cytology (Pap)	50-54	54	Not performed	Satisfactory	Positive	ASC-US	Low-grade / equivocal squamous	
P00829	2018	2018-Q2	2018-05	Pre-pandemic	Cytology (Pap)	45-49	46	Not performed	Satisfactory	NILM	NILM	Negative (NILM)	
P00830	2018	2018-Q2	2018-05	Pre-pandemic	Cytology (Pap)	40-44	42	Not performed	Satisfactory	NILM	NILM	Negative (NILM)	
P00831	2018	2018-Q2	2018-05	Pre-pandemic	Cytology (Pap)	30-34	34	Not performed	Satisfactory	NILM	NILM	Negative (NILM)	
P00832	2018	2018-Q2	2018-05	Pre-pandemic	Cytology (Pap)	30-34	30	Not performed	Satisfactory	NILM	NILM	Negative (NILM)	
P00833	2018	2018-Q2	2018-05	Pre-pandemic	Cytology (Pap)	30-34	30	Not performed	Satisfactory	NILM	NILM	Negative (NILM)	
P00834	2018	2018-Q2	2018-05	Pre-pandemic	Cytology (Pap)	30-34	30	Not performed	Satisfactory	NILM	NILM	Negative (NILM)	
P00835	2018	2018-Q2	2018-05	Pre-pandemic	Cytology (Pap)	30-34	31	Not performed	Satisfactory	NILM	NILM	Negative (NILM)	
P00836	2018	2018-Q2	2018-05	Pre-pandemic	Cytology (Pap)	30-34	30	Not performed	Satisfactory	NILM	NILM	Negative (NILM)	
P00837	2018	2018-Q2	2018-05	Pre-pandemic	Cytology (Pap)	25-29	28	Not performed	Satisfactory	NILM	NILM	Negative (NILM)	
P00838	2018	2018-Q2	2018-05	Pre-pandemic	Cytology (Pap)	50-54	51	Not performed	Satisfactory	NILM	NILM	Negative (NILM)	
P00839	2018	2018-Q2	2018-05	Pre-pandemic	Cytology (Pap)	35-39	39	Not performed	Satisfactory	NILM	NILM	Negative (NILM)	
P00840	2018	2018-Q2	2018-05	Pre-pandemic	Cytology (Pap)	45-49	48	Not performed	Satisfactory	NILM	NILM	Negative (NILM)	
P00841	2018	2018-Q2	2018-05	Pre-pandemic	Cytology (Pap)	50-54	53	Not performed	Satisfactory	NILM	NILM	Negative (NILM)	Candida
P00842	2018	2018-Q2	2018-05	Pre-pandemic	Cytology (Pap)	60-64	60	Not performed	Satisfactory	NILM	NILM	Negative (NILM)	Trichomonas
P00843	2018	2018-Q2	2018-05	Pre-pandemic	Cytology (Pap)	35-39	36	Not performed	Satisfactory	NILM	NILM	Negative (NILM)	
P00844	2018	2018-Q2	2018-05	Pre-pandemic	Cytology (Pap)	60-64	60	Not performed	Satisfactory	Positive	HSIL	High-grade squamous	
P00845	2018	2018-Q2	2018-05	Pre-pandemic	Cytology (Pap)	40-44	44	Not performed	Satisfactory	NILM	NILM	Negative (NILM)	
P00846	2018	2018-Q2	2018-05	Pre-pandemic	Cytology (Pap)	30-34	31	Not performed	Satisfactory	Positive	ASC-US	Low-grade / equivocal squamous	
P00847	2018	2018-Q2	2018-05	Pre-pandemic	Cytology (Pap)	40-44	41	Not performed	Satisfactory	NILM	NILM	Negative (NILM)	
P00848	2018	2018-Q2	2018-05	Pre-pandemic	Cytology (Pap)	40-44	43	Not performed	Satisfactory	NILM	NILM	Negative (NILM)	
P00849	2018	2018-Q2	2018-05	Pre-pandemic	Cytology (Pap)	30-34	31	Not performed	Satisfactory	NILM	NILM	Negative (NILM)	
P00850	2018	2018-Q2	2018-05	Pre-pandemic	Cytology (Pap)	40-44	41	Not performed	Satisfactory	NILM	NILM	Negative (NILM)	
P00851	2018	2018-Q2	2018-05	Pre-pandemic	Cytology (Pap)	30-34	34	Not performed	Satisfactory	NILM	NILM	Negative (NILM)	
P00852	2018	2018-Q2	2018-05	Pre-pandemic	Cytology (Pap)	40-44	43	Not performed	Satisfactory	NILM	NILM	Negative (NILM)	
P00853	2018	2018-Q2	2018-05	Pre-pandemic	Cytology (Pap)	Not recorded		Not performed	Satisfactory	NILM	NILM	Negative (NILM)	
P00854	2018	2018-Q2	2018-05	Pre-pandemic	Cytology (Pap)	45-49	47	Not performed	Satisfactory	Positive	HSIL	High-grade squamous	
P00855	2018	2018-Q2	2018-05	Pre-pandemic	Cytology (Pap)	30-34	34	Not performed	Satisfactory	NILM	NILM	Negative (NILM)	
P00856	2018	2018-Q2	2018-05	Pre-pandemic	Cytology (Pap)	35-39	35	Not performed	Satisfactory	Positive	ASC-US	Low-grade / equivocal squamous	
P00857	2018	2018-Q2	2018-05	Pre-pandemic	Cytology (Pap)	30-34	30	Not performed	Unsatisfactory	Unsatisfactory	Unsatisfactory	Unsatisfactory	
P00858	2018	2018-Q2	2018-05	Pre-pandemic	Cytology (Pap)	25-29	27	Not performed	Satisfactory	NILM	NILM	Negative (NILM)	
P00859	2018	2018-Q2	2018-05	Pre-pandemic	Cytology (Pap)	40-44	40	Not performed	Satisfactory	NILM	NILM	Negative (NILM)	
P00860	2018	2018-Q2	2018-05	Pre-pandemic	Cytology (Pap)	60-64	60	Not performed	Satisfactory	NILM	NILM	Negative (NILM)	
P00861	2018	2018-Q2	2018-05	Pre-pandemic	Cytology (Pap)	45-49	46	Not performed	Satisfactory	NILM	NILM	Negative (NILM)	Gardnerella
P00862	2018	2018-Q2	2018-05	Pre-pandemic	Cytology (Pap)	40-44	44	Not performed	Satisfactory	NILM	NILM	Negative (NILM)	
P00863	2018	2018-Q2	2018-05	Pre-pandemic	Cytology (Pap)	25-29	28	Not performed	Satisfactory	NILM	NILM	Negative (NILM)	
P00864	2018	2018-Q2	2018-05	Pre-pandemic	Cytology (Pap)	Not recorded		Not performed	Satisfactory	NILM	NILM	Negative (NILM)	
P00865	2018	2018-Q2	2018-05	Pre-pandemic	Cytology (Pap)	40-44	42	Not performed	Satisfactory	Positive	ASC-US	Low-grade / equivocal squamous	
P00866	2018	2018-Q2	2018-05	Pre-pandemic	Cytology (Pap)	40-44	43	Not performed	Satisfactory	NILM	NILM	Negative (NILM)	
P00867	2018	2018-Q2	2018-05	Pre-pandemic	Cytology (Pap)	45-49	46	Not performed	Satisfactory	NILM	NILM	Negative (NILM)	
P00868	2018	2018-Q2	2018-05	Pre-pandemic	Cytology (Pap)	40-44	42	Not performed	Satisfactory	NILM	NILM	Negative (NILM)	
P00869	2018	2018-Q2	2018-05	Pre-pandemic	Cytology (Pap)	40-44	44	Not performed	Satisfactory	NILM	NILM	Negative (NILM)	
P00870	2018	2018-Q2	2018-05	Pre-pandemic	Cytology (Pap)	30-34	32	Not performed	Satisfactory	NILM	NILM	Negative (NILM)	Candida
P00871	2018	2018-Q2	2018-05	Pre-pandemic	Cytology (Pap)	60-64	61	Not performed	Satisfactory	NILM	NILM	Negative (NILM)	
P00872	2018	2018-Q2	2018-05	Pre-pandemic	Cytology (Pap)	55-59	55	Not performed	Satisfactory	NILM	NILM	Negative (NILM)	
P00873	2018	2018-Q2	2018-05	Pre-pandemic	Cytology (Pap)	40-44	42	Not performed	Satisfactory	NILM	NILM	Negative (NILM)	
P00874	2018	2018-Q2	2018-05	Pre-pandemic	Cytology (Pap)	25-29	29	Not performed	Satisfactory	NILM	NILM	Negative (NILM)	
P00875	2018	2018-Q2	2018-05	Pre-pandemic	Cytology (Pap)	55-59	57	Not performed	Satisfactory	NILM	NILM	Negative (NILM)	
P00876	2018	2018-Q2	2018-05	Pre-pandemic	Cytology (Pap)	45-49	49	Not performed	Satisfactory	NILM	NILM	Negative (NILM)	
P00877	2018	2018-Q2	2018-05	Pre-pandemic	Cytology (Pap)	55-59	56	Not performed	Satisfactory	NILM	NILM	Negative (NILM)	
P00878	2018	2018-Q2	2018-05	Pre-pandemic	Cytology (Pap)	40-44	43	Not performed	Satisfactory	NILM	NILM	Negative (NILM)	

patient_id	year	quarter	month	period	primary_test	age_group	age_years	hpv_result	adequacy	result	bethesda	grade	infection
P00879	2018	2018-Q2	2018-05	Pre-pandemic	Cytology (Pap)	55-59	57	Not performed	Satisfactory	NILM	NILM	Negative (NILM)	
P00880	2018	2018-Q2	2018-05	Pre-pandemic	Cytology (Pap)	35-39	36	Not performed	Unsatisfactory	Unsatisfactory	Unsatisfactory	Unsatisfactory	
P00881	2018	2018-Q2	2018-05	Pre-pandemic	Cytology (Pap)	40-44	44	Not performed	Satisfactory	NILM	NILM	Negative (NILM)	
P00882	2018	2018-Q2	2018-05	Pre-pandemic	Cytology (Pap)	50-54	52	Not performed	Satisfactory	NILM	NILM	Negative (NILM)	
P00883	2018	2018-Q2	2018-05	Pre-pandemic	Cytology (Pap)	55-59	58	Not performed	Satisfactory	NILM	NILM	Negative (NILM)	
P00884	2018	2018-Q2	2018-05	Pre-pandemic	Cytology (Pap)	Not recorded		Not performed	Satisfactory	NILM	NILM	Negative (NILM)	
P00885	2018	2018-Q2	2018-05	Pre-pandemic	Cytology (Pap)	40-44	40	Not performed	Satisfactory	Positive	LSIL	Low-grade / equivocal squamous	
P00886	2018	2018-Q2	2018-05	Pre-pandemic	Cytology (Pap)	60-64	63	Not performed	Satisfactory	Positive	LSIL	Low-grade / equivocal squamous	
P00887	2018	2018-Q2	2018-05	Pre-pandemic	Cytology (Pap)	35-39	38	Not performed	Satisfactory	NILM	NILM	Negative (NILM)	
P00888	2018	2018-Q2	2018-05	Pre-pandemic	Cytology (Pap)	25-29	27	Not performed	Satisfactory	NILM	NILM	Negative (NILM)	Candida
P00889	2018	2018-Q2	2018-05	Pre-pandemic	Cytology (Pap)	35-39	39	Not performed	Satisfactory	NILM	NILM	Negative (NILM)	
P00890	2018	2018-Q2	2018-05	Pre-pandemic	Cytology (Pap)	60-64	62	Not performed	Satisfactory	Positive	HSIL	High-grade squamous	
P00891	2018	2018-Q2	2018-05	Pre-pandemic	Cytology (Pap)	50-54	53	Not performed	Satisfactory	NILM	NILM	Negative (NILM)	
P00892	2018	2018-Q2	2018-05	Pre-pandemic	Cytology (Pap)	Not recorded		Not performed	Unsatisfactory	Unsatisfactory	Unsatisfactory	Unsatisfactory	
P00893	2018	2018-Q2	2018-05	Pre-pandemic	Cytology (Pap)	40-44	40	Not performed	Satisfactory	NILM	NILM	Negative (NILM)	
P00894	2018	2018-Q2	2018-05	Pre-pandemic	Cytology (Pap)	35-39	39	Not performed	Satisfactory	NILM	NILM	Negative (NILM)	
P00895	2018	2018-Q2	2018-05	Pre-pandemic	Cytology (Pap)	40-44	41	Not performed	Satisfactory	NILM	NILM	Negative (NILM)	
P00896	2018	2018-Q2	2018-05	Pre-pandemic	Cytology (Pap)	35-39	35	Not performed	Satisfactory	NILM	NILM	Negative (NILM)	
P00897	2018	2018-Q2	2018-05	Pre-pandemic	Cytology (Pap)	50-54	52	Not performed	Satisfactory	NILM	NILM	Negative (NILM)	
P00898	2018	2018-Q2	2018-05	Pre-pandemic	Cytology (Pap)	40-44	41	Not performed	Satisfactory	NILM	NILM	Negative (NILM)	
P00899	2018	2018-Q2	2018-05	Pre-pandemic	Cytology (Pap)	50-54	51	Not performed	Satisfactory	NILM	NILM	Negative (NILM)	
P00900	2018	2018-Q2	2018-05	Pre-pandemic	Cytology (Pap)	60-64	60	Not performed	Satisfactory	Positive	ASC-US	Low-grade / equivocal squamous	
P00901	2018	2018-Q2	2018-05	Pre-pandemic	Cytology (Pap)	55-59	56	Not performed	Satisfactory	NILM	NILM	Negative (NILM)	
P00902	2018	2018-Q2	2018-05	Pre-pandemic	Cytology (Pap)	Not recorded		Not performed	Satisfactory	NILM	NILM	Negative (NILM)	
P00903	2018	2018-Q2	2018-05	Pre-pandemic	Cytology (Pap)	60-64	64	Not performed	Satisfactory	NILM	NILM	Negative (NILM)	
P00904	2018	2018-Q2	2018-05	Pre-pandemic	Cytology (Pap)	35-39	36	Not performed	Satisfactory	NILM	NILM	Negative (NILM)	
P00905	2018	2018-Q2	2018-05	Pre-pandemic	Cytology (Pap)	55-59	57	Not performed	Satisfactory	NILM	NILM	Negative (NILM)	
P00906	2018	2018-Q2	2018-05	Pre-pandemic	Cytology (Pap)	55-59	57	Not performed	Satisfactory	NILM	NILM	Negative (NILM)	
P00907	2018	2018-Q2	2018-05	Pre-pandemic	Cytology (Pap)	40-44	42	Not performed	Satisfactory	NILM	NILM	Negative (NILM)	
P00908	2018	2018-Q2	2018-05	Pre-pandemic	Cytology (Pap)	45-49	48	Not performed	Satisfactory	NILM	NILM	Negative (NILM)	
P00909	2018	2018-Q2	2018-05	Pre-pandemic	Cytology (Pap)	45-49	47	Not performed	Satisfactory	NILM	NILM	Negative (NILM)	
P00910	2018	2018-Q2	2018-05	Pre-pandemic	Cytology (Pap)	45-49	48	Not performed	Satisfactory	NILM	NILM	Negative (NILM)	
P00911	2018	2018-Q2	2018-05	Pre-pandemic	Cytology (Pap)	50-54	51	Not performed	Satisfactory	NILM	NILM	Negative (NILM)	
P00912	2018	2018-Q2	2018-05	Pre-pandemic	Cytology (Pap)	55-59	57	Not performed	Satisfactory	Positive	AGC	Glandular	
P00913	2018	2018-Q2	2018-05	Pre-pandemic	Cytology (Pap)	50-54	52	Not performed	Satisfactory	NILM	NILM	Negative (NILM)	
P00914	2018	2018-Q2	2018-05	Pre-pandemic	Cytology (Pap)	45-49	46	Not performed	Satisfactory	NILM	NILM	Negative (NILM)	
P00915	2018	2018-Q2	2018-05	Pre-pandemic	Cytology (Pap)	25-29	25	Not performed	Satisfactory	Positive	HSIL	High-grade squamous	
P00916	2018	2018-Q2	2018-05	Pre-pandemic	Cytology (Pap)	25-29	26	Not performed	Satisfactory	NILM	NILM	Negative (NILM)	
P00917	2018	2018-Q2	2018-05	Pre-pandemic	Cytology (Pap)	45-49	49	Not performed	Satisfactory	Positive	ASC-US	Low-grade / equivocal squamous	
P00918	2018	2018-Q2	2018-06	Pre-pandemic	Cytology (Pap)	25-29	28	Not performed	Satisfactory	NILM	NILM	Negative (NILM)	
P00919	2018	2018-Q2	2018-06	Pre-pandemic	Cytology (Pap)	25-29	28	Not performed	Satisfactory	NILM	NILM	Negative (NILM)	
P00920	2018	2018-Q2	2018-06	Pre-pandemic	Cytology (Pap)	35-39	37	Not performed	Satisfactory	NILM	NILM	Negative (NILM)	
P00921	2018	2018-Q2	2018-06	Pre-pandemic	Cytology (Pap)	Not recorded		Not performed	Satisfactory	NILM	NILM	Negative (NILM)	
P00922	2018	2018-Q2	2018-06	Pre-pandemic	Cytology (Pap)	40-44	43	Not performed	Satisfactory	NILM	NILM	Negative (NILM)	
P00923	2018	2018-Q2	2018-06	Pre-pandemic	Cytology (Pap)	30-34	32	Not performed	Satisfactory	NILM	NILM	Negative (NILM)	
P00924	2018	2018-Q2	2018-06	Pre-pandemic	Cytology (Pap)	30-34	31	Not performed	Satisfactory	NILM	NILM	Negative (NILM)	
P00925	2018	2018-Q2	2018-06	Pre-pandemic	Cytology (Pap)	55-59	57	Not performed	Satisfactory	NILM	NILM	Negative (NILM)	
P00926	2018	2018-Q2	2018-06	Pre-pandemic	Cytology (Pap)	35-39	39	Not performed	Satisfactory	NILM	NILM	Negative (NILM)	
P00927	2018	2018-Q2	2018-06	Pre-pandemic	Cytology (Pap)	55-59	56	Not performed	Satisfactory	NILM	NILM	Negative (NILM)	
P00928	2018	2018-Q2	2018-06	Pre-pandemic	Cytology (Pap)	40-44	44	Not performed	Satisfactory	NILM	NILM	Negative (NILM)	
P00929	2018	2018-Q2	2018-06	Pre-pandemic	Cytology (Pap)	40-44	40	Not performed	Satisfactory	NILM	NILM	Negative (NILM)	
P00930	2018	2018-Q2	2018-06	Pre-pandemic	Cytology (Pap)	40-44	44	Not performed	Satisfactory	NILM	NILM	Negative (NILM)	
P00931	2018	2018-Q2	2018-06	Pre-pandemic	Cytology (Pap)	45-49	48	Not performed	Satisfactory	NILM	NILM	Negative (NILM)	Trichomonas
P00932	2018	2018-Q2	2018-06	Pre-pandemic	Cytology (Pap)	40-44	44	Not performed	Satisfactory	NILM	NILM	Negative (NILM)	
P00933	2018	2018-Q2	2018-06	Pre-pandemic	Cytology (Pap)	35-39	39	Not performed	Satisfactory	NILM	NILM	Negative (NILM)	
P00934	2018	2018-Q2	2018-06	Pre-pandemic	Cytology (Pap)	40-44	41	Not performed	Satisfactory	NILM	NILM	Negative (NILM)	
P00935	2018	2018-Q2	2018-06	Pre-pandemic	Cytology (Pap)	55-59	58	Not performed	Satisfactory	NILM	NILM	Negative (NILM)	
P00936	2018	2018-Q2	2018-06	Pre-pandemic	Cytology (Pap)	25-29	26	Not performed	Unsatisfactory	Unsatisfactory	Unsatisfactory	Unsatisfactory	
P00937	2018	2018-Q2	2018-06	Pre-pandemic	Cytology (Pap)	55-59	59	Not performed	Satisfactory	Positive	LSIL	Low-grade / equivocal squamous	Other
P00938	2018	2018-Q2	2018-06	Pre-pandemic	Cytology (Pap)	40-44	40	Not performed	Satisfactory	NILM	NILM	Negative (NILM)	Candida
P00939	2018	2018-Q2	2018-06	Pre-pandemic	Cytology (Pap)	50-54	53	Not performed	Satisfactory	NILM	NILM	Negative (NILM)	
P00940	2018	2018-Q2	2018-06	Pre-pandemic	Cytology (Pap)	30-34	32	Not performed	Satisfactory	NILM	NILM	Negative (NILM)	
P00941	2018	2018-Q2	2018-06	Pre-pandemic	Cytology (Pap)	35-39	38	Not performed	Satisfactory	NILM	NILM	Negative (NILM)	
P00942	2018	2018-Q2	2018-06	Pre-pandemic	Cytology (Pap)	Not recorded		Not performed	Satisfactory	Positive	ASC-US	Low-grade / equivocal squamous	
P00943	2018	2018-Q2	2018-06	Pre-pandemic	Cytology (Pap)	55-59	59	Not performed	Satisfactory	NILM	NILM	Negative (NILM)	
P00944	2018	2018-Q2	2018-06	Pre-pandemic	Cytology (Pap)	45-49	47	Not performed	Satisfactory	NILM	NILM	Negative (NILM)	
P00945	2018	2018-Q2	2018-06	Pre-pandemic	Cytology (Pap)	30-34	32	Not performed	Satisfactory	Positive	ASC-US	Low-grade / equivocal squamous	
P00946	2018	2018-Q2	2018-06	Pre-pandemic	Cytology (Pap)	35-39	36	Not performed	Satisfactory	Positive	HSIL	High-grade squamous	
P00947	2018	2018-Q2	2018-06	Pre-pandemic	Cytology (Pap)	Not recorded		Not performed	Satisfactory	NILM	NILM	Negative (NILM)	
P00948	2018	2018-Q2	2018-06	Pre-pandemic	Cytology (Pap)	45-49	45	Not performed	Satisfactory	NILM	NILM	Negative (NILM)	
P00949	2018	2018-Q2	2018-06	Pre-pandemic	Cytology (Pap)	35-39	35	Not performed	Satisfactory	NILM	NILM	Negative (NILM)	
P00950	2018	2018-Q2	2018-06	Pre-pandemic	Cytology (Pap)	40-44	41	Not performed	Satisfactory	Positive	ASC-US	Low-grade / equivocal squamous	
P00951	2018	2018-Q2	2018-06	Pre-pandemic	Cytology (Pap)	50-54	51	Not performed	Satisfactory	Positive	ASC-US	Low-grade / equivocal squamous	
P00952	2018	2018-Q2	2018-06	Pre-pandemic	Cytology (Pap)	25-29	28	Not performed	Satisfactory	NILM	NILM	Negative (NILM)	
P00953	2018	2018-Q2	2018-06	Pre-pandemic	Cytology (Pap)	45-49	46	Not performed	Satisfactory	NILM	NILM	Negative (NILM)	
P00954	2018	2018-Q2	2018-06	Pre-pandemic	Cytology (Pap)	35-39	36	Not performed	Satisfactory	NILM	NILM	Negative (NILM)	
P00955	2018	2018-Q2	2018-06	Pre-pandemic	Cytology (Pap)	45-49	48	Not performed	Satisfactory	NILM	NILM	Negative (NILM)	
P00956	2018	2018-Q2	2018-06	Pre-pandemic	Cytology (Pap)	45-49	46	Not performed	Satisfactory	Positive	ASC-US	Low-grade / equivocal squamous	
P00957	2018	2018-Q2	2018-06	Pre-pandemic	Cytology (Pap)	35-39	37	Not performed	Satisfactory	Positive	ASC-H	High-grade squamous	
P00958	2018	2018-Q2	2018-06	Pre-pandemic	Cytology (Pap)	40-44	40	Not performed	Satisfactory	NILM	NILM	Negative (NILM)	

patient_id	year	quarter	month	period	primary_test	age_group	age_years	hpv_result	adequacy	result	bethesda	grade	infection
P00959	2018	2018-Q2	2018-06	Pre-pandemic	Cytology (Pap)	40-44	41	Not performed	Satisfactory	NILM	NILM	Negative (NILM)	
P00960	2018	2018-Q2	2018-06	Pre-pandemic	Cytology (Pap)	35-39	35	Not performed	Satisfactory	NILM	NILM	Negative (NILM)	Candida
P00961	2018	2018-Q2	2018-06	Pre-pandemic	Cytology (Pap)	50-54	50	Not performed	Satisfactory	NILM	NILM	Negative (NILM)	
P00962	2018	2018-Q2	2018-06	Pre-pandemic	Cytology (Pap)	50-54	50	Not performed	Satisfactory	NILM	NILM	Negative (NILM)	
P00963	2018	2018-Q2	2018-06	Pre-pandemic	Cytology (Pap)	40-44	43	Not performed	Satisfactory	NILM	NILM	Negative (NILM)	
P00964	2018	2018-Q2	2018-06	Pre-pandemic	Cytology (Pap)	60-64	63	Not performed	Satisfactory	NILM	NILM	Negative (NILM)	
P00965	2018	2018-Q2	2018-06	Pre-pandemic	Cytology (Pap)	40-44	41	Not performed	Satisfactory	NILM	NILM	Negative (NILM)	
P00966	2018	2018-Q2	2018-06	Pre-pandemic	Cytology (Pap)	40-44	42	Not performed	Satisfactory	NILM	NILM	Negative (NILM)	
P00967	2018	2018-Q2	2018-06	Pre-pandemic	Cytology (Pap)	45-49	48	Not performed	Satisfactory	NILM	NILM	Negative (NILM)	
P00968	2018	2018-Q2	2018-06	Pre-pandemic	Cytology (Pap)	35-39	37	Not performed	Satisfactory	NILM	NILM	Negative (NILM)	
P00969	2018	2018-Q2	2018-06	Pre-pandemic	Cytology (Pap)	35-39	39	Not performed	Satisfactory	NILM	NILM	Negative (NILM)	
P00970	2018	2018-Q2	2018-06	Pre-pandemic	Cytology (Pap)	45-49	45	Not performed	Unsatisfactory	Unsatisfactory	Unsatisfactory	Unsatisfactory	
P00971	2018	2018-Q2	2018-06	Pre-pandemic	Cytology (Pap)	30-34	31	Not performed	Satisfactory	NILM	NILM	Negative (NILM)	
P00972	2018	2018-Q2	2018-06	Pre-pandemic	Cytology (Pap)	40-44	44	Not performed	Satisfactory	NILM	NILM	Negative (NILM)	
P00973	2018	2018-Q2	2018-06	Pre-pandemic	Cytology (Pap)	45-49	48	Not performed	Satisfactory	NILM	NILM	Negative (NILM)	
P00974	2018	2018-Q2	2018-06	Pre-pandemic	Cytology (Pap)	25-29	29	Not performed	Satisfactory	NILM	NILM	Negative (NILM)	
P00975	2018	2018-Q2	2018-06	Pre-pandemic	Cytology (Pap)	30-34	33	Not performed	Unsatisfactory	Unsatisfactory	Unsatisfactory	Unsatisfactory	
P00976	2018	2018-Q2	2018-06	Pre-pandemic	Cytology (Pap)	55-59	57	Not performed	Satisfactory	NILM	NILM	Negative (NILM)	
P00977	2018	2018-Q2	2018-06	Pre-pandemic	Cytology (Pap)	45-49	47	Not performed	Satisfactory	NILM	NILM	Negative (NILM)	Candida
P00978	2018	2018-Q2	2018-06	Pre-pandemic	Cytology (Pap)	50-54	51	Not performed	Satisfactory	NILM	NILM	Negative (NILM)	
P00979	2018	2018-Q2	2018-06	Pre-pandemic	Cytology (Pap)	30-34	34	Not performed	Satisfactory	NILM	NILM	Negative (NILM)	
P00980	2018	2018-Q2	2018-06	Pre-pandemic	Cytology (Pap)	40-44	41	Not performed	Satisfactory	NILM	NILM	Negative (NILM)	
P00981	2018	2018-Q2	2018-06	Pre-pandemic	Cytology (Pap)	45-49	48	Not performed	Satisfactory	NILM	NILM	Negative (NILM)	
P00982	2018	2018-Q2	2018-06	Pre-pandemic	Cytology (Pap)	40-44	44	Not performed	Satisfactory	Positive	ASC-H	High-grade squamous	
P00983	2018	2018-Q2	2018-06	Pre-pandemic	Cytology (Pap)	60-64	62	Not performed	Satisfactory	NILM	NILM	Negative (NILM)	
P00984	2018	2018-Q2	2018-06	Pre-pandemic	Cytology (Pap)	40-44	41	Not performed	Satisfactory	NILM	NILM	Negative (NILM)	
P00985	2018	2018-Q2	2018-06	Pre-pandemic	Cytology (Pap)	45-49	48	Not performed	Satisfactory	NILM	NILM	Negative (NILM)	
P00986	2018	2018-Q2	2018-06	Pre-pandemic	Cytology (Pap)	35-39	38	Not performed	Satisfactory	NILM	NILM	Negative (NILM)	
P00987	2018	2018-Q2	2018-06	Pre-pandemic	Cytology (Pap)	50-54	53	Not performed	Satisfactory	Positive	ASC-US	Low-grade / equivocal squamous	
P00988	2018	2018-Q2	2018-06	Pre-pandemic	Cytology (Pap)	55-59	56	Not performed	Satisfactory	NILM	NILM	Negative (NILM)	
P00989	2018	2018-Q2	2018-06	Pre-pandemic	Cytology (Pap)	50-54	53	Not performed	Satisfactory	NILM	NILM	Negative (NILM)	
P00990	2018	2018-Q2	2018-06	Pre-pandemic	Cytology (Pap)	35-39	39	Not performed	Satisfactory	NILM	NILM	Negative (NILM)	
P00991	2018	2018-Q2	2018-06	Pre-pandemic	Cytology (Pap)	45-49	48	Not performed	Satisfactory	Positive	HSIL	High-grade squamous	
P00992	2018	2018-Q2	2018-06	Pre-pandemic	Cytology (Pap)	60-64	64	Not performed	Satisfactory	NILM	NILM	Negative (NILM)	
P00993	2018	2018-Q2	2018-06	Pre-pandemic	Cytology (Pap)	50-54	54	Not performed	Satisfactory	NILM	NILM	Negative (NILM)	
P00994	2018	2018-Q2	2018-06	Pre-pandemic	Cytology (Pap)	55-59	57	Not performed	Satisfactory	NILM	NILM	Negative (NILM)	
P00995	2018	2018-Q2	2018-06	Pre-pandemic	Cytology (Pap)	35-39	39	Not performed	Satisfactory	NILM	NILM	Negative (NILM)	
P00996	2018	2018-Q2	2018-06	Pre-pandemic	Cytology (Pap)	25-29	28	Not performed	Satisfactory	NILM	NILM	Negative (NILM)	
P00997	2018	2018-Q2	2018-06	Pre-pandemic	Cytology (Pap)	55-59	59	Not performed	Satisfactory	NILM	NILM	Negative (NILM)	
P00998	2018	2018-Q2	2018-06	Pre-pandemic	Cytology (Pap)	40-44	43	Not performed	Satisfactory	NILM	NILM	Negative (NILM)	
P00999	2018	2018-Q2	2018-06	Pre-pandemic	Cytology (Pap)	30-34	30	Not performed	Satisfactory	NILM	NILM	Negative (NILM)	
P01000	2018	2018-Q2	2018-06	Pre-pandemic	Cytology (Pap)	45-49	48	Not performed	Satisfactory	NILM	NILM	Negative (NILM)	
P01001	2018	2018-Q2	2018-06	Pre-pandemic	Cytology (Pap)	35-39	35	Not performed	Satisfactory	NILM	NILM	Negative (NILM)	
P01002	2018	2018-Q2	2018-06	Pre-pandemic	Cytology (Pap)	40-44	43	Not performed	Satisfactory	NILM	NILM	Negative (NILM)	
P01003	2018	2018-Q2	2018-06	Pre-pandemic	Cytology (Pap)	40-44	41	Not performed	Satisfactory	NILM	NILM	Negative (NILM)	
P01004	2018	2018-Q2	2018-06	Pre-pandemic	Cytology (Pap)	30-34	30	Not performed	Satisfactory	NILM	NILM	Negative (NILM)	
P01005	2018	2018-Q2	2018-06	Pre-pandemic	Cytology (Pap)	45-49	47	Not performed	Satisfactory	NILM	NILM	Negative (NILM)	
P01006	2018	2018-Q2	2018-06	Pre-pandemic	Cytology (Pap)	55-59	58	Not performed	Satisfactory	NILM	NILM	Negative (NILM)	
P01007	2018	2018-Q2	2018-06	Pre-pandemic	Cytology (Pap)	40-44	40	Not performed	Satisfactory	NILM	NILM	Negative (NILM)	
P01008	2018	2018-Q2	2018-06	Pre-pandemic	Cytology (Pap)	40-44	43	Not performed	Satisfactory	NILM	NILM	Negative (NILM)	
P01009	2018	2018-Q2	2018-06	Pre-pandemic	Cytology (Pap)	25-29	28	Not performed	Satisfactory	NILM	NILM	Negative (NILM)	
P01010	2018	2018-Q2	2018-06	Pre-pandemic	Cytology (Pap)	60-64	62	Not performed	Unsatisfactory	Unsatisfactory	Unsatisfactory	Unsatisfactory	
P01011	2018	2018-Q2	2018-06	Pre-pandemic	Cytology (Pap)	35-39	35	Not performed	Satisfactory	Positive	AGC	Glandular	
P01012	2018	2018-Q2	2018-06	Pre-pandemic	Cytology (Pap)	60-64	61	Not performed	Satisfactory	Positive	LSIL	Low-grade / equivocal squamous	
P01013	2018	2018-Q2	2018-06	Pre-pandemic	Cytology (Pap)	30-34	31	Not performed	Satisfactory	NILM	NILM	Negative (NILM)	
P01014	2018	2018-Q2	2018-06	Pre-pandemic	Cytology (Pap)	25-29	25	Not performed	Satisfactory	NILM	NILM	Negative (NILM)	
P01015	2018	2018-Q2	2018-06	Pre-pandemic	Cytology (Pap)	50-54	54	Not performed	Satisfactory	Positive	AGC	Glandular	
P01016	2018	2018-Q2	2018-06	Pre-pandemic	Cytology (Pap)	40-44	43	Not performed	Satisfactory	NILM	NILM	Negative (NILM)	
P01017	2018	2018-Q2	2018-06	Pre-pandemic	Cytology (Pap)	60-64	64	Not performed	Satisfactory	NILM	NILM	Negative (NILM)	
P01018	2018	2018-Q2	2018-06	Pre-pandemic	Cytology (Pap)	50-54	50	Not performed	Satisfactory	NILM	NILM	Negative (NILM)	
P01019	2018	2018-Q2	2018-06	Pre-pandemic	Cytology (Pap)	25-29	25	Not performed	Satisfactory	NILM	NILM	Negative (NILM)	
P01020	2018	2018-Q2	2018-06	Pre-pandemic	Cytology (Pap)	45-49	46	Not performed	Satisfactory	NILM	NILM	Negative (NILM)	
P01021	2018	2018-Q2	2018-06	Pre-pandemic	Cytology (Pap)	40-44	44	Not performed	Satisfactory	NILM	NILM	Negative (NILM)	
P01022	2018	2018-Q2	2018-06	Pre-pandemic	Cytology (Pap)	40-44	41	Not performed	Satisfactory	NILM	NILM	Negative (NILM)	
P01023	2018	2018-Q2	2018-06	Pre-pandemic	Cytology (Pap)	55-59	56	Not performed	Satisfactory	NILM	NILM	Negative (NILM)	
P01024	2018	2018-Q2	2018-06	Pre-pandemic	Cytology (Pap)	35-39	36	Not performed	Satisfactory	Positive	AGC	Glandular	
P01025	2018	2018-Q2	2018-06	Pre-pandemic	Cytology (Pap)	40-44	42	Not performed	Satisfactory	NILM	NILM	Negative (NILM)	
P01026	2018	2018-Q2	2018-06	Pre-pandemic	Cytology (Pap)	40-44	40	Not performed	Satisfactory	NILM	NILM	Negative (NILM)	
P01027	2018	2018-Q2	2018-06	Pre-pandemic	Cytology (Pap)	50-54	52	Not performed	Satisfactory	NILM	NILM	Negative (NILM)	
P01028	2018	2018-Q2	2018-06	Pre-pandemic	Cytology (Pap)	40-44	40	Not performed	Satisfactory	NILM	NILM	Negative (NILM)	
P01029	2018	2018-Q2	2018-06	Pre-pandemic	Cytology (Pap)	40-44	40	Not performed	Satisfactory	NILM	NILM	Negative (NILM)	
P01030	2018	2018-Q2	2018-06	Pre-pandemic	Cytology (Pap)	40-44	41	Not performed	Satisfactory	Positive	HSIL	High-grade squamous	
P01031	2018	2018-Q2	2018-06	Pre-pandemic	Cytology (Pap)	40-44	40	Not performed	Satisfactory	NILM	NILM	Negative (NILM)	
P01032	2018	2018-Q2	2018-06	Pre-pandemic	Cytology (Pap)	40-44	41	Not performed	Satisfactory	Positive	HSIL	High-grade squamous	
P01033	2018	2018-Q2	2018-06	Pre-pandemic	Cytology (Pap)	30-34	31	Not performed	Satisfactory	NILM	NILM	Negative (NILM)	
P01034	2018	2018-Q2	2018-06	Pre-pandemic	Cytology (Pap)	55-59	59	Not performed	Satisfactory	NILM	NILM	Negative (NILM)	
P01035	2018	2018-Q2	2018-06	Pre-pandemic	Cytology (Pap)	25-29	26	Not performed	Satisfactory	NILM	NILM	Negative (NILM)	
P01036	2018	2018-Q2	2018-06	Pre-pandemic	Cytology (Pap)	60-64	61	Not performed	Satisfactory	NILM	NILM	Negative (NILM)	
P01037	2018	2018-Q2	2018-06	Pre-pandemic	Cytology (Pap)	35-39	39	Not performed	Satisfactory	NILM	NILM	Negative (NILM)	
P01038	2018	2018-Q2	2018-06	Pre-pandemic	Cytology (Pap)	25-29	25	Not performed	Satisfactory	NILM	NILM	Negative (NILM)	

patient_id	year	quarter	month	period	primary_test	age_group	age_years	hpv_result	adequacy	result	bethesda	grade	infection
P01039	2018	2018-Q2	2018-06	Pre-pandemic	Cytology (Pap)	40-44	41	Not performed	Satisfactory	NILM	NILM	Negative (NILM)	
P01040	2018	2018-Q2	2018-06	Pre-pandemic	Cytology (Pap)	45-49	48	Not performed	Satisfactory	NILM	NILM	Negative (NILM)	
P01041	2018	2018-Q2	2018-06	Pre-pandemic	Cytology (Pap)	30-34	32	Not performed	Satisfactory	NILM	NILM	Negative (NILM)	
P01042	2018	2018-Q2	2018-06	Pre-pandemic	Cytology (Pap)	40-44	43	Not performed	Satisfactory	NILM	NILM	Negative (NILM)	
P01043	2018	2018-Q2	2018-06	Pre-pandemic	Cytology (Pap)	40-44	42	Not performed	Satisfactory	NILM	NILM	Negative (NILM)	
P01044	2018	2018-Q2	2018-06	Pre-pandemic	Cytology (Pap)	45-49	47	Not performed	Satisfactory	NILM	NILM	Negative (NILM)	
P01045	2018	2018-Q2	2018-06	Pre-pandemic	Cytology (Pap)	60-64	62	Not performed	Satisfactory	NILM	NILM	Negative (NILM)	
P01046	2018	2018-Q2	2018-06	Pre-pandemic	Cytology (Pap)	40-44	42	Not performed	Satisfactory	NILM	NILM	Negative (NILM)	
P01047	2018	2018-Q2	2018-06	Pre-pandemic	Cytology (Pap)	55-59	56	Not performed	Satisfactory	NILM	NILM	Negative (NILM)	
P01048	2018	2018-Q2	2018-06	Pre-pandemic	Cytology (Pap)	35-39	37	Not performed	Satisfactory	NILM	NILM	Negative (NILM)	
P01049	2018	2018-Q2	2018-06	Pre-pandemic	Cytology (Pap)	30-34	30	Not performed	Satisfactory	NILM	NILM	Negative (NILM)	
P01050	2018	2018-Q2	2018-06	Pre-pandemic	Cytology (Pap)	30-34	33	Not performed	Satisfactory	NILM	NILM	Negative (NILM)	
P01051	2018	2018-Q2	2018-06	Pre-pandemic	Cytology (Pap)	60-64	60	Not performed	Satisfactory	NILM	NILM	Negative (NILM)	
P01052	2018	2018-Q2	2018-06	Pre-pandemic	Cytology (Pap)	35-39	36	Not performed	Satisfactory	NILM	NILM	Negative (NILM)	
P01053	2018	2018-Q2	2018-06	Pre-pandemic	Cytology (Pap)	40-44	44	Not performed	Satisfactory	NILM	NILM	Negative (NILM)	
P01054	2018	2018-Q2	2018-06	Pre-pandemic	Cytology (Pap)	30-34	30	Not performed	Satisfactory	Positive	ASC-US	Low-grade / equivocal squamous	
P01055	2018	2018-Q2	2018-06	Pre-pandemic	Cytology (Pap)	45-49	46	Not performed	Satisfactory	NILM	NILM	Negative (NILM)	
P01056	2018	2018-Q2	2018-06	Pre-pandemic	Cytology (Pap)	55-59	58	Not performed	Satisfactory	Positive	ASC-US	Low-grade / equivocal squamous	
P01057	2018	2018-Q2	2018-06	Pre-pandemic	Cytology (Pap)	40-44	42	Not performed	Satisfactory	NILM	NILM	Negative (NILM)	
P01058	2018	2018-Q2	2018-06	Pre-pandemic	Cytology (Pap)	35-39	37	Not performed	Satisfactory	NILM	NILM	Negative (NILM)	
P01059	2018	2018-Q2	2018-06	Pre-pandemic	Cytology (Pap)	30-34	32	Not performed	Satisfactory	NILM	NILM	Negative (NILM)	
P01060	2018	2018-Q2	2018-06	Pre-pandemic	Cytology (Pap)	45-49	46	Not performed	Satisfactory	NILM	NILM	Negative (NILM)	
P01061	2018	2018-Q2	2018-06	Pre-pandemic	Cytology (Pap)	25-29	28	Not performed	Satisfactory	NILM	NILM	Negative (NILM)	
P01062	2018	2018-Q2	2018-06	Pre-pandemic	Cytology (Pap)	35-39	37	Not performed	Satisfactory	Positive	ASC-US	Low-grade / equivocal squamous	
P01063	2018	2018-Q2	2018-06	Pre-pandemic	Cytology (Pap)	35-39	37	Not performed	Satisfactory	NILM	NILM	Negative (NILM)	
P01064	2018	2018-Q2	2018-06	Pre-pandemic	Cytology (Pap)	60-64	63	Not performed	Satisfactory	NILM	NILM	Negative (NILM)	
P01065	2018	2018-Q2	2018-06	Pre-pandemic	Cytology (Pap)	40-44	43	Not performed	Satisfactory	NILM	NILM	Negative (NILM)	
P01066	2018	2018-Q2	2018-06	Pre-pandemic	Cytology (Pap)	60-64	60	Not performed	Satisfactory	Positive	LSIL	Low-grade / equivocal squamous	
P01067	2018	2018-Q2	2018-06	Pre-pandemic	Cytology (Pap)	35-39	38	Not performed	Satisfactory	NILM	NILM	Negative (NILM)	
P01068	2018	2018-Q2	2018-06	Pre-pandemic	Cytology (Pap)	40-44	40	Not performed	Satisfactory	NILM	NILM	Negative (NILM)	
P01069	2018	2018-Q2	2018-06	Pre-pandemic	Cytology (Pap)	30-34	32	Not performed	Satisfactory	NILM	NILM	Negative (NILM)	
P01070	2018	2018-Q2	2018-06	Pre-pandemic	Cytology (Pap)	50-54	54	Not performed	Unsatisfactory	Unsatisfactory	Unsatisfactory	Unsatisfactory	Candida
P01071	2018	2018-Q2	2018-06	Pre-pandemic	Cytology (Pap)	40-44	44	Not performed	Satisfactory	NILM	NILM	Negative (NILM)	
P01072	2018	2018-Q2	2018-06	Pre-pandemic	Cytology (Pap)	45-49	45	Not performed	Satisfactory	NILM	NILM	Negative (NILM)	
P01073	2018	2018-Q2	2018-06	Pre-pandemic	Cytology (Pap)	45-49	47	Not performed	Satisfactory	NILM	NILM	Negative (NILM)	
P01074	2018	2018-Q2	2018-06	Pre-pandemic	Cytology (Pap)	25-29	25	Not performed	Satisfactory	NILM	NILM	Negative (NILM)	
P01075	2018	2018-Q2	2018-06	Pre-pandemic	Cytology (Pap)	35-39	35	Not performed	Satisfactory	NILM	NILM	Negative (NILM)	
P01076	2018	2018-Q2	2018-06	Pre-pandemic	Cytology (Pap)	40-44	43	Not performed	Satisfactory	NILM	NILM	Negative (NILM)	
P01077	2018	2018-Q2	2018-06	Pre-pandemic	Cytology (Pap)	55-59	57	Not performed	Satisfactory	NILM	NILM	Negative (NILM)	Other
P01078	2018	2018-Q2	2018-06	Pre-pandemic	Cytology (Pap)	35-39	35	Not performed	Satisfactory	NILM	NILM	Negative (NILM)	
P01079	2018	2018-Q2	2018-06	Pre-pandemic	Cytology (Pap)	40-44	41	Not performed	Satisfactory	Positive	ASC-US	Low-grade / equivocal squamous	
P01080	2018	2018-Q2	2018-06	Pre-pandemic	Cytology (Pap)	Not recorded		Not performed	Satisfactory	NILM	NILM	Negative (NILM)	
P01081	2018	2018-Q2	2018-06	Pre-pandemic	Cytology (Pap)	50-54	52	Not performed	Satisfactory	Positive	HSIL	High-grade squamous	
P01082	2018	2018-Q2	2018-06	Pre-pandemic	Cytology (Pap)	50-54	52	Not performed	Satisfactory	NILM	NILM	Negative (NILM)	
P01083	2018	2018-Q2	2018-06	Pre-pandemic	Cytology (Pap)	40-44	42	Not performed	Satisfactory	NILM	NILM	Negative (NILM)	Trichomonas
P01084	2018	2018-Q2	2018-06	Pre-pandemic	Cytology (Pap)	40-44	44	Not performed	Satisfactory	NILM	NILM	Negative (NILM)	
P01085	2018	2018-Q2	2018-06	Pre-pandemic	Cytology (Pap)	55-59	57	Not performed	Satisfactory	NILM	NILM	Negative (NILM)	
P01086	2018	2018-Q2	2018-06	Pre-pandemic	Cytology (Pap)	40-44	42	Not performed	Satisfactory	NILM	NILM	Negative (NILM)	
P01087	2018	2018-Q2	2018-06	Pre-pandemic	Cytology (Pap)	60-64	62	Not performed	Satisfactory	NILM	NILM	Negative (NILM)	
P01088	2018	2018-Q2	2018-06	Pre-pandemic	Cytology (Pap)	35-39	37	Not performed	Satisfactory	NILM	NILM	Negative (NILM)	Gardnerella
P01089	2018	2018-Q2	2018-06	Pre-pandemic	Cytology (Pap)	30-34	33	Not performed	Satisfactory	NILM	NILM	Negative (NILM)	
P01090	2018	2018-Q2	2018-06	Pre-pandemic	Cytology (Pap)	35-39	36	Not performed	Satisfactory	Positive	ASC-US	Low-grade / equivocal squamous	
P01091	2018	2018-Q2	2018-06	Pre-pandemic	Cytology (Pap)	30-34	34	Not performed	Satisfactory	NILM	NILM	Negative (NILM)	
P01092	2018	2018-Q2	2018-06	Pre-pandemic	Cytology (Pap)	35-39	36	Not performed	Satisfactory	NILM	NILM	Negative (NILM)	
P01093	2018	2018-Q2	2018-06	Pre-pandemic	Cytology (Pap)	Not recorded		Not performed	Satisfactory	NILM	NILM	Negative (NILM)	
P01094	2018	2018-Q2	2018-06	Pre-pandemic	Cytology (Pap)	45-49	47	Not performed	Satisfactory	NILM	NILM	Negative (NILM)	Candida
P01095	2018	2018-Q2	2018-06	Pre-pandemic	Cytology (Pap)	40-44	44	Not performed	Satisfactory	NILM	NILM	Negative (NILM)	
P01096	2018	2018-Q2	2018-06	Pre-pandemic	Cytology (Pap)	25-29	25	Not performed	Satisfactory	Positive	ASC-US	Low-grade / equivocal squamous	
P01097	2018	2018-Q2	2018-06	Pre-pandemic	Cytology (Pap)	35-39	35	Not performed	Satisfactory	NILM	NILM	Negative (NILM)	
P01098	2018	2018-Q2	2018-06	Pre-pandemic	Cytology (Pap)	45-49	48	Not performed	Satisfactory	NILM	NILM	Negative (NILM)	
P01099	2018	2018-Q2	2018-06	Pre-pandemic	Cytology (Pap)	35-39	35	Not performed	Satisfactory	NILM	NILM	Negative (NILM)	
P01100	2018	2018-Q2	2018-06	Pre-pandemic	Cytology (Pap)	30-34	30	Not performed	Satisfactory	NILM	NILM	Negative (NILM)	
P01101	2018	2018-Q2	2018-06	Pre-pandemic	Cytology (Pap)	40-44	41	Not performed	Satisfactory	NILM	NILM	Negative (NILM)	
P01102	2018	2018-Q2	2018-06	Pre-pandemic	Cytology (Pap)	50-54	50	Not performed	Satisfactory	NILM	NILM	Negative (NILM)	
P01103	2018	2018-Q2	2018-06	Pre-pandemic	Cytology (Pap)	40-44	42	Not performed	Satisfactory	NILM	NILM	Negative (NILM)	
P01104	2018	2018-Q2	2018-06	Pre-pandemic	Cytology (Pap)	40-44	43	Not performed	Satisfactory	NILM	NILM	Negative (NILM)	
P01105	2018	2018-Q2	2018-06	Pre-pandemic	Cytology (Pap)	60-64	62	Not performed	Satisfactory	NILM	NILM	Negative (NILM)	
P01106	2018	2018-Q2	2018-06	Pre-pandemic	Cytology (Pap)	50-54	51	Not performed	Satisfactory	NILM	NILM	Negative (NILM)	
P01107	2018	2018-Q2	2018-06	Pre-pandemic	Cytology (Pap)	45-49	46	Not performed	Satisfactory	Positive	ASC-US	Low-grade / equivocal squamous	
P01108	2018	2018-Q2	2018-06	Pre-pandemic	Cytology (Pap)	40-44	40	Not performed	Satisfactory	NILM	NILM	Negative (NILM)	
P01109	2018	2018-Q2	2018-06	Pre-pandemic	Cytology (Pap)	45-49	46	Not performed	Satisfactory	NILM	NILM	Negative (NILM)	
P01110	2018	2018-Q2	2018-06	Pre-pandemic	Cytology (Pap)	55-59	59	Not performed	Satisfactory	NILM	NILM	Negative (NILM)	
P01111	2018	2018-Q2	2018-06	Pre-pandemic	Cytology (Pap)	35-39	37	Not performed	Satisfactory	NILM	NILM	Negative (NILM)	
P01112	2018	2018-Q2	2018-06	Pre-pandemic	Cytology (Pap)	40-44	44	Not performed	Satisfactory	NILM	NILM	Negative (NILM)	
P01113	2018	2018-Q2	2018-06	Pre-pandemic	Cytology (Pap)	35-39	38	Not performed	Satisfactory	NILM	NILM	Negative (NILM)	
P01114	2018	2018-Q2	2018-06	Pre-pandemic	Cytology (Pap)	40-44	41	Not performed	Satisfactory	NILM	NILM	Negative (NILM)	
P01115	2018	2018-Q2	2018-06	Pre-pandemic	Cytology (Pap)	40-44	40	Not performed	Satisfactory	NILM	NILM	Negative (NILM)	
P01116	2018	2018-Q2	2018-06	Pre-pandemic	Cytology (Pap)	40-44	42	Not performed	Satisfactory	NILM	NILM	Negative (NILM)	
P01117	2018	2018-Q2	2018-06	Pre-pandemic	Cytology (Pap)	Not recorded		Not performed	Satisfactory	NILM	NILM	Negative (NILM)	
P01118	2018	2018-Q2	2018-06	Pre-pandemic	Cytology (Pap)	60-64	62	Not performed	Satisfactory	NILM	NILM	Negative (NILM)	

patient_id	year	quarter	month	period	primary_test	age_group	age_years	hpv_result	adequacy	result	bethesda	grade	infection
P01119	2018	2018-Q2	2018-06	Pre-pandemic	Cytology (Pap)	55-59	56	Not performed	Satisfactory	NILM	NILM	Negative (NILM)	
P01120	2018	2018-Q2	2018-06	Pre-pandemic	Cytology (Pap)	35-39	38	Not performed	Satisfactory	NILM	NILM	Negative (NILM)	
P01121	2018	2018-Q2	2018-06	Pre-pandemic	Cytology (Pap)	50-54	52	Not performed	Satisfactory	Positive	ASC-H	High-grade squamous	
P01122	2018	2018-Q2	2018-06	Pre-pandemic	Cytology (Pap)	40-44	43	Not performed	Unsatisfactory	Unsatisfactory	Unsatisfactory	Unsatisfactory	
P01123	2018	2018-Q2	2018-06	Pre-pandemic	Cytology (Pap)	60-64	61	Not performed	Satisfactory	NILM	NILM	Negative (NILM)	
P01124	2018	2018-Q2	2018-06	Pre-pandemic	Cytology (Pap)	50-54	51	Not performed	Satisfactory	Positive	HSIL	High-grade squamous	
P01125	2018	2018-Q2	2018-06	Pre-pandemic	Cytology (Pap)	40-44	44	Not performed	Satisfactory	NILM	NILM	Negative (NILM)	
P01126	2018	2018-Q2	2018-06	Pre-pandemic	Cytology (Pap)	60-64	60	Not performed	Satisfactory	NILM	NILM	Negative (NILM)	
P01127	2018	2018-Q2	2018-06	Pre-pandemic	Cytology (Pap)	30-34	30	Not performed	Satisfactory	NILM	NILM	Negative (NILM)	
P01128	2018	2018-Q2	2018-06	Pre-pandemic	Cytology (Pap)	30-34	31	Not performed	Satisfactory	NILM	NILM	Negative (NILM)	
P01129	2018	2018-Q2	2018-06	Pre-pandemic	Cytology (Pap)	35-39	35	Not performed	Satisfactory	NILM	NILM	Negative (NILM)	
P01130	2018	2018-Q2	2018-06	Pre-pandemic	Cytology (Pap)	45-49	47	Not performed	Satisfactory	Positive	LSIL	Low-grade / equivocal squamous	
P01131	2018	2018-Q2	2018-06	Pre-pandemic	Cytology (Pap)	55-59	57	Not performed	Satisfactory	NILM	NILM	Negative (NILM)	
P01132	2018	2018-Q2	2018-06	Pre-pandemic	Cytology (Pap)	30-34	33	Not performed	Satisfactory	NILM	NILM	Negative (NILM)	
P01133	2018	2018-Q2	2018-06	Pre-pandemic	Cytology (Pap)	45-49	46	Not performed	Satisfactory	NILM	NILM	Negative (NILM)	
P01134	2018	2018-Q2	2018-06	Pre-pandemic	Cytology (Pap)	40-44	40	Not performed	Satisfactory	NILM	NILM	Negative (NILM)	
P01135	2018	2018-Q2	2018-06	Pre-pandemic	Cytology (Pap)	50-54	51	Not performed	Satisfactory	NILM	NILM	Negative (NILM)	
P01136	2018	2018-Q2	2018-06	Pre-pandemic	Cytology (Pap)	35-39	37	Not performed	Satisfactory	NILM	NILM	Negative (NILM)	
P01137	2018	2018-Q2	2018-06	Pre-pandemic	Cytology (Pap)	40-44	42	Not performed	Satisfactory	NILM	NILM	Negative (NILM)	
P01138	2018	2018-Q2	2018-06	Pre-pandemic	Cytology (Pap)	35-39	39	Not performed	Satisfactory	NILM	NILM	Negative (NILM)	
P01139	2018	2018-Q2	2018-06	Pre-pandemic	Cytology (Pap)	55-59	57	Not performed	Satisfactory	Positive	LSIL	Low-grade / equivocal squamous	
P01140	2018	2018-Q2	2018-06	Pre-pandemic	Cytology (Pap)	40-44	41	Not performed	Satisfactory	NILM	NILM	Negative (NILM)	
P01141	2018	2018-Q2	2018-06	Pre-pandemic	Cytology (Pap)	40-44	42	Not performed	Satisfactory	Positive	ASC-US	Low-grade / equivocal squamous	
P01142	2018	2018-Q2	2018-06	Pre-pandemic	Cytology (Pap)	40-44	41	Not performed	Satisfactory	NILM	NILM	Negative (NILM)	
P01143	2018	2018-Q2	2018-06	Pre-pandemic	Cytology (Pap)	35-39	35	Not performed	Satisfactory	NILM	NILM	Negative (NILM)	
P01144	2018	2018-Q2	2018-06	Pre-pandemic	Cytology (Pap)	55-59	56	Not performed	Satisfactory	Positive	ASC-H	High-grade squamous	
P01145	2018	2018-Q2	2018-06	Pre-pandemic	Cytology (Pap)	40-44	42	Not performed	Satisfactory	NILM	NILM	Negative (NILM)	
P01146	2018	2018-Q2	2018-06	Pre-pandemic	Cytology (Pap)	30-34	33	Not performed	Satisfactory	NILM	NILM	Negative (NILM)	
P01147	2018	2018-Q2	2018-06	Pre-pandemic	Cytology (Pap)	40-44	40	Not performed	Satisfactory	Positive	LSIL	Low-grade / equivocal squamous	
P01148	2018	2018-Q2	2018-06	Pre-pandemic	Cytology (Pap)	50-54	54	Not performed	Satisfactory	NILM	NILM	Negative (NILM)	
P01149	2018	2018-Q2	2018-06	Pre-pandemic	Cytology (Pap)	55-59	55	Not performed	Satisfactory	NILM	NILM	Negative (NILM)	
P01150	2018	2018-Q2	2018-06	Pre-pandemic	Cytology (Pap)	55-59	59	Not performed	Satisfactory	NILM	NILM	Negative (NILM)	
P01151	2018	2018-Q2	2018-06	Pre-pandemic	Cytology (Pap)	50-54	51	Not performed	Satisfactory	NILM	NILM	Negative (NILM)	
P01152	2018	2018-Q2	2018-06	Pre-pandemic	Cytology (Pap)	35-39	35	Not performed	Satisfactory	NILM	NILM	Negative (NILM)	
P01153	2018	2018-Q2	2018-06	Pre-pandemic	Cytology (Pap)	25-29	29	Not performed	Satisfactory	NILM	NILM	Negative (NILM)	
P01154	2018	2018-Q2	2018-06	Pre-pandemic	Cytology (Pap)	40-44	41	Not performed	Unsatisfactory	Unsatisfactory	Unsatisfactory	Unsatisfactory	
P01155	2018	2018-Q2	2018-06	Pre-pandemic	Cytology (Pap)	45-49	47	Not performed	Satisfactory	Positive	LSIL	Low-grade / equivocal squamous	Trichomonas
P01156	2018	2018-Q2	2018-06	Pre-pandemic	Cytology (Pap)	30-34	33	Not performed	Satisfactory	NILM	NILM	Negative (NILM)	
P01157	2018	2018-Q2	2018-06	Pre-pandemic	Cytology (Pap)	35-39	38	Not performed	Satisfactory	Positive	ASC-US	Low-grade / equivocal squamous	
P01158	2018	2018-Q2	2018-06	Pre-pandemic	Cytology (Pap)	50-54	52	Not performed	Satisfactory	NILM	NILM	Negative (NILM)	
P01159	2018	2018-Q2	2018-06	Pre-pandemic	Cytology (Pap)	35-39	36	Not performed	Satisfactory	NILM	NILM	Negative (NILM)	
P01160	2018	2018-Q2	2018-06	Pre-pandemic	Cytology (Pap)	30-34	34	Not performed	Satisfactory	NILM	NILM	Negative (NILM)	
P01161	2018	2018-Q2	2018-06	Pre-pandemic	Cytology (Pap)	35-39	36	Not performed	Satisfactory	NILM	NILM	Negative (NILM)	
P01162	2018	2018-Q2	2018-06	Pre-pandemic	Cytology (Pap)	50-54	51	Not performed	Satisfactory	NILM	NILM	Negative (NILM)	
P01163	2018	2018-Q2	2018-06	Pre-pandemic	Cytology (Pap)	40-44	43	Not performed	Satisfactory	NILM	NILM	Negative (NILM)	
P01164	2018	2018-Q2	2018-06	Pre-pandemic	Cytology (Pap)	50-54	54	Not performed	Satisfactory	NILM	NILM	Negative (NILM)	
P01165	2018	2018-Q2	2018-06	Pre-pandemic	Cytology (Pap)	45-49	48	Not performed	Satisfactory	NILM	NILM	Negative (NILM)	
P01166	2018	2018-Q2	2018-06	Pre-pandemic	Cytology (Pap)	35-39	37	Not performed	Satisfactory	NILM	NILM	Negative (NILM)	
P01167	2018	2018-Q2	2018-06	Pre-pandemic	Cytology (Pap)	40-44	43	Not performed	Satisfactory	NILM	NILM	Negative (NILM)	
P01168	2018	2018-Q2	2018-06	Pre-pandemic	Cytology (Pap)	60-64	61	Not performed	Satisfactory	NILM	NILM	Negative (NILM)	
P01169	2018	2018-Q2	2018-06	Pre-pandemic	Cytology (Pap)	50-54	53	Not performed	Satisfactory	NILM	NILM	Negative (NILM)	
P01170	2018	2018-Q2	2018-06	Pre-pandemic	Cytology (Pap)	35-39	36	Not performed	Satisfactory	NILM	NILM	Negative (NILM)	
P01171	2018	2018-Q2	2018-06	Pre-pandemic	Cytology (Pap)	60-64	62	Not performed	Satisfactory	NILM	NILM	Negative (NILM)	
P01172	2018	2018-Q2	2018-06	Pre-pandemic	Cytology (Pap)	25-29	29	Not performed	Satisfactory	NILM	NILM	Negative (NILM)	Trichomonas
P01173	2018	2018-Q2	2018-06	Pre-pandemic	Cytology (Pap)	60-64	60	Not performed	Satisfactory	NILM	NILM	Negative (NILM)	
P01174	2018	2018-Q2	2018-06	Pre-pandemic	Cytology (Pap)	35-39	36	Not performed	Satisfactory	NILM	NILM	Negative (NILM)	
P01175	2018	2018-Q2	2018-06	Pre-pandemic	Cytology (Pap)	40-44	42	Not performed	Satisfactory	NILM	NILM	Negative (NILM)	
P01176	2018	2018-Q2	2018-06	Pre-pandemic	Cytology (Pap)	45-49	48	Not performed	Unsatisfactory	Unsatisfactory	Unsatisfactory	Unsatisfactory	
P01177	2018	2018-Q2	2018-06	Pre-pandemic	Cytology (Pap)	30-34	31	Not performed	Satisfactory	NILM	NILM	Negative (NILM)	
P01178	2018	2018-Q2	2018-06	Pre-pandemic	Cytology (Pap)	45-49	45	Not performed	Satisfactory	Positive	LSIL	Low-grade / equivocal squamous	
P01179	2018	2018-Q2	2018-06	Pre-pandemic	Cytology (Pap)	35-39	39	Not performed	Satisfactory	NILM	NILM	Negative (NILM)	
P01180	2018	2018-Q2	2018-06	Pre-pandemic	Cytology (Pap)	30-34	30	Not performed	Satisfactory	NILM	NILM	Negative (NILM)	
P01181	2018	2018-Q2	2018-06	Pre-pandemic	Cytology (Pap)	40-44	41	Not performed	Satisfactory	Positive	LSIL	Low-grade / equivocal squamous	
P01182	2018	2018-Q2	2018-06	Pre-pandemic	Cytology (Pap)	35-39	35	Not performed	Satisfactory	NILM	NILM	Negative (NILM)	
P01183	2018	2018-Q2	2018-06	Pre-pandemic	Cytology (Pap)	50-54	51	Not performed	Satisfactory	NILM	NILM	Negative (NILM)	
P01184	2018	2018-Q2	2018-06	Pre-pandemic	Cytology (Pap)	45-49	47	Not performed	Satisfactory	NILM	NILM	Negative (NILM)	
P01185	2018	2018-Q2	2018-06	Pre-pandemic	Cytology (Pap)	35-39	38	Not performed	Satisfactory	Positive	ASC-US	Low-grade / equivocal squamous	Candida
P01186	2018	2018-Q2	2018-06	Pre-pandemic	Cytology (Pap)	50-54	50	Not performed	Satisfactory	NILM	NILM	Negative (NILM)	
P01187	2018	2018-Q2	2018-06	Pre-pandemic	Cytology (Pap)	40-44	40	Not performed	Satisfactory	NILM	NILM	Negative (NILM)	
P01188	2018	2018-Q2	2018-06	Pre-pandemic	Cytology (Pap)	45-49	45	Not performed	Satisfactory	NILM	NILM	Negative (NILM)	
P01189	2018	2018-Q2	2018-06	Pre-pandemic	Cytology (Pap)	40-44	41	Not performed	Satisfactory	Positive	HSIL	High-grade squamous	
P01190	2018	2018-Q2	2018-06	Pre-pandemic	Cytology (Pap)	45-49	45	Not performed	Satisfactory	NILM	NILM	Negative (NILM)	
P01191	2018	2018-Q2	2018-06	Pre-pandemic	Cytology (Pap)	35-39	36	Not performed	Satisfactory	NILM	NILM	Negative (NILM)	
P01192	2018	2018-Q2	2018-06	Pre-pandemic	Cytology (Pap)	35-39	35	Not performed	Satisfactory	NILM	NILM	Negative (NILM)	
P01193	2018	2018-Q2	2018-06	Pre-pandemic	Cytology (Pap)	30-34	30	Not performed	Satisfactory	NILM	NILM	Negative (NILM)	
P01194	2018	2018-Q2	2018-06	Pre-pandemic	Cytology (Pap)	55-59	55	Not performed	Satisfactory	NILM	NILM	Negative (NILM)	
P01195	2018	2018-Q2	2018-06	Pre-pandemic	Cytology (Pap)	45-49	45	Not performed	Satisfactory	Positive	ASC-US	Low-grade / equivocal squamous	
P01196	2018	2018-Q2	2018-06	Pre-pandemic	Cytology (Pap)	40-44	40	Not performed	Satisfactory	NILM	NILM	Negative (NILM)	
P01197	2018	2018-Q2	2018-06	Pre-pandemic	Cytology (Pap)	45-49	45	Not performed	Satisfactory	NILM	NILM	Negative (NILM)	
P01198	2018	2018-Q2	2018-06	Pre-pandemic	Cytology (Pap)	35-39	37	Not performed	Satisfactory	NILM	NILM	Negative (NILM)	

patient_id	year	quarter	month	period	primary_test	age_group	age_years	hpv_result	adequacy	result	bethesda	grade	infection
P01199	2018	2018-Q2	2018-06	Pre-pandemic	Cytology (Pap)	50-54	52	Not performed	Satisfactory	NILM	NILM	Negative (NILM)	
P01200	2018	2018-Q2	2018-06	Pre-pandemic	Cytology (Pap)	55-59	57	Not performed	Satisfactory	NILM	NILM	Negative (NILM)	
P01201	2018	2018-Q2	2018-06	Pre-pandemic	Cytology (Pap)	35-39	36	Not performed	Satisfactory	NILM	NILM	Negative (NILM)	
P01202	2018	2018-Q2	2018-06	Pre-pandemic	Cytology (Pap)	40-44	43	Not performed	Satisfactory	NILM	NILM	Negative (NILM)	
P01203	2018	2018-Q2	2018-06	Pre-pandemic	Cytology (Pap)	60-64	63	Not performed	Satisfactory	NILM	NILM	Negative (NILM)	
P01204	2018	2018-Q2	2018-06	Pre-pandemic	Cytology (Pap)	30-34	34	Not performed	Satisfactory	NILM	NILM	Negative (NILM)	
P01205	2018	2018-Q2	2018-06	Pre-pandemic	Cytology (Pap)	35-39	35	Not performed	Satisfactory	NILM	NILM	Negative (NILM)	
P01206	2018	2018-Q2	2018-06	Pre-pandemic	Cytology (Pap)	40-44	43	Not performed	Satisfactory	NILM	NILM	Negative (NILM)	
P01207	2018	2018-Q2	2018-06	Pre-pandemic	Cytology (Pap)	25-29	26	Not performed	Satisfactory	NILM	NILM	Negative (NILM)	
P01208	2018	2018-Q3	2018-07	Pre-pandemic	Cytology (Pap)	40-44	43	Not performed	Satisfactory	NILM	NILM	Negative (NILM)	
P01209	2018	2018-Q3	2018-07	Pre-pandemic	Cytology (Pap)	55-59	56	Not performed	Satisfactory	NILM	NILM	Negative (NILM)	
P01210	2018	2018-Q3	2018-07	Pre-pandemic	Cytology (Pap)	45-49	49	Not performed	Satisfactory	NILM	NILM	Negative (NILM)	
P01211	2018	2018-Q3	2018-07	Pre-pandemic	Cytology (Pap)	35-39	38	Not performed	Satisfactory	NILM	NILM	Negative (NILM)	Candida
P01212	2018	2018-Q3	2018-07	Pre-pandemic	Cytology (Pap)	35-39	37	Not performed	Satisfactory	NILM	NILM	Negative (NILM)	Trichomonas
P01213	2018	2018-Q3	2018-07	Pre-pandemic	Cytology (Pap)	35-39	35	Not performed	Satisfactory	NILM	NILM	Negative (NILM)	
P01214	2018	2018-Q3	2018-07	Pre-pandemic	Cytology (Pap)	50-54	52	Not performed	Satisfactory	NILM	NILM	Negative (NILM)	
P01215	2018	2018-Q3	2018-07	Pre-pandemic	Cytology (Pap)	40-44	41	Not performed	Satisfactory	NILM	NILM	Negative (NILM)	
P01216	2018	2018-Q3	2018-07	Pre-pandemic	Cytology (Pap)	60-64	62	Not performed	Satisfactory	NILM	NILM	Negative (NILM)	
P01217	2018	2018-Q3	2018-07	Pre-pandemic	Cytology (Pap)	30-34	32	Not performed	Satisfactory	NILM	NILM	Negative (NILM)	
P01218	2018	2018-Q3	2018-07	Pre-pandemic	Cytology (Pap)	25-29	29	Not performed	Satisfactory	Positive	LSIL	Low-grade / equivocal squamous	
P01219	2018	2018-Q3	2018-07	Pre-pandemic	Cytology (Pap)	30-34	30	Not performed	Satisfactory	NILM	NILM	Negative (NILM)	
P01220	2018	2018-Q3	2018-07	Pre-pandemic	Cytology (Pap)	40-44	41	Not performed	Satisfactory	NILM	NILM	Negative (NILM)	
P01221	2018	2018-Q3	2018-07	Pre-pandemic	Cytology (Pap)	25-29	26	Not performed	Satisfactory	NILM	NILM	Negative (NILM)	
P01222	2018	2018-Q3	2018-07	Pre-pandemic	Cytology (Pap)	55-59	55	Not performed	Satisfactory	NILM	NILM	Negative (NILM)	
P01223	2018	2018-Q3	2018-07	Pre-pandemic	Cytology (Pap)	25-29	29	Not performed	Satisfactory	NILM	NILM	Negative (NILM)	
P01224	2018	2018-Q3	2018-07	Pre-pandemic	Cytology (Pap)	30-34	34	Not performed	Satisfactory	Positive	ASC-US	Low-grade / equivocal squamous	
P01225	2018	2018-Q3	2018-07	Pre-pandemic	Cytology (Pap)	45-49	47	Not performed	Satisfactory	NILM	NILM	Negative (NILM)	
P01226	2018	2018-Q3	2018-07	Pre-pandemic	Cytology (Pap)	25-29	26	Not performed	Satisfactory	NILM	NILM	Negative (NILM)	
P01227	2018	2018-Q3	2018-07	Pre-pandemic	Cytology (Pap)	40-44	44	Not performed	Satisfactory	NILM	NILM	Negative (NILM)	
P01228	2018	2018-Q3	2018-07	Pre-pandemic	Cytology (Pap)	40-44	43	Not performed	Satisfactory	NILM	NILM	Negative (NILM)	
P01229	2018	2018-Q3	2018-07	Pre-pandemic	Cytology (Pap)	50-54	50	Not performed	Satisfactory	Positive	HSIL	High-grade squamous	
P01230	2018	2018-Q3	2018-07	Pre-pandemic	Cytology (Pap)	45-49	47	Not performed	Satisfactory	NILM	NILM	Negative (NILM)	
P01231	2018	2018-Q3	2018-07	Pre-pandemic	Cytology (Pap)	40-44	44	Not performed	Satisfactory	NILM	NILM	Negative (NILM)	
P01232	2018	2018-Q3	2018-07	Pre-pandemic	Cytology (Pap)	55-59	58	Not performed	Satisfactory	NILM	NILM	Negative (NILM)	
P01233	2018	2018-Q3	2018-07	Pre-pandemic	Cytology (Pap)	25-29	26	Not performed	Satisfactory	NILM	NILM	Negative (NILM)	
P01234	2018	2018-Q3	2018-07	Pre-pandemic	Cytology (Pap)	40-44	40	Not performed	Satisfactory	NILM	NILM	Negative (NILM)	
P01235	2018	2018-Q3	2018-07	Pre-pandemic	Cytology (Pap)	50-54	50	Not performed	Satisfactory	NILM	NILM	Negative (NILM)	
P01236	2018	2018-Q3	2018-07	Pre-pandemic	Cytology (Pap)	50-54	51	Not performed	Satisfactory	NILM	NILM	Negative (NILM)	
P01237	2018	2018-Q3	2018-07	Pre-pandemic	Cytology (Pap)	35-39	38	Not performed	Satisfactory	NILM	NILM	Negative (NILM)	
P01238	2018	2018-Q3	2018-07	Pre-pandemic	Cytology (Pap)	50-54	50	Not performed	Unsatisfactory	Unsatisfactory	Unsatisfactory	Unsatisfactory	
P01239	2018	2018-Q3	2018-07	Pre-pandemic	Cytology (Pap)	30-34	31	Not performed	Satisfactory	NILM	NILM	Negative (NILM)	
P01240	2018	2018-Q3	2018-07	Pre-pandemic	Cytology (Pap)	45-49	46	Not performed	Satisfactory	NILM	NILM	Negative (NILM)	
P01241	2018	2018-Q3	2018-07	Pre-pandemic	Cytology (Pap)	40-44	41	Not performed	Satisfactory	NILM	NILM	Negative (NILM)	
P01242	2018	2018-Q3	2018-07	Pre-pandemic	Cytology (Pap)	35-39	36	Not performed	Satisfactory	NILM	NILM	Negative (NILM)	
P01243	2018	2018-Q3	2018-07	Pre-pandemic	Cytology (Pap)	30-34	33	Not performed	Satisfactory	Positive	ASC-US	Low-grade / equivocal squamous	
P01244	2018	2018-Q3	2018-07	Pre-pandemic	Cytology (Pap)	45-49	45	Not performed	Satisfactory	Positive	ASC-US	Low-grade / equivocal squamous	
P01245	2018	2018-Q3	2018-07	Pre-pandemic	Cytology (Pap)	40-44	44	Not performed	Satisfactory	NILM	NILM	Negative (NILM)	
P01246	2018	2018-Q3	2018-07	Pre-pandemic	Cytology (Pap)	30-34	32	Not performed	Satisfactory	NILM	NILM	Negative (NILM)	
P01247	2018	2018-Q3	2018-07	Pre-pandemic	Cytology (Pap)	35-39	35	Not performed	Unsatisfactory	Unsatisfactory	Unsatisfactory	Unsatisfactory	
P01248	2018	2018-Q3	2018-07	Pre-pandemic	Cytology (Pap)	40-44	41	Not performed	Satisfactory	NILM	NILM	Negative (NILM)	Candida
P01249	2018	2018-Q3	2018-07	Pre-pandemic	Cytology (Pap)	45-49	46	Not performed	Satisfactory	NILM	NILM	Negative (NILM)	
P01250	2018	2018-Q3	2018-07	Pre-pandemic	Cytology (Pap)	45-49	49	Not performed	Satisfactory	NILM	NILM	Negative (NILM)	
P01251	2018	2018-Q3	2018-07	Pre-pandemic	Cytology (Pap)	35-39	39	Not performed	Satisfactory	NILM	NILM	Negative (NILM)	
P01252	2018	2018-Q3	2018-07	Pre-pandemic	Cytology (Pap)	40-44	40	Not performed	Satisfactory	NILM	NILM	Negative (NILM)	
P01253	2018	2018-Q3	2018-07	Pre-pandemic	Cytology (Pap)	25-29	29	Not performed	Satisfactory	NILM	NILM	Negative (NILM)	
P01254	2018	2018-Q3	2018-07	Pre-pandemic	Cytology (Pap)	45-49	48	Not performed	Satisfactory	NILM	NILM	Negative (NILM)	
P01255	2018	2018-Q3	2018-07	Pre-pandemic	Cytology (Pap)	45-49	49	Not performed	Satisfactory	NILM	NILM	Negative (NILM)	
P01256	2018	2018-Q3	2018-07	Pre-pandemic	Cytology (Pap)	35-39	38	Not performed	Satisfactory	NILM	NILM	Negative (NILM)	
P01257	2018	2018-Q3	2018-07	Pre-pandemic	Cytology (Pap)	45-49	49	Not performed	Satisfactory	NILM	NILM	Negative (NILM)	
P01258	2018	2018-Q3	2018-07	Pre-pandemic	Cytology (Pap)	45-49	47	Not performed	Satisfactory	Positive	HSIL	High-grade squamous	
P01259	2018	2018-Q3	2018-07	Pre-pandemic	Cytology (Pap)	40-44	44	Not performed	Satisfactory	NILM	NILM	Negative (NILM)	
P01260	2018	2018-Q3	2018-07	Pre-pandemic	Cytology (Pap)	45-49	47	Not performed	Satisfactory	NILM	NILM	Negative (NILM)	
P01261	2018	2018-Q3	2018-07	Pre-pandemic	Cytology (Pap)	40-44	44	Not performed	Satisfactory	NILM	NILM	Negative (NILM)	
P01262	2018	2018-Q3	2018-07	Pre-pandemic	Cytology (Pap)	55-59	58	Not performed	Satisfactory	NILM	NILM	Negative (NILM)	
P01263	2018	2018-Q3	2018-07	Pre-pandemic	Cytology (Pap)	25-29	26	Not performed	Satisfactory	NILM	NILM	Negative (NILM)	
P01264	2018	2018-Q3	2018-07	Pre-pandemic	Cytology (Pap)	45-49	49	Not performed	Satisfactory	NILM	NILM	Negative (NILM)	Gardnerella
P01265	2018	2018-Q3	2018-07	Pre-pandemic	Cytology (Pap)	50-54	53	Not performed	Satisfactory	NILM	NILM	Negative (NILM)	
P01266	2018	2018-Q3	2018-07	Pre-pandemic	Cytology (Pap)	50-54	54	Not performed	Satisfactory	NILM	NILM	Negative (NILM)	
P01267	2018	2018-Q3	2018-07	Pre-pandemic	Cytology (Pap)	50-54	52	Not performed	Satisfactory	NILM	NILM	Negative (NILM)	
P01268	2018	2018-Q3	2018-07	Pre-pandemic	Cytology (Pap)	60-64	63	Not performed	Satisfactory	Positive	HSIL	High-grade squamous	
P01269	2018	2018-Q3	2018-07	Pre-pandemic	Cytology (Pap)	30-34	31	Not performed	Satisfactory	NILM	NILM	Negative (NILM)	
P01270	2018	2018-Q3	2018-07	Pre-pandemic	Cytology (Pap)	55-59	59	Not performed	Satisfactory	NILM	NILM	Negative (NILM)	
P01271	2018	2018-Q3	2018-07	Pre-pandemic	Cytology (Pap)	60-64	64	Not performed	Satisfactory	NILM	NILM	Negative (NILM)	
P01272	2018	2018-Q3	2018-07	Pre-pandemic	Cytology (Pap)	30-34	30	Not performed	Satisfactory	NILM	NILM	Negative (NILM)	
P01273	2018	2018-Q3	2018-07	Pre-pandemic	Cytology (Pap)	40-44	41	Not performed	Satisfactory	NILM	NILM	Negative (NILM)	
P01274	2018	2018-Q3	2018-07	Pre-pandemic	Cytology (Pap)	45-49	45	Not performed	Satisfactory	NILM	NILM	Negative (NILM)	
P01275	2018	2018-Q3	2018-07	Pre-pandemic	Cytology (Pap)	55-59	55	Not performed	Satisfactory	NILM	NILM	Negative (NILM)	
P01276	2018	2018-Q3	2018-07	Pre-pandemic	Cytology (Pap)	30-34	31	Not performed	Satisfactory	NILM	NILM	Negative (NILM)	
P01277	2018	2018-Q3	2018-07	Pre-pandemic	Cytology (Pap)	30-34	34	Not performed	Satisfactory	NILM	NILM	Negative (NILM)	
P01278	2018	2018-Q3	2018-07	Pre-pandemic	Cytology (Pap)	Not recorded		Not performed	Satisfactory	NILM	NILM	Negative (NILM)	

patient_id	year	quarter	month	period	primary_test	age_group	age_years	hpv_result	adequacy	result	bethesda	grade	infection
P01279	2018	2018-Q3	2018-07	Pre-pandemic	Cytology (Pap)	25-29	26	Not performed	Satisfactory	NILM	NILM	Negative (NILM)	
P01280	2018	2018-Q3	2018-07	Pre-pandemic	Cytology (Pap)	60-64	64	Not performed	Satisfactory	NILM	NILM	Negative (NILM)	
P01281	2018	2018-Q3	2018-07	Pre-pandemic	Cytology (Pap)	50-54	51	Not performed	Satisfactory	NILM	NILM	Negative (NILM)	
P01282	2018	2018-Q3	2018-07	Pre-pandemic	Cytology (Pap)	40-44	44	Not performed	Satisfactory	NILM	NILM	Negative (NILM)	
P01283	2018	2018-Q3	2018-07	Pre-pandemic	Cytology (Pap)	55-59	59	Not performed	Satisfactory	NILM	NILM	Negative (NILM)	
P01284	2018	2018-Q3	2018-07	Pre-pandemic	Cytology (Pap)	25-29	28	Not performed	Satisfactory	Positive	ASC-US	Low-grade / equivocal squamous	
P01285	2018	2018-Q3	2018-07	Pre-pandemic	Cytology (Pap)	45-49	47	Not performed	Satisfactory	Positive	ASC-US	Low-grade / equivocal squamous	
P01286	2018	2018-Q3	2018-07	Pre-pandemic	Cytology (Pap)	45-49	45	Not performed	Satisfactory	NILM	NILM	Negative (NILM)	
P01287	2018	2018-Q3	2018-07	Pre-pandemic	Cytology (Pap)	30-34	32	Not performed	Satisfactory	NILM	NILM	Negative (NILM)	
P01288	2018	2018-Q3	2018-07	Pre-pandemic	Cytology (Pap)	30-34	33	Not performed	Satisfactory	NILM	NILM	Negative (NILM)	
P01289	2018	2018-Q3	2018-07	Pre-pandemic	Cytology (Pap)	35-39	36	Not performed	Satisfactory	NILM	NILM	Negative (NILM)	
P01290	2018	2018-Q3	2018-07	Pre-pandemic	Cytology (Pap)	55-59	57	Not performed	Satisfactory	NILM	NILM	Negative (NILM)	Candida
P01291	2018	2018-Q3	2018-07	Pre-pandemic	Cytology (Pap)	50-54	54	Not performed	Satisfactory	NILM	NILM	Negative (NILM)	
P01292	2018	2018-Q3	2018-07	Pre-pandemic	Cytology (Pap)	50-54	50	Not performed	Satisfactory	NILM	NILM	Negative (NILM)	
P01293	2018	2018-Q3	2018-07	Pre-pandemic	Cytology (Pap)	35-39	35	Not performed	Satisfactory	NILM	NILM	Negative (NILM)	
P01294	2018	2018-Q3	2018-07	Pre-pandemic	Cytology (Pap)	40-44	44	Not performed	Satisfactory	NILM	NILM	Negative (NILM)	
P01295	2018	2018-Q3	2018-07	Pre-pandemic	Cytology (Pap)	45-49	46	Not performed	Satisfactory	NILM	NILM	Negative (NILM)	
P01296	2018	2018-Q3	2018-07	Pre-pandemic	Cytology (Pap)	60-64	64	Not performed	Satisfactory	NILM	NILM	Negative (NILM)	
P01297	2018	2018-Q3	2018-07	Pre-pandemic	Cytology (Pap)	40-44	42	Not performed	Satisfactory	NILM	NILM	Negative (NILM)	
P01298	2018	2018-Q3	2018-07	Pre-pandemic	Cytology (Pap)	40-44	42	Not performed	Unsatisfactory	Unsatisfactory	Unsatisfactory	Unsatisfactory	
P01299	2018	2018-Q3	2018-07	Pre-pandemic	Cytology (Pap)	40-44	41	Not performed	Unsatisfactory	Unsatisfactory	Unsatisfactory	Unsatisfactory	
P01300	2018	2018-Q3	2018-07	Pre-pandemic	Cytology (Pap)	60-64	61	Not performed	Satisfactory	NILM	NILM	Negative (NILM)	
P01301	2018	2018-Q3	2018-07	Pre-pandemic	Cytology (Pap)	50-54	53	Not performed	Satisfactory	NILM	NILM	Negative (NILM)	
P01302	2018	2018-Q3	2018-07	Pre-pandemic	Cytology (Pap)	35-39	37	Not performed	Satisfactory	NILM	NILM	Negative (NILM)	
P01303	2018	2018-Q3	2018-07	Pre-pandemic	Cytology (Pap)	60-64	64	Not performed	Satisfactory	NILM	NILM	Negative (NILM)	
P01304	2018	2018-Q3	2018-07	Pre-pandemic	Cytology (Pap)	60-64	63	Not performed	Satisfactory	Positive	HSIL	High-grade squamous	
P01305	2018	2018-Q3	2018-07	Pre-pandemic	Cytology (Pap)	40-44	41	Not performed	Satisfactory	NILM	NILM	Negative (NILM)	
P01306	2018	2018-Q3	2018-07	Pre-pandemic	Cytology (Pap)	50-54	52	Not performed	Satisfactory	NILM	NILM	Negative (NILM)	
P01307	2018	2018-Q3	2018-07	Pre-pandemic	Cytology (Pap)	50-54	52	Not performed	Satisfactory	NILM	NILM	Negative (NILM)	
P01308	2018	2018-Q3	2018-07	Pre-pandemic	Cytology (Pap)	40-44	41	Not performed	Satisfactory	NILM	NILM	Negative (NILM)	
P01309	2018	2018-Q3	2018-07	Pre-pandemic	Cytology (Pap)	35-39	36	Not performed	Satisfactory	NILM	NILM	Negative (NILM)	
P01310	2018	2018-Q3	2018-07	Pre-pandemic	Cytology (Pap)	45-49	47	Not performed	Unsatisfactory	Unsatisfactory	Unsatisfactory	Unsatisfactory	
P01311	2018	2018-Q3	2018-07	Pre-pandemic	Cytology (Pap)	35-39	37	Not performed	Satisfactory	Positive	ASC-US	Low-grade / equivocal squamous	
P01312	2018	2018-Q3	2018-07	Pre-pandemic	Cytology (Pap)	40-44	41	Not performed	Satisfactory	NILM	NILM	Negative (NILM)	
P01313	2018	2018-Q3	2018-07	Pre-pandemic	Cytology (Pap)	55-59	59	Not performed	Satisfactory	NILM	NILM	Negative (NILM)	Candida
P01314	2018	2018-Q3	2018-07	Pre-pandemic	Cytology (Pap)	25-29	29	Not performed	Satisfactory	NILM	NILM	Negative (NILM)	
P01315	2018	2018-Q3	2018-07	Pre-pandemic	Cytology (Pap)	35-39	39	Not performed	Satisfactory	NILM	NILM	Negative (NILM)	
P01316	2018	2018-Q3	2018-07	Pre-pandemic	Cytology (Pap)	40-44	40	Not performed	Satisfactory	NILM	NILM	Negative (NILM)	Candida
P01317	2018	2018-Q3	2018-07	Pre-pandemic	Cytology (Pap)	30-34	33	Not performed	Satisfactory	Positive	LSIL	Low-grade / equivocal squamous	
P01318	2018	2018-Q3	2018-07	Pre-pandemic	Cytology (Pap)	50-54	54	Not performed	Satisfactory	NILM	NILM	Negative (NILM)	
P01319	2018	2018-Q3	2018-07	Pre-pandemic	Cytology (Pap)	55-59	56	Not performed	Satisfactory	NILM	NILM	Negative (NILM)	
P01320	2018	2018-Q3	2018-07	Pre-pandemic	Cytology (Pap)	55-59	58	Not performed	Satisfactory	NILM	NILM	Negative (NILM)	
P01321	2018	2018-Q3	2018-07	Pre-pandemic	Cytology (Pap)	60-64	62	Not performed	Satisfactory	NILM	NILM	Negative (NILM)	
P01322	2018	2018-Q3	2018-07	Pre-pandemic	Cytology (Pap)	35-39	37	Not performed	Satisfactory	Positive	ASC-US	Low-grade / equivocal squamous	
P01323	2018	2018-Q3	2018-07	Pre-pandemic	Cytology (Pap)	25-29	28	Not performed	Satisfactory	NILM	NILM	Negative (NILM)	
P01324	2018	2018-Q3	2018-07	Pre-pandemic	Cytology (Pap)	40-44	41	Not performed	Satisfactory	NILM	NILM	Negative (NILM)	
P01325	2018	2018-Q3	2018-07	Pre-pandemic	Cytology (Pap)	50-54	51	Not performed	Satisfactory	Positive	HSIL	High-grade squamous	
P01326	2018	2018-Q3	2018-07	Pre-pandemic	Cytology (Pap)	40-44	44	Not performed	Satisfactory	NILM	NILM	Negative (NILM)	
P01327	2018	2018-Q3	2018-07	Pre-pandemic	Cytology (Pap)	30-34	32	Not performed	Satisfactory	NILM	NILM	Negative (NILM)	
P01328	2018	2018-Q3	2018-07	Pre-pandemic	Cytology (Pap)	60-64	60	Not performed	Satisfactory	NILM	NILM	Negative (NILM)	
P01329	2018	2018-Q3	2018-07	Pre-pandemic	Cytology (Pap)	60-64	64	Not performed	Satisfactory	NILM	NILM	Negative (NILM)	
P01330	2018	2018-Q3	2018-07	Pre-pandemic	Cytology (Pap)	30-34	33	Not performed	Satisfactory	NILM	NILM	Negative (NILM)	
P01331	2018	2018-Q3	2018-07	Pre-pandemic	Cytology (Pap)	35-39	37	Not performed	Satisfactory	NILM	NILM	Negative (NILM)	
P01332	2018	2018-Q3	2018-07	Pre-pandemic	Cytology (Pap)	55-59	55	Not performed	Satisfactory	NILM	NILM	Negative (NILM)	
P01333	2018	2018-Q3	2018-07	Pre-pandemic	Cytology (Pap)	30-34	32	Not performed	Satisfactory	NILM	NILM	Negative (NILM)	
P01334	2018	2018-Q3	2018-07	Pre-pandemic	Cytology (Pap)	50-54	50	Not performed	Satisfactory	NILM	NILM	Negative (NILM)	
P01335	2018	2018-Q3	2018-07	Pre-pandemic	Cytology (Pap)	Not recorded		Not performed	Satisfactory	NILM	NILM	Negative (NILM)	
P01336	2018	2018-Q3	2018-07	Pre-pandemic	Cytology (Pap)	35-39	38	Not performed	Satisfactory	NILM	NILM	Negative (NILM)	
P01337	2018	2018-Q3	2018-07	Pre-pandemic	Cytology (Pap)	35-39	36	Not performed	Satisfactory	NILM	NILM	Negative (NILM)	
P01338	2018	2018-Q3	2018-07	Pre-pandemic	Cytology (Pap)	60-64	64	Not performed	Satisfactory	NILM	NILM	Negative (NILM)	
P01339	2018	2018-Q3	2018-07	Pre-pandemic	Cytology (Pap)	35-39	39	Not performed	Satisfactory	NILM	NILM	Negative (NILM)	
P01340	2018	2018-Q3	2018-07	Pre-pandemic	Cytology (Pap)	60-64	62	Not performed	Satisfactory	NILM	NILM	Negative (NILM)	
P01341	2018	2018-Q3	2018-07	Pre-pandemic	Cytology (Pap)	35-39	39	Not performed	Satisfactory	NILM	NILM	Negative (NILM)	
P01342	2018	2018-Q3	2018-07	Pre-pandemic	Cytology (Pap)	50-54	50	Not performed	Satisfactory	NILM	NILM	Negative (NILM)	
P01343	2018	2018-Q3	2018-07	Pre-pandemic	Cytology (Pap)	40-44	41	Not performed	Satisfactory	NILM	NILM	Negative (NILM)	
P01344	2018	2018-Q3	2018-07	Pre-pandemic	Cytology (Pap)	45-49	48	Not performed	Satisfactory	NILM	NILM	Negative (NILM)	
P01345	2018	2018-Q3	2018-07	Pre-pandemic	Cytology (Pap)	Not recorded		Not performed	Satisfactory	NILM	NILM	Negative (NILM)	
P01346	2018	2018-Q3	2018-07	Pre-pandemic	Cytology (Pap)	35-39	39	Not performed	Satisfactory	NILM	NILM	Negative (NILM)	
P01347	2018	2018-Q3	2018-07	Pre-pandemic	Cytology (Pap)	60-64	64	Not performed	Satisfactory	NILM	NILM	Negative (NILM)	
P01348	2018	2018-Q3	2018-07	Pre-pandemic	Cytology (Pap)	40-44	44	Not performed	Satisfactory	NILM	NILM	Negative (NILM)	
P01349	2018	2018-Q3	2018-07	Pre-pandemic	Cytology (Pap)	45-49	46	Not performed	Satisfactory	NILM	NILM	Negative (NILM)	
P01350	2018	2018-Q3	2018-07	Pre-pandemic	Cytology (Pap)	50-54	54	Not performed	Satisfactory	Positive	ASC-US	Low-grade / equivocal squamous	
P01351	2018	2018-Q3	2018-07	Pre-pandemic	Cytology (Pap)	35-39	37	Not performed	Satisfactory	NILM	NILM	Negative (NILM)	
P01352	2018	2018-Q3	2018-07	Pre-pandemic	Cytology (Pap)	45-49	46	Not performed	Satisfactory	NILM	NILM	Negative (NILM)	Candida
P01353	2018	2018-Q3	2018-07	Pre-pandemic	Cytology (Pap)	30-34	31	Not performed	Satisfactory	NILM	NILM	Negative (NILM)	
P01354	2018	2018-Q3	2018-07	Pre-pandemic	Cytology (Pap)	55-59	55	Not performed	Satisfactory	NILM	NILM	Negative (NILM)	
P01355	2018	2018-Q3	2018-07	Pre-pandemic	Cytology (Pap)	45-49	49	Not performed	Satisfactory	NILM	NILM	Negative (NILM)	
P01356	2018	2018-Q3	2018-07	Pre-pandemic	Cytology (Pap)	40-44	40	Not performed	Satisfactory	NILM	NILM	Negative (NILM)	
P01357	2018	2018-Q3	2018-07	Pre-pandemic	Cytology (Pap)	25-29	28	Not performed	Satisfactory	NILM	NILM	Negative (NILM)	
P01358	2018	2018-Q3	2018-07	Pre-pandemic	Cytology (Pap)	50-54	50	Not performed	Satisfactory	NILM	NILM	Negative (NILM)	

patient_id	year	quarter	month	period	primary_test	age_group	age_years	hpv_result	adequacy	result	bethesda	grade	infection
P01359	2018	2018-Q3	2018-07	Pre-pandemic	Cytology (Pap)	Not recorded		Not performed	Satisfactory	NILM	NILM	Negative (NILM)	
P01360	2018	2018-Q3	2018-07	Pre-pandemic	Cytology (Pap)	55-59	59	Not performed	Unsatisfactory	Unsatisfactory	Unsatisfactory	Unsatisfactory	
P01361	2018	2018-Q3	2018-07	Pre-pandemic	Cytology (Pap)	30-34	33	Not performed	Satisfactory	NILM	NILM	Negative (NILM)	Candida
P01362	2018	2018-Q3	2018-07	Pre-pandemic	Cytology (Pap)	40-44	42	Not performed	Satisfactory	NILM	NILM	Negative (NILM)	
P01363	2018	2018-Q3	2018-07	Pre-pandemic	Cytology (Pap)	60-64	64	Not performed	Satisfactory	NILM	NILM	Negative (NILM)	
P01364	2018	2018-Q3	2018-07	Pre-pandemic	Cytology (Pap)	60-64	63	Not performed	Satisfactory	NILM	NILM	Negative (NILM)	
P01365	2018	2018-Q3	2018-07	Pre-pandemic	Cytology (Pap)	40-44	44	Not performed	Satisfactory	NILM	NILM	Negative (NILM)	
P01366	2018	2018-Q3	2018-07	Pre-pandemic	Cytology (Pap)	35-39	39	Not performed	Satisfactory	NILM	NILM	Negative (NILM)	
P01367	2018	2018-Q3	2018-07	Pre-pandemic	Cytology (Pap)	45-49	48	Not performed	Satisfactory	NILM	NILM	Negative (NILM)	
P01368	2018	2018-Q3	2018-07	Pre-pandemic	Cytology (Pap)	35-39	35	Not performed	Satisfactory	Positive	HSIL	High-grade squamous	Candida
P01369	2018	2018-Q3	2018-07	Pre-pandemic	Cytology (Pap)	40-44	44	Not performed	Satisfactory	NILM	NILM	Negative (NILM)	
P01370	2018	2018-Q3	2018-07	Pre-pandemic	Cytology (Pap)	40-44	40	Not performed	Satisfactory	NILM	NILM	Negative (NILM)	
P01371	2018	2018-Q3	2018-07	Pre-pandemic	Cytology (Pap)	Not recorded		Not performed	Satisfactory	NILM	NILM	Negative (NILM)	
P01372	2018	2018-Q3	2018-07	Pre-pandemic	Cytology (Pap)	35-39	38	Not performed	Unsatisfactory	Unsatisfactory	Unsatisfactory	Unsatisfactory	
P01373	2018	2018-Q3	2018-07	Pre-pandemic	Cytology (Pap)	30-34	34	Not performed	Satisfactory	NILM	NILM	Negative (NILM)	
P01374	2018	2018-Q3	2018-07	Pre-pandemic	Cytology (Pap)	40-44	44	Not performed	Unsatisfactory	Unsatisfactory	Unsatisfactory	Unsatisfactory	
P01375	2018	2018-Q3	2018-07	Pre-pandemic	Cytology (Pap)	40-44	44	Not performed	Satisfactory	NILM	NILM	Negative (NILM)	
P01376	2018	2018-Q3	2018-07	Pre-pandemic	Cytology (Pap)	40-44	42	Not performed	Satisfactory	NILM	NILM	Negative (NILM)	
P01377	2018	2018-Q3	2018-07	Pre-pandemic	Cytology (Pap)	40-44	41	Not performed	Satisfactory	NILM	NILM	Negative (NILM)	
P01378	2018	2018-Q3	2018-07	Pre-pandemic	Cytology (Pap)	30-34	32	Not performed	Satisfactory	Positive	ASC-US	Low-grade / equivocal squamous	Candida
P01379	2018	2018-Q3	2018-07	Pre-pandemic	Cytology (Pap)	40-44	42	Not performed	Satisfactory	Positive	LSIL	Low-grade / equivocal squamous	
P01380	2018	2018-Q3	2018-07	Pre-pandemic	Cytology (Pap)	55-59	55	Not performed	Satisfactory	NILM	NILM	Negative (NILM)	
P01381	2018	2018-Q3	2018-07	Pre-pandemic	Cytology (Pap)	30-34	30	Not performed	Satisfactory	NILM	NILM	Negative (NILM)	
P01382	2018	2018-Q3	2018-07	Pre-pandemic	Cytology (Pap)	55-59	55	Not performed	Satisfactory	NILM	NILM	Negative (NILM)	
P01383	2018	2018-Q3	2018-07	Pre-pandemic	Cytology (Pap)	35-39	36	Not performed	Satisfactory	NILM	NILM	Negative (NILM)	Gardnerella
P01384	2018	2018-Q3	2018-07	Pre-pandemic	Cytology (Pap)	35-39	36	Not performed	Satisfactory	NILM	NILM	Negative (NILM)	
P01385	2018	2018-Q3	2018-07	Pre-pandemic	Cytology (Pap)	40-44	42	Not performed	Satisfactory	NILM	NILM	Negative (NILM)	
P01386	2018	2018-Q3	2018-07	Pre-pandemic	Cytology (Pap)	25-29	29	Not performed	Satisfactory	NILM	NILM	Negative (NILM)	
P01387	2018	2018-Q3	2018-07	Pre-pandemic	Cytology (Pap)	40-44	41	Not performed	Satisfactory	NILM	NILM	Negative (NILM)	
P01388	2018	2018-Q3	2018-07	Pre-pandemic	Cytology (Pap)	40-44	41	Not performed	Satisfactory	NILM	NILM	Negative (NILM)	
P01389	2018	2018-Q3	2018-07	Pre-pandemic	Cytology (Pap)	55-59	59	Not performed	Satisfactory	NILM	NILM	Negative (NILM)	
P01390	2018	2018-Q3	2018-07	Pre-pandemic	Cytology (Pap)	30-34	33	Not performed	Satisfactory	NILM	NILM	Negative (NILM)	
P01391	2018	2018-Q3	2018-07	Pre-pandemic	Cytology (Pap)	40-44	40	Not performed	Satisfactory	NILM	NILM	Negative (NILM)	
P01392	2018	2018-Q3	2018-07	Pre-pandemic	Cytology (Pap)	50-54	52	Not performed	Satisfactory	NILM	NILM	Negative (NILM)	
P01393	2018	2018-Q3	2018-07	Pre-pandemic	Cytology (Pap)	55-59	56	Not performed	Satisfactory	NILM	NILM	Negative (NILM)	
P01394	2018	2018-Q3	2018-07	Pre-pandemic	Cytology (Pap)	55-59	58	Not performed	Satisfactory	NILM	NILM	Negative (NILM)	
P01395	2018	2018-Q3	2018-07	Pre-pandemic	Cytology (Pap)	55-59	58	Not performed	Satisfactory	Positive	ASC-H	High-grade squamous	
P01396	2018	2018-Q3	2018-07	Pre-pandemic	Cytology (Pap)	Not recorded		Not performed	Satisfactory	NILM	NILM	Negative (NILM)	
P01397	2018	2018-Q3	2018-07	Pre-pandemic	Cytology (Pap)	45-49	47	Not performed	Satisfactory	NILM	NILM	Negative (NILM)	
P01398	2018	2018-Q3	2018-07	Pre-pandemic	Cytology (Pap)	30-34	30	Not performed	Satisfactory	NILM	NILM	Negative (NILM)	
P01399	2018	2018-Q3	2018-07	Pre-pandemic	Cytology (Pap)	40-44	41	Not performed	Satisfactory	Positive	LSIL	Low-grade / equivocal squamous	
P01400	2018	2018-Q3	2018-07	Pre-pandemic	Cytology (Pap)	50-54	51	Not performed	Satisfactory	NILM	NILM	Negative (NILM)	
P01401	2018	2018-Q3	2018-07	Pre-pandemic	Cytology (Pap)	55-59	59	Not performed	Satisfactory	NILM	NILM	Negative (NILM)	
P01402	2018	2018-Q3	2018-07	Pre-pandemic	Cytology (Pap)	60-64	60	Not performed	Satisfactory	Positive	HSIL	High-grade squamous	
P01403	2018	2018-Q3	2018-07	Pre-pandemic	Cytology (Pap)	Not recorded		Not performed	Satisfactory	NILM	NILM	Negative (NILM)	
P01404	2018	2018-Q3	2018-07	Pre-pandemic	Cytology (Pap)	40-44	40	Not performed	Satisfactory	NILM	NILM	Negative (NILM)	
P01405	2018	2018-Q3	2018-07	Pre-pandemic	Cytology (Pap)	35-39	38	Not performed	Satisfactory	NILM	NILM	Negative (NILM)	
P01406	2018	2018-Q3	2018-07	Pre-pandemic	Cytology (Pap)	40-44	41	Not performed	Satisfactory	NILM	NILM	Negative (NILM)	
P01407	2018	2018-Q3	2018-07	Pre-pandemic	Cytology (Pap)	45-49	49	Not performed	Satisfactory	NILM	NILM	Negative (NILM)	
P01408	2018	2018-Q3	2018-07	Pre-pandemic	Cytology (Pap)	25-29	28	Not performed	Satisfactory	NILM	NILM	Negative (NILM)	
P01409	2018	2018-Q3	2018-07	Pre-pandemic	Cytology (Pap)	40-44	44	Not performed	Satisfactory	NILM	NILM	Negative (NILM)	
P01410	2018	2018-Q3	2018-07	Pre-pandemic	Cytology (Pap)	40-44	41	Not performed	Satisfactory	NILM	NILM	Negative (NILM)	
P01411	2018	2018-Q3	2018-07	Pre-pandemic	Cytology (Pap)	40-44	41	Not performed	Satisfactory	NILM	NILM	Negative (NILM)	
P01412	2018	2018-Q3	2018-07	Pre-pandemic	Cytology (Pap)	55-59	59	Not performed	Satisfactory	NILM	NILM	Negative (NILM)	
P01413	2018	2018-Q3	2018-07	Pre-pandemic	Cytology (Pap)	40-44	43	Not performed	Satisfactory	NILM	NILM	Negative (NILM)	
P01414	2018	2018-Q3	2018-07	Pre-pandemic	Cytology (Pap)	40-44	42	Not performed	Satisfactory	NILM	NILM	Negative (NILM)	
P01415	2018	2018-Q3	2018-07	Pre-pandemic	Cytology (Pap)	40-44	40	Not performed	Satisfactory	NILM	NILM	Negative (NILM)	
P01416	2018	2018-Q3	2018-07	Pre-pandemic	Cytology (Pap)	30-34	30	Not performed	Satisfactory	Positive	LSIL	Low-grade / equivocal squamous	
P01417	2018	2018-Q3	2018-07	Pre-pandemic	Cytology (Pap)	40-44	40	Not performed	Satisfactory	NILM	NILM	Negative (NILM)	
P01418	2018	2018-Q3	2018-07	Pre-pandemic	Cytology (Pap)	30-34	32	Not performed	Satisfactory	NILM	NILM	Negative (NILM)	
P01419	2018	2018-Q3	2018-07	Pre-pandemic	Cytology (Pap)	35-39	38	Not performed	Satisfactory	NILM	NILM	Negative (NILM)	Gardnerella
P01420	2018	2018-Q3	2018-07	Pre-pandemic	Cytology (Pap)	45-49	46	Not performed	Satisfactory	NILM	NILM	Negative (NILM)	
P01421	2018	2018-Q3	2018-07	Pre-pandemic	Cytology (Pap)	40-44	41	Not performed	Satisfactory	NILM	NILM	Negative (NILM)	
P01422	2018	2018-Q3	2018-07	Pre-pandemic	Cytology (Pap)	40-44	44	Not performed	Satisfactory	Positive	ASC-US	Low-grade / equivocal squamous	Gardnerella
P01423	2018	2018-Q3	2018-07	Pre-pandemic	Cytology (Pap)	40-44	40	Not performed	Satisfactory	NILM	NILM	Negative (NILM)	
P01424	2018	2018-Q3	2018-07	Pre-pandemic	Cytology (Pap)	45-49	45	Not performed	Satisfactory	Positive	ASC-US	Low-grade / equivocal squamous	
P01425	2018	2018-Q3	2018-07	Pre-pandemic	Cytology (Pap)	40-44	43	Not performed	Satisfactory	NILM	NILM	Negative (NILM)	
P01426	2018	2018-Q3	2018-07	Pre-pandemic	Cytology (Pap)	40-44	41	Not performed	Satisfactory	NILM	NILM	Negative (NILM)	
P01427	2018	2018-Q3	2018-07	Pre-pandemic	Cytology (Pap)	30-34	34	Not performed	Satisfactory	NILM	NILM	Negative (NILM)	
P01428	2018	2018-Q3	2018-07	Pre-pandemic	Cytology (Pap)	50-54	52	Not performed	Satisfactory	Positive	ASC-H	High-grade squamous	
P01429	2018	2018-Q3	2018-07	Pre-pandemic	Cytology (Pap)	30-34	33	Not performed	Satisfactory	NILM	NILM	Negative (NILM)	
P01430	2018	2018-Q3	2018-07	Pre-pandemic	Cytology (Pap)	30-34	31	Not performed	Satisfactory	NILM	NILM	Negative (NILM)	
P01431	2018	2018-Q3	2018-07	Pre-pandemic	Cytology (Pap)	35-39	39	Not performed	Satisfactory	NILM	NILM	Negative (NILM)	
P01432	2018	2018-Q3	2018-07	Pre-pandemic	Cytology (Pap)	45-49	47	Not performed	Satisfactory	NILM	NILM	Negative (NILM)	Candida
P01433	2018	2018-Q3	2018-07	Pre-pandemic	Cytology (Pap)	35-39	39	Not performed	Satisfactory	NILM	NILM	Negative (NILM)	
P01434	2018	2018-Q3	2018-07	Pre-pandemic	Cytology (Pap)	50-54	53	Not performed	Satisfactory	NILM	NILM	Negative (NILM)	
P01435	2018	2018-Q3	2018-07	Pre-pandemic	Cytology (Pap)	40-44	44	Not performed	Satisfactory	Positive	LSIL	Low-grade / equivocal squamous	
P01436	2018	2018-Q3	2018-07	Pre-pandemic	Cytology (Pap)	55-59	59	Not performed	Satisfactory	NILM	NILM	Negative (NILM)	
P01437	2018	2018-Q3	2018-07	Pre-pandemic	Cytology (Pap)	50-54	52	Not performed	Satisfactory	Positive	ASC-US	Low-grade / equivocal squamous	
P01438	2018	2018-Q3	2018-07	Pre-pandemic	Cytology (Pap)	60-64	61	Not performed	Satisfactory	NILM	NILM	Negative (NILM)	

patient_id	year	quarter	month	period	primary_test	age_group	age_years	hpv_result	adequacy	result	bethesda	grade	infection
P01439	2018	2018-Q3	2018-07	Pre-pandemic	Cytology (Pap)	50-54	53	Not performed	Satisfactory	NILM	NILM	Negative (NILM)	Gardnerella
P01440	2018	2018-Q3	2018-07	Pre-pandemic	Cytology (Pap)	60-64	61	Not performed	Satisfactory	Positive	ASC-US	Low-grade / equivocal squamous	
P01441	2018	2018-Q3	2018-07	Pre-pandemic	Cytology (Pap)	Not recorded		Not performed	Satisfactory	NILM	NILM	Negative (NILM)	
P01442	2018	2018-Q3	2018-07	Pre-pandemic	Cytology (Pap)	55-59	56	Not performed	Satisfactory	NILM	NILM	Negative (NILM)	
P01443	2018	2018-Q3	2018-07	Pre-pandemic	Cytology (Pap)	30-34	31	Not performed	Satisfactory	NILM	NILM	Negative (NILM)	
P01444	2018	2018-Q3	2018-07	Pre-pandemic	Cytology (Pap)	45-49	48	Not performed	Satisfactory	NILM	NILM	Negative (NILM)	
P01445	2018	2018-Q3	2018-07	Pre-pandemic	Cytology (Pap)	50-54	53	Not performed	Satisfactory	Positive	ASC-US	Low-grade / equivocal squamous	
P01446	2018	2018-Q3	2018-07	Pre-pandemic	Cytology (Pap)	55-59	58	Not performed	Satisfactory	Positive	ASC-US	Low-grade / equivocal squamous	
P01447	2018	2018-Q3	2018-07	Pre-pandemic	Cytology (Pap)	30-34	32	Not performed	Satisfactory	NILM	NILM	Negative (NILM)	
P01448	2018	2018-Q3	2018-07	Pre-pandemic	Cytology (Pap)	55-59	57	Not performed	Unsatisfactory	Unsatisfactory	Unsatisfactory	Unsatisfactory	
P01449	2018	2018-Q3	2018-07	Pre-pandemic	Cytology (Pap)	40-44	43	Not performed	Satisfactory	NILM	NILM	Negative (NILM)	
P01450	2018	2018-Q3	2018-07	Pre-pandemic	Cytology (Pap)	50-54	52	Not performed	Satisfactory	NILM	NILM	Negative (NILM)	
P01451	2018	2018-Q3	2018-07	Pre-pandemic	Cytology (Pap)	60-64	60	Not performed	Satisfactory	Positive	ASC-H	High-grade squamous	
P01452	2018	2018-Q3	2018-07	Pre-pandemic	Cytology (Pap)	60-64	64	Not performed	Satisfactory	NILM	NILM	Negative (NILM)	
P01453	2018	2018-Q3	2018-07	Pre-pandemic	Cytology (Pap)	50-54	54	Not performed	Satisfactory	NILM	NILM	Negative (NILM)	
P01454	2018	2018-Q3	2018-07	Pre-pandemic	Cytology (Pap)	Not recorded		Not performed	Satisfactory	NILM	NILM	Negative (NILM)	
P01455	2018	2018-Q3	2018-07	Pre-pandemic	Cytology (Pap)	45-49	45	Not performed	Satisfactory	Positive	ASC-US	Low-grade / equivocal squamous	
P01456	2018	2018-Q3	2018-07	Pre-pandemic	Cytology (Pap)	35-39	36	Not performed	Satisfactory	NILM	NILM	Negative (NILM)	
P01457	2018	2018-Q3	2018-07	Pre-pandemic	Cytology (Pap)	35-39	38	Not performed	Satisfactory	NILM	NILM	Negative (NILM)	
P01458	2018	2018-Q3	2018-07	Pre-pandemic	Cytology (Pap)	55-59	59	Not performed	Satisfactory	NILM	NILM	Negative (NILM)	
P01459	2018	2018-Q3	2018-07	Pre-pandemic	Cytology (Pap)	Not recorded		Not performed	Satisfactory	NILM	NILM	Negative (NILM)	
P01460	2018	2018-Q3	2018-07	Pre-pandemic	Cytology (Pap)	45-49	49	Not performed	Satisfactory	NILM	NILM	Negative (NILM)	
P01461	2018	2018-Q3	2018-07	Pre-pandemic	Cytology (Pap)	55-59	55	Not performed	Satisfactory	NILM	NILM	Negative (NILM)	
P01462	2018	2018-Q3	2018-07	Pre-pandemic	Cytology (Pap)	40-44	43	Not performed	Satisfactory	NILM	NILM	Negative (NILM)	Other
P01463	2018	2018-Q3	2018-07	Pre-pandemic	Cytology (Pap)	50-54	53	Not performed	Satisfactory	NILM	NILM	Negative (NILM)	
P01464	2018	2018-Q3	2018-07	Pre-pandemic	Cytology (Pap)	45-49	49	Not performed	Satisfactory	NILM	NILM	Negative (NILM)	Gardnerella
P01465	2018	2018-Q3	2018-07	Pre-pandemic	Cytology (Pap)	50-54	50	Not performed	Unsatisfactory	Unsatisfactory	Unsatisfactory	Unsatisfactory	
P01466	2018	2018-Q3	2018-07	Pre-pandemic	Cytology (Pap)	50-54	51	Not performed	Satisfactory	NILM	NILM	Negative (NILM)	
P01467	2018	2018-Q3	2018-07	Pre-pandemic	Cytology (Pap)	45-49	45	Not performed	Satisfactory	NILM	NILM	Negative (NILM)	
P01468	2018	2018-Q3	2018-07	Pre-pandemic	Cytology (Pap)	50-54	53	Not performed	Satisfactory	NILM	NILM	Negative (NILM)	
P01469	2018	2018-Q3	2018-07	Pre-pandemic	Cytology (Pap)	50-54	54	Not performed	Satisfactory	NILM	NILM	Negative (NILM)	
P01470	2018	2018-Q3	2018-07	Pre-pandemic	Cytology (Pap)	60-64	63	Not performed	Satisfactory	Positive	ASC-US	Low-grade / equivocal squamous	
P01471	2018	2018-Q3	2018-07	Pre-pandemic	Cytology (Pap)	40-44	43	Not performed	Satisfactory	Positive	LSIL	Low-grade / equivocal squamous	
P01472	2018	2018-Q3	2018-07	Pre-pandemic	Cytology (Pap)	40-44	40	Not performed	Satisfactory	NILM	NILM	Negative (NILM)	
P01473	2018	2018-Q3	2018-07	Pre-pandemic	Cytology (Pap)	30-34	31	Not performed	Satisfactory	NILM	NILM	Negative (NILM)	
P01474	2018	2018-Q3	2018-07	Pre-pandemic	Cytology (Pap)	30-34	31	Not performed	Satisfactory	NILM	NILM	Negative (NILM)	
P01475	2018	2018-Q3	2018-07	Pre-pandemic	Cytology (Pap)	40-44	40	Not performed	Satisfactory	Positive	ASC-H	High-grade squamous	
P01476	2018	2018-Q3	2018-07	Pre-pandemic	Cytology (Pap)	35-39	36	Not performed	Satisfactory	NILM	NILM	Negative (NILM)	
P01477	2018	2018-Q3	2018-07	Pre-pandemic	Cytology (Pap)	25-29	29	Not performed	Satisfactory	NILM	NILM	Negative (NILM)	
P01478	2018	2018-Q3	2018-07	Pre-pandemic	Cytology (Pap)	Not recorded		Not performed	Satisfactory	NILM	NILM	Negative (NILM)	
P01479	2018	2018-Q3	2018-07	Pre-pandemic	Cytology (Pap)	30-34	33	Not performed	Satisfactory	NILM	NILM	Negative (NILM)	
P01480	2018	2018-Q3	2018-07	Pre-pandemic	Cytology (Pap)	40-44	41	Not performed	Satisfactory	NILM	NILM	Negative (NILM)	
P01481	2018	2018-Q3	2018-07	Pre-pandemic	Cytology (Pap)	40-44	41	Not performed	Satisfactory	NILM	NILM	Negative (NILM)	
P01482	2018	2018-Q3	2018-07	Pre-pandemic	Cytology (Pap)	60-64	61	Not performed	Satisfactory	NILM	NILM	Negative (NILM)	
P01483	2018	2018-Q3	2018-07	Pre-pandemic	Cytology (Pap)	40-44	42	Not performed	Satisfactory	NILM	NILM	Negative (NILM)	
P01484	2018	2018-Q3	2018-07	Pre-pandemic	Cytology (Pap)	30-34	33	Not performed	Satisfactory	NILM	NILM	Negative (NILM)	
P01485	2018	2018-Q3	2018-07	Pre-pandemic	Cytology (Pap)	40-44	42	Not performed	Satisfactory	NILM	NILM	Negative (NILM)	
P01486	2018	2018-Q3	2018-07	Pre-pandemic	Cytology (Pap)	50-54	50	Not performed	Satisfactory	NILM	NILM	Negative (NILM)	
P01487	2018	2018-Q3	2018-07	Pre-pandemic	Cytology (Pap)	40-44	44	Not performed	Satisfactory	NILM	NILM	Negative (NILM)	
P01488	2018	2018-Q3	2018-07	Pre-pandemic	Cytology (Pap)	50-54	54	Not performed	Satisfactory	NILM	NILM	Negative (NILM)	
P01489	2018	2018-Q3	2018-07	Pre-pandemic	Cytology (Pap)	45-49	48	Not performed	Satisfactory	NILM	NILM	Negative (NILM)	
P01490	2018	2018-Q3	2018-07	Pre-pandemic	Cytology (Pap)	60-64	60	Not performed	Satisfactory	NILM	NILM	Negative (NILM)	
P01491	2018	2018-Q3	2018-07	Pre-pandemic	Cytology (Pap)	30-34	31	Not performed	Satisfactory	NILM	NILM	Negative (NILM)	
P01492	2018	2018-Q3	2018-07	Pre-pandemic	Cytology (Pap)	45-49	48	Not performed	Satisfactory	NILM	NILM	Negative (NILM)	
P01493	2018	2018-Q3	2018-07	Pre-pandemic	Cytology (Pap)	45-49	49	Not performed	Unsatisfactory	Unsatisfactory	Unsatisfactory	Unsatisfactory	
P01494	2018	2018-Q3	2018-07	Pre-pandemic	Cytology (Pap)	35-39	38	Not performed	Unsatisfactory	Unsatisfactory	Unsatisfactory	Unsatisfactory	
P01495	2018	2018-Q3	2018-07	Pre-pandemic	Cytology (Pap)	40-44	41	Not performed	Satisfactory	Positive	HSIL	High-grade squamous	
P01496	2018	2018-Q3	2018-07	Pre-pandemic	Cytology (Pap)	30-34	30	Not performed	Satisfactory	NILM	NILM	Negative (NILM)	
P01497	2018	2018-Q3	2018-07	Pre-pandemic	Cytology (Pap)	40-44	42	Not performed	Satisfactory	Positive	ASC-US	Low-grade / equivocal squamous	
P01498	2018	2018-Q3	2018-07	Pre-pandemic	Cytology (Pap)	45-49	49	Not performed	Satisfactory	NILM	NILM	Negative (NILM)	
P01499	2018	2018-Q3	2018-07	Pre-pandemic	Cytology (Pap)	50-54	52	Not performed	Satisfactory	Positive	ASC-US	Low-grade / equivocal squamous	
P01500	2018	2018-Q3	2018-07	Pre-pandemic	Cytology (Pap)	30-34	31	Not performed	Satisfactory	NILM	NILM	Negative (NILM)	
P01501	2018	2018-Q3	2018-08	Pre-pandemic	Cytology (Pap)	50-54	52	Not performed	Satisfactory	NILM	NILM	Negative (NILM)	
P01502	2018	2018-Q3	2018-08	Pre-pandemic	Cytology (Pap)	50-54	52	Not performed	Satisfactory	NILM	NILM	Negative (NILM)	
P01503	2018	2018-Q3	2018-08	Pre-pandemic	Cytology (Pap)	50-54	50	Not performed	Satisfactory	NILM	NILM	Negative (NILM)	
P01504	2018	2018-Q3	2018-08	Pre-pandemic	Cytology (Pap)	50-54	51	Not performed	Satisfactory	NILM	NILM	Negative (NILM)	
P01505	2018	2018-Q3	2018-08	Pre-pandemic	Cytology (Pap)	45-49	48	Not performed	Satisfactory	NILM	NILM	Negative (NILM)	
P01506	2018	2018-Q3	2018-08	Pre-pandemic	Cytology (Pap)	35-39	37	Not performed	Satisfactory	NILM	NILM	Negative (NILM)	
P01507	2018	2018-Q3	2018-08	Pre-pandemic	Cytology (Pap)	35-39	38	Not performed	Satisfactory	NILM	NILM	Negative (NILM)	
P01508	2018	2018-Q3	2018-08	Pre-pandemic	Cytology (Pap)	55-59	57	Not performed	Satisfactory	NILM	NILM	Negative (NILM)	
P01509	2018	2018-Q3	2018-08	Pre-pandemic	Cytology (Pap)	40-44	44	Not performed	Satisfactory	NILM	NILM	Negative (NILM)	
P01510	2018	2018-Q3	2018-08	Pre-pandemic	Cytology (Pap)	45-49	48	Not performed	Satisfactory	NILM	NILM	Negative (NILM)	
P01511	2018	2018-Q3	2018-08	Pre-pandemic	Cytology (Pap)	45-49	46	Not performed	Satisfactory	NILM	NILM	Negative (NILM)	
P01512	2018	2018-Q3	2018-08	Pre-pandemic	Cytology (Pap)	60-64	60	Not performed	Satisfactory	NILM	NILM	Negative (NILM)	
P01513	2018	2018-Q3	2018-08	Pre-pandemic	Cytology (Pap)	45-49	45	Not performed	Satisfactory	NILM	NILM	Negative (NILM)	
P01514	2018	2018-Q3	2018-08	Pre-pandemic	Cytology (Pap)	55-59	59	Not performed	Satisfactory	NILM	NILM	Negative (NILM)	
P01515	2018	2018-Q3	2018-08	Pre-pandemic	Cytology (Pap)	Not recorded		Not performed	Satisfactory	NILM	NILM	Negative (NILM)	
P01516	2018	2018-Q3	2018-08	Pre-pandemic	Cytology (Pap)	60-64	64	Not performed	Satisfactory	NILM	NILM	Negative (NILM)	
P01517	2018	2018-Q3	2018-08	Pre-pandemic	Cytology (Pap)	30-34	30	Not performed	Satisfactory	NILM	NILM	Negative (NILM)	
P01518	2018	2018-Q3	2018-08	Pre-pandemic	Cytology (Pap)	35-39	39	Not performed	Satisfactory	NILM	NILM	Negative (NILM)	

patient_id	year	quarter	month	period	primary_test	age_group	age_years	hpv_result	adequacy	result	bethesda	grade	infection
P01519	2018	2018-Q3	2018-08	Pre-pandemic	Cytology (Pap)	40-44	42	Not performed	Satisfactory	NILM	NILM	Negative (NILM)	
P01520	2018	2018-Q3	2018-08	Pre-pandemic	Cytology (Pap)	55-59	59	Not performed	Satisfactory	NILM	NILM	Negative (NILM)	
P01521	2018	2018-Q3	2018-08	Pre-pandemic	Cytology (Pap)	35-39	37	Not performed	Satisfactory	NILM	NILM	Negative (NILM)	
P01522	2018	2018-Q3	2018-08	Pre-pandemic	Cytology (Pap)	45-49	48	Not performed	Satisfactory	Positive	LSIL	Low-grade / equivocal squamous	
P01523	2018	2018-Q3	2018-08	Pre-pandemic	Cytology (Pap)	35-39	35	Not performed	Satisfactory	NILM	NILM	Negative (NILM)	
P01524	2018	2018-Q3	2018-08	Pre-pandemic	Cytology (Pap)	60-64	60	Not performed	Satisfactory	NILM	NILM	Negative (NILM)	
P01525	2018	2018-Q3	2018-08	Pre-pandemic	Cytology (Pap)	30-34	33	Not performed	Satisfactory	Positive	ASC-US	Low-grade / equivocal squamous	
P01526	2018	2018-Q3	2018-08	Pre-pandemic	Cytology (Pap)	35-39	36	Not performed	Satisfactory	NILM	NILM	Negative (NILM)	
P01527	2018	2018-Q3	2018-08	Pre-pandemic	Cytology (Pap)	30-34	31	Not performed	Satisfactory	NILM	NILM	Negative (NILM)	
P01528	2018	2018-Q3	2018-08	Pre-pandemic	Cytology (Pap)	35-39	37	Not performed	Satisfactory	NILM	NILM	Negative (NILM)	
P01529	2018	2018-Q3	2018-08	Pre-pandemic	Cytology (Pap)	40-44	43	Not performed	Satisfactory	NILM	NILM	Negative (NILM)	Trichomonas
P01530	2018	2018-Q3	2018-08	Pre-pandemic	Cytology (Pap)	40-44	42	Not performed	Satisfactory	NILM	NILM	Negative (NILM)	
P01531	2018	2018-Q3	2018-08	Pre-pandemic	Cytology (Pap)	40-44	40	Not performed	Satisfactory	NILM	NILM	Negative (NILM)	
P01532	2018	2018-Q3	2018-08	Pre-pandemic	Cytology (Pap)	35-39	36	Not performed	Satisfactory	NILM	NILM	Negative (NILM)	
P01533	2018	2018-Q3	2018-08	Pre-pandemic	Cytology (Pap)	60-64	62	Not performed	Satisfactory	NILM	NILM	Negative (NILM)	
P01534	2018	2018-Q3	2018-08	Pre-pandemic	Cytology (Pap)	25-29	29	Not performed	Satisfactory	NILM	NILM	Negative (NILM)	
P01535	2018	2018-Q3	2018-08	Pre-pandemic	Cytology (Pap)	60-64	63	Not performed	Satisfactory	NILM	NILM	Negative (NILM)	
P01536	2018	2018-Q3	2018-08	Pre-pandemic	Cytology (Pap)	35-39	39	Not performed	Satisfactory	Positive	ASC-US	Low-grade / equivocal squamous	
P01537	2018	2018-Q3	2018-08	Pre-pandemic	Cytology (Pap)	35-39	39	Not performed	Satisfactory	NILM	NILM	Negative (NILM)	
P01538	2018	2018-Q3	2018-08	Pre-pandemic	Cytology (Pap)	30-34	34	Not performed	Satisfactory	NILM	NILM	Negative (NILM)	Candida
P01539	2018	2018-Q3	2018-08	Pre-pandemic	Cytology (Pap)	50-54	53	Not performed	Satisfactory	NILM	NILM	Negative (NILM)	
P01540	2018	2018-Q3	2018-08	Pre-pandemic	Cytology (Pap)	35-39	35	Not performed	Satisfactory	NILM	NILM	Negative (NILM)	
P01541	2018	2018-Q3	2018-08	Pre-pandemic	Cytology (Pap)	60-64	61	Not performed	Satisfactory	NILM	NILM	Negative (NILM)	
P01542	2018	2018-Q3	2018-08	Pre-pandemic	Cytology (Pap)	55-59	59	Not performed	Satisfactory	NILM	NILM	Negative (NILM)	
P01543	2018	2018-Q3	2018-08	Pre-pandemic	Cytology (Pap)	Not recorded		Not performed	Satisfactory	Positive	ASC-H	High-grade squamous	
P01544	2018	2018-Q3	2018-08	Pre-pandemic	Cytology (Pap)	30-34	30	Not performed	Satisfactory	NILM	NILM	Negative (NILM)	
P01545	2018	2018-Q3	2018-08	Pre-pandemic	Cytology (Pap)	55-59	58	Not performed	Satisfactory	NILM	NILM	Negative (NILM)	
P01546	2018	2018-Q3	2018-08	Pre-pandemic	Cytology (Pap)	50-54	54	Not performed	Satisfactory	Positive	ASC-US	Low-grade / equivocal squamous	
P01547	2018	2018-Q3	2018-08	Pre-pandemic	Cytology (Pap)	40-44	41	Not performed	Satisfactory	NILM	NILM	Negative (NILM)	
P01548	2018	2018-Q3	2018-08	Pre-pandemic	Cytology (Pap)	40-44	41	Not performed	Satisfactory	NILM	NILM	Negative (NILM)	
P01549	2018	2018-Q3	2018-08	Pre-pandemic	Cytology (Pap)	40-44	41	Not performed	Satisfactory	NILM	NILM	Negative (NILM)	
P01550	2018	2018-Q3	2018-08	Pre-pandemic	Cytology (Pap)	55-59	58	Not performed	Satisfactory	NILM	NILM	Negative (NILM)	
P01551	2018	2018-Q3	2018-08	Pre-pandemic	Cytology (Pap)	50-54	52	Not performed	Satisfactory	Positive	HSIL	High-grade squamous	
P01552	2018	2018-Q3	2018-08	Pre-pandemic	Cytology (Pap)	40-44	40	Not performed	Satisfactory	NILM	NILM	Negative (NILM)	
P01553	2018	2018-Q3	2018-08	Pre-pandemic	Cytology (Pap)	55-59	58	Not performed	Satisfactory	Positive	ASC-US	Low-grade / equivocal squamous	
P01554	2018	2018-Q3	2018-08	Pre-pandemic	Cytology (Pap)	45-49	49	Not performed	Satisfactory	NILM	NILM	Negative (NILM)	
P01555	2018	2018-Q3	2018-08	Pre-pandemic	Cytology (Pap)	50-54	50	Not performed	Satisfactory	NILM	NILM	Negative (NILM)	
P01556	2018	2018-Q3	2018-08	Pre-pandemic	Cytology (Pap)	55-59	58	Not performed	Satisfactory	NILM	NILM	Negative (NILM)	
P01557	2018	2018-Q3	2018-08	Pre-pandemic	Cytology (Pap)	35-39	39	Not performed	Satisfactory	Positive	HSIL	High-grade squamous	
P01558	2018	2018-Q3	2018-08	Pre-pandemic	Cytology (Pap)	45-49	49	Not performed	Satisfactory	NILM	NILM	Negative (NILM)	
P01559	2018	2018-Q3	2018-08	Pre-pandemic	Cytology (Pap)	50-54	51	Not performed	Satisfactory	NILM	NILM	Negative (NILM)	
P01560	2018	2018-Q3	2018-08	Pre-pandemic	Cytology (Pap)	Not recorded		Not performed	Satisfactory	NILM	NILM	Negative (NILM)	
P01561	2018	2018-Q3	2018-08	Pre-pandemic	Cytology (Pap)	55-59	55	Not performed	Satisfactory	NILM	NILM	Negative (NILM)	
P01562	2018	2018-Q3	2018-08	Pre-pandemic	Cytology (Pap)	40-44	44	Not performed	Satisfactory	NILM	NILM	Negative (NILM)	
P01563	2018	2018-Q3	2018-08	Pre-pandemic	Cytology (Pap)	55-59	56	Not performed	Satisfactory	NILM	NILM	Negative (NILM)	
P01564	2018	2018-Q3	2018-08	Pre-pandemic	Cytology (Pap)	40-44	43	Not performed	Satisfactory	NILM	NILM	Negative (NILM)	
P01565	2018	2018-Q3	2018-08	Pre-pandemic	Cytology (Pap)	35-39	36	Not performed	Satisfactory	NILM	NILM	Negative (NILM)	
P01566	2018	2018-Q3	2018-08	Pre-pandemic	Cytology (Pap)	60-64	61	Not performed	Satisfactory	NILM	NILM	Negative (NILM)	
P01567	2018	2018-Q3	2018-08	Pre-pandemic	Cytology (Pap)	60-64	62	Not performed	Satisfactory	NILM	NILM	Negative (NILM)	
P01568	2018	2018-Q3	2018-08	Pre-pandemic	Cytology (Pap)	40-44	41	Not performed	Satisfactory	NILM	NILM	Negative (NILM)	
P01569	2018	2018-Q3	2018-08	Pre-pandemic	Cytology (Pap)	40-44	41	Not performed	Satisfactory	NILM	NILM	Negative (NILM)	
P01570	2018	2018-Q3	2018-08	Pre-pandemic	Cytology (Pap)	50-54	50	Not performed	Satisfactory	NILM	NILM	Negative (NILM)	
P01571	2018	2018-Q3	2018-08	Pre-pandemic	Cytology (Pap)	55-59	57	Not performed	Satisfactory	Positive	LSIL	Low-grade / equivocal squamous	
P01572	2018	2018-Q3	2018-08	Pre-pandemic	Cytology (Pap)	40-44	42	Not performed	Unsatisfactory	Unsatisfactory	Unsatisfactory	Unsatisfactory	
P01573	2018	2018-Q3	2018-08	Pre-pandemic	Cytology (Pap)	35-39	37	Not performed	Satisfactory	NILM	NILM	Negative (NILM)	
P01574	2018	2018-Q3	2018-08	Pre-pandemic	Cytology (Pap)	Not recorded		Not performed	Satisfactory	NILM	NILM	Negative (NILM)	
P01575	2018	2018-Q3	2018-08	Pre-pandemic	Cytology (Pap)	60-64	63	Not performed	Satisfactory	NILM	NILM	Negative (NILM)	
P01576	2018	2018-Q3	2018-08	Pre-pandemic	Cytology (Pap)	Not recorded		Not performed	Satisfactory	NILM	NILM	Negative (NILM)	
P01577	2018	2018-Q3	2018-08	Pre-pandemic	Cytology (Pap)	45-49	46	Not performed	Satisfactory	Positive	ASC-US	Low-grade / equivocal squamous	
P01578	2018	2018-Q3	2018-08	Pre-pandemic	Cytology (Pap)	40-44	43	Not performed	Satisfactory	NILM	NILM	Negative (NILM)	
P01579	2018	2018-Q3	2018-08	Pre-pandemic	Cytology (Pap)	25-29	28	Not performed	Satisfactory	NILM	NILM	Negative (NILM)	
P01580	2018	2018-Q3	2018-08	Pre-pandemic	Cytology (Pap)	Not recorded		Not performed	Satisfactory	NILM	NILM	Negative (NILM)	
P01581	2018	2018-Q3	2018-08	Pre-pandemic	Cytology (Pap)	50-54	54	Not performed	Satisfactory	NILM	NILM	Negative (NILM)	
P01582	2018	2018-Q3	2018-08	Pre-pandemic	Cytology (Pap)	45-49	47	Not performed	Satisfactory	NILM	NILM	Negative (NILM)	
P01583	2018	2018-Q3	2018-08	Pre-pandemic	Cytology (Pap)	35-39	35	Not performed	Satisfactory	Positive	ASC-US	Low-grade / equivocal squamous	
P01584	2018	2018-Q3	2018-08	Pre-pandemic	Cytology (Pap)	40-44	44	Not performed	Satisfactory	NILM	NILM	Negative (NILM)	
P01585	2018	2018-Q3	2018-08	Pre-pandemic	Cytology (Pap)	60-64	63	Not performed	Satisfactory	NILM	NILM	Negative (NILM)	
P01586	2018	2018-Q3	2018-08	Pre-pandemic	Cytology (Pap)	35-39	35	Not performed	Satisfactory	NILM	NILM	Negative (NILM)	
P01587	2018	2018-Q3	2018-08	Pre-pandemic	Cytology (Pap)	50-54	52	Not performed	Satisfactory	Positive	ASC-US	Low-grade / equivocal squamous	
P01588	2018	2018-Q3	2018-08	Pre-pandemic	Cytology (Pap)	50-54	51	Not performed	Satisfactory	NILM	NILM	Negative (NILM)	
P01589	2018	2018-Q3	2018-08	Pre-pandemic	Cytology (Pap)	50-54	51	Not performed	Satisfactory	Positive	LSIL	Low-grade / equivocal squamous	
P01590	2018	2018-Q3	2018-08	Pre-pandemic	Cytology (Pap)	Not recorded		Not performed	Satisfactory	NILM	NILM	Negative (NILM)	
P01591	2018	2018-Q3	2018-08	Pre-pandemic	Cytology (Pap)	40-44	42	Not performed	Satisfactory	NILM	NILM	Negative (NILM)	
P01592	2018	2018-Q3	2018-08	Pre-pandemic	Cytology (Pap)	Not recorded		Not performed	Satisfactory	NILM	NILM	Negative (NILM)	
P01593	2018	2018-Q3	2018-08	Pre-pandemic	Cytology (Pap)	35-39	35	Not performed	Satisfactory	NILM	NILM	Negative (NILM)	
P01594	2018	2018-Q3	2018-08	Pre-pandemic	Cytology (Pap)	60-64	60	Not performed	Satisfactory	Positive	ASC-US	Low-grade / equivocal squamous	
P01595	2018	2018-Q3	2018-08	Pre-pandemic	Cytology (Pap)	40-44	41	Not performed	Satisfactory	NILM	NILM	Negative (NILM)	
P01596	2018	2018-Q3	2018-08	Pre-pandemic	Cytology (Pap)	25-29	28	Not performed	Satisfactory	NILM	NILM	Negative (NILM)	
P01597	2018	2018-Q3	2018-08	Pre-pandemic	Cytology (Pap)	30-34	34	Not performed	Satisfactory	NILM	NILM	Negative (NILM)	
P01598	2018	2018-Q3	2018-08	Pre-pandemic	Cytology (Pap)	55-59	55	Not performed	Satisfactory	NILM	NILM	Negative (NILM)	Candida

patient_id	year	quarter	month	period	primary_test	age_group	age_years	hpv_result	adequacy	result	bethesda	grade	infection
P01599	2018	2018-Q3	2018-08	Pre-pandemic	Cytology (Pap)	45-49	47	Not performed	Satisfactory	NILM	NILM	Negative (NILM)	
P01600	2018	2018-Q3	2018-08	Pre-pandemic	Cytology (Pap)	50-54	54	Not performed	Satisfactory	NILM	NILM	Negative (NILM)	
P01601	2018	2018-Q3	2018-08	Pre-pandemic	Cytology (Pap)	50-54	53	Not performed	Satisfactory	NILM	NILM	Negative (NILM)	Trichomonas
P01602	2018	2018-Q3	2018-08	Pre-pandemic	Cytology (Pap)	40-44	43	Not performed	Satisfactory	NILM	NILM	Negative (NILM)	
P01603	2018	2018-Q3	2018-08	Pre-pandemic	Cytology (Pap)	25-29	27	Not performed	Satisfactory	NILM	NILM	Negative (NILM)	
P01604	2018	2018-Q3	2018-08	Pre-pandemic	Cytology (Pap)	60-64	61	Not performed	Satisfactory	NILM	NILM	Negative (NILM)	
P01605	2018	2018-Q3	2018-08	Pre-pandemic	Cytology (Pap)	55-59	55	Not performed	Satisfactory	NILM	NILM	Negative (NILM)	
P01606	2018	2018-Q3	2018-08	Pre-pandemic	Cytology (Pap)	35-39	37	Not performed	Satisfactory	NILM	NILM	Negative (NILM)	
P01607	2018	2018-Q3	2018-08	Pre-pandemic	Cytology (Pap)	55-59	59	Not performed	Satisfactory	NILM	NILM	Negative (NILM)	
P01608	2018	2018-Q3	2018-08	Pre-pandemic	Cytology (Pap)	50-54	51	Not performed	Satisfactory	NILM	NILM	Negative (NILM)	
P01609	2018	2018-Q3	2018-08	Pre-pandemic	Cytology (Pap)	40-44	44	Not performed	Satisfactory	NILM	NILM	Negative (NILM)	
P01610	2018	2018-Q3	2018-08	Pre-pandemic	Cytology (Pap)	35-39	36	Not performed	Satisfactory	NILM	NILM	Negative (NILM)	
P01611	2018	2018-Q3	2018-08	Pre-pandemic	Cytology (Pap)	55-59	55	Not performed	Satisfactory	NILM	NILM	Negative (NILM)	
P01612	2018	2018-Q3	2018-08	Pre-pandemic	Cytology (Pap)	30-34	30	Not performed	Satisfactory	NILM	NILM	Negative (NILM)	
P01613	2018	2018-Q3	2018-08	Pre-pandemic	Cytology (Pap)	60-64	64	Not performed	Satisfactory	NILM	NILM	Negative (NILM)	
P01614	2018	2018-Q3	2018-08	Pre-pandemic	Cytology (Pap)	40-44	42	Not performed	Satisfactory	NILM	NILM	Negative (NILM)	
P01615	2018	2018-Q3	2018-08	Pre-pandemic	Cytology (Pap)	35-39	38	Not performed	Satisfactory	NILM	NILM	Negative (NILM)	
P01616	2018	2018-Q3	2018-08	Pre-pandemic	Cytology (Pap)	50-54	50	Not performed	Satisfactory	Positive	ASC-US	Low-grade / equivocal squamous	
P01617	2018	2018-Q3	2018-08	Pre-pandemic	Cytology (Pap)	30-34	32	Not performed	Satisfactory	NILM	NILM	Negative (NILM)	
P01618	2018	2018-Q3	2018-08	Pre-pandemic	Cytology (Pap)	35-39	37	Not performed	Satisfactory	Positive	HSIL	High-grade squamous	
P01619	2018	2018-Q3	2018-08	Pre-pandemic	Cytology (Pap)	60-64	62	Not performed	Satisfactory	NILM	NILM	Negative (NILM)	
P01620	2018	2018-Q3	2018-08	Pre-pandemic	Cytology (Pap)	35-39	36	Not performed	Satisfactory	Positive	ASC-US	Low-grade / equivocal squamous	
P01621	2018	2018-Q3	2018-08	Pre-pandemic	Cytology (Pap)	40-44	40	Not performed	Satisfactory	NILM	NILM	Negative (NILM)	
P01622	2018	2018-Q3	2018-08	Pre-pandemic	Cytology (Pap)	30-34	31	Not performed	Satisfactory	NILM	NILM	Negative (NILM)	Other
P01623	2018	2018-Q3	2018-08	Pre-pandemic	Cytology (Pap)	40-44	40	Not performed	Satisfactory	NILM	NILM	Negative (NILM)	
P01624	2018	2018-Q3	2018-08	Pre-pandemic	Cytology (Pap)	60-64	62	Not performed	Satisfactory	NILM	NILM	Negative (NILM)	
P01625	2018	2018-Q3	2018-08	Pre-pandemic	Cytology (Pap)	40-44	40	Not performed	Satisfactory	NILM	NILM	Negative (NILM)	
P01626	2018	2018-Q3	2018-08	Pre-pandemic	Cytology (Pap)	35-39	39	Not performed	Satisfactory	NILM	NILM	Negative (NILM)	
P01627	2018	2018-Q3	2018-08	Pre-pandemic	Cytology (Pap)	30-34	30	Not performed	Satisfactory	NILM	NILM	Negative (NILM)	
P01628	2018	2018-Q3	2018-08	Pre-pandemic	Cytology (Pap)	Not recorded		Not performed	Satisfactory	Positive	ASC-H	High-grade squamous	
P01629	2018	2018-Q3	2018-08	Pre-pandemic	Cytology (Pap)	55-59	59	Not performed	Satisfactory	NILM	NILM	Negative (NILM)	
P01630	2018	2018-Q3	2018-08	Pre-pandemic	Cytology (Pap)	60-64	63	Not performed	Satisfactory	NILM	NILM	Negative (NILM)	
P01631	2018	2018-Q3	2018-08	Pre-pandemic	Cytology (Pap)	35-39	39	Not performed	Satisfactory	NILM	NILM	Negative (NILM)	
P01632	2018	2018-Q3	2018-08	Pre-pandemic	Cytology (Pap)	35-39	35	Not performed	Satisfactory	Positive	ASC-US	Low-grade / equivocal squamous	
P01633	2018	2018-Q3	2018-08	Pre-pandemic	Cytology (Pap)	25-29	27	Not performed	Satisfactory	NILM	NILM	Negative (NILM)	
P01634	2018	2018-Q3	2018-08	Pre-pandemic	Cytology (Pap)	50-54	54	Not performed	Satisfactory	NILM	NILM	Negative (NILM)	Candida
P01635	2018	2018-Q3	2018-08	Pre-pandemic	Cytology (Pap)	25-29	25	Not performed	Satisfactory	NILM	NILM	Negative (NILM)	
P01636	2018	2018-Q3	2018-08	Pre-pandemic	Cytology (Pap)	35-39	38	Not performed	Satisfactory	NILM	NILM	Negative (NILM)	
P01637	2018	2018-Q3	2018-08	Pre-pandemic	Cytology (Pap)	30-34	31	Not performed	Satisfactory	NILM	NILM	Negative (NILM)	
P01638	2018	2018-Q3	2018-08	Pre-pandemic	Cytology (Pap)	40-44	41	Not performed	Satisfactory	NILM	NILM	Negative (NILM)	
P01639	2018	2018-Q3	2018-08	Pre-pandemic	Cytology (Pap)	Not recorded		Not performed	Satisfactory	NILM	NILM	Negative (NILM)	
P01640	2018	2018-Q3	2018-08	Pre-pandemic	Cytology (Pap)	50-54	51	Not performed	Satisfactory	NILM	NILM	Negative (NILM)	
P01641	2018	2018-Q3	2018-08	Pre-pandemic	Cytology (Pap)	35-39	37	Not performed	Satisfactory	NILM	NILM	Negative (NILM)	
P01642	2018	2018-Q3	2018-08	Pre-pandemic	Cytology (Pap)	35-39	35	Not performed	Satisfactory	NILM	NILM	Negative (NILM)	
P01643	2018	2018-Q3	2018-08	Pre-pandemic	Cytology (Pap)	30-34	32	Not performed	Satisfactory	NILM	NILM	Negative (NILM)	Candida
P01644	2018	2018-Q3	2018-08	Pre-pandemic	Cytology (Pap)	45-49	49	Not performed	Satisfactory	NILM	NILM	Negative (NILM)	
P01645	2018	2018-Q3	2018-08	Pre-pandemic	Cytology (Pap)	30-34	32	Not performed	Satisfactory	NILM	NILM	Negative (NILM)	
P01646	2018	2018-Q3	2018-08	Pre-pandemic	Cytology (Pap)	60-64	63	Not performed	Satisfactory	NILM	NILM	Negative (NILM)	
P01647	2018	2018-Q3	2018-08	Pre-pandemic	Cytology (Pap)	40-44	40	Not performed	Satisfactory	NILM	NILM	Negative (NILM)	
P01648	2018	2018-Q3	2018-08	Pre-pandemic	Cytology (Pap)	50-54	51	Not performed	Satisfactory	NILM	NILM	Negative (NILM)	
P01649	2018	2018-Q3	2018-08	Pre-pandemic	Cytology (Pap)	25-29	29	Not performed	Satisfactory	Positive	LSIL	Low-grade / equivocal squamous	Trichomonas
P01650	2018	2018-Q3	2018-08	Pre-pandemic	Cytology (Pap)	45-49	48	Not performed	Satisfactory	NILM	NILM	Negative (NILM)	
P01651	2018	2018-Q3	2018-08	Pre-pandemic	Cytology (Pap)	50-54	51	Not performed	Satisfactory	NILM	NILM	Negative (NILM)	
P01652	2018	2018-Q3	2018-08	Pre-pandemic	Cytology (Pap)	40-44	42	Not performed	Satisfactory	Positive	ASC-US	Low-grade / equivocal squamous	
P01653	2018	2018-Q3	2018-08	Pre-pandemic	Cytology (Pap)	30-34	34	Not performed	Satisfactory	NILM	NILM	Negative (NILM)	
P01654	2018	2018-Q3	2018-08	Pre-pandemic	Cytology (Pap)	50-54	54	Not performed	Satisfactory	NILM	NILM	Negative (NILM)	
P01655	2018	2018-Q3	2018-08	Pre-pandemic	Cytology (Pap)	45-49	47	Not performed	Satisfactory	NILM	NILM	Negative (NILM)	
P01656	2018	2018-Q3	2018-08	Pre-pandemic	Cytology (Pap)	50-54	53	Not performed	Satisfactory	NILM	NILM	Negative (NILM)	
P01657	2018	2018-Q3	2018-08	Pre-pandemic	Cytology (Pap)	60-64	60	Not performed	Satisfactory	NILM	NILM	Negative (NILM)	
P01658	2018	2018-Q3	2018-08	Pre-pandemic	Cytology (Pap)	35-39	39	Not performed	Satisfactory	Positive	LSIL	Low-grade / equivocal squamous	
P01659	2018	2018-Q3	2018-08	Pre-pandemic	Cytology (Pap)	40-44	40	Not performed	Satisfactory	NILM	NILM	Negative (NILM)	
P01660	2018	2018-Q3	2018-08	Pre-pandemic	Cytology (Pap)	50-54	51	Not performed	Satisfactory	Positive	ASC-H	High-grade squamous	
P01661	2018	2018-Q3	2018-08	Pre-pandemic	Cytology (Pap)	25-29	29	Not performed	Satisfactory	NILM	NILM	Negative (NILM)	
P01662	2018	2018-Q3	2018-08	Pre-pandemic	Cytology (Pap)	60-64	60	Not performed	Satisfactory	NILM	NILM	Negative (NILM)	
P01663	2018	2018-Q3	2018-08	Pre-pandemic	Cytology (Pap)	Not recorded		Not performed	Satisfactory	Positive	ASC-US	Low-grade / equivocal squamous	
P01664	2018	2018-Q3	2018-08	Pre-pandemic	Cytology (Pap)	50-54	50	Not performed	Satisfactory	NILM	NILM	Negative (NILM)	
P01665	2018	2018-Q3	2018-08	Pre-pandemic	Cytology (Pap)	35-39	36	Not performed	Satisfactory	NILM	NILM	Negative (NILM)	
P01666	2018	2018-Q3	2018-08	Pre-pandemic	Cytology (Pap)	35-39	37	Not performed	Satisfactory	NILM	NILM	Negative (NILM)	
P01667	2018	2018-Q3	2018-08	Pre-pandemic	Cytology (Pap)	30-34	34	Not performed	Satisfactory	NILM	NILM	Negative (NILM)	
P01668	2018	2018-Q3	2018-08	Pre-pandemic	Cytology (Pap)	35-39	38	Not performed	Satisfactory	Positive	LSIL	Low-grade / equivocal squamous	
P01669	2018	2018-Q3	2018-08	Pre-pandemic	Cytology (Pap)	50-54	54	Not performed	Satisfactory	NILM	NILM	Negative (NILM)	
P01670	2018	2018-Q3	2018-08	Pre-pandemic	Cytology (Pap)	35-39	36	Not performed	Satisfactory	NILM	NILM	Negative (NILM)	
P01671	2018	2018-Q3	2018-08	Pre-pandemic	Cytology (Pap)	30-34	34	Not performed	Satisfactory	NILM	NILM	Negative (NILM)	
P01672	2018	2018-Q3	2018-08	Pre-pandemic	Cytology (Pap)	35-39	38	Not performed	Satisfactory	Positive	ASC-US	Low-grade / equivocal squamous	
P01673	2018	2018-Q3	2018-08	Pre-pandemic	Cytology (Pap)	35-39	38	Not performed	Satisfactory	NILM	NILM	Negative (NILM)	
P01674	2018	2018-Q3	2018-08	Pre-pandemic	Cytology (Pap)	40-44	41	Not performed	Satisfactory	NILM	NILM	Negative (NILM)	
P01675	2018	2018-Q3	2018-08	Pre-pandemic	Cytology (Pap)	40-44	41	Not performed	Satisfactory	Positive	ASC-US	Low-grade / equivocal squamous	
P01676	2018	2018-Q3	2018-08	Pre-pandemic	Cytology (Pap)	55-59	58	Not performed	Satisfactory	NILM	NILM	Negative (NILM)	
P01677	2018	2018-Q3	2018-08	Pre-pandemic	Cytology (Pap)	40-44	44	Not performed	Satisfactory	NILM	NILM	Negative (NILM)	
P01678	2018	2018-Q3	2018-08	Pre-pandemic	Cytology (Pap)	30-34	32	Not performed	Satisfactory	NILM	NILM	Negative (NILM)	

patient_id	year	quarter	month	period	primary_test	age_group	age_years	hpv_result	adequacy	result	bethesda	grade	infection
P01679	2018	2018-Q3	2018-08	Pre-pandemic	Cytology (Pap)	55-59	57	Not performed	Satisfactory	NILM	NILM	Negative (NILM)	
P01680	2018	2018-Q3	2018-08	Pre-pandemic	Cytology (Pap)	60-64	63	Not performed	Satisfactory	NILM	NILM	Negative (NILM)	
P01681	2018	2018-Q3	2018-08	Pre-pandemic	Cytology (Pap)	55-59	55	Not performed	Satisfactory	NILM	NILM	Negative (NILM)	
P01682	2018	2018-Q3	2018-08	Pre-pandemic	Cytology (Pap)	60-64	60	Not performed	Satisfactory	NILM	NILM	Negative (NILM)	
P01683	2018	2018-Q3	2018-08	Pre-pandemic	Cytology (Pap)	Not recorded		Not performed	Satisfactory	NILM	NILM	Negative (NILM)	
P01684	2018	2018-Q3	2018-08	Pre-pandemic	Cytology (Pap)	25-29	29	Not performed	Satisfactory	NILM	NILM	Negative (NILM)	
P01685	2018	2018-Q3	2018-08	Pre-pandemic	Cytology (Pap)	40-44	40	Not performed	Satisfactory	NILM	NILM	Negative (NILM)	
P01686	2018	2018-Q3	2018-08	Pre-pandemic	Cytology (Pap)	45-49	47	Not performed	Satisfactory	NILM	NILM	Negative (NILM)	
P01687	2018	2018-Q3	2018-08	Pre-pandemic	Cytology (Pap)	25-29	28	Not performed	Satisfactory	NILM	NILM	Negative (NILM)	
P01688	2018	2018-Q3	2018-08	Pre-pandemic	Cytology (Pap)	40-44	42	Not performed	Satisfactory	NILM	NILM	Negative (NILM)	
P01689	2018	2018-Q3	2018-08	Pre-pandemic	Cytology (Pap)	25-29	26	Not performed	Satisfactory	NILM	NILM	Negative (NILM)	
P01690	2018	2018-Q3	2018-08	Pre-pandemic	Cytology (Pap)	45-49	47	Not performed	Satisfactory	NILM	NILM	Negative (NILM)	
P01691	2018	2018-Q3	2018-08	Pre-pandemic	Cytology (Pap)	40-44	42	Not performed	Satisfactory	NILM	NILM	Negative (NILM)	
P01692	2018	2018-Q3	2018-08	Pre-pandemic	Cytology (Pap)	35-39	35	Not performed	Satisfactory	NILM	NILM	Negative (NILM)	
P01693	2018	2018-Q3	2018-08	Pre-pandemic	Cytology (Pap)	40-44	41	Not performed	Satisfactory	NILM	NILM	Negative (NILM)	
P01694	2018	2018-Q3	2018-08	Pre-pandemic	Cytology (Pap)	50-54	54	Not performed	Satisfactory	Positive	ASC-US	Low-grade / equivocal squamous	
P01695	2018	2018-Q3	2018-08	Pre-pandemic	Cytology (Pap)	Not recorded		Not performed	Satisfactory	NILM	NILM	Negative (NILM)	
P01696	2018	2018-Q3	2018-08	Pre-pandemic	Cytology (Pap)	40-44	40	Not performed	Satisfactory	NILM	NILM	Negative (NILM)	
P01697	2018	2018-Q3	2018-08	Pre-pandemic	Cytology (Pap)	50-54	50	Not performed	Satisfactory	NILM	NILM	Negative (NILM)	
P01698	2018	2018-Q3	2018-08	Pre-pandemic	Cytology (Pap)	35-39	37	Not performed	Satisfactory	NILM	NILM	Negative (NILM)	
P01699	2018	2018-Q3	2018-08	Pre-pandemic	Cytology (Pap)	50-54	54	Not performed	Satisfactory	NILM	NILM	Negative (NILM)	
P01700	2018	2018-Q3	2018-08	Pre-pandemic	Cytology (Pap)	35-39	37	Not performed	Satisfactory	NILM	NILM	Negative (NILM)	
P01701	2018	2018-Q3	2018-08	Pre-pandemic	Cytology (Pap)	50-54	54	Not performed	Satisfactory	NILM	NILM	Negative (NILM)	
P01702	2018	2018-Q3	2018-08	Pre-pandemic	Cytology (Pap)	50-54	51	Not performed	Satisfactory	NILM	NILM	Negative (NILM)	
P01703	2018	2018-Q3	2018-08	Pre-pandemic	Cytology (Pap)	40-44	41	Not performed	Satisfactory	NILM	NILM	Negative (NILM)	Candida
P01704	2018	2018-Q3	2018-08	Pre-pandemic	Cytology (Pap)	40-44	41	Not performed	Satisfactory	NILM	NILM	Negative (NILM)	
P01705	2018	2018-Q3	2018-08	Pre-pandemic	Cytology (Pap)	30-34	33	Not performed	Satisfactory	NILM	NILM	Negative (NILM)	
P01706	2018	2018-Q3	2018-08	Pre-pandemic	Cytology (Pap)	25-29	26	Not performed	Satisfactory	Positive	HSIL	High-grade squamous	
P01707	2018	2018-Q3	2018-08	Pre-pandemic	Cytology (Pap)	40-44	41	Not performed	Satisfactory	NILM	NILM	Negative (NILM)	
P01708	2018	2018-Q3	2018-08	Pre-pandemic	Cytology (Pap)	50-54	50	Not performed	Satisfactory	NILM	NILM	Negative (NILM)	
P01709	2018	2018-Q3	2018-08	Pre-pandemic	Cytology (Pap)	45-49	49	Not performed	Satisfactory	NILM	NILM	Negative (NILM)	
P01710	2018	2018-Q3	2018-08	Pre-pandemic	Cytology (Pap)	35-39	35	Not performed	Satisfactory	NILM	NILM	Negative (NILM)	
P01711	2018	2018-Q3	2018-08	Pre-pandemic	Cytology (Pap)	50-54	50	Not performed	Satisfactory	NILM	NILM	Negative (NILM)	
P01712	2018	2018-Q3	2018-08	Pre-pandemic	Cytology (Pap)	30-34	32	Not performed	Satisfactory	NILM	NILM	Negative (NILM)	
P01713	2018	2018-Q3	2018-08	Pre-pandemic	Cytology (Pap)	25-29	25	Not performed	Satisfactory	NILM	NILM	Negative (NILM)	
P01714	2018	2018-Q3	2018-08	Pre-pandemic	Cytology (Pap)	30-34	33	Not performed	Satisfactory	NILM	NILM	Negative (NILM)	
P01715	2018	2018-Q3	2018-08	Pre-pandemic	Cytology (Pap)	30-34	33	Not performed	Satisfactory	NILM	NILM	Negative (NILM)	
P01716	2018	2018-Q3	2018-08	Pre-pandemic	Cytology (Pap)	40-44	42	Not performed	Satisfactory	NILM	NILM	Negative (NILM)	Candida
P01717	2018	2018-Q3	2018-08	Pre-pandemic	Cytology (Pap)	40-44	42	Not performed	Satisfactory	NILM	NILM	Negative (NILM)	
P01718	2018	2018-Q3	2018-08	Pre-pandemic	Cytology (Pap)	25-29	26	Not performed	Satisfactory	NILM	NILM	Negative (NILM)	
P01719	2018	2018-Q3	2018-08	Pre-pandemic	Cytology (Pap)	50-54	54	Not performed	Satisfactory	Positive	HSIL	High-grade squamous	
P01720	2018	2018-Q3	2018-08	Pre-pandemic	Cytology (Pap)	50-54	51	Not performed	Satisfactory	NILM	NILM	Negative (NILM)	
P01721	2018	2018-Q3	2018-08	Pre-pandemic	Cytology (Pap)	35-39	38	Not performed	Satisfactory	NILM	NILM	Negative (NILM)	
P01722	2018	2018-Q3	2018-08	Pre-pandemic	Cytology (Pap)	30-34	31	Not performed	Satisfactory	NILM	NILM	Negative (NILM)	
P01723	2018	2018-Q3	2018-08	Pre-pandemic	Cytology (Pap)	40-44	44	Not performed	Satisfactory	NILM	NILM	Negative (NILM)	
P01724	2018	2018-Q3	2018-08	Pre-pandemic	Cytology (Pap)	30-34	32	Not performed	Satisfactory	Positive	LSIL	Low-grade / equivocal squamous	
P01725	2018	2018-Q3	2018-08	Pre-pandemic	Cytology (Pap)	40-44	41	Not performed	Satisfactory	NILM	NILM	Negative (NILM)	
P01726	2018	2018-Q3	2018-08	Pre-pandemic	Cytology (Pap)	40-44	40	Not performed	Satisfactory	NILM	NILM	Negative (NILM)	
P01727	2018	2018-Q3	2018-08	Pre-pandemic	Cytology (Pap)	Not recorded		Not performed	Unsatisfactory	Unsatisfactory	Unsatisfactory	Unsatisfactory	
P01728	2018	2018-Q3	2018-08	Pre-pandemic	Cytology (Pap)	25-29	27	Not performed	Satisfactory	NILM	NILM	Negative (NILM)	
P01729	2018	2018-Q3	2018-08	Pre-pandemic	Cytology (Pap)	40-44	40	Not performed	Satisfactory	NILM	NILM	Negative (NILM)	
P01730	2018	2018-Q3	2018-08	Pre-pandemic	Cytology (Pap)	25-29	25	Not performed	Satisfactory	NILM	NILM	Negative (NILM)	
P01731	2018	2018-Q3	2018-08	Pre-pandemic	Cytology (Pap)	55-59	55	Not performed	Satisfactory	Positive	ASC-US	Low-grade / equivocal squamous	
P01732	2018	2018-Q3	2018-08	Pre-pandemic	Cytology (Pap)	30-34	30	Not performed	Satisfactory	NILM	NILM	Negative (NILM)	
P01733	2018	2018-Q3	2018-08	Pre-pandemic	Cytology (Pap)	35-39	39	Not performed	Satisfactory	NILM	NILM	Negative (NILM)	
P01734	2018	2018-Q3	2018-08	Pre-pandemic	Cytology (Pap)	25-29	29	Not performed	Satisfactory	NILM	NILM	Negative (NILM)	
P01735	2018	2018-Q3	2018-08	Pre-pandemic	Cytology (Pap)	35-39	35	Not performed	Satisfactory	Positive	ASC-US	Low-grade / equivocal squamous	
P01736	2018	2018-Q3	2018-08	Pre-pandemic	Cytology (Pap)	40-44	43	Not performed	Satisfactory	NILM	NILM	Negative (NILM)	
P01737	2018	2018-Q3	2018-08	Pre-pandemic	Cytology (Pap)	45-49	46	Not performed	Satisfactory	NILM	NILM	Negative (NILM)	
P01738	2018	2018-Q3	2018-08	Pre-pandemic	Cytology (Pap)	40-44	41	Not performed	Satisfactory	NILM	NILM	Negative (NILM)	
P01739	2018	2018-Q3	2018-08	Pre-pandemic	Cytology (Pap)	40-44	44	Not performed	Satisfactory	Positive	ASC-US	Low-grade / equivocal squamous	
P01740	2018	2018-Q3	2018-08	Pre-pandemic	Cytology (Pap)	45-49	49	Not performed	Satisfactory	NILM	NILM	Negative (NILM)	
P01741	2018	2018-Q3	2018-08	Pre-pandemic	Cytology (Pap)	35-39	35	Not performed	Satisfactory	Positive	ASC-US	Low-grade / equivocal squamous	
P01742	2018	2018-Q3	2018-08	Pre-pandemic	Cytology (Pap)	45-49	49	Not performed	Satisfactory	NILM	NILM	Negative (NILM)	
P01743	2018	2018-Q3	2018-08	Pre-pandemic	Cytology (Pap)	40-44	41	Not performed	Satisfactory	NILM	NILM	Negative (NILM)	
P01744	2018	2018-Q3	2018-08	Pre-pandemic	Cytology (Pap)	40-44	42	Not performed	Satisfactory	NILM	NILM	Negative (NILM)	
P01745	2018	2018-Q3	2018-08	Pre-pandemic	Cytology (Pap)	30-34	33	Not performed	Satisfactory	NILM	NILM	Negative (NILM)	Gardnerella
P01746	2018	2018-Q3	2018-08	Pre-pandemic	Cytology (Pap)	35-39	39	Not performed	Satisfactory	NILM	NILM	Negative (NILM)	
P01747	2018	2018-Q3	2018-08	Pre-pandemic	Cytology (Pap)	25-29	28	Not performed	Satisfactory	NILM	NILM	Negative (NILM)	
P01748	2018	2018-Q3	2018-08	Pre-pandemic	Cytology (Pap)	25-29	26	Not performed	Satisfactory	NILM	NILM	Negative (NILM)	
P01749	2018	2018-Q3	2018-08	Pre-pandemic	Cytology (Pap)	25-29	27	Not performed	Satisfactory	NILM	NILM	Negative (NILM)	
P01750	2018	2018-Q3	2018-08	Pre-pandemic	Cytology (Pap)	55-59	58	Not performed	Satisfactory	NILM	NILM	Negative (NILM)	
P01751	2018	2018-Q3	2018-08	Pre-pandemic	Cytology (Pap)	60-64	61	Not performed	Satisfactory	NILM	NILM	Negative (NILM)	Trichomonas
P01752	2018	2018-Q3	2018-08	Pre-pandemic	Cytology (Pap)	35-39	38	Not performed	Satisfactory	NILM	NILM	Negative (NILM)	Candida
P01753	2018	2018-Q3	2018-08	Pre-pandemic	Cytology (Pap)	45-49	47	Not performed	Satisfactory	NILM	NILM	Negative (NILM)	
P01754	2018	2018-Q3	2018-08	Pre-pandemic	Cytology (Pap)	40-44	43	Not performed	Satisfactory	NILM	NILM	Negative (NILM)	
P01755	2018	2018-Q3	2018-08	Pre-pandemic	Cytology (Pap)	50-54	53	Not performed	Satisfactory	NILM	NILM	Negative (NILM)	
P01756	2018	2018-Q3	2018-08	Pre-pandemic	Cytology (Pap)	45-49	47	Not performed	Satisfactory	NILM	NILM	Negative (NILM)	
P01757	2018	2018-Q3	2018-08	Pre-pandemic	Cytology (Pap)	Not recorded		Not performed	Satisfactory	NILM	NILM	Negative (NILM)	
P01758	2018	2018-Q3	2018-08	Pre-pandemic	Cytology (Pap)	35-39	37	Not performed	Satisfactory	Positive	ASC-US	Low-grade / equivocal squamous	

patient_id	year	quarter	month	period	primary_test	age_group	age_years	hpv_result	adequacy	result	bethesda	grade	infection
P01759	2018	2018-Q3	2018-08	Pre-pandemic	Cytology (Pap)	35-39	38	Not performed	Satisfactory	NILM	NILM	Negative (NILM)	Candida
P01760	2018	2018-Q3	2018-08	Pre-pandemic	Cytology (Pap)	25-29	29	Not performed	Satisfactory	NILM	NILM	Negative (NILM)	
P01761	2018	2018-Q3	2018-08	Pre-pandemic	Cytology (Pap)	45-49	49	Not performed	Satisfactory	NILM	NILM	Negative (NILM)	
P01762	2018	2018-Q3	2018-08	Pre-pandemic	Cytology (Pap)	50-54	54	Not performed	Satisfactory	NILM	NILM	Negative (NILM)	
P01763	2018	2018-Q3	2018-08	Pre-pandemic	Cytology (Pap)	25-29	26	Not performed	Satisfactory	NILM	NILM	Negative (NILM)	
P01764	2018	2018-Q3	2018-08	Pre-pandemic	Cytology (Pap)	50-54	53	Not performed	Satisfactory	NILM	NILM	Negative (NILM)	
P01765	2018	2018-Q3	2018-08	Pre-pandemic	Cytology (Pap)	40-44	44	Not performed	Satisfactory	NILM	NILM	Negative (NILM)	
P01766	2018	2018-Q3	2018-08	Pre-pandemic	Cytology (Pap)	45-49	46	Not performed	Satisfactory	Positive	AGC	Glandular	
P01767	2018	2018-Q3	2018-08	Pre-pandemic	Cytology (Pap)	40-44	42	Not performed	Satisfactory	NILM	NILM	Negative (NILM)	
P01768	2018	2018-Q3	2018-08	Pre-pandemic	Cytology (Pap)	50-54	51	Not performed	Satisfactory	NILM	NILM	Negative (NILM)	
P01769	2018	2018-Q3	2018-08	Pre-pandemic	Cytology (Pap)	60-64	64	Not performed	Satisfactory	NILM	NILM	Negative (NILM)	
P01770	2018	2018-Q3	2018-08	Pre-pandemic	Cytology (Pap)	50-54	54	Not performed	Satisfactory	NILM	NILM	Negative (NILM)	
P01771	2018	2018-Q3	2018-08	Pre-pandemic	Cytology (Pap)	40-44	43	Not performed	Satisfactory	NILM	NILM	Negative (NILM)	
P01772	2018	2018-Q3	2018-08	Pre-pandemic	Cytology (Pap)	30-34	32	Not performed	Satisfactory	NILM	NILM	Negative (NILM)	
P01773	2018	2018-Q3	2018-08	Pre-pandemic	Cytology (Pap)	30-34	33	Not performed	Satisfactory	NILM	NILM	Negative (NILM)	
P01774	2018	2018-Q3	2018-08	Pre-pandemic	Cytology (Pap)	45-49	49	Not performed	Satisfactory	NILM	NILM	Negative (NILM)	
P01775	2018	2018-Q3	2018-08	Pre-pandemic	Cytology (Pap)	50-54	52	Not performed	Satisfactory	NILM	NILM	Negative (NILM)	
P01776	2018	2018-Q3	2018-08	Pre-pandemic	Cytology (Pap)	Not recorded		Not performed	Satisfactory	NILM	NILM	Negative (NILM)	
P01777	2018	2018-Q3	2018-08	Pre-pandemic	Cytology (Pap)	40-44	42	Not performed	Unsatisfactory	Unsatisfactory	Unsatisfactory	Unsatisfactory	
P01778	2018	2018-Q3	2018-08	Pre-pandemic	Cytology (Pap)	45-49	47	Not performed	Satisfactory	NILM	NILM	Negative (NILM)	
P01779	2018	2018-Q3	2018-08	Pre-pandemic	Cytology (Pap)	Not recorded		Not performed	Satisfactory	NILM	NILM	Negative (NILM)	
P01780	2018	2018-Q3	2018-08	Pre-pandemic	Cytology (Pap)	30-34	32	Not performed	Satisfactory	Positive	LSIL	Low-grade / equivocal squamous	
P01781	2018	2018-Q3	2018-08	Pre-pandemic	Cytology (Pap)	50-54	51	Not performed	Satisfactory	NILM	NILM	Negative (NILM)	
P01782	2018	2018-Q3	2018-08	Pre-pandemic	Cytology (Pap)	40-44	42	Not performed	Satisfactory	NILM	NILM	Negative (NILM)	
P01783	2018	2018-Q3	2018-08	Pre-pandemic	Cytology (Pap)	25-29	26	Not performed	Satisfactory	NILM	NILM	Negative (NILM)	
P01784	2018	2018-Q3	2018-08	Pre-pandemic	Cytology (Pap)	45-49	47	Not performed	Satisfactory	NILM	NILM	Negative (NILM)	
P01785	2018	2018-Q3	2018-08	Pre-pandemic	Cytology (Pap)	Not recorded		Not performed	Satisfactory	NILM	NILM	Negative (NILM)	
P01786	2018	2018-Q3	2018-08	Pre-pandemic	Cytology (Pap)	30-34	30	Not performed	Satisfactory	NILM	NILM	Negative (NILM)	
P01787	2018	2018-Q3	2018-08	Pre-pandemic	Cytology (Pap)	30-34	32	Not performed	Satisfactory	NILM	NILM	Negative (NILM)	Candida
P01788	2018	2018-Q3	2018-08	Pre-pandemic	Cytology (Pap)	40-44	40	Not performed	Satisfactory	Positive	HSIL	High-grade squamous	
P01789	2018	2018-Q3	2018-08	Pre-pandemic	Cytology (Pap)	45-49	47	Not performed	Satisfactory	NILM	NILM	Negative (NILM)	
P01790	2018	2018-Q3	2018-08	Pre-pandemic	Cytology (Pap)	35-39	38	Not performed	Satisfactory	NILM	NILM	Negative (NILM)	
P01791	2018	2018-Q3	2018-08	Pre-pandemic	Cytology (Pap)	40-44	44	Not performed	Satisfactory	NILM	NILM	Negative (NILM)	Trichomonas
P01792	2018	2018-Q3	2018-08	Pre-pandemic	Cytology (Pap)	35-39	36	Not performed	Satisfactory	NILM	NILM	Negative (NILM)	
P01793	2018	2018-Q3	2018-08	Pre-pandemic	Cytology (Pap)	40-44	43	Not performed	Satisfactory	NILM	NILM	Negative (NILM)	
P01794	2018	2018-Q3	2018-09	Pre-pandemic	Cytology (Pap)	25-29	25	Not performed	Satisfactory	NILM	NILM	Negative (NILM)	
P01795	2018	2018-Q3	2018-09	Pre-pandemic	Cytology (Pap)	60-64	63	Not performed	Satisfactory	NILM	NILM	Negative (NILM)	
P01796	2018	2018-Q3	2018-09	Pre-pandemic	Cytology (Pap)	40-44	41	Not performed	Satisfactory	NILM	NILM	Negative (NILM)	Gardnerella
P01797	2018	2018-Q3	2018-09	Pre-pandemic	Cytology (Pap)	50-54	53	Not performed	Satisfactory	Positive	HSIL	High-grade squamous	
P01798	2018	2018-Q3	2018-09	Pre-pandemic	Cytology (Pap)	30-34	30	Not performed	Satisfactory	NILM	NILM	Negative (NILM)	
P01799	2018	2018-Q3	2018-09	Pre-pandemic	Cytology (Pap)	35-39	38	Not performed	Satisfactory	NILM	NILM	Negative (NILM)	
P01800	2018	2018-Q3	2018-09	Pre-pandemic	Cytology (Pap)	40-44	44	Not performed	Satisfactory	NILM	NILM	Negative (NILM)	
P01801	2018	2018-Q3	2018-09	Pre-pandemic	Cytology (Pap)	55-59	58	Not performed	Satisfactory	NILM	NILM	Negative (NILM)	
P01802	2018	2018-Q3	2018-09	Pre-pandemic	Cytology (Pap)	55-59	56	Not performed	Satisfactory	NILM	NILM	Negative (NILM)	
P01803	2018	2018-Q3	2018-09	Pre-pandemic	Cytology (Pap)	30-34	30	Not performed	Satisfactory	NILM	NILM	Negative (NILM)	
P01804	2018	2018-Q3	2018-09	Pre-pandemic	Cytology (Pap)	60-64	60	Not performed	Satisfactory	Positive	HSIL	High-grade squamous	
P01805	2018	2018-Q3	2018-09	Pre-pandemic	Cytology (Pap)	Not recorded		Not performed	Satisfactory	NILM	NILM	Negative (NILM)	
P01806	2018	2018-Q3	2018-09	Pre-pandemic	Cytology (Pap)	35-39	39	Not performed	Satisfactory	NILM	NILM	Negative (NILM)	
P01807	2018	2018-Q3	2018-09	Pre-pandemic	Cytology (Pap)	60-64	63	Not performed	Satisfactory	NILM	NILM	Negative (NILM)	
P01808	2018	2018-Q3	2018-09	Pre-pandemic	Cytology (Pap)	45-49	49	Not performed	Satisfactory	Positive	HSIL	High-grade squamous	
P01809	2018	2018-Q3	2018-09	Pre-pandemic	Cytology (Pap)	45-49	49	Not performed	Unsatisfactory	Unsatisfactory	Unsatisfactory	Unsatisfactory	
P01810	2018	2018-Q3	2018-09	Pre-pandemic	Cytology (Pap)	40-44	44	Not performed	Satisfactory	NILM	NILM	Negative (NILM)	
P01811	2018	2018-Q3	2018-09	Pre-pandemic	Cytology (Pap)	40-44	41	Not performed	Satisfactory	Positive	AGC	Glandular	
P01812	2018	2018-Q3	2018-09	Pre-pandemic	Cytology (Pap)	Not recorded		Not performed	Satisfactory	NILM	NILM	Negative (NILM)	
P01813	2018	2018-Q3	2018-09	Pre-pandemic	Cytology (Pap)	45-49	45	Not performed	Satisfactory	NILM	NILM	Negative (NILM)	
P01814	2018	2018-Q3	2018-09	Pre-pandemic	Cytology (Pap)	50-54	50	Not performed	Satisfactory	NILM	NILM	Negative (NILM)	
P01815	2018	2018-Q3	2018-09	Pre-pandemic	Cytology (Pap)	45-49	45	Not performed	Satisfactory	NILM	NILM	Negative (NILM)	
P01816	2018	2018-Q3	2018-09	Pre-pandemic	Cytology (Pap)	50-54	52	Not performed	Satisfactory	NILM	NILM	Negative (NILM)	Gardnerella
P01817	2018	2018-Q3	2018-09	Pre-pandemic	Cytology (Pap)	25-29	28	Not performed	Satisfactory	NILM	NILM	Negative (NILM)	
P01818	2018	2018-Q3	2018-09	Pre-pandemic	Cytology (Pap)	40-44	44	Not performed	Satisfactory	NILM	NILM	Negative (NILM)	
P01819	2018	2018-Q3	2018-09	Pre-pandemic	Cytology (Pap)	50-54	51	Not performed	Satisfactory	Positive	ASC-US	Low-grade / equivocal squamous	
P01820	2018	2018-Q3	2018-09	Pre-pandemic	Cytology (Pap)	40-44	44	Not performed	Satisfactory	NILM	NILM	Negative (NILM)	Candida
P01821	2018	2018-Q3	2018-09	Pre-pandemic	Cytology (Pap)	35-39	37	Not performed	Satisfactory	NILM	NILM	Negative (NILM)	
P01822	2018	2018-Q3	2018-09	Pre-pandemic	Cytology (Pap)	35-39	36	Not performed	Satisfactory	Positive	ASC-US	Low-grade / equivocal squamous	
P01823	2018	2018-Q3	2018-09	Pre-pandemic	Cytology (Pap)	35-39	37	Not performed	Satisfactory	NILM	NILM	Negative (NILM)	
P01824	2018	2018-Q3	2018-09	Pre-pandemic	Cytology (Pap)	40-44	42	Not performed	Satisfactory	NILM	NILM	Negative (NILM)	
P01825	2018	2018-Q3	2018-09	Pre-pandemic	Cytology (Pap)	40-44	44	Not performed	Satisfactory	NILM	NILM	Negative (NILM)	
P01826	2018	2018-Q3	2018-09	Pre-pandemic	Cytology (Pap)	Not recorded		Not performed	Satisfactory	NILM	NILM	Negative (NILM)	
P01827	2018	2018-Q3	2018-09	Pre-pandemic	Cytology (Pap)	40-44	43	Not performed	Satisfactory	NILM	NILM	Negative (NILM)	
P01828	2018	2018-Q3	2018-09	Pre-pandemic	Cytology (Pap)	40-44	40	Not performed	Unsatisfactory	Unsatisfactory	Unsatisfactory	Unsatisfactory	Candida
P01829	2018	2018-Q3	2018-09	Pre-pandemic	Cytology (Pap)	40-44	40	Not performed	Satisfactory	NILM	NILM	Negative (NILM)	
P01830	2018	2018-Q3	2018-09	Pre-pandemic	Cytology (Pap)	50-54	50	Not performed	Satisfactory	NILM	NILM	Negative (NILM)	
P01831	2018	2018-Q3	2018-09	Pre-pandemic	Cytology (Pap)	45-49	48	Not performed	Satisfactory	NILM	NILM	Negative (NILM)	
P01832	2018	2018-Q3	2018-09	Pre-pandemic	Cytology (Pap)	50-54	51	Not performed	Satisfactory	Positive	LSIL	Low-grade / equivocal squamous	
P01833	2018	2018-Q3	2018-09	Pre-pandemic	Cytology (Pap)	50-54	51	Not performed	Satisfactory	Positive	HSIL	High-grade squamous	
P01834	2018	2018-Q3	2018-09	Pre-pandemic	Cytology (Pap)	60-64	64	Not performed	Satisfactory	NILM	NILM	Negative (NILM)	
P01835	2018	2018-Q3	2018-09	Pre-pandemic	Cytology (Pap)	30-34	31	Not performed	Satisfactory	NILM	NILM	Negative (NILM)	
P01836	2018	2018-Q3	2018-09	Pre-pandemic	Cytology (Pap)	40-44	40	Not performed	Satisfactory	NILM	NILM	Negative (NILM)	
P01837	2018	2018-Q3	2018-09	Pre-pandemic	Cytology (Pap)	50-54	51	Not performed	Satisfactory	NILM	NILM	Negative (NILM)	
P01838	2018	2018-Q3	2018-09	Pre-pandemic	Cytology (Pap)	30-34	34	Not performed	Satisfactory	NILM	NILM	Negative (NILM)	

patient_id	year	quarter	month	period	primary_test	age_group	age_years	hpv_result	adequacy	result	bethesda	grade	infection
P01839	2018	2018-Q3	2018-09	Pre-pandemic	Cytology (Pap)	30-34	34	Not performed	Satisfactory	NILM	NILM	Negative (NILM)	
P01840	2018	2018-Q3	2018-09	Pre-pandemic	Cytology (Pap)	60-64	64	Not performed	Satisfactory	NILM	NILM	Negative (NILM)	
P01841	2018	2018-Q3	2018-09	Pre-pandemic	Cytology (Pap)	35-39	36	Not performed	Satisfactory	NILM	NILM	Negative (NILM)	
P01842	2018	2018-Q3	2018-09	Pre-pandemic	Cytology (Pap)	40-44	42	Not performed	Satisfactory	NILM	NILM	Negative (NILM)	
P01843	2018	2018-Q3	2018-09	Pre-pandemic	Cytology (Pap)	60-64	61	Not performed	Unsatisfactory	Unsatisfactory	Unsatisfactory	Unsatisfactory	
P01844	2018	2018-Q3	2018-09	Pre-pandemic	Cytology (Pap)	25-29	27	Not performed	Satisfactory	NILM	NILM	Negative (NILM)	
P01845	2018	2018-Q3	2018-09	Pre-pandemic	Cytology (Pap)	35-39	36	Not performed	Satisfactory	NILM	NILM	Negative (NILM)	
P01846	2018	2018-Q3	2018-09	Pre-pandemic	Cytology (Pap)	45-49	45	Not performed	Satisfactory	NILM	NILM	Negative (NILM)	Gardnerella
P01847	2018	2018-Q3	2018-09	Pre-pandemic	Cytology (Pap)	55-59	57	Not performed	Satisfactory	NILM	NILM	Negative (NILM)	
P01848	2018	2018-Q3	2018-09	Pre-pandemic	Cytology (Pap)	50-54	52	Not performed	Satisfactory	Positive	ASC-US	Low-grade / equivocal squamous	
P01849	2018	2018-Q3	2018-09	Pre-pandemic	Cytology (Pap)	50-54	52	Not performed	Satisfactory	NILM	NILM	Negative (NILM)	
P01850	2018	2018-Q3	2018-09	Pre-pandemic	Cytology (Pap)	60-64	60	Not performed	Satisfactory	NILM	NILM	Negative (NILM)	
P01851	2018	2018-Q3	2018-09	Pre-pandemic	Cytology (Pap)	25-29	25	Not performed	Satisfactory	NILM	NILM	Negative (NILM)	
P01852	2018	2018-Q3	2018-09	Pre-pandemic	Cytology (Pap)	35-39	36	Not performed	Satisfactory	NILM	NILM	Negative (NILM)	
P01853	2018	2018-Q3	2018-09	Pre-pandemic	Cytology (Pap)	25-29	29	Not performed	Satisfactory	NILM	NILM	Negative (NILM)	
P01854	2018	2018-Q3	2018-09	Pre-pandemic	Cytology (Pap)	35-39	37	Not performed	Satisfactory	NILM	NILM	Negative (NILM)	Candida
P01855	2018	2018-Q3	2018-09	Pre-pandemic	Cytology (Pap)	30-34	32	Not performed	Satisfactory	NILM	NILM	Negative (NILM)	
P01856	2018	2018-Q3	2018-09	Pre-pandemic	Cytology (Pap)	30-34	33	Not performed	Satisfactory	NILM	NILM	Negative (NILM)	
P01857	2018	2018-Q3	2018-09	Pre-pandemic	Cytology (Pap)	60-64	61	Not performed	Satisfactory	NILM	NILM	Negative (NILM)	
P01858	2018	2018-Q3	2018-09	Pre-pandemic	Cytology (Pap)	25-29	27	Not performed	Satisfactory	NILM	NILM	Negative (NILM)	
P01859	2018	2018-Q3	2018-09	Pre-pandemic	Cytology (Pap)	50-54	52	Not performed	Satisfactory	NILM	NILM	Negative (NILM)	
P01860	2018	2018-Q3	2018-09	Pre-pandemic	Cytology (Pap)	25-29	28	Not performed	Satisfactory	NILM	NILM	Negative (NILM)	
P01861	2018	2018-Q3	2018-09	Pre-pandemic	Cytology (Pap)	45-49	47	Not performed	Satisfactory	NILM	NILM	Negative (NILM)	
P01862	2018	2018-Q3	2018-09	Pre-pandemic	Cytology (Pap)	35-39	39	Not performed	Satisfactory	NILM	NILM	Negative (NILM)	
P01863	2018	2018-Q3	2018-09	Pre-pandemic	Cytology (Pap)	Not recorded		Not performed	Satisfactory	NILM	NILM	Negative (NILM)	
P01864	2018	2018-Q3	2018-09	Pre-pandemic	Cytology (Pap)	45-49	45	Not performed	Satisfactory	NILM	NILM	Negative (NILM)	
P01865	2018	2018-Q3	2018-09	Pre-pandemic	Cytology (Pap)	50-54	54	Not performed	Satisfactory	NILM	NILM	Negative (NILM)	
P01866	2018	2018-Q3	2018-09	Pre-pandemic	Cytology (Pap)	55-59	58	Not performed	Satisfactory	Positive	LSIL	Low-grade / equivocal squamous	
P01867	2018	2018-Q3	2018-09	Pre-pandemic	Cytology (Pap)	50-54	50	Not performed	Satisfactory	NILM	NILM	Negative (NILM)	
P01868	2018	2018-Q3	2018-09	Pre-pandemic	Cytology (Pap)	55-59	57	Not performed	Satisfactory	NILM	NILM	Negative (NILM)	
P01869	2018	2018-Q3	2018-09	Pre-pandemic	Cytology (Pap)	45-49	49	Not performed	Satisfactory	NILM	NILM	Negative (NILM)	
P01870	2018	2018-Q3	2018-09	Pre-pandemic	Cytology (Pap)	40-44	40	Not performed	Satisfactory	NILM	NILM	Negative (NILM)	
P01871	2018	2018-Q3	2018-09	Pre-pandemic	Cytology (Pap)	50-54	53	Not performed	Satisfactory	NILM	NILM	Negative (NILM)	
P01872	2018	2018-Q3	2018-09	Pre-pandemic	Cytology (Pap)	35-39	39	Not performed	Satisfactory	Positive	ASC-US	Low-grade / equivocal squamous	
P01873	2018	2018-Q3	2018-09	Pre-pandemic	Cytology (Pap)	40-44	40	Not performed	Satisfactory	NILM	NILM	Negative (NILM)	
P01874	2018	2018-Q3	2018-09	Pre-pandemic	Cytology (Pap)	40-44	42	Not performed	Satisfactory	NILM	NILM	Negative (NILM)	
P01875	2018	2018-Q3	2018-09	Pre-pandemic	Cytology (Pap)	25-29	26	Not performed	Satisfactory	NILM	NILM	Negative (NILM)	Trichomonas
P01876	2018	2018-Q3	2018-09	Pre-pandemic	Cytology (Pap)	55-59	56	Not performed	Satisfactory	NILM	NILM	Negative (NILM)	
P01877	2018	2018-Q3	2018-09	Pre-pandemic	Cytology (Pap)	50-54	53	Not performed	Satisfactory	NILM	NILM	Negative (NILM)	
P01878	2018	2018-Q3	2018-09	Pre-pandemic	Cytology (Pap)	40-44	43	Not performed	Satisfactory	NILM	NILM	Negative (NILM)	
P01879	2018	2018-Q3	2018-09	Pre-pandemic	Cytology (Pap)	50-54	52	Not performed	Satisfactory	Positive	ASC-US	Low-grade / equivocal squamous	
P01880	2018	2018-Q3	2018-09	Pre-pandemic	Cytology (Pap)	35-39	35	Not performed	Satisfactory	NILM	NILM	Negative (NILM)	
P01881	2018	2018-Q3	2018-09	Pre-pandemic	Cytology (Pap)	25-29	28	Not performed	Satisfactory	NILM	NILM	Negative (NILM)	
P01882	2018	2018-Q3	2018-09	Pre-pandemic	Cytology (Pap)	55-59	56	Not performed	Satisfactory	NILM	NILM	Negative (NILM)	
P01883	2018	2018-Q3	2018-09	Pre-pandemic	Cytology (Pap)	40-44	43	Not performed	Satisfactory	NILM	NILM	Negative (NILM)	
P01884	2018	2018-Q3	2018-09	Pre-pandemic	Cytology (Pap)	50-54	53	Not performed	Satisfactory	Positive	ASC-US	Low-grade / equivocal squamous	
P01885	2018	2018-Q3	2018-09	Pre-pandemic	Cytology (Pap)	40-44	43	Not performed	Satisfactory	NILM	NILM	Negative (NILM)	
P01886	2018	2018-Q3	2018-09	Pre-pandemic	Cytology (Pap)	30-34	32	Not performed	Satisfactory	NILM	NILM	Negative (NILM)	
P01887	2018	2018-Q3	2018-09	Pre-pandemic	Cytology (Pap)	40-44	43	Not performed	Satisfactory	NILM	NILM	Negative (NILM)	
P01888	2018	2018-Q3	2018-09	Pre-pandemic	Cytology (Pap)	Not recorded		Not performed	Unsatisfactory	Unsatisfactory	Unsatisfactory	Unsatisfactory	
P01889	2018	2018-Q3	2018-09	Pre-pandemic	Cytology (Pap)	25-29	29	Not performed	Satisfactory	NILM	NILM	Negative (NILM)	
P01890	2018	2018-Q3	2018-09	Pre-pandemic	Cytology (Pap)	40-44	43	Not performed	Satisfactory	Positive	HSIL	High-grade squamous	
P01891	2018	2018-Q3	2018-09	Pre-pandemic	Cytology (Pap)	40-44	41	Not performed	Satisfactory	NILM	NILM	Negative (NILM)	
P01892	2018	2018-Q3	2018-09	Pre-pandemic	Cytology (Pap)	25-29	29	Not performed	Satisfactory	NILM	NILM	Negative (NILM)	
P01893	2018	2018-Q3	2018-09	Pre-pandemic	Cytology (Pap)	35-39	38	Not performed	Satisfactory	NILM	NILM	Negative (NILM)	
P01894	2018	2018-Q3	2018-09	Pre-pandemic	Cytology (Pap)	25-29	27	Not performed	Satisfactory	NILM	NILM	Negative (NILM)	
P01895	2018	2018-Q3	2018-09	Pre-pandemic	Cytology (Pap)	45-49	45	Not performed	Satisfactory	NILM	NILM	Negative (NILM)	
P01896	2018	2018-Q3	2018-09	Pre-pandemic	Cytology (Pap)	55-59	55	Not performed	Satisfactory	NILM	NILM	Negative (NILM)	
P01897	2018	2018-Q3	2018-09	Pre-pandemic	Cytology (Pap)	55-59	59	Not performed	Satisfactory	NILM	NILM	Negative (NILM)	
P01898	2018	2018-Q3	2018-09	Pre-pandemic	Cytology (Pap)	55-59	56	Not performed	Satisfactory	NILM	NILM	Negative (NILM)	
P01899	2018	2018-Q3	2018-09	Pre-pandemic	Cytology (Pap)	50-54	53	Not performed	Satisfactory	NILM	NILM	Negative (NILM)	
P01900	2018	2018-Q3	2018-09	Pre-pandemic	Cytology (Pap)	35-39	37	Not performed	Satisfactory	NILM	NILM	Negative (NILM)	
P01901	2018	2018-Q3	2018-09	Pre-pandemic	Cytology (Pap)	60-64	61	Not performed	Satisfactory	Positive	LSIL	Low-grade / equivocal squamous	
P01902	2018	2018-Q3	2018-09	Pre-pandemic	Cytology (Pap)	25-29	28	Not performed	Satisfactory	NILM	NILM	Negative (NILM)	
P01903	2018	2018-Q3	2018-09	Pre-pandemic	Cytology (Pap)	45-49	49	Not performed	Satisfactory	NILM	NILM	Negative (NILM)	
P01904	2018	2018-Q3	2018-09	Pre-pandemic	Cytology (Pap)	Not recorded		Not performed	Satisfactory	NILM	NILM	Negative (NILM)	
P01905	2018	2018-Q3	2018-09	Pre-pandemic	Cytology (Pap)	30-34	33	Not performed	Satisfactory	NILM	NILM	Negative (NILM)	
P01906	2018	2018-Q3	2018-09	Pre-pandemic	Cytology (Pap)	25-29	25	Not performed	Satisfactory	NILM	NILM	Negative (NILM)	
P01907	2018	2018-Q3	2018-09	Pre-pandemic	Cytology (Pap)	55-59	55	Not performed	Satisfactory	NILM	NILM	Negative (NILM)	
P01908	2018	2018-Q3	2018-09	Pre-pandemic	Cytology (Pap)	40-44	44	Not performed	Satisfactory	NILM	NILM	Negative (NILM)	
P01909	2018	2018-Q3	2018-09	Pre-pandemic	Cytology (Pap)	25-29	27	Not performed	Satisfactory	NILM	NILM	Negative (NILM)	
P01910	2018	2018-Q3	2018-09	Pre-pandemic	Cytology (Pap)	40-44	40	Not performed	Satisfactory	NILM	NILM	Negative (NILM)	
P01911	2018	2018-Q3	2018-09	Pre-pandemic	Cytology (Pap)	40-44	40	Not performed	Satisfactory	NILM	NILM	Negative (NILM)	
P01912	2018	2018-Q3	2018-09	Pre-pandemic	Cytology (Pap)	40-44	40	Not performed	Satisfactory	NILM	NILM	Negative (NILM)	
P01913	2018	2018-Q3	2018-09	Pre-pandemic	Cytology (Pap)	40-44	44	Not performed	Satisfactory	NILM	NILM	Negative (NILM)	
P01914	2018	2018-Q3	2018-09	Pre-pandemic	Cytology (Pap)	55-59	55	Not performed	Satisfactory	NILM	NILM	Negative (NILM)	
P01915	2018	2018-Q3	2018-09	Pre-pandemic	Cytology (Pap)	30-34	30	Not performed	Satisfactory	NILM	NILM	Negative (NILM)	
P01916	2018	2018-Q3	2018-09	Pre-pandemic	Cytology (Pap)	55-59	58	Not performed	Satisfactory	NILM	NILM	Negative (NILM)	
P01917	2018	2018-Q3	2018-09	Pre-pandemic	Cytology (Pap)	25-29	28	Not performed	Satisfactory	NILM	NILM	Negative (NILM)	
P01918	2018	2018-Q3	2018-09	Pre-pandemic	Cytology (Pap)	40-44	42	Not performed	Satisfactory	NILM	NILM	Negative (NILM)	

patient_id	year	quarter	month	period	primary_test	age_group	age_years	hpv_result	adequacy	result	bethesda	grade	infection
P01919	2018	2018-Q3	2018-09	Pre-pandemic	Cytology (Pap)	40-44	44	Not performed	Satisfactory	NILM	NILM	Negative (NILM)	
P01920	2018	2018-Q3	2018-09	Pre-pandemic	Cytology (Pap)	35-39	37	Not performed	Satisfactory	NILM	NILM	Negative (NILM)	
P01921	2018	2018-Q3	2018-09	Pre-pandemic	Cytology (Pap)	40-44	43	Not performed	Satisfactory	Positive	HSIL	High-grade squamous	
P01922	2018	2018-Q3	2018-09	Pre-pandemic	Cytology (Pap)	45-49	45	Not performed	Satisfactory	NILM	NILM	Negative (NILM)	
P01923	2018	2018-Q3	2018-09	Pre-pandemic	Cytology (Pap)	40-44	44	Not performed	Satisfactory	NILM	NILM	Negative (NILM)	
P01924	2018	2018-Q3	2018-09	Pre-pandemic	Cytology (Pap)	35-39	36	Not performed	Unsatisfactory	Unsatisfactory	Unsatisfactory	Unsatisfactory	
P01925	2018	2018-Q3	2018-09	Pre-pandemic	Cytology (Pap)	55-59	58	Not performed	Satisfactory	NILM	NILM	Negative (NILM)	
P01926	2018	2018-Q3	2018-09	Pre-pandemic	Cytology (Pap)	25-29	25	Not performed	Satisfactory	NILM	NILM	Negative (NILM)	Trichomonas
P01927	2018	2018-Q3	2018-09	Pre-pandemic	Cytology (Pap)	25-29	28	Not performed	Satisfactory	NILM	NILM	Negative (NILM)	
P01928	2018	2018-Q3	2018-09	Pre-pandemic	Cytology (Pap)	35-39	38	Not performed	Satisfactory	Positive	LSIL	Low-grade / equivocal squamous	
P01929	2018	2018-Q3	2018-09	Pre-pandemic	Cytology (Pap)	55-59	56	Not performed	Satisfactory	NILM	NILM	Negative (NILM)	
P01930	2018	2018-Q3	2018-09	Pre-pandemic	Cytology (Pap)	45-49	46	Not performed	Satisfactory	NILM	NILM	Negative (NILM)	
P01931	2018	2018-Q3	2018-09	Pre-pandemic	Cytology (Pap)	50-54	50	Not performed	Satisfactory	NILM	NILM	Negative (NILM)	
P01932	2018	2018-Q3	2018-09	Pre-pandemic	Cytology (Pap)	Not recorded		Not performed	Satisfactory	NILM	NILM	Negative (NILM)	
P01933	2018	2018-Q3	2018-09	Pre-pandemic	Cytology (Pap)	35-39	38	Not performed	Satisfactory	NILM	NILM	Negative (NILM)	
P01934	2018	2018-Q3	2018-09	Pre-pandemic	Cytology (Pap)	45-49	45	Not performed	Satisfactory	NILM	NILM	Negative (NILM)	
P01935	2018	2018-Q3	2018-09	Pre-pandemic	Cytology (Pap)	25-29	27	Not performed	Satisfactory	NILM	NILM	Negative (NILM)	
P01936	2018	2018-Q3	2018-09	Pre-pandemic	Cytology (Pap)	45-49	45	Not performed	Satisfactory	NILM	NILM	Negative (NILM)	
P01937	2018	2018-Q3	2018-09	Pre-pandemic	Cytology (Pap)	35-39	35	Not performed	Satisfactory	NILM	NILM	Negative (NILM)	
P01938	2018	2018-Q3	2018-09	Pre-pandemic	Cytology (Pap)	45-49	45	Not performed	Satisfactory	NILM	NILM	Negative (NILM)	
P01939	2018	2018-Q3	2018-09	Pre-pandemic	Cytology (Pap)	40-44	43	Not performed	Satisfactory	NILM	NILM	Negative (NILM)	
P01940	2018	2018-Q3	2018-09	Pre-pandemic	Cytology (Pap)	60-64	61	Not performed	Satisfactory	Positive	HSIL	High-grade squamous	
P01941	2018	2018-Q3	2018-09	Pre-pandemic	Cytology (Pap)	40-44	40	Not performed	Satisfactory	Positive	ASC-US	Low-grade / equivocal squamous	
P01942	2018	2018-Q3	2018-09	Pre-pandemic	Cytology (Pap)	35-39	38	Not performed	Satisfactory	NILM	NILM	Negative (NILM)	
P01943	2018	2018-Q3	2018-09	Pre-pandemic	Cytology (Pap)	25-29	29	Not performed	Satisfactory	NILM	NILM	Negative (NILM)	
P01944	2018	2018-Q3	2018-09	Pre-pandemic	Cytology (Pap)	30-34	30	Not performed	Satisfactory	NILM	NILM	Negative (NILM)	
P01945	2018	2018-Q3	2018-09	Pre-pandemic	Cytology (Pap)	60-64	62	Not performed	Satisfactory	NILM	NILM	Negative (NILM)	
P01946	2018	2018-Q3	2018-09	Pre-pandemic	Cytology (Pap)	30-34	31	Not performed	Satisfactory	NILM	NILM	Negative (NILM)	
P01947	2018	2018-Q3	2018-09	Pre-pandemic	Cytology (Pap)	55-59	56	Not performed	Satisfactory	NILM	NILM	Negative (NILM)	
P01948	2018	2018-Q3	2018-09	Pre-pandemic	Cytology (Pap)	55-59	57	Not performed	Satisfactory	NILM	NILM	Negative (NILM)	
P01949	2018	2018-Q3	2018-09	Pre-pandemic	Cytology (Pap)	30-34	34	Not performed	Satisfactory	NILM	NILM	Negative (NILM)	
P01950	2018	2018-Q3	2018-09	Pre-pandemic	Cytology (Pap)	40-44	42	Not performed	Satisfactory	Positive	ASC-US	Low-grade / equivocal squamous	
P01951	2018	2018-Q3	2018-09	Pre-pandemic	Cytology (Pap)	30-34	34	Not performed	Unsatisfactory	Unsatisfactory	Unsatisfactory	Unsatisfactory	
P01952	2018	2018-Q3	2018-09	Pre-pandemic	Cytology (Pap)	Not recorded		Not performed	Satisfactory	NILM	NILM	Negative (NILM)	
P01953	2018	2018-Q3	2018-09	Pre-pandemic	Cytology (Pap)	45-49	45	Not performed	Satisfactory	NILM	NILM	Negative (NILM)	
P01954	2018	2018-Q3	2018-09	Pre-pandemic	Cytology (Pap)	30-34	34	Not performed	Satisfactory	NILM	NILM	Negative (NILM)	
P01955	2018	2018-Q3	2018-09	Pre-pandemic	Cytology (Pap)	55-59	57	Not performed	Satisfactory	NILM	NILM	Negative (NILM)	
P01956	2018	2018-Q3	2018-09	Pre-pandemic	Cytology (Pap)	60-64	60	Not performed	Satisfactory	NILM	NILM	Negative (NILM)	
P01957	2018	2018-Q3	2018-09	Pre-pandemic	Cytology (Pap)	40-44	40	Not performed	Unsatisfactory	Unsatisfactory	Unsatisfactory	Unsatisfactory	
P01958	2018	2018-Q3	2018-09	Pre-pandemic	Cytology (Pap)	35-39	37	Not performed	Satisfactory	NILM	NILM	Negative (NILM)	
P01959	2018	2018-Q3	2018-09	Pre-pandemic	Cytology (Pap)	40-44	41	Not performed	Satisfactory	NILM	NILM	Negative (NILM)	
P01960	2018	2018-Q3	2018-09	Pre-pandemic	Cytology (Pap)	40-44	42	Not performed	Satisfactory	NILM	NILM	Negative (NILM)	
P01961	2018	2018-Q3	2018-09	Pre-pandemic	Cytology (Pap)	35-39	39	Not performed	Satisfactory	NILM	NILM	Negative (NILM)	
P01962	2018	2018-Q3	2018-09	Pre-pandemic	Cytology (Pap)	25-29	25	Not performed	Satisfactory	NILM	NILM	Negative (NILM)	Candida
P01963	2018	2018-Q3	2018-09	Pre-pandemic	Cytology (Pap)	45-49	48	Not performed	Satisfactory	NILM	NILM	Negative (NILM)	
P01964	2018	2018-Q3	2018-09	Pre-pandemic	Cytology (Pap)	35-39	38	Not performed	Satisfactory	NILM	NILM	Negative (NILM)	
P01965	2018	2018-Q3	2018-09	Pre-pandemic	Cytology (Pap)	55-59	59	Not performed	Satisfactory	Positive	LSIL	Low-grade / equivocal squamous	
P01966	2018	2018-Q3	2018-09	Pre-pandemic	Cytology (Pap)	55-59	59	Not performed	Satisfactory	NILM	NILM	Negative (NILM)	
P01967	2018	2018-Q3	2018-09	Pre-pandemic	Cytology (Pap)	25-29	26	Not performed	Satisfactory	Positive	ASC-US	Low-grade / equivocal squamous	
P01968	2018	2018-Q3	2018-09	Pre-pandemic	Cytology (Pap)	30-34	33	Not performed	Satisfactory	NILM	NILM	Negative (NILM)	
P01969	2018	2018-Q3	2018-09	Pre-pandemic	Cytology (Pap)	30-34	30	Not performed	Satisfactory	NILM	NILM	Negative (NILM)	
P01970	2018	2018-Q3	2018-09	Pre-pandemic	Cytology (Pap)	40-44	40	Not performed	Satisfactory	Positive	LSIL	Low-grade / equivocal squamous	
P01971	2018	2018-Q3	2018-09	Pre-pandemic	Cytology (Pap)	40-44	40	Not performed	Satisfactory	NILM	NILM	Negative (NILM)	
P01972	2018	2018-Q3	2018-09	Pre-pandemic	Cytology (Pap)	40-44	43	Not performed	Satisfactory	NILM	NILM	Negative (NILM)	
P01973	2018	2018-Q3	2018-09	Pre-pandemic	Cytology (Pap)	40-44	42	Not performed	Satisfactory	NILM	NILM	Negative (NILM)	
P01974	2018	2018-Q3	2018-09	Pre-pandemic	Cytology (Pap)	35-39	36	Not performed	Satisfactory	Positive	ASC-US	Low-grade / equivocal squamous	
P01975	2018	2018-Q3	2018-09	Pre-pandemic	Cytology (Pap)	50-54	50	Not performed	Satisfactory	NILM	NILM	Negative (NILM)	
P01976	2018	2018-Q3	2018-09	Pre-pandemic	Cytology (Pap)	40-44	43	Not performed	Satisfactory	NILM	NILM	Negative (NILM)	
P01977	2018	2018-Q3	2018-09	Pre-pandemic	Cytology (Pap)	25-29	28	Not performed	Unsatisfactory	Unsatisfactory	Unsatisfactory	Unsatisfactory	
P01978	2018	2018-Q3	2018-09	Pre-pandemic	Cytology (Pap)	35-39	36	Not performed	Satisfactory	Positive	ASC-H	High-grade squamous	
P01979	2018	2018-Q3	2018-09	Pre-pandemic	Cytology (Pap)	35-39	37	Not performed	Satisfactory	Positive	LSIL	Low-grade / equivocal squamous	
P01980	2018	2018-Q3	2018-09	Pre-pandemic	Cytology (Pap)	60-64	62	Not performed	Satisfactory	NILM	NILM	Negative (NILM)	
P01981	2018	2018-Q3	2018-09	Pre-pandemic	Cytology (Pap)	40-44	40	Not performed	Satisfactory	NILM	NILM	Negative (NILM)	
P01982	2018	2018-Q3	2018-09	Pre-pandemic	Cytology (Pap)	35-39	36	Not performed	Satisfactory	NILM	NILM	Negative (NILM)	
P01983	2018	2018-Q3	2018-09	Pre-pandemic	Cytology (Pap)	45-49	47	Not performed	Satisfactory	NILM	NILM	Negative (NILM)	
P01984	2018	2018-Q3	2018-09	Pre-pandemic	Cytology (Pap)	35-39	36	Not performed	Satisfactory	NILM	NILM	Negative (NILM)	
P01985	2018	2018-Q3	2018-09	Pre-pandemic	Cytology (Pap)	40-44	44	Not performed	Satisfactory	NILM	NILM	Negative (NILM)	
P01986	2018	2018-Q3	2018-09	Pre-pandemic	Cytology (Pap)	45-49	48	Not performed	Satisfactory	NILM	NILM	Negative (NILM)	
P01987	2018	2018-Q3	2018-09	Pre-pandemic	Cytology (Pap)	40-44	40	Not performed	Satisfactory	NILM	NILM	Negative (NILM)	
P01988	2018	2018-Q3	2018-09	Pre-pandemic	Cytology (Pap)	35-39	35	Not performed	Satisfactory	NILM	NILM	Negative (NILM)	
P01989	2018	2018-Q3	2018-09	Pre-pandemic	Cytology (Pap)	60-64	64	Not performed	Satisfactory	NILM	NILM	Negative (NILM)	
P01990	2018	2018-Q3	2018-09	Pre-pandemic	Cytology (Pap)	25-29	29	Not performed	Satisfactory	NILM	NILM	Negative (NILM)	
P01991	2018	2018-Q3	2018-09	Pre-pandemic	Cytology (Pap)	35-39	35	Not performed	Unsatisfactory	Unsatisfactory	Unsatisfactory	Unsatisfactory	
P01992	2018	2018-Q3	2018-09	Pre-pandemic	Cytology (Pap)	30-34	34	Not performed	Satisfactory	NILM	NILM	Negative (NILM)	
P01993	2018	2018-Q3	2018-09	Pre-pandemic	Cytology (Pap)	45-49	49	Not performed	Satisfactory	Positive	ASC-US	Low-grade / equivocal squamous	
P01994	2018	2018-Q3	2018-09	Pre-pandemic	Cytology (Pap)	40-44	42	Not performed	Satisfactory	NILM	NILM	Negative (NILM)	
P01995	2018	2018-Q3	2018-09	Pre-pandemic	Cytology (Pap)	30-34	34	Not performed	Satisfactory	Positive	ASC-US	Low-grade / equivocal squamous	
P01996	2018	2018-Q3	2018-09	Pre-pandemic	Cytology (Pap)	25-29	29	Not performed	Satisfactory	NILM	NILM	Negative (NILM)	
P01997	2018	2018-Q3	2018-09	Pre-pandemic	Cytology (Pap)	55-59	55	Not performed	Satisfactory	NILM	NILM	Negative (NILM)	
P01998	2018	2018-Q3	2018-09	Pre-pandemic	Cytology (Pap)	40-44	40	Not performed	Satisfactory	NILM	NILM	Negative (NILM)	Other

patient_id	year	quarter	month	period	primary_test	age_group	age_years	hpv_result	adequacy	result	bethesda	grade	infection
P01999	2018	2018-Q3	2018-09	Pre-pandemic	Cytology (Pap)	60-64	61	Not performed	Satisfactory	NILM	NILM	Negative (NILM)	
P02000	2018	2018-Q3	2018-09	Pre-pandemic	Cytology (Pap)	35-39	37	Not performed	Satisfactory	NILM	NILM	Negative (NILM)	
P02001	2018	2018-Q3	2018-09	Pre-pandemic	Cytology (Pap)	40-44	42	Not performed	Satisfactory	NILM	NILM	Negative (NILM)	
P02002	2018	2018-Q3	2018-09	Pre-pandemic	Cytology (Pap)	30-34	32	Not performed	Satisfactory	NILM	NILM	Negative (NILM)	
P02003	2018	2018-Q3	2018-09	Pre-pandemic	Cytology (Pap)	25-29	26	Not performed	Satisfactory	NILM	NILM	Negative (NILM)	
P02004	2018	2018-Q3	2018-09	Pre-pandemic	Cytology (Pap)	40-44	42	Not performed	Satisfactory	NILM	NILM	Negative (NILM)	
P02005	2018	2018-Q3	2018-09	Pre-pandemic	Cytology (Pap)	35-39	35	Not performed	Satisfactory	NILM	NILM	Negative (NILM)	
P02006	2018	2018-Q3	2018-09	Pre-pandemic	Cytology (Pap)	30-34	30	Not performed	Satisfactory	Positive	LSIL	Low-grade / equivocal squamous	
P02007	2018	2018-Q3	2018-09	Pre-pandemic	Cytology (Pap)	50-54	51	Not performed	Satisfactory	Positive	ASC-US	Low-grade / equivocal squamous	
P02008	2018	2018-Q3	2018-09	Pre-pandemic	Cytology (Pap)	40-44	43	Not performed	Satisfactory	NILM	NILM	Negative (NILM)	
P02009	2018	2018-Q3	2018-09	Pre-pandemic	Cytology (Pap)	40-44	42	Not performed	Satisfactory	NILM	NILM	Negative (NILM)	
P02010	2018	2018-Q3	2018-09	Pre-pandemic	Cytology (Pap)	60-64	61	Not performed	Satisfactory	NILM	NILM	Negative (NILM)	
P02011	2018	2018-Q3	2018-09	Pre-pandemic	Cytology (Pap)	40-44	44	Not performed	Satisfactory	NILM	NILM	Negative (NILM)	
P02012	2018	2018-Q3	2018-09	Pre-pandemic	Cytology (Pap)	Not recorded		Not performed	Satisfactory	NILM	NILM	Negative (NILM)	
P02013	2018	2018-Q3	2018-09	Pre-pandemic	Cytology (Pap)	60-64	61	Not performed	Satisfactory	NILM	NILM	Negative (NILM)	
P02014	2018	2018-Q3	2018-09	Pre-pandemic	Cytology (Pap)	30-34	32	Not performed	Satisfactory	NILM	NILM	Negative (NILM)	
P02015	2018	2018-Q3	2018-09	Pre-pandemic	Cytology (Pap)	60-64	63	Not performed	Satisfactory	NILM	NILM	Negative (NILM)	
P02016	2018	2018-Q3	2018-09	Pre-pandemic	Cytology (Pap)	55-59	59	Not performed	Satisfactory	Positive	ASC-US	Low-grade / equivocal squamous	Candida
P02017	2018	2018-Q3	2018-09	Pre-pandemic	Cytology (Pap)	45-49	45	Not performed	Satisfactory	NILM	NILM	Negative (NILM)	
P02018	2018	2018-Q3	2018-09	Pre-pandemic	Cytology (Pap)	55-59	56	Not performed	Satisfactory	NILM	NILM	Negative (NILM)	
P02019	2018	2018-Q3	2018-09	Pre-pandemic	Cytology (Pap)	45-49	49	Not performed	Satisfactory	NILM	NILM	Negative (NILM)	
P02020	2018	2018-Q3	2018-09	Pre-pandemic	Cytology (Pap)	60-64	61	Not performed	Satisfactory	NILM	NILM	Negative (NILM)	
P02021	2018	2018-Q3	2018-09	Pre-pandemic	Cytology (Pap)	55-59	58	Not performed	Satisfactory	NILM	NILM	Negative (NILM)	Trichomonas
P02022	2018	2018-Q3	2018-09	Pre-pandemic	Cytology (Pap)	Not recorded		Not performed	Satisfactory	NILM	NILM	Negative (NILM)	
P02023	2018	2018-Q3	2018-09	Pre-pandemic	Cytology (Pap)	25-29	27	Not performed	Satisfactory	NILM	NILM	Negative (NILM)	
P02024	2018	2018-Q3	2018-09	Pre-pandemic	Cytology (Pap)	55-59	57	Not performed	Satisfactory	NILM	NILM	Negative (NILM)	
P02025	2018	2018-Q3	2018-09	Pre-pandemic	Cytology (Pap)	30-34	30	Not performed	Satisfactory	NILM	NILM	Negative (NILM)	
P02026	2018	2018-Q3	2018-09	Pre-pandemic	Cytology (Pap)	55-59	58	Not performed	Satisfactory	NILM	NILM	Negative (NILM)	
P02027	2018	2018-Q3	2018-09	Pre-pandemic	Cytology (Pap)	35-39	39	Not performed	Satisfactory	NILM	NILM	Negative (NILM)	
P02028	2018	2018-Q3	2018-09	Pre-pandemic	Cytology (Pap)	40-44	41	Not performed	Satisfactory	NILM	NILM	Negative (NILM)	
P02029	2018	2018-Q3	2018-09	Pre-pandemic	Cytology (Pap)	45-49	49	Not performed	Satisfactory	NILM	NILM	Negative (NILM)	
P02030	2018	2018-Q3	2018-09	Pre-pandemic	Cytology (Pap)	25-29	28	Not performed	Satisfactory	NILM	NILM	Negative (NILM)	
P02031	2018	2018-Q3	2018-09	Pre-pandemic	Cytology (Pap)	45-49	48	Not performed	Satisfactory	NILM	NILM	Negative (NILM)	
P02032	2018	2018-Q3	2018-09	Pre-pandemic	Cytology (Pap)	25-29	28	Not performed	Satisfactory	NILM	NILM	Negative (NILM)	
P02033	2018	2018-Q3	2018-09	Pre-pandemic	Cytology (Pap)	50-54	51	Not performed	Satisfactory	NILM	NILM	Negative (NILM)	
P02034	2018	2018-Q3	2018-09	Pre-pandemic	Cytology (Pap)	50-54	50	Not performed	Unsatisfactory	Unsatisfactory	Unsatisfactory	Unsatisfactory	
P02035	2018	2018-Q3	2018-09	Pre-pandemic	Cytology (Pap)	45-49	46	Not performed	Satisfactory	NILM	NILM	Negative (NILM)	
P02036	2018	2018-Q3	2018-09	Pre-pandemic	Cytology (Pap)	30-34	32	Not performed	Satisfactory	NILM	NILM	Negative (NILM)	
P02037	2018	2018-Q3	2018-09	Pre-pandemic	Cytology (Pap)	45-49	48	Not performed	Satisfactory	Positive	LSIL	Low-grade / equivocal squamous	
P02038	2018	2018-Q3	2018-09	Pre-pandemic	Cytology (Pap)	50-54	54	Not performed	Satisfactory	NILM	NILM	Negative (NILM)	
P02039	2018	2018-Q3	2018-09	Pre-pandemic	Cytology (Pap)	30-34	33	Not performed	Satisfactory	NILM	NILM	Negative (NILM)	
P02040	2018	2018-Q3	2018-09	Pre-pandemic	Cytology (Pap)	60-64	64	Not performed	Satisfactory	NILM	NILM	Negative (NILM)	
P02041	2018	2018-Q3	2018-09	Pre-pandemic	Cytology (Pap)	40-44	44	Not performed	Satisfactory	Positive	ASC-US	Low-grade / equivocal squamous	
P02042	2018	2018-Q3	2018-09	Pre-pandemic	Cytology (Pap)	35-39	35	Not performed	Satisfactory	NILM	NILM	Negative (NILM)	
P02043	2018	2018-Q3	2018-09	Pre-pandemic	Cytology (Pap)	40-44	41	Not performed	Satisfactory	NILM	NILM	Negative (NILM)	Candida
P02044	2018	2018-Q3	2018-09	Pre-pandemic	Cytology (Pap)	35-39	37	Not performed	Satisfactory	NILM	NILM	Negative (NILM)	
P02045	2018	2018-Q3	2018-09	Pre-pandemic	Cytology (Pap)	35-39	38	Not performed	Satisfactory	NILM	NILM	Negative (NILM)	
P02046	2018	2018-Q3	2018-09	Pre-pandemic	Cytology (Pap)	50-54	50	Not performed	Satisfactory	NILM	NILM	Negative (NILM)	
P02047	2018	2018-Q3	2018-09	Pre-pandemic	Cytology (Pap)	35-39	35	Not performed	Satisfactory	NILM	NILM	Negative (NILM)	
P02048	2018	2018-Q3	2018-09	Pre-pandemic	Cytology (Pap)	40-44	42	Not performed	Satisfactory	NILM	NILM	Negative (NILM)	
P02049	2018	2018-Q3	2018-09	Pre-pandemic	Cytology (Pap)	45-49	46	Not performed	Satisfactory	NILM	NILM	Negative (NILM)	
P02050	2018	2018-Q3	2018-09	Pre-pandemic	Cytology (Pap)	25-29	26	Not performed	Satisfactory	NILM	NILM	Negative (NILM)	
P02051	2018	2018-Q3	2018-09	Pre-pandemic	Cytology (Pap)	50-54	54	Not performed	Satisfactory	NILM	NILM	Negative (NILM)	
P02052	2018	2018-Q3	2018-09	Pre-pandemic	Cytology (Pap)	40-44	43	Not performed	Satisfactory	NILM	NILM	Negative (NILM)	
P02053	2018	2018-Q3	2018-09	Pre-pandemic	Cytology (Pap)	Not recorded		Not performed	Satisfactory	Positive	AGC	Glandular	
P02054	2018	2018-Q3	2018-09	Pre-pandemic	Cytology (Pap)	35-39	39	Not performed	Satisfactory	NILM	NILM	Negative (NILM)	
P02055	2018	2018-Q3	2018-09	Pre-pandemic	Cytology (Pap)	45-49	49	Not performed	Satisfactory	NILM	NILM	Negative (NILM)	
P02056	2018	2018-Q3	2018-09	Pre-pandemic	Cytology (Pap)	55-59	58	Not performed	Satisfactory	NILM	NILM	Negative (NILM)	
P02057	2018	2018-Q3	2018-09	Pre-pandemic	Cytology (Pap)	45-49	49	Not performed	Satisfactory	NILM	NILM	Negative (NILM)	
P02058	2018	2018-Q3	2018-09	Pre-pandemic	Cytology (Pap)	30-34	33	Not performed	Unsatisfactory	Unsatisfactory	Unsatisfactory	Unsatisfactory	
P02059	2018	2018-Q3	2018-09	Pre-pandemic	Cytology (Pap)	55-59	55	Not performed	Unsatisfactory	Unsatisfactory	Unsatisfactory	Unsatisfactory	
P02060	2018	2018-Q3	2018-09	Pre-pandemic	Cytology (Pap)	30-34	34	Not performed	Satisfactory	NILM	NILM	Negative (NILM)	
P02061	2018	2018-Q3	2018-09	Pre-pandemic	Cytology (Pap)	35-39	39	Not performed	Satisfactory	NILM	NILM	Negative (NILM)	
P02062	2018	2018-Q3	2018-09	Pre-pandemic	Cytology (Pap)	40-44	42	Not performed	Satisfactory	NILM	NILM	Negative (NILM)	
P02063	2018	2018-Q3	2018-09	Pre-pandemic	Cytology (Pap)	40-44	41	Not performed	Satisfactory	NILM	NILM	Negative (NILM)	
P02064	2018	2018-Q3	2018-09	Pre-pandemic	Cytology (Pap)	40-44	40	Not performed	Unsatisfactory	Unsatisfactory	Unsatisfactory	Unsatisfactory	
P02065	2018	2018-Q3	2018-09	Pre-pandemic	Cytology (Pap)	40-44	40	Not performed	Satisfactory	NILM	NILM	Negative (NILM)	
P02066	2018	2018-Q3	2018-09	Pre-pandemic	Cytology (Pap)	Not recorded		Not performed	Satisfactory	NILM	NILM	Negative (NILM)	
P02067	2018	2018-Q3	2018-09	Pre-pandemic	Cytology (Pap)	40-44	41	Not performed	Satisfactory	NILM	NILM	Negative (NILM)	
P02068	2018	2018-Q3	2018-09	Pre-pandemic	Cytology (Pap)	40-44	40	Not performed	Satisfactory	NILM	NILM	Negative (NILM)	
P02069	2018	2018-Q3	2018-09	Pre-pandemic	Cytology (Pap)	35-39	39	Not performed	Satisfactory	Positive	LSIL	Low-grade / equivocal squamous	
P02070	2018	2018-Q3	2018-09	Pre-pandemic	Cytology (Pap)	45-49	47	Not performed	Satisfactory	NILM	NILM	Negative (NILM)	
P02071	2018	2018-Q3	2018-09	Pre-pandemic	Cytology (Pap)	40-44	40	Not performed	Satisfactory	NILM	NILM	Negative (NILM)	
P02072	2018	2018-Q3	2018-09	Pre-pandemic	Cytology (Pap)	35-39	37	Not performed	Satisfactory	NILM	NILM	Negative (NILM)	
P02073	2018	2018-Q3	2018-09	Pre-pandemic	Cytology (Pap)	30-34	31	Not performed	Satisfactory	NILM	NILM	Negative (NILM)	
P02074	2018	2018-Q3	2018-09	Pre-pandemic	Cytology (Pap)	50-54	52	Not performed	Satisfactory	Positive	HSIL	High-grade squamous	
P02075	2018	2018-Q3	2018-09	Pre-pandemic	Cytology (Pap)	40-44	43	Not performed	Satisfactory	NILM	NILM	Negative (NILM)	
P02076	2018	2018-Q3	2018-09	Pre-pandemic	Cytology (Pap)	25-29	26	Not performed	Satisfactory	NILM	NILM	Negative (NILM)	
P02077	2018	2018-Q3	2018-09	Pre-pandemic	Cytology (Pap)	35-39	38	Not performed	Satisfactory	Positive	HSIL	High-grade squamous	
P02078	2018	2018-Q3	2018-09	Pre-pandemic	Cytology (Pap)	35-39	36	Not performed	Satisfactory	NILM	NILM	Negative (NILM)	

patient_id	year	quarter	month	period	primary_test	age_group	age_years	hpv_result	adequacy	result	bethesda	grade	infection
P02079	2018	2018-Q3	2018-09	Pre-pandemic	Cytology (Pap)	35-39	35	Not performed	Satisfactory	NILM	NILM	Negative (NILM)	
P02080	2018	2018-Q3	2018-09	Pre-pandemic	Cytology (Pap)	30-34	31	Not performed	Satisfactory	NILM	NILM	Negative (NILM)	
P02081	2018	2018-Q3	2018-09	Pre-pandemic	Cytology (Pap)	35-39	38	Not performed	Satisfactory	NILM	NILM	Negative (NILM)	
P02082	2018	2018-Q3	2018-09	Pre-pandemic	Cytology (Pap)	45-49	45	Not performed	Satisfactory	Positive	ASC-US	Low-grade / equivocal squamous	
P02083	2018	2018-Q3	2018-09	Pre-pandemic	Cytology (Pap)	40-44	41	Not performed	Satisfactory	NILM	NILM	Negative (NILM)	
P02084	2018	2018-Q3	2018-09	Pre-pandemic	Cytology (Pap)	40-44	43	Not performed	Satisfactory	NILM	NILM	Negative (NILM)	
P02085	2018	2018-Q3	2018-09	Pre-pandemic	Cytology (Pap)	35-39	37	Not performed	Satisfactory	NILM	NILM	Negative (NILM)	
P02086	2018	2018-Q3	2018-09	Pre-pandemic	Cytology (Pap)	25-29	26	Not performed	Satisfactory	NILM	NILM	Negative (NILM)	
P02087	2018	2018-Q4	2018-10	Pre-pandemic	Cytology (Pap)	40-44	43	Not performed	Satisfactory	NILM	NILM	Negative (NILM)	
P02088	2018	2018-Q4	2018-10	Pre-pandemic	Cytology (Pap)	40-44	42	Not performed	Satisfactory	NILM	NILM	Negative (NILM)	
P02089	2018	2018-Q4	2018-10	Pre-pandemic	Cytology (Pap)	25-29	29	Not performed	Satisfactory	Positive	ASC-US	Low-grade / equivocal squamous	
P02090	2018	2018-Q4	2018-10	Pre-pandemic	Cytology (Pap)	40-44	43	Not performed	Satisfactory	NILM	NILM	Negative (NILM)	
P02091	2018	2018-Q4	2018-10	Pre-pandemic	Cytology (Pap)	60-64	61	Not performed	Satisfactory	NILM	NILM	Negative (NILM)	
P02092	2018	2018-Q4	2018-10	Pre-pandemic	Cytology (Pap)	45-49	46	Not performed	Satisfactory	Positive	ASC-US	Low-grade / equivocal squamous	
P02093	2018	2018-Q4	2018-10	Pre-pandemic	Cytology (Pap)	45-49	46	Not performed	Satisfactory	NILM	NILM	Negative (NILM)	
P02094	2018	2018-Q4	2018-10	Pre-pandemic	Cytology (Pap)	25-29	25	Not performed	Satisfactory	NILM	NILM	Negative (NILM)	
P02095	2018	2018-Q4	2018-10	Pre-pandemic	Cytology (Pap)	45-49	47	Not performed	Satisfactory	NILM	NILM	Negative (NILM)	
P02096	2018	2018-Q4	2018-10	Pre-pandemic	Cytology (Pap)	35-39	38	Not performed	Satisfactory	Positive	HSIL	High-grade squamous	
P02097	2018	2018-Q4	2018-10	Pre-pandemic	Cytology (Pap)	35-39	37	Not performed	Satisfactory	NILM	NILM	Negative (NILM)	
P02098	2018	2018-Q4	2018-10	Pre-pandemic	Cytology (Pap)	45-49	45	Not performed	Satisfactory	NILM	NILM	Negative (NILM)	
P02099	2018	2018-Q4	2018-10	Pre-pandemic	Cytology (Pap)	55-59	59	Not performed	Unsatisfactory	Unsatisfactory	Unsatisfactory	Unsatisfactory	
P02100	2018	2018-Q4	2018-10	Pre-pandemic	Cytology (Pap)	50-54	51	Not performed	Satisfactory	NILM	NILM	Negative (NILM)	
P02101	2018	2018-Q4	2018-10	Pre-pandemic	Cytology (Pap)	35-39	37	Not performed	Satisfactory	NILM	NILM	Negative (NILM)	
P02102	2018	2018-Q4	2018-10	Pre-pandemic	Cytology (Pap)	35-39	35	Not performed	Satisfactory	NILM	NILM	Negative (NILM)	
P02103	2018	2018-Q4	2018-10	Pre-pandemic	Cytology (Pap)	40-44	44	Not performed	Satisfactory	NILM	NILM	Negative (NILM)	
P02104	2018	2018-Q4	2018-10	Pre-pandemic	Cytology (Pap)	60-64	61	Not performed	Satisfactory	NILM	NILM	Negative (NILM)	
P02105	2018	2018-Q4	2018-10	Pre-pandemic	Cytology (Pap)	35-39	38	Not performed	Satisfactory	Positive	LSIL	Low-grade / equivocal squamous	
P02106	2018	2018-Q4	2018-10	Pre-pandemic	Cytology (Pap)	60-64	61	Not performed	Satisfactory	NILM	NILM	Negative (NILM)	
P02107	2018	2018-Q4	2018-10	Pre-pandemic	Cytology (Pap)	60-64	60	Not performed	Satisfactory	NILM	NILM	Negative (NILM)	
P02108	2018	2018-Q4	2018-10	Pre-pandemic	Cytology (Pap)	40-44	43	Not performed	Satisfactory	NILM	NILM	Negative (NILM)	
P02109	2018	2018-Q4	2018-10	Pre-pandemic	Cytology (Pap)	60-64	61	Not performed	Satisfactory	NILM	NILM	Negative (NILM)	Trichomonas
P02110	2018	2018-Q4	2018-10	Pre-pandemic	Cytology (Pap)	35-39	39	Not performed	Satisfactory	NILM	NILM	Negative (NILM)	
P02111	2018	2018-Q4	2018-10	Pre-pandemic	Cytology (Pap)	40-44	43	Not performed	Satisfactory	NILM	NILM	Negative (NILM)	
P02112	2018	2018-Q4	2018-10	Pre-pandemic	Cytology (Pap)	45-49	46	Not performed	Satisfactory	NILM	NILM	Negative (NILM)	
P02113	2018	2018-Q4	2018-10	Pre-pandemic	Cytology (Pap)	60-64	60	Not performed	Satisfactory	NILM	NILM	Negative (NILM)	
P02114	2018	2018-Q4	2018-10	Pre-pandemic	Cytology (Pap)	25-29	26	Not performed	Satisfactory	NILM	NILM	Negative (NILM)	
P02115	2018	2018-Q4	2018-10	Pre-pandemic	Cytology (Pap)	50-54	54	Not performed	Satisfactory	NILM	NILM	Negative (NILM)	
P02116	2018	2018-Q4	2018-10	Pre-pandemic	Cytology (Pap)	30-34	33	Not performed	Satisfactory	NILM	NILM	Negative (NILM)	
P02117	2018	2018-Q4	2018-10	Pre-pandemic	Cytology (Pap)	35-39	35	Not performed	Satisfactory	NILM	NILM	Negative (NILM)	
P02118	2018	2018-Q4	2018-10	Pre-pandemic	Cytology (Pap)	60-64	63	Not performed	Satisfactory	NILM	NILM	Negative (NILM)	
P02119	2018	2018-Q4	2018-10	Pre-pandemic	Cytology (Pap)	Not recorded		Not performed	Satisfactory	NILM	NILM	Negative (NILM)	
P02120	2018	2018-Q4	2018-10	Pre-pandemic	Cytology (Pap)	35-39	36	Not performed	Satisfactory	NILM	NILM	Negative (NILM)	
P02121	2018	2018-Q4	2018-10	Pre-pandemic	Cytology (Pap)	25-29	29	Not performed	Satisfactory	NILM	NILM	Negative (NILM)	Candida
P02122	2018	2018-Q4	2018-10	Pre-pandemic	Cytology (Pap)	40-44	42	Not performed	Satisfactory	NILM	NILM	Negative (NILM)	
P02123	2018	2018-Q4	2018-10	Pre-pandemic	Cytology (Pap)	50-54	52	Not performed	Unsatisfactory	Unsatisfactory	Unsatisfactory	Unsatisfactory	
P02124	2018	2018-Q4	2018-10	Pre-pandemic	Cytology (Pap)	55-59	58	Not performed	Satisfactory	NILM	NILM	Negative (NILM)	Trichomonas
P02125	2018	2018-Q4	2018-10	Pre-pandemic	Cytology (Pap)	30-34	30	Not performed	Satisfactory	NILM	NILM	Negative (NILM)	
P02126	2018	2018-Q4	2018-10	Pre-pandemic	Cytology (Pap)	50-54	50	Not performed	Satisfactory	NILM	NILM	Negative (NILM)	Candida
P02127	2018	2018-Q4	2018-10	Pre-pandemic	Cytology (Pap)	60-64	61	Not performed	Satisfactory	NILM	NILM	Negative (NILM)	
P02128	2018	2018-Q4	2018-10	Pre-pandemic	Cytology (Pap)	50-54	52	Not performed	Satisfactory	NILM	NILM	Negative (NILM)	
P02129	2018	2018-Q4	2018-10	Pre-pandemic	Cytology (Pap)	30-34	32	Not performed	Satisfactory	NILM	NILM	Negative (NILM)	Candida
P02130	2018	2018-Q4	2018-10	Pre-pandemic	Cytology (Pap)	30-34	30	Not performed	Satisfactory	NILM	NILM	Negative (NILM)	
P02131	2018	2018-Q4	2018-10	Pre-pandemic	Cytology (Pap)	30-34	33	Not performed	Satisfactory	Positive	ASC-US	Low-grade / equivocal squamous	
P02132	2018	2018-Q4	2018-10	Pre-pandemic	Cytology (Pap)	45-49	48	Not performed	Satisfactory	Positive	HSIL	High-grade squamous	
P02133	2018	2018-Q4	2018-10	Pre-pandemic	Cytology (Pap)	50-54	52	Not performed	Satisfactory	NILM	NILM	Negative (NILM)	
P02134	2018	2018-Q4	2018-10	Pre-pandemic	Cytology (Pap)	35-39	36	Not performed	Satisfactory	NILM	NILM	Negative (NILM)	
P02135	2018	2018-Q4	2018-10	Pre-pandemic	Cytology (Pap)	30-34	33	Not performed	Satisfactory	NILM	NILM	Negative (NILM)	
P02136	2018	2018-Q4	2018-10	Pre-pandemic	Cytology (Pap)	40-44	41	Not performed	Satisfactory	NILM	NILM	Negative (NILM)	
P02137	2018	2018-Q4	2018-10	Pre-pandemic	Cytology (Pap)	30-34	33	Not performed	Satisfactory	Positive	HSIL	High-grade squamous	
P02138	2018	2018-Q4	2018-10	Pre-pandemic	Cytology (Pap)	55-59	58	Not performed	Satisfactory	NILM	NILM	Negative (NILM)	
P02139	2018	2018-Q4	2018-10	Pre-pandemic	Cytology (Pap)	30-34	33	Not performed	Satisfactory	NILM	NILM	Negative (NILM)	
P02140	2018	2018-Q4	2018-10	Pre-pandemic	Cytology (Pap)	45-49	45	Not performed	Satisfactory	NILM	NILM	Negative (NILM)	
P02141	2018	2018-Q4	2018-10	Pre-pandemic	Cytology (Pap)	50-54	52	Not performed	Satisfactory	NILM	NILM	Negative (NILM)	
P02142	2018	2018-Q4	2018-10	Pre-pandemic	Cytology (Pap)	40-44	44	Not performed	Satisfactory	NILM	NILM	Negative (NILM)	
P02143	2018	2018-Q4	2018-10	Pre-pandemic	Cytology (Pap)	45-49	45	Not performed	Satisfactory	Positive	LSIL	Low-grade / equivocal squamous	Trichomonas
P02144	2018	2018-Q4	2018-10	Pre-pandemic	Cytology (Pap)	50-54	51	Not performed	Satisfactory	NILM	NILM	Negative (NILM)	
P02145	2018	2018-Q4	2018-10	Pre-pandemic	Cytology (Pap)	Not recorded		Not performed	Satisfactory	NILM	NILM	Negative (NILM)	
P02146	2018	2018-Q4	2018-10	Pre-pandemic	Cytology (Pap)	40-44	40	Not performed	Satisfactory	NILM	NILM	Negative (NILM)	
P02147	2018	2018-Q4	2018-10	Pre-pandemic	Cytology (Pap)	30-34	30	Not performed	Satisfactory	NILM	NILM	Negative (NILM)	
P02148	2018	2018-Q4	2018-10	Pre-pandemic	Cytology (Pap)	Not recorded		Not performed	Satisfactory	NILM	NILM	Negative (NILM)	
P02149	2018	2018-Q4	2018-10	Pre-pandemic	Cytology (Pap)	50-54	54	Not performed	Satisfactory	NILM	NILM	Negative (NILM)	
P02150	2018	2018-Q4	2018-10	Pre-pandemic	Cytology (Pap)	30-34	31	Not performed	Satisfactory	NILM	NILM	Negative (NILM)	Trichomonas
P02151	2018	2018-Q4	2018-10	Pre-pandemic	Cytology (Pap)	35-39	37	Not performed	Satisfactory	NILM	NILM	Negative (NILM)	
P02152	2018	2018-Q4	2018-10	Pre-pandemic	Cytology (Pap)	60-64	62	Not performed	Satisfactory	NILM	NILM	Negative (NILM)	
P02153	2018	2018-Q4	2018-10	Pre-pandemic	Cytology (Pap)	55-59	59	Not performed	Satisfactory	NILM	NILM	Negative (NILM)	
P02154	2018	2018-Q4	2018-10	Pre-pandemic	Cytology (Pap)	40-44	42	Not performed	Satisfactory	NILM	NILM	Negative (NILM)	
P02155	2018	2018-Q4	2018-10	Pre-pandemic	Cytology (Pap)	35-39	35	Not performed	Satisfactory	NILM	NILM	Negative (NILM)	
P02156	2018	2018-Q4	2018-10	Pre-pandemic	Cytology (Pap)	45-49	47	Not performed	Satisfactory	NILM	NILM	Negative (NILM)	
P02157	2018	2018-Q4	2018-10	Pre-pandemic	Cytology (Pap)	55-59	55	Not performed	Satisfactory	NILM	NILM	Negative (NILM)	
P02158	2018	2018-Q4	2018-10	Pre-pandemic	Cytology (Pap)	35-39	36	Not performed	Satisfactory	NILM	NILM	Negative (NILM)	

patient_id	year	quarter	month	period	primary_test	age_group	age_years	hpv_result	adequacy	result	bethesda	grade	infection
P02159	2018	2018-Q4	2018-10	Pre-pandemic	Cytology (Pap)	35-39	39	Not performed	Satisfactory	NILM	NILM	Negative (NILM)	
P02160	2018	2018-Q4	2018-10	Pre-pandemic	Cytology (Pap)	60-64	61	Not performed	Satisfactory	NILM	NILM	Negative (NILM)	Candida
P02161	2018	2018-Q4	2018-10	Pre-pandemic	Cytology (Pap)	40-44	41	Not performed	Satisfactory	NILM	NILM	Negative (NILM)	
P02162	2018	2018-Q4	2018-10	Pre-pandemic	Cytology (Pap)	40-44	40	Not performed	Satisfactory	NILM	NILM	Negative (NILM)	
P02163	2018	2018-Q4	2018-10	Pre-pandemic	Cytology (Pap)	Not recorded		Not performed	Satisfactory	NILM	NILM	Negative (NILM)	
P02164	2018	2018-Q4	2018-10	Pre-pandemic	Cytology (Pap)	40-44	40	Not performed	Satisfactory	Positive	AGC	Glandular	
P02165	2018	2018-Q4	2018-10	Pre-pandemic	Cytology (Pap)	35-39	37	Not performed	Satisfactory	NILM	NILM	Negative (NILM)	
P02166	2018	2018-Q4	2018-10	Pre-pandemic	Cytology (Pap)	45-49	48	Not performed	Satisfactory	NILM	NILM	Negative (NILM)	
P02167	2018	2018-Q4	2018-10	Pre-pandemic	Cytology (Pap)	35-39	39	Not performed	Satisfactory	NILM	NILM	Negative (NILM)	
P02168	2018	2018-Q4	2018-10	Pre-pandemic	Cytology (Pap)	35-39	38	Not performed	Satisfactory	NILM	NILM	Negative (NILM)	
P02169	2018	2018-Q4	2018-10	Pre-pandemic	Cytology (Pap)	35-39	36	Not performed	Satisfactory	NILM	NILM	Negative (NILM)	
P02170	2018	2018-Q4	2018-10	Pre-pandemic	Cytology (Pap)	25-29	28	Not performed	Satisfactory	NILM	NILM	Negative (NILM)	
P02171	2018	2018-Q4	2018-10	Pre-pandemic	Cytology (Pap)	60-64	63	Not performed	Satisfactory	NILM	NILM	Negative (NILM)	
P02172	2018	2018-Q4	2018-10	Pre-pandemic	Cytology (Pap)	40-44	44	Not performed	Satisfactory	NILM	NILM	Negative (NILM)	
P02173	2018	2018-Q4	2018-10	Pre-pandemic	Cytology (Pap)	35-39	36	Not performed	Satisfactory	NILM	NILM	Negative (NILM)	
P02174	2018	2018-Q4	2018-10	Pre-pandemic	Cytology (Pap)	55-59	57	Not performed	Satisfactory	NILM	NILM	Negative (NILM)	
P02175	2018	2018-Q4	2018-10	Pre-pandemic	Cytology (Pap)	25-29	27	Not performed	Satisfactory	NILM	NILM	Negative (NILM)	
P02176	2018	2018-Q4	2018-10	Pre-pandemic	Cytology (Pap)	45-49	45	Not performed	Satisfactory	NILM	NILM	Negative (NILM)	
P02177	2018	2018-Q4	2018-10	Pre-pandemic	Cytology (Pap)	35-39	35	Not performed	Satisfactory	NILM	NILM	Negative (NILM)	
P02178	2018	2018-Q4	2018-10	Pre-pandemic	Cytology (Pap)	Not recorded		Not performed	Satisfactory	NILM	NILM	Negative (NILM)	
P02179	2018	2018-Q4	2018-10	Pre-pandemic	Cytology (Pap)	40-44	40	Not performed	Satisfactory	NILM	NILM	Negative (NILM)	
P02180	2018	2018-Q4	2018-10	Pre-pandemic	Cytology (Pap)	35-39	36	Not performed	Satisfactory	Positive	ASC-US	Low-grade / equivocal squamous	
P02181	2018	2018-Q4	2018-10	Pre-pandemic	Cytology (Pap)	60-64	63	Not performed	Satisfactory	NILM	NILM	Negative (NILM)	
P02182	2018	2018-Q4	2018-10	Pre-pandemic	Cytology (Pap)	55-59	58	Not performed	Satisfactory	NILM	NILM	Negative (NILM)	
P02183	2018	2018-Q4	2018-10	Pre-pandemic	Cytology (Pap)	30-34	34	Not performed	Satisfactory	NILM	NILM	Negative (NILM)	Other
P02184	2018	2018-Q4	2018-10	Pre-pandemic	Cytology (Pap)	Not recorded		Not performed	Satisfactory	NILM	NILM	Negative (NILM)	
P02185	2018	2018-Q4	2018-10	Pre-pandemic	Cytology (Pap)	35-39	39	Not performed	Satisfactory	NILM	NILM	Negative (NILM)	
P02186	2018	2018-Q4	2018-10	Pre-pandemic	Cytology (Pap)	60-64	62	Not performed	Satisfactory	NILM	NILM	Negative (NILM)	
P02187	2018	2018-Q4	2018-10	Pre-pandemic	Cytology (Pap)	50-54	50	Not performed	Satisfactory	NILM	NILM	Negative (NILM)	
P02188	2018	2018-Q4	2018-10	Pre-pandemic	Cytology (Pap)	60-64	63	Not performed	Satisfactory	NILM	NILM	Negative (NILM)	
P02189	2018	2018-Q4	2018-10	Pre-pandemic	Cytology (Pap)	40-44	43	Not performed	Satisfactory	NILM	NILM	Negative (NILM)	
P02190	2018	2018-Q4	2018-10	Pre-pandemic	Cytology (Pap)	45-49	45	Not performed	Unsatisfactory	Unsatisfactory	Unsatisfactory	Unsatisfactory	
P02191	2018	2018-Q4	2018-10	Pre-pandemic	Cytology (Pap)	25-29	27	Not performed	Satisfactory	NILM	NILM	Negative (NILM)	
P02192	2018	2018-Q4	2018-10	Pre-pandemic	Cytology (Pap)	35-39	37	Not performed	Satisfactory	NILM	NILM	Negative (NILM)	
P02193	2018	2018-Q4	2018-10	Pre-pandemic	Cytology (Pap)	25-29	25	Not performed	Satisfactory	NILM	NILM	Negative (NILM)	
P02194	2018	2018-Q4	2018-10	Pre-pandemic	Cytology (Pap)	30-34	33	Not performed	Satisfactory	NILM	NILM	Negative (NILM)	Candida
P02195	2018	2018-Q4	2018-10	Pre-pandemic	Cytology (Pap)	30-34	33	Not performed	Satisfactory	NILM	NILM	Negative (NILM)	
P02196	2018	2018-Q4	2018-10	Pre-pandemic	Cytology (Pap)	30-34	31	Not performed	Satisfactory	NILM	NILM	Negative (NILM)	
P02197	2018	2018-Q4	2018-10	Pre-pandemic	Cytology (Pap)	35-39	37	Not performed	Satisfactory	NILM	NILM	Negative (NILM)	
P02198	2018	2018-Q4	2018-10	Pre-pandemic	Cytology (Pap)	35-39	38	Not performed	Satisfactory	NILM	NILM	Negative (NILM)	
P02199	2018	2018-Q4	2018-10	Pre-pandemic	Cytology (Pap)	60-64	62	Not performed	Satisfactory	NILM	NILM	Negative (NILM)	
P02200	2018	2018-Q4	2018-10	Pre-pandemic	Cytology (Pap)	30-34	30	Not performed	Satisfactory	Positive	ASC-H	High-grade squamous	
P02201	2018	2018-Q4	2018-10	Pre-pandemic	Cytology (Pap)	40-44	41	Not performed	Satisfactory	NILM	NILM	Negative (NILM)	
P02202	2018	2018-Q4	2018-10	Pre-pandemic	Cytology (Pap)	55-59	57	Not performed	Satisfactory	NILM	NILM	Negative (NILM)	
P02203	2018	2018-Q4	2018-10	Pre-pandemic	Cytology (Pap)	30-34	32	Not performed	Satisfactory	NILM	NILM	Negative (NILM)	
P02204	2018	2018-Q4	2018-10	Pre-pandemic	Cytology (Pap)	45-49	49	Not performed	Satisfactory	NILM	NILM	Negative (NILM)	
P02205	2018	2018-Q4	2018-10	Pre-pandemic	Cytology (Pap)	55-59	58	Not performed	Satisfactory	Positive	AGC	Glandular	
P02206	2018	2018-Q4	2018-10	Pre-pandemic	Cytology (Pap)	30-34	33	Not performed	Satisfactory	NILM	NILM	Negative (NILM)	Other
P02207	2018	2018-Q4	2018-10	Pre-pandemic	Cytology (Pap)	60-64	61	Not performed	Satisfactory	NILM	NILM	Negative (NILM)	
P02208	2018	2018-Q4	2018-10	Pre-pandemic	Cytology (Pap)	35-39	36	Not performed	Satisfactory	NILM	NILM	Negative (NILM)	
P02209	2018	2018-Q4	2018-10	Pre-pandemic	Cytology (Pap)	55-59	58	Not performed	Satisfactory	Positive	ASC-US	Low-grade / equivocal squamous	
P02210	2018	2018-Q4	2018-10	Pre-pandemic	Cytology (Pap)	55-59	58	Not performed	Satisfactory	Positive	ASC-US	Low-grade / equivocal squamous	
P02211	2018	2018-Q4	2018-10	Pre-pandemic	Cytology (Pap)	30-34	30	Not performed	Satisfactory	NILM	NILM	Negative (NILM)	
P02212	2018	2018-Q4	2018-10	Pre-pandemic	Cytology (Pap)	45-49	47	Not performed	Satisfactory	NILM	NILM	Negative (NILM)	
P02213	2018	2018-Q4	2018-10	Pre-pandemic	Cytology (Pap)	40-44	43	Not performed	Unsatisfactory	Unsatisfactory	Unsatisfactory	Unsatisfactory	
P02214	2018	2018-Q4	2018-10	Pre-pandemic	Cytology (Pap)	40-44	43	Not performed	Satisfactory	Positive	LSIL	Low-grade / equivocal squamous	
P02215	2018	2018-Q4	2018-10	Pre-pandemic	Cytology (Pap)	50-54	51	Not performed	Satisfactory	NILM	NILM	Negative (NILM)	
P02216	2018	2018-Q4	2018-10	Pre-pandemic	Cytology (Pap)	40-44	44	Not performed	Satisfactory	NILM	NILM	Negative (NILM)	
P02217	2018	2018-Q4	2018-10	Pre-pandemic	Cytology (Pap)	30-34	32	Not performed	Satisfactory	NILM	NILM	Negative (NILM)	
P02218	2018	2018-Q4	2018-10	Pre-pandemic	Cytology (Pap)	35-39	37	Not performed	Satisfactory	Positive	ASC-H	High-grade squamous	Candida
P02219	2018	2018-Q4	2018-10	Pre-pandemic	Cytology (Pap)	50-54	53	Not performed	Satisfactory	Positive	ASC-H	High-grade squamous	
P02220	2018	2018-Q4	2018-10	Pre-pandemic	Cytology (Pap)	50-54	51	Not performed	Satisfactory	NILM	NILM	Negative (NILM)	
P02221	2018	2018-Q4	2018-10	Pre-pandemic	Cytology (Pap)	45-49	45	Not performed	Satisfactory	NILM	NILM	Negative (NILM)	
P02222	2018	2018-Q4	2018-10	Pre-pandemic	Cytology (Pap)	50-54	51	Not performed	Satisfactory	NILM	NILM	Negative (NILM)	
P02223	2018	2018-Q4	2018-10	Pre-pandemic	Cytology (Pap)	40-44	40	Not performed	Satisfactory	NILM	NILM	Negative (NILM)	
P02224	2018	2018-Q4	2018-10	Pre-pandemic	Cytology (Pap)	45-49	49	Not performed	Satisfactory	Positive	HSIL	High-grade squamous	
P02225	2018	2018-Q4	2018-10	Pre-pandemic	Cytology (Pap)	35-39	37	Not performed	Satisfactory	Positive	ASC-US	Low-grade / equivocal squamous	
P02226	2018	2018-Q4	2018-10	Pre-pandemic	Cytology (Pap)	50-54	51	Not performed	Satisfactory	NILM	NILM	Negative (NILM)	
P02227	2018	2018-Q4	2018-10	Pre-pandemic	Cytology (Pap)	35-39	38	Not performed	Satisfactory	NILM	NILM	Negative (NILM)	
P02228	2018	2018-Q4	2018-10	Pre-pandemic	Cytology (Pap)	35-39	35	Not performed	Satisfactory	NILM	NILM	Negative (NILM)	
P02229	2018	2018-Q4	2018-10	Pre-pandemic	Cytology (Pap)	40-44	42	Not performed	Satisfactory	NILM	NILM	Negative (NILM)	
P02230	2018	2018-Q4	2018-10	Pre-pandemic	Cytology (Pap)	50-54	50	Not performed	Unsatisfactory	Unsatisfactory	Unsatisfactory	Unsatisfactory	
P02231	2018	2018-Q4	2018-10	Pre-pandemic	Cytology (Pap)	40-44	42	Not performed	Satisfactory	NILM	NILM	Negative (NILM)	
P02232	2018	2018-Q4	2018-10	Pre-pandemic	Cytology (Pap)	45-49	47	Not performed	Satisfactory	NILM	NILM	Negative (NILM)	
P02233	2018	2018-Q4	2018-10	Pre-pandemic	Cytology (Pap)	Not recorded		Not performed	Satisfactory	NILM	NILM	Negative (NILM)	
P02234	2018	2018-Q4	2018-10	Pre-pandemic	Cytology (Pap)	55-59	55	Not performed	Satisfactory	NILM	NILM	Negative (NILM)	
P02235	2018	2018-Q4	2018-10	Pre-pandemic	Cytology (Pap)	50-54	50	Not performed	Satisfactory	NILM	NILM	Negative (NILM)	
P02236	2018	2018-Q4	2018-10	Pre-pandemic	Cytology (Pap)	Not recorded		Not performed	Satisfactory	NILM	NILM	Negative (NILM)	
P02237	2018	2018-Q4	2018-10	Pre-pandemic	Cytology (Pap)	40-44	42	Not performed	Satisfactory	NILM	NILM	Negative (NILM)	
P02238	2018	2018-Q4	2018-10	Pre-pandemic	Cytology (Pap)	60-64	61	Not performed	Satisfactory	NILM	NILM	Negative (NILM)	

patient_id	year	quarter	month	period	primary_test	age_group	age_years	hpv_result	adequacy	result	bethesda	grade	infection
P02239	2018	2018-Q4	2018-10	Pre-pandemic	Cytology (Pap)	Not recorded		Not performed	Satisfactory	NILM	NILM	Negative (NILM)	
P02240	2018	2018-Q4	2018-10	Pre-pandemic	Cytology (Pap)	60-64	62	Not performed	Satisfactory	NILM	NILM	Negative (NILM)	
P02241	2018	2018-Q4	2018-10	Pre-pandemic	Cytology (Pap)	55-59	59	Not performed	Satisfactory	Positive	AGC	Glandular	
P02242	2018	2018-Q4	2018-10	Pre-pandemic	Cytology (Pap)	50-54	52	Not performed	Satisfactory	NILM	NILM	Negative (NILM)	
P02243	2018	2018-Q4	2018-10	Pre-pandemic	Cytology (Pap)	45-49	48	Not performed	Satisfactory	NILM	NILM	Negative (NILM)	
P02244	2018	2018-Q4	2018-10	Pre-pandemic	Cytology (Pap)	45-49	45	Not performed	Satisfactory	Positive	LSIL	Low-grade / equivocal squamous	
P02245	2018	2018-Q4	2018-10	Pre-pandemic	Cytology (Pap)	55-59	58	Not performed	Satisfactory	NILM	NILM	Negative (NILM)	
P02246	2018	2018-Q4	2018-10	Pre-pandemic	Cytology (Pap)	50-54	54	Not performed	Satisfactory	NILM	NILM	Negative (NILM)	
P02247	2018	2018-Q4	2018-10	Pre-pandemic	Cytology (Pap)	30-34	34	Not performed	Satisfactory	NILM	NILM	Negative (NILM)	Candida
P02248	2018	2018-Q4	2018-10	Pre-pandemic	Cytology (Pap)	25-29	27	Not performed	Satisfactory	NILM	NILM	Negative (NILM)	Other
P02249	2018	2018-Q4	2018-10	Pre-pandemic	Cytology (Pap)	55-59	56	Not performed	Satisfactory	NILM	NILM	Negative (NILM)	
P02250	2018	2018-Q4	2018-10	Pre-pandemic	Cytology (Pap)	40-44	43	Not performed	Satisfactory	NILM	NILM	Negative (NILM)	
P02251	2018	2018-Q4	2018-10	Pre-pandemic	Cytology (Pap)	55-59	59	Not performed	Satisfactory	NILM	NILM	Negative (NILM)	
P02252	2018	2018-Q4	2018-10	Pre-pandemic	Cytology (Pap)	40-44	40	Not performed	Satisfactory	NILM	NILM	Negative (NILM)	
P02253	2018	2018-Q4	2018-10	Pre-pandemic	Cytology (Pap)	40-44	41	Not performed	Satisfactory	Positive	ASC-US	Low-grade / equivocal squamous	
P02254	2018	2018-Q4	2018-10	Pre-pandemic	Cytology (Pap)	45-49	45	Not performed	Satisfactory	NILM	NILM	Negative (NILM)	
P02255	2018	2018-Q4	2018-10	Pre-pandemic	Cytology (Pap)	50-54	52	Not performed	Satisfactory	NILM	NILM	Negative (NILM)	
P02256	2018	2018-Q4	2018-10	Pre-pandemic	Cytology (Pap)	55-59	58	Not performed	Satisfactory	Positive	ASC-US	Low-grade / equivocal squamous	
P02257	2018	2018-Q4	2018-10	Pre-pandemic	Cytology (Pap)	30-34	31	Not performed	Satisfactory	NILM	NILM	Negative (NILM)	
P02258	2018	2018-Q4	2018-10	Pre-pandemic	Cytology (Pap)	30-34	30	Not performed	Satisfactory	Positive	ASC-US	Low-grade / equivocal squamous	
P02259	2018	2018-Q4	2018-10	Pre-pandemic	Cytology (Pap)	60-64	61	Not performed	Satisfactory	Positive	AGC	Glandular	
P02260	2018	2018-Q4	2018-10	Pre-pandemic	Cytology (Pap)	50-54	50	Not performed	Satisfactory	NILM	NILM	Negative (NILM)	
P02261	2018	2018-Q4	2018-10	Pre-pandemic	Cytology (Pap)	35-39	38	Not performed	Satisfactory	NILM	NILM	Negative (NILM)	
P02262	2018	2018-Q4	2018-10	Pre-pandemic	Cytology (Pap)	25-29	25	Not performed	Satisfactory	Positive	ASC-US	Low-grade / equivocal squamous	
P02263	2018	2018-Q4	2018-10	Pre-pandemic	Cytology (Pap)	45-49	49	Not performed	Satisfactory	NILM	NILM	Negative (NILM)	
P02264	2018	2018-Q4	2018-10	Pre-pandemic	Cytology (Pap)	25-29	26	Not performed	Satisfactory	NILM	NILM	Negative (NILM)	
P02265	2018	2018-Q4	2018-10	Pre-pandemic	Cytology (Pap)	35-39	39	Not performed	Satisfactory	NILM	NILM	Negative (NILM)	
P02266	2018	2018-Q4	2018-10	Pre-pandemic	Cytology (Pap)	35-39	37	Not performed	Satisfactory	NILM	NILM	Negative (NILM)	
P02267	2018	2018-Q4	2018-10	Pre-pandemic	Cytology (Pap)	35-39	39	Not performed	Satisfactory	NILM	NILM	Negative (NILM)	
P02268	2018	2018-Q4	2018-10	Pre-pandemic	Cytology (Pap)	30-34	30	Not performed	Satisfactory	NILM	NILM	Negative (NILM)	
P02269	2018	2018-Q4	2018-10	Pre-pandemic	Cytology (Pap)	35-39	35	Not performed	Satisfactory	NILM	NILM	Negative (NILM)	
P02270	2018	2018-Q4	2018-10	Pre-pandemic	Cytology (Pap)	60-64	60	Not performed	Satisfactory	NILM	NILM	Negative (NILM)	
P02271	2018	2018-Q4	2018-10	Pre-pandemic	Cytology (Pap)	40-44	42	Not performed	Satisfactory	NILM	NILM	Negative (NILM)	
P02272	2018	2018-Q4	2018-10	Pre-pandemic	Cytology (Pap)	30-34	32	Not performed	Satisfactory	NILM	NILM	Negative (NILM)	
P02273	2018	2018-Q4	2018-10	Pre-pandemic	Cytology (Pap)	50-54	54	Not performed	Satisfactory	NILM	NILM	Negative (NILM)	
P02274	2018	2018-Q4	2018-10	Pre-pandemic	Cytology (Pap)	35-39	36	Not performed	Satisfactory	NILM	NILM	Negative (NILM)	
P02275	2018	2018-Q4	2018-10	Pre-pandemic	Cytology (Pap)	30-34	31	Not performed	Satisfactory	NILM	NILM	Negative (NILM)	
P02276	2018	2018-Q4	2018-10	Pre-pandemic	Cytology (Pap)	35-39	37	Not performed	Satisfactory	NILM	NILM	Negative (NILM)	
P02277	2018	2018-Q4	2018-10	Pre-pandemic	Cytology (Pap)	40-44	41	Not performed	Satisfactory	NILM	NILM	Negative (NILM)	
P02278	2018	2018-Q4	2018-10	Pre-pandemic	Cytology (Pap)	40-44	44	Not performed	Satisfactory	Positive	HSIL	High-grade squamous	
P02279	2018	2018-Q4	2018-10	Pre-pandemic	Cytology (Pap)	45-49	45	Not performed	Satisfactory	NILM	NILM	Negative (NILM)	
P02280	2018	2018-Q4	2018-10	Pre-pandemic	Cytology (Pap)	Not recorded		Not performed	Satisfactory	NILM	NILM	Negative (NILM)	
P02281	2018	2018-Q4	2018-10	Pre-pandemic	Cytology (Pap)	40-44	42	Not performed	Satisfactory	Positive	HSIL	High-grade squamous	
P02282	2018	2018-Q4	2018-10	Pre-pandemic	Cytology (Pap)	45-49	48	Not performed	Satisfactory	NILM	NILM	Negative (NILM)	
P02283	2018	2018-Q4	2018-10	Pre-pandemic	Cytology (Pap)	40-44	44	Not performed	Satisfactory	NILM	NILM	Negative (NILM)	
P02284	2018	2018-Q4	2018-10	Pre-pandemic	Cytology (Pap)	25-29	25	Not performed	Satisfactory	NILM	NILM	Negative (NILM)	
P02285	2018	2018-Q4	2018-10	Pre-pandemic	Cytology (Pap)	40-44	41	Not performed	Satisfactory	NILM	NILM	Negative (NILM)	
P02286	2018	2018-Q4	2018-10	Pre-pandemic	Cytology (Pap)	40-44	44	Not performed	Satisfactory	NILM	NILM	Negative (NILM)	
P02287	2018	2018-Q4	2018-10	Pre-pandemic	Cytology (Pap)	40-44	40	Not performed	Unsatisfactory	Unsatisfactory	Unsatisfactory	Unsatisfactory	
P02288	2018	2018-Q4	2018-10	Pre-pandemic	Cytology (Pap)	50-54	54	Not performed	Satisfactory	NILM	NILM	Negative (NILM)	
P02289	2018	2018-Q4	2018-10	Pre-pandemic	Cytology (Pap)	Not recorded		Not performed	Satisfactory	NILM	NILM	Negative (NILM)	
P02290	2018	2018-Q4	2018-10	Pre-pandemic	Cytology (Pap)	35-39	36	Not performed	Satisfactory	NILM	NILM	Negative (NILM)	
P02291	2018	2018-Q4	2018-10	Pre-pandemic	Cytology (Pap)	35-39	35	Not performed	Satisfactory	NILM	NILM	Negative (NILM)	
P02292	2018	2018-Q4	2018-10	Pre-pandemic	Cytology (Pap)	60-64	62	Not performed	Satisfactory	NILM	NILM	Negative (NILM)	
P02293	2018	2018-Q4	2018-10	Pre-pandemic	Cytology (Pap)	35-39	35	Not performed	Satisfactory	NILM	NILM	Negative (NILM)	
P02294	2018	2018-Q4	2018-10	Pre-pandemic	Cytology (Pap)	30-34	31	Not performed	Satisfactory	NILM	NILM	Negative (NILM)	
P02295	2018	2018-Q4	2018-10	Pre-pandemic	Cytology (Pap)	55-59	55	Not performed	Satisfactory	NILM	NILM	Negative (NILM)	
P02296	2018	2018-Q4	2018-10	Pre-pandemic	Cytology (Pap)	45-49	48	Not performed	Satisfactory	NILM	NILM	Negative (NILM)	Candida
P02297	2018	2018-Q4	2018-10	Pre-pandemic	Cytology (Pap)	50-54	50	Not performed	Satisfactory	NILM	NILM	Negative (NILM)	Gardnerella
P02298	2018	2018-Q4	2018-10	Pre-pandemic	Cytology (Pap)	45-49	49	Not performed	Satisfactory	NILM	NILM	Negative (NILM)	
P02299	2018	2018-Q4	2018-10	Pre-pandemic	Cytology (Pap)	40-44	40	Not performed	Satisfactory	NILM	NILM	Negative (NILM)	
P02300	2018	2018-Q4	2018-10	Pre-pandemic	Cytology (Pap)	45-49	47	Not performed	Satisfactory	NILM	NILM	Negative (NILM)	
P02301	2018	2018-Q4	2018-10	Pre-pandemic	Cytology (Pap)	25-29	28	Not performed	Satisfactory	NILM	NILM	Negative (NILM)	
P02302	2018	2018-Q4	2018-10	Pre-pandemic	Cytology (Pap)	35-39	35	Not performed	Satisfactory	NILM	NILM	Negative (NILM)	
P02303	2018	2018-Q4	2018-10	Pre-pandemic	Cytology (Pap)	45-49	48	Not performed	Satisfactory	NILM	NILM	Negative (NILM)	Trichomonas
P02304	2018	2018-Q4	2018-10	Pre-pandemic	Cytology (Pap)	55-59	55	Not performed	Satisfactory	NILM	NILM	Negative (NILM)	
P02305	2018	2018-Q4	2018-10	Pre-pandemic	Cytology (Pap)	45-49	47	Not performed	Satisfactory	NILM	NILM	Negative (NILM)	
P02306	2018	2018-Q4	2018-10	Pre-pandemic	Cytology (Pap)	30-34	30	Not performed	Satisfactory	NILM	NILM	Negative (NILM)	
P02307	2018	2018-Q4	2018-10	Pre-pandemic	Cytology (Pap)	40-44	40	Not performed	Satisfactory	NILM	NILM	Negative (NILM)	
P02308	2018	2018-Q4	2018-10	Pre-pandemic	Cytology (Pap)	45-49	48	Not performed	Satisfactory	NILM	NILM	Negative (NILM)	
P02309	2018	2018-Q4	2018-10	Pre-pandemic	Cytology (Pap)	50-54	50	Not performed	Satisfactory	NILM	NILM	Negative (NILM)	
P02310	2018	2018-Q4	2018-10	Pre-pandemic	Cytology (Pap)	30-34	33	Not performed	Satisfactory	NILM	NILM	Negative (NILM)	
P02311	2018	2018-Q4	2018-10	Pre-pandemic	Cytology (Pap)	35-39	36	Not performed	Satisfactory	NILM	NILM	Negative (NILM)	
P02312	2018	2018-Q4	2018-10	Pre-pandemic	Cytology (Pap)	40-44	41	Not performed	Satisfactory	NILM	NILM	Negative (NILM)	
P02313	2018	2018-Q4	2018-10	Pre-pandemic	Cytology (Pap)	30-34	32	Not performed	Satisfactory	Positive	AGC	Glandular	
P02314	2018	2018-Q4	2018-10	Pre-pandemic	Cytology (Pap)	45-49	46	Not performed	Satisfactory	NILM	NILM	Negative (NILM)	
P02315	2018	2018-Q4	2018-10	Pre-pandemic	Cytology (Pap)	25-29	26	Not performed	Satisfactory	NILM	NILM	Negative (NILM)	
P02316	2018	2018-Q4	2018-10	Pre-pandemic	Cytology (Pap)	35-39	39	Not performed	Satisfactory	NILM	NILM	Negative (NILM)	
P02317	2018	2018-Q4	2018-10	Pre-pandemic	Cytology (Pap)	40-44	40	Not performed	Unsatisfactory	Unsatisfactory	Unsatisfactory	Unsatisfactory	
P02318	2018	2018-Q4	2018-10	Pre-pandemic	Cytology (Pap)	30-34	32	Not performed	Satisfactory	Positive	ASC-US	Low-grade / equivocal squamous	

patient_id	year	quarter	month	period	primary_test	age_group	age_years	hpv_result	adequacy	result	bethesda	grade	infection
P02319	2018	2018-Q4	2018-10	Pre-pandemic	Cytology (Pap)	30-34	34	Not performed	Satisfactory	NILM	NILM	Negative (NILM)	
P02320	2018	2018-Q4	2018-10	Pre-pandemic	Cytology (Pap)	Not recorded		Not performed	Satisfactory	NILM	NILM	Negative (NILM)	
P02321	2018	2018-Q4	2018-10	Pre-pandemic	Cytology (Pap)	45-49	46	Not performed	Satisfactory	Positive	ASC-US	Low-grade / equivocal squamous	
P02322	2018	2018-Q4	2018-10	Pre-pandemic	Cytology (Pap)	40-44	41	Not performed	Satisfactory	NILM	NILM	Negative (NILM)	
P02323	2018	2018-Q4	2018-10	Pre-pandemic	Cytology (Pap)	50-54	53	Not performed	Satisfactory	Positive	HSIL	High-grade squamous	
P02324	2018	2018-Q4	2018-10	Pre-pandemic	Cytology (Pap)	35-39	39	Not performed	Satisfactory	NILM	NILM	Negative (NILM)	
P02325	2018	2018-Q4	2018-10	Pre-pandemic	Cytology (Pap)	35-39	38	Not performed	Satisfactory	Positive	ASC-US	Low-grade / equivocal squamous	
P02326	2018	2018-Q4	2018-10	Pre-pandemic	Cytology (Pap)	45-49	49	Not performed	Satisfactory	NILM	NILM	Negative (NILM)	
P02327	2018	2018-Q4	2018-10	Pre-pandemic	Cytology (Pap)	40-44	40	Not performed	Satisfactory	NILM	NILM	Negative (NILM)	
P02328	2018	2018-Q4	2018-10	Pre-pandemic	Cytology (Pap)	45-49	45	Not performed	Unsatisfactory	Unsatisfactory	Unsatisfactory	Unsatisfactory	
P02329	2018	2018-Q4	2018-10	Pre-pandemic	Cytology (Pap)	40-44	44	Not performed	Satisfactory	NILM	NILM	Negative (NILM)	
P02330	2018	2018-Q4	2018-10	Pre-pandemic	Cytology (Pap)	60-64	62	Not performed	Satisfactory	NILM	NILM	Negative (NILM)	
P02331	2018	2018-Q4	2018-10	Pre-pandemic	Cytology (Pap)	30-34	34	Not performed	Satisfactory	Positive	ASC-US	Low-grade / equivocal squamous	
P02332	2018	2018-Q4	2018-10	Pre-pandemic	Cytology (Pap)	45-49	46	Not performed	Satisfactory	NILM	NILM	Negative (NILM)	
P02333	2018	2018-Q4	2018-10	Pre-pandemic	Cytology (Pap)	30-34	31	Not performed	Satisfactory	NILM	NILM	Negative (NILM)	
P02334	2018	2018-Q4	2018-10	Pre-pandemic	Cytology (Pap)	55-59	57	Not performed	Satisfactory	NILM	NILM	Negative (NILM)	
P02335	2018	2018-Q4	2018-10	Pre-pandemic	Cytology (Pap)	30-34	33	Not performed	Satisfactory	NILM	NILM	Negative (NILM)	
P02336	2018	2018-Q4	2018-10	Pre-pandemic	Cytology (Pap)	35-39	38	Not performed	Satisfactory	NILM	NILM	Negative (NILM)	
P02337	2018	2018-Q4	2018-10	Pre-pandemic	Cytology (Pap)	60-64	61	Not performed	Satisfactory	NILM	NILM	Negative (NILM)	
P02338	2018	2018-Q4	2018-10	Pre-pandemic	Cytology (Pap)	40-44	40	Not performed	Satisfactory	NILM	NILM	Negative (NILM)	
P02339	2018	2018-Q4	2018-10	Pre-pandemic	Cytology (Pap)	35-39	35	Not performed	Satisfactory	NILM	NILM	Negative (NILM)	
P02340	2018	2018-Q4	2018-10	Pre-pandemic	Cytology (Pap)	40-44	44	Not performed	Satisfactory	NILM	NILM	Negative (NILM)	
P02341	2018	2018-Q4	2018-10	Pre-pandemic	Cytology (Pap)	35-39	37	Not performed	Satisfactory	NILM	NILM	Negative (NILM)	
P02342	2018	2018-Q4	2018-10	Pre-pandemic	Cytology (Pap)	30-34	30	Not performed	Satisfactory	Positive	HSIL	High-grade squamous	
P02343	2018	2018-Q4	2018-10	Pre-pandemic	Cytology (Pap)	25-29	27	Not performed	Satisfactory	NILM	NILM	Negative (NILM)	
P02344	2018	2018-Q4	2018-10	Pre-pandemic	Cytology (Pap)	40-44	41	Not performed	Satisfactory	NILM	NILM	Negative (NILM)	
P02345	2018	2018-Q4	2018-10	Pre-pandemic	Cytology (Pap)	40-44	44	Not performed	Satisfactory	NILM	NILM	Negative (NILM)	
P02346	2018	2018-Q4	2018-10	Pre-pandemic	Cytology (Pap)	45-49	47	Not performed	Satisfactory	NILM	NILM	Negative (NILM)	
P02347	2018	2018-Q4	2018-10	Pre-pandemic	Cytology (Pap)	50-54	54	Not performed	Satisfactory	NILM	NILM	Negative (NILM)	
P02348	2018	2018-Q4	2018-10	Pre-pandemic	Cytology (Pap)	25-29	27	Not performed	Satisfactory	NILM	NILM	Negative (NILM)	
P02349	2018	2018-Q4	2018-10	Pre-pandemic	Cytology (Pap)	45-49	47	Not performed	Satisfactory	NILM	NILM	Negative (NILM)	
P02350	2018	2018-Q4	2018-10	Pre-pandemic	Cytology (Pap)	45-49	49	Not performed	Satisfactory	NILM	NILM	Negative (NILM)	
P02351	2018	2018-Q4	2018-10	Pre-pandemic	Cytology (Pap)	Not recorded		Not performed	Satisfactory	NILM	NILM	Negative (NILM)	
P02352	2018	2018-Q4	2018-10	Pre-pandemic	Cytology (Pap)	55-59	59	Not performed	Satisfactory	NILM	NILM	Negative (NILM)	
P02353	2018	2018-Q4	2018-10	Pre-pandemic	Cytology (Pap)	55-59	55	Not performed	Satisfactory	NILM	NILM	Negative (NILM)	Candida
P02354	2018	2018-Q4	2018-10	Pre-pandemic	Cytology (Pap)	35-39	36	Not performed	Satisfactory	NILM	NILM	Negative (NILM)	
P02355	2018	2018-Q4	2018-10	Pre-pandemic	Cytology (Pap)	50-54	51	Not performed	Satisfactory	NILM	NILM	Negative (NILM)	
P02356	2018	2018-Q4	2018-10	Pre-pandemic	Cytology (Pap)	35-39	35	Not performed	Satisfactory	NILM	NILM	Negative (NILM)	
P02357	2018	2018-Q4	2018-10	Pre-pandemic	Cytology (Pap)	45-49	46	Not performed	Satisfactory	NILM	NILM	Negative (NILM)	
P02358	2018	2018-Q4	2018-10	Pre-pandemic	Cytology (Pap)	50-54	54	Not performed	Satisfactory	NILM	NILM	Negative (NILM)	
P02359	2018	2018-Q4	2018-10	Pre-pandemic	Cytology (Pap)	25-29	27	Not performed	Satisfactory	NILM	NILM	Negative (NILM)	
P02360	2018	2018-Q4	2018-10	Pre-pandemic	Cytology (Pap)	60-64	61	Not performed	Satisfactory	NILM	NILM	Negative (NILM)	
P02361	2018	2018-Q4	2018-10	Pre-pandemic	Cytology (Pap)	40-44	44	Not performed	Satisfactory	NILM	NILM	Negative (NILM)	
P02362	2018	2018-Q4	2018-10	Pre-pandemic	Cytology (Pap)	30-34	34	Not performed	Satisfactory	NILM	NILM	Negative (NILM)	
P02363	2018	2018-Q4	2018-10	Pre-pandemic	Cytology (Pap)	40-44	44	Not performed	Satisfactory	NILM	NILM	Negative (NILM)	Candida
P02364	2018	2018-Q4	2018-10	Pre-pandemic	Cytology (Pap)	25-29	25	Not performed	Satisfactory	NILM	NILM	Negative (NILM)	
P02365	2018	2018-Q4	2018-11	Pre-pandemic	Cytology (Pap)	45-49	46	Not performed	Satisfactory	Positive	LSIL	Low-grade / equivocal squamous	
P02366	2018	2018-Q4	2018-11	Pre-pandemic	Cytology (Pap)	60-64	63	Not performed	Satisfactory	NILM	NILM	Negative (NILM)	
P02367	2018	2018-Q4	2018-11	Pre-pandemic	Cytology (Pap)	55-59	58	Not performed	Satisfactory	NILM	NILM	Negative (NILM)	Trichomonas
P02368	2018	2018-Q4	2018-11	Pre-pandemic	Cytology (Pap)	40-44	42	Not performed	Satisfactory	Positive	ASC-US	Low-grade / equivocal squamous	Candida
P02369	2018	2018-Q4	2018-11	Pre-pandemic	Cytology (Pap)	35-39	39	Not performed	Satisfactory	NILM	NILM	Negative (NILM)	
P02370	2018	2018-Q4	2018-11	Pre-pandemic	Cytology (Pap)	40-44	42	Not performed	Satisfactory	NILM	NILM	Negative (NILM)	
P02371	2018	2018-Q4	2018-11	Pre-pandemic	Cytology (Pap)	40-44	42	Not performed	Satisfactory	NILM	NILM	Negative (NILM)	Trichomonas
P02372	2018	2018-Q4	2018-11	Pre-pandemic	Cytology (Pap)	40-44	43	Not performed	Satisfactory	NILM	NILM	Negative (NILM)	
P02373	2018	2018-Q4	2018-11	Pre-pandemic	Cytology (Pap)	55-59	57	Not performed	Satisfactory	NILM	NILM	Negative (NILM)	
P02374	2018	2018-Q4	2018-11	Pre-pandemic	Cytology (Pap)	30-34	33	Not performed	Satisfactory	Positive	HSIL	High-grade squamous	
P02375	2018	2018-Q4	2018-11	Pre-pandemic	Cytology (Pap)	60-64	63	Not performed	Satisfactory	NILM	NILM	Negative (NILM)	
P02376	2018	2018-Q4	2018-11	Pre-pandemic	Cytology (Pap)	35-39	39	Not performed	Satisfactory	NILM	NILM	Negative (NILM)	
P02377	2018	2018-Q4	2018-11	Pre-pandemic	Cytology (Pap)	25-29	27	Not performed	Satisfactory	NILM	NILM	Negative (NILM)	
P02378	2018	2018-Q4	2018-11	Pre-pandemic	Cytology (Pap)	Not recorded		Not performed	Satisfactory	NILM	NILM	Negative (NILM)	
P02379	2018	2018-Q4	2018-11	Pre-pandemic	Cytology (Pap)	25-29	27	Not performed	Satisfactory	NILM	NILM	Negative (NILM)	
P02380	2018	2018-Q4	2018-11	Pre-pandemic	Cytology (Pap)	25-29	29	Not performed	Satisfactory	NILM	NILM	Negative (NILM)	
P02381	2018	2018-Q4	2018-11	Pre-pandemic	Cytology (Pap)	30-34	31	Not performed	Satisfactory	NILM	NILM	Negative (NILM)	
P02382	2018	2018-Q4	2018-11	Pre-pandemic	Cytology (Pap)	Not recorded		Not performed	Satisfactory	NILM	NILM	Negative (NILM)	
P02383	2018	2018-Q4	2018-11	Pre-pandemic	Cytology (Pap)	Not recorded		Not performed	Satisfactory	NILM	NILM	Negative (NILM)	
P02384	2018	2018-Q4	2018-11	Pre-pandemic	Cytology (Pap)	40-44	41	Not performed	Satisfactory	NILM	NILM	Negative (NILM)	
P02385	2018	2018-Q4	2018-11	Pre-pandemic	Cytology (Pap)	40-44	42	Not performed	Satisfactory	NILM	NILM	Negative (NILM)	
P02386	2018	2018-Q4	2018-11	Pre-pandemic	Cytology (Pap)	40-44	44	Not performed	Satisfactory	NILM	NILM	Negative (NILM)	
P02387	2018	2018-Q4	2018-11	Pre-pandemic	Cytology (Pap)	55-59	58	Not performed	Satisfactory	NILM	NILM	Negative (NILM)	
P02388	2018	2018-Q4	2018-11	Pre-pandemic	Cytology (Pap)	55-59	55	Not performed	Satisfactory	NILM	NILM	Negative (NILM)	
P02389	2018	2018-Q4	2018-11	Pre-pandemic	Cytology (Pap)	25-29	28	Not performed	Satisfactory	Positive	ASC-US	Low-grade / equivocal squamous	
P02390	2018	2018-Q4	2018-11	Pre-pandemic	Cytology (Pap)	60-64	62	Not performed	Satisfactory	NILM	NILM	Negative (NILM)	
P02391	2018	2018-Q4	2018-11	Pre-pandemic	Cytology (Pap)	30-34	33	Not performed	Satisfactory	NILM	NILM	Negative (NILM)	
P02392	2018	2018-Q4	2018-11	Pre-pandemic	Cytology (Pap)	25-29	28	Not performed	Satisfactory	NILM	NILM	Negative (NILM)	
P02393	2018	2018-Q4	2018-11	Pre-pandemic	Cytology (Pap)	30-34	30	Not performed	Satisfactory	NILM	NILM	Negative (NILM)	
P02394	2018	2018-Q4	2018-11	Pre-pandemic	Cytology (Pap)	45-49	46	Not performed	Satisfactory	NILM	NILM	Negative (NILM)	
P02395	2018	2018-Q4	2018-11	Pre-pandemic	Cytology (Pap)	35-39	38	Not performed	Satisfactory	NILM	NILM	Negative (NILM)	
P02396	2018	2018-Q4	2018-11	Pre-pandemic	Cytology (Pap)	45-49	46	Not performed	Satisfactory	NILM	NILM	Negative (NILM)	
P02397	2018	2018-Q4	2018-11	Pre-pandemic	Cytology (Pap)	55-59	58	Not performed	Satisfactory	NILM	NILM	Negative (NILM)	
P02398	2018	2018-Q4	2018-11	Pre-pandemic	Cytology (Pap)	25-29	29	Not performed	Satisfactory	NILM	NILM	Negative (NILM)	Gardnerella

patient_id	year	quarter	month	period	primary_test	age_group	age_years	hpv_result	adequacy	result	bethesda	grade	infection
P02399	2018	2018-Q4	2018-11	Pre-pandemic	Cytology (Pap)	Not recorded		Not performed	Satisfactory	NILM	NILM	Negative (NILM)	
P02400	2018	2018-Q4	2018-11	Pre-pandemic	Cytology (Pap)	30-34	31	Not performed	Satisfactory	NILM	NILM	Negative (NILM)	
P02401	2018	2018-Q4	2018-11	Pre-pandemic	Cytology (Pap)	40-44	43	Not performed	Satisfactory	NILM	NILM	Negative (NILM)	
P02402	2018	2018-Q4	2018-11	Pre-pandemic	Cytology (Pap)	25-29	29	Not performed	Satisfactory	NILM	NILM	Negative (NILM)	
P02403	2018	2018-Q4	2018-11	Pre-pandemic	Cytology (Pap)	55-59	57	Not performed	Satisfactory	Positive	ASC-H	High-grade squamous	
P02404	2018	2018-Q4	2018-11	Pre-pandemic	Cytology (Pap)	35-39	37	Not performed	Satisfactory	NILM	NILM	Negative (NILM)	
P02405	2018	2018-Q4	2018-11	Pre-pandemic	Cytology (Pap)	40-44	43	Not performed	Satisfactory	NILM	NILM	Negative (NILM)	
P02406	2018	2018-Q4	2018-11	Pre-pandemic	Cytology (Pap)	25-29	28	Not performed	Satisfactory	Positive	ASC-US	Low-grade / equivocal squamous	
P02407	2018	2018-Q4	2018-11	Pre-pandemic	Cytology (Pap)	50-54	52	Not performed	Satisfactory	NILM	NILM	Negative (NILM)	
P02408	2018	2018-Q4	2018-11	Pre-pandemic	Cytology (Pap)	55-59	57	Not performed	Satisfactory	NILM	NILM	Negative (NILM)	
P02409	2018	2018-Q4	2018-11	Pre-pandemic	Cytology (Pap)	60-64	61	Not performed	Satisfactory	NILM	NILM	Negative (NILM)	
P02410	2018	2018-Q4	2018-11	Pre-pandemic	Cytology (Pap)	45-49	46	Not performed	Satisfactory	NILM	NILM	Negative (NILM)	Candida
P02411	2018	2018-Q4	2018-11	Pre-pandemic	Cytology (Pap)	50-54	51	Not performed	Satisfactory	NILM	NILM	Negative (NILM)	
P02412	2018	2018-Q4	2018-11	Pre-pandemic	Cytology (Pap)	30-34	34	Not performed	Satisfactory	NILM	NILM	Negative (NILM)	
P02413	2018	2018-Q4	2018-11	Pre-pandemic	Cytology (Pap)	40-44	43	Not performed	Satisfactory	NILM	NILM	Negative (NILM)	Candida
P02414	2018	2018-Q4	2018-11	Pre-pandemic	Cytology (Pap)	40-44	44	Not performed	Satisfactory	NILM	NILM	Negative (NILM)	
P02415	2018	2018-Q4	2018-11	Pre-pandemic	Cytology (Pap)	Not recorded		Not performed	Satisfactory	NILM	NILM	Negative (NILM)	
P02416	2018	2018-Q4	2018-11	Pre-pandemic	Cytology (Pap)	30-34	33	Not performed	Satisfactory	NILM	NILM	Negative (NILM)	
P02417	2018	2018-Q4	2018-11	Pre-pandemic	Cytology (Pap)	60-64	64	Not performed	Satisfactory	NILM	NILM	Negative (NILM)	
P02418	2018	2018-Q4	2018-11	Pre-pandemic	Cytology (Pap)	Not recorded		Not performed	Satisfactory	NILM	NILM	Negative (NILM)	
P02419	2018	2018-Q4	2018-11	Pre-pandemic	Cytology (Pap)	35-39	39	Not performed	Satisfactory	Positive	ASC-H	High-grade squamous	
P02420	2018	2018-Q4	2018-11	Pre-pandemic	Cytology (Pap)	55-59	56	Not performed	Satisfactory	Positive	LSIL	Low-grade / equivocal squamous	
P02421	2018	2018-Q4	2018-11	Pre-pandemic	Cytology (Pap)	30-34	33	Not performed	Satisfactory	NILM	NILM	Negative (NILM)	
P02422	2018	2018-Q4	2018-11	Pre-pandemic	Cytology (Pap)	Not recorded		Not performed	Satisfactory	NILM	NILM	Negative (NILM)	
P02423	2018	2018-Q4	2018-11	Pre-pandemic	Cytology (Pap)	30-34	30	Not performed	Satisfactory	NILM	NILM	Negative (NILM)	
P02424	2018	2018-Q4	2018-11	Pre-pandemic	Cytology (Pap)	40-44	41	Not performed	Satisfactory	Positive	ASC-US	Low-grade / equivocal squamous	
P02425	2018	2018-Q4	2018-11	Pre-pandemic	Cytology (Pap)	40-44	44	Not performed	Satisfactory	NILM	NILM	Negative (NILM)	
P02426	2018	2018-Q4	2018-11	Pre-pandemic	Cytology (Pap)	30-34	33	Not performed	Satisfactory	Positive	ASC-US	Low-grade / equivocal squamous	
P02427	2018	2018-Q4	2018-11	Pre-pandemic	Cytology (Pap)	55-59	57	Not performed	Satisfactory	Positive	ASC-US	Low-grade / equivocal squamous	
P02428	2018	2018-Q4	2018-11	Pre-pandemic	Cytology (Pap)	40-44	40	Not performed	Satisfactory	NILM	NILM	Negative (NILM)	
P02429	2018	2018-Q4	2018-11	Pre-pandemic	Cytology (Pap)	50-54	54	Not performed	Satisfactory	NILM	NILM	Negative (NILM)	
P02430	2018	2018-Q4	2018-11	Pre-pandemic	Cytology (Pap)	40-44	40	Not performed	Satisfactory	NILM	NILM	Negative (NILM)	
P02431	2018	2018-Q4	2018-11	Pre-pandemic	Cytology (Pap)	55-59	58	Not performed	Satisfactory	NILM	NILM	Negative (NILM)	
P02432	2018	2018-Q4	2018-11	Pre-pandemic	Cytology (Pap)	40-44	44	Not performed	Satisfactory	NILM	NILM	Negative (NILM)	
P02433	2018	2018-Q4	2018-11	Pre-pandemic	Cytology (Pap)	45-49	48	Not performed	Satisfactory	NILM	NILM	Negative (NILM)	
P02434	2018	2018-Q4	2018-11	Pre-pandemic	Cytology (Pap)	55-59	59	Not performed	Satisfactory	Positive	AGC	Glandular	
P02435	2018	2018-Q4	2018-11	Pre-pandemic	Cytology (Pap)	55-59	58	Not performed	Satisfactory	NILM	NILM	Negative (NILM)	
P02436	2018	2018-Q4	2018-11	Pre-pandemic	Cytology (Pap)	Not recorded		Not performed	Satisfactory	NILM	NILM	Negative (NILM)	
P02437	2018	2018-Q4	2018-11	Pre-pandemic	Cytology (Pap)	Not recorded		Not performed	Satisfactory	NILM	NILM	Negative (NILM)	
P02438	2018	2018-Q4	2018-11	Pre-pandemic	Cytology (Pap)	40-44	44	Not performed	Satisfactory	NILM	NILM	Negative (NILM)	
P02439	2018	2018-Q4	2018-11	Pre-pandemic	Cytology (Pap)	40-44	44	Not performed	Satisfactory	NILM	NILM	Negative (NILM)	
P02440	2018	2018-Q4	2018-11	Pre-pandemic	Cytology (Pap)	40-44	43	Not performed	Satisfactory	NILM	NILM	Negative (NILM)	
P02441	2018	2018-Q4	2018-11	Pre-pandemic	Cytology (Pap)	55-59	55	Not performed	Satisfactory	NILM	NILM	Negative (NILM)	
P02442	2018	2018-Q4	2018-11	Pre-pandemic	Cytology (Pap)	40-44	44	Not performed	Satisfactory	NILM	NILM	Negative (NILM)	Candida
P02443	2018	2018-Q4	2018-11	Pre-pandemic	Cytology (Pap)	60-64	61	Not performed	Satisfactory	NILM	NILM	Negative (NILM)	
P02444	2018	2018-Q4	2018-11	Pre-pandemic	Cytology (Pap)	30-34	32	Not performed	Satisfactory	NILM	NILM	Negative (NILM)	
P02445	2018	2018-Q4	2018-11	Pre-pandemic	Cytology (Pap)	50-54	50	Not performed	Satisfactory	Positive	LSIL	Low-grade / equivocal squamous	
P02446	2018	2018-Q4	2018-11	Pre-pandemic	Cytology (Pap)	50-54	54	Not performed	Satisfactory	Positive	LSIL	Low-grade / equivocal squamous	
P02447	2018	2018-Q4	2018-11	Pre-pandemic	Cytology (Pap)	30-34	34	Not performed	Satisfactory	NILM	NILM	Negative (NILM)	
P02448	2018	2018-Q4	2018-11	Pre-pandemic	Cytology (Pap)	35-39	36	Not performed	Satisfactory	NILM	NILM	Negative (NILM)	
P02449	2018	2018-Q4	2018-11	Pre-pandemic	Cytology (Pap)	25-29	27	Not performed	Satisfactory	NILM	NILM	Negative (NILM)	
P02450	2018	2018-Q4	2018-11	Pre-pandemic	Cytology (Pap)	30-34	32	Not performed	Satisfactory	NILM	NILM	Negative (NILM)	
P02451	2018	2018-Q4	2018-11	Pre-pandemic	Cytology (Pap)	35-39	37	Not performed	Satisfactory	NILM	NILM	Negative (NILM)	
P02452	2018	2018-Q4	2018-11	Pre-pandemic	Cytology (Pap)	40-44	41	Not performed	Satisfactory	NILM	NILM	Negative (NILM)	
P02453	2018	2018-Q4	2018-11	Pre-pandemic	Cytology (Pap)	40-44	40	Not performed	Satisfactory	NILM	NILM	Negative (NILM)	
P02454	2018	2018-Q4	2018-11	Pre-pandemic	Cytology (Pap)	25-29	26	Not performed	Satisfactory	Positive	LSIL	Low-grade / equivocal squamous	
P02455	2018	2018-Q4	2018-11	Pre-pandemic	Cytology (Pap)	35-39	36	Not performed	Satisfactory	NILM	NILM	Negative (NILM)	
P02456	2018	2018-Q4	2018-11	Pre-pandemic	Cytology (Pap)	40-44	43	Not performed	Satisfactory	NILM	NILM	Negative (NILM)	
P02457	2018	2018-Q4	2018-11	Pre-pandemic	Cytology (Pap)	Not recorded		Not performed	Satisfactory	NILM	NILM	Negative (NILM)	
P02458	2018	2018-Q4	2018-11	Pre-pandemic	Cytology (Pap)	55-59	56	Not performed	Satisfactory	Positive	LSIL	Low-grade / equivocal squamous	
P02459	2018	2018-Q4	2018-11	Pre-pandemic	Cytology (Pap)	45-49	49	Not performed	Satisfactory	NILM	NILM	Negative (NILM)	
P02460	2018	2018-Q4	2018-11	Pre-pandemic	Cytology (Pap)	45-49	47	Not performed	Satisfactory	NILM	NILM	Negative (NILM)	
P02461	2018	2018-Q4	2018-11	Pre-pandemic	Cytology (Pap)	40-44	42	Not performed	Satisfactory	NILM	NILM	Negative (NILM)	
P02462	2018	2018-Q4	2018-11	Pre-pandemic	Cytology (Pap)	30-34	33	Not performed	Satisfactory	NILM	NILM	Negative (NILM)	
P02463	2018	2018-Q4	2018-11	Pre-pandemic	Cytology (Pap)	30-34	34	Not performed	Satisfactory	NILM	NILM	Negative (NILM)	
P02464	2018	2018-Q4	2018-11	Pre-pandemic	Cytology (Pap)	45-49	49	Not performed	Satisfactory	NILM	NILM	Negative (NILM)	
P02465	2018	2018-Q4	2018-11	Pre-pandemic	Cytology (Pap)	30-34	31	Not performed	Satisfactory	Positive	LSIL	Low-grade / equivocal squamous	
P02466	2018	2018-Q4	2018-11	Pre-pandemic	Cytology (Pap)	30-34	34	Not performed	Satisfactory	NILM	NILM	Negative (NILM)	
P02467	2018	2018-Q4	2018-11	Pre-pandemic	Cytology (Pap)	45-49	45	Not performed	Satisfactory	Positive	LSIL	Low-grade / equivocal squamous	
P02468	2018	2018-Q4	2018-11	Pre-pandemic	Cytology (Pap)	55-59	55	Not performed	Satisfactory	Positive	HSIL	High-grade squamous	
P02469	2018	2018-Q4	2018-11	Pre-pandemic	Cytology (Pap)	35-39	38	Not performed	Satisfactory	NILM	NILM	Negative (NILM)	
P02470	2018	2018-Q4	2018-11	Pre-pandemic	Cytology (Pap)	Not recorded		Not performed	Satisfactory	NILM	NILM	Negative (NILM)	
P02471	2018	2018-Q4	2018-11	Pre-pandemic	Cytology (Pap)	55-59	58	Not performed	Satisfactory	Positive	ASC-US	Low-grade / equivocal squamous	
P02472	2018	2018-Q4	2018-11	Pre-pandemic	Cytology (Pap)	Not recorded		Not performed	Satisfactory	NILM	NILM	Negative (NILM)	
P02473	2018	2018-Q4	2018-11	Pre-pandemic	Cytology (Pap)	40-44	42	Not performed	Satisfactory	NILM	NILM	Negative (NILM)	
P02474	2018	2018-Q4	2018-11	Pre-pandemic	Cytology (Pap)	40-44	42	Not performed	Satisfactory	NILM	NILM	Negative (NILM)	
P02475	2018	2018-Q4	2018-11	Pre-pandemic	Cytology (Pap)	60-64	61	Not performed	Satisfactory	NILM	NILM	Negative (NILM)	
P02476	2018	2018-Q4	2018-11	Pre-pandemic	Cytology (Pap)	25-29	25	Not performed	Satisfactory	NILM	NILM	Negative (NILM)	
P02477	2018	2018-Q4	2018-11	Pre-pandemic	Cytology (Pap)	40-44	43	Not performed	Satisfactory	Positive	AGC	Glandular	
P02478	2018	2018-Q4	2018-11	Pre-pandemic	Cytology (Pap)	30-34	32	Not performed	Satisfactory	NILM	NILM	Negative (NILM)	

patient_id	year	quarter	month	period	primary_test	age_group	age_years	hpv_result	adequacy	result	bethesda	grade	infection
P02479	2018	2018-Q4	2018-11	Pre-pandemic	Cytology (Pap)	55-59	56	Not performed	Satisfactory	NILM	NILM	Negative (NILM)	
P02480	2018	2018-Q4	2018-11	Pre-pandemic	Cytology (Pap)	50-54	50	Not performed	Satisfactory	Positive	HSIL	High-grade squamous	
P02481	2018	2018-Q4	2018-11	Pre-pandemic	Cytology (Pap)	60-64	62	Not performed	Satisfactory	NILM	NILM	Negative (NILM)	
P02482	2018	2018-Q4	2018-11	Pre-pandemic	Cytology (Pap)	35-39	37	Not performed	Satisfactory	NILM	NILM	Negative (NILM)	Gardnerella
P02483	2018	2018-Q4	2018-11	Pre-pandemic	Cytology (Pap)	40-44	41	Not performed	Unsatisfactory	Unsatisfactory	Unsatisfactory	Unsatisfactory	
P02484	2018	2018-Q4	2018-11	Pre-pandemic	Cytology (Pap)	Not recorded		Not performed	Satisfactory	NILM	NILM	Negative (NILM)	
P02485	2018	2018-Q4	2018-11	Pre-pandemic	Cytology (Pap)	40-44	40	Not performed	Satisfactory	NILM	NILM	Negative (NILM)	
P02486	2018	2018-Q4	2018-11	Pre-pandemic	Cytology (Pap)	35-39	37	Not performed	Satisfactory	NILM	NILM	Negative (NILM)	
P02487	2018	2018-Q4	2018-11	Pre-pandemic	Cytology (Pap)	50-54	51	Not performed	Satisfactory	NILM	NILM	Negative (NILM)	
P02488	2018	2018-Q4	2018-11	Pre-pandemic	Cytology (Pap)	45-49	49	Not performed	Satisfactory	NILM	NILM	Negative (NILM)	
P02489	2018	2018-Q4	2018-11	Pre-pandemic	Cytology (Pap)	45-49	48	Not performed	Satisfactory	NILM	NILM	Negative (NILM)	
P02490	2018	2018-Q4	2018-11	Pre-pandemic	Cytology (Pap)	45-49	49	Not performed	Satisfactory	NILM	NILM	Negative (NILM)	
P02491	2018	2018-Q4	2018-11	Pre-pandemic	Cytology (Pap)	35-39	37	Not performed	Satisfactory	NILM	NILM	Negative (NILM)	
P02492	2018	2018-Q4	2018-11	Pre-pandemic	Cytology (Pap)	50-54	51	Not performed	Satisfactory	NILM	NILM	Negative (NILM)	
P02493	2018	2018-Q4	2018-11	Pre-pandemic	Cytology (Pap)	25-29	25	Not performed	Satisfactory	NILM	NILM	Negative (NILM)	
P02494	2018	2018-Q4	2018-11	Pre-pandemic	Cytology (Pap)	25-29	28	Not performed	Satisfactory	NILM	NILM	Negative (NILM)	
P02495	2018	2018-Q4	2018-11	Pre-pandemic	Cytology (Pap)	25-29	26	Not performed	Unsatisfactory	Unsatisfactory	Unsatisfactory	Unsatisfactory	
P02496	2018	2018-Q4	2018-11	Pre-pandemic	Cytology (Pap)	30-34	34	Not performed	Satisfactory	NILM	NILM	Negative (NILM)	
P02497	2018	2018-Q4	2018-11	Pre-pandemic	Cytology (Pap)	35-39	36	Not performed	Satisfactory	NILM	NILM	Negative (NILM)	
P02498	2018	2018-Q4	2018-11	Pre-pandemic	Cytology (Pap)	55-59	55	Not performed	Satisfactory	NILM	NILM	Negative (NILM)	
P02499	2018	2018-Q4	2018-11	Pre-pandemic	Cytology (Pap)	45-49	48	Not performed	Satisfactory	NILM	NILM	Negative (NILM)	
P02500	2018	2018-Q4	2018-11	Pre-pandemic	Cytology (Pap)	40-44	43	Not performed	Satisfactory	NILM	NILM	Negative (NILM)	
P02501	2018	2018-Q4	2018-11	Pre-pandemic	Cytology (Pap)	Not recorded		Not performed	Satisfactory	NILM	NILM	Negative (NILM)	
P02502	2018	2018-Q4	2018-11	Pre-pandemic	Cytology (Pap)	50-54	50	Not performed	Satisfactory	NILM	NILM	Negative (NILM)	
P02503	2018	2018-Q4	2018-11	Pre-pandemic	Cytology (Pap)	35-39	36	Not performed	Satisfactory	Positive	LSIL	Low-grade / equivocal squamous	
P02504	2018	2018-Q4	2018-11	Pre-pandemic	Cytology (Pap)	25-29	25	Not performed	Satisfactory	NILM	NILM	Negative (NILM)	
P02505	2018	2018-Q4	2018-11	Pre-pandemic	Cytology (Pap)	40-44	43	Not performed	Satisfactory	NILM	NILM	Negative (NILM)	
P02506	2018	2018-Q4	2018-11	Pre-pandemic	Cytology (Pap)	40-44	40	Not performed	Satisfactory	NILM	NILM	Negative (NILM)	
P02507	2018	2018-Q4	2018-11	Pre-pandemic	Cytology (Pap)	50-54	51	Not performed	Satisfactory	NILM	NILM	Negative (NILM)	
P02508	2018	2018-Q4	2018-11	Pre-pandemic	Cytology (Pap)	50-54	53	Not performed	Satisfactory	NILM	NILM	Negative (NILM)	
P02509	2018	2018-Q4	2018-11	Pre-pandemic	Cytology (Pap)	30-34	34	Not performed	Satisfactory	NILM	NILM	Negative (NILM)	
P02510	2018	2018-Q4	2018-11	Pre-pandemic	Cytology (Pap)	30-34	32	Not performed	Satisfactory	NILM	NILM	Negative (NILM)	
P02511	2018	2018-Q4	2018-11	Pre-pandemic	Cytology (Pap)	40-44	44	Not performed	Satisfactory	NILM	NILM	Negative (NILM)	
P02512	2018	2018-Q4	2018-11	Pre-pandemic	Cytology (Pap)	40-44	42	Not performed	Satisfactory	NILM	NILM	Negative (NILM)	
P02513	2018	2018-Q4	2018-11	Pre-pandemic	Cytology (Pap)	30-34	34	Not performed	Satisfactory	NILM	NILM	Negative (NILM)	
P02514	2018	2018-Q4	2018-11	Pre-pandemic	Cytology (Pap)	40-44	44	Not performed	Satisfactory	NILM	NILM	Negative (NILM)	
P02515	2018	2018-Q4	2018-11	Pre-pandemic	Cytology (Pap)	40-44	44	Not performed	Satisfactory	NILM	NILM	Negative (NILM)	
P02516	2018	2018-Q4	2018-11	Pre-pandemic	Cytology (Pap)	Not recorded		Not performed	Satisfactory	NILM	NILM	Negative (NILM)	
P02517	2018	2018-Q4	2018-11	Pre-pandemic	Cytology (Pap)	Not recorded		Not performed	Satisfactory	NILM	NILM	Negative (NILM)	
P02518	2018	2018-Q4	2018-11	Pre-pandemic	Cytology (Pap)	50-54	53	Not performed	Satisfactory	NILM	NILM	Negative (NILM)	
P02519	2018	2018-Q4	2018-11	Pre-pandemic	Cytology (Pap)	35-39	38	Not performed	Satisfactory	Positive	ASC-US	Low-grade / equivocal squamous	
P02520	2018	2018-Q4	2018-11	Pre-pandemic	Cytology (Pap)	45-49	47	Not performed	Satisfactory	Positive	ASC-US	Low-grade / equivocal squamous	
P02521	2018	2018-Q4	2018-11	Pre-pandemic	Cytology (Pap)	60-64	63	Not performed	Satisfactory	Positive	LSIL	Low-grade / equivocal squamous	
P02522	2018	2018-Q4	2018-11	Pre-pandemic	Cytology (Pap)	40-44	41	Not performed	Satisfactory	NILM	NILM	Negative (NILM)	
P02523	2018	2018-Q4	2018-11	Pre-pandemic	Cytology (Pap)	45-49	45	Not performed	Satisfactory	NILM	NILM	Negative (NILM)	
P02524	2018	2018-Q4	2018-11	Pre-pandemic	Cytology (Pap)	60-64	61	Not performed	Satisfactory	Positive	LSIL	Low-grade / equivocal squamous	
P02525	2018	2018-Q4	2018-11	Pre-pandemic	Cytology (Pap)	30-34	33	Not performed	Satisfactory	NILM	NILM	Negative (NILM)	
P02526	2018	2018-Q4	2018-11	Pre-pandemic	Cytology (Pap)	40-44	44	Not performed	Satisfactory	NILM	NILM	Negative (NILM)	
P02527	2018	2018-Q4	2018-11	Pre-pandemic	Cytology (Pap)	35-39	39	Not performed	Satisfactory	NILM	NILM	Negative (NILM)	
P02528	2018	2018-Q4	2018-11	Pre-pandemic	Cytology (Pap)	50-54	50	Not performed	Satisfactory	NILM	NILM	Negative (NILM)	
P02529	2018	2018-Q4	2018-11	Pre-pandemic	Cytology (Pap)	40-44	41	Not performed	Satisfactory	NILM	NILM	Negative (NILM)	
P02530	2018	2018-Q4	2018-11	Pre-pandemic	Cytology (Pap)	40-44	44	Not performed	Satisfactory	Positive	ASC-US	Low-grade / equivocal squamous	
P02531	2018	2018-Q4	2018-11	Pre-pandemic	Cytology (Pap)	50-54	54	Not performed	Satisfactory	NILM	NILM	Negative (NILM)	
P02532	2018	2018-Q4	2018-11	Pre-pandemic	Cytology (Pap)	60-64	61	Not performed	Satisfactory	NILM	NILM	Negative (NILM)	
P02533	2018	2018-Q4	2018-11	Pre-pandemic	Cytology (Pap)	45-49	47	Not performed	Satisfactory	NILM	NILM	Negative (NILM)	
P02534	2018	2018-Q4	2018-11	Pre-pandemic	Cytology (Pap)	45-49	47	Not performed	Satisfactory	NILM	NILM	Negative (NILM)	
P02535	2018	2018-Q4	2018-11	Pre-pandemic	Cytology (Pap)	60-64	63	Not performed	Satisfactory	NILM	NILM	Negative (NILM)	
P02536	2018	2018-Q4	2018-11	Pre-pandemic	Cytology (Pap)	45-49	46	Not performed	Satisfactory	NILM	NILM	Negative (NILM)	
P02537	2018	2018-Q4	2018-11	Pre-pandemic	Cytology (Pap)	30-34	32	Not performed	Satisfactory	NILM	NILM	Negative (NILM)	
P02538	2018	2018-Q4	2018-11	Pre-pandemic	Cytology (Pap)	45-49	46	Not performed	Satisfactory	NILM	NILM	Negative (NILM)	
P02539	2018	2018-Q4	2018-11	Pre-pandemic	Cytology (Pap)	55-59	59	Not performed	Satisfactory	NILM	NILM	Negative (NILM)	
P02540	2018	2018-Q4	2018-11	Pre-pandemic	Cytology (Pap)	50-54	53	Not performed	Satisfactory	NILM	NILM	Negative (NILM)	
P02541	2018	2018-Q4	2018-11	Pre-pandemic	Cytology (Pap)	55-59	58	Not performed	Satisfactory	Positive	ASC-US	Low-grade / equivocal squamous	
P02542	2018	2018-Q4	2018-11	Pre-pandemic	Cytology (Pap)	60-64	62	Not performed	Satisfactory	NILM	NILM	Negative (NILM)	
P02543	2018	2018-Q4	2018-11	Pre-pandemic	Cytology (Pap)	35-39	35	Not performed	Satisfactory	NILM	NILM	Negative (NILM)	
P02544	2018	2018-Q4	2018-11	Pre-pandemic	Cytology (Pap)	45-49	47	Not performed	Satisfactory	Positive	AGC	Glandular	
P02545	2018	2018-Q4	2018-11	Pre-pandemic	Cytology (Pap)	60-64	61	Not performed	Satisfactory	NILM	NILM	Negative (NILM)	
P02546	2018	2018-Q4	2018-11	Pre-pandemic	Cytology (Pap)	45-49	45	Not performed	Satisfactory	NILM	NILM	Negative (NILM)	
P02547	2018	2018-Q4	2018-11	Pre-pandemic	Cytology (Pap)	55-59	58	Not performed	Satisfactory	Positive	LSIL	Low-grade / equivocal squamous	
P02548	2018	2018-Q4	2018-11	Pre-pandemic	Cytology (Pap)	60-64	60	Not performed	Satisfactory	NILM	NILM	Negative (NILM)	
P02549	2018	2018-Q4	2018-11	Pre-pandemic	Cytology (Pap)	45-49	46	Not performed	Satisfactory	NILM	NILM	Negative (NILM)	
P02550	2018	2018-Q4	2018-11	Pre-pandemic	Cytology (Pap)	40-44	43	Not performed	Satisfactory	NILM	NILM	Negative (NILM)	
P02551	2018	2018-Q4	2018-11	Pre-pandemic	Cytology (Pap)	50-54	50	Not performed	Satisfactory	NILM	NILM	Negative (NILM)	
P02552	2018	2018-Q4	2018-11	Pre-pandemic	Cytology (Pap)	30-34	31	Not performed	Satisfactory	Positive	LSIL	Low-grade / equivocal squamous	
P02553	2018	2018-Q4	2018-11	Pre-pandemic	Cytology (Pap)	40-44	43	Not performed	Satisfactory	NILM	NILM	Negative (NILM)	
P02554	2018	2018-Q4	2018-11	Pre-pandemic	Cytology (Pap)	50-54	53	Not performed	Satisfactory	NILM	NILM	Negative (NILM)	
P02555	2018	2018-Q4	2018-11	Pre-pandemic	Cytology (Pap)	35-39	39	Not performed	Satisfactory	NILM	NILM	Negative (NILM)	
P02556	2018	2018-Q4	2018-11	Pre-pandemic	Cytology (Pap)	45-49	47	Not performed	Satisfactory	NILM	NILM	Negative (NILM)	
P02557	2018	2018-Q4	2018-11	Pre-pandemic	Cytology (Pap)	35-39	35	Not performed	Satisfactory	NILM	NILM	Negative (NILM)	
P02558	2018	2018-Q4	2018-11	Pre-pandemic	Cytology (Pap)	50-54	54	Not performed	Satisfactory	NILM	NILM	Negative (NILM)	

patient_id	year	quarter	month	period	primary_test	age_group	age_years	hpv_result	adequacy	result	bethesda	grade	infection
P02559	2018	2018-Q4	2018-11	Pre-pandemic	Cytology (Pap)	40-44	41	Not performed	Satisfactory	NILM	NILM	Negative (NILM)	
P02560	2018	2018-Q4	2018-11	Pre-pandemic	Cytology (Pap)	45-49	48	Not performed	Satisfactory	NILM	NILM	Negative (NILM)	
P02561	2018	2018-Q4	2018-11	Pre-pandemic	Cytology (Pap)	45-49	45	Not performed	Satisfactory	NILM	NILM	Negative (NILM)	
P02562	2018	2018-Q4	2018-11	Pre-pandemic	Cytology (Pap)	40-44	41	Not performed	Satisfactory	NILM	NILM	Negative (NILM)	
P02563	2018	2018-Q4	2018-11	Pre-pandemic	Cytology (Pap)	55-59	56	Not performed	Satisfactory	NILM	NILM	Negative (NILM)	Gardnerella
P02564	2018	2018-Q4	2018-11	Pre-pandemic	Cytology (Pap)	55-59	55	Not performed	Satisfactory	NILM	NILM	Negative (NILM)	
P02565	2018	2018-Q4	2018-11	Pre-pandemic	Cytology (Pap)	50-54	54	Not performed	Satisfactory	Positive	HSIL	High-grade squamous	
P02566	2018	2018-Q4	2018-11	Pre-pandemic	Cytology (Pap)	40-44	44	Not performed	Satisfactory	NILM	NILM	Negative (NILM)	
P02567	2018	2018-Q4	2018-11	Pre-pandemic	Cytology (Pap)	40-44	41	Not performed	Satisfactory	NILM	NILM	Negative (NILM)	
P02568	2018	2018-Q4	2018-11	Pre-pandemic	Cytology (Pap)	45-49	46	Not performed	Satisfactory	NILM	NILM	Negative (NILM)	
P02569	2018	2018-Q4	2018-11	Pre-pandemic	Cytology (Pap)	60-64	62	Not performed	Satisfactory	NILM	NILM	Negative (NILM)	
P02570	2018	2018-Q4	2018-11	Pre-pandemic	Cytology (Pap)	55-59	57	Not performed	Satisfactory	NILM	NILM	Negative (NILM)	
P02571	2018	2018-Q4	2018-11	Pre-pandemic	Cytology (Pap)	40-44	44	Not performed	Satisfactory	NILM	NILM	Negative (NILM)	
P02572	2018	2018-Q4	2018-11	Pre-pandemic	Cytology (Pap)	35-39	37	Not performed	Satisfactory	NILM	NILM	Negative (NILM)	
P02573	2018	2018-Q4	2018-11	Pre-pandemic	Cytology (Pap)	40-44	40	Not performed	Satisfactory	NILM	NILM	Negative (NILM)	
P02574	2018	2018-Q4	2018-11	Pre-pandemic	Cytology (Pap)	50-54	53	Not performed	Satisfactory	Positive	HSIL	High-grade squamous	
P02575	2018	2018-Q4	2018-11	Pre-pandemic	Cytology (Pap)	50-54	54	Not performed	Satisfactory	NILM	NILM	Negative (NILM)	
P02576	2018	2018-Q4	2018-11	Pre-pandemic	Cytology (Pap)	30-34	32	Not performed	Satisfactory	NILM	NILM	Negative (NILM)	
P02577	2018	2018-Q4	2018-11	Pre-pandemic	Cytology (Pap)	45-49	47	Not performed	Satisfactory	Positive	HSIL	High-grade squamous	
P02578	2018	2018-Q4	2018-11	Pre-pandemic	Cytology (Pap)	Not recorded		Not performed	Satisfactory	NILM	NILM	Negative (NILM)	
P02579	2018	2018-Q4	2018-11	Pre-pandemic	Cytology (Pap)	40-44	43	Not performed	Satisfactory	NILM	NILM	Negative (NILM)	
P02580	2018	2018-Q4	2018-11	Pre-pandemic	Cytology (Pap)	25-29	26	Not performed	Satisfactory	NILM	NILM	Negative (NILM)	
P02581	2018	2018-Q4	2018-11	Pre-pandemic	Cytology (Pap)	50-54	53	Not performed	Satisfactory	NILM	NILM	Negative (NILM)	
P02582	2018	2018-Q4	2018-11	Pre-pandemic	Cytology (Pap)	30-34	31	Not performed	Satisfactory	NILM	NILM	Negative (NILM)	
P02583	2018	2018-Q4	2018-11	Pre-pandemic	Cytology (Pap)	30-34	34	Not performed	Satisfactory	NILM	NILM	Negative (NILM)	
P02584	2018	2018-Q4	2018-11	Pre-pandemic	Cytology (Pap)	40-44	41	Not performed	Satisfactory	NILM	NILM	Negative (NILM)	
P02585	2018	2018-Q4	2018-11	Pre-pandemic	Cytology (Pap)	Not recorded		Not performed	Satisfactory	NILM	NILM	Negative (NILM)	
P02586	2018	2018-Q4	2018-11	Pre-pandemic	Cytology (Pap)	40-44	42	Not performed	Satisfactory	NILM	NILM	Negative (NILM)	
P02587	2018	2018-Q4	2018-11	Pre-pandemic	Cytology (Pap)	60-64	64	Not performed	Satisfactory	NILM	NILM	Negative (NILM)	
P02588	2018	2018-Q4	2018-11	Pre-pandemic	Cytology (Pap)	55-59	58	Not performed	Satisfactory	Positive	HSIL	High-grade squamous	
P02589	2018	2018-Q4	2018-11	Pre-pandemic	Cytology (Pap)	60-64	60	Not performed	Satisfactory	NILM	NILM	Negative (NILM)	
P02590	2018	2018-Q4	2018-11	Pre-pandemic	Cytology (Pap)	40-44	43	Not performed	Satisfactory	NILM	NILM	Negative (NILM)	
P02591	2018	2018-Q4	2018-11	Pre-pandemic	Cytology (Pap)	30-34	34	Not performed	Satisfactory	NILM	NILM	Negative (NILM)	
P02592	2018	2018-Q4	2018-11	Pre-pandemic	Cytology (Pap)	55-59	55	Not performed	Satisfactory	Positive	HSIL	High-grade squamous	
P02593	2018	2018-Q4	2018-11	Pre-pandemic	Cytology (Pap)	35-39	36	Not performed	Satisfactory	NILM	NILM	Negative (NILM)	
P02594	2018	2018-Q4	2018-11	Pre-pandemic	Cytology (Pap)	40-44	41	Not performed	Satisfactory	NILM	NILM	Negative (NILM)	
P02595	2018	2018-Q4	2018-11	Pre-pandemic	Cytology (Pap)	30-34	33	Not performed	Satisfactory	NILM	NILM	Negative (NILM)	
P02596	2018	2018-Q4	2018-11	Pre-pandemic	Cytology (Pap)	50-54	52	Not performed	Satisfactory	NILM	NILM	Negative (NILM)	
P02597	2018	2018-Q4	2018-11	Pre-pandemic	Cytology (Pap)	30-34	34	Not performed	Satisfactory	NILM	NILM	Negative (NILM)	
P02598	2018	2018-Q4	2018-11	Pre-pandemic	Cytology (Pap)	25-29	29	Not performed	Satisfactory	NILM	NILM	Negative (NILM)	
P02599	2018	2018-Q4	2018-11	Pre-pandemic	Cytology (Pap)	25-29	27	Not performed	Satisfactory	NILM	NILM	Negative (NILM)	
P02600	2018	2018-Q4	2018-11	Pre-pandemic	Cytology (Pap)	45-49	49	Not performed	Satisfactory	NILM	NILM	Negative (NILM)	
P02601	2018	2018-Q4	2018-11	Pre-pandemic	Cytology (Pap)	45-49	46	Not performed	Satisfactory	Positive	LSIL	Low-grade / equivocal squamous	
P02602	2018	2018-Q4	2018-11	Pre-pandemic	Cytology (Pap)	55-59	59	Not performed	Satisfactory	Positive	ASC-H	High-grade squamous	
P02603	2018	2018-Q4	2018-11	Pre-pandemic	Cytology (Pap)	60-64	63	Not performed	Satisfactory	NILM	NILM	Negative (NILM)	
P02604	2018	2018-Q4	2018-11	Pre-pandemic	Cytology (Pap)	45-49	45	Not performed	Satisfactory	NILM	NILM	Negative (NILM)	
P02605	2018	2018-Q4	2018-11	Pre-pandemic	Cytology (Pap)	40-44	43	Not performed	Satisfactory	NILM	NILM	Negative (NILM)	
P02606	2018	2018-Q4	2018-11	Pre-pandemic	Cytology (Pap)	45-49	46	Not performed	Satisfactory	NILM	NILM	Negative (NILM)	
P02607	2018	2018-Q4	2018-11	Pre-pandemic	Cytology (Pap)	40-44	40	Not performed	Satisfactory	NILM	NILM	Negative (NILM)	
P02608	2018	2018-Q4	2018-11	Pre-pandemic	Cytology (Pap)	30-34	34	Not performed	Satisfactory	NILM	NILM	Negative (NILM)	
P02609	2018	2018-Q4	2018-11	Pre-pandemic	Cytology (Pap)	40-44	41	Not performed	Satisfactory	NILM	NILM	Negative (NILM)	
P02610	2018	2018-Q4	2018-11	Pre-pandemic	Cytology (Pap)	25-29	25	Not performed	Satisfactory	NILM	NILM	Negative (NILM)	
P02611	2018	2018-Q4	2018-11	Pre-pandemic	Cytology (Pap)	35-39	35	Not performed	Satisfactory	NILM	NILM	Negative (NILM)	
P02612	2018	2018-Q4	2018-11	Pre-pandemic	Cytology (Pap)	30-34	30	Not performed	Satisfactory	NILM	NILM	Negative (NILM)	
P02613	2018	2018-Q4	2018-11	Pre-pandemic	Cytology (Pap)	25-29	25	Not performed	Satisfactory	Positive	ASC-US	Low-grade / equivocal squamous	
P02614	2018	2018-Q4	2018-11	Pre-pandemic	Cytology (Pap)	25-29	28	Not performed	Satisfactory	NILM	NILM	Negative (NILM)	Candida
P02615	2018	2018-Q4	2018-11	Pre-pandemic	Cytology (Pap)	45-49	46	Not performed	Satisfactory	NILM	NILM	Negative (NILM)	
P02616	2018	2018-Q4	2018-11	Pre-pandemic	Cytology (Pap)	Not recorded		Not performed	Satisfactory	Positive	AGC	Glandular	
P02617	2018	2018-Q4	2018-11	Pre-pandemic	Cytology (Pap)	45-49	48	Not performed	Satisfactory	NILM	NILM	Negative (NILM)	
P02618	2018	2018-Q4	2018-11	Pre-pandemic	Cytology (Pap)	50-54	54	Not performed	Satisfactory	NILM	NILM	Negative (NILM)	
P02619	2018	2018-Q4	2018-11	Pre-pandemic	Cytology (Pap)	55-59	58	Not performed	Satisfactory	Positive	AGC	Glandular	
P02620	2018	2018-Q4	2018-11	Pre-pandemic	Cytology (Pap)	30-34	30	Not performed	Satisfactory	NILM	NILM	Negative (NILM)	
P02621	2018	2018-Q4	2018-11	Pre-pandemic	Cytology (Pap)	30-34	30	Not performed	Satisfactory	NILM	NILM	Negative (NILM)	
P02622	2018	2018-Q4	2018-11	Pre-pandemic	Cytology (Pap)	45-49	45	Not performed	Satisfactory	NILM	NILM	Negative (NILM)	Candida
P02623	2018	2018-Q4	2018-11	Pre-pandemic	Cytology (Pap)	55-59	55	Not performed	Satisfactory	NILM	NILM	Negative (NILM)	
P02624	2018	2018-Q4	2018-11	Pre-pandemic	Cytology (Pap)	45-49	45	Not performed	Satisfactory	NILM	NILM	Negative (NILM)	Trichomonas
P02625	2018	2018-Q4	2018-11	Pre-pandemic	Cytology (Pap)	45-49	48	Not performed	Unsatisfactory	Unsatisfactory	Unsatisfactory	Unsatisfactory	
P02626	2018	2018-Q4	2018-11	Pre-pandemic	Cytology (Pap)	30-34	34	Not performed	Satisfactory	NILM	NILM	Negative (NILM)	
P02627	2018	2018-Q4	2018-11	Pre-pandemic	Cytology (Pap)	55-59	59	Not performed	Satisfactory	NILM	NILM	Negative (NILM)	
P02628	2018	2018-Q4	2018-11	Pre-pandemic	Cytology (Pap)	55-59	59	Not performed	Satisfactory	NILM	NILM	Negative (NILM)	
P02629	2018	2018-Q4	2018-11	Pre-pandemic	Cytology (Pap)	35-39	37	Not performed	Satisfactory	NILM	NILM	Negative (NILM)	
P02630	2018	2018-Q4	2018-11	Pre-pandemic	Cytology (Pap)	50-54	52	Not performed	Satisfactory	NILM	NILM	Negative (NILM)	
P02631	2018	2018-Q4	2018-11	Pre-pandemic	Cytology (Pap)	55-59	58	Not performed	Satisfactory	NILM	NILM	Negative (NILM)	
P02632	2018	2018-Q4	2018-11	Pre-pandemic	Cytology (Pap)	Not recorded		Not performed	Satisfactory	NILM	NILM	Negative (NILM)	
P02633	2018	2018-Q4	2018-11	Pre-pandemic	Cytology (Pap)	35-39	37	Not performed	Satisfactory	NILM	NILM	Negative (NILM)	
P02634	2018	2018-Q4	2018-11	Pre-pandemic	Cytology (Pap)	35-39	37	Not performed	Satisfactory	NILM	NILM	Negative (NILM)	
P02635	2018	2018-Q4	2018-11	Pre-pandemic	Cytology (Pap)	35-39	38	Not performed	Satisfactory	NILM	NILM	Negative (NILM)	
P02636	2018	2018-Q4	2018-11	Pre-pandemic	Cytology (Pap)	35-39	39	Not performed	Satisfactory	NILM	NILM	Negative (NILM)	
P02637	2018	2018-Q4	2018-11	Pre-pandemic	Cytology (Pap)	40-44	42	Not performed	Satisfactory	NILM	NILM	Negative (NILM)	
P02638	2018	2018-Q4	2018-11	Pre-pandemic	Cytology (Pap)	45-49	49	Not performed	Satisfactory	NILM	NILM	Negative (NILM)	

patient_id	year	quarter	month	period	primary_test	age_group	age_years	hpv_result	adequacy	result	bethesda	grade	infection
P02639	2018	2018-Q4	2018-11	Pre-pandemic	Cytology (Pap)	55-59	58	Not performed	Satisfactory	NILM	NILM	Negative (NILM)	
P02640	2018	2018-Q4	2018-11	Pre-pandemic	Cytology (Pap)	35-39	38	Not performed	Satisfactory	Positive	LSIL	Low-grade / equivocal squamous	
P02641	2018	2018-Q4	2018-11	Pre-pandemic	Cytology (Pap)	55-59	57	Not performed	Satisfactory	NILM	NILM	Negative (NILM)	
P02642	2018	2018-Q4	2018-12	Pre-pandemic	Cytology (Pap)	50-54	50	Not performed	Satisfactory	NILM	NILM	Negative (NILM)	
P02643	2018	2018-Q4	2018-12	Pre-pandemic	Cytology (Pap)	50-54	50	Not performed	Satisfactory	NILM	NILM	Negative (NILM)	
P02644	2018	2018-Q4	2018-12	Pre-pandemic	Cytology (Pap)	50-54	50	Not performed	Satisfactory	NILM	NILM	Negative (NILM)	
P02645	2018	2018-Q4	2018-12	Pre-pandemic	Cytology (Pap)	55-59	59	Not performed	Satisfactory	NILM	NILM	Negative (NILM)	
P02646	2018	2018-Q4	2018-12	Pre-pandemic	Cytology (Pap)	50-54	52	Not performed	Satisfactory	NILM	NILM	Negative (NILM)	
P02647	2018	2018-Q4	2018-12	Pre-pandemic	Cytology (Pap)	35-39	38	Not performed	Satisfactory	Positive	ASC-US	Low-grade / equivocal squamous	
P02648	2018	2018-Q4	2018-12	Pre-pandemic	Cytology (Pap)	25-29	29	Not performed	Satisfactory	NILM	NILM	Negative (NILM)	
P02649	2018	2018-Q4	2018-12	Pre-pandemic	Cytology (Pap)	60-64	60	Not performed	Satisfactory	NILM	NILM	Negative (NILM)	
P02650	2018	2018-Q4	2018-12	Pre-pandemic	Cytology (Pap)	35-39	38	Not performed	Satisfactory	NILM	NILM	Negative (NILM)	
P02651	2018	2018-Q4	2018-12	Pre-pandemic	Cytology (Pap)	40-44	44	Not performed	Satisfactory	NILM	NILM	Negative (NILM)	
P02652	2018	2018-Q4	2018-12	Pre-pandemic	Cytology (Pap)	40-44	44	Not performed	Satisfactory	NILM	NILM	Negative (NILM)	
P02653	2018	2018-Q4	2018-12	Pre-pandemic	Cytology (Pap)	35-39	39	Not performed	Satisfactory	NILM	NILM	Negative (NILM)	
P02654	2018	2018-Q4	2018-12	Pre-pandemic	Cytology (Pap)	25-29	26	Not performed	Unsatisfactory	Unsatisfactory	Unsatisfactory	Unsatisfactory	
P02655	2018	2018-Q4	2018-12	Pre-pandemic	Cytology (Pap)	45-49	46	Not performed	Satisfactory	NILM	NILM	Negative (NILM)	
P02656	2018	2018-Q4	2018-12	Pre-pandemic	Cytology (Pap)	45-49	45	Not performed	Satisfactory	NILM	NILM	Negative (NILM)	
P02657	2018	2018-Q4	2018-12	Pre-pandemic	Cytology (Pap)	35-39	39	Not performed	Satisfactory	NILM	NILM	Negative (NILM)	
P02658	2018	2018-Q4	2018-12	Pre-pandemic	Cytology (Pap)	40-44	44	Not performed	Satisfactory	NILM	NILM	Negative (NILM)	
P02659	2018	2018-Q4	2018-12	Pre-pandemic	Cytology (Pap)	35-39	35	Not performed	Satisfactory	NILM	NILM	Negative (NILM)	
P02660	2018	2018-Q4	2018-12	Pre-pandemic	Cytology (Pap)	50-54	51	Not performed	Unsatisfactory	Unsatisfactory	Unsatisfactory	Unsatisfactory	
P02661	2018	2018-Q4	2018-12	Pre-pandemic	Cytology (Pap)	50-54	53	Not performed	Satisfactory	NILM	NILM	Negative (NILM)	
P02662	2018	2018-Q4	2018-12	Pre-pandemic	Cytology (Pap)	30-34	34	Not performed	Satisfactory	NILM	NILM	Negative (NILM)	
P02663	2018	2018-Q4	2018-12	Pre-pandemic	Cytology (Pap)	45-49	45	Not performed	Satisfactory	NILM	NILM	Negative (NILM)	
P02664	2018	2018-Q4	2018-12	Pre-pandemic	Cytology (Pap)	40-44	44	Not performed	Satisfactory	NILM	NILM	Negative (NILM)	
P02665	2018	2018-Q4	2018-12	Pre-pandemic	Cytology (Pap)	35-39	39	Not performed	Satisfactory	NILM	NILM	Negative (NILM)	
P02666	2018	2018-Q4	2018-12	Pre-pandemic	Cytology (Pap)	40-44	42	Not performed	Satisfactory	NILM	NILM	Negative (NILM)	
P02667	2018	2018-Q4	2018-12	Pre-pandemic	Cytology (Pap)	55-59	57	Not performed	Satisfactory	NILM	NILM	Negative (NILM)	
P02668	2018	2018-Q4	2018-12	Pre-pandemic	Cytology (Pap)	40-44	42	Not performed	Satisfactory	NILM	NILM	Negative (NILM)	
P02669	2018	2018-Q4	2018-12	Pre-pandemic	Cytology (Pap)	45-49	48	Not performed	Satisfactory	NILM	NILM	Negative (NILM)	
P02670	2018	2018-Q4	2018-12	Pre-pandemic	Cytology (Pap)	25-29	25	Not performed	Satisfactory	NILM	NILM	Negative (NILM)	
P02671	2018	2018-Q4	2018-12	Pre-pandemic	Cytology (Pap)	50-54	51	Not performed	Satisfactory	NILM	NILM	Negative (NILM)	
P02672	2018	2018-Q4	2018-12	Pre-pandemic	Cytology (Pap)	30-34	30	Not performed	Satisfactory	NILM	NILM	Negative (NILM)	
P02673	2018	2018-Q4	2018-12	Pre-pandemic	Cytology (Pap)	40-44	41	Not performed	Satisfactory	NILM	NILM	Negative (NILM)	
P02674	2018	2018-Q4	2018-12	Pre-pandemic	Cytology (Pap)	60-64	60	Not performed	Satisfactory	NILM	NILM	Negative (NILM)	
P02675	2018	2018-Q4	2018-12	Pre-pandemic	Cytology (Pap)	35-39	37	Not performed	Satisfactory	NILM	NILM	Negative (NILM)	
P02676	2018	2018-Q4	2018-12	Pre-pandemic	Cytology (Pap)	35-39	35	Not performed	Satisfactory	Positive	HSIL	High-grade squamous	
P02677	2018	2018-Q4	2018-12	Pre-pandemic	Cytology (Pap)	30-34	34	Not performed	Satisfactory	NILM	NILM	Negative (NILM)	
P02678	2018	2018-Q4	2018-12	Pre-pandemic	Cytology (Pap)	35-39	39	Not performed	Satisfactory	NILM	NILM	Negative (NILM)	
P02679	2018	2018-Q4	2018-12	Pre-pandemic	Cytology (Pap)	45-49	48	Not performed	Satisfactory	NILM	NILM	Negative (NILM)	
P02680	2018	2018-Q4	2018-12	Pre-pandemic	Cytology (Pap)	30-34	32	Not performed	Satisfactory	NILM	NILM	Negative (NILM)	
P02681	2018	2018-Q4	2018-12	Pre-pandemic	Cytology (Pap)	45-49	49	Not performed	Satisfactory	NILM	NILM	Negative (NILM)	
P02682	2018	2018-Q4	2018-12	Pre-pandemic	Cytology (Pap)	45-49	47	Not performed	Unsatisfactory	Unsatisfactory	Unsatisfactory	Unsatisfactory	
P02683	2018	2018-Q4	2018-12	Pre-pandemic	Cytology (Pap)	45-49	47	Not performed	Satisfactory	NILM	NILM	Negative (NILM)	
P02684	2018	2018-Q4	2018-12	Pre-pandemic	Cytology (Pap)	40-44	44	Not performed	Satisfactory	Positive	ASC-US	Low-grade / equivocal squamous	
P02685	2018	2018-Q4	2018-12	Pre-pandemic	Cytology (Pap)	25-29	29	Not performed	Satisfactory	NILM	NILM	Negative (NILM)	
P02686	2018	2018-Q4	2018-12	Pre-pandemic	Cytology (Pap)	35-39	36	Not performed	Satisfactory	NILM	NILM	Negative (NILM)	
P02687	2018	2018-Q4	2018-12	Pre-pandemic	Cytology (Pap)	40-44	43	Not performed	Satisfactory	NILM	NILM	Negative (NILM)	
P02688	2018	2018-Q4	2018-12	Pre-pandemic	Cytology (Pap)	25-29	25	Not performed	Satisfactory	NILM	NILM	Negative (NILM)	
P02689	2018	2018-Q4	2018-12	Pre-pandemic	Cytology (Pap)	45-49	48	Not performed	Satisfactory	NILM	NILM	Negative (NILM)	
P02690	2018	2018-Q4	2018-12	Pre-pandemic	Cytology (Pap)	30-34	32	Not performed	Satisfactory	Positive	ASC-US	Low-grade / equivocal squamous	
P02691	2018	2018-Q4	2018-12	Pre-pandemic	Cytology (Pap)	40-44	41	Not performed	Satisfactory	NILM	NILM	Negative (NILM)	
P02692	2018	2018-Q4	2018-12	Pre-pandemic	Cytology (Pap)	25-29	25	Not performed	Satisfactory	NILM	NILM	Negative (NILM)	
P02693	2018	2018-Q4	2018-12	Pre-pandemic	Cytology (Pap)	45-49	46	Not performed	Satisfactory	NILM	NILM	Negative (NILM)	
P02694	2018	2018-Q4	2018-12	Pre-pandemic	Cytology (Pap)	35-39	36	Not performed	Satisfactory	NILM	NILM	Negative (NILM)	
P02695	2018	2018-Q4	2018-12	Pre-pandemic	Cytology (Pap)	25-29	25	Not performed	Satisfactory	NILM	NILM	Negative (NILM)	
P02696	2018	2018-Q4	2018-12	Pre-pandemic	Cytology (Pap)	30-34	33	Not performed	Unsatisfactory	Unsatisfactory	Unsatisfactory	Unsatisfactory	
P02697	2018	2018-Q4	2018-12	Pre-pandemic	Cytology (Pap)	50-54	50	Not performed	Satisfactory	Positive	LSIL	Low-grade / equivocal squamous	
P02698	2018	2018-Q4	2018-12	Pre-pandemic	Cytology (Pap)	40-44	44	Not performed	Satisfactory	NILM	NILM	Negative (NILM)	
P02699	2018	2018-Q4	2018-12	Pre-pandemic	Cytology (Pap)	40-44	40	Not performed	Unsatisfactory	Unsatisfactory	Unsatisfactory	Unsatisfactory	
P02700	2018	2018-Q4	2018-12	Pre-pandemic	Cytology (Pap)	55-59	57	Not performed	Satisfactory	NILM	NILM	Negative (NILM)	
P02701	2018	2018-Q4	2018-12	Pre-pandemic	Cytology (Pap)	30-34	33	Not performed	Satisfactory	NILM	NILM	Negative (NILM)	
P02702	2018	2018-Q4	2018-12	Pre-pandemic	Cytology (Pap)	25-29	28	Not performed	Satisfactory	Positive	ASC-H	High-grade squamous	Trichomonas
P02703	2018	2018-Q4	2018-12	Pre-pandemic	Cytology (Pap)	40-44	40	Not performed	Satisfactory	NILM	NILM	Negative (NILM)	
P02704	2018	2018-Q4	2018-12	Pre-pandemic	Cytology (Pap)	45-49	46	Not performed	Satisfactory	NILM	NILM	Negative (NILM)	
P02705	2018	2018-Q4	2018-12	Pre-pandemic	Cytology (Pap)	40-44	40	Not performed	Satisfactory	Positive	HSIL	High-grade squamous	
P02706	2018	2018-Q4	2018-12	Pre-pandemic	Cytology (Pap)	50-54	50	Not performed	Satisfactory	Positive	ASC-H	High-grade squamous	
P02707	2018	2018-Q4	2018-12	Pre-pandemic	Cytology (Pap)	40-44	44	Not performed	Satisfactory	NILM	NILM	Negative (NILM)	
P02708	2018	2018-Q4	2018-12	Pre-pandemic	Cytology (Pap)	30-34	33	Not performed	Satisfactory	Positive	ASC-US	Low-grade / equivocal squamous	
P02709	2018	2018-Q4	2018-12	Pre-pandemic	Cytology (Pap)	60-64	64	Not performed	Satisfactory	NILM	NILM	Negative (NILM)	
P02710	2018	2018-Q4	2018-12	Pre-pandemic	Cytology (Pap)	25-29	27	Not performed	Satisfactory	NILM	NILM	Negative (NILM)	
P02711	2018	2018-Q4	2018-12	Pre-pandemic	Cytology (Pap)	Not recorded		Not performed	Satisfactory	NILM	NILM	Negative (NILM)	Trichomonas
P02712	2018	2018-Q4	2018-12	Pre-pandemic	Cytology (Pap)	60-64	61	Not performed	Satisfactory	NILM	NILM	Negative (NILM)	
P02713	2018	2018-Q4	2018-12	Pre-pandemic	Cytology (Pap)	45-49	47	Not performed	Satisfactory	Positive	LSIL	Low-grade / equivocal squamous	
P02714	2018	2018-Q4	2018-12	Pre-pandemic	Cytology (Pap)	35-39	36	Not performed	Satisfactory	NILM	NILM	Negative (NILM)	
P02715	2018	2018-Q4	2018-12	Pre-pandemic	Cytology (Pap)	45-49	45	Not performed	Satisfactory	NILM	NILM	Negative (NILM)	
P02716	2018	2018-Q4	2018-12	Pre-pandemic	Cytology (Pap)	35-39	39	Not performed	Satisfactory	NILM	NILM	Negative (NILM)	
P02717	2018	2018-Q4	2018-12	Pre-pandemic	Cytology (Pap)	Not recorded		Not performed	Satisfactory	Positive	LSIL	Low-grade / equivocal squamous	
P02718	2018	2018-Q4	2018-12	Pre-pandemic	Cytology (Pap)	50-54	52	Not performed	Satisfactory	NILM	NILM	Negative (NILM)	

patient_id	year	quarter	month	period	primary_test	age_group	age_years	hpv_result	adequacy	result	bethesda	grade	infection
P02719	2018	2018-Q4	2018-12	Pre-pandemic	Cytology (Pap)	40-44	42	Not performed	Satisfactory	NILM	NILM	Negative (NILM)	
P02720	2018	2018-Q4	2018-12	Pre-pandemic	Cytology (Pap)	35-39	37	Not performed	Satisfactory	Positive	ASC-US	Low-grade / equivocal squamous	
P02721	2018	2018-Q4	2018-12	Pre-pandemic	Cytology (Pap)	30-34	31	Not performed	Satisfactory	NILM	NILM	Negative (NILM)	
P02722	2018	2018-Q4	2018-12	Pre-pandemic	Cytology (Pap)	55-59	58	Not performed	Satisfactory	NILM	NILM	Negative (NILM)	
P02723	2018	2018-Q4	2018-12	Pre-pandemic	Cytology (Pap)	45-49	46	Not performed	Satisfactory	NILM	NILM	Negative (NILM)	
P02724	2018	2018-Q4	2018-12	Pre-pandemic	Cytology (Pap)	25-29	29	Not performed	Satisfactory	NILM	NILM	Negative (NILM)	
P02725	2018	2018-Q4	2018-12	Pre-pandemic	Cytology (Pap)	55-59	59	Not performed	Satisfactory	NILM	NILM	Negative (NILM)	
P02726	2018	2018-Q4	2018-12	Pre-pandemic	Cytology (Pap)	45-49	46	Not performed	Satisfactory	NILM	NILM	Negative (NILM)	
P02727	2018	2018-Q4	2018-12	Pre-pandemic	Cytology (Pap)	60-64	63	Not performed	Satisfactory	NILM	NILM	Negative (NILM)	Other
P02728	2018	2018-Q4	2018-12	Pre-pandemic	Cytology (Pap)	55-59	59	Not performed	Satisfactory	NILM	NILM	Negative (NILM)	
P02729	2018	2018-Q4	2018-12	Pre-pandemic	Cytology (Pap)	55-59	55	Not performed	Satisfactory	NILM	NILM	Negative (NILM)	
P02730	2018	2018-Q4	2018-12	Pre-pandemic	Cytology (Pap)	40-44	41	Not performed	Satisfactory	NILM	NILM	Negative (NILM)	
P02731	2018	2018-Q4	2018-12	Pre-pandemic	Cytology (Pap)	25-29	28	Not performed	Satisfactory	NILM	NILM	Negative (NILM)	
P02732	2018	2018-Q4	2018-12	Pre-pandemic	Cytology (Pap)	45-49	45	Not performed	Satisfactory	NILM	NILM	Negative (NILM)	
P02733	2018	2018-Q4	2018-12	Pre-pandemic	Cytology (Pap)	45-49	45	Not performed	Satisfactory	Positive	ASC-US	Low-grade / equivocal squamous	
P02734	2018	2018-Q4	2018-12	Pre-pandemic	Cytology (Pap)	50-54	52	Not performed	Satisfactory	NILM	NILM	Negative (NILM)	
P02735	2018	2018-Q4	2018-12	Pre-pandemic	Cytology (Pap)	55-59	57	Not performed	Satisfactory	NILM	NILM	Negative (NILM)	
P02736	2018	2018-Q4	2018-12	Pre-pandemic	Cytology (Pap)	35-39	36	Not performed	Satisfactory	NILM	NILM	Negative (NILM)	
P02737	2018	2018-Q4	2018-12	Pre-pandemic	Cytology (Pap)	25-29	27	Not performed	Satisfactory	NILM	NILM	Negative (NILM)	
P02738	2018	2018-Q4	2018-12	Pre-pandemic	Cytology (Pap)	30-34	30	Not performed	Satisfactory	Positive	ASC-US	Low-grade / equivocal squamous	
P02739	2018	2018-Q4	2018-12	Pre-pandemic	Cytology (Pap)	55-59	56	Not performed	Satisfactory	NILM	NILM	Negative (NILM)	
P02740	2018	2018-Q4	2018-12	Pre-pandemic	Cytology (Pap)	50-54	50	Not performed	Satisfactory	NILM	NILM	Negative (NILM)	
P02741	2018	2018-Q4	2018-12	Pre-pandemic	Cytology (Pap)	30-34	33	Not performed	Satisfactory	Positive	LSIL	Low-grade / equivocal squamous	
P02742	2018	2018-Q4	2018-12	Pre-pandemic	Cytology (Pap)	35-39	37	Not performed	Satisfactory	NILM	NILM	Negative (NILM)	
P02743	2018	2018-Q4	2018-12	Pre-pandemic	Cytology (Pap)	30-34	30	Not performed	Satisfactory	Positive	ASC-US	Low-grade / equivocal squamous	
P02744	2018	2018-Q4	2018-12	Pre-pandemic	Cytology (Pap)	30-34	33	Not performed	Satisfactory	NILM	NILM	Negative (NILM)	
P02745	2018	2018-Q4	2018-12	Pre-pandemic	Cytology (Pap)	45-49	46	Not performed	Satisfactory	NILM	NILM	Negative (NILM)	
P02746	2018	2018-Q4	2018-12	Pre-pandemic	Cytology (Pap)	25-29	25	Not performed	Unsatisfactory	Unsatisfactory	Unsatisfactory	Unsatisfactory	
P02747	2018	2018-Q4	2018-12	Pre-pandemic	Cytology (Pap)	40-44	40	Not performed	Satisfactory	NILM	NILM	Negative (NILM)	
P02748	2018	2018-Q4	2018-12	Pre-pandemic	Cytology (Pap)	60-64	61	Not performed	Satisfactory	NILM	NILM	Negative (NILM)	
P02749	2018	2018-Q4	2018-12	Pre-pandemic	Cytology (Pap)	40-44	44	Not performed	Satisfactory	NILM	NILM	Negative (NILM)	
P02750	2018	2018-Q4	2018-12	Pre-pandemic	Cytology (Pap)	55-59	59	Not performed	Satisfactory	NILM	NILM	Negative (NILM)	
P02751	2018	2018-Q4	2018-12	Pre-pandemic	Cytology (Pap)	Not recorded		Not performed	Satisfactory	Positive	ASC-US	Low-grade / equivocal squamous	
P02752	2018	2018-Q4	2018-12	Pre-pandemic	Cytology (Pap)	35-39	39	Not performed	Satisfactory	NILM	NILM	Negative (NILM)	
P02753	2018	2018-Q4	2018-12	Pre-pandemic	Cytology (Pap)	25-29	25	Not performed	Satisfactory	NILM	NILM	Negative (NILM)	
P02754	2018	2018-Q4	2018-12	Pre-pandemic	Cytology (Pap)	55-59	58	Not performed	Satisfactory	NILM	NILM	Negative (NILM)	
P02755	2018	2018-Q4	2018-12	Pre-pandemic	Cytology (Pap)	25-29	25	Not performed	Satisfactory	NILM	NILM	Negative (NILM)	
P02756	2018	2018-Q4	2018-12	Pre-pandemic	Cytology (Pap)	50-54	50	Not performed	Satisfactory	NILM	NILM	Negative (NILM)	
P02757	2018	2018-Q4	2018-12	Pre-pandemic	Cytology (Pap)	45-49	45	Not performed	Satisfactory	NILM	NILM	Negative (NILM)	
P02758	2018	2018-Q4	2018-12	Pre-pandemic	Cytology (Pap)	40-44	42	Not performed	Satisfactory	NILM	NILM	Negative (NILM)	
P02759	2018	2018-Q4	2018-12	Pre-pandemic	Cytology (Pap)	60-64	64	Not performed	Satisfactory	NILM	NILM	Negative (NILM)	
P02760	2018	2018-Q4	2018-12	Pre-pandemic	Cytology (Pap)	35-39	35	Not performed	Satisfactory	NILM	NILM	Negative (NILM)	
P02761	2018	2018-Q4	2018-12	Pre-pandemic	Cytology (Pap)	40-44	43	Not performed	Satisfactory	NILM	NILM	Negative (NILM)	Candida
P02762	2018	2018-Q4	2018-12	Pre-pandemic	Cytology (Pap)	50-54	50	Not performed	Satisfactory	Positive	ASC-US	Low-grade / equivocal squamous	
P02763	2018	2018-Q4	2018-12	Pre-pandemic	Cytology (Pap)	35-39	39	Not performed	Satisfactory	NILM	NILM	Negative (NILM)	
P02764	2018	2018-Q4	2018-12	Pre-pandemic	Cytology (Pap)	30-34	33	Not performed	Satisfactory	NILM	NILM	Negative (NILM)	
P02765	2018	2018-Q4	2018-12	Pre-pandemic	Cytology (Pap)	Not recorded		Not performed	Satisfactory	NILM	NILM	Negative (NILM)	
P02766	2018	2018-Q4	2018-12	Pre-pandemic	Cytology (Pap)	40-44	41	Not performed	Satisfactory	NILM	NILM	Negative (NILM)	
P02767	2018	2018-Q4	2018-12	Pre-pandemic	Cytology (Pap)	25-29	27	Not performed	Satisfactory	NILM	NILM	Negative (NILM)	
P02768	2018	2018-Q4	2018-12	Pre-pandemic	Cytology (Pap)	60-64	61	Not performed	Satisfactory	NILM	NILM	Negative (NILM)	
P02769	2018	2018-Q4	2018-12	Pre-pandemic	Cytology (Pap)	Not recorded		Not performed	Satisfactory	NILM	NILM	Negative (NILM)	
P02770	2018	2018-Q4	2018-12	Pre-pandemic	Cytology (Pap)	30-34	30	Not performed	Satisfactory	NILM	NILM	Negative (NILM)	
P02771	2018	2018-Q4	2018-12	Pre-pandemic	Cytology (Pap)	35-39	35	Not performed	Satisfactory	NILM	NILM	Negative (NILM)	
P02772	2018	2018-Q4	2018-12	Pre-pandemic	Cytology (Pap)	45-49	45	Not performed	Satisfactory	NILM	NILM	Negative (NILM)	
P02773	2018	2018-Q4	2018-12	Pre-pandemic	Cytology (Pap)	50-54	52	Not performed	Satisfactory	NILM	NILM	Negative (NILM)	
P02774	2018	2018-Q4	2018-12	Pre-pandemic	Cytology (Pap)	35-39	39	Not performed	Satisfactory	NILM	NILM	Negative (NILM)	
P02775	2018	2018-Q4	2018-12	Pre-pandemic	Cytology (Pap)	40-44	41	Not performed	Satisfactory	NILM	NILM	Negative (NILM)	
P02776	2018	2018-Q4	2018-12	Pre-pandemic	Cytology (Pap)	55-59	55	Not performed	Satisfactory	NILM	NILM	Negative (NILM)	
P02777	2018	2018-Q4	2018-12	Pre-pandemic	Cytology (Pap)	40-44	43	Not performed	Satisfactory	NILM	NILM	Negative (NILM)	
P02778	2018	2018-Q4	2018-12	Pre-pandemic	Cytology (Pap)	40-44	43	Not performed	Satisfactory	NILM	NILM	Negative (NILM)	
P02779	2018	2018-Q4	2018-12	Pre-pandemic	Cytology (Pap)	50-54	53	Not performed	Satisfactory	NILM	NILM	Negative (NILM)	
P02780	2018	2018-Q4	2018-12	Pre-pandemic	Cytology (Pap)	Not recorded		Not performed	Satisfactory	NILM	NILM	Negative (NILM)	
P02781	2018	2018-Q4	2018-12	Pre-pandemic	Cytology (Pap)	50-54	51	Not performed	Satisfactory	NILM	NILM	Negative (NILM)	
P02782	2018	2018-Q4	2018-12	Pre-pandemic	Cytology (Pap)	50-54	52	Not performed	Satisfactory	Positive	ASC-US	Low-grade / equivocal squamous	
P02783	2018	2018-Q4	2018-12	Pre-pandemic	Cytology (Pap)	50-54	51	Not performed	Satisfactory	Positive	ASC-US	Low-grade / equivocal squamous	
P02784	2018	2018-Q4	2018-12	Pre-pandemic	Cytology (Pap)	50-54	53	Not performed	Satisfactory	NILM	NILM	Negative (NILM)	
P02785	2018	2018-Q4	2018-12	Pre-pandemic	Cytology (Pap)	40-44	41	Not performed	Satisfactory	NILM	NILM	Negative (NILM)	
P02786	2018	2018-Q4	2018-12	Pre-pandemic	Cytology (Pap)	45-49	45	Not performed	Satisfactory	Positive	AGC	Glandular	
P02787	2018	2018-Q4	2018-12	Pre-pandemic	Cytology (Pap)	25-29	28	Not performed	Satisfactory	NILM	NILM	Negative (NILM)	
P02788	2018	2018-Q4	2018-12	Pre-pandemic	Cytology (Pap)	25-29	25	Not performed	Satisfactory	NILM	NILM	Negative (NILM)	
P02789	2018	2018-Q4	2018-12	Pre-pandemic	Cytology (Pap)	40-44	40	Not performed	Satisfactory	NILM	NILM	Negative (NILM)	
P02790	2018	2018-Q4	2018-12	Pre-pandemic	Cytology (Pap)	55-59	57	Not performed	Satisfactory	Positive	LSIL	Low-grade / equivocal squamous	
P02791	2018	2018-Q4	2018-12	Pre-pandemic	Cytology (Pap)	30-34	31	Not performed	Satisfactory	NILM	NILM	Negative (NILM)	
P02792	2018	2018-Q4	2018-12	Pre-pandemic	Cytology (Pap)	30-34	34	Not performed	Satisfactory	NILM	NILM	Negative (NILM)	
P02793	2018	2018-Q4	2018-12	Pre-pandemic	Cytology (Pap)	35-39	37	Not performed	Satisfactory	NILM	NILM	Negative (NILM)	
P02794	2018	2018-Q4	2018-12	Pre-pandemic	Cytology (Pap)	Not recorded		Not performed	Satisfactory	NILM	NILM	Negative (NILM)	
P02795	2018	2018-Q4	2018-12	Pre-pandemic	Cytology (Pap)	45-49	49	Not performed	Satisfactory	NILM	NILM	Negative (NILM)	
P02796	2018	2018-Q4	2018-12	Pre-pandemic	Cytology (Pap)	Not recorded		Not performed	Satisfactory	Positive	ASC-US	Low-grade / equivocal squamous	
P02797	2018	2018-Q4	2018-12	Pre-pandemic	Cytology (Pap)	35-39	36	Not performed	Satisfactory	NILM	NILM	Negative (NILM)	
P02798	2018	2018-Q4	2018-12	Pre-pandemic	Cytology (Pap)	50-54	50	Not performed	Satisfactory	NILM	NILM	Negative (NILM)	

patient_id	year	quarter	month	period	primary_test	age_group	age_years	hpv_result	adequacy	result	bethesda	grade	infection
P02799	2018	2018-Q4	2018-12	Pre-pandemic	Cytology (Pap)	40-44	42	Not performed	Satisfactory	NILM	NILM	Negative (NILM)	
P02800	2018	2018-Q4	2018-12	Pre-pandemic	Cytology (Pap)	50-54	54	Not performed	Satisfactory	NILM	NILM	Negative (NILM)	
P02801	2018	2018-Q4	2018-12	Pre-pandemic	Cytology (Pap)	35-39	38	Not performed	Satisfactory	NILM	NILM	Negative (NILM)	
P02802	2018	2018-Q4	2018-12	Pre-pandemic	Cytology (Pap)	60-64	64	Not performed	Satisfactory	NILM	NILM	Negative (NILM)	
P02803	2018	2018-Q4	2018-12	Pre-pandemic	Cytology (Pap)	40-44	41	Not performed	Satisfactory	NILM	NILM	Negative (NILM)	
P02804	2018	2018-Q4	2018-12	Pre-pandemic	Cytology (Pap)	35-39	37	Not performed	Satisfactory	Positive	HSIL	High-grade squamous	Candida
P02805	2018	2018-Q4	2018-12	Pre-pandemic	Cytology (Pap)	40-44	44	Not performed	Satisfactory	NILM	NILM	Negative (NILM)	
P02806	2018	2018-Q4	2018-12	Pre-pandemic	Cytology (Pap)	40-44	41	Not performed	Satisfactory	NILM	NILM	Negative (NILM)	
P02807	2018	2018-Q4	2018-12	Pre-pandemic	Cytology (Pap)	50-54	52	Not performed	Satisfactory	NILM	NILM	Negative (NILM)	
P02808	2018	2018-Q4	2018-12	Pre-pandemic	Cytology (Pap)	30-34	30	Not performed	Satisfactory	NILM	NILM	Negative (NILM)	
P02809	2018	2018-Q4	2018-12	Pre-pandemic	Cytology (Pap)	50-54	50	Not performed	Satisfactory	NILM	NILM	Negative (NILM)	
P02810	2018	2018-Q4	2018-12	Pre-pandemic	Cytology (Pap)	50-54	51	Not performed	Satisfactory	NILM	NILM	Negative (NILM)	
P02811	2018	2018-Q4	2018-12	Pre-pandemic	Cytology (Pap)	Not recorded		Not performed	Satisfactory	Positive	LSIL	Low-grade / equivocal squamous	
P02812	2018	2018-Q4	2018-12	Pre-pandemic	Cytology (Pap)	60-64	63	Not performed	Satisfactory	NILM	NILM	Negative (NILM)	Candida
P02813	2018	2018-Q4	2018-12	Pre-pandemic	Cytology (Pap)	55-59	59	Not performed	Satisfactory	Positive	ASC-US	Low-grade / equivocal squamous	
P02814	2018	2018-Q4	2018-12	Pre-pandemic	Cytology (Pap)	35-39	38	Not performed	Satisfactory	NILM	NILM	Negative (NILM)	
P02815	2018	2018-Q4	2018-12	Pre-pandemic	Cytology (Pap)	50-54	50	Not performed	Satisfactory	NILM	NILM	Negative (NILM)	
P02816	2018	2018-Q4	2018-12	Pre-pandemic	Cytology (Pap)	25-29	25	Not performed	Satisfactory	NILM	NILM	Negative (NILM)	
P02817	2018	2018-Q4	2018-12	Pre-pandemic	Cytology (Pap)	35-39	35	Not performed	Satisfactory	NILM	NILM	Negative (NILM)	
P02818	2018	2018-Q4	2018-12	Pre-pandemic	Cytology (Pap)	25-29	29	Not performed	Satisfactory	NILM	NILM	Negative (NILM)	
P02819	2018	2018-Q4	2018-12	Pre-pandemic	Cytology (Pap)	45-49	46	Not performed	Satisfactory	NILM	NILM	Negative (NILM)	
P02820	2018	2018-Q4	2018-12	Pre-pandemic	Cytology (Pap)	45-49	47	Not performed	Satisfactory	NILM	NILM	Negative (NILM)	
P02821	2018	2018-Q4	2018-12	Pre-pandemic	Cytology (Pap)	40-44	43	Not performed	Satisfactory	NILM	NILM	Negative (NILM)	
P02822	2018	2018-Q4	2018-12	Pre-pandemic	Cytology (Pap)	40-44	44	Not performed	Satisfactory	NILM	NILM	Negative (NILM)	
P02823	2018	2018-Q4	2018-12	Pre-pandemic	Cytology (Pap)	40-44	40	Not performed	Satisfactory	NILM	NILM	Negative (NILM)	Trichomonas
P02824	2018	2018-Q4	2018-12	Pre-pandemic	Cytology (Pap)	30-34	32	Not performed	Satisfactory	NILM	NILM	Negative (NILM)	
P02825	2018	2018-Q4	2018-12	Pre-pandemic	Cytology (Pap)	50-54	54	Not performed	Satisfactory	NILM	NILM	Negative (NILM)	
P02826	2018	2018-Q4	2018-12	Pre-pandemic	Cytology (Pap)	55-59	57	Not performed	Satisfactory	NILM	NILM	Negative (NILM)	
P02827	2018	2018-Q4	2018-12	Pre-pandemic	Cytology (Pap)	25-29	26	Not performed	Satisfactory	NILM	NILM	Negative (NILM)	
P02828	2018	2018-Q4	2018-12	Pre-pandemic	Cytology (Pap)	40-44	40	Not performed	Satisfactory	Positive	HSIL	High-grade squamous	
P02829	2018	2018-Q4	2018-12	Pre-pandemic	Cytology (Pap)	40-44	41	Not performed	Satisfactory	NILM	NILM	Negative (NILM)	
P02830	2018	2018-Q4	2018-12	Pre-pandemic	Cytology (Pap)	45-49	49	Not performed	Satisfactory	NILM	NILM	Negative (NILM)	
P02831	2018	2018-Q4	2018-12	Pre-pandemic	Cytology (Pap)	55-59	59	Not performed	Satisfactory	NILM	NILM	Negative (NILM)	
P02832	2018	2018-Q4	2018-12	Pre-pandemic	Cytology (Pap)	30-34	33	Not performed	Satisfactory	Positive	ASC-US	Low-grade / equivocal squamous	
P02833	2018	2018-Q4	2018-12	Pre-pandemic	Cytology (Pap)	40-44	44	Not performed	Satisfactory	NILM	NILM	Negative (NILM)	
P02834	2018	2018-Q4	2018-12	Pre-pandemic	Cytology (Pap)	40-44	41	Not performed	Satisfactory	NILM	NILM	Negative (NILM)	
P02835	2018	2018-Q4	2018-12	Pre-pandemic	Cytology (Pap)	40-44	43	Not performed	Satisfactory	NILM	NILM	Negative (NILM)	
P02836	2018	2018-Q4	2018-12	Pre-pandemic	Cytology (Pap)	30-34	31	Not performed	Satisfactory	NILM	NILM	Negative (NILM)	
P02837	2018	2018-Q4	2018-12	Pre-pandemic	Cytology (Pap)	45-49	48	Not performed	Satisfactory	NILM	NILM	Negative (NILM)	Candida
P02838	2018	2018-Q4	2018-12	Pre-pandemic	Cytology (Pap)	40-44	41	Not performed	Satisfactory	NILM	NILM	Negative (NILM)	
P02839	2018	2018-Q4	2018-12	Pre-pandemic	Cytology (Pap)	35-39	37	Not performed	Satisfactory	Positive	ASC-US	Low-grade / equivocal squamous	
P02840	2018	2018-Q4	2018-12	Pre-pandemic	Cytology (Pap)	35-39	35	Not performed	Satisfactory	Positive	ASC-H	High-grade squamous	
P02841	2018	2018-Q4	2018-12	Pre-pandemic	Cytology (Pap)	35-39	39	Not performed	Satisfactory	NILM	NILM	Negative (NILM)	
P02842	2018	2018-Q4	2018-12	Pre-pandemic	Cytology (Pap)	30-34	32	Not performed	Satisfactory	NILM	NILM	Negative (NILM)	
P02843	2018	2018-Q4	2018-12	Pre-pandemic	Cytology (Pap)	30-34	31	Not performed	Unsatisfactory	Unsatisfactory	Unsatisfactory	Unsatisfactory	
P02844	2018	2018-Q4	2018-12	Pre-pandemic	Cytology (Pap)	35-39	35	Not performed	Satisfactory	NILM	NILM	Negative (NILM)	
P02845	2018	2018-Q4	2018-12	Pre-pandemic	Cytology (Pap)	40-44	42	Not performed	Satisfactory	Positive	HSIL	High-grade squamous	
P02846	2018	2018-Q4	2018-12	Pre-pandemic	Cytology (Pap)	45-49	48	Not performed	Satisfactory	NILM	NILM	Negative (NILM)	
P02847	2018	2018-Q4	2018-12	Pre-pandemic	Cytology (Pap)	55-59	59	Not performed	Satisfactory	NILM	NILM	Negative (NILM)	
P02848	2018	2018-Q4	2018-12	Pre-pandemic	Cytology (Pap)	Not recorded		Not performed	Satisfactory	NILM	NILM	Negative (NILM)	
P02849	2018	2018-Q4	2018-12	Pre-pandemic	Cytology (Pap)	50-54	52	Not performed	Satisfactory	NILM	NILM	Negative (NILM)	
P02850	2018	2018-Q4	2018-12	Pre-pandemic	Cytology (Pap)	30-34	31	Not performed	Satisfactory	NILM	NILM	Negative (NILM)	
P02851	2018	2018-Q4	2018-12	Pre-pandemic	Cytology (Pap)	35-39	38	Not performed	Satisfactory	Positive	HSIL	High-grade squamous	
P02852	2018	2018-Q4	2018-12	Pre-pandemic	Cytology (Pap)	40-44	40	Not performed	Satisfactory	NILM	NILM	Negative (NILM)	
P02853	2018	2018-Q4	2018-12	Pre-pandemic	Cytology (Pap)	35-39	38	Not performed	Satisfactory	Positive	ASC-US	Low-grade / equivocal squamous	
P02854	2018	2018-Q4	2018-12	Pre-pandemic	Cytology (Pap)	45-49	46	Not performed	Satisfactory	NILM	NILM	Negative (NILM)	
P02855	2018	2018-Q4	2018-12	Pre-pandemic	Cytology (Pap)	25-29	28	Not performed	Satisfactory	NILM	NILM	Negative (NILM)	
P02856	2018	2018-Q4	2018-12	Pre-pandemic	Cytology (Pap)	30-34	34	Not performed	Satisfactory	Positive	ASC-US	Low-grade / equivocal squamous	
P02857	2018	2018-Q4	2018-12	Pre-pandemic	Cytology (Pap)	40-44	41	Not performed	Satisfactory	NILM	NILM	Negative (NILM)	
P02858	2018	2018-Q4	2018-12	Pre-pandemic	Cytology (Pap)	60-64	60	Not performed	Satisfactory	NILM	NILM	Negative (NILM)	Candida
P02859	2018	2018-Q4	2018-12	Pre-pandemic	Cytology (Pap)	Not recorded		Not performed	Satisfactory	Positive	ASC-US	Low-grade / equivocal squamous	
P02860	2018	2018-Q4	2018-12	Pre-pandemic	Cytology (Pap)	Not recorded		Not performed	Unsatisfactory	Unsatisfactory	Unsatisfactory	Unsatisfactory	
P02861	2018	2018-Q4	2018-12	Pre-pandemic	Cytology (Pap)	30-34	32	Not performed	Satisfactory	NILM	NILM	Negative (NILM)	
P02862	2018	2018-Q4	2018-12	Pre-pandemic	Cytology (Pap)	30-34	33	Not performed	Unsatisfactory	Unsatisfactory	Unsatisfactory	Unsatisfactory	
P02863	2018	2018-Q4	2018-12	Pre-pandemic	Cytology (Pap)	40-44	43	Not performed	Satisfactory	NILM	NILM	Negative (NILM)	
P02864	2018	2018-Q4	2018-12	Pre-pandemic	Cytology (Pap)	45-49	48	Not performed	Satisfactory	NILM	NILM	Negative (NILM)	
P02865	2018	2018-Q4	2018-12	Pre-pandemic	Cytology (Pap)	55-59	56	Not performed	Satisfactory	NILM	NILM	Negative (NILM)	
P02866	2018	2018-Q4	2018-12	Pre-pandemic	Cytology (Pap)	35-39	35	Not performed	Satisfactory	NILM	NILM	Negative (NILM)	
P02867	2018	2018-Q4	2018-12	Pre-pandemic	Cytology (Pap)	45-49	48	Not performed	Satisfactory	NILM	NILM	Negative (NILM)	Gardnerella
P02868	2018	2018-Q4	2018-12	Pre-pandemic	Cytology (Pap)	35-39	36	Not performed	Satisfactory	NILM	NILM	Negative (NILM)	
P02869	2018	2018-Q4	2018-12	Pre-pandemic	Cytology (Pap)	30-34	31	Not performed	Satisfactory	NILM	NILM	Negative (NILM)	
P02870	2018	2018-Q4	2018-12	Pre-pandemic	Cytology (Pap)	55-59	56	Not performed	Satisfactory	NILM	NILM	Negative (NILM)	Other
P02871	2018	2018-Q4	2018-12	Pre-pandemic	Cytology (Pap)	30-34	32	Not performed	Satisfactory	NILM	NILM	Negative (NILM)	
P02872	2018	2018-Q4	2018-12	Pre-pandemic	Cytology (Pap)	50-54	54	Not performed	Satisfactory	NILM	NILM	Negative (NILM)	
P02873	2018	2018-Q4	2018-12	Pre-pandemic	Cytology (Pap)	45-49	49	Not performed	Satisfactory	NILM	NILM	Negative (NILM)	
P02874	2018	2018-Q4	2018-12	Pre-pandemic	Cytology (Pap)	30-34	31	Not performed	Satisfactory	NILM	NILM	Negative (NILM)	
P02875	2018	2018-Q4	2018-12	Pre-pandemic	Cytology (Pap)	40-44	41	Not performed	Satisfactory	NILM	NILM	Negative (NILM)	
P02876	2018	2018-Q4	2018-12	Pre-pandemic	Cytology (Pap)	40-44	43	Not performed	Satisfactory	NILM	NILM	Negative (NILM)	
P02877	2018	2018-Q4	2018-12	Pre-pandemic	Cytology (Pap)	45-49	48	Not performed	Satisfactory	NILM	NILM	Negative (NILM)	
P02878	2018	2018-Q4	2018-12	Pre-pandemic	Cytology (Pap)	55-59	59	Not performed	Satisfactory	NILM	NILM	Negative (NILM)	

patient_id	year	quarter	month	period	primary_test	age_group	age_years	hpv_result	adequacy	result	bethesda	grade	infection
P02879	2018	2018-Q4	2018-12	Pre-pandemic	Cytology (Pap)	35-39	36	Not performed	Satisfactory	NILM	NILM	Negative (NILM)	
P02880	2018	2018-Q4	2018-12	Pre-pandemic	Cytology (Pap)	45-49	45	Not performed	Satisfactory	NILM	NILM	Negative (NILM)	
P02881	2018	2018-Q4	2018-12	Pre-pandemic	Cytology (Pap)	50-54	50	Not performed	Satisfactory	NILM	NILM	Negative (NILM)	
P02882	2018	2018-Q4	2018-12	Pre-pandemic	Cytology (Pap)	40-44	44	Not performed	Satisfactory	NILM	NILM	Negative (NILM)	
P02883	2018	2018-Q4	2018-12	Pre-pandemic	Cytology (Pap)	45-49	46	Not performed	Satisfactory	NILM	NILM	Negative (NILM)	
P02884	2018	2018-Q4	2018-12	Pre-pandemic	Cytology (Pap)	35-39	37	Not performed	Satisfactory	NILM	NILM	Negative (NILM)	
P02885	2018	2018-Q4	2018-12	Pre-pandemic	Cytology (Pap)	25-29	29	Not performed	Satisfactory	NILM	NILM	Negative (NILM)	
P02886	2018	2018-Q4	2018-12	Pre-pandemic	Cytology (Pap)	30-34	30	Not performed	Satisfactory	NILM	NILM	Negative (NILM)	
P02887	2018	2018-Q4	2018-12	Pre-pandemic	Cytology (Pap)	25-29	25	Not performed	Satisfactory	Positive	ASC-US	Low-grade / equivocal squamous	Gardnerella
P02888	2018	2018-Q4	2018-12	Pre-pandemic	Cytology (Pap)	45-49	46	Not performed	Unsatisfactory	Unsatisfactory	Unsatisfactory	Unsatisfactory	
P02889	2018	2018-Q4	2018-12	Pre-pandemic	Cytology (Pap)	Not recorded		Not performed	Satisfactory	NILM	NILM	Negative (NILM)	
P02890	2018	2018-Q4	2018-12	Pre-pandemic	Cytology (Pap)	45-49	48	Not performed	Satisfactory	NILM	NILM	Negative (NILM)	Candida
P02891	2018	2018-Q4	2018-12	Pre-pandemic	Cytology (Pap)	35-39	39	Not performed	Satisfactory	NILM	NILM	Negative (NILM)	
P02892	2018	2018-Q4	2018-12	Pre-pandemic	Cytology (Pap)	25-29	28	Not performed	Satisfactory	NILM	NILM	Negative (NILM)	
P02893	2018	2018-Q4	2018-12	Pre-pandemic	Cytology (Pap)	30-34	30	Not performed	Satisfactory	NILM	NILM	Negative (NILM)	
P02894	2018	2018-Q4	2018-12	Pre-pandemic	Cytology (Pap)	60-64	64	Not performed	Satisfactory	NILM	NILM	Negative (NILM)	
P02895	2018	2018-Q4	2018-12	Pre-pandemic	Cytology (Pap)	25-29	25	Not performed	Satisfactory	NILM	NILM	Negative (NILM)	
P02896	2018	2018-Q4	2018-12	Pre-pandemic	Cytology (Pap)	35-39	39	Not performed	Satisfactory	NILM	NILM	Negative (NILM)	
P02897	2018	2018-Q4	2018-12	Pre-pandemic	Cytology (Pap)	45-49	47	Not performed	Unsatisfactory	Unsatisfactory	Unsatisfactory	Unsatisfactory	
P02898	2018	2018-Q4	2018-12	Pre-pandemic	Cytology (Pap)	25-29	25	Not performed	Satisfactory	Positive	LSIL	Low-grade / equivocal squamous	
P02899	2018	2018-Q4	2018-12	Pre-pandemic	Cytology (Pap)	Not recorded		Not performed	Satisfactory	Positive	ASC-US	Low-grade / equivocal squamous	
P02900	2018	2018-Q4	2018-12	Pre-pandemic	Cytology (Pap)	Not recorded		Not performed	Satisfactory	NILM	NILM	Negative (NILM)	
P02901	2018	2018-Q4	2018-12	Pre-pandemic	Cytology (Pap)	55-59	59	Not performed	Satisfactory	NILM	NILM	Negative (NILM)	
P02902	2018	2018-Q4	2018-12	Pre-pandemic	Cytology (Pap)	30-34	30	Not performed	Satisfactory	Positive	ASC-US	Low-grade / equivocal squamous	
P02903	2018	2018-Q4	2018-12	Pre-pandemic	Cytology (Pap)	35-39	39	Not performed	Satisfactory	NILM	NILM	Negative (NILM)	Trichomonas
P02904	2018	2018-Q4	2018-12	Pre-pandemic	Cytology (Pap)	60-64	62	Not performed	Satisfactory	Positive	ASC-H	High-grade squamous	Candida
P02905	2018	2018-Q4	2018-12	Pre-pandemic	Cytology (Pap)	55-59	58	Not performed	Satisfactory	NILM	NILM	Negative (NILM)	
P02906	2018	2018-Q4	2018-12	Pre-pandemic	Cytology (Pap)	35-39	35	Not performed	Satisfactory	NILM	NILM	Negative (NILM)	
P02907	2018	2018-Q4	2018-12	Pre-pandemic	Cytology (Pap)	40-44	44	Not performed	Satisfactory	NILM	NILM	Negative (NILM)	
P02908	2018	2018-Q4	2018-12	Pre-pandemic	Cytology (Pap)	50-54	52	Not performed	Satisfactory	Positive	ASC-US	Low-grade / equivocal squamous	
P02909	2018	2018-Q4	2018-12	Pre-pandemic	Cytology (Pap)	55-59	59	Not performed	Satisfactory	Positive	HSIL	High-grade squamous	
P02910	2018	2018-Q4	2018-12	Pre-pandemic	Cytology (Pap)	50-54	50	Not performed	Satisfactory	NILM	NILM	Negative (NILM)	
P02911	2018	2018-Q4	2018-12	Pre-pandemic	Cytology (Pap)	45-49	47	Not performed	Satisfactory	NILM	NILM	Negative (NILM)	
P02912	2018	2018-Q4	2018-12	Pre-pandemic	Cytology (Pap)	60-64	64	Not performed	Satisfactory	NILM	NILM	Negative (NILM)	
P02913	2018	2018-Q4	2018-12	Pre-pandemic	Cytology (Pap)	40-44	43	Not performed	Satisfactory	NILM	NILM	Negative (NILM)	
P02914	2018	2018-Q4	2018-12	Pre-pandemic	Cytology (Pap)	40-44	42	Not performed	Satisfactory	Positive	LSIL	Low-grade / equivocal squamous	
P02915	2018	2018-Q4	2018-12	Pre-pandemic	Cytology (Pap)	Not recorded		Not performed	Satisfactory	NILM	NILM	Negative (NILM)	
P02916	2018	2018-Q4	2018-12	Pre-pandemic	Cytology (Pap)	35-39	37	Not performed	Satisfactory	NILM	NILM	Negative (NILM)	
P02917	2018	2018-Q4	2018-12	Pre-pandemic	Cytology (Pap)	45-49	47	Not performed	Satisfactory	Positive	LSIL	Low-grade / equivocal squamous	
P02918	2018	2018-Q4	2018-12	Pre-pandemic	Cytology (Pap)	50-54	50	Not performed	Satisfactory	NILM	NILM	Negative (NILM)	
P02919	2019	2019-Q1	2019-01	Pre-pandemic	Cytology (Pap)	45-49	48	Not performed	Satisfactory	Positive	ASC-US	Low-grade / equivocal squamous	
P02920	2019	2019-Q1	2019-01	Pre-pandemic	Cytology (Pap)	30-34	30	Not performed	Satisfactory	Positive	LSIL	Low-grade / equivocal squamous	
P02921	2019	2019-Q1	2019-01	Pre-pandemic	Cytology (Pap)	35-39	39	Not performed	Satisfactory	NILM	NILM	Negative (NILM)	
P02922	2019	2019-Q1	2019-01	Pre-pandemic	Cytology (Pap)	40-44	41	Not performed	Satisfactory	NILM	NILM	Negative (NILM)	
P02923	2019	2019-Q1	2019-01	Pre-pandemic	Cytology (Pap)	40-44	41	Not performed	Satisfactory	NILM	NILM	Negative (NILM)	
P02924	2019	2019-Q1	2019-01	Pre-pandemic	Cytology (Pap)	55-59	59	Not performed	Satisfactory	NILM	NILM	Negative (NILM)	
P02925	2019	2019-Q1	2019-01	Pre-pandemic	Cytology (Pap)	35-39	39	Not performed	Satisfactory	NILM	NILM	Negative (NILM)	
P02926	2019	2019-Q1	2019-01	Pre-pandemic	Cytology (Pap)	30-34	32	Not performed	Satisfactory	NILM	NILM	Negative (NILM)	
P02927	2019	2019-Q1	2019-01	Pre-pandemic	Cytology (Pap)	30-34	30	Not performed	Satisfactory	Positive	LSIL	Low-grade / equivocal squamous	
P02928	2019	2019-Q1	2019-01	Pre-pandemic	Cytology (Pap)	40-44	40	Not performed	Satisfactory	Positive	HSIL	High-grade squamous	
P02929	2019	2019-Q1	2019-01	Pre-pandemic	Cytology (Pap)	35-39	35	Not performed	Satisfactory	NILM	NILM	Negative (NILM)	
P02930	2019	2019-Q1	2019-01	Pre-pandemic	Cytology (Pap)	40-44	44	Not performed	Satisfactory	NILM	NILM	Negative (NILM)	
P02931	2019	2019-Q1	2019-01	Pre-pandemic	Cytology (Pap)	30-34	33	Not performed	Satisfactory	NILM	NILM	Negative (NILM)	
P02932	2019	2019-Q1	2019-01	Pre-pandemic	Cytology (Pap)	25-29	25	Not performed	Satisfactory	NILM	NILM	Negative (NILM)	
P02933	2019	2019-Q1	2019-01	Pre-pandemic	Cytology (Pap)	25-29	25	Not performed	Satisfactory	NILM	NILM	Negative (NILM)	
P02934	2019	2019-Q1	2019-01	Pre-pandemic	Cytology (Pap)	30-34	34	Not performed	Satisfactory	Positive	ASC-US	Low-grade / equivocal squamous	
P02935	2019	2019-Q1	2019-01	Pre-pandemic	Cytology (Pap)	45-49	49	Not performed	Satisfactory	NILM	NILM	Negative (NILM)	
P02936	2019	2019-Q1	2019-01	Pre-pandemic	Cytology (Pap)	40-44	43	Not performed	Satisfactory	NILM	NILM	Negative (NILM)	
P02937	2019	2019-Q1	2019-01	Pre-pandemic	Cytology (Pap)	55-59	56	Not performed	Satisfactory	NILM	NILM	Negative (NILM)	
P02938	2019	2019-Q1	2019-01	Pre-pandemic	Cytology (Pap)	55-59	55	Not performed	Satisfactory	Positive	LSIL	Low-grade / equivocal squamous	
P02939	2019	2019-Q1	2019-01	Pre-pandemic	Cytology (Pap)	50-54	52	Not performed	Satisfactory	NILM	NILM	Negative (NILM)	
P02940	2019	2019-Q1	2019-01	Pre-pandemic	Cytology (Pap)	30-34	32	Not performed	Satisfactory	NILM	NILM	Negative (NILM)	
P02941	2019	2019-Q1	2019-01	Pre-pandemic	Cytology (Pap)	60-64	63	Not performed	Satisfactory	NILM	NILM	Negative (NILM)	
P02942	2019	2019-Q1	2019-01	Pre-pandemic	Cytology (Pap)	40-44	43	Not performed	Satisfactory	NILM	NILM	Negative (NILM)	
P02943	2019	2019-Q1	2019-01	Pre-pandemic	Cytology (Pap)	60-64	60	Not performed	Satisfactory	NILM	NILM	Negative (NILM)	
P02944	2019	2019-Q1	2019-01	Pre-pandemic	Cytology (Pap)	50-54	50	Not performed	Satisfactory	NILM	NILM	Negative (NILM)	
P02945	2019	2019-Q1	2019-01	Pre-pandemic	Cytology (Pap)	50-54	53	Not performed	Satisfactory	NILM	NILM	Negative (NILM)	
P02946	2019	2019-Q1	2019-01	Pre-pandemic	Cytology (Pap)	45-49	47	Not performed	Satisfactory	NILM	NILM	Negative (NILM)	
P02947	2019	2019-Q1	2019-01	Pre-pandemic	Cytology (Pap)	45-49	46	Not performed	Satisfactory	NILM	NILM	Negative (NILM)	
P02948	2019	2019-Q1	2019-01	Pre-pandemic	Cytology (Pap)	60-64	61	Not performed	Satisfactory	NILM	NILM	Negative (NILM)	
P02949	2019	2019-Q1	2019-01	Pre-pandemic	Cytology (Pap)	35-39	35	Not performed	Satisfactory	NILM	NILM	Negative (NILM)	
P02950	2019	2019-Q1	2019-01	Pre-pandemic	Cytology (Pap)	50-54	52	Not performed	Satisfactory	NILM	NILM	Negative (NILM)	
P02951	2019	2019-Q1	2019-01	Pre-pandemic	Cytology (Pap)	45-49	47	Not performed	Satisfactory	NILM	NILM	Negative (NILM)	
P02952	2019	2019-Q1	2019-01	Pre-pandemic	Cytology (Pap)	60-64	61	Not performed	Satisfactory	NILM	NILM	Negative (NILM)	
P02953	2019	2019-Q1	2019-01	Pre-pandemic	Cytology (Pap)	30-34	30	Not performed	Satisfactory	NILM	NILM	Negative (NILM)	
P02954	2019	2019-Q1	2019-01	Pre-pandemic	Cytology (Pap)	35-39	35	Not performed	Satisfactory	NILM	NILM	Negative (NILM)	
P02955	2019	2019-Q1	2019-01	Pre-pandemic	Cytology (Pap)	40-44	44	Not performed	Satisfactory	NILM	NILM	Negative (NILM)	
P02956	2019	2019-Q1	2019-01	Pre-pandemic	Cytology (Pap)	35-39	39	Not performed	Satisfactory	NILM	NILM	Negative (NILM)	
P02957	2019	2019-Q1	2019-01	Pre-pandemic	Cytology (Pap)	50-54	52	Not performed	Satisfactory	NILM	NILM	Negative (NILM)	
P02958	2019	2019-Q1	2019-01	Pre-pandemic	Cytology (Pap)	50-54	51	Not performed	Satisfactory	NILM	NILM	Negative (NILM)	

patient_id	year	quarter	month	period	primary_test	age_group	age_years	hpv_result	adequacy	result	bethesda	grade	infection
P02959	2019	2019-Q1	2019-01	Pre-pandemic	Cytology (Pap)	55-59	56	Not performed	Satisfactory	NILM	NILM	Negative (NILM)	
P02960	2019	2019-Q1	2019-01	Pre-pandemic	Cytology (Pap)	25-29	27	Not performed	Satisfactory	NILM	NILM	Negative (NILM)	
P02961	2019	2019-Q1	2019-01	Pre-pandemic	Cytology (Pap)	55-59	59	Not performed	Satisfactory	NILM	NILM	Negative (NILM)	
P02962	2019	2019-Q1	2019-01	Pre-pandemic	Cytology (Pap)	55-59	58	Not performed	Satisfactory	NILM	NILM	Negative (NILM)	
P02963	2019	2019-Q1	2019-01	Pre-pandemic	Cytology (Pap)	45-49	46	Not performed	Satisfactory	NILM	NILM	Negative (NILM)	
P02964	2019	2019-Q1	2019-01	Pre-pandemic	Cytology (Pap)	30-34	33	Not performed	Satisfactory	NILM	NILM	Negative (NILM)	
P02965	2019	2019-Q1	2019-01	Pre-pandemic	Cytology (Pap)	30-34	34	Not performed	Satisfactory	NILM	NILM	Negative (NILM)	
P02966	2019	2019-Q1	2019-01	Pre-pandemic	Cytology (Pap)	40-44	41	Not performed	Satisfactory	NILM	NILM	Negative (NILM)	
P02967	2019	2019-Q1	2019-01	Pre-pandemic	Cytology (Pap)	30-34	31	Not performed	Satisfactory	NILM	NILM	Negative (NILM)	
P02968	2019	2019-Q1	2019-01	Pre-pandemic	Cytology (Pap)	55-59	59	Not performed	Satisfactory	NILM	NILM	Negative (NILM)	
P02969	2019	2019-Q1	2019-01	Pre-pandemic	Cytology (Pap)	40-44	44	Not performed	Satisfactory	NILM	NILM	Negative (NILM)	
P02970	2019	2019-Q1	2019-01	Pre-pandemic	Cytology (Pap)	40-44	44	Not performed	Satisfactory	NILM	NILM	Negative (NILM)	
P02971	2019	2019-Q1	2019-01	Pre-pandemic	Cytology (Pap)	60-64	62	Not performed	Satisfactory	NILM	NILM	Negative (NILM)	
P02972	2019	2019-Q1	2019-01	Pre-pandemic	Cytology (Pap)	45-49	49	Not performed	Satisfactory	NILM	NILM	Negative (NILM)	
P02973	2019	2019-Q1	2019-01	Pre-pandemic	Cytology (Pap)	30-34	34	Not performed	Satisfactory	Positive	ASC-US	Low-grade / equivocal squamous	
P02974	2019	2019-Q1	2019-01	Pre-pandemic	Cytology (Pap)	30-34	33	Not performed	Satisfactory	NILM	NILM	Negative (NILM)	
P02975	2019	2019-Q1	2019-01	Pre-pandemic	Cytology (Pap)	35-39	38	Not performed	Satisfactory	NILM	NILM	Negative (NILM)	
P02976	2019	2019-Q1	2019-01	Pre-pandemic	Cytology (Pap)	55-59	57	Not performed	Satisfactory	NILM	NILM	Negative (NILM)	
P02977	2019	2019-Q1	2019-01	Pre-pandemic	Cytology (Pap)	45-49	49	Not performed	Satisfactory	NILM	NILM	Negative (NILM)	
P02978	2019	2019-Q1	2019-01	Pre-pandemic	Cytology (Pap)	50-54	50	Not performed	Satisfactory	NILM	NILM	Negative (NILM)	
P02979	2019	2019-Q1	2019-01	Pre-pandemic	Cytology (Pap)	50-54	53	Not performed	Satisfactory	NILM	NILM	Negative (NILM)	
P02980	2019	2019-Q1	2019-01	Pre-pandemic	Cytology (Pap)	45-49	46	Not performed	Satisfactory	NILM	NILM	Negative (NILM)	
P02981	2019	2019-Q1	2019-01	Pre-pandemic	Cytology (Pap)	50-54	53	Not performed	Satisfactory	Positive	LSIL	Low-grade / equivocal squamous	Trichomonas
P02982	2019	2019-Q1	2019-01	Pre-pandemic	Cytology (Pap)	30-34	33	Not performed	Satisfactory	NILM	NILM	Negative (NILM)	
P02983	2019	2019-Q1	2019-01	Pre-pandemic	Cytology (Pap)	30-34	30	Not performed	Satisfactory	Positive	ASC-US	Low-grade / equivocal squamous	Gardnerella
P02984	2019	2019-Q1	2019-01	Pre-pandemic	Cytology (Pap)	35-39	35	Not performed	Satisfactory	NILM	NILM	Negative (NILM)	
P02985	2019	2019-Q1	2019-01	Pre-pandemic	Cytology (Pap)	40-44	44	Not performed	Satisfactory	NILM	NILM	Negative (NILM)	
P02986	2019	2019-Q1	2019-01	Pre-pandemic	Cytology (Pap)	60-64	60	Not performed	Satisfactory	NILM	NILM	Negative (NILM)	
P02987	2019	2019-Q1	2019-01	Pre-pandemic	Cytology (Pap)	40-44	40	Not performed	Satisfactory	NILM	NILM	Negative (NILM)	
P02988	2019	2019-Q1	2019-01	Pre-pandemic	Cytology (Pap)	25-29	28	Not performed	Satisfactory	NILM	NILM	Negative (NILM)	
P02989	2019	2019-Q1	2019-01	Pre-pandemic	Cytology (Pap)	35-39	36	Not performed	Satisfactory	NILM	NILM	Negative (NILM)	
P02990	2019	2019-Q1	2019-01	Pre-pandemic	Cytology (Pap)	30-34	33	Not performed	Satisfactory	NILM	NILM	Negative (NILM)	
P02991	2019	2019-Q1	2019-01	Pre-pandemic	Cytology (Pap)	40-44	42	Not performed	Satisfactory	Positive	ASC-US	Low-grade / equivocal squamous	
P02992	2019	2019-Q1	2019-01	Pre-pandemic	Cytology (Pap)	40-44	40	Not performed	Satisfactory	NILM	NILM	Negative (NILM)	
P02993	2019	2019-Q1	2019-01	Pre-pandemic	Cytology (Pap)	35-39	39	Not performed	Satisfactory	NILM	NILM	Negative (NILM)	
P02994	2019	2019-Q1	2019-01	Pre-pandemic	Cytology (Pap)	60-64	64	Not performed	Satisfactory	NILM	NILM	Negative (NILM)	
P02995	2019	2019-Q1	2019-01	Pre-pandemic	Cytology (Pap)	45-49	45	Not performed	Satisfactory	NILM	NILM	Negative (NILM)	
P02996	2019	2019-Q1	2019-01	Pre-pandemic	Cytology (Pap)	35-39	37	Not performed	Satisfactory	NILM	NILM	Negative (NILM)	
P02997	2019	2019-Q1	2019-01	Pre-pandemic	Cytology (Pap)	45-49	46	Not performed	Satisfactory	NILM	NILM	Negative (NILM)	
P02998	2019	2019-Q1	2019-01	Pre-pandemic	Cytology (Pap)	60-64	61	Not performed	Satisfactory	Positive	HSIL	High-grade squamous	
P02999	2019	2019-Q1	2019-01	Pre-pandemic	Cytology (Pap)	35-39	36	Not performed	Satisfactory	Positive	ASC-US	Low-grade / equivocal squamous	
P03000	2019	2019-Q1	2019-01	Pre-pandemic	Cytology (Pap)	55-59	57	Not performed	Satisfactory	Positive	ASC-US	Low-grade / equivocal squamous	
P03001	2019	2019-Q1	2019-01	Pre-pandemic	Cytology (Pap)	35-39	36	Not performed	Satisfactory	NILM	NILM	Negative (NILM)	
P03002	2019	2019-Q1	2019-01	Pre-pandemic	Cytology (Pap)	55-59	57	Not performed	Satisfactory	NILM	NILM	Negative (NILM)	
P03003	2019	2019-Q1	2019-01	Pre-pandemic	Cytology (Pap)	45-49	48	Not performed	Satisfactory	NILM	NILM	Negative (NILM)	
P03004	2019	2019-Q1	2019-01	Pre-pandemic	Cytology (Pap)	45-49	48	Not performed	Satisfactory	Positive	LSIL	Low-grade / equivocal squamous	
P03005	2019	2019-Q1	2019-01	Pre-pandemic	Cytology (Pap)	40-44	44	Not performed	Satisfactory	NILM	NILM	Negative (NILM)	
P03006	2019	2019-Q1	2019-01	Pre-pandemic	Cytology (Pap)	45-49	47	Not performed	Satisfactory	NILM	NILM	Negative (NILM)	
P03007	2019	2019-Q1	2019-01	Pre-pandemic	Cytology (Pap)	50-54	52	Not performed	Satisfactory	NILM	NILM	Negative (NILM)	
P03008	2019	2019-Q1	2019-01	Pre-pandemic	Cytology (Pap)	35-39	37	Not performed	Satisfactory	Positive	HSIL	High-grade squamous	
P03009	2019	2019-Q1	2019-01	Pre-pandemic	Cytology (Pap)	40-44	41	Not performed	Satisfactory	NILM	NILM	Negative (NILM)	
P03010	2019	2019-Q1	2019-01	Pre-pandemic	Cytology (Pap)	55-59	58	Not performed	Satisfactory	NILM	NILM	Negative (NILM)	
P03011	2019	2019-Q1	2019-01	Pre-pandemic	Cytology (Pap)	55-59	59	Not performed	Satisfactory	NILM	NILM	Negative (NILM)	
P03012	2019	2019-Q1	2019-01	Pre-pandemic	Cytology (Pap)	35-39	37	Not performed	Satisfactory	NILM	NILM	Negative (NILM)	
P03013	2019	2019-Q1	2019-01	Pre-pandemic	Cytology (Pap)	30-34	32	Not performed	Satisfactory	Positive	ASC-US	Low-grade / equivocal squamous	
P03014	2019	2019-Q1	2019-01	Pre-pandemic	Cytology (Pap)	40-44	40	Not performed	Satisfactory	NILM	NILM	Negative (NILM)	
P03015	2019	2019-Q1	2019-01	Pre-pandemic	Cytology (Pap)	55-59	58	Not performed	Unsatisfactory	Unsatisfactory	Unsatisfactory	Unsatisfactory	
P03016	2019	2019-Q1	2019-01	Pre-pandemic	Cytology (Pap)	45-49	48	Not performed	Satisfactory	NILM	NILM	Negative (NILM)	
P03017	2019	2019-Q1	2019-01	Pre-pandemic	Cytology (Pap)	35-39	38	Not performed	Satisfactory	NILM	NILM	Negative (NILM)	
P03018	2019	2019-Q1	2019-01	Pre-pandemic	Cytology (Pap)	40-44	43	Not performed	Satisfactory	NILM	NILM	Negative (NILM)	
P03019	2019	2019-Q1	2019-01	Pre-pandemic	Cytology (Pap)	45-49	47	Not performed	Satisfactory	NILM	NILM	Negative (NILM)	
P03020	2019	2019-Q1	2019-01	Pre-pandemic	Cytology (Pap)	50-54	51	Not performed	Satisfactory	NILM	NILM	Negative (NILM)	
P03021	2019	2019-Q1	2019-01	Pre-pandemic	Cytology (Pap)	40-44	40	Not performed	Satisfactory	NILM	NILM	Negative (NILM)	
P03022	2019	2019-Q1	2019-01	Pre-pandemic	Cytology (Pap)	40-44	43	Not performed	Satisfactory	NILM	NILM	Negative (NILM)	
P03023	2019	2019-Q1	2019-01	Pre-pandemic	Cytology (Pap)	45-49	47	Not performed	Satisfactory	NILM	NILM	Negative (NILM)	
P03024	2019	2019-Q1	2019-01	Pre-pandemic	Cytology (Pap)	35-39	39	Not performed	Satisfactory	NILM	NILM	Negative (NILM)	
P03025	2019	2019-Q1	2019-01	Pre-pandemic	Cytology (Pap)	40-44	42	Not performed	Satisfactory	NILM	NILM	Negative (NILM)	
P03026	2019	2019-Q1	2019-01	Pre-pandemic	Cytology (Pap)	50-54	50	Not performed	Satisfactory	NILM	NILM	Negative (NILM)	
P03027	2019	2019-Q1	2019-01	Pre-pandemic	Cytology (Pap)	30-34	33	Not performed	Satisfactory	NILM	NILM	Negative (NILM)	
P03028	2019	2019-Q1	2019-01	Pre-pandemic	Cytology (Pap)	25-29	29	Not performed	Satisfactory	NILM	NILM	Negative (NILM)	
P03029	2019	2019-Q1	2019-01	Pre-pandemic	Cytology (Pap)	45-49	48	Not performed	Satisfactory	NILM	NILM	Negative (NILM)	
P03030	2019	2019-Q1	2019-01	Pre-pandemic	Cytology (Pap)	30-34	32	Not performed	Satisfactory	NILM	NILM	Negative (NILM)	
P03031	2019	2019-Q1	2019-01	Pre-pandemic	Cytology (Pap)	40-44	42	Not performed	Satisfactory	NILM	NILM	Negative (NILM)	
P03032	2019	2019-Q1	2019-01	Pre-pandemic	Cytology (Pap)	40-44	43	Not performed	Satisfactory	NILM	NILM	Negative (NILM)	Trichomonas
P03033	2019	2019-Q1	2019-01	Pre-pandemic	Cytology (Pap)	30-34	32	Not performed	Satisfactory	NILM	NILM	Negative (NILM)	
P03034	2019	2019-Q1	2019-01	Pre-pandemic	Cytology (Pap)	50-54	52	Not performed	Satisfactory	NILM	NILM	Negative (NILM)	
P03035	2019	2019-Q1	2019-01	Pre-pandemic	Cytology (Pap)	60-64	64	Not performed	Satisfactory	NILM	NILM	Negative (NILM)	
P03036	2019	2019-Q1	2019-01	Pre-pandemic	Cytology (Pap)	35-39	38	Not performed	Satisfactory	NILM	NILM	Negative (NILM)	
P03037	2019	2019-Q1	2019-01	Pre-pandemic	Cytology (Pap)	40-44	42	Not performed	Satisfactory	NILM	NILM	Negative (NILM)	
P03038	2019	2019-Q1	2019-01	Pre-pandemic	Cytology (Pap)	50-54	51	Not performed	Satisfactory	Positive	ASC-US	Low-grade / equivocal squamous	

patient_id	year	quarter	month	period	primary_test	age_group	age_years	hpv_result	adequacy	result	bethesda	grade	infection
P03039	2019	2019-Q1	2019-01	Pre-pandemic	Cytology (Pap)	50-54	52	Not performed	Satisfactory	NILM	NILM	Negative (NILM)	
P03040	2019	2019-Q1	2019-01	Pre-pandemic	Cytology (Pap)	45-49	45	Not performed	Satisfactory	NILM	NILM	Negative (NILM)	
P03041	2019	2019-Q1	2019-01	Pre-pandemic	Cytology (Pap)	45-49	45	Not performed	Satisfactory	NILM	NILM	Negative (NILM)	
P03042	2019	2019-Q1	2019-01	Pre-pandemic	Cytology (Pap)	40-44	40	Not performed	Satisfactory	Positive	HSIL	High-grade squamous	
P03043	2019	2019-Q1	2019-01	Pre-pandemic	Cytology (Pap)	45-49	46	Not performed	Satisfactory	Positive	ASC-US	Low-grade / equivocal squamous	
P03044	2019	2019-Q1	2019-01	Pre-pandemic	Cytology (Pap)	30-34	31	Not performed	Satisfactory	NILM	NILM	Negative (NILM)	
P03045	2019	2019-Q1	2019-01	Pre-pandemic	Cytology (Pap)	45-49	45	Not performed	Satisfactory	Positive	HSIL	High-grade squamous	
P03046	2019	2019-Q1	2019-01	Pre-pandemic	Cytology (Pap)	40-44	44	Not performed	Satisfactory	NILM	NILM	Negative (NILM)	
P03047	2019	2019-Q1	2019-01	Pre-pandemic	Cytology (Pap)	35-39	38	Not performed	Satisfactory	NILM	NILM	Negative (NILM)	
P03048	2019	2019-Q1	2019-01	Pre-pandemic	Cytology (Pap)	35-39	38	Not performed	Satisfactory	NILM	NILM	Negative (NILM)	
P03049	2019	2019-Q1	2019-01	Pre-pandemic	Cytology (Pap)	40-44	43	Not performed	Satisfactory	Positive	LSIL	Low-grade / equivocal squamous	
P03050	2019	2019-Q1	2019-01	Pre-pandemic	Cytology (Pap)	60-64	63	Not performed	Satisfactory	NILM	NILM	Negative (NILM)	
P03051	2019	2019-Q1	2019-01	Pre-pandemic	Cytology (Pap)	45-49	48	Not performed	Satisfactory	NILM	NILM	Negative (NILM)	
P03052	2019	2019-Q1	2019-01	Pre-pandemic	Cytology (Pap)	30-34	33	Not performed	Satisfactory	NILM	NILM	Negative (NILM)	
P03053	2019	2019-Q1	2019-01	Pre-pandemic	Cytology (Pap)	45-49	47	Not performed	Satisfactory	NILM	NILM	Negative (NILM)	
P03054	2019	2019-Q1	2019-01	Pre-pandemic	Cytology (Pap)	55-59	57	Not performed	Satisfactory	NILM	NILM	Negative (NILM)	
P03055	2019	2019-Q1	2019-01	Pre-pandemic	Cytology (Pap)	30-34	31	Not performed	Satisfactory	Positive	ASC-H	High-grade squamous	
P03056	2019	2019-Q1	2019-01	Pre-pandemic	Cytology (Pap)	30-34	34	Not performed	Satisfactory	NILM	NILM	Negative (NILM)	
P03057	2019	2019-Q1	2019-01	Pre-pandemic	Cytology (Pap)	35-39	38	Not performed	Satisfactory	NILM	NILM	Negative (NILM)	
P03058	2019	2019-Q1	2019-01	Pre-pandemic	Cytology (Pap)	45-49	48	Not performed	Satisfactory	NILM	NILM	Negative (NILM)	
P03059	2019	2019-Q1	2019-01	Pre-pandemic	Cytology (Pap)	40-44	41	Not performed	Satisfactory	NILM	NILM	Negative (NILM)	
P03060	2019	2019-Q1	2019-01	Pre-pandemic	Cytology (Pap)	40-44	41	Not performed	Satisfactory	NILM	NILM	Negative (NILM)	
P03061	2019	2019-Q1	2019-01	Pre-pandemic	Cytology (Pap)	25-29	25	Not performed	Satisfactory	Positive	LSIL	Low-grade / equivocal squamous	
P03062	2019	2019-Q1	2019-01	Pre-pandemic	Cytology (Pap)	45-49	49	Not performed	Satisfactory	NILM	NILM	Negative (NILM)	
P03063	2019	2019-Q1	2019-01	Pre-pandemic	Cytology (Pap)	35-39	38	Not performed	Satisfactory	Positive	ASC-US	Low-grade / equivocal squamous	
P03064	2019	2019-Q1	2019-01	Pre-pandemic	Cytology (Pap)	30-34	31	Not performed	Satisfactory	NILM	NILM	Negative (NILM)	
P03065	2019	2019-Q1	2019-01	Pre-pandemic	Cytology (Pap)	40-44	41	Not performed	Satisfactory	NILM	NILM	Negative (NILM)	
P03066	2019	2019-Q1	2019-01	Pre-pandemic	Cytology (Pap)	30-34	34	Not performed	Satisfactory	NILM	NILM	Negative (NILM)	
P03067	2019	2019-Q1	2019-01	Pre-pandemic	Cytology (Pap)	40-44	44	Not performed	Satisfactory	NILM	NILM	Negative (NILM)	
P03068	2019	2019-Q1	2019-01	Pre-pandemic	Cytology (Pap)	35-39	35	Not performed	Satisfactory	NILM	NILM	Negative (NILM)	
P03069	2019	2019-Q1	2019-01	Pre-pandemic	Cytology (Pap)	40-44	44	Not performed	Satisfactory	NILM	NILM	Negative (NILM)	
P03070	2019	2019-Q1	2019-01	Pre-pandemic	Cytology (Pap)	30-34	31	Not performed	Satisfactory	Positive	ASC-H	High-grade squamous	
P03071	2019	2019-Q1	2019-01	Pre-pandemic	Cytology (Pap)	40-44	44	Not performed	Satisfactory	NILM	NILM	Negative (NILM)	
P03072	2019	2019-Q1	2019-01	Pre-pandemic	Cytology (Pap)	25-29	29	Not performed	Satisfactory	NILM	NILM	Negative (NILM)	
P03073	2019	2019-Q1	2019-01	Pre-pandemic	Cytology (Pap)	60-64	64	Not performed	Unsatisfactory	Unsatisfactory	Unsatisfactory	Unsatisfactory	
P03074	2019	2019-Q1	2019-01	Pre-pandemic	Cytology (Pap)	40-44	42	Not performed	Satisfactory	NILM	NILM	Negative (NILM)	
P03075	2019	2019-Q1	2019-01	Pre-pandemic	Cytology (Pap)	30-34	32	Not performed	Satisfactory	NILM	NILM	Negative (NILM)	
P03076	2019	2019-Q1	2019-01	Pre-pandemic	Cytology (Pap)	30-34	33	Not performed	Satisfactory	Positive	ASC-US	Low-grade / equivocal squamous	
P03077	2019	2019-Q1	2019-01	Pre-pandemic	Cytology (Pap)	40-44	44	Not performed	Satisfactory	NILM	NILM	Negative (NILM)	
P03078	2019	2019-Q1	2019-01	Pre-pandemic	Cytology (Pap)	50-54	50	Not performed	Satisfactory	Positive	ASC-H	High-grade squamous	
P03079	2019	2019-Q1	2019-01	Pre-pandemic	Cytology (Pap)	40-44	40	Not performed	Satisfactory	NILM	NILM	Negative (NILM)	
P03080	2019	2019-Q1	2019-01	Pre-pandemic	Cytology (Pap)	40-44	40	Not performed	Satisfactory	NILM	NILM	Negative (NILM)	
P03081	2019	2019-Q1	2019-01	Pre-pandemic	Cytology (Pap)	60-64	62	Not performed	Satisfactory	Positive	LSIL	Low-grade / equivocal squamous	
P03082	2019	2019-Q1	2019-01	Pre-pandemic	Cytology (Pap)	40-44	44	Not performed	Satisfactory	NILM	NILM	Negative (NILM)	
P03083	2019	2019-Q1	2019-01	Pre-pandemic	Cytology (Pap)	35-39	39	Not performed	Satisfactory	NILM	NILM	Negative (NILM)	
P03084	2019	2019-Q1	2019-01	Pre-pandemic	Cytology (Pap)	55-59	56	Not performed	Satisfactory	NILM	NILM	Negative (NILM)	
P03085	2019	2019-Q1	2019-01	Pre-pandemic	Cytology (Pap)	35-39	38	Not performed	Satisfactory	NILM	NILM	Negative (NILM)	
P03086	2019	2019-Q1	2019-01	Pre-pandemic	Cytology (Pap)	35-39	38	Not performed	Satisfactory	NILM	NILM	Negative (NILM)	
P03087	2019	2019-Q1	2019-01	Pre-pandemic	Cytology (Pap)	35-39	36	Not performed	Satisfactory	NILM	NILM	Negative (NILM)	
P03088	2019	2019-Q1	2019-01	Pre-pandemic	Cytology (Pap)	55-59	59	Not performed	Satisfactory	Positive	HSIL	High-grade squamous	
P03089	2019	2019-Q1	2019-01	Pre-pandemic	Cytology (Pap)	50-54	51	Not performed	Satisfactory	NILM	NILM	Negative (NILM)	
P03090	2019	2019-Q1	2019-01	Pre-pandemic	Cytology (Pap)	55-59	57	Not performed	Satisfactory	NILM	NILM	Negative (NILM)	
P03091	2019	2019-Q1	2019-01	Pre-pandemic	Cytology (Pap)	55-59	58	Not performed	Satisfactory	Positive	LSIL	Low-grade / equivocal squamous	
P03092	2019	2019-Q1	2019-01	Pre-pandemic	Cytology (Pap)	60-64	62	Not performed	Satisfactory	NILM	NILM	Negative (NILM)	
P03093	2019	2019-Q1	2019-01	Pre-pandemic	Cytology (Pap)	45-49	45	Not performed	Satisfactory	NILM	NILM	Negative (NILM)	
P03094	2019	2019-Q1	2019-01	Pre-pandemic	Cytology (Pap)	55-59	59	Not performed	Satisfactory	NILM	NILM	Negative (NILM)	
P03095	2019	2019-Q1	2019-01	Pre-pandemic	Cytology (Pap)	45-49	45	Not performed	Satisfactory	NILM	NILM	Negative (NILM)	
P03096	2019	2019-Q1	2019-01	Pre-pandemic	Cytology (Pap)	30-34	30	Not performed	Unsatisfactory	Unsatisfactory	Unsatisfactory	Unsatisfactory	
P03097	2019	2019-Q1	2019-01	Pre-pandemic	Cytology (Pap)	40-44	43	Not performed	Satisfactory	NILM	NILM	Negative (NILM)	
P03098	2019	2019-Q1	2019-01	Pre-pandemic	Cytology (Pap)	30-34	31	Not performed	Satisfactory	NILM	NILM	Negative (NILM)	
P03099	2019	2019-Q1	2019-01	Pre-pandemic	Cytology (Pap)	35-39	39	Not performed	Satisfactory	Positive	HSIL	High-grade squamous	
P03100	2019	2019-Q1	2019-01	Pre-pandemic	Cytology (Pap)	45-49	48	Not performed	Satisfactory	NILM	NILM	Negative (NILM)	
P03101	2019	2019-Q1	2019-01	Pre-pandemic	Cytology (Pap)	25-29	27	Not performed	Satisfactory	NILM	NILM	Negative (NILM)	
P03102	2019	2019-Q1	2019-01	Pre-pandemic	Cytology (Pap)	30-34	32	Not performed	Satisfactory	NILM	NILM	Negative (NILM)	
P03103	2019	2019-Q1	2019-01	Pre-pandemic	Cytology (Pap)	55-59	55	Not performed	Satisfactory	NILM	NILM	Negative (NILM)	
P03104	2019	2019-Q1	2019-01	Pre-pandemic	Cytology (Pap)	60-64	60	Not performed	Satisfactory	NILM	NILM	Negative (NILM)	
P03105	2019	2019-Q1	2019-01	Pre-pandemic	Cytology (Pap)	40-44	42	Not performed	Satisfactory	NILM	NILM	Negative (NILM)	
P03106	2019	2019-Q1	2019-01	Pre-pandemic	Cytology (Pap)	45-49	49	Not performed	Satisfactory	NILM	NILM	Negative (NILM)	
P03107	2019	2019-Q1	2019-01	Pre-pandemic	Cytology (Pap)	25-29	25	Not performed	Satisfactory	NILM	NILM	Negative (NILM)	
P03108	2019	2019-Q1	2019-01	Pre-pandemic	Cytology (Pap)	25-29	26	Not performed	Satisfactory	NILM	NILM	Negative (NILM)	
P03109	2019	2019-Q1	2019-01	Pre-pandemic	Cytology (Pap)	25-29	27	Not performed	Satisfactory	Positive	LSIL	Low-grade / equivocal squamous	
P03110	2019	2019-Q1	2019-01	Pre-pandemic	Cytology (Pap)	30-34	31	Not performed	Satisfactory	NILM	NILM	Negative (NILM)	
P03111	2019	2019-Q1	2019-01	Pre-pandemic	Cytology (Pap)	50-54	53	Not performed	Satisfactory	NILM	NILM	Negative (NILM)	
P03112	2019	2019-Q1	2019-01	Pre-pandemic	Cytology (Pap)	40-44	43	Not performed	Satisfactory	Positive	ASC-US	Low-grade / equivocal squamous	
P03113	2019	2019-Q1	2019-01	Pre-pandemic	Cytology (Pap)	25-29	29	Not performed	Satisfactory	NILM	NILM	Negative (NILM)	
P03114	2019	2019-Q1	2019-01	Pre-pandemic	Cytology (Pap)	50-54	53	Not performed	Satisfactory	NILM	NILM	Negative (NILM)	
P03115	2019	2019-Q1	2019-01	Pre-pandemic	Cytology (Pap)	40-44	44	Not performed	Satisfactory	NILM	NILM	Negative (NILM)	
P03116	2019	2019-Q1	2019-01	Pre-pandemic	Cytology (Pap)	45-49	46	Not performed	Satisfactory	Positive	LSIL	Low-grade / equivocal squamous	
P03117	2019	2019-Q1	2019-01	Pre-pandemic	Cytology (Pap)	40-44	44	Not performed	Satisfactory	NILM	NILM	Negative (NILM)	
P03118	2019	2019-Q1	2019-01	Pre-pandemic	Cytology (Pap)	40-44	43	Not performed	Satisfactory	NILM	NILM	Negative (NILM)	

patient_id	year	quarter	month	period	primary_test	age_group	age_years	hpv_result	adequacy	result	bethesda	grade	infection
P03119	2019	2019-Q1	2019-01	Pre-pandemic	Cytology (Pap)	40-44	44	Not performed	Satisfactory	NILM	NILM	Negative (NILM)	
P03120	2019	2019-Q1	2019-01	Pre-pandemic	Cytology (Pap)	30-34	30	Not performed	Satisfactory	NILM	NILM	Negative (NILM)	
P03121	2019	2019-Q1	2019-01	Pre-pandemic	Cytology (Pap)	45-49	46	Not performed	Satisfactory	NILM	NILM	Negative (NILM)	
P03122	2019	2019-Q1	2019-01	Pre-pandemic	Cytology (Pap)	40-44	44	Not performed	Satisfactory	NILM	NILM	Negative (NILM)	
P03123	2019	2019-Q1	2019-01	Pre-pandemic	Cytology (Pap)	40-44	44	Not performed	Satisfactory	NILM	NILM	Negative (NILM)	
P03124	2019	2019-Q1	2019-01	Pre-pandemic	Cytology (Pap)	40-44	44	Not performed	Satisfactory	Positive	ASC-H	High-grade squamous	
P03125	2019	2019-Q1	2019-01	Pre-pandemic	Cytology (Pap)	55-59	59	Not performed	Satisfactory	NILM	NILM	Negative (NILM)	
P03126	2019	2019-Q1	2019-01	Pre-pandemic	Cytology (Pap)	30-34	34	Not performed	Satisfactory	NILM	NILM	Negative (NILM)	
P03127	2019	2019-Q1	2019-01	Pre-pandemic	Cytology (Pap)	35-39	38	Not performed	Satisfactory	NILM	NILM	Negative (NILM)	
P03128	2019	2019-Q1	2019-01	Pre-pandemic	Cytology (Pap)	55-59	56	Not performed	Satisfactory	NILM	NILM	Negative (NILM)	
P03129	2019	2019-Q1	2019-01	Pre-pandemic	Cytology (Pap)	30-34	32	Not performed	Unsatisfactory	Unsatisfactory	Unsatisfactory	Unsatisfactory	
P03130	2019	2019-Q1	2019-01	Pre-pandemic	Cytology (Pap)	35-39	39	Not performed	Satisfactory	NILM	NILM	Negative (NILM)	
P03131	2019	2019-Q1	2019-01	Pre-pandemic	Cytology (Pap)	40-44	40	Not performed	Satisfactory	NILM	NILM	Negative (NILM)	
P03132	2019	2019-Q1	2019-01	Pre-pandemic	Cytology (Pap)	45-49	48	Not performed	Satisfactory	NILM	NILM	Negative (NILM)	
P03133	2019	2019-Q1	2019-01	Pre-pandemic	Cytology (Pap)	30-34	33	Not performed	Satisfactory	NILM	NILM	Negative (NILM)	
P03134	2019	2019-Q1	2019-01	Pre-pandemic	Cytology (Pap)	55-59	58	Not performed	Satisfactory	NILM	NILM	Negative (NILM)	
P03135	2019	2019-Q1	2019-01	Pre-pandemic	Cytology (Pap)	35-39	35	Not performed	Satisfactory	NILM	NILM	Negative (NILM)	
P03136	2019	2019-Q1	2019-01	Pre-pandemic	Cytology (Pap)	45-49	46	Not performed	Satisfactory	NILM	NILM	Negative (NILM)	
P03137	2019	2019-Q1	2019-01	Pre-pandemic	Cytology (Pap)	40-44	42	Not performed	Satisfactory	Positive	ASC-US	Low-grade / equivocal squamous	
P03138	2019	2019-Q1	2019-01	Pre-pandemic	Cytology (Pap)	25-29	25	Not performed	Satisfactory	Positive	LSIL	Low-grade / equivocal squamous	
P03139	2019	2019-Q1	2019-01	Pre-pandemic	Cytology (Pap)	55-59	58	Not performed	Satisfactory	NILM	NILM	Negative (NILM)	
P03140	2019	2019-Q1	2019-01	Pre-pandemic	Cytology (Pap)	40-44	44	Not performed	Satisfactory	NILM	NILM	Negative (NILM)	
P03141	2019	2019-Q1	2019-01	Pre-pandemic	Cytology (Pap)	55-59	56	Not performed	Satisfactory	NILM	NILM	Negative (NILM)	
P03142	2019	2019-Q1	2019-01	Pre-pandemic	Cytology (Pap)	35-39	38	Not performed	Satisfactory	NILM	NILM	Negative (NILM)	
P03143	2019	2019-Q1	2019-01	Pre-pandemic	Cytology (Pap)	50-54	54	Not performed	Satisfactory	Positive	AGC	Glandular	
P03144	2019	2019-Q1	2019-01	Pre-pandemic	Cytology (Pap)	55-59	56	Not performed	Satisfactory	NILM	NILM	Negative (NILM)	
P03145	2019	2019-Q1	2019-01	Pre-pandemic	Cytology (Pap)	45-49	49	Not performed	Satisfactory	Positive	LSIL	Low-grade / equivocal squamous	
P03146	2019	2019-Q1	2019-01	Pre-pandemic	Cytology (Pap)	40-44	42	Not performed	Satisfactory	NILM	NILM	Negative (NILM)	
P03147	2019	2019-Q1	2019-01	Pre-pandemic	Cytology (Pap)	60-64	63	Not performed	Satisfactory	NILM	NILM	Negative (NILM)	
P03148	2019	2019-Q1	2019-01	Pre-pandemic	Cytology (Pap)	55-59	59	Not performed	Satisfactory	NILM	NILM	Negative (NILM)	
P03149	2019	2019-Q1	2019-01	Pre-pandemic	Cytology (Pap)	35-39	39	Not performed	Satisfactory	NILM	NILM	Negative (NILM)	
P03150	2019	2019-Q1	2019-01	Pre-pandemic	Cytology (Pap)	45-49	47	Not performed	Satisfactory	NILM	NILM	Negative (NILM)	
P03151	2019	2019-Q1	2019-01	Pre-pandemic	Cytology (Pap)	35-39	36	Not performed	Satisfactory	NILM	NILM	Negative (NILM)	
P03152	2019	2019-Q1	2019-01	Pre-pandemic	Cytology (Pap)	45-49	46	Not performed	Satisfactory	NILM	NILM	Negative (NILM)	
P03153	2019	2019-Q1	2019-01	Pre-pandemic	Cytology (Pap)	45-49	45	Not performed	Satisfactory	NILM	NILM	Negative (NILM)	
P03154	2019	2019-Q1	2019-01	Pre-pandemic	Cytology (Pap)	55-59	56	Not performed	Satisfactory	NILM	NILM	Negative (NILM)	
P03155	2019	2019-Q1	2019-01	Pre-pandemic	Cytology (Pap)	30-34	30	Not performed	Satisfactory	NILM	NILM	Negative (NILM)	
P03156	2019	2019-Q1	2019-01	Pre-pandemic	Cytology (Pap)	35-39	35	Not performed	Satisfactory	NILM	NILM	Negative (NILM)	
P03157	2019	2019-Q1	2019-01	Pre-pandemic	Cytology (Pap)	45-49	46	Not performed	Satisfactory	Positive	LSIL	Low-grade / equivocal squamous	
P03158	2019	2019-Q1	2019-01	Pre-pandemic	Cytology (Pap)	45-49	45	Not performed	Satisfactory	Positive	LSIL	Low-grade / equivocal squamous	
P03159	2019	2019-Q1	2019-01	Pre-pandemic	Cytology (Pap)	40-44	42	Not performed	Satisfactory	NILM	NILM	Negative (NILM)	
P03160	2019	2019-Q1	2019-01	Pre-pandemic	Cytology (Pap)	35-39	39	Not performed	Satisfactory	NILM	NILM	Negative (NILM)	
P03161	2019	2019-Q1	2019-01	Pre-pandemic	Cytology (Pap)	40-44	43	Not performed	Satisfactory	NILM	NILM	Negative (NILM)	
P03162	2019	2019-Q1	2019-01	Pre-pandemic	Cytology (Pap)	60-64	64	Not performed	Satisfactory	NILM	NILM	Negative (NILM)	
P03163	2019	2019-Q1	2019-01	Pre-pandemic	Cytology (Pap)	40-44	41	Not performed	Satisfactory	NILM	NILM	Negative (NILM)	
P03164	2019	2019-Q1	2019-01	Pre-pandemic	Cytology (Pap)	40-44	42	Not performed	Satisfactory	NILM	NILM	Negative (NILM)	
P03165	2019	2019-Q1	2019-01	Pre-pandemic	Cytology (Pap)	45-49	45	Not performed	Satisfactory	NILM	NILM	Negative (NILM)	
P03166	2019	2019-Q1	2019-01	Pre-pandemic	Cytology (Pap)	40-44	40	Not performed	Satisfactory	NILM	NILM	Negative (NILM)	
P03167	2019	2019-Q1	2019-01	Pre-pandemic	Cytology (Pap)	60-64	64	Not performed	Satisfactory	NILM	NILM	Negative (NILM)	
P03168	2019	2019-Q1	2019-01	Pre-pandemic	Cytology (Pap)	25-29	29	Not performed	Satisfactory	Positive	LSIL	Low-grade / equivocal squamous	
P03169	2019	2019-Q1	2019-01	Pre-pandemic	Cytology (Pap)	35-39	36	Not performed	Satisfactory	NILM	NILM	Negative (NILM)	Trichomonas
P03170	2019	2019-Q1	2019-01	Pre-pandemic	Cytology (Pap)	60-64	64	Not performed	Satisfactory	NILM	NILM	Negative (NILM)	
P03171	2019	2019-Q1	2019-01	Pre-pandemic	Cytology (Pap)	30-34	32	Not performed	Satisfactory	NILM	NILM	Negative (NILM)	
P03172	2019	2019-Q1	2019-01	Pre-pandemic	Cytology (Pap)	40-44	42	Not performed	Satisfactory	NILM	NILM	Negative (NILM)	
P03173	2019	2019-Q1	2019-01	Pre-pandemic	Cytology (Pap)	50-54	51	Not performed	Satisfactory	Positive	LSIL	Low-grade / equivocal squamous	
P03174	2019	2019-Q1	2019-01	Pre-pandemic	Cytology (Pap)	45-49	46	Not performed	Satisfactory	NILM	NILM	Negative (NILM)	
P03175	2019	2019-Q1	2019-01	Pre-pandemic	Cytology (Pap)	35-39	39	Not performed	Satisfactory	NILM	NILM	Negative (NILM)	
P03176	2019	2019-Q1	2019-01	Pre-pandemic	Cytology (Pap)	45-49	47	Not performed	Satisfactory	NILM	NILM	Negative (NILM)	
P03177	2019	2019-Q1	2019-01	Pre-pandemic	Cytology (Pap)	35-39	39	Not performed	Satisfactory	NILM	NILM	Negative (NILM)	
P03178	2019	2019-Q1	2019-01	Pre-pandemic	Cytology (Pap)	30-34	32	Not performed	Satisfactory	NILM	NILM	Negative (NILM)	
P03179	2019	2019-Q1	2019-01	Pre-pandemic	Cytology (Pap)	55-59	58	Not performed	Satisfactory	NILM	NILM	Negative (NILM)	
P03180	2019	2019-Q1	2019-01	Pre-pandemic	Cytology (Pap)	50-54	50	Not performed	Satisfactory	NILM	NILM	Negative (NILM)	
P03181	2019	2019-Q1	2019-01	Pre-pandemic	Cytology (Pap)	40-44	43	Not performed	Satisfactory	NILM	NILM	Negative (NILM)	
P03182	2019	2019-Q1	2019-01	Pre-pandemic	Cytology (Pap)	40-44	42	Not performed	Satisfactory	NILM	NILM	Negative (NILM)	
P03183	2019	2019-Q1	2019-01	Pre-pandemic	Cytology (Pap)	35-39	35	Not performed	Satisfactory	NILM	NILM	Negative (NILM)	
P03184	2019	2019-Q1	2019-01	Pre-pandemic	Cytology (Pap)	45-49	49	Not performed	Satisfactory	NILM	NILM	Negative (NILM)	
P03185	2019	2019-Q1	2019-01	Pre-pandemic	Cytology (Pap)	35-39	36	Not performed	Satisfactory	Positive	HSIL	High-grade squamous	
P03186	2019	2019-Q1	2019-01	Pre-pandemic	Cytology (Pap)	45-49	48	Not performed	Satisfactory	NILM	NILM	Negative (NILM)	
P03187	2019	2019-Q1	2019-01	Pre-pandemic	Cytology (Pap)	35-39	37	Not performed	Satisfactory	NILM	NILM	Negative (NILM)	
P03188	2019	2019-Q1	2019-01	Pre-pandemic	Cytology (Pap)	55-59	55	Not performed	Satisfactory	NILM	NILM	Negative (NILM)	
P03189	2019	2019-Q1	2019-01	Pre-pandemic	Cytology (Pap)	35-39	37	Not performed	Satisfactory	NILM	NILM	Negative (NILM)	
P03190	2019	2019-Q1	2019-01	Pre-pandemic	Cytology (Pap)	35-39	35	Not performed	Satisfactory	NILM	NILM	Negative (NILM)	
P03191	2019	2019-Q1	2019-01	Pre-pandemic	Cytology (Pap)	40-44	44	Not performed	Satisfactory	Positive	AGC	Glandular	
P03192	2019	2019-Q1	2019-01	Pre-pandemic	Cytology (Pap)	30-34	33	Not performed	Satisfactory	NILM	NILM	Negative (NILM)	
P03193	2019	2019-Q1	2019-01	Pre-pandemic	Cytology (Pap)	40-44	40	Not performed	Satisfactory	NILM	NILM	Negative (NILM)	
P03194	2019	2019-Q1	2019-01	Pre-pandemic	Cytology (Pap)	50-54	52	Not performed	Satisfactory	Positive	AGC	Glandular	
P03195	2019	2019-Q1	2019-01	Pre-pandemic	Cytology (Pap)	55-59	57	Not performed	Satisfactory	NILM	NILM	Negative (NILM)	
P03196	2019	2019-Q1	2019-01	Pre-pandemic	Cytology (Pap)	25-29	26	Not performed	Satisfactory	NILM	NILM	Negative (NILM)	
P03197	2019	2019-Q1	2019-01	Pre-pandemic	Cytology (Pap)	60-64	63	Not performed	Satisfactory	NILM	NILM	Negative (NILM)	
P03198	2019	2019-Q1	2019-01	Pre-pandemic	Cytology (Pap)	50-54	52	Not performed	Satisfactory	NILM	NILM	Negative (NILM)	

patient_id	year	quarter	month	period	primary_test	age_group	age_years	hpv_result	adequacy	result	bethesda	grade	infection
P03199	2019	2019-Q1	2019-01	Pre-pandemic	Cytology (Pap)	60-64	60	Not performed	Satisfactory	NILM	NILM	Negative (NILM)	
P03200	2019	2019-Q1	2019-01	Pre-pandemic	Cytology (Pap)	30-34	34	Not performed	Satisfactory	NILM	NILM	Negative (NILM)	
P03201	2019	2019-Q1	2019-01	Pre-pandemic	Cytology (Pap)	45-49	45	Not performed	Satisfactory	NILM	NILM	Negative (NILM)	
P03202	2019	2019-Q1	2019-01	Pre-pandemic	Cytology (Pap)	40-44	43	Not performed	Satisfactory	NILM	NILM	Negative (NILM)	
P03203	2019	2019-Q1	2019-01	Pre-pandemic	Cytology (Pap)	40-44	41	Not performed	Satisfactory	NILM	NILM	Negative (NILM)	
P03204	2019	2019-Q1	2019-01	Pre-pandemic	Cytology (Pap)	40-44	40	Not performed	Satisfactory	NILM	NILM	Negative (NILM)	
P03205	2019	2019-Q1	2019-01	Pre-pandemic	Cytology (Pap)	50-54	54	Not performed	Satisfactory	NILM	NILM	Negative (NILM)	
P03206	2019	2019-Q1	2019-01	Pre-pandemic	Cytology (Pap)	50-54	50	Not performed	Satisfactory	NILM	NILM	Negative (NILM)	
P03207	2019	2019-Q1	2019-01	Pre-pandemic	Cytology (Pap)	45-49	46	Not performed	Satisfactory	NILM	NILM	Negative (NILM)	
P03208	2019	2019-Q1	2019-01	Pre-pandemic	Cytology (Pap)	40-44	42	Not performed	Satisfactory	Positive	HSIL	High-grade squamous	
P03209	2019	2019-Q1	2019-01	Pre-pandemic	Cytology (Pap)	60-64	62	Not performed	Satisfactory	NILM	NILM	Negative (NILM)	
P03210	2019	2019-Q1	2019-01	Pre-pandemic	Cytology (Pap)	60-64	61	Not performed	Satisfactory	NILM	NILM	Negative (NILM)	
P03211	2019	2019-Q1	2019-01	Pre-pandemic	Cytology (Pap)	35-39	35	Not performed	Unsatisfactory	Unsatisfactory	Unsatisfactory	Unsatisfactory	
P03212	2019	2019-Q1	2019-01	Pre-pandemic	Cytology (Pap)	25-29	27	Not performed	Satisfactory	NILM	NILM	Negative (NILM)	
P03213	2019	2019-Q1	2019-01	Pre-pandemic	Cytology (Pap)	35-39	37	Not performed	Satisfactory	NILM	NILM	Negative (NILM)	
P03214	2019	2019-Q1	2019-01	Pre-pandemic	Cytology (Pap)	35-39	35	Not performed	Satisfactory	NILM	NILM	Negative (NILM)	
P03215	2019	2019-Q1	2019-01	Pre-pandemic	Cytology (Pap)	40-44	42	Not performed	Satisfactory	NILM	NILM	Negative (NILM)	
P03216	2019	2019-Q1	2019-01	Pre-pandemic	Cytology (Pap)	35-39	36	Not performed	Satisfactory	NILM	NILM	Negative (NILM)	
P03217	2019	2019-Q1	2019-01	Pre-pandemic	Cytology (Pap)	45-49	45	Not performed	Satisfactory	NILM	NILM	Negative (NILM)	
P03218	2019	2019-Q1	2019-01	Pre-pandemic	Cytology (Pap)	55-59	59	Not performed	Satisfactory	NILM	NILM	Negative (NILM)	
P03219	2019	2019-Q1	2019-01	Pre-pandemic	Cytology (Pap)	45-49	49	Not performed	Satisfactory	NILM	NILM	Negative (NILM)	
P03220	2019	2019-Q1	2019-01	Pre-pandemic	Cytology (Pap)	50-54	54	Not performed	Satisfactory	Positive	ASC-US	Low-grade / equivocal squamous	Trichomonas
P03221	2019	2019-Q1	2019-01	Pre-pandemic	Cytology (Pap)	60-64	62	Not performed	Satisfactory	Positive	HSIL	High-grade squamous	
P03222	2019	2019-Q1	2019-01	Pre-pandemic	Cytology (Pap)	30-34	31	Not performed	Satisfactory	NILM	NILM	Negative (NILM)	
P03223	2019	2019-Q1	2019-01	Pre-pandemic	Cytology (Pap)	40-44	41	Not performed	Satisfactory	Positive	ASC-US	Low-grade / equivocal squamous	
P03224	2019	2019-Q1	2019-01	Pre-pandemic	Cytology (Pap)	50-54	54	Not performed	Satisfactory	NILM	NILM	Negative (NILM)	
P03225	2019	2019-Q1	2019-01	Pre-pandemic	Cytology (Pap)	40-44	44	Not performed	Satisfactory	NILM	NILM	Negative (NILM)	
P03226	2019	2019-Q1	2019-01	Pre-pandemic	Cytology (Pap)	45-49	48	Not performed	Satisfactory	NILM	NILM	Negative (NILM)	
P03227	2019	2019-Q1	2019-01	Pre-pandemic	Cytology (Pap)	35-39	38	Not performed	Satisfactory	NILM	NILM	Negative (NILM)	
P03228	2019	2019-Q1	2019-01	Pre-pandemic	Cytology (Pap)	40-44	42	Not performed	Unsatisfactory	Unsatisfactory	Unsatisfactory	Unsatisfactory	
P03229	2019	2019-Q1	2019-01	Pre-pandemic	Cytology (Pap)	50-54	50	Not performed	Satisfactory	NILM	NILM	Negative (NILM)	
P03230	2019	2019-Q1	2019-01	Pre-pandemic	Cytology (Pap)	40-44	44	Not performed	Satisfactory	NILM	NILM	Negative (NILM)	
P03231	2019	2019-Q1	2019-01	Pre-pandemic	Cytology (Pap)	50-54	54	Not performed	Satisfactory	Positive	LSIL	Low-grade / equivocal squamous	
P03232	2019	2019-Q1	2019-01	Pre-pandemic	Cytology (Pap)	35-39	36	Not performed	Satisfactory	NILM	NILM	Negative (NILM)	
P03233	2019	2019-Q1	2019-01	Pre-pandemic	Cytology (Pap)	45-49	49	Not performed	Satisfactory	NILM	NILM	Negative (NILM)	
P03234	2019	2019-Q1	2019-01	Pre-pandemic	Cytology (Pap)	35-39	37	Not performed	Satisfactory	Positive	ASC-US	Low-grade / equivocal squamous	
P03235	2019	2019-Q1	2019-01	Pre-pandemic	Cytology (Pap)	55-59	59	Not performed	Satisfactory	NILM	NILM	Negative (NILM)	
P03236	2019	2019-Q1	2019-01	Pre-pandemic	Cytology (Pap)	30-34	31	Not performed	Satisfactory	NILM	NILM	Negative (NILM)	
P03237	2019	2019-Q1	2019-01	Pre-pandemic	Cytology (Pap)	30-34	34	Not performed	Satisfactory	NILM	NILM	Negative (NILM)	
P03238	2019	2019-Q1	2019-01	Pre-pandemic	Cytology (Pap)	40-44	43	Not performed	Satisfactory	NILM	NILM	Negative (NILM)	
P03239	2019	2019-Q1	2019-02	Pre-pandemic	Cytology (Pap)	45-49	48	Not performed	Satisfactory	NILM	NILM	Negative (NILM)	
P03240	2019	2019-Q1	2019-02	Pre-pandemic	Cytology (Pap)	30-34	34	Not performed	Satisfactory	Positive	LSIL	Low-grade / equivocal squamous	
P03241	2019	2019-Q1	2019-02	Pre-pandemic	Cytology (Pap)	55-59	57	Not performed	Satisfactory	NILM	NILM	Negative (NILM)	
P03242	2019	2019-Q1	2019-02	Pre-pandemic	Cytology (Pap)	50-54	52	Not performed	Satisfactory	Positive	AGC	Glandular	
P03243	2019	2019-Q1	2019-02	Pre-pandemic	Cytology (Pap)	45-49	48	Not performed	Satisfactory	NILM	NILM	Negative (NILM)	
P03244	2019	2019-Q1	2019-02	Pre-pandemic	Cytology (Pap)	55-59	59	Not performed	Satisfactory	NILM	NILM	Negative (NILM)	
P03245	2019	2019-Q1	2019-02	Pre-pandemic	Cytology (Pap)	40-44	41	Not performed	Unsatisfactory	Unsatisfactory	Unsatisfactory	Unsatisfactory	
P03246	2019	2019-Q1	2019-02	Pre-pandemic	Cytology (Pap)	50-54	50	Not performed	Satisfactory	NILM	NILM	Negative (NILM)	
P03247	2019	2019-Q1	2019-02	Pre-pandemic	Cytology (Pap)	40-44	44	Not performed	Satisfactory	NILM	NILM	Negative (NILM)	
P03248	2019	2019-Q1	2019-02	Pre-pandemic	Cytology (Pap)	55-59	57	Not performed	Satisfactory	NILM	NILM	Negative (NILM)	
P03249	2019	2019-Q1	2019-02	Pre-pandemic	Cytology (Pap)	35-39	37	Not performed	Satisfactory	NILM	NILM	Negative (NILM)	
P03250	2019	2019-Q1	2019-02	Pre-pandemic	Cytology (Pap)	30-34	34	Not performed	Satisfactory	NILM	NILM	Negative (NILM)	
P03251	2019	2019-Q1	2019-02	Pre-pandemic	Cytology (Pap)	35-39	35	Not performed	Satisfactory	Positive	ASC-H	High-grade squamous	
P03252	2019	2019-Q1	2019-02	Pre-pandemic	Cytology (Pap)	30-34	30	Not performed	Satisfactory	NILM	NILM	Negative (NILM)	
P03253	2019	2019-Q1	2019-02	Pre-pandemic	Cytology (Pap)	30-34	30	Not performed	Satisfactory	NILM	NILM	Negative (NILM)	Gardnerella
P03254	2019	2019-Q1	2019-02	Pre-pandemic	Cytology (Pap)	60-64	63	Not performed	Satisfactory	Positive	HSIL	High-grade squamous	
P03255	2019	2019-Q1	2019-02	Pre-pandemic	Cytology (Pap)	30-34	34	Not performed	Satisfactory	NILM	NILM	Negative (NILM)	
P03256	2019	2019-Q1	2019-02	Pre-pandemic	Cytology (Pap)	45-49	46	Not performed	Satisfactory	NILM	NILM	Negative (NILM)	
P03257	2019	2019-Q1	2019-02	Pre-pandemic	Cytology (Pap)	25-29	29	Not performed	Satisfactory	NILM	NILM	Negative (NILM)	
P03258	2019	2019-Q1	2019-02	Pre-pandemic	Cytology (Pap)	35-39	35	Not performed	Satisfactory	NILM	NILM	Negative (NILM)	
P03259	2019	2019-Q1	2019-02	Pre-pandemic	Cytology (Pap)	30-34	32	Not performed	Satisfactory	Positive	HSIL	High-grade squamous	
P03260	2019	2019-Q1	2019-02	Pre-pandemic	Cytology (Pap)	35-39	36	Not performed	Satisfactory	NILM	NILM	Negative (NILM)	
P03261	2019	2019-Q1	2019-02	Pre-pandemic	Cytology (Pap)	35-39	35	Not performed	Satisfactory	NILM	NILM	Negative (NILM)	
P03262	2019	2019-Q1	2019-02	Pre-pandemic	Cytology (Pap)	45-49	47	Not performed	Satisfactory	NILM	NILM	Negative (NILM)	
P03263	2019	2019-Q1	2019-02	Pre-pandemic	Cytology (Pap)	30-34	30	Not performed	Satisfactory	NILM	NILM	Negative (NILM)	Candida
P03264	2019	2019-Q1	2019-02	Pre-pandemic	Cytology (Pap)	55-59	58	Not performed	Satisfactory	NILM	NILM	Negative (NILM)	Trichomonas
P03265	2019	2019-Q1	2019-02	Pre-pandemic	Cytology (Pap)	30-34	34	Not performed	Satisfactory	NILM	NILM	Negative (NILM)	
P03266	2019	2019-Q1	2019-02	Pre-pandemic	Cytology (Pap)	45-49	48	Not performed	Satisfactory	NILM	NILM	Negative (NILM)	
P03267	2019	2019-Q1	2019-02	Pre-pandemic	Cytology (Pap)	45-49	45	Not performed	Satisfactory	NILM	NILM	Negative (NILM)	
P03268	2019	2019-Q1	2019-02	Pre-pandemic	Cytology (Pap)	50-54	52	Not performed	Satisfactory	NILM	NILM	Negative (NILM)	
P03269	2019	2019-Q1	2019-02	Pre-pandemic	Cytology (Pap)	30-34	34	Not performed	Satisfactory	NILM	NILM	Negative (NILM)	
P03270	2019	2019-Q1	2019-02	Pre-pandemic	Cytology (Pap)	40-44	42	Not performed	Satisfactory	Positive	HSIL	High-grade squamous	
P03271	2019	2019-Q1	2019-02	Pre-pandemic	Cytology (Pap)	50-54	53	Not performed	Satisfactory	NILM	NILM	Negative (NILM)	
P03272	2019	2019-Q1	2019-02	Pre-pandemic	Cytology (Pap)	40-44	43	Not performed	Satisfactory	NILM	NILM	Negative (NILM)	
P03273	2019	2019-Q1	2019-02	Pre-pandemic	Cytology (Pap)	35-39	39	Not performed	Unsatisfactory	Unsatisfactory	Unsatisfactory	Unsatisfactory	
P03274	2019	2019-Q1	2019-02	Pre-pandemic	Cytology (Pap)	45-49	47	Not performed	Satisfactory	NILM	NILM	Negative (NILM)	
P03275	2019	2019-Q1	2019-02	Pre-pandemic	Cytology (Pap)	40-44	42	Not performed	Satisfactory	NILM	NILM	Negative (NILM)	
P03276	2019	2019-Q1	2019-02	Pre-pandemic	Cytology (Pap)	30-34	33	Not performed	Satisfactory	NILM	NILM	Negative (NILM)	
P03277	2019	2019-Q1	2019-02	Pre-pandemic	Cytology (Pap)	55-59	56	Not performed	Satisfactory	NILM	NILM	Negative (NILM)	
P03278	2019	2019-Q1	2019-02	Pre-pandemic	Cytology (Pap)	60-64	63	Not performed	Satisfactory	NILM	NILM	Negative (NILM)	

patient_id	year	quarter	month	period	primary_test	age_group	age_years	hpv_result	adequacy	result	bethesda	grade	infection
P03279	2019	2019-Q1	2019-02	Pre-pandemic	Cytology (Pap)	45-49	45	Not performed	Satisfactory	NILM	NILM	Negative (NILM)	
P03280	2019	2019-Q1	2019-02	Pre-pandemic	Cytology (Pap)	50-54	50	Not performed	Satisfactory	NILM	NILM	Negative (NILM)	
P03281	2019	2019-Q1	2019-02	Pre-pandemic	Cytology (Pap)	40-44	44	Not performed	Satisfactory	NILM	NILM	Negative (NILM)	
P03282	2019	2019-Q1	2019-02	Pre-pandemic	Cytology (Pap)	55-59	55	Not performed	Satisfactory	NILM	NILM	Negative (NILM)	
P03283	2019	2019-Q1	2019-02	Pre-pandemic	Cytology (Pap)	35-39	35	Not performed	Satisfactory	NILM	NILM	Negative (NILM)	
P03284	2019	2019-Q1	2019-02	Pre-pandemic	Cytology (Pap)	40-44	43	Not performed	Satisfactory	NILM	NILM	Negative (NILM)	
P03285	2019	2019-Q1	2019-02	Pre-pandemic	Cytology (Pap)	55-59	58	Not performed	Satisfactory	NILM	NILM	Negative (NILM)	
P03286	2019	2019-Q1	2019-02	Pre-pandemic	Cytology (Pap)	40-44	41	Not performed	Satisfactory	NILM	NILM	Negative (NILM)	
P03287	2019	2019-Q1	2019-02	Pre-pandemic	Cytology (Pap)	45-49	47	Not performed	Satisfactory	NILM	NILM	Negative (NILM)	Candida
P03288	2019	2019-Q1	2019-02	Pre-pandemic	Cytology (Pap)	35-39	39	Not performed	Satisfactory	Positive	HSIL	High-grade squamous	
P03289	2019	2019-Q1	2019-02	Pre-pandemic	Cytology (Pap)	50-54	52	Not performed	Satisfactory	NILM	NILM	Negative (NILM)	
P03290	2019	2019-Q1	2019-02	Pre-pandemic	Cytology (Pap)	45-49	45	Not performed	Satisfactory	NILM	NILM	Negative (NILM)	
P03291	2019	2019-Q1	2019-02	Pre-pandemic	Cytology (Pap)	55-59	55	Not performed	Satisfactory	NILM	NILM	Negative (NILM)	
P03292	2019	2019-Q1	2019-02	Pre-pandemic	Cytology (Pap)	35-39	36	Not performed	Satisfactory	NILM	NILM	Negative (NILM)	
P03293	2019	2019-Q1	2019-02	Pre-pandemic	Cytology (Pap)	40-44	41	Not performed	Satisfactory	NILM	NILM	Negative (NILM)	
P03294	2019	2019-Q1	2019-02	Pre-pandemic	Cytology (Pap)	30-34	31	Not performed	Satisfactory	NILM	NILM	Negative (NILM)	
P03295	2019	2019-Q1	2019-02	Pre-pandemic	Cytology (Pap)	45-49	45	Not performed	Satisfactory	NILM	NILM	Negative (NILM)	
P03296	2019	2019-Q1	2019-02	Pre-pandemic	Cytology (Pap)	40-44	44	Not performed	Satisfactory	NILM	NILM	Negative (NILM)	
P03297	2019	2019-Q1	2019-02	Pre-pandemic	Cytology (Pap)	40-44	44	Not performed	Satisfactory	NILM	NILM	Negative (NILM)	
P03298	2019	2019-Q1	2019-02	Pre-pandemic	Cytology (Pap)	45-49	47	Not performed	Satisfactory	NILM	NILM	Negative (NILM)	Trichomonas
P03299	2019	2019-Q1	2019-02	Pre-pandemic	Cytology (Pap)	40-44	43	Not performed	Satisfactory	NILM	NILM	Negative (NILM)	
P03300	2019	2019-Q1	2019-02	Pre-pandemic	Cytology (Pap)	40-44	42	Not performed	Satisfactory	NILM	NILM	Negative (NILM)	
P03301	2019	2019-Q1	2019-02	Pre-pandemic	Cytology (Pap)	60-64	63	Not performed	Satisfactory	NILM	NILM	Negative (NILM)	
P03302	2019	2019-Q1	2019-02	Pre-pandemic	Cytology (Pap)	55-59	56	Not performed	Satisfactory	NILM	NILM	Negative (NILM)	
P03303	2019	2019-Q1	2019-02	Pre-pandemic	Cytology (Pap)	25-29	27	Not performed	Satisfactory	NILM	NILM	Negative (NILM)	
P03304	2019	2019-Q1	2019-02	Pre-pandemic	Cytology (Pap)	35-39	39	Not performed	Satisfactory	Positive	ASC-US	Low-grade / equivocal squamous	
P03305	2019	2019-Q1	2019-02	Pre-pandemic	Cytology (Pap)	35-39	38	Not performed	Satisfactory	NILM	NILM	Negative (NILM)	
P03306	2019	2019-Q1	2019-02	Pre-pandemic	Cytology (Pap)	50-54	52	Not performed	Satisfactory	NILM	NILM	Negative (NILM)	
P03307	2019	2019-Q1	2019-02	Pre-pandemic	Cytology (Pap)	25-29	27	Not performed	Unsatisfactory	Unsatisfactory	Unsatisfactory	Unsatisfactory	
P03308	2019	2019-Q1	2019-02	Pre-pandemic	Cytology (Pap)	30-34	33	Not performed	Satisfactory	NILM	NILM	Negative (NILM)	
P03309	2019	2019-Q1	2019-02	Pre-pandemic	Cytology (Pap)	40-44	41	Not performed	Satisfactory	NILM	NILM	Negative (NILM)	
P03310	2019	2019-Q1	2019-02	Pre-pandemic	Cytology (Pap)	40-44	44	Not performed	Satisfactory	NILM	NILM	Negative (NILM)	
P03311	2019	2019-Q1	2019-02	Pre-pandemic	Cytology (Pap)	45-49	48	Not performed	Satisfactory	NILM	NILM	Negative (NILM)	
P03312	2019	2019-Q1	2019-02	Pre-pandemic	Cytology (Pap)	55-59	56	Not performed	Satisfactory	NILM	NILM	Negative (NILM)	
P03313	2019	2019-Q1	2019-02	Pre-pandemic	Cytology (Pap)	45-49	49	Not performed	Satisfactory	Positive	ASC-H	High-grade squamous	
P03314	2019	2019-Q1	2019-02	Pre-pandemic	Cytology (Pap)	30-34	33	Not performed	Satisfactory	NILM	NILM	Negative (NILM)	
P03315	2019	2019-Q1	2019-02	Pre-pandemic	Cytology (Pap)	40-44	40	Not performed	Satisfactory	NILM	NILM	Negative (NILM)	
P03316	2019	2019-Q1	2019-02	Pre-pandemic	Cytology (Pap)	55-59	56	Not performed	Satisfactory	NILM	NILM	Negative (NILM)	
P03317	2019	2019-Q1	2019-02	Pre-pandemic	Cytology (Pap)	35-39	38	Not performed	Satisfactory	NILM	NILM	Negative (NILM)	
P03318	2019	2019-Q1	2019-02	Pre-pandemic	Cytology (Pap)	45-49	48	Not performed	Satisfactory	NILM	NILM	Negative (NILM)	
P03319	2019	2019-Q1	2019-02	Pre-pandemic	Cytology (Pap)	40-44	42	Not performed	Satisfactory	NILM	NILM	Negative (NILM)	
P03320	2019	2019-Q1	2019-02	Pre-pandemic	Cytology (Pap)	35-39	35	Not performed	Satisfactory	Positive	LSIL	Low-grade / equivocal squamous	
P03321	2019	2019-Q1	2019-02	Pre-pandemic	Cytology (Pap)	30-34	32	Not performed	Satisfactory	NILM	NILM	Negative (NILM)	
P03322	2019	2019-Q1	2019-02	Pre-pandemic	Cytology (Pap)	25-29	26	Not performed	Satisfactory	NILM	NILM	Negative (NILM)	
P03323	2019	2019-Q1	2019-02	Pre-pandemic	Cytology (Pap)	60-64	62	Not performed	Satisfactory	NILM	NILM	Negative (NILM)	
P03324	2019	2019-Q1	2019-02	Pre-pandemic	Cytology (Pap)	35-39	38	Not performed	Satisfactory	NILM	NILM	Negative (NILM)	
P03325	2019	2019-Q1	2019-02	Pre-pandemic	Cytology (Pap)	35-39	36	Not performed	Satisfactory	NILM	NILM	Negative (NILM)	
P03326	2019	2019-Q1	2019-02	Pre-pandemic	Cytology (Pap)	50-54	54	Not performed	Satisfactory	NILM	NILM	Negative (NILM)	
P03327	2019	2019-Q1	2019-02	Pre-pandemic	Cytology (Pap)	25-29	28	Not performed	Satisfactory	NILM	NILM	Negative (NILM)	
P03328	2019	2019-Q1	2019-02	Pre-pandemic	Cytology (Pap)	35-39	35	Not performed	Satisfactory	NILM	NILM	Negative (NILM)	
P03329	2019	2019-Q1	2019-02	Pre-pandemic	Cytology (Pap)	40-44	41	Not performed	Satisfactory	NILM	NILM	Negative (NILM)	
P03330	2019	2019-Q1	2019-02	Pre-pandemic	Cytology (Pap)	30-34	33	Not performed	Satisfactory	NILM	NILM	Negative (NILM)	
P03331	2019	2019-Q1	2019-02	Pre-pandemic	Cytology (Pap)	55-59	55	Not performed	Satisfactory	NILM	NILM	Negative (NILM)	
P03332	2019	2019-Q1	2019-02	Pre-pandemic	Cytology (Pap)	35-39	35	Not performed	Satisfactory	NILM	NILM	Negative (NILM)	
P03333	2019	2019-Q1	2019-02	Pre-pandemic	Cytology (Pap)	35-39	35	Not performed	Satisfactory	NILM	NILM	Negative (NILM)	
P03334	2019	2019-Q1	2019-02	Pre-pandemic	Cytology (Pap)	45-49	46	Not performed	Satisfactory	NILM	NILM	Negative (NILM)	
P03335	2019	2019-Q1	2019-02	Pre-pandemic	Cytology (Pap)	40-44	41	Not performed	Satisfactory	NILM	NILM	Negative (NILM)	
P03336	2019	2019-Q1	2019-02	Pre-pandemic	Cytology (Pap)	25-29	28	Not performed	Satisfactory	NILM	NILM	Negative (NILM)	
P03337	2019	2019-Q1	2019-02	Pre-pandemic	Cytology (Pap)	40-44	43	Not performed	Satisfactory	NILM	NILM	Negative (NILM)	
P03338	2019	2019-Q1	2019-02	Pre-pandemic	Cytology (Pap)	30-34	34	Not performed	Satisfactory	NILM	NILM	Negative (NILM)	
P03339	2019	2019-Q1	2019-02	Pre-pandemic	Cytology (Pap)	40-44	43	Not performed	Satisfactory	NILM	NILM	Negative (NILM)	
P03340	2019	2019-Q1	2019-02	Pre-pandemic	Cytology (Pap)	55-59	58	Not performed	Satisfactory	NILM	NILM	Negative (NILM)	
P03341	2019	2019-Q1	2019-02	Pre-pandemic	Cytology (Pap)	55-59	57	Not performed	Satisfactory	NILM	NILM	Negative (NILM)	
P03342	2019	2019-Q1	2019-02	Pre-pandemic	Cytology (Pap)	35-39	37	Not performed	Satisfactory	NILM	NILM	Negative (NILM)	
P03343	2019	2019-Q1	2019-02	Pre-pandemic	Cytology (Pap)	40-44	43	Not performed	Satisfactory	NILM	NILM	Negative (NILM)	
P03344	2019	2019-Q1	2019-02	Pre-pandemic	Cytology (Pap)	45-49	45	Not performed	Satisfactory	NILM	NILM	Negative (NILM)	Trichomonas
P03345	2019	2019-Q1	2019-02	Pre-pandemic	Cytology (Pap)	30-34	33	Not performed	Satisfactory	NILM	NILM	Negative (NILM)	
P03346	2019	2019-Q1	2019-02	Pre-pandemic	Cytology (Pap)	55-59	55	Not performed	Satisfactory	NILM	NILM	Negative (NILM)	
P03347	2019	2019-Q1	2019-02	Pre-pandemic	Cytology (Pap)	55-59	58	Not performed	Satisfactory	NILM	NILM	Negative (NILM)	
P03348	2019	2019-Q1	2019-02	Pre-pandemic	Cytology (Pap)	35-39	38	Not performed	Satisfactory	Positive	LSIL	Low-grade / equivocal squamous	
P03349	2019	2019-Q1	2019-02	Pre-pandemic	Cytology (Pap)	55-59	55	Not performed	Satisfactory	NILM	NILM	Negative (NILM)	
P03350	2019	2019-Q1	2019-02	Pre-pandemic	Cytology (Pap)	40-44	41	Not performed	Satisfactory	Positive	ASC-H	High-grade squamous	
P03351	2019	2019-Q1	2019-02	Pre-pandemic	Cytology (Pap)	35-39	39	Not performed	Satisfactory	Positive	ASC-US	Low-grade / equivocal squamous	
P03352	2019	2019-Q1	2019-02	Pre-pandemic	Cytology (Pap)	25-29	27	Not performed	Satisfactory	NILM	NILM	Negative (NILM)	Candida
P03353	2019	2019-Q1	2019-02	Pre-pandemic	Cytology (Pap)	30-34	34	Not performed	Satisfactory	NILM	NILM	Negative (NILM)	
P03354	2019	2019-Q1	2019-02	Pre-pandemic	Cytology (Pap)	45-49	45	Not performed	Satisfactory	Positive	HSIL	High-grade squamous	
P03355	2019	2019-Q1	2019-02	Pre-pandemic	Cytology (Pap)	50-54	52	Not performed	Satisfactory	NILM	NILM	Negative (NILM)	
P03356	2019	2019-Q1	2019-02	Pre-pandemic	Cytology (Pap)	35-39	39	Not performed	Satisfactory	Positive	ASC-H	High-grade squamous	
P03357	2019	2019-Q1	2019-02	Pre-pandemic	Cytology (Pap)	40-44	41	Not performed	Satisfactory	NILM	NILM	Negative (NILM)	
P03358	2019	2019-Q1	2019-02	Pre-pandemic	Cytology (Pap)	60-64	60	Not performed	Satisfactory	Positive	HSIL	High-grade squamous	

patient_id	year	quarter	month	period	primary_test	age_group	age_years	hpv_result	adequacy	result	bethesda	grade	infection
P03359	2019	2019-Q1	2019-02	Pre-pandemic	Cytology (Pap)	50-54	51	Not performed	Satisfactory	NILM	NILM	Negative (NILM)	
P03360	2019	2019-Q1	2019-02	Pre-pandemic	Cytology (Pap)	40-44	43	Not performed	Satisfactory	NILM	NILM	Negative (NILM)	
P03361	2019	2019-Q1	2019-02	Pre-pandemic	Cytology (Pap)	60-64	63	Not performed	Satisfactory	Positive	ASC-H	High-grade squamous	
P03362	2019	2019-Q1	2019-02	Pre-pandemic	Cytology (Pap)	45-49	46	Not performed	Satisfactory	NILM	NILM	Negative (NILM)	
P03363	2019	2019-Q1	2019-02	Pre-pandemic	Cytology (Pap)	45-49	46	Not performed	Satisfactory	NILM	NILM	Negative (NILM)	
P03364	2019	2019-Q1	2019-02	Pre-pandemic	Cytology (Pap)	45-49	49	Not performed	Satisfactory	NILM	NILM	Negative (NILM)	
P03365	2019	2019-Q1	2019-02	Pre-pandemic	Cytology (Pap)	35-39	37	Not performed	Unsatisfactory	Unsatisfactory	Unsatisfactory	Unsatisfactory	
P03366	2019	2019-Q1	2019-02	Pre-pandemic	Cytology (Pap)	40-44	41	Not performed	Satisfactory	NILM	NILM	Negative (NILM)	
P03367	2019	2019-Q1	2019-02	Pre-pandemic	Cytology (Pap)	50-54	50	Not performed	Satisfactory	NILM	NILM	Negative (NILM)	
P03368	2019	2019-Q1	2019-02	Pre-pandemic	Cytology (Pap)	50-54	52	Not performed	Satisfactory	NILM	NILM	Negative (NILM)	
P03369	2019	2019-Q1	2019-02	Pre-pandemic	Cytology (Pap)	45-49	46	Not performed	Satisfactory	NILM	NILM	Negative (NILM)	
P03370	2019	2019-Q1	2019-02	Pre-pandemic	Cytology (Pap)	60-64	60	Not performed	Satisfactory	NILM	NILM	Negative (NILM)	
P03371	2019	2019-Q1	2019-02	Pre-pandemic	Cytology (Pap)	35-39	35	Not performed	Satisfactory	NILM	NILM	Negative (NILM)	
P03372	2019	2019-Q1	2019-02	Pre-pandemic	Cytology (Pap)	35-39	36	Not performed	Satisfactory	NILM	NILM	Negative (NILM)	
P03373	2019	2019-Q1	2019-02	Pre-pandemic	Cytology (Pap)	40-44	43	Not performed	Satisfactory	NILM	NILM	Negative (NILM)	
P03374	2019	2019-Q1	2019-02	Pre-pandemic	Cytology (Pap)	30-34	32	Not performed	Satisfactory	NILM	NILM	Negative (NILM)	
P03375	2019	2019-Q1	2019-02	Pre-pandemic	Cytology (Pap)	35-39	37	Not performed	Satisfactory	NILM	NILM	Negative (NILM)	
P03376	2019	2019-Q1	2019-02	Pre-pandemic	Cytology (Pap)	35-39	39	Not performed	Satisfactory	NILM	NILM	Negative (NILM)	
P03377	2019	2019-Q1	2019-02	Pre-pandemic	Cytology (Pap)	45-49	49	Not performed	Satisfactory	NILM	NILM	Negative (NILM)	
P03378	2019	2019-Q1	2019-02	Pre-pandemic	Cytology (Pap)	40-44	40	Not performed	Satisfactory	NILM	NILM	Negative (NILM)	Candida
P03379	2019	2019-Q1	2019-02	Pre-pandemic	Cytology (Pap)	50-54	52	Not performed	Satisfactory	Positive	ASC-H	High-grade squamous	
P03380	2019	2019-Q1	2019-02	Pre-pandemic	Cytology (Pap)	60-64	60	Not performed	Satisfactory	NILM	NILM	Negative (NILM)	
P03381	2019	2019-Q1	2019-02	Pre-pandemic	Cytology (Pap)	45-49	48	Not performed	Satisfactory	NILM	NILM	Negative (NILM)	
P03382	2019	2019-Q1	2019-02	Pre-pandemic	Cytology (Pap)	40-44	44	Not performed	Satisfactory	NILM	NILM	Negative (NILM)	
P03383	2019	2019-Q1	2019-02	Pre-pandemic	Cytology (Pap)	50-54	50	Not performed	Satisfactory	NILM	NILM	Negative (NILM)	
P03384	2019	2019-Q1	2019-02	Pre-pandemic	Cytology (Pap)	50-54	50	Not performed	Satisfactory	NILM	NILM	Negative (NILM)	
P03385	2019	2019-Q1	2019-02	Pre-pandemic	Cytology (Pap)	40-44	40	Not performed	Satisfactory	NILM	NILM	Negative (NILM)	Candida
P03386	2019	2019-Q1	2019-02	Pre-pandemic	Cytology (Pap)	25-29	26	Not performed	Satisfactory	NILM	NILM	Negative (NILM)	
P03387	2019	2019-Q1	2019-02	Pre-pandemic	Cytology (Pap)	30-34	30	Not performed	Satisfactory	NILM	NILM	Negative (NILM)	
P03388	2019	2019-Q1	2019-02	Pre-pandemic	Cytology (Pap)	50-54	52	Not performed	Satisfactory	Positive	ASC-US	Low-grade / equivocal squamous	
P03389	2019	2019-Q1	2019-02	Pre-pandemic	Cytology (Pap)	40-44	43	Not performed	Unsatisfactory	Unsatisfactory	Unsatisfactory	Unsatisfactory	
P03390	2019	2019-Q1	2019-02	Pre-pandemic	Cytology (Pap)	45-49	49	Not performed	Satisfactory	Positive	LSIL	Low-grade / equivocal squamous	
P03391	2019	2019-Q1	2019-02	Pre-pandemic	Cytology (Pap)	40-44	41	Not performed	Satisfactory	Positive	ASC-US	Low-grade / equivocal squamous	
P03392	2019	2019-Q1	2019-02	Pre-pandemic	Cytology (Pap)	40-44	41	Not performed	Satisfactory	Positive	LSIL	Low-grade / equivocal squamous	
P03393	2019	2019-Q1	2019-02	Pre-pandemic	Cytology (Pap)	60-64	60	Not performed	Satisfactory	NILM	NILM	Negative (NILM)	
P03394	2019	2019-Q1	2019-02	Pre-pandemic	Cytology (Pap)	60-64	62	Not performed	Satisfactory	NILM	NILM	Negative (NILM)	
P03395	2019	2019-Q1	2019-02	Pre-pandemic	Cytology (Pap)	30-34	30	Not performed	Satisfactory	NILM	NILM	Negative (NILM)	
P03396	2019	2019-Q1	2019-02	Pre-pandemic	Cytology (Pap)	45-49	48	Not performed	Satisfactory	NILM	NILM	Negative (NILM)	
P03397	2019	2019-Q1	2019-02	Pre-pandemic	Cytology (Pap)	60-64	64	Not performed	Satisfactory	NILM	NILM	Negative (NILM)	
P03398	2019	2019-Q1	2019-02	Pre-pandemic	Cytology (Pap)	30-34	33	Not performed	Satisfactory	NILM	NILM	Negative (NILM)	
P03399	2019	2019-Q1	2019-02	Pre-pandemic	Cytology (Pap)	45-49	45	Not performed	Satisfactory	NILM	NILM	Negative (NILM)	
P03400	2019	2019-Q1	2019-02	Pre-pandemic	Cytology (Pap)	40-44	40	Not performed	Satisfactory	NILM	NILM	Negative (NILM)	
P03401	2019	2019-Q1	2019-02	Pre-pandemic	Cytology (Pap)	55-59	59	Not performed	Satisfactory	NILM	NILM	Negative (NILM)	
P03402	2019	2019-Q1	2019-02	Pre-pandemic	Cytology (Pap)	30-34	30	Not performed	Satisfactory	NILM	NILM	Negative (NILM)	
P03403	2019	2019-Q1	2019-02	Pre-pandemic	Cytology (Pap)	35-39	37	Not performed	Satisfactory	NILM	NILM	Negative (NILM)	
P03404	2019	2019-Q1	2019-02	Pre-pandemic	Cytology (Pap)	35-39	36	Not performed	Satisfactory	NILM	NILM	Negative (NILM)	
P03405	2019	2019-Q1	2019-02	Pre-pandemic	Cytology (Pap)	40-44	42	Not performed	Satisfactory	NILM	NILM	Negative (NILM)	
P03406	2019	2019-Q1	2019-02	Pre-pandemic	Cytology (Pap)	35-39	37	Not performed	Satisfactory	NILM	NILM	Negative (NILM)	
P03407	2019	2019-Q1	2019-02	Pre-pandemic	Cytology (Pap)	40-44	44	Not performed	Satisfactory	NILM	NILM	Negative (NILM)	
P03408	2019	2019-Q1	2019-02	Pre-pandemic	Cytology (Pap)	30-34	30	Not performed	Satisfactory	NILM	NILM	Negative (NILM)	
P03409	2019	2019-Q1	2019-02	Pre-pandemic	Cytology (Pap)	40-44	44	Not performed	Satisfactory	NILM	NILM	Negative (NILM)	
P03410	2019	2019-Q1	2019-02	Pre-pandemic	Cytology (Pap)	50-54	54	Not performed	Satisfactory	NILM	NILM	Negative (NILM)	
P03411	2019	2019-Q1	2019-02	Pre-pandemic	Cytology (Pap)	55-59	59	Not performed	Satisfactory	NILM	NILM	Negative (NILM)	
P03412	2019	2019-Q1	2019-02	Pre-pandemic	Cytology (Pap)	40-44	44	Not performed	Satisfactory	NILM	NILM	Negative (NILM)	
P03413	2019	2019-Q1	2019-02	Pre-pandemic	Cytology (Pap)	50-54	50	Not performed	Satisfactory	NILM	NILM	Negative (NILM)	
P03414	2019	2019-Q1	2019-02	Pre-pandemic	Cytology (Pap)	30-34	32	Not performed	Satisfactory	NILM	NILM	Negative (NILM)	
P03415	2019	2019-Q1	2019-02	Pre-pandemic	Cytology (Pap)	50-54	52	Not performed	Satisfactory	NILM	NILM	Negative (NILM)	
P03416	2019	2019-Q1	2019-02	Pre-pandemic	Cytology (Pap)	45-49	48	Not performed	Satisfactory	NILM	NILM	Negative (NILM)	
P03417	2019	2019-Q1	2019-02	Pre-pandemic	Cytology (Pap)	45-49	45	Not performed	Satisfactory	NILM	NILM	Negative (NILM)	
P03418	2019	2019-Q1	2019-02	Pre-pandemic	Cytology (Pap)	35-39	37	Not performed	Satisfactory	NILM	NILM	Negative (NILM)	Trichomonas
P03419	2019	2019-Q1	2019-02	Pre-pandemic	Cytology (Pap)	40-44	40	Not performed	Satisfactory	NILM	NILM	Negative (NILM)	
P03420	2019	2019-Q1	2019-02	Pre-pandemic	Cytology (Pap)	45-49	48	Not performed	Satisfactory	NILM	NILM	Negative (NILM)	
P03421	2019	2019-Q1	2019-02	Pre-pandemic	Cytology (Pap)	45-49	48	Not performed	Satisfactory	NILM	NILM	Negative (NILM)	
P03422	2019	2019-Q1	2019-02	Pre-pandemic	Cytology (Pap)	35-39	38	Not performed	Satisfactory	NILM	NILM	Negative (NILM)	
P03423	2019	2019-Q1	2019-02	Pre-pandemic	Cytology (Pap)	40-44	42	Not performed	Satisfactory	NILM	NILM	Negative (NILM)	
P03424	2019	2019-Q1	2019-02	Pre-pandemic	Cytology (Pap)	55-59	57	Not performed	Satisfactory	NILM	NILM	Negative (NILM)	
P03425	2019	2019-Q1	2019-02	Pre-pandemic	Cytology (Pap)	45-49	49	Not performed	Satisfactory	NILM	NILM	Negative (NILM)	
P03426	2019	2019-Q1	2019-02	Pre-pandemic	Cytology (Pap)	30-34	32	Not performed	Satisfactory	NILM	NILM	Negative (NILM)	
P03427	2019	2019-Q1	2019-02	Pre-pandemic	Cytology (Pap)	50-54	50	Not performed	Satisfactory	NILM	NILM	Negative (NILM)	
P03428	2019	2019-Q1	2019-02	Pre-pandemic	Cytology (Pap)	55-59	56	Not performed	Satisfactory	NILM	NILM	Negative (NILM)	
P03429	2019	2019-Q1	2019-02	Pre-pandemic	Cytology (Pap)	35-39	39	Not performed	Satisfactory	NILM	NILM	Negative (NILM)	
P03430	2019	2019-Q1	2019-02	Pre-pandemic	Cytology (Pap)	45-49	49	Not performed	Satisfactory	Positive	AGC	Glandular	
P03431	2019	2019-Q1	2019-02	Pre-pandemic	Cytology (Pap)	60-64	62	Not performed	Satisfactory	Positive	ASC-US	Low-grade / equivocal squamous	
P03432	2019	2019-Q1	2019-02	Pre-pandemic	Cytology (Pap)	30-34	34	Not performed	Satisfactory	NILM	NILM	Negative (NILM)	
P03433	2019	2019-Q1	2019-02	Pre-pandemic	Cytology (Pap)	45-49	48	Not performed	Satisfactory	NILM	NILM	Negative (NILM)	
P03434	2019	2019-Q1	2019-02	Pre-pandemic	Cytology (Pap)	45-49	47	Not performed	Satisfactory	NILM	NILM	Negative (NILM)	
P03435	2019	2019-Q1	2019-02	Pre-pandemic	Cytology (Pap)	60-64	62	Not performed	Satisfactory	Positive	ASC-US	Low-grade / equivocal squamous	
P03436	2019	2019-Q1	2019-02	Pre-pandemic	Cytology (Pap)	45-49	48	Not performed	Satisfactory	NILM	NILM	Negative (NILM)	
P03437	2019	2019-Q1	2019-02	Pre-pandemic	Cytology (Pap)	60-64	60	Not performed	Satisfactory	NILM	NILM	Negative (NILM)	
P03438	2019	2019-Q1	2019-02	Pre-pandemic	Cytology (Pap)	25-29	25	Not performed	Satisfactory	NILM	NILM	Negative (NILM)	

patient_id	year	quarter	month	period	primary_test	age_group	age_years	hpv_result	adequacy	result	bethesda	grade	infection
P03439	2019	2019-Q1	2019-02	Pre-pandemic	Cytology (Pap)	35-39	38	Not performed	Satisfactory	NILM	NILM	Negative (NILM)	
P03440	2019	2019-Q1	2019-02	Pre-pandemic	Cytology (Pap)	25-29	29	Not performed	Satisfactory	NILM	NILM	Negative (NILM)	
P03441	2019	2019-Q1	2019-02	Pre-pandemic	Cytology (Pap)	40-44	41	Not performed	Satisfactory	NILM	NILM	Negative (NILM)	
P03442	2019	2019-Q1	2019-02	Pre-pandemic	Cytology (Pap)	35-39	36	Not performed	Satisfactory	NILM	NILM	Negative (NILM)	
P03443	2019	2019-Q1	2019-02	Pre-pandemic	Cytology (Pap)	30-34	33	Not performed	Satisfactory	NILM	NILM	Negative (NILM)	
P03444	2019	2019-Q1	2019-02	Pre-pandemic	Cytology (Pap)	55-59	56	Not performed	Satisfactory	NILM	NILM	Negative (NILM)	Trichomonas
P03445	2019	2019-Q1	2019-02	Pre-pandemic	Cytology (Pap)	55-59	59	Not performed	Satisfactory	NILM	NILM	Negative (NILM)	
P03446	2019	2019-Q1	2019-02	Pre-pandemic	Cytology (Pap)	35-39	37	Not performed	Satisfactory	NILM	NILM	Negative (NILM)	Candida
P03447	2019	2019-Q1	2019-02	Pre-pandemic	Cytology (Pap)	45-49	49	Not performed	Satisfactory	NILM	NILM	Negative (NILM)	
P03448	2019	2019-Q1	2019-02	Pre-pandemic	Cytology (Pap)	50-54	50	Not performed	Satisfactory	NILM	NILM	Negative (NILM)	
P03449	2019	2019-Q1	2019-02	Pre-pandemic	Cytology (Pap)	50-54	51	Not performed	Satisfactory	NILM	NILM	Negative (NILM)	
P03450	2019	2019-Q1	2019-02	Pre-pandemic	Cytology (Pap)	30-34	34	Not performed	Satisfactory	NILM	NILM	Negative (NILM)	
P03451	2019	2019-Q1	2019-02	Pre-pandemic	Cytology (Pap)	55-59	56	Not performed	Satisfactory	NILM	NILM	Negative (NILM)	Gardnerella
P03452	2019	2019-Q1	2019-02	Pre-pandemic	Cytology (Pap)	40-44	40	Not performed	Satisfactory	NILM	NILM	Negative (NILM)	
P03453	2019	2019-Q1	2019-02	Pre-pandemic	Cytology (Pap)	35-39	37	Not performed	Satisfactory	NILM	NILM	Negative (NILM)	
P03454	2019	2019-Q1	2019-02	Pre-pandemic	Cytology (Pap)	30-34	33	Not performed	Satisfactory	NILM	NILM	Negative (NILM)	Candida
P03455	2019	2019-Q1	2019-02	Pre-pandemic	Cytology (Pap)	35-39	38	Not performed	Unsatisfactory	Unsatisfactory	Unsatisfactory	Unsatisfactory	
P03456	2019	2019-Q1	2019-02	Pre-pandemic	Cytology (Pap)	55-59	59	Not performed	Satisfactory	NILM	NILM	Negative (NILM)	
P03457	2019	2019-Q1	2019-02	Pre-pandemic	Cytology (Pap)	55-59	58	Not performed	Satisfactory	NILM	NILM	Negative (NILM)	
P03458	2019	2019-Q1	2019-02	Pre-pandemic	Cytology (Pap)	25-29	27	Not performed	Satisfactory	Positive	ASC-US	Low-grade / equivocal squamous	
P03459	2019	2019-Q1	2019-02	Pre-pandemic	Cytology (Pap)	50-54	54	Not performed	Satisfactory	NILM	NILM	Negative (NILM)	
P03460	2019	2019-Q1	2019-02	Pre-pandemic	Cytology (Pap)	40-44	40	Not performed	Satisfactory	NILM	NILM	Negative (NILM)	
P03461	2019	2019-Q1	2019-02	Pre-pandemic	Cytology (Pap)	30-34	34	Not performed	Satisfactory	NILM	NILM	Negative (NILM)	
P03462	2019	2019-Q1	2019-02	Pre-pandemic	Cytology (Pap)	30-34	33	Not performed	Satisfactory	Positive	LSIL	Low-grade / equivocal squamous	
P03463	2019	2019-Q1	2019-02	Pre-pandemic	Cytology (Pap)	45-49	46	Not performed	Satisfactory	Positive	ASC-US	Low-grade / equivocal squamous	
P03464	2019	2019-Q1	2019-02	Pre-pandemic	Cytology (Pap)	55-59	58	Not performed	Satisfactory	NILM	NILM	Negative (NILM)	
P03465	2019	2019-Q1	2019-02	Pre-pandemic	Cytology (Pap)	45-49	46	Not performed	Satisfactory	Positive	LSIL	Low-grade / equivocal squamous	
P03466	2019	2019-Q1	2019-02	Pre-pandemic	Cytology (Pap)	40-44	43	Not performed	Satisfactory	NILM	NILM	Negative (NILM)	
P03467	2019	2019-Q1	2019-02	Pre-pandemic	Cytology (Pap)	60-64	61	Not performed	Satisfactory	NILM	NILM	Negative (NILM)	
P03468	2019	2019-Q1	2019-02	Pre-pandemic	Cytology (Pap)	30-34	34	Not performed	Satisfactory	NILM	NILM	Negative (NILM)	
P03469	2019	2019-Q1	2019-02	Pre-pandemic	Cytology (Pap)	35-39	36	Not performed	Satisfactory	NILM	NILM	Negative (NILM)	
P03470	2019	2019-Q1	2019-02	Pre-pandemic	Cytology (Pap)	60-64	61	Not performed	Satisfactory	NILM	NILM	Negative (NILM)	
P03471	2019	2019-Q1	2019-02	Pre-pandemic	Cytology (Pap)	45-49	48	Not performed	Satisfactory	Positive	HSIL	High-grade squamous	
P03472	2019	2019-Q1	2019-02	Pre-pandemic	Cytology (Pap)	45-49	45	Not performed	Satisfactory	NILM	NILM	Negative (NILM)	
P03473	2019	2019-Q1	2019-02	Pre-pandemic	Cytology (Pap)	30-34	34	Not performed	Unsatisfactory	Unsatisfactory	Unsatisfactory	Unsatisfactory	
P03474	2019	2019-Q1	2019-02	Pre-pandemic	Cytology (Pap)	40-44	42	Not performed	Satisfactory	NILM	NILM	Negative (NILM)	
P03475	2019	2019-Q1	2019-02	Pre-pandemic	Cytology (Pap)	50-54	51	Not performed	Satisfactory	NILM	NILM	Negative (NILM)	
P03476	2019	2019-Q1	2019-02	Pre-pandemic	Cytology (Pap)	55-59	58	Not performed	Satisfactory	NILM	NILM	Negative (NILM)	
P03477	2019	2019-Q1	2019-02	Pre-pandemic	Cytology (Pap)	40-44	42	Not performed	Satisfactory	NILM	NILM	Negative (NILM)	
P03478	2019	2019-Q1	2019-02	Pre-pandemic	Cytology (Pap)	50-54	54	Not performed	Satisfactory	Positive	ASC-US	Low-grade / equivocal squamous	
P03479	2019	2019-Q1	2019-02	Pre-pandemic	Cytology (Pap)	35-39	35	Not performed	Satisfactory	NILM	NILM	Negative (NILM)	
P03480	2019	2019-Q1	2019-02	Pre-pandemic	Cytology (Pap)	35-39	36	Not performed	Satisfactory	NILM	NILM	Negative (NILM)	
P03481	2019	2019-Q1	2019-02	Pre-pandemic	Cytology (Pap)	50-54	53	Not performed	Satisfactory	NILM	NILM	Negative (NILM)	
P03482	2019	2019-Q1	2019-02	Pre-pandemic	Cytology (Pap)	50-54	54	Not performed	Satisfactory	Positive	HSIL	High-grade squamous	Candida
P03483	2019	2019-Q1	2019-02	Pre-pandemic	Cytology (Pap)	55-59	59	Not performed	Satisfactory	NILM	NILM	Negative (NILM)	
P03484	2019	2019-Q1	2019-02	Pre-pandemic	Cytology (Pap)	30-34	30	Not performed	Satisfactory	NILM	NILM	Negative (NILM)	
P03485	2019	2019-Q1	2019-02	Pre-pandemic	Cytology (Pap)	35-39	39	Not performed	Satisfactory	Positive	ASC-US	Low-grade / equivocal squamous	
P03486	2019	2019-Q1	2019-02	Pre-pandemic	Cytology (Pap)	50-54	54	Not performed	Satisfactory	NILM	NILM	Negative (NILM)	
P03487	2019	2019-Q1	2019-02	Pre-pandemic	Cytology (Pap)	40-44	41	Not performed	Satisfactory	NILM	NILM	Negative (NILM)	
P03488	2019	2019-Q1	2019-02	Pre-pandemic	Cytology (Pap)	55-59	57	Not performed	Satisfactory	NILM	NILM	Negative (NILM)	
P03489	2019	2019-Q1	2019-02	Pre-pandemic	Cytology (Pap)	30-34	33	Not performed	Unsatisfactory	Unsatisfactory	Unsatisfactory	Unsatisfactory	
P03490	2019	2019-Q1	2019-02	Pre-pandemic	Cytology (Pap)	40-44	41	Not performed	Satisfactory	NILM	NILM	Negative (NILM)	
P03491	2019	2019-Q1	2019-02	Pre-pandemic	Cytology (Pap)	35-39	36	Not performed	Satisfactory	NILM	NILM	Negative (NILM)	
P03492	2019	2019-Q1	2019-02	Pre-pandemic	Cytology (Pap)	35-39	36	Not performed	Satisfactory	NILM	NILM	Negative (NILM)	
P03493	2019	2019-Q1	2019-02	Pre-pandemic	Cytology (Pap)	40-44	40	Not performed	Satisfactory	NILM	NILM	Negative (NILM)	
P03494	2019	2019-Q1	2019-02	Pre-pandemic	Cytology (Pap)	35-39	37	Not performed	Satisfactory	NILM	NILM	Negative (NILM)	
P03495	2019	2019-Q1	2019-02	Pre-pandemic	Cytology (Pap)	60-64	61	Not performed	Unsatisfactory	Unsatisfactory	Unsatisfactory	Unsatisfactory	
P03496	2019	2019-Q1	2019-02	Pre-pandemic	Cytology (Pap)	60-64	62	Not performed	Satisfactory	NILM	NILM	Negative (NILM)	
P03497	2019	2019-Q1	2019-02	Pre-pandemic	Cytology (Pap)	30-34	32	Not performed	Satisfactory	NILM	NILM	Negative (NILM)	
P03498	2019	2019-Q1	2019-02	Pre-pandemic	Cytology (Pap)	60-64	62	Not performed	Satisfactory	Positive	LSIL	Low-grade / equivocal squamous	
P03499	2019	2019-Q1	2019-02	Pre-pandemic	Cytology (Pap)	25-29	26	Not performed	Satisfactory	NILM	NILM	Negative (NILM)	
P03500	2019	2019-Q1	2019-02	Pre-pandemic	Cytology (Pap)	25-29	29	Not performed	Satisfactory	Positive	ASC-US	Low-grade / equivocal squamous	
P03501	2019	2019-Q1	2019-02	Pre-pandemic	Cytology (Pap)	60-64	63	Not performed	Satisfactory	NILM	NILM	Negative (NILM)	
P03502	2019	2019-Q1	2019-02	Pre-pandemic	Cytology (Pap)	35-39	39	Not performed	Satisfactory	NILM	NILM	Negative (NILM)	
P03503	2019	2019-Q1	2019-02	Pre-pandemic	Cytology (Pap)	40-44	42	Not performed	Satisfactory	NILM	NILM	Negative (NILM)	
P03504	2019	2019-Q1	2019-02	Pre-pandemic	Cytology (Pap)	35-39	38	Not performed	Satisfactory	NILM	NILM	Negative (NILM)	
P03505	2019	2019-Q1	2019-02	Pre-pandemic	Cytology (Pap)	25-29	27	Not performed	Satisfactory	NILM	NILM	Negative (NILM)	
P03506	2019	2019-Q1	2019-02	Pre-pandemic	Cytology (Pap)	45-49	49	Not performed	Satisfactory	NILM	NILM	Negative (NILM)	
P03507	2019	2019-Q1	2019-02	Pre-pandemic	Cytology (Pap)	55-59	56	Not performed	Satisfactory	NILM	NILM	Negative (NILM)	
P03508	2019	2019-Q1	2019-02	Pre-pandemic	Cytology (Pap)	40-44	43	Not performed	Satisfactory	NILM	NILM	Negative (NILM)	
P03509	2019	2019-Q1	2019-02	Pre-pandemic	Cytology (Pap)	30-34	33	Not performed	Satisfactory	NILM	NILM	Negative (NILM)	
P03510	2019	2019-Q1	2019-02	Pre-pandemic	Cytology (Pap)	30-34	32	Not performed	Satisfactory	NILM	NILM	Negative (NILM)	Candida
P03511	2019	2019-Q1	2019-02	Pre-pandemic	Cytology (Pap)	45-49	45	Not performed	Satisfactory	NILM	NILM	Negative (NILM)	
P03512	2019	2019-Q1	2019-02	Pre-pandemic	Cytology (Pap)	50-54	54	Not performed	Satisfactory	NILM	NILM	Negative (NILM)	
P03513	2019	2019-Q1	2019-02	Pre-pandemic	Cytology (Pap)	35-39	35	Not performed	Satisfactory	NILM	NILM	Negative (NILM)	
P03514	2019	2019-Q1	2019-02	Pre-pandemic	Cytology (Pap)	40-44	41	Not performed	Satisfactory	NILM	NILM	Negative (NILM)	
P03515	2019	2019-Q1	2019-02	Pre-pandemic	Cytology (Pap)	45-49	45	Not performed	Satisfactory	NILM	NILM	Negative (NILM)	
P03516	2019	2019-Q1	2019-02	Pre-pandemic	Cytology (Pap)	30-34	33	Not performed	Satisfactory	NILM	NILM	Negative (NILM)	
P03517	2019	2019-Q1	2019-02	Pre-pandemic	Cytology (Pap)	45-49	45	Not performed	Satisfactory	NILM	NILM	Negative (NILM)	
P03518	2019	2019-Q1	2019-02	Pre-pandemic	Cytology (Pap)	35-39	37	Not performed	Satisfactory	NILM	NILM	Negative (NILM)	

patient_id	year	quarter	month	period	primary_test	age_group	age_years	hpv_result	adequacy	result	bethesda	grade	infection
P03519	2019	2019-Q1	2019-02	Pre-pandemic	Cytology (Pap)	60-64	64	Not performed	Satisfactory	NILM	NILM	Negative (NILM)	
P03520	2019	2019-Q1	2019-02	Pre-pandemic	Cytology (Pap)	50-54	52	Not performed	Satisfactory	NILM	NILM	Negative (NILM)	
P03521	2019	2019-Q1	2019-02	Pre-pandemic	Cytology (Pap)	60-64	64	Not performed	Satisfactory	NILM	NILM	Negative (NILM)	
P03522	2019	2019-Q1	2019-02	Pre-pandemic	Cytology (Pap)	45-49	47	Not performed	Satisfactory	NILM	NILM	Negative (NILM)	
P03523	2019	2019-Q1	2019-02	Pre-pandemic	Cytology (Pap)	40-44	42	Not performed	Satisfactory	NILM	NILM	Negative (NILM)	
P03524	2019	2019-Q1	2019-02	Pre-pandemic	Cytology (Pap)	35-39	37	Not performed	Satisfactory	NILM	NILM	Negative (NILM)	
P03525	2019	2019-Q1	2019-02	Pre-pandemic	Cytology (Pap)	60-64	63	Not performed	Unsatisfactory	Unsatisfactory	Unsatisfactory	Unsatisfactory	
P03526	2019	2019-Q1	2019-02	Pre-pandemic	Cytology (Pap)	55-59	55	Not performed	Satisfactory	NILM	NILM	Negative (NILM)	
P03527	2019	2019-Q1	2019-02	Pre-pandemic	Cytology (Pap)	45-49	47	Not performed	Satisfactory	NILM	NILM	Negative (NILM)	
P03528	2019	2019-Q1	2019-02	Pre-pandemic	Cytology (Pap)	50-54	50	Not performed	Satisfactory	NILM	NILM	Negative (NILM)	
P03529	2019	2019-Q1	2019-02	Pre-pandemic	Cytology (Pap)	40-44	41	Not performed	Satisfactory	Positive	ASC-US	Low-grade / equivocal squamous	
P03530	2019	2019-Q1	2019-02	Pre-pandemic	Cytology (Pap)	35-39	35	Not performed	Satisfactory	NILM	NILM	Negative (NILM)	
P03531	2019	2019-Q1	2019-02	Pre-pandemic	Cytology (Pap)	45-49	46	Not performed	Satisfactory	NILM	NILM	Negative (NILM)	
P03532	2019	2019-Q1	2019-02	Pre-pandemic	Cytology (Pap)	45-49	47	Not performed	Satisfactory	NILM	NILM	Negative (NILM)	
P03533	2019	2019-Q1	2019-02	Pre-pandemic	Cytology (Pap)	55-59	57	Not performed	Satisfactory	NILM	NILM	Negative (NILM)	
P03534	2019	2019-Q1	2019-02	Pre-pandemic	Cytology (Pap)	30-34	34	Not performed	Satisfactory	Positive	ASC-US	Low-grade / equivocal squamous	
P03535	2019	2019-Q1	2019-02	Pre-pandemic	Cytology (Pap)	30-34	34	Not performed	Satisfactory	NILM	NILM	Negative (NILM)	
P03536	2019	2019-Q1	2019-02	Pre-pandemic	Cytology (Pap)	30-34	32	Not performed	Satisfactory	NILM	NILM	Negative (NILM)	
P03537	2019	2019-Q1	2019-02	Pre-pandemic	Cytology (Pap)	30-34	34	Not performed	Satisfactory	NILM	NILM	Negative (NILM)	
P03538	2019	2019-Q1	2019-02	Pre-pandemic	Cytology (Pap)	55-59	58	Not performed	Satisfactory	NILM	NILM	Negative (NILM)	Trichomonas
P03539	2019	2019-Q1	2019-02	Pre-pandemic	Cytology (Pap)	45-49	46	Not performed	Satisfactory	Positive	HSIL	High-grade squamous	
P03540	2019	2019-Q1	2019-02	Pre-pandemic	Cytology (Pap)	50-54	52	Not performed	Satisfactory	NILM	NILM	Negative (NILM)	
P03541	2019	2019-Q1	2019-02	Pre-pandemic	Cytology (Pap)	30-34	32	Not performed	Satisfactory	NILM	NILM	Negative (NILM)	Candida
P03542	2019	2019-Q1	2019-02	Pre-pandemic	Cytology (Pap)	35-39	39	Not performed	Satisfactory	NILM	NILM	Negative (NILM)	
P03543	2019	2019-Q1	2019-02	Pre-pandemic	Cytology (Pap)	40-44	42	Not performed	Satisfactory	NILM	NILM	Negative (NILM)	
P03544	2019	2019-Q1	2019-02	Pre-pandemic	Cytology (Pap)	45-49	47	Not performed	Satisfactory	NILM	NILM	Negative (NILM)	
P03545	2019	2019-Q1	2019-02	Pre-pandemic	Cytology (Pap)	30-34	34	Not performed	Satisfactory	NILM	NILM	Negative (NILM)	
P03546	2019	2019-Q1	2019-02	Pre-pandemic	Cytology (Pap)	40-44	42	Not performed	Satisfactory	NILM	NILM	Negative (NILM)	
P03547	2019	2019-Q1	2019-02	Pre-pandemic	Cytology (Pap)	30-34	33	Not performed	Satisfactory	NILM	NILM	Negative (NILM)	
P03548	2019	2019-Q1	2019-02	Pre-pandemic	Cytology (Pap)	45-49	46	Not performed	Satisfactory	NILM	NILM	Negative (NILM)	
P03549	2019	2019-Q1	2019-02	Pre-pandemic	Cytology (Pap)	45-49	47	Not performed	Unsatisfactory	Unsatisfactory	Unsatisfactory	Unsatisfactory	
P03550	2019	2019-Q1	2019-02	Pre-pandemic	Cytology (Pap)	60-64	64	Not performed	Satisfactory	NILM	NILM	Negative (NILM)	
P03551	2019	2019-Q1	2019-02	Pre-pandemic	Cytology (Pap)	35-39	38	Not performed	Satisfactory	NILM	NILM	Negative (NILM)	
P03552	2019	2019-Q1	2019-02	Pre-pandemic	Cytology (Pap)	50-54	52	Not performed	Satisfactory	Positive	ASC-US	Low-grade / equivocal squamous	
P03553	2019	2019-Q1	2019-02	Pre-pandemic	Cytology (Pap)	50-54	52	Not performed	Satisfactory	NILM	NILM	Negative (NILM)	
P03554	2019	2019-Q1	2019-02	Pre-pandemic	Cytology (Pap)	35-39	37	Not performed	Satisfactory	NILM	NILM	Negative (NILM)	
P03555	2019	2019-Q1	2019-02	Pre-pandemic	Cytology (Pap)	50-54	50	Not performed	Satisfactory	NILM	NILM	Negative (NILM)	
P03556	2019	2019-Q1	2019-02	Pre-pandemic	Cytology (Pap)	40-44	42	Not performed	Satisfactory	NILM	NILM	Negative (NILM)	
P03557	2019	2019-Q1	2019-02	Pre-pandemic	Cytology (Pap)	35-39	38	Not performed	Satisfactory	NILM	NILM	Negative (NILM)	
P03558	2019	2019-Q1	2019-02	Pre-pandemic	Cytology (Pap)	55-59	56	Not performed	Satisfactory	NILM	NILM	Negative (NILM)	
P03559	2019	2019-Q1	2019-03	Pre-pandemic	Cytology (Pap)	35-39	38	Not performed	Satisfactory	NILM	NILM	Negative (NILM)	
P03560	2019	2019-Q1	2019-03	Pre-pandemic	Cytology (Pap)	30-34	30	Not performed	Satisfactory	NILM	NILM	Negative (NILM)	
P03561	2019	2019-Q1	2019-03	Pre-pandemic	Cytology (Pap)	35-39	38	Not performed	Satisfactory	NILM	NILM	Negative (NILM)	
P03562	2019	2019-Q1	2019-03	Pre-pandemic	Cytology (Pap)	45-49	47	Not performed	Satisfactory	NILM	NILM	Negative (NILM)	
P03563	2019	2019-Q1	2019-03	Pre-pandemic	Cytology (Pap)	45-49	49	Not performed	Satisfactory	NILM	NILM	Negative (NILM)	
P03564	2019	2019-Q1	2019-03	Pre-pandemic	Cytology (Pap)	40-44	43	Not performed	Satisfactory	NILM	NILM	Negative (NILM)	
P03565	2019	2019-Q1	2019-03	Pre-pandemic	Cytology (Pap)	55-59	56	Not performed	Satisfactory	Positive	HSIL	High-grade squamous	
P03566	2019	2019-Q1	2019-03	Pre-pandemic	Cytology (Pap)	60-64	63	Not performed	Satisfactory	NILM	NILM	Negative (NILM)	
P03567	2019	2019-Q1	2019-03	Pre-pandemic	Cytology (Pap)	30-34	32	Not performed	Satisfactory	NILM	NILM	Negative (NILM)	
P03568	2019	2019-Q1	2019-03	Pre-pandemic	Cytology (Pap)	45-49	47	Not performed	Satisfactory	NILM	NILM	Negative (NILM)	
P03569	2019	2019-Q1	2019-03	Pre-pandemic	Cytology (Pap)	55-59	56	Not performed	Satisfactory	Positive	HSIL	High-grade squamous	
P03570	2019	2019-Q1	2019-03	Pre-pandemic	Cytology (Pap)	30-34	33	Not performed	Satisfactory	Positive	LSIL	Low-grade / equivocal squamous	
P03571	2019	2019-Q1	2019-03	Pre-pandemic	Cytology (Pap)	40-44	40	Not performed	Satisfactory	NILM	NILM	Negative (NILM)	
P03572	2019	2019-Q1	2019-03	Pre-pandemic	Cytology (Pap)	60-64	64	Not performed	Satisfactory	NILM	NILM	Negative (NILM)	
P03573	2019	2019-Q1	2019-03	Pre-pandemic	Cytology (Pap)	50-54	51	Not performed	Satisfactory	NILM	NILM	Negative (NILM)	
P03574	2019	2019-Q1	2019-03	Pre-pandemic	Cytology (Pap)	50-54	51	Not performed	Satisfactory	NILM	NILM	Negative (NILM)	
P03575	2019	2019-Q1	2019-03	Pre-pandemic	Cytology (Pap)	45-49	48	Not performed	Satisfactory	NILM	NILM	Negative (NILM)	
P03576	2019	2019-Q1	2019-03	Pre-pandemic	Cytology (Pap)	40-44	40	Not performed	Satisfactory	NILM	NILM	Negative (NILM)	
P03577	2019	2019-Q1	2019-03	Pre-pandemic	Cytology (Pap)	50-54	54	Not performed	Satisfactory	NILM	NILM	Negative (NILM)	
P03578	2019	2019-Q1	2019-03	Pre-pandemic	Cytology (Pap)	50-54	53	Not performed	Satisfactory	NILM	NILM	Negative (NILM)	
P03579	2019	2019-Q1	2019-03	Pre-pandemic	Cytology (Pap)	35-39	37	Not performed	Satisfactory	NILM	NILM	Negative (NILM)	Candida
P03580	2019	2019-Q1	2019-03	Pre-pandemic	Cytology (Pap)	35-39	35	Not performed	Satisfactory	NILM	NILM	Negative (NILM)	
P03581	2019	2019-Q1	2019-03	Pre-pandemic	Cytology (Pap)	45-49	49	Not performed	Satisfactory	Positive	HSIL	High-grade squamous	
P03582	2019	2019-Q1	2019-03	Pre-pandemic	Cytology (Pap)	30-34	33	Not performed	Satisfactory	Positive	LSIL	Low-grade / equivocal squamous	
P03583	2019	2019-Q1	2019-03	Pre-pandemic	Cytology (Pap)	60-64	64	Not performed	Satisfactory	Positive	HSIL	High-grade squamous	
P03584	2019	2019-Q1	2019-03	Pre-pandemic	Cytology (Pap)	55-59	56	Not performed	Satisfactory	NILM	NILM	Negative (NILM)	
P03585	2019	2019-Q1	2019-03	Pre-pandemic	Cytology (Pap)	45-49	47	Not performed	Satisfactory	NILM	NILM	Negative (NILM)	
P03586	2019	2019-Q1	2019-03	Pre-pandemic	Cytology (Pap)	30-34	30	Not performed	Satisfactory	NILM	NILM	Negative (NILM)	
P03587	2019	2019-Q1	2019-03	Pre-pandemic	Cytology (Pap)	30-34	32	Not performed	Satisfactory	NILM	NILM	Negative (NILM)	
P03588	2019	2019-Q1	2019-03	Pre-pandemic	Cytology (Pap)	60-64	63	Not performed	Satisfactory	Positive	LSIL	Low-grade / equivocal squamous	
P03589	2019	2019-Q1	2019-03	Pre-pandemic	Cytology (Pap)	40-44	41	Not performed	Satisfactory	NILM	NILM	Negative (NILM)	
P03590	2019	2019-Q1	2019-03	Pre-pandemic	Cytology (Pap)	25-29	26	Not performed	Satisfactory	Positive	ASC-US	Low-grade / equivocal squamous	
P03591	2019	2019-Q1	2019-03	Pre-pandemic	Cytology (Pap)	50-54	53	Not performed	Satisfactory	Positive	HSIL	High-grade squamous	
P03592	2019	2019-Q1	2019-03	Pre-pandemic	Cytology (Pap)	45-49	46	Not performed	Satisfactory	NILM	NILM	Negative (NILM)	
P03593	2019	2019-Q1	2019-03	Pre-pandemic	Cytology (Pap)	35-39	35	Not performed	Satisfactory	NILM	NILM	Negative (NILM)	
P03594	2019	2019-Q1	2019-03	Pre-pandemic	Cytology (Pap)	45-49	49	Not performed	Satisfactory	NILM	NILM	Negative (NILM)	
P03595	2019	2019-Q1	2019-03	Pre-pandemic	Cytology (Pap)	60-64	60	Not performed	Satisfactory	NILM	NILM	Negative (NILM)	
P03596	2019	2019-Q1	2019-03	Pre-pandemic	Cytology (Pap)	40-44	43	Not performed	Satisfactory	NILM	NILM	Negative (NILM)	
P03597	2019	2019-Q1	2019-03	Pre-pandemic	Cytology (Pap)	30-34	30	Not performed	Satisfactory	NILM	NILM	Negative (NILM)	
P03598	2019	2019-Q1	2019-03	Pre-pandemic	Cytology (Pap)	45-49	45	Not performed	Satisfactory	NILM	NILM	Negative (NILM)	

patient_id	year	quarter	month	period	primary_test	age_group	age_years	hpv_result	adequacy	result	bethesda	grade	infection
P03599	2019	2019-Q1	2019-03	Pre-pandemic	Cytology (Pap)	50-54	50	Not performed	Satisfactory	NILM	NILM	Negative (NILM)	
P03600	2019	2019-Q1	2019-03	Pre-pandemic	Cytology (Pap)	35-39	37	Not performed	Unsatisfactory	Unsatisfactory	Unsatisfactory	Unsatisfactory	
P03601	2019	2019-Q1	2019-03	Pre-pandemic	Cytology (Pap)	40-44	43	Not performed	Satisfactory	NILM	NILM	Negative (NILM)	
P03602	2019	2019-Q1	2019-03	Pre-pandemic	Cytology (Pap)	30-34	34	Not performed	Satisfactory	NILM	NILM	Negative (NILM)	
P03603	2019	2019-Q1	2019-03	Pre-pandemic	Cytology (Pap)	30-34	34	Not performed	Satisfactory	NILM	NILM	Negative (NILM)	
P03604	2019	2019-Q1	2019-03	Pre-pandemic	Cytology (Pap)	55-59	58	Not performed	Satisfactory	NILM	NILM	Negative (NILM)	
P03605	2019	2019-Q1	2019-03	Pre-pandemic	Cytology (Pap)	60-64	63	Not performed	Satisfactory	NILM	NILM	Negative (NILM)	
P03606	2019	2019-Q1	2019-03	Pre-pandemic	Cytology (Pap)	40-44	42	Not performed	Satisfactory	NILM	NILM	Negative (NILM)	
P03607	2019	2019-Q1	2019-03	Pre-pandemic	Cytology (Pap)	50-54	53	Not performed	Satisfactory	NILM	NILM	Negative (NILM)	
P03608	2019	2019-Q1	2019-03	Pre-pandemic	Cytology (Pap)	50-54	51	Not performed	Satisfactory	NILM	NILM	Negative (NILM)	
P03609	2019	2019-Q1	2019-03	Pre-pandemic	Cytology (Pap)	60-64	63	Not performed	Satisfactory	Positive	ASC-H	High-grade squamous	
P03610	2019	2019-Q1	2019-03	Pre-pandemic	Cytology (Pap)	25-29	29	Not performed	Satisfactory	NILM	NILM	Negative (NILM)	
P03611	2019	2019-Q1	2019-03	Pre-pandemic	Cytology (Pap)	40-44	43	Not performed	Satisfactory	NILM	NILM	Negative (NILM)	
P03612	2019	2019-Q1	2019-03	Pre-pandemic	Cytology (Pap)	50-54	50	Not performed	Satisfactory	Positive	HSIL	High-grade squamous	
P03613	2019	2019-Q1	2019-03	Pre-pandemic	Cytology (Pap)	35-39	38	Not performed	Satisfactory	NILM	NILM	Negative (NILM)	
P03614	2019	2019-Q1	2019-03	Pre-pandemic	Cytology (Pap)	35-39	35	Not performed	Satisfactory	NILM	NILM	Negative (NILM)	
P03615	2019	2019-Q1	2019-03	Pre-pandemic	Cytology (Pap)	40-44	40	Not performed	Satisfactory	NILM	NILM	Negative (NILM)	
P03616	2019	2019-Q1	2019-03	Pre-pandemic	Cytology (Pap)	25-29	28	Not performed	Satisfactory	NILM	NILM	Negative (NILM)	
P03617	2019	2019-Q1	2019-03	Pre-pandemic	Cytology (Pap)	35-39	37	Not performed	Satisfactory	Positive	HSIL	High-grade squamous	
P03618	2019	2019-Q1	2019-03	Pre-pandemic	Cytology (Pap)	35-39	39	Not performed	Satisfactory	NILM	NILM	Negative (NILM)	
P03619	2019	2019-Q1	2019-03	Pre-pandemic	Cytology (Pap)	25-29	29	Not performed	Satisfactory	Positive	ASC-US	Low-grade / equivocal squamous	
P03620	2019	2019-Q1	2019-03	Pre-pandemic	Cytology (Pap)	30-34	33	Not performed	Satisfactory	NILM	NILM	Negative (NILM)	
P03621	2019	2019-Q1	2019-03	Pre-pandemic	Cytology (Pap)	25-29	29	Not performed	Satisfactory	NILM	NILM	Negative (NILM)	
P03622	2019	2019-Q1	2019-03	Pre-pandemic	Cytology (Pap)	45-49	48	Not performed	Satisfactory	NILM	NILM	Negative (NILM)	
P03623	2019	2019-Q1	2019-03	Pre-pandemic	Cytology (Pap)	40-44	41	Not performed	Satisfactory	NILM	NILM	Negative (NILM)	
P03624	2019	2019-Q1	2019-03	Pre-pandemic	Cytology (Pap)	50-54	53	Not performed	Satisfactory	NILM	NILM	Negative (NILM)	
P03625	2019	2019-Q1	2019-03	Pre-pandemic	Cytology (Pap)	40-44	40	Not performed	Satisfactory	Positive	ASC-H	High-grade squamous	
P03626	2019	2019-Q1	2019-03	Pre-pandemic	Cytology (Pap)	35-39	38	Not performed	Satisfactory	NILM	NILM	Negative (NILM)	
P03627	2019	2019-Q1	2019-03	Pre-pandemic	Cytology (Pap)	50-54	52	Not performed	Satisfactory	NILM	NILM	Negative (NILM)	
P03628	2019	2019-Q1	2019-03	Pre-pandemic	Cytology (Pap)	35-39	35	Not performed	Satisfactory	NILM	NILM	Negative (NILM)	
P03629	2019	2019-Q1	2019-03	Pre-pandemic	Cytology (Pap)	35-39	36	Not performed	Satisfactory	NILM	NILM	Negative (NILM)	
P03630	2019	2019-Q1	2019-03	Pre-pandemic	Cytology (Pap)	50-54	50	Not performed	Satisfactory	Positive	ASC-US	Low-grade / equivocal squamous	
P03631	2019	2019-Q1	2019-03	Pre-pandemic	Cytology (Pap)	50-54	52	Not performed	Satisfactory	NILM	NILM	Negative (NILM)	
P03632	2019	2019-Q1	2019-03	Pre-pandemic	Cytology (Pap)	45-49	47	Not performed	Satisfactory	NILM	NILM	Negative (NILM)	
P03633	2019	2019-Q1	2019-03	Pre-pandemic	Cytology (Pap)	40-44	41	Not performed	Satisfactory	NILM	NILM	Negative (NILM)	
P03634	2019	2019-Q1	2019-03	Pre-pandemic	Cytology (Pap)	45-49	46	Not performed	Unsatisfactory	Unsatisfactory	Unsatisfactory	Unsatisfactory	
P03635	2019	2019-Q1	2019-03	Pre-pandemic	Cytology (Pap)	35-39	38	Not performed	Satisfactory	Positive	ASC-US	Low-grade / equivocal squamous	
P03636	2019	2019-Q1	2019-03	Pre-pandemic	Cytology (Pap)	40-44	41	Not performed	Satisfactory	NILM	NILM	Negative (NILM)	
P03637	2019	2019-Q1	2019-03	Pre-pandemic	Cytology (Pap)	40-44	44	Not performed	Satisfactory	Positive	ASC-US	Low-grade / equivocal squamous	
P03638	2019	2019-Q1	2019-03	Pre-pandemic	Cytology (Pap)	40-44	44	Not performed	Satisfactory	NILM	NILM	Negative (NILM)	
P03639	2019	2019-Q1	2019-03	Pre-pandemic	Cytology (Pap)	35-39	39	Not performed	Satisfactory	NILM	NILM	Negative (NILM)	
P03640	2019	2019-Q1	2019-03	Pre-pandemic	Cytology (Pap)	45-49	45	Not performed	Satisfactory	NILM	NILM	Negative (NILM)	
P03641	2019	2019-Q1	2019-03	Pre-pandemic	Cytology (Pap)	30-34	33	Not performed	Satisfactory	NILM	NILM	Negative (NILM)	
P03642	2019	2019-Q1	2019-03	Pre-pandemic	Cytology (Pap)	55-59	57	Not performed	Satisfactory	NILM	NILM	Negative (NILM)	
P03643	2019	2019-Q1	2019-03	Pre-pandemic	Cytology (Pap)	45-49	45	Not performed	Satisfactory	NILM	NILM	Negative (NILM)	
P03644	2019	2019-Q1	2019-03	Pre-pandemic	Cytology (Pap)	55-59	59	Not performed	Satisfactory	NILM	NILM	Negative (NILM)	
P03645	2019	2019-Q1	2019-03	Pre-pandemic	Cytology (Pap)	30-34	31	Not performed	Satisfactory	NILM	NILM	Negative (NILM)	
P03646	2019	2019-Q1	2019-03	Pre-pandemic	Cytology (Pap)	30-34	31	Not performed	Satisfactory	NILM	NILM	Negative (NILM)	
P03647	2019	2019-Q1	2019-03	Pre-pandemic	Cytology (Pap)	35-39	39	Not performed	Satisfactory	NILM	NILM	Negative (NILM)	
P03648	2019	2019-Q1	2019-03	Pre-pandemic	Cytology (Pap)	35-39	37	Not performed	Satisfactory	NILM	NILM	Negative (NILM)	
P03649	2019	2019-Q1	2019-03	Pre-pandemic	Cytology (Pap)	50-54	52	Not performed	Satisfactory	NILM	NILM	Negative (NILM)	Candida
P03650	2019	2019-Q1	2019-03	Pre-pandemic	Cytology (Pap)	30-34	31	Not performed	Satisfactory	NILM	NILM	Negative (NILM)	
P03651	2019	2019-Q1	2019-03	Pre-pandemic	Cytology (Pap)	35-39	37	Not performed	Satisfactory	NILM	NILM	Negative (NILM)	
P03652	2019	2019-Q1	2019-03	Pre-pandemic	Cytology (Pap)	40-44	41	Not performed	Satisfactory	NILM	NILM	Negative (NILM)	
P03653	2019	2019-Q1	2019-03	Pre-pandemic	Cytology (Pap)	50-54	52	Not performed	Satisfactory	Positive	ASC-US	Low-grade / equivocal squamous	
P03654	2019	2019-Q1	2019-03	Pre-pandemic	Cytology (Pap)	45-49	46	Not performed	Satisfactory	NILM	NILM	Negative (NILM)	
P03655	2019	2019-Q1	2019-03	Pre-pandemic	Cytology (Pap)	30-34	30	Not performed	Satisfactory	NILM	NILM	Negative (NILM)	
P03656	2019	2019-Q1	2019-03	Pre-pandemic	Cytology (Pap)	35-39	36	Not performed	Satisfactory	NILM	NILM	Negative (NILM)	
P03657	2019	2019-Q1	2019-03	Pre-pandemic	Cytology (Pap)	55-59	59	Not performed	Satisfactory	NILM	NILM	Negative (NILM)	
P03658	2019	2019-Q1	2019-03	Pre-pandemic	Cytology (Pap)	50-54	54	Not performed	Satisfactory	Positive	ASC-US	Low-grade / equivocal squamous	
P03659	2019	2019-Q1	2019-03	Pre-pandemic	Cytology (Pap)	55-59	59	Not performed	Satisfactory	Positive	LSIL	Low-grade / equivocal squamous	
P03660	2019	2019-Q1	2019-03	Pre-pandemic	Cytology (Pap)	35-39	37	Not performed	Satisfactory	NILM	NILM	Negative (NILM)	
P03661	2019	2019-Q1	2019-03	Pre-pandemic	Cytology (Pap)	35-39	38	Not performed	Unsatisfactory	Unsatisfactory	Unsatisfactory	Unsatisfactory	
P03662	2019	2019-Q1	2019-03	Pre-pandemic	Cytology (Pap)	45-49	47	Not performed	Satisfactory	NILM	NILM	Negative (NILM)	
P03663	2019	2019-Q1	2019-03	Pre-pandemic	Cytology (Pap)	55-59	57	Not performed	Satisfactory	NILM	NILM	Negative (NILM)	
P03664	2019	2019-Q1	2019-03	Pre-pandemic	Cytology (Pap)	45-49	45	Not performed	Satisfactory	Positive	LSIL	Low-grade / equivocal squamous	
P03665	2019	2019-Q1	2019-03	Pre-pandemic	Cytology (Pap)	40-44	44	Not performed	Satisfactory	NILM	NILM	Negative (NILM)	
P03666	2019	2019-Q1	2019-03	Pre-pandemic	Cytology (Pap)	35-39	35	Not performed	Satisfactory	Positive	HSIL	High-grade squamous	
P03667	2019	2019-Q1	2019-03	Pre-pandemic	Cytology (Pap)	50-54	54	Not performed	Satisfactory	NILM	NILM	Negative (NILM)	
P03668	2019	2019-Q1	2019-03	Pre-pandemic	Cytology (Pap)	30-34	31	Not performed	Satisfactory	NILM	NILM	Negative (NILM)	
P03669	2019	2019-Q1	2019-03	Pre-pandemic	Cytology (Pap)	50-54	53	Not performed	Satisfactory	NILM	NILM	Negative (NILM)	
P03670	2019	2019-Q1	2019-03	Pre-pandemic	Cytology (Pap)	35-39	38	Not performed	Satisfactory	NILM	NILM	Negative (NILM)	
P03671	2019	2019-Q1	2019-03	Pre-pandemic	Cytology (Pap)	35-39	39	Not performed	Satisfactory	NILM	NILM	Negative (NILM)	
P03672	2019	2019-Q1	2019-03	Pre-pandemic	Cytology (Pap)	55-59	55	Not performed	Satisfactory	NILM	NILM	Negative (NILM)	
P03673	2019	2019-Q1	2019-03	Pre-pandemic	Cytology (Pap)	30-34	30	Not performed	Satisfactory	NILM	NILM	Negative (NILM)	
P03674	2019	2019-Q1	2019-03	Pre-pandemic	Cytology (Pap)	30-34	31	Not performed	Satisfactory	NILM	NILM	Negative (NILM)	
P03675	2019	2019-Q1	2019-03	Pre-pandemic	Cytology (Pap)	40-44	44	Not performed	Satisfactory	NILM	NILM	Negative (NILM)	
P03676	2019	2019-Q1	2019-03	Pre-pandemic	Cytology (Pap)	40-44	44	Not performed	Satisfactory	Positive	ASC-H	High-grade squamous	
P03677	2019	2019-Q1	2019-03	Pre-pandemic	Cytology (Pap)	40-44	41	Not performed	Satisfactory	NILM	NILM	Negative (NILM)	
P03678	2019	2019-Q1	2019-03	Pre-pandemic	Cytology (Pap)	50-54	54	Not performed	Satisfactory	Positive	ASC-H	High-grade squamous	

patient_id	year	quarter	month	period	primary_test	age_group	age_years	hpv_result	adequacy	result	bethesda	grade	infection
P03679	2019	2019-Q1	2019-03	Pre-pandemic	Cytology (Pap)	60-64	61	Not performed	Satisfactory	Positive	ASC-US	Low-grade / equivocal squamous	
P03680	2019	2019-Q1	2019-03	Pre-pandemic	Cytology (Pap)	25-29	26	Not performed	Satisfactory	NILM	NILM	Negative (NILM)	
P03681	2019	2019-Q1	2019-03	Pre-pandemic	Cytology (Pap)	60-64	63	Not performed	Satisfactory	NILM	NILM	Negative (NILM)	
P03682	2019	2019-Q1	2019-03	Pre-pandemic	Cytology (Pap)	55-59	57	Not performed	Satisfactory	NILM	NILM	Negative (NILM)	
P03683	2019	2019-Q1	2019-03	Pre-pandemic	Cytology (Pap)	40-44	40	Not performed	Satisfactory	NILM	NILM	Negative (NILM)	
P03684	2019	2019-Q1	2019-03	Pre-pandemic	Cytology (Pap)	45-49	49	Not performed	Satisfactory	NILM	NILM	Negative (NILM)	
P03685	2019	2019-Q1	2019-03	Pre-pandemic	Cytology (Pap)	35-39	37	Not performed	Satisfactory	NILM	NILM	Negative (NILM)	
P03686	2019	2019-Q1	2019-03	Pre-pandemic	Cytology (Pap)	40-44	42	Not performed	Satisfactory	NILM	NILM	Negative (NILM)	
P03687	2019	2019-Q1	2019-03	Pre-pandemic	Cytology (Pap)	50-54	52	Not performed	Satisfactory	Positive	LSIL	Low-grade / equivocal squamous	
P03688	2019	2019-Q1	2019-03	Pre-pandemic	Cytology (Pap)	45-49	47	Not performed	Satisfactory	NILM	NILM	Negative (NILM)	
P03689	2019	2019-Q1	2019-03	Pre-pandemic	Cytology (Pap)	60-64	61	Not performed	Satisfactory	NILM	NILM	Negative (NILM)	
P03690	2019	2019-Q1	2019-03	Pre-pandemic	Cytology (Pap)	45-49	48	Not performed	Unsatisfactory	Unsatisfactory	Unsatisfactory	Unsatisfactory	
P03691	2019	2019-Q1	2019-03	Pre-pandemic	Cytology (Pap)	35-39	38	Not performed	Satisfactory	NILM	NILM	Negative (NILM)	
P03692	2019	2019-Q1	2019-03	Pre-pandemic	Cytology (Pap)	45-49	46	Not performed	Satisfactory	NILM	NILM	Negative (NILM)	
P03693	2019	2019-Q1	2019-03	Pre-pandemic	Cytology (Pap)	45-49	47	Not performed	Satisfactory	NILM	NILM	Negative (NILM)	
P03694	2019	2019-Q1	2019-03	Pre-pandemic	Cytology (Pap)	35-39	36	Not performed	Satisfactory	NILM	NILM	Negative (NILM)	
P03695	2019	2019-Q1	2019-03	Pre-pandemic	Cytology (Pap)	35-39	36	Not performed	Satisfactory	NILM	NILM	Negative (NILM)	
P03696	2019	2019-Q1	2019-03	Pre-pandemic	Cytology (Pap)	55-59	55	Not performed	Satisfactory	NILM	NILM	Negative (NILM)	
P03697	2019	2019-Q1	2019-03	Pre-pandemic	Cytology (Pap)	30-34	32	Not performed	Satisfactory	NILM	NILM	Negative (NILM)	
P03698	2019	2019-Q1	2019-03	Pre-pandemic	Cytology (Pap)	30-34	32	Not performed	Satisfactory	NILM	NILM	Negative (NILM)	
P03699	2019	2019-Q1	2019-03	Pre-pandemic	Cytology (Pap)	35-39	35	Not performed	Satisfactory	NILM	NILM	Negative (NILM)	
P03700	2019	2019-Q1	2019-03	Pre-pandemic	Cytology (Pap)	50-54	54	Not performed	Satisfactory	NILM	NILM	Negative (NILM)	
P03701	2019	2019-Q1	2019-03	Pre-pandemic	Cytology (Pap)	25-29	27	Not performed	Satisfactory	NILM	NILM	Negative (NILM)	
P03702	2019	2019-Q1	2019-03	Pre-pandemic	Cytology (Pap)	40-44	44	Not performed	Satisfactory	NILM	NILM	Negative (NILM)	
P03703	2019	2019-Q1	2019-03	Pre-pandemic	Cytology (Pap)	45-49	46	Not performed	Satisfactory	NILM	NILM	Negative (NILM)	
P03704	2019	2019-Q1	2019-03	Pre-pandemic	Cytology (Pap)	50-54	53	Not performed	Unsatisfactory	Unsatisfactory	Unsatisfactory	Unsatisfactory	
P03705	2019	2019-Q1	2019-03	Pre-pandemic	Cytology (Pap)	50-54	53	Not performed	Satisfactory	NILM	NILM	Negative (NILM)	
P03706	2019	2019-Q1	2019-03	Pre-pandemic	Cytology (Pap)	45-49	47	Not performed	Satisfactory	NILM	NILM	Negative (NILM)	
P03707	2019	2019-Q1	2019-03	Pre-pandemic	Cytology (Pap)	50-54	52	Not performed	Satisfactory	NILM	NILM	Negative (NILM)	
P03708	2019	2019-Q1	2019-03	Pre-pandemic	Cytology (Pap)	35-39	37	Not performed	Unsatisfactory	Unsatisfactory	Unsatisfactory	Unsatisfactory	
P03709	2019	2019-Q1	2019-03	Pre-pandemic	Cytology (Pap)	35-39	36	Not performed	Satisfactory	Positive	LSIL	Low-grade / equivocal squamous	
P03710	2019	2019-Q1	2019-03	Pre-pandemic	Cytology (Pap)	50-54	54	Not performed	Satisfactory	NILM	NILM	Negative (NILM)	
P03711	2019	2019-Q1	2019-03	Pre-pandemic	Cytology (Pap)	50-54	52	Not performed	Satisfactory	NILM	NILM	Negative (NILM)	
P03712	2019	2019-Q1	2019-03	Pre-pandemic	Cytology (Pap)	40-44	40	Not performed	Satisfactory	NILM	NILM	Negative (NILM)	
P03713	2019	2019-Q1	2019-03	Pre-pandemic	Cytology (Pap)	45-49	49	Not performed	Unsatisfactory	Unsatisfactory	Unsatisfactory	Unsatisfactory	
P03714	2019	2019-Q1	2019-03	Pre-pandemic	Cytology (Pap)	40-44	41	Not performed	Satisfactory	NILM	NILM	Negative (NILM)	
P03715	2019	2019-Q1	2019-03	Pre-pandemic	Cytology (Pap)	25-29	28	Not performed	Satisfactory	NILM	NILM	Negative (NILM)	
P03716	2019	2019-Q1	2019-03	Pre-pandemic	Cytology (Pap)	55-59	58	Not performed	Satisfactory	NILM	NILM	Negative (NILM)	
P03717	2019	2019-Q1	2019-03	Pre-pandemic	Cytology (Pap)	30-34	33	Not performed	Satisfactory	NILM	NILM	Negative (NILM)	
P03718	2019	2019-Q1	2019-03	Pre-pandemic	Cytology (Pap)	55-59	58	Not performed	Unsatisfactory	Unsatisfactory	Unsatisfactory	Unsatisfactory	
P03719	2019	2019-Q1	2019-03	Pre-pandemic	Cytology (Pap)	40-44	41	Not performed	Satisfactory	NILM	NILM	Negative (NILM)	
P03720	2019	2019-Q1	2019-03	Pre-pandemic	Cytology (Pap)	40-44	43	Not performed	Satisfactory	NILM	NILM	Negative (NILM)	
P03721	2019	2019-Q1	2019-03	Pre-pandemic	Cytology (Pap)	50-54	54	Not performed	Satisfactory	NILM	NILM	Negative (NILM)	
P03722	2019	2019-Q1	2019-03	Pre-pandemic	Cytology (Pap)	35-39	36	Not performed	Satisfactory	NILM	NILM	Negative (NILM)	
P03723	2019	2019-Q1	2019-03	Pre-pandemic	Cytology (Pap)	50-54	54	Not performed	Satisfactory	NILM	NILM	Negative (NILM)	
P03724	2019	2019-Q1	2019-03	Pre-pandemic	Cytology (Pap)	50-54	52	Not performed	Satisfactory	NILM	NILM	Negative (NILM)	
P03725	2019	2019-Q1	2019-03	Pre-pandemic	Cytology (Pap)	55-59	58	Not performed	Satisfactory	Positive	ASC-H	High-grade squamous	
P03726	2019	2019-Q1	2019-03	Pre-pandemic	Cytology (Pap)	40-44	41	Not performed	Satisfactory	NILM	NILM	Negative (NILM)	Trichomonas
P03727	2019	2019-Q1	2019-03	Pre-pandemic	Cytology (Pap)	60-64	60	Not performed	Satisfactory	NILM	NILM	Negative (NILM)	
P03728	2019	2019-Q1	2019-03	Pre-pandemic	Cytology (Pap)	40-44	44	Not performed	Satisfactory	NILM	NILM	Negative (NILM)	
P03729	2019	2019-Q1	2019-03	Pre-pandemic	Cytology (Pap)	35-39	35	Not performed	Satisfactory	NILM	NILM	Negative (NILM)	
P03730	2019	2019-Q1	2019-03	Pre-pandemic	Cytology (Pap)	40-44	41	Not performed	Satisfactory	NILM	NILM	Negative (NILM)	
P03731	2019	2019-Q1	2019-03	Pre-pandemic	Cytology (Pap)	40-44	44	Not performed	Satisfactory	NILM	NILM	Negative (NILM)	
P03732	2019	2019-Q1	2019-03	Pre-pandemic	Cytology (Pap)	55-59	59	Not performed	Satisfactory	NILM	NILM	Negative (NILM)	
P03733	2019	2019-Q1	2019-03	Pre-pandemic	Cytology (Pap)	45-49	49	Not performed	Satisfactory	NILM	NILM	Negative (NILM)	
P03734	2019	2019-Q1	2019-03	Pre-pandemic	Cytology (Pap)	55-59	58	Not performed	Satisfactory	NILM	NILM	Negative (NILM)	
P03735	2019	2019-Q1	2019-03	Pre-pandemic	Cytology (Pap)	40-44	42	Not performed	Satisfactory	NILM	NILM	Negative (NILM)	
P03736	2019	2019-Q1	2019-03	Pre-pandemic	Cytology (Pap)	30-34	32	Not performed	Satisfactory	Positive	LSIL	Low-grade / equivocal squamous	
P03737	2019	2019-Q1	2019-03	Pre-pandemic	Cytology (Pap)	30-34	31	Not performed	Satisfactory	NILM	NILM	Negative (NILM)	
P03738	2019	2019-Q1	2019-03	Pre-pandemic	Cytology (Pap)	30-34	34	Not performed	Satisfactory	NILM	NILM	Negative (NILM)	
P03739	2019	2019-Q1	2019-03	Pre-pandemic	Cytology (Pap)	30-34	30	Not performed	Satisfactory	NILM	NILM	Negative (NILM)	
P03740	2019	2019-Q1	2019-03	Pre-pandemic	Cytology (Pap)	40-44	43	Not performed	Satisfactory	Positive	HSIL	High-grade squamous	
P03741	2019	2019-Q1	2019-03	Pre-pandemic	Cytology (Pap)	40-44	44	Not performed	Satisfactory	NILM	NILM	Negative (NILM)	
P03742	2019	2019-Q1	2019-03	Pre-pandemic	Cytology (Pap)	60-64	63	Not performed	Satisfactory	NILM	NILM	Negative (NILM)	
P03743	2019	2019-Q1	2019-03	Pre-pandemic	Cytology (Pap)	45-49	49	Not performed	Satisfactory	Positive	HSIL	High-grade squamous	
P03744	2019	2019-Q1	2019-03	Pre-pandemic	Cytology (Pap)	30-34	33	Not performed	Satisfactory	NILM	NILM	Negative (NILM)	
P03745	2019	2019-Q1	2019-03	Pre-pandemic	Cytology (Pap)	25-29	26	Not performed	Satisfactory	NILM	NILM	Negative (NILM)	
P03746	2019	2019-Q1	2019-03	Pre-pandemic	Cytology (Pap)	35-39	37	Not performed	Satisfactory	NILM	NILM	Negative (NILM)	
P03747	2019	2019-Q1	2019-03	Pre-pandemic	Cytology (Pap)	40-44	40	Not performed	Satisfactory	Positive	LSIL	Low-grade / equivocal squamous	
P03748	2019	2019-Q1	2019-03	Pre-pandemic	Cytology (Pap)	50-54	51	Not performed	Satisfactory	NILM	NILM	Negative (NILM)	
P03749	2019	2019-Q1	2019-03	Pre-pandemic	Cytology (Pap)	45-49	49	Not performed	Satisfactory	NILM	NILM	Negative (NILM)	
P03750	2019	2019-Q1	2019-03	Pre-pandemic	Cytology (Pap)	35-39	38	Not performed	Satisfactory	NILM	NILM	Negative (NILM)	
P03751	2019	2019-Q1	2019-03	Pre-pandemic	Cytology (Pap)	35-39	38	Not performed	Satisfactory	NILM	NILM	Negative (NILM)	
P03752	2019	2019-Q1	2019-03	Pre-pandemic	Cytology (Pap)	35-39	36	Not performed	Satisfactory	NILM	NILM	Negative (NILM)	
P03753	2019	2019-Q1	2019-03	Pre-pandemic	Cytology (Pap)	30-34	31	Not performed	Satisfactory	NILM	NILM	Negative (NILM)	
P03754	2019	2019-Q1	2019-03	Pre-pandemic	Cytology (Pap)	50-54	54	Not performed	Satisfactory	NILM	NILM	Negative (NILM)	
P03755	2019	2019-Q1	2019-03	Pre-pandemic	Cytology (Pap)	50-54	51	Not performed	Satisfactory	NILM	NILM	Negative (NILM)	
P03756	2019	2019-Q1	2019-03	Pre-pandemic	Cytology (Pap)	40-44	42	Not performed	Satisfactory	NILM	NILM	Negative (NILM)	
P03757	2019	2019-Q1	2019-03	Pre-pandemic	Cytology (Pap)	50-54	52	Not performed	Satisfactory	NILM	NILM	Negative (NILM)	
P03758	2019	2019-Q1	2019-03	Pre-pandemic	Cytology (Pap)	45-49	47	Not performed	Satisfactory	NILM	NILM	Negative (NILM)	

patient_id	year	quarter	month	period	primary_test	age_group	age_years	hpv_result	adequacy	result	bethesda	grade	infection
P03759	2019	2019-Q1	2019-03	Pre-pandemic	Cytology (Pap)	45-49	46	Not performed	Satisfactory	Positive	ASC-US	Low-grade / equivocal squamous	
P03760	2019	2019-Q1	2019-03	Pre-pandemic	Cytology (Pap)	45-49	45	Not performed	Satisfactory	NILM	NILM	Negative (NILM)	
P03761	2019	2019-Q1	2019-03	Pre-pandemic	Cytology (Pap)	40-44	44	Not performed	Satisfactory	NILM	NILM	Negative (NILM)	
P03762	2019	2019-Q1	2019-03	Pre-pandemic	Cytology (Pap)	40-44	41	Not performed	Satisfactory	NILM	NILM	Negative (NILM)	
P03763	2019	2019-Q1	2019-03	Pre-pandemic	Cytology (Pap)	30-34	34	Not performed	Satisfactory	NILM	NILM	Negative (NILM)	
P03764	2019	2019-Q1	2019-03	Pre-pandemic	Cytology (Pap)	55-59	58	Not performed	Satisfactory	NILM	NILM	Negative (NILM)	
P03765	2019	2019-Q1	2019-03	Pre-pandemic	Cytology (Pap)	50-54	52	Not performed	Satisfactory	Positive	HSIL	High-grade squamous	
P03766	2019	2019-Q1	2019-03	Pre-pandemic	Cytology (Pap)	30-34	34	Not performed	Satisfactory	NILM	NILM	Negative (NILM)	
P03767	2019	2019-Q1	2019-03	Pre-pandemic	Cytology (Pap)	60-64	62	Not performed	Satisfactory	NILM	NILM	Negative (NILM)	
P03768	2019	2019-Q1	2019-03	Pre-pandemic	Cytology (Pap)	55-59	57	Not performed	Satisfactory	NILM	NILM	Negative (NILM)	
P03769	2019	2019-Q1	2019-03	Pre-pandemic	Cytology (Pap)	50-54	54	Not performed	Satisfactory	Positive	HSIL	High-grade squamous	
P03770	2019	2019-Q1	2019-03	Pre-pandemic	Cytology (Pap)	25-29	26	Not performed	Satisfactory	NILM	NILM	Negative (NILM)	
P03771	2019	2019-Q1	2019-03	Pre-pandemic	Cytology (Pap)	25-29	29	Not performed	Satisfactory	NILM	NILM	Negative (NILM)	
P03772	2019	2019-Q1	2019-03	Pre-pandemic	Cytology (Pap)	30-34	33	Not performed	Satisfactory	NILM	NILM	Negative (NILM)	
P03773	2019	2019-Q1	2019-03	Pre-pandemic	Cytology (Pap)	35-39	35	Not performed	Satisfactory	NILM	NILM	Negative (NILM)	
P03774	2019	2019-Q1	2019-03	Pre-pandemic	Cytology (Pap)	40-44	40	Not performed	Unsatisfactory	Unsatisfactory	Unsatisfactory	Unsatisfactory	
P03775	2019	2019-Q1	2019-03	Pre-pandemic	Cytology (Pap)	45-49	45	Not performed	Satisfactory	NILM	NILM	Negative (NILM)	
P03776	2019	2019-Q1	2019-03	Pre-pandemic	Cytology (Pap)	35-39	38	Not performed	Satisfactory	NILM	NILM	Negative (NILM)	
P03777	2019	2019-Q1	2019-03	Pre-pandemic	Cytology (Pap)	40-44	42	Not performed	Satisfactory	NILM	NILM	Negative (NILM)	
P03778	2019	2019-Q1	2019-03	Pre-pandemic	Cytology (Pap)	40-44	40	Not performed	Satisfactory	NILM	NILM	Negative (NILM)	
P03779	2019	2019-Q1	2019-03	Pre-pandemic	Cytology (Pap)	40-44	42	Not performed	Satisfactory	NILM	NILM	Negative (NILM)	
P03780	2019	2019-Q1	2019-03	Pre-pandemic	Cytology (Pap)	40-44	41	Not performed	Satisfactory	Positive	LSIL	Low-grade / equivocal squamous	
P03781	2019	2019-Q1	2019-03	Pre-pandemic	Cytology (Pap)	30-34	32	Not performed	Satisfactory	NILM	NILM	Negative (NILM)	
P03782	2019	2019-Q1	2019-03	Pre-pandemic	Cytology (Pap)	40-44	41	Not performed	Satisfactory	NILM	NILM	Negative (NILM)	
P03783	2019	2019-Q1	2019-03	Pre-pandemic	Cytology (Pap)	40-44	41	Not performed	Satisfactory	NILM	NILM	Negative (NILM)	
P03784	2019	2019-Q1	2019-03	Pre-pandemic	Cytology (Pap)	35-39	39	Not performed	Satisfactory	NILM	NILM	Negative (NILM)	
P03785	2019	2019-Q1	2019-03	Pre-pandemic	Cytology (Pap)	40-44	44	Not performed	Satisfactory	NILM	NILM	Negative (NILM)	
P03786	2019	2019-Q1	2019-03	Pre-pandemic	Cytology (Pap)	45-49	48	Not performed	Satisfactory	NILM	NILM	Negative (NILM)	
P03787	2019	2019-Q1	2019-03	Pre-pandemic	Cytology (Pap)	30-34	31	Not performed	Satisfactory	NILM	NILM	Negative (NILM)	
P03788	2019	2019-Q1	2019-03	Pre-pandemic	Cytology (Pap)	30-34	33	Not performed	Unsatisfactory	Unsatisfactory	Unsatisfactory	Unsatisfactory	
P03789	2019	2019-Q1	2019-03	Pre-pandemic	Cytology (Pap)	40-44	40	Not performed	Satisfactory	NILM	NILM	Negative (NILM)	
P03790	2019	2019-Q1	2019-03	Pre-pandemic	Cytology (Pap)	55-59	57	Not performed	Satisfactory	NILM	NILM	Negative (NILM)	
P03791	2019	2019-Q1	2019-03	Pre-pandemic	Cytology (Pap)	55-59	58	Not performed	Unsatisfactory	Unsatisfactory	Unsatisfactory	Unsatisfactory	
P03792	2019	2019-Q1	2019-03	Pre-pandemic	Cytology (Pap)	35-39	37	Not performed	Satisfactory	NILM	NILM	Negative (NILM)	
P03793	2019	2019-Q1	2019-03	Pre-pandemic	Cytology (Pap)	60-64	62	Not performed	Satisfactory	Positive	ASC-US	Low-grade / equivocal squamous	
P03794	2019	2019-Q1	2019-03	Pre-pandemic	Cytology (Pap)	30-34	33	Not performed	Satisfactory	NILM	NILM	Negative (NILM)	
P03795	2019	2019-Q1	2019-03	Pre-pandemic	Cytology (Pap)	30-34	33	Not performed	Satisfactory	NILM	NILM	Negative (NILM)	
P03796	2019	2019-Q1	2019-03	Pre-pandemic	Cytology (Pap)	40-44	40	Not performed	Satisfactory	NILM	NILM	Negative (NILM)	
P03797	2019	2019-Q1	2019-03	Pre-pandemic	Cytology (Pap)	55-59	56	Not performed	Satisfactory	Positive	LSIL	Low-grade / equivocal squamous	
P03798	2019	2019-Q1	2019-03	Pre-pandemic	Cytology (Pap)	55-59	55	Not performed	Satisfactory	NILM	NILM	Negative (NILM)	Candida
P03799	2019	2019-Q1	2019-03	Pre-pandemic	Cytology (Pap)	25-29	27	Not performed	Satisfactory	NILM	NILM	Negative (NILM)	
P03800	2019	2019-Q1	2019-03	Pre-pandemic	Cytology (Pap)	55-59	58	Not performed	Satisfactory	Positive	HSIL	High-grade squamous	
P03801	2019	2019-Q1	2019-03	Pre-pandemic	Cytology (Pap)	35-39	39	Not performed	Satisfactory	NILM	NILM	Negative (NILM)	
P03802	2019	2019-Q1	2019-03	Pre-pandemic	Cytology (Pap)	50-54	50	Not performed	Satisfactory	NILM	NILM	Negative (NILM)	
P03803	2019	2019-Q1	2019-03	Pre-pandemic	Cytology (Pap)	25-29	25	Not performed	Satisfactory	NILM	NILM	Negative (NILM)	
P03804	2019	2019-Q1	2019-03	Pre-pandemic	Cytology (Pap)	35-39	39	Not performed	Satisfactory	NILM	NILM	Negative (NILM)	
P03805	2019	2019-Q1	2019-03	Pre-pandemic	Cytology (Pap)	50-54	50	Not performed	Satisfactory	NILM	NILM	Negative (NILM)	
P03806	2019	2019-Q1	2019-03	Pre-pandemic	Cytology (Pap)	45-49	47	Not performed	Satisfactory	NILM	NILM	Negative (NILM)	
P03807	2019	2019-Q1	2019-03	Pre-pandemic	Cytology (Pap)	60-64	63	Not performed	Satisfactory	NILM	NILM	Negative (NILM)	
P03808	2019	2019-Q1	2019-03	Pre-pandemic	Cytology (Pap)	45-49	45	Not performed	Satisfactory	Positive	ASC-US	Low-grade / equivocal squamous	
P03809	2019	2019-Q1	2019-03	Pre-pandemic	Cytology (Pap)	25-29	26	Not performed	Satisfactory	NILM	NILM	Negative (NILM)	
P03810	2019	2019-Q1	2019-03	Pre-pandemic	Cytology (Pap)	30-34	31	Not performed	Satisfactory	NILM	NILM	Negative (NILM)	
P03811	2019	2019-Q1	2019-03	Pre-pandemic	Cytology (Pap)	50-54	51	Not performed	Satisfactory	NILM	NILM	Negative (NILM)	
P03812	2019	2019-Q1	2019-03	Pre-pandemic	Cytology (Pap)	40-44	40	Not performed	Satisfactory	NILM	NILM	Negative (NILM)	
P03813	2019	2019-Q1	2019-03	Pre-pandemic	Cytology (Pap)	40-44	40	Not performed	Satisfactory	NILM	NILM	Negative (NILM)	
P03814	2019	2019-Q1	2019-03	Pre-pandemic	Cytology (Pap)	60-64	63	Not performed	Unsatisfactory	Unsatisfactory	Unsatisfactory	Unsatisfactory	
P03815	2019	2019-Q1	2019-03	Pre-pandemic	Cytology (Pap)	35-39	35	Not performed	Unsatisfactory	Unsatisfactory	Unsatisfactory	Unsatisfactory	
P03816	2019	2019-Q1	2019-03	Pre-pandemic	Cytology (Pap)	30-34	30	Not performed	Satisfactory	NILM	NILM	Negative (NILM)	
P03817	2019	2019-Q1	2019-03	Pre-pandemic	Cytology (Pap)	40-44	40	Not performed	Satisfactory	NILM	NILM	Negative (NILM)	
P03818	2019	2019-Q1	2019-03	Pre-pandemic	Cytology (Pap)	50-54	50	Not performed	Satisfactory	NILM	NILM	Negative (NILM)	
P03819	2019	2019-Q1	2019-03	Pre-pandemic	Cytology (Pap)	50-54	54	Not performed	Satisfactory	NILM	NILM	Negative (NILM)	
P03820	2019	2019-Q1	2019-03	Pre-pandemic	Cytology (Pap)	40-44	40	Not performed	Satisfactory	NILM	NILM	Negative (NILM)	
P03821	2019	2019-Q1	2019-03	Pre-pandemic	Cytology (Pap)	40-44	41	Not performed	Satisfactory	NILM	NILM	Negative (NILM)	
P03822	2019	2019-Q1	2019-03	Pre-pandemic	Cytology (Pap)	40-44	43	Not performed	Satisfactory	NILM	NILM	Negative (NILM)	
P03823	2019	2019-Q1	2019-03	Pre-pandemic	Cytology (Pap)	60-64	62	Not performed	Satisfactory	NILM	NILM	Negative (NILM)	
P03824	2019	2019-Q1	2019-03	Pre-pandemic	Cytology (Pap)	40-44	42	Not performed	Satisfactory	NILM	NILM	Negative (NILM)	
P03825	2019	2019-Q1	2019-03	Pre-pandemic	Cytology (Pap)	40-44	40	Not performed	Satisfactory	NILM	NILM	Negative (NILM)	
P03826	2019	2019-Q1	2019-03	Pre-pandemic	Cytology (Pap)	45-49	47	Not performed	Satisfactory	NILM	NILM	Negative (NILM)	
P03827	2019	2019-Q1	2019-03	Pre-pandemic	Cytology (Pap)	35-39	36	Not performed	Satisfactory	NILM	NILM	Negative (NILM)	
P03828	2019	2019-Q1	2019-03	Pre-pandemic	Cytology (Pap)	50-54	52	Not performed	Satisfactory	NILM	NILM	Negative (NILM)	
P03829	2019	2019-Q1	2019-03	Pre-pandemic	Cytology (Pap)	40-44	42	Not performed	Satisfactory	NILM	NILM	Negative (NILM)	
P03830	2019	2019-Q1	2019-03	Pre-pandemic	Cytology (Pap)	50-54	54	Not performed	Satisfactory	NILM	NILM	Negative (NILM)	
P03831	2019	2019-Q1	2019-03	Pre-pandemic	Cytology (Pap)	45-49	49	Not performed	Satisfactory	NILM	NILM	Negative (NILM)	
P03832	2019	2019-Q1	2019-03	Pre-pandemic	Cytology (Pap)	45-49	46	Not performed	Satisfactory	NILM	NILM	Negative (NILM)	
P03833	2019	2019-Q1	2019-03	Pre-pandemic	Cytology (Pap)	55-59	57	Not performed	Satisfactory	NILM	NILM	Negative (NILM)	
P03834	2019	2019-Q1	2019-03	Pre-pandemic	Cytology (Pap)	55-59	58	Not performed	Satisfactory	NILM	NILM	Negative (NILM)	
P03835	2019	2019-Q1	2019-03	Pre-pandemic	Cytology (Pap)	40-44	43	Not performed	Satisfactory	Positive	HSIL	High-grade squamous	
P03836	2019	2019-Q1	2019-03	Pre-pandemic	Cytology (Pap)	45-49	45	Not performed	Satisfactory	NILM	NILM	Negative (NILM)	
P03837	2019	2019-Q1	2019-03	Pre-pandemic	Cytology (Pap)	35-39	35	Not performed	Satisfactory	NILM	NILM	Negative (NILM)	
P03838	2019	2019-Q1	2019-03	Pre-pandemic	Cytology (Pap)	40-44	41	Not performed	Satisfactory	NILM	NILM	Negative (NILM)	Trichomonas

patient_id	year	quarter	month	period	primary_test	age_group	age_years	hpv_result	adequacy	result	bethesda	grade	infection
P03839	2019	2019-Q1	2019-03	Pre-pandemic	Cytology (Pap)	30-34	31	Not performed	Satisfactory	NILM	NILM	Negative (NILM)	
P03840	2019	2019-Q1	2019-03	Pre-pandemic	Cytology (Pap)	35-39	36	Not performed	Satisfactory	Positive	LSIL	Low-grade / equivocal squamous	
P03841	2019	2019-Q1	2019-03	Pre-pandemic	Cytology (Pap)	45-49	45	Not performed	Satisfactory	NILM	NILM	Negative (NILM)	
P03842	2019	2019-Q1	2019-03	Pre-pandemic	Cytology (Pap)	30-34	32	Not performed	Unsatisfactory	Unsatisfactory	Unsatisfactory	Unsatisfactory	
P03843	2019	2019-Q1	2019-03	Pre-pandemic	Cytology (Pap)	30-34	34	Not performed	Satisfactory	NILM	NILM	Negative (NILM)	
P03844	2019	2019-Q1	2019-03	Pre-pandemic	Cytology (Pap)	55-59	57	Not performed	Satisfactory	Positive	ASC-US	Low-grade / equivocal squamous	
P03845	2019	2019-Q1	2019-03	Pre-pandemic	Cytology (Pap)	45-49	49	Not performed	Satisfactory	NILM	NILM	Negative (NILM)	
P03846	2019	2019-Q1	2019-03	Pre-pandemic	Cytology (Pap)	30-34	34	Not performed	Satisfactory	NILM	NILM	Negative (NILM)	
P03847	2019	2019-Q1	2019-03	Pre-pandemic	Cytology (Pap)	40-44	43	Not performed	Satisfactory	NILM	NILM	Negative (NILM)	
P03848	2019	2019-Q1	2019-03	Pre-pandemic	Cytology (Pap)	60-64	64	Not performed	Satisfactory	NILM	NILM	Negative (NILM)	
P03849	2019	2019-Q1	2019-03	Pre-pandemic	Cytology (Pap)	40-44	41	Not performed	Satisfactory	NILM	NILM	Negative (NILM)	
P03850	2019	2019-Q1	2019-03	Pre-pandemic	Cytology (Pap)	55-59	58	Not performed	Satisfactory	NILM	NILM	Negative (NILM)	
P03851	2019	2019-Q1	2019-03	Pre-pandemic	Cytology (Pap)	55-59	57	Not performed	Satisfactory	NILM	NILM	Negative (NILM)	
P03852	2019	2019-Q1	2019-03	Pre-pandemic	Cytology (Pap)	50-54	51	Not performed	Satisfactory	NILM	NILM	Negative (NILM)	
P03853	2019	2019-Q1	2019-03	Pre-pandemic	Cytology (Pap)	40-44	43	Not performed	Satisfactory	NILM	NILM	Negative (NILM)	
P03854	2019	2019-Q1	2019-03	Pre-pandemic	Cytology (Pap)	45-49	45	Not performed	Satisfactory	NILM	NILM	Negative (NILM)	
P03855	2019	2019-Q1	2019-03	Pre-pandemic	Cytology (Pap)	40-44	43	Not performed	Satisfactory	NILM	NILM	Negative (NILM)	
P03856	2019	2019-Q1	2019-03	Pre-pandemic	Cytology (Pap)	50-54	52	Not performed	Satisfactory	Positive	ASC-H	High-grade squamous	
P03857	2019	2019-Q1	2019-03	Pre-pandemic	Cytology (Pap)	40-44	41	Not performed	Satisfactory	Positive	ASC-US	Low-grade / equivocal squamous	
P03858	2019	2019-Q1	2019-03	Pre-pandemic	Cytology (Pap)	30-34	30	Not performed	Satisfactory	NILM	NILM	Negative (NILM)	
P03859	2019	2019-Q1	2019-03	Pre-pandemic	Cytology (Pap)	35-39	37	Not performed	Satisfactory	Positive	LSIL	Low-grade / equivocal squamous	
P03860	2019	2019-Q1	2019-03	Pre-pandemic	Cytology (Pap)	55-59	58	Not performed	Satisfactory	Positive	ASC-H	High-grade squamous	
P03861	2019	2019-Q1	2019-03	Pre-pandemic	Cytology (Pap)	25-29	25	Not performed	Satisfactory	NILM	NILM	Negative (NILM)	
P03862	2019	2019-Q1	2019-03	Pre-pandemic	Cytology (Pap)	30-34	33	Not performed	Satisfactory	NILM	NILM	Negative (NILM)	
P03863	2019	2019-Q1	2019-03	Pre-pandemic	Cytology (Pap)	50-54	51	Not performed	Satisfactory	NILM	NILM	Negative (NILM)	
P03864	2019	2019-Q1	2019-03	Pre-pandemic	Cytology (Pap)	35-39	37	Not performed	Satisfactory	Positive	ASC-US	Low-grade / equivocal squamous	
P03865	2019	2019-Q1	2019-03	Pre-pandemic	Cytology (Pap)	45-49	47	Not performed	Satisfactory	NILM	NILM	Negative (NILM)	
P03866	2019	2019-Q1	2019-03	Pre-pandemic	Cytology (Pap)	50-54	53	Not performed	Satisfactory	NILM	NILM	Negative (NILM)	
P03867	2019	2019-Q1	2019-03	Pre-pandemic	Cytology (Pap)	45-49	49	Not performed	Satisfactory	NILM	NILM	Negative (NILM)	
P03868	2019	2019-Q1	2019-03	Pre-pandemic	Cytology (Pap)	45-49	45	Not performed	Satisfactory	Positive	ASC-US	Low-grade / equivocal squamous	
P03869	2019	2019-Q1	2019-03	Pre-pandemic	Cytology (Pap)	45-49	49	Not performed	Satisfactory	NILM	NILM	Negative (NILM)	
P03870	2019	2019-Q1	2019-03	Pre-pandemic	Cytology (Pap)	50-54	50	Not performed	Satisfactory	Positive	ASC-US	Low-grade / equivocal squamous	
P03871	2019	2019-Q1	2019-03	Pre-pandemic	Cytology (Pap)	45-49	49	Not performed	Satisfactory	NILM	NILM	Negative (NILM)	
P03872	2019	2019-Q1	2019-03	Pre-pandemic	Cytology (Pap)	35-39	39	Not performed	Satisfactory	NILM	NILM	Negative (NILM)	
P03873	2019	2019-Q1	2019-03	Pre-pandemic	Cytology (Pap)	55-59	57	Not performed	Satisfactory	NILM	NILM	Negative (NILM)	
P03874	2019	2019-Q1	2019-03	Pre-pandemic	Cytology (Pap)	35-39	37	Not performed	Satisfactory	Positive	HSIL	High-grade squamous	
P03875	2019	2019-Q1	2019-03	Pre-pandemic	Cytology (Pap)	60-64	63	Not performed	Satisfactory	NILM	NILM	Negative (NILM)	
P03876	2019	2019-Q1	2019-03	Pre-pandemic	Cytology (Pap)	60-64	60	Not performed	Satisfactory	NILM	NILM	Negative (NILM)	
P03877	2019	2019-Q1	2019-03	Pre-pandemic	Cytology (Pap)	45-49	45	Not performed	Satisfactory	NILM	NILM	Negative (NILM)	
P03878	2019	2019-Q1	2019-03	Pre-pandemic	Cytology (Pap)	45-49	45	Not performed	Satisfactory	NILM	NILM	Negative (NILM)	
P03879	2019	2019-Q2	2019-04	Pre-pandemic	Cytology (Pap)	30-34	34	Not performed	Satisfactory	NILM	NILM	Negative (NILM)	
P03880	2019	2019-Q2	2019-04	Pre-pandemic	Cytology (Pap)	55-59	58	Not performed	Satisfactory	NILM	NILM	Negative (NILM)	
P03881	2019	2019-Q2	2019-04	Pre-pandemic	Cytology (Pap)	40-44	43	Not performed	Satisfactory	NILM	NILM	Negative (NILM)	
P03882	2019	2019-Q2	2019-04	Pre-pandemic	Cytology (Pap)	60-64	62	Not performed	Satisfactory	NILM	NILM	Negative (NILM)	
P03883	2019	2019-Q2	2019-04	Pre-pandemic	Cytology (Pap)	60-64	63	Not performed	Satisfactory	NILM	NILM	Negative (NILM)	
P03884	2019	2019-Q2	2019-04	Pre-pandemic	Cytology (Pap)	25-29	26	Not performed	Satisfactory	NILM	NILM	Negative (NILM)	
P03885	2019	2019-Q2	2019-04	Pre-pandemic	Cytology (Pap)	60-64	63	Not performed	Satisfactory	NILM	NILM	Negative (NILM)	
P03886	2019	2019-Q2	2019-04	Pre-pandemic	Cytology (Pap)	40-44	40	Not performed	Satisfactory	NILM	NILM	Negative (NILM)	
P03887	2019	2019-Q2	2019-04	Pre-pandemic	Cytology (Pap)	35-39	36	Not performed	Satisfactory	NILM	NILM	Negative (NILM)	
P03888	2019	2019-Q2	2019-04	Pre-pandemic	Cytology (Pap)	45-49	48	Not performed	Satisfactory	NILM	NILM	Negative (NILM)	
P03889	2019	2019-Q2	2019-04	Pre-pandemic	Cytology (Pap)	40-44	41	Not performed	Satisfactory	NILM	NILM	Negative (NILM)	
P03890	2019	2019-Q2	2019-04	Pre-pandemic	Cytology (Pap)	35-39	39	Not performed	Satisfactory	NILM	NILM	Negative (NILM)	
P03891	2019	2019-Q2	2019-04	Pre-pandemic	Cytology (Pap)	35-39	37	Not performed	Satisfactory	NILM	NILM	Negative (NILM)	
P03892	2019	2019-Q2	2019-04	Pre-pandemic	Cytology (Pap)	35-39	35	Not performed	Satisfactory	Positive	ASC-US	Low-grade / equivocal squamous	
P03893	2019	2019-Q2	2019-04	Pre-pandemic	Cytology (Pap)	30-34	30	Not performed	Satisfactory	NILM	NILM	Negative (NILM)	
P03894	2019	2019-Q2	2019-04	Pre-pandemic	Cytology (Pap)	35-39	39	Not performed	Satisfactory	NILM	NILM	Negative (NILM)	
P03895	2019	2019-Q2	2019-04	Pre-pandemic	Cytology (Pap)	45-49	46	Not performed	Satisfactory	NILM	NILM	Negative (NILM)	
P03896	2019	2019-Q2	2019-04	Pre-pandemic	Cytology (Pap)	35-39	36	Not performed	Satisfactory	NILM	NILM	Negative (NILM)	
P03897	2019	2019-Q2	2019-04	Pre-pandemic	Cytology (Pap)	30-34	33	Not performed	Satisfactory	NILM	NILM	Negative (NILM)	
P03898	2019	2019-Q2	2019-04	Pre-pandemic	Cytology (Pap)	30-34	34	Not performed	Satisfactory	NILM	NILM	Negative (NILM)	
P03899	2019	2019-Q2	2019-04	Pre-pandemic	Cytology (Pap)	30-34	33	Not performed	Satisfactory	NILM	NILM	Negative (NILM)	
P03900	2019	2019-Q2	2019-04	Pre-pandemic	Cytology (Pap)	45-49	46	Not performed	Satisfactory	NILM	NILM	Negative (NILM)	
P03901	2019	2019-Q2	2019-04	Pre-pandemic	Cytology (Pap)	50-54	50	Not performed	Satisfactory	NILM	NILM	Negative (NILM)	
P03902	2019	2019-Q2	2019-04	Pre-pandemic	Cytology (Pap)	30-34	30	Not performed	Satisfactory	NILM	NILM	Negative (NILM)	
P03903	2019	2019-Q2	2019-04	Pre-pandemic	Cytology (Pap)	60-64	62	Not performed	Satisfactory	Positive	ASC-US	Low-grade / equivocal squamous	
P03904	2019	2019-Q2	2019-04	Pre-pandemic	Cytology (Pap)	40-44	42	Not performed	Satisfactory	NILM	NILM	Negative (NILM)	
P03905	2019	2019-Q2	2019-04	Pre-pandemic	Cytology (Pap)	60-64	60	Not performed	Satisfactory	NILM	NILM	Negative (NILM)	
P03906	2019	2019-Q2	2019-04	Pre-pandemic	Cytology (Pap)	35-39	35	Not performed	Satisfactory	NILM	NILM	Negative (NILM)	
P03907	2019	2019-Q2	2019-04	Pre-pandemic	Cytology (Pap)	55-59	56	Not performed	Satisfactory	NILM	NILM	Negative (NILM)	
P03908	2019	2019-Q2	2019-04	Pre-pandemic	Cytology (Pap)	40-44	41	Not performed	Satisfactory	NILM	NILM	Negative (NILM)	
P03909	2019	2019-Q2	2019-04	Pre-pandemic	Cytology (Pap)	50-54	52	Not performed	Satisfactory	NILM	NILM	Negative (NILM)	
P03910	2019	2019-Q2	2019-04	Pre-pandemic	Cytology (Pap)	45-49	48	Not performed	Satisfactory	NILM	NILM	Negative (NILM)	
P03911	2019	2019-Q2	2019-04	Pre-pandemic	Cytology (Pap)	40-44	42	Not performed	Satisfactory	NILM	NILM	Negative (NILM)	
P03912	2019	2019-Q2	2019-04	Pre-pandemic	Cytology (Pap)	40-44	42	Not performed	Satisfactory	Positive	LSIL	Low-grade / equivocal squamous	
P03913	2019	2019-Q2	2019-04	Pre-pandemic	Cytology (Pap)	25-29	29	Not performed	Satisfactory	NILM	NILM	Negative (NILM)	
P03914	2019	2019-Q2	2019-04	Pre-pandemic	Cytology (Pap)	50-54	52	Not performed	Satisfactory	NILM	NILM	Negative (NILM)	Trichomonas
P03915	2019	2019-Q2	2019-04	Pre-pandemic	Cytology (Pap)	60-64	63	Not performed	Satisfactory	NILM	NILM	Negative (NILM)	
P03916	2019	2019-Q2	2019-04	Pre-pandemic	Cytology (Pap)	40-44	41	Not performed	Satisfactory	NILM	NILM	Negative (NILM)	Candida
P03917	2019	2019-Q2	2019-04	Pre-pandemic	Cytology (Pap)	50-54	54	Not performed	Satisfactory	NILM	NILM	Negative (NILM)	
P03918	2019	2019-Q2	2019-04	Pre-pandemic	Cytology (Pap)	45-49	48	Not performed	Satisfactory	Positive	ASC-US	Low-grade / equivocal squamous	

patient_id	year	quarter	month	period	primary_test	age_group	age_years	hpv_result	adequacy	result	bethesda	grade	infection
P03919	2019	2019-Q2	2019-04	Pre-pandemic	Cytology (Pap)	40-44	40	Not performed	Satisfactory	NILM	NILM	Negative (NILM)	
P03920	2019	2019-Q2	2019-04	Pre-pandemic	Cytology (Pap)	50-54	53	Not performed	Satisfactory	NILM	NILM	Negative (NILM)	
P03921	2019	2019-Q2	2019-04	Pre-pandemic	Cytology (Pap)	55-59	57	Not performed	Satisfactory	Positive	ASC-US	Low-grade / equivocal squamous	
P03922	2019	2019-Q2	2019-04	Pre-pandemic	Cytology (Pap)	50-54	51	Not performed	Satisfactory	NILM	NILM	Negative (NILM)	
P03923	2019	2019-Q2	2019-04	Pre-pandemic	Cytology (Pap)	35-39	36	Not performed	Satisfactory	NILM	NILM	Negative (NILM)	
P03924	2019	2019-Q2	2019-04	Pre-pandemic	Cytology (Pap)	25-29	29	Not performed	Satisfactory	NILM	NILM	Negative (NILM)	
P03925	2019	2019-Q2	2019-04	Pre-pandemic	Cytology (Pap)	30-34	30	Not performed	Satisfactory	NILM	NILM	Negative (NILM)	
P03926	2019	2019-Q2	2019-04	Pre-pandemic	Cytology (Pap)	50-54	53	Not performed	Satisfactory	NILM	NILM	Negative (NILM)	
P03927	2019	2019-Q2	2019-04	Pre-pandemic	Cytology (Pap)	35-39	37	Not performed	Satisfactory	NILM	NILM	Negative (NILM)	
P03928	2019	2019-Q2	2019-04	Pre-pandemic	Cytology (Pap)	25-29	25	Not performed	Satisfactory	NILM	NILM	Negative (NILM)	
P03929	2019	2019-Q2	2019-04	Pre-pandemic	Cytology (Pap)	45-49	48	Not performed	Satisfactory	NILM	NILM	Negative (NILM)	
P03930	2019	2019-Q2	2019-04	Pre-pandemic	Cytology (Pap)	40-44	44	Not performed	Satisfactory	Positive	ASC-US	Low-grade / equivocal squamous	
P03931	2019	2019-Q2	2019-04	Pre-pandemic	Cytology (Pap)	60-64	62	Not performed	Satisfactory	NILM	NILM	Negative (NILM)	
P03932	2019	2019-Q2	2019-04	Pre-pandemic	Cytology (Pap)	35-39	36	Not performed	Satisfactory	NILM	NILM	Negative (NILM)	
P03933	2019	2019-Q2	2019-04	Pre-pandemic	Cytology (Pap)	35-39	38	Not performed	Satisfactory	NILM	NILM	Negative (NILM)	
P03934	2019	2019-Q2	2019-04	Pre-pandemic	Cytology (Pap)	35-39	36	Not performed	Satisfactory	NILM	NILM	Negative (NILM)	
P03935	2019	2019-Q2	2019-04	Pre-pandemic	Cytology (Pap)	55-59	57	Not performed	Satisfactory	NILM	NILM	Negative (NILM)	
P03936	2019	2019-Q2	2019-04	Pre-pandemic	Cytology (Pap)	45-49	48	Not performed	Satisfactory	NILM	NILM	Negative (NILM)	
P03937	2019	2019-Q2	2019-04	Pre-pandemic	Cytology (Pap)	60-64	61	Not performed	Satisfactory	NILM	NILM	Negative (NILM)	
P03938	2019	2019-Q2	2019-04	Pre-pandemic	Cytology (Pap)	55-59	56	Not performed	Satisfactory	NILM	NILM	Negative (NILM)	
P03939	2019	2019-Q2	2019-04	Pre-pandemic	Cytology (Pap)	50-54	50	Not performed	Satisfactory	Positive	LSIL	Low-grade / equivocal squamous	
P03940	2019	2019-Q2	2019-04	Pre-pandemic	Cytology (Pap)	35-39	35	Not performed	Satisfactory	NILM	NILM	Negative (NILM)	
P03941	2019	2019-Q2	2019-04	Pre-pandemic	Cytology (Pap)	35-39	36	Not performed	Satisfactory	NILM	NILM	Negative (NILM)	
P03942	2019	2019-Q2	2019-04	Pre-pandemic	Cytology (Pap)	40-44	41	Not performed	Satisfactory	Positive	HSIL	High-grade squamous	
P03943	2019	2019-Q2	2019-04	Pre-pandemic	Cytology (Pap)	55-59	58	Not performed	Satisfactory	NILM	NILM	Negative (NILM)	
P03944	2019	2019-Q2	2019-04	Pre-pandemic	Cytology (Pap)	25-29	29	Not performed	Satisfactory	NILM	NILM	Negative (NILM)	
P03945	2019	2019-Q2	2019-04	Pre-pandemic	Cytology (Pap)	60-64	61	Not performed	Satisfactory	NILM	NILM	Negative (NILM)	
P03946	2019	2019-Q2	2019-04	Pre-pandemic	Cytology (Pap)	45-49	48	Not performed	Satisfactory	NILM	NILM	Negative (NILM)	
P03947	2019	2019-Q2	2019-04	Pre-pandemic	Cytology (Pap)	55-59	55	Not performed	Satisfactory	NILM	NILM	Negative (NILM)	
P03948	2019	2019-Q2	2019-04	Pre-pandemic	Cytology (Pap)	30-34	31	Not performed	Satisfactory	NILM	NILM	Negative (NILM)	
P03949	2019	2019-Q2	2019-04	Pre-pandemic	Cytology (Pap)	30-34	31	Not performed	Satisfactory	NILM	NILM	Negative (NILM)	
P03950	2019	2019-Q2	2019-04	Pre-pandemic	Cytology (Pap)	30-34	30	Not performed	Satisfactory	NILM	NILM	Negative (NILM)	
P03951	2019	2019-Q2	2019-04	Pre-pandemic	Cytology (Pap)	45-49	46	Not performed	Satisfactory	NILM	NILM	Negative (NILM)	
P03952	2019	2019-Q2	2019-04	Pre-pandemic	Cytology (Pap)	55-59	56	Not performed	Satisfactory	NILM	NILM	Negative (NILM)	
P03953	2019	2019-Q2	2019-04	Pre-pandemic	Cytology (Pap)	40-44	42	Not performed	Satisfactory	NILM	NILM	Negative (NILM)	
P03954	2019	2019-Q2	2019-04	Pre-pandemic	Cytology (Pap)	50-54	51	Not performed	Satisfactory	NILM	NILM	Negative (NILM)	
P03955	2019	2019-Q2	2019-04	Pre-pandemic	Cytology (Pap)	35-39	36	Not performed	Satisfactory	Positive	ASC-US	Low-grade / equivocal squamous	
P03956	2019	2019-Q2	2019-04	Pre-pandemic	Cytology (Pap)	25-29	29	Not performed	Satisfactory	NILM	NILM	Negative (NILM)	
P03957	2019	2019-Q2	2019-04	Pre-pandemic	Cytology (Pap)	25-29	28	Not performed	Satisfactory	Positive	ASC-US	Low-grade / equivocal squamous	
P03958	2019	2019-Q2	2019-04	Pre-pandemic	Cytology (Pap)	25-29	26	Not performed	Satisfactory	NILM	NILM	Negative (NILM)	
P03959	2019	2019-Q2	2019-04	Pre-pandemic	Cytology (Pap)	35-39	37	Not performed	Satisfactory	Positive	HSIL	High-grade squamous	
P03960	2019	2019-Q2	2019-04	Pre-pandemic	Cytology (Pap)	60-64	64	Not performed	Satisfactory	NILM	NILM	Negative (NILM)	
P03961	2019	2019-Q2	2019-04	Pre-pandemic	Cytology (Pap)	40-44	41	Not performed	Satisfactory	NILM	NILM	Negative (NILM)	
P03962	2019	2019-Q2	2019-04	Pre-pandemic	Cytology (Pap)	40-44	43	Not performed	Satisfactory	NILM	NILM	Negative (NILM)	
P03963	2019	2019-Q2	2019-04	Pre-pandemic	Cytology (Pap)	45-49	45	Not performed	Satisfactory	NILM	NILM	Negative (NILM)	
P03964	2019	2019-Q2	2019-04	Pre-pandemic	Cytology (Pap)	35-39	39	Not performed	Satisfactory	NILM	NILM	Negative (NILM)	
P03965	2019	2019-Q2	2019-04	Pre-pandemic	Cytology (Pap)	50-54	54	Not performed	Satisfactory	NILM	NILM	Negative (NILM)	
P03966	2019	2019-Q2	2019-04	Pre-pandemic	Cytology (Pap)	35-39	39	Not performed	Satisfactory	NILM	NILM	Negative (NILM)	
P03967	2019	2019-Q2	2019-04	Pre-pandemic	Cytology (Pap)	40-44	42	Not performed	Satisfactory	NILM	NILM	Negative (NILM)	
P03968	2019	2019-Q2	2019-04	Pre-pandemic	Cytology (Pap)	25-29	27	Not performed	Satisfactory	Positive	ASC-US	Low-grade / equivocal squamous	
P03969	2019	2019-Q2	2019-04	Pre-pandemic	Cytology (Pap)	45-49	49	Not performed	Satisfactory	NILM	NILM	Negative (NILM)	
P03970	2019	2019-Q2	2019-04	Pre-pandemic	Cytology (Pap)	30-34	31	Not performed	Satisfactory	NILM	NILM	Negative (NILM)	
P03971	2019	2019-Q2	2019-04	Pre-pandemic	Cytology (Pap)	55-59	56	Not performed	Satisfactory	NILM	NILM	Negative (NILM)	
P03972	2019	2019-Q2	2019-04	Pre-pandemic	Cytology (Pap)	60-64	62	Not performed	Satisfactory	NILM	NILM	Negative (NILM)	
P03973	2019	2019-Q2	2019-04	Pre-pandemic	Cytology (Pap)	35-39	37	Not performed	Satisfactory	NILM	NILM	Negative (NILM)	
P03974	2019	2019-Q2	2019-04	Pre-pandemic	Cytology (Pap)	40-44	41	Not performed	Satisfactory	NILM	NILM	Negative (NILM)	
P03975	2019	2019-Q2	2019-04	Pre-pandemic	Cytology (Pap)	35-39	35	Not performed	Satisfactory	NILM	NILM	Negative (NILM)	
P03976	2019	2019-Q2	2019-04	Pre-pandemic	Cytology (Pap)	45-49	46	Not performed	Unsatisfactory	Unsatisfactory	Unsatisfactory	Unsatisfactory	
P03977	2019	2019-Q2	2019-04	Pre-pandemic	Cytology (Pap)	35-39	39	Not performed	Satisfactory	NILM	NILM	Negative (NILM)	
P03978	2019	2019-Q2	2019-04	Pre-pandemic	Cytology (Pap)	35-39	37	Not performed	Satisfactory	NILM	NILM	Negative (NILM)	
P03979	2019	2019-Q2	2019-04	Pre-pandemic	Cytology (Pap)	30-34	32	Not performed	Satisfactory	NILM	NILM	Negative (NILM)	
P03980	2019	2019-Q2	2019-04	Pre-pandemic	Cytology (Pap)	40-44	42	Not performed	Satisfactory	NILM	NILM	Negative (NILM)	Candida
P03981	2019	2019-Q2	2019-04	Pre-pandemic	Cytology (Pap)	35-39	38	Not performed	Satisfactory	NILM	NILM	Negative (NILM)	
P03982	2019	2019-Q2	2019-04	Pre-pandemic	Cytology (Pap)	50-54	53	Not performed	Satisfactory	Positive	LSIL	Low-grade / equivocal squamous	Trichomonas
P03983	2019	2019-Q2	2019-04	Pre-pandemic	Cytology (Pap)	40-44	41	Not performed	Satisfactory	NILM	NILM	Negative (NILM)	
P03984	2019	2019-Q2	2019-04	Pre-pandemic	Cytology (Pap)	25-29	28	Not performed	Satisfactory	Positive	ASC-H	High-grade squamous	
P03985	2019	2019-Q2	2019-04	Pre-pandemic	Cytology (Pap)	45-49	47	Not performed	Satisfactory	NILM	NILM	Negative (NILM)	
P03986	2019	2019-Q2	2019-04	Pre-pandemic	Cytology (Pap)	35-39	38	Not performed	Satisfactory	Positive	LSIL	Low-grade / equivocal squamous	
P03987	2019	2019-Q2	2019-04	Pre-pandemic	Cytology (Pap)	25-29	28	Not performed	Satisfactory	NILM	NILM	Negative (NILM)	
P03988	2019	2019-Q2	2019-04	Pre-pandemic	Cytology (Pap)	40-44	41	Not performed	Satisfactory	NILM	NILM	Negative (NILM)	Candida
P03989	2019	2019-Q2	2019-04	Pre-pandemic	Cytology (Pap)	30-34	34	Not performed	Satisfactory	Positive	LSIL	Low-grade / equivocal squamous	
P03990	2019	2019-Q2	2019-04	Pre-pandemic	Cytology (Pap)	50-54	53	Not performed	Satisfactory	NILM	NILM	Negative (NILM)	
P03991	2019	2019-Q2	2019-04	Pre-pandemic	Cytology (Pap)	40-44	41	Not performed	Satisfactory	NILM	NILM	Negative (NILM)	
P03992	2019	2019-Q2	2019-04	Pre-pandemic	Cytology (Pap)	45-49	45	Not performed	Satisfactory	NILM	NILM	Negative (NILM)	
P03993	2019	2019-Q2	2019-04	Pre-pandemic	Cytology (Pap)	30-34	33	Not performed	Satisfactory	NILM	NILM	Negative (NILM)	
P03994	2019	2019-Q2	2019-04	Pre-pandemic	Cytology (Pap)	45-49	46	Not performed	Satisfactory	NILM	NILM	Negative (NILM)	
P03995	2019	2019-Q2	2019-04	Pre-pandemic	Cytology (Pap)	45-49	46	Not performed	Satisfactory	NILM	NILM	Negative (NILM)	
P03996	2019	2019-Q2	2019-04	Pre-pandemic	Cytology (Pap)	40-44	42	Not performed	Satisfactory	NILM	NILM	Negative (NILM)	
P03997	2019	2019-Q2	2019-04	Pre-pandemic	Cytology (Pap)	45-49	46	Not performed	Satisfactory	NILM	NILM	Negative (NILM)	
P03998	2019	2019-Q2	2019-04	Pre-pandemic	Cytology (Pap)	30-34	33	Not performed	Satisfactory	NILM	NILM	Negative (NILM)	

patient_id	year	quarter	month	period	primary_test	age_group	age_years	hpv_result	adequacy	result	bethesda	grade	infection
P03999	2019	2019-Q2	2019-04	Pre-pandemic	Cytology (Pap)	35-39	35	Not performed	Satisfactory	NILM	NILM	Negative (NILM)	
P04000	2019	2019-Q2	2019-04	Pre-pandemic	Cytology (Pap)	50-54	51	Not performed	Satisfactory	NILM	NILM	Negative (NILM)	
P04001	2019	2019-Q2	2019-04	Pre-pandemic	Cytology (Pap)	50-54	50	Not performed	Satisfactory	NILM	NILM	Negative (NILM)	
P04002	2019	2019-Q2	2019-04	Pre-pandemic	Cytology (Pap)	45-49	47	Not performed	Satisfactory	NILM	NILM	Negative (NILM)	
P04003	2019	2019-Q2	2019-04	Pre-pandemic	Cytology (Pap)	40-44	42	Not performed	Satisfactory	NILM	NILM	Negative (NILM)	
P04004	2019	2019-Q2	2019-04	Pre-pandemic	Cytology (Pap)	60-64	60	Not performed	Satisfactory	NILM	NILM	Negative (NILM)	
P04005	2019	2019-Q2	2019-04	Pre-pandemic	Cytology (Pap)	45-49	48	Not performed	Satisfactory	NILM	NILM	Negative (NILM)	
P04006	2019	2019-Q2	2019-04	Pre-pandemic	Cytology (Pap)	40-44	44	Not performed	Satisfactory	NILM	NILM	Negative (NILM)	
P04007	2019	2019-Q2	2019-04	Pre-pandemic	Cytology (Pap)	50-54	54	Not performed	Satisfactory	NILM	NILM	Negative (NILM)	
P04008	2019	2019-Q2	2019-04	Pre-pandemic	Cytology (Pap)	25-29	26	Not performed	Satisfactory	NILM	NILM	Negative (NILM)	
P04009	2019	2019-Q2	2019-04	Pre-pandemic	Cytology (Pap)	30-34	33	Not performed	Satisfactory	NILM	NILM	Negative (NILM)	
P04010	2019	2019-Q2	2019-04	Pre-pandemic	Cytology (Pap)	30-34	33	Not performed	Satisfactory	Positive	ASC-US	Low-grade / equivocal squamous	
P04011	2019	2019-Q2	2019-04	Pre-pandemic	Cytology (Pap)	40-44	43	Not performed	Satisfactory	NILM	NILM	Negative (NILM)	
P04012	2019	2019-Q2	2019-04	Pre-pandemic	Cytology (Pap)	30-34	31	Not performed	Satisfactory	Positive	LSIL	Low-grade / equivocal squamous	
P04013	2019	2019-Q2	2019-04	Pre-pandemic	Cytology (Pap)	45-49	45	Not performed	Satisfactory	NILM	NILM	Negative (NILM)	
P04014	2019	2019-Q2	2019-04	Pre-pandemic	Cytology (Pap)	50-54	54	Not performed	Satisfactory	NILM	NILM	Negative (NILM)	
P04015	2019	2019-Q2	2019-04	Pre-pandemic	Cytology (Pap)	30-34	31	Not performed	Satisfactory	Positive	LSIL	Low-grade / equivocal squamous	
P04016	2019	2019-Q2	2019-04	Pre-pandemic	Cytology (Pap)	55-59	57	Not performed	Satisfactory	NILM	NILM	Negative (NILM)	
P04017	2019	2019-Q2	2019-04	Pre-pandemic	Cytology (Pap)	35-39	36	Not performed	Satisfactory	NILM	NILM	Negative (NILM)	
P04018	2019	2019-Q2	2019-04	Pre-pandemic	Cytology (Pap)	30-34	34	Not performed	Satisfactory	NILM	NILM	Negative (NILM)	
P04019	2019	2019-Q2	2019-04	Pre-pandemic	Cytology (Pap)	45-49	48	Not performed	Satisfactory	NILM	NILM	Negative (NILM)	
P04020	2019	2019-Q2	2019-04	Pre-pandemic	Cytology (Pap)	35-39	36	Not performed	Satisfactory	NILM	NILM	Negative (NILM)	
P04021	2019	2019-Q2	2019-04	Pre-pandemic	Cytology (Pap)	40-44	44	Not performed	Satisfactory	NILM	NILM	Negative (NILM)	
P04022	2019	2019-Q2	2019-04	Pre-pandemic	Cytology (Pap)	45-49	48	Not performed	Satisfactory	NILM	NILM	Negative (NILM)	
P04023	2019	2019-Q2	2019-04	Pre-pandemic	Cytology (Pap)	35-39	39	Not performed	Satisfactory	NILM	NILM	Negative (NILM)	
P04024	2019	2019-Q2	2019-04	Pre-pandemic	Cytology (Pap)	60-64	63	Not performed	Satisfactory	NILM	NILM	Negative (NILM)	
P04025	2019	2019-Q2	2019-04	Pre-pandemic	Cytology (Pap)	40-44	44	Not performed	Satisfactory	NILM	NILM	Negative (NILM)	
P04026	2019	2019-Q2	2019-04	Pre-pandemic	Cytology (Pap)	40-44	42	Not performed	Satisfactory	NILM	NILM	Negative (NILM)	
P04027	2019	2019-Q2	2019-04	Pre-pandemic	Cytology (Pap)	40-44	42	Not performed	Satisfactory	NILM	NILM	Negative (NILM)	
P04028	2019	2019-Q2	2019-04	Pre-pandemic	Cytology (Pap)	40-44	42	Not performed	Satisfactory	Positive	LSIL	Low-grade / equivocal squamous	
P04029	2019	2019-Q2	2019-04	Pre-pandemic	Cytology (Pap)	45-49	47	Not performed	Satisfactory	NILM	NILM	Negative (NILM)	
P04030	2019	2019-Q2	2019-04	Pre-pandemic	Cytology (Pap)	40-44	43	Not performed	Satisfactory	NILM	NILM	Negative (NILM)	
P04031	2019	2019-Q2	2019-04	Pre-pandemic	Cytology (Pap)	55-59	59	Not performed	Satisfactory	NILM	NILM	Negative (NILM)	
P04032	2019	2019-Q2	2019-04	Pre-pandemic	Cytology (Pap)	35-39	36	Not performed	Satisfactory	NILM	NILM	Negative (NILM)	
P04033	2019	2019-Q2	2019-04	Pre-pandemic	Cytology (Pap)	40-44	42	Not performed	Satisfactory	NILM	NILM	Negative (NILM)	
P04034	2019	2019-Q2	2019-04	Pre-pandemic	Cytology (Pap)	35-39	39	Not performed	Satisfactory	NILM	NILM	Negative (NILM)	
P04035	2019	2019-Q2	2019-04	Pre-pandemic	Cytology (Pap)	30-34	33	Not performed	Satisfactory	NILM	NILM	Negative (NILM)	
P04036	2019	2019-Q2	2019-04	Pre-pandemic	Cytology (Pap)	55-59	56	Not performed	Satisfactory	NILM	NILM	Negative (NILM)	
P04037	2019	2019-Q2	2019-04	Pre-pandemic	Cytology (Pap)	40-44	42	Not performed	Satisfactory	NILM	NILM	Negative (NILM)	
P04038	2019	2019-Q2	2019-04	Pre-pandemic	Cytology (Pap)	50-54	52	Not performed	Satisfactory	NILM	NILM	Negative (NILM)	
P04039	2019	2019-Q2	2019-04	Pre-pandemic	Cytology (Pap)	40-44	40	Not performed	Satisfactory	NILM	NILM	Negative (NILM)	
P04040	2019	2019-Q2	2019-04	Pre-pandemic	Cytology (Pap)	35-39	35	Not performed	Satisfactory	NILM	NILM	Negative (NILM)	
P04041	2019	2019-Q2	2019-04	Pre-pandemic	Cytology (Pap)	45-49	47	Not performed	Satisfactory	NILM	NILM	Negative (NILM)	
P04042	2019	2019-Q2	2019-04	Pre-pandemic	Cytology (Pap)	40-44	43	Not performed	Satisfactory	Positive	ASC-US	Low-grade / equivocal squamous	
P04043	2019	2019-Q2	2019-04	Pre-pandemic	Cytology (Pap)	40-44	43	Not performed	Satisfactory	Positive	ASC-US	Low-grade / equivocal squamous	
P04044	2019	2019-Q2	2019-04	Pre-pandemic	Cytology (Pap)	35-39	38	Not performed	Satisfactory	NILM	NILM	Negative (NILM)	
P04045	2019	2019-Q2	2019-04	Pre-pandemic	Cytology (Pap)	45-49	45	Not performed	Satisfactory	NILM	NILM	Negative (NILM)	
P04046	2019	2019-Q2	2019-04	Pre-pandemic	Cytology (Pap)	35-39	38	Not performed	Satisfactory	NILM	NILM	Negative (NILM)	
P04047	2019	2019-Q2	2019-04	Pre-pandemic	Cytology (Pap)	35-39	36	Not performed	Satisfactory	NILM	NILM	Negative (NILM)	
P04048	2019	2019-Q2	2019-04	Pre-pandemic	Cytology (Pap)	30-34	30	Not performed	Satisfactory	NILM	NILM	Negative (NILM)	
P04049	2019	2019-Q2	2019-04	Pre-pandemic	Cytology (Pap)	25-29	26	Not performed	Satisfactory	NILM	NILM	Negative (NILM)	
P04050	2019	2019-Q2	2019-04	Pre-pandemic	Cytology (Pap)	40-44	41	Not performed	Satisfactory	NILM	NILM	Negative (NILM)	
P04051	2019	2019-Q2	2019-04	Pre-pandemic	Cytology (Pap)	30-34	30	Not performed	Satisfactory	NILM	NILM	Negative (NILM)	
P04052	2019	2019-Q2	2019-04	Pre-pandemic	Cytology (Pap)	30-34	33	Not performed	Satisfactory	NILM	NILM	Negative (NILM)	
P04053	2019	2019-Q2	2019-04	Pre-pandemic	Cytology (Pap)	35-39	39	Not performed	Unsatisfactory	Unsatisfactory	Unsatisfactory	Unsatisfactory	
P04054	2019	2019-Q2	2019-04	Pre-pandemic	Cytology (Pap)	50-54	52	Not performed	Satisfactory	NILM	NILM	Negative (NILM)	
P04055	2019	2019-Q2	2019-04	Pre-pandemic	Cytology (Pap)	45-49	48	Not performed	Satisfactory	NILM	NILM	Negative (NILM)	
P04056	2019	2019-Q2	2019-04	Pre-pandemic	Cytology (Pap)	35-39	36	Not performed	Satisfactory	NILM	NILM	Negative (NILM)	
P04057	2019	2019-Q2	2019-04	Pre-pandemic	Cytology (Pap)	30-34	31	Not performed	Satisfactory	NILM	NILM	Negative (NILM)	
P04058	2019	2019-Q2	2019-04	Pre-pandemic	Cytology (Pap)	40-44	41	Not performed	Satisfactory	NILM	NILM	Negative (NILM)	
P04059	2019	2019-Q2	2019-04	Pre-pandemic	Cytology (Pap)	60-64	60	Not performed	Satisfactory	NILM	NILM	Negative (NILM)	
P04060	2019	2019-Q2	2019-04	Pre-pandemic	Cytology (Pap)	35-39	37	Not performed	Satisfactory	Positive	ASC-H	High-grade squamous	
P04061	2019	2019-Q2	2019-04	Pre-pandemic	Cytology (Pap)	30-34	30	Not performed	Satisfactory	NILM	NILM	Negative (NILM)	
P04062	2019	2019-Q2	2019-04	Pre-pandemic	Cytology (Pap)	35-39	36	Not performed	Satisfactory	NILM	NILM	Negative (NILM)	
P04063	2019	2019-Q2	2019-04	Pre-pandemic	Cytology (Pap)	25-29	28	Not performed	Satisfactory	NILM	NILM	Negative (NILM)	
P04064	2019	2019-Q2	2019-04	Pre-pandemic	Cytology (Pap)	60-64	62	Not performed	Satisfactory	NILM	NILM	Negative (NILM)	
P04065	2019	2019-Q2	2019-04	Pre-pandemic	Cytology (Pap)	45-49	45	Not performed	Satisfactory	NILM	NILM	Negative (NILM)	
P04066	2019	2019-Q2	2019-04	Pre-pandemic	Cytology (Pap)	35-39	37	Not performed	Satisfactory	NILM	NILM	Negative (NILM)	
P04067	2019	2019-Q2	2019-04	Pre-pandemic	Cytology (Pap)	25-29	25	Not performed	Satisfactory	NILM	NILM	Negative (NILM)	
P04068	2019	2019-Q2	2019-04	Pre-pandemic	Cytology (Pap)	30-34	31	Not performed	Satisfactory	Positive	HSIL	High-grade squamous	
P04069	2019	2019-Q2	2019-04	Pre-pandemic	Cytology (Pap)	30-34	32	Not performed	Satisfactory	NILM	NILM	Negative (NILM)	
P04070	2019	2019-Q2	2019-04	Pre-pandemic	Cytology (Pap)	35-39	37	Not performed	Unsatisfactory	Unsatisfactory	Unsatisfactory	Unsatisfactory	
P04071	2019	2019-Q2	2019-04	Pre-pandemic	Cytology (Pap)	45-49	47	Not performed	Satisfactory	NILM	NILM	Negative (NILM)	
P04072	2019	2019-Q2	2019-04	Pre-pandemic	Cytology (Pap)	35-39	38	Not performed	Satisfactory	NILM	NILM	Negative (NILM)	
P04073	2019	2019-Q2	2019-04	Pre-pandemic	Cytology (Pap)	55-59	57	Not performed	Satisfactory	NILM	NILM	Negative (NILM)	
P04074	2019	2019-Q2	2019-04	Pre-pandemic	Cytology (Pap)	55-59	59	Not performed	Satisfactory	Positive	HSIL	High-grade squamous	
P04075	2019	2019-Q2	2019-04	Pre-pandemic	Cytology (Pap)	50-54	52	Not performed	Satisfactory	NILM	NILM	Negative (NILM)	
P04076	2019	2019-Q2	2019-04	Pre-pandemic	Cytology (Pap)	50-54	50	Not performed	Satisfactory	NILM	NILM	Negative (NILM)	
P04077	2019	2019-Q2	2019-04	Pre-pandemic	Cytology (Pap)	50-54	53	Not performed	Satisfactory	NILM	NILM	Negative (NILM)	
P04078	2019	2019-Q2	2019-04	Pre-pandemic	Cytology (Pap)	30-34	30	Not performed	Satisfactory	NILM	NILM	Negative (NILM)	

patient_id	year	quarter	month	period	primary_test	age_group	age_years	hpv_result	adequacy	result	bethesda	grade	infection
P04079	2019	2019-Q2	2019-04	Pre-pandemic	Cytology (Pap)	50-54	54	Not performed	Satisfactory	NILM	NILM	Negative (NILM)	
P04080	2019	2019-Q2	2019-04	Pre-pandemic	Cytology (Pap)	35-39	37	Not performed	Satisfactory	NILM	NILM	Negative (NILM)	
P04081	2019	2019-Q2	2019-04	Pre-pandemic	Cytology (Pap)	30-34	34	Not performed	Satisfactory	NILM	NILM	Negative (NILM)	
P04082	2019	2019-Q2	2019-04	Pre-pandemic	Cytology (Pap)	25-29	27	Not performed	Satisfactory	NILM	NILM	Negative (NILM)	
P04083	2019	2019-Q2	2019-04	Pre-pandemic	Cytology (Pap)	35-39	38	Not performed	Satisfactory	NILM	NILM	Negative (NILM)	
P04084	2019	2019-Q2	2019-04	Pre-pandemic	Cytology (Pap)	40-44	43	Not performed	Satisfactory	NILM	NILM	Negative (NILM)	
P04085	2019	2019-Q2	2019-04	Pre-pandemic	Cytology (Pap)	60-64	61	Not performed	Satisfactory	NILM	NILM	Negative (NILM)	
P04086	2019	2019-Q2	2019-04	Pre-pandemic	Cytology (Pap)	35-39	38	Not performed	Satisfactory	NILM	NILM	Negative (NILM)	
P04087	2019	2019-Q2	2019-04	Pre-pandemic	Cytology (Pap)	30-34	31	Not performed	Satisfactory	NILM	NILM	Negative (NILM)	
P04088	2019	2019-Q2	2019-04	Pre-pandemic	Cytology (Pap)	35-39	35	Not performed	Satisfactory	NILM	NILM	Negative (NILM)	
P04089	2019	2019-Q2	2019-04	Pre-pandemic	Cytology (Pap)	35-39	38	Not performed	Satisfactory	NILM	NILM	Negative (NILM)	
P04090	2019	2019-Q2	2019-04	Pre-pandemic	Cytology (Pap)	55-59	58	Not performed	Satisfactory	NILM	NILM	Negative (NILM)	
P04091	2019	2019-Q2	2019-04	Pre-pandemic	Cytology (Pap)	35-39	39	Not performed	Satisfactory	NILM	NILM	Negative (NILM)	
P04092	2019	2019-Q2	2019-04	Pre-pandemic	Cytology (Pap)	30-34	32	Not performed	Satisfactory	NILM	NILM	Negative (NILM)	Trichomonas
P04093	2019	2019-Q2	2019-04	Pre-pandemic	Cytology (Pap)	45-49	47	Not performed	Satisfactory	NILM	NILM	Negative (NILM)	
P04094	2019	2019-Q2	2019-04	Pre-pandemic	Cytology (Pap)	40-44	44	Not performed	Unsatisfactory	Unsatisfactory	Unsatisfactory	Unsatisfactory	Gardnerella
P04095	2019	2019-Q2	2019-04	Pre-pandemic	Cytology (Pap)	50-54	53	Not performed	Satisfactory	NILM	NILM	Negative (NILM)	
P04096	2019	2019-Q2	2019-04	Pre-pandemic	Cytology (Pap)	50-54	52	Not performed	Satisfactory	NILM	NILM	Negative (NILM)	
P04097	2019	2019-Q2	2019-04	Pre-pandemic	Cytology (Pap)	50-54	53	Not performed	Satisfactory	NILM	NILM	Negative (NILM)	
P04098	2019	2019-Q2	2019-04	Pre-pandemic	Cytology (Pap)	30-34	32	Not performed	Satisfactory	Positive	ASC-US	Low-grade / equivocal squamous	
P04099	2019	2019-Q2	2019-04	Pre-pandemic	Cytology (Pap)	60-64	63	Not performed	Unsatisfactory	Unsatisfactory	Unsatisfactory	Unsatisfactory	
P04100	2019	2019-Q2	2019-04	Pre-pandemic	Cytology (Pap)	30-34	32	Not performed	Satisfactory	Positive	ASC-H	High-grade squamous	
P04101	2019	2019-Q2	2019-04	Pre-pandemic	Cytology (Pap)	60-64	62	Not performed	Satisfactory	NILM	NILM	Negative (NILM)	Candida
P04102	2019	2019-Q2	2019-04	Pre-pandemic	Cytology (Pap)	35-39	36	Not performed	Satisfactory	NILM	NILM	Negative (NILM)	
P04103	2019	2019-Q2	2019-04	Pre-pandemic	Cytology (Pap)	35-39	36	Not performed	Satisfactory	Positive	LSIL	Low-grade / equivocal squamous	
P04104	2019	2019-Q2	2019-04	Pre-pandemic	Cytology (Pap)	60-64	63	Not performed	Satisfactory	NILM	NILM	Negative (NILM)	
P04105	2019	2019-Q2	2019-04	Pre-pandemic	Cytology (Pap)	40-44	41	Not performed	Satisfactory	NILM	NILM	Negative (NILM)	
P04106	2019	2019-Q2	2019-04	Pre-pandemic	Cytology (Pap)	50-54	51	Not performed	Satisfactory	NILM	NILM	Negative (NILM)	
P04107	2019	2019-Q2	2019-04	Pre-pandemic	Cytology (Pap)	50-54	53	Not performed	Satisfactory	Positive	ASC-US	Low-grade / equivocal squamous	
P04108	2019	2019-Q2	2019-04	Pre-pandemic	Cytology (Pap)	30-34	33	Not performed	Satisfactory	NILM	NILM	Negative (NILM)	
P04109	2019	2019-Q2	2019-04	Pre-pandemic	Cytology (Pap)	50-54	52	Not performed	Satisfactory	NILM	NILM	Negative (NILM)	
P04110	2019	2019-Q2	2019-04	Pre-pandemic	Cytology (Pap)	50-54	51	Not performed	Satisfactory	NILM	NILM	Negative (NILM)	
P04111	2019	2019-Q2	2019-04	Pre-pandemic	Cytology (Pap)	60-64	61	Not performed	Satisfactory	NILM	NILM	Negative (NILM)	
P04112	2019	2019-Q2	2019-04	Pre-pandemic	Cytology (Pap)	60-64	64	Not performed	Satisfactory	NILM	NILM	Negative (NILM)	
P04113	2019	2019-Q2	2019-04	Pre-pandemic	Cytology (Pap)	45-49	49	Not performed	Satisfactory	NILM	NILM	Negative (NILM)	
P04114	2019	2019-Q2	2019-04	Pre-pandemic	Cytology (Pap)	25-29	27	Not performed	Satisfactory	NILM	NILM	Negative (NILM)	
P04115	2019	2019-Q2	2019-04	Pre-pandemic	Cytology (Pap)	40-44	43	Not performed	Satisfactory	NILM	NILM	Negative (NILM)	
P04116	2019	2019-Q2	2019-04	Pre-pandemic	Cytology (Pap)	40-44	42	Not performed	Satisfactory	NILM	NILM	Negative (NILM)	
P04117	2019	2019-Q2	2019-04	Pre-pandemic	Cytology (Pap)	35-39	36	Not performed	Satisfactory	NILM	NILM	Negative (NILM)	
P04118	2019	2019-Q2	2019-05	Pre-pandemic	Cytology (Pap)	45-49	45	Not performed	Satisfactory	NILM	NILM	Negative (NILM)	
P04119	2019	2019-Q2	2019-05	Pre-pandemic	Cytology (Pap)	30-34	30	Not performed	Satisfactory	NILM	NILM	Negative (NILM)	
P04120	2019	2019-Q2	2019-05	Pre-pandemic	Cytology (Pap)	25-29	27	Not performed	Satisfactory	NILM	NILM	Negative (NILM)	
P04121	2019	2019-Q2	2019-05	Pre-pandemic	Cytology (Pap)	50-54	50	Not performed	Satisfactory	NILM	NILM	Negative (NILM)	
P04122	2019	2019-Q2	2019-05	Pre-pandemic	Cytology (Pap)	55-59	57	Not performed	Satisfactory	Positive	AGC	Glandular	
P04123	2019	2019-Q2	2019-05	Pre-pandemic	Cytology (Pap)	55-59	55	Not performed	Satisfactory	NILM	NILM	Negative (NILM)	
P04124	2019	2019-Q2	2019-05	Pre-pandemic	Cytology (Pap)	50-54	51	Not performed	Satisfactory	NILM	NILM	Negative (NILM)	
P04125	2019	2019-Q2	2019-05	Pre-pandemic	Cytology (Pap)	30-34	34	Not performed	Satisfactory	NILM	NILM	Negative (NILM)	
P04126	2019	2019-Q2	2019-05	Pre-pandemic	Cytology (Pap)	55-59	58	Not performed	Satisfactory	Positive	HSIL	High-grade squamous	
P04127	2019	2019-Q2	2019-05	Pre-pandemic	Cytology (Pap)	25-29	25	Not performed	Satisfactory	Positive	LSIL	Low-grade / equivocal squamous	
P04128	2019	2019-Q2	2019-05	Pre-pandemic	Cytology (Pap)	40-44	44	Not performed	Satisfactory	NILM	NILM	Negative (NILM)	Gardnerella
P04129	2019	2019-Q2	2019-05	Pre-pandemic	Cytology (Pap)	40-44	41	Not performed	Satisfactory	NILM	NILM	Negative (NILM)	
P04130	2019	2019-Q2	2019-05	Pre-pandemic	Cytology (Pap)	25-29	26	Not performed	Satisfactory	NILM	NILM	Negative (NILM)	
P04131	2019	2019-Q2	2019-05	Pre-pandemic	Cytology (Pap)	60-64	60	Not performed	Satisfactory	NILM	NILM	Negative (NILM)	
P04132	2019	2019-Q2	2019-05	Pre-pandemic	Cytology (Pap)	45-49	45	Not performed	Satisfactory	NILM	NILM	Negative (NILM)	
P04133	2019	2019-Q2	2019-05	Pre-pandemic	Cytology (Pap)	60-64	61	Not performed	Satisfactory	NILM	NILM	Negative (NILM)	
P04134	2019	2019-Q2	2019-05	Pre-pandemic	Cytology (Pap)	30-34	31	Not performed	Satisfactory	NILM	NILM	Negative (NILM)	
P04135	2019	2019-Q2	2019-05	Pre-pandemic	Cytology (Pap)	45-49	48	Not performed	Unsatisfactory	Unsatisfactory	Unsatisfactory	Unsatisfactory	
P04136	2019	2019-Q2	2019-05	Pre-pandemic	Cytology (Pap)	35-39	35	Not performed	Satisfactory	NILM	NILM	Negative (NILM)	
P04137	2019	2019-Q2	2019-05	Pre-pandemic	Cytology (Pap)	55-59	55	Not performed	Satisfactory	NILM	NILM	Negative (NILM)	
P04138	2019	2019-Q2	2019-05	Pre-pandemic	Cytology (Pap)	40-44	43	Not performed	Satisfactory	NILM	NILM	Negative (NILM)	
P04139	2019	2019-Q2	2019-05	Pre-pandemic	Cytology (Pap)	50-54	50	Not performed	Satisfactory	Positive	ASC-US	Low-grade / equivocal squamous	
P04140	2019	2019-Q2	2019-05	Pre-pandemic	Cytology (Pap)	40-44	42	Not performed	Satisfactory	NILM	NILM	Negative (NILM)	
P04141	2019	2019-Q2	2019-05	Pre-pandemic	Cytology (Pap)	30-34	34	Not performed	Satisfactory	Positive	LSIL	Low-grade / equivocal squamous	
P04142	2019	2019-Q2	2019-05	Pre-pandemic	Cytology (Pap)	45-49	48	Not performed	Satisfactory	NILM	NILM	Negative (NILM)	
P04143	2019	2019-Q2	2019-05	Pre-pandemic	Cytology (Pap)	50-54	54	Not performed	Satisfactory	Positive	AGC	Glandular	
P04144	2019	2019-Q2	2019-05	Pre-pandemic	Cytology (Pap)	30-34	32	Not performed	Satisfactory	NILM	NILM	Negative (NILM)	
P04145	2019	2019-Q2	2019-05	Pre-pandemic	Cytology (Pap)	40-44	40	Not performed	Satisfactory	NILM	NILM	Negative (NILM)	
P04146	2019	2019-Q2	2019-05	Pre-pandemic	Cytology (Pap)	45-49	46	Not performed	Satisfactory	NILM	NILM	Negative (NILM)	
P04147	2019	2019-Q2	2019-05	Pre-pandemic	Cytology (Pap)	30-34	31	Not performed	Satisfactory	NILM	NILM	Negative (NILM)	
P04148	2019	2019-Q2	2019-05	Pre-pandemic	Cytology (Pap)	60-64	63	Not performed	Satisfactory	NILM	NILM	Negative (NILM)	
P04149	2019	2019-Q2	2019-05	Pre-pandemic	Cytology (Pap)	40-44	42	Not performed	Satisfactory	NILM	NILM	Negative (NILM)	
P04150	2019	2019-Q2	2019-05	Pre-pandemic	Cytology (Pap)	30-34	30	Not performed	Satisfactory	NILM	NILM	Negative (NILM)	
P04151	2019	2019-Q2	2019-05	Pre-pandemic	Cytology (Pap)	30-34	31	Not performed	Satisfactory	Positive	LSIL	Low-grade / equivocal squamous	
P04152	2019	2019-Q2	2019-05	Pre-pandemic	Cytology (Pap)	45-49	45	Not performed	Unsatisfactory	Unsatisfactory	Unsatisfactory	Unsatisfactory	
P04153	2019	2019-Q2	2019-05	Pre-pandemic	Cytology (Pap)	45-49	47	Not performed	Satisfactory	NILM	NILM	Negative (NILM)	
P04154	2019	2019-Q2	2019-05	Pre-pandemic	Cytology (Pap)	45-49	46	Not performed	Satisfactory	NILM	NILM	Negative (NILM)	
P04155	2019	2019-Q2	2019-05	Pre-pandemic	Cytology (Pap)	35-39	37	Not performed	Satisfactory	NILM	NILM	Negative (NILM)	
P04156	2019	2019-Q2	2019-05	Pre-pandemic	Cytology (Pap)	40-44	43	Not performed	Satisfactory	NILM	NILM	Negative (NILM)	
P04157	2019	2019-Q2	2019-05	Pre-pandemic	Cytology (Pap)	55-59	58	Not performed	Satisfactory	NILM	NILM	Negative (NILM)	
P04158	2019	2019-Q2	2019-05	Pre-pandemic	Cytology (Pap)	50-54	53	Not performed	Satisfactory	NILM	NILM	Negative (NILM)	

patient_id	year	quarter	month	period	primary_test	age_group	age_years	hpv_result	adequacy	result	bethesda	grade	infection
P04159	2019	2019-Q2	2019-05	Pre-pandemic	Cytology (Pap)	40-44	44	Not performed	Satisfactory	NILM	NILM	Negative (NILM)	
P04160	2019	2019-Q2	2019-05	Pre-pandemic	Cytology (Pap)	45-49	49	Not performed	Satisfactory	NILM	NILM	Negative (NILM)	
P04161	2019	2019-Q2	2019-05	Pre-pandemic	Cytology (Pap)	55-59	55	Not performed	Satisfactory	Positive	ASC-US	Low-grade / equivocal squamous	
P04162	2019	2019-Q2	2019-05	Pre-pandemic	Cytology (Pap)	25-29	28	Not performed	Satisfactory	NILM	NILM	Negative (NILM)	
P04163	2019	2019-Q2	2019-05	Pre-pandemic	Cytology (Pap)	35-39	38	Not performed	Satisfactory	NILM	NILM	Negative (NILM)	
P04164	2019	2019-Q2	2019-05	Pre-pandemic	Cytology (Pap)	45-49	49	Not performed	Satisfactory	NILM	NILM	Negative (NILM)	
P04165	2019	2019-Q2	2019-05	Pre-pandemic	Cytology (Pap)	25-29	26	Not performed	Satisfactory	NILM	NILM	Negative (NILM)	
P04166	2019	2019-Q2	2019-05	Pre-pandemic	Cytology (Pap)	40-44	43	Not performed	Satisfactory	Positive	HSIL	High-grade squamous	
P04167	2019	2019-Q2	2019-05	Pre-pandemic	Cytology (Pap)	35-39	38	Not performed	Satisfactory	NILM	NILM	Negative (NILM)	
P04168	2019	2019-Q2	2019-05	Pre-pandemic	Cytology (Pap)	60-64	61	Not performed	Satisfactory	NILM	NILM	Negative (NILM)	
P04169	2019	2019-Q2	2019-05	Pre-pandemic	Cytology (Pap)	30-34	32	Not performed	Satisfactory	NILM	NILM	Negative (NILM)	
P04170	2019	2019-Q2	2019-05	Pre-pandemic	Cytology (Pap)	30-34	31	Not performed	Satisfactory	NILM	NILM	Negative (NILM)	
P04171	2019	2019-Q2	2019-05	Pre-pandemic	Cytology (Pap)	35-39	37	Not performed	Satisfactory	Positive	ASC-US	Low-grade / equivocal squamous	
P04172	2019	2019-Q2	2019-05	Pre-pandemic	Cytology (Pap)	45-49	49	Not performed	Satisfactory	NILM	NILM	Negative (NILM)	
P04173	2019	2019-Q2	2019-05	Pre-pandemic	Cytology (Pap)	45-49	47	Not performed	Satisfactory	NILM	NILM	Negative (NILM)	
P04174	2019	2019-Q2	2019-05	Pre-pandemic	Cytology (Pap)	40-44	42	Not performed	Satisfactory	NILM	NILM	Negative (NILM)	
P04175	2019	2019-Q2	2019-05	Pre-pandemic	Cytology (Pap)	60-64	62	Not performed	Satisfactory	NILM	NILM	Negative (NILM)	
P04176	2019	2019-Q2	2019-05	Pre-pandemic	Cytology (Pap)	40-44	41	Not performed	Satisfactory	NILM	NILM	Negative (NILM)	
P04177	2019	2019-Q2	2019-05	Pre-pandemic	Cytology (Pap)	30-34	34	Not performed	Satisfactory	NILM	NILM	Negative (NILM)	
P04178	2019	2019-Q2	2019-05	Pre-pandemic	Cytology (Pap)	35-39	39	Not performed	Satisfactory	NILM	NILM	Negative (NILM)	
P04179	2019	2019-Q2	2019-05	Pre-pandemic	Cytology (Pap)	30-34	33	Not performed	Satisfactory	NILM	NILM	Negative (NILM)	
P04180	2019	2019-Q2	2019-05	Pre-pandemic	Cytology (Pap)	55-59	58	Not performed	Satisfactory	Positive	ASC-US	Low-grade / equivocal squamous	
P04181	2019	2019-Q2	2019-05	Pre-pandemic	Cytology (Pap)	60-64	61	Not performed	Satisfactory	NILM	NILM	Negative (NILM)	
P04182	2019	2019-Q2	2019-05	Pre-pandemic	Cytology (Pap)	35-39	35	Not performed	Satisfactory	Positive	AGC	Glandular	
P04183	2019	2019-Q2	2019-05	Pre-pandemic	Cytology (Pap)	45-49	47	Not performed	Satisfactory	NILM	NILM	Negative (NILM)	
P04184	2019	2019-Q2	2019-05	Pre-pandemic	Cytology (Pap)	30-34	34	Not performed	Satisfactory	NILM	NILM	Negative (NILM)	
P04185	2019	2019-Q2	2019-05	Pre-pandemic	Cytology (Pap)	40-44	40	Not performed	Satisfactory	NILM	NILM	Negative (NILM)	
P04186	2019	2019-Q2	2019-05	Pre-pandemic	Cytology (Pap)	30-34	32	Not performed	Satisfactory	NILM	NILM	Negative (NILM)	
P04187	2019	2019-Q2	2019-05	Pre-pandemic	Cytology (Pap)	55-59	57	Not performed	Satisfactory	Positive	ASC-US	Low-grade / equivocal squamous	
P04188	2019	2019-Q2	2019-05	Pre-pandemic	Cytology (Pap)	50-54	51	Not performed	Satisfactory	NILM	NILM	Negative (NILM)	
P04189	2019	2019-Q2	2019-05	Pre-pandemic	Cytology (Pap)	50-54	52	Not performed	Satisfactory	NILM	NILM	Negative (NILM)	
P04190	2019	2019-Q2	2019-05	Pre-pandemic	Cytology (Pap)	40-44	43	Not performed	Satisfactory	NILM	NILM	Negative (NILM)	
P04191	2019	2019-Q2	2019-05	Pre-pandemic	Cytology (Pap)	25-29	26	Not performed	Satisfactory	Positive	ASC-US	Low-grade / equivocal squamous	
P04192	2019	2019-Q2	2019-05	Pre-pandemic	Cytology (Pap)	55-59	59	Not performed	Satisfactory	Positive	ASC-US	Low-grade / equivocal squamous	
P04193	2019	2019-Q2	2019-05	Pre-pandemic	Cytology (Pap)	35-39	39	Not performed	Satisfactory	NILM	NILM	Negative (NILM)	
P04194	2019	2019-Q2	2019-05	Pre-pandemic	Cytology (Pap)	35-39	36	Not performed	Satisfactory	NILM	NILM	Negative (NILM)	
P04195	2019	2019-Q2	2019-05	Pre-pandemic	Cytology (Pap)	50-54	51	Not performed	Satisfactory	NILM	NILM	Negative (NILM)	
P04196	2019	2019-Q2	2019-05	Pre-pandemic	Cytology (Pap)	40-44	42	Not performed	Satisfactory	NILM	NILM	Negative (NILM)	
P04197	2019	2019-Q2	2019-05	Pre-pandemic	Cytology (Pap)	50-54	51	Not performed	Satisfactory	NILM	NILM	Negative (NILM)	
P04198	2019	2019-Q2	2019-05	Pre-pandemic	Cytology (Pap)	35-39	35	Not performed	Satisfactory	NILM	NILM	Negative (NILM)	
P04199	2019	2019-Q2	2019-05	Pre-pandemic	Cytology (Pap)	35-39	38	Not performed	Satisfactory	NILM	NILM	Negative (NILM)	
P04200	2019	2019-Q2	2019-05	Pre-pandemic	Cytology (Pap)	25-29	27	Not performed	Satisfactory	NILM	NILM	Negative (NILM)	
P04201	2019	2019-Q2	2019-05	Pre-pandemic	Cytology (Pap)	55-59	59	Not performed	Satisfactory	NILM	NILM	Negative (NILM)	
P04202	2019	2019-Q2	2019-05	Pre-pandemic	Cytology (Pap)	50-54	51	Not performed	Satisfactory	Positive	LSIL	Low-grade / equivocal squamous	
P04203	2019	2019-Q2	2019-05	Pre-pandemic	Cytology (Pap)	35-39	37	Not performed	Satisfactory	NILM	NILM	Negative (NILM)	
P04204	2019	2019-Q2	2019-05	Pre-pandemic	Cytology (Pap)	40-44	40	Not performed	Unsatisfactory	Unsatisfactory	Unsatisfactory	Unsatisfactory	
P04205	2019	2019-Q2	2019-05	Pre-pandemic	Cytology (Pap)	35-39	36	Not performed	Satisfactory	NILM	NILM	Negative (NILM)	
P04206	2019	2019-Q2	2019-05	Pre-pandemic	Cytology (Pap)	35-39	36	Not performed	Satisfactory	Positive	ASC-H	High-grade squamous	
P04207	2019	2019-Q2	2019-05	Pre-pandemic	Cytology (Pap)	35-39	36	Not performed	Satisfactory	NILM	NILM	Negative (NILM)	
P04208	2019	2019-Q2	2019-05	Pre-pandemic	Cytology (Pap)	50-54	53	Not performed	Satisfactory	NILM	NILM	Negative (NILM)	
P04209	2019	2019-Q2	2019-05	Pre-pandemic	Cytology (Pap)	40-44	41	Not performed	Satisfactory	NILM	NILM	Negative (NILM)	
P04210	2019	2019-Q2	2019-05	Pre-pandemic	Cytology (Pap)	55-59	59	Not performed	Satisfactory	NILM	NILM	Negative (NILM)	
P04211	2019	2019-Q2	2019-05	Pre-pandemic	Cytology (Pap)	40-44	42	Not performed	Satisfactory	NILM	NILM	Negative (NILM)	
P04212	2019	2019-Q2	2019-05	Pre-pandemic	Cytology (Pap)	45-49	46	Not performed	Satisfactory	NILM	NILM	Negative (NILM)	
P04213	2019	2019-Q2	2019-05	Pre-pandemic	Cytology (Pap)	45-49	46	Not performed	Satisfactory	NILM	NILM	Negative (NILM)	
P04214	2019	2019-Q2	2019-05	Pre-pandemic	Cytology (Pap)	40-44	42	Not performed	Satisfactory	NILM	NILM	Negative (NILM)	
P04215	2019	2019-Q2	2019-05	Pre-pandemic	Cytology (Pap)	60-64	61	Not performed	Satisfactory	NILM	NILM	Negative (NILM)	
P04216	2019	2019-Q2	2019-05	Pre-pandemic	Cytology (Pap)	30-34	32	Not performed	Satisfactory	NILM	NILM	Negative (NILM)	
P04217	2019	2019-Q2	2019-05	Pre-pandemic	Cytology (Pap)	45-49	49	Not performed	Satisfactory	Positive	ASC-H	High-grade squamous	
P04218	2019	2019-Q2	2019-05	Pre-pandemic	Cytology (Pap)	55-59	59	Not performed	Satisfactory	NILM	NILM	Negative (NILM)	
P04219	2019	2019-Q2	2019-05	Pre-pandemic	Cytology (Pap)	40-44	41	Not performed	Satisfactory	NILM	NILM	Negative (NILM)	
P04220	2019	2019-Q2	2019-05	Pre-pandemic	Cytology (Pap)	50-54	52	Not performed	Satisfactory	NILM	NILM	Negative (NILM)	
P04221	2019	2019-Q2	2019-05	Pre-pandemic	Cytology (Pap)	35-39	37	Not performed	Satisfactory	NILM	NILM	Negative (NILM)	
P04222	2019	2019-Q2	2019-05	Pre-pandemic	Cytology (Pap)	50-54	54	Not performed	Satisfactory	NILM	NILM	Negative (NILM)	
P04223	2019	2019-Q2	2019-05	Pre-pandemic	Cytology (Pap)	40-44	41	Not performed	Satisfactory	NILM	NILM	Negative (NILM)	
P04224	2019	2019-Q2	2019-05	Pre-pandemic	Cytology (Pap)	40-44	42	Not performed	Satisfactory	NILM	NILM	Negative (NILM)	
P04225	2019	2019-Q2	2019-05	Pre-pandemic	Cytology (Pap)	40-44	42	Not performed	Satisfactory	NILM	NILM	Negative (NILM)	
P04226	2019	2019-Q2	2019-05	Pre-pandemic	Cytology (Pap)	25-29	28	Not performed	Satisfactory	NILM	NILM	Negative (NILM)	
P04227	2019	2019-Q2	2019-05	Pre-pandemic	Cytology (Pap)	35-39	38	Not performed	Satisfactory	NILM	NILM	Negative (NILM)	
P04228	2019	2019-Q2	2019-05	Pre-pandemic	Cytology (Pap)	50-54	54	Not performed	Satisfactory	NILM	NILM	Negative (NILM)	
P04229	2019	2019-Q2	2019-05	Pre-pandemic	Cytology (Pap)	35-39	38	Not performed	Unsatisfactory	Unsatisfactory	Unsatisfactory	Unsatisfactory	
P04230	2019	2019-Q2	2019-05	Pre-pandemic	Cytology (Pap)	40-44	40	Not performed	Satisfactory	NILM	NILM	Negative (NILM)	
P04231	2019	2019-Q2	2019-05	Pre-pandemic	Cytology (Pap)	55-59	57	Not performed	Satisfactory	NILM	NILM	Negative (NILM)	
P04232	2019	2019-Q2	2019-05	Pre-pandemic	Cytology (Pap)	35-39	35	Not performed	Satisfactory	NILM	NILM	Negative (NILM)	
P04233	2019	2019-Q2	2019-05	Pre-pandemic	Cytology (Pap)	50-54	53	Not performed	Satisfactory	NILM	NILM	Negative (NILM)	
P04234	2019	2019-Q2	2019-05	Pre-pandemic	Cytology (Pap)	50-54	53	Not performed	Satisfactory	Positive	ASC-US	Low-grade / equivocal squamous	
P04235	2019	2019-Q2	2019-05	Pre-pandemic	Cytology (Pap)	35-39	36	Not performed	Satisfactory	NILM	NILM	Negative (NILM)	
P04236	2019	2019-Q2	2019-05	Pre-pandemic	Cytology (Pap)	45-49	47	Not performed	Satisfactory	NILM	NILM	Negative (NILM)	
P04237	2019	2019-Q2	2019-05	Pre-pandemic	Cytology (Pap)	40-44	41	Not performed	Satisfactory	Positive	LSIL	Low-grade / equivocal squamous	
P04238	2019	2019-Q2	2019-05	Pre-pandemic	Cytology (Pap)	60-64	60	Not performed	Satisfactory	NILM	NILM	Negative (NILM)	

patient_id	year	quarter	month	period	primary_test	age_group	age_years	hpv_result	adequacy	result	bethesda	grade	infection
P04239	2019	2019-Q2	2019-05	Pre-pandemic	Cytology (Pap)	45-49	48	Not performed	Satisfactory	NILM	NILM	Negative (NILM)	
P04240	2019	2019-Q2	2019-05	Pre-pandemic	Cytology (Pap)	55-59	59	Not performed	Satisfactory	NILM	NILM	Negative (NILM)	
P04241	2019	2019-Q2	2019-05	Pre-pandemic	Cytology (Pap)	35-39	35	Not performed	Satisfactory	NILM	NILM	Negative (NILM)	
P04242	2019	2019-Q2	2019-05	Pre-pandemic	Cytology (Pap)	60-64	62	Not performed	Satisfactory	NILM	NILM	Negative (NILM)	
P04243	2019	2019-Q2	2019-05	Pre-pandemic	Cytology (Pap)	60-64	61	Not performed	Satisfactory	NILM	NILM	Negative (NILM)	
P04244	2019	2019-Q2	2019-05	Pre-pandemic	Cytology (Pap)	45-49	45	Not performed	Satisfactory	NILM	NILM	Negative (NILM)	
P04245	2019	2019-Q2	2019-05	Pre-pandemic	Cytology (Pap)	55-59	57	Not performed	Satisfactory	NILM	NILM	Negative (NILM)	
P04246	2019	2019-Q2	2019-05	Pre-pandemic	Cytology (Pap)	30-34	32	Not performed	Satisfactory	NILM	NILM	Negative (NILM)	
P04247	2019	2019-Q2	2019-05	Pre-pandemic	Cytology (Pap)	35-39	37	Not performed	Unsatisfactory	Unsatisfactory	Unsatisfactory	Unsatisfactory	
P04248	2019	2019-Q2	2019-05	Pre-pandemic	Cytology (Pap)	40-44	41	Not performed	Satisfactory	NILM	NILM	Negative (NILM)	
P04249	2019	2019-Q2	2019-05	Pre-pandemic	Cytology (Pap)	45-49	48	Not performed	Satisfactory	Positive	HSIL	High-grade squamous	
P04250	2019	2019-Q2	2019-05	Pre-pandemic	Cytology (Pap)	45-49	46	Not performed	Satisfactory	NILM	NILM	Negative (NILM)	
P04251	2019	2019-Q2	2019-05	Pre-pandemic	Cytology (Pap)	40-44	42	Not performed	Satisfactory	NILM	NILM	Negative (NILM)	
P04252	2019	2019-Q2	2019-05	Pre-pandemic	Cytology (Pap)	30-34	33	Not performed	Satisfactory	NILM	NILM	Negative (NILM)	
P04253	2019	2019-Q2	2019-05	Pre-pandemic	Cytology (Pap)	55-59	59	Not performed	Satisfactory	Positive	LSIL	Low-grade / equivocal squamous	
P04254	2019	2019-Q2	2019-05	Pre-pandemic	Cytology (Pap)	35-39	39	Not performed	Satisfactory	NILM	NILM	Negative (NILM)	
P04255	2019	2019-Q2	2019-05	Pre-pandemic	Cytology (Pap)	50-54	51	Not performed	Satisfactory	NILM	NILM	Negative (NILM)	
P04256	2019	2019-Q2	2019-05	Pre-pandemic	Cytology (Pap)	60-64	63	Not performed	Satisfactory	Positive	LSIL	Low-grade / equivocal squamous	
P04257	2019	2019-Q2	2019-05	Pre-pandemic	Cytology (Pap)	40-44	41	Not performed	Satisfactory	NILM	NILM	Negative (NILM)	
P04258	2019	2019-Q2	2019-05	Pre-pandemic	Cytology (Pap)	45-49	45	Not performed	Satisfactory	Positive	ASC-US	Low-grade / equivocal squamous	
P04259	2019	2019-Q2	2019-05	Pre-pandemic	Cytology (Pap)	25-29	27	Not performed	Satisfactory	NILM	NILM	Negative (NILM)	
P04260	2019	2019-Q2	2019-05	Pre-pandemic	Cytology (Pap)	35-39	36	Not performed	Satisfactory	NILM	NILM	Negative (NILM)	
P04261	2019	2019-Q2	2019-05	Pre-pandemic	Cytology (Pap)	45-49	49	Not performed	Satisfactory	Positive	ASC-US	Low-grade / equivocal squamous	
P04262	2019	2019-Q2	2019-05	Pre-pandemic	Cytology (Pap)	35-39	37	Not performed	Satisfactory	NILM	NILM	Negative (NILM)	
P04263	2019	2019-Q2	2019-05	Pre-pandemic	Cytology (Pap)	45-49	49	Not performed	Satisfactory	NILM	NILM	Negative (NILM)	
P04264	2019	2019-Q2	2019-05	Pre-pandemic	Cytology (Pap)	40-44	41	Not performed	Unsatisfactory	Unsatisfactory	Unsatisfactory	Unsatisfactory	
P04265	2019	2019-Q2	2019-05	Pre-pandemic	Cytology (Pap)	30-34	31	Not performed	Satisfactory	NILM	NILM	Negative (NILM)	
P04266	2019	2019-Q2	2019-05	Pre-pandemic	Cytology (Pap)	40-44	44	Not performed	Satisfactory	NILM	NILM	Negative (NILM)	
P04267	2019	2019-Q2	2019-05	Pre-pandemic	Cytology (Pap)	55-59	55	Not performed	Satisfactory	NILM	NILM	Negative (NILM)	
P04268	2019	2019-Q2	2019-05	Pre-pandemic	Cytology (Pap)	30-34	31	Not performed	Satisfactory	NILM	NILM	Negative (NILM)	
P04269	2019	2019-Q2	2019-05	Pre-pandemic	Cytology (Pap)	35-39	36	Not performed	Satisfactory	NILM	NILM	Negative (NILM)	
P04270	2019	2019-Q2	2019-05	Pre-pandemic	Cytology (Pap)	45-49	46	Not performed	Satisfactory	NILM	NILM	Negative (NILM)	
P04271	2019	2019-Q2	2019-05	Pre-pandemic	Cytology (Pap)	40-44	42	Not performed	Satisfactory	NILM	NILM	Negative (NILM)	Trichomonas
P04272	2019	2019-Q2	2019-05	Pre-pandemic	Cytology (Pap)	35-39	35	Not performed	Satisfactory	NILM	NILM	Negative (NILM)	
P04273	2019	2019-Q2	2019-05	Pre-pandemic	Cytology (Pap)	40-44	44	Not performed	Satisfactory	NILM	NILM	Negative (NILM)	
P04274	2019	2019-Q2	2019-05	Pre-pandemic	Cytology (Pap)	40-44	44	Not performed	Satisfactory	NILM	NILM	Negative (NILM)	
P04275	2019	2019-Q2	2019-05	Pre-pandemic	Cytology (Pap)	35-39	38	Not performed	Satisfactory	NILM	NILM	Negative (NILM)	
P04276	2019	2019-Q2	2019-05	Pre-pandemic	Cytology (Pap)	40-44	42	Not performed	Satisfactory	Positive	ASC-US	Low-grade / equivocal squamous	
P04277	2019	2019-Q2	2019-05	Pre-pandemic	Cytology (Pap)	40-44	43	Not performed	Satisfactory	NILM	NILM	Negative (NILM)	
P04278	2019	2019-Q2	2019-05	Pre-pandemic	Cytology (Pap)	40-44	40	Not performed	Satisfactory	NILM	NILM	Negative (NILM)	
P04279	2019	2019-Q2	2019-05	Pre-pandemic	Cytology (Pap)	30-34	33	Not performed	Satisfactory	NILM	NILM	Negative (NILM)	
P04280	2019	2019-Q2	2019-05	Pre-pandemic	Cytology (Pap)	45-49	48	Not performed	Satisfactory	NILM	NILM	Negative (NILM)	
P04281	2019	2019-Q2	2019-05	Pre-pandemic	Cytology (Pap)	30-34	34	Not performed	Satisfactory	NILM	NILM	Negative (NILM)	Trichomonas
P04282	2019	2019-Q2	2019-05	Pre-pandemic	Cytology (Pap)	35-39	38	Not performed	Satisfactory	NILM	NILM	Negative (NILM)	
P04283	2019	2019-Q2	2019-05	Pre-pandemic	Cytology (Pap)	45-49	45	Not performed	Satisfactory	NILM	NILM	Negative (NILM)	
P04284	2019	2019-Q2	2019-05	Pre-pandemic	Cytology (Pap)	40-44	42	Not performed	Satisfactory	NILM	NILM	Negative (NILM)	
P04285	2019	2019-Q2	2019-05	Pre-pandemic	Cytology (Pap)	30-34	33	Not performed	Satisfactory	Positive	HSIL	High-grade squamous	Trichomonas
P04286	2019	2019-Q2	2019-05	Pre-pandemic	Cytology (Pap)	55-59	55	Not performed	Satisfactory	Positive	LSIL	Low-grade / equivocal squamous	
P04287	2019	2019-Q2	2019-05	Pre-pandemic	Cytology (Pap)	25-29	27	Not performed	Satisfactory	NILM	NILM	Negative (NILM)	
P04288	2019	2019-Q2	2019-05	Pre-pandemic	Cytology (Pap)	35-39	36	Not performed	Satisfactory	NILM	NILM	Negative (NILM)	
P04289	2019	2019-Q2	2019-05	Pre-pandemic	Cytology (Pap)	50-54	51	Not performed	Satisfactory	NILM	NILM	Negative (NILM)	
P04290	2019	2019-Q2	2019-05	Pre-pandemic	Cytology (Pap)	50-54	51	Not performed	Satisfactory	NILM	NILM	Negative (NILM)	
P04291	2019	2019-Q2	2019-05	Pre-pandemic	Cytology (Pap)	40-44	40	Not performed	Satisfactory	NILM	NILM	Negative (NILM)	
P04292	2019	2019-Q2	2019-05	Pre-pandemic	Cytology (Pap)	60-64	62	Not performed	Satisfactory	NILM	NILM	Negative (NILM)	
P04293	2019	2019-Q2	2019-05	Pre-pandemic	Cytology (Pap)	40-44	44	Not performed	Satisfactory	NILM	NILM	Negative (NILM)	
P04294	2019	2019-Q2	2019-05	Pre-pandemic	Cytology (Pap)	35-39	38	Not performed	Satisfactory	NILM	NILM	Negative (NILM)	
P04295	2019	2019-Q2	2019-05	Pre-pandemic	Cytology (Pap)	50-54	50	Not performed	Satisfactory	NILM	NILM	Negative (NILM)	
P04296	2019	2019-Q2	2019-05	Pre-pandemic	Cytology (Pap)	30-34	32	Not performed	Satisfactory	NILM	NILM	Negative (NILM)	
P04297	2019	2019-Q2	2019-05	Pre-pandemic	Cytology (Pap)	35-39	36	Not performed	Satisfactory	NILM	NILM	Negative (NILM)	
P04298	2019	2019-Q2	2019-05	Pre-pandemic	Cytology (Pap)	45-49	46	Not performed	Satisfactory	NILM	NILM	Negative (NILM)	
P04299	2019	2019-Q2	2019-05	Pre-pandemic	Cytology (Pap)	30-34	34	Not performed	Satisfactory	NILM	NILM	Negative (NILM)	
P04300	2019	2019-Q2	2019-05	Pre-pandemic	Cytology (Pap)	30-34	32	Not performed	Satisfactory	NILM	NILM	Negative (NILM)	Trichomonas
P04301	2019	2019-Q2	2019-05	Pre-pandemic	Cytology (Pap)	55-59	56	Not performed	Satisfactory	NILM	NILM	Negative (NILM)	
P04302	2019	2019-Q2	2019-05	Pre-pandemic	Cytology (Pap)	40-44	41	Not performed	Satisfactory	Positive	ASC-US	Low-grade / equivocal squamous	
P04303	2019	2019-Q2	2019-05	Pre-pandemic	Cytology (Pap)	55-59	56	Not performed	Satisfactory	NILM	NILM	Negative (NILM)	
P04304	2019	2019-Q2	2019-05	Pre-pandemic	Cytology (Pap)	45-49	45	Not performed	Satisfactory	Positive	HSIL	High-grade squamous	
P04305	2019	2019-Q2	2019-05	Pre-pandemic	Cytology (Pap)	50-54	51	Not performed	Satisfactory	NILM	NILM	Negative (NILM)	
P04306	2019	2019-Q2	2019-05	Pre-pandemic	Cytology (Pap)	50-54	50	Not performed	Satisfactory	NILM	NILM	Negative (NILM)	
P04307	2019	2019-Q2	2019-05	Pre-pandemic	Cytology (Pap)	35-39	37	Not performed	Satisfactory	NILM	NILM	Negative (NILM)	
P04308	2019	2019-Q2	2019-05	Pre-pandemic	Cytology (Pap)	25-29	27	Not performed	Satisfactory	NILM	NILM	Negative (NILM)	Candida
P04309	2019	2019-Q2	2019-05	Pre-pandemic	Cytology (Pap)	50-54	51	Not performed	Satisfactory	NILM	NILM	Negative (NILM)	
P04310	2019	2019-Q2	2019-05	Pre-pandemic	Cytology (Pap)	40-44	44	Not performed	Satisfactory	NILM	NILM	Negative (NILM)	Candida
P04311	2019	2019-Q2	2019-05	Pre-pandemic	Cytology (Pap)	45-49	47	Not performed	Satisfactory	NILM	NILM	Negative (NILM)	
P04312	2019	2019-Q2	2019-05	Pre-pandemic	Cytology (Pap)	55-59	57	Not performed	Satisfactory	NILM	NILM	Negative (NILM)	
P04313	2019	2019-Q2	2019-05	Pre-pandemic	Cytology (Pap)	40-44	41	Not performed	Satisfactory	Positive	ASC-US	Low-grade / equivocal squamous	
P04314	2019	2019-Q2	2019-05	Pre-pandemic	Cytology (Pap)	60-64	60	Not performed	Satisfactory	NILM	NILM	Negative (NILM)	
P04315	2019	2019-Q2	2019-05	Pre-pandemic	Cytology (Pap)	35-39	37	Not performed	Satisfactory	NILM	NILM	Negative (NILM)	Trichomonas
P04316	2019	2019-Q2	2019-05	Pre-pandemic	Cytology (Pap)	55-59	57	Not performed	Satisfactory	NILM	NILM	Negative (NILM)	
P04317	2019	2019-Q2	2019-05	Pre-pandemic	Cytology (Pap)	40-44	41	Not performed	Satisfactory	NILM	NILM	Negative (NILM)	
P04318	2019	2019-Q2	2019-05	Pre-pandemic	Cytology (Pap)	35-39	35	Not performed	Satisfactory	NILM	NILM	Negative (NILM)	

patient_id	year	quarter	month	period	primary_test	age_group	age_years	hpv_result	adequacy	result	bethesda	grade	infection
P04319	2019	2019-Q2	2019-05	Pre-pandemic	Cytology (Pap)	40-44	40	Not performed	Satisfactory	NILM	NILM	Negative (NILM)	
P04320	2019	2019-Q2	2019-05	Pre-pandemic	Cytology (Pap)	50-54	51	Not performed	Satisfactory	NILM	NILM	Negative (NILM)	
P04321	2019	2019-Q2	2019-05	Pre-pandemic	Cytology (Pap)	35-39	38	Not performed	Satisfactory	NILM	NILM	Negative (NILM)	
P04322	2019	2019-Q2	2019-05	Pre-pandemic	Cytology (Pap)	40-44	41	Not performed	Satisfactory	NILM	NILM	Negative (NILM)	
P04323	2019	2019-Q2	2019-05	Pre-pandemic	Cytology (Pap)	45-49	47	Not performed	Satisfactory	NILM	NILM	Negative (NILM)	
P04324	2019	2019-Q2	2019-05	Pre-pandemic	Cytology (Pap)	55-59	55	Not performed	Satisfactory	NILM	NILM	Negative (NILM)	
P04325	2019	2019-Q2	2019-05	Pre-pandemic	Cytology (Pap)	40-44	42	Not performed	Satisfactory	Positive	HSIL	High-grade squamous	
P04326	2019	2019-Q2	2019-05	Pre-pandemic	Cytology (Pap)	50-54	52	Not performed	Satisfactory	NILM	NILM	Negative (NILM)	
P04327	2019	2019-Q2	2019-05	Pre-pandemic	Cytology (Pap)	35-39	37	Not performed	Satisfactory	NILM	NILM	Negative (NILM)	
P04328	2019	2019-Q2	2019-05	Pre-pandemic	Cytology (Pap)	45-49	45	Not performed	Satisfactory	NILM	NILM	Negative (NILM)	
P04329	2019	2019-Q2	2019-05	Pre-pandemic	Cytology (Pap)	35-39	37	Not performed	Satisfactory	NILM	NILM	Negative (NILM)	
P04330	2019	2019-Q2	2019-05	Pre-pandemic	Cytology (Pap)	25-29	27	Not performed	Satisfactory	NILM	NILM	Negative (NILM)	Gardnerella
P04331	2019	2019-Q2	2019-05	Pre-pandemic	Cytology (Pap)	50-54	52	Not performed	Satisfactory	NILM	NILM	Negative (NILM)	
P04332	2019	2019-Q2	2019-05	Pre-pandemic	Cytology (Pap)	45-49	45	Not performed	Satisfactory	Positive	ASC-US	Low-grade / equivocal squamous	
P04333	2019	2019-Q2	2019-05	Pre-pandemic	Cytology (Pap)	50-54	51	Not performed	Satisfactory	NILM	NILM	Negative (NILM)	
P04334	2019	2019-Q2	2019-05	Pre-pandemic	Cytology (Pap)	35-39	36	Not performed	Satisfactory	NILM	NILM	Negative (NILM)	
P04335	2019	2019-Q2	2019-05	Pre-pandemic	Cytology (Pap)	55-59	58	Not performed	Satisfactory	NILM	NILM	Negative (NILM)	
P04336	2019	2019-Q2	2019-05	Pre-pandemic	Cytology (Pap)	50-54	52	Not performed	Satisfactory	NILM	NILM	Negative (NILM)	
P04337	2019	2019-Q2	2019-05	Pre-pandemic	Cytology (Pap)	60-64	60	Not performed	Satisfactory	NILM	NILM	Negative (NILM)	
P04338	2019	2019-Q2	2019-05	Pre-pandemic	Cytology (Pap)	30-34	30	Not performed	Satisfactory	Positive	LSIL	Low-grade / equivocal squamous	
P04339	2019	2019-Q2	2019-05	Pre-pandemic	Cytology (Pap)	40-44	41	Not performed	Satisfactory	NILM	NILM	Negative (NILM)	
P04340	2019	2019-Q2	2019-05	Pre-pandemic	Cytology (Pap)	55-59	56	Not performed	Satisfactory	Positive	ASC-H	High-grade squamous	
P04341	2019	2019-Q2	2019-05	Pre-pandemic	Cytology (Pap)	40-44	41	Not performed	Satisfactory	NILM	NILM	Negative (NILM)	
P04342	2019	2019-Q2	2019-05	Pre-pandemic	Cytology (Pap)	40-44	42	Not performed	Satisfactory	Positive	ASC-H	High-grade squamous	
P04343	2019	2019-Q2	2019-05	Pre-pandemic	Cytology (Pap)	50-54	53	Not performed	Satisfactory	NILM	NILM	Negative (NILM)	
P04344	2019	2019-Q2	2019-05	Pre-pandemic	Cytology (Pap)	45-49	45	Not performed	Satisfactory	NILM	NILM	Negative (NILM)	
P04345	2019	2019-Q2	2019-05	Pre-pandemic	Cytology (Pap)	40-44	41	Not performed	Satisfactory	NILM	NILM	Negative (NILM)	
P04346	2019	2019-Q2	2019-05	Pre-pandemic	Cytology (Pap)	55-59	56	Not performed	Satisfactory	NILM	NILM	Negative (NILM)	
P04347	2019	2019-Q2	2019-05	Pre-pandemic	Cytology (Pap)	35-39	39	Not performed	Satisfactory	NILM	NILM	Negative (NILM)	
P04348	2019	2019-Q2	2019-05	Pre-pandemic	Cytology (Pap)	35-39	37	Not performed	Satisfactory	NILM	NILM	Negative (NILM)	
P04349	2019	2019-Q2	2019-05	Pre-pandemic	Cytology (Pap)	40-44	41	Not performed	Satisfactory	NILM	NILM	Negative (NILM)	
P04350	2019	2019-Q2	2019-05	Pre-pandemic	Cytology (Pap)	25-29	29	Not performed	Satisfactory	NILM	NILM	Negative (NILM)	
P04351	2019	2019-Q2	2019-05	Pre-pandemic	Cytology (Pap)	30-34	32	Not performed	Satisfactory	NILM	NILM	Negative (NILM)	
P04352	2019	2019-Q2	2019-05	Pre-pandemic	Cytology (Pap)	40-44	41	Not performed	Satisfactory	NILM	NILM	Negative (NILM)	
P04353	2019	2019-Q2	2019-05	Pre-pandemic	Cytology (Pap)	35-39	39	Not performed	Satisfactory	NILM	NILM	Negative (NILM)	
P04354	2019	2019-Q2	2019-05	Pre-pandemic	Cytology (Pap)	40-44	42	Not performed	Satisfactory	NILM	NILM	Negative (NILM)	
P04355	2019	2019-Q2	2019-05	Pre-pandemic	Cytology (Pap)	35-39	35	Not performed	Satisfactory	NILM	NILM	Negative (NILM)	
P04356	2019	2019-Q2	2019-06	Pre-pandemic	Cytology (Pap)	40-44	41	Not performed	Satisfactory	NILM	NILM	Negative (NILM)	
P04357	2019	2019-Q2	2019-06	Pre-pandemic	Cytology (Pap)	60-64	64	Not performed	Satisfactory	NILM	NILM	Negative (NILM)	
P04358	2019	2019-Q2	2019-06	Pre-pandemic	Cytology (Pap)	35-39	39	Not performed	Satisfactory	NILM	NILM	Negative (NILM)	
P04359	2019	2019-Q2	2019-06	Pre-pandemic	Cytology (Pap)	40-44	44	Not performed	Satisfactory	NILM	NILM	Negative (NILM)	
P04360	2019	2019-Q2	2019-06	Pre-pandemic	Cytology (Pap)	30-34	33	Not performed	Satisfactory	NILM	NILM	Negative (NILM)	
P04361	2019	2019-Q2	2019-06	Pre-pandemic	Cytology (Pap)	30-34	32	Not performed	Satisfactory	NILM	NILM	Negative (NILM)	
P04362	2019	2019-Q2	2019-06	Pre-pandemic	Cytology (Pap)	25-29	29	Not performed	Satisfactory	Positive	ASC-US	Low-grade / equivocal squamous	
P04363	2019	2019-Q2	2019-06	Pre-pandemic	Cytology (Pap)	35-39	39	Not performed	Satisfactory	NILM	NILM	Negative (NILM)	
P04364	2019	2019-Q2	2019-06	Pre-pandemic	Cytology (Pap)	45-49	46	Not performed	Satisfactory	NILM	NILM	Negative (NILM)	
P04365	2019	2019-Q2	2019-06	Pre-pandemic	Cytology (Pap)	40-44	43	Not performed	Satisfactory	NILM	NILM	Negative (NILM)	
P04366	2019	2019-Q2	2019-06	Pre-pandemic	Cytology (Pap)	35-39	36	Not performed	Satisfactory	NILM	NILM	Negative (NILM)	
P04367	2019	2019-Q2	2019-06	Pre-pandemic	Cytology (Pap)	30-34	32	Not performed	Satisfactory	NILM	NILM	Negative (NILM)	
P04368	2019	2019-Q2	2019-06	Pre-pandemic	Cytology (Pap)	50-54	52	Not performed	Satisfactory	NILM	NILM	Negative (NILM)	
P04369	2019	2019-Q2	2019-06	Pre-pandemic	Cytology (Pap)	55-59	59	Not performed	Satisfactory	NILM	NILM	Negative (NILM)	
P04370	2019	2019-Q2	2019-06	Pre-pandemic	Cytology (Pap)	45-49	47	Not performed	Satisfactory	NILM	NILM	Negative (NILM)	
P04371	2019	2019-Q2	2019-06	Pre-pandemic	Cytology (Pap)	60-64	62	Not performed	Satisfactory	NILM	NILM	Negative (NILM)	
P04372	2019	2019-Q2	2019-06	Pre-pandemic	Cytology (Pap)	50-54	53	Not performed	Satisfactory	NILM	NILM	Negative (NILM)	
P04373	2019	2019-Q2	2019-06	Pre-pandemic	Cytology (Pap)	35-39	35	Not performed	Satisfactory	NILM	NILM	Negative (NILM)	
P04374	2019	2019-Q2	2019-06	Pre-pandemic	Cytology (Pap)	40-44	43	Not performed	Satisfactory	NILM	NILM	Negative (NILM)	
P04375	2019	2019-Q2	2019-06	Pre-pandemic	Cytology (Pap)	50-54	54	Not performed	Satisfactory	NILM	NILM	Negative (NILM)	
P04376	2019	2019-Q2	2019-06	Pre-pandemic	Cytology (Pap)	35-39	37	Not performed	Satisfactory	NILM	NILM	Negative (NILM)	
P04377	2019	2019-Q2	2019-06	Pre-pandemic	Cytology (Pap)	40-44	43	Not performed	Satisfactory	NILM	NILM	Negative (NILM)	
P04378	2019	2019-Q2	2019-06	Pre-pandemic	Cytology (Pap)	30-34	33	Not performed	Satisfactory	NILM	NILM	Negative (NILM)	
P04379	2019	2019-Q2	2019-06	Pre-pandemic	Cytology (Pap)	50-54	54	Not performed	Satisfactory	NILM	NILM	Negative (NILM)	
P04380	2019	2019-Q2	2019-06	Pre-pandemic	Cytology (Pap)	40-44	40	Not performed	Satisfactory	NILM	NILM	Negative (NILM)	
P04381	2019	2019-Q2	2019-06	Pre-pandemic	Cytology (Pap)	40-44	40	Not performed	Satisfactory	NILM	NILM	Negative (NILM)	
P04382	2019	2019-Q2	2019-06	Pre-pandemic	Cytology (Pap)	45-49	47	Not performed	Satisfactory	NILM	NILM	Negative (NILM)	
P04383	2019	2019-Q2	2019-06	Pre-pandemic	Cytology (Pap)	30-34	31	Not performed	Satisfactory	NILM	NILM	Negative (NILM)	
P04384	2019	2019-Q2	2019-06	Pre-pandemic	Cytology (Pap)	30-34	33	Not performed	Satisfactory	NILM	NILM	Negative (NILM)	
P04385	2019	2019-Q2	2019-06	Pre-pandemic	Cytology (Pap)	45-49	47	Not performed	Satisfactory	NILM	NILM	Negative (NILM)	
P04386	2019	2019-Q2	2019-06	Pre-pandemic	Cytology (Pap)	40-44	42	Not performed	Satisfactory	NILM	NILM	Negative (NILM)	
P04387	2019	2019-Q2	2019-06	Pre-pandemic	Cytology (Pap)	40-44	40	Not performed	Satisfactory	NILM	NILM	Negative (NILM)	
P04388	2019	2019-Q2	2019-06	Pre-pandemic	Cytology (Pap)	50-54	51	Not performed	Satisfactory	NILM	NILM	Negative (NILM)	
P04389	2019	2019-Q2	2019-06	Pre-pandemic	Cytology (Pap)	30-34	32	Not performed	Satisfactory	NILM	NILM	Negative (NILM)	
P04390	2019	2019-Q2	2019-06	Pre-pandemic	Cytology (Pap)	40-44	40	Not performed	Satisfactory	NILM	NILM	Negative (NILM)	
P04391	2019	2019-Q2	2019-06	Pre-pandemic	Cytology (Pap)	50-54	51	Not performed	Satisfactory	NILM	NILM	Negative (NILM)	
P04392	2019	2019-Q2	2019-06	Pre-pandemic	Cytology (Pap)	40-44	42	Not performed	Satisfactory	NILM	NILM	Negative (NILM)	
P04393	2019	2019-Q2	2019-06	Pre-pandemic	Cytology (Pap)	30-34	32	Not performed	Satisfactory	NILM	NILM	Negative (NILM)	
P04394	2019	2019-Q2	2019-06	Pre-pandemic	Cytology (Pap)	45-49	48	Not performed	Satisfactory	NILM	NILM	Negative (NILM)	
P04395	2019	2019-Q2	2019-06	Pre-pandemic	Cytology (Pap)	45-49	46	Not performed	Satisfactory	NILM	NILM	Negative (NILM)	
P04396	2019	2019-Q2	2019-06	Pre-pandemic	Cytology (Pap)	35-39	39	Not performed	Satisfactory	Positive	ASC-US	Low-grade / equivocal squamous	
P04397	2019	2019-Q2	2019-06	Pre-pandemic	Cytology (Pap)	25-29	27	Not performed	Satisfactory	NILM	NILM	Negative (NILM)	
P04398	2019	2019-Q2	2019-06	Pre-pandemic	Cytology (Pap)	40-44	44	Not performed	Satisfactory	Positive	HSIL	High-grade squamous	

patient_id	year	quarter	month	period	primary_test	age_group	age_years	hpv_result	adequacy	result	bethesda	grade	infection
P04399	2019	2019-Q2	2019-06	Pre-pandemic	Cytology (Pap)	35-39	36	Not performed	Unsatisfactory	Unsatisfactory	Unsatisfactory	Unsatisfactory	
P04400	2019	2019-Q2	2019-06	Pre-pandemic	Cytology (Pap)	35-39	36	Not performed	Satisfactory	NILM	NILM	Negative (NILM)	
P04401	2019	2019-Q2	2019-06	Pre-pandemic	Cytology (Pap)	60-64	60	Not performed	Satisfactory	NILM	NILM	Negative (NILM)	Trichomonas
P04402	2019	2019-Q2	2019-06	Pre-pandemic	Cytology (Pap)	40-44	42	Not performed	Satisfactory	NILM	NILM	Negative (NILM)	
P04403	2019	2019-Q2	2019-06	Pre-pandemic	Cytology (Pap)	50-54	54	Not performed	Satisfactory	NILM	NILM	Negative (NILM)	
P04404	2019	2019-Q2	2019-06	Pre-pandemic	Cytology (Pap)	30-34	30	Not performed	Satisfactory	NILM	NILM	Negative (NILM)	
P04405	2019	2019-Q2	2019-06	Pre-pandemic	Cytology (Pap)	40-44	41	Not performed	Satisfactory	NILM	NILM	Negative (NILM)	
P04406	2019	2019-Q2	2019-06	Pre-pandemic	Cytology (Pap)	55-59	56	Not performed	Satisfactory	NILM	NILM	Negative (NILM)	
P04407	2019	2019-Q2	2019-06	Pre-pandemic	Cytology (Pap)	35-39	39	Not performed	Satisfactory	NILM	NILM	Negative (NILM)	
P04408	2019	2019-Q2	2019-06	Pre-pandemic	Cytology (Pap)	40-44	43	Not performed	Satisfactory	Positive	ASC-US	Low-grade / equivocal squamous	
P04409	2019	2019-Q2	2019-06	Pre-pandemic	Cytology (Pap)	55-59	58	Not performed	Satisfactory	NILM	NILM	Negative (NILM)	
P04410	2019	2019-Q2	2019-06	Pre-pandemic	Cytology (Pap)	55-59	59	Not performed	Satisfactory	NILM	NILM	Negative (NILM)	
P04411	2019	2019-Q2	2019-06	Pre-pandemic	Cytology (Pap)	40-44	43	Not performed	Satisfactory	NILM	NILM	Negative (NILM)	
P04412	2019	2019-Q2	2019-06	Pre-pandemic	Cytology (Pap)	45-49	46	Not performed	Satisfactory	NILM	NILM	Negative (NILM)	Trichomonas
P04413	2019	2019-Q2	2019-06	Pre-pandemic	Cytology (Pap)	55-59	59	Not performed	Satisfactory	NILM	NILM	Negative (NILM)	
P04414	2019	2019-Q2	2019-06	Pre-pandemic	Cytology (Pap)	55-59	59	Not performed	Satisfactory	Positive	ASC-US	Low-grade / equivocal squamous	
P04415	2019	2019-Q2	2019-06	Pre-pandemic	Cytology (Pap)	40-44	41	Not performed	Unsatisfactory	Unsatisfactory	Unsatisfactory	Unsatisfactory	
P04416	2019	2019-Q2	2019-06	Pre-pandemic	Cytology (Pap)	50-54	51	Not performed	Satisfactory	NILM	NILM	Negative (NILM)	
P04417	2019	2019-Q2	2019-06	Pre-pandemic	Cytology (Pap)	40-44	44	Not performed	Satisfactory	NILM	NILM	Negative (NILM)	
P04418	2019	2019-Q2	2019-06	Pre-pandemic	Cytology (Pap)	40-44	42	Not performed	Satisfactory	NILM	NILM	Negative (NILM)	
P04419	2019	2019-Q2	2019-06	Pre-pandemic	Cytology (Pap)	60-64	62	Not performed	Unsatisfactory	Unsatisfactory	Unsatisfactory	Unsatisfactory	
P04420	2019	2019-Q2	2019-06	Pre-pandemic	Cytology (Pap)	45-49	49	Not performed	Satisfactory	NILM	NILM	Negative (NILM)	
P04421	2019	2019-Q2	2019-06	Pre-pandemic	Cytology (Pap)	30-34	31	Not performed	Satisfactory	NILM	NILM	Negative (NILM)	
P04422	2019	2019-Q2	2019-06	Pre-pandemic	Cytology (Pap)	35-39	35	Not performed	Satisfactory	NILM	NILM	Negative (NILM)	
P04423	2019	2019-Q2	2019-06	Pre-pandemic	Cytology (Pap)	40-44	41	Not performed	Satisfactory	NILM	NILM	Negative (NILM)	
P04424	2019	2019-Q2	2019-06	Pre-pandemic	Cytology (Pap)	35-39	37	Not performed	Satisfactory	NILM	NILM	Negative (NILM)	
P04425	2019	2019-Q2	2019-06	Pre-pandemic	Cytology (Pap)	60-64	64	Not performed	Satisfactory	NILM	NILM	Negative (NILM)	
P04426	2019	2019-Q2	2019-06	Pre-pandemic	Cytology (Pap)	40-44	44	Not performed	Satisfactory	NILM	NILM	Negative (NILM)	
P04427	2019	2019-Q2	2019-06	Pre-pandemic	Cytology (Pap)	35-39	38	Not performed	Satisfactory	NILM	NILM	Negative (NILM)	Trichomonas
P04428	2019	2019-Q2	2019-06	Pre-pandemic	Cytology (Pap)	60-64	64	Not performed	Satisfactory	Positive	HSIL	High-grade squamous	
P04429	2019	2019-Q2	2019-06	Pre-pandemic	Cytology (Pap)	55-59	57	Not performed	Satisfactory	NILM	NILM	Negative (NILM)	
P04430	2019	2019-Q2	2019-06	Pre-pandemic	Cytology (Pap)	30-34	32	Not performed	Satisfactory	NILM	NILM	Negative (NILM)	
P04431	2019	2019-Q2	2019-06	Pre-pandemic	Cytology (Pap)	40-44	40	Not performed	Satisfactory	NILM	NILM	Negative (NILM)	
P04432	2019	2019-Q2	2019-06	Pre-pandemic	Cytology (Pap)	60-64	63	Not performed	Satisfactory	NILM	NILM	Negative (NILM)	
P04433	2019	2019-Q2	2019-06	Pre-pandemic	Cytology (Pap)	50-54	53	Not performed	Satisfactory	NILM	NILM	Negative (NILM)	
P04434	2019	2019-Q2	2019-06	Pre-pandemic	Cytology (Pap)	55-59	55	Not performed	Satisfactory	NILM	NILM	Negative (NILM)	
P04435	2019	2019-Q2	2019-06	Pre-pandemic	Cytology (Pap)	55-59	59	Not performed	Satisfactory	Positive	ASC-US	Low-grade / equivocal squamous	
P04436	2019	2019-Q2	2019-06	Pre-pandemic	Cytology (Pap)	60-64	64	Not performed	Satisfactory	Positive	LSIL	Low-grade / equivocal squamous	
P04437	2019	2019-Q2	2019-06	Pre-pandemic	Cytology (Pap)	40-44	42	Not performed	Satisfactory	NILM	NILM	Negative (NILM)	
P04438	2019	2019-Q2	2019-06	Pre-pandemic	Cytology (Pap)	30-34	34	Not performed	Satisfactory	NILM	NILM	Negative (NILM)	Candida
P04439	2019	2019-Q2	2019-06	Pre-pandemic	Cytology (Pap)	45-49	46	Not performed	Satisfactory	NILM	NILM	Negative (NILM)	
P04440	2019	2019-Q2	2019-06	Pre-pandemic	Cytology (Pap)	60-64	60	Not performed	Satisfactory	NILM	NILM	Negative (NILM)	
P04441	2019	2019-Q2	2019-06	Pre-pandemic	Cytology (Pap)	50-54	53	Not performed	Satisfactory	NILM	NILM	Negative (NILM)	
P04442	2019	2019-Q2	2019-06	Pre-pandemic	Cytology (Pap)	45-49	48	Not performed	Satisfactory	NILM	NILM	Negative (NILM)	
P04443	2019	2019-Q2	2019-06	Pre-pandemic	Cytology (Pap)	35-39	39	Not performed	Satisfactory	NILM	NILM	Negative (NILM)	
P04444	2019	2019-Q2	2019-06	Pre-pandemic	Cytology (Pap)	60-64	61	Not performed	Satisfactory	NILM	NILM	Negative (NILM)	Gardnerella
P04445	2019	2019-Q2	2019-06	Pre-pandemic	Cytology (Pap)	50-54	51	Not performed	Satisfactory	Positive	ASC-US	Low-grade / equivocal squamous	
P04446	2019	2019-Q2	2019-06	Pre-pandemic	Cytology (Pap)	50-54	53	Not performed	Satisfactory	NILM	NILM	Negative (NILM)	
P04447	2019	2019-Q2	2019-06	Pre-pandemic	Cytology (Pap)	40-44	44	Not performed	Satisfactory	NILM	NILM	Negative (NILM)	
P04448	2019	2019-Q2	2019-06	Pre-pandemic	Cytology (Pap)	40-44	40	Not performed	Unsatisfactory	Unsatisfactory	Unsatisfactory	Unsatisfactory	
P04449	2019	2019-Q2	2019-06	Pre-pandemic	Cytology (Pap)	45-49	47	Not performed	Satisfactory	Positive	ASC-US	Low-grade / equivocal squamous	
P04450	2019	2019-Q2	2019-06	Pre-pandemic	Cytology (Pap)	30-34	33	Not performed	Satisfactory	NILM	NILM	Negative (NILM)	
P04451	2019	2019-Q2	2019-06	Pre-pandemic	Cytology (Pap)	55-59	59	Not performed	Satisfactory	NILM	NILM	Negative (NILM)	
P04452	2019	2019-Q2	2019-06	Pre-pandemic	Cytology (Pap)	40-44	43	Not performed	Satisfactory	NILM	NILM	Negative (NILM)	
P04453	2019	2019-Q2	2019-06	Pre-pandemic	Cytology (Pap)	30-34	31	Not performed	Satisfactory	NILM	NILM	Negative (NILM)	
P04454	2019	2019-Q2	2019-06	Pre-pandemic	Cytology (Pap)	50-54	51	Not performed	Satisfactory	NILM	NILM	Negative (NILM)	
P04455	2019	2019-Q2	2019-06	Pre-pandemic	Cytology (Pap)	40-44	40	Not performed	Satisfactory	NILM	NILM	Negative (NILM)	
P04456	2019	2019-Q2	2019-06	Pre-pandemic	Cytology (Pap)	35-39	36	Not performed	Satisfactory	NILM	NILM	Negative (NILM)	
P04457	2019	2019-Q2	2019-06	Pre-pandemic	Cytology (Pap)	40-44	42	Not performed	Satisfactory	NILM	NILM	Negative (NILM)	
P04458	2019	2019-Q2	2019-06	Pre-pandemic	Cytology (Pap)	30-34	32	Not performed	Satisfactory	NILM	NILM	Negative (NILM)	
P04459	2019	2019-Q2	2019-06	Pre-pandemic	Cytology (Pap)	50-54	50	Not performed	Satisfactory	NILM	NILM	Negative (NILM)	
P04460	2019	2019-Q2	2019-06	Pre-pandemic	Cytology (Pap)	55-59	57	Not performed	Unsatisfactory	Unsatisfactory	Unsatisfactory	Unsatisfactory	
P04461	2019	2019-Q2	2019-06	Pre-pandemic	Cytology (Pap)	60-64	64	Not performed	Satisfactory	NILM	NILM	Negative (NILM)	
P04462	2019	2019-Q2	2019-06	Pre-pandemic	Cytology (Pap)	30-34	33	Not performed	Satisfactory	NILM	NILM	Negative (NILM)	
P04463	2019	2019-Q2	2019-06	Pre-pandemic	Cytology (Pap)	40-44	41	Not performed	Satisfactory	NILM	NILM	Negative (NILM)	
P04464	2019	2019-Q2	2019-06	Pre-pandemic	Cytology (Pap)	40-44	40	Not performed	Satisfactory	NILM	NILM	Negative (NILM)	
P04465	2019	2019-Q2	2019-06	Pre-pandemic	Cytology (Pap)	60-64	61	Not performed	Satisfactory	NILM	NILM	Negative (NILM)	
P04466	2019	2019-Q2	2019-06	Pre-pandemic	Cytology (Pap)	30-34	33	Not performed	Satisfactory	NILM	NILM	Negative (NILM)	
P04467	2019	2019-Q2	2019-06	Pre-pandemic	Cytology (Pap)	35-39	35	Not performed	Satisfactory	NILM	NILM	Negative (NILM)	
P04468	2019	2019-Q2	2019-06	Pre-pandemic	Cytology (Pap)	45-49	48	Not performed	Satisfactory	Positive	ASC-US	Low-grade / equivocal squamous	
P04469	2019	2019-Q2	2019-06	Pre-pandemic	Cytology (Pap)	50-54	54	Not performed	Satisfactory	NILM	NILM	Negative (NILM)	
P04470	2019	2019-Q2	2019-06	Pre-pandemic	Cytology (Pap)	50-54	51	Not performed	Satisfactory	NILM	NILM	Negative (NILM)	
P04471	2019	2019-Q2	2019-06	Pre-pandemic	Cytology (Pap)	45-49	45	Not performed	Satisfactory	NILM	NILM	Negative (NILM)	
P04472	2019	2019-Q2	2019-06	Pre-pandemic	Cytology (Pap)	30-34	30	Not performed	Satisfactory	NILM	NILM	Negative (NILM)	
P04473	2019	2019-Q2	2019-06	Pre-pandemic	Cytology (Pap)	30-34	33	Not performed	Satisfactory	NILM	NILM	Negative (NILM)	
P04474	2019	2019-Q2	2019-06	Pre-pandemic	Cytology (Pap)	50-54	51	Not performed	Satisfactory	Positive	ASC-US	Low-grade / equivocal squamous	
P04475	2019	2019-Q2	2019-06	Pre-pandemic	Cytology (Pap)	35-39	37	Not performed	Satisfactory	NILM	NILM	Negative (NILM)	
P04476	2019	2019-Q2	2019-06	Pre-pandemic	Cytology (Pap)	35-39	37	Not performed	Satisfactory	NILM	NILM	Negative (NILM)	
P04477	2019	2019-Q2	2019-06	Pre-pandemic	Cytology (Pap)	45-49	49	Not performed	Satisfactory	NILM	NILM	Negative (NILM)	
P04478	2019	2019-Q2	2019-06	Pre-pandemic	Cytology (Pap)	40-44	41	Not performed	Satisfactory	NILM	NILM	Negative (NILM)	

patient_id	year	quarter	month	period	primary_test	age_group	age_years	hpv_result	adequacy	result	bethesda	grade	infection
P04479	2019	2019-Q2	2019-06	Pre-pandemic	Cytology (Pap)	50-54	51	Not performed	Satisfactory	NILM	NILM	Negative (NILM)	Trichomonas
P04480	2019	2019-Q2	2019-06	Pre-pandemic	Cytology (Pap)	50-54	54	Not performed	Satisfactory	NILM	NILM	Negative (NILM)	
P04481	2019	2019-Q2	2019-06	Pre-pandemic	Cytology (Pap)	55-59	58	Not performed	Satisfactory	NILM	NILM	Negative (NILM)	Candida
P04482	2019	2019-Q2	2019-06	Pre-pandemic	Cytology (Pap)	25-29	27	Not performed	Satisfactory	NILM	NILM	Negative (NILM)	
P04483	2019	2019-Q2	2019-06	Pre-pandemic	Cytology (Pap)	30-34	34	Not performed	Satisfactory	NILM	NILM	Negative (NILM)	
P04484	2019	2019-Q2	2019-06	Pre-pandemic	Cytology (Pap)	40-44	43	Not performed	Satisfactory	Positive	LSIL	Low-grade / equivocal squamous	
P04485	2019	2019-Q2	2019-06	Pre-pandemic	Cytology (Pap)	45-49	47	Not performed	Satisfactory	NILM	NILM	Negative (NILM)	
P04486	2019	2019-Q2	2019-06	Pre-pandemic	Cytology (Pap)	50-54	54	Not performed	Satisfactory	NILM	NILM	Negative (NILM)	
P04487	2019	2019-Q2	2019-06	Pre-pandemic	Cytology (Pap)	45-49	48	Not performed	Satisfactory	NILM	NILM	Negative (NILM)	
P04488	2019	2019-Q2	2019-06	Pre-pandemic	Cytology (Pap)	40-44	42	Not performed	Satisfactory	NILM	NILM	Negative (NILM)	
P04489	2019	2019-Q2	2019-06	Pre-pandemic	Cytology (Pap)	40-44	40	Not performed	Satisfactory	NILM	NILM	Negative (NILM)	
P04490	2019	2019-Q2	2019-06	Pre-pandemic	Cytology (Pap)	60-64	62	Not performed	Satisfactory	NILM	NILM	Negative (NILM)	
P04491	2019	2019-Q2	2019-06	Pre-pandemic	Cytology (Pap)	55-59	55	Not performed	Satisfactory	NILM	NILM	Negative (NILM)	
P04492	2019	2019-Q2	2019-06	Pre-pandemic	Cytology (Pap)	60-64	62	Not performed	Satisfactory	NILM	NILM	Negative (NILM)	
P04493	2019	2019-Q2	2019-06	Pre-pandemic	Cytology (Pap)	40-44	44	Not performed	Satisfactory	NILM	NILM	Negative (NILM)	
P04494	2019	2019-Q2	2019-06	Pre-pandemic	Cytology (Pap)	55-59	57	Not performed	Satisfactory	NILM	NILM	Negative (NILM)	
P04495	2019	2019-Q2	2019-06	Pre-pandemic	Cytology (Pap)	50-54	52	Not performed	Satisfactory	NILM	NILM	Negative (NILM)	
P04496	2019	2019-Q2	2019-06	Pre-pandemic	Cytology (Pap)	45-49	49	Not performed	Satisfactory	NILM	NILM	Negative (NILM)	
P04497	2019	2019-Q2	2019-06	Pre-pandemic	Cytology (Pap)	40-44	41	Not performed	Satisfactory	NILM	NILM	Negative (NILM)	
P04498	2019	2019-Q2	2019-06	Pre-pandemic	Cytology (Pap)	30-34	32	Not performed	Satisfactory	Positive	LSIL	Low-grade / equivocal squamous	
P04499	2019	2019-Q2	2019-06	Pre-pandemic	Cytology (Pap)	30-34	34	Not performed	Satisfactory	NILM	NILM	Negative (NILM)	
P04500	2019	2019-Q2	2019-06	Pre-pandemic	Cytology (Pap)	50-54	51	Not performed	Satisfactory	NILM	NILM	Negative (NILM)	
P04501	2019	2019-Q2	2019-06	Pre-pandemic	Cytology (Pap)	60-64	62	Not performed	Satisfactory	Positive	ASC-US	Low-grade / equivocal squamous	
P04502	2019	2019-Q2	2019-06	Pre-pandemic	Cytology (Pap)	50-54	52	Not performed	Satisfactory	NILM	NILM	Negative (NILM)	Candida
P04503	2019	2019-Q2	2019-06	Pre-pandemic	Cytology (Pap)	40-44	40	Not performed	Satisfactory	NILM	NILM	Negative (NILM)	Candida
P04504	2019	2019-Q2	2019-06	Pre-pandemic	Cytology (Pap)	35-39	38	Not performed	Satisfactory	NILM	NILM	Negative (NILM)	
P04505	2019	2019-Q2	2019-06	Pre-pandemic	Cytology (Pap)	45-49	47	Not performed	Satisfactory	Positive	ASC-H	High-grade squamous	
P04506	2019	2019-Q2	2019-06	Pre-pandemic	Cytology (Pap)	35-39	35	Not performed	Satisfactory	Positive	ASC-US	Low-grade / equivocal squamous	
P04507	2019	2019-Q2	2019-06	Pre-pandemic	Cytology (Pap)	30-34	32	Not performed	Satisfactory	NILM	NILM	Negative (NILM)	
P04508	2019	2019-Q2	2019-06	Pre-pandemic	Cytology (Pap)	35-39	37	Not performed	Satisfactory	NILM	NILM	Negative (NILM)	
P04509	2019	2019-Q2	2019-06	Pre-pandemic	Cytology (Pap)	30-34	34	Not performed	Satisfactory	NILM	NILM	Negative (NILM)	
P04510	2019	2019-Q2	2019-06	Pre-pandemic	Cytology (Pap)	35-39	38	Not performed	Satisfactory	NILM	NILM	Negative (NILM)	
P04511	2019	2019-Q2	2019-06	Pre-pandemic	Cytology (Pap)	30-34	30	Not performed	Satisfactory	NILM	NILM	Negative (NILM)	
P04512	2019	2019-Q2	2019-06	Pre-pandemic	Cytology (Pap)	40-44	44	Not performed	Satisfactory	NILM	NILM	Negative (NILM)	
P04513	2019	2019-Q2	2019-06	Pre-pandemic	Cytology (Pap)	25-29	26	Not performed	Satisfactory	NILM	NILM	Negative (NILM)	
P04514	2019	2019-Q2	2019-06	Pre-pandemic	Cytology (Pap)	45-49	45	Not performed	Satisfactory	NILM	NILM	Negative (NILM)	
P04515	2019	2019-Q2	2019-06	Pre-pandemic	Cytology (Pap)	55-59	57	Not performed	Satisfactory	NILM	NILM	Negative (NILM)	
P04516	2019	2019-Q2	2019-06	Pre-pandemic	Cytology (Pap)	45-49	49	Not performed	Satisfactory	NILM	NILM	Negative (NILM)	Candida
P04517	2019	2019-Q2	2019-06	Pre-pandemic	Cytology (Pap)	55-59	57	Not performed	Satisfactory	Positive	LSIL	Low-grade / equivocal squamous	
P04518	2019	2019-Q2	2019-06	Pre-pandemic	Cytology (Pap)	30-34	32	Not performed	Satisfactory	NILM	NILM	Negative (NILM)	
P04519	2019	2019-Q2	2019-06	Pre-pandemic	Cytology (Pap)	50-54	51	Not performed	Satisfactory	NILM	NILM	Negative (NILM)	
P04520	2019	2019-Q2	2019-06	Pre-pandemic	Cytology (Pap)	35-39	35	Not performed	Satisfactory	NILM	NILM	Negative (NILM)	
P04521	2019	2019-Q2	2019-06	Pre-pandemic	Cytology (Pap)	40-44	43	Not performed	Satisfactory	NILM	NILM	Negative (NILM)	
P04522	2019	2019-Q2	2019-06	Pre-pandemic	Cytology (Pap)	55-59	57	Not performed	Satisfactory	NILM	NILM	Negative (NILM)	
P04523	2019	2019-Q2	2019-06	Pre-pandemic	Cytology (Pap)	30-34	33	Not performed	Satisfactory	NILM	NILM	Negative (NILM)	
P04524	2019	2019-Q2	2019-06	Pre-pandemic	Cytology (Pap)	40-44	42	Not performed	Satisfactory	NILM	NILM	Negative (NILM)	
P04525	2019	2019-Q2	2019-06	Pre-pandemic	Cytology (Pap)	50-54	53	Not performed	Satisfactory	NILM	NILM	Negative (NILM)	
P04526	2019	2019-Q2	2019-06	Pre-pandemic	Cytology (Pap)	40-44	44	Not performed	Satisfactory	NILM	NILM	Negative (NILM)	
P04527	2019	2019-Q2	2019-06	Pre-pandemic	Cytology (Pap)	35-39	38	Not performed	Satisfactory	NILM	NILM	Negative (NILM)	
P04528	2019	2019-Q2	2019-06	Pre-pandemic	Cytology (Pap)	50-54	52	Not performed	Satisfactory	NILM	NILM	Negative (NILM)	
P04529	2019	2019-Q2	2019-06	Pre-pandemic	Cytology (Pap)	55-59	59	Not performed	Satisfactory	NILM	NILM	Negative (NILM)	
P04530	2019	2019-Q2	2019-06	Pre-pandemic	Cytology (Pap)	50-54	54	Not performed	Satisfactory	NILM	NILM	Negative (NILM)	
P04531	2019	2019-Q2	2019-06	Pre-pandemic	Cytology (Pap)	40-44	40	Not performed	Satisfactory	NILM	NILM	Negative (NILM)	
P04532	2019	2019-Q2	2019-06	Pre-pandemic	Cytology (Pap)	45-49	47	Not performed	Satisfactory	NILM	NILM	Negative (NILM)	
P04533	2019	2019-Q2	2019-06	Pre-pandemic	Cytology (Pap)	55-59	55	Not performed	Satisfactory	NILM	NILM	Negative (NILM)	
P04534	2019	2019-Q2	2019-06	Pre-pandemic	Cytology (Pap)	50-54	52	Not performed	Satisfactory	NILM	NILM	Negative (NILM)	
P04535	2019	2019-Q2	2019-06	Pre-pandemic	Cytology (Pap)	35-39	36	Not performed	Satisfactory	NILM	NILM	Negative (NILM)	
P04536	2019	2019-Q2	2019-06	Pre-pandemic	Cytology (Pap)	40-44	43	Not performed	Satisfactory	NILM	NILM	Negative (NILM)	
P04537	2019	2019-Q2	2019-06	Pre-pandemic	Cytology (Pap)	25-29	29	Not performed	Satisfactory	Positive	LSIL	Low-grade / equivocal squamous	
P04538	2019	2019-Q2	2019-06	Pre-pandemic	Cytology (Pap)	55-59	57	Not performed	Satisfactory	NILM	NILM	Negative (NILM)	
P04539	2019	2019-Q2	2019-06	Pre-pandemic	Cytology (Pap)	30-34	31	Not performed	Satisfactory	NILM	NILM	Negative (NILM)	
P04540	2019	2019-Q2	2019-06	Pre-pandemic	Cytology (Pap)	30-34	31	Not performed	Satisfactory	NILM	NILM	Negative (NILM)	
P04541	2019	2019-Q2	2019-06	Pre-pandemic	Cytology (Pap)	50-54	51	Not performed	Satisfactory	NILM	NILM	Negative (NILM)	
P04542	2019	2019-Q2	2019-06	Pre-pandemic	Cytology (Pap)	35-39	37	Not performed	Satisfactory	Positive	LSIL	Low-grade / equivocal squamous	
P04543	2019	2019-Q2	2019-06	Pre-pandemic	Cytology (Pap)	35-39	38	Not performed	Satisfactory	NILM	NILM	Negative (NILM)	
P04544	2019	2019-Q2	2019-06	Pre-pandemic	Cytology (Pap)	40-44	43	Not performed	Satisfactory	NILM	NILM	Negative (NILM)	Candida
P04545	2019	2019-Q2	2019-06	Pre-pandemic	Cytology (Pap)	40-44	41	Not performed	Satisfactory	NILM	NILM	Negative (NILM)	
P04546	2019	2019-Q2	2019-06	Pre-pandemic	Cytology (Pap)	50-54	53	Not performed	Satisfactory	NILM	NILM	Negative (NILM)	
P04547	2019	2019-Q2	2019-06	Pre-pandemic	Cytology (Pap)	50-54	50	Not performed	Satisfactory	NILM	NILM	Negative (NILM)	
P04548	2019	2019-Q2	2019-06	Pre-pandemic	Cytology (Pap)	30-34	33	Not performed	Satisfactory	NILM	NILM	Negative (NILM)	Trichomonas
P04549	2019	2019-Q2	2019-06	Pre-pandemic	Cytology (Pap)	45-49	47	Not performed	Satisfactory	NILM	NILM	Negative (NILM)	
P04550	2019	2019-Q2	2019-06	Pre-pandemic	Cytology (Pap)	50-54	54	Not performed	Satisfactory	NILM	NILM	Negative (NILM)	
P04551	2019	2019-Q2	2019-06	Pre-pandemic	Cytology (Pap)	40-44	43	Not performed	Satisfactory	NILM	NILM	Negative (NILM)	
P04552	2019	2019-Q2	2019-06	Pre-pandemic	Cytology (Pap)	35-39	39	Not performed	Satisfactory	NILM	NILM	Negative (NILM)	
P04553	2019	2019-Q2	2019-06	Pre-pandemic	Cytology (Pap)	60-64	60	Not performed	Satisfactory	NILM	NILM	Negative (NILM)	
P04554	2019	2019-Q2	2019-06	Pre-pandemic	Cytology (Pap)	35-39	36	Not performed	Unsatisfactory	Unsatisfactory	Unsatisfactory	Unsatisfactory	
P04555	2019	2019-Q2	2019-06	Pre-pandemic	Cytology (Pap)	45-49	47	Not performed	Satisfactory	NILM	NILM	Negative (NILM)	
P04556	2019	2019-Q2	2019-06	Pre-pandemic	Cytology (Pap)	25-29	26	Not performed	Satisfactory	NILM	NILM	Negative (NILM)	
P04557	2019	2019-Q2	2019-06	Pre-pandemic	Cytology (Pap)	50-54	52	Not performed	Satisfactory	Positive	HSIL	High-grade squamous	
P04558	2019	2019-Q2	2019-06	Pre-pandemic	Cytology (Pap)	30-34	32	Not performed	Satisfactory	NILM	NILM	Negative (NILM)	

patient_id	year	quarter	month	period	primary_test	age_group	age_years	hpv_result	adequacy	result	bethesda	grade	infection
P04559	2019	2019-Q2	2019-06	Pre-pandemic	Cytology (Pap)	30-34	34	Not performed	Satisfactory	Positive	ASC-US	Low-grade / equivocal squamous	
P04560	2019	2019-Q2	2019-06	Pre-pandemic	Cytology (Pap)	55-59	59	Not performed	Satisfactory	NILM	NILM	Negative (NILM)	
P04561	2019	2019-Q2	2019-06	Pre-pandemic	Cytology (Pap)	35-39	38	Not performed	Satisfactory	Positive	ASC-US	Low-grade / equivocal squamous	
P04562	2019	2019-Q2	2019-06	Pre-pandemic	Cytology (Pap)	45-49	49	Not performed	Satisfactory	NILM	NILM	Negative (NILM)	
P04563	2019	2019-Q2	2019-06	Pre-pandemic	Cytology (Pap)	40-44	41	Not performed	Satisfactory	NILM	NILM	Negative (NILM)	
P04564	2019	2019-Q2	2019-06	Pre-pandemic	Cytology (Pap)	45-49	45	Not performed	Satisfactory	NILM	NILM	Negative (NILM)	
P04565	2019	2019-Q2	2019-06	Pre-pandemic	Cytology (Pap)	40-44	43	Not performed	Satisfactory	NILM	NILM	Negative (NILM)	
P04566	2019	2019-Q2	2019-06	Pre-pandemic	Cytology (Pap)	60-64	61	Not performed	Satisfactory	NILM	NILM	Negative (NILM)	
P04567	2019	2019-Q2	2019-06	Pre-pandemic	Cytology (Pap)	50-54	52	Not performed	Satisfactory	Positive	ASC-US	Low-grade / equivocal squamous	
P04568	2019	2019-Q2	2019-06	Pre-pandemic	Cytology (Pap)	55-59	57	Not performed	Satisfactory	NILM	NILM	Negative (NILM)	
P04569	2019	2019-Q2	2019-06	Pre-pandemic	Cytology (Pap)	40-44	40	Not performed	Satisfactory	NILM	NILM	Negative (NILM)	
P04570	2019	2019-Q2	2019-06	Pre-pandemic	Cytology (Pap)	50-54	54	Not performed	Satisfactory	NILM	NILM	Negative (NILM)	
P04571	2019	2019-Q2	2019-06	Pre-pandemic	Cytology (Pap)	50-54	51	Not performed	Satisfactory	NILM	NILM	Negative (NILM)	Candida
P04572	2019	2019-Q2	2019-06	Pre-pandemic	Cytology (Pap)	35-39	37	Not performed	Satisfactory	NILM	NILM	Negative (NILM)	
P04573	2019	2019-Q2	2019-06	Pre-pandemic	Cytology (Pap)	60-64	60	Not performed	Satisfactory	NILM	NILM	Negative (NILM)	
P04574	2019	2019-Q2	2019-06	Pre-pandemic	Cytology (Pap)	30-34	31	Not performed	Satisfactory	NILM	NILM	Negative (NILM)	
P04575	2019	2019-Q2	2019-06	Pre-pandemic	Cytology (Pap)	45-49	45	Not performed	Satisfactory	NILM	NILM	Negative (NILM)	
P04576	2019	2019-Q2	2019-06	Pre-pandemic	Cytology (Pap)	35-39	35	Not performed	Satisfactory	NILM	NILM	Negative (NILM)	
P04577	2019	2019-Q2	2019-06	Pre-pandemic	Cytology (Pap)	55-59	55	Not performed	Satisfactory	NILM	NILM	Negative (NILM)	
P04578	2019	2019-Q2	2019-06	Pre-pandemic	Cytology (Pap)	55-59	57	Not performed	Satisfactory	NILM	NILM	Negative (NILM)	
P04579	2019	2019-Q2	2019-06	Pre-pandemic	Cytology (Pap)	35-39	39	Not performed	Satisfactory	NILM	NILM	Negative (NILM)	
P04580	2019	2019-Q2	2019-06	Pre-pandemic	Cytology (Pap)	55-59	58	Not performed	Satisfactory	NILM	NILM	Negative (NILM)	
P04581	2019	2019-Q2	2019-06	Pre-pandemic	Cytology (Pap)	50-54	52	Not performed	Satisfactory	NILM	NILM	Negative (NILM)	
P04582	2019	2019-Q2	2019-06	Pre-pandemic	Cytology (Pap)	35-39	37	Not performed	Satisfactory	NILM	NILM	Negative (NILM)	
P04583	2019	2019-Q2	2019-06	Pre-pandemic	Cytology (Pap)	50-54	51	Not performed	Satisfactory	Positive	HSIL	High-grade squamous	
P04584	2019	2019-Q2	2019-06	Pre-pandemic	Cytology (Pap)	60-64	63	Not performed	Satisfactory	NILM	NILM	Negative (NILM)	
P04585	2019	2019-Q2	2019-06	Pre-pandemic	Cytology (Pap)	45-49	46	Not performed	Satisfactory	NILM	NILM	Negative (NILM)	
P04586	2019	2019-Q2	2019-06	Pre-pandemic	Cytology (Pap)	45-49	48	Not performed	Satisfactory	NILM	NILM	Negative (NILM)	
P04587	2019	2019-Q2	2019-06	Pre-pandemic	Cytology (Pap)	45-49	45	Not performed	Satisfactory	NILM	NILM	Negative (NILM)	
P04588	2019	2019-Q2	2019-06	Pre-pandemic	Cytology (Pap)	50-54	54	Not performed	Satisfactory	NILM	NILM	Negative (NILM)	
P04589	2019	2019-Q2	2019-06	Pre-pandemic	Cytology (Pap)	30-34	32	Not performed	Satisfactory	NILM	NILM	Negative (NILM)	
P04590	2019	2019-Q2	2019-06	Pre-pandemic	Cytology (Pap)	40-44	41	Not performed	Satisfactory	NILM	NILM	Negative (NILM)	
P04591	2019	2019-Q2	2019-06	Pre-pandemic	Cytology (Pap)	30-34	30	Not performed	Satisfactory	NILM	NILM	Negative (NILM)	
P04592	2019	2019-Q2	2019-06	Pre-pandemic	Cytology (Pap)	40-44	40	Not performed	Satisfactory	NILM	NILM	Negative (NILM)	
P04593	2019	2019-Q2	2019-06	Pre-pandemic	Cytology (Pap)	45-49	47	Not performed	Satisfactory	NILM	NILM	Negative (NILM)	
P04594	2019	2019-Q3	2019-07	Pre-pandemic	Cytology (Pap)	35-39	37	Not performed	Satisfactory	Positive	LSIL	Low-grade / equivocal squamous	
P04595	2019	2019-Q3	2019-07	Pre-pandemic	Cytology (Pap)	35-39	38	Not performed	Satisfactory	NILM	NILM	Negative (NILM)	
P04596	2019	2019-Q3	2019-07	Pre-pandemic	Cytology (Pap)	50-54	52	Not performed	Satisfactory	NILM	NILM	Negative (NILM)	
P04597	2019	2019-Q3	2019-07	Pre-pandemic	Cytology (Pap)	50-54	54	Not performed	Satisfactory	NILM	NILM	Negative (NILM)	
P04598	2019	2019-Q3	2019-07	Pre-pandemic	Cytology (Pap)	35-39	36	Not performed	Satisfactory	NILM	NILM	Negative (NILM)	
P04599	2019	2019-Q3	2019-07	Pre-pandemic	Cytology (Pap)	50-54	51	Not performed	Satisfactory	NILM	NILM	Negative (NILM)	
P04600	2019	2019-Q3	2019-07	Pre-pandemic	Cytology (Pap)	50-54	50	Not performed	Satisfactory	Positive	ASC-US	Low-grade / equivocal squamous	
P04601	2019	2019-Q3	2019-07	Pre-pandemic	Cytology (Pap)	45-49	46	Not performed	Satisfactory	NILM	NILM	Negative (NILM)	
P04602	2019	2019-Q3	2019-07	Pre-pandemic	Cytology (Pap)	50-54	50	Not performed	Satisfactory	NILM	NILM	Negative (NILM)	
P04603	2019	2019-Q3	2019-07	Pre-pandemic	Cytology (Pap)	60-64	62	Not performed	Satisfactory	Positive	ASC-US	Low-grade / equivocal squamous	
P04604	2019	2019-Q3	2019-07	Pre-pandemic	Cytology (Pap)	50-54	51	Not performed	Satisfactory	NILM	NILM	Negative (NILM)	
P04605	2019	2019-Q3	2019-07	Pre-pandemic	Cytology (Pap)	45-49	48	Not performed	Satisfactory	NILM	NILM	Negative (NILM)	
P04606	2019	2019-Q3	2019-07	Pre-pandemic	Cytology (Pap)	55-59	58	Not performed	Satisfactory	NILM	NILM	Negative (NILM)	
P04607	2019	2019-Q3	2019-07	Pre-pandemic	Cytology (Pap)	40-44	42	Not performed	Satisfactory	NILM	NILM	Negative (NILM)	
P04608	2019	2019-Q3	2019-07	Pre-pandemic	Cytology (Pap)	40-44	42	Not performed	Satisfactory	NILM	NILM	Negative (NILM)	
P04609	2019	2019-Q3	2019-07	Pre-pandemic	Cytology (Pap)	35-39	36	Not performed	Satisfactory	NILM	NILM	Negative (NILM)	
P04610	2019	2019-Q3	2019-07	Pre-pandemic	Cytology (Pap)	35-39	36	Not performed	Satisfactory	NILM	NILM	Negative (NILM)	
P04611	2019	2019-Q3	2019-07	Pre-pandemic	Cytology (Pap)	40-44	43	Not performed	Satisfactory	NILM	NILM	Negative (NILM)	
P04612	2019	2019-Q3	2019-07	Pre-pandemic	Cytology (Pap)	30-34	30	Not performed	Satisfactory	NILM	NILM	Negative (NILM)	
P04613	2019	2019-Q3	2019-07	Pre-pandemic	Cytology (Pap)	50-54	54	Not performed	Satisfactory	Positive	AGC	Glandular	
P04614	2019	2019-Q3	2019-07	Pre-pandemic	Cytology (Pap)	60-64	64	Not performed	Satisfactory	Positive	ASC-US	Low-grade / equivocal squamous	
P04615	2019	2019-Q3	2019-07	Pre-pandemic	Cytology (Pap)	35-39	36	Not performed	Satisfactory	NILM	NILM	Negative (NILM)	
P04616	2019	2019-Q3	2019-07	Pre-pandemic	Cytology (Pap)	50-54	52	Not performed	Satisfactory	Positive	ASC-US	Low-grade / equivocal squamous	
P04617	2019	2019-Q3	2019-07	Pre-pandemic	Cytology (Pap)	45-49	48	Not performed	Satisfactory	NILM	NILM	Negative (NILM)	
P04618	2019	2019-Q3	2019-07	Pre-pandemic	Cytology (Pap)	25-29	29	Not performed	Satisfactory	NILM	NILM	Negative (NILM)	
P04619	2019	2019-Q3	2019-07	Pre-pandemic	Cytology (Pap)	60-64	62	Not performed	Satisfactory	NILM	NILM	Negative (NILM)	
P04620	2019	2019-Q3	2019-07	Pre-pandemic	Cytology (Pap)	30-34	30	Not performed	Satisfactory	NILM	NILM	Negative (NILM)	
P04621	2019	2019-Q3	2019-07	Pre-pandemic	Cytology (Pap)	60-64	62	Not performed	Satisfactory	NILM	NILM	Negative (NILM)	
P04622	2019	2019-Q3	2019-07	Pre-pandemic	Cytology (Pap)	40-44	41	Not performed	Satisfactory	NILM	NILM	Negative (NILM)	
P04623	2019	2019-Q3	2019-07	Pre-pandemic	Cytology (Pap)	45-49	49	Not performed	Satisfactory	NILM	NILM	Negative (NILM)	
P04624	2019	2019-Q3	2019-07	Pre-pandemic	Cytology (Pap)	50-54	54	Not performed	Satisfactory	NILM	NILM	Negative (NILM)	
P04625	2019	2019-Q3	2019-07	Pre-pandemic	Cytology (Pap)	45-49	45	Not performed	Unsatisfactory	Unsatisfactory	Unsatisfactory	Unsatisfactory	
P04626	2019	2019-Q3	2019-07	Pre-pandemic	Cytology (Pap)	50-54	51	Not performed	Satisfactory	NILM	NILM	Negative (NILM)	
P04627	2019	2019-Q3	2019-07	Pre-pandemic	Cytology (Pap)	35-39	38	Not performed	Satisfactory	NILM	NILM	Negative (NILM)	
P04628	2019	2019-Q3	2019-07	Pre-pandemic	Cytology (Pap)	55-59	58	Not performed	Satisfactory	NILM	NILM	Negative (NILM)	
P04629	2019	2019-Q3	2019-07	Pre-pandemic	Cytology (Pap)	35-39	36	Not performed	Satisfactory	NILM	NILM	Negative (NILM)	
P04630	2019	2019-Q3	2019-07	Pre-pandemic	Cytology (Pap)	45-49	45	Not performed	Satisfactory	NILM	NILM	Negative (NILM)	
P04631	2019	2019-Q3	2019-07	Pre-pandemic	Cytology (Pap)	30-34	30	Not performed	Satisfactory	Positive	ASC-US	Low-grade / equivocal squamous	
P04632	2019	2019-Q3	2019-07	Pre-pandemic	Cytology (Pap)	60-64	62	Not performed	Satisfactory	NILM	NILM	Negative (NILM)	
P04633	2019	2019-Q3	2019-07	Pre-pandemic	Cytology (Pap)	25-29	26	Not performed	Satisfactory	NILM	NILM	Negative (NILM)	
P04634	2019	2019-Q3	2019-07	Pre-pandemic	Cytology (Pap)	40-44	44	Not performed	Unsatisfactory	Unsatisfactory	Unsatisfactory	Unsatisfactory	
P04635	2019	2019-Q3	2019-07	Pre-pandemic	Cytology (Pap)	45-49	46	Not performed	Satisfactory	NILM	NILM	Negative (NILM)	
P04636	2019	2019-Q3	2019-07	Pre-pandemic	Cytology (Pap)	40-44	44	Not performed	Satisfactory	NILM	NILM	Negative (NILM)	Trichomonas
P04637	2019	2019-Q3	2019-07	Pre-pandemic	Cytology (Pap)	40-44	42	Not performed	Satisfactory	NILM	NILM	Negative (NILM)	
P04638	2019	2019-Q3	2019-07	Pre-pandemic	Cytology (Pap)	40-44	41	Not performed	Satisfactory	NILM	NILM	Negative (NILM)	

patient_id	year	quarter	month	period	primary_test	age_group	age_years	hpv_result	adequacy	result	bethesda	grade	infection
P04639	2019	2019-Q3	2019-07	Pre-pandemic	Cytology (Pap)	30-34	32	Not performed	Satisfactory	NILM	NILM	Negative (NILM)	
P04640	2019	2019-Q3	2019-07	Pre-pandemic	Cytology (Pap)	50-54	51	Not performed	Satisfactory	NILM	NILM	Negative (NILM)	
P04641	2019	2019-Q3	2019-07	Pre-pandemic	Cytology (Pap)	50-54	51	Not performed	Satisfactory	NILM	NILM	Negative (NILM)	
P04642	2019	2019-Q3	2019-07	Pre-pandemic	Cytology (Pap)	30-34	33	Not performed	Satisfactory	Positive	HSIL	High-grade squamous	
P04643	2019	2019-Q3	2019-07	Pre-pandemic	Cytology (Pap)	40-44	44	Not performed	Satisfactory	NILM	NILM	Negative (NILM)	
P04644	2019	2019-Q3	2019-07	Pre-pandemic	Cytology (Pap)	60-64	62	Not performed	Satisfactory	NILM	NILM	Negative (NILM)	
P04645	2019	2019-Q3	2019-07	Pre-pandemic	Cytology (Pap)	40-44	40	Not performed	Satisfactory	NILM	NILM	Negative (NILM)	
P04646	2019	2019-Q3	2019-07	Pre-pandemic	Cytology (Pap)	50-54	50	Not performed	Satisfactory	NILM	NILM	Negative (NILM)	
P04647	2019	2019-Q3	2019-07	Pre-pandemic	Cytology (Pap)	45-49	49	Not performed	Satisfactory	NILM	NILM	Negative (NILM)	
P04648	2019	2019-Q3	2019-07	Pre-pandemic	Cytology (Pap)	50-54	53	Not performed	Satisfactory	NILM	NILM	Negative (NILM)	
P04649	2019	2019-Q3	2019-07	Pre-pandemic	Cytology (Pap)	55-59	58	Not performed	Satisfactory	NILM	NILM	Negative (NILM)	
P04650	2019	2019-Q3	2019-07	Pre-pandemic	Cytology (Pap)	45-49	46	Not performed	Satisfactory	NILM	NILM	Negative (NILM)	
P04651	2019	2019-Q3	2019-07	Pre-pandemic	Cytology (Pap)	40-44	42	Not performed	Satisfactory	NILM	NILM	Negative (NILM)	
P04652	2019	2019-Q3	2019-07	Pre-pandemic	Cytology (Pap)	25-29	26	Not performed	Satisfactory	NILM	NILM	Negative (NILM)	
P04653	2019	2019-Q3	2019-07	Pre-pandemic	Cytology (Pap)	30-34	31	Not performed	Satisfactory	Positive	LSIL	Low-grade / equivocal squamous	
P04654	2019	2019-Q3	2019-07	Pre-pandemic	Cytology (Pap)	40-44	44	Not performed	Satisfactory	NILM	NILM	Negative (NILM)	
P04655	2019	2019-Q3	2019-07	Pre-pandemic	Cytology (Pap)	35-39	35	Not performed	Satisfactory	NILM	NILM	Negative (NILM)	
P04656	2019	2019-Q3	2019-07	Pre-pandemic	Cytology (Pap)	55-59	55	Not performed	Satisfactory	NILM	NILM	Negative (NILM)	
P04657	2019	2019-Q3	2019-07	Pre-pandemic	Cytology (Pap)	55-59	55	Not performed	Satisfactory	NILM	NILM	Negative (NILM)	
P04658	2019	2019-Q3	2019-07	Pre-pandemic	Cytology (Pap)	40-44	42	Not performed	Satisfactory	NILM	NILM	Negative (NILM)	
P04659	2019	2019-Q3	2019-07	Pre-pandemic	Cytology (Pap)	45-49	48	Not performed	Satisfactory	NILM	NILM	Negative (NILM)	
P04660	2019	2019-Q3	2019-07	Pre-pandemic	Cytology (Pap)	45-49	47	Not performed	Satisfactory	NILM	NILM	Negative (NILM)	
P04661	2019	2019-Q3	2019-07	Pre-pandemic	Cytology (Pap)	35-39	35	Not performed	Satisfactory	NILM	NILM	Negative (NILM)	
P04662	2019	2019-Q3	2019-07	Pre-pandemic	Cytology (Pap)	50-54	50	Not performed	Satisfactory	NILM	NILM	Negative (NILM)	
P04663	2019	2019-Q3	2019-07	Pre-pandemic	Cytology (Pap)	40-44	43	Not performed	Satisfactory	NILM	NILM	Negative (NILM)	
P04664	2019	2019-Q3	2019-07	Pre-pandemic	Cytology (Pap)	45-49	49	Not performed	Satisfactory	NILM	NILM	Negative (NILM)	
P04665	2019	2019-Q3	2019-07	Pre-pandemic	Cytology (Pap)	55-59	58	Not performed	Satisfactory	NILM	NILM	Negative (NILM)	
P04666	2019	2019-Q3	2019-07	Pre-pandemic	Cytology (Pap)	50-54	53	Not performed	Satisfactory	Positive	ASC-US	Low-grade / equivocal squamous	
P04667	2019	2019-Q3	2019-07	Pre-pandemic	Cytology (Pap)	60-64	60	Not performed	Satisfactory	NILM	NILM	Negative (NILM)	
P04668	2019	2019-Q3	2019-07	Pre-pandemic	Cytology (Pap)	55-59	56	Not performed	Unsatisfactory	Unsatisfactory	Unsatisfactory	Unsatisfactory	
P04669	2019	2019-Q3	2019-07	Pre-pandemic	Cytology (Pap)	55-59	57	Not performed	Satisfactory	Positive	HSIL	High-grade squamous	
P04670	2019	2019-Q3	2019-07	Pre-pandemic	Cytology (Pap)	55-59	55	Not performed	Satisfactory	NILM	NILM	Negative (NILM)	
P04671	2019	2019-Q3	2019-07	Pre-pandemic	Cytology (Pap)	40-44	44	Not performed	Satisfactory	NILM	NILM	Negative (NILM)	
P04672	2019	2019-Q3	2019-07	Pre-pandemic	Cytology (Pap)	45-49	45	Not performed	Satisfactory	NILM	NILM	Negative (NILM)	
P04673	2019	2019-Q3	2019-07	Pre-pandemic	Cytology (Pap)	35-39	37	Not performed	Satisfactory	NILM	NILM	Negative (NILM)	
P04674	2019	2019-Q3	2019-07	Pre-pandemic	Cytology (Pap)	50-54	52	Not performed	Satisfactory	NILM	NILM	Negative (NILM)	
P04675	2019	2019-Q3	2019-07	Pre-pandemic	Cytology (Pap)	40-44	41	Not performed	Satisfactory	NILM	NILM	Negative (NILM)	
P04676	2019	2019-Q3	2019-07	Pre-pandemic	Cytology (Pap)	55-59	56	Not performed	Satisfactory	NILM	NILM	Negative (NILM)	
P04677	2019	2019-Q3	2019-07	Pre-pandemic	Cytology (Pap)	40-44	41	Not performed	Satisfactory	Positive	HSIL	High-grade squamous	
P04678	2019	2019-Q3	2019-07	Pre-pandemic	Cytology (Pap)	35-39	36	Not performed	Satisfactory	NILM	NILM	Negative (NILM)	
P04679	2019	2019-Q3	2019-07	Pre-pandemic	Cytology (Pap)	45-49	47	Not performed	Satisfactory	NILM	NILM	Negative (NILM)	
P04680	2019	2019-Q3	2019-07	Pre-pandemic	Cytology (Pap)	40-44	40	Not performed	Satisfactory	NILM	NILM	Negative (NILM)	
P04681	2019	2019-Q3	2019-07	Pre-pandemic	Cytology (Pap)	35-39	39	Not performed	Satisfactory	NILM	NILM	Negative (NILM)	
P04682	2019	2019-Q3	2019-07	Pre-pandemic	Cytology (Pap)	45-49	47	Not performed	Satisfactory	NILM	NILM	Negative (NILM)	
P04683	2019	2019-Q3	2019-07	Pre-pandemic	Cytology (Pap)	45-49	49	Not performed	Satisfactory	NILM	NILM	Negative (NILM)	
P04684	2019	2019-Q3	2019-07	Pre-pandemic	Cytology (Pap)	50-54	50	Not performed	Satisfactory	NILM	NILM	Negative (NILM)	
P04685	2019	2019-Q3	2019-07	Pre-pandemic	Cytology (Pap)	35-39	39	Not performed	Satisfactory	NILM	NILM	Negative (NILM)	
P04686	2019	2019-Q3	2019-07	Pre-pandemic	Cytology (Pap)	45-49	45	Not performed	Satisfactory	NILM	NILM	Negative (NILM)	
P04687	2019	2019-Q3	2019-07	Pre-pandemic	Cytology (Pap)	55-59	59	Not performed	Satisfactory	NILM	NILM	Negative (NILM)	
P04688	2019	2019-Q3	2019-07	Pre-pandemic	Cytology (Pap)	30-34	30	Not performed	Satisfactory	NILM	NILM	Negative (NILM)	
P04689	2019	2019-Q3	2019-07	Pre-pandemic	Cytology (Pap)	50-54	52	Not performed	Satisfactory	NILM	NILM	Negative (NILM)	
P04690	2019	2019-Q3	2019-07	Pre-pandemic	Cytology (Pap)	55-59	56	Not performed	Satisfactory	NILM	NILM	Negative (NILM)	
P04691	2019	2019-Q3	2019-07	Pre-pandemic	Cytology (Pap)	55-59	57	Not performed	Satisfactory	NILM	NILM	Negative (NILM)	
P04692	2019	2019-Q3	2019-07	Pre-pandemic	Cytology (Pap)	45-49	46	Not performed	Satisfactory	NILM	NILM	Negative (NILM)	
P04693	2019	2019-Q3	2019-07	Pre-pandemic	Cytology (Pap)	40-44	42	Not performed	Satisfactory	NILM	NILM	Negative (NILM)	
P04694	2019	2019-Q3	2019-07	Pre-pandemic	Cytology (Pap)	35-39	35	Not performed	Satisfactory	NILM	NILM	Negative (NILM)	
P04695	2019	2019-Q3	2019-07	Pre-pandemic	Cytology (Pap)	35-39	35	Not performed	Satisfactory	NILM	NILM	Negative (NILM)	
P04696	2019	2019-Q3	2019-07	Pre-pandemic	Cytology (Pap)	40-44	41	Not performed	Satisfactory	Positive	ASC-US	Low-grade / equivocal squamous	
P04697	2019	2019-Q3	2019-07	Pre-pandemic	Cytology (Pap)	55-59	58	Not performed	Satisfactory	NILM	NILM	Negative (NILM)	
P04698	2019	2019-Q3	2019-07	Pre-pandemic	Cytology (Pap)	40-44	41	Not performed	Satisfactory	NILM	NILM	Negative (NILM)	
P04699	2019	2019-Q3	2019-07	Pre-pandemic	Cytology (Pap)	45-49	48	Not performed	Satisfactory	NILM	NILM	Negative (NILM)	
P04700	2019	2019-Q3	2019-07	Pre-pandemic	Cytology (Pap)	45-49	45	Not performed	Satisfactory	NILM	NILM	Negative (NILM)	
P04701	2019	2019-Q3	2019-07	Pre-pandemic	Cytology (Pap)	50-54	54	Not performed	Satisfactory	NILM	NILM	Negative (NILM)	
P04702	2019	2019-Q3	2019-07	Pre-pandemic	Cytology (Pap)	60-64	60	Not performed	Unsatisfactory	Unsatisfactory	Unsatisfactory	Unsatisfactory	
P04703	2019	2019-Q3	2019-07	Pre-pandemic	Cytology (Pap)	30-34	33	Not performed	Satisfactory	NILM	NILM	Negative (NILM)	Trichomonas
P04704	2019	2019-Q3	2019-07	Pre-pandemic	Cytology (Pap)	55-59	59	Not performed	Satisfactory	NILM	NILM	Negative (NILM)	
P04705	2019	2019-Q3	2019-07	Pre-pandemic	Cytology (Pap)	35-39	36	Not performed	Unsatisfactory	Unsatisfactory	Unsatisfactory	Unsatisfactory	
P04706	2019	2019-Q3	2019-07	Pre-pandemic	Cytology (Pap)	35-39	39	Not performed	Satisfactory	NILM	NILM	Negative (NILM)	
P04707	2019	2019-Q3	2019-07	Pre-pandemic	Cytology (Pap)	45-49	47	Not performed	Satisfactory	NILM	NILM	Negative (NILM)	
P04708	2019	2019-Q3	2019-07	Pre-pandemic	Cytology (Pap)	45-49	48	Not performed	Satisfactory	NILM	NILM	Negative (NILM)	
P04709	2019	2019-Q3	2019-07	Pre-pandemic	Cytology (Pap)	50-54	51	Not performed	Satisfactory	NILM	NILM	Negative (NILM)	
P04710	2019	2019-Q3	2019-07	Pre-pandemic	Cytology (Pap)	50-54	52	Not performed	Satisfactory	NILM	NILM	Negative (NILM)	
P04711	2019	2019-Q3	2019-07	Pre-pandemic	Cytology (Pap)	30-34	32	Not performed	Satisfactory	NILM	NILM	Negative (NILM)	
P04712	2019	2019-Q3	2019-07	Pre-pandemic	Cytology (Pap)	50-54	51	Not performed	Satisfactory	NILM	NILM	Negative (NILM)	
P04713	2019	2019-Q3	2019-07	Pre-pandemic	Cytology (Pap)	55-59	58	Not performed	Satisfactory	NILM	NILM	Negative (NILM)	
P04714	2019	2019-Q3	2019-07	Pre-pandemic	Cytology (Pap)	40-44	41	Not performed	Satisfactory	NILM	NILM	Negative (NILM)	
P04715	2019	2019-Q3	2019-07	Pre-pandemic	Cytology (Pap)	45-49	46	Not performed	Satisfactory	NILM	NILM	Negative (NILM)	
P04716	2019	2019-Q3	2019-07	Pre-pandemic	Cytology (Pap)	45-49	45	Not performed	Satisfactory	NILM	NILM	Negative (NILM)	
P04717	2019	2019-Q3	2019-07	Pre-pandemic	Cytology (Pap)	35-39	35	Not performed	Satisfactory	Positive	HSIL	High-grade squamous	
P04718	2019	2019-Q3	2019-07	Pre-pandemic	Cytology (Pap)	50-54	51	Not performed	Satisfactory	NILM	NILM	Negative (NILM)	

patient_id	year	quarter	month	period	primary_test	age_group	age_years	hpv_result	adequacy	result	bethesda	grade	infection
P04719	2019	2019-Q3	2019-07	Pre-pandemic	Cytology (Pap)	35-39	39	Not performed	Satisfactory	NILM	NILM	Negative (NILM)	
P04720	2019	2019-Q3	2019-07	Pre-pandemic	Cytology (Pap)	55-59	55	Not performed	Satisfactory	Positive	LSIL	Low-grade / equivocal squamous	
P04721	2019	2019-Q3	2019-07	Pre-pandemic	Cytology (Pap)	40-44	42	Not performed	Satisfactory	Positive	LSIL	Low-grade / equivocal squamous	
P04722	2019	2019-Q3	2019-07	Pre-pandemic	Cytology (Pap)	30-34	34	Not performed	Satisfactory	NILM	NILM	Negative (NILM)	
P04723	2019	2019-Q3	2019-07	Pre-pandemic	Cytology (Pap)	50-54	50	Not performed	Satisfactory	NILM	NILM	Negative (NILM)	
P04724	2019	2019-Q3	2019-07	Pre-pandemic	Cytology (Pap)	60-64	64	Not performed	Unsatisfactory	Unsatisfactory	Unsatisfactory	Unsatisfactory	
P04725	2019	2019-Q3	2019-07	Pre-pandemic	Cytology (Pap)	30-34	30	Not performed	Satisfactory	NILM	NILM	Negative (NILM)	
P04726	2019	2019-Q3	2019-07	Pre-pandemic	Cytology (Pap)	50-54	52	Not performed	Satisfactory	NILM	NILM	Negative (NILM)	
P04727	2019	2019-Q3	2019-07	Pre-pandemic	Cytology (Pap)	50-54	50	Not performed	Satisfactory	NILM	NILM	Negative (NILM)	
P04728	2019	2019-Q3	2019-07	Pre-pandemic	Cytology (Pap)	60-64	64	Not performed	Satisfactory	Positive	HSIL	High-grade squamous	
P04729	2019	2019-Q3	2019-07	Pre-pandemic	Cytology (Pap)	40-44	42	Not performed	Satisfactory	NILM	NILM	Negative (NILM)	
P04730	2019	2019-Q3	2019-07	Pre-pandemic	Cytology (Pap)	35-39	36	Not performed	Satisfactory	NILM	NILM	Negative (NILM)	
P04731	2019	2019-Q3	2019-07	Pre-pandemic	Cytology (Pap)	55-59	59	Not performed	Satisfactory	NILM	NILM	Negative (NILM)	
P04732	2019	2019-Q3	2019-07	Pre-pandemic	Cytology (Pap)	55-59	59	Not performed	Satisfactory	NILM	NILM	Negative (NILM)	
P04733	2019	2019-Q3	2019-07	Pre-pandemic	Cytology (Pap)	40-44	40	Not performed	Satisfactory	NILM	NILM	Negative (NILM)	
P04734	2019	2019-Q3	2019-07	Pre-pandemic	Cytology (Pap)	30-34	34	Not performed	Satisfactory	NILM	NILM	Negative (NILM)	
P04735	2019	2019-Q3	2019-07	Pre-pandemic	Cytology (Pap)	50-54	54	Not performed	Satisfactory	NILM	NILM	Negative (NILM)	
P04736	2019	2019-Q3	2019-07	Pre-pandemic	Cytology (Pap)	40-44	44	Not performed	Satisfactory	Positive	ASC-US	Low-grade / equivocal squamous	
P04737	2019	2019-Q3	2019-07	Pre-pandemic	Cytology (Pap)	40-44	41	Not performed	Satisfactory	NILM	NILM	Negative (NILM)	
P04738	2019	2019-Q3	2019-07	Pre-pandemic	Cytology (Pap)	55-59	57	Not performed	Satisfactory	NILM	NILM	Negative (NILM)	
P04739	2019	2019-Q3	2019-07	Pre-pandemic	Cytology (Pap)	40-44	40	Not performed	Satisfactory	NILM	NILM	Negative (NILM)	
P04740	2019	2019-Q3	2019-07	Pre-pandemic	Cytology (Pap)	45-49	47	Not performed	Satisfactory	NILM	NILM	Negative (NILM)	
P04741	2019	2019-Q3	2019-07	Pre-pandemic	Cytology (Pap)	35-39	36	Not performed	Satisfactory	NILM	NILM	Negative (NILM)	
P04742	2019	2019-Q3	2019-07	Pre-pandemic	Cytology (Pap)	30-34	33	Not performed	Satisfactory	NILM	NILM	Negative (NILM)	
P04743	2019	2019-Q3	2019-07	Pre-pandemic	Cytology (Pap)	45-49	49	Not performed	Satisfactory	NILM	NILM	Negative (NILM)	
P04744	2019	2019-Q3	2019-07	Pre-pandemic	Cytology (Pap)	40-44	44	Not performed	Satisfactory	NILM	NILM	Negative (NILM)	
P04745	2019	2019-Q3	2019-07	Pre-pandemic	Cytology (Pap)	45-49	49	Not performed	Satisfactory	NILM	NILM	Negative (NILM)	
P04746	2019	2019-Q3	2019-07	Pre-pandemic	Cytology (Pap)	40-44	44	Not performed	Satisfactory	NILM	NILM	Negative (NILM)	
P04747	2019	2019-Q3	2019-07	Pre-pandemic	Cytology (Pap)	40-44	40	Not performed	Satisfactory	NILM	NILM	Negative (NILM)	
P04748	2019	2019-Q3	2019-07	Pre-pandemic	Cytology (Pap)	35-39	39	Not performed	Satisfactory	NILM	NILM	Negative (NILM)	
P04749	2019	2019-Q3	2019-07	Pre-pandemic	Cytology (Pap)	40-44	44	Not performed	Satisfactory	NILM	NILM	Negative (NILM)	
P04750	2019	2019-Q3	2019-07	Pre-pandemic	Cytology (Pap)	40-44	42	Not performed	Satisfactory	NILM	NILM	Negative (NILM)	
P04751	2019	2019-Q3	2019-07	Pre-pandemic	Cytology (Pap)	50-54	54	Not performed	Satisfactory	NILM	NILM	Negative (NILM)	
P04752	2019	2019-Q3	2019-07	Pre-pandemic	Cytology (Pap)	45-49	48	Not performed	Satisfactory	NILM	NILM	Negative (NILM)	
P04753	2019	2019-Q3	2019-07	Pre-pandemic	Cytology (Pap)	50-54	52	Not performed	Satisfactory	NILM	NILM	Negative (NILM)	
P04754	2019	2019-Q3	2019-07	Pre-pandemic	Cytology (Pap)	40-44	44	Not performed	Satisfactory	NILM	NILM	Negative (NILM)	
P04755	2019	2019-Q3	2019-07	Pre-pandemic	Cytology (Pap)	40-44	43	Not performed	Satisfactory	NILM	NILM	Negative (NILM)	
P04756	2019	2019-Q3	2019-07	Pre-pandemic	Cytology (Pap)	25-29	27	Not performed	Satisfactory	NILM	NILM	Negative (NILM)	
P04757	2019	2019-Q3	2019-07	Pre-pandemic	Cytology (Pap)	40-44	41	Not performed	Satisfactory	Positive	HSIL	High-grade squamous	
P04758	2019	2019-Q3	2019-07	Pre-pandemic	Cytology (Pap)	40-44	43	Not performed	Satisfactory	NILM	NILM	Negative (NILM)	
P04759	2019	2019-Q3	2019-07	Pre-pandemic	Cytology (Pap)	35-39	37	Not performed	Satisfactory	NILM	NILM	Negative (NILM)	
P04760	2019	2019-Q3	2019-07	Pre-pandemic	Cytology (Pap)	55-59	55	Not performed	Satisfactory	NILM	NILM	Negative (NILM)	
P04761	2019	2019-Q3	2019-07	Pre-pandemic	Cytology (Pap)	60-64	63	Not performed	Satisfactory	NILM	NILM	Negative (NILM)	
P04762	2019	2019-Q3	2019-07	Pre-pandemic	Cytology (Pap)	25-29	27	Not performed	Satisfactory	NILM	NILM	Negative (NILM)	
P04763	2019	2019-Q3	2019-07	Pre-pandemic	Cytology (Pap)	35-39	37	Not performed	Satisfactory	NILM	NILM	Negative (NILM)	
P04764	2019	2019-Q3	2019-07	Pre-pandemic	Cytology (Pap)	25-29	26	Not performed	Satisfactory	NILM	NILM	Negative (NILM)	
P04765	2019	2019-Q3	2019-07	Pre-pandemic	Cytology (Pap)	30-34	34	Not performed	Satisfactory	Positive	ASC-US	Low-grade / equivocal squamous	
P04766	2019	2019-Q3	2019-07	Pre-pandemic	Cytology (Pap)	30-34	31	Not performed	Satisfactory	Positive	ASC-US	Low-grade / equivocal squamous	
P04767	2019	2019-Q3	2019-07	Pre-pandemic	Cytology (Pap)	55-59	57	Not performed	Satisfactory	NILM	NILM	Negative (NILM)	
P04768	2019	2019-Q3	2019-07	Pre-pandemic	Cytology (Pap)	50-54	54	Not performed	Satisfactory	NILM	NILM	Negative (NILM)	
P04769	2019	2019-Q3	2019-07	Pre-pandemic	Cytology (Pap)	45-49	46	Not performed	Satisfactory	NILM	NILM	Negative (NILM)	
P04770	2019	2019-Q3	2019-07	Pre-pandemic	Cytology (Pap)	55-59	56	Not performed	Satisfactory	NILM	NILM	Negative (NILM)	
P04771	2019	2019-Q3	2019-07	Pre-pandemic	Cytology (Pap)	40-44	41	Not performed	Satisfactory	NILM	NILM	Negative (NILM)	
P04772	2019	2019-Q3	2019-07	Pre-pandemic	Cytology (Pap)	60-64	60	Not performed	Satisfactory	NILM	NILM	Negative (NILM)	
P04773	2019	2019-Q3	2019-07	Pre-pandemic	Cytology (Pap)	55-59	57	Not performed	Satisfactory	Positive	ASC-US	Low-grade / equivocal squamous	
P04774	2019	2019-Q3	2019-07	Pre-pandemic	Cytology (Pap)	30-34	32	Not performed	Satisfactory	NILM	NILM	Negative (NILM)	
P04775	2019	2019-Q3	2019-07	Pre-pandemic	Cytology (Pap)	35-39	36	Not performed	Satisfactory	NILM	NILM	Negative (NILM)	
P04776	2019	2019-Q3	2019-07	Pre-pandemic	Cytology (Pap)	40-44	40	Not performed	Satisfactory	NILM	NILM	Negative (NILM)	
P04777	2019	2019-Q3	2019-07	Pre-pandemic	Cytology (Pap)	55-59	58	Not performed	Satisfactory	NILM	NILM	Negative (NILM)	
P04778	2019	2019-Q3	2019-07	Pre-pandemic	Cytology (Pap)	35-39	39	Not performed	Satisfactory	NILM	NILM	Negative (NILM)	
P04779	2019	2019-Q3	2019-07	Pre-pandemic	Cytology (Pap)	45-49	45	Not performed	Satisfactory	NILM	NILM	Negative (NILM)	
P04780	2019	2019-Q3	2019-07	Pre-pandemic	Cytology (Pap)	45-49	46	Not performed	Satisfactory	NILM	NILM	Negative (NILM)	
P04781	2019	2019-Q3	2019-07	Pre-pandemic	Cytology (Pap)	35-39	39	Not performed	Satisfactory	Positive	HSIL	High-grade squamous	
P04782	2019	2019-Q3	2019-07	Pre-pandemic	Cytology (Pap)	35-39	36	Not performed	Satisfactory	NILM	NILM	Negative (NILM)	
P04783	2019	2019-Q3	2019-07	Pre-pandemic	Cytology (Pap)	40-44	44	Not performed	Satisfactory	NILM	NILM	Negative (NILM)	
P04784	2019	2019-Q3	2019-07	Pre-pandemic	Cytology (Pap)	30-34	33	Not performed	Satisfactory	NILM	NILM	Negative (NILM)	
P04785	2019	2019-Q3	2019-07	Pre-pandemic	Cytology (Pap)	45-49	47	Not performed	Unsatisfactory	Unsatisfactory	Unsatisfactory	Unsatisfactory	
P04786	2019	2019-Q3	2019-07	Pre-pandemic	Cytology (Pap)	50-54	51	Not performed	Satisfactory	NILM	NILM	Negative (NILM)	
P04787	2019	2019-Q3	2019-07	Pre-pandemic	Cytology (Pap)	30-34	31	Not performed	Satisfactory	NILM	NILM	Negative (NILM)	
P04788	2019	2019-Q3	2019-07	Pre-pandemic	Cytology (Pap)	45-49	49	Not performed	Satisfactory	NILM	NILM	Negative (NILM)	
P04789	2019	2019-Q3	2019-07	Pre-pandemic	Cytology (Pap)	45-49	49	Not performed	Satisfactory	NILM	NILM	Negative (NILM)	
P04790	2019	2019-Q3	2019-07	Pre-pandemic	Cytology (Pap)	40-44	42	Not performed	Satisfactory	NILM	NILM	Negative (NILM)	
P04791	2019	2019-Q3	2019-07	Pre-pandemic	Cytology (Pap)	25-29	28	Not performed	Satisfactory	NILM	NILM	Negative (NILM)	
P04792	2019	2019-Q3	2019-07	Pre-pandemic	Cytology (Pap)	60-64	63	Not performed	Satisfactory	NILM	NILM	Negative (NILM)	
P04793	2019	2019-Q3	2019-07	Pre-pandemic	Cytology (Pap)	45-49	49	Not performed	Satisfactory	NILM	NILM	Negative (NILM)	
P04794	2019	2019-Q3	2019-07	Pre-pandemic	Cytology (Pap)	40-44	41	Not performed	Satisfactory	NILM	NILM	Negative (NILM)	
P04795	2019	2019-Q3	2019-07	Pre-pandemic	Cytology (Pap)	45-49	49	Not performed	Satisfactory	NILM	NILM	Negative (NILM)	Gardnerella
P04796	2019	2019-Q3	2019-07	Pre-pandemic	Cytology (Pap)	35-39	37	Not performed	Unsatisfactory	Unsatisfactory	Unsatisfactory	Unsatisfactory	
P04797	2019	2019-Q3	2019-07	Pre-pandemic	Cytology (Pap)	25-29	27	Not performed	Satisfactory	NILM	NILM	Negative (NILM)	
P04798	2019	2019-Q3	2019-07	Pre-pandemic	Cytology (Pap)	40-44	42	Not performed	Satisfactory	Positive	LSIL	Low-grade / equivocal squamous	

patient_id	year	quarter	month	period	primary_test	age_group	age_years	hpv_result	adequacy	result	bethesda	grade	infection
P04799	2019	2019-Q3	2019-07	Pre-pandemic	Cytology (Pap)	40-44	42	Not performed	Satisfactory	NILM	NILM	Negative (NILM)	
P04800	2019	2019-Q3	2019-07	Pre-pandemic	Cytology (Pap)	60-64	61	Not performed	Satisfactory	NILM	NILM	Negative (NILM)	
P04801	2019	2019-Q3	2019-07	Pre-pandemic	Cytology (Pap)	30-34	31	Not performed	Satisfactory	NILM	NILM	Negative (NILM)	
P04802	2019	2019-Q3	2019-07	Pre-pandemic	Cytology (Pap)	30-34	34	Not performed	Satisfactory	Positive	HSIL	High-grade squamous	Candida
P04803	2019	2019-Q3	2019-07	Pre-pandemic	Cytology (Pap)	35-39	37	Not performed	Satisfactory	NILM	NILM	Negative (NILM)	
P04804	2019	2019-Q3	2019-07	Pre-pandemic	Cytology (Pap)	35-39	39	Not performed	Satisfactory	NILM	NILM	Negative (NILM)	
P04805	2019	2019-Q3	2019-07	Pre-pandemic	Cytology (Pap)	50-54	51	Not performed	Satisfactory	NILM	NILM	Negative (NILM)	
P04806	2019	2019-Q3	2019-07	Pre-pandemic	Cytology (Pap)	30-34	31	Not performed	Satisfactory	NILM	NILM	Negative (NILM)	
P04807	2019	2019-Q3	2019-07	Pre-pandemic	Cytology (Pap)	45-49	48	Not performed	Satisfactory	NILM	NILM	Negative (NILM)	
P04808	2019	2019-Q3	2019-07	Pre-pandemic	Cytology (Pap)	35-39	38	Not performed	Satisfactory	NILM	NILM	Negative (NILM)	
P04809	2019	2019-Q3	2019-07	Pre-pandemic	Cytology (Pap)	40-44	41	Not performed	Satisfactory	NILM	NILM	Negative (NILM)	
P04810	2019	2019-Q3	2019-07	Pre-pandemic	Cytology (Pap)	35-39	36	Not performed	Satisfactory	NILM	NILM	Negative (NILM)	
P04811	2019	2019-Q3	2019-07	Pre-pandemic	Cytology (Pap)	50-54	50	Not performed	Satisfactory	NILM	NILM	Negative (NILM)	
P04812	2019	2019-Q3	2019-07	Pre-pandemic	Cytology (Pap)	40-44	41	Not performed	Satisfactory	NILM	NILM	Negative (NILM)	
P04813	2019	2019-Q3	2019-07	Pre-pandemic	Cytology (Pap)	35-39	38	Not performed	Satisfactory	Positive	ASC-US	Low-grade / equivocal squamous	
P04814	2019	2019-Q3	2019-07	Pre-pandemic	Cytology (Pap)	40-44	41	Not performed	Satisfactory	NILM	NILM	Negative (NILM)	
P04815	2019	2019-Q3	2019-07	Pre-pandemic	Cytology (Pap)	25-29	27	Not performed	Satisfactory	NILM	NILM	Negative (NILM)	
P04816	2019	2019-Q3	2019-07	Pre-pandemic	Cytology (Pap)	60-64	63	Not performed	Satisfactory	NILM	NILM	Negative (NILM)	
P04817	2019	2019-Q3	2019-07	Pre-pandemic	Cytology (Pap)	45-49	47	Not performed	Satisfactory	NILM	NILM	Negative (NILM)	
P04818	2019	2019-Q3	2019-07	Pre-pandemic	Cytology (Pap)	35-39	38	Not performed	Satisfactory	Positive	LSIL	Low-grade / equivocal squamous	
P04819	2019	2019-Q3	2019-07	Pre-pandemic	Cytology (Pap)	60-64	61	Not performed	Satisfactory	NILM	NILM	Negative (NILM)	
P04820	2019	2019-Q3	2019-07	Pre-pandemic	Cytology (Pap)	45-49	45	Not performed	Satisfactory	Positive	ASC-US	Low-grade / equivocal squamous	Candida
P04821	2019	2019-Q3	2019-07	Pre-pandemic	Cytology (Pap)	60-64	62	Not performed	Satisfactory	NILM	NILM	Negative (NILM)	
P04822	2019	2019-Q3	2019-07	Pre-pandemic	Cytology (Pap)	55-59	55	Not performed	Satisfactory	NILM	NILM	Negative (NILM)	
P04823	2019	2019-Q3	2019-07	Pre-pandemic	Cytology (Pap)	35-39	35	Not performed	Satisfactory	NILM	NILM	Negative (NILM)	
P04824	2019	2019-Q3	2019-07	Pre-pandemic	Cytology (Pap)	60-64	61	Not performed	Satisfactory	NILM	NILM	Negative (NILM)	
P04825	2019	2019-Q3	2019-07	Pre-pandemic	Cytology (Pap)	50-54	51	Not performed	Satisfactory	NILM	NILM	Negative (NILM)	
P04826	2019	2019-Q3	2019-07	Pre-pandemic	Cytology (Pap)	25-29	26	Not performed	Satisfactory	Positive	LSIL	Low-grade / equivocal squamous	
P04827	2019	2019-Q3	2019-07	Pre-pandemic	Cytology (Pap)	60-64	63	Not performed	Satisfactory	NILM	NILM	Negative (NILM)	
P04828	2019	2019-Q3	2019-07	Pre-pandemic	Cytology (Pap)	50-54	50	Not performed	Satisfactory	NILM	NILM	Negative (NILM)	
P04829	2019	2019-Q3	2019-07	Pre-pandemic	Cytology (Pap)	25-29	27	Not performed	Satisfactory	NILM	NILM	Negative (NILM)	
P04830	2019	2019-Q3	2019-07	Pre-pandemic	Cytology (Pap)	50-54	50	Not performed	Satisfactory	NILM	NILM	Negative (NILM)	
P04831	2019	2019-Q3	2019-07	Pre-pandemic	Cytology (Pap)	60-64	62	Not performed	Satisfactory	NILM	NILM	Negative (NILM)	
P04832	2019	2019-Q3	2019-07	Pre-pandemic	Cytology (Pap)	40-44	41	Not performed	Satisfactory	NILM	NILM	Negative (NILM)	
P04833	2019	2019-Q3	2019-07	Pre-pandemic	Cytology (Pap)	45-49	47	Not performed	Satisfactory	NILM	NILM	Negative (NILM)	
P04834	2019	2019-Q3	2019-07	Pre-pandemic	Cytology (Pap)	50-54	51	Not performed	Satisfactory	NILM	NILM	Negative (NILM)	
P04835	2019	2019-Q3	2019-07	Pre-pandemic	Cytology (Pap)	30-34	33	Not performed	Satisfactory	NILM	NILM	Negative (NILM)	
P04836	2019	2019-Q3	2019-07	Pre-pandemic	Cytology (Pap)	45-49	46	Not performed	Unsatisfactory	Unsatisfactory	Unsatisfactory	Unsatisfactory	
P04837	2019	2019-Q3	2019-07	Pre-pandemic	Cytology (Pap)	35-39	37	Not performed	Satisfactory	NILM	NILM	Negative (NILM)	
P04838	2019	2019-Q3	2019-07	Pre-pandemic	Cytology (Pap)	60-64	61	Not performed	Satisfactory	NILM	NILM	Negative (NILM)	
P04839	2019	2019-Q3	2019-07	Pre-pandemic	Cytology (Pap)	35-39	35	Not performed	Satisfactory	NILM	NILM	Negative (NILM)	
P04840	2019	2019-Q3	2019-07	Pre-pandemic	Cytology (Pap)	40-44	44	Not performed	Satisfactory	NILM	NILM	Negative (NILM)	
P04841	2019	2019-Q3	2019-07	Pre-pandemic	Cytology (Pap)	45-49	47	Not performed	Satisfactory	NILM	NILM	Negative (NILM)	
P04842	2019	2019-Q3	2019-07	Pre-pandemic	Cytology (Pap)	45-49	47	Not performed	Satisfactory	Positive	HSIL	High-grade squamous	
P04843	2019	2019-Q3	2019-07	Pre-pandemic	Cytology (Pap)	35-39	35	Not performed	Satisfactory	NILM	NILM	Negative (NILM)	
P04844	2019	2019-Q3	2019-07	Pre-pandemic	Cytology (Pap)	55-59	59	Not performed	Satisfactory	NILM	NILM	Negative (NILM)	Trichomonas
P04845	2019	2019-Q3	2019-07	Pre-pandemic	Cytology (Pap)	40-44	44	Not performed	Satisfactory	NILM	NILM	Negative (NILM)	
P04846	2019	2019-Q3	2019-07	Pre-pandemic	Cytology (Pap)	40-44	40	Not performed	Satisfactory	NILM	NILM	Negative (NILM)	
P04847	2019	2019-Q3	2019-07	Pre-pandemic	Cytology (Pap)	45-49	47	Not performed	Satisfactory	NILM	NILM	Negative (NILM)	
P04848	2019	2019-Q3	2019-07	Pre-pandemic	Cytology (Pap)	35-39	35	Not performed	Satisfactory	NILM	NILM	Negative (NILM)	
P04849	2019	2019-Q3	2019-07	Pre-pandemic	Cytology (Pap)	30-34	32	Not performed	Satisfactory	NILM	NILM	Negative (NILM)	
P04850	2019	2019-Q3	2019-07	Pre-pandemic	Cytology (Pap)	50-54	54	Not performed	Satisfactory	NILM	NILM	Negative (NILM)	
P04851	2019	2019-Q3	2019-07	Pre-pandemic	Cytology (Pap)	30-34	34	Not performed	Satisfactory	Positive	ASC-H	High-grade squamous	
P04852	2019	2019-Q3	2019-07	Pre-pandemic	Cytology (Pap)	35-39	38	Not performed	Satisfactory	NILM	NILM	Negative (NILM)	
P04853	2019	2019-Q3	2019-07	Pre-pandemic	Cytology (Pap)	35-39	36	Not performed	Satisfactory	NILM	NILM	Negative (NILM)	
P04854	2019	2019-Q3	2019-07	Pre-pandemic	Cytology (Pap)	30-34	32	Not performed	Satisfactory	NILM	NILM	Negative (NILM)	
P04855	2019	2019-Q3	2019-07	Pre-pandemic	Cytology (Pap)	40-44	40	Not performed	Satisfactory	NILM	NILM	Negative (NILM)	
P04856	2019	2019-Q3	2019-07	Pre-pandemic	Cytology (Pap)	50-54	51	Not performed	Satisfactory	NILM	NILM	Negative (NILM)	
P04857	2019	2019-Q3	2019-07	Pre-pandemic	Cytology (Pap)	35-39	37	Not performed	Satisfactory	Positive	LSIL	Low-grade / equivocal squamous	
P04858	2019	2019-Q3	2019-07	Pre-pandemic	Cytology (Pap)	55-59	59	Not performed	Satisfactory	NILM	NILM	Negative (NILM)	
P04859	2019	2019-Q3	2019-07	Pre-pandemic	Cytology (Pap)	45-49	45	Not performed	Satisfactory	NILM	NILM	Negative (NILM)	
P04860	2019	2019-Q3	2019-07	Pre-pandemic	Cytology (Pap)	40-44	44	Not performed	Satisfactory	NILM	NILM	Negative (NILM)	
P04861	2019	2019-Q3	2019-07	Pre-pandemic	Cytology (Pap)	60-64	64	Not performed	Unsatisfactory	Unsatisfactory	Unsatisfactory	Unsatisfactory	
P04862	2019	2019-Q3	2019-07	Pre-pandemic	Cytology (Pap)	55-59	56	Not performed	Satisfactory	NILM	NILM	Negative (NILM)	
P04863	2019	2019-Q3	2019-07	Pre-pandemic	Cytology (Pap)	50-54	54	Not performed	Satisfactory	NILM	NILM	Negative (NILM)	
P04864	2019	2019-Q3	2019-07	Pre-pandemic	Cytology (Pap)	45-49	48	Not performed	Satisfactory	NILM	NILM	Negative (NILM)	
P04865	2019	2019-Q3	2019-07	Pre-pandemic	Cytology (Pap)	40-44	42	Not performed	Satisfactory	NILM	NILM	Negative (NILM)	
P04866	2019	2019-Q3	2019-07	Pre-pandemic	Cytology (Pap)	40-44	43	Not performed	Satisfactory	NILM	NILM	Negative (NILM)	
P04867	2019	2019-Q3	2019-07	Pre-pandemic	Cytology (Pap)	35-39	38	Not performed	Satisfactory	NILM	NILM	Negative (NILM)	Gardnerella
P04868	2019	2019-Q3	2019-07	Pre-pandemic	Cytology (Pap)	60-64	63	Not performed	Satisfactory	Positive	ASC-US	Low-grade / equivocal squamous	
P04869	2019	2019-Q3	2019-07	Pre-pandemic	Cytology (Pap)	50-54	52	Not performed	Satisfactory	NILM	NILM	Negative (NILM)	
P04870	2019	2019-Q3	2019-07	Pre-pandemic	Cytology (Pap)	40-44	41	Not performed	Satisfactory	NILM	NILM	Negative (NILM)	
P04871	2019	2019-Q3	2019-07	Pre-pandemic	Cytology (Pap)	45-49	49	Not performed	Satisfactory	Positive	ASC-US	Low-grade / equivocal squamous	
P04872	2019	2019-Q3	2019-07	Pre-pandemic	Cytology (Pap)	40-44	43	Not performed	Satisfactory	NILM	NILM	Negative (NILM)	Candida
P04873	2019	2019-Q3	2019-07	Pre-pandemic	Cytology (Pap)	30-34	30	Not performed	Satisfactory	NILM	NILM	Negative (NILM)	Gardnerella
P04874	2019	2019-Q3	2019-07	Pre-pandemic	Cytology (Pap)	35-39	36	Not performed	Satisfactory	Positive	HSIL	High-grade squamous	
P04875	2019	2019-Q3	2019-07	Pre-pandemic	Cytology (Pap)	45-49	48	Not performed	Satisfactory	NILM	NILM	Negative (NILM)	
P04876	2019	2019-Q3	2019-07	Pre-pandemic	Cytology (Pap)	55-59	55	Not performed	Satisfactory	NILM	NILM	Negative (NILM)	
P04877	2019	2019-Q3	2019-07	Pre-pandemic	Cytology (Pap)	25-29	28	Not performed	Satisfactory	NILM	NILM	Negative (NILM)	
P04878	2019	2019-Q3	2019-07	Pre-pandemic	Cytology (Pap)	50-54	52	Not performed	Satisfactory	NILM	NILM	Negative (NILM)	

patient_id	year	quarter	month	period	primary_test	age_group	age_years	hpv_result	adequacy	result	bethesda	grade	infection
P04879	2019	2019-Q3	2019-07	Pre-pandemic	Cytology (Pap)	45-49	46	Not performed	Satisfactory	Positive	LSIL	Low-grade / equivocal squamous	
P04880	2019	2019-Q3	2019-07	Pre-pandemic	Cytology (Pap)	60-64	62	Not performed	Satisfactory	NILM	NILM	Negative (NILM)	
P04881	2019	2019-Q3	2019-07	Pre-pandemic	Cytology (Pap)	55-59	56	Not performed	Satisfactory	NILM	NILM	Negative (NILM)	
P04882	2019	2019-Q3	2019-07	Pre-pandemic	Cytology (Pap)	40-44	44	Not performed	Satisfactory	NILM	NILM	Negative (NILM)	
P04883	2019	2019-Q3	2019-07	Pre-pandemic	Cytology (Pap)	40-44	43	Not performed	Satisfactory	NILM	NILM	Negative (NILM)	
P04884	2019	2019-Q3	2019-07	Pre-pandemic	Cytology (Pap)	30-34	31	Not performed	Satisfactory	NILM	NILM	Negative (NILM)	
P04885	2019	2019-Q3	2019-07	Pre-pandemic	Cytology (Pap)	55-59	57	Not performed	Satisfactory	NILM	NILM	Negative (NILM)	
P04886	2019	2019-Q3	2019-07	Pre-pandemic	Cytology (Pap)	40-44	43	Not performed	Satisfactory	NILM	NILM	Negative (NILM)	
P04887	2019	2019-Q3	2019-07	Pre-pandemic	Cytology (Pap)	55-59	55	Not performed	Satisfactory	NILM	NILM	Negative (NILM)	
P04888	2019	2019-Q3	2019-07	Pre-pandemic	Cytology (Pap)	40-44	42	Not performed	Satisfactory	NILM	NILM	Negative (NILM)	
P04889	2019	2019-Q3	2019-07	Pre-pandemic	Cytology (Pap)	35-39	39	Not performed	Satisfactory	NILM	NILM	Negative (NILM)	
P04890	2019	2019-Q3	2019-07	Pre-pandemic	Cytology (Pap)	35-39	37	Not performed	Satisfactory	Positive	ASC-US	Low-grade / equivocal squamous	
P04891	2019	2019-Q3	2019-07	Pre-pandemic	Cytology (Pap)	40-44	44	Not performed	Unsatisfactory	Unsatisfactory	Unsatisfactory	Unsatisfactory	
P04892	2019	2019-Q3	2019-07	Pre-pandemic	Cytology (Pap)	35-39	36	Not performed	Satisfactory	NILM	NILM	Negative (NILM)	
P04893	2019	2019-Q3	2019-07	Pre-pandemic	Cytology (Pap)	40-44	42	Not performed	Satisfactory	NILM	NILM	Negative (NILM)	
P04894	2019	2019-Q3	2019-07	Pre-pandemic	Cytology (Pap)	35-39	35	Not performed	Satisfactory	Positive	ASC-US	Low-grade / equivocal squamous	
P04895	2019	2019-Q3	2019-07	Pre-pandemic	Cytology (Pap)	45-49	48	Not performed	Satisfactory	NILM	NILM	Negative (NILM)	
P04896	2019	2019-Q3	2019-07	Pre-pandemic	Cytology (Pap)	45-49	46	Not performed	Satisfactory	NILM	NILM	Negative (NILM)	
P04897	2019	2019-Q3	2019-07	Pre-pandemic	Cytology (Pap)	25-29	28	Not performed	Satisfactory	NILM	NILM	Negative (NILM)	
P04898	2019	2019-Q3	2019-07	Pre-pandemic	Cytology (Pap)	40-44	42	Not performed	Satisfactory	NILM	NILM	Negative (NILM)	
P04899	2019	2019-Q3	2019-07	Pre-pandemic	Cytology (Pap)	40-44	42	Not performed	Satisfactory	NILM	NILM	Negative (NILM)	
P04900	2019	2019-Q3	2019-07	Pre-pandemic	Cytology (Pap)	35-39	38	Not performed	Satisfactory	NILM	NILM	Negative (NILM)	
P04901	2019	2019-Q3	2019-07	Pre-pandemic	Cytology (Pap)	40-44	41	Not performed	Satisfactory	NILM	NILM	Negative (NILM)	
P04902	2019	2019-Q3	2019-07	Pre-pandemic	Cytology (Pap)	50-54	52	Not performed	Satisfactory	NILM	NILM	Negative (NILM)	
P04903	2019	2019-Q3	2019-07	Pre-pandemic	Cytology (Pap)	45-49	48	Not performed	Satisfactory	NILM	NILM	Negative (NILM)	
P04904	2019	2019-Q3	2019-07	Pre-pandemic	Cytology (Pap)	45-49	47	Not performed	Satisfactory	NILM	NILM	Negative (NILM)	
P04905	2019	2019-Q3	2019-07	Pre-pandemic	Cytology (Pap)	40-44	42	Not performed	Satisfactory	NILM	NILM	Negative (NILM)	
P04906	2019	2019-Q3	2019-07	Pre-pandemic	Cytology (Pap)	40-44	41	Not performed	Satisfactory	NILM	NILM	Negative (NILM)	
P04907	2019	2019-Q3	2019-07	Pre-pandemic	Cytology (Pap)	60-64	61	Not performed	Satisfactory	NILM	NILM	Negative (NILM)	
P04908	2019	2019-Q3	2019-07	Pre-pandemic	Cytology (Pap)	60-64	63	Not performed	Satisfactory	NILM	NILM	Negative (NILM)	
P04909	2019	2019-Q3	2019-07	Pre-pandemic	Cytology (Pap)	25-29	27	Not performed	Satisfactory	NILM	NILM	Negative (NILM)	
P04910	2019	2019-Q3	2019-07	Pre-pandemic	Cytology (Pap)	50-54	54	Not performed	Satisfactory	NILM	NILM	Negative (NILM)	
P04911	2019	2019-Q3	2019-07	Pre-pandemic	Cytology (Pap)	50-54	53	Not performed	Satisfactory	NILM	NILM	Negative (NILM)	Candida
P04912	2019	2019-Q3	2019-07	Pre-pandemic	Cytology (Pap)	40-44	41	Not performed	Satisfactory	NILM	NILM	Negative (NILM)	
P04913	2019	2019-Q3	2019-07	Pre-pandemic	Cytology (Pap)	45-49	47	Not performed	Satisfactory	NILM	NILM	Negative (NILM)	
P04914	2019	2019-Q3	2019-07	Pre-pandemic	Cytology (Pap)	55-59	55	Not performed	Satisfactory	NILM	NILM	Negative (NILM)	
P04915	2019	2019-Q3	2019-07	Pre-pandemic	Cytology (Pap)	50-54	50	Not performed	Satisfactory	NILM	NILM	Negative (NILM)	
P04916	2019	2019-Q3	2019-07	Pre-pandemic	Cytology (Pap)	45-49	46	Not performed	Satisfactory	NILM	NILM	Negative (NILM)	
P04917	2019	2019-Q3	2019-07	Pre-pandemic	Cytology (Pap)	35-39	37	Not performed	Satisfactory	NILM	NILM	Negative (NILM)	
P04918	2019	2019-Q3	2019-07	Pre-pandemic	Cytology (Pap)	35-39	39	Not performed	Satisfactory	NILM	NILM	Negative (NILM)	
P04919	2019	2019-Q3	2019-07	Pre-pandemic	Cytology (Pap)	45-49	47	Not performed	Satisfactory	NILM	NILM	Negative (NILM)	
P04920	2019	2019-Q3	2019-07	Pre-pandemic	Cytology (Pap)	45-49	49	Not performed	Satisfactory	NILM	NILM	Negative (NILM)	
P04921	2019	2019-Q3	2019-07	Pre-pandemic	Cytology (Pap)	60-64	64	Not performed	Satisfactory	NILM	NILM	Negative (NILM)	
P04922	2019	2019-Q3	2019-07	Pre-pandemic	Cytology (Pap)	45-49	49	Not performed	Satisfactory	NILM	NILM	Negative (NILM)	
P04923	2019	2019-Q3	2019-07	Pre-pandemic	Cytology (Pap)	30-34	34	Not performed	Satisfactory	NILM	NILM	Negative (NILM)	
P04924	2019	2019-Q3	2019-07	Pre-pandemic	Cytology (Pap)	40-44	41	Not performed	Satisfactory	NILM	NILM	Negative (NILM)	
P04925	2019	2019-Q3	2019-07	Pre-pandemic	Cytology (Pap)	55-59	57	Not performed	Satisfactory	NILM	NILM	Negative (NILM)	Gardnerella
P04926	2019	2019-Q3	2019-07	Pre-pandemic	Cytology (Pap)	25-29	28	Not performed	Satisfactory	NILM	NILM	Negative (NILM)	
P04927	2019	2019-Q3	2019-07	Pre-pandemic	Cytology (Pap)	45-49	49	Not performed	Satisfactory	NILM	NILM	Negative (NILM)	Candida
P04928	2019	2019-Q3	2019-07	Pre-pandemic	Cytology (Pap)	35-39	35	Not performed	Satisfactory	NILM	NILM	Negative (NILM)	
P04929	2019	2019-Q3	2019-07	Pre-pandemic	Cytology (Pap)	25-29	28	Not performed	Satisfactory	Positive	HSIL	High-grade squamous	
P04930	2019	2019-Q3	2019-07	Pre-pandemic	Cytology (Pap)	35-39	37	Not performed	Satisfactory	NILM	NILM	Negative (NILM)	
P04931	2019	2019-Q3	2019-07	Pre-pandemic	Cytology (Pap)	40-44	44	Not performed	Satisfactory	NILM	NILM	Negative (NILM)	
P04932	2019	2019-Q3	2019-07	Pre-pandemic	Cytology (Pap)	45-49	48	Not performed	Satisfactory	Positive	ASC-US	Low-grade / equivocal squamous	
P04933	2019	2019-Q3	2019-08	Pre-pandemic	Cytology (Pap)	55-59	55	Not performed	Satisfactory	NILM	NILM	Negative (NILM)	
P04934	2019	2019-Q3	2019-08	Pre-pandemic	Cytology (Pap)	40-44	44	Not performed	Satisfactory	NILM	NILM	Negative (NILM)	
P04935	2019	2019-Q3	2019-08	Pre-pandemic	Cytology (Pap)	60-64	60	Not performed	Satisfactory	NILM	NILM	Negative (NILM)	
P04936	2019	2019-Q3	2019-08	Pre-pandemic	Cytology (Pap)	40-44	42	Not performed	Satisfactory	NILM	NILM	Negative (NILM)	
P04937	2019	2019-Q3	2019-08	Pre-pandemic	Cytology (Pap)	40-44	42	Not performed	Satisfactory	NILM	NILM	Negative (NILM)	
P04938	2019	2019-Q3	2019-08	Pre-pandemic	Cytology (Pap)	55-59	58	Not performed	Satisfactory	NILM	NILM	Negative (NILM)	
P04939	2019	2019-Q3	2019-08	Pre-pandemic	Cytology (Pap)	30-34	34	Not performed	Satisfactory	NILM	NILM	Negative (NILM)	
P04940	2019	2019-Q3	2019-08	Pre-pandemic	Cytology (Pap)	45-49	45	Not performed	Satisfactory	Positive	ASC-US	Low-grade / equivocal squamous	
P04941	2019	2019-Q3	2019-08	Pre-pandemic	Cytology (Pap)	40-44	43	Not performed	Satisfactory	NILM	NILM	Negative (NILM)	
P04942	2019	2019-Q3	2019-08	Pre-pandemic	Cytology (Pap)	40-44	42	Not performed	Satisfactory	Positive	LSIL	Low-grade / equivocal squamous	
P04943	2019	2019-Q3	2019-08	Pre-pandemic	Cytology (Pap)	40-44	40	Not performed	Satisfactory	NILM	NILM	Negative (NILM)	Candida
P04944	2019	2019-Q3	2019-08	Pre-pandemic	Cytology (Pap)	35-39	37	Not performed	Unsatisfactory	Unsatisfactory	Unsatisfactory	Unsatisfactory	
P04945	2019	2019-Q3	2019-08	Pre-pandemic	Cytology (Pap)	40-44	42	Not performed	Satisfactory	NILM	NILM	Negative (NILM)	
P04946	2019	2019-Q3	2019-08	Pre-pandemic	Cytology (Pap)	40-44	41	Not performed	Satisfactory	NILM	NILM	Negative (NILM)	
P04947	2019	2019-Q3	2019-08	Pre-pandemic	Cytology (Pap)	45-49	47	Not performed	Satisfactory	NILM	NILM	Negative (NILM)	
P04948	2019	2019-Q3	2019-08	Pre-pandemic	Cytology (Pap)	35-39	38	Not performed	Satisfactory	NILM	NILM	Negative (NILM)	
P04949	2019	2019-Q3	2019-08	Pre-pandemic	Cytology (Pap)	55-59	57	Not performed	Satisfactory	NILM	NILM	Negative (NILM)	
P04950	2019	2019-Q3	2019-08	Pre-pandemic	Cytology (Pap)	50-54	54	Not performed	Satisfactory	NILM	NILM	Negative (NILM)	
P04951	2019	2019-Q3	2019-08	Pre-pandemic	Cytology (Pap)	40-44	42	Not performed	Satisfactory	NILM	NILM	Negative (NILM)	
P04952	2019	2019-Q3	2019-08	Pre-pandemic	Cytology (Pap)	40-44	41	Not performed	Satisfactory	NILM	NILM	Negative (NILM)	
P04953	2019	2019-Q3	2019-08	Pre-pandemic	Cytology (Pap)	35-39	39	Not performed	Unsatisfactory	Unsatisfactory	Unsatisfactory	Unsatisfactory	
P04954	2019	2019-Q3	2019-08	Pre-pandemic	Cytology (Pap)	40-44	40	Not performed	Satisfactory	NILM	NILM	Negative (NILM)	
P04955	2019	2019-Q3	2019-08	Pre-pandemic	Cytology (Pap)	35-39	39	Not performed	Satisfactory	NILM	NILM	Negative (NILM)	
P04956	2019	2019-Q3	2019-08	Pre-pandemic	Cytology (Pap)	45-49	45	Not performed	Satisfactory	Positive	LSIL	Low-grade / equivocal squamous	
P04957	2019	2019-Q3	2019-08	Pre-pandemic	Cytology (Pap)	25-29	26	Not performed	Satisfactory	Positive	LSIL	Low-grade / equivocal squamous	
P04958	2019	2019-Q3	2019-08	Pre-pandemic	Cytology (Pap)	25-29	27	Not performed	Satisfactory	Positive	LSIL	Low-grade / equivocal squamous	

patient_id	year	quarter	month	period	primary_test	age_group	age_years	hpv_result	adequacy	result	bethesda	grade	infection
P04959	2019	2019-Q3	2019-08	Pre-pandemic	Cytology (Pap)	45-49	47	Not performed	Satisfactory	NILM	NILM	Negative (NILM)	
P04960	2019	2019-Q3	2019-08	Pre-pandemic	Cytology (Pap)	60-64	61	Not performed	Unsatisfactory	Unsatisfactory	Unsatisfactory	Unsatisfactory	
P04961	2019	2019-Q3	2019-08	Pre-pandemic	Cytology (Pap)	45-49	45	Not performed	Satisfactory	NILM	NILM	Negative (NILM)	
P04962	2019	2019-Q3	2019-08	Pre-pandemic	Cytology (Pap)	55-59	58	Not performed	Satisfactory	Positive	ASC-US	Low-grade / equivocal squamous	
P04963	2019	2019-Q3	2019-08	Pre-pandemic	Cytology (Pap)	45-49	48	Not performed	Satisfactory	Positive	LSIL	Low-grade / equivocal squamous	
P04964	2019	2019-Q3	2019-08	Pre-pandemic	Cytology (Pap)	40-44	41	Not performed	Satisfactory	NILM	NILM	Negative (NILM)	
P04965	2019	2019-Q3	2019-08	Pre-pandemic	Cytology (Pap)	25-29	26	Not performed	Satisfactory	NILM	NILM	Negative (NILM)	
P04966	2019	2019-Q3	2019-08	Pre-pandemic	Cytology (Pap)	55-59	59	Not performed	Satisfactory	NILM	NILM	Negative (NILM)	
P04967	2019	2019-Q3	2019-08	Pre-pandemic	Cytology (Pap)	30-34	34	Not performed	Satisfactory	Positive	ASC-US	Low-grade / equivocal squamous	
P04968	2019	2019-Q3	2019-08	Pre-pandemic	Cytology (Pap)	50-54	51	Not performed	Satisfactory	NILM	NILM	Negative (NILM)	
P04969	2019	2019-Q3	2019-08	Pre-pandemic	Cytology (Pap)	55-59	58	Not performed	Satisfactory	NILM	NILM	Negative (NILM)	
P04970	2019	2019-Q3	2019-08	Pre-pandemic	Cytology (Pap)	30-34	32	Not performed	Satisfactory	NILM	NILM	Negative (NILM)	
P04971	2019	2019-Q3	2019-08	Pre-pandemic	Cytology (Pap)	50-54	51	Not performed	Satisfactory	NILM	NILM	Negative (NILM)	
P04972	2019	2019-Q3	2019-08	Pre-pandemic	Cytology (Pap)	35-39	39	Not performed	Satisfactory	NILM	NILM	Negative (NILM)	
P04973	2019	2019-Q3	2019-08	Pre-pandemic	Cytology (Pap)	45-49	47	Not performed	Unsatisfactory	Unsatisfactory	Unsatisfactory	Unsatisfactory	
P04974	2019	2019-Q3	2019-08	Pre-pandemic	Cytology (Pap)	50-54	50	Not performed	Satisfactory	NILM	NILM	Negative (NILM)	
P04975	2019	2019-Q3	2019-08	Pre-pandemic	Cytology (Pap)	40-44	41	Not performed	Satisfactory	NILM	NILM	Negative (NILM)	
P04976	2019	2019-Q3	2019-08	Pre-pandemic	Cytology (Pap)	35-39	37	Not performed	Satisfactory	NILM	NILM	Negative (NILM)	
P04977	2019	2019-Q3	2019-08	Pre-pandemic	Cytology (Pap)	40-44	42	Not performed	Satisfactory	Positive	ASC-US	Low-grade / equivocal squamous	
P04978	2019	2019-Q3	2019-08	Pre-pandemic	Cytology (Pap)	35-39	35	Not performed	Satisfactory	NILM	NILM	Negative (NILM)	
P04979	2019	2019-Q3	2019-08	Pre-pandemic	Cytology (Pap)	45-49	47	Not performed	Satisfactory	Positive	LSIL	Low-grade / equivocal squamous	
P04980	2019	2019-Q3	2019-08	Pre-pandemic	Cytology (Pap)	30-34	32	Not performed	Satisfactory	NILM	NILM	Negative (NILM)	
P04981	2019	2019-Q3	2019-08	Pre-pandemic	Cytology (Pap)	50-54	54	Not performed	Satisfactory	NILM	NILM	Negative (NILM)	
P04982	2019	2019-Q3	2019-08	Pre-pandemic	Cytology (Pap)	40-44	42	Not performed	Satisfactory	NILM	NILM	Negative (NILM)	Trichomonas
P04983	2019	2019-Q3	2019-08	Pre-pandemic	Cytology (Pap)	50-54	50	Not performed	Satisfactory	NILM	NILM	Negative (NILM)	
P04984	2019	2019-Q3	2019-08	Pre-pandemic	Cytology (Pap)	40-44	42	Not performed	Satisfactory	NILM	NILM	Negative (NILM)	Trichomonas
P04985	2019	2019-Q3	2019-08	Pre-pandemic	Cytology (Pap)	55-59	57	Not performed	Satisfactory	Positive	ASC-US	Low-grade / equivocal squamous	
P04986	2019	2019-Q3	2019-08	Pre-pandemic	Cytology (Pap)	50-54	51	Not performed	Satisfactory	NILM	NILM	Negative (NILM)	
P04987	2019	2019-Q3	2019-08	Pre-pandemic	Cytology (Pap)	40-44	44	Not performed	Satisfactory	NILM	NILM	Negative (NILM)	
P04988	2019	2019-Q3	2019-08	Pre-pandemic	Cytology (Pap)	45-49	46	Not performed	Satisfactory	NILM	NILM	Negative (NILM)	
P04989	2019	2019-Q3	2019-08	Pre-pandemic	Cytology (Pap)	35-39	37	Not performed	Satisfactory	NILM	NILM	Negative (NILM)	
P04990	2019	2019-Q3	2019-08	Pre-pandemic	Cytology (Pap)	45-49	45	Not performed	Satisfactory	NILM	NILM	Negative (NILM)	
P04991	2019	2019-Q3	2019-08	Pre-pandemic	Cytology (Pap)	50-54	52	Not performed	Satisfactory	NILM	NILM	Negative (NILM)	
P04992	2019	2019-Q3	2019-08	Pre-pandemic	Cytology (Pap)	25-29	29	Not performed	Satisfactory	NILM	NILM	Negative (NILM)	
P04993	2019	2019-Q3	2019-08	Pre-pandemic	Cytology (Pap)	30-34	30	Not performed	Satisfactory	Positive	ASC-US	Low-grade / equivocal squamous	
P04994	2019	2019-Q3	2019-08	Pre-pandemic	Cytology (Pap)	45-49	45	Not performed	Satisfactory	NILM	NILM	Negative (NILM)	
P04995	2019	2019-Q3	2019-08	Pre-pandemic	Cytology (Pap)	45-49	47	Not performed	Satisfactory	NILM	NILM	Negative (NILM)	
P04996	2019	2019-Q3	2019-08	Pre-pandemic	Cytology (Pap)	40-44	40	Not performed	Satisfactory	NILM	NILM	Negative (NILM)	
P04997	2019	2019-Q3	2019-08	Pre-pandemic	Cytology (Pap)	55-59	57	Not performed	Satisfactory	NILM	NILM	Negative (NILM)	
P04998	2019	2019-Q3	2019-08	Pre-pandemic	Cytology (Pap)	35-39	36	Not performed	Satisfactory	NILM	NILM	Negative (NILM)	
P04999	2019	2019-Q3	2019-08	Pre-pandemic	Cytology (Pap)	30-34	34	Not performed	Satisfactory	NILM	NILM	Negative (NILM)	
P05000	2019	2019-Q3	2019-08	Pre-pandemic	Cytology (Pap)	30-34	32	Not performed	Satisfactory	NILM	NILM	Negative (NILM)	
P05001	2019	2019-Q3	2019-08	Pre-pandemic	Cytology (Pap)	50-54	53	Not performed	Satisfactory	NILM	NILM	Negative (NILM)	
P05002	2019	2019-Q3	2019-08	Pre-pandemic	Cytology (Pap)	50-54	53	Not performed	Satisfactory	NILM	NILM	Negative (NILM)	
P05003	2019	2019-Q3	2019-08	Pre-pandemic	Cytology (Pap)	40-44	43	Not performed	Satisfactory	NILM	NILM	Negative (NILM)	
P05004	2019	2019-Q3	2019-08	Pre-pandemic	Cytology (Pap)	40-44	42	Not performed	Satisfactory	NILM	NILM	Negative (NILM)	
P05005	2019	2019-Q3	2019-08	Pre-pandemic	Cytology (Pap)	50-54	52	Not performed	Satisfactory	NILM	NILM	Negative (NILM)	
P05006	2019	2019-Q3	2019-08	Pre-pandemic	Cytology (Pap)	55-59	57	Not performed	Satisfactory	NILM	NILM	Negative (NILM)	
P05007	2019	2019-Q3	2019-08	Pre-pandemic	Cytology (Pap)	60-64	61	Not performed	Satisfactory	Positive	ASC-US	Low-grade / equivocal squamous	Trichomonas
P05008	2019	2019-Q3	2019-08	Pre-pandemic	Cytology (Pap)	35-39	38	Not performed	Satisfactory	NILM	NILM	Negative (NILM)	
P05009	2019	2019-Q3	2019-08	Pre-pandemic	Cytology (Pap)	35-39	37	Not performed	Satisfactory	NILM	NILM	Negative (NILM)	
P05010	2019	2019-Q3	2019-08	Pre-pandemic	Cytology (Pap)	45-49	49	Not performed	Satisfactory	NILM	NILM	Negative (NILM)	
P05011	2019	2019-Q3	2019-08	Pre-pandemic	Cytology (Pap)	50-54	54	Not performed	Satisfactory	NILM	NILM	Negative (NILM)	
P05012	2019	2019-Q3	2019-08	Pre-pandemic	Cytology (Pap)	35-39	38	Not performed	Satisfactory	NILM	NILM	Negative (NILM)	
P05013	2019	2019-Q3	2019-08	Pre-pandemic	Cytology (Pap)	55-59	55	Not performed	Satisfactory	NILM	NILM	Negative (NILM)	
P05014	2019	2019-Q3	2019-08	Pre-pandemic	Cytology (Pap)	25-29	28	Not performed	Satisfactory	NILM	NILM	Negative (NILM)	
P05015	2019	2019-Q3	2019-08	Pre-pandemic	Cytology (Pap)	25-29	29	Not performed	Satisfactory	NILM	NILM	Negative (NILM)	
P05016	2019	2019-Q3	2019-08	Pre-pandemic	Cytology (Pap)	40-44	42	Not performed	Satisfactory	NILM	NILM	Negative (NILM)	
P05017	2019	2019-Q3	2019-08	Pre-pandemic	Cytology (Pap)	60-64	62	Not performed	Satisfactory	NILM	NILM	Negative (NILM)	
P05018	2019	2019-Q3	2019-08	Pre-pandemic	Cytology (Pap)	45-49	45	Not performed	Satisfactory	Positive	LSIL	Low-grade / equivocal squamous	
P05019	2019	2019-Q3	2019-08	Pre-pandemic	Cytology (Pap)	45-49	45	Not performed	Satisfactory	NILM	NILM	Negative (NILM)	
P05020	2019	2019-Q3	2019-08	Pre-pandemic	Cytology (Pap)	30-34	30	Not performed	Satisfactory	Positive	ASC-US	Low-grade / equivocal squamous	
P05021	2019	2019-Q3	2019-08	Pre-pandemic	Cytology (Pap)	60-64	64	Not performed	Satisfactory	NILM	NILM	Negative (NILM)	
P05022	2019	2019-Q3	2019-08	Pre-pandemic	Cytology (Pap)	50-54	53	Not performed	Satisfactory	NILM	NILM	Negative (NILM)	
P05023	2019	2019-Q3	2019-08	Pre-pandemic	Cytology (Pap)	50-54	50	Not performed	Satisfactory	NILM	NILM	Negative (NILM)	
P05024	2019	2019-Q3	2019-08	Pre-pandemic	Cytology (Pap)	40-44	41	Not performed	Satisfactory	NILM	NILM	Negative (NILM)	
P05025	2019	2019-Q3	2019-08	Pre-pandemic	Cytology (Pap)	45-49	47	Not performed	Satisfactory	NILM	NILM	Negative (NILM)	
P05026	2019	2019-Q3	2019-08	Pre-pandemic	Cytology (Pap)	45-49	47	Not performed	Satisfactory	NILM	NILM	Negative (NILM)	
P05027	2019	2019-Q3	2019-08	Pre-pandemic	Cytology (Pap)	45-49	45	Not performed	Satisfactory	NILM	NILM	Negative (NILM)	
P05028	2019	2019-Q3	2019-08	Pre-pandemic	Cytology (Pap)	40-44	41	Not performed	Satisfactory	NILM	NILM	Negative (NILM)	
P05029	2019	2019-Q3	2019-08	Pre-pandemic	Cytology (Pap)	30-34	30	Not performed	Satisfactory	NILM	NILM	Negative (NILM)	
P05030	2019	2019-Q3	2019-08	Pre-pandemic	Cytology (Pap)	50-54	51	Not performed	Satisfactory	NILM	NILM	Negative (NILM)	
P05031	2019	2019-Q3	2019-08	Pre-pandemic	Cytology (Pap)	30-34	33	Not performed	Satisfactory	NILM	NILM	Negative (NILM)	
P05032	2019	2019-Q3	2019-08	Pre-pandemic	Cytology (Pap)	50-54	53	Not performed	Satisfactory	NILM	NILM	Negative (NILM)	
P05033	2019	2019-Q3	2019-08	Pre-pandemic	Cytology (Pap)	40-44	42	Not performed	Unsatisfactory	Unsatisfactory	Unsatisfactory	Unsatisfactory	
P05034	2019	2019-Q3	2019-08	Pre-pandemic	Cytology (Pap)	35-39	37	Not performed	Satisfactory	NILM	NILM	Negative (NILM)	
P05035	2019	2019-Q3	2019-08	Pre-pandemic	Cytology (Pap)	45-49	47	Not performed	Satisfactory	NILM	NILM	Negative (NILM)	
P05036	2019	2019-Q3	2019-08	Pre-pandemic	Cytology (Pap)	40-44	43	Not performed	Satisfactory	NILM	NILM	Negative (NILM)	
P05037	2019	2019-Q3	2019-08	Pre-pandemic	Cytology (Pap)	30-34	32	Not performed	Satisfactory	NILM	NILM	Negative (NILM)	
P05038	2019	2019-Q3	2019-08	Pre-pandemic	Cytology (Pap)	45-49	47	Not performed	Satisfactory	NILM	NILM	Negative (NILM)	

patient_id	year	quarter	month	period	primary_test	age_group	age_years	hpv_result	adequacy	result	bethesda	grade	infection
P05039	2019	2019-Q3	2019-08	Pre-pandemic	Cytology (Pap)	40-44	43	Not performed	Satisfactory	NILM	NILM	Negative (NILM)	
P05040	2019	2019-Q3	2019-08	Pre-pandemic	Cytology (Pap)	30-34	32	Not performed	Satisfactory	NILM	NILM	Negative (NILM)	
P05041	2019	2019-Q3	2019-08	Pre-pandemic	Cytology (Pap)	45-49	45	Not performed	Satisfactory	Positive	ASC-H	High-grade squamous	
P05042	2019	2019-Q3	2019-08	Pre-pandemic	Cytology (Pap)	60-64	63	Not performed	Satisfactory	NILM	NILM	Negative (NILM)	
P05043	2019	2019-Q3	2019-08	Pre-pandemic	Cytology (Pap)	50-54	52	Not performed	Satisfactory	Positive	HSIL	High-grade squamous	
P05044	2019	2019-Q3	2019-08	Pre-pandemic	Cytology (Pap)	50-54	51	Not performed	Satisfactory	Positive	LSIL	Low-grade / equivocal squamous	
P05045	2019	2019-Q3	2019-08	Pre-pandemic	Cytology (Pap)	55-59	58	Not performed	Satisfactory	NILM	NILM	Negative (NILM)	
P05046	2019	2019-Q3	2019-08	Pre-pandemic	Cytology (Pap)	50-54	54	Not performed	Satisfactory	NILM	NILM	Negative (NILM)	
P05047	2019	2019-Q3	2019-08	Pre-pandemic	Cytology (Pap)	40-44	44	Not performed	Satisfactory	Positive	ASC-US	Low-grade / equivocal squamous	
P05048	2019	2019-Q3	2019-08	Pre-pandemic	Cytology (Pap)	50-54	50	Not performed	Satisfactory	NILM	NILM	Negative (NILM)	
P05049	2019	2019-Q3	2019-08	Pre-pandemic	Cytology (Pap)	40-44	41	Not performed	Satisfactory	NILM	NILM	Negative (NILM)	
P05050	2019	2019-Q3	2019-08	Pre-pandemic	Cytology (Pap)	45-49	47	Not performed	Satisfactory	NILM	NILM	Negative (NILM)	
P05051	2019	2019-Q3	2019-08	Pre-pandemic	Cytology (Pap)	40-44	42	Not performed	Satisfactory	Positive	ASC-US	Low-grade / equivocal squamous	
P05052	2019	2019-Q3	2019-08	Pre-pandemic	Cytology (Pap)	35-39	37	Not performed	Satisfactory	NILM	NILM	Negative (NILM)	
P05053	2019	2019-Q3	2019-08	Pre-pandemic	Cytology (Pap)	40-44	40	Not performed	Satisfactory	NILM	NILM	Negative (NILM)	
P05054	2019	2019-Q3	2019-08	Pre-pandemic	Cytology (Pap)	40-44	44	Not performed	Satisfactory	NILM	NILM	Negative (NILM)	
P05055	2019	2019-Q3	2019-08	Pre-pandemic	Cytology (Pap)	25-29	25	Not performed	Satisfactory	NILM	NILM	Negative (NILM)	
P05056	2019	2019-Q3	2019-08	Pre-pandemic	Cytology (Pap)	25-29	27	Not performed	Satisfactory	NILM	NILM	Negative (NILM)	
P05057	2019	2019-Q3	2019-08	Pre-pandemic	Cytology (Pap)	30-34	30	Not performed	Satisfactory	NILM	NILM	Negative (NILM)	
P05058	2019	2019-Q3	2019-08	Pre-pandemic	Cytology (Pap)	45-49	45	Not performed	Satisfactory	NILM	NILM	Negative (NILM)	
P05059	2019	2019-Q3	2019-08	Pre-pandemic	Cytology (Pap)	40-44	41	Not performed	Satisfactory	NILM	NILM	Negative (NILM)	
P05060	2019	2019-Q3	2019-08	Pre-pandemic	Cytology (Pap)	40-44	44	Not performed	Satisfactory	Positive	ASC-US	Low-grade / equivocal squamous	
P05061	2019	2019-Q3	2019-08	Pre-pandemic	Cytology (Pap)	45-49	47	Not performed	Satisfactory	NILM	NILM	Negative (NILM)	
P05062	2019	2019-Q3	2019-08	Pre-pandemic	Cytology (Pap)	25-29	28	Not performed	Satisfactory	NILM	NILM	Negative (NILM)	
P05063	2019	2019-Q3	2019-08	Pre-pandemic	Cytology (Pap)	60-64	63	Not performed	Satisfactory	NILM	NILM	Negative (NILM)	
P05064	2019	2019-Q3	2019-08	Pre-pandemic	Cytology (Pap)	60-64	62	Not performed	Satisfactory	NILM	NILM	Negative (NILM)	
P05065	2019	2019-Q3	2019-08	Pre-pandemic	Cytology (Pap)	35-39	37	Not performed	Satisfactory	NILM	NILM	Negative (NILM)	
P05066	2019	2019-Q3	2019-08	Pre-pandemic	Cytology (Pap)	40-44	41	Not performed	Satisfactory	NILM	NILM	Negative (NILM)	
P05067	2019	2019-Q3	2019-08	Pre-pandemic	Cytology (Pap)	55-59	57	Not performed	Satisfactory	NILM	NILM	Negative (NILM)	
P05068	2019	2019-Q3	2019-08	Pre-pandemic	Cytology (Pap)	45-49	46	Not performed	Satisfactory	NILM	NILM	Negative (NILM)	
P05069	2019	2019-Q3	2019-08	Pre-pandemic	Cytology (Pap)	35-39	39	Not performed	Satisfactory	NILM	NILM	Negative (NILM)	
P05070	2019	2019-Q3	2019-08	Pre-pandemic	Cytology (Pap)	40-44	40	Not performed	Satisfactory	NILM	NILM	Negative (NILM)	
P05071	2019	2019-Q3	2019-08	Pre-pandemic	Cytology (Pap)	30-34	33	Not performed	Satisfactory	NILM	NILM	Negative (NILM)	
P05072	2019	2019-Q3	2019-08	Pre-pandemic	Cytology (Pap)	35-39	38	Not performed	Satisfactory	NILM	NILM	Negative (NILM)	
P05073	2019	2019-Q3	2019-08	Pre-pandemic	Cytology (Pap)	25-29	26	Not performed	Satisfactory	NILM	NILM	Negative (NILM)	
P05074	2019	2019-Q3	2019-08	Pre-pandemic	Cytology (Pap)	40-44	43	Not performed	Satisfactory	NILM	NILM	Negative (NILM)	
P05075	2019	2019-Q3	2019-08	Pre-pandemic	Cytology (Pap)	55-59	59	Not performed	Satisfactory	NILM	NILM	Negative (NILM)	
P05076	2019	2019-Q3	2019-08	Pre-pandemic	Cytology (Pap)	30-34	33	Not performed	Satisfactory	NILM	NILM	Negative (NILM)	
P05077	2019	2019-Q3	2019-08	Pre-pandemic	Cytology (Pap)	30-34	33	Not performed	Satisfactory	NILM	NILM	Negative (NILM)	
P05078	2019	2019-Q3	2019-08	Pre-pandemic	Cytology (Pap)	60-64	61	Not performed	Satisfactory	NILM	NILM	Negative (NILM)	
P05079	2019	2019-Q3	2019-08	Pre-pandemic	Cytology (Pap)	40-44	41	Not performed	Satisfactory	NILM	NILM	Negative (NILM)	
P05080	2019	2019-Q3	2019-08	Pre-pandemic	Cytology (Pap)	35-39	36	Not performed	Satisfactory	NILM	NILM	Negative (NILM)	
P05081	2019	2019-Q3	2019-08	Pre-pandemic	Cytology (Pap)	35-39	37	Not performed	Satisfactory	NILM	NILM	Negative (NILM)	
P05082	2019	2019-Q3	2019-08	Pre-pandemic	Cytology (Pap)	35-39	36	Not performed	Satisfactory	NILM	NILM	Negative (NILM)	
P05083	2019	2019-Q3	2019-08	Pre-pandemic	Cytology (Pap)	55-59	57	Not performed	Satisfactory	NILM	NILM	Negative (NILM)	
P05084	2019	2019-Q3	2019-08	Pre-pandemic	Cytology (Pap)	60-64	64	Not performed	Satisfactory	NILM	NILM	Negative (NILM)	
P05085	2019	2019-Q3	2019-08	Pre-pandemic	Cytology (Pap)	50-54	50	Not performed	Satisfactory	Positive	ASC-H	High-grade squamous	
P05086	2019	2019-Q3	2019-08	Pre-pandemic	Cytology (Pap)	45-49	49	Not performed	Satisfactory	NILM	NILM	Negative (NILM)	
P05087	2019	2019-Q3	2019-08	Pre-pandemic	Cytology (Pap)	60-64	62	Not performed	Satisfactory	NILM	NILM	Negative (NILM)	
P05088	2019	2019-Q3	2019-08	Pre-pandemic	Cytology (Pap)	40-44	41	Not performed	Satisfactory	Positive	ASC-US	Low-grade / equivocal squamous	
P05089	2019	2019-Q3	2019-08	Pre-pandemic	Cytology (Pap)	50-54	52	Not performed	Satisfactory	NILM	NILM	Negative (NILM)	
P05090	2019	2019-Q3	2019-08	Pre-pandemic	Cytology (Pap)	45-49	46	Not performed	Satisfactory	NILM	NILM	Negative (NILM)	
P05091	2019	2019-Q3	2019-08	Pre-pandemic	Cytology (Pap)	45-49	49	Not performed	Satisfactory	NILM	NILM	Negative (NILM)	
P05092	2019	2019-Q3	2019-08	Pre-pandemic	Cytology (Pap)	55-59	55	Not performed	Satisfactory	NILM	NILM	Negative (NILM)	
P05093	2019	2019-Q3	2019-08	Pre-pandemic	Cytology (Pap)	55-59	58	Not performed	Satisfactory	NILM	NILM	Negative (NILM)	
P05094	2019	2019-Q3	2019-08	Pre-pandemic	Cytology (Pap)	25-29	27	Not performed	Satisfactory	NILM	NILM	Negative (NILM)	
P05095	2019	2019-Q3	2019-08	Pre-pandemic	Cytology (Pap)	40-44	40	Not performed	Satisfactory	NILM	NILM	Negative (NILM)	
P05096	2019	2019-Q3	2019-08	Pre-pandemic	Cytology (Pap)	55-59	57	Not performed	Satisfactory	Positive	ASC-US	Low-grade / equivocal squamous	
P05097	2019	2019-Q3	2019-08	Pre-pandemic	Cytology (Pap)	55-59	57	Not performed	Satisfactory	NILM	NILM	Negative (NILM)	
P05098	2019	2019-Q3	2019-08	Pre-pandemic	Cytology (Pap)	35-39	38	Not performed	Satisfactory	Positive	ASC-US	Low-grade / equivocal squamous	
P05099	2019	2019-Q3	2019-08	Pre-pandemic	Cytology (Pap)	25-29	25	Not performed	Satisfactory	NILM	NILM	Negative (NILM)	
P05100	2019	2019-Q3	2019-08	Pre-pandemic	Cytology (Pap)	45-49	46	Not performed	Satisfactory	NILM	NILM	Negative (NILM)	
P05101	2019	2019-Q3	2019-08	Pre-pandemic	Cytology (Pap)	40-44	43	Not performed	Satisfactory	Positive	ASC-US	Low-grade / equivocal squamous	
P05102	2019	2019-Q3	2019-08	Pre-pandemic	Cytology (Pap)	30-34	32	Not performed	Satisfactory	NILM	NILM	Negative (NILM)	
P05103	2019	2019-Q3	2019-08	Pre-pandemic	Cytology (Pap)	40-44	44	Not performed	Satisfactory	NILM	NILM	Negative (NILM)	
P05104	2019	2019-Q3	2019-08	Pre-pandemic	Cytology (Pap)	40-44	40	Not performed	Satisfactory	NILM	NILM	Negative (NILM)	
P05105	2019	2019-Q3	2019-08	Pre-pandemic	Cytology (Pap)	30-34	32	Not performed	Satisfactory	NILM	NILM	Negative (NILM)	
P05106	2019	2019-Q3	2019-08	Pre-pandemic	Cytology (Pap)	40-44	43	Not performed	Satisfactory	NILM	NILM	Negative (NILM)	
P05107	2019	2019-Q3	2019-08	Pre-pandemic	Cytology (Pap)	30-34	32	Not performed	Unsatisfactory	Unsatisfactory	Unsatisfactory	Unsatisfactory	
P05108	2019	2019-Q3	2019-08	Pre-pandemic	Cytology (Pap)	40-44	43	Not performed	Satisfactory	NILM	NILM	Negative (NILM)	
P05109	2019	2019-Q3	2019-08	Pre-pandemic	Cytology (Pap)	50-54	54	Not performed	Satisfactory	NILM	NILM	Negative (NILM)	Candida
P05110	2019	2019-Q3	2019-08	Pre-pandemic	Cytology (Pap)	40-44	44	Not performed	Satisfactory	NILM	NILM	Negative (NILM)	
P05111	2019	2019-Q3	2019-08	Pre-pandemic	Cytology (Pap)	45-49	45	Not performed	Satisfactory	NILM	NILM	Negative (NILM)	
P05112	2019	2019-Q3	2019-08	Pre-pandemic	Cytology (Pap)	50-54	51	Not performed	Satisfactory	NILM	NILM	Negative (NILM)	
P05113	2019	2019-Q3	2019-08	Pre-pandemic	Cytology (Pap)	45-49	45	Not performed	Satisfactory	NILM	NILM	Negative (NILM)	
P05114	2019	2019-Q3	2019-08	Pre-pandemic	Cytology (Pap)	40-44	42	Not performed	Satisfactory	NILM	NILM	Negative (NILM)	
P05115	2019	2019-Q3	2019-08	Pre-pandemic	Cytology (Pap)	50-54	53	Not performed	Satisfactory	NILM	NILM	Negative (NILM)	
P05116	2019	2019-Q3	2019-08	Pre-pandemic	Cytology (Pap)	40-44	44	Not performed	Satisfactory	Positive	ASC-US	Low-grade / equivocal squamous	
P05117	2019	2019-Q3	2019-08	Pre-pandemic	Cytology (Pap)	35-39	37	Not performed	Satisfactory	NILM	NILM	Negative (NILM)	
P05118	2019	2019-Q3	2019-08	Pre-pandemic	Cytology (Pap)	60-64	60	Not performed	Unsatisfactory	Unsatisfactory	Unsatisfactory	Unsatisfactory	

patient_id	year	quarter	month	period	primary_test	age_group	age_years	hpv_result	adequacy	result	bethesda	grade	infection
P05119	2019	2019-Q3	2019-08	Pre-pandemic	Cytology (Pap)	55-59	59	Not performed	Satisfactory	NILM	NILM	Negative (NILM)	
P05120	2019	2019-Q3	2019-08	Pre-pandemic	Cytology (Pap)	45-49	49	Not performed	Satisfactory	NILM	NILM	Negative (NILM)	
P05121	2019	2019-Q3	2019-08	Pre-pandemic	Cytology (Pap)	40-44	43	Not performed	Satisfactory	NILM	NILM	Negative (NILM)	
P05122	2019	2019-Q3	2019-08	Pre-pandemic	Cytology (Pap)	50-54	50	Not performed	Satisfactory	NILM	NILM	Negative (NILM)	
P05123	2019	2019-Q3	2019-08	Pre-pandemic	Cytology (Pap)	35-39	37	Not performed	Satisfactory	Positive	ASC-US	Low-grade / equivocal squamous	
P05124	2019	2019-Q3	2019-08	Pre-pandemic	Cytology (Pap)	55-59	59	Not performed	Satisfactory	Positive	LSIL	Low-grade / equivocal squamous	
P05125	2019	2019-Q3	2019-08	Pre-pandemic	Cytology (Pap)	40-44	41	Not performed	Satisfactory	NILM	NILM	Negative (NILM)	
P05126	2019	2019-Q3	2019-08	Pre-pandemic	Cytology (Pap)	30-34	30	Not performed	Satisfactory	NILM	NILM	Negative (NILM)	
P05127	2019	2019-Q3	2019-08	Pre-pandemic	Cytology (Pap)	45-49	46	Not performed	Satisfactory	NILM	NILM	Negative (NILM)	
P05128	2019	2019-Q3	2019-08	Pre-pandemic	Cytology (Pap)	30-34	34	Not performed	Unsatisfactory	Unsatisfactory	Unsatisfactory	Unsatisfactory	
P05129	2019	2019-Q3	2019-08	Pre-pandemic	Cytology (Pap)	40-44	41	Not performed	Satisfactory	NILM	NILM	Negative (NILM)	
P05130	2019	2019-Q3	2019-08	Pre-pandemic	Cytology (Pap)	35-39	36	Not performed	Satisfactory	NILM	NILM	Negative (NILM)	Candida
P05131	2019	2019-Q3	2019-08	Pre-pandemic	Cytology (Pap)	50-54	51	Not performed	Satisfactory	Positive	LSIL	Low-grade / equivocal squamous	
P05132	2019	2019-Q3	2019-08	Pre-pandemic	Cytology (Pap)	55-59	58	Not performed	Satisfactory	NILM	NILM	Negative (NILM)	
P05133	2019	2019-Q3	2019-08	Pre-pandemic	Cytology (Pap)	45-49	49	Not performed	Satisfactory	NILM	NILM	Negative (NILM)	
P05134	2019	2019-Q3	2019-08	Pre-pandemic	Cytology (Pap)	35-39	36	Not performed	Satisfactory	NILM	NILM	Negative (NILM)	
P05135	2019	2019-Q3	2019-08	Pre-pandemic	Cytology (Pap)	35-39	39	Not performed	Satisfactory	NILM	NILM	Negative (NILM)	
P05136	2019	2019-Q3	2019-08	Pre-pandemic	Cytology (Pap)	60-64	60	Not performed	Satisfactory	NILM	NILM	Negative (NILM)	
P05137	2019	2019-Q3	2019-08	Pre-pandemic	Cytology (Pap)	60-64	62	Not performed	Satisfactory	NILM	NILM	Negative (NILM)	
P05138	2019	2019-Q3	2019-08	Pre-pandemic	Cytology (Pap)	55-59	58	Not performed	Satisfactory	NILM	NILM	Negative (NILM)	
P05139	2019	2019-Q3	2019-08	Pre-pandemic	Cytology (Pap)	35-39	38	Not performed	Satisfactory	NILM	NILM	Negative (NILM)	
P05140	2019	2019-Q3	2019-08	Pre-pandemic	Cytology (Pap)	35-39	36	Not performed	Satisfactory	NILM	NILM	Negative (NILM)	
P05141	2019	2019-Q3	2019-08	Pre-pandemic	Cytology (Pap)	40-44	41	Not performed	Satisfactory	Positive	HSIL	High-grade squamous	
P05142	2019	2019-Q3	2019-08	Pre-pandemic	Cytology (Pap)	30-34	33	Not performed	Satisfactory	Positive	ASC-US	Low-grade / equivocal squamous	
P05143	2019	2019-Q3	2019-08	Pre-pandemic	Cytology (Pap)	45-49	48	Not performed	Satisfactory	NILM	NILM	Negative (NILM)	
P05144	2019	2019-Q3	2019-08	Pre-pandemic	Cytology (Pap)	40-44	41	Not performed	Satisfactory	Positive	HSIL	High-grade squamous	
P05145	2019	2019-Q3	2019-08	Pre-pandemic	Cytology (Pap)	40-44	41	Not performed	Satisfactory	NILM	NILM	Negative (NILM)	
P05146	2019	2019-Q3	2019-08	Pre-pandemic	Cytology (Pap)	35-39	37	Not performed	Unsatisfactory	Unsatisfactory	Unsatisfactory	Unsatisfactory	
P05147	2019	2019-Q3	2019-08	Pre-pandemic	Cytology (Pap)	30-34	34	Not performed	Satisfactory	NILM	NILM	Negative (NILM)	
P05148	2019	2019-Q3	2019-08	Pre-pandemic	Cytology (Pap)	50-54	51	Not performed	Satisfactory	NILM	NILM	Negative (NILM)	
P05149	2019	2019-Q3	2019-08	Pre-pandemic	Cytology (Pap)	45-49	46	Not performed	Satisfactory	NILM	NILM	Negative (NILM)	
P05150	2019	2019-Q3	2019-08	Pre-pandemic	Cytology (Pap)	50-54	52	Not performed	Satisfactory	NILM	NILM	Negative (NILM)	
P05151	2019	2019-Q3	2019-08	Pre-pandemic	Cytology (Pap)	55-59	57	Not performed	Satisfactory	NILM	NILM	Negative (NILM)	
P05152	2019	2019-Q3	2019-08	Pre-pandemic	Cytology (Pap)	25-29	28	Not performed	Satisfactory	NILM	NILM	Negative (NILM)	
P05153	2019	2019-Q3	2019-08	Pre-pandemic	Cytology (Pap)	30-34	33	Not performed	Satisfactory	NILM	NILM	Negative (NILM)	
P05154	2019	2019-Q3	2019-08	Pre-pandemic	Cytology (Pap)	55-59	55	Not performed	Unsatisfactory	Unsatisfactory	Unsatisfactory	Unsatisfactory	
P05155	2019	2019-Q3	2019-08	Pre-pandemic	Cytology (Pap)	55-59	55	Not performed	Satisfactory	NILM	NILM	Negative (NILM)	
P05156	2019	2019-Q3	2019-08	Pre-pandemic	Cytology (Pap)	35-39	35	Not performed	Satisfactory	Positive	LSIL	Low-grade / equivocal squamous	
P05157	2019	2019-Q3	2019-08	Pre-pandemic	Cytology (Pap)	30-34	31	Not performed	Satisfactory	NILM	NILM	Negative (NILM)	
P05158	2019	2019-Q3	2019-08	Pre-pandemic	Cytology (Pap)	40-44	44	Not performed	Satisfactory	NILM	NILM	Negative (NILM)	
P05159	2019	2019-Q3	2019-08	Pre-pandemic	Cytology (Pap)	55-59	56	Not performed	Satisfactory	NILM	NILM	Negative (NILM)	
P05160	2019	2019-Q3	2019-08	Pre-pandemic	Cytology (Pap)	25-29	27	Not performed	Satisfactory	NILM	NILM	Negative (NILM)	
P05161	2019	2019-Q3	2019-08	Pre-pandemic	Cytology (Pap)	40-44	43	Not performed	Satisfactory	NILM	NILM	Negative (NILM)	
P05162	2019	2019-Q3	2019-08	Pre-pandemic	Cytology (Pap)	30-34	31	Not performed	Satisfactory	NILM	NILM	Negative (NILM)	
P05163	2019	2019-Q3	2019-08	Pre-pandemic	Cytology (Pap)	35-39	35	Not performed	Satisfactory	NILM	NILM	Negative (NILM)	
P05164	2019	2019-Q3	2019-08	Pre-pandemic	Cytology (Pap)	35-39	37	Not performed	Satisfactory	Positive	HSIL	High-grade squamous	
P05165	2019	2019-Q3	2019-08	Pre-pandemic	Cytology (Pap)	40-44	44	Not performed	Satisfactory	NILM	NILM	Negative (NILM)	
P05166	2019	2019-Q3	2019-08	Pre-pandemic	Cytology (Pap)	40-44	41	Not performed	Satisfactory	NILM	NILM	Negative (NILM)	
P05167	2019	2019-Q3	2019-08	Pre-pandemic	Cytology (Pap)	55-59	57	Not performed	Satisfactory	NILM	NILM	Negative (NILM)	
P05168	2019	2019-Q3	2019-08	Pre-pandemic	Cytology (Pap)	30-34	31	Not performed	Satisfactory	NILM	NILM	Negative (NILM)	
P05169	2019	2019-Q3	2019-08	Pre-pandemic	Cytology (Pap)	40-44	40	Not performed	Satisfactory	NILM	NILM	Negative (NILM)	
P05170	2019	2019-Q3	2019-08	Pre-pandemic	Cytology (Pap)	35-39	38	Not performed	Satisfactory	NILM	NILM	Negative (NILM)	
P05171	2019	2019-Q3	2019-08	Pre-pandemic	Cytology (Pap)	35-39	38	Not performed	Satisfactory	NILM	NILM	Negative (NILM)	
P05172	2019	2019-Q3	2019-08	Pre-pandemic	Cytology (Pap)	25-29	27	Not performed	Satisfactory	NILM	NILM	Negative (NILM)	
P05173	2019	2019-Q3	2019-08	Pre-pandemic	Cytology (Pap)	45-49	45	Not performed	Satisfactory	NILM	NILM	Negative (NILM)	
P05174	2019	2019-Q3	2019-08	Pre-pandemic	Cytology (Pap)	50-54	50	Not performed	Satisfactory	NILM	NILM	Negative (NILM)	
P05175	2019	2019-Q3	2019-08	Pre-pandemic	Cytology (Pap)	45-49	47	Not performed	Unsatisfactory	Unsatisfactory	Unsatisfactory	Unsatisfactory	
P05176	2019	2019-Q3	2019-08	Pre-pandemic	Cytology (Pap)	30-34	30	Not performed	Satisfactory	NILM	NILM	Negative (NILM)	
P05177	2019	2019-Q3	2019-08	Pre-pandemic	Cytology (Pap)	40-44	44	Not performed	Satisfactory	NILM	NILM	Negative (NILM)	
P05178	2019	2019-Q3	2019-08	Pre-pandemic	Cytology (Pap)	40-44	41	Not performed	Satisfactory	NILM	NILM	Negative (NILM)	
P05179	2019	2019-Q3	2019-08	Pre-pandemic	Cytology (Pap)	55-59	56	Not performed	Satisfactory	Positive	AGC	Glandular	
P05180	2019	2019-Q3	2019-08	Pre-pandemic	Cytology (Pap)	40-44	44	Not performed	Satisfactory	NILM	NILM	Negative (NILM)	
P05181	2019	2019-Q3	2019-08	Pre-pandemic	Cytology (Pap)	30-34	30	Not performed	Satisfactory	NILM	NILM	Negative (NILM)	
P05182	2019	2019-Q3	2019-08	Pre-pandemic	Cytology (Pap)	30-34	33	Not performed	Satisfactory	NILM	NILM	Negative (NILM)	
P05183	2019	2019-Q3	2019-08	Pre-pandemic	Cytology (Pap)	40-44	41	Not performed	Satisfactory	Positive	HSIL	High-grade squamous	
P05184	2019	2019-Q3	2019-08	Pre-pandemic	Cytology (Pap)	40-44	40	Not performed	Satisfactory	NILM	NILM	Negative (NILM)	
P05185	2019	2019-Q3	2019-08	Pre-pandemic	Cytology (Pap)	35-39	36	Not performed	Satisfactory	NILM	NILM	Negative (NILM)	
P05186	2019	2019-Q3	2019-08	Pre-pandemic	Cytology (Pap)	35-39	35	Not performed	Satisfactory	NILM	NILM	Negative (NILM)	
P05187	2019	2019-Q3	2019-08	Pre-pandemic	Cytology (Pap)	50-54	51	Not performed	Satisfactory	Positive	LSIL	Low-grade / equivocal squamous	
P05188	2019	2019-Q3	2019-08	Pre-pandemic	Cytology (Pap)	30-34	34	Not performed	Satisfactory	NILM	NILM	Negative (NILM)	
P05189	2019	2019-Q3	2019-08	Pre-pandemic	Cytology (Pap)	25-29	27	Not performed	Satisfactory	Positive	LSIL	Low-grade / equivocal squamous	
P05190	2019	2019-Q3	2019-08	Pre-pandemic	Cytology (Pap)	30-34	34	Not performed	Satisfactory	NILM	NILM	Negative (NILM)	
P05191	2019	2019-Q3	2019-08	Pre-pandemic	Cytology (Pap)	45-49	48	Not performed	Satisfactory	NILM	NILM	Negative (NILM)	
P05192	2019	2019-Q3	2019-08	Pre-pandemic	Cytology (Pap)	35-39	37	Not performed	Satisfactory	NILM	NILM	Negative (NILM)	
P05193	2019	2019-Q3	2019-08	Pre-pandemic	Cytology (Pap)	50-54	53	Not performed	Satisfactory	NILM	NILM	Negative (NILM)	
P05194	2019	2019-Q3	2019-08	Pre-pandemic	Cytology (Pap)	40-44	44	Not performed	Satisfactory	NILM	NILM	Negative (NILM)	
P05195	2019	2019-Q3	2019-08	Pre-pandemic	Cytology (Pap)	25-29	27	Not performed	Satisfactory	NILM	NILM	Negative (NILM)	
P05196	2019	2019-Q3	2019-08	Pre-pandemic	Cytology (Pap)	40-44	42	Not performed	Satisfactory	Positive	ASC-US	Low-grade / equivocal squamous	
P05197	2019	2019-Q3	2019-08	Pre-pandemic	Cytology (Pap)	40-44	44	Not performed	Satisfactory	NILM	NILM	Negative (NILM)	
P05198	2019	2019-Q3	2019-08	Pre-pandemic	Cytology (Pap)	50-54	53	Not performed	Satisfactory	NILM	NILM	Negative (NILM)	Trichomonas

patient_id	year	quarter	month	period	primary_test	age_group	age_years	hpv_result	adequacy	result	bethesda	grade	infection
P05199	2019	2019-Q3	2019-08	Pre-pandemic	Cytology (Pap)	45-49	48	Not performed	Satisfactory	NILM	NILM	Negative (NILM)	
P05200	2019	2019-Q3	2019-08	Pre-pandemic	Cytology (Pap)	35-39	37	Not performed	Satisfactory	NILM	NILM	Negative (NILM)	
P05201	2019	2019-Q3	2019-08	Pre-pandemic	Cytology (Pap)	50-54	51	Not performed	Satisfactory	NILM	NILM	Negative (NILM)	
P05202	2019	2019-Q3	2019-08	Pre-pandemic	Cytology (Pap)	30-34	33	Not performed	Satisfactory	NILM	NILM	Negative (NILM)	
P05203	2019	2019-Q3	2019-08	Pre-pandemic	Cytology (Pap)	55-59	59	Not performed	Satisfactory	NILM	NILM	Negative (NILM)	
P05204	2019	2019-Q3	2019-08	Pre-pandemic	Cytology (Pap)	60-64	62	Not performed	Satisfactory	NILM	NILM	Negative (NILM)	
P05205	2019	2019-Q3	2019-08	Pre-pandemic	Cytology (Pap)	45-49	47	Not performed	Satisfactory	NILM	NILM	Negative (NILM)	
P05206	2019	2019-Q3	2019-08	Pre-pandemic	Cytology (Pap)	40-44	41	Not performed	Satisfactory	NILM	NILM	Negative (NILM)	
P05207	2019	2019-Q3	2019-08	Pre-pandemic	Cytology (Pap)	45-49	47	Not performed	Satisfactory	NILM	NILM	Negative (NILM)	
P05208	2019	2019-Q3	2019-08	Pre-pandemic	Cytology (Pap)	30-34	33	Not performed	Satisfactory	NILM	NILM	Negative (NILM)	
P05209	2019	2019-Q3	2019-08	Pre-pandemic	Cytology (Pap)	30-34	30	Not performed	Satisfactory	NILM	NILM	Negative (NILM)	
P05210	2019	2019-Q3	2019-08	Pre-pandemic	Cytology (Pap)	50-54	52	Not performed	Satisfactory	Positive	LSIL	Low-grade / equivocal squamous	
P05211	2019	2019-Q3	2019-08	Pre-pandemic	Cytology (Pap)	50-54	54	Not performed	Satisfactory	NILM	NILM	Negative (NILM)	
P05212	2019	2019-Q3	2019-08	Pre-pandemic	Cytology (Pap)	60-64	62	Not performed	Satisfactory	NILM	NILM	Negative (NILM)	
P05213	2019	2019-Q3	2019-08	Pre-pandemic	Cytology (Pap)	45-49	47	Not performed	Satisfactory	NILM	NILM	Negative (NILM)	
P05214	2019	2019-Q3	2019-08	Pre-pandemic	Cytology (Pap)	40-44	42	Not performed	Satisfactory	NILM	NILM	Negative (NILM)	
P05215	2019	2019-Q3	2019-08	Pre-pandemic	Cytology (Pap)	30-34	31	Not performed	Satisfactory	NILM	NILM	Negative (NILM)	
P05216	2019	2019-Q3	2019-08	Pre-pandemic	Cytology (Pap)	55-59	57	Not performed	Satisfactory	NILM	NILM	Negative (NILM)	
P05217	2019	2019-Q3	2019-08	Pre-pandemic	Cytology (Pap)	35-39	35	Not performed	Satisfactory	NILM	NILM	Negative (NILM)	
P05218	2019	2019-Q3	2019-08	Pre-pandemic	Cytology (Pap)	40-44	40	Not performed	Satisfactory	NILM	NILM	Negative (NILM)	
P05219	2019	2019-Q3	2019-08	Pre-pandemic	Cytology (Pap)	60-64	61	Not performed	Satisfactory	NILM	NILM	Negative (NILM)	
P05220	2019	2019-Q3	2019-08	Pre-pandemic	Cytology (Pap)	55-59	56	Not performed	Satisfactory	NILM	NILM	Negative (NILM)	
P05221	2019	2019-Q3	2019-08	Pre-pandemic	Cytology (Pap)	55-59	56	Not performed	Satisfactory	NILM	NILM	Negative (NILM)	
P05222	2019	2019-Q3	2019-08	Pre-pandemic	Cytology (Pap)	40-44	40	Not performed	Satisfactory	Positive	LSIL	Low-grade / equivocal squamous	
P05223	2019	2019-Q3	2019-08	Pre-pandemic	Cytology (Pap)	45-49	47	Not performed	Satisfactory	NILM	NILM	Negative (NILM)	
P05224	2019	2019-Q3	2019-08	Pre-pandemic	Cytology (Pap)	55-59	59	Not performed	Satisfactory	NILM	NILM	Negative (NILM)	
P05225	2019	2019-Q3	2019-08	Pre-pandemic	Cytology (Pap)	25-29	26	Not performed	Satisfactory	NILM	NILM	Negative (NILM)	
P05226	2019	2019-Q3	2019-08	Pre-pandemic	Cytology (Pap)	60-64	61	Not performed	Satisfactory	Positive	HSIL	High-grade squamous	
P05227	2019	2019-Q3	2019-08	Pre-pandemic	Cytology (Pap)	35-39	36	Not performed	Satisfactory	NILM	NILM	Negative (NILM)	
P05228	2019	2019-Q3	2019-08	Pre-pandemic	Cytology (Pap)	50-54	54	Not performed	Satisfactory	NILM	NILM	Negative (NILM)	
P05229	2019	2019-Q3	2019-08	Pre-pandemic	Cytology (Pap)	40-44	43	Not performed	Satisfactory	Positive	ASC-US	Low-grade / equivocal squamous	
P05230	2019	2019-Q3	2019-08	Pre-pandemic	Cytology (Pap)	30-34	32	Not performed	Satisfactory	NILM	NILM	Negative (NILM)	Trichomonas
P05231	2019	2019-Q3	2019-08	Pre-pandemic	Cytology (Pap)	50-54	51	Not performed	Satisfactory	NILM	NILM	Negative (NILM)	
P05232	2019	2019-Q3	2019-08	Pre-pandemic	Cytology (Pap)	55-59	56	Not performed	Satisfactory	NILM	NILM	Negative (NILM)	
P05233	2019	2019-Q3	2019-08	Pre-pandemic	Cytology (Pap)	45-49	48	Not performed	Satisfactory	NILM	NILM	Negative (NILM)	
P05234	2019	2019-Q3	2019-08	Pre-pandemic	Cytology (Pap)	35-39	35	Not performed	Satisfactory	NILM	NILM	Negative (NILM)	
P05235	2019	2019-Q3	2019-08	Pre-pandemic	Cytology (Pap)	55-59	58	Not performed	Satisfactory	NILM	NILM	Negative (NILM)	
P05236	2019	2019-Q3	2019-08	Pre-pandemic	Cytology (Pap)	30-34	32	Not performed	Satisfactory	NILM	NILM	Negative (NILM)	
P05237	2019	2019-Q3	2019-08	Pre-pandemic	Cytology (Pap)	40-44	40	Not performed	Satisfactory	NILM	NILM	Negative (NILM)	
P05238	2019	2019-Q3	2019-08	Pre-pandemic	Cytology (Pap)	25-29	29	Not performed	Satisfactory	NILM	NILM	Negative (NILM)	
P05239	2019	2019-Q3	2019-08	Pre-pandemic	Cytology (Pap)	55-59	58	Not performed	Satisfactory	NILM	NILM	Negative (NILM)	
P05240	2019	2019-Q3	2019-08	Pre-pandemic	Cytology (Pap)	40-44	42	Not performed	Satisfactory	NILM	NILM	Negative (NILM)	
P05241	2019	2019-Q3	2019-08	Pre-pandemic	Cytology (Pap)	40-44	40	Not performed	Satisfactory	NILM	NILM	Negative (NILM)	
P05242	2019	2019-Q3	2019-08	Pre-pandemic	Cytology (Pap)	25-29	27	Not performed	Satisfactory	NILM	NILM	Negative (NILM)	
P05243	2019	2019-Q3	2019-08	Pre-pandemic	Cytology (Pap)	30-34	32	Not performed	Satisfactory	Positive	ASC-US	Low-grade / equivocal squamous	
P05244	2019	2019-Q3	2019-08	Pre-pandemic	Cytology (Pap)	55-59	59	Not performed	Satisfactory	Positive	LSIL	Low-grade / equivocal squamous	
P05245	2019	2019-Q3	2019-08	Pre-pandemic	Cytology (Pap)	35-39	35	Not performed	Satisfactory	NILM	NILM	Negative (NILM)	
P05246	2019	2019-Q3	2019-08	Pre-pandemic	Cytology (Pap)	45-49	48	Not performed	Satisfactory	NILM	NILM	Negative (NILM)	
P05247	2019	2019-Q3	2019-08	Pre-pandemic	Cytology (Pap)	30-34	30	Not performed	Satisfactory	NILM	NILM	Negative (NILM)	
P05248	2019	2019-Q3	2019-08	Pre-pandemic	Cytology (Pap)	40-44	44	Not performed	Satisfactory	NILM	NILM	Negative (NILM)	
P05249	2019	2019-Q3	2019-08	Pre-pandemic	Cytology (Pap)	30-34	30	Not performed	Satisfactory	NILM	NILM	Negative (NILM)	
P05250	2019	2019-Q3	2019-08	Pre-pandemic	Cytology (Pap)	55-59	59	Not performed	Satisfactory	NILM	NILM	Negative (NILM)	
P05251	2019	2019-Q3	2019-08	Pre-pandemic	Cytology (Pap)	35-39	37	Not performed	Satisfactory	NILM	NILM	Negative (NILM)	
P05252	2019	2019-Q3	2019-08	Pre-pandemic	Cytology (Pap)	40-44	41	Not performed	Satisfactory	NILM	NILM	Negative (NILM)	
P05253	2019	2019-Q3	2019-08	Pre-pandemic	Cytology (Pap)	45-49	45	Not performed	Satisfactory	NILM	NILM	Negative (NILM)	
P05254	2019	2019-Q3	2019-08	Pre-pandemic	Cytology (Pap)	40-44	43	Not performed	Satisfactory	NILM	NILM	Negative (NILM)	
P05255	2019	2019-Q3	2019-08	Pre-pandemic	Cytology (Pap)	45-49	48	Not performed	Satisfactory	NILM	NILM	Negative (NILM)	
P05256	2019	2019-Q3	2019-08	Pre-pandemic	Cytology (Pap)	35-39	36	Not performed	Satisfactory	NILM	NILM	Negative (NILM)	
P05257	2019	2019-Q3	2019-08	Pre-pandemic	Cytology (Pap)	35-39	37	Not performed	Satisfactory	Positive	HSIL	High-grade squamous	
P05258	2019	2019-Q3	2019-08	Pre-pandemic	Cytology (Pap)	25-29	28	Not performed	Satisfactory	NILM	NILM	Negative (NILM)	
P05259	2019	2019-Q3	2019-08	Pre-pandemic	Cytology (Pap)	45-49	46	Not performed	Satisfactory	NILM	NILM	Negative (NILM)	
P05260	2019	2019-Q3	2019-08	Pre-pandemic	Cytology (Pap)	35-39	38	Not performed	Satisfactory	NILM	NILM	Negative (NILM)	
P05261	2019	2019-Q3	2019-08	Pre-pandemic	Cytology (Pap)	60-64	61	Not performed	Satisfactory	NILM	NILM	Negative (NILM)	
P05262	2019	2019-Q3	2019-08	Pre-pandemic	Cytology (Pap)	60-64	63	Not performed	Satisfactory	NILM	NILM	Negative (NILM)	
P05263	2019	2019-Q3	2019-08	Pre-pandemic	Cytology (Pap)	40-44	41	Not performed	Satisfactory	NILM	NILM	Negative (NILM)	
P05264	2019	2019-Q3	2019-08	Pre-pandemic	Cytology (Pap)	45-49	49	Not performed	Satisfactory	NILM	NILM	Negative (NILM)	
P05265	2019	2019-Q3	2019-08	Pre-pandemic	Cytology (Pap)	25-29	29	Not performed	Satisfactory	NILM	NILM	Negative (NILM)	
P05266	2019	2019-Q3	2019-08	Pre-pandemic	Cytology (Pap)	55-59	55	Not performed	Satisfactory	NILM	NILM	Negative (NILM)	
P05267	2019	2019-Q3	2019-08	Pre-pandemic	Cytology (Pap)	45-49	45	Not performed	Satisfactory	NILM	NILM	Negative (NILM)	
P05268	2019	2019-Q3	2019-08	Pre-pandemic	Cytology (Pap)	40-44	40	Not performed	Unsatisfactory	Unsatisfactory	Unsatisfactory	Unsatisfactory	
P05269	2019	2019-Q3	2019-08	Pre-pandemic	Cytology (Pap)	35-39	35	Not performed	Satisfactory	Positive	LSIL	Low-grade / equivocal squamous	
P05270	2019	2019-Q3	2019-08	Pre-pandemic	Cytology (Pap)	60-64	64	Not performed	Satisfactory	NILM	NILM	Negative (NILM)	
P05271	2019	2019-Q3	2019-08	Pre-pandemic	Cytology (Pap)	25-29	25	Not performed	Satisfactory	Positive	ASC-US	Low-grade / equivocal squamous	
P05272	2019	2019-Q3	2019-09	Pre-pandemic	Cytology (Pap)	45-49	48	Not performed	Satisfactory	NILM	NILM	Negative (NILM)	
P05273	2019	2019-Q3	2019-09	Pre-pandemic	Cytology (Pap)	40-44	44	Not performed	Unsatisfactory	Unsatisfactory	Unsatisfactory	Unsatisfactory	
P05274	2019	2019-Q3	2019-09	Pre-pandemic	Cytology (Pap)	35-39	39	Not performed	Satisfactory	NILM	NILM	Negative (NILM)	
P05275	2019	2019-Q3	2019-09	Pre-pandemic	Cytology (Pap)	40-44	42	Not performed	Satisfactory	NILM	NILM	Negative (NILM)	
P05276	2019	2019-Q3	2019-09	Pre-pandemic	Cytology (Pap)	50-54	50	Not performed	Satisfactory	NILM	NILM	Negative (NILM)	
P05277	2019	2019-Q3	2019-09	Pre-pandemic	Cytology (Pap)	50-54	52	Not performed	Satisfactory	NILM	NILM	Negative (NILM)	
P05278	2019	2019-Q3	2019-09	Pre-pandemic	Cytology (Pap)	40-44	43	Not performed	Satisfactory	Positive	HSIL	High-grade squamous	

patient_id	year	quarter	month	period	primary_test	age_group	age_years	hpv_result	adequacy	result	bethesda	grade	infection
P05279	2019	2019-Q3	2019-09	Pre-pandemic	Cytology (Pap)	45-49	49	Not performed	Satisfactory	NILM	NILM	Negative (NILM)	
P05280	2019	2019-Q3	2019-09	Pre-pandemic	Cytology (Pap)	45-49	47	Not performed	Satisfactory	NILM	NILM	Negative (NILM)	
P05281	2019	2019-Q3	2019-09	Pre-pandemic	Cytology (Pap)	45-49	48	Not performed	Satisfactory	NILM	NILM	Negative (NILM)	
P05282	2019	2019-Q3	2019-09	Pre-pandemic	Cytology (Pap)	55-59	57	Not performed	Satisfactory	NILM	NILM	Negative (NILM)	
P05283	2019	2019-Q3	2019-09	Pre-pandemic	Cytology (Pap)	45-49	46	Not performed	Satisfactory	Positive	LSIL	Low-grade / equivocal squamous	
P05284	2019	2019-Q3	2019-09	Pre-pandemic	Cytology (Pap)	60-64	61	Not performed	Satisfactory	NILM	NILM	Negative (NILM)	
P05285	2019	2019-Q3	2019-09	Pre-pandemic	Cytology (Pap)	50-54	50	Not performed	Satisfactory	NILM	NILM	Negative (NILM)	
P05286	2019	2019-Q3	2019-09	Pre-pandemic	Cytology (Pap)	45-49	45	Not performed	Satisfactory	NILM	NILM	Negative (NILM)	
P05287	2019	2019-Q3	2019-09	Pre-pandemic	Cytology (Pap)	45-49	47	Not performed	Satisfactory	NILM	NILM	Negative (NILM)	
P05288	2019	2019-Q3	2019-09	Pre-pandemic	Cytology (Pap)	45-49	45	Not performed	Satisfactory	NILM	NILM	Negative (NILM)	
P05289	2019	2019-Q3	2019-09	Pre-pandemic	Cytology (Pap)	45-49	48	Not performed	Satisfactory	NILM	NILM	Negative (NILM)	
P05290	2019	2019-Q3	2019-09	Pre-pandemic	Cytology (Pap)	35-39	37	Not performed	Satisfactory	NILM	NILM	Negative (NILM)	
P05291	2019	2019-Q3	2019-09	Pre-pandemic	Cytology (Pap)	50-54	51	Not performed	Unsatisfactory	Unsatisfactory	Unsatisfactory	Unsatisfactory	
P05292	2019	2019-Q3	2019-09	Pre-pandemic	Cytology (Pap)	35-39	38	Not performed	Satisfactory	NILM	NILM	Negative (NILM)	Trichomonas
P05293	2019	2019-Q3	2019-09	Pre-pandemic	Cytology (Pap)	35-39	36	Not performed	Satisfactory	NILM	NILM	Negative (NILM)	
P05294	2019	2019-Q3	2019-09	Pre-pandemic	Cytology (Pap)	40-44	44	Not performed	Satisfactory	NILM	NILM	Negative (NILM)	
P05295	2019	2019-Q3	2019-09	Pre-pandemic	Cytology (Pap)	45-49	45	Not performed	Satisfactory	NILM	NILM	Negative (NILM)	
P05296	2019	2019-Q3	2019-09	Pre-pandemic	Cytology (Pap)	35-39	35	Not performed	Satisfactory	Positive	ASC-US	Low-grade / equivocal squamous	
P05297	2019	2019-Q3	2019-09	Pre-pandemic	Cytology (Pap)	45-49	47	Not performed	Satisfactory	NILM	NILM	Negative (NILM)	
P05298	2019	2019-Q3	2019-09	Pre-pandemic	Cytology (Pap)	35-39	37	Not performed	Satisfactory	NILM	NILM	Negative (NILM)	
P05299	2019	2019-Q3	2019-09	Pre-pandemic	Cytology (Pap)	30-34	32	Not performed	Satisfactory	NILM	NILM	Negative (NILM)	
P05300	2019	2019-Q3	2019-09	Pre-pandemic	Cytology (Pap)	25-29	28	Not performed	Satisfactory	Positive	LSIL	Low-grade / equivocal squamous	
P05301	2019	2019-Q3	2019-09	Pre-pandemic	Cytology (Pap)	45-49	45	Not performed	Unsatisfactory	Unsatisfactory	Unsatisfactory	Unsatisfactory	
P05302	2019	2019-Q3	2019-09	Pre-pandemic	Cytology (Pap)	50-54	51	Not performed	Satisfactory	NILM	NILM	Negative (NILM)	
P05303	2019	2019-Q3	2019-09	Pre-pandemic	Cytology (Pap)	60-64	63	Not performed	Satisfactory	Positive	ASC-US	Low-grade / equivocal squamous	
P05304	2019	2019-Q3	2019-09	Pre-pandemic	Cytology (Pap)	60-64	61	Not performed	Satisfactory	NILM	NILM	Negative (NILM)	
P05305	2019	2019-Q3	2019-09	Pre-pandemic	Cytology (Pap)	45-49	47	Not performed	Satisfactory	NILM	NILM	Negative (NILM)	
P05306	2019	2019-Q3	2019-09	Pre-pandemic	Cytology (Pap)	50-54	52	Not performed	Satisfactory	NILM	NILM	Negative (NILM)	
P05307	2019	2019-Q3	2019-09	Pre-pandemic	Cytology (Pap)	55-59	59	Not performed	Satisfactory	NILM	NILM	Negative (NILM)	
P05308	2019	2019-Q3	2019-09	Pre-pandemic	Cytology (Pap)	45-49	48	Not performed	Satisfactory	NILM	NILM	Negative (NILM)	
P05309	2019	2019-Q3	2019-09	Pre-pandemic	Cytology (Pap)	45-49	48	Not performed	Satisfactory	NILM	NILM	Negative (NILM)	
P05310	2019	2019-Q3	2019-09	Pre-pandemic	Cytology (Pap)	40-44	44	Not performed	Satisfactory	NILM	NILM	Negative (NILM)	
P05311	2019	2019-Q3	2019-09	Pre-pandemic	Cytology (Pap)	55-59	56	Not performed	Satisfactory	NILM	NILM	Negative (NILM)	
P05312	2019	2019-Q3	2019-09	Pre-pandemic	Cytology (Pap)	30-34	33	Not performed	Satisfactory	NILM	NILM	Negative (NILM)	
P05313	2019	2019-Q3	2019-09	Pre-pandemic	Cytology (Pap)	40-44	41	Not performed	Satisfactory	NILM	NILM	Negative (NILM)	
P05314	2019	2019-Q3	2019-09	Pre-pandemic	Cytology (Pap)	25-29	27	Not performed	Satisfactory	NILM	NILM	Negative (NILM)	
P05315	2019	2019-Q3	2019-09	Pre-pandemic	Cytology (Pap)	50-54	54	Not performed	Satisfactory	NILM	NILM	Negative (NILM)	
P05316	2019	2019-Q3	2019-09	Pre-pandemic	Cytology (Pap)	35-39	38	Not performed	Satisfactory	NILM	NILM	Negative (NILM)	
P05317	2019	2019-Q3	2019-09	Pre-pandemic	Cytology (Pap)	30-34	30	Not performed	Satisfactory	Positive	HSIL	High-grade squamous	
P05318	2019	2019-Q3	2019-09	Pre-pandemic	Cytology (Pap)	45-49	49	Not performed	Satisfactory	NILM	NILM	Negative (NILM)	
P05319	2019	2019-Q3	2019-09	Pre-pandemic	Cytology (Pap)	35-39	39	Not performed	Satisfactory	NILM	NILM	Negative (NILM)	
P05320	2019	2019-Q3	2019-09	Pre-pandemic	Cytology (Pap)	50-54	54	Not performed	Satisfactory	Positive	ASC-US	Low-grade / equivocal squamous	
P05321	2019	2019-Q3	2019-09	Pre-pandemic	Cytology (Pap)	40-44	41	Not performed	Satisfactory	NILM	NILM	Negative (NILM)	
P05322	2019	2019-Q3	2019-09	Pre-pandemic	Cytology (Pap)	45-49	46	Not performed	Satisfactory	NILM	NILM	Negative (NILM)	
P05323	2019	2019-Q3	2019-09	Pre-pandemic	Cytology (Pap)	35-39	39	Not performed	Satisfactory	NILM	NILM	Negative (NILM)	
P05324	2019	2019-Q3	2019-09	Pre-pandemic	Cytology (Pap)	40-44	43	Not performed	Satisfactory	NILM	NILM	Negative (NILM)	
P05325	2019	2019-Q3	2019-09	Pre-pandemic	Cytology (Pap)	55-59	56	Not performed	Satisfactory	NILM	NILM	Negative (NILM)	
P05326	2019	2019-Q3	2019-09	Pre-pandemic	Cytology (Pap)	35-39	38	Not performed	Satisfactory	NILM	NILM	Negative (NILM)	
P05327	2019	2019-Q3	2019-09	Pre-pandemic	Cytology (Pap)	45-49	46	Not performed	Satisfactory	NILM	NILM	Negative (NILM)	
P05328	2019	2019-Q3	2019-09	Pre-pandemic	Cytology (Pap)	60-64	64	Not performed	Satisfactory	NILM	NILM	Negative (NILM)	
P05329	2019	2019-Q3	2019-09	Pre-pandemic	Cytology (Pap)	40-44	43	Not performed	Unsatisfactory	Unsatisfactory	Unsatisfactory	Unsatisfactory	
P05330	2019	2019-Q3	2019-09	Pre-pandemic	Cytology (Pap)	40-44	40	Not performed	Satisfactory	Positive	ASC-US	Low-grade / equivocal squamous	
P05331	2019	2019-Q3	2019-09	Pre-pandemic	Cytology (Pap)	35-39	39	Not performed	Satisfactory	NILM	NILM	Negative (NILM)	
P05332	2019	2019-Q3	2019-09	Pre-pandemic	Cytology (Pap)	40-44	42	Not performed	Satisfactory	NILM	NILM	Negative (NILM)	
P05333	2019	2019-Q3	2019-09	Pre-pandemic	Cytology (Pap)	35-39	38	Not performed	Satisfactory	NILM	NILM	Negative (NILM)	
P05334	2019	2019-Q3	2019-09	Pre-pandemic	Cytology (Pap)	40-44	41	Not performed	Satisfactory	NILM	NILM	Negative (NILM)	
P05335	2019	2019-Q3	2019-09	Pre-pandemic	Cytology (Pap)	45-49	45	Not performed	Satisfactory	NILM	NILM	Negative (NILM)	
P05336	2019	2019-Q3	2019-09	Pre-pandemic	Cytology (Pap)	25-29	27	Not performed	Unsatisfactory	Unsatisfactory	Unsatisfactory	Unsatisfactory	
P05337	2019	2019-Q3	2019-09	Pre-pandemic	Cytology (Pap)	30-34	33	Not performed	Satisfactory	NILM	NILM	Negative (NILM)	
P05338	2019	2019-Q3	2019-09	Pre-pandemic	Cytology (Pap)	30-34	31	Not performed	Satisfactory	NILM	NILM	Negative (NILM)	
P05339	2019	2019-Q3	2019-09	Pre-pandemic	Cytology (Pap)	35-39	39	Not performed	Satisfactory	Positive	HSIL	High-grade squamous	
P05340	2019	2019-Q3	2019-09	Pre-pandemic	Cytology (Pap)	60-64	64	Not performed	Satisfactory	NILM	NILM	Negative (NILM)	
P05341	2019	2019-Q3	2019-09	Pre-pandemic	Cytology (Pap)	55-59	57	Not performed	Satisfactory	NILM	NILM	Negative (NILM)	
P05342	2019	2019-Q3	2019-09	Pre-pandemic	Cytology (Pap)	40-44	44	Not performed	Satisfactory	NILM	NILM	Negative (NILM)	
P05343	2019	2019-Q3	2019-09	Pre-pandemic	Cytology (Pap)	40-44	44	Not performed	Satisfactory	NILM	NILM	Negative (NILM)	
P05344	2019	2019-Q3	2019-09	Pre-pandemic	Cytology (Pap)	35-39	36	Not performed	Satisfactory	NILM	NILM	Negative (NILM)	
P05345	2019	2019-Q3	2019-09	Pre-pandemic	Cytology (Pap)	45-49	47	Not performed	Satisfactory	NILM	NILM	Negative (NILM)	
P05346	2019	2019-Q3	2019-09	Pre-pandemic	Cytology (Pap)	30-34	31	Not performed	Satisfactory	NILM	NILM	Negative (NILM)	
P05347	2019	2019-Q3	2019-09	Pre-pandemic	Cytology (Pap)	25-29	26	Not performed	Satisfactory	NILM	NILM	Negative (NILM)	
P05348	2019	2019-Q3	2019-09	Pre-pandemic	Cytology (Pap)	60-64	63	Not performed	Satisfactory	NILM	NILM	Negative (NILM)	
P05349	2019	2019-Q3	2019-09	Pre-pandemic	Cytology (Pap)	35-39	39	Not performed	Satisfactory	NILM	NILM	Negative (NILM)	
P05350	2019	2019-Q3	2019-09	Pre-pandemic	Cytology (Pap)	40-44	44	Not performed	Satisfactory	NILM	NILM	Negative (NILM)	
P05351	2019	2019-Q3	2019-09	Pre-pandemic	Cytology (Pap)	35-39	36	Not performed	Satisfactory	NILM	NILM	Negative (NILM)	
P05352	2019	2019-Q3	2019-09	Pre-pandemic	Cytology (Pap)	45-49	48	Not performed	Satisfactory	Positive	LSIL	Low-grade / equivocal squamous	
P05353	2019	2019-Q3	2019-09	Pre-pandemic	Cytology (Pap)	35-39	37	Not performed	Satisfactory	NILM	NILM	Negative (NILM)	
P05354	2019	2019-Q3	2019-09	Pre-pandemic	Cytology (Pap)	35-39	36	Not performed	Satisfactory	NILM	NILM	Negative (NILM)	
P05355	2019	2019-Q3	2019-09	Pre-pandemic	Cytology (Pap)	50-54	52	Not performed	Satisfactory	NILM	NILM	Negative (NILM)	
P05356	2019	2019-Q3	2019-09	Pre-pandemic	Cytology (Pap)	50-54	51	Not performed	Unsatisfactory	Unsatisfactory	Unsatisfactory	Unsatisfactory	
P05357	2019	2019-Q3	2019-09	Pre-pandemic	Cytology (Pap)	40-44	44	Not performed	Satisfactory	NILM	NILM	Negative (NILM)	
P05358	2019	2019-Q3	2019-09	Pre-pandemic	Cytology (Pap)	35-39	35	Not performed	Satisfactory	NILM	NILM	Negative (NILM)	

patient_id	year	quarter	month	period	primary_test	age_group	age_years	hpv_result	adequacy	result	bethesda	grade	infection
P05359	2019	2019-Q3	2019-09	Pre-pandemic	Cytology (Pap)	40-44	42	Not performed	Satisfactory	NILM	NILM	Negative (NILM)	
P05360	2019	2019-Q3	2019-09	Pre-pandemic	Cytology (Pap)	45-49	45	Not performed	Satisfactory	NILM	NILM	Negative (NILM)	
P05361	2019	2019-Q3	2019-09	Pre-pandemic	Cytology (Pap)	40-44	40	Not performed	Satisfactory	Positive	ASC-US	Low-grade / equivocal squamous	
P05362	2019	2019-Q3	2019-09	Pre-pandemic	Cytology (Pap)	40-44	43	Not performed	Satisfactory	NILM	NILM	Negative (NILM)	
P05363	2019	2019-Q3	2019-09	Pre-pandemic	Cytology (Pap)	50-54	54	Not performed	Satisfactory	NILM	NILM	Negative (NILM)	
P05364	2019	2019-Q3	2019-09	Pre-pandemic	Cytology (Pap)	40-44	42	Not performed	Satisfactory	NILM	NILM	Negative (NILM)	
P05365	2019	2019-Q3	2019-09	Pre-pandemic	Cytology (Pap)	45-49	46	Not performed	Satisfactory	Positive	HSIL	High-grade squamous	
P05366	2019	2019-Q3	2019-09	Pre-pandemic	Cytology (Pap)	40-44	43	Not performed	Satisfactory	NILM	NILM	Negative (NILM)	
P05367	2019	2019-Q3	2019-09	Pre-pandemic	Cytology (Pap)	40-44	41	Not performed	Satisfactory	NILM	NILM	Negative (NILM)	Candida
P05368	2019	2019-Q3	2019-09	Pre-pandemic	Cytology (Pap)	60-64	60	Not performed	Satisfactory	NILM	NILM	Negative (NILM)	
P05369	2019	2019-Q3	2019-09	Pre-pandemic	Cytology (Pap)	35-39	37	Not performed	Satisfactory	NILM	NILM	Negative (NILM)	
P05370	2019	2019-Q3	2019-09	Pre-pandemic	Cytology (Pap)	45-49	49	Not performed	Satisfactory	Positive	LSIL	Low-grade / equivocal squamous	
P05371	2019	2019-Q3	2019-09	Pre-pandemic	Cytology (Pap)	50-54	50	Not performed	Satisfactory	NILM	NILM	Negative (NILM)	
P05372	2019	2019-Q3	2019-09	Pre-pandemic	Cytology (Pap)	40-44	42	Not performed	Unsatisfactory	Unsatisfactory	Unsatisfactory	Unsatisfactory	
P05373	2019	2019-Q3	2019-09	Pre-pandemic	Cytology (Pap)	45-49	48	Not performed	Satisfactory	Positive	ASC-H	High-grade squamous	
P05374	2019	2019-Q3	2019-09	Pre-pandemic	Cytology (Pap)	45-49	49	Not performed	Satisfactory	NILM	NILM	Negative (NILM)	
P05375	2019	2019-Q3	2019-09	Pre-pandemic	Cytology (Pap)	45-49	45	Not performed	Satisfactory	NILM	NILM	Negative (NILM)	
P05376	2019	2019-Q3	2019-09	Pre-pandemic	Cytology (Pap)	60-64	64	Not performed	Satisfactory	Positive	LSIL	Low-grade / equivocal squamous	
P05377	2019	2019-Q3	2019-09	Pre-pandemic	Cytology (Pap)	55-59	55	Not performed	Satisfactory	NILM	NILM	Negative (NILM)	
P05378	2019	2019-Q3	2019-09	Pre-pandemic	Cytology (Pap)	50-54	51	Not performed	Satisfactory	Positive	HSIL	High-grade squamous	
P05379	2019	2019-Q3	2019-09	Pre-pandemic	Cytology (Pap)	35-39	35	Not performed	Satisfactory	NILM	NILM	Negative (NILM)	
P05380	2019	2019-Q3	2019-09	Pre-pandemic	Cytology (Pap)	55-59	59	Not performed	Unsatisfactory	Unsatisfactory	Unsatisfactory	Unsatisfactory	
P05381	2019	2019-Q3	2019-09	Pre-pandemic	Cytology (Pap)	35-39	38	Not performed	Satisfactory	NILM	NILM	Negative (NILM)	
P05382	2019	2019-Q3	2019-09	Pre-pandemic	Cytology (Pap)	40-44	44	Not performed	Satisfactory	NILM	NILM	Negative (NILM)	
P05383	2019	2019-Q3	2019-09	Pre-pandemic	Cytology (Pap)	30-34	31	Not performed	Satisfactory	NILM	NILM	Negative (NILM)	
P05384	2019	2019-Q3	2019-09	Pre-pandemic	Cytology (Pap)	40-44	43	Not performed	Satisfactory	NILM	NILM	Negative (NILM)	
P05385	2019	2019-Q3	2019-09	Pre-pandemic	Cytology (Pap)	50-54	54	Not performed	Satisfactory	NILM	NILM	Negative (NILM)	
P05386	2019	2019-Q3	2019-09	Pre-pandemic	Cytology (Pap)	45-49	49	Not performed	Satisfactory	NILM	NILM	Negative (NILM)	
P05387	2019	2019-Q3	2019-09	Pre-pandemic	Cytology (Pap)	30-34	34	Not performed	Satisfactory	NILM	NILM	Negative (NILM)	
P05388	2019	2019-Q3	2019-09	Pre-pandemic	Cytology (Pap)	50-54	53	Not performed	Satisfactory	NILM	NILM	Negative (NILM)	
P05389	2019	2019-Q3	2019-09	Pre-pandemic	Cytology (Pap)	55-59	55	Not performed	Satisfactory	Positive	ASC-US	Low-grade / equivocal squamous	
P05390	2019	2019-Q3	2019-09	Pre-pandemic	Cytology (Pap)	40-44	42	Not performed	Satisfactory	Positive	HSIL	High-grade squamous	
P05391	2019	2019-Q3	2019-09	Pre-pandemic	Cytology (Pap)	30-34	31	Not performed	Satisfactory	NILM	NILM	Negative (NILM)	
P05392	2019	2019-Q3	2019-09	Pre-pandemic	Cytology (Pap)	40-44	41	Not performed	Satisfactory	NILM	NILM	Negative (NILM)	
P05393	2019	2019-Q3	2019-09	Pre-pandemic	Cytology (Pap)	35-39	35	Not performed	Satisfactory	NILM	NILM	Negative (NILM)	
P05394	2019	2019-Q3	2019-09	Pre-pandemic	Cytology (Pap)	35-39	35	Not performed	Satisfactory	NILM	NILM	Negative (NILM)	
P05395	2019	2019-Q3	2019-09	Pre-pandemic	Cytology (Pap)	35-39	37	Not performed	Satisfactory	NILM	NILM	Negative (NILM)	
P05396	2019	2019-Q3	2019-09	Pre-pandemic	Cytology (Pap)	35-39	37	Not performed	Satisfactory	NILM	NILM	Negative (NILM)	
P05397	2019	2019-Q3	2019-09	Pre-pandemic	Cytology (Pap)	55-59	59	Not performed	Satisfactory	NILM	NILM	Negative (NILM)	
P05398	2019	2019-Q3	2019-09	Pre-pandemic	Cytology (Pap)	50-54	54	Not performed	Satisfactory	NILM	NILM	Negative (NILM)	
P05399	2019	2019-Q3	2019-09	Pre-pandemic	Cytology (Pap)	55-59	56	Not performed	Satisfactory	NILM	NILM	Negative (NILM)	
P05400	2019	2019-Q3	2019-09	Pre-pandemic	Cytology (Pap)	40-44	42	Not performed	Satisfactory	NILM	NILM	Negative (NILM)	
P05401	2019	2019-Q3	2019-09	Pre-pandemic	Cytology (Pap)	55-59	55	Not performed	Satisfactory	NILM	NILM	Negative (NILM)	
P05402	2019	2019-Q3	2019-09	Pre-pandemic	Cytology (Pap)	45-49	48	Not performed	Satisfactory	NILM	NILM	Negative (NILM)	
P05403	2019	2019-Q3	2019-09	Pre-pandemic	Cytology (Pap)	50-54	52	Not performed	Satisfactory	NILM	NILM	Negative (NILM)	
P05404	2019	2019-Q3	2019-09	Pre-pandemic	Cytology (Pap)	30-34	34	Not performed	Satisfactory	NILM	NILM	Negative (NILM)	
P05405	2019	2019-Q3	2019-09	Pre-pandemic	Cytology (Pap)	35-39	39	Not performed	Satisfactory	NILM	NILM	Negative (NILM)	
P05406	2019	2019-Q3	2019-09	Pre-pandemic	Cytology (Pap)	50-54	50	Not performed	Satisfactory	NILM	NILM	Negative (NILM)	
P05407	2019	2019-Q3	2019-09	Pre-pandemic	Cytology (Pap)	45-49	45	Not performed	Satisfactory	NILM	NILM	Negative (NILM)	
P05408	2019	2019-Q3	2019-09	Pre-pandemic	Cytology (Pap)	60-64	64	Not performed	Satisfactory	NILM	NILM	Negative (NILM)	
P05409	2019	2019-Q3	2019-09	Pre-pandemic	Cytology (Pap)	50-54	54	Not performed	Satisfactory	NILM	NILM	Negative (NILM)	
P05410	2019	2019-Q3	2019-09	Pre-pandemic	Cytology (Pap)	30-34	30	Not performed	Satisfactory	NILM	NILM	Negative (NILM)	
P05411	2019	2019-Q3	2019-09	Pre-pandemic	Cytology (Pap)	25-29	27	Not performed	Satisfactory	NILM	NILM	Negative (NILM)	
P05412	2019	2019-Q3	2019-09	Pre-pandemic	Cytology (Pap)	55-59	57	Not performed	Satisfactory	NILM	NILM	Negative (NILM)	
P05413	2019	2019-Q3	2019-09	Pre-pandemic	Cytology (Pap)	50-54	54	Not performed	Satisfactory	NILM	NILM	Negative (NILM)	
P05414	2019	2019-Q3	2019-09	Pre-pandemic	Cytology (Pap)	30-34	34	Not performed	Satisfactory	NILM	NILM	Negative (NILM)	
P05415	2019	2019-Q3	2019-09	Pre-pandemic	Cytology (Pap)	55-59	58	Not performed	Satisfactory	NILM	NILM	Negative (NILM)	
P05416	2019	2019-Q3	2019-09	Pre-pandemic	Cytology (Pap)	25-29	26	Not performed	Satisfactory	NILM	NILM	Negative (NILM)	
P05417	2019	2019-Q3	2019-09	Pre-pandemic	Cytology (Pap)	40-44	43	Not performed	Satisfactory	NILM	NILM	Negative (NILM)	
P05418	2019	2019-Q3	2019-09	Pre-pandemic	Cytology (Pap)	25-29	25	Not performed	Satisfactory	NILM	NILM	Negative (NILM)	
P05419	2019	2019-Q3	2019-09	Pre-pandemic	Cytology (Pap)	35-39	39	Not performed	Satisfactory	NILM	NILM	Negative (NILM)	
P05420	2019	2019-Q3	2019-09	Pre-pandemic	Cytology (Pap)	45-49	45	Not performed	Satisfactory	NILM	NILM	Negative (NILM)	
P05421	2019	2019-Q3	2019-09	Pre-pandemic	Cytology (Pap)	30-34	31	Not performed	Satisfactory	NILM	NILM	Negative (NILM)	
P05422	2019	2019-Q3	2019-09	Pre-pandemic	Cytology (Pap)	40-44	40	Not performed	Satisfactory	NILM	NILM	Negative (NILM)	
P05423	2019	2019-Q3	2019-09	Pre-pandemic	Cytology (Pap)	30-34	31	Not performed	Unsatisfactory	Unsatisfactory	Unsatisfactory	Unsatisfactory	
P05424	2019	2019-Q3	2019-09	Pre-pandemic	Cytology (Pap)	25-29	27	Not performed	Satisfactory	NILM	NILM	Negative (NILM)	
P05425	2019	2019-Q3	2019-09	Pre-pandemic	Cytology (Pap)	40-44	44	Not performed	Satisfactory	NILM	NILM	Negative (NILM)	
P05426	2019	2019-Q3	2019-09	Pre-pandemic	Cytology (Pap)	35-39	37	Not performed	Satisfactory	NILM	NILM	Negative (NILM)	
P05427	2019	2019-Q3	2019-09	Pre-pandemic	Cytology (Pap)	40-44	44	Not performed	Satisfactory	NILM	NILM	Negative (NILM)	
P05428	2019	2019-Q3	2019-09	Pre-pandemic	Cytology (Pap)	60-64	64	Not performed	Satisfactory	Positive	HSIL	High-grade squamous	
P05429	2019	2019-Q3	2019-09	Pre-pandemic	Cytology (Pap)	40-44	42	Not performed	Satisfactory	NILM	NILM	Negative (NILM)	
P05430	2019	2019-Q3	2019-09	Pre-pandemic	Cytology (Pap)	35-39	35	Not performed	Satisfactory	NILM	NILM	Negative (NILM)	
P05431	2019	2019-Q3	2019-09	Pre-pandemic	Cytology (Pap)	40-44	42	Not performed	Satisfactory	NILM	NILM	Negative (NILM)	
P05432	2019	2019-Q3	2019-09	Pre-pandemic	Cytology (Pap)	30-34	31	Not performed	Satisfactory	NILM	NILM	Negative (NILM)	
P05433	2019	2019-Q3	2019-09	Pre-pandemic	Cytology (Pap)	45-49	45	Not performed	Satisfactory	Positive	ASC-US	Low-grade / equivocal squamous	
P05434	2019	2019-Q3	2019-09	Pre-pandemic	Cytology (Pap)	45-49	45	Not performed	Satisfactory	NILM	NILM	Negative (NILM)	
P05435	2019	2019-Q3	2019-09	Pre-pandemic	Cytology (Pap)	35-39	39	Not performed	Satisfactory	Positive	ASC-US	Low-grade / equivocal squamous	
P05436	2019	2019-Q3	2019-09	Pre-pandemic	Cytology (Pap)	30-34	34	Not performed	Satisfactory	NILM	NILM	Negative (NILM)	Gardnerella
P05437	2019	2019-Q3	2019-09	Pre-pandemic	Cytology (Pap)	50-54	54	Not performed	Satisfactory	NILM	NILM	Negative (NILM)	
P05438	2019	2019-Q3	2019-09	Pre-pandemic	Cytology (Pap)	60-64	64	Not performed	Satisfactory	NILM	NILM	Negative (NILM)	

patient_id	year	quarter	month	period	primary_test	age_group	age_years	hpv_result	adequacy	result	bethesda	grade	infection
P05439	2019	2019-Q3	2019-09	Pre-pandemic	Cytology (Pap)	40-44	43	Not performed	Satisfactory	NILM	NILM	Negative (NILM)	
P05440	2019	2019-Q3	2019-09	Pre-pandemic	Cytology (Pap)	35-39	37	Not performed	Satisfactory	NILM	NILM	Negative (NILM)	
P05441	2019	2019-Q3	2019-09	Pre-pandemic	Cytology (Pap)	40-44	41	Not performed	Satisfactory	Positive	ASC-US	Low-grade / equivocal squamous	
P05442	2019	2019-Q3	2019-09	Pre-pandemic	Cytology (Pap)	50-54	52	Not performed	Satisfactory	NILM	NILM	Negative (NILM)	
P05443	2019	2019-Q3	2019-09	Pre-pandemic	Cytology (Pap)	45-49	49	Not performed	Satisfactory	NILM	NILM	Negative (NILM)	
P05444	2019	2019-Q3	2019-09	Pre-pandemic	Cytology (Pap)	55-59	57	Not performed	Satisfactory	NILM	NILM	Negative (NILM)	
P05445	2019	2019-Q3	2019-09	Pre-pandemic	Cytology (Pap)	45-49	48	Not performed	Satisfactory	NILM	NILM	Negative (NILM)	
P05446	2019	2019-Q3	2019-09	Pre-pandemic	Cytology (Pap)	25-29	26	Not performed	Satisfactory	NILM	NILM	Negative (NILM)	
P05447	2019	2019-Q3	2019-09	Pre-pandemic	Cytology (Pap)	50-54	54	Not performed	Satisfactory	NILM	NILM	Negative (NILM)	
P05448	2019	2019-Q3	2019-09	Pre-pandemic	Cytology (Pap)	40-44	43	Not performed	Satisfactory	NILM	NILM	Negative (NILM)	
P05449	2019	2019-Q3	2019-09	Pre-pandemic	Cytology (Pap)	30-34	31	Not performed	Satisfactory	NILM	NILM	Negative (NILM)	
P05450	2019	2019-Q3	2019-09	Pre-pandemic	Cytology (Pap)	55-59	59	Not performed	Satisfactory	Positive	ASC-US	Low-grade / equivocal squamous	
P05451	2019	2019-Q3	2019-09	Pre-pandemic	Cytology (Pap)	55-59	56	Not performed	Satisfactory	Positive	ASC-US	Low-grade / equivocal squamous	
P05452	2019	2019-Q3	2019-09	Pre-pandemic	Cytology (Pap)	45-49	47	Not performed	Satisfactory	NILM	NILM	Negative (NILM)	
P05453	2019	2019-Q3	2019-09	Pre-pandemic	Cytology (Pap)	30-34	30	Not performed	Satisfactory	NILM	NILM	Negative (NILM)	
P05454	2019	2019-Q3	2019-09	Pre-pandemic	Cytology (Pap)	45-49	45	Not performed	Satisfactory	NILM	NILM	Negative (NILM)	
P05455	2019	2019-Q3	2019-09	Pre-pandemic	Cytology (Pap)	45-49	48	Not performed	Satisfactory	Positive	LSIL	Low-grade / equivocal squamous	
P05456	2019	2019-Q3	2019-09	Pre-pandemic	Cytology (Pap)	30-34	30	Not performed	Satisfactory	NILM	NILM	Negative (NILM)	
P05457	2019	2019-Q3	2019-09	Pre-pandemic	Cytology (Pap)	40-44	41	Not performed	Satisfactory	NILM	NILM	Negative (NILM)	
P05458	2019	2019-Q3	2019-09	Pre-pandemic	Cytology (Pap)	40-44	41	Not performed	Satisfactory	NILM	NILM	Negative (NILM)	
P05459	2019	2019-Q3	2019-09	Pre-pandemic	Cytology (Pap)	30-34	33	Not performed	Satisfactory	NILM	NILM	Negative (NILM)	
P05460	2019	2019-Q3	2019-09	Pre-pandemic	Cytology (Pap)	30-34	32	Not performed	Satisfactory	NILM	NILM	Negative (NILM)	
P05461	2019	2019-Q3	2019-09	Pre-pandemic	Cytology (Pap)	40-44	43	Not performed	Satisfactory	NILM	NILM	Negative (NILM)	
P05462	2019	2019-Q3	2019-09	Pre-pandemic	Cytology (Pap)	55-59	55	Not performed	Satisfactory	Positive	ASC-H	High-grade squamous	
P05463	2019	2019-Q3	2019-09	Pre-pandemic	Cytology (Pap)	55-59	55	Not performed	Satisfactory	NILM	NILM	Negative (NILM)	
P05464	2019	2019-Q3	2019-09	Pre-pandemic	Cytology (Pap)	25-29	29	Not performed	Satisfactory	Positive	HSIL	High-grade squamous	
P05465	2019	2019-Q3	2019-09	Pre-pandemic	Cytology (Pap)	35-39	38	Not performed	Satisfactory	NILM	NILM	Negative (NILM)	
P05466	2019	2019-Q3	2019-09	Pre-pandemic	Cytology (Pap)	45-49	46	Not performed	Satisfactory	NILM	NILM	Negative (NILM)	
P05467	2019	2019-Q3	2019-09	Pre-pandemic	Cytology (Pap)	50-54	51	Not performed	Satisfactory	Positive	HSIL	High-grade squamous	
P05468	2019	2019-Q3	2019-09	Pre-pandemic	Cytology (Pap)	55-59	56	Not performed	Satisfactory	NILM	NILM	Negative (NILM)	
P05469	2019	2019-Q3	2019-09	Pre-pandemic	Cytology (Pap)	40-44	40	Not performed	Satisfactory	NILM	NILM	Negative (NILM)	
P05470	2019	2019-Q3	2019-09	Pre-pandemic	Cytology (Pap)	45-49	48	Not performed	Satisfactory	NILM	NILM	Negative (NILM)	
P05471	2019	2019-Q3	2019-09	Pre-pandemic	Cytology (Pap)	45-49	45	Not performed	Satisfactory	NILM	NILM	Negative (NILM)	
P05472	2019	2019-Q3	2019-09	Pre-pandemic	Cytology (Pap)	45-49	48	Not performed	Satisfactory	Positive	ASC-H	High-grade squamous	
P05473	2019	2019-Q3	2019-09	Pre-pandemic	Cytology (Pap)	45-49	47	Not performed	Satisfactory	NILM	NILM	Negative (NILM)	
P05474	2019	2019-Q3	2019-09	Pre-pandemic	Cytology (Pap)	50-54	54	Not performed	Satisfactory	NILM	NILM	Negative (NILM)	
P05475	2019	2019-Q3	2019-09	Pre-pandemic	Cytology (Pap)	35-39	37	Not performed	Satisfactory	NILM	NILM	Negative (NILM)	
P05476	2019	2019-Q3	2019-09	Pre-pandemic	Cytology (Pap)	45-49	47	Not performed	Satisfactory	NILM	NILM	Negative (NILM)	
P05477	2019	2019-Q3	2019-09	Pre-pandemic	Cytology (Pap)	25-29	25	Not performed	Satisfactory	NILM	NILM	Negative (NILM)	
P05478	2019	2019-Q3	2019-09	Pre-pandemic	Cytology (Pap)	45-49	46	Not performed	Satisfactory	NILM	NILM	Negative (NILM)	
P05479	2019	2019-Q3	2019-09	Pre-pandemic	Cytology (Pap)	30-34	31	Not performed	Satisfactory	Positive	ASC-US	Low-grade / equivocal squamous	
P05480	2019	2019-Q3	2019-09	Pre-pandemic	Cytology (Pap)	50-54	53	Not performed	Satisfactory	NILM	NILM	Negative (NILM)	
P05481	2019	2019-Q3	2019-09	Pre-pandemic	Cytology (Pap)	35-39	36	Not performed	Satisfactory	NILM	NILM	Negative (NILM)	
P05482	2019	2019-Q3	2019-09	Pre-pandemic	Cytology (Pap)	45-49	47	Not performed	Satisfactory	NILM	NILM	Negative (NILM)	Candida
P05483	2019	2019-Q3	2019-09	Pre-pandemic	Cytology (Pap)	60-64	61	Not performed	Satisfactory	NILM	NILM	Negative (NILM)	
P05484	2019	2019-Q3	2019-09	Pre-pandemic	Cytology (Pap)	50-54	50	Not performed	Satisfactory	NILM	NILM	Negative (NILM)	
P05485	2019	2019-Q3	2019-09	Pre-pandemic	Cytology (Pap)	40-44	42	Not performed	Satisfactory	Positive	LSIL	Low-grade / equivocal squamous	
P05486	2019	2019-Q3	2019-09	Pre-pandemic	Cytology (Pap)	55-59	57	Not performed	Satisfactory	NILM	NILM	Negative (NILM)	
P05487	2019	2019-Q3	2019-09	Pre-pandemic	Cytology (Pap)	40-44	41	Not performed	Satisfactory	NILM	NILM	Negative (NILM)	
P05488	2019	2019-Q3	2019-09	Pre-pandemic	Cytology (Pap)	45-49	45	Not performed	Satisfactory	NILM	NILM	Negative (NILM)	
P05489	2019	2019-Q3	2019-09	Pre-pandemic	Cytology (Pap)	45-49	45	Not performed	Satisfactory	NILM	NILM	Negative (NILM)	
P05490	2019	2019-Q3	2019-09	Pre-pandemic	Cytology (Pap)	25-29	27	Not performed	Satisfactory	NILM	NILM	Negative (NILM)	
P05491	2019	2019-Q3	2019-09	Pre-pandemic	Cytology (Pap)	35-39	38	Not performed	Satisfactory	NILM	NILM	Negative (NILM)	
P05492	2019	2019-Q3	2019-09	Pre-pandemic	Cytology (Pap)	40-44	40	Not performed	Satisfactory	Positive	HSIL	High-grade squamous	
P05493	2019	2019-Q3	2019-09	Pre-pandemic	Cytology (Pap)	35-39	37	Not performed	Satisfactory	NILM	NILM	Negative (NILM)	
P05494	2019	2019-Q3	2019-09	Pre-pandemic	Cytology (Pap)	40-44	42	Not performed	Satisfactory	NILM	NILM	Negative (NILM)	
P05495	2019	2019-Q3	2019-09	Pre-pandemic	Cytology (Pap)	45-49	48	Not performed	Satisfactory	NILM	NILM	Negative (NILM)	
P05496	2019	2019-Q3	2019-09	Pre-pandemic	Cytology (Pap)	35-39	36	Not performed	Satisfactory	NILM	NILM	Negative (NILM)	
P05497	2019	2019-Q3	2019-09	Pre-pandemic	Cytology (Pap)	50-54	51	Not performed	Satisfactory	NILM	NILM	Negative (NILM)	
P05498	2019	2019-Q3	2019-09	Pre-pandemic	Cytology (Pap)	40-44	43	Not performed	Satisfactory	NILM	NILM	Negative (NILM)	
P05499	2019	2019-Q3	2019-09	Pre-pandemic	Cytology (Pap)	40-44	40	Not performed	Satisfactory	NILM	NILM	Negative (NILM)	
P05500	2019	2019-Q3	2019-09	Pre-pandemic	Cytology (Pap)	25-29	25	Not performed	Satisfactory	Positive	LSIL	Low-grade / equivocal squamous	
P05501	2019	2019-Q3	2019-09	Pre-pandemic	Cytology (Pap)	35-39	38	Not performed	Satisfactory	Positive	HSIL	High-grade squamous	
P05502	2019	2019-Q3	2019-09	Pre-pandemic	Cytology (Pap)	60-64	63	Not performed	Satisfactory	Positive	ASC-US	Low-grade / equivocal squamous	
P05503	2019	2019-Q3	2019-09	Pre-pandemic	Cytology (Pap)	45-49	47	Not performed	Satisfactory	NILM	NILM	Negative (NILM)	
P05504	2019	2019-Q3	2019-09	Pre-pandemic	Cytology (Pap)	30-34	32	Not performed	Satisfactory	Positive	AGC	Glandular	
P05505	2019	2019-Q3	2019-09	Pre-pandemic	Cytology (Pap)	45-49	45	Not performed	Satisfactory	NILM	NILM	Negative (NILM)	
P05506	2019	2019-Q3	2019-09	Pre-pandemic	Cytology (Pap)	60-64	62	Not performed	Satisfactory	NILM	NILM	Negative (NILM)	
P05507	2019	2019-Q3	2019-09	Pre-pandemic	Cytology (Pap)	50-54	51	Not performed	Satisfactory	NILM	NILM	Negative (NILM)	
P05508	2019	2019-Q3	2019-09	Pre-pandemic	Cytology (Pap)	55-59	59	Not performed	Satisfactory	NILM	NILM	Negative (NILM)	
P05509	2019	2019-Q3	2019-09	Pre-pandemic	Cytology (Pap)	45-49	47	Not performed	Satisfactory	NILM	NILM	Negative (NILM)	
P05510	2019	2019-Q3	2019-09	Pre-pandemic	Cytology (Pap)	35-39	36	Not performed	Satisfactory	Positive	ASC-US	Low-grade / equivocal squamous	
P05511	2019	2019-Q3	2019-09	Pre-pandemic	Cytology (Pap)	55-59	56	Not performed	Satisfactory	Positive	ASC-US	Low-grade / equivocal squamous	
P05512	2019	2019-Q3	2019-09	Pre-pandemic	Cytology (Pap)	45-49	46	Not performed	Satisfactory	NILM	NILM	Negative (NILM)	
P05513	2019	2019-Q3	2019-09	Pre-pandemic	Cytology (Pap)	60-64	60	Not performed	Satisfactory	NILM	NILM	Negative (NILM)	
P05514	2019	2019-Q3	2019-09	Pre-pandemic	Cytology (Pap)	50-54	52	Not performed	Satisfactory	NILM	NILM	Negative (NILM)	
P05515	2019	2019-Q3	2019-09	Pre-pandemic	Cytology (Pap)	25-29	28	Not performed	Satisfactory	NILM	NILM	Negative (NILM)	
P05516	2019	2019-Q3	2019-09	Pre-pandemic	Cytology (Pap)	45-49	48	Not performed	Satisfactory	NILM	NILM	Negative (NILM)	
P05517	2019	2019-Q3	2019-09	Pre-pandemic	Cytology (Pap)	25-29	25	Not performed	Satisfactory	NILM	NILM	Negative (NILM)	
P05518	2019	2019-Q3	2019-09	Pre-pandemic	Cytology (Pap)	55-59	56	Not performed	Satisfactory	Positive	ASC-US	Low-grade / equivocal squamous	

patient_id	year	quarter	month	period	primary_test	age_group	age_years	hpv_result	adequacy	result	bethesda	grade	infection
P05519	2019	2019-Q3	2019-09	Pre-pandemic	Cytology (Pap)	55-59	56	Not performed	Satisfactory	NILM	NILM	Negative (NILM)	
P05520	2019	2019-Q3	2019-09	Pre-pandemic	Cytology (Pap)	35-39	37	Not performed	Satisfactory	NILM	NILM	Negative (NILM)	
P05521	2019	2019-Q3	2019-09	Pre-pandemic	Cytology (Pap)	55-59	59	Not performed	Satisfactory	NILM	NILM	Negative (NILM)	
P05522	2019	2019-Q3	2019-09	Pre-pandemic	Cytology (Pap)	40-44	44	Not performed	Satisfactory	NILM	NILM	Negative (NILM)	
P05523	2019	2019-Q3	2019-09	Pre-pandemic	Cytology (Pap)	45-49	47	Not performed	Satisfactory	NILM	NILM	Negative (NILM)	
P05524	2019	2019-Q3	2019-09	Pre-pandemic	Cytology (Pap)	25-29	29	Not performed	Satisfactory	NILM	NILM	Negative (NILM)	
P05525	2019	2019-Q3	2019-09	Pre-pandemic	Cytology (Pap)	35-39	36	Not performed	Satisfactory	NILM	NILM	Negative (NILM)	
P05526	2019	2019-Q3	2019-09	Pre-pandemic	Cytology (Pap)	45-49	46	Not performed	Satisfactory	NILM	NILM	Negative (NILM)	
P05527	2019	2019-Q3	2019-09	Pre-pandemic	Cytology (Pap)	40-44	42	Not performed	Satisfactory	NILM	NILM	Negative (NILM)	
P05528	2019	2019-Q3	2019-09	Pre-pandemic	Cytology (Pap)	55-59	55	Not performed	Satisfactory	NILM	NILM	Negative (NILM)	
P05529	2019	2019-Q3	2019-09	Pre-pandemic	Cytology (Pap)	50-54	52	Not performed	Satisfactory	NILM	NILM	Negative (NILM)	
P05530	2019	2019-Q3	2019-09	Pre-pandemic	Cytology (Pap)	30-34	33	Not performed	Satisfactory	NILM	NILM	Negative (NILM)	
P05531	2019	2019-Q3	2019-09	Pre-pandemic	Cytology (Pap)	50-54	52	Not performed	Satisfactory	NILM	NILM	Negative (NILM)	
P05532	2019	2019-Q3	2019-09	Pre-pandemic	Cytology (Pap)	50-54	51	Not performed	Satisfactory	NILM	NILM	Negative (NILM)	
P05533	2019	2019-Q3	2019-09	Pre-pandemic	Cytology (Pap)	50-54	53	Not performed	Satisfactory	NILM	NILM	Negative (NILM)	
P05534	2019	2019-Q3	2019-09	Pre-pandemic	Cytology (Pap)	40-44	44	Not performed	Satisfactory	NILM	NILM	Negative (NILM)	
P05535	2019	2019-Q3	2019-09	Pre-pandemic	Cytology (Pap)	50-54	50	Not performed	Satisfactory	NILM	NILM	Negative (NILM)	
P05536	2019	2019-Q3	2019-09	Pre-pandemic	Cytology (Pap)	45-49	46	Not performed	Satisfactory	Positive	HSIL	High-grade squamous	
P05537	2019	2019-Q3	2019-09	Pre-pandemic	Cytology (Pap)	35-39	37	Not performed	Satisfactory	NILM	NILM	Negative (NILM)	
P05538	2019	2019-Q3	2019-09	Pre-pandemic	Cytology (Pap)	50-54	52	Not performed	Satisfactory	Positive	ASC-US	Low-grade / equivocal squamous	
P05539	2019	2019-Q3	2019-09	Pre-pandemic	Cytology (Pap)	60-64	64	Not performed	Satisfactory	NILM	NILM	Negative (NILM)	
P05540	2019	2019-Q3	2019-09	Pre-pandemic	Cytology (Pap)	40-44	42	Not performed	Satisfactory	NILM	NILM	Negative (NILM)	
P05541	2019	2019-Q3	2019-09	Pre-pandemic	Cytology (Pap)	40-44	41	Not performed	Satisfactory	NILM	NILM	Negative (NILM)	
P05542	2019	2019-Q3	2019-09	Pre-pandemic	Cytology (Pap)	40-44	40	Not performed	Satisfactory	NILM	NILM	Negative (NILM)	
P05543	2019	2019-Q3	2019-09	Pre-pandemic	Cytology (Pap)	25-29	26	Not performed	Satisfactory	NILM	NILM	Negative (NILM)	
P05544	2019	2019-Q3	2019-09	Pre-pandemic	Cytology (Pap)	55-59	59	Not performed	Satisfactory	NILM	NILM	Negative (NILM)	Candida
P05545	2019	2019-Q3	2019-09	Pre-pandemic	Cytology (Pap)	60-64	63	Not performed	Satisfactory	NILM	NILM	Negative (NILM)	
P05546	2019	2019-Q3	2019-09	Pre-pandemic	Cytology (Pap)	50-54	51	Not performed	Satisfactory	NILM	NILM	Negative (NILM)	
P05547	2019	2019-Q3	2019-09	Pre-pandemic	Cytology (Pap)	35-39	39	Not performed	Satisfactory	NILM	NILM	Negative (NILM)	
P05548	2019	2019-Q3	2019-09	Pre-pandemic	Cytology (Pap)	50-54	54	Not performed	Satisfactory	NILM	NILM	Negative (NILM)	
P05549	2019	2019-Q3	2019-09	Pre-pandemic	Cytology (Pap)	50-54	52	Not performed	Unsatisfactory	Unsatisfactory	Unsatisfactory	Unsatisfactory	
P05550	2019	2019-Q3	2019-09	Pre-pandemic	Cytology (Pap)	50-54	54	Not performed	Satisfactory	NILM	NILM	Negative (NILM)	
P05551	2019	2019-Q3	2019-09	Pre-pandemic	Cytology (Pap)	40-44	44	Not performed	Satisfactory	NILM	NILM	Negative (NILM)	
P05552	2019	2019-Q3	2019-09	Pre-pandemic	Cytology (Pap)	50-54	53	Not performed	Satisfactory	NILM	NILM	Negative (NILM)	
P05553	2019	2019-Q3	2019-09	Pre-pandemic	Cytology (Pap)	35-39	38	Not performed	Satisfactory	NILM	NILM	Negative (NILM)	
P05554	2019	2019-Q3	2019-09	Pre-pandemic	Cytology (Pap)	25-29	27	Not performed	Satisfactory	NILM	NILM	Negative (NILM)	
P05555	2019	2019-Q3	2019-09	Pre-pandemic	Cytology (Pap)	50-54	52	Not performed	Satisfactory	NILM	NILM	Negative (NILM)	
P05556	2019	2019-Q3	2019-09	Pre-pandemic	Cytology (Pap)	55-59	58	Not performed	Satisfactory	NILM	NILM	Negative (NILM)	
P05557	2019	2019-Q3	2019-09	Pre-pandemic	Cytology (Pap)	25-29	25	Not performed	Satisfactory	Positive	ASC-US	Low-grade / equivocal squamous	
P05558	2019	2019-Q3	2019-09	Pre-pandemic	Cytology (Pap)	35-39	37	Not performed	Satisfactory	NILM	NILM	Negative (NILM)	
P05559	2019	2019-Q3	2019-09	Pre-pandemic	Cytology (Pap)	55-59	58	Not performed	Satisfactory	Positive	LSIL	Low-grade / equivocal squamous	
P05560	2019	2019-Q3	2019-09	Pre-pandemic	Cytology (Pap)	25-29	27	Not performed	Satisfactory	NILM	NILM	Negative (NILM)	
P05561	2019	2019-Q3	2019-09	Pre-pandemic	Cytology (Pap)	30-34	32	Not performed	Satisfactory	NILM	NILM	Negative (NILM)	
P05562	2019	2019-Q3	2019-09	Pre-pandemic	Cytology (Pap)	30-34	32	Not performed	Satisfactory	NILM	NILM	Negative (NILM)	
P05563	2019	2019-Q3	2019-09	Pre-pandemic	Cytology (Pap)	50-54	51	Not performed	Satisfactory	NILM	NILM	Negative (NILM)	
P05564	2019	2019-Q3	2019-09	Pre-pandemic	Cytology (Pap)	55-59	59	Not performed	Satisfactory	NILM	NILM	Negative (NILM)	
P05565	2019	2019-Q3	2019-09	Pre-pandemic	Cytology (Pap)	40-44	44	Not performed	Satisfactory	NILM	NILM	Negative (NILM)	
P05566	2019	2019-Q3	2019-09	Pre-pandemic	Cytology (Pap)	40-44	44	Not performed	Satisfactory	NILM	NILM	Negative (NILM)	
P05567	2019	2019-Q3	2019-09	Pre-pandemic	Cytology (Pap)	50-54	53	Not performed	Satisfactory	NILM	NILM	Negative (NILM)	Trichomonas
P05568	2019	2019-Q3	2019-09	Pre-pandemic	Cytology (Pap)	50-54	51	Not performed	Satisfactory	NILM	NILM	Negative (NILM)	
P05569	2019	2019-Q3	2019-09	Pre-pandemic	Cytology (Pap)	30-34	31	Not performed	Satisfactory	NILM	NILM	Negative (NILM)	
P05570	2019	2019-Q3	2019-09	Pre-pandemic	Cytology (Pap)	25-29	26	Not performed	Unsatisfactory	Unsatisfactory	Unsatisfactory	Unsatisfactory	
P05571	2019	2019-Q3	2019-09	Pre-pandemic	Cytology (Pap)	55-59	58	Not performed	Satisfactory	NILM	NILM	Negative (NILM)	
P05572	2019	2019-Q3	2019-09	Pre-pandemic	Cytology (Pap)	50-54	50	Not performed	Satisfactory	NILM	NILM	Negative (NILM)	
P05573	2019	2019-Q3	2019-09	Pre-pandemic	Cytology (Pap)	30-34	30	Not performed	Satisfactory	NILM	NILM	Negative (NILM)	
P05574	2019	2019-Q3	2019-09	Pre-pandemic	Cytology (Pap)	35-39	37	Not performed	Satisfactory	NILM	NILM	Negative (NILM)	
P05575	2019	2019-Q3	2019-09	Pre-pandemic	Cytology (Pap)	45-49	48	Not performed	Satisfactory	NILM	NILM	Negative (NILM)	
P05576	2019	2019-Q3	2019-09	Pre-pandemic	Cytology (Pap)	45-49	49	Not performed	Satisfactory	NILM	NILM	Negative (NILM)	
P05577	2019	2019-Q3	2019-09	Pre-pandemic	Cytology (Pap)	35-39	39	Not performed	Satisfactory	NILM	NILM	Negative (NILM)	
P05578	2019	2019-Q3	2019-09	Pre-pandemic	Cytology (Pap)	30-34	34	Not performed	Satisfactory	NILM	NILM	Negative (NILM)	
P05579	2019	2019-Q3	2019-09	Pre-pandemic	Cytology (Pap)	55-59	56	Not performed	Satisfactory	NILM	NILM	Negative (NILM)	
P05580	2019	2019-Q3	2019-09	Pre-pandemic	Cytology (Pap)	50-54	54	Not performed	Satisfactory	NILM	NILM	Negative (NILM)	
P05581	2019	2019-Q3	2019-09	Pre-pandemic	Cytology (Pap)	50-54	52	Not performed	Satisfactory	NILM	NILM	Negative (NILM)	
P05582	2019	2019-Q3	2019-09	Pre-pandemic	Cytology (Pap)	25-29	27	Not performed	Satisfactory	NILM	NILM	Negative (NILM)	
P05583	2019	2019-Q3	2019-09	Pre-pandemic	Cytology (Pap)	45-49	46	Not performed	Satisfactory	NILM	NILM	Negative (NILM)	
P05584	2019	2019-Q3	2019-09	Pre-pandemic	Cytology (Pap)	30-34	32	Not performed	Satisfactory	NILM	NILM	Negative (NILM)	
P05585	2019	2019-Q3	2019-09	Pre-pandemic	Cytology (Pap)	35-39	36	Not performed	Satisfactory	Positive	LSIL	Low-grade / equivocal squamous	Candida
P05586	2019	2019-Q3	2019-09	Pre-pandemic	Cytology (Pap)	50-54	50	Not performed	Satisfactory	NILM	NILM	Negative (NILM)	
P05587	2019	2019-Q3	2019-09	Pre-pandemic	Cytology (Pap)	40-44	43	Not performed	Satisfactory	NILM	NILM	Negative (NILM)	
P05588	2019	2019-Q3	2019-09	Pre-pandemic	Cytology (Pap)	35-39	36	Not performed	Satisfactory	NILM	NILM	Negative (NILM)	
P05589	2019	2019-Q3	2019-09	Pre-pandemic	Cytology (Pap)	60-64	61	Not performed	Satisfactory	NILM	NILM	Negative (NILM)	
P05590	2019	2019-Q3	2019-09	Pre-pandemic	Cytology (Pap)	45-49	47	Not performed	Satisfactory	NILM	NILM	Negative (NILM)	
P05591	2019	2019-Q3	2019-09	Pre-pandemic	Cytology (Pap)	40-44	43	Not performed	Satisfactory	NILM	NILM	Negative (NILM)	
P05592	2019	2019-Q3	2019-09	Pre-pandemic	Cytology (Pap)	40-44	44	Not performed	Satisfactory	NILM	NILM	Negative (NILM)	
P05593	2019	2019-Q3	2019-09	Pre-pandemic	Cytology (Pap)	45-49	49	Not performed	Satisfactory	NILM	NILM	Negative (NILM)	
P05594	2019	2019-Q3	2019-09	Pre-pandemic	Cytology (Pap)	60-64	63	Not performed	Satisfactory	NILM	NILM	Negative (NILM)	
P05595	2019	2019-Q3	2019-09	Pre-pandemic	Cytology (Pap)	30-34	31	Not performed	Satisfactory	NILM	NILM	Negative (NILM)	
P05596	2019	2019-Q3	2019-09	Pre-pandemic	Cytology (Pap)	40-44	41	Not performed	Unsatisfactory	Unsatisfactory	Unsatisfactory	Unsatisfactory	
P05597	2019	2019-Q3	2019-09	Pre-pandemic	Cytology (Pap)	60-64	63	Not performed	Satisfactory	NILM	NILM	Negative (NILM)	
P05598	2019	2019-Q3	2019-09	Pre-pandemic	Cytology (Pap)	45-49	45	Not performed	Satisfactory	Positive	LSIL	Low-grade / equivocal squamous	

patient_id	year	quarter	month	period	primary_test	age_group	age_years	hpv_result	adequacy	result	bethesda	grade	infection
P05599	2019	2019-Q3	2019-09	Pre-pandemic	Cytology (Pap)	35-39	39	Not performed	Satisfactory	NILM	NILM	Negative (NILM)	
P05600	2019	2019-Q3	2019-09	Pre-pandemic	Cytology (Pap)	45-49	47	Not performed	Satisfactory	Positive	HSIL	High-grade squamous	
P05601	2019	2019-Q3	2019-09	Pre-pandemic	Cytology (Pap)	55-59	56	Not performed	Satisfactory	NILM	NILM	Negative (NILM)	
P05602	2019	2019-Q3	2019-09	Pre-pandemic	Cytology (Pap)	55-59	58	Not performed	Unsatisfactory	Unsatisfactory	Unsatisfactory	Unsatisfactory	
P05603	2019	2019-Q3	2019-09	Pre-pandemic	Cytology (Pap)	45-49	48	Not performed	Satisfactory	NILM	NILM	Negative (NILM)	
P05604	2019	2019-Q3	2019-09	Pre-pandemic	Cytology (Pap)	35-39	38	Not performed	Satisfactory	NILM	NILM	Negative (NILM)	
P05605	2019	2019-Q3	2019-09	Pre-pandemic	Cytology (Pap)	30-34	30	Not performed	Satisfactory	NILM	NILM	Negative (NILM)	
P05606	2019	2019-Q3	2019-09	Pre-pandemic	Cytology (Pap)	50-54	54	Not performed	Satisfactory	Positive	ASC-US	Low-grade / equivocal squamous	
P05607	2019	2019-Q3	2019-09	Pre-pandemic	Cytology (Pap)	25-29	25	Not performed	Satisfactory	Positive	LSIL	Low-grade / equivocal squamous	
P05608	2019	2019-Q3	2019-09	Pre-pandemic	Cytology (Pap)	55-59	55	Not performed	Satisfactory	NILM	NILM	Negative (NILM)	
P05609	2019	2019-Q3	2019-09	Pre-pandemic	Cytology (Pap)	30-34	30	Not performed	Satisfactory	NILM	NILM	Negative (NILM)	
P05610	2019	2019-Q4	2019-10	Pre-pandemic	Cytology (Pap)	35-39	39	Not performed	Satisfactory	NILM	NILM	Negative (NILM)	
P05611	2019	2019-Q4	2019-10	Pre-pandemic	Cytology (Pap)	55-59	58	Not performed	Satisfactory	NILM	NILM	Negative (NILM)	
P05612	2019	2019-Q4	2019-10	Pre-pandemic	Cytology (Pap)	45-49	49	Not performed	Satisfactory	NILM	NILM	Negative (NILM)	
P05613	2019	2019-Q4	2019-10	Pre-pandemic	Cytology (Pap)	45-49	45	Not performed	Satisfactory	NILM	NILM	Negative (NILM)	
P05614	2019	2019-Q4	2019-10	Pre-pandemic	Cytology (Pap)	55-59	58	Not performed	Satisfactory	NILM	NILM	Negative (NILM)	
P05615	2019	2019-Q4	2019-10	Pre-pandemic	Cytology (Pap)	45-49	46	Not performed	Satisfactory	Positive	ASC-US	Low-grade / equivocal squamous	
P05616	2019	2019-Q4	2019-10	Pre-pandemic	Cytology (Pap)	55-59	56	Not performed	Satisfactory	NILM	NILM	Negative (NILM)	
P05617	2019	2019-Q4	2019-10	Pre-pandemic	Cytology (Pap)	50-54	52	Not performed	Satisfactory	NILM	NILM	Negative (NILM)	
P05618	2019	2019-Q4	2019-10	Pre-pandemic	Cytology (Pap)	55-59	58	Not performed	Satisfactory	NILM	NILM	Negative (NILM)	
P05619	2019	2019-Q4	2019-10	Pre-pandemic	Cytology (Pap)	35-39	38	Not performed	Satisfactory	NILM	NILM	Negative (NILM)	
P05620	2019	2019-Q4	2019-10	Pre-pandemic	Cytology (Pap)	50-54	52	Not performed	Satisfactory	NILM	NILM	Negative (NILM)	Trichomonas
P05621	2019	2019-Q4	2019-10	Pre-pandemic	Cytology (Pap)	50-54	50	Not performed	Satisfactory	NILM	NILM	Negative (NILM)	
P05622	2019	2019-Q4	2019-10	Pre-pandemic	Cytology (Pap)	35-39	35	Not performed	Satisfactory	NILM	NILM	Negative (NILM)	
P05623	2019	2019-Q4	2019-10	Pre-pandemic	Cytology (Pap)	30-34	30	Not performed	Satisfactory	NILM	NILM	Negative (NILM)	
P05624	2019	2019-Q4	2019-10	Pre-pandemic	Cytology (Pap)	45-49	47	Not performed	Unsatisfactory	Unsatisfactory	Unsatisfactory	Unsatisfactory	
P05625	2019	2019-Q4	2019-10	Pre-pandemic	Cytology (Pap)	40-44	40	Not performed	Satisfactory	NILM	NILM	Negative (NILM)	
P05626	2019	2019-Q4	2019-10	Pre-pandemic	Cytology (Pap)	40-44	40	Not performed	Satisfactory	Positive	ASC-US	Low-grade / equivocal squamous	
P05627	2019	2019-Q4	2019-10	Pre-pandemic	Cytology (Pap)	45-49	48	Not performed	Satisfactory	NILM	NILM	Negative (NILM)	
P05628	2019	2019-Q4	2019-10	Pre-pandemic	Cytology (Pap)	50-54	52	Not performed	Satisfactory	NILM	NILM	Negative (NILM)	
P05629	2019	2019-Q4	2019-10	Pre-pandemic	Cytology (Pap)	40-44	43	Not performed	Satisfactory	NILM	NILM	Negative (NILM)	
P05630	2019	2019-Q4	2019-10	Pre-pandemic	Cytology (Pap)	45-49	48	Not performed	Satisfactory	NILM	NILM	Negative (NILM)	
P05631	2019	2019-Q4	2019-10	Pre-pandemic	Cytology (Pap)	45-49	46	Not performed	Satisfactory	Positive	LSIL	Low-grade / equivocal squamous	
P05632	2019	2019-Q4	2019-10	Pre-pandemic	Cytology (Pap)	45-49	48	Not performed	Satisfactory	NILM	NILM	Negative (NILM)	
P05633	2019	2019-Q4	2019-10	Pre-pandemic	Cytology (Pap)	35-39	37	Not performed	Satisfactory	NILM	NILM	Negative (NILM)	
P05634	2019	2019-Q4	2019-10	Pre-pandemic	Cytology (Pap)	60-64	64	Not performed	Satisfactory	NILM	NILM	Negative (NILM)	
P05635	2019	2019-Q4	2019-10	Pre-pandemic	Cytology (Pap)	60-64	64	Not performed	Satisfactory	NILM	NILM	Negative (NILM)	
P05636	2019	2019-Q4	2019-10	Pre-pandemic	Cytology (Pap)	25-29	25	Not performed	Satisfactory	NILM	NILM	Negative (NILM)	
P05637	2019	2019-Q4	2019-10	Pre-pandemic	Cytology (Pap)	35-39	37	Not performed	Satisfactory	NILM	NILM	Negative (NILM)	
P05638	2019	2019-Q4	2019-10	Pre-pandemic	Cytology (Pap)	50-54	53	Not performed	Satisfactory	NILM	NILM	Negative (NILM)	
P05639	2019	2019-Q4	2019-10	Pre-pandemic	Cytology (Pap)	45-49	46	Not performed	Satisfactory	NILM	NILM	Negative (NILM)	
P05640	2019	2019-Q4	2019-10	Pre-pandemic	Cytology (Pap)	45-49	48	Not performed	Satisfactory	NILM	NILM	Negative (NILM)	
P05641	2019	2019-Q4	2019-10	Pre-pandemic	Cytology (Pap)	50-54	51	Not performed	Satisfactory	NILM	NILM	Negative (NILM)	
P05642	2019	2019-Q4	2019-10	Pre-pandemic	Cytology (Pap)	40-44	42	Not performed	Satisfactory	NILM	NILM	Negative (NILM)	
P05643	2019	2019-Q4	2019-10	Pre-pandemic	Cytology (Pap)	40-44	41	Not performed	Satisfactory	Positive	ASC-US	Low-grade / equivocal squamous	Candida
P05644	2019	2019-Q4	2019-10	Pre-pandemic	Cytology (Pap)	45-49	46	Not performed	Satisfactory	Positive	HSIL	High-grade squamous	
P05645	2019	2019-Q4	2019-10	Pre-pandemic	Cytology (Pap)	50-54	53	Not performed	Satisfactory	NILM	NILM	Negative (NILM)	
P05646	2019	2019-Q4	2019-10	Pre-pandemic	Cytology (Pap)	50-54	50	Not performed	Satisfactory	NILM	NILM	Negative (NILM)	
P05647	2019	2019-Q4	2019-10	Pre-pandemic	Cytology (Pap)	30-34	32	Not performed	Satisfactory	Positive	HSIL	High-grade squamous	
P05648	2019	2019-Q4	2019-10	Pre-pandemic	Cytology (Pap)	45-49	49	Not performed	Satisfactory	NILM	NILM	Negative (NILM)	
P05649	2019	2019-Q4	2019-10	Pre-pandemic	Cytology (Pap)	40-44	43	Not performed	Satisfactory	NILM	NILM	Negative (NILM)	
P05650	2019	2019-Q4	2019-10	Pre-pandemic	Cytology (Pap)	35-39	37	Not performed	Satisfactory	NILM	NILM	Negative (NILM)	
P05651	2019	2019-Q4	2019-10	Pre-pandemic	Cytology (Pap)	35-39	35	Not performed	Satisfactory	NILM	NILM	Negative (NILM)	
P05652	2019	2019-Q4	2019-10	Pre-pandemic	Cytology (Pap)	35-39	35	Not performed	Satisfactory	NILM	NILM	Negative (NILM)	
P05653	2019	2019-Q4	2019-10	Pre-pandemic	Cytology (Pap)	40-44	44	Not performed	Satisfactory	Positive	ASC-US	Low-grade / equivocal squamous	
P05654	2019	2019-Q4	2019-10	Pre-pandemic	Cytology (Pap)	35-39	35	Not performed	Satisfactory	NILM	NILM	Negative (NILM)	
P05655	2019	2019-Q4	2019-10	Pre-pandemic	Cytology (Pap)	45-49	47	Not performed	Satisfactory	NILM	NILM	Negative (NILM)	
P05656	2019	2019-Q4	2019-10	Pre-pandemic	Cytology (Pap)	45-49	46	Not performed	Unsatisfactory	Unsatisfactory	Unsatisfactory	Unsatisfactory	
P05657	2019	2019-Q4	2019-10	Pre-pandemic	Cytology (Pap)	30-34	31	Not performed	Satisfactory	NILM	NILM	Negative (NILM)	
P05658	2019	2019-Q4	2019-10	Pre-pandemic	Cytology (Pap)	35-39	35	Not performed	Satisfactory	NILM	NILM	Negative (NILM)	
P05659	2019	2019-Q4	2019-10	Pre-pandemic	Cytology (Pap)	50-54	50	Not performed	Satisfactory	NILM	NILM	Negative (NILM)	
P05660	2019	2019-Q4	2019-10	Pre-pandemic	Cytology (Pap)	25-29	26	Not performed	Satisfactory	NILM	NILM	Negative (NILM)	
P05661	2019	2019-Q4	2019-10	Pre-pandemic	Cytology (Pap)	45-49	48	Not performed	Satisfactory	NILM	NILM	Negative (NILM)	
P05662	2019	2019-Q4	2019-10	Pre-pandemic	Cytology (Pap)	55-59	56	Not performed	Satisfactory	NILM	NILM	Negative (NILM)	
P05663	2019	2019-Q4	2019-10	Pre-pandemic	Cytology (Pap)	50-54	51	Not performed	Satisfactory	Positive	ASC-H	High-grade squamous	
P05664	2019	2019-Q4	2019-10	Pre-pandemic	Cytology (Pap)	45-49	49	Not performed	Satisfactory	NILM	NILM	Negative (NILM)	
P05665	2019	2019-Q4	2019-10	Pre-pandemic	Cytology (Pap)	30-34	33	Not performed	Satisfactory	NILM	NILM	Negative (NILM)	
P05666	2019	2019-Q4	2019-10	Pre-pandemic	Cytology (Pap)	45-49	48	Not performed	Satisfactory	Positive	LSIL	Low-grade / equivocal squamous	
P05667	2019	2019-Q4	2019-10	Pre-pandemic	Cytology (Pap)	55-59	58	Not performed	Unsatisfactory	Unsatisfactory	Unsatisfactory	Unsatisfactory	
P05668	2019	2019-Q4	2019-10	Pre-pandemic	Cytology (Pap)	35-39	37	Not performed	Satisfactory	Positive	HSIL	High-grade squamous	
P05669	2019	2019-Q4	2019-10	Pre-pandemic	Cytology (Pap)	50-54	52	Not performed	Satisfactory	NILM	NILM	Negative (NILM)	
P05670	2019	2019-Q4	2019-10	Pre-pandemic	Cytology (Pap)	45-49	45	Not performed	Unsatisfactory	Unsatisfactory	Unsatisfactory	Unsatisfactory	
P05671	2019	2019-Q4	2019-10	Pre-pandemic	Cytology (Pap)	55-59	58	Not performed	Satisfactory	NILM	NILM	Negative (NILM)	
P05672	2019	2019-Q4	2019-10	Pre-pandemic	Cytology (Pap)	45-49	49	Not performed	Satisfactory	NILM	NILM	Negative (NILM)	
P05673	2019	2019-Q4	2019-10	Pre-pandemic	Cytology (Pap)	45-49	48	Not performed	Satisfactory	Positive	ASC-US	Low-grade / equivocal squamous	
P05674	2019	2019-Q4	2019-10	Pre-pandemic	Cytology (Pap)	30-34	33	Not performed	Satisfactory	NILM	NILM	Negative (NILM)	
P05675	2019	2019-Q4	2019-10	Pre-pandemic	Cytology (Pap)	30-34	33	Not performed	Satisfactory	NILM	NILM	Negative (NILM)	
P05676	2019	2019-Q4	2019-10	Pre-pandemic	Cytology (Pap)	60-64	64	Not performed	Satisfactory	NILM	NILM	Negative (NILM)	
P05677	2019	2019-Q4	2019-10	Pre-pandemic	Cytology (Pap)	40-44	42	Not performed	Satisfactory	NILM	NILM	Negative (NILM)	
P05678	2019	2019-Q4	2019-10	Pre-pandemic	Cytology (Pap)	30-34	33	Not performed	Satisfactory	NILM	NILM	Negative (NILM)	

patient_id	year	quarter	month	period	primary_test	age_group	age_years	hpv_result	adequacy	result	bethesda	grade	infection
P05679	2019	2019-Q4	2019-10	Pre-pandemic	Cytology (Pap)	35-39	36	Not performed	Satisfactory	NILM	NILM	Negative (NILM)	
P05680	2019	2019-Q4	2019-10	Pre-pandemic	Cytology (Pap)	30-34	30	Not performed	Satisfactory	NILM	NILM	Negative (NILM)	Trichomonas
P05681	2019	2019-Q4	2019-10	Pre-pandemic	Cytology (Pap)	30-34	33	Not performed	Satisfactory	NILM	NILM	Negative (NILM)	
P05682	2019	2019-Q4	2019-10	Pre-pandemic	Cytology (Pap)	40-44	44	Not performed	Satisfactory	NILM	NILM	Negative (NILM)	
P05683	2019	2019-Q4	2019-10	Pre-pandemic	Cytology (Pap)	40-44	40	Not performed	Satisfactory	NILM	NILM	Negative (NILM)	
P05684	2019	2019-Q4	2019-10	Pre-pandemic	Cytology (Pap)	55-59	57	Not performed	Unsatisfactory	Unsatisfactory	Unsatisfactory	Unsatisfactory	
P05685	2019	2019-Q4	2019-10	Pre-pandemic	Cytology (Pap)	40-44	41	Not performed	Satisfactory	NILM	NILM	Negative (NILM)	
P05686	2019	2019-Q4	2019-10	Pre-pandemic	Cytology (Pap)	50-54	53	Not performed	Satisfactory	NILM	NILM	Negative (NILM)	
P05687	2019	2019-Q4	2019-10	Pre-pandemic	Cytology (Pap)	60-64	61	Not performed	Satisfactory	NILM	NILM	Negative (NILM)	
P05688	2019	2019-Q4	2019-10	Pre-pandemic	Cytology (Pap)	60-64	62	Not performed	Satisfactory	NILM	NILM	Negative (NILM)	
P05689	2019	2019-Q4	2019-10	Pre-pandemic	Cytology (Pap)	35-39	35	Not performed	Satisfactory	Positive	HSIL	High-grade squamous	
P05690	2019	2019-Q4	2019-10	Pre-pandemic	Cytology (Pap)	50-54	50	Not performed	Satisfactory	Positive	ASC-US	Low-grade / equivocal squamous	
P05691	2019	2019-Q4	2019-10	Pre-pandemic	Cytology (Pap)	30-34	34	Not performed	Satisfactory	NILM	NILM	Negative (NILM)	
P05692	2019	2019-Q4	2019-10	Pre-pandemic	Cytology (Pap)	45-49	49	Not performed	Satisfactory	NILM	NILM	Negative (NILM)	
P05693	2019	2019-Q4	2019-10	Pre-pandemic	Cytology (Pap)	40-44	40	Not performed	Satisfactory	NILM	NILM	Negative (NILM)	
P05694	2019	2019-Q4	2019-10	Pre-pandemic	Cytology (Pap)	50-54	51	Not performed	Satisfactory	NILM	NILM	Negative (NILM)	
P05695	2019	2019-Q4	2019-10	Pre-pandemic	Cytology (Pap)	45-49	48	Not performed	Satisfactory	NILM	NILM	Negative (NILM)	
P05696	2019	2019-Q4	2019-10	Pre-pandemic	Cytology (Pap)	50-54	50	Not performed	Satisfactory	NILM	NILM	Negative (NILM)	
P05697	2019	2019-Q4	2019-10	Pre-pandemic	Cytology (Pap)	30-34	31	Not performed	Satisfactory	NILM	NILM	Negative (NILM)	
P05698	2019	2019-Q4	2019-10	Pre-pandemic	Cytology (Pap)	40-44	40	Not performed	Satisfactory	Positive	ASC-US	Low-grade / equivocal squamous	
P05699	2019	2019-Q4	2019-10	Pre-pandemic	Cytology (Pap)	60-64	62	Not performed	Satisfactory	NILM	NILM	Negative (NILM)	
P05700	2019	2019-Q4	2019-10	Pre-pandemic	Cytology (Pap)	55-59	55	Not performed	Satisfactory	NILM	NILM	Negative (NILM)	
P05701	2019	2019-Q4	2019-10	Pre-pandemic	Cytology (Pap)	30-34	34	Not performed	Satisfactory	NILM	NILM	Negative (NILM)	
P05702	2019	2019-Q4	2019-10	Pre-pandemic	Cytology (Pap)	40-44	44	Not performed	Satisfactory	NILM	NILM	Negative (NILM)	
P05703	2019	2019-Q4	2019-10	Pre-pandemic	Cytology (Pap)	50-54	52	Not performed	Satisfactory	NILM	NILM	Negative (NILM)	
P05704	2019	2019-Q4	2019-10	Pre-pandemic	Cytology (Pap)	45-49	49	Not performed	Satisfactory	NILM	NILM	Negative (NILM)	
P05705	2019	2019-Q4	2019-10	Pre-pandemic	Cytology (Pap)	40-44	40	Not performed	Satisfactory	NILM	NILM	Negative (NILM)	
P05706	2019	2019-Q4	2019-10	Pre-pandemic	Cytology (Pap)	50-54	52	Not performed	Satisfactory	NILM	NILM	Negative (NILM)	
P05707	2019	2019-Q4	2019-10	Pre-pandemic	Cytology (Pap)	30-34	31	Not performed	Satisfactory	NILM	NILM	Negative (NILM)	
P05708	2019	2019-Q4	2019-10	Pre-pandemic	Cytology (Pap)	30-34	31	Not performed	Satisfactory	NILM	NILM	Negative (NILM)	
P05709	2019	2019-Q4	2019-10	Pre-pandemic	Cytology (Pap)	50-54	53	Not performed	Satisfactory	NILM	NILM	Negative (NILM)	
P05710	2019	2019-Q4	2019-10	Pre-pandemic	Cytology (Pap)	40-44	42	Not performed	Satisfactory	NILM	NILM	Negative (NILM)	
P05711	2019	2019-Q4	2019-10	Pre-pandemic	Cytology (Pap)	40-44	42	Not performed	Satisfactory	NILM	NILM	Negative (NILM)	
P05712	2019	2019-Q4	2019-10	Pre-pandemic	Cytology (Pap)	35-39	39	Not performed	Satisfactory	NILM	NILM	Negative (NILM)	
P05713	2019	2019-Q4	2019-10	Pre-pandemic	Cytology (Pap)	25-29	29	Not performed	Satisfactory	NILM	NILM	Negative (NILM)	
P05714	2019	2019-Q4	2019-10	Pre-pandemic	Cytology (Pap)	50-54	53	Not performed	Satisfactory	NILM	NILM	Negative (NILM)	
P05715	2019	2019-Q4	2019-10	Pre-pandemic	Cytology (Pap)	40-44	43	Not performed	Satisfactory	NILM	NILM	Negative (NILM)	
P05716	2019	2019-Q4	2019-10	Pre-pandemic	Cytology (Pap)	25-29	27	Not performed	Satisfactory	NILM	NILM	Negative (NILM)	
P05717	2019	2019-Q4	2019-10	Pre-pandemic	Cytology (Pap)	40-44	43	Not performed	Satisfactory	NILM	NILM	Negative (NILM)	
P05718	2019	2019-Q4	2019-10	Pre-pandemic	Cytology (Pap)	40-44	40	Not performed	Satisfactory	NILM	NILM	Negative (NILM)	
P05719	2019	2019-Q4	2019-10	Pre-pandemic	Cytology (Pap)	40-44	42	Not performed	Satisfactory	NILM	NILM	Negative (NILM)	
P05720	2019	2019-Q4	2019-10	Pre-pandemic	Cytology (Pap)	35-39	35	Not performed	Satisfactory	NILM	NILM	Negative (NILM)	
P05721	2019	2019-Q4	2019-10	Pre-pandemic	Cytology (Pap)	40-44	44	Not performed	Satisfactory	NILM	NILM	Negative (NILM)	
P05722	2019	2019-Q4	2019-10	Pre-pandemic	Cytology (Pap)	40-44	43	Not performed	Satisfactory	NILM	NILM	Negative (NILM)	
P05723	2019	2019-Q4	2019-10	Pre-pandemic	Cytology (Pap)	40-44	44	Not performed	Satisfactory	NILM	NILM	Negative (NILM)	
P05724	2019	2019-Q4	2019-10	Pre-pandemic	Cytology (Pap)	40-44	40	Not performed	Satisfactory	NILM	NILM	Negative (NILM)	
P05725	2019	2019-Q4	2019-10	Pre-pandemic	Cytology (Pap)	50-54	53	Not performed	Satisfactory	NILM	NILM	Negative (NILM)	
P05726	2019	2019-Q4	2019-10	Pre-pandemic	Cytology (Pap)	35-39	37	Not performed	Satisfactory	NILM	NILM	Negative (NILM)	
P05727	2019	2019-Q4	2019-10	Pre-pandemic	Cytology (Pap)	40-44	40	Not performed	Satisfactory	NILM	NILM	Negative (NILM)	
P05728	2019	2019-Q4	2019-10	Pre-pandemic	Cytology (Pap)	45-49	45	Not performed	Satisfactory	NILM	NILM	Negative (NILM)	
P05729	2019	2019-Q4	2019-10	Pre-pandemic	Cytology (Pap)	55-59	57	Not performed	Satisfactory	NILM	NILM	Negative (NILM)	
P05730	2019	2019-Q4	2019-10	Pre-pandemic	Cytology (Pap)	30-34	32	Not performed	Satisfactory	NILM	NILM	Negative (NILM)	
P05731	2019	2019-Q4	2019-10	Pre-pandemic	Cytology (Pap)	40-44	43	Not performed	Satisfactory	Positive	ASC-US	Low-grade / equivocal squamous	
P05732	2019	2019-Q4	2019-10	Pre-pandemic	Cytology (Pap)	55-59	57	Not performed	Satisfactory	NILM	NILM	Negative (NILM)	
P05733	2019	2019-Q4	2019-10	Pre-pandemic	Cytology (Pap)	55-59	57	Not performed	Satisfactory	NILM	NILM	Negative (NILM)	
P05734	2019	2019-Q4	2019-10	Pre-pandemic	Cytology (Pap)	50-54	53	Not performed	Satisfactory	NILM	NILM	Negative (NILM)	
P05735	2019	2019-Q4	2019-10	Pre-pandemic	Cytology (Pap)	50-54	53	Not performed	Satisfactory	NILM	NILM	Negative (NILM)	
P05736	2019	2019-Q4	2019-10	Pre-pandemic	Cytology (Pap)	45-49	49	Not performed	Satisfactory	NILM	NILM	Negative (NILM)	
P05737	2019	2019-Q4	2019-10	Pre-pandemic	Cytology (Pap)	45-49	48	Not performed	Satisfactory	NILM	NILM	Negative (NILM)	
P05738	2019	2019-Q4	2019-10	Pre-pandemic	Cytology (Pap)	35-39	38	Not performed	Satisfactory	NILM	NILM	Negative (NILM)	
P05739	2019	2019-Q4	2019-10	Pre-pandemic	Cytology (Pap)	30-34	31	Not performed	Satisfactory	NILM	NILM	Negative (NILM)	
P05740	2019	2019-Q4	2019-10	Pre-pandemic	Cytology (Pap)	35-39	39	Not performed	Satisfactory	NILM	NILM	Negative (NILM)	
P05741	2019	2019-Q4	2019-10	Pre-pandemic	Cytology (Pap)	45-49	45	Not performed	Satisfactory	NILM	NILM	Negative (NILM)	
P05742	2019	2019-Q4	2019-10	Pre-pandemic	Cytology (Pap)	45-49	48	Not performed	Satisfactory	Positive	HSIL	High-grade squamous	
P05743	2019	2019-Q4	2019-10	Pre-pandemic	Cytology (Pap)	45-49	47	Not performed	Satisfactory	NILM	NILM	Negative (NILM)	
P05744	2019	2019-Q4	2019-10	Pre-pandemic	Cytology (Pap)	40-44	40	Not performed	Satisfactory	NILM	NILM	Negative (NILM)	
P05745	2019	2019-Q4	2019-10	Pre-pandemic	Cytology (Pap)	35-39	35	Not performed	Satisfactory	NILM	NILM	Negative (NILM)	
P05746	2019	2019-Q4	2019-10	Pre-pandemic	Cytology (Pap)	40-44	42	Not performed	Satisfactory	NILM	NILM	Negative (NILM)	
P05747	2019	2019-Q4	2019-10	Pre-pandemic	Cytology (Pap)	55-59	59	Not performed	Satisfactory	NILM	NILM	Negative (NILM)	
P05748	2019	2019-Q4	2019-10	Pre-pandemic	Cytology (Pap)	40-44	41	Not performed	Satisfactory	Positive	ASC-US	Low-grade / equivocal squamous	
P05749	2019	2019-Q4	2019-10	Pre-pandemic	Cytology (Pap)	45-49	46	Not performed	Satisfactory	NILM	NILM	Negative (NILM)	
P05750	2019	2019-Q4	2019-10	Pre-pandemic	Cytology (Pap)	35-39	35	Not performed	Satisfactory	NILM	NILM	Negative (NILM)	
P05751	2019	2019-Q4	2019-10	Pre-pandemic	Cytology (Pap)	40-44	41	Not performed	Satisfactory	NILM	NILM	Negative (NILM)	
P05752	2019	2019-Q4	2019-10	Pre-pandemic	Cytology (Pap)	40-44	42	Not performed	Satisfactory	NILM	NILM	Negative (NILM)	
P05753	2019	2019-Q4	2019-10	Pre-pandemic	Cytology (Pap)	30-34	34	Not performed	Satisfactory	NILM	NILM	Negative (NILM)	
P05754	2019	2019-Q4	2019-10	Pre-pandemic	Cytology (Pap)	50-54	51	Not performed	Satisfactory	NILM	NILM	Negative (NILM)	
P05755	2019	2019-Q4	2019-10	Pre-pandemic	Cytology (Pap)	40-44	43	Not performed	Satisfactory	NILM	NILM	Negative (NILM)	Gardnerella
P05756	2019	2019-Q4	2019-10	Pre-pandemic	Cytology (Pap)	60-64	63	Not performed	Satisfactory	Positive	ASC-US	Low-grade / equivocal squamous	
P05757	2019	2019-Q4	2019-10	Pre-pandemic	Cytology (Pap)	45-49	49	Not performed	Satisfactory	NILM	NILM	Negative (NILM)	
P05758	2019	2019-Q4	2019-10	Pre-pandemic	Cytology (Pap)	50-54	53	Not performed	Satisfactory	NILM	NILM	Negative (NILM)	

patient_id	year	quarter	month	period	primary_test	age_group	age_years	hpv_result	adequacy	result	bethesda	grade	infection
P05759	2019	2019-Q4	2019-10	Pre-pandemic	Cytology (Pap)	35-39	37	Not performed	Satisfactory	NILM	NILM	Negative (NILM)	
P05760	2019	2019-Q4	2019-10	Pre-pandemic	Cytology (Pap)	40-44	44	Not performed	Satisfactory	NILM	NILM	Negative (NILM)	
P05761	2019	2019-Q4	2019-10	Pre-pandemic	Cytology (Pap)	45-49	48	Not performed	Satisfactory	NILM	NILM	Negative (NILM)	
P05762	2019	2019-Q4	2019-10	Pre-pandemic	Cytology (Pap)	35-39	36	Not performed	Satisfactory	NILM	NILM	Negative (NILM)	
P05763	2019	2019-Q4	2019-10	Pre-pandemic	Cytology (Pap)	40-44	44	Not performed	Unsatisfactory	Unsatisfactory	Unsatisfactory	Unsatisfactory	
P05764	2019	2019-Q4	2019-10	Pre-pandemic	Cytology (Pap)	50-54	51	Not performed	Satisfactory	NILM	NILM	Negative (NILM)	
P05765	2019	2019-Q4	2019-10	Pre-pandemic	Cytology (Pap)	25-29	28	Not performed	Satisfactory	NILM	NILM	Negative (NILM)	
P05766	2019	2019-Q4	2019-10	Pre-pandemic	Cytology (Pap)	35-39	36	Not performed	Unsatisfactory	Unsatisfactory	Unsatisfactory	Unsatisfactory	
P05767	2019	2019-Q4	2019-10	Pre-pandemic	Cytology (Pap)	45-49	47	Not performed	Satisfactory	NILM	NILM	Negative (NILM)	
P05768	2019	2019-Q4	2019-10	Pre-pandemic	Cytology (Pap)	40-44	42	Not performed	Satisfactory	Positive	HSIL	High-grade squamous	
P05769	2019	2019-Q4	2019-10	Pre-pandemic	Cytology (Pap)	50-54	52	Not performed	Satisfactory	NILM	NILM	Negative (NILM)	
P05770	2019	2019-Q4	2019-10	Pre-pandemic	Cytology (Pap)	35-39	35	Not performed	Satisfactory	NILM	NILM	Negative (NILM)	
P05771	2019	2019-Q4	2019-10	Pre-pandemic	Cytology (Pap)	30-34	34	Not performed	Satisfactory	NILM	NILM	Negative (NILM)	
P05772	2019	2019-Q4	2019-10	Pre-pandemic	Cytology (Pap)	40-44	44	Not performed	Satisfactory	NILM	NILM	Negative (NILM)	
P05773	2019	2019-Q4	2019-10	Pre-pandemic	Cytology (Pap)	35-39	37	Not performed	Unsatisfactory	Unsatisfactory	Unsatisfactory	Unsatisfactory	
P05774	2019	2019-Q4	2019-10	Pre-pandemic	Cytology (Pap)	30-34	34	Not performed	Satisfactory	Positive	LSIL	Low-grade / equivocal squamous	
P05775	2019	2019-Q4	2019-10	Pre-pandemic	Cytology (Pap)	40-44	40	Not performed	Satisfactory	NILM	NILM	Negative (NILM)	
P05776	2019	2019-Q4	2019-10	Pre-pandemic	Cytology (Pap)	45-49	48	Not performed	Satisfactory	NILM	NILM	Negative (NILM)	
P05777	2019	2019-Q4	2019-10	Pre-pandemic	Cytology (Pap)	50-54	51	Not performed	Satisfactory	NILM	NILM	Negative (NILM)	
P05778	2019	2019-Q4	2019-10	Pre-pandemic	Cytology (Pap)	40-44	41	Not performed	Satisfactory	NILM	NILM	Negative (NILM)	
P05779	2019	2019-Q4	2019-10	Pre-pandemic	Cytology (Pap)	55-59	58	Not performed	Satisfactory	NILM	NILM	Negative (NILM)	
P05780	2019	2019-Q4	2019-10	Pre-pandemic	Cytology (Pap)	30-34	30	Not performed	Satisfactory	Positive	LSIL	Low-grade / equivocal squamous	
P05781	2019	2019-Q4	2019-10	Pre-pandemic	Cytology (Pap)	30-34	34	Not performed	Satisfactory	NILM	NILM	Negative (NILM)	
P05782	2019	2019-Q4	2019-10	Pre-pandemic	Cytology (Pap)	50-54	53	Not performed	Satisfactory	NILM	NILM	Negative (NILM)	
P05783	2019	2019-Q4	2019-10	Pre-pandemic	Cytology (Pap)	30-34	31	Not performed	Satisfactory	NILM	NILM	Negative (NILM)	
P05784	2019	2019-Q4	2019-10	Pre-pandemic	Cytology (Pap)	30-34	31	Not performed	Satisfactory	NILM	NILM	Negative (NILM)	
P05785	2019	2019-Q4	2019-10	Pre-pandemic	Cytology (Pap)	40-44	44	Not performed	Satisfactory	NILM	NILM	Negative (NILM)	
P05786	2019	2019-Q4	2019-10	Pre-pandemic	Cytology (Pap)	35-39	38	Not performed	Satisfactory	NILM	NILM	Negative (NILM)	
P05787	2019	2019-Q4	2019-10	Pre-pandemic	Cytology (Pap)	25-29	29	Not performed	Satisfactory	NILM	NILM	Negative (NILM)	
P05788	2019	2019-Q4	2019-10	Pre-pandemic	Cytology (Pap)	55-59	59	Not performed	Satisfactory	NILM	NILM	Negative (NILM)	
P05789	2019	2019-Q4	2019-10	Pre-pandemic	Cytology (Pap)	40-44	41	Not performed	Satisfactory	Positive	ASC-US	Low-grade / equivocal squamous	
P05790	2019	2019-Q4	2019-10	Pre-pandemic	Cytology (Pap)	40-44	44	Not performed	Satisfactory	NILM	NILM	Negative (NILM)	
P05791	2019	2019-Q4	2019-10	Pre-pandemic	Cytology (Pap)	40-44	43	Not performed	Satisfactory	NILM	NILM	Negative (NILM)	
P05792	2019	2019-Q4	2019-10	Pre-pandemic	Cytology (Pap)	45-49	45	Not performed	Satisfactory	NILM	NILM	Negative (NILM)	
P05793	2019	2019-Q4	2019-10	Pre-pandemic	Cytology (Pap)	35-39	39	Not performed	Satisfactory	NILM	NILM	Negative (NILM)	
P05794	2019	2019-Q4	2019-10	Pre-pandemic	Cytology (Pap)	45-49	48	Not performed	Satisfactory	NILM	NILM	Negative (NILM)	
P05795	2019	2019-Q4	2019-10	Pre-pandemic	Cytology (Pap)	40-44	44	Not performed	Satisfactory	Positive	ASC-US	Low-grade / equivocal squamous	
P05796	2019	2019-Q4	2019-10	Pre-pandemic	Cytology (Pap)	40-44	42	Not performed	Satisfactory	NILM	NILM	Negative (NILM)	
P05797	2019	2019-Q4	2019-10	Pre-pandemic	Cytology (Pap)	25-29	25	Not performed	Satisfactory	NILM	NILM	Negative (NILM)	
P05798	2019	2019-Q4	2019-10	Pre-pandemic	Cytology (Pap)	45-49	47	Not performed	Satisfactory	NILM	NILM	Negative (NILM)	
P05799	2019	2019-Q4	2019-10	Pre-pandemic	Cytology (Pap)	30-34	31	Not performed	Satisfactory	NILM	NILM	Negative (NILM)	
P05800	2019	2019-Q4	2019-10	Pre-pandemic	Cytology (Pap)	35-39	35	Not performed	Satisfactory	NILM	NILM	Negative (NILM)	
P05801	2019	2019-Q4	2019-10	Pre-pandemic	Cytology (Pap)	50-54	54	Not performed	Satisfactory	NILM	NILM	Negative (NILM)	
P05802	2019	2019-Q4	2019-10	Pre-pandemic	Cytology (Pap)	45-49	47	Not performed	Satisfactory	NILM	NILM	Negative (NILM)	
P05803	2019	2019-Q4	2019-10	Pre-pandemic	Cytology (Pap)	35-39	38	Not performed	Satisfactory	NILM	NILM	Negative (NILM)	
P05804	2019	2019-Q4	2019-10	Pre-pandemic	Cytology (Pap)	40-44	44	Not performed	Satisfactory	Positive	ASC-US	Low-grade / equivocal squamous	
P05805	2019	2019-Q4	2019-10	Pre-pandemic	Cytology (Pap)	60-64	62	Not performed	Satisfactory	Positive	ASC-H	High-grade squamous	
P05806	2019	2019-Q4	2019-10	Pre-pandemic	Cytology (Pap)	45-49	47	Not performed	Satisfactory	NILM	NILM	Negative (NILM)	
P05807	2019	2019-Q4	2019-10	Pre-pandemic	Cytology (Pap)	30-34	30	Not performed	Satisfactory	Positive	ASC-US	Low-grade / equivocal squamous	
P05808	2019	2019-Q4	2019-10	Pre-pandemic	Cytology (Pap)	60-64	62	Not performed	Satisfactory	NILM	NILM	Negative (NILM)	
P05809	2019	2019-Q4	2019-10	Pre-pandemic	Cytology (Pap)	35-39	39	Not performed	Satisfactory	Positive	HSIL	High-grade squamous	
P05810	2019	2019-Q4	2019-10	Pre-pandemic	Cytology (Pap)	35-39	35	Not performed	Satisfactory	NILM	NILM	Negative (NILM)	
P05811	2019	2019-Q4	2019-10	Pre-pandemic	Cytology (Pap)	45-49	46	Not performed	Satisfactory	Positive	ASC-H	High-grade squamous	
P05812	2019	2019-Q4	2019-10	Pre-pandemic	Cytology (Pap)	50-54	53	Not performed	Unsatisfactory	Unsatisfactory	Unsatisfactory	Unsatisfactory	
P05813	2019	2019-Q4	2019-10	Pre-pandemic	Cytology (Pap)	35-39	37	Not performed	Satisfactory	Positive	ASC-US	Low-grade / equivocal squamous	
P05814	2019	2019-Q4	2019-10	Pre-pandemic	Cytology (Pap)	40-44	42	Not performed	Satisfactory	NILM	NILM	Negative (NILM)	
P05815	2019	2019-Q4	2019-10	Pre-pandemic	Cytology (Pap)	30-34	34	Not performed	Satisfactory	NILM	NILM	Negative (NILM)	
P05816	2019	2019-Q4	2019-10	Pre-pandemic	Cytology (Pap)	50-54	53	Not performed	Satisfactory	Positive	ASC-US	Low-grade / equivocal squamous	
P05817	2019	2019-Q4	2019-10	Pre-pandemic	Cytology (Pap)	40-44	44	Not performed	Unsatisfactory	Unsatisfactory	Unsatisfactory	Unsatisfactory	
P05818	2019	2019-Q4	2019-10	Pre-pandemic	Cytology (Pap)	35-39	37	Not performed	Satisfactory	Positive	ASC-US	Low-grade / equivocal squamous	
P05819	2019	2019-Q4	2019-10	Pre-pandemic	Cytology (Pap)	45-49	47	Not performed	Satisfactory	Positive	ASC-US	Low-grade / equivocal squamous	
P05820	2019	2019-Q4	2019-10	Pre-pandemic	Cytology (Pap)	40-44	42	Not performed	Satisfactory	NILM	NILM	Negative (NILM)	
P05821	2019	2019-Q4	2019-10	Pre-pandemic	Cytology (Pap)	40-44	42	Not performed	Satisfactory	Positive	LSIL	Low-grade / equivocal squamous	
P05822	2019	2019-Q4	2019-10	Pre-pandemic	Cytology (Pap)	50-54	50	Not performed	Unsatisfactory	Unsatisfactory	Unsatisfactory	Unsatisfactory	
P05823	2019	2019-Q4	2019-10	Pre-pandemic	Cytology (Pap)	40-44	40	Not performed	Satisfactory	NILM	NILM	Negative (NILM)	
P05824	2019	2019-Q4	2019-10	Pre-pandemic	Cytology (Pap)	35-39	39	Not performed	Satisfactory	NILM	NILM	Negative (NILM)	
P05825	2019	2019-Q4	2019-10	Pre-pandemic	Cytology (Pap)	60-64	63	Not performed	Satisfactory	NILM	NILM	Negative (NILM)	
P05826	2019	2019-Q4	2019-10	Pre-pandemic	Cytology (Pap)	30-34	31	Not performed	Satisfactory	NILM	NILM	Negative (NILM)	
P05827	2019	2019-Q4	2019-10	Pre-pandemic	Cytology (Pap)	35-39	38	Not performed	Satisfactory	NILM	NILM	Negative (NILM)	
P05828	2019	2019-Q4	2019-10	Pre-pandemic	Cytology (Pap)	50-54	54	Not performed	Satisfactory	NILM	NILM	Negative (NILM)	
P05829	2019	2019-Q4	2019-10	Pre-pandemic	Cytology (Pap)	50-54	53	Not performed	Satisfactory	Positive	ASC-US	Low-grade / equivocal squamous	
P05830	2019	2019-Q4	2019-10	Pre-pandemic	Cytology (Pap)	55-59	56	Not performed	Satisfactory	NILM	NILM	Negative (NILM)	
P05831	2019	2019-Q4	2019-10	Pre-pandemic	Cytology (Pap)	45-49	48	Not performed	Satisfactory	NILM	NILM	Negative (NILM)	
P05832	2019	2019-Q4	2019-10	Pre-pandemic	Cytology (Pap)	60-64	60	Not performed	Satisfactory	NILM	NILM	Negative (NILM)	
P05833	2019	2019-Q4	2019-10	Pre-pandemic	Cytology (Pap)	35-39	37	Not performed	Satisfactory	NILM	NILM	Negative (NILM)	
P05834	2019	2019-Q4	2019-10	Pre-pandemic	Cytology (Pap)	45-49	46	Not performed	Satisfactory	NILM	NILM	Negative (NILM)	
P05835	2019	2019-Q4	2019-10	Pre-pandemic	Cytology (Pap)	50-54	50	Not performed	Satisfactory	NILM	NILM	Negative (NILM)	
P05836	2019	2019-Q4	2019-10	Pre-pandemic	Cytology (Pap)	60-64	61	Not performed	Satisfactory	NILM	NILM	Negative (NILM)	
P05837	2019	2019-Q4	2019-10	Pre-pandemic	Cytology (Pap)	40-44	42	Not performed	Satisfactory	NILM	NILM	Negative (NILM)	
P05838	2019	2019-Q4	2019-10	Pre-pandemic	Cytology (Pap)	40-44	41	Not performed	Satisfactory	NILM	NILM	Negative (NILM)	

patient_id	year	quarter	month	period	primary_test	age_group	age_years	hpv_result	adequacy	result	bethesda	grade	infection
P05839	2019	2019-Q4	2019-10	Pre-pandemic	Cytology (Pap)	30-34	31	Not performed	Satisfactory	Positive	ASC-US	Low-grade / equivocal squamous	
P05840	2019	2019-Q4	2019-10	Pre-pandemic	Cytology (Pap)	40-44	44	Not performed	Satisfactory	Positive	ASC-US	Low-grade / equivocal squamous	
P05841	2019	2019-Q4	2019-10	Pre-pandemic	Cytology (Pap)	50-54	51	Not performed	Satisfactory	NILM	NILM	Negative (NILM)	
P05842	2019	2019-Q4	2019-10	Pre-pandemic	Cytology (Pap)	50-54	51	Not performed	Satisfactory	NILM	NILM	Negative (NILM)	
P05843	2019	2019-Q4	2019-10	Pre-pandemic	Cytology (Pap)	30-34	34	Not performed	Satisfactory	NILM	NILM	Negative (NILM)	
P05844	2019	2019-Q4	2019-10	Pre-pandemic	Cytology (Pap)	40-44	41	Not performed	Satisfactory	Positive	ASC-US	Low-grade / equivocal squamous	Trichomonas
P05845	2019	2019-Q4	2019-10	Pre-pandemic	Cytology (Pap)	60-64	61	Not performed	Satisfactory	NILM	NILM	Negative (NILM)	
P05846	2019	2019-Q4	2019-10	Pre-pandemic	Cytology (Pap)	30-34	33	Not performed	Satisfactory	NILM	NILM	Negative (NILM)	
P05847	2019	2019-Q4	2019-10	Pre-pandemic	Cytology (Pap)	30-34	34	Not performed	Satisfactory	NILM	NILM	Negative (NILM)	
P05848	2019	2019-Q4	2019-10	Pre-pandemic	Cytology (Pap)	55-59	56	Not performed	Satisfactory	NILM	NILM	Negative (NILM)	
P05849	2019	2019-Q4	2019-10	Pre-pandemic	Cytology (Pap)	40-44	40	Not performed	Satisfactory	NILM	NILM	Negative (NILM)	
P05850	2019	2019-Q4	2019-10	Pre-pandemic	Cytology (Pap)	55-59	59	Not performed	Satisfactory	NILM	NILM	Negative (NILM)	
P05851	2019	2019-Q4	2019-10	Pre-pandemic	Cytology (Pap)	55-59	58	Not performed	Satisfactory	NILM	NILM	Negative (NILM)	
P05852	2019	2019-Q4	2019-10	Pre-pandemic	Cytology (Pap)	50-54	50	Not performed	Satisfactory	NILM	NILM	Negative (NILM)	
P05853	2019	2019-Q4	2019-10	Pre-pandemic	Cytology (Pap)	45-49	45	Not performed	Satisfactory	NILM	NILM	Negative (NILM)	
P05854	2019	2019-Q4	2019-10	Pre-pandemic	Cytology (Pap)	35-39	39	Not performed	Satisfactory	NILM	NILM	Negative (NILM)	
P05855	2019	2019-Q4	2019-10	Pre-pandemic	Cytology (Pap)	40-44	43	Not performed	Satisfactory	NILM	NILM	Negative (NILM)	
P05856	2019	2019-Q4	2019-11	Pre-pandemic	Cytology (Pap)	45-49	46	Not performed	Satisfactory	NILM	NILM	Negative (NILM)	
P05857	2019	2019-Q4	2019-11	Pre-pandemic	Cytology (Pap)	35-39	35	Not performed	Satisfactory	NILM	NILM	Negative (NILM)	
P05858	2019	2019-Q4	2019-11	Pre-pandemic	Cytology (Pap)	35-39	35	Not performed	Satisfactory	NILM	NILM	Negative (NILM)	
P05859	2019	2019-Q4	2019-11	Pre-pandemic	Cytology (Pap)	40-44	43	Not performed	Satisfactory	NILM	NILM	Negative (NILM)	
P05860	2019	2019-Q4	2019-11	Pre-pandemic	Cytology (Pap)	30-34	32	Not performed	Satisfactory	NILM	NILM	Negative (NILM)	
P05861	2019	2019-Q4	2019-11	Pre-pandemic	Cytology (Pap)	25-29	28	Not performed	Satisfactory	NILM	NILM	Negative (NILM)	
P05862	2019	2019-Q4	2019-11	Pre-pandemic	Cytology (Pap)	45-49	46	Not performed	Satisfactory	NILM	NILM	Negative (NILM)	
P05863	2019	2019-Q4	2019-11	Pre-pandemic	Cytology (Pap)	60-64	64	Not performed	Satisfactory	Positive	ASC-US	Low-grade / equivocal squamous	
P05864	2019	2019-Q4	2019-11	Pre-pandemic	Cytology (Pap)	55-59	55	Not performed	Satisfactory	NILM	NILM	Negative (NILM)	
P05865	2019	2019-Q4	2019-11	Pre-pandemic	Cytology (Pap)	45-49	46	Not performed	Satisfactory	NILM	NILM	Negative (NILM)	
P05866	2019	2019-Q4	2019-11	Pre-pandemic	Cytology (Pap)	45-49	49	Not performed	Satisfactory	NILM	NILM	Negative (NILM)	
P05867	2019	2019-Q4	2019-11	Pre-pandemic	Cytology (Pap)	30-34	34	Not performed	Satisfactory	Positive	LSIL	Low-grade / equivocal squamous	
P05868	2019	2019-Q4	2019-11	Pre-pandemic	Cytology (Pap)	60-64	61	Not performed	Satisfactory	NILM	NILM	Negative (NILM)	
P05869	2019	2019-Q4	2019-11	Pre-pandemic	Cytology (Pap)	60-64	63	Not performed	Satisfactory	NILM	NILM	Negative (NILM)	
P05870	2019	2019-Q4	2019-11	Pre-pandemic	Cytology (Pap)	45-49	47	Not performed	Satisfactory	NILM	NILM	Negative (NILM)	
P05871	2019	2019-Q4	2019-11	Pre-pandemic	Cytology (Pap)	30-34	34	Not performed	Satisfactory	Positive	LSIL	Low-grade / equivocal squamous	
P05872	2019	2019-Q4	2019-11	Pre-pandemic	Cytology (Pap)	35-39	36	Not performed	Satisfactory	NILM	NILM	Negative (NILM)	
P05873	2019	2019-Q4	2019-11	Pre-pandemic	Cytology (Pap)	40-44	44	Not performed	Satisfactory	NILM	NILM	Negative (NILM)	
P05874	2019	2019-Q4	2019-11	Pre-pandemic	Cytology (Pap)	25-29	25	Not performed	Satisfactory	Positive	ASC-US	Low-grade / equivocal squamous	
P05875	2019	2019-Q4	2019-11	Pre-pandemic	Cytology (Pap)	50-54	51	Not performed	Satisfactory	NILM	NILM	Negative (NILM)	
P05876	2019	2019-Q4	2019-11	Pre-pandemic	Cytology (Pap)	35-39	35	Not performed	Satisfactory	NILM	NILM	Negative (NILM)	
P05877	2019	2019-Q4	2019-11	Pre-pandemic	Cytology (Pap)	40-44	44	Not performed	Satisfactory	NILM	NILM	Negative (NILM)	
P05878	2019	2019-Q4	2019-11	Pre-pandemic	Cytology (Pap)	60-64	64	Not performed	Satisfactory	NILM	NILM	Negative (NILM)	
P05879	2019	2019-Q4	2019-11	Pre-pandemic	Cytology (Pap)	55-59	57	Not performed	Satisfactory	NILM	NILM	Negative (NILM)	
P05880	2019	2019-Q4	2019-11	Pre-pandemic	Cytology (Pap)	45-49	47	Not performed	Satisfactory	NILM	NILM	Negative (NILM)	
P05881	2019	2019-Q4	2019-11	Pre-pandemic	Cytology (Pap)	35-39	39	Not performed	Satisfactory	NILM	NILM	Negative (NILM)	
P05882	2019	2019-Q4	2019-11	Pre-pandemic	Cytology (Pap)	40-44	44	Not performed	Satisfactory	NILM	NILM	Negative (NILM)	
P05883	2019	2019-Q4	2019-11	Pre-pandemic	Cytology (Pap)	40-44	41	Not performed	Satisfactory	NILM	NILM	Negative (NILM)	
P05884	2019	2019-Q4	2019-11	Pre-pandemic	Cytology (Pap)	50-54	50	Not performed	Satisfactory	NILM	NILM	Negative (NILM)	
P05885	2019	2019-Q4	2019-11	Pre-pandemic	Cytology (Pap)	25-29	28	Not performed	Unsatisfactory	Unsatisfactory	Unsatisfactory	Unsatisfactory	
P05886	2019	2019-Q4	2019-11	Pre-pandemic	Cytology (Pap)	45-49	46	Not performed	Satisfactory	NILM	NILM	Negative (NILM)	
P05887	2019	2019-Q4	2019-11	Pre-pandemic	Cytology (Pap)	55-59	59	Not performed	Satisfactory	Positive	HSIL	High-grade squamous	
P05888	2019	2019-Q4	2019-11	Pre-pandemic	Cytology (Pap)	40-44	42	Not performed	Satisfactory	NILM	NILM	Negative (NILM)	
P05889	2019	2019-Q4	2019-11	Pre-pandemic	Cytology (Pap)	55-59	57	Not performed	Satisfactory	NILM	NILM	Negative (NILM)	
P05890	2019	2019-Q4	2019-11	Pre-pandemic	Cytology (Pap)	45-49	47	Not performed	Satisfactory	Positive	HSIL	High-grade squamous	
P05891	2019	2019-Q4	2019-11	Pre-pandemic	Cytology (Pap)	45-49	45	Not performed	Satisfactory	Positive	ASC-US	Low-grade / equivocal squamous	
P05892	2019	2019-Q4	2019-11	Pre-pandemic	Cytology (Pap)	45-49	46	Not performed	Satisfactory	NILM	NILM	Negative (NILM)	
P05893	2019	2019-Q4	2019-11	Pre-pandemic	Cytology (Pap)	30-34	31	Not performed	Satisfactory	NILM	NILM	Negative (NILM)	
P05894	2019	2019-Q4	2019-11	Pre-pandemic	Cytology (Pap)	40-44	44	Not performed	Unsatisfactory	Unsatisfactory	Unsatisfactory	Unsatisfactory	
P05895	2019	2019-Q4	2019-11	Pre-pandemic	Cytology (Pap)	45-49	45	Not performed	Satisfactory	NILM	NILM	Negative (NILM)	
P05896	2019	2019-Q4	2019-11	Pre-pandemic	Cytology (Pap)	40-44	40	Not performed	Satisfactory	NILM	NILM	Negative (NILM)	
P05897	2019	2019-Q4	2019-11	Pre-pandemic	Cytology (Pap)	55-59	57	Not performed	Satisfactory	NILM	NILM	Negative (NILM)	
P05898	2019	2019-Q4	2019-11	Pre-pandemic	Cytology (Pap)	60-64	63	Not performed	Satisfactory	NILM	NILM	Negative (NILM)	
P05899	2019	2019-Q4	2019-11	Pre-pandemic	Cytology (Pap)	25-29	26	Not performed	Satisfactory	NILM	NILM	Negative (NILM)	
P05900	2019	2019-Q4	2019-11	Pre-pandemic	Cytology (Pap)	55-59	59	Not performed	Satisfactory	NILM	NILM	Negative (NILM)	
P05901	2019	2019-Q4	2019-11	Pre-pandemic	Cytology (Pap)	50-54	50	Not performed	Satisfactory	NILM	NILM	Negative (NILM)	
P05902	2019	2019-Q4	2019-11	Pre-pandemic	Cytology (Pap)	30-34	30	Not performed	Satisfactory	NILM	NILM	Negative (NILM)	
P05903	2019	2019-Q4	2019-11	Pre-pandemic	Cytology (Pap)	45-49	49	Not performed	Satisfactory	NILM	NILM	Negative (NILM)	
P05904	2019	2019-Q4	2019-11	Pre-pandemic	Cytology (Pap)	50-54	54	Not performed	Satisfactory	NILM	NILM	Negative (NILM)	
P05905	2019	2019-Q4	2019-11	Pre-pandemic	Cytology (Pap)	40-44	42	Not performed	Satisfactory	NILM	NILM	Negative (NILM)	
P05906	2019	2019-Q4	2019-11	Pre-pandemic	Cytology (Pap)	55-59	57	Not performed	Satisfactory	NILM	NILM	Negative (NILM)	
P05907	2019	2019-Q4	2019-11	Pre-pandemic	Cytology (Pap)	45-49	48	Not performed	Satisfactory	NILM	NILM	Negative (NILM)	
P05908	2019	2019-Q4	2019-11	Pre-pandemic	Cytology (Pap)	30-34	34	Not performed	Satisfactory	NILM	NILM	Negative (NILM)	
P05909	2019	2019-Q4	2019-11	Pre-pandemic	Cytology (Pap)	30-34	33	Not performed	Satisfactory	NILM	NILM	Negative (NILM)	
P05910	2019	2019-Q4	2019-11	Pre-pandemic	Cytology (Pap)	50-54	54	Not performed	Satisfactory	Positive	HSIL	High-grade squamous	
P05911	2019	2019-Q4	2019-11	Pre-pandemic	Cytology (Pap)	30-34	31	Not performed	Satisfactory	NILM	NILM	Negative (NILM)	
P05912	2019	2019-Q4	2019-11	Pre-pandemic	Cytology (Pap)	40-44	42	Not performed	Satisfactory	NILM	NILM	Negative (NILM)	
P05913	2019	2019-Q4	2019-11	Pre-pandemic	Cytology (Pap)	40-44	40	Not performed	Satisfactory	Positive	HSIL	High-grade squamous	
P05914	2019	2019-Q4	2019-11	Pre-pandemic	Cytology (Pap)	30-34	32	Not performed	Satisfactory	NILM	NILM	Negative (NILM)	
P05915	2019	2019-Q4	2019-11	Pre-pandemic	Cytology (Pap)	30-34	31	Not performed	Satisfactory	NILM	NILM	Negative (NILM)	
P05916	2019	2019-Q4	2019-11	Pre-pandemic	Cytology (Pap)	40-44	44	Not performed	Satisfactory	Positive	ASC-US	Low-grade / equivocal squamous	
P05917	2019	2019-Q4	2019-11	Pre-pandemic	Cytology (Pap)	40-44	44	Not performed	Satisfactory	NILM	NILM	Negative (NILM)	
P05918	2019	2019-Q4	2019-11	Pre-pandemic	Cytology (Pap)	35-39	35	Not performed	Satisfactory	NILM	NILM	Negative (NILM)	

patient_id	year	quarter	month	period	primary_test	age_group	age_years	hpv_result	adequacy	result	bethesda	grade	infection
P05919	2019	2019-Q4	2019-11	Pre-pandemic	Cytology (Pap)	45-49	45	Not performed	Satisfactory	NILM	NILM	Negative (NILM)	
P05920	2019	2019-Q4	2019-11	Pre-pandemic	Cytology (Pap)	45-49	48	Not performed	Satisfactory	NILM	NILM	Negative (NILM)	
P05921	2019	2019-Q4	2019-11	Pre-pandemic	Cytology (Pap)	25-29	26	Not performed	Satisfactory	NILM	NILM	Negative (NILM)	
P05922	2019	2019-Q4	2019-11	Pre-pandemic	Cytology (Pap)	60-64	60	Not performed	Satisfactory	NILM	NILM	Negative (NILM)	
P05923	2019	2019-Q4	2019-11	Pre-pandemic	Cytology (Pap)	60-64	61	Not performed	Satisfactory	Positive	ASC-US	Low-grade / equivocal squamous	
P05924	2019	2019-Q4	2019-11	Pre-pandemic	Cytology (Pap)	30-34	31	Not performed	Satisfactory	NILM	NILM	Negative (NILM)	
P05925	2019	2019-Q4	2019-11	Pre-pandemic	Cytology (Pap)	35-39	35	Not performed	Satisfactory	NILM	NILM	Negative (NILM)	
P05926	2019	2019-Q4	2019-11	Pre-pandemic	Cytology (Pap)	35-39	39	Not performed	Satisfactory	NILM	NILM	Negative (NILM)	
P05927	2019	2019-Q4	2019-11	Pre-pandemic	Cytology (Pap)	40-44	41	Not performed	Satisfactory	NILM	NILM	Negative (NILM)	
P05928	2019	2019-Q4	2019-11	Pre-pandemic	Cytology (Pap)	50-54	52	Not performed	Satisfactory	NILM	NILM	Negative (NILM)	
P05929	2019	2019-Q4	2019-11	Pre-pandemic	Cytology (Pap)	50-54	52	Not performed	Satisfactory	NILM	NILM	Negative (NILM)	
P05930	2019	2019-Q4	2019-11	Pre-pandemic	Cytology (Pap)	50-54	53	Not performed	Satisfactory	NILM	NILM	Negative (NILM)	
P05931	2019	2019-Q4	2019-11	Pre-pandemic	Cytology (Pap)	30-34	32	Not performed	Satisfactory	NILM	NILM	Negative (NILM)	
P05932	2019	2019-Q4	2019-11	Pre-pandemic	Cytology (Pap)	60-64	61	Not performed	Satisfactory	NILM	NILM	Negative (NILM)	
P05933	2019	2019-Q4	2019-11	Pre-pandemic	Cytology (Pap)	30-34	32	Not performed	Satisfactory	NILM	NILM	Negative (NILM)	
P05934	2019	2019-Q4	2019-11	Pre-pandemic	Cytology (Pap)	35-39	37	Not performed	Satisfactory	NILM	NILM	Negative (NILM)	
P05935	2019	2019-Q4	2019-11	Pre-pandemic	Cytology (Pap)	45-49	49	Not performed	Satisfactory	NILM	NILM	Negative (NILM)	
P05936	2019	2019-Q4	2019-11	Pre-pandemic	Cytology (Pap)	50-54	54	Not performed	Satisfactory	NILM	NILM	Negative (NILM)	
P05937	2019	2019-Q4	2019-11	Pre-pandemic	Cytology (Pap)	50-54	50	Not performed	Satisfactory	Positive	ASC-US	Low-grade / equivocal squamous	
P05938	2019	2019-Q4	2019-11	Pre-pandemic	Cytology (Pap)	55-59	56	Not performed	Satisfactory	NILM	NILM	Negative (NILM)	
P05939	2019	2019-Q4	2019-11	Pre-pandemic	Cytology (Pap)	45-49	45	Not performed	Satisfactory	NILM	NILM	Negative (NILM)	
P05940	2019	2019-Q4	2019-11	Pre-pandemic	Cytology (Pap)	40-44	44	Not performed	Satisfactory	NILM	NILM	Negative (NILM)	
P05941	2019	2019-Q4	2019-11	Pre-pandemic	Cytology (Pap)	45-49	45	Not performed	Satisfactory	NILM	NILM	Negative (NILM)	
P05942	2019	2019-Q4	2019-11	Pre-pandemic	Cytology (Pap)	30-34	34	Not performed	Satisfactory	NILM	NILM	Negative (NILM)	
P05943	2019	2019-Q4	2019-11	Pre-pandemic	Cytology (Pap)	40-44	40	Not performed	Satisfactory	Positive	LSIL	Low-grade / equivocal squamous	
P05944	2019	2019-Q4	2019-11	Pre-pandemic	Cytology (Pap)	60-64	63	Not performed	Satisfactory	NILM	NILM	Negative (NILM)	
P05945	2019	2019-Q4	2019-11	Pre-pandemic	Cytology (Pap)	60-64	61	Not performed	Satisfactory	Positive	HSIL	High-grade squamous	
P05946	2019	2019-Q4	2019-11	Pre-pandemic	Cytology (Pap)	35-39	39	Not performed	Satisfactory	NILM	NILM	Negative (NILM)	
P05947	2019	2019-Q4	2019-11	Pre-pandemic	Cytology (Pap)	40-44	43	Not performed	Satisfactory	NILM	NILM	Negative (NILM)	
P05948	2019	2019-Q4	2019-11	Pre-pandemic	Cytology (Pap)	50-54	54	Not performed	Satisfactory	NILM	NILM	Negative (NILM)	
P05949	2019	2019-Q4	2019-11	Pre-pandemic	Cytology (Pap)	55-59	58	Not performed	Satisfactory	NILM	NILM	Negative (NILM)	
P05950	2019	2019-Q4	2019-11	Pre-pandemic	Cytology (Pap)	40-44	44	Not performed	Satisfactory	Positive	ASC-US	Low-grade / equivocal squamous	
P05951	2019	2019-Q4	2019-11	Pre-pandemic	Cytology (Pap)	55-59	58	Not performed	Satisfactory	Positive	ASC-US	Low-grade / equivocal squamous	
P05952	2019	2019-Q4	2019-11	Pre-pandemic	Cytology (Pap)	35-39	37	Not performed	Satisfactory	NILM	NILM	Negative (NILM)	
P05953	2019	2019-Q4	2019-11	Pre-pandemic	Cytology (Pap)	35-39	38	Not performed	Satisfactory	NILM	NILM	Negative (NILM)	
P05954	2019	2019-Q4	2019-11	Pre-pandemic	Cytology (Pap)	45-49	48	Not performed	Satisfactory	NILM	NILM	Negative (NILM)	
P05955	2019	2019-Q4	2019-11	Pre-pandemic	Cytology (Pap)	35-39	38	Not performed	Satisfactory	NILM	NILM	Negative (NILM)	
P05956	2019	2019-Q4	2019-11	Pre-pandemic	Cytology (Pap)	45-49	48	Not performed	Satisfactory	NILM	NILM	Negative (NILM)	
P05957	2019	2019-Q4	2019-11	Pre-pandemic	Cytology (Pap)	30-34	32	Not performed	Satisfactory	NILM	NILM	Negative (NILM)	
P05958	2019	2019-Q4	2019-11	Pre-pandemic	Cytology (Pap)	30-34	34	Not performed	Satisfactory	NILM	NILM	Negative (NILM)	Trichomonas
P05959	2019	2019-Q4	2019-11	Pre-pandemic	Cytology (Pap)	45-49	48	Not performed	Satisfactory	NILM	NILM	Negative (NILM)	
P05960	2019	2019-Q4	2019-11	Pre-pandemic	Cytology (Pap)	50-54	54	Not performed	Satisfactory	NILM	NILM	Negative (NILM)	
P05961	2019	2019-Q4	2019-11	Pre-pandemic	Cytology (Pap)	55-59	59	Not performed	Satisfactory	NILM	NILM	Negative (NILM)	
P05962	2019	2019-Q4	2019-11	Pre-pandemic	Cytology (Pap)	40-44	44	Not performed	Satisfactory	NILM	NILM	Negative (NILM)	
P05963	2019	2019-Q4	2019-11	Pre-pandemic	Cytology (Pap)	50-54	51	Not performed	Satisfactory	Positive	LSIL	Low-grade / equivocal squamous	
P05964	2019	2019-Q4	2019-11	Pre-pandemic	Cytology (Pap)	45-49	48	Not performed	Satisfactory	NILM	NILM	Negative (NILM)	
P05965	2019	2019-Q4	2019-11	Pre-pandemic	Cytology (Pap)	40-44	42	Not performed	Satisfactory	Positive	HSIL	High-grade squamous	
P05966	2019	2019-Q4	2019-11	Pre-pandemic	Cytology (Pap)	55-59	57	Not performed	Satisfactory	NILM	NILM	Negative (NILM)	
P05967	2019	2019-Q4	2019-11	Pre-pandemic	Cytology (Pap)	40-44	44	Not performed	Satisfactory	NILM	NILM	Negative (NILM)	
P05968	2019	2019-Q4	2019-11	Pre-pandemic	Cytology (Pap)	50-54	54	Not performed	Satisfactory	NILM	NILM	Negative (NILM)	
P05969	2019	2019-Q4	2019-11	Pre-pandemic	Cytology (Pap)	50-54	53	Not performed	Satisfactory	NILM	NILM	Negative (NILM)	
P05970	2019	2019-Q4	2019-11	Pre-pandemic	Cytology (Pap)	45-49	48	Not performed	Satisfactory	Positive	LSIL	Low-grade / equivocal squamous	
P05971	2019	2019-Q4	2019-11	Pre-pandemic	Cytology (Pap)	40-44	41	Not performed	Satisfactory	NILM	NILM	Negative (NILM)	
P05972	2019	2019-Q4	2019-11	Pre-pandemic	Cytology (Pap)	60-64	62	Not performed	Satisfactory	NILM	NILM	Negative (NILM)	
P05973	2019	2019-Q4	2019-11	Pre-pandemic	Cytology (Pap)	40-44	41	Not performed	Satisfactory	Positive	ASC-US	Low-grade / equivocal squamous	
P05974	2019	2019-Q4	2019-11	Pre-pandemic	Cytology (Pap)	30-34	34	Not performed	Satisfactory	NILM	NILM	Negative (NILM)	
P05975	2019	2019-Q4	2019-11	Pre-pandemic	Cytology (Pap)	50-54	50	Not performed	Satisfactory	NILM	NILM	Negative (NILM)	
P05976	2019	2019-Q4	2019-11	Pre-pandemic	Cytology (Pap)	35-39	35	Not performed	Satisfactory	NILM	NILM	Negative (NILM)	
P05977	2019	2019-Q4	2019-11	Pre-pandemic	Cytology (Pap)	30-34	34	Not performed	Satisfactory	NILM	NILM	Negative (NILM)	
P05978	2019	2019-Q4	2019-11	Pre-pandemic	Cytology (Pap)	50-54	53	Not performed	Satisfactory	NILM	NILM	Negative (NILM)	
P05979	2019	2019-Q4	2019-11	Pre-pandemic	Cytology (Pap)	25-29	28	Not performed	Satisfactory	Positive	LSIL	Low-grade / equivocal squamous	
P05980	2019	2019-Q4	2019-11	Pre-pandemic	Cytology (Pap)	25-29	29	Not performed	Satisfactory	NILM	NILM	Negative (NILM)	
P05981	2019	2019-Q4	2019-11	Pre-pandemic	Cytology (Pap)	30-34	30	Not performed	Satisfactory	NILM	NILM	Negative (NILM)	
P05982	2019	2019-Q4	2019-11	Pre-pandemic	Cytology (Pap)	35-39	36	Not performed	Satisfactory	NILM	NILM	Negative (NILM)	
P05983	2019	2019-Q4	2019-11	Pre-pandemic	Cytology (Pap)	60-64	61	Not performed	Satisfactory	NILM	NILM	Negative (NILM)	
P05984	2019	2019-Q4	2019-11	Pre-pandemic	Cytology (Pap)	50-54	50	Not performed	Satisfactory	NILM	NILM	Negative (NILM)	
P05985	2019	2019-Q4	2019-11	Pre-pandemic	Cytology (Pap)	45-49	49	Not performed	Satisfactory	NILM	NILM	Negative (NILM)	
P05986	2019	2019-Q4	2019-11	Pre-pandemic	Cytology (Pap)	50-54	50	Not performed	Satisfactory	NILM	NILM	Negative (NILM)	
P05987	2019	2019-Q4	2019-11	Pre-pandemic	Cytology (Pap)	45-49	48	Not performed	Satisfactory	NILM	NILM	Negative (NILM)	
P05988	2019	2019-Q4	2019-11	Pre-pandemic	Cytology (Pap)	50-54	53	Not performed	Satisfactory	Positive	LSIL	Low-grade / equivocal squamous	
P05989	2019	2019-Q4	2019-11	Pre-pandemic	Cytology (Pap)	55-59	58	Not performed	Satisfactory	NILM	NILM	Negative (NILM)	
P05990	2019	2019-Q4	2019-11	Pre-pandemic	Cytology (Pap)	50-54	52	Not performed	Satisfactory	NILM	NILM	Negative (NILM)	
P05991	2019	2019-Q4	2019-11	Pre-pandemic	Cytology (Pap)	40-44	44	Not performed	Unsatisfactory	Unsatisfactory	Unsatisfactory	Unsatisfactory	
P05992	2019	2019-Q4	2019-11	Pre-pandemic	Cytology (Pap)	50-54	52	Not performed	Satisfactory	NILM	NILM	Negative (NILM)	
P05993	2019	2019-Q4	2019-11	Pre-pandemic	Cytology (Pap)	40-44	42	Not performed	Satisfactory	NILM	NILM	Negative (NILM)	
P05994	2019	2019-Q4	2019-11	Pre-pandemic	Cytology (Pap)	40-44	40	Not performed	Satisfactory	Positive	LSIL	Low-grade / equivocal squamous	
P05995	2019	2019-Q4	2019-11	Pre-pandemic	Cytology (Pap)	30-34	33	Not performed	Satisfactory	NILM	NILM	Negative (NILM)	
P05996	2019	2019-Q4	2019-11	Pre-pandemic	Cytology (Pap)	50-54	54	Not performed	Satisfactory	NILM	NILM	Negative (NILM)	
P05997	2019	2019-Q4	2019-11	Pre-pandemic	Cytology (Pap)	50-54	52	Not performed	Satisfactory	NILM	NILM	Negative (NILM)	
P05998	2019	2019-Q4	2019-11	Pre-pandemic	Cytology (Pap)	30-34	31	Not performed	Satisfactory	NILM	NILM	Negative (NILM)	

patient_id	year	quarter	month	period	primary_test	age_group	age_years	hpv_result	adequacy	result	bethesda	grade	infection
P05999	2019	2019-Q4	2019-11	Pre-pandemic	Cytology (Pap)	40-44	40	Not performed	Satisfactory	NILM	NILM	Negative (NILM)	
P06000	2019	2019-Q4	2019-11	Pre-pandemic	Cytology (Pap)	40-44	44	Not performed	Satisfactory	NILM	NILM	Negative (NILM)	
P06001	2019	2019-Q4	2019-11	Pre-pandemic	Cytology (Pap)	35-39	36	Not performed	Satisfactory	NILM	NILM	Negative (NILM)	
P06002	2019	2019-Q4	2019-11	Pre-pandemic	Cytology (Pap)	30-34	30	Not performed	Satisfactory	NILM	NILM	Negative (NILM)	
P06003	2019	2019-Q4	2019-11	Pre-pandemic	Cytology (Pap)	45-49	48	Not performed	Satisfactory	NILM	NILM	Negative (NILM)	
P06004	2019	2019-Q4	2019-11	Pre-pandemic	Cytology (Pap)	60-64	62	Not performed	Satisfactory	NILM	NILM	Negative (NILM)	
P06005	2019	2019-Q4	2019-11	Pre-pandemic	Cytology (Pap)	50-54	51	Not performed	Satisfactory	NILM	NILM	Negative (NILM)	
P06006	2019	2019-Q4	2019-11	Pre-pandemic	Cytology (Pap)	25-29	29	Not performed	Satisfactory	Positive	ASC-US	Low-grade / equivocal squamous	
P06007	2019	2019-Q4	2019-11	Pre-pandemic	Cytology (Pap)	40-44	42	Not performed	Satisfactory	Positive	ASC-US	Low-grade / equivocal squamous	
P06008	2019	2019-Q4	2019-11	Pre-pandemic	Cytology (Pap)	50-54	53	Not performed	Satisfactory	Positive	ASC-US	Low-grade / equivocal squamous	
P06009	2019	2019-Q4	2019-11	Pre-pandemic	Cytology (Pap)	40-44	41	Not performed	Satisfactory	NILM	NILM	Negative (NILM)	
P06010	2019	2019-Q4	2019-11	Pre-pandemic	Cytology (Pap)	35-39	38	Not performed	Satisfactory	NILM	NILM	Negative (NILM)	
P06011	2019	2019-Q4	2019-11	Pre-pandemic	Cytology (Pap)	60-64	60	Not performed	Satisfactory	Positive	LSIL	Low-grade / equivocal squamous	
P06012	2019	2019-Q4	2019-11	Pre-pandemic	Cytology (Pap)	50-54	51	Not performed	Satisfactory	NILM	NILM	Negative (NILM)	
P06013	2019	2019-Q4	2019-11	Pre-pandemic	Cytology (Pap)	45-49	48	Not performed	Satisfactory	NILM	NILM	Negative (NILM)	
P06014	2019	2019-Q4	2019-11	Pre-pandemic	Cytology (Pap)	40-44	43	Not performed	Satisfactory	Positive	LSIL	Low-grade / equivocal squamous	
P06015	2019	2019-Q4	2019-11	Pre-pandemic	Cytology (Pap)	55-59	58	Not performed	Satisfactory	Positive	HSIL	High-grade squamous	
P06016	2019	2019-Q4	2019-11	Pre-pandemic	Cytology (Pap)	35-39	38	Not performed	Satisfactory	NILM	NILM	Negative (NILM)	
P06017	2019	2019-Q4	2019-11	Pre-pandemic	Cytology (Pap)	60-64	62	Not performed	Satisfactory	Positive	ASC-H	High-grade squamous	
P06018	2019	2019-Q4	2019-11	Pre-pandemic	Cytology (Pap)	25-29	26	Not performed	Satisfactory	NILM	NILM	Negative (NILM)	
P06019	2019	2019-Q4	2019-11	Pre-pandemic	Cytology (Pap)	40-44	43	Not performed	Satisfactory	NILM	NILM	Negative (NILM)	
P06020	2019	2019-Q4	2019-11	Pre-pandemic	Cytology (Pap)	55-59	55	Not performed	Satisfactory	NILM	NILM	Negative (NILM)	
P06021	2019	2019-Q4	2019-11	Pre-pandemic	Cytology (Pap)	40-44	40	Not performed	Satisfactory	NILM	NILM	Negative (NILM)	
P06022	2019	2019-Q4	2019-11	Pre-pandemic	Cytology (Pap)	40-44	44	Not performed	Satisfactory	NILM	NILM	Negative (NILM)	
P06023	2019	2019-Q4	2019-11	Pre-pandemic	Cytology (Pap)	35-39	39	Not performed	Satisfactory	NILM	NILM	Negative (NILM)	
P06024	2019	2019-Q4	2019-11	Pre-pandemic	Cytology (Pap)	60-64	64	Not performed	Satisfactory	NILM	NILM	Negative (NILM)	
P06025	2019	2019-Q4	2019-11	Pre-pandemic	Cytology (Pap)	35-39	35	Not performed	Satisfactory	NILM	NILM	Negative (NILM)	
P06026	2019	2019-Q4	2019-11	Pre-pandemic	Cytology (Pap)	30-34	33	Not performed	Satisfactory	NILM	NILM	Negative (NILM)	
P06027	2019	2019-Q4	2019-11	Pre-pandemic	Cytology (Pap)	35-39	39	Not performed	Satisfactory	NILM	NILM	Negative (NILM)	
P06028	2019	2019-Q4	2019-11	Pre-pandemic	Cytology (Pap)	45-49	47	Not performed	Satisfactory	Positive	ASC-US	Low-grade / equivocal squamous	
P06029	2019	2019-Q4	2019-11	Pre-pandemic	Cytology (Pap)	35-39	39	Not performed	Satisfactory	Positive	ASC-H	High-grade squamous	
P06030	2019	2019-Q4	2019-11	Pre-pandemic	Cytology (Pap)	60-64	64	Not performed	Satisfactory	NILM	NILM	Negative (NILM)	
P06031	2019	2019-Q4	2019-11	Pre-pandemic	Cytology (Pap)	40-44	40	Not performed	Satisfactory	NILM	NILM	Negative (NILM)	
P06032	2019	2019-Q4	2019-11	Pre-pandemic	Cytology (Pap)	30-34	32	Not performed	Satisfactory	NILM	NILM	Negative (NILM)	
P06033	2019	2019-Q4	2019-11	Pre-pandemic	Cytology (Pap)	50-54	51	Not performed	Satisfactory	NILM	NILM	Negative (NILM)	
P06034	2019	2019-Q4	2019-11	Pre-pandemic	Cytology (Pap)	40-44	42	Not performed	Satisfactory	NILM	NILM	Negative (NILM)	
P06035	2019	2019-Q4	2019-11	Pre-pandemic	Cytology (Pap)	55-59	58	Not performed	Satisfactory	NILM	NILM	Negative (NILM)	
P06036	2019	2019-Q4	2019-11	Pre-pandemic	Cytology (Pap)	40-44	40	Not performed	Satisfactory	NILM	NILM	Negative (NILM)	
P06037	2019	2019-Q4	2019-11	Pre-pandemic	Cytology (Pap)	45-49	49	Not performed	Satisfactory	NILM	NILM	Negative (NILM)	
P06038	2019	2019-Q4	2019-11	Pre-pandemic	Cytology (Pap)	35-39	38	Not performed	Satisfactory	NILM	NILM	Negative (NILM)	
P06039	2019	2019-Q4	2019-11	Pre-pandemic	Cytology (Pap)	55-59	55	Not performed	Satisfactory	NILM	NILM	Negative (NILM)	
P06040	2019	2019-Q4	2019-11	Pre-pandemic	Cytology (Pap)	40-44	40	Not performed	Satisfactory	NILM	NILM	Negative (NILM)	
P06041	2019	2019-Q4	2019-11	Pre-pandemic	Cytology (Pap)	40-44	41	Not performed	Unsatisfactory	Unsatisfactory	Unsatisfactory	Unsatisfactory	Candida
P06042	2019	2019-Q4	2019-11	Pre-pandemic	Cytology (Pap)	50-54	52	Not performed	Satisfactory	Positive	LSIL	Low-grade / equivocal squamous	
P06043	2019	2019-Q4	2019-11	Pre-pandemic	Cytology (Pap)	40-44	41	Not performed	Satisfactory	NILM	NILM	Negative (NILM)	
P06044	2019	2019-Q4	2019-11	Pre-pandemic	Cytology (Pap)	40-44	44	Not performed	Satisfactory	NILM	NILM	Negative (NILM)	
P06045	2019	2019-Q4	2019-11	Pre-pandemic	Cytology (Pap)	45-49	45	Not performed	Satisfactory	NILM	NILM	Negative (NILM)	
P06046	2019	2019-Q4	2019-11	Pre-pandemic	Cytology (Pap)	55-59	57	Not performed	Satisfactory	NILM	NILM	Negative (NILM)	
P06047	2019	2019-Q4	2019-11	Pre-pandemic	Cytology (Pap)	30-34	33	Not performed	Satisfactory	NILM	NILM	Negative (NILM)	
P06048	2019	2019-Q4	2019-11	Pre-pandemic	Cytology (Pap)	40-44	40	Not performed	Satisfactory	NILM	NILM	Negative (NILM)	
P06049	2019	2019-Q4	2019-11	Pre-pandemic	Cytology (Pap)	40-44	44	Not performed	Satisfactory	Positive	LSIL	Low-grade / equivocal squamous	
P06050	2019	2019-Q4	2019-11	Pre-pandemic	Cytology (Pap)	40-44	44	Not performed	Satisfactory	NILM	NILM	Negative (NILM)	
P06051	2019	2019-Q4	2019-11	Pre-pandemic	Cytology (Pap)	30-34	32	Not performed	Satisfactory	NILM	NILM	Negative (NILM)	
P06052	2019	2019-Q4	2019-11	Pre-pandemic	Cytology (Pap)	40-44	42	Not performed	Satisfactory	NILM	NILM	Negative (NILM)	
P06053	2019	2019-Q4	2019-11	Pre-pandemic	Cytology (Pap)	45-49	48	Not performed	Satisfactory	NILM	NILM	Negative (NILM)	
P06054	2019	2019-Q4	2019-11	Pre-pandemic	Cytology (Pap)	35-39	39	Not performed	Satisfactory	NILM	NILM	Negative (NILM)	
P06055	2019	2019-Q4	2019-11	Pre-pandemic	Cytology (Pap)	55-59	59	Not performed	Satisfactory	NILM	NILM	Negative (NILM)	
P06056	2019	2019-Q4	2019-11	Pre-pandemic	Cytology (Pap)	45-49	49	Not performed	Satisfactory	NILM	NILM	Negative (NILM)	
P06057	2019	2019-Q4	2019-11	Pre-pandemic	Cytology (Pap)	55-59	58	Not performed	Satisfactory	NILM	NILM	Negative (NILM)	
P06058	2019	2019-Q4	2019-11	Pre-pandemic	Cytology (Pap)	30-34	34	Not performed	Satisfactory	Positive	HSIL	High-grade squamous	
P06059	2019	2019-Q4	2019-11	Pre-pandemic	Cytology (Pap)	50-54	53	Not performed	Satisfactory	NILM	NILM	Negative (NILM)	
P06060	2019	2019-Q4	2019-11	Pre-pandemic	Cytology (Pap)	40-44	43	Not performed	Satisfactory	NILM	NILM	Negative (NILM)	
P06061	2019	2019-Q4	2019-11	Pre-pandemic	Cytology (Pap)	55-59	59	Not performed	Satisfactory	NILM	NILM	Negative (NILM)	
P06062	2019	2019-Q4	2019-11	Pre-pandemic	Cytology (Pap)	30-34	33	Not performed	Satisfactory	NILM	NILM	Negative (NILM)	
P06063	2019	2019-Q4	2019-11	Pre-pandemic	Cytology (Pap)	55-59	55	Not performed	Satisfactory	NILM	NILM	Negative (NILM)	
P06064	2019	2019-Q4	2019-11	Pre-pandemic	Cytology (Pap)	40-44	43	Not performed	Satisfactory	NILM	NILM	Negative (NILM)	
P06065	2019	2019-Q4	2019-11	Pre-pandemic	Cytology (Pap)	40-44	43	Not performed	Satisfactory	NILM	NILM	Negative (NILM)	
P06066	2019	2019-Q4	2019-11	Pre-pandemic	Cytology (Pap)	45-49	46	Not performed	Satisfactory	NILM	NILM	Negative (NILM)	
P06067	2019	2019-Q4	2019-11	Pre-pandemic	Cytology (Pap)	30-34	34	Not performed	Satisfactory	NILM	NILM	Negative (NILM)	
P06068	2019	2019-Q4	2019-11	Pre-pandemic	Cytology (Pap)	40-44	40	Not performed	Satisfactory	NILM	NILM	Negative (NILM)	
P06069	2019	2019-Q4	2019-11	Pre-pandemic	Cytology (Pap)	45-49	46	Not performed	Satisfactory	NILM	NILM	Negative (NILM)	
P06070	2019	2019-Q4	2019-11	Pre-pandemic	Cytology (Pap)	50-54	51	Not performed	Satisfactory	NILM	NILM	Negative (NILM)	
P06071	2019	2019-Q4	2019-11	Pre-pandemic	Cytology (Pap)	40-44	44	Not performed	Satisfactory	NILM	NILM	Negative (NILM)	
P06072	2019	2019-Q4	2019-11	Pre-pandemic	Cytology (Pap)	55-59	56	Not performed	Satisfactory	Positive	HSIL	High-grade squamous	
P06073	2019	2019-Q4	2019-11	Pre-pandemic	Cytology (Pap)	35-39	38	Not performed	Satisfactory	NILM	NILM	Negative (NILM)	
P06074	2019	2019-Q4	2019-11	Pre-pandemic	Cytology (Pap)	45-49	49	Not performed	Satisfactory	NILM	NILM	Negative (NILM)	Candida
P06075	2019	2019-Q4	2019-11	Pre-pandemic	Cytology (Pap)	55-59	57	Not performed	Satisfactory	NILM	NILM	Negative (NILM)	
P06076	2019	2019-Q4	2019-11	Pre-pandemic	Cytology (Pap)	30-34	33	Not performed	Satisfactory	NILM	NILM	Negative (NILM)	
P06077	2019	2019-Q4	2019-11	Pre-pandemic	Cytology (Pap)	30-34	31	Not performed	Satisfactory	NILM	NILM	Negative (NILM)	
P06078	2019	2019-Q4	2019-11	Pre-pandemic	Cytology (Pap)	45-49	48	Not performed	Satisfactory	NILM	NILM	Negative (NILM)	

patient_id	year	quarter	month	period	primary_test	age_group	age_years	hpv_result	adequacy	result	bethesda	grade	infection
P06079	2019	2019-Q4	2019-11	Pre-pandemic	Cytology (Pap)	30-34	30	Not performed	Satisfactory	NILM	NILM	Negative (NILM)	
P06080	2019	2019-Q4	2019-11	Pre-pandemic	Cytology (Pap)	45-49	47	Not performed	Satisfactory	NILM	NILM	Negative (NILM)	
P06081	2019	2019-Q4	2019-11	Pre-pandemic	Cytology (Pap)	40-44	43	Not performed	Satisfactory	NILM	NILM	Negative (NILM)	
P06082	2019	2019-Q4	2019-11	Pre-pandemic	Cytology (Pap)	40-44	40	Not performed	Satisfactory	Positive	HSIL	High-grade squamous	
P06083	2019	2019-Q4	2019-11	Pre-pandemic	Cytology (Pap)	55-59	57	Not performed	Satisfactory	NILM	NILM	Negative (NILM)	
P06084	2019	2019-Q4	2019-11	Pre-pandemic	Cytology (Pap)	40-44	40	Not performed	Satisfactory	NILM	NILM	Negative (NILM)	
P06085	2019	2019-Q4	2019-11	Pre-pandemic	Cytology (Pap)	55-59	58	Not performed	Satisfactory	NILM	NILM	Negative (NILM)	
P06086	2019	2019-Q4	2019-11	Pre-pandemic	Cytology (Pap)	55-59	58	Not performed	Satisfactory	NILM	NILM	Negative (NILM)	
P06087	2019	2019-Q4	2019-11	Pre-pandemic	Cytology (Pap)	40-44	42	Not performed	Satisfactory	NILM	NILM	Negative (NILM)	
P06088	2019	2019-Q4	2019-11	Pre-pandemic	Cytology (Pap)	50-54	53	Not performed	Satisfactory	NILM	NILM	Negative (NILM)	
P06089	2019	2019-Q4	2019-11	Pre-pandemic	Cytology (Pap)	45-49	47	Not performed	Satisfactory	NILM	NILM	Negative (NILM)	
P06090	2019	2019-Q4	2019-11	Pre-pandemic	Cytology (Pap)	35-39	36	Not performed	Satisfactory	NILM	NILM	Negative (NILM)	
P06091	2019	2019-Q4	2019-11	Pre-pandemic	Cytology (Pap)	30-34	31	Not performed	Satisfactory	NILM	NILM	Negative (NILM)	
P06092	2019	2019-Q4	2019-11	Pre-pandemic	Cytology (Pap)	55-59	58	Not performed	Satisfactory	NILM	NILM	Negative (NILM)	
P06093	2019	2019-Q4	2019-11	Pre-pandemic	Cytology (Pap)	60-64	63	Not performed	Satisfactory	NILM	NILM	Negative (NILM)	
P06094	2019	2019-Q4	2019-11	Pre-pandemic	Cytology (Pap)	60-64	63	Not performed	Satisfactory	NILM	NILM	Negative (NILM)	
P06095	2019	2019-Q4	2019-11	Pre-pandemic	Cytology (Pap)	40-44	44	Not performed	Satisfactory	NILM	NILM	Negative (NILM)	
P06096	2019	2019-Q4	2019-11	Pre-pandemic	Cytology (Pap)	35-39	37	Not performed	Satisfactory	NILM	NILM	Negative (NILM)	
P06097	2019	2019-Q4	2019-11	Pre-pandemic	Cytology (Pap)	45-49	46	Not performed	Satisfactory	NILM	NILM	Negative (NILM)	
P06098	2019	2019-Q4	2019-11	Pre-pandemic	Cytology (Pap)	60-64	62	Not performed	Satisfactory	NILM	NILM	Negative (NILM)	
P06099	2019	2019-Q4	2019-11	Pre-pandemic	Cytology (Pap)	50-54	51	Not performed	Satisfactory	NILM	NILM	Negative (NILM)	
P06100	2019	2019-Q4	2019-11	Pre-pandemic	Cytology (Pap)	45-49	47	Not performed	Satisfactory	NILM	NILM	Negative (NILM)	
P06101	2019	2019-Q4	2019-11	Pre-pandemic	Cytology (Pap)	35-39	37	Not performed	Satisfactory	NILM	NILM	Negative (NILM)	
P06102	2019	2019-Q4	2019-12	Pre-pandemic	Cytology (Pap)	35-39	35	Not performed	Satisfactory	NILM	NILM	Negative (NILM)	
P06103	2019	2019-Q4	2019-12	Pre-pandemic	Cytology (Pap)	25-29	27	Not performed	Satisfactory	NILM	NILM	Negative (NILM)	
P06104	2019	2019-Q4	2019-12	Pre-pandemic	Cytology (Pap)	30-34	32	Not performed	Satisfactory	NILM	NILM	Negative (NILM)	
P06105	2019	2019-Q4	2019-12	Pre-pandemic	Cytology (Pap)	45-49	47	Not performed	Satisfactory	NILM	NILM	Negative (NILM)	
P06106	2019	2019-Q4	2019-12	Pre-pandemic	Cytology (Pap)	40-44	42	Not performed	Satisfactory	Positive	ASC-US	Low-grade / equivocal squamous	
P06107	2019	2019-Q4	2019-12	Pre-pandemic	Cytology (Pap)	55-59	58	Not performed	Satisfactory	NILM	NILM	Negative (NILM)	
P06108	2019	2019-Q4	2019-12	Pre-pandemic	Cytology (Pap)	60-64	62	Not performed	Satisfactory	NILM	NILM	Negative (NILM)	
P06109	2019	2019-Q4	2019-12	Pre-pandemic	Cytology (Pap)	55-59	56	Not performed	Satisfactory	NILM	NILM	Negative (NILM)	
P06110	2019	2019-Q4	2019-12	Pre-pandemic	Cytology (Pap)	40-44	41	Not performed	Satisfactory	NILM	NILM	Negative (NILM)	
P06111	2019	2019-Q4	2019-12	Pre-pandemic	Cytology (Pap)	50-54	54	Not performed	Satisfactory	NILM	NILM	Negative (NILM)	Gardnerella
P06112	2019	2019-Q4	2019-12	Pre-pandemic	Cytology (Pap)	50-54	53	Not performed	Satisfactory	NILM	NILM	Negative (NILM)	
P06113	2019	2019-Q4	2019-12	Pre-pandemic	Cytology (Pap)	35-39	35	Not performed	Satisfactory	NILM	NILM	Negative (NILM)	
P06114	2019	2019-Q4	2019-12	Pre-pandemic	Cytology (Pap)	35-39	38	Not performed	Satisfactory	NILM	NILM	Negative (NILM)	
P06115	2019	2019-Q4	2019-12	Pre-pandemic	Cytology (Pap)	60-64	62	Not performed	Satisfactory	NILM	NILM	Negative (NILM)	
P06116	2019	2019-Q4	2019-12	Pre-pandemic	Cytology (Pap)	30-34	34	Not performed	Satisfactory	NILM	NILM	Negative (NILM)	
P06117	2019	2019-Q4	2019-12	Pre-pandemic	Cytology (Pap)	25-29	25	Not performed	Satisfactory	NILM	NILM	Negative (NILM)	
P06118	2019	2019-Q4	2019-12	Pre-pandemic	Cytology (Pap)	35-39	37	Not performed	Satisfactory	NILM	NILM	Negative (NILM)	
P06119	2019	2019-Q4	2019-12	Pre-pandemic	Cytology (Pap)	35-39	35	Not performed	Satisfactory	NILM	NILM	Negative (NILM)	
P06120	2019	2019-Q4	2019-12	Pre-pandemic	Cytology (Pap)	35-39	36	Not performed	Satisfactory	NILM	NILM	Negative (NILM)	
P06121	2019	2019-Q4	2019-12	Pre-pandemic	Cytology (Pap)	50-54	51	Not performed	Satisfactory	NILM	NILM	Negative (NILM)	
P06122	2019	2019-Q4	2019-12	Pre-pandemic	Cytology (Pap)	60-64	63	Not performed	Satisfactory	NILM	NILM	Negative (NILM)	
P06123	2019	2019-Q4	2019-12	Pre-pandemic	Cytology (Pap)	50-54	51	Not performed	Satisfactory	NILM	NILM	Negative (NILM)	
P06124	2019	2019-Q4	2019-12	Pre-pandemic	Cytology (Pap)	50-54	53	Not performed	Satisfactory	NILM	NILM	Negative (NILM)	
P06125	2019	2019-Q4	2019-12	Pre-pandemic	Cytology (Pap)	35-39	35	Not performed	Satisfactory	NILM	NILM	Negative (NILM)	Trichomonas
P06126	2019	2019-Q4	2019-12	Pre-pandemic	Cytology (Pap)	50-54	50	Not performed	Satisfactory	NILM	NILM	Negative (NILM)	
P06127	2019	2019-Q4	2019-12	Pre-pandemic	Cytology (Pap)	45-49	49	Not performed	Satisfactory	NILM	NILM	Negative (NILM)	
P06128	2019	2019-Q4	2019-12	Pre-pandemic	Cytology (Pap)	55-59	58	Not performed	Satisfactory	NILM	NILM	Negative (NILM)	
P06129	2019	2019-Q4	2019-12	Pre-pandemic	Cytology (Pap)	40-44	42	Not performed	Satisfactory	Positive	ASC-US	Low-grade / equivocal squamous	
P06130	2019	2019-Q4	2019-12	Pre-pandemic	Cytology (Pap)	45-49	47	Not performed	Satisfactory	Positive	HSIL	High-grade squamous	
P06131	2019	2019-Q4	2019-12	Pre-pandemic	Cytology (Pap)	40-44	43	Not performed	Satisfactory	NILM	NILM	Negative (NILM)	
P06132	2019	2019-Q4	2019-12	Pre-pandemic	Cytology (Pap)	40-44	44	Not performed	Satisfactory	NILM	NILM	Negative (NILM)	
P06133	2019	2019-Q4	2019-12	Pre-pandemic	Cytology (Pap)	30-34	32	Not performed	Satisfactory	NILM	NILM	Negative (NILM)	
P06134	2019	2019-Q4	2019-12	Pre-pandemic	Cytology (Pap)	40-44	40	Not performed	Satisfactory	NILM	NILM	Negative (NILM)	
P06135	2019	2019-Q4	2019-12	Pre-pandemic	Cytology (Pap)	50-54	53	Not performed	Satisfactory	NILM	NILM	Negative (NILM)	
P06136	2019	2019-Q4	2019-12	Pre-pandemic	Cytology (Pap)	60-64	62	Not performed	Satisfactory	NILM	NILM	Negative (NILM)	
P06137	2019	2019-Q4	2019-12	Pre-pandemic	Cytology (Pap)	25-29	26	Not performed	Satisfactory	NILM	NILM	Negative (NILM)	
P06138	2019	2019-Q4	2019-12	Pre-pandemic	Cytology (Pap)	50-54	50	Not performed	Satisfactory	NILM	NILM	Negative (NILM)	
P06139	2019	2019-Q4	2019-12	Pre-pandemic	Cytology (Pap)	40-44	42	Not performed	Satisfactory	NILM	NILM	Negative (NILM)	
P06140	2019	2019-Q4	2019-12	Pre-pandemic	Cytology (Pap)	40-44	44	Not performed	Satisfactory	NILM	NILM	Negative (NILM)	
P06141	2019	2019-Q4	2019-12	Pre-pandemic	Cytology (Pap)	40-44	44	Not performed	Satisfactory	NILM	NILM	Negative (NILM)	
P06142	2019	2019-Q4	2019-12	Pre-pandemic	Cytology (Pap)	55-59	57	Not performed	Satisfactory	NILM	NILM	Negative (NILM)	
P06143	2019	2019-Q4	2019-12	Pre-pandemic	Cytology (Pap)	40-44	43	Not performed	Satisfactory	NILM	NILM	Negative (NILM)	
P06144	2019	2019-Q4	2019-12	Pre-pandemic	Cytology (Pap)	30-34	33	Not performed	Satisfactory	NILM	NILM	Negative (NILM)	
P06145	2019	2019-Q4	2019-12	Pre-pandemic	Cytology (Pap)	45-49	46	Not performed	Satisfactory	NILM	NILM	Negative (NILM)	
P06146	2019	2019-Q4	2019-12	Pre-pandemic	Cytology (Pap)	40-44	42	Not performed	Satisfactory	NILM	NILM	Negative (NILM)	
P06147	2019	2019-Q4	2019-12	Pre-pandemic	Cytology (Pap)	30-34	31	Not performed	Satisfactory	NILM	NILM	Negative (NILM)	
P06148	2019	2019-Q4	2019-12	Pre-pandemic	Cytology (Pap)	40-44	41	Not performed	Satisfactory	NILM	NILM	Negative (NILM)	
P06149	2019	2019-Q4	2019-12	Pre-pandemic	Cytology (Pap)	35-39	35	Not performed	Satisfactory	Positive	ASC-US	Low-grade / equivocal squamous	
P06150	2019	2019-Q4	2019-12	Pre-pandemic	Cytology (Pap)	45-49	48	Not performed	Satisfactory	Positive	ASC-H	High-grade squamous	
P06151	2019	2019-Q4	2019-12	Pre-pandemic	Cytology (Pap)	50-54	51	Not performed	Satisfactory	Positive	ASC-H	High-grade squamous	
P06152	2019	2019-Q4	2019-12	Pre-pandemic	Cytology (Pap)	35-39	39	Not performed	Satisfactory	NILM	NILM	Negative (NILM)	
P06153	2019	2019-Q4	2019-12	Pre-pandemic	Cytology (Pap)	45-49	46	Not performed	Satisfactory	NILM	NILM	Negative (NILM)	
P06154	2019	2019-Q4	2019-12	Pre-pandemic	Cytology (Pap)	40-44	42	Not performed	Satisfactory	Positive	LSIL	Low-grade / equivocal squamous	
P06155	2019	2019-Q4	2019-12	Pre-pandemic	Cytology (Pap)	55-59	59	Not performed	Satisfactory	NILM	NILM	Negative (NILM)	
P06156	2019	2019-Q4	2019-12	Pre-pandemic	Cytology (Pap)	35-39	39	Not performed	Satisfactory	NILM	NILM	Negative (NILM)	
P06157	2019	2019-Q4	2019-12	Pre-pandemic	Cytology (Pap)	45-49	45	Not performed	Satisfactory	NILM	NILM	Negative (NILM)	
P06158	2019	2019-Q4	2019-12	Pre-pandemic	Cytology (Pap)	30-34	34	Not performed	Satisfactory	NILM	NILM	Negative (NILM)	

patient_id	year	quarter	month	period	primary_test	age_group	age_years	hpv_result	adequacy	result	bethesda	grade	infection
P06159	2019	2019-Q4	2019-12	Pre-pandemic	Cytology (Pap)	25-29	25	Not performed	Satisfactory	NILM	NILM	Negative (NILM)	
P06160	2019	2019-Q4	2019-12	Pre-pandemic	Cytology (Pap)	50-54	53	Not performed	Satisfactory	NILM	NILM	Negative (NILM)	Candida
P06161	2019	2019-Q4	2019-12	Pre-pandemic	Cytology (Pap)	40-44	41	Not performed	Satisfactory	NILM	NILM	Negative (NILM)	
P06162	2019	2019-Q4	2019-12	Pre-pandemic	Cytology (Pap)	45-49	49	Not performed	Satisfactory	NILM	NILM	Negative (NILM)	
P06163	2019	2019-Q4	2019-12	Pre-pandemic	Cytology (Pap)	40-44	43	Not performed	Satisfactory	NILM	NILM	Negative (NILM)	
P06164	2019	2019-Q4	2019-12	Pre-pandemic	Cytology (Pap)	55-59	55	Not performed	Satisfactory	Positive	LSIL	Low-grade / equivocal squamous	
P06165	2019	2019-Q4	2019-12	Pre-pandemic	Cytology (Pap)	40-44	43	Not performed	Satisfactory	NILM	NILM	Negative (NILM)	
P06166	2019	2019-Q4	2019-12	Pre-pandemic	Cytology (Pap)	25-29	29	Not performed	Satisfactory	NILM	NILM	Negative (NILM)	
P06167	2019	2019-Q4	2019-12	Pre-pandemic	Cytology (Pap)	45-49	46	Not performed	Satisfactory	Positive	ASC-US	Low-grade / equivocal squamous	
P06168	2019	2019-Q4	2019-12	Pre-pandemic	Cytology (Pap)	35-39	39	Not performed	Satisfactory	NILM	NILM	Negative (NILM)	
P06169	2019	2019-Q4	2019-12	Pre-pandemic	Cytology (Pap)	35-39	37	Not performed	Unsatisfactory	Unsatisfactory	Unsatisfactory	Unsatisfactory	
P06170	2019	2019-Q4	2019-12	Pre-pandemic	Cytology (Pap)	45-49	49	Not performed	Satisfactory	NILM	NILM	Negative (NILM)	
P06171	2019	2019-Q4	2019-12	Pre-pandemic	Cytology (Pap)	40-44	42	Not performed	Satisfactory	NILM	NILM	Negative (NILM)	
P06172	2019	2019-Q4	2019-12	Pre-pandemic	Cytology (Pap)	35-39	39	Not performed	Satisfactory	NILM	NILM	Negative (NILM)	
P06173	2019	2019-Q4	2019-12	Pre-pandemic	Cytology (Pap)	30-34	31	Not performed	Satisfactory	Positive	LSIL	Low-grade / equivocal squamous	
P06174	2019	2019-Q4	2019-12	Pre-pandemic	Cytology (Pap)	40-44	43	Not performed	Satisfactory	NILM	NILM	Negative (NILM)	
P06175	2019	2019-Q4	2019-12	Pre-pandemic	Cytology (Pap)	55-59	57	Not performed	Satisfactory	NILM	NILM	Negative (NILM)	
P06176	2019	2019-Q4	2019-12	Pre-pandemic	Cytology (Pap)	45-49	46	Not performed	Satisfactory	NILM	NILM	Negative (NILM)	
P06177	2019	2019-Q4	2019-12	Pre-pandemic	Cytology (Pap)	45-49	45	Not performed	Satisfactory	NILM	NILM	Negative (NILM)	
P06178	2019	2019-Q4	2019-12	Pre-pandemic	Cytology (Pap)	30-34	32	Not performed	Satisfactory	NILM	NILM	Negative (NILM)	
P06179	2019	2019-Q4	2019-12	Pre-pandemic	Cytology (Pap)	45-49	45	Not performed	Satisfactory	Positive	AGC	Glandular	
P06180	2019	2019-Q4	2019-12	Pre-pandemic	Cytology (Pap)	30-34	34	Not performed	Satisfactory	Positive	ASC-US	Low-grade / equivocal squamous	
P06181	2019	2019-Q4	2019-12	Pre-pandemic	Cytology (Pap)	55-59	55	Not performed	Satisfactory	NILM	NILM	Negative (NILM)	
P06182	2019	2019-Q4	2019-12	Pre-pandemic	Cytology (Pap)	50-54	52	Not performed	Satisfactory	NILM	NILM	Negative (NILM)	
P06183	2019	2019-Q4	2019-12	Pre-pandemic	Cytology (Pap)	45-49	49	Not performed	Unsatisfactory	Unsatisfactory	Unsatisfactory	Unsatisfactory	
P06184	2019	2019-Q4	2019-12	Pre-pandemic	Cytology (Pap)	30-34	32	Not performed	Satisfactory	NILM	NILM	Negative (NILM)	
P06185	2019	2019-Q4	2019-12	Pre-pandemic	Cytology (Pap)	40-44	42	Not performed	Satisfactory	NILM	NILM	Negative (NILM)	
P06186	2019	2019-Q4	2019-12	Pre-pandemic	Cytology (Pap)	30-34	32	Not performed	Satisfactory	NILM	NILM	Negative (NILM)	
P06187	2019	2019-Q4	2019-12	Pre-pandemic	Cytology (Pap)	50-54	54	Not performed	Satisfactory	NILM	NILM	Negative (NILM)	
P06188	2019	2019-Q4	2019-12	Pre-pandemic	Cytology (Pap)	40-44	42	Not performed	Satisfactory	NILM	NILM	Negative (NILM)	
P06189	2019	2019-Q4	2019-12	Pre-pandemic	Cytology (Pap)	60-64	62	Not performed	Satisfactory	NILM	NILM	Negative (NILM)	
P06190	2019	2019-Q4	2019-12	Pre-pandemic	Cytology (Pap)	25-29	29	Not performed	Satisfactory	NILM	NILM	Negative (NILM)	
P06191	2019	2019-Q4	2019-12	Pre-pandemic	Cytology (Pap)	30-34	31	Not performed	Satisfactory	NILM	NILM	Negative (NILM)	
P06192	2019	2019-Q4	2019-12	Pre-pandemic	Cytology (Pap)	55-59	57	Not performed	Satisfactory	NILM	NILM	Negative (NILM)	
P06193	2019	2019-Q4	2019-12	Pre-pandemic	Cytology (Pap)	30-34	30	Not performed	Satisfactory	NILM	NILM	Negative (NILM)	
P06194	2019	2019-Q4	2019-12	Pre-pandemic	Cytology (Pap)	60-64	61	Not performed	Satisfactory	NILM	NILM	Negative (NILM)	
P06195	2019	2019-Q4	2019-12	Pre-pandemic	Cytology (Pap)	60-64	60	Not performed	Satisfactory	NILM	NILM	Negative (NILM)	
P06196	2019	2019-Q4	2019-12	Pre-pandemic	Cytology (Pap)	45-49	47	Not performed	Satisfactory	NILM	NILM	Negative (NILM)	
P06197	2019	2019-Q4	2019-12	Pre-pandemic	Cytology (Pap)	45-49	46	Not performed	Satisfactory	NILM	NILM	Negative (NILM)	
P06198	2019	2019-Q4	2019-12	Pre-pandemic	Cytology (Pap)	25-29	29	Not performed	Satisfactory	NILM	NILM	Negative (NILM)	
P06199	2019	2019-Q4	2019-12	Pre-pandemic	Cytology (Pap)	45-49	45	Not performed	Satisfactory	NILM	NILM	Negative (NILM)	
P06200	2019	2019-Q4	2019-12	Pre-pandemic	Cytology (Pap)	30-34	34	Not performed	Satisfactory	NILM	NILM	Negative (NILM)	
P06201	2019	2019-Q4	2019-12	Pre-pandemic	Cytology (Pap)	45-49	48	Not performed	Satisfactory	NILM	NILM	Negative (NILM)	
P06202	2019	2019-Q4	2019-12	Pre-pandemic	Cytology (Pap)	50-54	52	Not performed	Satisfactory	Positive	AGC	Glandular	
P06203	2019	2019-Q4	2019-12	Pre-pandemic	Cytology (Pap)	30-34	30	Not performed	Unsatisfactory	Unsatisfactory	Unsatisfactory	Unsatisfactory	
P06204	2019	2019-Q4	2019-12	Pre-pandemic	Cytology (Pap)	55-59	57	Not performed	Satisfactory	Positive	HSIL	High-grade squamous	
P06205	2019	2019-Q4	2019-12	Pre-pandemic	Cytology (Pap)	40-44	42	Not performed	Satisfactory	NILM	NILM	Negative (NILM)	Candida
P06206	2019	2019-Q4	2019-12	Pre-pandemic	Cytology (Pap)	50-54	53	Not performed	Satisfactory	NILM	NILM	Negative (NILM)	
P06207	2019	2019-Q4	2019-12	Pre-pandemic	Cytology (Pap)	40-44	43	Not performed	Satisfactory	NILM	NILM	Negative (NILM)	
P06208	2019	2019-Q4	2019-12	Pre-pandemic	Cytology (Pap)	45-49	47	Not performed	Satisfactory	NILM	NILM	Negative (NILM)	
P06209	2019	2019-Q4	2019-12	Pre-pandemic	Cytology (Pap)	40-44	42	Not performed	Satisfactory	NILM	NILM	Negative (NILM)	
P06210	2019	2019-Q4	2019-12	Pre-pandemic	Cytology (Pap)	40-44	44	Not performed	Satisfactory	NILM	NILM	Negative (NILM)	
P06211	2019	2019-Q4	2019-12	Pre-pandemic	Cytology (Pap)	60-64	61	Not performed	Satisfactory	NILM	NILM	Negative (NILM)	
P06212	2019	2019-Q4	2019-12	Pre-pandemic	Cytology (Pap)	45-49	48	Not performed	Satisfactory	NILM	NILM	Negative (NILM)	
P06213	2019	2019-Q4	2019-12	Pre-pandemic	Cytology (Pap)	30-34	33	Not performed	Satisfactory	NILM	NILM	Negative (NILM)	
P06214	2019	2019-Q4	2019-12	Pre-pandemic	Cytology (Pap)	60-64	64	Not performed	Satisfactory	NILM	NILM	Negative (NILM)	
P06215	2019	2019-Q4	2019-12	Pre-pandemic	Cytology (Pap)	60-64	60	Not performed	Satisfactory	NILM	NILM	Negative (NILM)	
P06216	2019	2019-Q4	2019-12	Pre-pandemic	Cytology (Pap)	60-64	63	Not performed	Satisfactory	NILM	NILM	Negative (NILM)	
P06217	2019	2019-Q4	2019-12	Pre-pandemic	Cytology (Pap)	35-39	35	Not performed	Satisfactory	NILM	NILM	Negative (NILM)	
P06218	2019	2019-Q4	2019-12	Pre-pandemic	Cytology (Pap)	45-49	47	Not performed	Satisfactory	NILM	NILM	Negative (NILM)	
P06219	2019	2019-Q4	2019-12	Pre-pandemic	Cytology (Pap)	50-54	54	Not performed	Satisfactory	NILM	NILM	Negative (NILM)	
P06220	2019	2019-Q4	2019-12	Pre-pandemic	Cytology (Pap)	40-44	42	Not performed	Satisfactory	NILM	NILM	Negative (NILM)	
P06221	2019	2019-Q4	2019-12	Pre-pandemic	Cytology (Pap)	30-34	30	Not performed	Satisfactory	NILM	NILM	Negative (NILM)	
P06222	2019	2019-Q4	2019-12	Pre-pandemic	Cytology (Pap)	30-34	34	Not performed	Satisfactory	NILM	NILM	Negative (NILM)	
P06223	2019	2019-Q4	2019-12	Pre-pandemic	Cytology (Pap)	50-54	50	Not performed	Satisfactory	Positive	ASC-US	Low-grade / equivocal squamous	
P06224	2019	2019-Q4	2019-12	Pre-pandemic	Cytology (Pap)	40-44	44	Not performed	Satisfactory	NILM	NILM	Negative (NILM)	
P06225	2019	2019-Q4	2019-12	Pre-pandemic	Cytology (Pap)	35-39	37	Not performed	Satisfactory	Positive	ASC-US	Low-grade / equivocal squamous	
P06226	2019	2019-Q4	2019-12	Pre-pandemic	Cytology (Pap)	60-64	64	Not performed	Satisfactory	NILM	NILM	Negative (NILM)	
P06227	2019	2019-Q4	2019-12	Pre-pandemic	Cytology (Pap)	40-44	41	Not performed	Satisfactory	NILM	NILM	Negative (NILM)	
P06228	2019	2019-Q4	2019-12	Pre-pandemic	Cytology (Pap)	25-29	27	Not performed	Satisfactory	NILM	NILM	Negative (NILM)	
P06229	2019	2019-Q4	2019-12	Pre-pandemic	Cytology (Pap)	50-54	50	Not performed	Satisfactory	NILM	NILM	Negative (NILM)	
P06230	2019	2019-Q4	2019-12	Pre-pandemic	Cytology (Pap)	50-54	54	Not performed	Satisfactory	NILM	NILM	Negative (NILM)	
P06231	2019	2019-Q4	2019-12	Pre-pandemic	Cytology (Pap)	40-44	41	Not performed	Satisfactory	NILM	NILM	Negative (NILM)	
P06232	2019	2019-Q4	2019-12	Pre-pandemic	Cytology (Pap)	40-44	43	Not performed	Satisfactory	Positive	ASC-H	High-grade squamous	
P06233	2019	2019-Q4	2019-12	Pre-pandemic	Cytology (Pap)	40-44	43	Not performed	Satisfactory	NILM	NILM	Negative (NILM)	
P06234	2019	2019-Q4	2019-12	Pre-pandemic	Cytology (Pap)	45-49	48	Not performed	Satisfactory	NILM	NILM	Negative (NILM)	
P06235	2019	2019-Q4	2019-12	Pre-pandemic	Cytology (Pap)	55-59	56	Not performed	Satisfactory	NILM	NILM	Negative (NILM)	
P06236	2019	2019-Q4	2019-12	Pre-pandemic	Cytology (Pap)	40-44	44	Not performed	Satisfactory	NILM	NILM	Negative (NILM)	
P06237	2019	2019-Q4	2019-12	Pre-pandemic	Cytology (Pap)	40-44	44	Not performed	Satisfactory	NILM	NILM	Negative (NILM)	
P06238	2019	2019-Q4	2019-12	Pre-pandemic	Cytology (Pap)	25-29	27	Not performed	Satisfactory	NILM	NILM	Negative (NILM)	

patient_id	year	quarter	month	period	primary_test	age_group	age_years	hpv_result	adequacy	result	bethesda	grade	infection
P06239	2019	2019-Q4	2019-12	Pre-pandemic	Cytology (Pap)	40-44	43	Not performed	Satisfactory	NILM	NILM	Negative (NILM)	
P06240	2019	2019-Q4	2019-12	Pre-pandemic	Cytology (Pap)	40-44	40	Not performed	Satisfactory	NILM	NILM	Negative (NILM)	
P06241	2019	2019-Q4	2019-12	Pre-pandemic	Cytology (Pap)	40-44	41	Not performed	Satisfactory	NILM	NILM	Negative (NILM)	
P06242	2019	2019-Q4	2019-12	Pre-pandemic	Cytology (Pap)	25-29	27	Not performed	Satisfactory	NILM	NILM	Negative (NILM)	
P06243	2019	2019-Q4	2019-12	Pre-pandemic	Cytology (Pap)	60-64	64	Not performed	Satisfactory	NILM	NILM	Negative (NILM)	
P06244	2019	2019-Q4	2019-12	Pre-pandemic	Cytology (Pap)	25-29	27	Not performed	Satisfactory	NILM	NILM	Negative (NILM)	
P06245	2019	2019-Q4	2019-12	Pre-pandemic	Cytology (Pap)	45-49	45	Not performed	Satisfactory	Positive	ASC-US	Low-grade / equivocal squamous	
P06246	2019	2019-Q4	2019-12	Pre-pandemic	Cytology (Pap)	45-49	47	Not performed	Satisfactory	Positive	LSIL	Low-grade / equivocal squamous	
P06247	2019	2019-Q4	2019-12	Pre-pandemic	Cytology (Pap)	50-54	54	Not performed	Satisfactory	NILM	NILM	Negative (NILM)	
P06248	2019	2019-Q4	2019-12	Pre-pandemic	Cytology (Pap)	55-59	55	Not performed	Unsatisfactory	Unsatisfactory	Unsatisfactory	Unsatisfactory	
P06249	2019	2019-Q4	2019-12	Pre-pandemic	Cytology (Pap)	30-34	30	Not performed	Satisfactory	NILM	NILM	Negative (NILM)	
P06250	2019	2019-Q4	2019-12	Pre-pandemic	Cytology (Pap)	35-39	38	Not performed	Satisfactory	NILM	NILM	Negative (NILM)	
P06251	2019	2019-Q4	2019-12	Pre-pandemic	Cytology (Pap)	45-49	48	Not performed	Satisfactory	NILM	NILM	Negative (NILM)	
P06252	2019	2019-Q4	2019-12	Pre-pandemic	Cytology (Pap)	40-44	41	Not performed	Satisfactory	NILM	NILM	Negative (NILM)	
P06253	2019	2019-Q4	2019-12	Pre-pandemic	Cytology (Pap)	60-64	62	Not performed	Satisfactory	NILM	NILM	Negative (NILM)	
P06254	2019	2019-Q4	2019-12	Pre-pandemic	Cytology (Pap)	35-39	37	Not performed	Satisfactory	NILM	NILM	Negative (NILM)	Gardnerella
P06255	2019	2019-Q4	2019-12	Pre-pandemic	Cytology (Pap)	40-44	42	Not performed	Satisfactory	NILM	NILM	Negative (NILM)	
P06256	2019	2019-Q4	2019-12	Pre-pandemic	Cytology (Pap)	30-34	31	Not performed	Satisfactory	NILM	NILM	Negative (NILM)	
P06257	2019	2019-Q4	2019-12	Pre-pandemic	Cytology (Pap)	45-49	48	Not performed	Satisfactory	NILM	NILM	Negative (NILM)	
P06258	2019	2019-Q4	2019-12	Pre-pandemic	Cytology (Pap)	30-34	30	Not performed	Satisfactory	NILM	NILM	Negative (NILM)	
P06259	2019	2019-Q4	2019-12	Pre-pandemic	Cytology (Pap)	45-49	48	Not performed	Satisfactory	NILM	NILM	Negative (NILM)	
P06260	2019	2019-Q4	2019-12	Pre-pandemic	Cytology (Pap)	60-64	61	Not performed	Satisfactory	NILM	NILM	Negative (NILM)	
P06261	2019	2019-Q4	2019-12	Pre-pandemic	Cytology (Pap)	50-54	53	Not performed	Satisfactory	NILM	NILM	Negative (NILM)	
P06262	2019	2019-Q4	2019-12	Pre-pandemic	Cytology (Pap)	45-49	49	Not performed	Satisfactory	NILM	NILM	Negative (NILM)	
P06263	2019	2019-Q4	2019-12	Pre-pandemic	Cytology (Pap)	30-34	32	Not performed	Satisfactory	NILM	NILM	Negative (NILM)	
P06264	2019	2019-Q4	2019-12	Pre-pandemic	Cytology (Pap)	35-39	39	Not performed	Satisfactory	NILM	NILM	Negative (NILM)	
P06265	2019	2019-Q4	2019-12	Pre-pandemic	Cytology (Pap)	35-39	39	Not performed	Satisfactory	NILM	NILM	Negative (NILM)	
P06266	2019	2019-Q4	2019-12	Pre-pandemic	Cytology (Pap)	35-39	39	Not performed	Satisfactory	NILM	NILM	Negative (NILM)	
P06267	2019	2019-Q4	2019-12	Pre-pandemic	Cytology (Pap)	60-64	64	Not performed	Satisfactory	NILM	NILM	Negative (NILM)	
P06268	2019	2019-Q4	2019-12	Pre-pandemic	Cytology (Pap)	55-59	55	Not performed	Satisfactory	NILM	NILM	Negative (NILM)	
P06269	2019	2019-Q4	2019-12	Pre-pandemic	Cytology (Pap)	40-44	42	Not performed	Unsatisfactory	Unsatisfactory	Unsatisfactory	Unsatisfactory	Trichomonas
P06270	2019	2019-Q4	2019-12	Pre-pandemic	Cytology (Pap)	35-39	36	Not performed	Satisfactory	NILM	NILM	Negative (NILM)	Trichomonas
P06271	2019	2019-Q4	2019-12	Pre-pandemic	Cytology (Pap)	45-49	49	Not performed	Satisfactory	NILM	NILM	Negative (NILM)	
P06272	2019	2019-Q4	2019-12	Pre-pandemic	Cytology (Pap)	45-49	48	Not performed	Satisfactory	NILM	NILM	Negative (NILM)	
P06273	2019	2019-Q4	2019-12	Pre-pandemic	Cytology (Pap)	30-34	31	Not performed	Satisfactory	NILM	NILM	Negative (NILM)	
P06274	2019	2019-Q4	2019-12	Pre-pandemic	Cytology (Pap)	30-34	34	Not performed	Satisfactory	NILM	NILM	Negative (NILM)	
P06275	2019	2019-Q4	2019-12	Pre-pandemic	Cytology (Pap)	50-54	51	Not performed	Satisfactory	NILM	NILM	Negative (NILM)	
P06276	2019	2019-Q4	2019-12	Pre-pandemic	Cytology (Pap)	55-59	55	Not performed	Satisfactory	NILM	NILM	Negative (NILM)	
P06277	2019	2019-Q4	2019-12	Pre-pandemic	Cytology (Pap)	55-59	55	Not performed	Satisfactory	NILM	NILM	Negative (NILM)	
P06278	2019	2019-Q4	2019-12	Pre-pandemic	Cytology (Pap)	45-49	49	Not performed	Satisfactory	NILM	NILM	Negative (NILM)	
P06279	2019	2019-Q4	2019-12	Pre-pandemic	Cytology (Pap)	55-59	55	Not performed	Satisfactory	NILM	NILM	Negative (NILM)	
P06280	2019	2019-Q4	2019-12	Pre-pandemic	Cytology (Pap)	35-39	35	Not performed	Satisfactory	NILM	NILM	Negative (NILM)	
P06281	2019	2019-Q4	2019-12	Pre-pandemic	Cytology (Pap)	35-39	35	Not performed	Satisfactory	NILM	NILM	Negative (NILM)	
P06282	2019	2019-Q4	2019-12	Pre-pandemic	Cytology (Pap)	50-54	54	Not performed	Satisfactory	Positive	ASC-US	Low-grade / equivocal squamous	
P06283	2019	2019-Q4	2019-12	Pre-pandemic	Cytology (Pap)	35-39	35	Not performed	Satisfactory	Positive	ASC-US	Low-grade / equivocal squamous	
P06284	2019	2019-Q4	2019-12	Pre-pandemic	Cytology (Pap)	40-44	41	Not performed	Unsatisfactory	Unsatisfactory	Unsatisfactory	Unsatisfactory	
P06285	2019	2019-Q4	2019-12	Pre-pandemic	Cytology (Pap)	35-39	35	Not performed	Satisfactory	NILM	NILM	Negative (NILM)	
P06286	2019	2019-Q4	2019-12	Pre-pandemic	Cytology (Pap)	40-44	44	Not performed	Satisfactory	NILM	NILM	Negative (NILM)	
P06287	2019	2019-Q4	2019-12	Pre-pandemic	Cytology (Pap)	40-44	42	Not performed	Satisfactory	NILM	NILM	Negative (NILM)	
P06288	2019	2019-Q4	2019-12	Pre-pandemic	Cytology (Pap)	45-49	46	Not performed	Satisfactory	Positive	ASC-US	Low-grade / equivocal squamous	
P06289	2019	2019-Q4	2019-12	Pre-pandemic	Cytology (Pap)	35-39	39	Not performed	Satisfactory	Positive	HSIL	High-grade squamous	
P06290	2019	2019-Q4	2019-12	Pre-pandemic	Cytology (Pap)	40-44	43	Not performed	Satisfactory	NILM	NILM	Negative (NILM)	
P06291	2019	2019-Q4	2019-12	Pre-pandemic	Cytology (Pap)	45-49	45	Not performed	Satisfactory	Positive	ASC-US	Low-grade / equivocal squamous	
P06292	2019	2019-Q4	2019-12	Pre-pandemic	Cytology (Pap)	45-49	49	Not performed	Satisfactory	Positive	ASC-H	High-grade squamous	
P06293	2019	2019-Q4	2019-12	Pre-pandemic	Cytology (Pap)	45-49	46	Not performed	Satisfactory	NILM	NILM	Negative (NILM)	
P06294	2019	2019-Q4	2019-12	Pre-pandemic	Cytology (Pap)	30-34	33	Not performed	Satisfactory	NILM	NILM	Negative (NILM)	
P06295	2019	2019-Q4	2019-12	Pre-pandemic	Cytology (Pap)	60-64	61	Not performed	Satisfactory	NILM	NILM	Negative (NILM)	
P06296	2019	2019-Q4	2019-12	Pre-pandemic	Cytology (Pap)	55-59	57	Not performed	Satisfactory	NILM	NILM	Negative (NILM)	
P06297	2019	2019-Q4	2019-12	Pre-pandemic	Cytology (Pap)	45-49	48	Not performed	Satisfactory	NILM	NILM	Negative (NILM)	
P06298	2019	2019-Q4	2019-12	Pre-pandemic	Cytology (Pap)	55-59	56	Not performed	Satisfactory	NILM	NILM	Negative (NILM)	
P06299	2019	2019-Q4	2019-12	Pre-pandemic	Cytology (Pap)	35-39	38	Not performed	Satisfactory	Positive	LSIL	Low-grade / equivocal squamous	
P06300	2019	2019-Q4	2019-12	Pre-pandemic	Cytology (Pap)	35-39	37	Not performed	Satisfactory	NILM	NILM	Negative (NILM)	
P06301	2019	2019-Q4	2019-12	Pre-pandemic	Cytology (Pap)	60-64	61	Not performed	Satisfactory	Positive	HSIL	High-grade squamous	
P06302	2019	2019-Q4	2019-12	Pre-pandemic	Cytology (Pap)	35-39	36	Not performed	Satisfactory	NILM	NILM	Negative (NILM)	
P06303	2019	2019-Q4	2019-12	Pre-pandemic	Cytology (Pap)	40-44	42	Not performed	Satisfactory	NILM	NILM	Negative (NILM)	
P06304	2019	2019-Q4	2019-12	Pre-pandemic	Cytology (Pap)	50-54	53	Not performed	Satisfactory	NILM	NILM	Negative (NILM)	
P06305	2019	2019-Q4	2019-12	Pre-pandemic	Cytology (Pap)	40-44	44	Not performed	Satisfactory	NILM	NILM	Negative (NILM)	Candida
P06306	2019	2019-Q4	2019-12	Pre-pandemic	Cytology (Pap)	55-59	58	Not performed	Satisfactory	NILM	NILM	Negative (NILM)	
P06307	2019	2019-Q4	2019-12	Pre-pandemic	Cytology (Pap)	45-49	48	Not performed	Satisfactory	NILM	NILM	Negative (NILM)	
P06308	2019	2019-Q4	2019-12	Pre-pandemic	Cytology (Pap)	35-39	39	Not performed	Satisfactory	NILM	NILM	Negative (NILM)	
P06309	2019	2019-Q4	2019-12	Pre-pandemic	Cytology (Pap)	50-54	54	Not performed	Satisfactory	NILM	NILM	Negative (NILM)	
P06310	2019	2019-Q4	2019-12	Pre-pandemic	Cytology (Pap)	30-34	30	Not performed	Satisfactory	NILM	NILM	Negative (NILM)	
P06311	2019	2019-Q4	2019-12	Pre-pandemic	Cytology (Pap)	50-54	52	Not performed	Satisfactory	NILM	NILM	Negative (NILM)	
P06312	2019	2019-Q4	2019-12	Pre-pandemic	Cytology (Pap)	40-44	40	Not performed	Satisfactory	NILM	NILM	Negative (NILM)	
P06313	2019	2019-Q4	2019-12	Pre-pandemic	Cytology (Pap)	55-59	55	Not performed	Satisfactory	NILM	NILM	Negative (NILM)	
P06314	2019	2019-Q4	2019-12	Pre-pandemic	Cytology (Pap)	40-44	43	Not performed	Satisfactory	NILM	NILM	Negative (NILM)	
P06315	2019	2019-Q4	2019-12	Pre-pandemic	Cytology (Pap)	50-54	54	Not performed	Satisfactory	NILM	NILM	Negative (NILM)	
P06316	2019	2019-Q4	2019-12	Pre-pandemic	Cytology (Pap)	55-59	56	Not performed	Satisfactory	Positive	HSIL	High-grade squamous	
P06317	2019	2019-Q4	2019-12	Pre-pandemic	Cytology (Pap)	55-59	57	Not performed	Satisfactory	Positive	HSIL	High-grade squamous	
P06318	2019	2019-Q4	2019-12	Pre-pandemic	Cytology (Pap)	30-34	32	Not performed	Satisfactory	NILM	NILM	Negative (NILM)	

patient_id	year	quarter	month	period	primary_test	age_group	age_years	hpv_result	adequacy	result	bethesda	grade	infection
P06319	2019	2019-Q4	2019-12	Pre-pandemic	Cytology (Pap)	30-34	33	Not performed	Satisfactory	Positive	LSIL	Low-grade / equivocal squamous	
P06320	2019	2019-Q4	2019-12	Pre-pandemic	Cytology (Pap)	55-59	57	Not performed	Satisfactory	NILM	NILM	Negative (NILM)	
P06321	2019	2019-Q4	2019-12	Pre-pandemic	Cytology (Pap)	50-54	53	Not performed	Satisfactory	NILM	NILM	Negative (NILM)	
P06322	2019	2019-Q4	2019-12	Pre-pandemic	Cytology (Pap)	40-44	44	Not performed	Satisfactory	NILM	NILM	Negative (NILM)	
P06323	2019	2019-Q4	2019-12	Pre-pandemic	Cytology (Pap)	30-34	31	Not performed	Satisfactory	NILM	NILM	Negative (NILM)	
P06324	2019	2019-Q4	2019-12	Pre-pandemic	Cytology (Pap)	45-49	45	Not performed	Satisfactory	NILM	NILM	Negative (NILM)	Trichomonas
P06325	2019	2019-Q4	2019-12	Pre-pandemic	Cytology (Pap)	45-49	49	Not performed	Satisfactory	NILM	NILM	Negative (NILM)	
P06326	2019	2019-Q4	2019-12	Pre-pandemic	Cytology (Pap)	40-44	41	Not performed	Satisfactory	NILM	NILM	Negative (NILM)	
P06327	2019	2019-Q4	2019-12	Pre-pandemic	Cytology (Pap)	35-39	35	Not performed	Satisfactory	NILM	NILM	Negative (NILM)	
P06328	2019	2019-Q4	2019-12	Pre-pandemic	Cytology (Pap)	60-64	63	Not performed	Satisfactory	NILM	NILM	Negative (NILM)	
P06329	2019	2019-Q4	2019-12	Pre-pandemic	Cytology (Pap)	40-44	44	Not performed	Satisfactory	NILM	NILM	Negative (NILM)	
P06330	2019	2019-Q4	2019-12	Pre-pandemic	Cytology (Pap)	55-59	58	Not performed	Satisfactory	NILM	NILM	Negative (NILM)	
P06331	2019	2019-Q4	2019-12	Pre-pandemic	Cytology (Pap)	40-44	44	Not performed	Satisfactory	NILM	NILM	Negative (NILM)	
P06332	2019	2019-Q4	2019-12	Pre-pandemic	Cytology (Pap)	40-44	42	Not performed	Satisfactory	NILM	NILM	Negative (NILM)	
P06333	2019	2019-Q4	2019-12	Pre-pandemic	Cytology (Pap)	40-44	43	Not performed	Satisfactory	Positive	AGC	Glandular	
P06334	2019	2019-Q4	2019-12	Pre-pandemic	Cytology (Pap)	40-44	44	Not performed	Satisfactory	NILM	NILM	Negative (NILM)	
P06335	2019	2019-Q4	2019-12	Pre-pandemic	Cytology (Pap)	45-49	46	Not performed	Satisfactory	Positive	LSIL	Low-grade / equivocal squamous	
P06336	2019	2019-Q4	2019-12	Pre-pandemic	Cytology (Pap)	55-59	56	Not performed	Satisfactory	NILM	NILM	Negative (NILM)	
P06337	2019	2019-Q4	2019-12	Pre-pandemic	Cytology (Pap)	55-59	56	Not performed	Satisfactory	NILM	NILM	Negative (NILM)	
P06338	2019	2019-Q4	2019-12	Pre-pandemic	Cytology (Pap)	40-44	42	Not performed	Satisfactory	NILM	NILM	Negative (NILM)	
P06339	2019	2019-Q4	2019-12	Pre-pandemic	Cytology (Pap)	45-49	47	Not performed	Satisfactory	Positive	ASC-US	Low-grade / equivocal squamous	Candida
P06340	2019	2019-Q4	2019-12	Pre-pandemic	Cytology (Pap)	40-44	44	Not performed	Satisfactory	NILM	NILM	Negative (NILM)	
P06341	2019	2019-Q4	2019-12	Pre-pandemic	Cytology (Pap)	40-44	40	Not performed	Satisfactory	NILM	NILM	Negative (NILM)	
P06342	2019	2019-Q4	2019-12	Pre-pandemic	Cytology (Pap)	35-39	38	Not performed	Satisfactory	NILM	NILM	Negative (NILM)	
P06343	2019	2019-Q4	2019-12	Pre-pandemic	Cytology (Pap)	55-59	56	Not performed	Satisfactory	NILM	NILM	Negative (NILM)	
P06344	2019	2019-Q4	2019-12	Pre-pandemic	Cytology (Pap)	45-49	47	Not performed	Satisfactory	NILM	NILM	Negative (NILM)	
P06345	2019	2019-Q4	2019-12	Pre-pandemic	Cytology (Pap)	35-39	38	Not performed	Unsatisfactory	Unsatisfactory	Unsatisfactory	Unsatisfactory	
P06346	2019	2019-Q4	2019-12	Pre-pandemic	Cytology (Pap)	45-49	45	Not performed	Satisfactory	NILM	NILM	Negative (NILM)	
P06347	2019	2019-Q4	2019-12	Pre-pandemic	Cytology (Pap)	50-54	53	Not performed	Satisfactory	NILM	NILM	Negative (NILM)	
P06348	2020	2020-Q1	2020-01	Pandemic	Cytology (Pap)	40-44	40	Not performed	Satisfactory	Positive	HSIL	High-grade squamous	
P06349	2020	2020-Q1	2020-01	Pandemic	Cytology (Pap)	30-34	32	Not performed	Satisfactory	NILM	NILM	Negative (NILM)	
P06350	2020	2020-Q1	2020-01	Pandemic	Cytology (Pap)	25-29	29	Not performed	Satisfactory	NILM	NILM	Negative (NILM)	
P06351	2020	2020-Q1	2020-01	Pandemic	Cytology (Pap)	25-29	28	Not performed	Satisfactory	NILM	NILM	Negative (NILM)	
P06352	2020	2020-Q1	2020-01	Pandemic	Cytology (Pap)	30-34	34	Not performed	Satisfactory	NILM	NILM	Negative (NILM)	
P06353	2020	2020-Q1	2020-01	Pandemic	Cytology (Pap)	50-54	50	Not performed	Satisfactory	NILM	NILM	Negative (NILM)	
P06354	2020	2020-Q1	2020-01	Pandemic	Cytology (Pap)	40-44	41	Not performed	Satisfactory	Positive	ASC-US	Low-grade / equivocal squamous	
P06355	2020	2020-Q1	2020-01	Pandemic	Cytology (Pap)	25-29	25	Not performed	Satisfactory	NILM	NILM	Negative (NILM)	
P06356	2020	2020-Q1	2020-01	Pandemic	Cytology (Pap)	55-59	58	Not performed	Satisfactory	NILM	NILM	Negative (NILM)	
P06357	2020	2020-Q1	2020-01	Pandemic	Cytology (Pap)	40-44	40	Not performed	Satisfactory	NILM	NILM	Negative (NILM)	
P06358	2020	2020-Q1	2020-01	Pandemic	Cytology (Pap)	40-44	43	Not performed	Satisfactory	NILM	NILM	Negative (NILM)	
P06359	2020	2020-Q1	2020-01	Pandemic	Cytology (Pap)	45-49	47	Not performed	Satisfactory	NILM	NILM	Negative (NILM)	
P06360	2020	2020-Q1	2020-01	Pandemic	Cytology (Pap)	55-59	55	Not performed	Satisfactory	NILM	NILM	Negative (NILM)	
P06361	2020	2020-Q1	2020-01	Pandemic	Cytology (Pap)	40-44	43	Not performed	Satisfactory	NILM	NILM	Negative (NILM)	
P06362	2020	2020-Q1	2020-01	Pandemic	Cytology (Pap)	45-49	48	Not performed	Satisfactory	NILM	NILM	Negative (NILM)	
P06363	2020	2020-Q1	2020-01	Pandemic	Cytology (Pap)	40-44	43	Not performed	Satisfactory	Positive	ASC-US	Low-grade / equivocal squamous	Trichomonas
P06364	2020	2020-Q1	2020-01	Pandemic	Cytology (Pap)	55-59	55	Not performed	Satisfactory	NILM	NILM	Negative (NILM)	
P06365	2020	2020-Q1	2020-01	Pandemic	Cytology (Pap)	40-44	44	Not performed	Satisfactory	NILM	NILM	Negative (NILM)	
P06366	2020	2020-Q1	2020-01	Pandemic	Cytology (Pap)	45-49	47	Not performed	Satisfactory	NILM	NILM	Negative (NILM)	
P06367	2020	2020-Q1	2020-01	Pandemic	Cytology (Pap)	40-44	42	Not performed	Satisfactory	NILM	NILM	Negative (NILM)	
P06368	2020	2020-Q1	2020-01	Pandemic	Cytology (Pap)	40-44	40	Not performed	Satisfactory	NILM	NILM	Negative (NILM)	
P06369	2020	2020-Q1	2020-01	Pandemic	Cytology (Pap)	40-44	40	Not performed	Satisfactory	NILM	NILM	Negative (NILM)	
P06370	2020	2020-Q1	2020-01	Pandemic	Cytology (Pap)	40-44	44	Not performed	Satisfactory	NILM	NILM	Negative (NILM)	
P06371	2020	2020-Q1	2020-01	Pandemic	Cytology (Pap)	50-54	53	Not performed	Satisfactory	NILM	NILM	Negative (NILM)	
P06372	2020	2020-Q1	2020-01	Pandemic	Cytology (Pap)	55-59	55	Not performed	Satisfactory	NILM	NILM	Negative (NILM)	
P06373	2020	2020-Q1	2020-01	Pandemic	Cytology (Pap)	40-44	41	Not performed	Satisfactory	NILM	NILM	Negative (NILM)	
P06374	2020	2020-Q1	2020-01	Pandemic	Cytology (Pap)	55-59	55	Not performed	Satisfactory	NILM	NILM	Negative (NILM)	
P06375	2020	2020-Q1	2020-01	Pandemic	Cytology (Pap)	50-54	50	Not performed	Satisfactory	NILM	NILM	Negative (NILM)	Gardnerella
P06376	2020	2020-Q1	2020-01	Pandemic	Cytology (Pap)	50-54	54	Not performed	Satisfactory	NILM	NILM	Negative (NILM)	
P06377	2020	2020-Q1	2020-01	Pandemic	Cytology (Pap)	35-39	37	Not performed	Satisfactory	NILM	NILM	Negative (NILM)	
P06378	2020	2020-Q1	2020-01	Pandemic	Cytology (Pap)	40-44	40	Not performed	Satisfactory	NILM	NILM	Negative (NILM)	
P06379	2020	2020-Q1	2020-01	Pandemic	Cytology (Pap)	40-44	43	Not performed	Satisfactory	NILM	NILM	Negative (NILM)	
P06380	2020	2020-Q1	2020-01	Pandemic	Cytology (Pap)	30-34	34	Not performed	Satisfactory	NILM	NILM	Negative (NILM)	
P06381	2020	2020-Q1	2020-01	Pandemic	Cytology (Pap)	60-64	64	Not performed	Satisfactory	NILM	NILM	Negative (NILM)	Trichomonas
P06382	2020	2020-Q1	2020-01	Pandemic	Cytology (Pap)	30-34	30	Not performed	Satisfactory	NILM	NILM	Negative (NILM)	
P06383	2020	2020-Q1	2020-01	Pandemic	Cytology (Pap)	50-54	52	Not performed	Satisfactory	NILM	NILM	Negative (NILM)	
P06384	2020	2020-Q1	2020-01	Pandemic	Cytology (Pap)	40-44	43	Not performed	Satisfactory	NILM	NILM	Negative (NILM)	
P06385	2020	2020-Q1	2020-01	Pandemic	Cytology (Pap)	30-34	34	Not performed	Satisfactory	Positive	LSIL	Low-grade / equivocal squamous	
P06386	2020	2020-Q1	2020-01	Pandemic	Cytology (Pap)	35-39	39	Not performed	Satisfactory	NILM	NILM	Negative (NILM)	
P06387	2020	2020-Q1	2020-01	Pandemic	Cytology (Pap)	30-34	32	Not performed	Satisfactory	NILM	NILM	Negative (NILM)	
P06388	2020	2020-Q1	2020-01	Pandemic	Cytology (Pap)	40-44	42	Not performed	Satisfactory	NILM	NILM	Negative (NILM)	
P06389	2020	2020-Q1	2020-01	Pandemic	Cytology (Pap)	45-49	47	Not performed	Satisfactory	Positive	ASC-US	Low-grade / equivocal squamous	
P06390	2020	2020-Q1	2020-01	Pandemic	Cytology (Pap)	50-54	50	Not performed	Satisfactory	NILM	NILM	Negative (NILM)	
P06391	2020	2020-Q1	2020-01	Pandemic	Cytology (Pap)	40-44	44	Not performed	Satisfactory	NILM	NILM	Negative (NILM)	
P06392	2020	2020-Q1	2020-01	Pandemic	Cytology (Pap)	35-39	37	Not performed	Unsatisfactory	Unsatisfactory	Unsatisfactory	Unsatisfactory	
P06393	2020	2020-Q1	2020-01	Pandemic	Cytology (Pap)	35-39	37	Not performed	Unsatisfactory	Unsatisfactory	Unsatisfactory	Unsatisfactory	
P06394	2020	2020-Q1	2020-01	Pandemic	Cytology (Pap)	40-44	41	Not performed	Satisfactory	NILM	NILM	Negative (NILM)	
P06395	2020	2020-Q1	2020-01	Pandemic	Cytology (Pap)	40-44	43	Not performed	Satisfactory	NILM	NILM	Negative (NILM)	
P06396	2020	2020-Q1	2020-01	Pandemic	Cytology (Pap)	35-39	39	Not performed	Satisfactory	NILM	NILM	Negative (NILM)	
P06397	2020	2020-Q1	2020-01	Pandemic	Cytology (Pap)	60-64	63	Not performed	Satisfactory	Positive	ASC-US	Low-grade / equivocal squamous	
P06398	2020	2020-Q1	2020-01	Pandemic	Cytology (Pap)	50-54	51	Not performed	Satisfactory	NILM	NILM	Negative (NILM)	

patient_id	year	quarter	month	period	primary_test	age_group	age_years	hpv_result	adequacy	result	bethesda	grade	infection
P06399	2020	2020-Q1	2020-01	Pandemic	Cytology (Pap)	50-54	54	Not performed	Satisfactory	NILM	NILM	Negative (NILM)	
P06400	2020	2020-Q1	2020-01	Pandemic	Cytology (Pap)	25-29	25	Not performed	Satisfactory	NILM	NILM	Negative (NILM)	
P06401	2020	2020-Q1	2020-01	Pandemic	Cytology (Pap)	45-49	48	Not performed	Satisfactory	NILM	NILM	Negative (NILM)	
P06402	2020	2020-Q1	2020-01	Pandemic	Cytology (Pap)	45-49	48	Not performed	Satisfactory	NILM	NILM	Negative (NILM)	
P06403	2020	2020-Q1	2020-01	Pandemic	Cytology (Pap)	45-49	47	Not performed	Satisfactory	NILM	NILM	Negative (NILM)	
P06404	2020	2020-Q1	2020-01	Pandemic	Cytology (Pap)	45-49	49	Not performed	Satisfactory	NILM	NILM	Negative (NILM)	
P06405	2020	2020-Q1	2020-01	Pandemic	Cytology (Pap)	25-29	27	Not performed	Satisfactory	Positive	ASC-US	Low-grade / equivocal squamous	
P06406	2020	2020-Q1	2020-01	Pandemic	Cytology (Pap)	35-39	36	Not performed	Satisfactory	NILM	NILM	Negative (NILM)	
P06407	2020	2020-Q1	2020-01	Pandemic	Cytology (Pap)	55-59	55	Not performed	Satisfactory	NILM	NILM	Negative (NILM)	
P06408	2020	2020-Q1	2020-01	Pandemic	Cytology (Pap)	40-44	42	Not performed	Satisfactory	NILM	NILM	Negative (NILM)	
P06409	2020	2020-Q1	2020-01	Pandemic	Cytology (Pap)	55-59	58	Not performed	Satisfactory	NILM	NILM	Negative (NILM)	
P06410	2020	2020-Q1	2020-01	Pandemic	Cytology (Pap)	55-59	57	Not performed	Satisfactory	NILM	NILM	Negative (NILM)	Candida
P06411	2020	2020-Q1	2020-01	Pandemic	Cytology (Pap)	50-54	51	Not performed	Satisfactory	NILM	NILM	Negative (NILM)	
P06412	2020	2020-Q1	2020-01	Pandemic	Cytology (Pap)	45-49	45	Not performed	Satisfactory	NILM	NILM	Negative (NILM)	
P06413	2020	2020-Q1	2020-01	Pandemic	Cytology (Pap)	50-54	53	Not performed	Satisfactory	NILM	NILM	Negative (NILM)	
P06414	2020	2020-Q1	2020-01	Pandemic	Cytology (Pap)	30-34	33	Not performed	Satisfactory	NILM	NILM	Negative (NILM)	
P06415	2020	2020-Q1	2020-01	Pandemic	Cytology (Pap)	40-44	41	Not performed	Satisfactory	NILM	NILM	Negative (NILM)	
P06416	2020	2020-Q1	2020-01	Pandemic	Cytology (Pap)	55-59	59	Not performed	Satisfactory	NILM	NILM	Negative (NILM)	
P06417	2020	2020-Q1	2020-01	Pandemic	Cytology (Pap)	35-39	39	Not performed	Satisfactory	NILM	NILM	Negative (NILM)	
P06418	2020	2020-Q1	2020-01	Pandemic	Cytology (Pap)	45-49	46	Not performed	Satisfactory	NILM	NILM	Negative (NILM)	
P06419	2020	2020-Q1	2020-01	Pandemic	Cytology (Pap)	45-49	45	Not performed	Satisfactory	NILM	NILM	Negative (NILM)	
P06420	2020	2020-Q1	2020-01	Pandemic	Cytology (Pap)	55-59	56	Not performed	Satisfactory	NILM	NILM	Negative (NILM)	
P06421	2020	2020-Q1	2020-01	Pandemic	Cytology (Pap)	40-44	42	Not performed	Satisfactory	NILM	NILM	Negative (NILM)	
P06422	2020	2020-Q1	2020-01	Pandemic	Cytology (Pap)	55-59	56	Not performed	Satisfactory	NILM	NILM	Negative (NILM)	
P06423	2020	2020-Q1	2020-01	Pandemic	Cytology (Pap)	30-34	34	Not performed	Satisfactory	Positive	ASC-US	Low-grade / equivocal squamous	
P06424	2020	2020-Q1	2020-01	Pandemic	Cytology (Pap)	45-49	47	Not performed	Satisfactory	NILM	NILM	Negative (NILM)	
P06425	2020	2020-Q1	2020-01	Pandemic	Cytology (Pap)	35-39	35	Not performed	Unsatisfactory	Unsatisfactory	Unsatisfactory	Unsatisfactory	
P06426	2020	2020-Q1	2020-01	Pandemic	Cytology (Pap)	50-54	52	Not performed	Satisfactory	NILM	NILM	Negative (NILM)	
P06427	2020	2020-Q1	2020-01	Pandemic	Cytology (Pap)	45-49	46	Not performed	Satisfactory	NILM	NILM	Negative (NILM)	
P06428	2020	2020-Q1	2020-01	Pandemic	Cytology (Pap)	40-44	43	Not performed	Satisfactory	NILM	NILM	Negative (NILM)	
P06429	2020	2020-Q1	2020-01	Pandemic	Cytology (Pap)	25-29	27	Not performed	Satisfactory	NILM	NILM	Negative (NILM)	
P06430	2020	2020-Q1	2020-01	Pandemic	Cytology (Pap)	40-44	44	Not performed	Satisfactory	NILM	NILM	Negative (NILM)	
P06431	2020	2020-Q1	2020-01	Pandemic	Cytology (Pap)	50-54	53	Not performed	Satisfactory	NILM	NILM	Negative (NILM)	
P06432	2020	2020-Q1	2020-01	Pandemic	Cytology (Pap)	30-34	30	Not performed	Satisfactory	NILM	NILM	Negative (NILM)	
P06433	2020	2020-Q1	2020-01	Pandemic	Cytology (Pap)	55-59	58	Not performed	Satisfactory	NILM	NILM	Negative (NILM)	
P06434	2020	2020-Q1	2020-01	Pandemic	Cytology (Pap)	55-59	57	Not performed	Satisfactory	Positive	ASC-US	Low-grade / equivocal squamous	
P06435	2020	2020-Q1	2020-01	Pandemic	Cytology (Pap)	45-49	45	Not performed	Satisfactory	NILM	NILM	Negative (NILM)	
P06436	2020	2020-Q1	2020-01	Pandemic	Cytology (Pap)	40-44	42	Not performed	Satisfactory	NILM	NILM	Negative (NILM)	Trichomonas
P06437	2020	2020-Q1	2020-01	Pandemic	Cytology (Pap)	45-49	49	Not performed	Satisfactory	NILM	NILM	Negative (NILM)	
P06438	2020	2020-Q1	2020-01	Pandemic	Cytology (Pap)	40-44	44	Not performed	Satisfactory	NILM	NILM	Negative (NILM)	
P06439	2020	2020-Q1	2020-01	Pandemic	Cytology (Pap)	45-49	45	Not performed	Satisfactory	NILM	NILM	Negative (NILM)	
P06440	2020	2020-Q1	2020-01	Pandemic	Cytology (Pap)	35-39	37	Not performed	Satisfactory	NILM	NILM	Negative (NILM)	
P06441	2020	2020-Q1	2020-01	Pandemic	Cytology (Pap)	35-39	38	Not performed	Satisfactory	NILM	NILM	Negative (NILM)	
P06442	2020	2020-Q1	2020-01	Pandemic	Cytology (Pap)	55-59	58	Not performed	Satisfactory	NILM	NILM	Negative (NILM)	
P06443	2020	2020-Q1	2020-01	Pandemic	Cytology (Pap)	60-64	64	Not performed	Satisfactory	NILM	NILM	Negative (NILM)	
P06444	2020	2020-Q1	2020-01	Pandemic	Cytology (Pap)	50-54	51	Not performed	Satisfactory	NILM	NILM	Negative (NILM)	
P06445	2020	2020-Q1	2020-01	Pandemic	Cytology (Pap)	25-29	29	Not performed	Satisfactory	NILM	NILM	Negative (NILM)	
P06446	2020	2020-Q1	2020-01	Pandemic	Cytology (Pap)	50-54	51	Not performed	Satisfactory	Positive	ASC-US	Low-grade / equivocal squamous	
P06447	2020	2020-Q1	2020-01	Pandemic	Cytology (Pap)	45-49	45	Not performed	Satisfactory	NILM	NILM	Negative (NILM)	
P06448	2020	2020-Q1	2020-01	Pandemic	Cytology (Pap)	50-54	50	Not performed	Satisfactory	Positive	LSIL	Low-grade / equivocal squamous	
P06449	2020	2020-Q1	2020-01	Pandemic	Cytology (Pap)	40-44	40	Not performed	Unsatisfactory	Unsatisfactory	Unsatisfactory	Unsatisfactory	
P06450	2020	2020-Q1	2020-01	Pandemic	Cytology (Pap)	30-34	31	Not performed	Satisfactory	Positive	ASC-US	Low-grade / equivocal squamous	
P06451	2020	2020-Q1	2020-01	Pandemic	Cytology (Pap)	30-34	31	Not performed	Satisfactory	NILM	NILM	Negative (NILM)	
P06452	2020	2020-Q1	2020-01	Pandemic	Cytology (Pap)	55-59	56	Not performed	Satisfactory	NILM	NILM	Negative (NILM)	
P06453	2020	2020-Q1	2020-01	Pandemic	Cytology (Pap)	40-44	42	Not performed	Satisfactory	NILM	NILM	Negative (NILM)	
P06454	2020	2020-Q1	2020-01	Pandemic	Cytology (Pap)	40-44	40	Not performed	Satisfactory	NILM	NILM	Negative (NILM)	
P06455	2020	2020-Q1	2020-01	Pandemic	Cytology (Pap)	45-49	46	Not performed	Satisfactory	NILM	NILM	Negative (NILM)	
P06456	2020	2020-Q1	2020-01	Pandemic	Cytology (Pap)	40-44	44	Not performed	Satisfactory	NILM	NILM	Negative (NILM)	
P06457	2020	2020-Q1	2020-01	Pandemic	Cytology (Pap)	45-49	49	Not performed	Satisfactory	NILM	NILM	Negative (NILM)	
P06458	2020	2020-Q1	2020-01	Pandemic	Cytology (Pap)	30-34	34	Not performed	Satisfactory	NILM	NILM	Negative (NILM)	Other
P06459	2020	2020-Q1	2020-01	Pandemic	Cytology (Pap)	35-39	36	Not performed	Satisfactory	Positive	ASC-US	Low-grade / equivocal squamous	
P06460	2020	2020-Q1	2020-01	Pandemic	Cytology (Pap)	50-54	52	Not performed	Satisfactory	NILM	NILM	Negative (NILM)	
P06461	2020	2020-Q1	2020-01	Pandemic	Cytology (Pap)	35-39	38	Not performed	Satisfactory	NILM	NILM	Negative (NILM)	
P06462	2020	2020-Q1	2020-01	Pandemic	Cytology (Pap)	55-59	59	Not performed	Unsatisfactory	Unsatisfactory	Unsatisfactory	Unsatisfactory	
P06463	2020	2020-Q1	2020-01	Pandemic	Cytology (Pap)	35-39	36	Not performed	Satisfactory	NILM	NILM	Negative (NILM)	
P06464	2020	2020-Q1	2020-01	Pandemic	Cytology (Pap)	50-54	51	Not performed	Satisfactory	NILM	NILM	Negative (NILM)	
P06465	2020	2020-Q1	2020-01	Pandemic	Cytology (Pap)	55-59	58	Not performed	Satisfactory	NILM	NILM	Negative (NILM)	
P06466	2020	2020-Q1	2020-01	Pandemic	Cytology (Pap)	55-59	58	Not performed	Satisfactory	Positive	HSIL	High-grade squamous	
P06467	2020	2020-Q1	2020-01	Pandemic	Cytology (Pap)	55-59	58	Not performed	Satisfactory	NILM	NILM	Negative (NILM)	
P06468	2020	2020-Q1	2020-01	Pandemic	Cytology (Pap)	55-59	56	Not performed	Satisfactory	NILM	NILM	Negative (NILM)	
P06469	2020	2020-Q1	2020-01	Pandemic	Cytology (Pap)	45-49	47	Not performed	Satisfactory	Positive	HSIL	High-grade squamous	
P06470	2020	2020-Q1	2020-01	Pandemic	Cytology (Pap)	35-39	37	Not performed	Satisfactory	Positive	ASC-US	Low-grade / equivocal squamous	
P06471	2020	2020-Q1	2020-01	Pandemic	Cytology (Pap)	45-49	48	Not performed	Satisfactory	NILM	NILM	Negative (NILM)	
P06472	2020	2020-Q1	2020-01	Pandemic	Cytology (Pap)	50-54	53	Not performed	Satisfactory	NILM	NILM	Negative (NILM)	
P06473	2020	2020-Q1	2020-01	Pandemic	Cytology (Pap)	45-49	46	Not performed	Satisfactory	NILM	NILM	Negative (NILM)	
P06474	2020	2020-Q1	2020-01	Pandemic	Cytology (Pap)	45-49	47	Not performed	Satisfactory	NILM	NILM	Negative (NILM)	
P06475	2020	2020-Q1	2020-01	Pandemic	Cytology (Pap)	30-34	32	Not performed	Satisfactory	NILM	NILM	Negative (NILM)	
P06476	2020	2020-Q1	2020-01	Pandemic	Cytology (Pap)	50-54	54	Not performed	Unsatisfactory	Unsatisfactory	Unsatisfactory	Unsatisfactory	
P06477	2020	2020-Q1	2020-01	Pandemic	Cytology (Pap)	60-64	63	Not performed	Satisfactory	NILM	NILM	Negative (NILM)	
P06478	2020	2020-Q1	2020-01	Pandemic	Cytology (Pap)	35-39	39	Not performed	Satisfactory	NILM	NILM	Negative (NILM)	

patient_id	year	quarter	month	period	primary_test	age_group	age_years	hpv_result	adequacy	result	bethesda	grade	infection
P06479	2020	2020-Q1	2020-01	Pandemic	Cytology (Pap)	40-44	43	Not performed	Satisfactory	NILM	NILM	Negative (NILM)	
P06480	2020	2020-Q1	2020-01	Pandemic	Cytology (Pap)	25-29	29	Not performed	Unsatisfactory	Unsatisfactory	Unsatisfactory	Unsatisfactory	
P06481	2020	2020-Q1	2020-01	Pandemic	Cytology (Pap)	50-54	50	Not performed	Satisfactory	NILM	NILM	Negative (NILM)	
P06482	2020	2020-Q1	2020-01	Pandemic	Cytology (Pap)	40-44	43	Not performed	Satisfactory	NILM	NILM	Negative (NILM)	
P06483	2020	2020-Q1	2020-01	Pandemic	Cytology (Pap)	30-34	33	Not performed	Satisfactory	Positive	ASC-US	Low-grade / equivocal squamous	
P06484	2020	2020-Q1	2020-01	Pandemic	Cytology (Pap)	40-44	42	Not performed	Satisfactory	NILM	NILM	Negative (NILM)	
P06485	2020	2020-Q1	2020-01	Pandemic	Cytology (Pap)	60-64	63	Not performed	Unsatisfactory	Unsatisfactory	Unsatisfactory	Unsatisfactory	
P06486	2020	2020-Q1	2020-01	Pandemic	Cytology (Pap)	60-64	64	Not performed	Satisfactory	NILM	NILM	Negative (NILM)	
P06487	2020	2020-Q1	2020-01	Pandemic	Cytology (Pap)	25-29	28	Not performed	Satisfactory	NILM	NILM	Negative (NILM)	
P06488	2020	2020-Q1	2020-01	Pandemic	Cytology (Pap)	45-49	45	Not performed	Satisfactory	NILM	NILM	Negative (NILM)	Gardnerella
P06489	2020	2020-Q1	2020-01	Pandemic	Cytology (Pap)	40-44	43	Not performed	Satisfactory	NILM	NILM	Negative (NILM)	
P06490	2020	2020-Q1	2020-01	Pandemic	Cytology (Pap)	50-54	53	Not performed	Satisfactory	Positive	LSIL	Low-grade / equivocal squamous	Candida
P06491	2020	2020-Q1	2020-01	Pandemic	Cytology (Pap)	25-29	27	Not performed	Satisfactory	Positive	LSIL	Low-grade / equivocal squamous	Candida
P06492	2020	2020-Q1	2020-01	Pandemic	Cytology (Pap)	25-29	29	Not performed	Satisfactory	NILM	NILM	Negative (NILM)	
P06493	2020	2020-Q1	2020-01	Pandemic	Cytology (Pap)	55-59	57	Not performed	Satisfactory	NILM	NILM	Negative (NILM)	
P06494	2020	2020-Q1	2020-01	Pandemic	Cytology (Pap)	40-44	44	Not performed	Satisfactory	NILM	NILM	Negative (NILM)	
P06495	2020	2020-Q1	2020-01	Pandemic	Cytology (Pap)	50-54	54	Not performed	Satisfactory	NILM	NILM	Negative (NILM)	
P06496	2020	2020-Q1	2020-01	Pandemic	Cytology (Pap)	40-44	41	Not performed	Satisfactory	NILM	NILM	Negative (NILM)	
P06497	2020	2020-Q1	2020-01	Pandemic	Cytology (Pap)	40-44	44	Not performed	Satisfactory	NILM	NILM	Negative (NILM)	
P06498	2020	2020-Q1	2020-01	Pandemic	Cytology (Pap)	60-64	63	Not performed	Satisfactory	NILM	NILM	Negative (NILM)	
P06499	2020	2020-Q1	2020-01	Pandemic	Cytology (Pap)	55-59	59	Not performed	Satisfactory	Positive	ASC-US	Low-grade / equivocal squamous	
P06500	2020	2020-Q1	2020-01	Pandemic	Cytology (Pap)	50-54	53	Not performed	Satisfactory	Positive	HSIL	High-grade squamous	
P06501	2020	2020-Q1	2020-01	Pandemic	Cytology (Pap)	35-39	37	Not performed	Satisfactory	NILM	NILM	Negative (NILM)	
P06502	2020	2020-Q1	2020-01	Pandemic	Cytology (Pap)	40-44	40	Not performed	Satisfactory	NILM	NILM	Negative (NILM)	
P06503	2020	2020-Q1	2020-01	Pandemic	Cytology (Pap)	40-44	40	Not performed	Satisfactory	Positive	LSIL	Low-grade / equivocal squamous	Trichomonas
P06504	2020	2020-Q1	2020-01	Pandemic	Cytology (Pap)	35-39	38	Not performed	Satisfactory	NILM	NILM	Negative (NILM)	
P06505	2020	2020-Q1	2020-01	Pandemic	Cytology (Pap)	50-54	54	Not performed	Satisfactory	NILM	NILM	Negative (NILM)	
P06506	2020	2020-Q1	2020-01	Pandemic	Cytology (Pap)	60-64	62	Not performed	Satisfactory	NILM	NILM	Negative (NILM)	
P06507	2020	2020-Q1	2020-01	Pandemic	Cytology (Pap)	50-54	51	Not performed	Satisfactory	Positive	ASC-US	Low-grade / equivocal squamous	
P06508	2020	2020-Q1	2020-01	Pandemic	Cytology (Pap)	35-39	39	Not performed	Satisfactory	NILM	NILM	Negative (NILM)	
P06509	2020	2020-Q1	2020-01	Pandemic	Cytology (Pap)	40-44	41	Not performed	Satisfactory	NILM	NILM	Negative (NILM)	
P06510	2020	2020-Q1	2020-01	Pandemic	Cytology (Pap)	45-49	48	Not performed	Satisfactory	NILM	NILM	Negative (NILM)	
P06511	2020	2020-Q1	2020-01	Pandemic	Cytology (Pap)	40-44	42	Not performed	Satisfactory	NILM	NILM	Negative (NILM)	
P06512	2020	2020-Q1	2020-01	Pandemic	Cytology (Pap)	45-49	46	Not performed	Satisfactory	NILM	NILM	Negative (NILM)	Candida
P06513	2020	2020-Q1	2020-01	Pandemic	Cytology (Pap)	35-39	35	Not performed	Unsatisfactory	Unsatisfactory	Unsatisfactory	Unsatisfactory	
P06514	2020	2020-Q1	2020-01	Pandemic	Cytology (Pap)	60-64	62	Not performed	Satisfactory	Positive	LSIL	Low-grade / equivocal squamous	
P06515	2020	2020-Q1	2020-01	Pandemic	Cytology (Pap)	40-44	43	Not performed	Satisfactory	NILM	NILM	Negative (NILM)	
P06516	2020	2020-Q1	2020-01	Pandemic	Cytology (Pap)	40-44	42	Not performed	Satisfactory	NILM	NILM	Negative (NILM)	
P06517	2020	2020-Q1	2020-01	Pandemic	Cytology (Pap)	55-59	57	Not performed	Satisfactory	NILM	NILM	Negative (NILM)	
P06518	2020	2020-Q1	2020-01	Pandemic	Cytology (Pap)	60-64	62	Not performed	Satisfactory	NILM	NILM	Negative (NILM)	
P06519	2020	2020-Q1	2020-01	Pandemic	Cytology (Pap)	35-39	36	Not performed	Satisfactory	NILM	NILM	Negative (NILM)	
P06520	2020	2020-Q1	2020-01	Pandemic	Cytology (Pap)	35-39	36	Not performed	Satisfactory	NILM	NILM	Negative (NILM)	
P06521	2020	2020-Q1	2020-01	Pandemic	Cytology (Pap)	50-54	52	Not performed	Satisfactory	NILM	NILM	Negative (NILM)	
P06522	2020	2020-Q1	2020-01	Pandemic	Cytology (Pap)	40-44	40	Not performed	Unsatisfactory	Unsatisfactory	Unsatisfactory	Unsatisfactory	
P06523	2020	2020-Q1	2020-01	Pandemic	Cytology (Pap)	45-49	45	Not performed	Satisfactory	NILM	NILM	Negative (NILM)	
P06524	2020	2020-Q1	2020-01	Pandemic	Cytology (Pap)	55-59	55	Not performed	Satisfactory	NILM	NILM	Negative (NILM)	
P06525	2020	2020-Q1	2020-01	Pandemic	Cytology (Pap)	40-44	43	Not performed	Satisfactory	NILM	NILM	Negative (NILM)	Other
P06526	2020	2020-Q1	2020-01	Pandemic	Cytology (Pap)	40-44	44	Not performed	Satisfactory	NILM	NILM	Negative (NILM)	
P06527	2020	2020-Q1	2020-01	Pandemic	Cytology (Pap)	45-49	47	Not performed	Satisfactory	NILM	NILM	Negative (NILM)	
P06528	2020	2020-Q1	2020-01	Pandemic	Cytology (Pap)	40-44	42	Not performed	Satisfactory	NILM	NILM	Negative (NILM)	
P06529	2020	2020-Q1	2020-01	Pandemic	Cytology (Pap)	35-39	39	Not performed	Satisfactory	Positive	LSIL	Low-grade / equivocal squamous	
P06530	2020	2020-Q1	2020-01	Pandemic	Cytology (Pap)	45-49	45	Not performed	Satisfactory	NILM	NILM	Negative (NILM)	
P06531	2020	2020-Q1	2020-01	Pandemic	Cytology (Pap)	40-44	40	Not performed	Satisfactory	NILM	NILM	Negative (NILM)	
P06532	2020	2020-Q1	2020-01	Pandemic	Cytology (Pap)	45-49	45	Not performed	Satisfactory	NILM	NILM	Negative (NILM)	
P06533	2020	2020-Q1	2020-01	Pandemic	Cytology (Pap)	45-49	46	Not performed	Satisfactory	NILM	NILM	Negative (NILM)	
P06534	2020	2020-Q1	2020-01	Pandemic	Cytology (Pap)	30-34	30	Not performed	Unsatisfactory	Unsatisfactory	Unsatisfactory	Unsatisfactory	
P06535	2020	2020-Q1	2020-01	Pandemic	Cytology (Pap)	30-34	34	Not performed	Satisfactory	NILM	NILM	Negative (NILM)	
P06536	2020	2020-Q1	2020-01	Pandemic	Cytology (Pap)	50-54	54	Not performed	Satisfactory	NILM	NILM	Negative (NILM)	
P06537	2020	2020-Q1	2020-01	Pandemic	Cytology (Pap)	40-44	41	Not performed	Satisfactory	NILM	NILM	Negative (NILM)	
P06538	2020	2020-Q1	2020-01	Pandemic	Cytology (Pap)	30-34	32	Not performed	Satisfactory	Positive	ASC-US	Low-grade / equivocal squamous	
P06539	2020	2020-Q1	2020-01	Pandemic	Cytology (Pap)	60-64	64	Not performed	Satisfactory	NILM	NILM	Negative (NILM)	Gardnerella
P06540	2020	2020-Q1	2020-01	Pandemic	Cytology (Pap)	50-54	54	Not performed	Satisfactory	NILM	NILM	Negative (NILM)	
P06541	2020	2020-Q1	2020-01	Pandemic	Cytology (Pap)	50-54	52	Not performed	Satisfactory	NILM	NILM	Negative (NILM)	
P06542	2020	2020-Q1	2020-01	Pandemic	Cytology (Pap)	35-39	38	Not performed	Satisfactory	NILM	NILM	Negative (NILM)	
P06543	2020	2020-Q1	2020-01	Pandemic	Cytology (Pap)	40-44	42	Not performed	Satisfactory	NILM	NILM	Negative (NILM)	
P06544	2020	2020-Q1	2020-01	Pandemic	Cytology (Pap)	35-39	37	Not performed	Unsatisfactory	Unsatisfactory	Unsatisfactory	Unsatisfactory	
P06545	2020	2020-Q1	2020-01	Pandemic	Cytology (Pap)	45-49	48	Not performed	Satisfactory	Positive	ASC-US	Low-grade / equivocal squamous	
P06546	2020	2020-Q1	2020-01	Pandemic	Cytology (Pap)	35-39	39	Not performed	Satisfactory	NILM	NILM	Negative (NILM)	
P06547	2020	2020-Q1	2020-01	Pandemic	Cytology (Pap)	45-49	49	Not performed	Satisfactory	NILM	NILM	Negative (NILM)	
P06548	2020	2020-Q1	2020-01	Pandemic	Cytology (Pap)	50-54	50	Not performed	Satisfactory	NILM	NILM	Negative (NILM)	Candida
P06549	2020	2020-Q1	2020-01	Pandemic	Cytology (Pap)	40-44	43	Not performed	Satisfactory	NILM	NILM	Negative (NILM)	
P06550	2020	2020-Q1	2020-01	Pandemic	Cytology (Pap)	45-49	45	Not performed	Satisfactory	NILM	NILM	Negative (NILM)	
P06551	2020	2020-Q1	2020-01	Pandemic	Cytology (Pap)	50-54	53	Not performed	Satisfactory	Positive	HSIL	High-grade squamous	
P06552	2020	2020-Q1	2020-01	Pandemic	Cytology (Pap)	30-34	31	Not performed	Satisfactory	NILM	NILM	Negative (NILM)	
P06553	2020	2020-Q1	2020-01	Pandemic	Cytology (Pap)	30-34	30	Not performed	Satisfactory	NILM	NILM	Negative (NILM)	
P06554	2020	2020-Q1	2020-01	Pandemic	Cytology (Pap)	35-39	38	Not performed	Satisfactory	NILM	NILM	Negative (NILM)	
P06555	2020	2020-Q1	2020-01	Pandemic	Cytology (Pap)	45-49	45	Not performed	Satisfactory	Positive	LSIL	Low-grade / equivocal squamous	
P06556	2020	2020-Q1	2020-01	Pandemic	Cytology (Pap)	55-59	58	Not performed	Satisfactory	NILM	NILM	Negative (NILM)	
P06557	2020	2020-Q1	2020-01	Pandemic	Cytology (Pap)	55-59	57	Not performed	Satisfactory	NILM	NILM	Negative (NILM)	
P06558	2020	2020-Q1	2020-01	Pandemic	Cytology (Pap)	40-44	41	Not performed	Satisfactory	NILM	NILM	Negative (NILM)	

patient_id	year	quarter	month	period	primary_test	age_group	age_years	hpv_result	adequacy	result	bethesda	grade	infection
P06559	2020	2020-Q1	2020-01	Pandemic	Cytology (Pap)	55-59	59	Not performed	Satisfactory	NILM	NILM	Negative (NILM)	
P06560	2020	2020-Q1	2020-01	Pandemic	Cytology (Pap)	35-39	36	Not performed	Unsatisfactory	Unsatisfactory	Unsatisfactory	Unsatisfactory	
P06561	2020	2020-Q1	2020-01	Pandemic	Cytology (Pap)	50-54	50	Not performed	Satisfactory	NILM	NILM	Negative (NILM)	
P06562	2020	2020-Q1	2020-01	Pandemic	Cytology (Pap)	25-29	28	Not performed	Satisfactory	NILM	NILM	Negative (NILM)	
P06563	2020	2020-Q1	2020-01	Pandemic	Cytology (Pap)	50-54	54	Not performed	Satisfactory	NILM	NILM	Negative (NILM)	
P06564	2020	2020-Q1	2020-01	Pandemic	Cytology (Pap)	45-49	48	Not performed	Satisfactory	Positive	LSIL	Low-grade / equivocal squamous	
P06565	2020	2020-Q1	2020-01	Pandemic	Cytology (Pap)	30-34	33	Not performed	Satisfactory	Positive	LSIL	Low-grade / equivocal squamous	
P06566	2020	2020-Q1	2020-01	Pandemic	Cytology (Pap)	25-29	28	Not performed	Satisfactory	Positive	LSIL	Low-grade / equivocal squamous	
P06567	2020	2020-Q1	2020-01	Pandemic	Cytology (Pap)	35-39	39	Not performed	Satisfactory	NILM	NILM	Negative (NILM)	
P06568	2020	2020-Q1	2020-01	Pandemic	Cytology (Pap)	50-54	54	Not performed	Satisfactory	NILM	NILM	Negative (NILM)	
P06569	2020	2020-Q1	2020-01	Pandemic	Cytology (Pap)	50-54	54	Not performed	Satisfactory	NILM	NILM	Negative (NILM)	
P06570	2020	2020-Q1	2020-01	Pandemic	Cytology (Pap)	30-34	34	Not performed	Satisfactory	Positive	ASC-H	High-grade squamous	Trichomonas
P06571	2020	2020-Q1	2020-01	Pandemic	Cytology (Pap)	40-44	40	Not performed	Satisfactory	NILM	NILM	Negative (NILM)	
P06572	2020	2020-Q1	2020-01	Pandemic	Cytology (Pap)	30-34	31	Not performed	Satisfactory	NILM	NILM	Negative (NILM)	
P06573	2020	2020-Q1	2020-01	Pandemic	Cytology (Pap)	55-59	56	Not performed	Satisfactory	NILM	NILM	Negative (NILM)	
P06574	2020	2020-Q1	2020-01	Pandemic	Cytology (Pap)	45-49	49	Not performed	Satisfactory	NILM	NILM	Negative (NILM)	Candida
P06575	2020	2020-Q1	2020-01	Pandemic	Cytology (Pap)	45-49	45	Not performed	Satisfactory	Positive	HSIL	High-grade squamous	
P06576	2020	2020-Q1	2020-01	Pandemic	Cytology (Pap)	45-49	45	Not performed	Satisfactory	NILM	NILM	Negative (NILM)	
P06577	2020	2020-Q1	2020-01	Pandemic	Cytology (Pap)	25-29	26	Not performed	Satisfactory	NILM	NILM	Negative (NILM)	
P06578	2020	2020-Q1	2020-01	Pandemic	Cytology (Pap)	35-39	35	Not performed	Satisfactory	Positive	ASC-US	Low-grade / equivocal squamous	
P06579	2020	2020-Q1	2020-01	Pandemic	Cytology (Pap)	45-49	45	Not performed	Satisfactory	NILM	NILM	Negative (NILM)	
P06580	2020	2020-Q1	2020-01	Pandemic	Cytology (Pap)	50-54	52	Not performed	Satisfactory	NILM	NILM	Negative (NILM)	
P06581	2020	2020-Q1	2020-01	Pandemic	Cytology (Pap)	45-49	45	Not performed	Satisfactory	Positive	ASC-H	High-grade squamous	
P06582	2020	2020-Q1	2020-01	Pandemic	Cytology (Pap)	45-49	47	Not performed	Satisfactory	NILM	NILM	Negative (NILM)	
P06583	2020	2020-Q1	2020-01	Pandemic	Cytology (Pap)	40-44	41	Not performed	Satisfactory	NILM	NILM	Negative (NILM)	
P06584	2020	2020-Q1	2020-01	Pandemic	Cytology (Pap)	60-64	63	Not performed	Satisfactory	NILM	NILM	Negative (NILM)	
P06585	2020	2020-Q1	2020-01	Pandemic	Cytology (Pap)	45-49	48	Not performed	Satisfactory	NILM	NILM	Negative (NILM)	
P06586	2020	2020-Q1	2020-01	Pandemic	Cytology (Pap)	40-44	42	Not performed	Satisfactory	NILM	NILM	Negative (NILM)	
P06587	2020	2020-Q1	2020-01	Pandemic	Cytology (Pap)	30-34	31	Not performed	Satisfactory	Positive	ASC-US	Low-grade / equivocal squamous	
P06588	2020	2020-Q1	2020-01	Pandemic	Cytology (Pap)	30-34	33	Not performed	Satisfactory	NILM	NILM	Negative (NILM)	
P06589	2020	2020-Q1	2020-01	Pandemic	Cytology (Pap)	55-59	56	Not performed	Unsatisfactory	Unsatisfactory	Unsatisfactory	Unsatisfactory	
P06590	2020	2020-Q1	2020-01	Pandemic	Cytology (Pap)	30-34	34	Not performed	Satisfactory	NILM	NILM	Negative (NILM)	
P06591	2020	2020-Q1	2020-01	Pandemic	Cytology (Pap)	25-29	28	Not performed	Satisfactory	NILM	NILM	Negative (NILM)	
P06592	2020	2020-Q1	2020-01	Pandemic	Cytology (Pap)	45-49	48	Not performed	Satisfactory	NILM	NILM	Negative (NILM)	
P06593	2020	2020-Q1	2020-01	Pandemic	Cytology (Pap)	45-49	48	Not performed	Satisfactory	Positive	ASC-US	Low-grade / equivocal squamous	Other
P06594	2020	2020-Q1	2020-01	Pandemic	Cytology (Pap)	40-44	40	Not performed	Satisfactory	NILM	NILM	Negative (NILM)	
P06595	2020	2020-Q1	2020-02	Pandemic	Cytology (Pap)	45-49	46	Not performed	Satisfactory	NILM	NILM	Negative (NILM)	
P06596	2020	2020-Q1	2020-02	Pandemic	Cytology (Pap)	30-34	33	Not performed	Satisfactory	NILM	NILM	Negative (NILM)	
P06597	2020	2020-Q1	2020-02	Pandemic	Cytology (Pap)	35-39	37	Not performed	Satisfactory	NILM	NILM	Negative (NILM)	
P06598	2020	2020-Q1	2020-02	Pandemic	Cytology (Pap)	25-29	28	Not performed	Satisfactory	NILM	NILM	Negative (NILM)	
P06599	2020	2020-Q1	2020-02	Pandemic	Cytology (Pap)	55-59	59	Not performed	Satisfactory	NILM	NILM	Negative (NILM)	
P06600	2020	2020-Q1	2020-02	Pandemic	Cytology (Pap)	55-59	57	Not performed	Satisfactory	NILM	NILM	Negative (NILM)	
P06601	2020	2020-Q1	2020-02	Pandemic	Cytology (Pap)	55-59	56	Not performed	Unsatisfactory	Unsatisfactory	Unsatisfactory	Unsatisfactory	
P06602	2020	2020-Q1	2020-02	Pandemic	Cytology (Pap)	30-34	30	Not performed	Satisfactory	NILM	NILM	Negative (NILM)	
P06603	2020	2020-Q1	2020-02	Pandemic	Cytology (Pap)	45-49	47	Not performed	Satisfactory	NILM	NILM	Negative (NILM)	
P06604	2020	2020-Q1	2020-02	Pandemic	Cytology (Pap)	35-39	39	Not performed	Satisfactory	NILM	NILM	Negative (NILM)	
P06605	2020	2020-Q1	2020-02	Pandemic	Cytology (Pap)	50-54	54	Not performed	Satisfactory	NILM	NILM	Negative (NILM)	
P06606	2020	2020-Q1	2020-02	Pandemic	Cytology (Pap)	30-34	34	Not performed	Satisfactory	NILM	NILM	Negative (NILM)	
P06607	2020	2020-Q1	2020-02	Pandemic	Cytology (Pap)	45-49	49	Not performed	Satisfactory	NILM	NILM	Negative (NILM)	
P06608	2020	2020-Q1	2020-02	Pandemic	Cytology (Pap)	30-34	31	Not performed	Satisfactory	NILM	NILM	Negative (NILM)	
P06609	2020	2020-Q1	2020-02	Pandemic	Cytology (Pap)	40-44	42	Not performed	Satisfactory	NILM	NILM	Negative (NILM)	
P06610	2020	2020-Q1	2020-02	Pandemic	Cytology (Pap)	45-49	45	Not performed	Satisfactory	NILM	NILM	Negative (NILM)	
P06611	2020	2020-Q1	2020-02	Pandemic	Cytology (Pap)	30-34	32	Not performed	Satisfactory	NILM	NILM	Negative (NILM)	
P06612	2020	2020-Q1	2020-02	Pandemic	Cytology (Pap)	25-29	28	Not performed	Satisfactory	NILM	NILM	Negative (NILM)	
P06613	2020	2020-Q1	2020-02	Pandemic	Cytology (Pap)	50-54	52	Not performed	Satisfactory	NILM	NILM	Negative (NILM)	
P06614	2020	2020-Q1	2020-02	Pandemic	Cytology (Pap)	35-39	36	Not performed	Satisfactory	NILM	NILM	Negative (NILM)	
P06615	2020	2020-Q1	2020-02	Pandemic	Cytology (Pap)	30-34	31	Not performed	Satisfactory	NILM	NILM	Negative (NILM)	
P06616	2020	2020-Q1	2020-02	Pandemic	Cytology (Pap)	40-44	43	Not performed	Satisfactory	Positive	ASC-US	Low-grade / equivocal squamous	
P06617	2020	2020-Q1	2020-02	Pandemic	Cytology (Pap)	45-49	45	Not performed	Satisfactory	NILM	NILM	Negative (NILM)	
P06618	2020	2020-Q1	2020-02	Pandemic	Cytology (Pap)	30-34	32	Not performed	Unsatisfactory	Unsatisfactory	Unsatisfactory	Unsatisfactory	
P06619	2020	2020-Q1	2020-02	Pandemic	Cytology (Pap)	40-44	42	Not performed	Unsatisfactory	Unsatisfactory	Unsatisfactory	Unsatisfactory	Trichomonas
P06620	2020	2020-Q1	2020-02	Pandemic	Cytology (Pap)	45-49	49	Not performed	Satisfactory	NILM	NILM	Negative (NILM)	
P06621	2020	2020-Q1	2020-02	Pandemic	Cytology (Pap)	35-39	35	Not performed	Satisfactory	NILM	NILM	Negative (NILM)	
P06622	2020	2020-Q1	2020-02	Pandemic	Cytology (Pap)	25-29	25	Not performed	Satisfactory	Positive	LSIL	Low-grade / equivocal squamous	
P06623	2020	2020-Q1	2020-02	Pandemic	Cytology (Pap)	25-29	29	Not performed	Satisfactory	NILM	NILM	Negative (NILM)	
P06624	2020	2020-Q1	2020-02	Pandemic	Cytology (Pap)	50-54	50	Not performed	Satisfactory	NILM	NILM	Negative (NILM)	
P06625	2020	2020-Q1	2020-02	Pandemic	Cytology (Pap)	40-44	43	Not performed	Satisfactory	NILM	NILM	Negative (NILM)	
P06626	2020	2020-Q1	2020-02	Pandemic	Cytology (Pap)	40-44	40	Not performed	Satisfactory	NILM	NILM	Negative (NILM)	
P06627	2020	2020-Q1	2020-02	Pandemic	Cytology (Pap)	45-49	46	Not performed	Satisfactory	NILM	NILM	Negative (NILM)	
P06628	2020	2020-Q1	2020-02	Pandemic	Cytology (Pap)	40-44	42	Not performed	Satisfactory	NILM	NILM	Negative (NILM)	
P06629	2020	2020-Q1	2020-02	Pandemic	Cytology (Pap)	55-59	55	Not performed	Satisfactory	NILM	NILM	Negative (NILM)	
P06630	2020	2020-Q1	2020-02	Pandemic	Cytology (Pap)	40-44	41	Not performed	Satisfactory	NILM	NILM	Negative (NILM)	Trichomonas
P06631	2020	2020-Q1	2020-02	Pandemic	Cytology (Pap)	35-39	37	Not performed	Satisfactory	NILM	NILM	Negative (NILM)	
P06632	2020	2020-Q1	2020-02	Pandemic	Cytology (Pap)	30-34	31	Not performed	Satisfactory	NILM	NILM	Negative (NILM)	
P06633	2020	2020-Q1	2020-02	Pandemic	Cytology (Pap)	40-44	44	Not performed	Satisfactory	NILM	NILM	Negative (NILM)	
P06634	2020	2020-Q1	2020-02	Pandemic	Cytology (Pap)	50-54	51	Not performed	Satisfactory	NILM	NILM	Negative (NILM)	
P06635	2020	2020-Q1	2020-02	Pandemic	Cytology (Pap)	35-39	36	Not performed	Satisfactory	NILM	NILM	Negative (NILM)	
P06636	2020	2020-Q1	2020-02	Pandemic	Cytology (Pap)	35-39	39	Not performed	Satisfactory	NILM	NILM	Negative (NILM)	
P06637	2020	2020-Q1	2020-02	Pandemic	Cytology (Pap)	50-54	53	Not performed	Satisfactory	NILM	NILM	Negative (NILM)	
P06638	2020	2020-Q1	2020-02	Pandemic	Cytology (Pap)	45-49	46	Not performed	Satisfactory	Positive	LSIL	Low-grade / equivocal squamous	

patient_id	year	quarter	month	period	primary_test	age_group	age_years	hpv_result	adequacy	result	bethesda	grade	infection
P06639	2020	2020-Q1	2020-02	Pandemic	Cytology (Pap)	45-49	46	Not performed	Satisfactory	Positive	ASC-US	Low-grade / equivocal squamous	
P06640	2020	2020-Q1	2020-02	Pandemic	Cytology (Pap)	45-49	45	Not performed	Satisfactory	NILM	NILM	Negative (NILM)	
P06641	2020	2020-Q1	2020-02	Pandemic	Cytology (Pap)	30-34	30	Not performed	Satisfactory	NILM	NILM	Negative (NILM)	Other
P06642	2020	2020-Q1	2020-02	Pandemic	Cytology (Pap)	55-59	55	Not performed	Satisfactory	Positive	ASC-H	High-grade squamous	Candida
P06643	2020	2020-Q1	2020-02	Pandemic	Cytology (Pap)	35-39	38	Not performed	Satisfactory	NILM	NILM	Negative (NILM)	
P06644	2020	2020-Q1	2020-02	Pandemic	Cytology (Pap)	55-59	57	Not performed	Satisfactory	NILM	NILM	Negative (NILM)	
P06645	2020	2020-Q1	2020-02	Pandemic	Cytology (Pap)	55-59	56	Not performed	Satisfactory	NILM	NILM	Negative (NILM)	
P06646	2020	2020-Q1	2020-02	Pandemic	Cytology (Pap)	40-44	42	Not performed	Satisfactory	NILM	NILM	Negative (NILM)	
P06647	2020	2020-Q1	2020-02	Pandemic	Cytology (Pap)	35-39	35	Not performed	Satisfactory	NILM	NILM	Negative (NILM)	
P06648	2020	2020-Q1	2020-02	Pandemic	Cytology (Pap)	60-64	60	Not performed	Satisfactory	NILM	NILM	Negative (NILM)	
P06649	2020	2020-Q1	2020-02	Pandemic	Cytology (Pap)	60-64	62	Not performed	Satisfactory	NILM	NILM	Negative (NILM)	
P06650	2020	2020-Q1	2020-02	Pandemic	Cytology (Pap)	55-59	59	Not performed	Satisfactory	NILM	NILM	Negative (NILM)	
P06651	2020	2020-Q1	2020-02	Pandemic	Cytology (Pap)	35-39	36	Not performed	Satisfactory	NILM	NILM	Negative (NILM)	
P06652	2020	2020-Q1	2020-02	Pandemic	Cytology (Pap)	25-29	25	Not performed	Satisfactory	NILM	NILM	Negative (NILM)	
P06653	2020	2020-Q1	2020-02	Pandemic	Cytology (Pap)	55-59	56	Not performed	Unsatisfactory	Unsatisfactory	Unsatisfactory	Unsatisfactory	
P06654	2020	2020-Q1	2020-02	Pandemic	Cytology (Pap)	40-44	41	Not performed	Satisfactory	NILM	NILM	Negative (NILM)	
P06655	2020	2020-Q1	2020-02	Pandemic	Cytology (Pap)	30-34	30	Not performed	Satisfactory	NILM	NILM	Negative (NILM)	
P06656	2020	2020-Q1	2020-02	Pandemic	Cytology (Pap)	55-59	56	Not performed	Satisfactory	Positive	ASC-H	High-grade squamous	
P06657	2020	2020-Q1	2020-02	Pandemic	Cytology (Pap)	40-44	44	Not performed	Satisfactory	Positive	LSIL	Low-grade / equivocal squamous	
P06658	2020	2020-Q1	2020-02	Pandemic	Cytology (Pap)	45-49	46	Not performed	Satisfactory	NILM	NILM	Negative (NILM)	
P06659	2020	2020-Q1	2020-02	Pandemic	Cytology (Pap)	40-44	40	Not performed	Unsatisfactory	Unsatisfactory	Unsatisfactory	Unsatisfactory	Other
P06660	2020	2020-Q1	2020-02	Pandemic	Cytology (Pap)	45-49	49	Not performed	Satisfactory	NILM	NILM	Negative (NILM)	
P06661	2020	2020-Q1	2020-02	Pandemic	Cytology (Pap)	30-34	32	Not performed	Unsatisfactory	Unsatisfactory	Unsatisfactory	Unsatisfactory	
P06662	2020	2020-Q1	2020-02	Pandemic	Cytology (Pap)	30-34	34	Not performed	Satisfactory	NILM	NILM	Negative (NILM)	
P06663	2020	2020-Q1	2020-02	Pandemic	Cytology (Pap)	55-59	56	Not performed	Satisfactory	NILM	NILM	Negative (NILM)	
P06664	2020	2020-Q1	2020-02	Pandemic	Cytology (Pap)	30-34	34	Not performed	Satisfactory	NILM	NILM	Negative (NILM)	
P06665	2020	2020-Q1	2020-02	Pandemic	Cytology (Pap)	35-39	39	Not performed	Satisfactory	NILM	NILM	Negative (NILM)	
P06666	2020	2020-Q1	2020-02	Pandemic	Cytology (Pap)	40-44	41	Not performed	Satisfactory	NILM	NILM	Negative (NILM)	
P06667	2020	2020-Q1	2020-02	Pandemic	Cytology (Pap)	50-54	51	Not performed	Satisfactory	NILM	NILM	Negative (NILM)	
P06668	2020	2020-Q1	2020-02	Pandemic	Cytology (Pap)	40-44	42	Not performed	Satisfactory	NILM	NILM	Negative (NILM)	
P06669	2020	2020-Q1	2020-02	Pandemic	Cytology (Pap)	40-44	41	Not performed	Satisfactory	NILM	NILM	Negative (NILM)	
P06670	2020	2020-Q1	2020-02	Pandemic	Cytology (Pap)	40-44	44	Not performed	Satisfactory	Positive	LSIL	Low-grade / equivocal squamous	
P06671	2020	2020-Q1	2020-02	Pandemic	Cytology (Pap)	50-54	51	Not performed	Satisfactory	Positive	ASC-US	Low-grade / equivocal squamous	
P06672	2020	2020-Q1	2020-02	Pandemic	Cytology (Pap)	40-44	44	Not performed	Satisfactory	NILM	NILM	Negative (NILM)	
P06673	2020	2020-Q1	2020-02	Pandemic	Cytology (Pap)	35-39	37	Not performed	Satisfactory	NILM	NILM	Negative (NILM)	
P06674	2020	2020-Q1	2020-02	Pandemic	Cytology (Pap)	45-49	45	Not performed	Satisfactory	NILM	NILM	Negative (NILM)	
P06675	2020	2020-Q1	2020-02	Pandemic	Cytology (Pap)	35-39	39	Not performed	Satisfactory	NILM	NILM	Negative (NILM)	
P06676	2020	2020-Q1	2020-02	Pandemic	Cytology (Pap)	40-44	41	Not performed	Satisfactory	NILM	NILM	Negative (NILM)	
P06677	2020	2020-Q1	2020-02	Pandemic	Cytology (Pap)	40-44	44	Not performed	Satisfactory	NILM	NILM	Negative (NILM)	
P06678	2020	2020-Q1	2020-02	Pandemic	Cytology (Pap)	35-39	39	Not performed	Satisfactory	Positive	ASC-H	High-grade squamous	
P06679	2020	2020-Q1	2020-02	Pandemic	Cytology (Pap)	40-44	40	Not performed	Satisfactory	NILM	NILM	Negative (NILM)	
P06680	2020	2020-Q1	2020-02	Pandemic	Cytology (Pap)	30-34	31	Not performed	Satisfactory	NILM	NILM	Negative (NILM)	
P06681	2020	2020-Q1	2020-02	Pandemic	Cytology (Pap)	45-49	45	Not performed	Satisfactory	NILM	NILM	Negative (NILM)	
P06682	2020	2020-Q1	2020-02	Pandemic	Cytology (Pap)	40-44	40	Not performed	Satisfactory	NILM	NILM	Negative (NILM)	
P06683	2020	2020-Q1	2020-02	Pandemic	Cytology (Pap)	50-54	50	Not performed	Satisfactory	NILM	NILM	Negative (NILM)	
P06684	2020	2020-Q1	2020-02	Pandemic	Cytology (Pap)	45-49	49	Not performed	Satisfactory	NILM	NILM	Negative (NILM)	
P06685	2020	2020-Q1	2020-02	Pandemic	Cytology (Pap)	35-39	39	Not performed	Satisfactory	NILM	NILM	Negative (NILM)	
P06686	2020	2020-Q1	2020-02	Pandemic	Cytology (Pap)	45-49	46	Not performed	Satisfactory	NILM	NILM	Negative (NILM)	
P06687	2020	2020-Q1	2020-02	Pandemic	Cytology (Pap)	30-34	32	Not performed	Satisfactory	Positive	LSIL	Low-grade / equivocal squamous	
P06688	2020	2020-Q1	2020-02	Pandemic	Cytology (Pap)	60-64	63	Not performed	Satisfactory	NILM	NILM	Negative (NILM)	
P06689	2020	2020-Q1	2020-02	Pandemic	Cytology (Pap)	40-44	40	Not performed	Satisfactory	Positive	HSIL	High-grade squamous	
P06690	2020	2020-Q1	2020-02	Pandemic	Cytology (Pap)	45-49	45	Not performed	Satisfactory	Positive	ASC-US	Low-grade / equivocal squamous	
P06691	2020	2020-Q1	2020-02	Pandemic	Cytology (Pap)	35-39	39	Not performed	Unsatisfactory	Unsatisfactory	Unsatisfactory	Unsatisfactory	
P06692	2020	2020-Q1	2020-02	Pandemic	Cytology (Pap)	30-34	31	Not performed	Satisfactory	Positive	LSIL	Low-grade / equivocal squamous	
P06693	2020	2020-Q1	2020-02	Pandemic	Cytology (Pap)	35-39	36	Not performed	Satisfactory	Positive	HSIL	High-grade squamous	
P06694	2020	2020-Q1	2020-02	Pandemic	Cytology (Pap)	45-49	47	Not performed	Satisfactory	NILM	NILM	Negative (NILM)	
P06695	2020	2020-Q1	2020-02	Pandemic	Cytology (Pap)	45-49	49	Not performed	Satisfactory	Positive	ASC-US	Low-grade / equivocal squamous	
P06696	2020	2020-Q1	2020-02	Pandemic	Cytology (Pap)	60-64	62	Not performed	Satisfactory	NILM	NILM	Negative (NILM)	
P06697	2020	2020-Q1	2020-02	Pandemic	Cytology (Pap)	55-59	58	Not performed	Satisfactory	NILM	NILM	Negative (NILM)	
P06698	2020	2020-Q1	2020-02	Pandemic	Cytology (Pap)	55-59	56	Not performed	Satisfactory	NILM	NILM	Negative (NILM)	
P06699	2020	2020-Q1	2020-02	Pandemic	Cytology (Pap)	50-54	51	Not performed	Satisfactory	NILM	NILM	Negative (NILM)	
P06700	2020	2020-Q1	2020-02	Pandemic	Cytology (Pap)	40-44	41	Not performed	Satisfactory	NILM	NILM	Negative (NILM)	
P06701	2020	2020-Q1	2020-02	Pandemic	Cytology (Pap)	55-59	56	Not performed	Satisfactory	NILM	NILM	Negative (NILM)	
P06702	2020	2020-Q1	2020-02	Pandemic	Cytology (Pap)	35-39	37	Not performed	Satisfactory	NILM	NILM	Negative (NILM)	Candida
P06703	2020	2020-Q1	2020-02	Pandemic	Cytology (Pap)	25-29	28	Not performed	Satisfactory	NILM	NILM	Negative (NILM)	
P06704	2020	2020-Q1	2020-02	Pandemic	Cytology (Pap)	40-44	40	Not performed	Satisfactory	NILM	NILM	Negative (NILM)	
P06705	2020	2020-Q1	2020-02	Pandemic	Cytology (Pap)	45-49	49	Not performed	Satisfactory	NILM	NILM	Negative (NILM)	
P06706	2020	2020-Q1	2020-02	Pandemic	Cytology (Pap)	50-54	51	Not performed	Satisfactory	NILM	NILM	Negative (NILM)	
P06707	2020	2020-Q1	2020-02	Pandemic	Cytology (Pap)	45-49	46	Not performed	Satisfactory	NILM	NILM	Negative (NILM)	
P06708	2020	2020-Q1	2020-02	Pandemic	Cytology (Pap)	50-54	51	Not performed	Satisfactory	NILM	NILM	Negative (NILM)	
P06709	2020	2020-Q1	2020-02	Pandemic	Cytology (Pap)	40-44	40	Not performed	Satisfactory	NILM	NILM	Negative (NILM)	
P06710	2020	2020-Q1	2020-02	Pandemic	Cytology (Pap)	25-29	29	Not performed	Satisfactory	NILM	NILM	Negative (NILM)	Trichomonas
P06711	2020	2020-Q1	2020-02	Pandemic	Cytology (Pap)	40-44	41	Not performed	Satisfactory	NILM	NILM	Negative (NILM)	
P06712	2020	2020-Q1	2020-02	Pandemic	Cytology (Pap)	60-64	62	Not performed	Satisfactory	NILM	NILM	Negative (NILM)	
P06713	2020	2020-Q1	2020-02	Pandemic	Cytology (Pap)	40-44	40	Not performed	Satisfactory	Positive	HSIL	High-grade squamous	
P06714	2020	2020-Q1	2020-02	Pandemic	Cytology (Pap)	30-34	34	Not performed	Satisfactory	Positive	LSIL	Low-grade / equivocal squamous	
P06715	2020	2020-Q1	2020-02	Pandemic	Cytology (Pap)	35-39	38	Not performed	Satisfactory	NILM	NILM	Negative (NILM)	
P06716	2020	2020-Q1	2020-02	Pandemic	Cytology (Pap)	35-39	36	Not performed	Satisfactory	NILM	NILM	Negative (NILM)	
P06717	2020	2020-Q1	2020-02	Pandemic	Cytology (Pap)	45-49	45	Not performed	Satisfactory	NILM	NILM	Negative (NILM)	
P06718	2020	2020-Q1	2020-02	Pandemic	Cytology (Pap)	45-49	45	Not performed	Satisfactory	NILM	NILM	Negative (NILM)	

patient_id	year	quarter	month	period	primary_test	age_group	age_years	hpv_result	adequacy	result	bethesda	grade	infection
P06719	2020	2020-Q1	2020-02	Pandemic	Cytology (Pap)	60-64	61	Not performed	Satisfactory	NILM	NILM	Negative (NILM)	
P06720	2020	2020-Q1	2020-02	Pandemic	Cytology (Pap)	45-49	47	Not performed	Satisfactory	NILM	NILM	Negative (NILM)	
P06721	2020	2020-Q1	2020-02	Pandemic	Cytology (Pap)	40-44	43	Not performed	Satisfactory	NILM	NILM	Negative (NILM)	
P06722	2020	2020-Q1	2020-02	Pandemic	Cytology (Pap)	50-54	51	Not performed	Unsatisfactory	Unsatisfactory	Unsatisfactory	Unsatisfactory	
P06723	2020	2020-Q1	2020-02	Pandemic	Cytology (Pap)	35-39	37	Not performed	Satisfactory	NILM	NILM	Negative (NILM)	
P06724	2020	2020-Q1	2020-02	Pandemic	Cytology (Pap)	35-39	38	Not performed	Satisfactory	NILM	NILM	Negative (NILM)	
P06725	2020	2020-Q1	2020-02	Pandemic	Cytology (Pap)	40-44	40	Not performed	Satisfactory	NILM	NILM	Negative (NILM)	
P06726	2020	2020-Q1	2020-02	Pandemic	Cytology (Pap)	55-59	58	Not performed	Satisfactory	NILM	NILM	Negative (NILM)	
P06727	2020	2020-Q1	2020-02	Pandemic	Cytology (Pap)	45-49	49	Not performed	Unsatisfactory	Unsatisfactory	Unsatisfactory	Unsatisfactory	Candida
P06728	2020	2020-Q1	2020-02	Pandemic	Cytology (Pap)	40-44	43	Not performed	Satisfactory	NILM	NILM	Negative (NILM)	
P06729	2020	2020-Q1	2020-02	Pandemic	Cytology (Pap)	45-49	47	Not performed	Satisfactory	NILM	NILM	Negative (NILM)	
P06730	2020	2020-Q1	2020-02	Pandemic	Cytology (Pap)	45-49	48	Not performed	Satisfactory	NILM	NILM	Negative (NILM)	
P06731	2020	2020-Q1	2020-02	Pandemic	Cytology (Pap)	50-54	51	Not performed	Satisfactory	Positive	LSIL	Low-grade / equivocal squamous	
P06732	2020	2020-Q1	2020-02	Pandemic	Cytology (Pap)	40-44	43	Not performed	Satisfactory	NILM	NILM	Negative (NILM)	
P06733	2020	2020-Q1	2020-02	Pandemic	Cytology (Pap)	50-54	54	Not performed	Satisfactory	NILM	NILM	Negative (NILM)	
P06734	2020	2020-Q1	2020-02	Pandemic	Cytology (Pap)	45-49	47	Not performed	Satisfactory	NILM	NILM	Negative (NILM)	
P06735	2020	2020-Q1	2020-02	Pandemic	Cytology (Pap)	30-34	33	Not performed	Satisfactory	NILM	NILM	Negative (NILM)	
P06736	2020	2020-Q1	2020-02	Pandemic	Cytology (Pap)	30-34	30	Not performed	Satisfactory	NILM	NILM	Negative (NILM)	Gardnerella
P06737	2020	2020-Q1	2020-02	Pandemic	Cytology (Pap)	35-39	39	Not performed	Satisfactory	NILM	NILM	Negative (NILM)	
P06738	2020	2020-Q1	2020-02	Pandemic	Cytology (Pap)	45-49	49	Not performed	Satisfactory	NILM	NILM	Negative (NILM)	
P06739	2020	2020-Q1	2020-02	Pandemic	Cytology (Pap)	45-49	45	Not performed	Satisfactory	NILM	NILM	Negative (NILM)	
P06740	2020	2020-Q1	2020-02	Pandemic	Cytology (Pap)	50-54	54	Not performed	Satisfactory	NILM	NILM	Negative (NILM)	
P06741	2020	2020-Q1	2020-02	Pandemic	Cytology (Pap)	45-49	46	Not performed	Satisfactory	NILM	NILM	Negative (NILM)	
P06742	2020	2020-Q1	2020-02	Pandemic	Cytology (Pap)	45-49	49	Not performed	Satisfactory	NILM	NILM	Negative (NILM)	
P06743	2020	2020-Q1	2020-02	Pandemic	Cytology (Pap)	55-59	59	Not performed	Satisfactory	NILM	NILM	Negative (NILM)	
P06744	2020	2020-Q1	2020-02	Pandemic	Cytology (Pap)	55-59	55	Not performed	Satisfactory	NILM	NILM	Negative (NILM)	
P06745	2020	2020-Q1	2020-02	Pandemic	Cytology (Pap)	60-64	64	Not performed	Satisfactory	Positive	AGC	Glandular	
P06746	2020	2020-Q1	2020-02	Pandemic	Cytology (Pap)	50-54	54	Not performed	Satisfactory	NILM	NILM	Negative (NILM)	
P06747	2020	2020-Q1	2020-02	Pandemic	Cytology (Pap)	50-54	51	Not performed	Satisfactory	Positive	ASC-US	Low-grade / equivocal squamous	
P06748	2020	2020-Q1	2020-02	Pandemic	Cytology (Pap)	50-54	50	Not performed	Satisfactory	NILM	NILM	Negative (NILM)	
P06749	2020	2020-Q1	2020-02	Pandemic	Cytology (Pap)	50-54	53	Not performed	Satisfactory	Positive	ASC-US	Low-grade / equivocal squamous	
P06750	2020	2020-Q1	2020-02	Pandemic	Cytology (Pap)	30-34	34	Not performed	Satisfactory	NILM	NILM	Negative (NILM)	
P06751	2020	2020-Q1	2020-02	Pandemic	Cytology (Pap)	45-49	45	Not performed	Satisfactory	NILM	NILM	Negative (NILM)	Other
P06752	2020	2020-Q1	2020-02	Pandemic	Cytology (Pap)	60-64	63	Not performed	Satisfactory	NILM	NILM	Negative (NILM)	
P06753	2020	2020-Q1	2020-02	Pandemic	Cytology (Pap)	30-34	34	Not performed	Satisfactory	NILM	NILM	Negative (NILM)	
P06754	2020	2020-Q1	2020-02	Pandemic	Cytology (Pap)	40-44	44	Not performed	Unsatisfactory	Unsatisfactory	Unsatisfactory	Unsatisfactory	
P06755	2020	2020-Q1	2020-02	Pandemic	Cytology (Pap)	40-44	44	Not performed	Satisfactory	Positive	ASC-US	Low-grade / equivocal squamous	
P06756	2020	2020-Q1	2020-02	Pandemic	Cytology (Pap)	40-44	44	Not performed	Satisfactory	NILM	NILM	Negative (NILM)	
P06757	2020	2020-Q1	2020-02	Pandemic	Cytology (Pap)	25-29	28	Not performed	Satisfactory	NILM	NILM	Negative (NILM)	
P06758	2020	2020-Q1	2020-02	Pandemic	Cytology (Pap)	60-64	60	Not performed	Satisfactory	NILM	NILM	Negative (NILM)	
P06759	2020	2020-Q1	2020-02	Pandemic	Cytology (Pap)	50-54	50	Not performed	Satisfactory	NILM	NILM	Negative (NILM)	
P06760	2020	2020-Q1	2020-02	Pandemic	Cytology (Pap)	45-49	47	Not performed	Satisfactory	NILM	NILM	Negative (NILM)	Trichomonas
P06761	2020	2020-Q1	2020-02	Pandemic	Cytology (Pap)	40-44	41	Not performed	Satisfactory	NILM	NILM	Negative (NILM)	
P06762	2020	2020-Q1	2020-02	Pandemic	Cytology (Pap)	55-59	56	Not performed	Satisfactory	NILM	NILM	Negative (NILM)	
P06763	2020	2020-Q1	2020-02	Pandemic	Cytology (Pap)	60-64	62	Not performed	Unsatisfactory	Unsatisfactory	Unsatisfactory	Unsatisfactory	
P06764	2020	2020-Q1	2020-02	Pandemic	Cytology (Pap)	30-34	33	Not performed	Satisfactory	NILM	NILM	Negative (NILM)	
P06765	2020	2020-Q1	2020-02	Pandemic	Cytology (Pap)	40-44	43	Not performed	Satisfactory	NILM	NILM	Negative (NILM)	
P06766	2020	2020-Q1	2020-02	Pandemic	Cytology (Pap)	40-44	42	Not performed	Satisfactory	NILM	NILM	Negative (NILM)	
P06767	2020	2020-Q1	2020-02	Pandemic	Cytology (Pap)	50-54	51	Not performed	Satisfactory	NILM	NILM	Negative (NILM)	
P06768	2020	2020-Q1	2020-02	Pandemic	Cytology (Pap)	45-49	47	Not performed	Satisfactory	NILM	NILM	Negative (NILM)	
P06769	2020	2020-Q1	2020-02	Pandemic	Cytology (Pap)	35-39	35	Not performed	Satisfactory	NILM	NILM	Negative (NILM)	
P06770	2020	2020-Q1	2020-02	Pandemic	Cytology (Pap)	55-59	55	Not performed	Satisfactory	NILM	NILM	Negative (NILM)	
P06771	2020	2020-Q1	2020-02	Pandemic	Cytology (Pap)	40-44	42	Not performed	Satisfactory	NILM	NILM	Negative (NILM)	
P06772	2020	2020-Q1	2020-02	Pandemic	Cytology (Pap)	40-44	40	Not performed	Satisfactory	NILM	NILM	Negative (NILM)	
P06773	2020	2020-Q1	2020-02	Pandemic	Cytology (Pap)	35-39	35	Not performed	Satisfactory	NILM	NILM	Negative (NILM)	
P06774	2020	2020-Q1	2020-02	Pandemic	Cytology (Pap)	25-29	25	Not performed	Satisfactory	NILM	NILM	Negative (NILM)	
P06775	2020	2020-Q1	2020-02	Pandemic	Cytology (Pap)	40-44	44	Not performed	Satisfactory	NILM	NILM	Negative (NILM)	
P06776	2020	2020-Q1	2020-02	Pandemic	Cytology (Pap)	40-44	44	Not performed	Satisfactory	NILM	NILM	Negative (NILM)	
P06777	2020	2020-Q1	2020-02	Pandemic	Cytology (Pap)	40-44	44	Not performed	Satisfactory	NILM	NILM	Negative (NILM)	
P06778	2020	2020-Q1	2020-02	Pandemic	Cytology (Pap)	40-44	42	Not performed	Satisfactory	NILM	NILM	Negative (NILM)	
P06779	2020	2020-Q1	2020-02	Pandemic	Cytology (Pap)	35-39	35	Not performed	Satisfactory	NILM	NILM	Negative (NILM)	
P06780	2020	2020-Q1	2020-02	Pandemic	Cytology (Pap)	30-34	34	Not performed	Satisfactory	NILM	NILM	Negative (NILM)	
P06781	2020	2020-Q1	2020-02	Pandemic	Cytology (Pap)	60-64	62	Not performed	Satisfactory	NILM	NILM	Negative (NILM)	
P06782	2020	2020-Q1	2020-02	Pandemic	Cytology (Pap)	40-44	43	Not performed	Satisfactory	NILM	NILM	Negative (NILM)	
P06783	2020	2020-Q1	2020-02	Pandemic	Cytology (Pap)	45-49	46	Not performed	Satisfactory	NILM	NILM	Negative (NILM)	
P06784	2020	2020-Q1	2020-02	Pandemic	Cytology (Pap)	40-44	42	Not performed	Satisfactory	NILM	NILM	Negative (NILM)	
P06785	2020	2020-Q1	2020-02	Pandemic	Cytology (Pap)	30-34	32	Not performed	Satisfactory	NILM	NILM	Negative (NILM)	
P06786	2020	2020-Q1	2020-02	Pandemic	Cytology (Pap)	45-49	47	Not performed	Satisfactory	NILM	NILM	Negative (NILM)	
P06787	2020	2020-Q1	2020-02	Pandemic	Cytology (Pap)	40-44	40	Not performed	Satisfactory	Positive	ASC-US	Low-grade / equivocal squamous	
P06788	2020	2020-Q1	2020-02	Pandemic	Cytology (Pap)	30-34	32	Not performed	Satisfactory	NILM	NILM	Negative (NILM)	
P06789	2020	2020-Q1	2020-02	Pandemic	Cytology (Pap)	45-49	45	Not performed	Satisfactory	Positive	HSIL	High-grade squamous	
P06790	2020	2020-Q1	2020-02	Pandemic	Cytology (Pap)	35-39	39	Not performed	Satisfactory	NILM	NILM	Negative (NILM)	
P06791	2020	2020-Q1	2020-02	Pandemic	Cytology (Pap)	50-54	53	Not performed	Satisfactory	NILM	NILM	Negative (NILM)	
P06792	2020	2020-Q1	2020-02	Pandemic	Cytology (Pap)	35-39	35	Not performed	Satisfactory	Positive	LSIL	Low-grade / equivocal squamous	
P06793	2020	2020-Q1	2020-02	Pandemic	Cytology (Pap)	35-39	35	Not performed	Satisfactory	NILM	NILM	Negative (NILM)	
P06794	2020	2020-Q1	2020-02	Pandemic	Cytology (Pap)	40-44	43	Not performed	Satisfactory	Positive	ASC-US	Low-grade / equivocal squamous	
P06795	2020	2020-Q1	2020-02	Pandemic	Cytology (Pap)	45-49	49	Not performed	Satisfactory	NILM	NILM	Negative (NILM)	
P06796	2020	2020-Q1	2020-02	Pandemic	Cytology (Pap)	50-54	50	Not performed	Satisfactory	NILM	NILM	Negative (NILM)	
P06797	2020	2020-Q1	2020-02	Pandemic	Cytology (Pap)	35-39	36	Not performed	Satisfactory	NILM	NILM	Negative (NILM)	Candida
P06798	2020	2020-Q1	2020-02	Pandemic	Cytology (Pap)	55-59	59	Not performed	Satisfactory	NILM	NILM	Negative (NILM)	

patient_id	year	quarter	month	period	primary_test	age_group	age_years	hpv_result	adequacy	result	bethesda	grade	infection
P06799	2020	2020-Q1	2020-02	Pandemic	Cytology (Pap)	50-54	53	Not performed	Satisfactory	NILM	NILM	Negative (NILM)	
P06800	2020	2020-Q1	2020-02	Pandemic	Cytology (Pap)	35-39	37	Not performed	Satisfactory	NILM	NILM	Negative (NILM)	
P06801	2020	2020-Q1	2020-02	Pandemic	Cytology (Pap)	45-49	49	Not performed	Satisfactory	NILM	NILM	Negative (NILM)	
P06802	2020	2020-Q1	2020-02	Pandemic	Cytology (Pap)	50-54	52	Not performed	Satisfactory	NILM	NILM	Negative (NILM)	Candida
P06803	2020	2020-Q1	2020-02	Pandemic	Cytology (Pap)	35-39	38	Not performed	Satisfactory	NILM	NILM	Negative (NILM)	
P06804	2020	2020-Q1	2020-02	Pandemic	Cytology (Pap)	40-44	41	Not performed	Unsatisfactory	Unsatisfactory	Unsatisfactory	Unsatisfactory	
P06805	2020	2020-Q1	2020-02	Pandemic	Cytology (Pap)	35-39	35	Not performed	Satisfactory	NILM	NILM	Negative (NILM)	
P06806	2020	2020-Q1	2020-02	Pandemic	Cytology (Pap)	40-44	40	Not performed	Satisfactory	NILM	NILM	Negative (NILM)	Candida
P06807	2020	2020-Q1	2020-02	Pandemic	Cytology (Pap)	55-59	59	Not performed	Satisfactory	NILM	NILM	Negative (NILM)	
P06808	2020	2020-Q1	2020-02	Pandemic	Cytology (Pap)	45-49	45	Not performed	Satisfactory	NILM	NILM	Negative (NILM)	
P06809	2020	2020-Q1	2020-02	Pandemic	Cytology (Pap)	40-44	40	Not performed	Satisfactory	NILM	NILM	Negative (NILM)	
P06810	2020	2020-Q1	2020-02	Pandemic	Cytology (Pap)	25-29	27	Not performed	Satisfactory	NILM	NILM	Negative (NILM)	
P06811	2020	2020-Q1	2020-02	Pandemic	Cytology (Pap)	30-34	31	Not performed	Satisfactory	NILM	NILM	Negative (NILM)	
P06812	2020	2020-Q1	2020-02	Pandemic	Cytology (Pap)	40-44	41	Not performed	Satisfactory	NILM	NILM	Negative (NILM)	
P06813	2020	2020-Q1	2020-02	Pandemic	Cytology (Pap)	35-39	39	Not performed	Satisfactory	NILM	NILM	Negative (NILM)	
P06814	2020	2020-Q1	2020-02	Pandemic	Cytology (Pap)	30-34	31	Not performed	Satisfactory	NILM	NILM	Negative (NILM)	
P06815	2020	2020-Q1	2020-02	Pandemic	Cytology (Pap)	35-39	38	Not performed	Satisfactory	NILM	NILM	Negative (NILM)	
P06816	2020	2020-Q1	2020-02	Pandemic	Cytology (Pap)	45-49	46	Not performed	Satisfactory	NILM	NILM	Negative (NILM)	
P06817	2020	2020-Q1	2020-02	Pandemic	Cytology (Pap)	40-44	43	Not performed	Satisfactory	NILM	NILM	Negative (NILM)	
P06818	2020	2020-Q1	2020-02	Pandemic	Cytology (Pap)	30-34	32	Not performed	Satisfactory	NILM	NILM	Negative (NILM)	
P06819	2020	2020-Q1	2020-02	Pandemic	Cytology (Pap)	25-29	25	Not performed	Satisfactory	Positive	HSIL	High-grade squamous	
P06820	2020	2020-Q1	2020-02	Pandemic	Cytology (Pap)	45-49	48	Not performed	Satisfactory	Positive	ASC-US	Low-grade / equivocal squamous	
P06821	2020	2020-Q1	2020-02	Pandemic	Cytology (Pap)	25-29	27	Not performed	Satisfactory	Positive	HSIL	High-grade squamous	
P06822	2020	2020-Q1	2020-02	Pandemic	Cytology (Pap)	30-34	31	Not performed	Satisfactory	NILM	NILM	Negative (NILM)	
P06823	2020	2020-Q1	2020-02	Pandemic	Cytology (Pap)	45-49	46	Not performed	Satisfactory	Positive	ASC-US	Low-grade / equivocal squamous	
P06824	2020	2020-Q1	2020-02	Pandemic	Cytology (Pap)	45-49	48	Not performed	Satisfactory	NILM	NILM	Negative (NILM)	
P06825	2020	2020-Q1	2020-02	Pandemic	Cytology (Pap)	40-44	43	Not performed	Satisfactory	NILM	NILM	Negative (NILM)	
P06826	2020	2020-Q1	2020-02	Pandemic	Cytology (Pap)	45-49	46	Not performed	Satisfactory	NILM	NILM	Negative (NILM)	
P06827	2020	2020-Q1	2020-02	Pandemic	Cytology (Pap)	50-54	52	Not performed	Satisfactory	NILM	NILM	Negative (NILM)	
P06828	2020	2020-Q1	2020-02	Pandemic	Cytology (Pap)	55-59	57	Not performed	Satisfactory	NILM	NILM	Negative (NILM)	
P06829	2020	2020-Q1	2020-02	Pandemic	Cytology (Pap)	55-59	57	Not performed	Satisfactory	NILM	NILM	Negative (NILM)	
P06830	2020	2020-Q1	2020-02	Pandemic	Cytology (Pap)	50-54	53	Not performed	Satisfactory	NILM	NILM	Negative (NILM)	
P06831	2020	2020-Q1	2020-02	Pandemic	Cytology (Pap)	35-39	35	Not performed	Satisfactory	NILM	NILM	Negative (NILM)	Candida
P06832	2020	2020-Q1	2020-02	Pandemic	Cytology (Pap)	35-39	38	Not performed	Satisfactory	NILM	NILM	Negative (NILM)	
P06833	2020	2020-Q1	2020-02	Pandemic	Cytology (Pap)	25-29	29	Not performed	Satisfactory	Positive	ASC-US	Low-grade / equivocal squamous	
P06834	2020	2020-Q1	2020-02	Pandemic	Cytology (Pap)	25-29	27	Not performed	Satisfactory	Positive	ASC-US	Low-grade / equivocal squamous	
P06835	2020	2020-Q1	2020-02	Pandemic	Cytology (Pap)	30-34	33	Not performed	Satisfactory	Positive	LSIL	Low-grade / equivocal squamous	
P06836	2020	2020-Q1	2020-02	Pandemic	Cytology (Pap)	45-49	46	Not performed	Satisfactory	NILM	NILM	Negative (NILM)	
P06837	2020	2020-Q1	2020-02	Pandemic	Cytology (Pap)	40-44	43	Not performed	Satisfactory	NILM	NILM	Negative (NILM)	
P06838	2020	2020-Q1	2020-02	Pandemic	Cytology (Pap)	35-39	39	Not performed	Satisfactory	NILM	NILM	Negative (NILM)	
P06839	2020	2020-Q1	2020-02	Pandemic	Cytology (Pap)	35-39	39	Not performed	Satisfactory	NILM	NILM	Negative (NILM)	
P06840	2020	2020-Q1	2020-02	Pandemic	Cytology (Pap)	55-59	58	Not performed	Satisfactory	NILM	NILM	Negative (NILM)	
P06841	2020	2020-Q1	2020-02	Pandemic	Cytology (Pap)	60-64	60	Not performed	Satisfactory	NILM	NILM	Negative (NILM)	
P06842	2020	2020-Q1	2020-03	Pandemic	Cytology (Pap)	50-54	50	Not performed	Satisfactory	NILM	NILM	Negative (NILM)	
P06843	2020	2020-Q1	2020-03	Pandemic	Cytology (Pap)	40-44	44	Not performed	Satisfactory	NILM	NILM	Negative (NILM)	
P06844	2020	2020-Q1	2020-03	Pandemic	Cytology (Pap)	55-59	59	Not performed	Satisfactory	NILM	NILM	Negative (NILM)	
P06845	2020	2020-Q1	2020-03	Pandemic	Cytology (Pap)	30-34	30	Not performed	Satisfactory	NILM	NILM	Negative (NILM)	
P06846	2020	2020-Q1	2020-03	Pandemic	Cytology (Pap)	45-49	47	Not performed	Satisfactory	Positive	ASC-US	Low-grade / equivocal squamous	
P06847	2020	2020-Q1	2020-03	Pandemic	Cytology (Pap)	35-39	39	Not performed	Satisfactory	NILM	NILM	Negative (NILM)	
P06848	2020	2020-Q1	2020-03	Pandemic	Cytology (Pap)	55-59	59	Not performed	Satisfactory	NILM	NILM	Negative (NILM)	
P06849	2020	2020-Q1	2020-03	Pandemic	Cytology (Pap)	35-39	35	Not performed	Satisfactory	NILM	NILM	Negative (NILM)	
P06850	2020	2020-Q1	2020-03	Pandemic	Cytology (Pap)	50-54	51	Not performed	Satisfactory	NILM	NILM	Negative (NILM)	
P06851	2020	2020-Q1	2020-03	Pandemic	Cytology (Pap)	55-59	57	Not performed	Satisfactory	NILM	NILM	Negative (NILM)	
P06852	2020	2020-Q1	2020-03	Pandemic	Cytology (Pap)	40-44	42	Not performed	Satisfactory	NILM	NILM	Negative (NILM)	
P06853	2020	2020-Q1	2020-03	Pandemic	Cytology (Pap)	55-59	56	Not performed	Satisfactory	NILM	NILM	Negative (NILM)	
P06854	2020	2020-Q1	2020-03	Pandemic	Cytology (Pap)	35-39	35	Not performed	Satisfactory	Positive	ASC-US	Low-grade / equivocal squamous	
P06855	2020	2020-Q1	2020-03	Pandemic	Cytology (Pap)	50-54	53	Not performed	Satisfactory	Positive	LSIL	Low-grade / equivocal squamous	
P06856	2020	2020-Q1	2020-03	Pandemic	Cytology (Pap)	30-34	30	Not performed	Satisfactory	NILM	NILM	Negative (NILM)	
P06857	2020	2020-Q1	2020-03	Pandemic	Cytology (Pap)	55-59	57	Not performed	Satisfactory	NILM	NILM	Negative (NILM)	
P06858	2020	2020-Q1	2020-03	Pandemic	Cytology (Pap)	40-44	43	Not performed	Satisfactory	NILM	NILM	Negative (NILM)	
P06859	2020	2020-Q1	2020-03	Pandemic	Cytology (Pap)	40-44	41	Not performed	Satisfactory	NILM	NILM	Negative (NILM)	
P06860	2020	2020-Q1	2020-03	Pandemic	Cytology (Pap)	50-54	52	Not performed	Satisfactory	NILM	NILM	Negative (NILM)	
P06861	2020	2020-Q1	2020-03	Pandemic	Cytology (Pap)	40-44	41	Not performed	Satisfactory	Positive	ASC-US	Low-grade / equivocal squamous	
P06862	2020	2020-Q1	2020-03	Pandemic	Cytology (Pap)	40-44	42	Not performed	Satisfactory	NILM	NILM	Negative (NILM)	
P06863	2020	2020-Q1	2020-03	Pandemic	Cytology (Pap)	40-44	40	Not performed	Satisfactory	NILM	NILM	Negative (NILM)	
P06864	2020	2020-Q1	2020-03	Pandemic	Cytology (Pap)	55-59	55	Not performed	Satisfactory	NILM	NILM	Negative (NILM)	
P06865	2020	2020-Q1	2020-03	Pandemic	Cytology (Pap)	30-34	33	Not performed	Satisfactory	NILM	NILM	Negative (NILM)	
P06866	2020	2020-Q1	2020-03	Pandemic	Cytology (Pap)	40-44	42	Not performed	Satisfactory	NILM	NILM	Negative (NILM)	
P06867	2020	2020-Q1	2020-03	Pandemic	Cytology (Pap)	30-34	31	Not performed	Satisfactory	NILM	NILM	Negative (NILM)	
P06868	2020	2020-Q1	2020-03	Pandemic	Cytology (Pap)	60-64	60	Not performed	Satisfactory	Positive	HSIL	High-grade squamous	
P06869	2020	2020-Q1	2020-03	Pandemic	Cytology (Pap)	30-34	34	Not performed	Satisfactory	NILM	NILM	Negative (NILM)	
P06870	2020	2020-Q1	2020-03	Pandemic	Cytology (Pap)	50-54	54	Not performed	Satisfactory	NILM	NILM	Negative (NILM)	
P06871	2020	2020-Q1	2020-03	Pandemic	Cytology (Pap)	45-49	45	Not performed	Satisfactory	NILM	NILM	Negative (NILM)	
P06872	2020	2020-Q1	2020-03	Pandemic	Cytology (Pap)	45-49	48	Not performed	Satisfactory	NILM	NILM	Negative (NILM)	
P06873	2020	2020-Q1	2020-03	Pandemic	Cytology (Pap)	30-34	30	Not performed	Satisfactory	NILM	NILM	Negative (NILM)	
P06874	2020	2020-Q1	2020-03	Pandemic	Cytology (Pap)	25-29	26	Not performed	Satisfactory	NILM	NILM	Negative (NILM)	
P06875	2020	2020-Q1	2020-03	Pandemic	Cytology (Pap)	45-49	49	Not performed	Satisfactory	NILM	NILM	Negative (NILM)	
P06876	2020	2020-Q1	2020-03	Pandemic	Cytology (Pap)	45-49	49	Not performed	Satisfactory	NILM	NILM	Negative (NILM)	
P06877	2020	2020-Q1	2020-03	Pandemic	Cytology (Pap)	35-39	37	Not performed	Satisfactory	Positive	ASC-US	Low-grade / equivocal squamous	
P06878	2020	2020-Q1	2020-03	Pandemic	Cytology (Pap)	35-39	35	Not performed	Satisfactory	NILM	NILM	Negative (NILM)	

patient_id	year	quarter	month	period	primary_test	age_group	age_years	hpv_result	adequacy	result	bethesda	grade	infection
P06879	2020	2020-Q1	2020-03	Pandemic	Cytology (Pap)	40-44	43	Not performed	Satisfactory	NILM	NILM	Negative (NILM)	
P06880	2020	2020-Q1	2020-03	Pandemic	Cytology (Pap)	45-49	47	Not performed	Satisfactory	NILM	NILM	Negative (NILM)	
P06881	2020	2020-Q1	2020-03	Pandemic	Cytology (Pap)	55-59	55	Not performed	Satisfactory	NILM	NILM	Negative (NILM)	
P06882	2020	2020-Q1	2020-03	Pandemic	Cytology (Pap)	35-39	36	Not performed	Satisfactory	NILM	NILM	Negative (NILM)	
P06883	2020	2020-Q1	2020-03	Pandemic	Cytology (Pap)	50-54	53	Not performed	Satisfactory	NILM	NILM	Negative (NILM)	
P06884	2020	2020-Q1	2020-03	Pandemic	Cytology (Pap)	50-54	53	Not performed	Satisfactory	NILM	NILM	Negative (NILM)	
P06885	2020	2020-Q1	2020-03	Pandemic	Cytology (Pap)	50-54	54	Not performed	Satisfactory	NILM	NILM	Negative (NILM)	
P06886	2020	2020-Q1	2020-03	Pandemic	Cytology (Pap)	40-44	41	Not performed	Satisfactory	NILM	NILM	Negative (NILM)	
P06887	2020	2020-Q1	2020-03	Pandemic	Cytology (Pap)	40-44	41	Not performed	Unsatisfactory	Unsatisfactory	Unsatisfactory	Unsatisfactory	
P06888	2020	2020-Q1	2020-03	Pandemic	Cytology (Pap)	45-49	46	Not performed	Satisfactory	NILM	NILM	Negative (NILM)	
P06889	2020	2020-Q1	2020-03	Pandemic	Cytology (Pap)	45-49	45	Not performed	Satisfactory	NILM	NILM	Negative (NILM)	
P06890	2020	2020-Q1	2020-03	Pandemic	Cytology (Pap)	45-49	48	Not performed	Satisfactory	NILM	NILM	Negative (NILM)	
P06891	2020	2020-Q1	2020-03	Pandemic	Cytology (Pap)	35-39	35	Not performed	Satisfactory	NILM	NILM	Negative (NILM)	
P06892	2020	2020-Q1	2020-03	Pandemic	Cytology (Pap)	60-64	63	Not performed	Satisfactory	NILM	NILM	Negative (NILM)	
P06893	2020	2020-Q1	2020-03	Pandemic	Cytology (Pap)	40-44	44	Not performed	Satisfactory	Positive	ASC-US	Low-grade / equivocal squamous	
P06894	2020	2020-Q1	2020-03	Pandemic	Cytology (Pap)	50-54	54	Not performed	Satisfactory	NILM	NILM	Negative (NILM)	
P06895	2020	2020-Q1	2020-03	Pandemic	Cytology (Pap)	50-54	50	Not performed	Satisfactory	Positive	LSIL	Low-grade / equivocal squamous	
P06896	2020	2020-Q1	2020-03	Pandemic	Cytology (Pap)	45-49	45	Not performed	Satisfactory	NILM	NILM	Negative (NILM)	
P06897	2020	2020-Q1	2020-03	Pandemic	Cytology (Pap)	60-64	62	Not performed	Satisfactory	NILM	NILM	Negative (NILM)	
P06898	2020	2020-Q1	2020-03	Pandemic	Cytology (Pap)	45-49	48	Not performed	Satisfactory	NILM	NILM	Negative (NILM)	
P06899	2020	2020-Q1	2020-03	Pandemic	Cytology (Pap)	45-49	45	Not performed	Satisfactory	NILM	NILM	Negative (NILM)	
P06900	2020	2020-Q1	2020-03	Pandemic	Cytology (Pap)	50-54	51	Not performed	Satisfactory	NILM	NILM	Negative (NILM)	
P06901	2020	2020-Q1	2020-03	Pandemic	Cytology (Pap)	45-49	46	Not performed	Satisfactory	Positive	LSIL	Low-grade / equivocal squamous	
P06902	2020	2020-Q1	2020-03	Pandemic	Cytology (Pap)	55-59	58	Not performed	Satisfactory	NILM	NILM	Negative (NILM)	
P06903	2020	2020-Q1	2020-03	Pandemic	Cytology (Pap)	45-49	47	Not performed	Satisfactory	NILM	NILM	Negative (NILM)	
P06904	2020	2020-Q1	2020-03	Pandemic	Cytology (Pap)	40-44	43	Not performed	Unsatisfactory	Unsatisfactory	Unsatisfactory	Unsatisfactory	
P06905	2020	2020-Q1	2020-03	Pandemic	Cytology (Pap)	40-44	41	Not performed	Satisfactory	Positive	ASC-US	Low-grade / equivocal squamous	
P06906	2020	2020-Q1	2020-03	Pandemic	Cytology (Pap)	60-64	61	Not performed	Satisfactory	Positive	HSIL	High-grade squamous	
P06907	2020	2020-Q1	2020-03	Pandemic	Cytology (Pap)	60-64	62	Not performed	Satisfactory	NILM	NILM	Negative (NILM)	
P06908	2020	2020-Q1	2020-03	Pandemic	Cytology (Pap)	40-44	42	Not performed	Satisfactory	NILM	NILM	Negative (NILM)	
P06909	2020	2020-Q1	2020-03	Pandemic	Cytology (Pap)	30-34	34	Not performed	Satisfactory	NILM	NILM	Negative (NILM)	
P06910	2020	2020-Q1	2020-03	Pandemic	Cytology (Pap)	40-44	43	Not performed	Unsatisfactory	Unsatisfactory	Unsatisfactory	Unsatisfactory	
P06911	2020	2020-Q1	2020-03	Pandemic	Cytology (Pap)	40-44	40	Not performed	Unsatisfactory	Unsatisfactory	Unsatisfactory	Unsatisfactory	
P06912	2020	2020-Q1	2020-03	Pandemic	Cytology (Pap)	30-34	30	Not performed	Satisfactory	NILM	NILM	Negative (NILM)	
P06913	2020	2020-Q1	2020-03	Pandemic	Cytology (Pap)	50-54	50	Not performed	Satisfactory	NILM	NILM	Negative (NILM)	
P06914	2020	2020-Q1	2020-03	Pandemic	Cytology (Pap)	30-34	34	Not performed	Satisfactory	NILM	NILM	Negative (NILM)	
P06915	2020	2020-Q1	2020-03	Pandemic	Cytology (Pap)	60-64	61	Not performed	Satisfactory	NILM	NILM	Negative (NILM)	
P06916	2020	2020-Q1	2020-03	Pandemic	Cytology (Pap)	35-39	35	Not performed	Satisfactory	NILM	NILM	Negative (NILM)	
P06917	2020	2020-Q1	2020-03	Pandemic	Cytology (Pap)	40-44	44	Not performed	Satisfactory	NILM	NILM	Negative (NILM)	
P06918	2020	2020-Q1	2020-03	Pandemic	Cytology (Pap)	35-39	35	Not performed	Satisfactory	NILM	NILM	Negative (NILM)	
P06919	2020	2020-Q1	2020-03	Pandemic	Cytology (Pap)	45-49	45	Not performed	Satisfactory	NILM	NILM	Negative (NILM)	
P06920	2020	2020-Q1	2020-03	Pandemic	Cytology (Pap)	35-39	35	Not performed	Satisfactory	NILM	NILM	Negative (NILM)	
P06921	2020	2020-Q1	2020-03	Pandemic	Cytology (Pap)	30-34	33	Not performed	Satisfactory	Positive	LSIL	Low-grade / equivocal squamous	
P06922	2020	2020-Q1	2020-03	Pandemic	Cytology (Pap)	40-44	41	Not performed	Satisfactory	NILM	NILM	Negative (NILM)	
P06923	2020	2020-Q1	2020-03	Pandemic	Cytology (Pap)	55-59	57	Not performed	Satisfactory	NILM	NILM	Negative (NILM)	
P06924	2020	2020-Q1	2020-03	Pandemic	Cytology (Pap)	40-44	41	Not performed	Satisfactory	NILM	NILM	Negative (NILM)	
P06925	2020	2020-Q1	2020-03	Pandemic	Cytology (Pap)	45-49	48	Not performed	Satisfactory	Positive	HSIL	High-grade squamous	
P06926	2020	2020-Q1	2020-03	Pandemic	Cytology (Pap)	35-39	35	Not performed	Satisfactory	NILM	NILM	Negative (NILM)	
P06927	2020	2020-Q1	2020-03	Pandemic	Cytology (Pap)	25-29	26	Not performed	Satisfactory	NILM	NILM	Negative (NILM)	
P06928	2020	2020-Q1	2020-03	Pandemic	Cytology (Pap)	40-44	42	Not performed	Satisfactory	NILM	NILM	Negative (NILM)	
P06929	2020	2020-Q1	2020-03	Pandemic	Cytology (Pap)	35-39	37	Not performed	Satisfactory	NILM	NILM	Negative (NILM)	
P06930	2020	2020-Q1	2020-03	Pandemic	Cytology (Pap)	30-34	30	Not performed	Satisfactory	NILM	NILM	Negative (NILM)	
P06931	2020	2020-Q1	2020-03	Pandemic	Cytology (Pap)	55-59	57	Not performed	Satisfactory	Positive	LSIL	Low-grade / equivocal squamous	
P06932	2020	2020-Q1	2020-03	Pandemic	Cytology (Pap)	50-54	51	Not performed	Satisfactory	NILM	NILM	Negative (NILM)	
P06933	2020	2020-Q1	2020-03	Pandemic	Cytology (Pap)	55-59	55	Not performed	Satisfactory	NILM	NILM	Negative (NILM)	
P06934	2020	2020-Q1	2020-03	Pandemic	Cytology (Pap)	50-54	52	Not performed	Satisfactory	NILM	NILM	Negative (NILM)	
P06935	2020	2020-Q1	2020-03	Pandemic	Cytology (Pap)	35-39	37	Not performed	Satisfactory	Positive	HSIL	High-grade squamous	
P06936	2020	2020-Q1	2020-03	Pandemic	Cytology (Pap)	35-39	36	Not performed	Satisfactory	NILM	NILM	Negative (NILM)	
P06937	2020	2020-Q1	2020-03	Pandemic	Cytology (Pap)	55-59	56	Not performed	Satisfactory	NILM	NILM	Negative (NILM)	
P06938	2020	2020-Q1	2020-03	Pandemic	Cytology (Pap)	60-64	64	Not performed	Satisfactory	NILM	NILM	Negative (NILM)	
P06939	2020	2020-Q1	2020-03	Pandemic	Cytology (Pap)	45-49	46	Not performed	Satisfactory	NILM	NILM	Negative (NILM)	
P06940	2020	2020-Q1	2020-03	Pandemic	Cytology (Pap)	25-29	27	Not performed	Satisfactory	NILM	NILM	Negative (NILM)	
P06941	2020	2020-Q1	2020-03	Pandemic	Cytology (Pap)	50-54	53	Not performed	Satisfactory	NILM	NILM	Negative (NILM)	
P06942	2020	2020-Q1	2020-03	Pandemic	Cytology (Pap)	60-64	63	Not performed	Satisfactory	NILM	NILM	Negative (NILM)	
P06943	2020	2020-Q1	2020-03	Pandemic	Cytology (Pap)	45-49	46	Not performed	Satisfactory	NILM	NILM	Negative (NILM)	
P06944	2020	2020-Q1	2020-03	Pandemic	Cytology (Pap)	35-39	36	Not performed	Satisfactory	NILM	NILM	Negative (NILM)	Candida
P06945	2020	2020-Q1	2020-03	Pandemic	Cytology (Pap)	35-39	35	Not performed	Satisfactory	NILM	NILM	Negative (NILM)	
P06946	2020	2020-Q1	2020-03	Pandemic	Cytology (Pap)	50-54	51	Not performed	Satisfactory	NILM	NILM	Negative (NILM)	
P06947	2020	2020-Q1	2020-03	Pandemic	Cytology (Pap)	55-59	55	Not performed	Satisfactory	NILM	NILM	Negative (NILM)	
P06948	2020	2020-Q1	2020-03	Pandemic	Cytology (Pap)	55-59	59	Not performed	Satisfactory	NILM	NILM	Negative (NILM)	
P06949	2020	2020-Q1	2020-03	Pandemic	Cytology (Pap)	40-44	44	Not performed	Satisfactory	NILM	NILM	Negative (NILM)	
P06950	2020	2020-Q1	2020-03	Pandemic	Cytology (Pap)	45-49	45	Not performed	Satisfactory	NILM	NILM	Negative (NILM)	
P06951	2020	2020-Q1	2020-03	Pandemic	Cytology (Pap)	50-54	53	Not performed	Satisfactory	NILM	NILM	Negative (NILM)	
P06952	2020	2020-Q1	2020-03	Pandemic	Cytology (Pap)	40-44	40	Not performed	Satisfactory	NILM	NILM	Negative (NILM)	
P06953	2020	2020-Q1	2020-03	Pandemic	Cytology (Pap)	55-59	58	Not performed	Satisfactory	NILM	NILM	Negative (NILM)	
P06954	2020	2020-Q1	2020-03	Pandemic	Cytology (Pap)	40-44	44	Not performed	Satisfactory	Positive	ASC-H	High-grade squamous	
P06955	2020	2020-Q1	2020-03	Pandemic	Cytology (Pap)	50-54	50	Not performed	Satisfactory	Positive	LSIL	Low-grade / equivocal squamous	
P06956	2020	2020-Q1	2020-03	Pandemic	Cytology (Pap)	35-39	38	Not performed	Satisfactory	NILM	NILM	Negative (NILM)	
P06957	2020	2020-Q1	2020-03	Pandemic	Cytology (Pap)	35-39	35	Not performed	Satisfactory	NILM	NILM	Negative (NILM)	
P06958	2020	2020-Q1	2020-03	Pandemic	Cytology (Pap)	35-39	35	Not performed	Unsatisfactory	Unsatisfactory	Unsatisfactory	Unsatisfactory	

patient_id	year	quarter	month	period	primary_test	age_group	age_years	hpv_result	adequacy	result	bethesda	grade	infection
P06959	2020	2020-Q1	2020-03	Pandemic	Cytology (Pap)	35-39	39	Not performed	Satisfactory	NILM	NILM	Negative (NILM)	
P06960	2020	2020-Q1	2020-03	Pandemic	Cytology (Pap)	50-54	50	Not performed	Satisfactory	NILM	NILM	Negative (NILM)	
P06961	2020	2020-Q1	2020-03	Pandemic	Cytology (Pap)	40-44	40	Not performed	Satisfactory	NILM	NILM	Negative (NILM)	
P06962	2020	2020-Q1	2020-03	Pandemic	Cytology (Pap)	35-39	37	Not performed	Satisfactory	NILM	NILM	Negative (NILM)	
P06963	2020	2020-Q1	2020-03	Pandemic	Cytology (Pap)	45-49	46	Not performed	Satisfactory	NILM	NILM	Negative (NILM)	
P06964	2020	2020-Q1	2020-03	Pandemic	Cytology (Pap)	25-29	25	Not performed	Satisfactory	NILM	NILM	Negative (NILM)	
P06965	2020	2020-Q1	2020-03	Pandemic	Cytology (Pap)	45-49	49	Not performed	Satisfactory	NILM	NILM	Negative (NILM)	
P06966	2020	2020-Q1	2020-03	Pandemic	Cytology (Pap)	50-54	52	Not performed	Satisfactory	NILM	NILM	Negative (NILM)	
P06967	2020	2020-Q1	2020-03	Pandemic	Cytology (Pap)	40-44	44	Not performed	Satisfactory	NILM	NILM	Negative (NILM)	
P06968	2020	2020-Q1	2020-03	Pandemic	Cytology (Pap)	35-39	38	Not performed	Satisfactory	NILM	NILM	Negative (NILM)	
P06969	2020	2020-Q1	2020-03	Pandemic	Cytology (Pap)	45-49	46	Not performed	Satisfactory	NILM	NILM	Negative (NILM)	
P06970	2020	2020-Q1	2020-03	Pandemic	Cytology (Pap)	40-44	41	Not performed	Satisfactory	NILM	NILM	Negative (NILM)	
P06971	2020	2020-Q1	2020-03	Pandemic	Cytology (Pap)	30-34	31	Not performed	Satisfactory	NILM	NILM	Negative (NILM)	
P06972	2020	2020-Q1	2020-03	Pandemic	Cytology (Pap)	35-39	35	Not performed	Satisfactory	NILM	NILM	Negative (NILM)	
P06973	2020	2020-Q1	2020-03	Pandemic	Cytology (Pap)	40-44	42	Not performed	Satisfactory	NILM	NILM	Negative (NILM)	
P06974	2020	2020-Q1	2020-03	Pandemic	Cytology (Pap)	50-54	50	Not performed	Satisfactory	NILM	NILM	Negative (NILM)	
P06975	2020	2020-Q1	2020-03	Pandemic	Cytology (Pap)	35-39	39	Not performed	Satisfactory	NILM	NILM	Negative (NILM)	
P06976	2020	2020-Q1	2020-03	Pandemic	Cytology (Pap)	45-49	47	Not performed	Satisfactory	NILM	NILM	Negative (NILM)	
P06977	2020	2020-Q1	2020-03	Pandemic	Cytology (Pap)	55-59	56	Not performed	Satisfactory	NILM	NILM	Negative (NILM)	Candida
P06978	2020	2020-Q1	2020-03	Pandemic	Cytology (Pap)	60-64	64	Not performed	Satisfactory	NILM	NILM	Negative (NILM)	
P06979	2020	2020-Q1	2020-03	Pandemic	Cytology (Pap)	40-44	41	Not performed	Satisfactory	NILM	NILM	Negative (NILM)	
P06980	2020	2020-Q1	2020-03	Pandemic	Cytology (Pap)	45-49	46	Not performed	Satisfactory	Positive	LSIL	Low-grade / equivocal squamous	
P06981	2020	2020-Q1	2020-03	Pandemic	Cytology (Pap)	35-39	39	Not performed	Satisfactory	NILM	NILM	Negative (NILM)	
P06982	2020	2020-Q1	2020-03	Pandemic	Cytology (Pap)	40-44	43	Not performed	Satisfactory	NILM	NILM	Negative (NILM)	
P06983	2020	2020-Q1	2020-03	Pandemic	Cytology (Pap)	45-49	49	Not performed	Satisfactory	NILM	NILM	Negative (NILM)	
P06984	2020	2020-Q1	2020-03	Pandemic	Cytology (Pap)	45-49	45	Not performed	Satisfactory	NILM	NILM	Negative (NILM)	
P06985	2020	2020-Q1	2020-03	Pandemic	Cytology (Pap)	30-34	31	Not performed	Satisfactory	NILM	NILM	Negative (NILM)	
P06986	2020	2020-Q1	2020-03	Pandemic	Cytology (Pap)	35-39	36	Not performed	Satisfactory	NILM	NILM	Negative (NILM)	
P06987	2020	2020-Q1	2020-03	Pandemic	Cytology (Pap)	35-39	36	Not performed	Satisfactory	NILM	NILM	Negative (NILM)	
P06988	2020	2020-Q1	2020-03	Pandemic	Cytology (Pap)	45-49	49	Not performed	Satisfactory	NILM	NILM	Negative (NILM)	
P06989	2020	2020-Q1	2020-03	Pandemic	Cytology (Pap)	35-39	36	Not performed	Satisfactory	NILM	NILM	Negative (NILM)	
P06990	2020	2020-Q1	2020-03	Pandemic	Cytology (Pap)	50-54	50	Not performed	Satisfactory	NILM	NILM	Negative (NILM)	
P06991	2020	2020-Q1	2020-03	Pandemic	Cytology (Pap)	40-44	43	Not performed	Satisfactory	NILM	NILM	Negative (NILM)	
P06992	2020	2020-Q1	2020-03	Pandemic	Cytology (Pap)	55-59	58	Not performed	Satisfactory	NILM	NILM	Negative (NILM)	
P06993	2020	2020-Q1	2020-03	Pandemic	Cytology (Pap)	55-59	59	Not performed	Satisfactory	NILM	NILM	Negative (NILM)	
P06994	2020	2020-Q1	2020-03	Pandemic	Cytology (Pap)	60-64	62	Not performed	Satisfactory	Positive	ASC-US	Low-grade / equivocal squamous	
P06995	2020	2020-Q1	2020-03	Pandemic	Cytology (Pap)	35-39	39	Not performed	Satisfactory	NILM	NILM	Negative (NILM)	
P06996	2020	2020-Q1	2020-03	Pandemic	Cytology (Pap)	40-44	43	Not performed	Satisfactory	NILM	NILM	Negative (NILM)	Trichomonas
P06997	2020	2020-Q1	2020-03	Pandemic	Cytology (Pap)	25-29	25	Not performed	Satisfactory	NILM	NILM	Negative (NILM)	
P06998	2020	2020-Q1	2020-03	Pandemic	Cytology (Pap)	60-64	61	Not performed	Satisfactory	Positive	ASC-US	Low-grade / equivocal squamous	
P06999	2020	2020-Q1	2020-03	Pandemic	Cytology (Pap)	55-59	55	Not performed	Satisfactory	Positive	LSIL	Low-grade / equivocal squamous	
P07000	2020	2020-Q1	2020-03	Pandemic	Cytology (Pap)	55-59	56	Not performed	Satisfactory	NILM	NILM	Negative (NILM)	
P07001	2020	2020-Q1	2020-03	Pandemic	Cytology (Pap)	40-44	40	Not performed	Satisfactory	NILM	NILM	Negative (NILM)	
P07002	2020	2020-Q1	2020-03	Pandemic	Cytology (Pap)	30-34	34	Not performed	Satisfactory	NILM	NILM	Negative (NILM)	
P07003	2020	2020-Q1	2020-03	Pandemic	Cytology (Pap)	50-54	52	Not performed	Satisfactory	NILM	NILM	Negative (NILM)	
P07004	2020	2020-Q1	2020-03	Pandemic	Cytology (Pap)	45-49	49	Not performed	Satisfactory	NILM	NILM	Negative (NILM)	
P07005	2020	2020-Q1	2020-03	Pandemic	Cytology (Pap)	35-39	35	Not performed	Satisfactory	NILM	NILM	Negative (NILM)	
P07006	2020	2020-Q1	2020-03	Pandemic	Cytology (Pap)	25-29	29	Not performed	Satisfactory	NILM	NILM	Negative (NILM)	
P07007	2020	2020-Q1	2020-03	Pandemic	Cytology (Pap)	35-39	36	Not performed	Satisfactory	NILM	NILM	Negative (NILM)	
P07008	2020	2020-Q1	2020-03	Pandemic	Cytology (Pap)	25-29	27	Not performed	Satisfactory	NILM	NILM	Negative (NILM)	Candida
P07009	2020	2020-Q1	2020-03	Pandemic	Cytology (Pap)	40-44	40	Not performed	Satisfactory	NILM	NILM	Negative (NILM)	
P07010	2020	2020-Q1	2020-03	Pandemic	Cytology (Pap)	35-39	35	Not performed	Satisfactory	NILM	NILM	Negative (NILM)	
P07011	2020	2020-Q1	2020-03	Pandemic	Cytology (Pap)	45-49	45	Not performed	Satisfactory	Positive	ASC-US	Low-grade / equivocal squamous	
P07012	2020	2020-Q1	2020-03	Pandemic	Cytology (Pap)	45-49	45	Not performed	Satisfactory	NILM	NILM	Negative (NILM)	
P07013	2020	2020-Q1	2020-03	Pandemic	Cytology (Pap)	50-54	54	Not performed	Satisfactory	NILM	NILM	Negative (NILM)	
P07014	2020	2020-Q1	2020-03	Pandemic	Cytology (Pap)	40-44	43	Not performed	Satisfactory	NILM	NILM	Negative (NILM)	
P07015	2020	2020-Q1	2020-03	Pandemic	Cytology (Pap)	35-39	35	Not performed	Satisfactory	NILM	NILM	Negative (NILM)	
P07016	2020	2020-Q1	2020-03	Pandemic	Cytology (Pap)	30-34	30	Not performed	Satisfactory	NILM	NILM	Negative (NILM)	
P07017	2020	2020-Q1	2020-03	Pandemic	Cytology (Pap)	30-34	32	Not performed	Satisfactory	Positive	HSIL	High-grade squamous	
P07018	2020	2020-Q1	2020-03	Pandemic	Cytology (Pap)	60-64	64	Not performed	Satisfactory	NILM	NILM	Negative (NILM)	
P07019	2020	2020-Q1	2020-03	Pandemic	Cytology (Pap)	55-59	55	Not performed	Satisfactory	Positive	ASC-US	Low-grade / equivocal squamous	
P07020	2020	2020-Q1	2020-03	Pandemic	Cytology (Pap)	50-54	51	Not performed	Satisfactory	NILM	NILM	Negative (NILM)	
P07021	2020	2020-Q1	2020-03	Pandemic	Cytology (Pap)	50-54	52	Not performed	Satisfactory	NILM	NILM	Negative (NILM)	
P07022	2020	2020-Q1	2020-03	Pandemic	Cytology (Pap)	55-59	56	Not performed	Satisfactory	NILM	NILM	Negative (NILM)	
P07023	2020	2020-Q1	2020-03	Pandemic	Cytology (Pap)	55-59	58	Not performed	Satisfactory	NILM	NILM	Negative (NILM)	
P07024	2020	2020-Q1	2020-03	Pandemic	Cytology (Pap)	45-49	45	Not performed	Satisfactory	NILM	NILM	Negative (NILM)	
P07025	2020	2020-Q1	2020-03	Pandemic	Cytology (Pap)	40-44	40	Not performed	Satisfactory	NILM	NILM	Negative (NILM)	
P07026	2020	2020-Q1	2020-03	Pandemic	Cytology (Pap)	45-49	46	Not performed	Satisfactory	Positive	ASC-US	Low-grade / equivocal squamous	
P07027	2020	2020-Q1	2020-03	Pandemic	Cytology (Pap)	40-44	43	Not performed	Satisfactory	NILM	NILM	Negative (NILM)	
P07028	2020	2020-Q1	2020-03	Pandemic	Cytology (Pap)	50-54	54	Not performed	Satisfactory	NILM	NILM	Negative (NILM)	
P07029	2020	2020-Q1	2020-03	Pandemic	Cytology (Pap)	35-39	38	Not performed	Satisfactory	NILM	NILM	Negative (NILM)	
P07030	2020	2020-Q1	2020-03	Pandemic	Cytology (Pap)	30-34	33	Not performed	Satisfactory	NILM	NILM	Negative (NILM)	
P07031	2020	2020-Q1	2020-03	Pandemic	Cytology (Pap)	35-39	38	Not performed	Satisfactory	NILM	NILM	Negative (NILM)	
P07032	2020	2020-Q1	2020-03	Pandemic	Cytology (Pap)	30-34	31	Not performed	Satisfactory	Positive	HSIL	High-grade squamous	
P07033	2020	2020-Q1	2020-03	Pandemic	Cytology (Pap)	40-44	42	Not performed	Satisfactory	NILM	NILM	Negative (NILM)	
P07034	2020	2020-Q1	2020-03	Pandemic	Cytology (Pap)	40-44	43	Not performed	Satisfactory	NILM	NILM	Negative (NILM)	
P07035	2020	2020-Q1	2020-03	Pandemic	Cytology (Pap)	40-44	42	Not performed	Satisfactory	NILM	NILM	Negative (NILM)	
P07036	2020	2020-Q1	2020-03	Pandemic	Cytology (Pap)	40-44	41	Not performed	Satisfactory	NILM	NILM	Negative (NILM)	
P07037	2020	2020-Q1	2020-03	Pandemic	Cytology (Pap)	55-59	57	Not performed	Satisfactory	NILM	NILM	Negative (NILM)	
P07038	2020	2020-Q1	2020-03	Pandemic	Cytology (Pap)	45-49	49	Not performed	Satisfactory	NILM	NILM	Negative (NILM)	

patient_id	year	quarter	month	period	primary_test	age_group	age_years	hpv_result	adequacy	result	bethesda	grade	infection
P07039	2020	2020-Q1	2020-03	Pandemic	Cytology (Pap)	30-34	33	Not performed	Satisfactory	NILM	NILM	Negative (NILM)	
P07040	2020	2020-Q1	2020-03	Pandemic	Cytology (Pap)	35-39	36	Not performed	Satisfactory	NILM	NILM	Negative (NILM)	
P07041	2020	2020-Q1	2020-03	Pandemic	Cytology (Pap)	55-59	59	Not performed	Satisfactory	NILM	NILM	Negative (NILM)	
P07042	2020	2020-Q1	2020-03	Pandemic	Cytology (Pap)	60-64	62	Not performed	Satisfactory	NILM	NILM	Negative (NILM)	
P07043	2020	2020-Q1	2020-03	Pandemic	Cytology (Pap)	30-34	32	Not performed	Satisfactory	NILM	NILM	Negative (NILM)	
P07044	2020	2020-Q1	2020-03	Pandemic	Cytology (Pap)	30-34	32	Not performed	Satisfactory	NILM	NILM	Negative (NILM)	
P07045	2020	2020-Q1	2020-03	Pandemic	Cytology (Pap)	55-59	55	Not performed	Satisfactory	NILM	NILM	Negative (NILM)	
P07046	2020	2020-Q1	2020-03	Pandemic	Cytology (Pap)	50-54	54	Not performed	Satisfactory	NILM	NILM	Negative (NILM)	
P07047	2020	2020-Q1	2020-03	Pandemic	Cytology (Pap)	35-39	39	Not performed	Satisfactory	NILM	NILM	Negative (NILM)	
P07048	2020	2020-Q1	2020-03	Pandemic	Cytology (Pap)	40-44	44	Not performed	Satisfactory	NILM	NILM	Negative (NILM)	
P07049	2020	2020-Q1	2020-03	Pandemic	Cytology (Pap)	45-49	48	Not performed	Satisfactory	NILM	NILM	Negative (NILM)	
P07050	2020	2020-Q1	2020-03	Pandemic	Cytology (Pap)	50-54	54	Not performed	Satisfactory	Positive	LSIL	Low-grade / equivocal squamous	
P07051	2020	2020-Q1	2020-03	Pandemic	Cytology (Pap)	50-54	54	Not performed	Satisfactory	NILM	NILM	Negative (NILM)	
P07052	2020	2020-Q1	2020-03	Pandemic	Cytology (Pap)	45-49	48	Not performed	Satisfactory	NILM	NILM	Negative (NILM)	
P07053	2020	2020-Q1	2020-03	Pandemic	Cytology (Pap)	40-44	42	Not performed	Satisfactory	NILM	NILM	Negative (NILM)	
P07054	2020	2020-Q1	2020-03	Pandemic	Cytology (Pap)	35-39	37	Not performed	Satisfactory	NILM	NILM	Negative (NILM)	Candida
P07055	2020	2020-Q1	2020-03	Pandemic	Cytology (Pap)	55-59	57	Not performed	Satisfactory	Positive	HSIL	High-grade squamous	
P07056	2020	2020-Q1	2020-03	Pandemic	Cytology (Pap)	40-44	41	Not performed	Satisfactory	NILM	NILM	Negative (NILM)	
P07057	2020	2020-Q1	2020-03	Pandemic	Cytology (Pap)	35-39	36	Not performed	Satisfactory	NILM	NILM	Negative (NILM)	
P07058	2020	2020-Q1	2020-03	Pandemic	Cytology (Pap)	25-29	29	Not performed	Unsatisfactory	Unsatisfactory	Unsatisfactory	Unsatisfactory	
P07059	2020	2020-Q1	2020-03	Pandemic	Cytology (Pap)	40-44	41	Not performed	Unsatisfactory	Unsatisfactory	Unsatisfactory	Unsatisfactory	
P07060	2020	2020-Q1	2020-03	Pandemic	Cytology (Pap)	25-29	29	Not performed	Satisfactory	NILM	NILM	Negative (NILM)	
P07061	2020	2020-Q1	2020-03	Pandemic	Cytology (Pap)	60-64	62	Not performed	Satisfactory	NILM	NILM	Negative (NILM)	
P07062	2020	2020-Q1	2020-03	Pandemic	Cytology (Pap)	30-34	32	Not performed	Satisfactory	NILM	NILM	Negative (NILM)	
P07063	2020	2020-Q1	2020-03	Pandemic	Cytology (Pap)	35-39	35	Not performed	Satisfactory	Positive	HSIL	High-grade squamous	
P07064	2020	2020-Q1	2020-03	Pandemic	Cytology (Pap)	35-39	35	Not performed	Satisfactory	NILM	NILM	Negative (NILM)	
P07065	2020	2020-Q1	2020-03	Pandemic	Cytology (Pap)	30-34	31	Not performed	Satisfactory	NILM	NILM	Negative (NILM)	
P07066	2020	2020-Q1	2020-03	Pandemic	Cytology (Pap)	30-34	31	Not performed	Unsatisfactory	Unsatisfactory	Unsatisfactory	Unsatisfactory	
P07067	2020	2020-Q1	2020-03	Pandemic	Cytology (Pap)	35-39	35	Not performed	Satisfactory	Positive	HSIL	High-grade squamous	
P07068	2020	2020-Q1	2020-03	Pandemic	Cytology (Pap)	40-44	43	Not performed	Satisfactory	NILM	NILM	Negative (NILM)	
P07069	2020	2020-Q1	2020-03	Pandemic	Cytology (Pap)	40-44	44	Not performed	Satisfactory	NILM	NILM	Negative (NILM)	
P07070	2020	2020-Q1	2020-03	Pandemic	Cytology (Pap)	35-39	35	Not performed	Satisfactory	NILM	NILM	Negative (NILM)	
P07071	2020	2020-Q1	2020-03	Pandemic	Cytology (Pap)	25-29	28	Not performed	Satisfactory	NILM	NILM	Negative (NILM)	
P07072	2020	2020-Q1	2020-03	Pandemic	Cytology (Pap)	40-44	40	Not performed	Satisfactory	NILM	NILM	Negative (NILM)	
P07073	2020	2020-Q1	2020-03	Pandemic	Cytology (Pap)	50-54	51	Not performed	Satisfactory	NILM	NILM	Negative (NILM)	
P07074	2020	2020-Q1	2020-03	Pandemic	Cytology (Pap)	55-59	55	Not performed	Satisfactory	NILM	NILM	Negative (NILM)	
P07075	2020	2020-Q1	2020-03	Pandemic	Cytology (Pap)	45-49	45	Not performed	Satisfactory	NILM	NILM	Negative (NILM)	
P07076	2020	2020-Q1	2020-03	Pandemic	Cytology (Pap)	35-39	37	Not performed	Satisfactory	NILM	NILM	Negative (NILM)	
P07077	2020	2020-Q1	2020-03	Pandemic	Cytology (Pap)	40-44	42	Not performed	Satisfactory	Positive	LSIL	Low-grade / equivocal squamous	
P07078	2020	2020-Q1	2020-03	Pandemic	Cytology (Pap)	60-64	63	Not performed	Satisfactory	NILM	NILM	Negative (NILM)	
P07079	2020	2020-Q1	2020-03	Pandemic	Cytology (Pap)	40-44	43	Not performed	Satisfactory	NILM	NILM	Negative (NILM)	
P07080	2020	2020-Q1	2020-03	Pandemic	Cytology (Pap)	40-44	44	Not performed	Satisfactory	NILM	NILM	Negative (NILM)	
P07081	2020	2020-Q1	2020-03	Pandemic	Cytology (Pap)	30-34	31	Not performed	Satisfactory	NILM	NILM	Negative (NILM)	
P07082	2020	2020-Q1	2020-03	Pandemic	Cytology (Pap)	35-39	39	Not performed	Unsatisfactory	Unsatisfactory	Unsatisfactory	Unsatisfactory	Trichomonas
P07083	2020	2020-Q1	2020-03	Pandemic	Cytology (Pap)	55-59	58	Not performed	Satisfactory	NILM	NILM	Negative (NILM)	
P07084	2020	2020-Q1	2020-03	Pandemic	Cytology (Pap)	45-49	47	Not performed	Satisfactory	NILM	NILM	Negative (NILM)	
P07085	2020	2020-Q1	2020-03	Pandemic	Cytology (Pap)	30-34	31	Not performed	Satisfactory	Positive	ASC-US	Low-grade / equivocal squamous	
P07086	2020	2020-Q1	2020-03	Pandemic	Cytology (Pap)	55-59	55	Not performed	Satisfactory	NILM	NILM	Negative (NILM)	
P07087	2020	2020-Q1	2020-03	Pandemic	Cytology (Pap)	55-59	59	Not performed	Satisfactory	NILM	NILM	Negative (NILM)	
P07088	2020	2020-Q1	2020-03	Pandemic	Cytology (Pap)	40-44	42	Not performed	Satisfactory	NILM	NILM	Negative (NILM)	
P07089	2020	2020-Q3	2020-07	Pandemic	Cytology (Pap)	45-49	45	Not performed	Satisfactory	NILM	NILM	Negative (NILM)	
P07090	2020	2020-Q3	2020-07	Pandemic	Cytology (Pap)	45-49	45	Not performed	Satisfactory	NILM	NILM	Negative (NILM)	
P07091	2020	2020-Q3	2020-07	Pandemic	Cytology (Pap)	40-44	41	Not performed	Satisfactory	NILM	NILM	Negative (NILM)	
P07092	2020	2020-Q3	2020-07	Pandemic	Cytology (Pap)	40-44	41	Not performed	Satisfactory	NILM	NILM	Negative (NILM)	
P07093	2020	2020-Q3	2020-07	Pandemic	Cytology (Pap)	35-39	36	Not performed	Satisfactory	NILM	NILM	Negative (NILM)	
P07094	2020	2020-Q3	2020-07	Pandemic	Cytology (Pap)	35-39	35	Not performed	Satisfactory	NILM	NILM	Negative (NILM)	
P07095	2020	2020-Q3	2020-07	Pandemic	Cytology (Pap)	45-49	46	Not performed	Satisfactory	NILM	NILM	Negative (NILM)	
P07096	2020	2020-Q3	2020-07	Pandemic	Cytology (Pap)	45-49	47	Not performed	Satisfactory	NILM	NILM	Negative (NILM)	
P07097	2020	2020-Q3	2020-07	Pandemic	Cytology (Pap)	50-54	54	Not performed	Satisfactory	NILM	NILM	Negative (NILM)	
P07098	2020	2020-Q3	2020-07	Pandemic	Cytology (Pap)	30-34	30	Not performed	Satisfactory	Positive	LSIL	Low-grade / equivocal squamous	
P07099	2020	2020-Q3	2020-07	Pandemic	Cytology (Pap)	50-54	54	Not performed	Satisfactory	NILM	NILM	Negative (NILM)	
P07100	2020	2020-Q3	2020-07	Pandemic	Cytology (Pap)	55-59	57	Not performed	Satisfactory	NILM	NILM	Negative (NILM)	
P07101	2020	2020-Q3	2020-07	Pandemic	Cytology (Pap)	50-54	50	Not performed	Satisfactory	Positive	HSIL	High-grade squamous	
P07102	2020	2020-Q3	2020-07	Pandemic	Cytology (Pap)	40-44	40	Not performed	Unsatisfactory	Unsatisfactory	Unsatisfactory	Unsatisfactory	
P07103	2020	2020-Q3	2020-07	Pandemic	Cytology (Pap)	30-34	31	Not performed	Satisfactory	Positive	AGC	Glandular	
P07104	2020	2020-Q3	2020-07	Pandemic	Cytology (Pap)	40-44	41	Not performed	Satisfactory	NILM	NILM	Negative (NILM)	Candida
P07105	2020	2020-Q3	2020-07	Pandemic	Cytology (Pap)	30-34	34	Not performed	Satisfactory	NILM	NILM	Negative (NILM)	
P07106	2020	2020-Q3	2020-07	Pandemic	Cytology (Pap)	30-34	33	Not performed	Satisfactory	NILM	NILM	Negative (NILM)	
P07107	2020	2020-Q3	2020-07	Pandemic	Cytology (Pap)	45-49	46	Not performed	Satisfactory	NILM	NILM	Negative (NILM)	
P07108	2020	2020-Q3	2020-07	Pandemic	Cytology (Pap)	45-49	49	Not performed	Satisfactory	NILM	NILM	Negative (NILM)	
P07109	2020	2020-Q3	2020-07	Pandemic	Cytology (Pap)	60-64	61	Not performed	Satisfactory	NILM	NILM	Negative (NILM)	
P07110	2020	2020-Q3	2020-07	Pandemic	Cytology (Pap)	55-59	55	Not performed	Satisfactory	NILM	NILM	Negative (NILM)	
P07111	2020	2020-Q3	2020-07	Pandemic	Cytology (Pap)	35-39	39	Not performed	Satisfactory	NILM	NILM	Negative (NILM)	
P07112	2020	2020-Q3	2020-07	Pandemic	Cytology (Pap)	60-64	64	Not performed	Satisfactory	Positive	LSIL	Low-grade / equivocal squamous	
P07113	2020	2020-Q3	2020-07	Pandemic	Cytology (Pap)	45-49	49	Not performed	Unsatisfactory	Unsatisfactory	Unsatisfactory	Unsatisfactory	
P07114	2020	2020-Q3	2020-07	Pandemic	Cytology (Pap)	45-49	48	Not performed	Unsatisfactory	Unsatisfactory	Unsatisfactory	Unsatisfactory	
P07115	2020	2020-Q3	2020-07	Pandemic	Cytology (Pap)	45-49	46	Not performed	Satisfactory	NILM	NILM	Negative (NILM)	
P07116	2020	2020-Q3	2020-07	Pandemic	Cytology (Pap)	40-44	44	Not performed	Satisfactory	NILM	NILM	Negative (NILM)	
P07117	2020	2020-Q3	2020-07	Pandemic	Cytology (Pap)	60-64	60	Not performed	Unsatisfactory	Unsatisfactory	Unsatisfactory	Unsatisfactory	
P07118	2020	2020-Q3	2020-07	Pandemic	Cytology (Pap)	45-49	47	Not performed	Satisfactory	NILM	NILM	Negative (NILM)	

patient_id	year	quarter	month	period	primary_test	age_group	age_years	hpv_result	adequacy	result	bethesda	grade	infection
P07119	2020	2020-Q3	2020-07	Pandemic	Cytology (Pap)	55-59	59	Not performed	Satisfactory	NILM	NILM	Negative (NILM)	
P07120	2020	2020-Q3	2020-07	Pandemic	Cytology (Pap)	40-44	41	Not performed	Satisfactory	NILM	NILM	Negative (NILM)	
P07121	2020	2020-Q3	2020-07	Pandemic	Cytology (Pap)	45-49	47	Not performed	Satisfactory	NILM	NILM	Negative (NILM)	
P07122	2020	2020-Q3	2020-07	Pandemic	Cytology (Pap)	45-49	46	Not performed	Satisfactory	NILM	NILM	Negative (NILM)	
P07123	2020	2020-Q3	2020-07	Pandemic	Cytology (Pap)	45-49	47	Not performed	Satisfactory	NILM	NILM	Negative (NILM)	
P07124	2020	2020-Q3	2020-07	Pandemic	Cytology (Pap)	35-39	36	Not performed	Satisfactory	NILM	NILM	Negative (NILM)	
P07125	2020	2020-Q3	2020-07	Pandemic	Cytology (Pap)	30-34	34	Not performed	Satisfactory	NILM	NILM	Negative (NILM)	
P07126	2020	2020-Q3	2020-07	Pandemic	Cytology (Pap)	40-44	40	Not performed	Satisfactory	Positive	ASC-US	Low-grade / equivocal squamous	
P07127	2020	2020-Q3	2020-07	Pandemic	Cytology (Pap)	55-59	55	Not performed	Satisfactory	NILM	NILM	Negative (NILM)	
P07128	2020	2020-Q3	2020-07	Pandemic	Cytology (Pap)	45-49	45	Not performed	Satisfactory	NILM	NILM	Negative (NILM)	
P07129	2020	2020-Q3	2020-08	Pandemic	Cytology (Pap)	35-39	37	Not performed	Unsatisfactory	Unsatisfactory	Unsatisfactory	Unsatisfactory	
P07130	2020	2020-Q3	2020-08	Pandemic	Cytology (Pap)	35-39	37	Not performed	Satisfactory	NILM	NILM	Negative (NILM)	
P07131	2020	2020-Q3	2020-08	Pandemic	Cytology (Pap)	40-44	44	Not performed	Satisfactory	NILM	NILM	Negative (NILM)	
P07132	2020	2020-Q3	2020-08	Pandemic	Cytology (Pap)	25-29	27	Not performed	Satisfactory	NILM	NILM	Negative (NILM)	
P07133	2020	2020-Q3	2020-08	Pandemic	Cytology (Pap)	55-59	56	Not performed	Satisfactory	NILM	NILM	Negative (NILM)	
P07134	2020	2020-Q3	2020-08	Pandemic	Cytology (Pap)	30-34	30	Not performed	Satisfactory	NILM	NILM	Negative (NILM)	
P07135	2020	2020-Q3	2020-08	Pandemic	Cytology (Pap)	50-54	50	Not performed	Satisfactory	NILM	NILM	Negative (NILM)	
P07136	2020	2020-Q3	2020-08	Pandemic	Cytology (Pap)	30-34	31	Not performed	Satisfactory	Positive	LSIL	Low-grade / equivocal squamous	
P07137	2020	2020-Q3	2020-08	Pandemic	Cytology (Pap)	40-44	41	Not performed	Satisfactory	NILM	NILM	Negative (NILM)	
P07138	2020	2020-Q3	2020-08	Pandemic	Cytology (Pap)	30-34	34	Not performed	Satisfactory	NILM	NILM	Negative (NILM)	
P07139	2020	2020-Q3	2020-08	Pandemic	Cytology (Pap)	30-34	34	Not performed	Satisfactory	NILM	NILM	Negative (NILM)	
P07140	2020	2020-Q3	2020-08	Pandemic	Cytology (Pap)	45-49	49	Not performed	Satisfactory	NILM	NILM	Negative (NILM)	
P07141	2020	2020-Q3	2020-08	Pandemic	Cytology (Pap)	45-49	48	Not performed	Satisfactory	NILM	NILM	Negative (NILM)	
P07142	2020	2020-Q3	2020-08	Pandemic	Cytology (Pap)	40-44	40	Not performed	Satisfactory	NILM	NILM	Negative (NILM)	
P07143	2020	2020-Q3	2020-08	Pandemic	Cytology (Pap)	30-34	32	Not performed	Satisfactory	NILM	NILM	Negative (NILM)	
P07144	2020	2020-Q3	2020-08	Pandemic	Cytology (Pap)	55-59	59	Not performed	Satisfactory	NILM	NILM	Negative (NILM)	
P07145	2020	2020-Q3	2020-08	Pandemic	Cytology (Pap)	55-59	55	Not performed	Satisfactory	NILM	NILM	Negative (NILM)	
P07146	2020	2020-Q3	2020-08	Pandemic	Cytology (Pap)	50-54	54	Not performed	Satisfactory	NILM	NILM	Negative (NILM)	
P07147	2020	2020-Q3	2020-08	Pandemic	Cytology (Pap)	50-54	50	Not performed	Satisfactory	NILM	NILM	Negative (NILM)	
P07148	2020	2020-Q3	2020-08	Pandemic	Cytology (Pap)	40-44	40	Not performed	Satisfactory	NILM	NILM	Negative (NILM)	
P07149	2020	2020-Q3	2020-08	Pandemic	Cytology (Pap)	40-44	41	Not performed	Satisfactory	NILM	NILM	Negative (NILM)	
P07150	2020	2020-Q3	2020-08	Pandemic	Cytology (Pap)	35-39	38	Not performed	Satisfactory	NILM	NILM	Negative (NILM)	
P07151	2020	2020-Q3	2020-08	Pandemic	Cytology (Pap)	35-39	38	Not performed	Satisfactory	NILM	NILM	Negative (NILM)	
P07152	2020	2020-Q3	2020-08	Pandemic	Cytology (Pap)	45-49	46	Not performed	Satisfactory	NILM	NILM	Negative (NILM)	Candida
P07153	2020	2020-Q3	2020-08	Pandemic	Cytology (Pap)	55-59	57	Not performed	Satisfactory	NILM	NILM	Negative (NILM)	
P07154	2020	2020-Q3	2020-08	Pandemic	Cytology (Pap)	55-59	56	Not performed	Satisfactory	NILM	NILM	Negative (NILM)	
P07155	2020	2020-Q3	2020-08	Pandemic	Cytology (Pap)	50-54	53	Not performed	Unsatisfactory	Unsatisfactory	Unsatisfactory	Unsatisfactory	
P07156	2020	2020-Q3	2020-08	Pandemic	Cytology (Pap)	35-39	37	Not performed	Satisfactory	NILM	NILM	Negative (NILM)	
P07157	2020	2020-Q3	2020-08	Pandemic	Cytology (Pap)	30-34	31	Not performed	Satisfactory	Positive	LSIL	Low-grade / equivocal squamous	
P07158	2020	2020-Q3	2020-08	Pandemic	Cytology (Pap)	45-49	49	Not performed	Satisfactory	NILM	NILM	Negative (NILM)	
P07159	2020	2020-Q3	2020-08	Pandemic	Cytology (Pap)	40-44	44	Not performed	Satisfactory	NILM	NILM	Negative (NILM)	
P07160	2020	2020-Q3	2020-08	Pandemic	Cytology (Pap)	40-44	43	Not performed	Satisfactory	NILM	NILM	Negative (NILM)	
P07161	2020	2020-Q3	2020-08	Pandemic	Cytology (Pap)	60-64	61	Not performed	Satisfactory	NILM	NILM	Negative (NILM)	
P07162	2020	2020-Q3	2020-08	Pandemic	Cytology (Pap)	35-39	38	Not performed	Satisfactory	NILM	NILM	Negative (NILM)	
P07163	2020	2020-Q3	2020-08	Pandemic	Cytology (Pap)	55-59	59	Not performed	Satisfactory	NILM	NILM	Negative (NILM)	
P07164	2020	2020-Q3	2020-08	Pandemic	Cytology (Pap)	30-34	32	Not performed	Satisfactory	NILM	NILM	Negative (NILM)	
P07165	2020	2020-Q3	2020-08	Pandemic	Cytology (Pap)	35-39	37	Not performed	Satisfactory	NILM	NILM	Negative (NILM)	
P07166	2020	2020-Q3	2020-08	Pandemic	Cytology (Pap)	40-44	41	Not performed	Satisfactory	NILM	NILM	Negative (NILM)	
P07167	2020	2020-Q3	2020-08	Pandemic	Cytology (Pap)	25-29	26	Not performed	Satisfactory	NILM	NILM	Negative (NILM)	Gardnerella
P07168	2020	2020-Q3	2020-08	Pandemic	Cytology (Pap)	35-39	36	Not performed	Satisfactory	NILM	NILM	Negative (NILM)	Trichomonas
P07169	2020	2020-Q3	2020-09	Pandemic	Cytology (Pap)	55-59	57	Not performed	Satisfactory	NILM	NILM	Negative (NILM)	
P07170	2020	2020-Q3	2020-09	Pandemic	Cytology (Pap)	40-44	41	Not performed	Satisfactory	Positive	ASC-US	Low-grade / equivocal squamous	
P07171	2020	2020-Q3	2020-09	Pandemic	Cytology (Pap)	50-54	54	Not performed	Satisfactory	NILM	NILM	Negative (NILM)	
P07172	2020	2020-Q3	2020-09	Pandemic	Cytology (Pap)	25-29	27	Not performed	Satisfactory	NILM	NILM	Negative (NILM)	
P07173	2020	2020-Q3	2020-09	Pandemic	Cytology (Pap)	50-54	53	Not performed	Satisfactory	NILM	NILM	Negative (NILM)	
P07174	2020	2020-Q3	2020-09	Pandemic	Cytology (Pap)	50-54	53	Not performed	Satisfactory	NILM	NILM	Negative (NILM)	
P07175	2020	2020-Q3	2020-09	Pandemic	Cytology (Pap)	35-39	35	Not performed	Satisfactory	NILM	NILM	Negative (NILM)	
P07176	2020	2020-Q3	2020-09	Pandemic	Cytology (Pap)	40-44	42	Not performed	Satisfactory	NILM	NILM	Negative (NILM)	
P07177	2020	2020-Q3	2020-09	Pandemic	Cytology (Pap)	30-34	30	Not performed	Satisfactory	NILM	NILM	Negative (NILM)	
P07178	2020	2020-Q3	2020-09	Pandemic	Cytology (Pap)	40-44	41	Not performed	Satisfactory	NILM	NILM	Negative (NILM)	
P07179	2020	2020-Q3	2020-09	Pandemic	Cytology (Pap)	35-39	37	Not performed	Satisfactory	NILM	NILM	Negative (NILM)	
P07180	2020	2020-Q3	2020-09	Pandemic	Cytology (Pap)	45-49	45	Not performed	Satisfactory	NILM	NILM	Negative (NILM)	Candida
P07181	2020	2020-Q3	2020-09	Pandemic	Cytology (Pap)	40-44	40	Not performed	Satisfactory	NILM	NILM	Negative (NILM)	Trichomonas
P07182	2020	2020-Q3	2020-09	Pandemic	Cytology (Pap)	30-34	34	Not performed	Satisfactory	NILM	NILM	Negative (NILM)	
P07183	2020	2020-Q3	2020-09	Pandemic	Cytology (Pap)	50-54	52	Not performed	Satisfactory	NILM	NILM	Negative (NILM)	
P07184	2020	2020-Q3	2020-09	Pandemic	Cytology (Pap)	50-54	53	Not performed	Satisfactory	NILM	NILM	Negative (NILM)	
P07185	2020	2020-Q3	2020-09	Pandemic	Cytology (Pap)	60-64	64	Not performed	Satisfactory	NILM	NILM	Negative (NILM)	
P07186	2020	2020-Q3	2020-09	Pandemic	Cytology (Pap)	50-54	50	Not performed	Satisfactory	NILM	NILM	Negative (NILM)	
P07187	2020	2020-Q3	2020-09	Pandemic	Cytology (Pap)	55-59	57	Not performed	Satisfactory	Positive	LSIL	Low-grade / equivocal squamous	
P07188	2020	2020-Q3	2020-09	Pandemic	Cytology (Pap)	45-49	49	Not performed	Satisfactory	NILM	NILM	Negative (NILM)	
P07189	2020	2020-Q3	2020-09	Pandemic	Cytology (Pap)	40-44	43	Not performed	Satisfactory	NILM	NILM	Negative (NILM)	
P07190	2020	2020-Q3	2020-09	Pandemic	Cytology (Pap)	40-44	44	Not performed	Satisfactory	NILM	NILM	Negative (NILM)	
P07191	2020	2020-Q3	2020-09	Pandemic	Cytology (Pap)	30-34	33	Not performed	Satisfactory	NILM	NILM	Negative (NILM)	
P07192	2020	2020-Q3	2020-09	Pandemic	Cytology (Pap)	45-49	46	Not performed	Satisfactory	NILM	NILM	Negative (NILM)	
P07193	2020	2020-Q3	2020-09	Pandemic	Cytology (Pap)	50-54	51	Not performed	Satisfactory	Positive	HSIL	High-grade squamous	
P07194	2020	2020-Q3	2020-09	Pandemic	Cytology (Pap)	55-59	56	Not performed	Satisfactory	Positive	ASC-H	High-grade squamous	
P07195	2020	2020-Q3	2020-09	Pandemic	Cytology (Pap)	25-29	29	Not performed	Satisfactory	NILM	NILM	Negative (NILM)	
P07196	2020	2020-Q3	2020-09	Pandemic	Cytology (Pap)	40-44	42	Not performed	Satisfactory	Positive	AGC	Glandular	
P07197	2020	2020-Q3	2020-09	Pandemic	Cytology (Pap)	35-39	38	Not performed	Satisfactory	NILM	NILM	Negative (NILM)	
P07198	2020	2020-Q3	2020-09	Pandemic	Cytology (Pap)	30-34	32	Not performed	Satisfactory	NILM	NILM	Negative (NILM)	

patient_id	year	quarter	month	period	primary_test	age_group	age_years	hpv_result	adequacy	result	bethesda	grade	infection
P07199	2020	2020-Q3	2020-09	Pandemic	Cytology (Pap)	40-44	41	Not performed	Satisfactory	Positive	ASC-US	Low-grade / equivocal squamous	
P07200	2020	2020-Q3	2020-09	Pandemic	Cytology (Pap)	40-44	41	Not performed	Satisfactory	NILM	NILM	Negative (NILM)	
P07201	2020	2020-Q3	2020-09	Pandemic	Cytology (Pap)	45-49	46	Not performed	Satisfactory	NILM	NILM	Negative (NILM)	
P07202	2020	2020-Q3	2020-09	Pandemic	Cytology (Pap)	40-44	42	Not performed	Satisfactory	NILM	NILM	Negative (NILM)	
P07203	2020	2020-Q3	2020-09	Pandemic	Cytology (Pap)	35-39	39	Not performed	Satisfactory	NILM	NILM	Negative (NILM)	
P07204	2020	2020-Q3	2020-09	Pandemic	Cytology (Pap)	60-64	63	Not performed	Satisfactory	NILM	NILM	Negative (NILM)	
P07205	2020	2020-Q3	2020-09	Pandemic	Cytology (Pap)	35-39	35	Not performed	Satisfactory	NILM	NILM	Negative (NILM)	
P07206	2020	2020-Q3	2020-09	Pandemic	Cytology (Pap)	25-29	25	Not performed	Satisfactory	Positive	ASC-US	Low-grade / equivocal squamous	
P07207	2020	2020-Q3	2020-09	Pandemic	Cytology (Pap)	55-59	59	Not performed	Satisfactory	NILM	NILM	Negative (NILM)	
P07208	2020	2020-Q4	2020-10	Pandemic	Cytology (Pap)	45-49	47	Not performed	Satisfactory	Positive	ASC-US	Low-grade / equivocal squamous	
P07209	2020	2020-Q4	2020-10	Pandemic	Cytology (Pap)	40-44	41	Not performed	Satisfactory	NILM	NILM	Negative (NILM)	
P07210	2020	2020-Q4	2020-10	Pandemic	Cytology (Pap)	40-44	41	Not performed	Satisfactory	Positive	LSIL	Low-grade / equivocal squamous	
P07211	2020	2020-Q4	2020-10	Pandemic	Cytology (Pap)	45-49	48	Not performed	Satisfactory	NILM	NILM	Negative (NILM)	
P07212	2020	2020-Q4	2020-10	Pandemic	Cytology (Pap)	40-44	43	Not performed	Satisfactory	Positive	ASC-US	Low-grade / equivocal squamous	
P07213	2020	2020-Q4	2020-10	Pandemic	Cytology (Pap)	25-29	25	Not performed	Satisfactory	NILM	NILM	Negative (NILM)	
P07214	2020	2020-Q4	2020-10	Pandemic	Cytology (Pap)	55-59	55	Not performed	Satisfactory	Positive	AGC	Glandular	
P07215	2020	2020-Q4	2020-10	Pandemic	Cytology (Pap)	25-29	29	Not performed	Satisfactory	Positive	LSIL	Low-grade / equivocal squamous	
P07216	2020	2020-Q4	2020-10	Pandemic	Cytology (Pap)	45-49	49	Not performed	Satisfactory	NILM	NILM	Negative (NILM)	
P07217	2020	2020-Q4	2020-10	Pandemic	Cytology (Pap)	50-54	52	Not performed	Satisfactory	NILM	NILM	Negative (NILM)	
P07218	2020	2020-Q4	2020-10	Pandemic	Cytology (Pap)	40-44	44	Not performed	Satisfactory	NILM	NILM	Negative (NILM)	
P07219	2020	2020-Q4	2020-10	Pandemic	Cytology (Pap)	30-34	32	Not performed	Satisfactory	NILM	NILM	Negative (NILM)	
P07220	2020	2020-Q4	2020-10	Pandemic	Cytology (Pap)	40-44	40	Not performed	Satisfactory	NILM	NILM	Negative (NILM)	
P07221	2020	2020-Q4	2020-10	Pandemic	Cytology (Pap)	60-64	64	Not performed	Satisfactory	NILM	NILM	Negative (NILM)	Trichomonas
P07222	2020	2020-Q4	2020-10	Pandemic	Cytology (Pap)	30-34	30	Not performed	Satisfactory	NILM	NILM	Negative (NILM)	
P07223	2020	2020-Q4	2020-10	Pandemic	Cytology (Pap)	35-39	35	Not performed	Satisfactory	NILM	NILM	Negative (NILM)	
P07224	2020	2020-Q4	2020-10	Pandemic	Cytology (Pap)	50-54	54	Not performed	Satisfactory	Positive	ASC-H	High-grade squamous	
P07225	2020	2020-Q4	2020-10	Pandemic	Cytology (Pap)	40-44	42	Not performed	Satisfactory	NILM	NILM	Negative (NILM)	
P07226	2020	2020-Q4	2020-10	Pandemic	Cytology (Pap)	50-54	54	Not performed	Satisfactory	NILM	NILM	Negative (NILM)	
P07227	2020	2020-Q4	2020-10	Pandemic	Cytology (Pap)	30-34	32	Not performed	Satisfactory	NILM	NILM	Negative (NILM)	
P07228	2020	2020-Q4	2020-10	Pandemic	Cytology (Pap)	55-59	59	Not performed	Satisfactory	NILM	NILM	Negative (NILM)	Candida
P07229	2020	2020-Q4	2020-10	Pandemic	Cytology (Pap)	55-59	55	Not performed	Satisfactory	Positive	ASC-US	Low-grade / equivocal squamous	
P07230	2020	2020-Q4	2020-10	Pandemic	Cytology (Pap)	40-44	43	Not performed	Satisfactory	NILM	NILM	Negative (NILM)	
P07231	2020	2020-Q4	2020-10	Pandemic	Cytology (Pap)	45-49	46	Not performed	Satisfactory	NILM	NILM	Negative (NILM)	
P07232	2020	2020-Q4	2020-10	Pandemic	Cytology (Pap)	55-59	58	Not performed	Satisfactory	NILM	NILM	Negative (NILM)	
P07233	2020	2020-Q4	2020-10	Pandemic	Cytology (Pap)	30-34	33	Not performed	Satisfactory	NILM	NILM	Negative (NILM)	
P07234	2020	2020-Q4	2020-10	Pandemic	Cytology (Pap)	30-34	34	Not performed	Satisfactory	Positive	LSIL	Low-grade / equivocal squamous	
P07235	2020	2020-Q4	2020-10	Pandemic	Cytology (Pap)	40-44	41	Not performed	Satisfactory	NILM	NILM	Negative (NILM)	
P07236	2020	2020-Q4	2020-10	Pandemic	Cytology (Pap)	55-59	57	Not performed	Satisfactory	NILM	NILM	Negative (NILM)	
P07237	2020	2020-Q4	2020-10	Pandemic	Cytology (Pap)	30-34	31	Not performed	Satisfactory	Positive	LSIL	Low-grade / equivocal squamous	
P07238	2020	2020-Q4	2020-10	Pandemic	Cytology (Pap)	55-59	56	Not performed	Satisfactory	Positive	LSIL	Low-grade / equivocal squamous	
P07239	2020	2020-Q4	2020-10	Pandemic	Cytology (Pap)	25-29	26	Not performed	Satisfactory	NILM	NILM	Negative (NILM)	
P07240	2020	2020-Q4	2020-10	Pandemic	Cytology (Pap)	60-64	64	Not performed	Satisfactory	Positive	LSIL	Low-grade / equivocal squamous	
P07241	2020	2020-Q4	2020-10	Pandemic	Cytology (Pap)	35-39	38	Not performed	Satisfactory	NILM	NILM	Negative (NILM)	
P07242	2020	2020-Q4	2020-10	Pandemic	Cytology (Pap)	50-54	50	Not performed	Satisfactory	NILM	NILM	Negative (NILM)	
P07243	2020	2020-Q4	2020-10	Pandemic	Cytology (Pap)	35-39	36	Not performed	Satisfactory	NILM	NILM	Negative (NILM)	
P07244	2020	2020-Q4	2020-10	Pandemic	Cytology (Pap)	40-44	43	Not performed	Satisfactory	NILM	NILM	Negative (NILM)	
P07245	2020	2020-Q4	2020-10	Pandemic	Cytology (Pap)	45-49	49	Not performed	Satisfactory	NILM	NILM	Negative (NILM)	
P07246	2020	2020-Q4	2020-10	Pandemic	Cytology (Pap)	45-49	45	Not performed	Satisfactory	NILM	NILM	Negative (NILM)	
P07247	2020	2020-Q4	2020-10	Pandemic	Cytology (Pap)	55-59	57	Not performed	Satisfactory	NILM	NILM	Negative (NILM)	
P07248	2020	2020-Q4	2020-10	Pandemic	Cytology (Pap)	60-64	61	Not performed	Satisfactory	NILM	NILM	Negative (NILM)	
P07249	2020	2020-Q4	2020-10	Pandemic	Cytology (Pap)	35-39	35	Not performed	Satisfactory	NILM	NILM	Negative (NILM)	
P07250	2020	2020-Q4	2020-10	Pandemic	Cytology (Pap)	30-34	34	Not performed	Satisfactory	NILM	NILM	Negative (NILM)	
P07251	2020	2020-Q4	2020-10	Pandemic	Cytology (Pap)	35-39	38	Not performed	Satisfactory	NILM	NILM	Negative (NILM)	
P07252	2020	2020-Q4	2020-10	Pandemic	Cytology (Pap)	35-39	37	Not performed	Satisfactory	NILM	NILM	Negative (NILM)	
P07253	2020	2020-Q4	2020-10	Pandemic	Cytology (Pap)	45-49	47	Not performed	Satisfactory	NILM	NILM	Negative (NILM)	
P07254	2020	2020-Q4	2020-10	Pandemic	Cytology (Pap)	40-44	43	Not performed	Satisfactory	NILM	NILM	Negative (NILM)	
P07255	2020	2020-Q4	2020-10	Pandemic	Cytology (Pap)	40-44	40	Not performed	Satisfactory	NILM	NILM	Negative (NILM)	Gardnerella
P07256	2020	2020-Q4	2020-10	Pandemic	Cytology (Pap)	60-64	61	Not performed	Satisfactory	NILM	NILM	Negative (NILM)	
P07257	2020	2020-Q4	2020-10	Pandemic	Cytology (Pap)	55-59	58	Not performed	Satisfactory	NILM	NILM	Negative (NILM)	
P07258	2020	2020-Q4	2020-10	Pandemic	Cytology (Pap)	30-34	31	Not performed	Satisfactory	Positive	ASC-US	Low-grade / equivocal squamous	
P07259	2020	2020-Q4	2020-10	Pandemic	Cytology (Pap)	40-44	40	Not performed	Satisfactory	NILM	NILM	Negative (NILM)	
P07260	2020	2020-Q4	2020-10	Pandemic	Cytology (Pap)	40-44	42	Not performed	Satisfactory	Positive	ASC-US	Low-grade / equivocal squamous	
P07261	2020	2020-Q4	2020-10	Pandemic	Cytology (Pap)	35-39	39	Not performed	Satisfactory	NILM	NILM	Negative (NILM)	
P07262	2020	2020-Q4	2020-10	Pandemic	Cytology (Pap)	35-39	38	Not performed	Satisfactory	NILM	NILM	Negative (NILM)	
P07263	2020	2020-Q4	2020-10	Pandemic	Cytology (Pap)	45-49	49	Not performed	Satisfactory	NILM	NILM	Negative (NILM)	
P07264	2020	2020-Q4	2020-10	Pandemic	Cytology (Pap)	45-49	48	Not performed	Satisfactory	NILM	NILM	Negative (NILM)	
P07265	2020	2020-Q4	2020-10	Pandemic	Cytology (Pap)	50-54	54	Not performed	Satisfactory	NILM	NILM	Negative (NILM)	
P07266	2020	2020-Q4	2020-10	Pandemic	Cytology (Pap)	45-49	46	Not performed	Satisfactory	Positive	ASC-US	Low-grade / equivocal squamous	
P07267	2020	2020-Q4	2020-10	Pandemic	Cytology (Pap)	35-39	35	Not performed	Satisfactory	NILM	NILM	Negative (NILM)	
P07268	2020	2020-Q4	2020-10	Pandemic	Cytology (Pap)	40-44	43	Not performed	Satisfactory	NILM	NILM	Negative (NILM)	
P07269	2020	2020-Q4	2020-10	Pandemic	Cytology (Pap)	40-44	44	Not performed	Satisfactory	NILM	NILM	Negative (NILM)	
P07270	2020	2020-Q4	2020-10	Pandemic	Cytology (Pap)	40-44	41	Not performed	Satisfactory	NILM	NILM	Negative (NILM)	
P07271	2020	2020-Q4	2020-10	Pandemic	Cytology (Pap)	55-59	55	Not performed	Satisfactory	NILM	NILM	Negative (NILM)	
P07272	2020	2020-Q4	2020-10	Pandemic	Cytology (Pap)	45-49	46	Not performed	Satisfactory	NILM	NILM	Negative (NILM)	
P07273	2020	2020-Q4	2020-10	Pandemic	Cytology (Pap)	45-49	47	Not performed	Unsatisfactory	Unsatisfactory	Unsatisfactory	Unsatisfactory	
P07274	2020	2020-Q4	2020-10	Pandemic	Cytology (Pap)	55-59	57	Not performed	Unsatisfactory	Unsatisfactory	Unsatisfactory	Unsatisfactory	
P07275	2020	2020-Q4	2020-10	Pandemic	Cytology (Pap)	25-29	25	Not performed	Satisfactory	Positive	ASC-US	Low-grade / equivocal squamous	
P07276	2020	2020-Q4	2020-10	Pandemic	Cytology (Pap)	55-59	57	Not performed	Satisfactory	NILM	NILM	Negative (NILM)	
P07277	2020	2020-Q4	2020-10	Pandemic	Cytology (Pap)	40-44	41	Not performed	Satisfactory	NILM	NILM	Negative (NILM)	
P07278	2020	2020-Q4	2020-10	Pandemic	Cytology (Pap)	50-54	54	Not performed	Satisfactory	NILM	NILM	Negative (NILM)	

patient_id	year	quarter	month	period	primary_test	age_group	age_years	hpv_result	adequacy	result	bethesda	grade	infection
P07279	2020	2020-Q4	2020-10	Pandemic	Cytology (Pap)	50-54	54	Not performed	Satisfactory	NILM	NILM	Negative (NILM)	
P07280	2020	2020-Q4	2020-10	Pandemic	Cytology (Pap)	45-49	49	Not performed	Satisfactory	NILM	NILM	Negative (NILM)	
P07281	2020	2020-Q4	2020-10	Pandemic	Cytology (Pap)	45-49	48	Not performed	Satisfactory	NILM	NILM	Negative (NILM)	
P07282	2020	2020-Q4	2020-10	Pandemic	Cytology (Pap)	35-39	36	Not performed	Satisfactory	NILM	NILM	Negative (NILM)	
P07283	2020	2020-Q4	2020-10	Pandemic	Cytology (Pap)	40-44	41	Not performed	Satisfactory	NILM	NILM	Negative (NILM)	
P07284	2020	2020-Q4	2020-10	Pandemic	Cytology (Pap)	30-34	32	Not performed	Unsatisfactory	Unsatisfactory	Unsatisfactory	Unsatisfactory	
P07285	2020	2020-Q4	2020-10	Pandemic	Cytology (Pap)	35-39	39	Not performed	Satisfactory	NILM	NILM	Negative (NILM)	
P07286	2020	2020-Q4	2020-10	Pandemic	Cytology (Pap)	40-44	44	Not performed	Satisfactory	NILM	NILM	Negative (NILM)	
P07287	2020	2020-Q4	2020-10	Pandemic	Cytology (Pap)	40-44	43	Not performed	Satisfactory	Positive	LSIL	Low-grade / equivocal squamous	
P07288	2020	2020-Q4	2020-10	Pandemic	Cytology (Pap)	35-39	38	Not performed	Satisfactory	NILM	NILM	Negative (NILM)	
P07289	2020	2020-Q4	2020-10	Pandemic	Cytology (Pap)	50-54	54	Not performed	Satisfactory	NILM	NILM	Negative (NILM)	
P07290	2020	2020-Q4	2020-10	Pandemic	Cytology (Pap)	45-49	46	Not performed	Satisfactory	NILM	NILM	Negative (NILM)	
P07291	2020	2020-Q4	2020-10	Pandemic	Cytology (Pap)	50-54	53	Not performed	Satisfactory	NILM	NILM	Negative (NILM)	
P07292	2020	2020-Q4	2020-10	Pandemic	Cytology (Pap)	45-49	46	Not performed	Satisfactory	NILM	NILM	Negative (NILM)	
P07293	2020	2020-Q4	2020-10	Pandemic	Cytology (Pap)	50-54	52	Not performed	Satisfactory	NILM	NILM	Negative (NILM)	
P07294	2020	2020-Q4	2020-10	Pandemic	Cytology (Pap)	45-49	46	Not performed	Satisfactory	NILM	NILM	Negative (NILM)	
P07295	2020	2020-Q4	2020-10	Pandemic	Cytology (Pap)	30-34	33	Not performed	Satisfactory	NILM	NILM	Negative (NILM)	
P07296	2020	2020-Q4	2020-10	Pandemic	Cytology (Pap)	50-54	51	Not performed	Satisfactory	NILM	NILM	Negative (NILM)	
P07297	2020	2020-Q4	2020-10	Pandemic	Cytology (Pap)	55-59	57	Not performed	Satisfactory	NILM	NILM	Negative (NILM)	
P07298	2020	2020-Q4	2020-10	Pandemic	Cytology (Pap)	45-49	48	Not performed	Satisfactory	Positive	LSIL	Low-grade / equivocal squamous	
P07299	2020	2020-Q4	2020-11	Pandemic	Cytology (Pap)	35-39	36	Not performed	Satisfactory	NILM	NILM	Negative (NILM)	
P07300	2020	2020-Q4	2020-11	Pandemic	Cytology (Pap)	50-54	52	Not performed	Unsatisfactory	Unsatisfactory	Unsatisfactory	Unsatisfactory	
P07301	2020	2020-Q4	2020-11	Pandemic	Cytology (Pap)	35-39	38	Not performed	Satisfactory	NILM	NILM	Negative (NILM)	
P07302	2020	2020-Q4	2020-11	Pandemic	Cytology (Pap)	35-39	35	Not performed	Satisfactory	NILM	NILM	Negative (NILM)	
P07303	2020	2020-Q4	2020-11	Pandemic	Cytology (Pap)	35-39	38	Not performed	Satisfactory	NILM	NILM	Negative (NILM)	Candida
P07304	2020	2020-Q4	2020-11	Pandemic	Cytology (Pap)	35-39	38	Not performed	Satisfactory	NILM	NILM	Negative (NILM)	
P07305	2020	2020-Q4	2020-11	Pandemic	Cytology (Pap)	30-34	33	Not performed	Satisfactory	NILM	NILM	Negative (NILM)	
P07306	2020	2020-Q4	2020-11	Pandemic	Cytology (Pap)	30-34	32	Not performed	Satisfactory	NILM	NILM	Negative (NILM)	
P07307	2020	2020-Q4	2020-11	Pandemic	Cytology (Pap)	25-29	29	Not performed	Satisfactory	Positive	HSIL	High-grade squamous	
P07308	2020	2020-Q4	2020-11	Pandemic	Cytology (Pap)	40-44	41	Not performed	Satisfactory	Positive	ASC-US	Low-grade / equivocal squamous	
P07309	2020	2020-Q4	2020-11	Pandemic	Cytology (Pap)	25-29	27	Not performed	Satisfactory	NILM	NILM	Negative (NILM)	
P07310	2020	2020-Q4	2020-11	Pandemic	Cytology (Pap)	45-49	46	Not performed	Satisfactory	Positive	ASC-US	Low-grade / equivocal squamous	Gardnerella
P07311	2020	2020-Q4	2020-11	Pandemic	Cytology (Pap)	45-49	47	Not performed	Satisfactory	NILM	NILM	Negative (NILM)	
P07312	2020	2020-Q4	2020-11	Pandemic	Cytology (Pap)	35-39	36	Not performed	Satisfactory	NILM	NILM	Negative (NILM)	
P07313	2020	2020-Q4	2020-11	Pandemic	Cytology (Pap)	40-44	41	Not performed	Satisfactory	NILM	NILM	Negative (NILM)	
P07314	2020	2020-Q4	2020-11	Pandemic	Cytology (Pap)	35-39	38	Not performed	Satisfactory	NILM	NILM	Negative (NILM)	
P07315	2020	2020-Q4	2020-11	Pandemic	Cytology (Pap)	55-59	56	Not performed	Satisfactory	NILM	NILM	Negative (NILM)	
P07316	2020	2020-Q4	2020-11	Pandemic	Cytology (Pap)	40-44	43	Not performed	Satisfactory	NILM	NILM	Negative (NILM)	
P07317	2020	2020-Q4	2020-11	Pandemic	Cytology (Pap)	40-44	41	Not performed	Satisfactory	NILM	NILM	Negative (NILM)	
P07318	2020	2020-Q4	2020-11	Pandemic	Cytology (Pap)	35-39	39	Not performed	Satisfactory	NILM	NILM	Negative (NILM)	
P07319	2020	2020-Q4	2020-11	Pandemic	Cytology (Pap)	55-59	57	Not performed	Satisfactory	Positive	ASC-H	High-grade squamous	
P07320	2020	2020-Q4	2020-11	Pandemic	Cytology (Pap)	55-59	57	Not performed	Satisfactory	NILM	NILM	Negative (NILM)	
P07321	2020	2020-Q4	2020-11	Pandemic	Cytology (Pap)	40-44	43	Not performed	Satisfactory	Positive	ASC-US	Low-grade / equivocal squamous	
P07322	2020	2020-Q4	2020-11	Pandemic	Cytology (Pap)	30-34	31	Not performed	Satisfactory	NILM	NILM	Negative (NILM)	
P07323	2020	2020-Q4	2020-11	Pandemic	Cytology (Pap)	25-29	28	Not performed	Satisfactory	NILM	NILM	Negative (NILM)	
P07324	2020	2020-Q4	2020-11	Pandemic	Cytology (Pap)	50-54	50	Not performed	Satisfactory	NILM	NILM	Negative (NILM)	
P07325	2020	2020-Q4	2020-11	Pandemic	Cytology (Pap)	45-49	45	Not performed	Satisfactory	NILM	NILM	Negative (NILM)	
P07326	2020	2020-Q4	2020-11	Pandemic	Cytology (Pap)	30-34	34	Not performed	Satisfactory	NILM	NILM	Negative (NILM)	
P07327	2020	2020-Q4	2020-11	Pandemic	Cytology (Pap)	50-54	54	Not performed	Satisfactory	NILM	NILM	Negative (NILM)	
P07328	2020	2020-Q4	2020-11	Pandemic	Cytology (Pap)	50-54	50	Not performed	Satisfactory	NILM	NILM	Negative (NILM)	
P07329	2020	2020-Q4	2020-11	Pandemic	Cytology (Pap)	55-59	57	Not performed	Satisfactory	Positive	ASC-US	Low-grade / equivocal squamous	
P07330	2020	2020-Q4	2020-11	Pandemic	Cytology (Pap)	40-44	42	Not performed	Satisfactory	NILM	NILM	Negative (NILM)	
P07331	2020	2020-Q4	2020-11	Pandemic	Cytology (Pap)	40-44	42	Not performed	Satisfactory	NILM	NILM	Negative (NILM)	
P07332	2020	2020-Q4	2020-11	Pandemic	Cytology (Pap)	60-64	62	Not performed	Satisfactory	NILM	NILM	Negative (NILM)	Candida
P07333	2020	2020-Q4	2020-11	Pandemic	Cytology (Pap)	40-44	41	Not performed	Satisfactory	NILM	NILM	Negative (NILM)	
P07334	2020	2020-Q4	2020-11	Pandemic	Cytology (Pap)	25-29	29	Not performed	Satisfactory	NILM	NILM	Negative (NILM)	
P07335	2020	2020-Q4	2020-11	Pandemic	Cytology (Pap)	50-54	52	Not performed	Satisfactory	NILM	NILM	Negative (NILM)	
P07336	2020	2020-Q4	2020-11	Pandemic	Cytology (Pap)	45-49	45	Not performed	Satisfactory	Positive	HSIL	High-grade squamous	
P07337	2020	2020-Q4	2020-11	Pandemic	Cytology (Pap)	60-64	60	Not performed	Satisfactory	NILM	NILM	Negative (NILM)	
P07338	2020	2020-Q4	2020-11	Pandemic	Cytology (Pap)	50-54	51	Not performed	Satisfactory	NILM	NILM	Negative (NILM)	
P07339	2020	2020-Q4	2020-11	Pandemic	Cytology (Pap)	40-44	43	Not performed	Satisfactory	NILM	NILM	Negative (NILM)	
P07340	2020	2020-Q4	2020-11	Pandemic	Cytology (Pap)	40-44	40	Not performed	Satisfactory	NILM	NILM	Negative (NILM)	Gardnerella
P07341	2020	2020-Q4	2020-11	Pandemic	Cytology (Pap)	45-49	47	Not performed	Satisfactory	Positive	LSIL	Low-grade / equivocal squamous	
P07342	2020	2020-Q4	2020-11	Pandemic	Cytology (Pap)	35-39	35	Not performed	Satisfactory	NILM	NILM	Negative (NILM)	
P07343	2020	2020-Q4	2020-11	Pandemic	Cytology (Pap)	60-64	63	Not performed	Satisfactory	NILM	NILM	Negative (NILM)	
P07344	2020	2020-Q4	2020-11	Pandemic	Cytology (Pap)	35-39	39	Not performed	Satisfactory	NILM	NILM	Negative (NILM)	
P07345	2020	2020-Q4	2020-11	Pandemic	Cytology (Pap)	55-59	56	Not performed	Satisfactory	NILM	NILM	Negative (NILM)	
P07346	2020	2020-Q4	2020-11	Pandemic	Cytology (Pap)	50-54	50	Not performed	Satisfactory	Positive	AGC	Glandular	
P07347	2020	2020-Q4	2020-11	Pandemic	Cytology (Pap)	55-59	58	Not performed	Satisfactory	NILM	NILM	Negative (NILM)	
P07348	2020	2020-Q4	2020-11	Pandemic	Cytology (Pap)	35-39	39	Not performed	Satisfactory	NILM	NILM	Negative (NILM)	
P07349	2020	2020-Q4	2020-11	Pandemic	Cytology (Pap)	35-39	37	Not performed	Satisfactory	Positive	ASC-US	Low-grade / equivocal squamous	
P07350	2020	2020-Q4	2020-11	Pandemic	Cytology (Pap)	50-54	52	Not performed	Satisfactory	NILM	NILM	Negative (NILM)	
P07351	2020	2020-Q4	2020-11	Pandemic	Cytology (Pap)	40-44	43	Not performed	Satisfactory	NILM	NILM	Negative (NILM)	
P07352	2020	2020-Q4	2020-11	Pandemic	Cytology (Pap)	40-44	40	Not performed	Satisfactory	NILM	NILM	Negative (NILM)	
P07353	2020	2020-Q4	2020-11	Pandemic	Cytology (Pap)	55-59	59	Not performed	Satisfactory	NILM	NILM	Negative (NILM)	
P07354	2020	2020-Q4	2020-11	Pandemic	Cytology (Pap)	35-39	37	Not performed	Satisfactory	NILM	NILM	Negative (NILM)	
P07355	2020	2020-Q4	2020-11	Pandemic	Cytology (Pap)	50-54	53	Not performed	Satisfactory	NILM	NILM	Negative (NILM)	
P07356	2020	2020-Q4	2020-11	Pandemic	Cytology (Pap)	55-59	59	Not performed	Satisfactory	NILM	NILM	Negative (NILM)	
P07357	2020	2020-Q4	2020-11	Pandemic	Cytology (Pap)	25-29	29	Not performed	Satisfactory	NILM	NILM	Negative (NILM)	
P07358	2020	2020-Q4	2020-11	Pandemic	Cytology (Pap)	55-59	56	Not performed	Satisfactory	NILM	NILM	Negative (NILM)	

patient_id	year	quarter	month	period	primary_test	age_group	age_years	hpv_result	adequacy	result	bethesda	grade	infection
P07359	2020	2020-Q4	2020-11	Pandemic	Cytology (Pap)	40-44	40	Not performed	Satisfactory	NILM	NILM	Negative (NILM)	
P07360	2020	2020-Q4	2020-11	Pandemic	Cytology (Pap)	45-49	45	Not performed	Satisfactory	NILM	NILM	Negative (NILM)	
P07361	2020	2020-Q4	2020-11	Pandemic	Cytology (Pap)	55-59	59	Not performed	Satisfactory	NILM	NILM	Negative (NILM)	
P07362	2020	2020-Q4	2020-11	Pandemic	Cytology (Pap)	45-49	46	Not performed	Satisfactory	NILM	NILM	Negative (NILM)	
P07363	2020	2020-Q4	2020-11	Pandemic	Cytology (Pap)	45-49	48	Not performed	Unsatisfactory	Unsatisfactory	Unsatisfactory	Unsatisfactory	
P07364	2020	2020-Q4	2020-11	Pandemic	Cytology (Pap)	45-49	49	Not performed	Satisfactory	NILM	NILM	Negative (NILM)	
P07365	2020	2020-Q4	2020-11	Pandemic	Cytology (Pap)	55-59	59	Not performed	Satisfactory	NILM	NILM	Negative (NILM)	
P07366	2020	2020-Q4	2020-11	Pandemic	Cytology (Pap)	50-54	50	Not performed	Satisfactory	Positive	AGC	Glandular	
P07367	2020	2020-Q4	2020-11	Pandemic	Cytology (Pap)	30-34	34	Not performed	Satisfactory	Positive	LSIL	Low-grade / equivocal squamous	
P07368	2020	2020-Q4	2020-11	Pandemic	Cytology (Pap)	50-54	54	Not performed	Satisfactory	NILM	NILM	Negative (NILM)	
P07369	2020	2020-Q4	2020-11	Pandemic	Cytology (Pap)	40-44	44	Not performed	Satisfactory	NILM	NILM	Negative (NILM)	
P07370	2020	2020-Q4	2020-11	Pandemic	Cytology (Pap)	40-44	44	Not performed	Satisfactory	NILM	NILM	Negative (NILM)	
P07371	2020	2020-Q4	2020-11	Pandemic	Cytology (Pap)	40-44	42	Not performed	Satisfactory	NILM	NILM	Negative (NILM)	
P07372	2020	2020-Q4	2020-11	Pandemic	Cytology (Pap)	35-39	37	Not performed	Satisfactory	NILM	NILM	Negative (NILM)	
P07373	2020	2020-Q4	2020-11	Pandemic	Cytology (Pap)	40-44	44	Not performed	Satisfactory	Positive	ASC-US	Low-grade / equivocal squamous	
P07374	2020	2020-Q4	2020-11	Pandemic	Cytology (Pap)	35-39	37	Not performed	Satisfactory	NILM	NILM	Negative (NILM)	
P07375	2020	2020-Q4	2020-11	Pandemic	Cytology (Pap)	40-44	44	Not performed	Satisfactory	NILM	NILM	Negative (NILM)	
P07376	2020	2020-Q4	2020-11	Pandemic	Cytology (Pap)	50-54	53	Not performed	Satisfactory	NILM	NILM	Negative (NILM)	
P07377	2020	2020-Q4	2020-11	Pandemic	Cytology (Pap)	35-39	35	Not performed	Satisfactory	Positive	ASC-US	Low-grade / equivocal squamous	
P07378	2020	2020-Q4	2020-11	Pandemic	Cytology (Pap)	45-49	46	Not performed	Satisfactory	NILM	NILM	Negative (NILM)	
P07379	2020	2020-Q4	2020-11	Pandemic	Cytology (Pap)	50-54	50	Not performed	Satisfactory	NILM	NILM	Negative (NILM)	
P07380	2020	2020-Q4	2020-11	Pandemic	Cytology (Pap)	35-39	37	Not performed	Satisfactory	NILM	NILM	Negative (NILM)	
P07381	2020	2020-Q4	2020-11	Pandemic	Cytology (Pap)	40-44	44	Not performed	Satisfactory	NILM	NILM	Negative (NILM)	
P07382	2020	2020-Q4	2020-11	Pandemic	Cytology (Pap)	40-44	43	Not performed	Satisfactory	Positive	ASC-US	Low-grade / equivocal squamous	
P07383	2020	2020-Q4	2020-11	Pandemic	Cytology (Pap)	50-54	53	Not performed	Satisfactory	NILM	NILM	Negative (NILM)	
P07384	2020	2020-Q4	2020-11	Pandemic	Cytology (Pap)	50-54	51	Not performed	Satisfactory	NILM	NILM	Negative (NILM)	
P07385	2020	2020-Q4	2020-11	Pandemic	Cytology (Pap)	45-49	49	Not performed	Satisfactory	NILM	NILM	Negative (NILM)	
P07386	2020	2020-Q4	2020-11	Pandemic	Cytology (Pap)	35-39	39	Not performed	Satisfactory	NILM	NILM	Negative (NILM)	Gardnerella
P07387	2020	2020-Q4	2020-11	Pandemic	Cytology (Pap)	40-44	42	Not performed	Satisfactory	NILM	NILM	Negative (NILM)	
P07388	2020	2020-Q4	2020-11	Pandemic	Cytology (Pap)	55-59	57	Not performed	Satisfactory	Positive	ASC-US	Low-grade / equivocal squamous	
P07389	2020	2020-Q4	2020-11	Pandemic	Cytology (Pap)	30-34	30	Not performed	Satisfactory	NILM	NILM	Negative (NILM)	
P07390	2020	2020-Q4	2020-12	Pandemic	Cytology (Pap)	45-49	45	Not performed	Satisfactory	NILM	NILM	Negative (NILM)	
P07391	2020	2020-Q4	2020-12	Pandemic	Cytology (Pap)	45-49	48	Not performed	Satisfactory	NILM	NILM	Negative (NILM)	
P07392	2020	2020-Q4	2020-12	Pandemic	Cytology (Pap)	55-59	56	Not performed	Satisfactory	NILM	NILM	Negative (NILM)	
P07393	2020	2020-Q4	2020-12	Pandemic	Cytology (Pap)	25-29	27	Not performed	Satisfactory	NILM	NILM	Negative (NILM)	
P07394	2020	2020-Q4	2020-12	Pandemic	Cytology (Pap)	30-34	32	Not performed	Satisfactory	NILM	NILM	Negative (NILM)	
P07395	2020	2020-Q4	2020-12	Pandemic	Cytology (Pap)	40-44	44	Not performed	Satisfactory	NILM	NILM	Negative (NILM)	
P07396	2020	2020-Q4	2020-12	Pandemic	Cytology (Pap)	55-59	59	Not performed	Satisfactory	NILM	NILM	Negative (NILM)	
P07397	2020	2020-Q4	2020-12	Pandemic	Cytology (Pap)	55-59	55	Not performed	Satisfactory	NILM	NILM	Negative (NILM)	
P07398	2020	2020-Q4	2020-12	Pandemic	Cytology (Pap)	30-34	32	Not performed	Satisfactory	NILM	NILM	Negative (NILM)	
P07399	2020	2020-Q4	2020-12	Pandemic	Cytology (Pap)	25-29	27	Not performed	Satisfactory	Positive	HSIL	High-grade squamous	
P07400	2020	2020-Q4	2020-12	Pandemic	Cytology (Pap)	30-34	34	Not performed	Satisfactory	NILM	NILM	Negative (NILM)	
P07401	2020	2020-Q4	2020-12	Pandemic	Cytology (Pap)	55-59	58	Not performed	Satisfactory	NILM	NILM	Negative (NILM)	
P07402	2020	2020-Q4	2020-12	Pandemic	Cytology (Pap)	60-64	62	Not performed	Satisfactory	NILM	NILM	Negative (NILM)	
P07403	2020	2020-Q4	2020-12	Pandemic	Cytology (Pap)	40-44	43	Not performed	Unsatisfactory	Unsatisfactory	Unsatisfactory	Unsatisfactory	
P07404	2020	2020-Q4	2020-12	Pandemic	Cytology (Pap)	35-39	37	Not performed	Satisfactory	NILM	NILM	Negative (NILM)	Candida
P07405	2020	2020-Q4	2020-12	Pandemic	Cytology (Pap)	35-39	36	Not performed	Satisfactory	NILM	NILM	Negative (NILM)	
P07406	2020	2020-Q4	2020-12	Pandemic	Cytology (Pap)	60-64	62	Not performed	Satisfactory	NILM	NILM	Negative (NILM)	
P07407	2020	2020-Q4	2020-12	Pandemic	Cytology (Pap)	50-54	54	Not performed	Satisfactory	NILM	NILM	Negative (NILM)	
P07408	2020	2020-Q4	2020-12	Pandemic	Cytology (Pap)	45-49	46	Not performed	Satisfactory	Positive	ASC-US	Low-grade / equivocal squamous	
P07409	2020	2020-Q4	2020-12	Pandemic	Cytology (Pap)	40-44	44	Not performed	Satisfactory	NILM	NILM	Negative (NILM)	
P07410	2020	2020-Q4	2020-12	Pandemic	Cytology (Pap)	35-39	36	Not performed	Satisfactory	NILM	NILM	Negative (NILM)	
P07411	2020	2020-Q4	2020-12	Pandemic	Cytology (Pap)	30-34	30	Not performed	Satisfactory	NILM	NILM	Negative (NILM)	
P07412	2020	2020-Q4	2020-12	Pandemic	Cytology (Pap)	40-44	40	Not performed	Satisfactory	NILM	NILM	Negative (NILM)	
P07413	2020	2020-Q4	2020-12	Pandemic	Cytology (Pap)	40-44	43	Not performed	Satisfactory	NILM	NILM	Negative (NILM)	
P07414	2020	2020-Q4	2020-12	Pandemic	Cytology (Pap)	35-39	37	Not performed	Satisfactory	NILM	NILM	Negative (NILM)	
P07415	2020	2020-Q4	2020-12	Pandemic	Cytology (Pap)	45-49	47	Not performed	Satisfactory	Positive	HSIL	High-grade squamous	
P07416	2020	2020-Q4	2020-12	Pandemic	Cytology (Pap)	60-64	61	Not performed	Satisfactory	NILM	NILM	Negative (NILM)	
P07417	2020	2020-Q4	2020-12	Pandemic	Cytology (Pap)	50-54	51	Not performed	Satisfactory	Positive	AGC	Glandular	
P07418	2020	2020-Q4	2020-12	Pandemic	Cytology (Pap)	40-44	43	Not performed	Satisfactory	NILM	NILM	Negative (NILM)	
P07419	2020	2020-Q4	2020-12	Pandemic	Cytology (Pap)	40-44	40	Not performed	Satisfactory	NILM	NILM	Negative (NILM)	
P07420	2020	2020-Q4	2020-12	Pandemic	Cytology (Pap)	45-49	46	Not performed	Satisfactory	NILM	NILM	Negative (NILM)	
P07421	2020	2020-Q4	2020-12	Pandemic	Cytology (Pap)	50-54	50	Not performed	Satisfactory	NILM	NILM	Negative (NILM)	
P07422	2020	2020-Q4	2020-12	Pandemic	Cytology (Pap)	30-34	34	Not performed	Satisfactory	NILM	NILM	Negative (NILM)	
P07423	2020	2020-Q4	2020-12	Pandemic	Cytology (Pap)	40-44	42	Not performed	Satisfactory	NILM	NILM	Negative (NILM)	
P07424	2020	2020-Q4	2020-12	Pandemic	Cytology (Pap)	40-44	44	Not performed	Satisfactory	NILM	NILM	Negative (NILM)	
P07425	2020	2020-Q4	2020-12	Pandemic	Cytology (Pap)	35-39	39	Not performed	Satisfactory	NILM	NILM	Negative (NILM)	
P07426	2020	2020-Q4	2020-12	Pandemic	Cytology (Pap)	45-49	47	Not performed	Satisfactory	NILM	NILM	Negative (NILM)	
P07427	2020	2020-Q4	2020-12	Pandemic	Cytology (Pap)	50-54	54	Not performed	Satisfactory	NILM	NILM	Negative (NILM)	
P07428	2020	2020-Q4	2020-12	Pandemic	Cytology (Pap)	30-34	34	Not performed	Satisfactory	NILM	NILM	Negative (NILM)	
P07429	2020	2020-Q4	2020-12	Pandemic	Cytology (Pap)	40-44	43	Not performed	Satisfactory	NILM	NILM	Negative (NILM)	
P07430	2020	2020-Q4	2020-12	Pandemic	Cytology (Pap)	40-44	41	Not performed	Satisfactory	NILM	NILM	Negative (NILM)	
P07431	2020	2020-Q4	2020-12	Pandemic	Cytology (Pap)	40-44	41	Not performed	Satisfactory	NILM	NILM	Negative (NILM)	
P07432	2020	2020-Q4	2020-12	Pandemic	Cytology (Pap)	40-44	43	Not performed	Satisfactory	Positive	ASC-US	Low-grade / equivocal squamous	
P07433	2020	2020-Q4	2020-12	Pandemic	Cytology (Pap)	50-54	51	Not performed	Satisfactory	NILM	NILM	Negative (NILM)	
P07434	2020	2020-Q4	2020-12	Pandemic	Cytology (Pap)	30-34	34	Not performed	Satisfactory	NILM	NILM	Negative (NILM)	
P07435	2020	2020-Q4	2020-12	Pandemic	Cytology (Pap)	35-39	36	Not performed	Satisfactory	NILM	NILM	Negative (NILM)	
P07436	2020	2020-Q4	2020-12	Pandemic	Cytology (Pap)	50-54	51	Not performed	Satisfactory	NILM	NILM	Negative (NILM)	
P07437	2020	2020-Q4	2020-12	Pandemic	Cytology (Pap)	45-49	45	Not performed	Satisfactory	Positive	LSIL	Low-grade / equivocal squamous	
P07438	2020	2020-Q4	2020-12	Pandemic	Cytology (Pap)	35-39	36	Not performed	Satisfactory	NILM	NILM	Negative (NILM)	

patient_id	year	quarter	month	period	primary_test	age_group	age_years	hpv_result	adequacy	result	bethesda	grade	infection
P07439	2020	2020-Q4	2020-12	Pandemic	Cytology (Pap)	30-34	31	Not performed	Satisfactory	NILM	NILM	Negative (NILM)	
P07440	2020	2020-Q4	2020-12	Pandemic	Cytology (Pap)	45-49	49	Not performed	Satisfactory	NILM	NILM	Negative (NILM)	
P07441	2020	2020-Q4	2020-12	Pandemic	Cytology (Pap)	40-44	41	Not performed	Satisfactory	Positive	ASC-US	Low-grade / equivocal squamous	
P07442	2020	2020-Q4	2020-12	Pandemic	Cytology (Pap)	45-49	45	Not performed	Satisfactory	NILM	NILM	Negative (NILM)	
P07443	2020	2020-Q4	2020-12	Pandemic	Cytology (Pap)	40-44	44	Not performed	Satisfactory	NILM	NILM	Negative (NILM)	
P07444	2020	2020-Q4	2020-12	Pandemic	Cytology (Pap)	45-49	47	Not performed	Satisfactory	Positive	LSIL	Low-grade / equivocal squamous	
P07445	2020	2020-Q4	2020-12	Pandemic	Cytology (Pap)	30-34	33	Not performed	Satisfactory	NILM	NILM	Negative (NILM)	
P07446	2020	2020-Q4	2020-12	Pandemic	Cytology (Pap)	40-44	43	Not performed	Satisfactory	NILM	NILM	Negative (NILM)	
P07447	2020	2020-Q4	2020-12	Pandemic	Cytology (Pap)	45-49	46	Not performed	Satisfactory	NILM	NILM	Negative (NILM)	
P07448	2020	2020-Q4	2020-12	Pandemic	Cytology (Pap)	50-54	53	Not performed	Satisfactory	NILM	NILM	Negative (NILM)	
P07449	2020	2020-Q4	2020-12	Pandemic	Cytology (Pap)	55-59	56	Not performed	Satisfactory	Positive	ASC-US	Low-grade / equivocal squamous	
P07450	2020	2020-Q4	2020-12	Pandemic	Cytology (Pap)	45-49	48	Not performed	Satisfactory	NILM	NILM	Negative (NILM)	
P07451	2020	2020-Q4	2020-12	Pandemic	Cytology (Pap)	60-64	64	Not performed	Satisfactory	Positive	LSIL	Low-grade / equivocal squamous	
P07452	2020	2020-Q4	2020-12	Pandemic	Cytology (Pap)	40-44	44	Not performed	Satisfactory	Positive	ASC-US	Low-grade / equivocal squamous	
P07453	2020	2020-Q4	2020-12	Pandemic	Cytology (Pap)	45-49	47	Not performed	Satisfactory	Positive	LSIL	Low-grade / equivocal squamous	Other
P07454	2020	2020-Q4	2020-12	Pandemic	Cytology (Pap)	45-49	47	Not performed	Unsatisfactory	Unsatisfactory	Unsatisfactory	Unsatisfactory	
P07455	2020	2020-Q4	2020-12	Pandemic	Cytology (Pap)	45-49	48	Not performed	Satisfactory	NILM	NILM	Negative (NILM)	
P07456	2020	2020-Q4	2020-12	Pandemic	Cytology (Pap)	50-54	52	Not performed	Satisfactory	NILM	NILM	Negative (NILM)	
P07457	2020	2020-Q4	2020-12	Pandemic	Cytology (Pap)	40-44	44	Not performed	Satisfactory	NILM	NILM	Negative (NILM)	
P07458	2020	2020-Q4	2020-12	Pandemic	Cytology (Pap)	50-54	52	Not performed	Satisfactory	Positive	LSIL	Low-grade / equivocal squamous	
P07459	2020	2020-Q4	2020-12	Pandemic	Cytology (Pap)	55-59	59	Not performed	Satisfactory	NILM	NILM	Negative (NILM)	
P07460	2020	2020-Q4	2020-12	Pandemic	Cytology (Pap)	45-49	46	Not performed	Satisfactory	Positive	LSIL	Low-grade / equivocal squamous	
P07461	2020	2020-Q4	2020-12	Pandemic	Cytology (Pap)	40-44	43	Not performed	Satisfactory	Positive	HSIL	High-grade squamous	
P07462	2020	2020-Q4	2020-12	Pandemic	Cytology (Pap)	45-49	49	Not performed	Satisfactory	Positive	ASC-US	Low-grade / equivocal squamous	
P07463	2020	2020-Q4	2020-12	Pandemic	Cytology (Pap)	50-54	54	Not performed	Satisfactory	NILM	NILM	Negative (NILM)	
P07464	2020	2020-Q4	2020-12	Pandemic	Cytology (Pap)	50-54	54	Not performed	Satisfactory	NILM	NILM	Negative (NILM)	
P07465	2020	2020-Q4	2020-12	Pandemic	Cytology (Pap)	50-54	51	Not performed	Satisfactory	NILM	NILM	Negative (NILM)	
P07466	2020	2020-Q4	2020-12	Pandemic	Cytology (Pap)	40-44	44	Not performed	Satisfactory	NILM	NILM	Negative (NILM)	
P07467	2020	2020-Q4	2020-12	Pandemic	Cytology (Pap)	45-49	46	Not performed	Satisfactory	NILM	NILM	Negative (NILM)	
P07468	2020	2020-Q4	2020-12	Pandemic	Cytology (Pap)	40-44	43	Not performed	Satisfactory	NILM	NILM	Negative (NILM)	
P07469	2020	2020-Q4	2020-12	Pandemic	Cytology (Pap)	35-39	38	Not performed	Satisfactory	NILM	NILM	Negative (NILM)	
P07470	2020	2020-Q4	2020-12	Pandemic	Cytology (Pap)	30-34	32	Not performed	Satisfactory	NILM	NILM	Negative (NILM)	
P07471	2020	2020-Q4	2020-12	Pandemic	Cytology (Pap)	40-44	42	Not performed	Satisfactory	NILM	NILM	Negative (NILM)	
P07472	2020	2020-Q4	2020-12	Pandemic	Cytology (Pap)	55-59	58	Not performed	Satisfactory	NILM	NILM	Negative (NILM)	
P07473	2020	2020-Q4	2020-12	Pandemic	Cytology (Pap)	45-49	46	Not performed	Satisfactory	NILM	NILM	Negative (NILM)	
P07474	2020	2020-Q4	2020-12	Pandemic	Cytology (Pap)	50-54	53	Not performed	Satisfactory	NILM	NILM	Negative (NILM)	
P07475	2020	2020-Q4	2020-12	Pandemic	Cytology (Pap)	35-39	38	Not performed	Satisfactory	NILM	NILM	Negative (NILM)	
P07476	2020	2020-Q4	2020-12	Pandemic	Cytology (Pap)	50-54	53	Not performed	Satisfactory	NILM	NILM	Negative (NILM)	
P07477	2020	2020-Q4	2020-12	Pandemic	Cytology (Pap)	40-44	40	Not performed	Satisfactory	Positive	LSIL	Low-grade / equivocal squamous	
P07478	2020	2020-Q4	2020-12	Pandemic	Cytology (Pap)	25-29	27	Not performed	Satisfactory	NILM	NILM	Negative (NILM)	
P07479	2020	2020-Q4	2020-12	Pandemic	Cytology (Pap)	40-44	41	Not performed	Satisfactory	NILM	NILM	Negative (NILM)	
P07480	2021	2021-Q1	2021-01	Pandemic	Cytology (Pap)	50-54	54	Not performed	Satisfactory	NILM	NILM	Negative (NILM)	Other
P07481	2021	2021-Q1	2021-01	Pandemic	Cytology (Pap)	50-54	52	Not performed	Satisfactory	NILM	NILM	Negative (NILM)	
P07482	2021	2021-Q1	2021-01	Pandemic	Cytology (Pap)	40-44	43	Not performed	Satisfactory	NILM	NILM	Negative (NILM)	
P07483	2021	2021-Q1	2021-01	Pandemic	Cytology (Pap)	55-59	58	Not performed	Satisfactory	NILM	NILM	Negative (NILM)	
P07484	2021	2021-Q1	2021-01	Pandemic	Cytology (Pap)	40-44	42	Not performed	Satisfactory	NILM	NILM	Negative (NILM)	
P07485	2021	2021-Q1	2021-01	Pandemic	Cytology (Pap)	40-44	40	Not performed	Satisfactory	NILM	NILM	Negative (NILM)	
P07486	2021	2021-Q1	2021-01	Pandemic	Cytology (Pap)	60-64	60	Not performed	Satisfactory	NILM	NILM	Negative (NILM)	
P07487	2021	2021-Q1	2021-01	Pandemic	Cytology (Pap)	25-29	29	Not performed	Satisfactory	NILM	NILM	Negative (NILM)	
P07488	2021	2021-Q1	2021-01	Pandemic	Cytology (Pap)	40-44	40	Not performed	Satisfactory	NILM	NILM	Negative (NILM)	Other
P07489	2021	2021-Q1	2021-01	Pandemic	Cytology (Pap)	35-39	38	Not performed	Satisfactory	Positive	ASC-US	Low-grade / equivocal squamous	
P07490	2021	2021-Q1	2021-01	Pandemic	Cytology (Pap)	40-44	42	Not performed	Satisfactory	NILM	NILM	Negative (NILM)	
P07491	2021	2021-Q1	2021-01	Pandemic	Cytology (Pap)	30-34	31	Not performed	Satisfactory	NILM	NILM	Negative (NILM)	
P07492	2021	2021-Q1	2021-01	Pandemic	Cytology (Pap)	50-54	54	Not performed	Satisfactory	NILM	NILM	Negative (NILM)	
P07493	2021	2021-Q1	2021-01	Pandemic	Cytology (Pap)	50-54	52	Not performed	Satisfactory	NILM	NILM	Negative (NILM)	
P07494	2021	2021-Q1	2021-01	Pandemic	Cytology (Pap)	45-49	49	Not performed	Satisfactory	Positive	HSIL	High-grade squamous	
P07495	2021	2021-Q1	2021-01	Pandemic	Cytology (Pap)	55-59	55	Not performed	Satisfactory	Positive	AGC	Glandular	
P07496	2021	2021-Q1	2021-01	Pandemic	Cytology (Pap)	30-34	30	Not performed	Satisfactory	NILM	NILM	Negative (NILM)	
P07497	2021	2021-Q1	2021-01	Pandemic	Cytology (Pap)	40-44	43	Not performed	Satisfactory	NILM	NILM	Negative (NILM)	
P07498	2021	2021-Q1	2021-01	Pandemic	Cytology (Pap)	40-44	41	Not performed	Satisfactory	NILM	NILM	Negative (NILM)	
P07499	2021	2021-Q1	2021-01	Pandemic	Cytology (Pap)	25-29	29	Not performed	Satisfactory	NILM	NILM	Negative (NILM)	
P07500	2021	2021-Q1	2021-01	Pandemic	Cytology (Pap)	30-34	34	Not performed	Satisfactory	Positive	ASC-US	Low-grade / equivocal squamous	
P07501	2021	2021-Q1	2021-01	Pandemic	Cytology (Pap)	40-44	42	Not performed	Unsatisfactory	Unsatisfactory	Unsatisfactory	Unsatisfactory	
P07502	2021	2021-Q1	2021-01	Pandemic	Cytology (Pap)	40-44	41	Not performed	Satisfactory	NILM	NILM	Negative (NILM)	
P07503	2021	2021-Q1	2021-01	Pandemic	Cytology (Pap)	40-44	43	Not performed	Unsatisfactory	Unsatisfactory	Unsatisfactory	Unsatisfactory	
P07504	2021	2021-Q1	2021-01	Pandemic	Cytology (Pap)	35-39	37	Not performed	Satisfactory	NILM	NILM	Negative (NILM)	Trichomonas
P07505	2021	2021-Q1	2021-01	Pandemic	Cytology (Pap)	35-39	39	Not performed	Satisfactory	NILM	NILM	Negative (NILM)	
P07506	2021	2021-Q1	2021-01	Pandemic	Cytology (Pap)	50-54	50	Not performed	Satisfactory	NILM	NILM	Negative (NILM)	
P07507	2021	2021-Q1	2021-01	Pandemic	Cytology (Pap)	55-59	59	Not performed	Satisfactory	NILM	NILM	Negative (NILM)	
P07508	2021	2021-Q1	2021-01	Pandemic	Cytology (Pap)	35-39	36	Not performed	Satisfactory	NILM	NILM	Negative (NILM)	
P07509	2021	2021-Q1	2021-01	Pandemic	Cytology (Pap)	50-54	54	Not performed	Satisfactory	NILM	NILM	Negative (NILM)	
P07510	2021	2021-Q1	2021-01	Pandemic	Cytology (Pap)	55-59	58	Not performed	Satisfactory	NILM	NILM	Negative (NILM)	
P07511	2021	2021-Q1	2021-01	Pandemic	Cytology (Pap)	35-39	35	Not performed	Satisfactory	NILM	NILM	Negative (NILM)	
P07512	2021	2021-Q1	2021-01	Pandemic	Cytology (Pap)	25-29	25	Not performed	Satisfactory	NILM	NILM	Negative (NILM)	
P07513	2021	2021-Q1	2021-01	Pandemic	Cytology (Pap)	55-59	58	Not performed	Satisfactory	NILM	NILM	Negative (NILM)	
P07514	2021	2021-Q1	2021-01	Pandemic	Cytology (Pap)	40-44	44	Not performed	Satisfactory	NILM	NILM	Negative (NILM)	Trichomonas
P07515	2021	2021-Q1	2021-01	Pandemic	Cytology (Pap)	35-39	35	Not performed	Satisfactory	NILM	NILM	Negative (NILM)	
P07516	2021	2021-Q1	2021-01	Pandemic	Cytology (Pap)	40-44	40	Not performed	Satisfactory	NILM	NILM	Negative (NILM)	
P07517	2021	2021-Q1	2021-01	Pandemic	Cytology (Pap)	50-54	52	Not performed	Satisfactory	NILM	NILM	Negative (NILM)	
P07518	2021	2021-Q1	2021-01	Pandemic	Cytology (Pap)	40-44	41	Not performed	Satisfactory	NILM	NILM	Negative (NILM)	

patient_id	year	quarter	month	period	primary_test	age_group	age_years	hpv_result	adequacy	result	bethesda	grade	infection
P07519	2021	2021-Q1	2021-01	Pandemic	Cytology (Pap)	40-44	40	Not performed	Satisfactory	NILM	NILM	Negative (NILM)	Other
P07520	2021	2021-Q1	2021-01	Pandemic	Cytology (Pap)	45-49	49	Not performed	Satisfactory	Positive	ASC-US	Low-grade / equivocal squamous	
P07521	2021	2021-Q1	2021-01	Pandemic	Cytology (Pap)	40-44	42	Not performed	Satisfactory	NILM	NILM	Negative (NILM)	
P07522	2021	2021-Q1	2021-01	Pandemic	Cytology (Pap)	45-49	49	Not performed	Satisfactory	Positive	LSIL	Low-grade / equivocal squamous	
P07523	2021	2021-Q1	2021-01	Pandemic	Cytology (Pap)	45-49	48	Not performed	Satisfactory	NILM	NILM	Negative (NILM)	
P07524	2021	2021-Q1	2021-01	Pandemic	Cytology (Pap)	40-44	41	Not performed	Satisfactory	NILM	NILM	Negative (NILM)	
P07525	2021	2021-Q1	2021-01	Pandemic	Cytology (Pap)	55-59	56	Not performed	Satisfactory	NILM	NILM	Negative (NILM)	
P07526	2021	2021-Q1	2021-01	Pandemic	Cytology (Pap)	30-34	30	Not performed	Satisfactory	NILM	NILM	Negative (NILM)	
P07527	2021	2021-Q1	2021-01	Pandemic	Cytology (Pap)	30-34	34	Not performed	Satisfactory	NILM	NILM	Negative (NILM)	
P07528	2021	2021-Q1	2021-01	Pandemic	Cytology (Pap)	30-34	34	Not performed	Satisfactory	NILM	NILM	Negative (NILM)	
P07529	2021	2021-Q1	2021-01	Pandemic	Cytology (Pap)	30-34	33	Not performed	Satisfactory	NILM	NILM	Negative (NILM)	
P07530	2021	2021-Q1	2021-01	Pandemic	Cytology (Pap)	40-44	43	Not performed	Satisfactory	NILM	NILM	Negative (NILM)	
P07531	2021	2021-Q1	2021-01	Pandemic	Cytology (Pap)	25-29	26	Not performed	Satisfactory	NILM	NILM	Negative (NILM)	
P07532	2021	2021-Q1	2021-01	Pandemic	Cytology (Pap)	50-54	51	Not performed	Satisfactory	Positive	ASC-US	Low-grade / equivocal squamous	
P07533	2021	2021-Q1	2021-01	Pandemic	Cytology (Pap)	45-49	48	Not performed	Satisfactory	NILM	NILM	Negative (NILM)	
P07534	2021	2021-Q1	2021-01	Pandemic	Cytology (Pap)	35-39	36	Not performed	Satisfactory	NILM	NILM	Negative (NILM)	
P07535	2021	2021-Q1	2021-01	Pandemic	Cytology (Pap)	30-34	34	Not performed	Satisfactory	NILM	NILM	Negative (NILM)	
P07536	2021	2021-Q1	2021-01	Pandemic	Cytology (Pap)	25-29	25	Not performed	Unsatisfactory	Unsatisfactory	Unsatisfactory	Unsatisfactory	
P07537	2021	2021-Q1	2021-01	Pandemic	Cytology (Pap)	50-54	50	Not performed	Satisfactory	NILM	NILM	Negative (NILM)	
P07538	2021	2021-Q1	2021-01	Pandemic	Cytology (Pap)	50-54	53	Not performed	Satisfactory	NILM	NILM	Negative (NILM)	
P07539	2021	2021-Q1	2021-01	Pandemic	Cytology (Pap)	45-49	48	Not performed	Satisfactory	Positive	HSIL	High-grade squamous	
P07540	2021	2021-Q1	2021-01	Pandemic	Cytology (Pap)	50-54	54	Not performed	Satisfactory	NILM	NILM	Negative (NILM)	
P07541	2021	2021-Q1	2021-01	Pandemic	Cytology (Pap)	50-54	54	Not performed	Satisfactory	NILM	NILM	Negative (NILM)	
P07542	2021	2021-Q1	2021-01	Pandemic	Cytology (Pap)	30-34	31	Not performed	Satisfactory	NILM	NILM	Negative (NILM)	
P07543	2021	2021-Q1	2021-01	Pandemic	Cytology (Pap)	55-59	58	Not performed	Satisfactory	NILM	NILM	Negative (NILM)	
P07544	2021	2021-Q1	2021-01	Pandemic	Cytology (Pap)	45-49	46	Not performed	Satisfactory	NILM	NILM	Negative (NILM)	
P07545	2021	2021-Q1	2021-01	Pandemic	Cytology (Pap)	45-49	47	Not performed	Satisfactory	Positive	ASC-US	Low-grade / equivocal squamous	
P07546	2021	2021-Q1	2021-01	Pandemic	Cytology (Pap)	55-59	57	Not performed	Satisfactory	NILM	NILM	Negative (NILM)	
P07547	2021	2021-Q1	2021-01	Pandemic	Cytology (Pap)	45-49	49	Not performed	Satisfactory	NILM	NILM	Negative (NILM)	Gardnerella
P07548	2021	2021-Q1	2021-01	Pandemic	Cytology (Pap)	35-39	39	Not performed	Satisfactory	NILM	NILM	Negative (NILM)	
P07549	2021	2021-Q1	2021-01	Pandemic	Cytology (Pap)	55-59	56	Not performed	Satisfactory	NILM	NILM	Negative (NILM)	
P07550	2021	2021-Q1	2021-01	Pandemic	Cytology (Pap)	55-59	56	Not performed	Satisfactory	NILM	NILM	Negative (NILM)	
P07551	2021	2021-Q1	2021-01	Pandemic	Cytology (Pap)	35-39	35	Not performed	Satisfactory	NILM	NILM	Negative (NILM)	
P07552	2021	2021-Q1	2021-01	Pandemic	Cytology (Pap)	45-49	46	Not performed	Satisfactory	Positive	LSIL	Low-grade / equivocal squamous	
P07553	2021	2021-Q1	2021-01	Pandemic	Cytology (Pap)	30-34	34	Not performed	Satisfactory	NILM	NILM	Negative (NILM)	
P07554	2021	2021-Q1	2021-01	Pandemic	Cytology (Pap)	35-39	38	Not performed	Satisfactory	NILM	NILM	Negative (NILM)	
P07555	2021	2021-Q1	2021-01	Pandemic	Cytology (Pap)	50-54	50	Not performed	Satisfactory	NILM	NILM	Negative (NILM)	
P07556	2021	2021-Q1	2021-01	Pandemic	Cytology (Pap)	40-44	41	Not performed	Satisfactory	NILM	NILM	Negative (NILM)	
P07557	2021	2021-Q1	2021-01	Pandemic	Cytology (Pap)	55-59	56	Not performed	Satisfactory	NILM	NILM	Negative (NILM)	
P07558	2021	2021-Q1	2021-01	Pandemic	Cytology (Pap)	35-39	35	Not performed	Satisfactory	NILM	NILM	Negative (NILM)	
P07559	2021	2021-Q1	2021-01	Pandemic	Cytology (Pap)	30-34	34	Not performed	Satisfactory	NILM	NILM	Negative (NILM)	
P07560	2021	2021-Q1	2021-01	Pandemic	Cytology (Pap)	45-49	49	Not performed	Satisfactory	NILM	NILM	Negative (NILM)	
P07561	2021	2021-Q1	2021-01	Pandemic	Cytology (Pap)	35-39	35	Not performed	Satisfactory	NILM	NILM	Negative (NILM)	
P07562	2021	2021-Q1	2021-01	Pandemic	Cytology (Pap)	50-54	53	Not performed	Satisfactory	NILM	NILM	Negative (NILM)	
P07563	2021	2021-Q1	2021-01	Pandemic	Cytology (Pap)	40-44	43	Not performed	Satisfactory	NILM	NILM	Negative (NILM)	
P07564	2021	2021-Q1	2021-01	Pandemic	Cytology (Pap)	50-54	54	Not performed	Satisfactory	NILM	NILM	Negative (NILM)	
P07565	2021	2021-Q1	2021-01	Pandemic	Cytology (Pap)	55-59	59	Not performed	Satisfactory	NILM	NILM	Negative (NILM)	Trichomonas
P07566	2021	2021-Q1	2021-01	Pandemic	Cytology (Pap)	40-44	42	Not performed	Satisfactory	NILM	NILM	Negative (NILM)	
P07567	2021	2021-Q1	2021-01	Pandemic	Cytology (Pap)	55-59	55	Not performed	Unsatisfactory	Unsatisfactory	Unsatisfactory	Unsatisfactory	
P07568	2021	2021-Q1	2021-02	Pandemic	Cytology (Pap)	45-49	48	Not performed	Satisfactory	NILM	NILM	Negative (NILM)	
P07569	2021	2021-Q1	2021-02	Pandemic	Cytology (Pap)	45-49	49	Not performed	Satisfactory	NILM	NILM	Negative (NILM)	
P07570	2021	2021-Q1	2021-02	Pandemic	Cytology (Pap)	55-59	59	Not performed	Satisfactory	NILM	NILM	Negative (NILM)	
P07571	2021	2021-Q1	2021-02	Pandemic	Cytology (Pap)	35-39	36	Not performed	Satisfactory	NILM	NILM	Negative (NILM)	
P07572	2021	2021-Q1	2021-02	Pandemic	Cytology (Pap)	55-59	56	Not performed	Satisfactory	NILM	NILM	Negative (NILM)	
P07573	2021	2021-Q1	2021-02	Pandemic	Cytology (Pap)	40-44	42	Not performed	Satisfactory	Positive	ASC-US	Low-grade / equivocal squamous	
P07574	2021	2021-Q1	2021-02	Pandemic	Cytology (Pap)	45-49	48	Not performed	Satisfactory	NILM	NILM	Negative (NILM)	
P07575	2021	2021-Q1	2021-02	Pandemic	Cytology (Pap)	45-49	47	Not performed	Satisfactory	NILM	NILM	Negative (NILM)	Candida
P07576	2021	2021-Q1	2021-02	Pandemic	Cytology (Pap)	55-59	57	Not performed	Satisfactory	Positive	ASC-US	Low-grade / equivocal squamous	
P07577	2021	2021-Q1	2021-02	Pandemic	Cytology (Pap)	40-44	44	Not performed	Satisfactory	NILM	NILM	Negative (NILM)	
P07578	2021	2021-Q1	2021-02	Pandemic	Cytology (Pap)	35-39	39	Not performed	Satisfactory	NILM	NILM	Negative (NILM)	
P07579	2021	2021-Q1	2021-02	Pandemic	Cytology (Pap)	50-54	53	Not performed	Satisfactory	NILM	NILM	Negative (NILM)	
P07580	2021	2021-Q1	2021-02	Pandemic	Cytology (Pap)	45-49	46	Not performed	Satisfactory	NILM	NILM	Negative (NILM)	
P07581	2021	2021-Q1	2021-02	Pandemic	Cytology (Pap)	45-49	49	Not performed	Satisfactory	Positive	LSIL	Low-grade / equivocal squamous	
P07582	2021	2021-Q1	2021-02	Pandemic	Cytology (Pap)	50-54	52	Not performed	Satisfactory	Positive	ASC-US	Low-grade / equivocal squamous	
P07583	2021	2021-Q1	2021-02	Pandemic	Cytology (Pap)	35-39	37	Not performed	Satisfactory	NILM	NILM	Negative (NILM)	
P07584	2021	2021-Q1	2021-02	Pandemic	Cytology (Pap)	55-59	56	Not performed	Satisfactory	Positive	HSIL	High-grade squamous	
P07585	2021	2021-Q1	2021-02	Pandemic	Cytology (Pap)	30-34	30	Not performed	Satisfactory	NILM	NILM	Negative (NILM)	
P07586	2021	2021-Q1	2021-02	Pandemic	Cytology (Pap)	50-54	54	Not performed	Satisfactory	NILM	NILM	Negative (NILM)	Trichomonas
P07587	2021	2021-Q1	2021-02	Pandemic	Cytology (Pap)	40-44	41	Not performed	Satisfactory	Positive	ASC-US	Low-grade / equivocal squamous	
P07588	2021	2021-Q1	2021-02	Pandemic	Cytology (Pap)	45-49	49	Not performed	Satisfactory	NILM	NILM	Negative (NILM)	
P07589	2021	2021-Q1	2021-02	Pandemic	Cytology (Pap)	50-54	50	Not performed	Satisfactory	NILM	NILM	Negative (NILM)	
P07590	2021	2021-Q1	2021-02	Pandemic	Cytology (Pap)	35-39	36	Not performed	Satisfactory	Positive	LSIL	Low-grade / equivocal squamous	
P07591	2021	2021-Q1	2021-02	Pandemic	Cytology (Pap)	60-64	61	Not performed	Satisfactory	NILM	NILM	Negative (NILM)	
P07592	2021	2021-Q1	2021-02	Pandemic	Cytology (Pap)	50-54	54	Not performed	Satisfactory	NILM	NILM	Negative (NILM)	
P07593	2021	2021-Q1	2021-02	Pandemic	Cytology (Pap)	35-39	37	Not performed	Satisfactory	NILM	NILM	Negative (NILM)	
P07594	2021	2021-Q1	2021-02	Pandemic	Cytology (Pap)	40-44	44	Not performed	Satisfactory	NILM	NILM	Negative (NILM)	
P07595	2021	2021-Q1	2021-02	Pandemic	Cytology (Pap)	35-39	39	Not performed	Satisfactory	NILM	NILM	Negative (NILM)	
P07596	2021	2021-Q1	2021-02	Pandemic	Cytology (Pap)	35-39	39	Not performed	Satisfactory	NILM	NILM	Negative (NILM)	
P07597	2021	2021-Q1	2021-02	Pandemic	Cytology (Pap)	40-44	43	Not performed	Satisfactory	NILM	NILM	Negative (NILM)	Trichomonas
P07598	2021	2021-Q1	2021-02	Pandemic	Cytology (Pap)	45-49	49	Not performed	Satisfactory	NILM	NILM	Negative (NILM)	

patient_id	year	quarter	month	period	primary_test	age_group	age_years	hpv_result	adequacy	result	bethesda	grade	infection
P07599	2021	2021-Q1	2021-02	Pandemic	Cytology (Pap)	35-39	39	Not performed	Satisfactory	NILM	NILM	Negative (NILM)	
P07600	2021	2021-Q1	2021-02	Pandemic	Cytology (Pap)	60-64	61	Not performed	Satisfactory	NILM	NILM	Negative (NILM)	
P07601	2021	2021-Q1	2021-02	Pandemic	Cytology (Pap)	45-49	45	Not performed	Satisfactory	NILM	NILM	Negative (NILM)	
P07602	2021	2021-Q1	2021-02	Pandemic	Cytology (Pap)	35-39	36	Not performed	Satisfactory	NILM	NILM	Negative (NILM)	
P07603	2021	2021-Q1	2021-02	Pandemic	Cytology (Pap)	45-49	48	Not performed	Satisfactory	NILM	NILM	Negative (NILM)	
P07604	2021	2021-Q1	2021-02	Pandemic	Cytology (Pap)	45-49	48	Not performed	Satisfactory	NILM	NILM	Negative (NILM)	
P07605	2021	2021-Q1	2021-02	Pandemic	Cytology (Pap)	45-49	46	Not performed	Satisfactory	NILM	NILM	Negative (NILM)	
P07606	2021	2021-Q1	2021-02	Pandemic	Cytology (Pap)	35-39	38	Not performed	Satisfactory	NILM	NILM	Negative (NILM)	
P07607	2021	2021-Q1	2021-02	Pandemic	Cytology (Pap)	45-49	47	Not performed	Satisfactory	NILM	NILM	Negative (NILM)	
P07608	2021	2021-Q1	2021-02	Pandemic	Cytology (Pap)	60-64	64	Not performed	Satisfactory	NILM	NILM	Negative (NILM)	
P07609	2021	2021-Q1	2021-02	Pandemic	Cytology (Pap)	55-59	55	Not performed	Satisfactory	NILM	NILM	Negative (NILM)	
P07610	2021	2021-Q1	2021-02	Pandemic	Cytology (Pap)	45-49	49	Not performed	Satisfactory	NILM	NILM	Negative (NILM)	
P07611	2021	2021-Q1	2021-02	Pandemic	Cytology (Pap)	40-44	41	Not performed	Satisfactory	NILM	NILM	Negative (NILM)	
P07612	2021	2021-Q1	2021-02	Pandemic	Cytology (Pap)	35-39	36	Not performed	Satisfactory	NILM	NILM	Negative (NILM)	
P07613	2021	2021-Q1	2021-02	Pandemic	Cytology (Pap)	40-44	44	Not performed	Satisfactory	NILM	NILM	Negative (NILM)	
P07614	2021	2021-Q1	2021-02	Pandemic	Cytology (Pap)	40-44	43	Not performed	Satisfactory	NILM	NILM	Negative (NILM)	
P07615	2021	2021-Q1	2021-02	Pandemic	Cytology (Pap)	50-54	53	Not performed	Satisfactory	NILM	NILM	Negative (NILM)	
P07616	2021	2021-Q1	2021-02	Pandemic	Cytology (Pap)	45-49	47	Not performed	Satisfactory	NILM	NILM	Negative (NILM)	
P07617	2021	2021-Q1	2021-02	Pandemic	Cytology (Pap)	35-39	36	Not performed	Satisfactory	NILM	NILM	Negative (NILM)	
P07618	2021	2021-Q1	2021-02	Pandemic	Cytology (Pap)	45-49	46	Not performed	Satisfactory	NILM	NILM	Negative (NILM)	
P07619	2021	2021-Q1	2021-02	Pandemic	Cytology (Pap)	40-44	41	Not performed	Satisfactory	Positive	LSIL	Low-grade / equivocal squamous	Gardnerella
P07620	2021	2021-Q1	2021-02	Pandemic	Cytology (Pap)	35-39	38	Not performed	Satisfactory	NILM	NILM	Negative (NILM)	
P07621	2021	2021-Q1	2021-02	Pandemic	Cytology (Pap)	35-39	39	Not performed	Satisfactory	NILM	NILM	Negative (NILM)	
P07622	2021	2021-Q1	2021-02	Pandemic	Cytology (Pap)	25-29	27	Not performed	Satisfactory	NILM	NILM	Negative (NILM)	
P07623	2021	2021-Q1	2021-02	Pandemic	Cytology (Pap)	35-39	36	Not performed	Satisfactory	Positive	AGC	Glandular	
P07624	2021	2021-Q1	2021-02	Pandemic	Cytology (Pap)	60-64	61	Not performed	Satisfactory	NILM	NILM	Negative (NILM)	
P07625	2021	2021-Q1	2021-02	Pandemic	Cytology (Pap)	30-34	31	Not performed	Satisfactory	NILM	NILM	Negative (NILM)	
P07626	2021	2021-Q1	2021-02	Pandemic	Cytology (Pap)	45-49	45	Not performed	Satisfactory	Positive	AGC	Glandular	
P07627	2021	2021-Q1	2021-02	Pandemic	Cytology (Pap)	45-49	47	Not performed	Satisfactory	NILM	NILM	Negative (NILM)	
P07628	2021	2021-Q1	2021-02	Pandemic	Cytology (Pap)	25-29	27	Not performed	Satisfactory	NILM	NILM	Negative (NILM)	
P07629	2021	2021-Q1	2021-02	Pandemic	Cytology (Pap)	35-39	36	Not performed	Satisfactory	NILM	NILM	Negative (NILM)	
P07630	2021	2021-Q1	2021-02	Pandemic	Cytology (Pap)	35-39	35	Not performed	Satisfactory	NILM	NILM	Negative (NILM)	
P07631	2021	2021-Q1	2021-02	Pandemic	Cytology (Pap)	45-49	49	Not performed	Satisfactory	NILM	NILM	Negative (NILM)	
P07632	2021	2021-Q1	2021-02	Pandemic	Cytology (Pap)	45-49	46	Not performed	Satisfactory	NILM	NILM	Negative (NILM)	
P07633	2021	2021-Q1	2021-02	Pandemic	Cytology (Pap)	25-29	28	Not performed	Satisfactory	NILM	NILM	Negative (NILM)	
P07634	2021	2021-Q1	2021-02	Pandemic	Cytology (Pap)	30-34	32	Not performed	Satisfactory	NILM	NILM	Negative (NILM)	
P07635	2021	2021-Q1	2021-02	Pandemic	Cytology (Pap)	40-44	42	Not performed	Satisfactory	NILM	NILM	Negative (NILM)	
P07636	2021	2021-Q1	2021-02	Pandemic	Cytology (Pap)	50-54	50	Not performed	Satisfactory	NILM	NILM	Negative (NILM)	
P07637	2021	2021-Q1	2021-02	Pandemic	Cytology (Pap)	55-59	59	Not performed	Satisfactory	Positive	HSIL	High-grade squamous	
P07638	2021	2021-Q1	2021-02	Pandemic	Cytology (Pap)	45-49	48	Not performed	Satisfactory	NILM	NILM	Negative (NILM)	
P07639	2021	2021-Q1	2021-02	Pandemic	Cytology (Pap)	45-49	49	Not performed	Satisfactory	NILM	NILM	Negative (NILM)	
P07640	2021	2021-Q1	2021-02	Pandemic	Cytology (Pap)	40-44	44	Not performed	Satisfactory	Positive	ASC-US	Low-grade / equivocal squamous	
P07641	2021	2021-Q1	2021-02	Pandemic	Cytology (Pap)	50-54	52	Not performed	Satisfactory	NILM	NILM	Negative (NILM)	
P07642	2021	2021-Q1	2021-02	Pandemic	Cytology (Pap)	45-49	47	Not performed	Satisfactory	NILM	NILM	Negative (NILM)	
P07643	2021	2021-Q1	2021-02	Pandemic	Cytology (Pap)	50-54	51	Not performed	Unsatisfactory	Unsatisfactory	Unsatisfactory	Unsatisfactory	
P07644	2021	2021-Q1	2021-02	Pandemic	Cytology (Pap)	45-49	49	Not performed	Satisfactory	NILM	NILM	Negative (NILM)	
P07645	2021	2021-Q1	2021-02	Pandemic	Cytology (Pap)	40-44	42	Not performed	Satisfactory	NILM	NILM	Negative (NILM)	
P07646	2021	2021-Q1	2021-02	Pandemic	Cytology (Pap)	35-39	36	Not performed	Satisfactory	NILM	NILM	Negative (NILM)	
P07647	2021	2021-Q1	2021-02	Pandemic	Cytology (Pap)	45-49	46	Not performed	Satisfactory	NILM	NILM	Negative (NILM)	
P07648	2021	2021-Q1	2021-02	Pandemic	Cytology (Pap)	45-49	49	Not performed	Satisfactory	NILM	NILM	Negative (NILM)	
P07649	2021	2021-Q1	2021-02	Pandemic	Cytology (Pap)	60-64	63	Not performed	Satisfactory	NILM	NILM	Negative (NILM)	
P07650	2021	2021-Q1	2021-02	Pandemic	Cytology (Pap)	35-39	38	Not performed	Satisfactory	NILM	NILM	Negative (NILM)	
P07651	2021	2021-Q1	2021-02	Pandemic	Cytology (Pap)	40-44	44	Not performed	Satisfactory	NILM	NILM	Negative (NILM)	
P07652	2021	2021-Q1	2021-02	Pandemic	Cytology (Pap)	35-39	36	Not performed	Satisfactory	NILM	NILM	Negative (NILM)	
P07653	2021	2021-Q1	2021-02	Pandemic	Cytology (Pap)	40-44	41	Not performed	Satisfactory	Positive	ASC-US	Low-grade / equivocal squamous	
P07654	2021	2021-Q1	2021-02	Pandemic	Cytology (Pap)	35-39	35	Not performed	Satisfactory	NILM	NILM	Negative (NILM)	
P07655	2021	2021-Q1	2021-02	Pandemic	Cytology (Pap)	35-39	36	Not performed	Satisfactory	NILM	NILM	Negative (NILM)	
P07656	2021	2021-Q1	2021-03	Pandemic	Cytology (Pap)	55-59	56	Not performed	Satisfactory	NILM	NILM	Negative (NILM)	
P07657	2021	2021-Q1	2021-03	Pandemic	Cytology (Pap)	55-59	59	Not performed	Satisfactory	NILM	NILM	Negative (NILM)	
P07658	2021	2021-Q1	2021-03	Pandemic	Cytology (Pap)	25-29	27	Not performed	Satisfactory	NILM	NILM	Negative (NILM)	
P07659	2021	2021-Q1	2021-03	Pandemic	Cytology (Pap)	55-59	57	Not performed	Satisfactory	NILM	NILM	Negative (NILM)	
P07660	2021	2021-Q1	2021-03	Pandemic	Cytology (Pap)	25-29	28	Not performed	Satisfactory	NILM	NILM	Negative (NILM)	
P07661	2021	2021-Q1	2021-03	Pandemic	Cytology (Pap)	45-49	49	Not performed	Satisfactory	NILM	NILM	Negative (NILM)	
P07662	2021	2021-Q1	2021-03	Pandemic	Cytology (Pap)	40-44	40	Not performed	Satisfactory	Positive	HSIL	High-grade squamous	
P07663	2021	2021-Q1	2021-03	Pandemic	Cytology (Pap)	40-44	44	Not performed	Satisfactory	NILM	NILM	Negative (NILM)	
P07664	2021	2021-Q1	2021-03	Pandemic	Cytology (Pap)	45-49	45	Not performed	Satisfactory	NILM	NILM	Negative (NILM)	
P07665	2021	2021-Q1	2021-03	Pandemic	Cytology (Pap)	35-39	39	Not performed	Satisfactory	NILM	NILM	Negative (NILM)	
P07666	2021	2021-Q1	2021-03	Pandemic	Cytology (Pap)	50-54	54	Not performed	Satisfactory	NILM	NILM	Negative (NILM)	
P07667	2021	2021-Q1	2021-03	Pandemic	Cytology (Pap)	50-54	50	Not performed	Satisfactory	NILM	NILM	Negative (NILM)	
P07668	2021	2021-Q1	2021-03	Pandemic	Cytology (Pap)	40-44	41	Not performed	Satisfactory	NILM	NILM	Negative (NILM)	
P07669	2021	2021-Q1	2021-03	Pandemic	Cytology (Pap)	40-44	40	Not performed	Satisfactory	NILM	NILM	Negative (NILM)	
P07670	2021	2021-Q1	2021-03	Pandemic	Cytology (Pap)	45-49	46	Not performed	Satisfactory	NILM	NILM	Negative (NILM)	
P07671	2021	2021-Q1	2021-03	Pandemic	Cytology (Pap)	25-29	28	Not performed	Satisfactory	NILM	NILM	Negative (NILM)	
P07672	2021	2021-Q1	2021-03	Pandemic	Cytology (Pap)	35-39	38	Not performed	Satisfactory	NILM	NILM	Negative (NILM)	
P07673	2021	2021-Q1	2021-03	Pandemic	Cytology (Pap)	40-44	43	Not performed	Satisfactory	NILM	NILM	Negative (NILM)	Candida
P07674	2021	2021-Q1	2021-03	Pandemic	Cytology (Pap)	60-64	60	Not performed	Satisfactory	Positive	ASC-US	Low-grade / equivocal squamous	
P07675	2021	2021-Q1	2021-03	Pandemic	Cytology (Pap)	35-39	35	Not performed	Satisfactory	NILM	NILM	Negative (NILM)	
P07676	2021	2021-Q1	2021-03	Pandemic	Cytology (Pap)	60-64	62	Not performed	Satisfactory	NILM	NILM	Negative (NILM)	
P07677	2021	2021-Q1	2021-03	Pandemic	Cytology (Pap)	45-49	47	Not performed	Satisfactory	Positive	ASC-US	Low-grade / equivocal squamous	
P07678	2021	2021-Q1	2021-03	Pandemic	Cytology (Pap)	35-39	39	Not performed	Satisfactory	Positive	HSIL	High-grade squamous	

patient_id	year	quarter	month	period	primary_test	age_group	age_years	hpv_result	adequacy	result	bethesda	grade	infection
P07679	2021	2021-Q1	2021-03	Pandemic	Cytology (Pap)	40-44	40	Not performed	Satisfactory	NILM	NILM	Negative (NILM)	
P07680	2021	2021-Q1	2021-03	Pandemic	Cytology (Pap)	40-44	41	Not performed	Satisfactory	Positive	ASC-US	Low-grade / equivocal squamous	
P07681	2021	2021-Q1	2021-03	Pandemic	Cytology (Pap)	50-54	51	Not performed	Satisfactory	NILM	NILM	Negative (NILM)	
P07682	2021	2021-Q1	2021-03	Pandemic	Cytology (Pap)	40-44	40	Not performed	Satisfactory	NILM	NILM	Negative (NILM)	
P07683	2021	2021-Q1	2021-03	Pandemic	Cytology (Pap)	30-34	32	Not performed	Satisfactory	Positive	ASC-US	Low-grade / equivocal squamous	
P07684	2021	2021-Q1	2021-03	Pandemic	Cytology (Pap)	25-29	28	Not performed	Satisfactory	Positive	LSIL	Low-grade / equivocal squamous	
P07685	2021	2021-Q1	2021-03	Pandemic	Cytology (Pap)	30-34	31	Not performed	Satisfactory	NILM	NILM	Negative (NILM)	
P07686	2021	2021-Q1	2021-03	Pandemic	Cytology (Pap)	25-29	29	Not performed	Satisfactory	Positive	AGC	Glandular	
P07687	2021	2021-Q1	2021-03	Pandemic	Cytology (Pap)	35-39	39	Not performed	Satisfactory	NILM	NILM	Negative (NILM)	
P07688	2021	2021-Q1	2021-03	Pandemic	Cytology (Pap)	60-64	63	Not performed	Satisfactory	NILM	NILM	Negative (NILM)	
P07689	2021	2021-Q1	2021-03	Pandemic	Cytology (Pap)	55-59	55	Not performed	Satisfactory	NILM	NILM	Negative (NILM)	
P07690	2021	2021-Q1	2021-03	Pandemic	Cytology (Pap)	50-54	52	Not performed	Satisfactory	NILM	NILM	Negative (NILM)	
P07691	2021	2021-Q1	2021-03	Pandemic	Cytology (Pap)	50-54	52	Not performed	Satisfactory	NILM	NILM	Negative (NILM)	
P07692	2021	2021-Q1	2021-03	Pandemic	Cytology (Pap)	40-44	42	Not performed	Satisfactory	NILM	NILM	Negative (NILM)	
P07693	2021	2021-Q1	2021-03	Pandemic	Cytology (Pap)	50-54	50	Not performed	Satisfactory	Positive	HSIL	High-grade squamous	
P07694	2021	2021-Q1	2021-03	Pandemic	Cytology (Pap)	55-59	56	Not performed	Satisfactory	NILM	NILM	Negative (NILM)	
P07695	2021	2021-Q1	2021-03	Pandemic	Cytology (Pap)	35-39	37	Not performed	Satisfactory	NILM	NILM	Negative (NILM)	
P07696	2021	2021-Q1	2021-03	Pandemic	Cytology (Pap)	55-59	58	Not performed	Satisfactory	NILM	NILM	Negative (NILM)	
P07697	2021	2021-Q1	2021-03	Pandemic	Cytology (Pap)	30-34	30	Not performed	Satisfactory	NILM	NILM	Negative (NILM)	
P07698	2021	2021-Q1	2021-03	Pandemic	Cytology (Pap)	40-44	42	Not performed	Satisfactory	NILM	NILM	Negative (NILM)	
P07699	2021	2021-Q1	2021-03	Pandemic	Cytology (Pap)	40-44	40	Not performed	Satisfactory	NILM	NILM	Negative (NILM)	
P07700	2021	2021-Q1	2021-03	Pandemic	Cytology (Pap)	30-34	32	Not performed	Satisfactory	NILM	NILM	Negative (NILM)	
P07701	2021	2021-Q1	2021-03	Pandemic	Cytology (Pap)	50-54	51	Not performed	Satisfactory	NILM	NILM	Negative (NILM)	
P07702	2021	2021-Q1	2021-03	Pandemic	Cytology (Pap)	25-29	26	Not performed	Satisfactory	NILM	NILM	Negative (NILM)	
P07703	2021	2021-Q1	2021-03	Pandemic	Cytology (Pap)	55-59	59	Not performed	Satisfactory	NILM	NILM	Negative (NILM)	
P07704	2021	2021-Q1	2021-03	Pandemic	Cytology (Pap)	50-54	51	Not performed	Satisfactory	Positive	ASC-US	Low-grade / equivocal squamous	
P07705	2021	2021-Q1	2021-03	Pandemic	Cytology (Pap)	40-44	40	Not performed	Satisfactory	NILM	NILM	Negative (NILM)	
P07706	2021	2021-Q1	2021-03	Pandemic	Cytology (Pap)	45-49	49	Not performed	Satisfactory	NILM	NILM	Negative (NILM)	
P07707	2021	2021-Q1	2021-03	Pandemic	Cytology (Pap)	30-34	31	Not performed	Satisfactory	NILM	NILM	Negative (NILM)	
P07708	2021	2021-Q1	2021-03	Pandemic	Cytology (Pap)	45-49	49	Not performed	Satisfactory	NILM	NILM	Negative (NILM)	
P07709	2021	2021-Q1	2021-03	Pandemic	Cytology (Pap)	30-34	34	Not performed	Satisfactory	NILM	NILM	Negative (NILM)	
P07710	2021	2021-Q1	2021-03	Pandemic	Cytology (Pap)	60-64	60	Not performed	Satisfactory	NILM	NILM	Negative (NILM)	
P07711	2021	2021-Q1	2021-03	Pandemic	Cytology (Pap)	35-39	37	Not performed	Satisfactory	NILM	NILM	Negative (NILM)	
P07712	2021	2021-Q1	2021-03	Pandemic	Cytology (Pap)	45-49	46	Not performed	Satisfactory	NILM	NILM	Negative (NILM)	
P07713	2021	2021-Q1	2021-03	Pandemic	Cytology (Pap)	25-29	25	Not performed	Satisfactory	NILM	NILM	Negative (NILM)	
P07714	2021	2021-Q1	2021-03	Pandemic	Cytology (Pap)	40-44	44	Not performed	Satisfactory	NILM	NILM	Negative (NILM)	Other
P07715	2021	2021-Q1	2021-03	Pandemic	Cytology (Pap)	40-44	41	Not performed	Unsatisfactory	Unsatisfactory	Unsatisfactory	Unsatisfactory	
P07716	2021	2021-Q1	2021-03	Pandemic	Cytology (Pap)	40-44	42	Not performed	Satisfactory	Positive	ASC-US	Low-grade / equivocal squamous	
P07717	2021	2021-Q1	2021-03	Pandemic	Cytology (Pap)	55-59	58	Not performed	Satisfactory	NILM	NILM	Negative (NILM)	
P07718	2021	2021-Q1	2021-03	Pandemic	Cytology (Pap)	50-54	51	Not performed	Satisfactory	NILM	NILM	Negative (NILM)	
P07719	2021	2021-Q1	2021-03	Pandemic	Cytology (Pap)	50-54	51	Not performed	Satisfactory	NILM	NILM	Negative (NILM)	
P07720	2021	2021-Q1	2021-03	Pandemic	Cytology (Pap)	50-54	51	Not performed	Satisfactory	Positive	AGC	Glandular	
P07721	2021	2021-Q1	2021-03	Pandemic	Cytology (Pap)	40-44	42	Not performed	Satisfactory	NILM	NILM	Negative (NILM)	
P07722	2021	2021-Q1	2021-03	Pandemic	Cytology (Pap)	50-54	53	Not performed	Satisfactory	NILM	NILM	Negative (NILM)	
P07723	2021	2021-Q1	2021-03	Pandemic	Cytology (Pap)	30-34	32	Not performed	Satisfactory	NILM	NILM	Negative (NILM)	Candida
P07724	2021	2021-Q1	2021-03	Pandemic	Cytology (Pap)	35-39	37	Not performed	Satisfactory	NILM	NILM	Negative (NILM)	
P07725	2021	2021-Q1	2021-03	Pandemic	Cytology (Pap)	25-29	27	Not performed	Satisfactory	NILM	NILM	Negative (NILM)	
P07726	2021	2021-Q1	2021-03	Pandemic	Cytology (Pap)	55-59	59	Not performed	Satisfactory	NILM	NILM	Negative (NILM)	
P07727	2021	2021-Q1	2021-03	Pandemic	Cytology (Pap)	30-34	33	Not performed	Satisfactory	NILM	NILM	Negative (NILM)	
P07728	2021	2021-Q1	2021-03	Pandemic	Cytology (Pap)	35-39	37	Not performed	Satisfactory	NILM	NILM	Negative (NILM)	
P07729	2021	2021-Q1	2021-03	Pandemic	Cytology (Pap)	40-44	43	Not performed	Satisfactory	NILM	NILM	Negative (NILM)	
P07730	2021	2021-Q1	2021-03	Pandemic	Cytology (Pap)	30-34	34	Not performed	Satisfactory	NILM	NILM	Negative (NILM)	
P07731	2021	2021-Q1	2021-03	Pandemic	Cytology (Pap)	45-49	48	Not performed	Satisfactory	NILM	NILM	Negative (NILM)	
P07732	2021	2021-Q1	2021-03	Pandemic	Cytology (Pap)	55-59	55	Not performed	Unsatisfactory	Unsatisfactory	Unsatisfactory	Unsatisfactory	
P07733	2021	2021-Q1	2021-03	Pandemic	Cytology (Pap)	45-49	48	Not performed	Satisfactory	NILM	NILM	Negative (NILM)	
P07734	2021	2021-Q1	2021-03	Pandemic	Cytology (Pap)	30-34	33	Not performed	Satisfactory	NILM	NILM	Negative (NILM)	
P07735	2021	2021-Q1	2021-03	Pandemic	Cytology (Pap)	60-64	62	Not performed	Satisfactory	Positive	HSIL	High-grade squamous	
P07736	2021	2021-Q1	2021-03	Pandemic	Cytology (Pap)	45-49	49	Not performed	Satisfactory	NILM	NILM	Negative (NILM)	
P07737	2021	2021-Q1	2021-03	Pandemic	Cytology (Pap)	60-64	62	Not performed	Satisfactory	NILM	NILM	Negative (NILM)	
P07738	2021	2021-Q1	2021-03	Pandemic	Cytology (Pap)	45-49	49	Not performed	Satisfactory	NILM	NILM	Negative (NILM)	
P07739	2021	2021-Q1	2021-03	Pandemic	Cytology (Pap)	35-39	37	Not performed	Satisfactory	NILM	NILM	Negative (NILM)	
P07740	2021	2021-Q1	2021-03	Pandemic	Cytology (Pap)	25-29	28	Not performed	Satisfactory	NILM	NILM	Negative (NILM)	
P07741	2021	2021-Q1	2021-03	Pandemic	Cytology (Pap)	50-54	51	Not performed	Satisfactory	NILM	NILM	Negative (NILM)	
P07742	2021	2021-Q1	2021-03	Pandemic	Cytology (Pap)	50-54	54	Not performed	Satisfactory	NILM	NILM	Negative (NILM)	
P07743	2021	2021-Q2	2021-04	Pandemic	Cytology (Pap)	35-39	36	Not performed	Satisfactory	NILM	NILM	Negative (NILM)	
P07744	2021	2021-Q2	2021-04	Pandemic	Cytology (Pap)	55-59	56	Not performed	Satisfactory	Positive	LSIL	Low-grade / equivocal squamous	
P07745	2021	2021-Q2	2021-04	Pandemic	Cytology (Pap)	25-29	29	Not performed	Satisfactory	NILM	NILM	Negative (NILM)	
P07746	2021	2021-Q2	2021-04	Pandemic	Cytology (Pap)	35-39	35	Not performed	Satisfactory	NILM	NILM	Negative (NILM)	
P07747	2021	2021-Q2	2021-04	Pandemic	Cytology (Pap)	30-34	31	Not performed	Satisfactory	Positive	ASC-US	Low-grade / equivocal squamous	
P07748	2021	2021-Q2	2021-04	Pandemic	Cytology (Pap)	30-34	31	Not performed	Satisfactory	NILM	NILM	Negative (NILM)	
P07749	2021	2021-Q2	2021-04	Pandemic	Cytology (Pap)	50-54	54	Not performed	Satisfactory	NILM	NILM	Negative (NILM)	
P07750	2021	2021-Q2	2021-04	Pandemic	Cytology (Pap)	40-44	40	Not performed	Satisfactory	NILM	NILM	Negative (NILM)	
P07751	2021	2021-Q2	2021-04	Pandemic	Cytology (Pap)	45-49	45	Not performed	Satisfactory	NILM	NILM	Negative (NILM)	
P07752	2021	2021-Q2	2021-04	Pandemic	Cytology (Pap)	40-44	44	Not performed	Satisfactory	Positive	HSIL	High-grade squamous	
P07753	2021	2021-Q2	2021-04	Pandemic	Cytology (Pap)	40-44	40	Not performed	Satisfactory	Positive	ASC-US	Low-grade / equivocal squamous	
P07754	2021	2021-Q2	2021-04	Pandemic	Cytology (Pap)	50-54	52	Not performed	Satisfactory	NILM	NILM	Negative (NILM)	
P07755	2021	2021-Q2	2021-04	Pandemic	Cytology (Pap)	45-49	45	Not performed	Satisfactory	NILM	NILM	Negative (NILM)	Candida
P07756	2021	2021-Q2	2021-04	Pandemic	Cytology (Pap)	40-44	44	Not performed	Satisfactory	NILM	NILM	Negative (NILM)	
P07757	2021	2021-Q2	2021-04	Pandemic	Cytology (Pap)	50-54	52	Not performed	Satisfactory	NILM	NILM	Negative (NILM)	
P07758	2021	2021-Q2	2021-04	Pandemic	Cytology (Pap)	45-49	48	Not performed	Satisfactory	NILM	NILM	Negative (NILM)	

patient_id	year	quarter	month	period	primary_test	age_group	age_years	hpv_result	adequacy	result	bethesda	grade	infection
P07759	2021	2021-Q2	2021-04	Pandemic	Cytology (Pap)	55-59	57	Not performed	Satisfactory	NILM	NILM	Negative (NILM)	
P07760	2021	2021-Q2	2021-04	Pandemic	Cytology (Pap)	45-49	47	Not performed	Satisfactory	NILM	NILM	Negative (NILM)	
P07761	2021	2021-Q2	2021-04	Pandemic	Cytology (Pap)	45-49	48	Not performed	Satisfactory	NILM	NILM	Negative (NILM)	Candida
P07762	2021	2021-Q2	2021-04	Pandemic	Cytology (Pap)	35-39	39	Not performed	Satisfactory	Positive	ASC-US	Low-grade / equivocal squamous	
P07763	2021	2021-Q2	2021-04	Pandemic	Cytology (Pap)	55-59	55	Not performed	Satisfactory	NILM	NILM	Negative (NILM)	
P07764	2021	2021-Q2	2021-04	Pandemic	Cytology (Pap)	50-54	51	Not performed	Satisfactory	NILM	NILM	Negative (NILM)	
P07765	2021	2021-Q2	2021-04	Pandemic	Cytology (Pap)	40-44	44	Not performed	Satisfactory	NILM	NILM	Negative (NILM)	
P07766	2021	2021-Q2	2021-04	Pandemic	Cytology (Pap)	25-29	28	Not performed	Satisfactory	Positive	ASC-US	Low-grade / equivocal squamous	
P07767	2021	2021-Q2	2021-04	Pandemic	Cytology (Pap)	55-59	56	Not performed	Satisfactory	NILM	NILM	Negative (NILM)	
P07768	2021	2021-Q2	2021-04	Pandemic	Cytology (Pap)	50-54	53	Not performed	Satisfactory	NILM	NILM	Negative (NILM)	
P07769	2021	2021-Q2	2021-04	Pandemic	Cytology (Pap)	35-39	35	Not performed	Satisfactory	NILM	NILM	Negative (NILM)	
P07770	2021	2021-Q2	2021-04	Pandemic	Cytology (Pap)	45-49	46	Not performed	Satisfactory	NILM	NILM	Negative (NILM)	
P07771	2021	2021-Q2	2021-04	Pandemic	Cytology (Pap)	30-34	32	Not performed	Satisfactory	Positive	ASC-US	Low-grade / equivocal squamous	
P07772	2021	2021-Q2	2021-04	Pandemic	Cytology (Pap)	35-39	39	Not performed	Satisfactory	NILM	NILM	Negative (NILM)	
P07773	2021	2021-Q2	2021-04	Pandemic	Cytology (Pap)	55-59	56	Not performed	Satisfactory	NILM	NILM	Negative (NILM)	
P07774	2021	2021-Q2	2021-04	Pandemic	Cytology (Pap)	45-49	48	Not performed	Satisfactory	NILM	NILM	Negative (NILM)	
P07775	2021	2021-Q2	2021-04	Pandemic	Cytology (Pap)	50-54	51	Not performed	Satisfactory	NILM	NILM	Negative (NILM)	
P07776	2021	2021-Q2	2021-04	Pandemic	Cytology (Pap)	45-49	46	Not performed	Satisfactory	NILM	NILM	Negative (NILM)	
P07777	2021	2021-Q2	2021-04	Pandemic	Cytology (Pap)	45-49	48	Not performed	Satisfactory	NILM	NILM	Negative (NILM)	
P07778	2021	2021-Q2	2021-04	Pandemic	Cytology (Pap)	45-49	48	Not performed	Satisfactory	NILM	NILM	Negative (NILM)	
P07779	2021	2021-Q2	2021-04	Pandemic	Cytology (Pap)	35-39	39	Not performed	Satisfactory	NILM	NILM	Negative (NILM)	
P07780	2021	2021-Q2	2021-04	Pandemic	Cytology (Pap)	45-49	48	Not performed	Satisfactory	NILM	NILM	Negative (NILM)	
P07781	2021	2021-Q2	2021-04	Pandemic	Cytology (Pap)	35-39	35	Not performed	Satisfactory	NILM	NILM	Negative (NILM)	
P07782	2021	2021-Q2	2021-04	Pandemic	Cytology (Pap)	40-44	43	Not performed	Satisfactory	NILM	NILM	Negative (NILM)	
P07783	2021	2021-Q2	2021-04	Pandemic	Cytology (Pap)	40-44	40	Not performed	Satisfactory	NILM	NILM	Negative (NILM)	
P07784	2021	2021-Q2	2021-04	Pandemic	Cytology (Pap)	45-49	49	Not performed	Satisfactory	NILM	NILM	Negative (NILM)	
P07785	2021	2021-Q2	2021-04	Pandemic	Cytology (Pap)	45-49	46	Not performed	Satisfactory	Positive	ASC-US	Low-grade / equivocal squamous	
P07786	2021	2021-Q2	2021-04	Pandemic	Cytology (Pap)	35-39	39	Not performed	Unsatisfactory	Unsatisfactory	Unsatisfactory	Unsatisfactory	
P07787	2021	2021-Q2	2021-04	Pandemic	Cytology (Pap)	35-39	38	Not performed	Satisfactory	NILM	NILM	Negative (NILM)	
P07788	2021	2021-Q2	2021-04	Pandemic	Cytology (Pap)	30-34	31	Not performed	Satisfactory	NILM	NILM	Negative (NILM)	
P07789	2021	2021-Q2	2021-04	Pandemic	Cytology (Pap)	40-44	43	Not performed	Satisfactory	NILM	NILM	Negative (NILM)	
P07790	2021	2021-Q2	2021-04	Pandemic	Cytology (Pap)	55-59	55	Not performed	Satisfactory	Positive	ASC-H	High-grade squamous	
P07791	2021	2021-Q2	2021-04	Pandemic	Cytology (Pap)	30-34	33	Not performed	Satisfactory	NILM	NILM	Negative (NILM)	
P07792	2021	2021-Q2	2021-04	Pandemic	Cytology (Pap)	45-49	49	Not performed	Satisfactory	NILM	NILM	Negative (NILM)	
P07793	2021	2021-Q2	2021-04	Pandemic	Cytology (Pap)	40-44	42	Not performed	Satisfactory	NILM	NILM	Negative (NILM)	
P07794	2021	2021-Q2	2021-04	Pandemic	Cytology (Pap)	50-54	52	Not performed	Unsatisfactory	Unsatisfactory	Unsatisfactory	Unsatisfactory	
P07795	2021	2021-Q2	2021-04	Pandemic	Cytology (Pap)	45-49	46	Not performed	Satisfactory	NILM	NILM	Negative (NILM)	
P07796	2021	2021-Q2	2021-04	Pandemic	Cytology (Pap)	40-44	43	Not performed	Satisfactory	NILM	NILM	Negative (NILM)	
P07797	2021	2021-Q2	2021-04	Pandemic	Cytology (Pap)	35-39	38	Not performed	Satisfactory	NILM	NILM	Negative (NILM)	
P07798	2021	2021-Q2	2021-04	Pandemic	Cytology (Pap)	30-34	32	Not performed	Satisfactory	NILM	NILM	Negative (NILM)	
P07799	2021	2021-Q2	2021-05	Pandemic	Cytology (Pap)	50-54	52	Not performed	Satisfactory	NILM	NILM	Negative (NILM)	
P07800	2021	2021-Q2	2021-05	Pandemic	Cytology (Pap)	45-49	48	Not performed	Satisfactory	NILM	NILM	Negative (NILM)	
P07801	2021	2021-Q2	2021-05	Pandemic	Cytology (Pap)	40-44	44	Not performed	Satisfactory	NILM	NILM	Negative (NILM)	
P07802	2021	2021-Q2	2021-05	Pandemic	Cytology (Pap)	40-44	40	Not performed	Satisfactory	NILM	NILM	Negative (NILM)	
P07803	2021	2021-Q2	2021-05	Pandemic	Cytology (Pap)	50-54	50	Not performed	Satisfactory	NILM	NILM	Negative (NILM)	
P07804	2021	2021-Q2	2021-05	Pandemic	Cytology (Pap)	40-44	40	Not performed	Satisfactory	NILM	NILM	Negative (NILM)	
P07805	2021	2021-Q2	2021-05	Pandemic	Cytology (Pap)	50-54	51	Not performed	Satisfactory	NILM	NILM	Negative (NILM)	
P07806	2021	2021-Q2	2021-05	Pandemic	Cytology (Pap)	45-49	48	Not performed	Satisfactory	NILM	NILM	Negative (NILM)	
P07807	2021	2021-Q2	2021-05	Pandemic	Cytology (Pap)	40-44	40	Not performed	Satisfactory	Positive	LSIL	Low-grade / equivocal squamous	
P07808	2021	2021-Q2	2021-05	Pandemic	Cytology (Pap)	40-44	40	Not performed	Satisfactory	NILM	NILM	Negative (NILM)	
P07809	2021	2021-Q2	2021-05	Pandemic	Cytology (Pap)	55-59	57	Not performed	Satisfactory	NILM	NILM	Negative (NILM)	
P07810	2021	2021-Q2	2021-05	Pandemic	Cytology (Pap)	35-39	36	Not performed	Satisfactory	Positive	ASC-H	High-grade squamous	
P07811	2021	2021-Q2	2021-05	Pandemic	Cytology (Pap)	35-39	35	Not performed	Satisfactory	NILM	NILM	Negative (NILM)	
P07812	2021	2021-Q2	2021-05	Pandemic	Cytology (Pap)	40-44	44	Not performed	Satisfactory	NILM	NILM	Negative (NILM)	
P07813	2021	2021-Q2	2021-05	Pandemic	Cytology (Pap)	30-34	32	Not performed	Satisfactory	NILM	NILM	Negative (NILM)	
P07814	2021	2021-Q2	2021-05	Pandemic	Cytology (Pap)	40-44	42	Not performed	Satisfactory	NILM	NILM	Negative (NILM)	
P07815	2021	2021-Q2	2021-05	Pandemic	Cytology (Pap)	40-44	42	Not performed	Satisfactory	NILM	NILM	Negative (NILM)	
P07816	2021	2021-Q2	2021-05	Pandemic	Cytology (Pap)	30-34	30	Not performed	Satisfactory	NILM	NILM	Negative (NILM)	
P07817	2021	2021-Q2	2021-05	Pandemic	Cytology (Pap)	35-39	38	Not performed	Satisfactory	NILM	NILM	Negative (NILM)	
P07818	2021	2021-Q2	2021-05	Pandemic	Cytology (Pap)	55-59	56	Not performed	Satisfactory	NILM	NILM	Negative (NILM)	Candida
P07819	2021	2021-Q2	2021-05	Pandemic	Cytology (Pap)	35-39	37	Not performed	Satisfactory	NILM	NILM	Negative (NILM)	
P07820	2021	2021-Q2	2021-05	Pandemic	Cytology (Pap)	55-59	56	Not performed	Satisfactory	NILM	NILM	Negative (NILM)	
P07821	2021	2021-Q2	2021-05	Pandemic	Cytology (Pap)	45-49	49	Not performed	Satisfactory	NILM	NILM	Negative (NILM)	
P07822	2021	2021-Q2	2021-05	Pandemic	Cytology (Pap)	45-49	45	Not performed	Satisfactory	NILM	NILM	Negative (NILM)	
P07823	2021	2021-Q2	2021-05	Pandemic	Cytology (Pap)	35-39	35	Not performed	Satisfactory	NILM	NILM	Negative (NILM)	
P07824	2021	2021-Q2	2021-05	Pandemic	Cytology (Pap)	50-54	54	Not performed	Satisfactory	NILM	NILM	Negative (NILM)	
P07825	2021	2021-Q2	2021-05	Pandemic	Cytology (Pap)	45-49	48	Not performed	Satisfactory	NILM	NILM	Negative (NILM)	
P07826	2021	2021-Q2	2021-05	Pandemic	Cytology (Pap)	35-39	38	Not performed	Satisfactory	NILM	NILM	Negative (NILM)	
P07827	2021	2021-Q2	2021-05	Pandemic	Cytology (Pap)	40-44	41	Not performed	Satisfactory	NILM	NILM	Negative (NILM)	
P07828	2021	2021-Q2	2021-05	Pandemic	Cytology (Pap)	60-64	61	Not performed	Satisfactory	NILM	NILM	Negative (NILM)	
P07829	2021	2021-Q2	2021-05	Pandemic	Cytology (Pap)	45-49	45	Not performed	Satisfactory	NILM	NILM	Negative (NILM)	
P07830	2021	2021-Q2	2021-05	Pandemic	Cytology (Pap)	40-44	40	Not performed	Satisfactory	NILM	NILM	Negative (NILM)	
P07831	2021	2021-Q2	2021-05	Pandemic	Cytology (Pap)	25-29	27	Not performed	Satisfactory	NILM	NILM	Negative (NILM)	
P07832	2021	2021-Q2	2021-05	Pandemic	Cytology (Pap)	40-44	43	Not performed	Satisfactory	NILM	NILM	Negative (NILM)	
P07833	2021	2021-Q2	2021-05	Pandemic	Cytology (Pap)	45-49	49	Not performed	Satisfactory	NILM	NILM	Negative (NILM)	
P07834	2021	2021-Q2	2021-05	Pandemic	Cytology (Pap)	40-44	44	Not performed	Satisfactory	NILM	NILM	Negative (NILM)	Other
P07835	2021	2021-Q2	2021-05	Pandemic	Cytology (Pap)	35-39	36	Not performed	Satisfactory	NILM	NILM	Negative (NILM)	
P07836	2021	2021-Q2	2021-05	Pandemic	Cytology (Pap)	45-49	45	Not performed	Satisfactory	Positive	ASC-US	Low-grade / equivocal squamous	
P07837	2021	2021-Q2	2021-05	Pandemic	Cytology (Pap)	45-49	45	Not performed	Satisfactory	NILM	NILM	Negative (NILM)	
P07838	2021	2021-Q2	2021-05	Pandemic	Cytology (Pap)	40-44	43	Not performed	Satisfactory	NILM	NILM	Negative (NILM)	

patient_id	year	quarter	month	period	primary_test	age_group	age_years	hpv_result	adequacy	result	bethesda	grade	infection
P07839	2021	2021-Q2	2021-05	Pandemic	Cytology (Pap)	25-29	29	Not performed	Satisfactory	NILM	NILM	Negative (NILM)	
P07840	2021	2021-Q2	2021-05	Pandemic	Cytology (Pap)	50-54	54	Not performed	Satisfactory	Positive	ASC-US	Low-grade / equivocal squamous	
P07841	2021	2021-Q2	2021-05	Pandemic	Cytology (Pap)	25-29	25	Not performed	Satisfactory	NILM	NILM	Negative (NILM)	
P07842	2021	2021-Q2	2021-05	Pandemic	Cytology (Pap)	50-54	54	Not performed	Satisfactory	NILM	NILM	Negative (NILM)	
P07843	2021	2021-Q2	2021-05	Pandemic	Cytology (Pap)	25-29	26	Not performed	Satisfactory	NILM	NILM	Negative (NILM)	Gardnerella
P07844	2021	2021-Q2	2021-05	Pandemic	Cytology (Pap)	25-29	28	Not performed	Satisfactory	Positive	ASC-US	Low-grade / equivocal squamous	
P07845	2021	2021-Q2	2021-05	Pandemic	Cytology (Pap)	25-29	29	Not performed	Satisfactory	NILM	NILM	Negative (NILM)	
P07846	2021	2021-Q2	2021-05	Pandemic	Cytology (Pap)	55-59	58	Not performed	Satisfactory	NILM	NILM	Negative (NILM)	
P07847	2021	2021-Q2	2021-05	Pandemic	Cytology (Pap)	25-29	29	Not performed	Satisfactory	Positive	LSIL	Low-grade / equivocal squamous	
P07848	2021	2021-Q2	2021-05	Pandemic	Cytology (Pap)	40-44	40	Not performed	Satisfactory	NILM	NILM	Negative (NILM)	
P07849	2021	2021-Q2	2021-05	Pandemic	Cytology (Pap)	45-49	45	Not performed	Satisfactory	NILM	NILM	Negative (NILM)	
P07850	2021	2021-Q2	2021-05	Pandemic	Cytology (Pap)	40-44	41	Not performed	Satisfactory	NILM	NILM	Negative (NILM)	
P07851	2021	2021-Q2	2021-05	Pandemic	Cytology (Pap)	50-54	53	Not performed	Satisfactory	NILM	NILM	Negative (NILM)	
P07852	2021	2021-Q2	2021-05	Pandemic	Cytology (Pap)	45-49	49	Not performed	Satisfactory	NILM	NILM	Negative (NILM)	
P07853	2021	2021-Q2	2021-05	Pandemic	Cytology (Pap)	30-34	32	Not performed	Satisfactory	NILM	NILM	Negative (NILM)	
P07854	2021	2021-Q2	2021-06	Pandemic	Cytology (Pap)	30-34	32	Not performed	Satisfactory	NILM	NILM	Negative (NILM)	
P07855	2021	2021-Q2	2021-06	Pandemic	Cytology (Pap)	55-59	57	Not performed	Satisfactory	NILM	NILM	Negative (NILM)	
P07856	2021	2021-Q2	2021-06	Pandemic	Cytology (Pap)	40-44	44	Not performed	Satisfactory	NILM	NILM	Negative (NILM)	
P07857	2021	2021-Q2	2021-06	Pandemic	Cytology (Pap)	40-44	40	Not performed	Satisfactory	NILM	NILM	Negative (NILM)	
P07858	2021	2021-Q2	2021-06	Pandemic	Cytology (Pap)	45-49	45	Not performed	Satisfactory	NILM	NILM	Negative (NILM)	
P07859	2021	2021-Q2	2021-06	Pandemic	Cytology (Pap)	40-44	42	Not performed	Unsatisfactory	Unsatisfactory	Unsatisfactory	Unsatisfactory	
P07860	2021	2021-Q2	2021-06	Pandemic	Cytology (Pap)	25-29	25	Not performed	Satisfactory	NILM	NILM	Negative (NILM)	
P07861	2021	2021-Q2	2021-06	Pandemic	Cytology (Pap)	30-34	31	Not performed	Satisfactory	NILM	NILM	Negative (NILM)	
P07862	2021	2021-Q2	2021-06	Pandemic	Cytology (Pap)	45-49	48	Not performed	Satisfactory	NILM	NILM	Negative (NILM)	
P07863	2021	2021-Q2	2021-06	Pandemic	Cytology (Pap)	45-49	47	Not performed	Satisfactory	NILM	NILM	Negative (NILM)	
P07864	2021	2021-Q2	2021-06	Pandemic	Cytology (Pap)	40-44	43	Not performed	Satisfactory	NILM	NILM	Negative (NILM)	
P07865	2021	2021-Q2	2021-06	Pandemic	Cytology (Pap)	60-64	64	Not performed	Satisfactory	NILM	NILM	Negative (NILM)	
P07866	2021	2021-Q2	2021-06	Pandemic	Cytology (Pap)	40-44	44	Not performed	Satisfactory	NILM	NILM	Negative (NILM)	Candida
P07867	2021	2021-Q2	2021-06	Pandemic	Cytology (Pap)	25-29	29	Not performed	Satisfactory	NILM	NILM	Negative (NILM)	
P07868	2021	2021-Q2	2021-06	Pandemic	Cytology (Pap)	55-59	59	Not performed	Satisfactory	NILM	NILM	Negative (NILM)	
P07869	2021	2021-Q2	2021-06	Pandemic	Cytology (Pap)	35-39	39	Not performed	Satisfactory	NILM	NILM	Negative (NILM)	
P07870	2021	2021-Q2	2021-06	Pandemic	Cytology (Pap)	30-34	34	Not performed	Satisfactory	NILM	NILM	Negative (NILM)	
P07871	2021	2021-Q2	2021-06	Pandemic	Cytology (Pap)	50-54	50	Not performed	Unsatisfactory	Unsatisfactory	Unsatisfactory	Unsatisfactory	
P07872	2021	2021-Q2	2021-06	Pandemic	Cytology (Pap)	35-39	38	Not performed	Satisfactory	NILM	NILM	Negative (NILM)	Candida
P07873	2021	2021-Q2	2021-06	Pandemic	Cytology (Pap)	50-54	51	Not performed	Satisfactory	NILM	NILM	Negative (NILM)	
P07874	2021	2021-Q2	2021-06	Pandemic	Cytology (Pap)	45-49	47	Not performed	Satisfactory	NILM	NILM	Negative (NILM)	
P07875	2021	2021-Q2	2021-06	Pandemic	Cytology (Pap)	55-59	56	Not performed	Satisfactory	NILM	NILM	Negative (NILM)	
P07876	2021	2021-Q2	2021-06	Pandemic	Cytology (Pap)	30-34	33	Not performed	Satisfactory	NILM	NILM	Negative (NILM)	
P07877	2021	2021-Q2	2021-06	Pandemic	Cytology (Pap)	40-44	40	Not performed	Satisfactory	Positive	ASC-US	Low-grade / equivocal squamous	
P07878	2021	2021-Q2	2021-06	Pandemic	Cytology (Pap)	45-49	46	Not performed	Satisfactory	NILM	NILM	Negative (NILM)	
P07879	2021	2021-Q2	2021-06	Pandemic	Cytology (Pap)	40-44	42	Not performed	Satisfactory	NILM	NILM	Negative (NILM)	
P07880	2021	2021-Q2	2021-06	Pandemic	Cytology (Pap)	45-49	46	Not performed	Satisfactory	NILM	NILM	Negative (NILM)	
P07881	2021	2021-Q2	2021-06	Pandemic	Cytology (Pap)	40-44	42	Not performed	Satisfactory	NILM	NILM	Negative (NILM)	
P07882	2021	2021-Q2	2021-06	Pandemic	Cytology (Pap)	60-64	60	Not performed	Satisfactory	NILM	NILM	Negative (NILM)	
P07883	2021	2021-Q2	2021-06	Pandemic	Cytology (Pap)	25-29	26	Not performed	Satisfactory	NILM	NILM	Negative (NILM)	
P07884	2021	2021-Q2	2021-06	Pandemic	Cytology (Pap)	35-39	39	Not performed	Satisfactory	NILM	NILM	Negative (NILM)	
P07885	2021	2021-Q2	2021-06	Pandemic	Cytology (Pap)	30-34	32	Not performed	Satisfactory	NILM	NILM	Negative (NILM)	
P07886	2021	2021-Q2	2021-06	Pandemic	Cytology (Pap)	40-44	42	Not performed	Satisfactory	NILM	NILM	Negative (NILM)	
P07887	2021	2021-Q2	2021-06	Pandemic	Cytology (Pap)	45-49	47	Not performed	Satisfactory	NILM	NILM	Negative (NILM)	
P07888	2021	2021-Q2	2021-06	Pandemic	Cytology (Pap)	50-54	54	Not performed	Satisfactory	NILM	NILM	Negative (NILM)	
P07889	2021	2021-Q2	2021-06	Pandemic	Cytology (Pap)	30-34	31	Not performed	Satisfactory	NILM	NILM	Negative (NILM)	
P07890	2021	2021-Q2	2021-06	Pandemic	Cytology (Pap)	50-54	51	Not performed	Satisfactory	Positive	ASC-US	Low-grade / equivocal squamous	
P07891	2021	2021-Q2	2021-06	Pandemic	Cytology (Pap)	40-44	44	Not performed	Satisfactory	Positive	ASC-US	Low-grade / equivocal squamous	Trichomonas
P07892	2021	2021-Q2	2021-06	Pandemic	Cytology (Pap)	30-34	34	Not performed	Satisfactory	NILM	NILM	Negative (NILM)	
P07893	2021	2021-Q2	2021-06	Pandemic	Cytology (Pap)	40-44	44	Not performed	Unsatisfactory	Unsatisfactory	Unsatisfactory	Unsatisfactory	
P07894	2021	2021-Q2	2021-06	Pandemic	Cytology (Pap)	45-49	48	Not performed	Satisfactory	NILM	NILM	Negative (NILM)	
P07895	2021	2021-Q2	2021-06	Pandemic	Cytology (Pap)	40-44	42	Not performed	Satisfactory	NILM	NILM	Negative (NILM)	
P07896	2021	2021-Q2	2021-06	Pandemic	Cytology (Pap)	25-29	26	Not performed	Satisfactory	NILM	NILM	Negative (NILM)	
P07897	2021	2021-Q2	2021-06	Pandemic	Cytology (Pap)	45-49	49	Not performed	Satisfactory	Positive	LSIL	Low-grade / equivocal squamous	
P07898	2021	2021-Q2	2021-06	Pandemic	Cytology (Pap)	35-39	38	Not performed	Satisfactory	NILM	NILM	Negative (NILM)	
P07899	2021	2021-Q2	2021-06	Pandemic	Cytology (Pap)	60-64	63	Not performed	Satisfactory	NILM	NILM	Negative (NILM)	
P07900	2021	2021-Q2	2021-06	Pandemic	Cytology (Pap)	40-44	40	Not performed	Satisfactory	NILM	NILM	Negative (NILM)	
P07901	2021	2021-Q2	2021-06	Pandemic	Cytology (Pap)	45-49	45	Not performed	Satisfactory	NILM	NILM	Negative (NILM)	
P07902	2021	2021-Q2	2021-06	Pandemic	Cytology (Pap)	45-49	45	Not performed	Satisfactory	Positive	LSIL	Low-grade / equivocal squamous	
P07903	2021	2021-Q2	2021-06	Pandemic	Cytology (Pap)	45-49	47	Not performed	Satisfactory	NILM	NILM	Negative (NILM)	
P07904	2021	2021-Q2	2021-06	Pandemic	Cytology (Pap)	40-44	41	Not performed	Satisfactory	NILM	NILM	Negative (NILM)	
P07905	2021	2021-Q2	2021-06	Pandemic	Cytology (Pap)	50-54	50	Not performed	Satisfactory	NILM	NILM	Negative (NILM)	
P07906	2021	2021-Q2	2021-06	Pandemic	Cytology (Pap)	50-54	51	Not performed	Satisfactory	NILM	NILM	Negative (NILM)	
P07907	2021	2021-Q2	2021-06	Pandemic	Cytology (Pap)	45-49	45	Not performed	Satisfactory	NILM	NILM	Negative (NILM)	
P07908	2021	2021-Q2	2021-06	Pandemic	Cytology (Pap)	35-39	38	Not performed	Satisfactory	NILM	NILM	Negative (NILM)	
P07909	2021	2021-Q3	2021-07	Pandemic	Cytology (Pap)	45-49	49	Not performed	Satisfactory	NILM	NILM	Negative (NILM)	
P07910	2021	2021-Q3	2021-07	Pandemic	Cytology (Pap)	45-49	48	Not performed	Satisfactory	NILM	NILM	Negative (NILM)	
P07911	2021	2021-Q3	2021-07	Pandemic	Cytology (Pap)	45-49	45	Not performed	Satisfactory	Positive	ASC-US	Low-grade / equivocal squamous	
P07912	2021	2021-Q3	2021-07	Pandemic	Cytology (Pap)	50-54	51	Not performed	Satisfactory	NILM	NILM	Negative (NILM)	
P07913	2021	2021-Q3	2021-07	Pandemic	Cytology (Pap)	35-39	36	Not performed	Satisfactory	NILM	NILM	Negative (NILM)	
P07914	2021	2021-Q3	2021-07	Pandemic	Cytology (Pap)	45-49	46	Not performed	Satisfactory	NILM	NILM	Negative (NILM)	
P07915	2021	2021-Q3	2021-07	Pandemic	Cytology (Pap)	40-44	42	Not performed	Satisfactory	Positive	ASC-US	Low-grade / equivocal squamous	
P07916	2021	2021-Q3	2021-07	Pandemic	Cytology (Pap)	50-54	50	Not performed	Satisfactory	NILM	NILM	Negative (NILM)	
P07917	2021	2021-Q3	2021-07	Pandemic	Cytology (Pap)	45-49	45	Not performed	Satisfactory	NILM	NILM	Negative (NILM)	
P07918	2021	2021-Q3	2021-07	Pandemic	Cytology (Pap)	40-44	42	Not performed	Satisfactory	NILM	NILM	Negative (NILM)	

patient_id	year	quarter	month	period	primary_test	age_group	age_years	hpv_result	adequacy	result	bethesda	grade	infection
P07919	2021	2021-Q3	2021-07	Pandemic	Cytology (Pap)	30-34	32	Not performed	Satisfactory	NILM	NILM	Negative (NILM)	
P07920	2021	2021-Q3	2021-07	Pandemic	Cytology (Pap)	45-49	46	Not performed	Satisfactory	NILM	NILM	Negative (NILM)	
P07921	2021	2021-Q3	2021-07	Pandemic	Cytology (Pap)	25-29	26	Not performed	Satisfactory	NILM	NILM	Negative (NILM)	
P07922	2021	2021-Q3	2021-07	Pandemic	Cytology (Pap)	25-29	29	Not performed	Satisfactory	NILM	NILM	Negative (NILM)	
P07923	2021	2021-Q3	2021-07	Pandemic	Cytology (Pap)	30-34	34	Not performed	Satisfactory	NILM	NILM	Negative (NILM)	
P07924	2021	2021-Q3	2021-07	Pandemic	Cytology (Pap)	35-39	37	Not performed	Satisfactory	Positive	ASC-US	Low-grade / equivocal squamous	
P07925	2021	2021-Q3	2021-07	Pandemic	Cytology (Pap)	30-34	30	Not performed	Satisfactory	NILM	NILM	Negative (NILM)	
P07926	2021	2021-Q3	2021-07	Pandemic	Cytology (Pap)	35-39	36	Not performed	Satisfactory	NILM	NILM	Negative (NILM)	
P07927	2021	2021-Q3	2021-07	Pandemic	Cytology (Pap)	60-64	60	Not performed	Satisfactory	Positive	AGC	Glandular	
P07928	2021	2021-Q3	2021-07	Pandemic	Cytology (Pap)	35-39	36	Not performed	Satisfactory	NILM	NILM	Negative (NILM)	
P07929	2021	2021-Q3	2021-07	Pandemic	Cytology (Pap)	45-49	48	Not performed	Satisfactory	NILM	NILM	Negative (NILM)	Gardnerella
P07930	2021	2021-Q3	2021-07	Pandemic	Cytology (Pap)	50-54	52	Not performed	Satisfactory	NILM	NILM	Negative (NILM)	
P07931	2021	2021-Q3	2021-07	Pandemic	Cytology (Pap)	45-49	47	Not performed	Satisfactory	Positive	HSIL	High-grade squamous	
P07932	2021	2021-Q3	2021-07	Pandemic	Cytology (Pap)	45-49	46	Not performed	Satisfactory	NILM	NILM	Negative (NILM)	
P07933	2021	2021-Q3	2021-07	Pandemic	Cytology (Pap)	40-44	43	Not performed	Satisfactory	NILM	NILM	Negative (NILM)	
P07934	2021	2021-Q3	2021-07	Pandemic	Cytology (Pap)	30-34	30	Not performed	Satisfactory	NILM	NILM	Negative (NILM)	
P07935	2021	2021-Q3	2021-07	Pandemic	Cytology (Pap)	30-34	30	Not performed	Unsatisfactory	Unsatisfactory	Unsatisfactory	Unsatisfactory	
P07936	2021	2021-Q3	2021-07	Pandemic	Cytology (Pap)	45-49	46	Not performed	Satisfactory	NILM	NILM	Negative (NILM)	
P07937	2021	2021-Q3	2021-07	Pandemic	Cytology (Pap)	55-59	59	Not performed	Satisfactory	NILM	NILM	Negative (NILM)	
P07938	2021	2021-Q3	2021-07	Pandemic	Cytology (Pap)	50-54	53	Not performed	Satisfactory	NILM	NILM	Negative (NILM)	
P07939	2021	2021-Q3	2021-07	Pandemic	Cytology (Pap)	35-39	39	Not performed	Satisfactory	Positive	LSIL	Low-grade / equivocal squamous	
P07940	2021	2021-Q3	2021-07	Pandemic	Cytology (Pap)	55-59	55	Not performed	Satisfactory	NILM	NILM	Negative (NILM)	
P07941	2021	2021-Q3	2021-07	Pandemic	Cytology (Pap)	30-34	32	Not performed	Satisfactory	NILM	NILM	Negative (NILM)	
P07942	2021	2021-Q3	2021-07	Pandemic	Cytology (Pap)	35-39	35	Not performed	Unsatisfactory	Unsatisfactory	Unsatisfactory	Unsatisfactory	
P07943	2021	2021-Q3	2021-07	Pandemic	Cytology (Pap)	45-49	49	Not performed	Satisfactory	NILM	NILM	Negative (NILM)	
P07944	2021	2021-Q3	2021-07	Pandemic	Cytology (Pap)	50-54	52	Not performed	Satisfactory	NILM	NILM	Negative (NILM)	
P07945	2021	2021-Q3	2021-07	Pandemic	Cytology (Pap)	30-34	30	Not performed	Satisfactory	NILM	NILM	Negative (NILM)	
P07946	2021	2021-Q3	2021-07	Pandemic	Cytology (Pap)	40-44	42	Not performed	Satisfactory	NILM	NILM	Negative (NILM)	
P07947	2021	2021-Q3	2021-07	Pandemic	Cytology (Pap)	55-59	57	Not performed	Satisfactory	NILM	NILM	Negative (NILM)	
P07948	2021	2021-Q3	2021-07	Pandemic	Cytology (Pap)	40-44	42	Not performed	Satisfactory	NILM	NILM	Negative (NILM)	
P07949	2021	2021-Q3	2021-07	Pandemic	Cytology (Pap)	45-49	47	Not performed	Satisfactory	NILM	NILM	Negative (NILM)	
P07950	2021	2021-Q3	2021-07	Pandemic	Cytology (Pap)	35-39	35	Not performed	Satisfactory	NILM	NILM	Negative (NILM)	
P07951	2021	2021-Q3	2021-07	Pandemic	Cytology (Pap)	50-54	54	Not performed	Satisfactory	NILM	NILM	Negative (NILM)	
P07952	2021	2021-Q3	2021-07	Pandemic	Cytology (Pap)	55-59	59	Not performed	Satisfactory	NILM	NILM	Negative (NILM)	
P07953	2021	2021-Q3	2021-07	Pandemic	Cytology (Pap)	35-39	38	Not performed	Satisfactory	NILM	NILM	Negative (NILM)	
P07954	2021	2021-Q3	2021-07	Pandemic	Cytology (Pap)	45-49	48	Not performed	Satisfactory	NILM	NILM	Negative (NILM)	
P07955	2021	2021-Q3	2021-07	Pandemic	Cytology (Pap)	35-39	36	Not performed	Satisfactory	NILM	NILM	Negative (NILM)	
P07956	2021	2021-Q3	2021-07	Pandemic	Cytology (Pap)	40-44	40	Not performed	Satisfactory	NILM	NILM	Negative (NILM)	
P07957	2021	2021-Q3	2021-07	Pandemic	Cytology (Pap)	40-44	41	Not performed	Satisfactory	NILM	NILM	Negative (NILM)	
P07958	2021	2021-Q3	2021-07	Pandemic	Cytology (Pap)	45-49	49	Not performed	Satisfactory	NILM	NILM	Negative (NILM)	
P07959	2021	2021-Q3	2021-07	Pandemic	Cytology (Pap)	55-59	55	Not performed	Satisfactory	NILM	NILM	Negative (NILM)	
P07960	2021	2021-Q3	2021-07	Pandemic	Cytology (Pap)	35-39	36	Not performed	Satisfactory	NILM	NILM	Negative (NILM)	
P07961	2021	2021-Q3	2021-07	Pandemic	Cytology (Pap)	35-39	39	Not performed	Satisfactory	NILM	NILM	Negative (NILM)	
P07962	2021	2021-Q3	2021-07	Pandemic	Cytology (Pap)	45-49	46	Not performed	Satisfactory	NILM	NILM	Negative (NILM)	
P07963	2021	2021-Q3	2021-07	Pandemic	Cytology (Pap)	40-44	41	Not performed	Satisfactory	NILM	NILM	Negative (NILM)	
P07964	2021	2021-Q3	2021-07	Pandemic	Cytology (Pap)	40-44	42	Not performed	Satisfactory	Positive	LSIL	Low-grade / equivocal squamous	
P07965	2021	2021-Q3	2021-07	Pandemic	Cytology (Pap)	25-29	28	Not performed	Satisfactory	NILM	NILM	Negative (NILM)	
P07966	2021	2021-Q3	2021-07	Pandemic	Cytology (Pap)	45-49	45	Not performed	Satisfactory	NILM	NILM	Negative (NILM)	
P07967	2021	2021-Q3	2021-07	Pandemic	Cytology (Pap)	50-54	54	Not performed	Satisfactory	NILM	NILM	Negative (NILM)	
P07968	2021	2021-Q3	2021-07	Pandemic	Cytology (Pap)	40-44	40	Not performed	Satisfactory	NILM	NILM	Negative (NILM)	
P07969	2021	2021-Q3	2021-07	Pandemic	Cytology (Pap)	30-34	33	Not performed	Satisfactory	NILM	NILM	Negative (NILM)	
P07970	2021	2021-Q3	2021-07	Pandemic	Cytology (Pap)	45-49	46	Not performed	Satisfactory	NILM	NILM	Negative (NILM)	
P07971	2021	2021-Q3	2021-07	Pandemic	Cytology (Pap)	30-34	34	Not performed	Satisfactory	Positive	ASC-US	Low-grade / equivocal squamous	
P07972	2021	2021-Q3	2021-07	Pandemic	Cytology (Pap)	50-54	50	Not performed	Satisfactory	NILM	NILM	Negative (NILM)	
P07973	2021	2021-Q3	2021-07	Pandemic	Cytology (Pap)	30-34	30	Not performed	Satisfactory	NILM	NILM	Negative (NILM)	
P07974	2021	2021-Q3	2021-07	Pandemic	Cytology (Pap)	55-59	56	Not performed	Satisfactory	NILM	NILM	Negative (NILM)	
P07975	2021	2021-Q3	2021-07	Pandemic	Cytology (Pap)	40-44	44	Not performed	Satisfactory	Positive	AGC	Glandular	
P07976	2021	2021-Q3	2021-07	Pandemic	Cytology (Pap)	30-34	30	Not performed	Satisfactory	NILM	NILM	Negative (NILM)	
P07977	2021	2021-Q3	2021-07	Pandemic	Cytology (Pap)	40-44	44	Not performed	Satisfactory	NILM	NILM	Negative (NILM)	
P07978	2021	2021-Q3	2021-07	Pandemic	Cytology (Pap)	55-59	57	Not performed	Satisfactory	NILM	NILM	Negative (NILM)	
P07979	2021	2021-Q3	2021-07	Pandemic	Cytology (Pap)	45-49	49	Not performed	Unsatisfactory	Unsatisfactory	Unsatisfactory	Unsatisfactory	
P07980	2021	2021-Q3	2021-07	Pandemic	Cytology (Pap)	55-59	58	Not performed	Satisfactory	NILM	NILM	Negative (NILM)	
P07981	2021	2021-Q3	2021-07	Pandemic	Cytology (Pap)	50-54	54	Not performed	Satisfactory	NILM	NILM	Negative (NILM)	
P07982	2021	2021-Q3	2021-07	Pandemic	Cytology (Pap)	45-49	49	Not performed	Satisfactory	NILM	NILM	Negative (NILM)	
P07983	2021	2021-Q3	2021-07	Pandemic	Cytology (Pap)	35-39	37	Not performed	Satisfactory	NILM	NILM	Negative (NILM)	
P07984	2021	2021-Q3	2021-07	Pandemic	Cytology (Pap)	55-59	55	Not performed	Satisfactory	NILM	NILM	Negative (NILM)	
P07985	2021	2021-Q3	2021-07	Pandemic	Cytology (Pap)	40-44	44	Not performed	Satisfactory	NILM	NILM	Negative (NILM)	
P07986	2021	2021-Q3	2021-07	Pandemic	Cytology (Pap)	45-49	45	Not performed	Satisfactory	NILM	NILM	Negative (NILM)	
P07987	2021	2021-Q3	2021-07	Pandemic	Cytology (Pap)	55-59	55	Not performed	Satisfactory	NILM	NILM	Negative (NILM)	
P07988	2021	2021-Q3	2021-07	Pandemic	Cytology (Pap)	25-29	26	Not performed	Satisfactory	NILM	NILM	Negative (NILM)	
P07989	2021	2021-Q3	2021-07	Pandemic	Cytology (Pap)	60-64	63	Not performed	Satisfactory	NILM	NILM	Negative (NILM)	
P07990	2021	2021-Q3	2021-07	Pandemic	Cytology (Pap)	25-29	26	Not performed	Satisfactory	NILM	NILM	Negative (NILM)	
P07991	2021	2021-Q3	2021-07	Pandemic	Cytology (Pap)	45-49	49	Not performed	Satisfactory	Positive	HSIL	High-grade squamous	
P07992	2021	2021-Q3	2021-07	Pandemic	Cytology (Pap)	35-39	37	Not performed	Satisfactory	NILM	NILM	Negative (NILM)	
P07993	2021	2021-Q3	2021-07	Pandemic	Cytology (Pap)	60-64	63	Not performed	Satisfactory	NILM	NILM	Negative (NILM)	
P07994	2021	2021-Q3	2021-07	Pandemic	Cytology (Pap)	40-44	41	Not performed	Satisfactory	NILM	NILM	Negative (NILM)	
P07995	2021	2021-Q3	2021-07	Pandemic	Cytology (Pap)	50-54	50	Not performed	Satisfactory	NILM	NILM	Negative (NILM)	
P07996	2021	2021-Q3	2021-07	Pandemic	Cytology (Pap)	55-59	56	Not performed	Satisfactory	NILM	NILM	Negative (NILM)	
P07997	2021	2021-Q3	2021-07	Pandemic	Cytology (Pap)	45-49	47	Not performed	Satisfactory	Positive	LSIL	Low-grade / equivocal squamous	
P07998	2021	2021-Q3	2021-07	Pandemic	Cytology (Pap)	30-34	32	Not performed	Satisfactory	Positive	LSIL	Low-grade / equivocal squamous	

patient_id	year	quarter	month	period	primary_test	age_group	age_years	hpv_result	adequacy	result	bethesda	grade	infection
P07999	2021	2021-Q3	2021-07	Pandemic	Cytology (Pap)	55-59	57	Not performed	Satisfactory	Positive	HSIL	High-grade squamous	
P08000	2021	2021-Q3	2021-07	Pandemic	Cytology (Pap)	30-34	31	Not performed	Satisfactory	NILM	NILM	Negative (NILM)	
P08001	2021	2021-Q3	2021-07	Pandemic	Cytology (Pap)	35-39	39	Not performed	Satisfactory	NILM	NILM	Negative (NILM)	
P08002	2021	2021-Q3	2021-07	Pandemic	Cytology (Pap)	30-34	30	Not performed	Satisfactory	NILM	NILM	Negative (NILM)	
P08003	2021	2021-Q3	2021-07	Pandemic	Cytology (Pap)	50-54	52	Not performed	Satisfactory	NILM	NILM	Negative (NILM)	
P08004	2021	2021-Q3	2021-07	Pandemic	Cytology (Pap)	40-44	40	Not performed	Satisfactory	NILM	NILM	Negative (NILM)	
P08005	2021	2021-Q3	2021-07	Pandemic	Cytology (Pap)	35-39	39	Not performed	Satisfactory	NILM	NILM	Negative (NILM)	
P08006	2021	2021-Q3	2021-07	Pandemic	Cytology (Pap)	50-54	52	Not performed	Satisfactory	NILM	NILM	Negative (NILM)	
P08007	2021	2021-Q3	2021-07	Pandemic	Cytology (Pap)	30-34	32	Not performed	Satisfactory	NILM	NILM	Negative (NILM)	
P08008	2021	2021-Q3	2021-07	Pandemic	Cytology (Pap)	40-44	42	Not performed	Satisfactory	NILM	NILM	Negative (NILM)	Gardnerella
P08009	2021	2021-Q3	2021-08	Pandemic	Cytology (Pap)	55-59	59	Not performed	Satisfactory	NILM	NILM	Negative (NILM)	Gardnerella
P08010	2021	2021-Q3	2021-08	Pandemic	Cytology (Pap)	40-44	41	Not performed	Satisfactory	NILM	NILM	Negative (NILM)	
P08011	2021	2021-Q3	2021-08	Pandemic	Cytology (Pap)	45-49	46	Not performed	Satisfactory	NILM	NILM	Negative (NILM)	
P08012	2021	2021-Q3	2021-08	Pandemic	Cytology (Pap)	45-49	49	Not performed	Satisfactory	NILM	NILM	Negative (NILM)	
P08013	2021	2021-Q3	2021-08	Pandemic	Cytology (Pap)	50-54	51	Not performed	Satisfactory	NILM	NILM	Negative (NILM)	
P08014	2021	2021-Q3	2021-08	Pandemic	Cytology (Pap)	45-49	47	Not performed	Satisfactory	NILM	NILM	Negative (NILM)	
P08015	2021	2021-Q3	2021-08	Pandemic	Cytology (Pap)	50-54	54	Not performed	Satisfactory	NILM	NILM	Negative (NILM)	
P08016	2021	2021-Q3	2021-08	Pandemic	Cytology (Pap)	40-44	44	Not performed	Satisfactory	NILM	NILM	Negative (NILM)	
P08017	2021	2021-Q3	2021-08	Pandemic	Cytology (Pap)	40-44	41	Not performed	Satisfactory	NILM	NILM	Negative (NILM)	
P08018	2021	2021-Q3	2021-08	Pandemic	Cytology (Pap)	35-39	39	Not performed	Satisfactory	Positive	LSIL	Low-grade / equivocal squamous	
P08019	2021	2021-Q3	2021-08	Pandemic	Cytology (Pap)	45-49	48	Not performed	Satisfactory	NILM	NILM	Negative (NILM)	
P08020	2021	2021-Q3	2021-08	Pandemic	Cytology (Pap)	30-34	32	Not performed	Satisfactory	NILM	NILM	Negative (NILM)	
P08021	2021	2021-Q3	2021-08	Pandemic	Cytology (Pap)	45-49	49	Not performed	Satisfactory	NILM	NILM	Negative (NILM)	
P08022	2021	2021-Q3	2021-08	Pandemic	Cytology (Pap)	40-44	41	Not performed	Satisfactory	NILM	NILM	Negative (NILM)	
P08023	2021	2021-Q3	2021-08	Pandemic	Cytology (Pap)	40-44	41	Not performed	Satisfactory	NILM	NILM	Negative (NILM)	
P08024	2021	2021-Q3	2021-08	Pandemic	Cytology (Pap)	45-49	46	Not performed	Satisfactory	NILM	NILM	Negative (NILM)	
P08025	2021	2021-Q3	2021-08	Pandemic	Cytology (Pap)	35-39	37	Not performed	Satisfactory	NILM	NILM	Negative (NILM)	
P08026	2021	2021-Q3	2021-08	Pandemic	Cytology (Pap)	40-44	41	Not performed	Satisfactory	Positive	AGC	Glandular	
P08027	2021	2021-Q3	2021-08	Pandemic	Cytology (Pap)	40-44	40	Not performed	Satisfactory	Positive	LSIL	Low-grade / equivocal squamous	
P08028	2021	2021-Q3	2021-08	Pandemic	Cytology (Pap)	40-44	43	Not performed	Satisfactory	NILM	NILM	Negative (NILM)	
P08029	2021	2021-Q3	2021-08	Pandemic	Cytology (Pap)	30-34	30	Not performed	Satisfactory	NILM	NILM	Negative (NILM)	
P08030	2021	2021-Q3	2021-08	Pandemic	Cytology (Pap)	30-34	33	Not performed	Satisfactory	NILM	NILM	Negative (NILM)	
P08031	2021	2021-Q3	2021-08	Pandemic	Cytology (Pap)	50-54	50	Not performed	Satisfactory	NILM	NILM	Negative (NILM)	
P08032	2021	2021-Q3	2021-08	Pandemic	Cytology (Pap)	35-39	35	Not performed	Satisfactory	NILM	NILM	Negative (NILM)	
P08033	2021	2021-Q3	2021-08	Pandemic	Cytology (Pap)	25-29	26	Not performed	Satisfactory	NILM	NILM	Negative (NILM)	
P08034	2021	2021-Q3	2021-08	Pandemic	Cytology (Pap)	45-49	46	Not performed	Satisfactory	NILM	NILM	Negative (NILM)	
P08035	2021	2021-Q3	2021-08	Pandemic	Cytology (Pap)	25-29	25	Not performed	Satisfactory	NILM	NILM	Negative (NILM)	
P08036	2021	2021-Q3	2021-08	Pandemic	Cytology (Pap)	60-64	64	Not performed	Satisfactory	NILM	NILM	Negative (NILM)	
P08037	2021	2021-Q3	2021-08	Pandemic	Cytology (Pap)	45-49	47	Not performed	Satisfactory	Positive	HSIL	High-grade squamous	
P08038	2021	2021-Q3	2021-08	Pandemic	Cytology (Pap)	45-49	47	Not performed	Satisfactory	NILM	NILM	Negative (NILM)	
P08039	2021	2021-Q3	2021-08	Pandemic	Cytology (Pap)	25-29	29	Not performed	Satisfactory	NILM	NILM	Negative (NILM)	
P08040	2021	2021-Q3	2021-08	Pandemic	Cytology (Pap)	45-49	46	Not performed	Satisfactory	Positive	HSIL	High-grade squamous	
P08041	2021	2021-Q3	2021-08	Pandemic	Cytology (Pap)	35-39	38	Not performed	Satisfactory	NILM	NILM	Negative (NILM)	
P08042	2021	2021-Q3	2021-08	Pandemic	Cytology (Pap)	45-49	47	Not performed	Satisfactory	NILM	NILM	Negative (NILM)	
P08043	2021	2021-Q3	2021-08	Pandemic	Cytology (Pap)	25-29	25	Not performed	Satisfactory	NILM	NILM	Negative (NILM)	
P08044	2021	2021-Q3	2021-08	Pandemic	Cytology (Pap)	50-54	50	Not performed	Satisfactory	Positive	HSIL	High-grade squamous	
P08045	2021	2021-Q3	2021-08	Pandemic	Cytology (Pap)	35-39	39	Not performed	Satisfactory	NILM	NILM	Negative (NILM)	
P08046	2021	2021-Q3	2021-08	Pandemic	Cytology (Pap)	45-49	48	Not performed	Satisfactory	Positive	ASC-US	Low-grade / equivocal squamous	
P08047	2021	2021-Q3	2021-08	Pandemic	Cytology (Pap)	40-44	41	Not performed	Satisfactory	NILM	NILM	Negative (NILM)	
P08048	2021	2021-Q3	2021-08	Pandemic	Cytology (Pap)	35-39	35	Not performed	Satisfactory	NILM	NILM	Negative (NILM)	Gardnerella
P08049	2021	2021-Q3	2021-08	Pandemic	Cytology (Pap)	50-54	53	Not performed	Satisfactory	NILM	NILM	Negative (NILM)	
P08050	2021	2021-Q3	2021-08	Pandemic	Cytology (Pap)	45-49	47	Not performed	Satisfactory	NILM	NILM	Negative (NILM)	
P08051	2021	2021-Q3	2021-08	Pandemic	Cytology (Pap)	45-49	49	Not performed	Satisfactory	NILM	NILM	Negative (NILM)	
P08052	2021	2021-Q3	2021-08	Pandemic	Cytology (Pap)	45-49	47	Not performed	Satisfactory	Positive	LSIL	Low-grade / equivocal squamous	
P08053	2021	2021-Q3	2021-08	Pandemic	Cytology (Pap)	45-49	47	Not performed	Satisfactory	NILM	NILM	Negative (NILM)	Trichomonas
P08054	2021	2021-Q3	2021-08	Pandemic	Cytology (Pap)	40-44	42	Not performed	Satisfactory	NILM	NILM	Negative (NILM)	
P08055	2021	2021-Q3	2021-08	Pandemic	Cytology (Pap)	45-49	47	Not performed	Satisfactory	NILM	NILM	Negative (NILM)	
P08056	2021	2021-Q3	2021-08	Pandemic	Cytology (Pap)	60-64	61	Not performed	Satisfactory	NILM	NILM	Negative (NILM)	
P08057	2021	2021-Q3	2021-08	Pandemic	Cytology (Pap)	40-44	41	Not performed	Satisfactory	NILM	NILM	Negative (NILM)	
P08058	2021	2021-Q3	2021-08	Pandemic	Cytology (Pap)	40-44	44	Not performed	Satisfactory	NILM	NILM	Negative (NILM)	
P08059	2021	2021-Q3	2021-08	Pandemic	Cytology (Pap)	45-49	47	Not performed	Satisfactory	NILM	NILM	Negative (NILM)	
P08060	2021	2021-Q3	2021-08	Pandemic	Cytology (Pap)	50-54	52	Not performed	Satisfactory	NILM	NILM	Negative (NILM)	
P08061	2021	2021-Q3	2021-08	Pandemic	Cytology (Pap)	25-29	29	Not performed	Satisfactory	NILM	NILM	Negative (NILM)	
P08062	2021	2021-Q3	2021-08	Pandemic	Cytology (Pap)	40-44	43	Not performed	Satisfactory	NILM	NILM	Negative (NILM)	
P08063	2021	2021-Q3	2021-08	Pandemic	Cytology (Pap)	40-44	43	Not performed	Satisfactory	NILM	NILM	Negative (NILM)	
P08064	2021	2021-Q3	2021-08	Pandemic	Cytology (Pap)	45-49	49	Not performed	Satisfactory	NILM	NILM	Negative (NILM)	
P08065	2021	2021-Q3	2021-08	Pandemic	Cytology (Pap)	35-39	39	Not performed	Satisfactory	NILM	NILM	Negative (NILM)	
P08066	2021	2021-Q3	2021-08	Pandemic	Cytology (Pap)	45-49	47	Not performed	Satisfactory	NILM	NILM	Negative (NILM)	
P08067	2021	2021-Q3	2021-08	Pandemic	Cytology (Pap)	45-49	48	Not performed	Satisfactory	NILM	NILM	Negative (NILM)	
P08068	2021	2021-Q3	2021-08	Pandemic	Cytology (Pap)	45-49	45	Not performed	Satisfactory	NILM	NILM	Negative (NILM)	
P08069	2021	2021-Q3	2021-08	Pandemic	Cytology (Pap)	40-44	42	Not performed	Satisfactory	NILM	NILM	Negative (NILM)	
P08070	2021	2021-Q3	2021-08	Pandemic	Cytology (Pap)	45-49	49	Not performed	Satisfactory	NILM	NILM	Negative (NILM)	
P08071	2021	2021-Q3	2021-08	Pandemic	Cytology (Pap)	55-59	55	Not performed	Satisfactory	NILM	NILM	Negative (NILM)	
P08072	2021	2021-Q3	2021-08	Pandemic	Cytology (Pap)	35-39	35	Not performed	Satisfactory	NILM	NILM	Negative (NILM)	Candida
P08073	2021	2021-Q3	2021-08	Pandemic	Cytology (Pap)	40-44	44	Not performed	Satisfactory	NILM	NILM	Negative (NILM)	
P08074	2021	2021-Q3	2021-08	Pandemic	Cytology (Pap)	35-39	37	Not performed	Satisfactory	NILM	NILM	Negative (NILM)	
P08075	2021	2021-Q3	2021-08	Pandemic	Cytology (Pap)	30-34	32	Not performed	Satisfactory	Positive	ASC-H	High-grade squamous	
P08076	2021	2021-Q3	2021-08	Pandemic	Cytology (Pap)	45-49	45	Not performed	Satisfactory	NILM	NILM	Negative (NILM)	
P08077	2021	2021-Q3	2021-08	Pandemic	Cytology (Pap)	40-44	43	Not performed	Satisfactory	NILM	NILM	Negative (NILM)	
P08078	2021	2021-Q3	2021-08	Pandemic	Cytology (Pap)	40-44	43	Not performed	Unsatisfactory	Unsatisfactory	Unsatisfactory	Unsatisfactory	

patient_id	year	quarter	month	period	primary_test	age_group	age_years	hpv_result	adequacy	result	bethesda	grade	infection
P08079	2021	2021-Q3	2021-08	Pandemic	Cytology (Pap)	50-54	51	Not performed	Satisfactory	NILM	NILM	Negative (NILM)	Candida
P08080	2021	2021-Q3	2021-08	Pandemic	Cytology (Pap)	35-39	35	Not performed	Satisfactory	NILM	NILM	Negative (NILM)	
P08081	2021	2021-Q3	2021-08	Pandemic	Cytology (Pap)	55-59	59	Not performed	Satisfactory	NILM	NILM	Negative (NILM)	
P08082	2021	2021-Q3	2021-08	Pandemic	Cytology (Pap)	35-39	38	Not performed	Satisfactory	NILM	NILM	Negative (NILM)	
P08083	2021	2021-Q3	2021-08	Pandemic	Cytology (Pap)	55-59	57	Not performed	Satisfactory	Positive	ASC-US	Low-grade / equivocal squamous	
P08084	2021	2021-Q3	2021-08	Pandemic	Cytology (Pap)	60-64	63	Not performed	Satisfactory	Positive	ASC-US	Low-grade / equivocal squamous	
P08085	2021	2021-Q3	2021-08	Pandemic	Cytology (Pap)	40-44	44	Not performed	Satisfactory	NILM	NILM	Negative (NILM)	
P08086	2021	2021-Q3	2021-08	Pandemic	Cytology (Pap)	35-39	38	Not performed	Satisfactory	NILM	NILM	Negative (NILM)	
P08087	2021	2021-Q3	2021-08	Pandemic	Cytology (Pap)	45-49	46	Not performed	Satisfactory	NILM	NILM	Negative (NILM)	Candida
P08088	2021	2021-Q3	2021-08	Pandemic	Cytology (Pap)	30-34	34	Not performed	Satisfactory	NILM	NILM	Negative (NILM)	
P08089	2021	2021-Q3	2021-08	Pandemic	Cytology (Pap)	50-54	53	Not performed	Satisfactory	NILM	NILM	Negative (NILM)	
P08090	2021	2021-Q3	2021-08	Pandemic	Cytology (Pap)	25-29	25	Not performed	Satisfactory	Positive	LSIL	Low-grade / equivocal squamous	
P08091	2021	2021-Q3	2021-08	Pandemic	Cytology (Pap)	40-44	43	Not performed	Satisfactory	NILM	NILM	Negative (NILM)	
P08092	2021	2021-Q3	2021-08	Pandemic	Cytology (Pap)	55-59	56	Not performed	Satisfactory	NILM	NILM	Negative (NILM)	
P08093	2021	2021-Q3	2021-08	Pandemic	Cytology (Pap)	45-49	48	Not performed	Satisfactory	NILM	NILM	Negative (NILM)	
P08094	2021	2021-Q3	2021-08	Pandemic	Cytology (Pap)	30-34	31	Not performed	Satisfactory	NILM	NILM	Negative (NILM)	
P08095	2021	2021-Q3	2021-08	Pandemic	Cytology (Pap)	35-39	35	Not performed	Satisfactory	NILM	NILM	Negative (NILM)	
P08096	2021	2021-Q3	2021-08	Pandemic	Cytology (Pap)	40-44	44	Not performed	Satisfactory	NILM	NILM	Negative (NILM)	
P08097	2021	2021-Q3	2021-08	Pandemic	Cytology (Pap)	55-59	55	Not performed	Satisfactory	Positive	ASC-US	Low-grade / equivocal squamous	
P08098	2021	2021-Q3	2021-08	Pandemic	Cytology (Pap)	35-39	36	Not performed	Unsatisfactory	Unsatisfactory	Unsatisfactory	Unsatisfactory	
P08099	2021	2021-Q3	2021-08	Pandemic	Cytology (Pap)	30-34	33	Not performed	Satisfactory	NILM	NILM	Negative (NILM)	
P08100	2021	2021-Q3	2021-08	Pandemic	Cytology (Pap)	35-39	36	Not performed	Satisfactory	NILM	NILM	Negative (NILM)	
P08101	2021	2021-Q3	2021-08	Pandemic	Cytology (Pap)	45-49	46	Not performed	Satisfactory	Positive	AGC	Glandular	
P08102	2021	2021-Q3	2021-08	Pandemic	Cytology (Pap)	50-54	51	Not performed	Satisfactory	Positive	LSIL	Low-grade / equivocal squamous	
P08103	2021	2021-Q3	2021-08	Pandemic	Cytology (Pap)	55-59	58	Not performed	Satisfactory	NILM	NILM	Negative (NILM)	
P08104	2021	2021-Q3	2021-08	Pandemic	Cytology (Pap)	60-64	63	Not performed	Satisfactory	NILM	NILM	Negative (NILM)	
P08105	2021	2021-Q3	2021-08	Pandemic	Cytology (Pap)	45-49	49	Not performed	Satisfactory	NILM	NILM	Negative (NILM)	
P08106	2021	2021-Q3	2021-08	Pandemic	Cytology (Pap)	45-49	45	Not performed	Satisfactory	Positive	HSIL	High-grade squamous	
P08107	2021	2021-Q3	2021-08	Pandemic	Cytology (Pap)	30-34	34	Not performed	Satisfactory	NILM	NILM	Negative (NILM)	
P08108	2021	2021-Q3	2021-08	Pandemic	Cytology (Pap)	45-49	49	Not performed	Satisfactory	NILM	NILM	Negative (NILM)	
P08109	2021	2021-Q3	2021-09	Pandemic	Cytology (Pap)	40-44	41	Not performed	Satisfactory	NILM	NILM	Negative (NILM)	Candida
P08110	2021	2021-Q3	2021-09	Pandemic	Cytology (Pap)	25-29	26	Not performed	Satisfactory	NILM	NILM	Negative (NILM)	
P08111	2021	2021-Q3	2021-09	Pandemic	Cytology (Pap)	30-34	30	Not performed	Satisfactory	NILM	NILM	Negative (NILM)	
P08112	2021	2021-Q3	2021-09	Pandemic	Cytology (Pap)	45-49	48	Not performed	Satisfactory	NILM	NILM	Negative (NILM)	
P08113	2021	2021-Q3	2021-09	Pandemic	Cytology (Pap)	50-54	51	Not performed	Satisfactory	NILM	NILM	Negative (NILM)	
P08114	2021	2021-Q3	2021-09	Pandemic	Cytology (Pap)	60-64	64	Not performed	Satisfactory	NILM	NILM	Negative (NILM)	
P08115	2021	2021-Q3	2021-09	Pandemic	Cytology (Pap)	35-39	39	Not performed	Satisfactory	Positive	HSIL	High-grade squamous	
P08116	2021	2021-Q3	2021-09	Pandemic	Cytology (Pap)	35-39	36	Not performed	Satisfactory	NILM	NILM	Negative (NILM)	
P08117	2021	2021-Q3	2021-09	Pandemic	Cytology (Pap)	40-44	42	Not performed	Satisfactory	NILM	NILM	Negative (NILM)	
P08118	2021	2021-Q3	2021-09	Pandemic	Cytology (Pap)	35-39	37	Not performed	Satisfactory	NILM	NILM	Negative (NILM)	
P08119	2021	2021-Q3	2021-09	Pandemic	Cytology (Pap)	55-59	58	Not performed	Satisfactory	NILM	NILM	Negative (NILM)	
P08120	2021	2021-Q3	2021-09	Pandemic	Cytology (Pap)	45-49	49	Not performed	Satisfactory	NILM	NILM	Negative (NILM)	
P08121	2021	2021-Q3	2021-09	Pandemic	Cytology (Pap)	45-49	45	Not performed	Satisfactory	NILM	NILM	Negative (NILM)	
P08122	2021	2021-Q3	2021-09	Pandemic	Cytology (Pap)	40-44	40	Not performed	Satisfactory	NILM	NILM	Negative (NILM)	
P08123	2021	2021-Q3	2021-09	Pandemic	Cytology (Pap)	40-44	40	Not performed	Satisfactory	NILM	NILM	Negative (NILM)	
P08124	2021	2021-Q3	2021-09	Pandemic	Cytology (Pap)	50-54	50	Not performed	Satisfactory	NILM	NILM	Negative (NILM)	
P08125	2021	2021-Q3	2021-09	Pandemic	Cytology (Pap)	55-59	55	Not performed	Satisfactory	NILM	NILM	Negative (NILM)	
P08126	2021	2021-Q3	2021-09	Pandemic	Cytology (Pap)	45-49	47	Not performed	Satisfactory	NILM	NILM	Negative (NILM)	
P08127	2021	2021-Q3	2021-09	Pandemic	Cytology (Pap)	35-39	37	Not performed	Satisfactory	NILM	NILM	Negative (NILM)	
P08128	2021	2021-Q3	2021-09	Pandemic	Cytology (Pap)	45-49	48	Not performed	Satisfactory	NILM	NILM	Negative (NILM)	
P08129	2021	2021-Q3	2021-09	Pandemic	Cytology (Pap)	35-39	39	Not performed	Satisfactory	Positive	ASC-H	High-grade squamous	
P08130	2021	2021-Q3	2021-09	Pandemic	Cytology (Pap)	50-54	52	Not performed	Satisfactory	NILM	NILM	Negative (NILM)	Other
P08131	2021	2021-Q3	2021-09	Pandemic	Cytology (Pap)	50-54	50	Not performed	Satisfactory	NILM	NILM	Negative (NILM)	
P08132	2021	2021-Q3	2021-09	Pandemic	Cytology (Pap)	45-49	49	Not performed	Satisfactory	NILM	NILM	Negative (NILM)	
P08133	2021	2021-Q3	2021-09	Pandemic	Cytology (Pap)	30-34	34	Not performed	Satisfactory	NILM	NILM	Negative (NILM)	
P08134	2021	2021-Q3	2021-09	Pandemic	Cytology (Pap)	45-49	45	Not performed	Satisfactory	NILM	NILM	Negative (NILM)	
P08135	2021	2021-Q3	2021-09	Pandemic	Cytology (Pap)	25-29	25	Not performed	Satisfactory	Positive	ASC-H	High-grade squamous	
P08136	2021	2021-Q3	2021-09	Pandemic	Cytology (Pap)	40-44	44	Not performed	Satisfactory	NILM	NILM	Negative (NILM)	
P08137	2021	2021-Q3	2021-09	Pandemic	Cytology (Pap)	35-39	38	Not performed	Satisfactory	NILM	NILM	Negative (NILM)	
P08138	2021	2021-Q3	2021-09	Pandemic	Cytology (Pap)	40-44	41	Not performed	Satisfactory	NILM	NILM	Negative (NILM)	Other
P08139	2021	2021-Q3	2021-09	Pandemic	Cytology (Pap)	55-59	59	Not performed	Satisfactory	NILM	NILM	Negative (NILM)	
P08140	2021	2021-Q3	2021-09	Pandemic	Cytology (Pap)	35-39	36	Not performed	Satisfactory	NILM	NILM	Negative (NILM)	
P08141	2021	2021-Q3	2021-09	Pandemic	Cytology (Pap)	50-54	51	Not performed	Satisfactory	NILM	NILM	Negative (NILM)	
P08142	2021	2021-Q3	2021-09	Pandemic	Cytology (Pap)	55-59	57	Not performed	Satisfactory	NILM	NILM	Negative (NILM)	
P08143	2021	2021-Q3	2021-09	Pandemic	Cytology (Pap)	35-39	36	Not performed	Satisfactory	NILM	NILM	Negative (NILM)	
P08144	2021	2021-Q3	2021-09	Pandemic	Cytology (Pap)	30-34	31	Not performed	Unsatisfactory	Unsatisfactory	Unsatisfactory	Unsatisfactory	
P08145	2021	2021-Q3	2021-09	Pandemic	Cytology (Pap)	55-59	59	Not performed	Satisfactory	NILM	NILM	Negative (NILM)	
P08146	2021	2021-Q3	2021-09	Pandemic	Cytology (Pap)	25-29	28	Not performed	Satisfactory	NILM	NILM	Negative (NILM)	
P08147	2021	2021-Q3	2021-09	Pandemic	Cytology (Pap)	45-49	46	Not performed	Satisfactory	NILM	NILM	Negative (NILM)	
P08148	2021	2021-Q3	2021-09	Pandemic	Cytology (Pap)	50-54	50	Not performed	Satisfactory	NILM	NILM	Negative (NILM)	
P08149	2021	2021-Q3	2021-09	Pandemic	Cytology (Pap)	45-49	46	Not performed	Satisfactory	NILM	NILM	Negative (NILM)	
P08150	2021	2021-Q3	2021-09	Pandemic	Cytology (Pap)	30-34	32	Not performed	Satisfactory	NILM	NILM	Negative (NILM)	
P08151	2021	2021-Q3	2021-09	Pandemic	Cytology (Pap)	55-59	56	Not performed	Satisfactory	NILM	NILM	Negative (NILM)	
P08152	2021	2021-Q3	2021-09	Pandemic	Cytology (Pap)	25-29	26	Not performed	Satisfactory	NILM	NILM	Negative (NILM)	
P08153	2021	2021-Q3	2021-09	Pandemic	Cytology (Pap)	35-39	38	Not performed	Satisfactory	NILM	NILM	Negative (NILM)	
P08154	2021	2021-Q3	2021-09	Pandemic	Cytology (Pap)	50-54	54	Not performed	Satisfactory	NILM	NILM	Negative (NILM)	
P08155	2021	2021-Q3	2021-09	Pandemic	Cytology (Pap)	35-39	35	Not performed	Satisfactory	NILM	NILM	Negative (NILM)	
P08156	2021	2021-Q3	2021-09	Pandemic	Cytology (Pap)	40-44	44	Not performed	Satisfactory	NILM	NILM	Negative (NILM)	
P08157	2021	2021-Q3	2021-09	Pandemic	Cytology (Pap)	50-54	51	Not performed	Satisfactory	Positive	HSIL	High-grade squamous	
P08158	2021	2021-Q3	2021-09	Pandemic	Cytology (Pap)	40-44	43	Not performed	Satisfactory	NILM	NILM	Negative (NILM)	

patient_id	year	quarter	month	period	primary_test	age_group	age_years	hpv_result	adequacy	result	bethesda	grade	infection
P08159	2021	2021-Q3	2021-09	Pandemic	Cytology (Pap)	30-34	31	Not performed	Satisfactory	Positive	HSIL	High-grade squamous	
P08160	2021	2021-Q3	2021-09	Pandemic	Cytology (Pap)	45-49	48	Not performed	Satisfactory	NILM	NILM	Negative (NILM)	
P08161	2021	2021-Q3	2021-09	Pandemic	Cytology (Pap)	25-29	27	Not performed	Satisfactory	NILM	NILM	Negative (NILM)	
P08162	2021	2021-Q3	2021-09	Pandemic	Cytology (Pap)	35-39	39	Not performed	Satisfactory	Positive	ASC-H	High-grade squamous	
P08163	2021	2021-Q3	2021-09	Pandemic	Cytology (Pap)	55-59	55	Not performed	Satisfactory	NILM	NILM	Negative (NILM)	
P08164	2021	2021-Q3	2021-09	Pandemic	Cytology (Pap)	35-39	37	Not performed	Satisfactory	NILM	NILM	Negative (NILM)	
P08165	2021	2021-Q3	2021-09	Pandemic	Cytology (Pap)	35-39	39	Not performed	Satisfactory	NILM	NILM	Negative (NILM)	
P08166	2021	2021-Q3	2021-09	Pandemic	Cytology (Pap)	45-49	49	Not performed	Satisfactory	Positive	ASC-H	High-grade squamous	
P08167	2021	2021-Q3	2021-09	Pandemic	Cytology (Pap)	25-29	26	Not performed	Satisfactory	NILM	NILM	Negative (NILM)	
P08168	2021	2021-Q3	2021-09	Pandemic	Cytology (Pap)	45-49	48	Not performed	Satisfactory	NILM	NILM	Negative (NILM)	
P08169	2021	2021-Q3	2021-09	Pandemic	Cytology (Pap)	30-34	33	Not performed	Satisfactory	NILM	NILM	Negative (NILM)	
P08170	2021	2021-Q3	2021-09	Pandemic	Cytology (Pap)	45-49	49	Not performed	Satisfactory	NILM	NILM	Negative (NILM)	
P08171	2021	2021-Q3	2021-09	Pandemic	Cytology (Pap)	60-64	60	Not performed	Satisfactory	Positive	ASC-US	Low-grade / equivocal squamous	
P08172	2021	2021-Q3	2021-09	Pandemic	Cytology (Pap)	30-34	30	Not performed	Satisfactory	NILM	NILM	Negative (NILM)	Candida
P08173	2021	2021-Q3	2021-09	Pandemic	Cytology (Pap)	30-34	33	Not performed	Satisfactory	NILM	NILM	Negative (NILM)	
P08174	2021	2021-Q3	2021-09	Pandemic	Cytology (Pap)	35-39	36	Not performed	Satisfactory	NILM	NILM	Negative (NILM)	
P08175	2021	2021-Q3	2021-09	Pandemic	Cytology (Pap)	35-39	37	Not performed	Satisfactory	NILM	NILM	Negative (NILM)	
P08176	2021	2021-Q3	2021-09	Pandemic	Cytology (Pap)	40-44	41	Not performed	Satisfactory	NILM	NILM	Negative (NILM)	
P08177	2021	2021-Q3	2021-09	Pandemic	Cytology (Pap)	45-49	47	Not performed	Satisfactory	Positive	ASC-US	Low-grade / equivocal squamous	
P08178	2021	2021-Q3	2021-09	Pandemic	Cytology (Pap)	25-29	25	Not performed	Satisfactory	NILM	NILM	Negative (NILM)	
P08179	2021	2021-Q3	2021-09	Pandemic	Cytology (Pap)	40-44	42	Not performed	Satisfactory	NILM	NILM	Negative (NILM)	
P08180	2021	2021-Q3	2021-09	Pandemic	Cytology (Pap)	55-59	56	Not performed	Satisfactory	NILM	NILM	Negative (NILM)	
P08181	2021	2021-Q3	2021-09	Pandemic	Cytology (Pap)	25-29	27	Not performed	Satisfactory	NILM	NILM	Negative (NILM)	
P08182	2021	2021-Q3	2021-09	Pandemic	Cytology (Pap)	50-54	52	Not performed	Satisfactory	NILM	NILM	Negative (NILM)	
P08183	2021	2021-Q3	2021-09	Pandemic	Cytology (Pap)	50-54	50	Not performed	Satisfactory	NILM	NILM	Negative (NILM)	
P08184	2021	2021-Q3	2021-09	Pandemic	Cytology (Pap)	45-49	49	Not performed	Satisfactory	Positive	ASC-US	Low-grade / equivocal squamous	
P08185	2021	2021-Q3	2021-09	Pandemic	Cytology (Pap)	55-59	58	Not performed	Satisfactory	NILM	NILM	Negative (NILM)	Candida
P08186	2021	2021-Q3	2021-09	Pandemic	Cytology (Pap)	30-34	34	Not performed	Satisfactory	NILM	NILM	Negative (NILM)	
P08187	2021	2021-Q3	2021-09	Pandemic	Cytology (Pap)	30-34	34	Not performed	Satisfactory	NILM	NILM	Negative (NILM)	
P08188	2021	2021-Q3	2021-09	Pandemic	Cytology (Pap)	50-54	53	Not performed	Satisfactory	NILM	NILM	Negative (NILM)	
P08189	2021	2021-Q3	2021-09	Pandemic	Cytology (Pap)	35-39	35	Not performed	Satisfactory	NILM	NILM	Negative (NILM)	
P08190	2021	2021-Q3	2021-09	Pandemic	Cytology (Pap)	35-39	36	Not performed	Satisfactory	NILM	NILM	Negative (NILM)	
P08191	2021	2021-Q3	2021-09	Pandemic	Cytology (Pap)	35-39	35	Not performed	Unsatisfactory	Unsatisfactory	Unsatisfactory	Unsatisfactory	Other
P08192	2021	2021-Q3	2021-09	Pandemic	Cytology (Pap)	25-29	29	Not performed	Satisfactory	NILM	NILM	Negative (NILM)	
P08193	2021	2021-Q3	2021-09	Pandemic	Cytology (Pap)	55-59	59	Not performed	Satisfactory	NILM	NILM	Negative (NILM)	
P08194	2021	2021-Q3	2021-09	Pandemic	Cytology (Pap)	25-29	27	Not performed	Satisfactory	NILM	NILM	Negative (NILM)	
P08195	2021	2021-Q3	2021-09	Pandemic	Cytology (Pap)	40-44	42	Not performed	Satisfactory	NILM	NILM	Negative (NILM)	
P08196	2021	2021-Q3	2021-09	Pandemic	Cytology (Pap)	60-64	63	Not performed	Satisfactory	NILM	NILM	Negative (NILM)	
P08197	2021	2021-Q3	2021-09	Pandemic	Cytology (Pap)	30-34	31	Not performed	Satisfactory	Positive	LSIL	Low-grade / equivocal squamous	
P08198	2021	2021-Q3	2021-09	Pandemic	Cytology (Pap)	30-34	30	Not performed	Satisfactory	NILM	NILM	Negative (NILM)	
P08199	2021	2021-Q3	2021-09	Pandemic	Cytology (Pap)	40-44	42	Not performed	Unsatisfactory	Unsatisfactory	Unsatisfactory	Unsatisfactory	
P08200	2021	2021-Q3	2021-09	Pandemic	Cytology (Pap)	25-29	25	Not performed	Satisfactory	NILM	NILM	Negative (NILM)	
P08201	2021	2021-Q3	2021-09	Pandemic	Cytology (Pap)	30-34	31	Not performed	Satisfactory	NILM	NILM	Negative (NILM)	
P08202	2021	2021-Q3	2021-09	Pandemic	Cytology (Pap)	30-34	30	Not performed	Satisfactory	NILM	NILM	Negative (NILM)	
P08203	2021	2021-Q3	2021-09	Pandemic	Cytology (Pap)	30-34	33	Not performed	Satisfactory	NILM	NILM	Negative (NILM)	
P08204	2021	2021-Q3	2021-09	Pandemic	Cytology (Pap)	50-54	53	Not performed	Unsatisfactory	Unsatisfactory	Unsatisfactory	Unsatisfactory	
P08205	2021	2021-Q3	2021-09	Pandemic	Cytology (Pap)	45-49	48	Not performed	Satisfactory	NILM	NILM	Negative (NILM)	
P08206	2021	2021-Q3	2021-09	Pandemic	Cytology (Pap)	45-49	49	Not performed	Satisfactory	NILM	NILM	Negative (NILM)	
P08207	2021	2021-Q3	2021-09	Pandemic	Cytology (Pap)	35-39	37	Not performed	Satisfactory	NILM	NILM	Negative (NILM)	
P08208	2022	2022-Q1	2022-01	Post-pandemic recovery	Cytology (Pap)	25-29	26	Not performed	Satisfactory	NILM	NILM	Negative (NILM)	
P08209	2022	2022-Q1	2022-01	Post-pandemic recovery	Cytology (Pap)	45-49	45	Not performed	Satisfactory	NILM	NILM	Negative (NILM)	
P08210	2022	2022-Q1	2022-01	Post-pandemic recovery	Cytology (Pap)	35-39	39	Not performed	Unsatisfactory	Unsatisfactory	Unsatisfactory	Unsatisfactory	
P08211	2022	2022-Q1	2022-01	Post-pandemic recovery	Cytology (Pap)	40-44	41	Not performed	Satisfactory	NILM	NILM	Negative (NILM)	
P08212	2022	2022-Q1	2022-01	Post-pandemic recovery	Cytology (Pap)	50-54	52	Not performed	Satisfactory	NILM	NILM	Negative (NILM)	
P08213	2022	2022-Q1	2022-01	Post-pandemic recovery	Cytology (Pap)	30-34	34	Not performed	Satisfactory	NILM	NILM	Negative (NILM)	
P08214	2022	2022-Q1	2022-01	Post-pandemic recovery	Cytology (Pap)	30-34	33	Not performed	Satisfactory	NILM	NILM	Negative (NILM)	
P08215	2022	2022-Q1	2022-01	Post-pandemic recovery	Cytology (Pap)	40-44	44	Not performed	Satisfactory	NILM	NILM	Negative (NILM)	
P08216	2022	2022-Q1	2022-01	Post-pandemic recovery	Cytology (Pap)	30-34	32	Not performed	Satisfactory	NILM	NILM	Negative (NILM)	
P08217	2022	2022-Q1	2022-01	Post-pandemic recovery	Cytology (Pap)	40-44	42	Not performed	Satisfactory	NILM	NILM	Negative (NILM)	
P08218	2022	2022-Q1	2022-01	Post-pandemic recovery	Cytology (Pap)	40-44	40	Not performed	Satisfactory	NILM	NILM	Negative (NILM)	
P08219	2022	2022-Q1	2022-01	Post-pandemic recovery	Cytology (Pap)	45-49	45	Not performed	Satisfactory	NILM	NILM	Negative (NILM)	
P08220	2022	2022-Q1	2022-01	Post-pandemic recovery	Cytology (Pap)	45-49	47	Not performed	Satisfactory	NILM	NILM	Negative (NILM)	
P08221	2022	2022-Q1	2022-01	Post-pandemic recovery	Cytology (Pap)	60-64	63	Not performed	Satisfactory	Positive	ASC-US	Low-grade / equivocal squamous	
P08222	2022	2022-Q1	2022-01	Post-pandemic recovery	Cytology (Pap)	45-49	45	Not performed	Satisfactory	Positive	ASC-US	Low-grade / equivocal squamous	Candida
P08223	2022	2022-Q1	2022-01	Post-pandemic recovery	Cytology (Pap)	35-39	35	Not performed	Satisfactory	NILM	NILM	Negative (NILM)	
P08224	2022	2022-Q1	2022-01	Post-pandemic recovery	Cytology (Pap)	25-29	28	Not performed	Satisfactory	NILM	NILM	Negative (NILM)	
P08225	2022	2022-Q1	2022-01	Post-pandemic recovery	Cytology (Pap)	35-39	35	Not performed	Satisfactory	NILM	NILM	Negative (NILM)	Candida
P08226	2022	2022-Q1	2022-01	Post-pandemic recovery	Cytology (Pap)	45-49	48	Not performed	Satisfactory	NILM	NILM	Negative (NILM)	
P08227	2022	2022-Q1	2022-01	Post-pandemic recovery	Cytology (Pap)	40-44	41	Not performed	Satisfactory	NILM	NILM	Negative (NILM)	
P08228	2022	2022-Q1	2022-01	Post-pandemic recovery	Cytology (Pap)	35-39	36	Not performed	Satisfactory	NILM	NILM	Negative (NILM)	
P08229	2022	2022-Q1	2022-01	Post-pandemic recovery	Cytology (Pap)	40-44	42	Not performed	Satisfactory	NILM	NILM	Negative (NILM)	
P08230	2022	2022-Q1	2022-01	Post-pandemic recovery	Cytology (Pap)	30-34	31	Not performed	Satisfactory	NILM	NILM	Negative (NILM)	
P08231	2022	2022-Q1	2022-01	Post-pandemic recovery	Cytology (Pap)	50-54	53	Not performed	Satisfactory	NILM	NILM	Negative (NILM)	
P08232	2022	2022-Q1	2022-01	Post-pandemic recovery	Cytology (Pap)	30-34	34	Not performed	Satisfactory	NILM	NILM	Negative (NILM)	
P08233	2022	2022-Q1	2022-01	Post-pandemic recovery	Cytology (Pap)	40-44	42	Not performed	Satisfactory	NILM	NILM	Negative (NILM)	
P08234	2022	2022-Q1	2022-01	Post-pandemic recovery	Cytology (Pap)	40-44	40	Not performed	Satisfactory	NILM	NILM	Negative (NILM)	
P08235	2022	2022-Q1	2022-01	Post-pandemic recovery	Cytology (Pap)	40-44	44	Not performed	Satisfactory	NILM	NILM	Negative (NILM)	
P08236	2022	2022-Q1	2022-01	Post-pandemic recovery	Cytology (Pap)	40-44	41	Not performed	Satisfactory	NILM	NILM	Negative (NILM)	
P08237	2022	2022-Q1	2022-01	Post-pandemic recovery	Cytology (Pap)	50-54	52	Not performed	Satisfactory	NILM	NILM	Negative (NILM)	
P08238	2022	2022-Q1	2022-01	Post-pandemic recovery	Cytology (Pap)	25-29	27	Not performed	Satisfactory	Positive	HSIL	High-grade squamous	

patient_id	year	quarter	month	period	primary_test	age_group	age_years	hpv_result	adequacy	result	bethesda	grade	infection
P08239	2022	2022-Q1	2022-01	Post-pandemic recovery	Cytology (Pap)	45-49	45	Not performed	Satisfactory	NILM	NILM	Negative (NILM)	
P08240	2022	2022-Q1	2022-01	Post-pandemic recovery	Cytology (Pap)	40-44	41	Not performed	Satisfactory	NILM	NILM	Negative (NILM)	
P08241	2022	2022-Q1	2022-01	Post-pandemic recovery	Cytology (Pap)	40-44	40	Not performed	Satisfactory	NILM	NILM	Negative (NILM)	
P08242	2022	2022-Q1	2022-01	Post-pandemic recovery	Cytology (Pap)	30-34	34	Not performed	Satisfactory	NILM	NILM	Negative (NILM)	
P08243	2022	2022-Q1	2022-01	Post-pandemic recovery	Cytology (Pap)	25-29	28	Not performed	Satisfactory	Positive	LSIL	Low-grade / equivocal squamous	
P08244	2022	2022-Q1	2022-01	Post-pandemic recovery	Cytology (Pap)	35-39	35	Not performed	Satisfactory	Positive	LSIL	Low-grade / equivocal squamous	
P08245	2022	2022-Q1	2022-01	Post-pandemic recovery	Cytology (Pap)	55-59	56	Not performed	Satisfactory	NILM	NILM	Negative (NILM)	
P08246	2022	2022-Q1	2022-01	Post-pandemic recovery	Cytology (Pap)	35-39	39	Not performed	Satisfactory	Positive	ASC-US	Low-grade / equivocal squamous	
P08247	2022	2022-Q1	2022-01	Post-pandemic recovery	Cytology (Pap)	45-49	47	Not performed	Satisfactory	NILM	NILM	Negative (NILM)	
P08248	2022	2022-Q1	2022-01	Post-pandemic recovery	Cytology (Pap)	50-54	51	Not performed	Satisfactory	NILM	NILM	Negative (NILM)	
P08249	2022	2022-Q1	2022-01	Post-pandemic recovery	Cytology (Pap)	45-49	48	Not performed	Satisfactory	Positive	ASC-US	Low-grade / equivocal squamous	
P08250	2022	2022-Q1	2022-01	Post-pandemic recovery	Cytology (Pap)	40-44	40	Not performed	Satisfactory	NILM	NILM	Negative (NILM)	
P08251	2022	2022-Q1	2022-01	Post-pandemic recovery	Cytology (Pap)	45-49	46	Not performed	Satisfactory	NILM	NILM	Negative (NILM)	
P08252	2022	2022-Q1	2022-01	Post-pandemic recovery	Cytology (Pap)	40-44	40	Not performed	Satisfactory	NILM	NILM	Negative (NILM)	
P08253	2022	2022-Q1	2022-01	Post-pandemic recovery	Cytology (Pap)	50-54	53	Not performed	Satisfactory	NILM	NILM	Negative (NILM)	
P08254	2022	2022-Q1	2022-01	Post-pandemic recovery	Cytology (Pap)	40-44	44	Not performed	Satisfactory	NILM	NILM	Negative (NILM)	
P08255	2022	2022-Q1	2022-01	Post-pandemic recovery	Cytology (Pap)	50-54	53	Not performed	Satisfactory	NILM	NILM	Negative (NILM)	
P08256	2022	2022-Q1	2022-01	Post-pandemic recovery	Cytology (Pap)	55-59	59	Not performed	Satisfactory	NILM	NILM	Negative (NILM)	
P08257	2022	2022-Q1	2022-01	Post-pandemic recovery	Cytology (Pap)	60-64	62	Not performed	Satisfactory	NILM	NILM	Negative (NILM)	
P08258	2022	2022-Q1	2022-01	Post-pandemic recovery	Cytology (Pap)	25-29	25	Not performed	Satisfactory	NILM	NILM	Negative (NILM)	
P08259	2022	2022-Q1	2022-01	Post-pandemic recovery	Cytology (Pap)	30-34	30	Not performed	Satisfactory	NILM	NILM	Negative (NILM)	
P08260	2022	2022-Q1	2022-01	Post-pandemic recovery	Cytology (Pap)	50-54	53	Not performed	Satisfactory	NILM	NILM	Negative (NILM)	
P08261	2022	2022-Q1	2022-01	Post-pandemic recovery	Cytology (Pap)	40-44	44	Not performed	Satisfactory	Positive	LSIL	Low-grade / equivocal squamous	
P08262	2022	2022-Q1	2022-01	Post-pandemic recovery	Cytology (Pap)	60-64	60	Not performed	Satisfactory	NILM	NILM	Negative (NILM)	
P08263	2022	2022-Q1	2022-01	Post-pandemic recovery	Cytology (Pap)	30-34	32	Not performed	Satisfactory	NILM	NILM	Negative (NILM)	
P08264	2022	2022-Q1	2022-01	Post-pandemic recovery	Cytology (Pap)	55-59	55	Not performed	Satisfactory	NILM	NILM	Negative (NILM)	
P08265	2022	2022-Q1	2022-01	Post-pandemic recovery	Cytology (Pap)	40-44	41	Not performed	Satisfactory	NILM	NILM	Negative (NILM)	Trichomonas
P08266	2022	2022-Q1	2022-01	Post-pandemic recovery	Cytology (Pap)	60-64	63	Not performed	Satisfactory	NILM	NILM	Negative (NILM)	
P08267	2022	2022-Q1	2022-01	Post-pandemic recovery	Cytology (Pap)	50-54	53	Not performed	Satisfactory	Positive	LSIL	Low-grade / equivocal squamous	
P08268	2022	2022-Q1	2022-01	Post-pandemic recovery	Cytology (Pap)	50-54	50	Not performed	Satisfactory	NILM	NILM	Negative (NILM)	
P08269	2022	2022-Q1	2022-01	Post-pandemic recovery	Cytology (Pap)	55-59	55	Not performed	Satisfactory	NILM	NILM	Negative (NILM)	
P08270	2022	2022-Q1	2022-01	Post-pandemic recovery	Cytology (Pap)	30-34	32	Not performed	Satisfactory	NILM	NILM	Negative (NILM)	Other
P08271	2022	2022-Q1	2022-01	Post-pandemic recovery	Cytology (Pap)	35-39	39	Not performed	Satisfactory	NILM	NILM	Negative (NILM)	
P08272	2022	2022-Q1	2022-01	Post-pandemic recovery	Cytology (Pap)	40-44	44	Not performed	Satisfactory	NILM	NILM	Negative (NILM)	
P08273	2022	2022-Q1	2022-01	Post-pandemic recovery	Cytology (Pap)	45-49	45	Not performed	Satisfactory	NILM	NILM	Negative (NILM)	
P08274	2022	2022-Q1	2022-01	Post-pandemic recovery	Cytology (Pap)	45-49	48	Not performed	Satisfactory	NILM	NILM	Negative (NILM)	
P08275	2022	2022-Q1	2022-01	Post-pandemic recovery	Cytology (Pap)	35-39	37	Not performed	Satisfactory	NILM	NILM	Negative (NILM)	
P08276	2022	2022-Q1	2022-01	Post-pandemic recovery	Cytology (Pap)	45-49	48	Not performed	Satisfactory	NILM	NILM	Negative (NILM)	
P08277	2022	2022-Q1	2022-01	Post-pandemic recovery	Cytology (Pap)	45-49	48	Not performed	Satisfactory	NILM	NILM	Negative (NILM)	
P08278	2022	2022-Q1	2022-01	Post-pandemic recovery	Cytology (Pap)	30-34	34	Not performed	Satisfactory	NILM	NILM	Negative (NILM)	
P08279	2022	2022-Q1	2022-01	Post-pandemic recovery	Cytology (Pap)	60-64	63	Not performed	Satisfactory	NILM	NILM	Negative (NILM)	
P08280	2022	2022-Q1	2022-01	Post-pandemic recovery	Cytology (Pap)	30-34	34	Not performed	Satisfactory	NILM	NILM	Negative (NILM)	
P08281	2022	2022-Q1	2022-01	Post-pandemic recovery	Cytology (Pap)	55-59	56	Not performed	Satisfactory	Positive	LSIL	Low-grade / equivocal squamous	
P08282	2022	2022-Q1	2022-01	Post-pandemic recovery	Cytology (Pap)	55-59	58	Not performed	Satisfactory	NILM	NILM	Negative (NILM)	
P08283	2022	2022-Q1	2022-01	Post-pandemic recovery	Cytology (Pap)	45-49	48	Not performed	Satisfactory	NILM	NILM	Negative (NILM)	
P08284	2022	2022-Q1	2022-01	Post-pandemic recovery	Cytology (Pap)	45-49	46	Not performed	Satisfactory	NILM	NILM	Negative (NILM)	
P08285	2022	2022-Q1	2022-01	Post-pandemic recovery	Cytology (Pap)	40-44	43	Not performed	Satisfactory	NILM	NILM	Negative (NILM)	
P08286	2022	2022-Q1	2022-01	Post-pandemic recovery	Cytology (Pap)	40-44	43	Not performed	Satisfactory	NILM	NILM	Negative (NILM)	
P08287	2022	2022-Q1	2022-01	Post-pandemic recovery	Cytology (Pap)	30-34	30	Not performed	Satisfactory	Positive	LSIL	Low-grade / equivocal squamous	
P08288	2022	2022-Q1	2022-01	Post-pandemic recovery	Cytology (Pap)	40-44	42	Not performed	Satisfactory	Positive	ASC-US	Low-grade / equivocal squamous	
P08289	2022	2022-Q1	2022-01	Post-pandemic recovery	Cytology (Pap)	40-44	40	Not performed	Satisfactory	NILM	NILM	Negative (NILM)	
P08290	2022	2022-Q1	2022-01	Post-pandemic recovery	Cytology (Pap)	40-44	44	Not performed	Unsatisfactory	Unsatisfactory	Unsatisfactory	Unsatisfactory	
P08291	2022	2022-Q1	2022-01	Post-pandemic recovery	Cytology (Pap)	55-59	55	Not performed	Satisfactory	NILM	NILM	Negative (NILM)	
P08292	2022	2022-Q1	2022-01	Post-pandemic recovery	Cytology (Pap)	60-64	64	Not performed	Satisfactory	NILM	NILM	Negative (NILM)	
P08293	2022	2022-Q1	2022-01	Post-pandemic recovery	Cytology (Pap)	30-34	31	Not performed	Satisfactory	NILM	NILM	Negative (NILM)	
P08294	2022	2022-Q1	2022-01	Post-pandemic recovery	Cytology (Pap)	35-39	39	Not performed	Satisfactory	Positive	LSIL	Low-grade / equivocal squamous	
P08295	2022	2022-Q1	2022-01	Post-pandemic recovery	Cytology (Pap)	60-64	63	Not performed	Satisfactory	NILM	NILM	Negative (NILM)	
P08296	2022	2022-Q1	2022-01	Post-pandemic recovery	Cytology (Pap)	30-34	30	Not performed	Satisfactory	NILM	NILM	Negative (NILM)	
P08297	2022	2022-Q1	2022-01	Post-pandemic recovery	Cytology (Pap)	50-54	51	Not performed	Satisfactory	NILM	NILM	Negative (NILM)	
P08298	2022	2022-Q1	2022-01	Post-pandemic recovery	Cytology (Pap)	30-34	33	Not performed	Satisfactory	NILM	NILM	Negative (NILM)	
P08299	2022	2022-Q1	2022-01	Post-pandemic recovery	Cytology (Pap)	25-29	28	Not performed	Satisfactory	NILM	NILM	Negative (NILM)	
P08300	2022	2022-Q1	2022-01	Post-pandemic recovery	Cytology (Pap)	40-44	43	Not performed	Satisfactory	NILM	NILM	Negative (NILM)	
P08301	2022	2022-Q1	2022-01	Post-pandemic recovery	Cytology (Pap)	35-39	37	Not performed	Satisfactory	Positive	ASC-US	Low-grade / equivocal squamous	
P08302	2022	2022-Q1	2022-01	Post-pandemic recovery	Cytology (Pap)	45-49	48	Not performed	Satisfactory	NILM	NILM	Negative (NILM)	
P08303	2022	2022-Q1	2022-01	Post-pandemic recovery	Cytology (Pap)	25-29	29	Not performed	Satisfactory	NILM	NILM	Negative (NILM)	
P08304	2022	2022-Q1	2022-01	Post-pandemic recovery	Cytology (Pap)	35-39	35	Not performed	Satisfactory	NILM	NILM	Negative (NILM)	
P08305	2022	2022-Q1	2022-01	Post-pandemic recovery	Cytology (Pap)	45-49	49	Not performed	Satisfactory	NILM	NILM	Negative (NILM)	
P08306	2022	2022-Q1	2022-01	Post-pandemic recovery	Cytology (Pap)	30-34	31	Not performed	Satisfactory	NILM	NILM	Negative (NILM)	
P08307	2022	2022-Q1	2022-01	Post-pandemic recovery	Cytology (Pap)	35-39	39	Not performed	Satisfactory	NILM	NILM	Negative (NILM)	
P08308	2022	2022-Q1	2022-02	Post-pandemic recovery	Cytology (Pap)	45-49	46	Not performed	Satisfactory	Positive	AGC	Glandular	Candida
P08309	2022	2022-Q1	2022-02	Post-pandemic recovery	Cytology (Pap)	40-44	44	Not performed	Satisfactory	NILM	NILM	Negative (NILM)	
P08310	2022	2022-Q1	2022-02	Post-pandemic recovery	Cytology (Pap)	35-39	38	Not performed	Satisfactory	NILM	NILM	Negative (NILM)	Candida
P08311	2022	2022-Q1	2022-02	Post-pandemic recovery	Cytology (Pap)	35-39	35	Not performed	Satisfactory	NILM	NILM	Negative (NILM)	Candida
P08312	2022	2022-Q1	2022-02	Post-pandemic recovery	Cytology (Pap)	35-39	38	Not performed	Satisfactory	NILM	NILM	Negative (NILM)	
P08313	2022	2022-Q1	2022-02	Post-pandemic recovery	Cytology (Pap)	45-49	47	Not performed	Satisfactory	NILM	NILM	Negative (NILM)	
P08314	2022	2022-Q1	2022-02	Post-pandemic recovery	Cytology (Pap)	35-39	35	Not performed	Satisfactory	NILM	NILM	Negative (NILM)	
P08315	2022	2022-Q1	2022-02	Post-pandemic recovery	Cytology (Pap)	60-64	62	Not performed	Satisfactory	NILM	NILM	Negative (NILM)	
P08316	2022	2022-Q1	2022-02	Post-pandemic recovery	Cytology (Pap)	50-54	50	Not performed	Satisfactory	NILM	NILM	Negative (NILM)	
P08317	2022	2022-Q1	2022-02	Post-pandemic recovery	Cytology (Pap)	50-54	51	Not performed	Satisfactory	NILM	NILM	Negative (NILM)	
P08318	2022	2022-Q1	2022-02	Post-pandemic recovery	Cytology (Pap)	40-44	43	Not performed	Satisfactory	NILM	NILM	Negative (NILM)	

patient_id	year	quarter	month	period	primary_test	age_group	age_years	hpv_result	adequacy	result	bethesda	grade	infection
P08319	2022	2022-Q1	2022-02	Post-pandemic recovery	Cytology (Pap)	35-39	35	Not performed	Satisfactory	NILM	NILM	Negative (NILM)	
P08320	2022	2022-Q1	2022-02	Post-pandemic recovery	Cytology (Pap)	40-44	40	Not performed	Satisfactory	Positive	LSIL	Low-grade / equivocal squamous	
P08321	2022	2022-Q1	2022-02	Post-pandemic recovery	Cytology (Pap)	35-39	38	Not performed	Satisfactory	NILM	NILM	Negative (NILM)	
P08322	2022	2022-Q1	2022-02	Post-pandemic recovery	Cytology (Pap)	30-34	33	Not performed	Satisfactory	NILM	NILM	Negative (NILM)	
P08323	2022	2022-Q1	2022-02	Post-pandemic recovery	Cytology (Pap)	45-49	49	Not performed	Satisfactory	NILM	NILM	Negative (NILM)	Other
P08324	2022	2022-Q1	2022-02	Post-pandemic recovery	Cytology (Pap)	35-39	38	Not performed	Satisfactory	NILM	NILM	Negative (NILM)	
P08325	2022	2022-Q1	2022-02	Post-pandemic recovery	Cytology (Pap)	35-39	35	Not performed	Satisfactory	Positive	ASC-H	High-grade squamous	
P08326	2022	2022-Q1	2022-02	Post-pandemic recovery	Cytology (Pap)	35-39	36	Not performed	Satisfactory	NILM	NILM	Negative (NILM)	
P08327	2022	2022-Q1	2022-02	Post-pandemic recovery	Cytology (Pap)	45-49	49	Not performed	Satisfactory	NILM	NILM	Negative (NILM)	
P08328	2022	2022-Q1	2022-02	Post-pandemic recovery	Cytology (Pap)	50-54	54	Not performed	Satisfactory	NILM	NILM	Negative (NILM)	
P08329	2022	2022-Q1	2022-02	Post-pandemic recovery	Cytology (Pap)	55-59	58	Not performed	Satisfactory	NILM	NILM	Negative (NILM)	
P08330	2022	2022-Q1	2022-02	Post-pandemic recovery	Cytology (Pap)	35-39	39	Not performed	Satisfactory	NILM	NILM	Negative (NILM)	
P08331	2022	2022-Q1	2022-02	Post-pandemic recovery	Cytology (Pap)	60-64	64	Not performed	Satisfactory	NILM	NILM	Negative (NILM)	
P08332	2022	2022-Q1	2022-02	Post-pandemic recovery	Cytology (Pap)	35-39	37	Not performed	Satisfactory	NILM	NILM	Negative (NILM)	
P08333	2022	2022-Q1	2022-02	Post-pandemic recovery	Cytology (Pap)	60-64	60	Not performed	Satisfactory	NILM	NILM	Negative (NILM)	
P08334	2022	2022-Q1	2022-02	Post-pandemic recovery	Cytology (Pap)	45-49	46	Not performed	Satisfactory	NILM	NILM	Negative (NILM)	
P08335	2022	2022-Q1	2022-02	Post-pandemic recovery	Cytology (Pap)	60-64	60	Not performed	Satisfactory	NILM	NILM	Negative (NILM)	
P08336	2022	2022-Q1	2022-02	Post-pandemic recovery	Cytology (Pap)	35-39	36	Not performed	Satisfactory	Positive	LSIL	Low-grade / equivocal squamous	
P08337	2022	2022-Q1	2022-02	Post-pandemic recovery	Cytology (Pap)	35-39	39	Not performed	Satisfactory	Positive	ASC-US	Low-grade / equivocal squamous	
P08338	2022	2022-Q1	2022-02	Post-pandemic recovery	Cytology (Pap)	30-34	32	Not performed	Satisfactory	NILM	NILM	Negative (NILM)	
P08339	2022	2022-Q1	2022-02	Post-pandemic recovery	Cytology (Pap)	55-59	59	Not performed	Satisfactory	NILM	NILM	Negative (NILM)	
P08340	2022	2022-Q1	2022-02	Post-pandemic recovery	Cytology (Pap)	40-44	40	Not performed	Satisfactory	NILM	NILM	Negative (NILM)	
P08341	2022	2022-Q1	2022-02	Post-pandemic recovery	Cytology (Pap)	40-44	44	Not performed	Satisfactory	NILM	NILM	Negative (NILM)	
P08342	2022	2022-Q1	2022-02	Post-pandemic recovery	Cytology (Pap)	35-39	36	Not performed	Satisfactory	Positive	LSIL	Low-grade / equivocal squamous	Gardnerella
P08343	2022	2022-Q1	2022-02	Post-pandemic recovery	Cytology (Pap)	35-39	37	Not performed	Unsatisfactory	Unsatisfactory	Unsatisfactory	Unsatisfactory	
P08344	2022	2022-Q1	2022-02	Post-pandemic recovery	Cytology (Pap)	60-64	62	Not performed	Satisfactory	NILM	NILM	Negative (NILM)	Candida
P08345	2022	2022-Q1	2022-02	Post-pandemic recovery	Cytology (Pap)	50-54	53	Not performed	Satisfactory	NILM	NILM	Negative (NILM)	
P08346	2022	2022-Q1	2022-02	Post-pandemic recovery	Cytology (Pap)	45-49	49	Not performed	Satisfactory	Positive	ASC-US	Low-grade / equivocal squamous	
P08347	2022	2022-Q1	2022-02	Post-pandemic recovery	Cytology (Pap)	40-44	44	Not performed	Satisfactory	NILM	NILM	Negative (NILM)	
P08348	2022	2022-Q1	2022-02	Post-pandemic recovery	Cytology (Pap)	40-44	44	Not performed	Satisfactory	NILM	NILM	Negative (NILM)	
P08349	2022	2022-Q1	2022-02	Post-pandemic recovery	Cytology (Pap)	30-34	34	Not performed	Satisfactory	Positive	ASC-US	Low-grade / equivocal squamous	
P08350	2022	2022-Q1	2022-02	Post-pandemic recovery	Cytology (Pap)	50-54	52	Not performed	Satisfactory	NILM	NILM	Negative (NILM)	
P08351	2022	2022-Q1	2022-02	Post-pandemic recovery	Cytology (Pap)	30-34	33	Not performed	Satisfactory	NILM	NILM	Negative (NILM)	
P08352	2022	2022-Q1	2022-02	Post-pandemic recovery	Cytology (Pap)	40-44	40	Not performed	Satisfactory	NILM	NILM	Negative (NILM)	
P08353	2022	2022-Q1	2022-02	Post-pandemic recovery	Cytology (Pap)	55-59	59	Not performed	Satisfactory	NILM	NILM	Negative (NILM)	
P08354	2022	2022-Q1	2022-02	Post-pandemic recovery	Cytology (Pap)	25-29	26	Not performed	Satisfactory	NILM	NILM	Negative (NILM)	
P08355	2022	2022-Q1	2022-02	Post-pandemic recovery	Cytology (Pap)	35-39	39	Not performed	Unsatisfactory	Unsatisfactory	Unsatisfactory	Unsatisfactory	
P08356	2022	2022-Q1	2022-02	Post-pandemic recovery	Cytology (Pap)	25-29	25	Not performed	Satisfactory	NILM	NILM	Negative (NILM)	Candida
P08357	2022	2022-Q1	2022-02	Post-pandemic recovery	Cytology (Pap)	55-59	55	Not performed	Satisfactory	NILM	NILM	Negative (NILM)	
P08358	2022	2022-Q1	2022-02	Post-pandemic recovery	Cytology (Pap)	45-49	46	Not performed	Satisfactory	NILM	NILM	Negative (NILM)	
P08359	2022	2022-Q1	2022-02	Post-pandemic recovery	Cytology (Pap)	60-64	60	Not performed	Satisfactory	NILM	NILM	Negative (NILM)	
P08360	2022	2022-Q1	2022-02	Post-pandemic recovery	Cytology (Pap)	55-59	56	Not performed	Satisfactory	NILM	NILM	Negative (NILM)	
P08361	2022	2022-Q1	2022-02	Post-pandemic recovery	Cytology (Pap)	55-59	58	Not performed	Satisfactory	NILM	NILM	Negative (NILM)	
P08362	2022	2022-Q1	2022-02	Post-pandemic recovery	Cytology (Pap)	30-34	33	Not performed	Satisfactory	NILM	NILM	Negative (NILM)	
P08363	2022	2022-Q1	2022-02	Post-pandemic recovery	Cytology (Pap)	40-44	44	Not performed	Satisfactory	Positive	LSIL	Low-grade / equivocal squamous	
P08364	2022	2022-Q1	2022-02	Post-pandemic recovery	Cytology (Pap)	35-39	36	Not performed	Unsatisfactory	Unsatisfactory	Unsatisfactory	Unsatisfactory	
P08365	2022	2022-Q1	2022-02	Post-pandemic recovery	Cytology (Pap)	35-39	39	Not performed	Satisfactory	NILM	NILM	Negative (NILM)	
P08366	2022	2022-Q1	2022-02	Post-pandemic recovery	Cytology (Pap)	30-34	34	Not performed	Satisfactory	NILM	NILM	Negative (NILM)	
P08367	2022	2022-Q1	2022-02	Post-pandemic recovery	Cytology (Pap)	30-34	30	Not performed	Satisfactory	NILM	NILM	Negative (NILM)	
P08368	2022	2022-Q1	2022-02	Post-pandemic recovery	Cytology (Pap)	30-34	34	Not performed	Satisfactory	NILM	NILM	Negative (NILM)	
P08369	2022	2022-Q1	2022-02	Post-pandemic recovery	Cytology (Pap)	30-34	34	Not performed	Satisfactory	NILM	NILM	Negative (NILM)	
P08370	2022	2022-Q1	2022-02	Post-pandemic recovery	Cytology (Pap)	40-44	41	Not performed	Satisfactory	NILM	NILM	Negative (NILM)	
P08371	2022	2022-Q1	2022-02	Post-pandemic recovery	Cytology (Pap)	60-64	60	Not performed	Satisfactory	NILM	NILM	Negative (NILM)	
P08372	2022	2022-Q1	2022-02	Post-pandemic recovery	Cytology (Pap)	35-39	37	Not performed	Satisfactory	NILM	NILM	Negative (NILM)	
P08373	2022	2022-Q1	2022-02	Post-pandemic recovery	Cytology (Pap)	25-29	26	Not performed	Satisfactory	Positive	ASC-H	High-grade squamous	
P08374	2022	2022-Q1	2022-02	Post-pandemic recovery	Cytology (Pap)	50-54	53	Not performed	Satisfactory	NILM	NILM	Negative (NILM)	Candida
P08375	2022	2022-Q1	2022-02	Post-pandemic recovery	Cytology (Pap)	30-34	33	Not performed	Satisfactory	NILM	NILM	Negative (NILM)	
P08376	2022	2022-Q1	2022-02	Post-pandemic recovery	Cytology (Pap)	60-64	61	Not performed	Satisfactory	NILM	NILM	Negative (NILM)	
P08377	2022	2022-Q1	2022-02	Post-pandemic recovery	Cytology (Pap)	30-34	30	Not performed	Satisfactory	NILM	NILM	Negative (NILM)	
P08378	2022	2022-Q1	2022-02	Post-pandemic recovery	Cytology (Pap)	25-29	27	Not performed	Satisfactory	NILM	NILM	Negative (NILM)	
P08379	2022	2022-Q1	2022-02	Post-pandemic recovery	Cytology (Pap)	30-34	30	Not performed	Satisfactory	Positive	HSIL	High-grade squamous	
P08380	2022	2022-Q1	2022-02	Post-pandemic recovery	Cytology (Pap)	45-49	46	Not performed	Satisfactory	NILM	NILM	Negative (NILM)	
P08381	2022	2022-Q1	2022-02	Post-pandemic recovery	Cytology (Pap)	50-54	54	Not performed	Satisfactory	NILM	NILM	Negative (NILM)	
P08382	2022	2022-Q1	2022-02	Post-pandemic recovery	Cytology (Pap)	50-54	50	Not performed	Satisfactory	NILM	NILM	Negative (NILM)	
P08383	2022	2022-Q1	2022-02	Post-pandemic recovery	Cytology (Pap)	50-54	54	Not performed	Satisfactory	NILM	NILM	Negative (NILM)	
P08384	2022	2022-Q1	2022-02	Post-pandemic recovery	Cytology (Pap)	40-44	42	Not performed	Satisfactory	NILM	NILM	Negative (NILM)	
P08385	2022	2022-Q1	2022-02	Post-pandemic recovery	Cytology (Pap)	30-34	30	Not performed	Satisfactory	NILM	NILM	Negative (NILM)	
P08386	2022	2022-Q1	2022-02	Post-pandemic recovery	Cytology (Pap)	35-39	38	Not performed	Satisfactory	Positive	HSIL	High-grade squamous	
P08387	2022	2022-Q1	2022-02	Post-pandemic recovery	Cytology (Pap)	50-54	50	Not performed	Satisfactory	NILM	NILM	Negative (NILM)	
P08388	2022	2022-Q1	2022-02	Post-pandemic recovery	Cytology (Pap)	50-54	50	Not performed	Satisfactory	NILM	NILM	Negative (NILM)	
P08389	2022	2022-Q1	2022-02	Post-pandemic recovery	Cytology (Pap)	45-49	46	Not performed	Satisfactory	Positive	ASC-US	Low-grade / equivocal squamous	
P08390	2022	2022-Q1	2022-02	Post-pandemic recovery	Cytology (Pap)	40-44	40	Not performed	Satisfactory	NILM	NILM	Negative (NILM)	
P08391	2022	2022-Q1	2022-02	Post-pandemic recovery	Cytology (Pap)	30-34	34	Not performed	Satisfactory	NILM	NILM	Negative (NILM)	
P08392	2022	2022-Q1	2022-02	Post-pandemic recovery	Cytology (Pap)	25-29	29	Not performed	Satisfactory	NILM	NILM	Negative (NILM)	
P08393	2022	2022-Q1	2022-02	Post-pandemic recovery	Cytology (Pap)	50-54	50	Not performed	Satisfactory	NILM	NILM	Negative (NILM)	
P08394	2022	2022-Q1	2022-02	Post-pandemic recovery	Cytology (Pap)	45-49	45	Not performed	Satisfactory	NILM	NILM	Negative (NILM)	
P08395	2022	2022-Q1	2022-02	Post-pandemic recovery	Cytology (Pap)	25-29	26	Not performed	Satisfactory	NILM	NILM	Negative (NILM)	
P08396	2022	2022-Q1	2022-02	Post-pandemic recovery	Cytology (Pap)	50-54	53	Not performed	Satisfactory	NILM	NILM	Negative (NILM)	
P08397	2022	2022-Q1	2022-02	Post-pandemic recovery	Cytology (Pap)	45-49	48	Not performed	Satisfactory	NILM	NILM	Negative (NILM)	
P08398	2022	2022-Q1	2022-02	Post-pandemic recovery	Cytology (Pap)	40-44	44	Not performed	Satisfactory	NILM	NILM	Negative (NILM)	

patient_id	year	quarter	month	period	primary_test	age_group	age_years	hpv_result	adequacy	result	bethesda	grade	infection
P08399	2022	2022-Q1	2022-02	Post-pandemic recovery	Cytology (Pap)	30-34	30	Not performed	Satisfactory	NILM	NILM	Negative (NILM)	
P08400	2022	2022-Q1	2022-02	Post-pandemic recovery	Cytology (Pap)	45-49	47	Not performed	Satisfactory	NILM	NILM	Negative (NILM)	
P08401	2022	2022-Q1	2022-02	Post-pandemic recovery	Cytology (Pap)	40-44	42	Not performed	Satisfactory	Positive	ASC-US	Low-grade / equivocal squamous	
P08402	2022	2022-Q1	2022-02	Post-pandemic recovery	Cytology (Pap)	35-39	37	Not performed	Satisfactory	NILM	NILM	Negative (NILM)	
P08403	2022	2022-Q1	2022-02	Post-pandemic recovery	Cytology (Pap)	45-49	49	Not performed	Satisfactory	NILM	NILM	Negative (NILM)	
P08404	2022	2022-Q1	2022-02	Post-pandemic recovery	Cytology (Pap)	45-49	45	Not performed	Satisfactory	Positive	HSIL	High-grade squamous	Candida
P08405	2022	2022-Q1	2022-02	Post-pandemic recovery	Cytology (Pap)	40-44	40	Not performed	Satisfactory	NILM	NILM	Negative (NILM)	
P08406	2022	2022-Q1	2022-02	Post-pandemic recovery	Cytology (Pap)	50-54	52	Not performed	Satisfactory	Positive	HSIL	High-grade squamous	
P08407	2022	2022-Q1	2022-02	Post-pandemic recovery	Cytology (Pap)	60-64	60	Not performed	Satisfactory	Positive	ASC-US	Low-grade / equivocal squamous	
P08408	2022	2022-Q1	2022-03	Post-pandemic recovery	Cytology (Pap)	25-29	26	Not performed	Satisfactory	NILM	NILM	Negative (NILM)	
P08409	2022	2022-Q1	2022-03	Post-pandemic recovery	Cytology (Pap)	30-34	31	Not performed	Satisfactory	NILM	NILM	Negative (NILM)	
P08410	2022	2022-Q1	2022-03	Post-pandemic recovery	Cytology (Pap)	30-34	32	Not performed	Satisfactory	NILM	NILM	Negative (NILM)	
P08411	2022	2022-Q1	2022-03	Post-pandemic recovery	Cytology (Pap)	40-44	40	Not performed	Satisfactory	NILM	NILM	Negative (NILM)	
P08412	2022	2022-Q1	2022-03	Post-pandemic recovery	Cytology (Pap)	40-44	41	Not performed	Satisfactory	NILM	NILM	Negative (NILM)	Trichomonas
P08413	2022	2022-Q1	2022-03	Post-pandemic recovery	Cytology (Pap)	30-34	33	Not performed	Satisfactory	NILM	NILM	Negative (NILM)	
P08414	2022	2022-Q1	2022-03	Post-pandemic recovery	Cytology (Pap)	40-44	41	Not performed	Satisfactory	NILM	NILM	Negative (NILM)	
P08415	2022	2022-Q1	2022-03	Post-pandemic recovery	Cytology (Pap)	40-44	41	Not performed	Satisfactory	NILM	NILM	Negative (NILM)	
P08416	2022	2022-Q1	2022-03	Post-pandemic recovery	Cytology (Pap)	40-44	43	Not performed	Satisfactory	NILM	NILM	Negative (NILM)	
P08417	2022	2022-Q1	2022-03	Post-pandemic recovery	Cytology (Pap)	40-44	44	Not performed	Satisfactory	NILM	NILM	Negative (NILM)	
P08418	2022	2022-Q1	2022-03	Post-pandemic recovery	Cytology (Pap)	55-59	55	Not performed	Satisfactory	NILM	NILM	Negative (NILM)	
P08419	2022	2022-Q1	2022-03	Post-pandemic recovery	Cytology (Pap)	50-54	54	Not performed	Satisfactory	NILM	NILM	Negative (NILM)	
P08420	2022	2022-Q1	2022-03	Post-pandemic recovery	Cytology (Pap)	45-49	47	Not performed	Satisfactory	NILM	NILM	Negative (NILM)	
P08421	2022	2022-Q1	2022-03	Post-pandemic recovery	Cytology (Pap)	60-64	62	Not performed	Satisfactory	NILM	NILM	Negative (NILM)	
P08422	2022	2022-Q1	2022-03	Post-pandemic recovery	Cytology (Pap)	40-44	44	Not performed	Unsatisfactory	Unsatisfactory	Unsatisfactory	Unsatisfactory	Trichomonas
P08423	2022	2022-Q1	2022-03	Post-pandemic recovery	Cytology (Pap)	25-29	27	Not performed	Satisfactory	Positive	ASC-US	Low-grade / equivocal squamous	
P08424	2022	2022-Q1	2022-03	Post-pandemic recovery	Cytology (Pap)	40-44	42	Not performed	Satisfactory	NILM	NILM	Negative (NILM)	
P08425	2022	2022-Q1	2022-03	Post-pandemic recovery	Cytology (Pap)	55-59	56	Not performed	Satisfactory	NILM	NILM	Negative (NILM)	
P08426	2022	2022-Q1	2022-03	Post-pandemic recovery	Cytology (Pap)	50-54	50	Not performed	Satisfactory	Positive	ASC-US	Low-grade / equivocal squamous	
P08427	2022	2022-Q1	2022-03	Post-pandemic recovery	Cytology (Pap)	45-49	47	Not performed	Satisfactory	NILM	NILM	Negative (NILM)	
P08428	2022	2022-Q1	2022-03	Post-pandemic recovery	Cytology (Pap)	55-59	58	Not performed	Unsatisfactory	Unsatisfactory	Unsatisfactory	Unsatisfactory	
P08429	2022	2022-Q1	2022-03	Post-pandemic recovery	Cytology (Pap)	40-44	42	Not performed	Satisfactory	NILM	NILM	Negative (NILM)	
P08430	2022	2022-Q1	2022-03	Post-pandemic recovery	Cytology (Pap)	40-44	42	Not performed	Satisfactory	NILM	NILM	Negative (NILM)	
P08431	2022	2022-Q1	2022-03	Post-pandemic recovery	Cytology (Pap)	30-34	30	Not performed	Satisfactory	Positive	ASC-US	Low-grade / equivocal squamous	
P08432	2022	2022-Q1	2022-03	Post-pandemic recovery	Cytology (Pap)	45-49	46	Not performed	Satisfactory	NILM	NILM	Negative (NILM)	
P08433	2022	2022-Q1	2022-03	Post-pandemic recovery	Cytology (Pap)	45-49	45	Not performed	Satisfactory	NILM	NILM	Negative (NILM)	
P08434	2022	2022-Q1	2022-03	Post-pandemic recovery	Cytology (Pap)	45-49	45	Not performed	Satisfactory	NILM	NILM	Negative (NILM)	
P08435	2022	2022-Q1	2022-03	Post-pandemic recovery	Cytology (Pap)	40-44	42	Not performed	Satisfactory	NILM	NILM	Negative (NILM)	
P08436	2022	2022-Q1	2022-03	Post-pandemic recovery	Cytology (Pap)	55-59	56	Not performed	Satisfactory	NILM	NILM	Negative (NILM)	
P08437	2022	2022-Q1	2022-03	Post-pandemic recovery	Cytology (Pap)	25-29	27	Not performed	Satisfactory	Positive	ASC-H	High-grade squamous	
P08438	2022	2022-Q1	2022-03	Post-pandemic recovery	Cytology (Pap)	35-39	38	Not performed	Satisfactory	NILM	NILM	Negative (NILM)	
P08439	2022	2022-Q1	2022-03	Post-pandemic recovery	Cytology (Pap)	50-54	51	Not performed	Unsatisfactory	Unsatisfactory	Unsatisfactory	Unsatisfactory	Gardnerella
P08440	2022	2022-Q1	2022-03	Post-pandemic recovery	Cytology (Pap)	50-54	54	Not performed	Satisfactory	NILM	NILM	Negative (NILM)	
P08441	2022	2022-Q1	2022-03	Post-pandemic recovery	Cytology (Pap)	25-29	28	Not performed	Satisfactory	Positive	LSIL	Low-grade / equivocal squamous	
P08442	2022	2022-Q1	2022-03	Post-pandemic recovery	Cytology (Pap)	50-54	53	Not performed	Satisfactory	NILM	NILM	Negative (NILM)	
P08443	2022	2022-Q1	2022-03	Post-pandemic recovery	Cytology (Pap)	45-49	46	Not performed	Satisfactory	NILM	NILM	Negative (NILM)	
P08444	2022	2022-Q1	2022-03	Post-pandemic recovery	Cytology (Pap)	55-59	59	Not performed	Satisfactory	NILM	NILM	Negative (NILM)	
P08445	2022	2022-Q1	2022-03	Post-pandemic recovery	Cytology (Pap)	40-44	41	Not performed	Unsatisfactory	Unsatisfactory	Unsatisfactory	Unsatisfactory	
P08446	2022	2022-Q1	2022-03	Post-pandemic recovery	Cytology (Pap)	25-29	27	Not performed	Satisfactory	NILM	NILM	Negative (NILM)	
P08447	2022	2022-Q1	2022-03	Post-pandemic recovery	Cytology (Pap)	40-44	43	Not performed	Satisfactory	NILM	NILM	Negative (NILM)	
P08448	2022	2022-Q1	2022-03	Post-pandemic recovery	Cytology (Pap)	60-64	61	Not performed	Satisfactory	NILM	NILM	Negative (NILM)	
P08449	2022	2022-Q1	2022-03	Post-pandemic recovery	Cytology (Pap)	50-54	50	Not performed	Satisfactory	NILM	NILM	Negative (NILM)	
P08450	2022	2022-Q1	2022-03	Post-pandemic recovery	Cytology (Pap)	30-34	32	Not performed	Satisfactory	NILM	NILM	Negative (NILM)	
P08451	2022	2022-Q1	2022-03	Post-pandemic recovery	Cytology (Pap)	35-39	36	Not performed	Satisfactory	NILM	NILM	Negative (NILM)	
P08452	2022	2022-Q1	2022-03	Post-pandemic recovery	Cytology (Pap)	40-44	42	Not performed	Satisfactory	NILM	NILM	Negative (NILM)	
P08453	2022	2022-Q1	2022-03	Post-pandemic recovery	Cytology (Pap)	55-59	59	Not performed	Satisfactory	Positive	AGC	Glandular	
P08454	2022	2022-Q1	2022-03	Post-pandemic recovery	Cytology (Pap)	30-34	31	Not performed	Satisfactory	NILM	NILM	Negative (NILM)	
P08455	2022	2022-Q1	2022-03	Post-pandemic recovery	Cytology (Pap)	55-59	59	Not performed	Satisfactory	NILM	NILM	Negative (NILM)	
P08456	2022	2022-Q1	2022-03	Post-pandemic recovery	Cytology (Pap)	45-49	47	Not performed	Satisfactory	NILM	NILM	Negative (NILM)	
P08457	2022	2022-Q1	2022-03	Post-pandemic recovery	Cytology (Pap)	40-44	41	Not performed	Satisfactory	NILM	NILM	Negative (NILM)	
P08458	2022	2022-Q1	2022-03	Post-pandemic recovery	Cytology (Pap)	35-39	39	Not performed	Satisfactory	NILM	NILM	Negative (NILM)	
P08459	2022	2022-Q1	2022-03	Post-pandemic recovery	Cytology (Pap)	40-44	42	Not performed	Satisfactory	NILM	NILM	Negative (NILM)	
P08460	2022	2022-Q1	2022-03	Post-pandemic recovery	Cytology (Pap)	50-54	54	Not performed	Satisfactory	NILM	NILM	Negative (NILM)	
P08461	2022	2022-Q1	2022-03	Post-pandemic recovery	Cytology (Pap)	35-39	36	Not performed	Satisfactory	Positive	LSIL	Low-grade / equivocal squamous	
P08462	2022	2022-Q1	2022-03	Post-pandemic recovery	Cytology (Pap)	30-34	32	Not performed	Satisfactory	NILM	NILM	Negative (NILM)	
P08463	2022	2022-Q1	2022-03	Post-pandemic recovery	Cytology (Pap)	25-29	25	Not performed	Satisfactory	NILM	NILM	Negative (NILM)	
P08464	2022	2022-Q1	2022-03	Post-pandemic recovery	Cytology (Pap)	35-39	37	Not performed	Satisfactory	Positive	ASC-US	Low-grade / equivocal squamous	
P08465	2022	2022-Q1	2022-03	Post-pandemic recovery	Cytology (Pap)	60-64	61	Not performed	Satisfactory	NILM	NILM	Negative (NILM)	Other
P08466	2022	2022-Q1	2022-03	Post-pandemic recovery	Cytology (Pap)	40-44	43	Not performed	Satisfactory	NILM	NILM	Negative (NILM)	
P08467	2022	2022-Q1	2022-03	Post-pandemic recovery	Cytology (Pap)	25-29	25	Not performed	Satisfactory	NILM	NILM	Negative (NILM)	
P08468	2022	2022-Q1	2022-03	Post-pandemic recovery	Cytology (Pap)	30-34	31	Not performed	Satisfactory	Positive	AGC	Glandular	
P08469	2022	2022-Q1	2022-03	Post-pandemic recovery	Cytology (Pap)	45-49	47	Not performed	Satisfactory	NILM	NILM	Negative (NILM)	
P08470	2022	2022-Q1	2022-03	Post-pandemic recovery	Cytology (Pap)	45-49	46	Not performed	Satisfactory	NILM	NILM	Negative (NILM)	
P08471	2022	2022-Q1	2022-03	Post-pandemic recovery	Cytology (Pap)	55-59	56	Not performed	Satisfactory	NILM	NILM	Negative (NILM)	
P08472	2022	2022-Q1	2022-03	Post-pandemic recovery	Cytology (Pap)	25-29	27	Not performed	Satisfactory	NILM	NILM	Negative (NILM)	
P08473	2022	2022-Q1	2022-03	Post-pandemic recovery	Cytology (Pap)	35-39	35	Not performed	Satisfactory	NILM	NILM	Negative (NILM)	
P08474	2022	2022-Q1	2022-03	Post-pandemic recovery	Cytology (Pap)	25-29	26	Not performed	Satisfactory	NILM	NILM	Negative (NILM)	
P08475	2022	2022-Q1	2022-03	Post-pandemic recovery	Cytology (Pap)	55-59	56	Not performed	Satisfactory	NILM	NILM	Negative (NILM)	
P08476	2022	2022-Q1	2022-03	Post-pandemic recovery	Cytology (Pap)	50-54	53	Not performed	Satisfactory	NILM	NILM	Negative (NILM)	Trichomonas
P08477	2022	2022-Q1	2022-03	Post-pandemic recovery	Cytology (Pap)	35-39	36	Not performed	Satisfactory	NILM	NILM	Negative (NILM)	
P08478	2022	2022-Q1	2022-03	Post-pandemic recovery	Cytology (Pap)	30-34	34	Not performed	Satisfactory	NILM	NILM	Negative (NILM)	

patient_id	year	quarter	month	period	primary_test	age_group	age_years	hpv_result	adequacy	result	bethesda	grade	infection
P08479	2022	2022-Q1	2022-03	Post-pandemic recovery	Cytology (Pap)	30-34	33	Not performed	Satisfactory	NILM	NILM	Negative (NILM)	
P08480	2022	2022-Q1	2022-03	Post-pandemic recovery	Cytology (Pap)	40-44	40	Not performed	Satisfactory	NILM	NILM	Negative (NILM)	
P08481	2022	2022-Q1	2022-03	Post-pandemic recovery	Cytology (Pap)	60-64	63	Not performed	Satisfactory	NILM	NILM	Negative (NILM)	
P08482	2022	2022-Q1	2022-03	Post-pandemic recovery	Cytology (Pap)	40-44	40	Not performed	Satisfactory	NILM	NILM	Negative (NILM)	Candida
P08483	2022	2022-Q1	2022-03	Post-pandemic recovery	Cytology (Pap)	45-49	48	Not performed	Satisfactory	NILM	NILM	Negative (NILM)	Trichomonas
P08484	2022	2022-Q1	2022-03	Post-pandemic recovery	Cytology (Pap)	55-59	56	Not performed	Satisfactory	NILM	NILM	Negative (NILM)	
P08485	2022	2022-Q1	2022-03	Post-pandemic recovery	Cytology (Pap)	50-54	50	Not performed	Satisfactory	NILM	NILM	Negative (NILM)	
P08486	2022	2022-Q1	2022-03	Post-pandemic recovery	Cytology (Pap)	25-29	26	Not performed	Satisfactory	NILM	NILM	Negative (NILM)	
P08487	2022	2022-Q1	2022-03	Post-pandemic recovery	Cytology (Pap)	50-54	54	Not performed	Satisfactory	Positive	ASC-US	Low-grade / equivocal squamous	
P08488	2022	2022-Q1	2022-03	Post-pandemic recovery	Cytology (Pap)	45-49	49	Not performed	Satisfactory	NILM	NILM	Negative (NILM)	
P08489	2022	2022-Q1	2022-03	Post-pandemic recovery	Cytology (Pap)	30-34	30	Not performed	Satisfactory	NILM	NILM	Negative (NILM)	
P08490	2022	2022-Q1	2022-03	Post-pandemic recovery	Cytology (Pap)	60-64	63	Not performed	Satisfactory	NILM	NILM	Negative (NILM)	
P08491	2022	2022-Q1	2022-03	Post-pandemic recovery	Cytology (Pap)	45-49	45	Not performed	Satisfactory	NILM	NILM	Negative (NILM)	
P08492	2022	2022-Q1	2022-03	Post-pandemic recovery	Cytology (Pap)	60-64	63	Not performed	Satisfactory	Positive	HSIL	High-grade squamous	
P08493	2022	2022-Q1	2022-03	Post-pandemic recovery	Cytology (Pap)	35-39	38	Not performed	Satisfactory	Positive	ASC-H	High-grade squamous	
P08494	2022	2022-Q1	2022-03	Post-pandemic recovery	Cytology (Pap)	35-39	39	Not performed	Satisfactory	NILM	NILM	Negative (NILM)	
P08495	2022	2022-Q1	2022-03	Post-pandemic recovery	Cytology (Pap)	40-44	43	Not performed	Satisfactory	NILM	NILM	Negative (NILM)	
P08496	2022	2022-Q1	2022-03	Post-pandemic recovery	Cytology (Pap)	35-39	39	Not performed	Satisfactory	NILM	NILM	Negative (NILM)	
P08497	2022	2022-Q1	2022-03	Post-pandemic recovery	Cytology (Pap)	55-59	57	Not performed	Satisfactory	Positive	ASC-US	Low-grade / equivocal squamous	
P08498	2022	2022-Q1	2022-03	Post-pandemic recovery	Cytology (Pap)	35-39	35	Not performed	Satisfactory	NILM	NILM	Negative (NILM)	
P08499	2022	2022-Q1	2022-03	Post-pandemic recovery	Cytology (Pap)	40-44	40	Not performed	Satisfactory	NILM	NILM	Negative (NILM)	
P08500	2022	2022-Q1	2022-03	Post-pandemic recovery	Cytology (Pap)	45-49	45	Not performed	Satisfactory	NILM	NILM	Negative (NILM)	Gardnerella
P08501	2022	2022-Q1	2022-03	Post-pandemic recovery	Cytology (Pap)	45-49	46	Not performed	Satisfactory	NILM	NILM	Negative (NILM)	
P08502	2022	2022-Q1	2022-03	Post-pandemic recovery	Cytology (Pap)	30-34	31	Not performed	Satisfactory	NILM	NILM	Negative (NILM)	
P08503	2022	2022-Q1	2022-03	Post-pandemic recovery	Cytology (Pap)	40-44	43	Not performed	Satisfactory	NILM	NILM	Negative (NILM)	
P08504	2022	2022-Q1	2022-03	Post-pandemic recovery	Cytology (Pap)	50-54	53	Not performed	Satisfactory	NILM	NILM	Negative (NILM)	
P08505	2022	2022-Q1	2022-03	Post-pandemic recovery	Cytology (Pap)	50-54	51	Not performed	Satisfactory	NILM	NILM	Negative (NILM)	
P08506	2022	2022-Q1	2022-03	Post-pandemic recovery	Cytology (Pap)	40-44	44	Not performed	Satisfactory	NILM	NILM	Negative (NILM)	
P08507	2022	2022-Q1	2022-03	Post-pandemic recovery	Cytology (Pap)	30-34	33	Not performed	Satisfactory	NILM	NILM	Negative (NILM)	
P08508	2022	2022-Q2	2022-04	Post-pandemic recovery	Cytology (Pap)	55-59	56	Not performed	Satisfactory	NILM	NILM	Negative (NILM)	
P08509	2022	2022-Q2	2022-04	Post-pandemic recovery	Cytology (Pap)	25-29	28	Not performed	Satisfactory	NILM	NILM	Negative (NILM)	
P08510	2022	2022-Q2	2022-04	Post-pandemic recovery	Cytology (Pap)	35-39	38	Not performed	Satisfactory	Positive	ASC-US	Low-grade / equivocal squamous	
P08511	2022	2022-Q2	2022-04	Post-pandemic recovery	Cytology (Pap)	50-54	51	Not performed	Satisfactory	NILM	NILM	Negative (NILM)	
P08512	2022	2022-Q2	2022-04	Post-pandemic recovery	Cytology (Pap)	30-34	34	Not performed	Satisfactory	NILM	NILM	Negative (NILM)	
P08513	2022	2022-Q2	2022-04	Post-pandemic recovery	Cytology (Pap)	45-49	49	Not performed	Satisfactory	NILM	NILM	Negative (NILM)	
P08514	2022	2022-Q2	2022-04	Post-pandemic recovery	Cytology (Pap)	50-54	54	Not performed	Satisfactory	NILM	NILM	Negative (NILM)	
P08515	2022	2022-Q2	2022-04	Post-pandemic recovery	Cytology (Pap)	50-54	52	Not performed	Satisfactory	NILM	NILM	Negative (NILM)	
P08516	2022	2022-Q2	2022-04	Post-pandemic recovery	Cytology (Pap)	30-34	30	Not performed	Satisfactory	NILM	NILM	Negative (NILM)	
P08517	2022	2022-Q2	2022-04	Post-pandemic recovery	Cytology (Pap)	45-49	47	Not performed	Satisfactory	NILM	NILM	Negative (NILM)	
P08518	2022	2022-Q2	2022-04	Post-pandemic recovery	Cytology (Pap)	40-44	42	Not performed	Satisfactory	NILM	NILM	Negative (NILM)	
P08519	2022	2022-Q2	2022-04	Post-pandemic recovery	Cytology (Pap)	45-49	49	Not performed	Satisfactory	NILM	NILM	Negative (NILM)	
P08520	2022	2022-Q2	2022-04	Post-pandemic recovery	Cytology (Pap)	50-54	51	Not performed	Satisfactory	Positive	ASC-US	Low-grade / equivocal squamous	
P08521	2022	2022-Q2	2022-04	Post-pandemic recovery	Cytology (Pap)	50-54	51	Not performed	Unsatisfactory	Unsatisfactory	Unsatisfactory	Unsatisfactory	
P08522	2022	2022-Q2	2022-04	Post-pandemic recovery	Cytology (Pap)	35-39	36	Not performed	Satisfactory	NILM	NILM	Negative (NILM)	
P08523	2022	2022-Q2	2022-04	Post-pandemic recovery	Cytology (Pap)	55-59	58	Not performed	Satisfactory	NILM	NILM	Negative (NILM)	
P08524	2022	2022-Q2	2022-04	Post-pandemic recovery	Cytology (Pap)	60-64	62	Not performed	Satisfactory	NILM	NILM	Negative (NILM)	
P08525	2022	2022-Q2	2022-04	Post-pandemic recovery	Cytology (Pap)	30-34	34	Not performed	Satisfactory	NILM	NILM	Negative (NILM)	Candida
P08526	2022	2022-Q2	2022-04	Post-pandemic recovery	Cytology (Pap)	35-39	36	Not performed	Satisfactory	NILM	NILM	Negative (NILM)	
P08527	2022	2022-Q2	2022-04	Post-pandemic recovery	Cytology (Pap)	40-44	44	Not performed	Unsatisfactory	Unsatisfactory	Unsatisfactory	Unsatisfactory	
P08528	2022	2022-Q2	2022-04	Post-pandemic recovery	Cytology (Pap)	35-39	35	Not performed	Satisfactory	NILM	NILM	Negative (NILM)	
P08529	2022	2022-Q2	2022-04	Post-pandemic recovery	Cytology (Pap)	30-34	31	Not performed	Satisfactory	NILM	NILM	Negative (NILM)	Candida
P08530	2022	2022-Q2	2022-04	Post-pandemic recovery	Cytology (Pap)	45-49	49	Not performed	Satisfactory	NILM	NILM	Negative (NILM)	
P08531	2022	2022-Q2	2022-04	Post-pandemic recovery	Cytology (Pap)	45-49	45	Not performed	Satisfactory	NILM	NILM	Negative (NILM)	
P08532	2022	2022-Q2	2022-04	Post-pandemic recovery	Cytology (Pap)	55-59	57	Not performed	Satisfactory	NILM	NILM	Negative (NILM)	
P08533	2022	2022-Q2	2022-04	Post-pandemic recovery	Cytology (Pap)	50-54	52	Not performed	Satisfactory	NILM	NILM	Negative (NILM)	
P08534	2022	2022-Q2	2022-04	Post-pandemic recovery	Cytology (Pap)	45-49	46	Not performed	Satisfactory	NILM	NILM	Negative (NILM)	
P08535	2022	2022-Q2	2022-04	Post-pandemic recovery	Cytology (Pap)	35-39	38	Not performed	Satisfactory	Positive	LSIL	Low-grade / equivocal squamous	
P08536	2022	2022-Q2	2022-04	Post-pandemic recovery	Cytology (Pap)	30-34	34	Not performed	Satisfactory	NILM	NILM	Negative (NILM)	
P08537	2022	2022-Q2	2022-04	Post-pandemic recovery	Cytology (Pap)	35-39	36	Not performed	Satisfactory	NILM	NILM	Negative (NILM)	
P08538	2022	2022-Q2	2022-04	Post-pandemic recovery	Cytology (Pap)	30-34	34	Not performed	Satisfactory	NILM	NILM	Negative (NILM)	
P08539	2022	2022-Q2	2022-04	Post-pandemic recovery	Cytology (Pap)	30-34	33	Not performed	Satisfactory	NILM	NILM	Negative (NILM)	
P08540	2022	2022-Q2	2022-04	Post-pandemic recovery	Cytology (Pap)	55-59	58	Not performed	Satisfactory	NILM	NILM	Negative (NILM)	
P08541	2022	2022-Q2	2022-04	Post-pandemic recovery	Cytology (Pap)	40-44	41	Not performed	Satisfactory	Positive	ASC-US	Low-grade / equivocal squamous	
P08542	2022	2022-Q2	2022-04	Post-pandemic recovery	Cytology (Pap)	25-29	27	Not performed	Satisfactory	NILM	NILM	Negative (NILM)	
P08543	2022	2022-Q2	2022-04	Post-pandemic recovery	Cytology (Pap)	45-49	49	Not performed	Satisfactory	NILM	NILM	Negative (NILM)	
P08544	2022	2022-Q2	2022-04	Post-pandemic recovery	Cytology (Pap)	45-49	49	Not performed	Satisfactory	NILM	NILM	Negative (NILM)	
P08545	2022	2022-Q2	2022-04	Post-pandemic recovery	Cytology (Pap)	35-39	36	Not performed	Satisfactory	NILM	NILM	Negative (NILM)	
P08546	2022	2022-Q2	2022-04	Post-pandemic recovery	Cytology (Pap)	25-29	28	Not performed	Satisfactory	NILM	NILM	Negative (NILM)	
P08547	2022	2022-Q2	2022-04	Post-pandemic recovery	Cytology (Pap)	35-39	39	Not performed	Satisfactory	NILM	NILM	Negative (NILM)	
P08548	2022	2022-Q2	2022-04	Post-pandemic recovery	Cytology (Pap)	55-59	58	Not performed	Satisfactory	NILM	NILM	Negative (NILM)	Candida
P08549	2022	2022-Q2	2022-04	Post-pandemic recovery	Cytology (Pap)	45-49	46	Not performed	Satisfactory	NILM	NILM	Negative (NILM)	
P08550	2022	2022-Q2	2022-04	Post-pandemic recovery	Cytology (Pap)	55-59	58	Not performed	Satisfactory	NILM	NILM	Negative (NILM)	
P08551	2022	2022-Q2	2022-04	Post-pandemic recovery	Cytology (Pap)	45-49	49	Not performed	Satisfactory	NILM	NILM	Negative (NILM)	Candida
P08552	2022	2022-Q2	2022-04	Post-pandemic recovery	Cytology (Pap)	45-49	48	Not performed	Satisfactory	NILM	NILM	Negative (NILM)	
P08553	2022	2022-Q2	2022-04	Post-pandemic recovery	Cytology (Pap)	25-29	26	Not performed	Satisfactory	NILM	NILM	Negative (NILM)	
P08554	2022	2022-Q2	2022-04	Post-pandemic recovery	Cytology (Pap)	45-49	45	Not performed	Satisfactory	NILM	NILM	Negative (NILM)	Candida
P08555	2022	2022-Q2	2022-04	Post-pandemic recovery	Cytology (Pap)	40-44	44	Not performed	Satisfactory	NILM	NILM	Negative (NILM)	Trichomonas
P08556	2022	2022-Q2	2022-04	Post-pandemic recovery	Cytology (Pap)	55-59	58	Not performed	Satisfactory	NILM	NILM	Negative (NILM)	
P08557	2022	2022-Q2	2022-04	Post-pandemic recovery	Cytology (Pap)	45-49	45	Not performed	Satisfactory	NILM	NILM	Negative (NILM)	
P08558	2022	2022-Q2	2022-04	Post-pandemic recovery	Cytology (Pap)	40-44	43	Not performed	Satisfactory	NILM	NILM	Negative (NILM)	

patient_id	year	quarter	month	period	primary_test	age_group	age_years	hpv_result	adequacy	result	bethesda	grade	infection
P08559	2022	2022-Q2	2022-04	Post-pandemic recovery	Cytology (Pap)	30-34	31	Not performed	Satisfactory	NILM	NILM	Negative (NILM)	
P08560	2022	2022-Q2	2022-04	Post-pandemic recovery	Cytology (Pap)	45-49	46	Not performed	Satisfactory	NILM	NILM	Negative (NILM)	
P08561	2022	2022-Q2	2022-04	Post-pandemic recovery	Cytology (Pap)	35-39	39	Not performed	Satisfactory	NILM	NILM	Negative (NILM)	
P08562	2022	2022-Q2	2022-04	Post-pandemic recovery	Cytology (Pap)	50-54	51	Not performed	Satisfactory	Positive	HSIL	High-grade squamous	
P08563	2022	2022-Q2	2022-04	Post-pandemic recovery	Cytology (Pap)	30-34	30	Not performed	Satisfactory	NILM	NILM	Negative (NILM)	
P08564	2022	2022-Q2	2022-04	Post-pandemic recovery	Cytology (Pap)	50-54	52	Not performed	Satisfactory	Positive	LSIL	Low-grade / equivocal squamous	
P08565	2022	2022-Q2	2022-04	Post-pandemic recovery	Cytology (Pap)	40-44	41	Not performed	Satisfactory	NILM	NILM	Negative (NILM)	
P08566	2022	2022-Q2	2022-04	Post-pandemic recovery	Cytology (Pap)	55-59	59	Not performed	Satisfactory	NILM	NILM	Negative (NILM)	
P08567	2022	2022-Q2	2022-04	Post-pandemic recovery	Cytology (Pap)	30-34	33	Not performed	Satisfactory	NILM	NILM	Negative (NILM)	
P08568	2022	2022-Q2	2022-04	Post-pandemic recovery	Cytology (Pap)	35-39	35	Not performed	Satisfactory	NILM	NILM	Negative (NILM)	
P08569	2022	2022-Q2	2022-04	Post-pandemic recovery	Cytology (Pap)	55-59	56	Not performed	Unsatisfactory	Unsatisfactory	Unsatisfactory	Unsatisfactory	
P08570	2022	2022-Q2	2022-04	Post-pandemic recovery	Cytology (Pap)	45-49	47	Not performed	Satisfactory	Positive	ASC-US	Low-grade / equivocal squamous	
P08571	2022	2022-Q2	2022-04	Post-pandemic recovery	Cytology (Pap)	50-54	53	Not performed	Satisfactory	NILM	NILM	Negative (NILM)	
P08572	2022	2022-Q2	2022-04	Post-pandemic recovery	Cytology (Pap)	45-49	45	Not performed	Satisfactory	NILM	NILM	Negative (NILM)	
P08573	2022	2022-Q2	2022-04	Post-pandemic recovery	Cytology (Pap)	50-54	54	Not performed	Satisfactory	NILM	NILM	Negative (NILM)	Gardnerella
P08574	2022	2022-Q2	2022-04	Post-pandemic recovery	Cytology (Pap)	45-49	47	Not performed	Satisfactory	NILM	NILM	Negative (NILM)	
P08575	2022	2022-Q2	2022-04	Post-pandemic recovery	Cytology (Pap)	45-49	49	Not performed	Satisfactory	NILM	NILM	Negative (NILM)	
P08576	2022	2022-Q2	2022-04	Post-pandemic recovery	Cytology (Pap)	25-29	26	Not performed	Satisfactory	NILM	NILM	Negative (NILM)	
P08577	2022	2022-Q2	2022-04	Post-pandemic recovery	Cytology (Pap)	25-29	27	Not performed	Satisfactory	NILM	NILM	Negative (NILM)	
P08578	2022	2022-Q2	2022-04	Post-pandemic recovery	Cytology (Pap)	30-34	33	Not performed	Satisfactory	NILM	NILM	Negative (NILM)	
P08579	2022	2022-Q2	2022-04	Post-pandemic recovery	Cytology (Pap)	40-44	42	Not performed	Satisfactory	NILM	NILM	Negative (NILM)	
P08580	2022	2022-Q2	2022-04	Post-pandemic recovery	Cytology (Pap)	35-39	35	Not performed	Satisfactory	NILM	NILM	Negative (NILM)	Candida
P08581	2022	2022-Q2	2022-04	Post-pandemic recovery	Cytology (Pap)	55-59	55	Not performed	Satisfactory	NILM	NILM	Negative (NILM)	
P08582	2022	2022-Q2	2022-04	Post-pandemic recovery	Cytology (Pap)	40-44	44	Not performed	Satisfactory	NILM	NILM	Negative (NILM)	
P08583	2022	2022-Q2	2022-04	Post-pandemic recovery	Cytology (Pap)	35-39	38	Not performed	Satisfactory	NILM	NILM	Negative (NILM)	
P08584	2022	2022-Q2	2022-04	Post-pandemic recovery	Cytology (Pap)	30-34	32	Not performed	Satisfactory	NILM	NILM	Negative (NILM)	
P08585	2022	2022-Q2	2022-04	Post-pandemic recovery	Cytology (Pap)	40-44	40	Not performed	Satisfactory	NILM	NILM	Negative (NILM)	
P08586	2022	2022-Q2	2022-04	Post-pandemic recovery	Cytology (Pap)	40-44	44	Not performed	Satisfactory	NILM	NILM	Negative (NILM)	
P08587	2022	2022-Q2	2022-04	Post-pandemic recovery	Cytology (Pap)	40-44	41	Not performed	Satisfactory	Positive	LSIL	Low-grade / equivocal squamous	
P08588	2022	2022-Q2	2022-04	Post-pandemic recovery	Cytology (Pap)	55-59	55	Not performed	Satisfactory	NILM	NILM	Negative (NILM)	
P08589	2022	2022-Q2	2022-04	Post-pandemic recovery	Cytology (Pap)	40-44	43	Not performed	Satisfactory	Positive	HSIL	High-grade squamous	
P08590	2022	2022-Q2	2022-04	Post-pandemic recovery	Cytology (Pap)	30-34	32	Not performed	Satisfactory	NILM	NILM	Negative (NILM)	
P08591	2022	2022-Q2	2022-04	Post-pandemic recovery	Cytology (Pap)	55-59	59	Not performed	Satisfactory	NILM	NILM	Negative (NILM)	Candida
P08592	2022	2022-Q2	2022-04	Post-pandemic recovery	Cytology (Pap)	30-34	33	Not performed	Satisfactory	Positive	ASC-H	High-grade squamous	
P08593	2022	2022-Q2	2022-04	Post-pandemic recovery	Cytology (Pap)	55-59	59	Not performed	Satisfactory	NILM	NILM	Negative (NILM)	
P08594	2022	2022-Q2	2022-04	Post-pandemic recovery	Cytology (Pap)	60-64	64	Not performed	Satisfactory	NILM	NILM	Negative (NILM)	
P08595	2022	2022-Q2	2022-04	Post-pandemic recovery	Cytology (Pap)	25-29	26	Not performed	Satisfactory	NILM	NILM	Negative (NILM)	
P08596	2022	2022-Q2	2022-04	Post-pandemic recovery	Cytology (Pap)	25-29	26	Not performed	Satisfactory	NILM	NILM	Negative (NILM)	
P08597	2022	2022-Q2	2022-04	Post-pandemic recovery	Cytology (Pap)	40-44	40	Not performed	Satisfactory	NILM	NILM	Negative (NILM)	
P08598	2022	2022-Q2	2022-04	Post-pandemic recovery	Cytology (Pap)	40-44	42	Not performed	Satisfactory	NILM	NILM	Negative (NILM)	
P08599	2022	2022-Q2	2022-04	Post-pandemic recovery	Cytology (Pap)	30-34	30	Not performed	Satisfactory	NILM	NILM	Negative (NILM)	
P08600	2022	2022-Q2	2022-04	Post-pandemic recovery	Cytology (Pap)	40-44	43	Not performed	Satisfactory	NILM	NILM	Negative (NILM)	
P08601	2022	2022-Q2	2022-04	Post-pandemic recovery	Cytology (Pap)	25-29	29	Not performed	Satisfactory	NILM	NILM	Negative (NILM)	
P08602	2022	2022-Q2	2022-04	Post-pandemic recovery	Cytology (Pap)	60-64	62	Not performed	Satisfactory	NILM	NILM	Negative (NILM)	
P08603	2022	2022-Q2	2022-04	Post-pandemic recovery	Cytology (Pap)	60-64	61	Not performed	Satisfactory	NILM	NILM	Negative (NILM)	
P08604	2022	2022-Q2	2022-04	Post-pandemic recovery	Cytology (Pap)	35-39	35	Not performed	Satisfactory	NILM	NILM	Negative (NILM)	Candida
P08605	2022	2022-Q2	2022-04	Post-pandemic recovery	Cytology (Pap)	35-39	37	Not performed	Satisfactory	NILM	NILM	Negative (NILM)	
P08606	2022	2022-Q2	2022-04	Post-pandemic recovery	Cytology (Pap)	25-29	25	Not performed	Satisfactory	NILM	NILM	Negative (NILM)	
P08607	2022	2022-Q2	2022-04	Post-pandemic recovery	Cytology (Pap)	35-39	39	Not performed	Satisfactory	NILM	NILM	Negative (NILM)	
P08608	2022	2022-Q2	2022-04	Post-pandemic recovery	Cytology (Pap)	55-59	55	Not performed	Satisfactory	NILM	NILM	Negative (NILM)	
P08609	2022	2022-Q2	2022-04	Post-pandemic recovery	Cytology (Pap)	45-49	49	Not performed	Satisfactory	NILM	NILM	Negative (NILM)	
P08610	2022	2022-Q2	2022-04	Post-pandemic recovery	Cytology (Pap)	60-64	61	Not performed	Satisfactory	NILM	NILM	Negative (NILM)	
P08611	2022	2022-Q2	2022-04	Post-pandemic recovery	Cytology (Pap)	45-49	48	Not performed	Satisfactory	NILM	NILM	Negative (NILM)	
P08612	2022	2022-Q2	2022-04	Post-pandemic recovery	Cytology (Pap)	25-29	25	Not performed	Satisfactory	NILM	NILM	Negative (NILM)	
P08613	2022	2022-Q2	2022-04	Post-pandemic recovery	Cytology (Pap)	50-54	51	Not performed	Satisfactory	NILM	NILM	Negative (NILM)	Candida
P08614	2022	2022-Q2	2022-04	Post-pandemic recovery	Cytology (Pap)	50-54	54	Not performed	Satisfactory	NILM	NILM	Negative (NILM)	
P08615	2022	2022-Q2	2022-04	Post-pandemic recovery	Cytology (Pap)	35-39	35	Not performed	Satisfactory	Positive	ASC-US	Low-grade / equivocal squamous	
P08616	2022	2022-Q2	2022-04	Post-pandemic recovery	Cytology (Pap)	30-34	32	Not performed	Satisfactory	NILM	NILM	Negative (NILM)	
P08617	2022	2022-Q2	2022-04	Post-pandemic recovery	Cytology (Pap)	30-34	32	Not performed	Unsatisfactory	Unsatisfactory	Unsatisfactory	Unsatisfactory	
P08618	2022	2022-Q2	2022-04	Post-pandemic recovery	Cytology (Pap)	55-59	55	Not performed	Satisfactory	NILM	NILM	Negative (NILM)	
P08619	2022	2022-Q2	2022-04	Post-pandemic recovery	Cytology (Pap)	45-49	49	Not performed	Satisfactory	NILM	NILM	Negative (NILM)	
P08620	2022	2022-Q2	2022-04	Post-pandemic recovery	Cytology (Pap)	30-34	34	Not performed	Satisfactory	NILM	NILM	Negative (NILM)	
P08621	2022	2022-Q2	2022-04	Post-pandemic recovery	Cytology (Pap)	25-29	25	Not performed	Satisfactory	NILM	NILM	Negative (NILM)	
P08622	2022	2022-Q2	2022-04	Post-pandemic recovery	Cytology (Pap)	40-44	44	Not performed	Satisfactory	NILM	NILM	Negative (NILM)	
P08623	2022	2022-Q2	2022-04	Post-pandemic recovery	Cytology (Pap)	55-59	57	Not performed	Satisfactory	NILM	NILM	Negative (NILM)	
P08624	2022	2022-Q2	2022-04	Post-pandemic recovery	Cytology (Pap)	30-34	30	Not performed	Satisfactory	NILM	NILM	Negative (NILM)	
P08625	2022	2022-Q2	2022-04	Post-pandemic recovery	Cytology (Pap)	40-44	43	Not performed	Satisfactory	NILM	NILM	Negative (NILM)	
P08626	2022	2022-Q2	2022-04	Post-pandemic recovery	Cytology (Pap)	35-39	39	Not performed	Satisfactory	NILM	NILM	Negative (NILM)	
P08627	2022	2022-Q2	2022-04	Post-pandemic recovery	Cytology (Pap)	35-39	35	Not performed	Satisfactory	NILM	NILM	Negative (NILM)	
P08628	2022	2022-Q2	2022-04	Post-pandemic recovery	Cytology (Pap)	45-49	47	Not performed	Satisfactory	NILM	NILM	Negative (NILM)	
P08629	2022	2022-Q2	2022-04	Post-pandemic recovery	Cytology (Pap)	60-64	63	Not performed	Satisfactory	NILM	NILM	Negative (NILM)	
P08630	2022	2022-Q2	2022-04	Post-pandemic recovery	Cytology (Pap)	60-64	63	Not performed	Satisfactory	NILM	NILM	Negative (NILM)	Candida
P08631	2022	2022-Q2	2022-04	Post-pandemic recovery	Cytology (Pap)	25-29	29	Not performed	Satisfactory	NILM	NILM	Negative (NILM)	
P08632	2022	2022-Q2	2022-04	Post-pandemic recovery	Cytology (Pap)	50-54	52	Not performed	Satisfactory	Positive	ASC-US	Low-grade / equivocal squamous	
P08633	2022	2022-Q2	2022-04	Post-pandemic recovery	Cytology (Pap)	45-49	46	Not performed	Satisfactory	NILM	NILM	Negative (NILM)	
P08634	2022	2022-Q2	2022-04	Post-pandemic recovery	Cytology (Pap)	45-49	48	Not performed	Satisfactory	Positive	ASC-US	Low-grade / equivocal squamous	
P08635	2022	2022-Q2	2022-05	Post-pandemic recovery	Cytology (Pap)	50-54	50	Not performed	Satisfactory	NILM	NILM	Negative (NILM)	Other
P08636	2022	2022-Q2	2022-05	Post-pandemic recovery	Cytology (Pap)	45-49	48	Not performed	Satisfactory	Positive	LSIL	Low-grade / equivocal squamous	
P08637	2022	2022-Q2	2022-05	Post-pandemic recovery	Cytology (Pap)	35-39	36	Not performed	Satisfactory	NILM	NILM	Negative (NILM)	
P08638	2022	2022-Q2	2022-05	Post-pandemic recovery	Cytology (Pap)	30-34	34	Not performed	Satisfactory	NILM	NILM	Negative (NILM)	

patient_id	year	quarter	month	period	primary_test	age_group	age_years	hpv_result	adequacy	result	bethesda	grade	infection
P08639	2022	2022-Q2	2022-05	Post-pandemic recovery	Cytology (Pap)	35-39	39	Not performed	Satisfactory	NILM	NILM	Negative (NILM)	
P08640	2022	2022-Q2	2022-05	Post-pandemic recovery	Cytology (Pap)	25-29	27	Not performed	Satisfactory	NILM	NILM	Negative (NILM)	
P08641	2022	2022-Q2	2022-05	Post-pandemic recovery	Cytology (Pap)	30-34	32	Not performed	Satisfactory	NILM	NILM	Negative (NILM)	
P08642	2022	2022-Q2	2022-05	Post-pandemic recovery	Cytology (Pap)	55-59	58	Not performed	Satisfactory	NILM	NILM	Negative (NILM)	
P08643	2022	2022-Q2	2022-05	Post-pandemic recovery	Cytology (Pap)	25-29	25	Not performed	Satisfactory	Positive	LSIL	Low-grade / equivocal squamous	
P08644	2022	2022-Q2	2022-05	Post-pandemic recovery	Cytology (Pap)	45-49	45	Not performed	Satisfactory	NILM	NILM	Negative (NILM)	
P08645	2022	2022-Q2	2022-05	Post-pandemic recovery	Cytology (Pap)	45-49	46	Not performed	Satisfactory	Positive	LSIL	Low-grade / equivocal squamous	
P08646	2022	2022-Q2	2022-05	Post-pandemic recovery	Cytology (Pap)	25-29	29	Not performed	Satisfactory	NILM	NILM	Negative (NILM)	
P08647	2022	2022-Q2	2022-05	Post-pandemic recovery	Cytology (Pap)	45-49	47	Not performed	Satisfactory	NILM	NILM	Negative (NILM)	
P08648	2022	2022-Q2	2022-05	Post-pandemic recovery	Cytology (Pap)	45-49	46	Not performed	Satisfactory	NILM	NILM	Negative (NILM)	
P08649	2022	2022-Q2	2022-05	Post-pandemic recovery	Cytology (Pap)	30-34	30	Not performed	Satisfactory	NILM	NILM	Negative (NILM)	
P08650	2022	2022-Q2	2022-05	Post-pandemic recovery	Cytology (Pap)	25-29	26	Not performed	Satisfactory	NILM	NILM	Negative (NILM)	
P08651	2022	2022-Q2	2022-05	Post-pandemic recovery	Cytology (Pap)	50-54	53	Not performed	Satisfactory	NILM	NILM	Negative (NILM)	
P08652	2022	2022-Q2	2022-05	Post-pandemic recovery	Cytology (Pap)	45-49	48	Not performed	Satisfactory	NILM	NILM	Negative (NILM)	
P08653	2022	2022-Q2	2022-05	Post-pandemic recovery	Cytology (Pap)	60-64	64	Not performed	Satisfactory	NILM	NILM	Negative (NILM)	
P08654	2022	2022-Q2	2022-05	Post-pandemic recovery	Cytology (Pap)	50-54	52	Not performed	Satisfactory	NILM	NILM	Negative (NILM)	
P08655	2022	2022-Q2	2022-05	Post-pandemic recovery	Cytology (Pap)	40-44	41	Not performed	Satisfactory	NILM	NILM	Negative (NILM)	
P08656	2022	2022-Q2	2022-05	Post-pandemic recovery	Cytology (Pap)	55-59	58	Not performed	Unsatisfactory	Unsatisfactory	Unsatisfactory	Unsatisfactory	
P08657	2022	2022-Q2	2022-05	Post-pandemic recovery	Cytology (Pap)	35-39	35	Not performed	Satisfactory	NILM	NILM	Negative (NILM)	
P08658	2022	2022-Q2	2022-05	Post-pandemic recovery	Cytology (Pap)	25-29	28	Not performed	Satisfactory	Positive	LSIL	Low-grade / equivocal squamous	
P08659	2022	2022-Q2	2022-05	Post-pandemic recovery	Cytology (Pap)	25-29	27	Not performed	Satisfactory	NILM	NILM	Negative (NILM)	
P08660	2022	2022-Q2	2022-05	Post-pandemic recovery	Cytology (Pap)	40-44	43	Not performed	Satisfactory	NILM	NILM	Negative (NILM)	
P08661	2022	2022-Q2	2022-05	Post-pandemic recovery	Cytology (Pap)	25-29	25	Not performed	Satisfactory	NILM	NILM	Negative (NILM)	
P08662	2022	2022-Q2	2022-05	Post-pandemic recovery	Cytology (Pap)	30-34	32	Not performed	Satisfactory	Positive	ASC-US	Low-grade / equivocal squamous	
P08663	2022	2022-Q2	2022-05	Post-pandemic recovery	Cytology (Pap)	50-54	52	Not performed	Satisfactory	NILM	NILM	Negative (NILM)	
P08664	2022	2022-Q2	2022-05	Post-pandemic recovery	Cytology (Pap)	50-54	50	Not performed	Satisfactory	NILM	NILM	Negative (NILM)	
P08665	2022	2022-Q2	2022-05	Post-pandemic recovery	Cytology (Pap)	30-34	32	Not performed	Satisfactory	NILM	NILM	Negative (NILM)	
P08666	2022	2022-Q2	2022-05	Post-pandemic recovery	Cytology (Pap)	40-44	41	Not performed	Satisfactory	NILM	NILM	Negative (NILM)	
P08667	2022	2022-Q2	2022-05	Post-pandemic recovery	Cytology (Pap)	55-59	57	Not performed	Unsatisfactory	Unsatisfactory	Unsatisfactory	Unsatisfactory	Candida
P08668	2022	2022-Q2	2022-05	Post-pandemic recovery	Cytology (Pap)	55-59	56	Not performed	Satisfactory	Positive	ASC-H	High-grade squamous	Other
P08669	2022	2022-Q2	2022-05	Post-pandemic recovery	Cytology (Pap)	35-39	36	Not performed	Satisfactory	NILM	NILM	Negative (NILM)	Candida
P08670	2022	2022-Q2	2022-05	Post-pandemic recovery	Cytology (Pap)	35-39	37	Not performed	Satisfactory	NILM	NILM	Negative (NILM)	
P08671	2022	2022-Q2	2022-05	Post-pandemic recovery	Cytology (Pap)	35-39	38	Not performed	Satisfactory	NILM	NILM	Negative (NILM)	
P08672	2022	2022-Q2	2022-05	Post-pandemic recovery	Cytology (Pap)	25-29	29	Not performed	Satisfactory	NILM	NILM	Negative (NILM)	
P08673	2022	2022-Q2	2022-05	Post-pandemic recovery	Cytology (Pap)	55-59	56	Not performed	Satisfactory	Positive	HSIL	High-grade squamous	
P08674	2022	2022-Q2	2022-05	Post-pandemic recovery	Cytology (Pap)	30-34	31	Not performed	Satisfactory	NILM	NILM	Negative (NILM)	
P08675	2022	2022-Q2	2022-05	Post-pandemic recovery	Cytology (Pap)	45-49	49	Not performed	Satisfactory	NILM	NILM	Negative (NILM)	
P08676	2022	2022-Q2	2022-05	Post-pandemic recovery	Cytology (Pap)	45-49	49	Not performed	Satisfactory	NILM	NILM	Negative (NILM)	
P08677	2022	2022-Q2	2022-05	Post-pandemic recovery	Cytology (Pap)	30-34	32	Not performed	Satisfactory	NILM	NILM	Negative (NILM)	
P08678	2022	2022-Q2	2022-05	Post-pandemic recovery	Cytology (Pap)	50-54	53	Not performed	Satisfactory	NILM	NILM	Negative (NILM)	
P08679	2022	2022-Q2	2022-05	Post-pandemic recovery	Cytology (Pap)	25-29	29	Not performed	Satisfactory	NILM	NILM	Negative (NILM)	
P08680	2022	2022-Q2	2022-05	Post-pandemic recovery	Cytology (Pap)	30-34	33	Not performed	Satisfactory	NILM	NILM	Negative (NILM)	
P08681	2022	2022-Q2	2022-05	Post-pandemic recovery	Cytology (Pap)	45-49	46	Not performed	Satisfactory	NILM	NILM	Negative (NILM)	
P08682	2022	2022-Q2	2022-05	Post-pandemic recovery	Cytology (Pap)	35-39	38	Not performed	Satisfactory	NILM	NILM	Negative (NILM)	
P08683	2022	2022-Q2	2022-05	Post-pandemic recovery	Cytology (Pap)	55-59	59	Not performed	Satisfactory	NILM	NILM	Negative (NILM)	
P08684	2022	2022-Q2	2022-05	Post-pandemic recovery	Cytology (Pap)	25-29	28	Not performed	Satisfactory	NILM	NILM	Negative (NILM)	
P08685	2022	2022-Q2	2022-05	Post-pandemic recovery	Cytology (Pap)	25-29	27	Not performed	Satisfactory	NILM	NILM	Negative (NILM)	
P08686	2022	2022-Q2	2022-05	Post-pandemic recovery	Cytology (Pap)	45-49	47	Not performed	Satisfactory	NILM	NILM	Negative (NILM)	
P08687	2022	2022-Q2	2022-05	Post-pandemic recovery	Cytology (Pap)	25-29	28	Not performed	Satisfactory	NILM	NILM	Negative (NILM)	
P08688	2022	2022-Q2	2022-05	Post-pandemic recovery	Cytology (Pap)	45-49	45	Not performed	Satisfactory	NILM	NILM	Negative (NILM)	
P08689	2022	2022-Q2	2022-05	Post-pandemic recovery	Cytology (Pap)	50-54	51	Not performed	Satisfactory	NILM	NILM	Negative (NILM)	
P08690	2022	2022-Q2	2022-05	Post-pandemic recovery	Cytology (Pap)	55-59	58	Not performed	Satisfactory	NILM	NILM	Negative (NILM)	
P08691	2022	2022-Q2	2022-05	Post-pandemic recovery	Cytology (Pap)	35-39	36	Not performed	Satisfactory	NILM	NILM	Negative (NILM)	
P08692	2022	2022-Q2	2022-05	Post-pandemic recovery	Cytology (Pap)	55-59	58	Not performed	Satisfactory	NILM	NILM	Negative (NILM)	
P08693	2022	2022-Q2	2022-05	Post-pandemic recovery	Cytology (Pap)	50-54	53	Not performed	Satisfactory	Positive	ASC-US	Low-grade / equivocal squamous	
P08694	2022	2022-Q2	2022-05	Post-pandemic recovery	Cytology (Pap)	50-54	52	Not performed	Satisfactory	NILM	NILM	Negative (NILM)	
P08695	2022	2022-Q2	2022-05	Post-pandemic recovery	Cytology (Pap)	50-54	50	Not performed	Satisfactory	NILM	NILM	Negative (NILM)	
P08696	2022	2022-Q2	2022-05	Post-pandemic recovery	Cytology (Pap)	55-59	57	Not performed	Satisfactory	NILM	NILM	Negative (NILM)	
P08697	2022	2022-Q2	2022-05	Post-pandemic recovery	Cytology (Pap)	45-49	49	Not performed	Satisfactory	NILM	NILM	Negative (NILM)	
P08698	2022	2022-Q2	2022-05	Post-pandemic recovery	Cytology (Pap)	50-54	51	Not performed	Satisfactory	NILM	NILM	Negative (NILM)	
P08699	2022	2022-Q2	2022-05	Post-pandemic recovery	Cytology (Pap)	40-44	44	Not performed	Satisfactory	NILM	NILM	Negative (NILM)	
P08700	2022	2022-Q2	2022-05	Post-pandemic recovery	Cytology (Pap)	40-44	41	Not performed	Satisfactory	NILM	NILM	Negative (NILM)	
P08701	2022	2022-Q2	2022-05	Post-pandemic recovery	Cytology (Pap)	55-59	59	Not performed	Satisfactory	NILM	NILM	Negative (NILM)	
P08702	2022	2022-Q2	2022-05	Post-pandemic recovery	Cytology (Pap)	45-49	46	Not performed	Satisfactory	NILM	NILM	Negative (NILM)	
P08703	2022	2022-Q2	2022-05	Post-pandemic recovery	Cytology (Pap)	35-39	38	Not performed	Satisfactory	NILM	NILM	Negative (NILM)	
P08704	2022	2022-Q2	2022-05	Post-pandemic recovery	Cytology (Pap)	40-44	41	Not performed	Satisfactory	Positive	LSIL	Low-grade / equivocal squamous	
P08705	2022	2022-Q2	2022-05	Post-pandemic recovery	Cytology (Pap)	30-34	31	Not performed	Satisfactory	NILM	NILM	Negative (NILM)	
P08706	2022	2022-Q2	2022-05	Post-pandemic recovery	Cytology (Pap)	45-49	49	Not performed	Satisfactory	NILM	NILM	Negative (NILM)	
P08707	2022	2022-Q2	2022-05	Post-pandemic recovery	Cytology (Pap)	50-54	51	Not performed	Satisfactory	NILM	NILM	Negative (NILM)	
P08708	2022	2022-Q2	2022-05	Post-pandemic recovery	Cytology (Pap)	50-54	52	Not performed	Satisfactory	NILM	NILM	Negative (NILM)	
P08709	2022	2022-Q2	2022-05	Post-pandemic recovery	Cytology (Pap)	45-49	46	Not performed	Satisfactory	NILM	NILM	Negative (NILM)	
P08710	2022	2022-Q2	2022-05	Post-pandemic recovery	Cytology (Pap)	40-44	40	Not performed	Satisfactory	NILM	NILM	Negative (NILM)	
P08711	2022	2022-Q2	2022-05	Post-pandemic recovery	Cytology (Pap)	45-49	45	Not performed	Satisfactory	NILM	NILM	Negative (NILM)	
P08712	2022	2022-Q2	2022-05	Post-pandemic recovery	Cytology (Pap)	40-44	40	Not performed	Satisfactory	Positive	HSIL	High-grade squamous	
P08713	2022	2022-Q2	2022-05	Post-pandemic recovery	Cytology (Pap)	45-49	45	Not performed	Satisfactory	NILM	NILM	Negative (NILM)	
P08714	2022	2022-Q2	2022-05	Post-pandemic recovery	Cytology (Pap)	25-29	27	Not performed	Satisfactory	NILM	NILM	Negative (NILM)	
P08715	2022	2022-Q2	2022-05	Post-pandemic recovery	Cytology (Pap)	50-54	52	Not performed	Satisfactory	NILM	NILM	Negative (NILM)	Trichomonas
P08716	2022	2022-Q2	2022-05	Post-pandemic recovery	Cytology (Pap)	25-29	27	Not performed	Satisfactory	NILM	NILM	Negative (NILM)	
P08717	2022	2022-Q2	2022-05	Post-pandemic recovery	Cytology (Pap)	30-34	31	Not performed	Satisfactory	NILM	NILM	Negative (NILM)	
P08718	2022	2022-Q2	2022-05	Post-pandemic recovery	Cytology (Pap)	35-39	39	Not performed	Satisfactory	NILM	NILM	Negative (NILM)	

patient_id	year	quarter	month	period	primary_test	age_group	age_years	hpv_result	adequacy	result	bethesda	grade	infection
P08719	2022	2022-Q2	2022-05	Post-pandemic recovery	Cytology (Pap)	45-49	46	Not performed	Satisfactory	NILM	NILM	Negative (NILM)	
P08720	2022	2022-Q2	2022-05	Post-pandemic recovery	Cytology (Pap)	40-44	43	Not performed	Satisfactory	NILM	NILM	Negative (NILM)	
P08721	2022	2022-Q2	2022-05	Post-pandemic recovery	Cytology (Pap)	50-54	54	Not performed	Satisfactory	NILM	NILM	Negative (NILM)	
P08722	2022	2022-Q2	2022-05	Post-pandemic recovery	Cytology (Pap)	40-44	42	Not performed	Satisfactory	NILM	NILM	Negative (NILM)	
P08723	2022	2022-Q2	2022-05	Post-pandemic recovery	Cytology (Pap)	40-44	41	Not performed	Satisfactory	NILM	NILM	Negative (NILM)	
P08724	2022	2022-Q2	2022-05	Post-pandemic recovery	Cytology (Pap)	50-54	50	Not performed	Satisfactory	NILM	NILM	Negative (NILM)	
P08725	2022	2022-Q2	2022-05	Post-pandemic recovery	Cytology (Pap)	35-39	36	Not performed	Satisfactory	NILM	NILM	Negative (NILM)	
P08726	2022	2022-Q2	2022-05	Post-pandemic recovery	Cytology (Pap)	45-49	49	Not performed	Satisfactory	Positive	HSIL	High-grade squamous	
P08727	2022	2022-Q2	2022-05	Post-pandemic recovery	Cytology (Pap)	50-54	51	Not performed	Satisfactory	NILM	NILM	Negative (NILM)	
P08728	2022	2022-Q2	2022-05	Post-pandemic recovery	Cytology (Pap)	40-44	43	Not performed	Unsatisfactory	Unsatisfactory	Unsatisfactory	Unsatisfactory	
P08729	2022	2022-Q2	2022-05	Post-pandemic recovery	Cytology (Pap)	40-44	40	Not performed	Satisfactory	NILM	NILM	Negative (NILM)	Other
P08730	2022	2022-Q2	2022-05	Post-pandemic recovery	Cytology (Pap)	60-64	61	Not performed	Satisfactory	NILM	NILM	Negative (NILM)	
P08731	2022	2022-Q2	2022-05	Post-pandemic recovery	Cytology (Pap)	60-64	61	Not performed	Satisfactory	NILM	NILM	Negative (NILM)	
P08732	2022	2022-Q2	2022-05	Post-pandemic recovery	Cytology (Pap)	55-59	57	Not performed	Satisfactory	NILM	NILM	Negative (NILM)	
P08733	2022	2022-Q2	2022-05	Post-pandemic recovery	Cytology (Pap)	30-34	33	Not performed	Satisfactory	NILM	NILM	Negative (NILM)	
P08734	2022	2022-Q2	2022-05	Post-pandemic recovery	Cytology (Pap)	35-39	35	Not performed	Satisfactory	Positive	HSIL	High-grade squamous	
P08735	2022	2022-Q2	2022-05	Post-pandemic recovery	Cytology (Pap)	40-44	43	Not performed	Satisfactory	NILM	NILM	Negative (NILM)	
P08736	2022	2022-Q2	2022-05	Post-pandemic recovery	Cytology (Pap)	60-64	62	Not performed	Satisfactory	NILM	NILM	Negative (NILM)	
P08737	2022	2022-Q2	2022-05	Post-pandemic recovery	Cytology (Pap)	50-54	54	Not performed	Satisfactory	NILM	NILM	Negative (NILM)	
P08738	2022	2022-Q2	2022-05	Post-pandemic recovery	Cytology (Pap)	35-39	36	Not performed	Satisfactory	Positive	ASC-US	Low-grade / equivocal squamous	
P08739	2022	2022-Q2	2022-05	Post-pandemic recovery	Cytology (Pap)	40-44	41	Not performed	Unsatisfactory	Unsatisfactory	Unsatisfactory	Unsatisfactory	
P08740	2022	2022-Q2	2022-05	Post-pandemic recovery	Cytology (Pap)	40-44	42	Not performed	Satisfactory	NILM	NILM	Negative (NILM)	
P08741	2022	2022-Q2	2022-05	Post-pandemic recovery	Cytology (Pap)	25-29	25	Not performed	Satisfactory	NILM	NILM	Negative (NILM)	
P08742	2022	2022-Q2	2022-05	Post-pandemic recovery	Cytology (Pap)	45-49	45	Not performed	Satisfactory	NILM	NILM	Negative (NILM)	
P08743	2022	2022-Q2	2022-05	Post-pandemic recovery	Cytology (Pap)	35-39	36	Not performed	Satisfactory	Positive	HSIL	High-grade squamous	
P08744	2022	2022-Q2	2022-05	Post-pandemic recovery	Cytology (Pap)	40-44	42	Not performed	Satisfactory	NILM	NILM	Negative (NILM)	
P08745	2022	2022-Q2	2022-05	Post-pandemic recovery	Cytology (Pap)	25-29	27	Not performed	Satisfactory	NILM	NILM	Negative (NILM)	Candida
P08746	2022	2022-Q2	2022-05	Post-pandemic recovery	Cytology (Pap)	55-59	56	Not performed	Unsatisfactory	Unsatisfactory	Unsatisfactory	Unsatisfactory	
P08747	2022	2022-Q2	2022-05	Post-pandemic recovery	Cytology (Pap)	50-54	53	Not performed	Satisfactory	NILM	NILM	Negative (NILM)	
P08748	2022	2022-Q2	2022-05	Post-pandemic recovery	Cytology (Pap)	50-54	53	Not performed	Satisfactory	NILM	NILM	Negative (NILM)	
P08749	2022	2022-Q2	2022-05	Post-pandemic recovery	Cytology (Pap)	50-54	52	Not performed	Satisfactory	NILM	NILM	Negative (NILM)	
P08750	2022	2022-Q2	2022-05	Post-pandemic recovery	Cytology (Pap)	55-59	59	Not performed	Satisfactory	NILM	NILM	Negative (NILM)	
P08751	2022	2022-Q2	2022-05	Post-pandemic recovery	Cytology (Pap)	30-34	34	Not performed	Satisfactory	NILM	NILM	Negative (NILM)	
P08752	2022	2022-Q2	2022-05	Post-pandemic recovery	Cytology (Pap)	25-29	25	Not performed	Satisfactory	Positive	ASC-US	Low-grade / equivocal squamous	
P08753	2022	2022-Q2	2022-05	Post-pandemic recovery	Cytology (Pap)	55-59	55	Not performed	Satisfactory	NILM	NILM	Negative (NILM)	
P08754	2022	2022-Q2	2022-05	Post-pandemic recovery	Cytology (Pap)	25-29	27	Not performed	Satisfactory	NILM	NILM	Negative (NILM)	
P08755	2022	2022-Q2	2022-05	Post-pandemic recovery	Cytology (Pap)	30-34	30	Not performed	Satisfactory	NILM	NILM	Negative (NILM)	
P08756	2022	2022-Q2	2022-05	Post-pandemic recovery	Cytology (Pap)	35-39	36	Not performed	Satisfactory	NILM	NILM	Negative (NILM)	
P08757	2022	2022-Q2	2022-05	Post-pandemic recovery	Cytology (Pap)	55-59	56	Not performed	Satisfactory	NILM	NILM	Negative (NILM)	
P08758	2022	2022-Q2	2022-05	Post-pandemic recovery	Cytology (Pap)	60-64	64	Not performed	Satisfactory	NILM	NILM	Negative (NILM)	
P08759	2022	2022-Q2	2022-05	Post-pandemic recovery	Cytology (Pap)	45-49	49	Not performed	Satisfactory	NILM	NILM	Negative (NILM)	
P08760	2022	2022-Q2	2022-05	Post-pandemic recovery	Cytology (Pap)	40-44	41	Not performed	Satisfactory	NILM	NILM	Negative (NILM)	
P08761	2022	2022-Q2	2022-05	Post-pandemic recovery	Cytology (Pap)	30-34	31	Not performed	Satisfactory	Positive	LSIL	Low-grade / equivocal squamous	
P08762	2022	2022-Q2	2022-06	Post-pandemic recovery	Cytology (Pap)	35-39	38	Not performed	Satisfactory	NILM	NILM	Negative (NILM)	
P08763	2022	2022-Q2	2022-06	Post-pandemic recovery	Cytology (Pap)	50-54	54	Not performed	Satisfactory	NILM	NILM	Negative (NILM)	
P08764	2022	2022-Q2	2022-06	Post-pandemic recovery	Cytology (Pap)	25-29	25	Not performed	Satisfactory	NILM	NILM	Negative (NILM)	
P08765	2022	2022-Q2	2022-06	Post-pandemic recovery	Cytology (Pap)	40-44	41	Not performed	Satisfactory	Positive	HSIL	High-grade squamous	
P08766	2022	2022-Q2	2022-06	Post-pandemic recovery	Cytology (Pap)	45-49	48	Not performed	Satisfactory	NILM	NILM	Negative (NILM)	
P08767	2022	2022-Q2	2022-06	Post-pandemic recovery	Cytology (Pap)	40-44	44	Not performed	Satisfactory	Positive	ASC-H	High-grade squamous	
P08768	2022	2022-Q2	2022-06	Post-pandemic recovery	Cytology (Pap)	40-44	40	Not performed	Satisfactory	NILM	NILM	Negative (NILM)	
P08769	2022	2022-Q2	2022-06	Post-pandemic recovery	Cytology (Pap)	45-49	46	Not performed	Satisfactory	Positive	ASC-US	Low-grade / equivocal squamous	
P08770	2022	2022-Q2	2022-06	Post-pandemic recovery	Cytology (Pap)	35-39	39	Not performed	Satisfactory	NILM	NILM	Negative (NILM)	
P08771	2022	2022-Q2	2022-06	Post-pandemic recovery	Cytology (Pap)	50-54	54	Not performed	Satisfactory	Positive	LSIL	Low-grade / equivocal squamous	
P08772	2022	2022-Q2	2022-06	Post-pandemic recovery	Cytology (Pap)	45-49	45	Not performed	Satisfactory	NILM	NILM	Negative (NILM)	
P08773	2022	2022-Q2	2022-06	Post-pandemic recovery	Cytology (Pap)	45-49	45	Not performed	Satisfactory	NILM	NILM	Negative (NILM)	
P08774	2022	2022-Q2	2022-06	Post-pandemic recovery	Cytology (Pap)	25-29	28	Not performed	Satisfactory	NILM	NILM	Negative (NILM)	
P08775	2022	2022-Q2	2022-06	Post-pandemic recovery	Cytology (Pap)	50-54	52	Not performed	Satisfactory	NILM	NILM	Negative (NILM)	
P08776	2022	2022-Q2	2022-06	Post-pandemic recovery	Cytology (Pap)	40-44	44	Not performed	Satisfactory	Positive	HSIL	High-grade squamous	
P08777	2022	2022-Q2	2022-06	Post-pandemic recovery	Cytology (Pap)	40-44	43	Not performed	Satisfactory	NILM	NILM	Negative (NILM)	
P08778	2022	2022-Q2	2022-06	Post-pandemic recovery	Cytology (Pap)	40-44	42	Not performed	Satisfactory	NILM	NILM	Negative (NILM)	
P08779	2022	2022-Q2	2022-06	Post-pandemic recovery	Cytology (Pap)	35-39	35	Not performed	Satisfactory	NILM	NILM	Negative (NILM)	
P08780	2022	2022-Q2	2022-06	Post-pandemic recovery	Cytology (Pap)	30-34	30	Not performed	Satisfactory	NILM	NILM	Negative (NILM)	
P08781	2022	2022-Q2	2022-06	Post-pandemic recovery	Cytology (Pap)	45-49	47	Not performed	Satisfactory	Positive	AGC	Glandular	
P08782	2022	2022-Q2	2022-06	Post-pandemic recovery	Cytology (Pap)	45-49	46	Not performed	Satisfactory	NILM	NILM	Negative (NILM)	
P08783	2022	2022-Q2	2022-06	Post-pandemic recovery	Cytology (Pap)	30-34	33	Not performed	Satisfactory	Positive	LSIL	Low-grade / equivocal squamous	
P08784	2022	2022-Q2	2022-06	Post-pandemic recovery	Cytology (Pap)	35-39	37	Not performed	Satisfactory	NILM	NILM	Negative (NILM)	
P08785	2022	2022-Q2	2022-06	Post-pandemic recovery	Cytology (Pap)	40-44	42	Not performed	Satisfactory	NILM	NILM	Negative (NILM)	
P08786	2022	2022-Q2	2022-06	Post-pandemic recovery	Cytology (Pap)	25-29	26	Not performed	Satisfactory	Positive	LSIL	Low-grade / equivocal squamous	
P08787	2022	2022-Q2	2022-06	Post-pandemic recovery	Cytology (Pap)	35-39	35	Not performed	Satisfactory	NILM	NILM	Negative (NILM)	
P08788	2022	2022-Q2	2022-06	Post-pandemic recovery	Cytology (Pap)	30-34	31	Not performed	Satisfactory	NILM	NILM	Negative (NILM)	
P08789	2022	2022-Q2	2022-06	Post-pandemic recovery	Cytology (Pap)	55-59	59	Not performed	Satisfactory	NILM	NILM	Negative (NILM)	
P08790	2022	2022-Q2	2022-06	Post-pandemic recovery	Cytology (Pap)	40-44	41	Not performed	Satisfactory	NILM	NILM	Negative (NILM)	
P08791	2022	2022-Q2	2022-06	Post-pandemic recovery	Cytology (Pap)	30-34	34	Not performed	Satisfactory	NILM	NILM	Negative (NILM)	
P08792	2022	2022-Q2	2022-06	Post-pandemic recovery	Cytology (Pap)	50-54	51	Not performed	Satisfactory	NILM	NILM	Negative (NILM)	
P08793	2022	2022-Q2	2022-06	Post-pandemic recovery	Cytology (Pap)	60-64	63	Not performed	Satisfactory	NILM	NILM	Negative (NILM)	
P08794	2022	2022-Q2	2022-06	Post-pandemic recovery	Cytology (Pap)	45-49	46	Not performed	Satisfactory	NILM	NILM	Negative (NILM)	
P08795	2022	2022-Q2	2022-06	Post-pandemic recovery	Cytology (Pap)	60-64	62	Not performed	Satisfactory	NILM	NILM	Negative (NILM)	Candida
P08796	2022	2022-Q2	2022-06	Post-pandemic recovery	Cytology (Pap)	40-44	42	Not performed	Satisfactory	NILM	NILM	Negative (NILM)	
P08797	2022	2022-Q2	2022-06	Post-pandemic recovery	Cytology (Pap)	35-39	38	Not performed	Satisfactory	NILM	NILM	Negative (NILM)	
P08798	2022	2022-Q2	2022-06	Post-pandemic recovery	Cytology (Pap)	45-49	46	Not performed	Satisfactory	NILM	NILM	Negative (NILM)	

patient_id	year	quarter	month	period	primary_test	age_group	age_years	hpv_result	adequacy	result	bethesda	grade	infection
P08799	2022	2022-Q2	2022-06	Post-pandemic recovery	Cytology (Pap)	30-34	31	Not performed	Satisfactory	Positive	LSIL	Low-grade / equivocal squamous	
P08800	2022	2022-Q2	2022-06	Post-pandemic recovery	Cytology (Pap)	30-34	31	Not performed	Satisfactory	NILM	NILM	Negative (NILM)	
P08801	2022	2022-Q2	2022-06	Post-pandemic recovery	Cytology (Pap)	55-59	59	Not performed	Satisfactory	NILM	NILM	Negative (NILM)	
P08802	2022	2022-Q2	2022-06	Post-pandemic recovery	Cytology (Pap)	50-54	51	Not performed	Satisfactory	NILM	NILM	Negative (NILM)	
P08803	2022	2022-Q2	2022-06	Post-pandemic recovery	Cytology (Pap)	40-44	40	Not performed	Unsatisfactory	Unsatisfactory	Unsatisfactory	Unsatisfactory	Candida
P08804	2022	2022-Q2	2022-06	Post-pandemic recovery	Cytology (Pap)	55-59	55	Not performed	Satisfactory	NILM	NILM	Negative (NILM)	Candida
P08805	2022	2022-Q2	2022-06	Post-pandemic recovery	Cytology (Pap)	40-44	42	Not performed	Satisfactory	NILM	NILM	Negative (NILM)	
P08806	2022	2022-Q2	2022-06	Post-pandemic recovery	Cytology (Pap)	45-49	46	Not performed	Unsatisfactory	Unsatisfactory	Unsatisfactory	Unsatisfactory	
P08807	2022	2022-Q2	2022-06	Post-pandemic recovery	Cytology (Pap)	55-59	58	Not performed	Satisfactory	NILM	NILM	Negative (NILM)	
P08808	2022	2022-Q2	2022-06	Post-pandemic recovery	Cytology (Pap)	30-34	32	Not performed	Satisfactory	NILM	NILM	Negative (NILM)	
P08809	2022	2022-Q2	2022-06	Post-pandemic recovery	Cytology (Pap)	55-59	59	Not performed	Satisfactory	NILM	NILM	Negative (NILM)	
P08810	2022	2022-Q2	2022-06	Post-pandemic recovery	Cytology (Pap)	45-49	45	Not performed	Satisfactory	NILM	NILM	Negative (NILM)	
P08811	2022	2022-Q2	2022-06	Post-pandemic recovery	Cytology (Pap)	35-39	35	Not performed	Satisfactory	Positive	ASC-US	Low-grade / equivocal squamous	
P08812	2022	2022-Q2	2022-06	Post-pandemic recovery	Cytology (Pap)	55-59	56	Not performed	Unsatisfactory	Unsatisfactory	Unsatisfactory	Unsatisfactory	
P08813	2022	2022-Q2	2022-06	Post-pandemic recovery	Cytology (Pap)	35-39	36	Not performed	Satisfactory	NILM	NILM	Negative (NILM)	
P08814	2022	2022-Q2	2022-06	Post-pandemic recovery	Cytology (Pap)	40-44	42	Not performed	Satisfactory	Positive	LSIL	Low-grade / equivocal squamous	
P08815	2022	2022-Q2	2022-06	Post-pandemic recovery	Cytology (Pap)	25-29	28	Not performed	Satisfactory	NILM	NILM	Negative (NILM)	
P08816	2022	2022-Q2	2022-06	Post-pandemic recovery	Cytology (Pap)	30-34	32	Not performed	Satisfactory	NILM	NILM	Negative (NILM)	
P08817	2022	2022-Q2	2022-06	Post-pandemic recovery	Cytology (Pap)	60-64	62	Not performed	Satisfactory	NILM	NILM	Negative (NILM)	
P08818	2022	2022-Q2	2022-06	Post-pandemic recovery	Cytology (Pap)	40-44	42	Not performed	Satisfactory	Positive	ASC-US	Low-grade / equivocal squamous	
P08819	2022	2022-Q2	2022-06	Post-pandemic recovery	Cytology (Pap)	30-34	33	Not performed	Satisfactory	Positive	HSIL	High-grade squamous	
P08820	2022	2022-Q2	2022-06	Post-pandemic recovery	Cytology (Pap)	55-59	59	Not performed	Satisfactory	NILM	NILM	Negative (NILM)	
P08821	2022	2022-Q2	2022-06	Post-pandemic recovery	Cytology (Pap)	60-64	63	Not performed	Satisfactory	Positive	ASC-US	Low-grade / equivocal squamous	
P08822	2022	2022-Q2	2022-06	Post-pandemic recovery	Cytology (Pap)	25-29	27	Not performed	Satisfactory	NILM	NILM	Negative (NILM)	
P08823	2022	2022-Q2	2022-06	Post-pandemic recovery	Cytology (Pap)	30-34	31	Not performed	Satisfactory	NILM	NILM	Negative (NILM)	
P08824	2022	2022-Q2	2022-06	Post-pandemic recovery	Cytology (Pap)	40-44	43	Not performed	Satisfactory	NILM	NILM	Negative (NILM)	Candida
P08825	2022	2022-Q2	2022-06	Post-pandemic recovery	Cytology (Pap)	40-44	43	Not performed	Satisfactory	Positive	ASC-US	Low-grade / equivocal squamous	
P08826	2022	2022-Q2	2022-06	Post-pandemic recovery	Cytology (Pap)	55-59	57	Not performed	Satisfactory	NILM	NILM	Negative (NILM)	
P08827	2022	2022-Q2	2022-06	Post-pandemic recovery	Cytology (Pap)	40-44	43	Not performed	Satisfactory	NILM	NILM	Negative (NILM)	Other
P08828	2022	2022-Q2	2022-06	Post-pandemic recovery	Cytology (Pap)	60-64	60	Not performed	Satisfactory	NILM	NILM	Negative (NILM)	
P08829	2022	2022-Q2	2022-06	Post-pandemic recovery	Cytology (Pap)	30-34	32	Not performed	Satisfactory	NILM	NILM	Negative (NILM)	
P08830	2022	2022-Q2	2022-06	Post-pandemic recovery	Cytology (Pap)	40-44	43	Not performed	Satisfactory	NILM	NILM	Negative (NILM)	
P08831	2022	2022-Q2	2022-06	Post-pandemic recovery	Cytology (Pap)	35-39	39	Not performed	Satisfactory	NILM	NILM	Negative (NILM)	
P08832	2022	2022-Q2	2022-06	Post-pandemic recovery	Cytology (Pap)	30-34	30	Not performed	Satisfactory	NILM	NILM	Negative (NILM)	
P08833	2022	2022-Q2	2022-06	Post-pandemic recovery	Cytology (Pap)	35-39	35	Not performed	Satisfactory	NILM	NILM	Negative (NILM)	
P08834	2022	2022-Q2	2022-06	Post-pandemic recovery	Cytology (Pap)	55-59	57	Not performed	Satisfactory	NILM	NILM	Negative (NILM)	Candida
P08835	2022	2022-Q2	2022-06	Post-pandemic recovery	Cytology (Pap)	55-59	56	Not performed	Satisfactory	NILM	NILM	Negative (NILM)	
P08836	2022	2022-Q2	2022-06	Post-pandemic recovery	Cytology (Pap)	25-29	28	Not performed	Satisfactory	NILM	NILM	Negative (NILM)	
P08837	2022	2022-Q2	2022-06	Post-pandemic recovery	Cytology (Pap)	35-39	39	Not performed	Satisfactory	NILM	NILM	Negative (NILM)	
P08838	2022	2022-Q2	2022-06	Post-pandemic recovery	Cytology (Pap)	50-54	52	Not performed	Satisfactory	NILM	NILM	Negative (NILM)	
P08839	2022	2022-Q2	2022-06	Post-pandemic recovery	Cytology (Pap)	40-44	44	Not performed	Satisfactory	NILM	NILM	Negative (NILM)	
P08840	2022	2022-Q2	2022-06	Post-pandemic recovery	Cytology (Pap)	45-49	47	Not performed	Satisfactory	NILM	NILM	Negative (NILM)	
P08841	2022	2022-Q2	2022-06	Post-pandemic recovery	Cytology (Pap)	35-39	39	Not performed	Satisfactory	NILM	NILM	Negative (NILM)	Gardnerella
P08842	2022	2022-Q2	2022-06	Post-pandemic recovery	Cytology (Pap)	60-64	62	Not performed	Satisfactory	NILM	NILM	Negative (NILM)	
P08843	2022	2022-Q2	2022-06	Post-pandemic recovery	Cytology (Pap)	60-64	60	Not performed	Satisfactory	NILM	NILM	Negative (NILM)	Other
P08844	2022	2022-Q2	2022-06	Post-pandemic recovery	Cytology (Pap)	35-39	38	Not performed	Satisfactory	NILM	NILM	Negative (NILM)	Candida
P08845	2022	2022-Q2	2022-06	Post-pandemic recovery	Cytology (Pap)	50-54	52	Not performed	Satisfactory	NILM	NILM	Negative (NILM)	
P08846	2022	2022-Q2	2022-06	Post-pandemic recovery	Cytology (Pap)	55-59	57	Not performed	Satisfactory	NILM	NILM	Negative (NILM)	
P08847	2022	2022-Q2	2022-06	Post-pandemic recovery	Cytology (Pap)	35-39	38	Not performed	Unsatisfactory	Unsatisfactory	Unsatisfactory	Unsatisfactory	
P08848	2022	2022-Q2	2022-06	Post-pandemic recovery	Cytology (Pap)	50-54	50	Not performed	Satisfactory	NILM	NILM	Negative (NILM)	
P08849	2022	2022-Q2	2022-06	Post-pandemic recovery	Cytology (Pap)	30-34	33	Not performed	Satisfactory	NILM	NILM	Negative (NILM)	
P08850	2022	2022-Q2	2022-06	Post-pandemic recovery	Cytology (Pap)	40-44	41	Not performed	Satisfactory	Positive	ASC-US	Low-grade / equivocal squamous	
P08851	2022	2022-Q2	2022-06	Post-pandemic recovery	Cytology (Pap)	40-44	44	Not performed	Satisfactory	NILM	NILM	Negative (NILM)	
P08852	2022	2022-Q2	2022-06	Post-pandemic recovery	Cytology (Pap)	55-59	57	Not performed	Satisfactory	NILM	NILM	Negative (NILM)	
P08853	2022	2022-Q2	2022-06	Post-pandemic recovery	Cytology (Pap)	60-64	63	Not performed	Satisfactory	NILM	NILM	Negative (NILM)	
P08854	2022	2022-Q2	2022-06	Post-pandemic recovery	Cytology (Pap)	35-39	36	Not performed	Satisfactory	NILM	NILM	Negative (NILM)	
P08855	2022	2022-Q2	2022-06	Post-pandemic recovery	Cytology (Pap)	30-34	34	Not performed	Satisfactory	NILM	NILM	Negative (NILM)	
P08856	2022	2022-Q2	2022-06	Post-pandemic recovery	Cytology (Pap)	30-34	30	Not performed	Satisfactory	NILM	NILM	Negative (NILM)	
P08857	2022	2022-Q2	2022-06	Post-pandemic recovery	Cytology (Pap)	50-54	50	Not performed	Satisfactory	NILM	NILM	Negative (NILM)	
P08858	2022	2022-Q2	2022-06	Post-pandemic recovery	Cytology (Pap)	50-54	53	Not performed	Satisfactory	NILM	NILM	Negative (NILM)	
P08859	2022	2022-Q2	2022-06	Post-pandemic recovery	Cytology (Pap)	50-54	54	Not performed	Unsatisfactory	Unsatisfactory	Unsatisfactory	Unsatisfactory	
P08860	2022	2022-Q2	2022-06	Post-pandemic recovery	Cytology (Pap)	50-54	54	Not performed	Satisfactory	Positive	LSIL	Low-grade / equivocal squamous	
P08861	2022	2022-Q2	2022-06	Post-pandemic recovery	Cytology (Pap)	30-34	33	Not performed	Satisfactory	NILM	NILM	Negative (NILM)	
P08862	2022	2022-Q2	2022-06	Post-pandemic recovery	Cytology (Pap)	35-39	36	Not performed	Satisfactory	NILM	NILM	Negative (NILM)	
P08863	2022	2022-Q2	2022-06	Post-pandemic recovery	Cytology (Pap)	30-34	31	Not performed	Satisfactory	NILM	NILM	Negative (NILM)	
P08864	2022	2022-Q2	2022-06	Post-pandemic recovery	Cytology (Pap)	55-59	59	Not performed	Satisfactory	NILM	NILM	Negative (NILM)	
P08865	2022	2022-Q2	2022-06	Post-pandemic recovery	Cytology (Pap)	60-64	63	Not performed	Satisfactory	NILM	NILM	Negative (NILM)	
P08866	2022	2022-Q2	2022-06	Post-pandemic recovery	Cytology (Pap)	40-44	42	Not performed	Satisfactory	NILM	NILM	Negative (NILM)	
P08867	2022	2022-Q2	2022-06	Post-pandemic recovery	Cytology (Pap)	35-39	36	Not performed	Satisfactory	Positive	LSIL	Low-grade / equivocal squamous	
P08868	2022	2022-Q2	2022-06	Post-pandemic recovery	Cytology (Pap)	25-29	26	Not performed	Satisfactory	Positive	ASC-US	Low-grade / equivocal squamous	Candida
P08869	2022	2022-Q2	2022-06	Post-pandemic recovery	Cytology (Pap)	35-39	39	Not performed	Satisfactory	NILM	NILM	Negative (NILM)	
P08870	2022	2022-Q2	2022-06	Post-pandemic recovery	Cytology (Pap)	60-64	63	Not performed	Satisfactory	NILM	NILM	Negative (NILM)	
P08871	2022	2022-Q2	2022-06	Post-pandemic recovery	Cytology (Pap)	25-29	26	Not performed	Satisfactory	NILM	NILM	Negative (NILM)	
P08872	2022	2022-Q2	2022-06	Post-pandemic recovery	Cytology (Pap)	50-54	52	Not performed	Satisfactory	NILM	NILM	Negative (NILM)	
P08873	2022	2022-Q2	2022-06	Post-pandemic recovery	Cytology (Pap)	30-34	30	Not performed	Satisfactory	NILM	NILM	Negative (NILM)	
P08874	2022	2022-Q2	2022-06	Post-pandemic recovery	Cytology (Pap)	40-44	43	Not performed	Satisfactory	NILM	NILM	Negative (NILM)	
P08875	2022	2022-Q2	2022-06	Post-pandemic recovery	Cytology (Pap)	40-44	42	Not performed	Satisfactory	NILM	NILM	Negative (NILM)	
P08876	2022	2022-Q2	2022-06	Post-pandemic recovery	Cytology (Pap)	25-29	25	Not performed	Satisfactory	NILM	NILM	Negative (NILM)	
P08877	2022	2022-Q2	2022-06	Post-pandemic recovery	Cytology (Pap)	45-49	45	Not performed	Unsatisfactory	Unsatisfactory	Unsatisfactory	Unsatisfactory	
P08878	2022	2022-Q2	2022-06	Post-pandemic recovery	Cytology (Pap)	25-29	26	Not performed	Unsatisfactory	Unsatisfactory	Unsatisfactory	Unsatisfactory	

patient_id	year	quarter	month	period	primary_test	age_group	age_years	hpv_result	adequacy	result	bethesda	grade	infection
P08879	2022	2022-Q2	2022-06	Post-pandemic recovery	Cytology (Pap)	55-59	55	Not performed	Satisfactory	NILM	NILM	Negative (NILM)	
P08880	2022	2022-Q2	2022-06	Post-pandemic recovery	Cytology (Pap)	30-34	34	Not performed	Satisfactory	NILM	NILM	Negative (NILM)	Trichomonas
P08881	2022	2022-Q2	2022-06	Post-pandemic recovery	Cytology (Pap)	40-44	44	Not performed	Unsatisfactory	Unsatisfactory	Unsatisfactory	Unsatisfactory	
P08882	2022	2022-Q2	2022-06	Post-pandemic recovery	Cytology (Pap)	55-59	57	Not performed	Satisfactory	NILM	NILM	Negative (NILM)	
P08883	2022	2022-Q2	2022-06	Post-pandemic recovery	Cytology (Pap)	55-59	58	Not performed	Satisfactory	NILM	NILM	Negative (NILM)	
P08884	2022	2022-Q2	2022-06	Post-pandemic recovery	Cytology (Pap)	55-59	59	Not performed	Satisfactory	NILM	NILM	Negative (NILM)	
P08885	2022	2022-Q2	2022-06	Post-pandemic recovery	Cytology (Pap)	35-39	39	Not performed	Satisfactory	NILM	NILM	Negative (NILM)	
P08886	2022	2022-Q2	2022-06	Post-pandemic recovery	Cytology (Pap)	30-34	34	Not performed	Satisfactory	Positive	ASC-US	Low-grade / equivocal squamous	
P08887	2022	2022-Q2	2022-06	Post-pandemic recovery	Cytology (Pap)	40-44	41	Not performed	Satisfactory	Positive	LSIL	Low-grade / equivocal squamous	Candida
P08888	2022	2022-Q3	2022-07	Post-pandemic recovery	Cytology (Pap)	45-49	46	Not performed	Satisfactory	NILM	NILM	Negative (NILM)	
P08889	2022	2022-Q3	2022-07	Post-pandemic recovery	Cytology (Pap)	45-49	46	Not performed	Satisfactory	NILM	NILM	Negative (NILM)	
P08890	2022	2022-Q3	2022-07	Post-pandemic recovery	Cytology (Pap)	25-29	29	Not performed	Satisfactory	NILM	NILM	Negative (NILM)	Trichomonas
P08891	2022	2022-Q3	2022-07	Post-pandemic recovery	Cytology (Pap)	25-29	29	Not performed	Satisfactory	NILM	NILM	Negative (NILM)	
P08892	2022	2022-Q3	2022-07	Post-pandemic recovery	Cytology (Pap)	40-44	44	Not performed	Satisfactory	NILM	NILM	Negative (NILM)	
P08893	2022	2022-Q3	2022-07	Post-pandemic recovery	Cytology (Pap)	45-49	48	Not performed	Satisfactory	NILM	NILM	Negative (NILM)	
P08894	2022	2022-Q3	2022-07	Post-pandemic recovery	Cytology (Pap)	40-44	44	Not performed	Satisfactory	NILM	NILM	Negative (NILM)	
P08895	2022	2022-Q3	2022-07	Post-pandemic recovery	Cytology (Pap)	45-49	46	Not performed	Satisfactory	NILM	NILM	Negative (NILM)	
P08896	2022	2022-Q3	2022-07	Post-pandemic recovery	Cytology (Pap)	45-49	48	Not performed	Satisfactory	NILM	NILM	Negative (NILM)	
P08897	2022	2022-Q3	2022-07	Post-pandemic recovery	Cytology (Pap)	35-39	35	Not performed	Satisfactory	NILM	NILM	Negative (NILM)	
P08898	2022	2022-Q3	2022-07	Post-pandemic recovery	Cytology (Pap)	35-39	36	Not performed	Unsatisfactory	Unsatisfactory	Unsatisfactory	Unsatisfactory	
P08899	2022	2022-Q3	2022-07	Post-pandemic recovery	Cytology (Pap)	55-59	59	Not performed	Satisfactory	NILM	NILM	Negative (NILM)	
P08900	2022	2022-Q3	2022-07	Post-pandemic recovery	Cytology (Pap)	25-29	28	Not performed	Satisfactory	NILM	NILM	Negative (NILM)	
P08901	2022	2022-Q3	2022-07	Post-pandemic recovery	Cytology (Pap)	30-34	32	Not performed	Satisfactory	NILM	NILM	Negative (NILM)	
P08902	2022	2022-Q3	2022-07	Post-pandemic recovery	Cytology (Pap)	60-64	64	Not performed	Satisfactory	NILM	NILM	Negative (NILM)	
P08903	2022	2022-Q3	2022-07	Post-pandemic recovery	Cytology (Pap)	45-49	48	Not performed	Satisfactory	NILM	NILM	Negative (NILM)	Candida
P08904	2022	2022-Q3	2022-07	Post-pandemic recovery	Cytology (Pap)	45-49	46	Not performed	Satisfactory	Positive	HSIL	High-grade squamous	
P08905	2022	2022-Q3	2022-07	Post-pandemic recovery	Cytology (Pap)	25-29	29	Not performed	Satisfactory	NILM	NILM	Negative (NILM)	Trichomonas
P08906	2022	2022-Q3	2022-07	Post-pandemic recovery	Cytology (Pap)	60-64	61	Not performed	Satisfactory	NILM	NILM	Negative (NILM)	
P08907	2022	2022-Q3	2022-07	Post-pandemic recovery	Cytology (Pap)	40-44	42	Not performed	Satisfactory	NILM	NILM	Negative (NILM)	
P08908	2022	2022-Q3	2022-07	Post-pandemic recovery	Cytology (Pap)	35-39	35	Not performed	Satisfactory	NILM	NILM	Negative (NILM)	
P08909	2022	2022-Q3	2022-07	Post-pandemic recovery	Cytology (Pap)	45-49	48	Not performed	Satisfactory	NILM	NILM	Negative (NILM)	
P08910	2022	2022-Q3	2022-07	Post-pandemic recovery	Cytology (Pap)	40-44	43	Not performed	Satisfactory	Positive	ASC-US	Low-grade / equivocal squamous	
P08911	2022	2022-Q3	2022-07	Post-pandemic recovery	Cytology (Pap)	40-44	40	Not performed	Satisfactory	NILM	NILM	Negative (NILM)	
P08912	2022	2022-Q3	2022-07	Post-pandemic recovery	Cytology (Pap)	30-34	32	Not performed	Satisfactory	NILM	NILM	Negative (NILM)	Candida
P08913	2022	2022-Q3	2022-07	Post-pandemic recovery	Cytology (Pap)	60-64	61	Not performed	Satisfactory	NILM	NILM	Negative (NILM)	
P08914	2022	2022-Q3	2022-07	Post-pandemic recovery	Cytology (Pap)	50-54	50	Not performed	Satisfactory	NILM	NILM	Negative (NILM)	
P08915	2022	2022-Q3	2022-07	Post-pandemic recovery	Cytology (Pap)	40-44	43	Not performed	Satisfactory	NILM	NILM	Negative (NILM)	
P08916	2022	2022-Q3	2022-07	Post-pandemic recovery	Cytology (Pap)	25-29	29	Not performed	Satisfactory	NILM	NILM	Negative (NILM)	
P08917	2022	2022-Q3	2022-07	Post-pandemic recovery	Cytology (Pap)	55-59	57	Not performed	Satisfactory	NILM	NILM	Negative (NILM)	
P08918	2022	2022-Q3	2022-07	Post-pandemic recovery	Cytology (Pap)	60-64	60	Not performed	Satisfactory	NILM	NILM	Negative (NILM)	
P08919	2022	2022-Q3	2022-07	Post-pandemic recovery	Cytology (Pap)	45-49	47	Not performed	Satisfactory	NILM	NILM	Negative (NILM)	
P08920	2022	2022-Q3	2022-07	Post-pandemic recovery	Cytology (Pap)	35-39	37	Not performed	Unsatisfactory	Unsatisfactory	Unsatisfactory	Unsatisfactory	
P08921	2022	2022-Q3	2022-07	Post-pandemic recovery	Cytology (Pap)	40-44	44	Not performed	Satisfactory	NILM	NILM	Negative (NILM)	
P08922	2022	2022-Q3	2022-07	Post-pandemic recovery	Cytology (Pap)	40-44	43	Not performed	Satisfactory	NILM	NILM	Negative (NILM)	
P08923	2022	2022-Q3	2022-07	Post-pandemic recovery	Cytology (Pap)	35-39	37	Not performed	Satisfactory	Positive	ASC-US	Low-grade / equivocal squamous	
P08924	2022	2022-Q3	2022-07	Post-pandemic recovery	Cytology (Pap)	45-49	47	Not performed	Satisfactory	NILM	NILM	Negative (NILM)	
P08925	2022	2022-Q3	2022-07	Post-pandemic recovery	Cytology (Pap)	35-39	37	Not performed	Satisfactory	NILM	NILM	Negative (NILM)	
P08926	2022	2022-Q3	2022-07	Post-pandemic recovery	Cytology (Pap)	55-59	56	Not performed	Unsatisfactory	Unsatisfactory	Unsatisfactory	Unsatisfactory	
P08927	2022	2022-Q3	2022-07	Post-pandemic recovery	Cytology (Pap)	50-54	53	Not performed	Satisfactory	Positive	LSIL	Low-grade / equivocal squamous	
P08928	2022	2022-Q3	2022-07	Post-pandemic recovery	Cytology (Pap)	25-29	29	Not performed	Satisfactory	NILM	NILM	Negative (NILM)	
P08929	2022	2022-Q3	2022-07	Post-pandemic recovery	Cytology (Pap)	35-39	35	Not performed	Satisfactory	NILM	NILM	Negative (NILM)	
P08930	2022	2022-Q3	2022-07	Post-pandemic recovery	Cytology (Pap)	35-39	39	Not performed	Satisfactory	NILM	NILM	Negative (NILM)	
P08931	2022	2022-Q3	2022-07	Post-pandemic recovery	Cytology (Pap)	45-49	45	Not performed	Satisfactory	NILM	NILM	Negative (NILM)	
P08932	2022	2022-Q3	2022-07	Post-pandemic recovery	Cytology (Pap)	40-44	40	Not performed	Satisfactory	NILM	NILM	Negative (NILM)	
P08933	2022	2022-Q3	2022-07	Post-pandemic recovery	Cytology (Pap)	50-54	54	Not performed	Satisfactory	NILM	NILM	Negative (NILM)	
P08934	2022	2022-Q3	2022-07	Post-pandemic recovery	Cytology (Pap)	45-49	46	Not performed	Satisfactory	NILM	NILM	Negative (NILM)	
P08935	2022	2022-Q3	2022-07	Post-pandemic recovery	Cytology (Pap)	55-59	55	Not performed	Satisfactory	NILM	NILM	Negative (NILM)	
P08936	2022	2022-Q3	2022-07	Post-pandemic recovery	Cytology (Pap)	30-34	32	Not performed	Satisfactory	NILM	NILM	Negative (NILM)	
P08937	2022	2022-Q3	2022-07	Post-pandemic recovery	Cytology (Pap)	35-39	37	Not performed	Satisfactory	NILM	NILM	Negative (NILM)	Gardnerella
P08938	2022	2022-Q3	2022-07	Post-pandemic recovery	Cytology (Pap)	55-59	58	Not performed	Satisfactory	Positive	LSIL	Low-grade / equivocal squamous	
P08939	2022	2022-Q3	2022-07	Post-pandemic recovery	Cytology (Pap)	40-44	42	Not performed	Satisfactory	NILM	NILM	Negative (NILM)	
P08940	2022	2022-Q3	2022-07	Post-pandemic recovery	Cytology (Pap)	40-44	40	Not performed	Satisfactory	NILM	NILM	Negative (NILM)	
P08941	2022	2022-Q3	2022-07	Post-pandemic recovery	Cytology (Pap)	25-29	25	Not performed	Satisfactory	NILM	NILM	Negative (NILM)	
P08942	2022	2022-Q3	2022-07	Post-pandemic recovery	Cytology (Pap)	45-49	48	Not performed	Satisfactory	Positive	ASC-US	Low-grade / equivocal squamous	Candida
P08943	2022	2022-Q3	2022-07	Post-pandemic recovery	Cytology (Pap)	40-44	44	Not performed	Satisfactory	NILM	NILM	Negative (NILM)	
P08944	2022	2022-Q3	2022-07	Post-pandemic recovery	Cytology (Pap)	50-54	51	Not performed	Satisfactory	NILM	NILM	Negative (NILM)	
P08945	2022	2022-Q3	2022-07	Post-pandemic recovery	Cytology (Pap)	40-44	40	Not performed	Satisfactory	NILM	NILM	Negative (NILM)	
P08946	2022	2022-Q3	2022-07	Post-pandemic recovery	Cytology (Pap)	35-39	39	Not performed	Satisfactory	NILM	NILM	Negative (NILM)	
P08947	2022	2022-Q3	2022-07	Post-pandemic recovery	Cytology (Pap)	45-49	48	Not performed	Satisfactory	Positive	ASC-US	Low-grade / equivocal squamous	
P08948	2022	2022-Q3	2022-07	Post-pandemic recovery	Cytology (Pap)	40-44	42	Not performed	Satisfactory	NILM	NILM	Negative (NILM)	
P08949	2022	2022-Q3	2022-07	Post-pandemic recovery	Cytology (Pap)	45-49	45	Not performed	Unsatisfactory	Unsatisfactory	Unsatisfactory	Unsatisfactory	
P08950	2022	2022-Q3	2022-07	Post-pandemic recovery	Cytology (Pap)	40-44	43	Not performed	Satisfactory	NILM	NILM	Negative (NILM)	
P08951	2022	2022-Q3	2022-07	Post-pandemic recovery	Cytology (Pap)	35-39	37	Not performed	Satisfactory	NILM	NILM	Negative (NILM)	
P08952	2022	2022-Q3	2022-07	Post-pandemic recovery	Cytology (Pap)	40-44	41	Not performed	Satisfactory	NILM	NILM	Negative (NILM)	
P08953	2022	2022-Q3	2022-07	Post-pandemic recovery	Cytology (Pap)	35-39	36	Not performed	Satisfactory	NILM	NILM	Negative (NILM)	Other
P08954	2022	2022-Q3	2022-07	Post-pandemic recovery	Cytology (Pap)	35-39	37	Not performed	Satisfactory	NILM	NILM	Negative (NILM)	
P08955	2022	2022-Q3	2022-07	Post-pandemic recovery	Cytology (Pap)	40-44	41	Not performed	Satisfactory	NILM	NILM	Negative (NILM)	
P08956	2022	2022-Q3	2022-07	Post-pandemic recovery	Cytology (Pap)	35-39	36	Not performed	Satisfactory	NILM	NILM	Negative (NILM)	
P08957	2022	2022-Q3	2022-07	Post-pandemic recovery	Cytology (Pap)	40-44	44	Not performed	Satisfactory	Positive	ASC-US	Low-grade / equivocal squamous	
P08958	2022	2022-Q3	2022-07	Post-pandemic recovery	Cytology (Pap)	60-64	64	Not performed	Satisfactory	NILM	NILM	Negative (NILM)	Candida

patient_id	year	quarter	month	period	primary_test	age_group	age_years	hpv_result	adequacy	result	bethesda	grade	infection
P08959	2022	2022-Q3	2022-07	Post-pandemic recovery	Cytology (Pap)	40-44	43	Not performed	Satisfactory	NILM	NILM	Negative (NILM)	
P08960	2022	2022-Q3	2022-07	Post-pandemic recovery	Cytology (Pap)	25-29	29	Not performed	Satisfactory	NILM	NILM	Negative (NILM)	
P08961	2022	2022-Q3	2022-07	Post-pandemic recovery	Cytology (Pap)	25-29	26	Not performed	Satisfactory	NILM	NILM	Negative (NILM)	
P08962	2022	2022-Q3	2022-07	Post-pandemic recovery	Cytology (Pap)	60-64	63	Not performed	Satisfactory	Positive	ASC-US	Low-grade / equivocal squamous	
P08963	2022	2022-Q3	2022-07	Post-pandemic recovery	Cytology (Pap)	60-64	62	Not performed	Satisfactory	NILM	NILM	Negative (NILM)	
P08964	2022	2022-Q3	2022-07	Post-pandemic recovery	Cytology (Pap)	35-39	39	Not performed	Satisfactory	Positive	ASC-US	Low-grade / equivocal squamous	
P08965	2022	2022-Q3	2022-07	Post-pandemic recovery	Cytology (Pap)	55-59	59	Not performed	Satisfactory	NILM	NILM	Negative (NILM)	
P08966	2022	2022-Q3	2022-07	Post-pandemic recovery	Cytology (Pap)	60-64	63	Not performed	Satisfactory	NILM	NILM	Negative (NILM)	
P08967	2022	2022-Q3	2022-07	Post-pandemic recovery	Cytology (Pap)	30-34	31	Not performed	Satisfactory	NILM	NILM	Negative (NILM)	
P08968	2022	2022-Q3	2022-07	Post-pandemic recovery	Cytology (Pap)	50-54	54	Not performed	Satisfactory	Positive	AGC	Glandular	
P08969	2022	2022-Q3	2022-07	Post-pandemic recovery	Cytology (Pap)	50-54	52	Not performed	Satisfactory	NILM	NILM	Negative (NILM)	
P08970	2022	2022-Q3	2022-07	Post-pandemic recovery	Cytology (Pap)	40-44	40	Not performed	Satisfactory	NILM	NILM	Negative (NILM)	
P08971	2022	2022-Q3	2022-07	Post-pandemic recovery	Cytology (Pap)	50-54	51	Not performed	Satisfactory	Positive	LSIL	Low-grade / equivocal squamous	
P08972	2022	2022-Q3	2022-07	Post-pandemic recovery	Cytology (Pap)	25-29	29	Not performed	Satisfactory	NILM	NILM	Negative (NILM)	
P08973	2022	2022-Q3	2022-07	Post-pandemic recovery	Cytology (Pap)	40-44	42	Not performed	Satisfactory	NILM	NILM	Negative (NILM)	
P08974	2022	2022-Q3	2022-07	Post-pandemic recovery	Cytology (Pap)	35-39	35	Not performed	Satisfactory	NILM	NILM	Negative (NILM)	
P08975	2022	2022-Q3	2022-07	Post-pandemic recovery	Cytology (Pap)	45-49	45	Not performed	Satisfactory	NILM	NILM	Negative (NILM)	
P08976	2022	2022-Q3	2022-07	Post-pandemic recovery	Cytology (Pap)	50-54	54	Not performed	Satisfactory	NILM	NILM	Negative (NILM)	
P08977	2022	2022-Q3	2022-07	Post-pandemic recovery	Cytology (Pap)	40-44	40	Not performed	Satisfactory	NILM	NILM	Negative (NILM)	
P08978	2022	2022-Q3	2022-07	Post-pandemic recovery	Cytology (Pap)	55-59	59	Not performed	Satisfactory	Positive	HSIL	High-grade squamous	
P08979	2022	2022-Q3	2022-07	Post-pandemic recovery	Cytology (Pap)	30-34	30	Not performed	Satisfactory	NILM	NILM	Negative (NILM)	
P08980	2022	2022-Q3	2022-07	Post-pandemic recovery	Cytology (Pap)	25-29	27	Not performed	Satisfactory	Positive	ASC-US	Low-grade / equivocal squamous	
P08981	2022	2022-Q3	2022-07	Post-pandemic recovery	Cytology (Pap)	30-34	30	Not performed	Satisfactory	NILM	NILM	Negative (NILM)	
P08982	2022	2022-Q3	2022-07	Post-pandemic recovery	Cytology (Pap)	55-59	59	Not performed	Satisfactory	NILM	NILM	Negative (NILM)	
P08983	2022	2022-Q3	2022-07	Post-pandemic recovery	Cytology (Pap)	35-39	36	Not performed	Satisfactory	NILM	NILM	Negative (NILM)	
P08984	2022	2022-Q3	2022-07	Post-pandemic recovery	Cytology (Pap)	25-29	29	Not performed	Satisfactory	NILM	NILM	Negative (NILM)	
P08985	2022	2022-Q3	2022-07	Post-pandemic recovery	Cytology (Pap)	45-49	48	Not performed	Satisfactory	NILM	NILM	Negative (NILM)	
P08986	2022	2022-Q3	2022-07	Post-pandemic recovery	Cytology (Pap)	55-59	57	Not performed	Satisfactory	Positive	ASC-US	Low-grade / equivocal squamous	Trichomonas
P08987	2022	2022-Q3	2022-07	Post-pandemic recovery	Cytology (Pap)	40-44	41	Not performed	Satisfactory	Positive	LSIL	Low-grade / equivocal squamous	
P08988	2022	2022-Q3	2022-07	Post-pandemic recovery	Cytology (Pap)	25-29	29	Not performed	Satisfactory	Positive	LSIL	Low-grade / equivocal squamous	
P08989	2022	2022-Q3	2022-07	Post-pandemic recovery	Cytology (Pap)	50-54	50	Not performed	Satisfactory	NILM	NILM	Negative (NILM)	
P08990	2022	2022-Q3	2022-07	Post-pandemic recovery	Cytology (Pap)	60-64	62	Not performed	Satisfactory	NILM	NILM	Negative (NILM)	
P08991	2022	2022-Q3	2022-07	Post-pandemic recovery	Cytology (Pap)	40-44	41	Not performed	Satisfactory	NILM	NILM	Negative (NILM)	Candida
P08992	2022	2022-Q3	2022-07	Post-pandemic recovery	Cytology (Pap)	50-54	54	Not performed	Satisfactory	NILM	NILM	Negative (NILM)	
P08993	2022	2022-Q3	2022-07	Post-pandemic recovery	Cytology (Pap)	30-34	33	Not performed	Satisfactory	NILM	NILM	Negative (NILM)	
P08994	2022	2022-Q3	2022-07	Post-pandemic recovery	Cytology (Pap)	30-34	32	Not performed	Satisfactory	NILM	NILM	Negative (NILM)	
P08995	2022	2022-Q3	2022-07	Post-pandemic recovery	Cytology (Pap)	25-29	29	Not performed	Satisfactory	NILM	NILM	Negative (NILM)	
P08996	2022	2022-Q3	2022-07	Post-pandemic recovery	Cytology (Pap)	50-54	53	Not performed	Satisfactory	NILM	NILM	Negative (NILM)	
P08997	2022	2022-Q3	2022-07	Post-pandemic recovery	Cytology (Pap)	40-44	43	Not performed	Satisfactory	NILM	NILM	Negative (NILM)	
P08998	2022	2022-Q3	2022-07	Post-pandemic recovery	Cytology (Pap)	50-54	51	Not performed	Satisfactory	NILM	NILM	Negative (NILM)	
P08999	2022	2022-Q3	2022-07	Post-pandemic recovery	Cytology (Pap)	60-64	60	Not performed	Satisfactory	NILM	NILM	Negative (NILM)	
P09000	2022	2022-Q3	2022-07	Post-pandemic recovery	Cytology (Pap)	30-34	32	Not performed	Satisfactory	NILM	NILM	Negative (NILM)	
P09001	2022	2022-Q3	2022-07	Post-pandemic recovery	Cytology (Pap)	45-49	45	Not performed	Satisfactory	NILM	NILM	Negative (NILM)	
P09002	2022	2022-Q3	2022-07	Post-pandemic recovery	Cytology (Pap)	45-49	49	Not performed	Satisfactory	NILM	NILM	Negative (NILM)	
P09003	2022	2022-Q3	2022-07	Post-pandemic recovery	Cytology (Pap)	45-49	45	Not performed	Satisfactory	NILM	NILM	Negative (NILM)	
P09004	2022	2022-Q3	2022-07	Post-pandemic recovery	Cytology (Pap)	30-34	34	Not performed	Satisfactory	Positive	ASC-US	Low-grade / equivocal squamous	
P09005	2022	2022-Q3	2022-07	Post-pandemic recovery	Cytology (Pap)	55-59	56	Not performed	Satisfactory	Positive	ASC-US	Low-grade / equivocal squamous	
P09006	2022	2022-Q3	2022-07	Post-pandemic recovery	Cytology (Pap)	30-34	33	Not performed	Satisfactory	NILM	NILM	Negative (NILM)	
P09007	2022	2022-Q3	2022-07	Post-pandemic recovery	Cytology (Pap)	60-64	61	Not performed	Satisfactory	NILM	NILM	Negative (NILM)	
P09008	2022	2022-Q3	2022-07	Post-pandemic recovery	Cytology (Pap)	35-39	37	Not performed	Satisfactory	NILM	NILM	Negative (NILM)	
P09009	2022	2022-Q3	2022-07	Post-pandemic recovery	Cytology (Pap)	60-64	62	Not performed	Satisfactory	NILM	NILM	Negative (NILM)	
P09010	2022	2022-Q3	2022-07	Post-pandemic recovery	Cytology (Pap)	40-44	41	Not performed	Satisfactory	NILM	NILM	Negative (NILM)	
P09011	2022	2022-Q3	2022-07	Post-pandemic recovery	Cytology (Pap)	40-44	41	Not performed	Satisfactory	NILM	NILM	Negative (NILM)	
P09012	2022	2022-Q3	2022-07	Post-pandemic recovery	Cytology (Pap)	50-54	50	Not performed	Satisfactory	NILM	NILM	Negative (NILM)	
P09013	2022	2022-Q3	2022-07	Post-pandemic recovery	Cytology (Pap)	55-59	58	Not performed	Satisfactory	Positive	ASC-US	Low-grade / equivocal squamous	
P09014	2022	2022-Q3	2022-07	Post-pandemic recovery	Cytology (Pap)	25-29	25	Not performed	Satisfactory	NILM	NILM	Negative (NILM)	
P09015	2022	2022-Q3	2022-07	Post-pandemic recovery	Cytology (Pap)	45-49	46	Not performed	Satisfactory	Positive	ASC-US	Low-grade / equivocal squamous	
P09016	2022	2022-Q3	2022-07	Post-pandemic recovery	Cytology (Pap)	40-44	42	Not performed	Satisfactory	NILM	NILM	Negative (NILM)	
P09017	2022	2022-Q3	2022-07	Post-pandemic recovery	Cytology (Pap)	40-44	43	Not performed	Satisfactory	NILM	NILM	Negative (NILM)	
P09018	2022	2022-Q3	2022-07	Post-pandemic recovery	Cytology (Pap)	60-64	64	Not performed	Satisfactory	NILM	NILM	Negative (NILM)	Candida
P09019	2022	2022-Q3	2022-07	Post-pandemic recovery	Cytology (Pap)	30-34	34	Not performed	Satisfactory	NILM	NILM	Negative (NILM)	
P09020	2022	2022-Q3	2022-07	Post-pandemic recovery	Cytology (Pap)	50-54	54	Not performed	Satisfactory	Positive	ASC-US	Low-grade / equivocal squamous	
P09021	2022	2022-Q3	2022-07	Post-pandemic recovery	Cytology (Pap)	25-29	27	Not performed	Satisfactory	NILM	NILM	Negative (NILM)	
P09022	2022	2022-Q3	2022-07	Post-pandemic recovery	Cytology (Pap)	40-44	44	Not performed	Satisfactory	NILM	NILM	Negative (NILM)	
P09023	2022	2022-Q3	2022-07	Post-pandemic recovery	Cytology (Pap)	30-34	32	Not performed	Satisfactory	NILM	NILM	Negative (NILM)	
P09024	2022	2022-Q3	2022-07	Post-pandemic recovery	Cytology (Pap)	55-59	55	Not performed	Satisfactory	NILM	NILM	Negative (NILM)	
P09025	2022	2022-Q3	2022-07	Post-pandemic recovery	Cytology (Pap)	40-44	40	Not performed	Satisfactory	NILM	NILM	Negative (NILM)	
P09026	2022	2022-Q3	2022-07	Post-pandemic recovery	Cytology (Pap)	40-44	44	Not performed	Satisfactory	NILM	NILM	Negative (NILM)	
P09027	2022	2022-Q3	2022-07	Post-pandemic recovery	Cytology (Pap)	45-49	49	Not performed	Satisfactory	Positive	ASC-H	High-grade squamous	
P09028	2022	2022-Q3	2022-07	Post-pandemic recovery	Cytology (Pap)	30-34	33	Not performed	Satisfactory	NILM	NILM	Negative (NILM)	Candida
P09029	2022	2022-Q3	2022-07	Post-pandemic recovery	Cytology (Pap)	30-34	30	Not performed	Satisfactory	NILM	NILM	Negative (NILM)	
P09030	2022	2022-Q3	2022-07	Post-pandemic recovery	Cytology (Pap)	45-49	46	Not performed	Satisfactory	NILM	NILM	Negative (NILM)	
P09031	2022	2022-Q3	2022-07	Post-pandemic recovery	Cytology (Pap)	50-54	53	Not performed	Satisfactory	NILM	NILM	Negative (NILM)	
P09032	2022	2022-Q3	2022-07	Post-pandemic recovery	Cytology (Pap)	45-49	48	Not performed	Satisfactory	NILM	NILM	Negative (NILM)	
P09033	2022	2022-Q3	2022-07	Post-pandemic recovery	Cytology (Pap)	45-49	45	Not performed	Satisfactory	NILM	NILM	Negative (NILM)	Trichomonas
P09034	2022	2022-Q3	2022-07	Post-pandemic recovery	Cytology (Pap)	40-44	42	Not performed	Satisfactory	NILM	NILM	Negative (NILM)	
P09035	2022	2022-Q3	2022-08	Post-pandemic recovery	Cytology (Pap)	35-39	37	Not performed	Satisfactory	NILM	NILM	Negative (NILM)	
P09036	2022	2022-Q3	2022-08	Post-pandemic recovery	Cytology (Pap)	40-44	44	Not performed	Satisfactory	Positive	LSIL	Low-grade / equivocal squamous	
P09037	2022	2022-Q3	2022-08	Post-pandemic recovery	Cytology (Pap)	35-39	39	Not performed	Unsatisfactory	Unsatisfactory	Unsatisfactory	Unsatisfactory	
P09038	2022	2022-Q3	2022-08	Post-pandemic recovery	Cytology (Pap)	50-54	51	Not performed	Satisfactory	NILM	NILM	Negative (NILM)	

patient_id	year	quarter	month	period	primary_test	age_group	age_years	hpv_result	adequacy	result	bethesda	grade	infection
P09039	2022	2022-Q3	2022-08	Post-pandemic recovery	Cytology (Pap)	45-49	47	Not performed	Satisfactory	NILM	NILM	Negative (NILM)	Candida
P09040	2022	2022-Q3	2022-08	Post-pandemic recovery	Cytology (Pap)	30-34	33	Not performed	Satisfactory	NILM	NILM	Negative (NILM)	
P09041	2022	2022-Q3	2022-08	Post-pandemic recovery	Cytology (Pap)	55-59	58	Not performed	Satisfactory	Positive	ASC-US	Low-grade / equivocal squamous	
P09042	2022	2022-Q3	2022-08	Post-pandemic recovery	Cytology (Pap)	40-44	41	Not performed	Satisfactory	NILM	NILM	Negative (NILM)	Other
P09043	2022	2022-Q3	2022-08	Post-pandemic recovery	Cytology (Pap)	40-44	44	Not performed	Satisfactory	NILM	NILM	Negative (NILM)	
P09044	2022	2022-Q3	2022-08	Post-pandemic recovery	Cytology (Pap)	35-39	37	Not performed	Satisfactory	NILM	NILM	Negative (NILM)	
P09045	2022	2022-Q3	2022-08	Post-pandemic recovery	Cytology (Pap)	40-44	43	Not performed	Satisfactory	Positive	HSIL	High-grade squamous	
P09046	2022	2022-Q3	2022-08	Post-pandemic recovery	Cytology (Pap)	35-39	38	Not performed	Satisfactory	NILM	NILM	Negative (NILM)	
P09047	2022	2022-Q3	2022-08	Post-pandemic recovery	Cytology (Pap)	40-44	40	Not performed	Satisfactory	NILM	NILM	Negative (NILM)	
P09048	2022	2022-Q3	2022-08	Post-pandemic recovery	Cytology (Pap)	35-39	37	Not performed	Satisfactory	NILM	NILM	Negative (NILM)	
P09049	2022	2022-Q3	2022-08	Post-pandemic recovery	Cytology (Pap)	40-44	40	Not performed	Satisfactory	NILM	NILM	Negative (NILM)	
P09050	2022	2022-Q3	2022-08	Post-pandemic recovery	Cytology (Pap)	60-64	61	Not performed	Satisfactory	Positive	HSIL	High-grade squamous	
P09051	2022	2022-Q3	2022-08	Post-pandemic recovery	Cytology (Pap)	50-54	54	Not performed	Unsatisfactory	Unsatisfactory	Unsatisfactory	Unsatisfactory	
P09052	2022	2022-Q3	2022-08	Post-pandemic recovery	Cytology (Pap)	60-64	63	Not performed	Satisfactory	Positive	ASC-US	Low-grade / equivocal squamous	
P09053	2022	2022-Q3	2022-08	Post-pandemic recovery	Cytology (Pap)	35-39	38	Not performed	Satisfactory	NILM	NILM	Negative (NILM)	
P09054	2022	2022-Q3	2022-08	Post-pandemic recovery	Cytology (Pap)	30-34	34	Not performed	Satisfactory	NILM	NILM	Negative (NILM)	Candida
P09055	2022	2022-Q3	2022-08	Post-pandemic recovery	Cytology (Pap)	55-59	56	Not performed	Satisfactory	NILM	NILM	Negative (NILM)	
P09056	2022	2022-Q3	2022-08	Post-pandemic recovery	Cytology (Pap)	50-54	52	Not performed	Satisfactory	NILM	NILM	Negative (NILM)	
P09057	2022	2022-Q3	2022-08	Post-pandemic recovery	Cytology (Pap)	45-49	49	Not performed	Unsatisfactory	Unsatisfactory	Unsatisfactory	Unsatisfactory	
P09058	2022	2022-Q3	2022-08	Post-pandemic recovery	Cytology (Pap)	55-59	56	Not performed	Satisfactory	NILM	NILM	Negative (NILM)	
P09059	2022	2022-Q3	2022-08	Post-pandemic recovery	Cytology (Pap)	45-49	47	Not performed	Satisfactory	NILM	NILM	Negative (NILM)	
P09060	2022	2022-Q3	2022-08	Post-pandemic recovery	Cytology (Pap)	35-39	35	Not performed	Satisfactory	NILM	NILM	Negative (NILM)	
P09061	2022	2022-Q3	2022-08	Post-pandemic recovery	Cytology (Pap)	50-54	53	Not performed	Satisfactory	NILM	NILM	Negative (NILM)	Candida
P09062	2022	2022-Q3	2022-08	Post-pandemic recovery	Cytology (Pap)	35-39	38	Not performed	Satisfactory	Positive	ASC-US	Low-grade / equivocal squamous	
P09063	2022	2022-Q3	2022-08	Post-pandemic recovery	Cytology (Pap)	35-39	37	Not performed	Satisfactory	NILM	NILM	Negative (NILM)	
P09064	2022	2022-Q3	2022-08	Post-pandemic recovery	Cytology (Pap)	55-59	59	Not performed	Satisfactory	NILM	NILM	Negative (NILM)	
P09065	2022	2022-Q3	2022-08	Post-pandemic recovery	Cytology (Pap)	30-34	30	Not performed	Satisfactory	NILM	NILM	Negative (NILM)	
P09066	2022	2022-Q3	2022-08	Post-pandemic recovery	Cytology (Pap)	45-49	48	Not performed	Satisfactory	NILM	NILM	Negative (NILM)	
P09067	2022	2022-Q3	2022-08	Post-pandemic recovery	Cytology (Pap)	55-59	59	Not performed	Satisfactory	NILM	NILM	Negative (NILM)	
P09068	2022	2022-Q3	2022-08	Post-pandemic recovery	Cytology (Pap)	60-64	61	Not performed	Satisfactory	NILM	NILM	Negative (NILM)	
P09069	2022	2022-Q3	2022-08	Post-pandemic recovery	Cytology (Pap)	30-34	31	Not performed	Satisfactory	NILM	NILM	Negative (NILM)	
P09070	2022	2022-Q3	2022-08	Post-pandemic recovery	Cytology (Pap)	45-49	45	Not performed	Satisfactory	NILM	NILM	Negative (NILM)	
P09071	2022	2022-Q3	2022-08	Post-pandemic recovery	Cytology (Pap)	25-29	26	Not performed	Satisfactory	NILM	NILM	Negative (NILM)	
P09072	2022	2022-Q3	2022-08	Post-pandemic recovery	Cytology (Pap)	45-49	46	Not performed	Satisfactory	NILM	NILM	Negative (NILM)	
P09073	2022	2022-Q3	2022-08	Post-pandemic recovery	Cytology (Pap)	55-59	55	Not performed	Satisfactory	NILM	NILM	Negative (NILM)	Gardnerella
P09074	2022	2022-Q3	2022-08	Post-pandemic recovery	Cytology (Pap)	60-64	64	Not performed	Satisfactory	NILM	NILM	Negative (NILM)	
P09075	2022	2022-Q3	2022-08	Post-pandemic recovery	Cytology (Pap)	55-59	56	Not performed	Satisfactory	NILM	NILM	Negative (NILM)	
P09076	2022	2022-Q3	2022-08	Post-pandemic recovery	Cytology (Pap)	40-44	42	Not performed	Satisfactory	NILM	NILM	Negative (NILM)	
P09077	2022	2022-Q3	2022-08	Post-pandemic recovery	Cytology (Pap)	45-49	47	Not performed	Satisfactory	NILM	NILM	Negative (NILM)	
P09078	2022	2022-Q3	2022-08	Post-pandemic recovery	Cytology (Pap)	30-34	33	Not performed	Satisfactory	NILM	NILM	Negative (NILM)	
P09079	2022	2022-Q3	2022-08	Post-pandemic recovery	Cytology (Pap)	35-39	38	Not performed	Satisfactory	NILM	NILM	Negative (NILM)	
P09080	2022	2022-Q3	2022-08	Post-pandemic recovery	Cytology (Pap)	45-49	49	Not performed	Satisfactory	NILM	NILM	Negative (NILM)	
P09081	2022	2022-Q3	2022-08	Post-pandemic recovery	Cytology (Pap)	50-54	54	Not performed	Satisfactory	NILM	NILM	Negative (NILM)	
P09082	2022	2022-Q3	2022-08	Post-pandemic recovery	Cytology (Pap)	35-39	35	Not performed	Satisfactory	NILM	NILM	Negative (NILM)	Candida
P09083	2022	2022-Q3	2022-08	Post-pandemic recovery	Cytology (Pap)	55-59	56	Not performed	Satisfactory	NILM	NILM	Negative (NILM)	
P09084	2022	2022-Q3	2022-08	Post-pandemic recovery	Cytology (Pap)	50-54	51	Not performed	Satisfactory	NILM	NILM	Negative (NILM)	Gardnerella
P09085	2022	2022-Q3	2022-08	Post-pandemic recovery	Cytology (Pap)	40-44	43	Not performed	Satisfactory	NILM	NILM	Negative (NILM)	
P09086	2022	2022-Q3	2022-08	Post-pandemic recovery	Cytology (Pap)	40-44	43	Not performed	Satisfactory	Positive	ASC-US	Low-grade / equivocal squamous	
P09087	2022	2022-Q3	2022-08	Post-pandemic recovery	Cytology (Pap)	45-49	45	Not performed	Satisfactory	Positive	HSIL	High-grade squamous	
P09088	2022	2022-Q3	2022-08	Post-pandemic recovery	Cytology (Pap)	45-49	49	Not performed	Satisfactory	Positive	LSIL	Low-grade / equivocal squamous	
P09089	2022	2022-Q3	2022-08	Post-pandemic recovery	Cytology (Pap)	30-34	32	Not performed	Satisfactory	NILM	NILM	Negative (NILM)	
P09090	2022	2022-Q3	2022-08	Post-pandemic recovery	Cytology (Pap)	50-54	51	Not performed	Satisfactory	Positive	LSIL	Low-grade / equivocal squamous	
P09091	2022	2022-Q3	2022-08	Post-pandemic recovery	Cytology (Pap)	40-44	40	Not performed	Satisfactory	NILM	NILM	Negative (NILM)	
P09092	2022	2022-Q3	2022-08	Post-pandemic recovery	Cytology (Pap)	30-34	33	Not performed	Satisfactory	NILM	NILM	Negative (NILM)	
P09093	2022	2022-Q3	2022-08	Post-pandemic recovery	Cytology (Pap)	40-44	41	Not performed	Satisfactory	Positive	ASC-US	Low-grade / equivocal squamous	
P09094	2022	2022-Q3	2022-08	Post-pandemic recovery	Cytology (Pap)	40-44	41	Not performed	Satisfactory	NILM	NILM	Negative (NILM)	
P09095	2022	2022-Q3	2022-08	Post-pandemic recovery	Cytology (Pap)	45-49	45	Not performed	Satisfactory	NILM	NILM	Negative (NILM)	Candida
P09096	2022	2022-Q3	2022-08	Post-pandemic recovery	Cytology (Pap)	30-34	34	Not performed	Satisfactory	NILM	NILM	Negative (NILM)	
P09097	2022	2022-Q3	2022-08	Post-pandemic recovery	Cytology (Pap)	45-49	45	Not performed	Satisfactory	NILM	NILM	Negative (NILM)	
P09098	2022	2022-Q3	2022-08	Post-pandemic recovery	Cytology (Pap)	55-59	55	Not performed	Satisfactory	NILM	NILM	Negative (NILM)	
P09099	2022	2022-Q3	2022-08	Post-pandemic recovery	Cytology (Pap)	45-49	45	Not performed	Satisfactory	NILM	NILM	Negative (NILM)	
P09100	2022	2022-Q3	2022-08	Post-pandemic recovery	Cytology (Pap)	25-29	25	Not performed	Satisfactory	NILM	NILM	Negative (NILM)	
P09101	2022	2022-Q3	2022-08	Post-pandemic recovery	Cytology (Pap)	40-44	41	Not performed	Unsatisfactory	Unsatisfactory	Unsatisfactory	Unsatisfactory	
P09102	2022	2022-Q3	2022-08	Post-pandemic recovery	Cytology (Pap)	25-29	25	Not performed	Satisfactory	Positive	LSIL	Low-grade / equivocal squamous	
P09103	2022	2022-Q3	2022-08	Post-pandemic recovery	Cytology (Pap)	50-54	50	Not performed	Satisfactory	Positive	ASC-US	Low-grade / equivocal squamous	
P09104	2022	2022-Q3	2022-08	Post-pandemic recovery	Cytology (Pap)	60-64	64	Not performed	Satisfactory	NILM	NILM	Negative (NILM)	
P09105	2022	2022-Q3	2022-08	Post-pandemic recovery	Cytology (Pap)	40-44	43	Not performed	Satisfactory	NILM	NILM	Negative (NILM)	
P09106	2022	2022-Q3	2022-08	Post-pandemic recovery	Cytology (Pap)	40-44	40	Not performed	Satisfactory	NILM	NILM	Negative (NILM)	
P09107	2022	2022-Q3	2022-08	Post-pandemic recovery	Cytology (Pap)	30-34	32	Not performed	Satisfactory	NILM	NILM	Negative (NILM)	
P09108	2022	2022-Q3	2022-08	Post-pandemic recovery	Cytology (Pap)	40-44	43	Not performed	Satisfactory	NILM	NILM	Negative (NILM)	
P09109	2022	2022-Q3	2022-08	Post-pandemic recovery	Cytology (Pap)	35-39	39	Not performed	Satisfactory	NILM	NILM	Negative (NILM)	
P09110	2022	2022-Q3	2022-08	Post-pandemic recovery	Cytology (Pap)	45-49	47	Not performed	Satisfactory	Positive	ASC-US	Low-grade / equivocal squamous	
P09111	2022	2022-Q3	2022-08	Post-pandemic recovery	Cytology (Pap)	50-54	53	Not performed	Satisfactory	NILM	NILM	Negative (NILM)	
P09112	2022	2022-Q3	2022-08	Post-pandemic recovery	Cytology (Pap)	30-34	31	Not performed	Satisfactory	NILM	NILM	Negative (NILM)	
P09113	2022	2022-Q3	2022-08	Post-pandemic recovery	Cytology (Pap)	40-44	42	Not performed	Satisfactory	NILM	NILM	Negative (NILM)	
P09114	2022	2022-Q3	2022-08	Post-pandemic recovery	Cytology (Pap)	45-49	46	Not performed	Satisfactory	Positive	HSIL	High-grade squamous	
P09115	2022	2022-Q3	2022-08	Post-pandemic recovery	Cytology (Pap)	25-29	27	Not performed	Satisfactory	NILM	NILM	Negative (NILM)	
P09116	2022	2022-Q3	2022-08	Post-pandemic recovery	Cytology (Pap)	35-39	36	Not performed	Satisfactory	NILM	NILM	Negative (NILM)	
P09117	2022	2022-Q3	2022-08	Post-pandemic recovery	Cytology (Pap)	40-44	41	Not performed	Satisfactory	NILM	NILM	Negative (NILM)	Gardnerella
P09118	2022	2022-Q3	2022-08	Post-pandemic recovery	Cytology (Pap)	30-34	32	Not performed	Satisfactory	NILM	NILM	Negative (NILM)	

patient_id	year	quarter	month	period	primary_test	age_group	age_years	hpv_result	adequacy	result	bethesda	grade	infection
P09119	2022	2022-Q3	2022-08	Post-pandemic recovery	Cytology (Pap)	35-39	36	Not performed	Satisfactory	NILM	NILM	Negative (NILM)	
P09120	2022	2022-Q3	2022-08	Post-pandemic recovery	Cytology (Pap)	50-54	53	Not performed	Satisfactory	NILM	NILM	Negative (NILM)	
P09121	2022	2022-Q3	2022-08	Post-pandemic recovery	Cytology (Pap)	45-49	48	Not performed	Satisfactory	NILM	NILM	Negative (NILM)	
P09122	2022	2022-Q3	2022-08	Post-pandemic recovery	Cytology (Pap)	40-44	44	Not performed	Satisfactory	NILM	NILM	Negative (NILM)	Candida
P09123	2022	2022-Q3	2022-08	Post-pandemic recovery	Cytology (Pap)	40-44	43	Not performed	Satisfactory	NILM	NILM	Negative (NILM)	
P09124	2022	2022-Q3	2022-08	Post-pandemic recovery	Cytology (Pap)	30-34	32	Not performed	Satisfactory	NILM	NILM	Negative (NILM)	
P09125	2022	2022-Q3	2022-08	Post-pandemic recovery	Cytology (Pap)	35-39	35	Not performed	Satisfactory	Positive	ASC-US	Low-grade / equivocal squamous	
P09126	2022	2022-Q3	2022-08	Post-pandemic recovery	Cytology (Pap)	25-29	25	Not performed	Satisfactory	NILM	NILM	Negative (NILM)	
P09127	2022	2022-Q3	2022-08	Post-pandemic recovery	Cytology (Pap)	60-64	60	Not performed	Unsatisfactory	Unsatisfactory	Unsatisfactory	Unsatisfactory	
P09128	2022	2022-Q3	2022-08	Post-pandemic recovery	Cytology (Pap)	55-59	57	Not performed	Satisfactory	NILM	NILM	Negative (NILM)	
P09129	2022	2022-Q3	2022-08	Post-pandemic recovery	Cytology (Pap)	40-44	41	Not performed	Satisfactory	NILM	NILM	Negative (NILM)	
P09130	2022	2022-Q3	2022-08	Post-pandemic recovery	Cytology (Pap)	45-49	49	Not performed	Satisfactory	NILM	NILM	Negative (NILM)	
P09131	2022	2022-Q3	2022-08	Post-pandemic recovery	Cytology (Pap)	40-44	42	Not performed	Satisfactory	NILM	NILM	Negative (NILM)	
P09132	2022	2022-Q3	2022-08	Post-pandemic recovery	Cytology (Pap)	40-44	40	Not performed	Satisfactory	NILM	NILM	Negative (NILM)	
P09133	2022	2022-Q3	2022-08	Post-pandemic recovery	Cytology (Pap)	40-44	40	Not performed	Satisfactory	Positive	ASC-US	Low-grade / equivocal squamous	
P09134	2022	2022-Q3	2022-08	Post-pandemic recovery	Cytology (Pap)	45-49	45	Not performed	Satisfactory	NILM	NILM	Negative (NILM)	
P09135	2022	2022-Q3	2022-08	Post-pandemic recovery	Cytology (Pap)	30-34	31	Not performed	Satisfactory	NILM	NILM	Negative (NILM)	
P09136	2022	2022-Q3	2022-08	Post-pandemic recovery	Cytology (Pap)	40-44	42	Not performed	Satisfactory	NILM	NILM	Negative (NILM)	
P09137	2022	2022-Q3	2022-08	Post-pandemic recovery	Cytology (Pap)	35-39	39	Not performed	Satisfactory	Positive	LSIL	Low-grade / equivocal squamous	
P09138	2022	2022-Q3	2022-08	Post-pandemic recovery	Cytology (Pap)	55-59	59	Not performed	Satisfactory	Positive	LSIL	Low-grade / equivocal squamous	
P09139	2022	2022-Q3	2022-08	Post-pandemic recovery	Cytology (Pap)	50-54	51	Not performed	Satisfactory	NILM	NILM	Negative (NILM)	
P09140	2022	2022-Q3	2022-08	Post-pandemic recovery	Cytology (Pap)	55-59	58	Not performed	Satisfactory	NILM	NILM	Negative (NILM)	
P09141	2022	2022-Q3	2022-08	Post-pandemic recovery	Cytology (Pap)	40-44	41	Not performed	Satisfactory	NILM	NILM	Negative (NILM)	Trichomonas
P09142	2022	2022-Q3	2022-08	Post-pandemic recovery	Cytology (Pap)	45-49	48	Not performed	Satisfactory	NILM	NILM	Negative (NILM)	Gardnerella
P09143	2022	2022-Q3	2022-08	Post-pandemic recovery	Cytology (Pap)	35-39	39	Not performed	Satisfactory	NILM	NILM	Negative (NILM)	Trichomonas
P09144	2022	2022-Q3	2022-08	Post-pandemic recovery	Cytology (Pap)	35-39	35	Not performed	Satisfactory	NILM	NILM	Negative (NILM)	
P09145	2022	2022-Q3	2022-08	Post-pandemic recovery	Cytology (Pap)	40-44	42	Not performed	Satisfactory	NILM	NILM	Negative (NILM)	
P09146	2022	2022-Q3	2022-08	Post-pandemic recovery	Cytology (Pap)	30-34	34	Not performed	Satisfactory	NILM	NILM	Negative (NILM)	
P09147	2022	2022-Q3	2022-08	Post-pandemic recovery	Cytology (Pap)	55-59	56	Not performed	Satisfactory	NILM	NILM	Negative (NILM)	
P09148	2022	2022-Q3	2022-08	Post-pandemic recovery	Cytology (Pap)	45-49	48	Not performed	Satisfactory	NILM	NILM	Negative (NILM)	
P09149	2022	2022-Q3	2022-08	Post-pandemic recovery	Cytology (Pap)	35-39	37	Not performed	Satisfactory	NILM	NILM	Negative (NILM)	
P09150	2022	2022-Q3	2022-08	Post-pandemic recovery	Cytology (Pap)	50-54	51	Not performed	Satisfactory	NILM	NILM	Negative (NILM)	
P09151	2022	2022-Q3	2022-08	Post-pandemic recovery	Cytology (Pap)	35-39	36	Not performed	Satisfactory	Positive	HSIL	High-grade squamous	
P09152	2022	2022-Q3	2022-08	Post-pandemic recovery	Cytology (Pap)	55-59	57	Not performed	Satisfactory	NILM	NILM	Negative (NILM)	
P09153	2022	2022-Q3	2022-08	Post-pandemic recovery	Cytology (Pap)	30-34	33	Not performed	Satisfactory	NILM	NILM	Negative (NILM)	
P09154	2022	2022-Q3	2022-08	Post-pandemic recovery	Cytology (Pap)	40-44	44	Not performed	Satisfactory	NILM	NILM	Negative (NILM)	
P09155	2022	2022-Q3	2022-08	Post-pandemic recovery	Cytology (Pap)	45-49	46	Not performed	Satisfactory	NILM	NILM	Negative (NILM)	
P09156	2022	2022-Q3	2022-08	Post-pandemic recovery	Cytology (Pap)	25-29	29	Not performed	Satisfactory	NILM	NILM	Negative (NILM)	
P09157	2022	2022-Q3	2022-08	Post-pandemic recovery	Cytology (Pap)	40-44	42	Not performed	Satisfactory	NILM	NILM	Negative (NILM)	
P09158	2022	2022-Q3	2022-08	Post-pandemic recovery	Cytology (Pap)	25-29	25	Not performed	Satisfactory	NILM	NILM	Negative (NILM)	
P09159	2022	2022-Q3	2022-08	Post-pandemic recovery	Cytology (Pap)	35-39	39	Not performed	Satisfactory	NILM	NILM	Negative (NILM)	
P09160	2022	2022-Q3	2022-08	Post-pandemic recovery	Cytology (Pap)	35-39	39	Not performed	Satisfactory	NILM	NILM	Negative (NILM)	
P09161	2022	2022-Q3	2022-08	Post-pandemic recovery	Cytology (Pap)	45-49	48	Not performed	Satisfactory	Positive	ASC-US	Low-grade / equivocal squamous	
P09162	2022	2022-Q3	2022-08	Post-pandemic recovery	Cytology (Pap)	40-44	44	Not performed	Satisfactory	NILM	NILM	Negative (NILM)	
P09163	2022	2022-Q3	2022-08	Post-pandemic recovery	Cytology (Pap)	40-44	42	Not performed	Satisfactory	NILM	NILM	Negative (NILM)	
P09164	2022	2022-Q3	2022-08	Post-pandemic recovery	Cytology (Pap)	25-29	27	Not performed	Satisfactory	NILM	NILM	Negative (NILM)	
P09165	2022	2022-Q3	2022-08	Post-pandemic recovery	Cytology (Pap)	35-39	38	Not performed	Satisfactory	Positive	ASC-US	Low-grade / equivocal squamous	
P09166	2022	2022-Q3	2022-08	Post-pandemic recovery	Cytology (Pap)	25-29	27	Not performed	Satisfactory	NILM	NILM	Negative (NILM)	
P09167	2022	2022-Q3	2022-08	Post-pandemic recovery	Cytology (Pap)	50-54	50	Not performed	Satisfactory	NILM	NILM	Negative (NILM)	
P09168	2022	2022-Q3	2022-08	Post-pandemic recovery	Cytology (Pap)	35-39	39	Not performed	Satisfactory	NILM	NILM	Negative (NILM)	
P09169	2022	2022-Q3	2022-08	Post-pandemic recovery	Cytology (Pap)	30-34	32	Not performed	Satisfactory	NILM	NILM	Negative (NILM)	
P09170	2022	2022-Q3	2022-08	Post-pandemic recovery	Cytology (Pap)	40-44	41	Not performed	Satisfactory	Positive	AGC	Glandular	
P09171	2022	2022-Q3	2022-08	Post-pandemic recovery	Cytology (Pap)	50-54	54	Not performed	Satisfactory	NILM	NILM	Negative (NILM)	
P09172	2022	2022-Q3	2022-08	Post-pandemic recovery	Cytology (Pap)	40-44	44	Not performed	Satisfactory	NILM	NILM	Negative (NILM)	
P09173	2022	2022-Q3	2022-08	Post-pandemic recovery	Cytology (Pap)	40-44	41	Not performed	Satisfactory	NILM	NILM	Negative (NILM)	
P09174	2022	2022-Q3	2022-08	Post-pandemic recovery	Cytology (Pap)	40-44	42	Not performed	Satisfactory	NILM	NILM	Negative (NILM)	
P09175	2022	2022-Q3	2022-08	Post-pandemic recovery	Cytology (Pap)	30-34	33	Not performed	Satisfactory	Positive	LSIL	Low-grade / equivocal squamous	
P09176	2022	2022-Q3	2022-08	Post-pandemic recovery	Cytology (Pap)	40-44	44	Not performed	Satisfactory	NILM	NILM	Negative (NILM)	
P09177	2022	2022-Q3	2022-08	Post-pandemic recovery	Cytology (Pap)	40-44	42	Not performed	Satisfactory	Positive	ASC-US	Low-grade / equivocal squamous	
P09178	2022	2022-Q3	2022-08	Post-pandemic recovery	Cytology (Pap)	25-29	29	Not performed	Satisfactory	Positive	LSIL	Low-grade / equivocal squamous	
P09179	2022	2022-Q3	2022-08	Post-pandemic recovery	Cytology (Pap)	40-44	44	Not performed	Satisfactory	NILM	NILM	Negative (NILM)	
P09180	2022	2022-Q3	2022-08	Post-pandemic recovery	Cytology (Pap)	55-59	58	Not performed	Satisfactory	NILM	NILM	Negative (NILM)	
P09181	2022	2022-Q3	2022-08	Post-pandemic recovery	Cytology (Pap)	30-34	32	Not performed	Unsatisfactory	Unsatisfactory	Unsatisfactory	Unsatisfactory	
P09182	2022	2022-Q3	2022-09	Post-pandemic recovery	Cytology (Pap)	40-44	41	Not performed	Satisfactory	NILM	NILM	Negative (NILM)	
P09183	2022	2022-Q3	2022-09	Post-pandemic recovery	Cytology (Pap)	50-54	51	Not performed	Unsatisfactory	Unsatisfactory	Unsatisfactory	Unsatisfactory	
P09184	2022	2022-Q3	2022-09	Post-pandemic recovery	Cytology (Pap)	55-59	56	Not performed	Satisfactory	NILM	NILM	Negative (NILM)	
P09185	2022	2022-Q3	2022-09	Post-pandemic recovery	Cytology (Pap)	45-49	49	Not performed	Satisfactory	NILM	NILM	Negative (NILM)	
P09186	2022	2022-Q3	2022-09	Post-pandemic recovery	Cytology (Pap)	40-44	44	Not performed	Satisfactory	Positive	ASC-US	Low-grade / equivocal squamous	
P09187	2022	2022-Q3	2022-09	Post-pandemic recovery	Cytology (Pap)	35-39	38	Not performed	Satisfactory	NILM	NILM	Negative (NILM)	
P09188	2022	2022-Q3	2022-09	Post-pandemic recovery	Cytology (Pap)	35-39	39	Not performed	Satisfactory	NILM	NILM	Negative (NILM)	
P09189	2022	2022-Q3	2022-09	Post-pandemic recovery	Cytology (Pap)	40-44	41	Not performed	Satisfactory	NILM	NILM	Negative (NILM)	
P09190	2022	2022-Q3	2022-09	Post-pandemic recovery	Cytology (Pap)	45-49	48	Not performed	Satisfactory	NILM	NILM	Negative (NILM)	
P09191	2022	2022-Q3	2022-09	Post-pandemic recovery	Cytology (Pap)	40-44	40	Not performed	Satisfactory	NILM	NILM	Negative (NILM)	
P09192	2022	2022-Q3	2022-09	Post-pandemic recovery	Cytology (Pap)	60-64	60	Not performed	Satisfactory	NILM	NILM	Negative (NILM)	Candida
P09193	2022	2022-Q3	2022-09	Post-pandemic recovery	Cytology (Pap)	35-39	37	Not performed	Satisfactory	NILM	NILM	Negative (NILM)	
P09194	2022	2022-Q3	2022-09	Post-pandemic recovery	Cytology (Pap)	25-29	29	Not performed	Satisfactory	NILM	NILM	Negative (NILM)	
P09195	2022	2022-Q3	2022-09	Post-pandemic recovery	Cytology (Pap)	60-64	60	Not performed	Satisfactory	NILM	NILM	Negative (NILM)	
P09196	2022	2022-Q3	2022-09	Post-pandemic recovery	Cytology (Pap)	35-39	36	Not performed	Satisfactory	NILM	NILM	Negative (NILM)	
P09197	2022	2022-Q3	2022-09	Post-pandemic recovery	Cytology (Pap)	40-44	43	Not performed	Satisfactory	Positive	LSIL	Low-grade / equivocal squamous	Candida
P09198	2022	2022-Q3	2022-09	Post-pandemic recovery	Cytology (Pap)	55-59	57	Not performed	Satisfactory	NILM	NILM	Negative (NILM)	

patient_id	year	quarter	month	period	primary_test	age_group	age_years	hpv_result	adequacy	result	bethesda	grade	infection
P09199	2022	2022-Q3	2022-09	Post-pandemic recovery	Cytology (Pap)	25-29	29	Not performed	Satisfactory	NILM	NILM	Negative (NILM)	
P09200	2022	2022-Q3	2022-09	Post-pandemic recovery	Cytology (Pap)	35-39	37	Not performed	Satisfactory	NILM	NILM	Negative (NILM)	
P09201	2022	2022-Q3	2022-09	Post-pandemic recovery	Cytology (Pap)	25-29	25	Not performed	Satisfactory	NILM	NILM	Negative (NILM)	
P09202	2022	2022-Q3	2022-09	Post-pandemic recovery	Cytology (Pap)	50-54	53	Not performed	Satisfactory	NILM	NILM	Negative (NILM)	
P09203	2022	2022-Q3	2022-09	Post-pandemic recovery	Cytology (Pap)	25-29	26	Not performed	Satisfactory	NILM	NILM	Negative (NILM)	
P09204	2022	2022-Q3	2022-09	Post-pandemic recovery	Cytology (Pap)	40-44	40	Not performed	Satisfactory	NILM	NILM	Negative (NILM)	
P09205	2022	2022-Q3	2022-09	Post-pandemic recovery	Cytology (Pap)	50-54	50	Not performed	Satisfactory	NILM	NILM	Negative (NILM)	
P09206	2022	2022-Q3	2022-09	Post-pandemic recovery	Cytology (Pap)	40-44	41	Not performed	Unsatisfactory	Unsatisfactory	Unsatisfactory	Unsatisfactory	
P09207	2022	2022-Q3	2022-09	Post-pandemic recovery	Cytology (Pap)	60-64	62	Not performed	Satisfactory	NILM	NILM	Negative (NILM)	
P09208	2022	2022-Q3	2022-09	Post-pandemic recovery	Cytology (Pap)	40-44	43	Not performed	Satisfactory	NILM	NILM	Negative (NILM)	
P09209	2022	2022-Q3	2022-09	Post-pandemic recovery	Cytology (Pap)	40-44	41	Not performed	Satisfactory	NILM	NILM	Negative (NILM)	
P09210	2022	2022-Q3	2022-09	Post-pandemic recovery	Cytology (Pap)	35-39	39	Not performed	Satisfactory	NILM	NILM	Negative (NILM)	Trichomonas
P09211	2022	2022-Q3	2022-09	Post-pandemic recovery	Cytology (Pap)	35-39	39	Not performed	Satisfactory	Positive	LSIL	Low-grade / equivocal squamous	
P09212	2022	2022-Q3	2022-09	Post-pandemic recovery	Cytology (Pap)	40-44	44	Not performed	Satisfactory	NILM	NILM	Negative (NILM)	
P09213	2022	2022-Q3	2022-09	Post-pandemic recovery	Cytology (Pap)	30-34	31	Not performed	Satisfactory	NILM	NILM	Negative (NILM)	
P09214	2022	2022-Q3	2022-09	Post-pandemic recovery	Cytology (Pap)	35-39	38	Not performed	Satisfactory	NILM	NILM	Negative (NILM)	
P09215	2022	2022-Q3	2022-09	Post-pandemic recovery	Cytology (Pap)	35-39	38	Not performed	Satisfactory	NILM	NILM	Negative (NILM)	
P09216	2022	2022-Q3	2022-09	Post-pandemic recovery	Cytology (Pap)	40-44	40	Not performed	Satisfactory	NILM	NILM	Negative (NILM)	
P09217	2022	2022-Q3	2022-09	Post-pandemic recovery	Cytology (Pap)	35-39	35	Not performed	Satisfactory	NILM	NILM	Negative (NILM)	
P09218	2022	2022-Q3	2022-09	Post-pandemic recovery	Cytology (Pap)	60-64	64	Not performed	Satisfactory	NILM	NILM	Negative (NILM)	
P09219	2022	2022-Q3	2022-09	Post-pandemic recovery	Cytology (Pap)	60-64	62	Not performed	Satisfactory	NILM	NILM	Negative (NILM)	
P09220	2022	2022-Q3	2022-09	Post-pandemic recovery	Cytology (Pap)	30-34	34	Not performed	Satisfactory	NILM	NILM	Negative (NILM)	
P09221	2022	2022-Q3	2022-09	Post-pandemic recovery	Cytology (Pap)	60-64	64	Not performed	Satisfactory	NILM	NILM	Negative (NILM)	
P09222	2022	2022-Q3	2022-09	Post-pandemic recovery	Cytology (Pap)	40-44	41	Not performed	Satisfactory	NILM	NILM	Negative (NILM)	
P09223	2022	2022-Q3	2022-09	Post-pandemic recovery	Cytology (Pap)	35-39	37	Not performed	Satisfactory	Positive	ASC-US	Low-grade / equivocal squamous	
P09224	2022	2022-Q3	2022-09	Post-pandemic recovery	Cytology (Pap)	40-44	44	Not performed	Satisfactory	NILM	NILM	Negative (NILM)	
P09225	2022	2022-Q3	2022-09	Post-pandemic recovery	Cytology (Pap)	25-29	28	Not performed	Satisfactory	Positive	LSIL	Low-grade / equivocal squamous	
P09226	2022	2022-Q3	2022-09	Post-pandemic recovery	Cytology (Pap)	50-54	52	Not performed	Satisfactory	NILM	NILM	Negative (NILM)	
P09227	2022	2022-Q3	2022-09	Post-pandemic recovery	Cytology (Pap)	40-44	43	Not performed	Satisfactory	Positive	ASC-US	Low-grade / equivocal squamous	Other
P09228	2022	2022-Q3	2022-09	Post-pandemic recovery	Cytology (Pap)	40-44	41	Not performed	Satisfactory	Positive	AGC	Glandular	
P09229	2022	2022-Q3	2022-09	Post-pandemic recovery	Cytology (Pap)	35-39	36	Not performed	Satisfactory	NILM	NILM	Negative (NILM)	Trichomonas
P09230	2022	2022-Q3	2022-09	Post-pandemic recovery	Cytology (Pap)	35-39	38	Not performed	Satisfactory	Positive	LSIL	Low-grade / equivocal squamous	
P09231	2022	2022-Q3	2022-09	Post-pandemic recovery	Cytology (Pap)	40-44	43	Not performed	Satisfactory	NILM	NILM	Negative (NILM)	
P09232	2022	2022-Q3	2022-09	Post-pandemic recovery	Cytology (Pap)	40-44	42	Not performed	Satisfactory	NILM	NILM	Negative (NILM)	
P09233	2022	2022-Q3	2022-09	Post-pandemic recovery	Cytology (Pap)	25-29	25	Not performed	Satisfactory	Positive	LSIL	Low-grade / equivocal squamous	
P09234	2022	2022-Q3	2022-09	Post-pandemic recovery	Cytology (Pap)	35-39	37	Not performed	Satisfactory	Positive	HSIL	High-grade squamous	
P09235	2022	2022-Q3	2022-09	Post-pandemic recovery	Cytology (Pap)	40-44	43	Not performed	Satisfactory	NILM	NILM	Negative (NILM)	
P09236	2022	2022-Q3	2022-09	Post-pandemic recovery	Cytology (Pap)	35-39	39	Not performed	Satisfactory	NILM	NILM	Negative (NILM)	
P09237	2022	2022-Q3	2022-09	Post-pandemic recovery	Cytology (Pap)	25-29	28	Not performed	Satisfactory	NILM	NILM	Negative (NILM)	
P09238	2022	2022-Q3	2022-09	Post-pandemic recovery	Cytology (Pap)	40-44	44	Not performed	Satisfactory	NILM	NILM	Negative (NILM)	
P09239	2022	2022-Q3	2022-09	Post-pandemic recovery	Cytology (Pap)	45-49	47	Not performed	Satisfactory	NILM	NILM	Negative (NILM)	
P09240	2022	2022-Q3	2022-09	Post-pandemic recovery	Cytology (Pap)	35-39	35	Not performed	Satisfactory	NILM	NILM	Negative (NILM)	
P09241	2022	2022-Q3	2022-09	Post-pandemic recovery	Cytology (Pap)	55-59	59	Not performed	Satisfactory	Positive	HSIL	High-grade squamous	
P09242	2022	2022-Q3	2022-09	Post-pandemic recovery	Cytology (Pap)	45-49	47	Not performed	Satisfactory	NILM	NILM	Negative (NILM)	
P09243	2022	2022-Q3	2022-09	Post-pandemic recovery	Cytology (Pap)	40-44	42	Not performed	Satisfactory	NILM	NILM	Negative (NILM)	
P09244	2022	2022-Q3	2022-09	Post-pandemic recovery	Cytology (Pap)	45-49	47	Not performed	Satisfactory	NILM	NILM	Negative (NILM)	
P09245	2022	2022-Q3	2022-09	Post-pandemic recovery	Cytology (Pap)	45-49	47	Not performed	Satisfactory	NILM	NILM	Negative (NILM)	
P09246	2022	2022-Q3	2022-09	Post-pandemic recovery	Cytology (Pap)	55-59	56	Not performed	Satisfactory	Positive	HSIL	High-grade squamous	
P09247	2022	2022-Q3	2022-09	Post-pandemic recovery	Cytology (Pap)	30-34	30	Not performed	Satisfactory	NILM	NILM	Negative (NILM)	
P09248	2022	2022-Q3	2022-09	Post-pandemic recovery	Cytology (Pap)	30-34	32	Not performed	Satisfactory	NILM	NILM	Negative (NILM)	
P09249	2022	2022-Q3	2022-09	Post-pandemic recovery	Cytology (Pap)	45-49	48	Not performed	Satisfactory	NILM	NILM	Negative (NILM)	
P09250	2022	2022-Q3	2022-09	Post-pandemic recovery	Cytology (Pap)	40-44	41	Not performed	Satisfactory	NILM	NILM	Negative (NILM)	
P09251	2022	2022-Q3	2022-09	Post-pandemic recovery	Cytology (Pap)	50-54	50	Not performed	Satisfactory	NILM	NILM	Negative (NILM)	
P09252	2022	2022-Q3	2022-09	Post-pandemic recovery	Cytology (Pap)	45-49	49	Not performed	Satisfactory	Positive	AGC	Glandular	
P09253	2022	2022-Q3	2022-09	Post-pandemic recovery	Cytology (Pap)	30-34	34	Not performed	Satisfactory	NILM	NILM	Negative (NILM)	
P09254	2022	2022-Q3	2022-09	Post-pandemic recovery	Cytology (Pap)	30-34	31	Not performed	Satisfactory	NILM	NILM	Negative (NILM)	Gardnerella
P09255	2022	2022-Q3	2022-09	Post-pandemic recovery	Cytology (Pap)	40-44	41	Not performed	Satisfactory	Positive	ASC-US	Low-grade / equivocal squamous	
P09256	2022	2022-Q3	2022-09	Post-pandemic recovery	Cytology (Pap)	40-44	44	Not performed	Satisfactory	NILM	NILM	Negative (NILM)	
P09257	2022	2022-Q3	2022-09	Post-pandemic recovery	Cytology (Pap)	40-44	41	Not performed	Satisfactory	NILM	NILM	Negative (NILM)	
P09258	2022	2022-Q3	2022-09	Post-pandemic recovery	Cytology (Pap)	50-54	50	Not performed	Unsatisfactory	Unsatisfactory	Unsatisfactory	Unsatisfactory	
P09259	2022	2022-Q3	2022-09	Post-pandemic recovery	Cytology (Pap)	55-59	55	Not performed	Satisfactory	NILM	NILM	Negative (NILM)	
P09260	2022	2022-Q3	2022-09	Post-pandemic recovery	Cytology (Pap)	45-49	45	Not performed	Satisfactory	Positive	HSIL	High-grade squamous	
P09261	2022	2022-Q3	2022-09	Post-pandemic recovery	Cytology (Pap)	40-44	42	Not performed	Satisfactory	NILM	NILM	Negative (NILM)	
P09262	2022	2022-Q3	2022-09	Post-pandemic recovery	Cytology (Pap)	35-39	37	Not performed	Satisfactory	NILM	NILM	Negative (NILM)	
P09263	2022	2022-Q3	2022-09	Post-pandemic recovery	Cytology (Pap)	50-54	50	Not performed	Satisfactory	Positive	AGC	Glandular	
P09264	2022	2022-Q3	2022-09	Post-pandemic recovery	Cytology (Pap)	35-39	37	Not performed	Satisfactory	NILM	NILM	Negative (NILM)	
P09265	2022	2022-Q3	2022-09	Post-pandemic recovery	Cytology (Pap)	25-29	27	Not performed	Satisfactory	NILM	NILM	Negative (NILM)	
P09266	2022	2022-Q3	2022-09	Post-pandemic recovery	Cytology (Pap)	35-39	39	Not performed	Satisfactory	NILM	NILM	Negative (NILM)	
P09267	2022	2022-Q3	2022-09	Post-pandemic recovery	Cytology (Pap)	55-59	55	Not performed	Satisfactory	NILM	NILM	Negative (NILM)	
P09268	2022	2022-Q3	2022-09	Post-pandemic recovery	Cytology (Pap)	50-54	52	Not performed	Satisfactory	NILM	NILM	Negative (NILM)	
P09269	2022	2022-Q3	2022-09	Post-pandemic recovery	Cytology (Pap)	35-39	38	Not performed	Satisfactory	NILM	NILM	Negative (NILM)	
P09270	2022	2022-Q3	2022-09	Post-pandemic recovery	Cytology (Pap)	30-34	33	Not performed	Satisfactory	NILM	NILM	Negative (NILM)	
P09271	2022	2022-Q3	2022-09	Post-pandemic recovery	Cytology (Pap)	30-34	33	Not performed	Unsatisfactory	Unsatisfactory	Unsatisfactory	Unsatisfactory	Candida
P09272	2022	2022-Q3	2022-09	Post-pandemic recovery	Cytology (Pap)	55-59	56	Not performed	Satisfactory	Positive	HSIL	High-grade squamous	
P09273	2022	2022-Q3	2022-09	Post-pandemic recovery	Cytology (Pap)	40-44	44	Not performed	Satisfactory	NILM	NILM	Negative (NILM)	
P09274	2022	2022-Q3	2022-09	Post-pandemic recovery	Cytology (Pap)	25-29	26	Not performed	Satisfactory	Positive	HSIL	High-grade squamous	
P09275	2022	2022-Q3	2022-09	Post-pandemic recovery	Cytology (Pap)	30-34	34	Not performed	Satisfactory	NILM	NILM	Negative (NILM)	
P09276	2022	2022-Q3	2022-09	Post-pandemic recovery	Cytology (Pap)	30-34	33	Not performed	Satisfactory	NILM	NILM	Negative (NILM)	
P09277	2022	2022-Q3	2022-09	Post-pandemic recovery	Cytology (Pap)	40-44	40	Not performed	Satisfactory	NILM	NILM	Negative (NILM)	
P09278	2022	2022-Q3	2022-09	Post-pandemic recovery	Cytology (Pap)	25-29	28	Not performed	Satisfactory	Positive	ASC-H	High-grade squamous	

patient_id	year	quarter	month	period	primary_test	age_group	age_years	hpv_result	adequacy	result	bethesda	grade	infection
P09279	2022	2022-Q3	2022-09	Post-pandemic recovery	Cytology (Pap)	40-44	43	Not performed	Satisfactory	NILM	NILM	Negative (NILM)	
P09280	2022	2022-Q3	2022-09	Post-pandemic recovery	Cytology (Pap)	60-64	64	Not performed	Satisfactory	NILM	NILM	Negative (NILM)	
P09281	2022	2022-Q3	2022-09	Post-pandemic recovery	Cytology (Pap)	55-59	57	Not performed	Satisfactory	Positive	ASC-US	Low-grade / equivocal squamous	
P09282	2022	2022-Q3	2022-09	Post-pandemic recovery	Cytology (Pap)	40-44	41	Not performed	Satisfactory	NILM	NILM	Negative (NILM)	
P09283	2022	2022-Q3	2022-09	Post-pandemic recovery	Cytology (Pap)	35-39	36	Not performed	Satisfactory	NILM	NILM	Negative (NILM)	
P09284	2022	2022-Q3	2022-09	Post-pandemic recovery	Cytology (Pap)	50-54	54	Not performed	Satisfactory	NILM	NILM	Negative (NILM)	
P09285	2022	2022-Q3	2022-09	Post-pandemic recovery	Cytology (Pap)	30-34	30	Not performed	Satisfactory	NILM	NILM	Negative (NILM)	
P09286	2022	2022-Q3	2022-09	Post-pandemic recovery	Cytology (Pap)	60-64	60	Not performed	Satisfactory	Positive	LSIL	Low-grade / equivocal squamous	
P09287	2022	2022-Q3	2022-09	Post-pandemic recovery	Cytology (Pap)	45-49	45	Not performed	Satisfactory	NILM	NILM	Negative (NILM)	
P09288	2022	2022-Q3	2022-09	Post-pandemic recovery	Cytology (Pap)	45-49	48	Not performed	Satisfactory	NILM	NILM	Negative (NILM)	
P09289	2022	2022-Q3	2022-09	Post-pandemic recovery	Cytology (Pap)	30-34	32	Not performed	Satisfactory	NILM	NILM	Negative (NILM)	
P09290	2022	2022-Q3	2022-09	Post-pandemic recovery	Cytology (Pap)	40-44	42	Not performed	Satisfactory	Positive	LSIL	Low-grade / equivocal squamous	Trichomonas
P09291	2022	2022-Q3	2022-09	Post-pandemic recovery	Cytology (Pap)	35-39	39	Not performed	Satisfactory	Positive	LSIL	Low-grade / equivocal squamous	
P09292	2022	2022-Q3	2022-09	Post-pandemic recovery	Cytology (Pap)	30-34	33	Not performed	Satisfactory	NILM	NILM	Negative (NILM)	
P09293	2022	2022-Q3	2022-09	Post-pandemic recovery	Cytology (Pap)	35-39	39	Not performed	Satisfactory	NILM	NILM	Negative (NILM)	
P09294	2022	2022-Q3	2022-09	Post-pandemic recovery	Cytology (Pap)	40-44	43	Not performed	Satisfactory	Positive	AGC	Glandular	
P09295	2022	2022-Q3	2022-09	Post-pandemic recovery	Cytology (Pap)	45-49	48	Not performed	Satisfactory	NILM	NILM	Negative (NILM)	
P09296	2022	2022-Q3	2022-09	Post-pandemic recovery	Cytology (Pap)	40-44	41	Not performed	Satisfactory	NILM	NILM	Negative (NILM)	
P09297	2022	2022-Q3	2022-09	Post-pandemic recovery	Cytology (Pap)	40-44	40	Not performed	Satisfactory	NILM	NILM	Negative (NILM)	
P09298	2022	2022-Q3	2022-09	Post-pandemic recovery	Cytology (Pap)	45-49	46	Not performed	Satisfactory	NILM	NILM	Negative (NILM)	
P09299	2022	2022-Q3	2022-09	Post-pandemic recovery	Cytology (Pap)	45-49	49	Not performed	Satisfactory	NILM	NILM	Negative (NILM)	
P09300	2022	2022-Q3	2022-09	Post-pandemic recovery	Cytology (Pap)	35-39	37	Not performed	Satisfactory	NILM	NILM	Negative (NILM)	
P09301	2022	2022-Q3	2022-09	Post-pandemic recovery	Cytology (Pap)	30-34	31	Not performed	Satisfactory	NILM	NILM	Negative (NILM)	
P09302	2022	2022-Q3	2022-09	Post-pandemic recovery	Cytology (Pap)	25-29	25	Not performed	Satisfactory	NILM	NILM	Negative (NILM)	
P09303	2022	2022-Q3	2022-09	Post-pandemic recovery	Cytology (Pap)	50-54	54	Not performed	Satisfactory	NILM	NILM	Negative (NILM)	
P09304	2022	2022-Q3	2022-09	Post-pandemic recovery	Cytology (Pap)	60-64	61	Not performed	Satisfactory	NILM	NILM	Negative (NILM)	Other
P09305	2022	2022-Q3	2022-09	Post-pandemic recovery	Cytology (Pap)	50-54	50	Not performed	Satisfactory	NILM	NILM	Negative (NILM)	
P09306	2022	2022-Q3	2022-09	Post-pandemic recovery	Cytology (Pap)	30-34	32	Not performed	Satisfactory	NILM	NILM	Negative (NILM)	
P09307	2022	2022-Q3	2022-09	Post-pandemic recovery	Cytology (Pap)	25-29	25	Not performed	Satisfactory	NILM	NILM	Negative (NILM)	
P09308	2022	2022-Q3	2022-09	Post-pandemic recovery	Cytology (Pap)	35-39	35	Not performed	Satisfactory	NILM	NILM	Negative (NILM)	
P09309	2022	2022-Q3	2022-09	Post-pandemic recovery	Cytology (Pap)	60-64	60	Not performed	Satisfactory	NILM	NILM	Negative (NILM)	
P09310	2022	2022-Q3	2022-09	Post-pandemic recovery	Cytology (Pap)	35-39	37	Not performed	Satisfactory	NILM	NILM	Negative (NILM)	
P09311	2022	2022-Q3	2022-09	Post-pandemic recovery	Cytology (Pap)	45-49	48	Not performed	Satisfactory	Positive	ASC-US	Low-grade / equivocal squamous	
P09312	2022	2022-Q3	2022-09	Post-pandemic recovery	Cytology (Pap)	60-64	64	Not performed	Satisfactory	NILM	NILM	Negative (NILM)	
P09313	2022	2022-Q3	2022-09	Post-pandemic recovery	Cytology (Pap)	45-49	47	Not performed	Satisfactory	NILM	NILM	Negative (NILM)	
P09314	2022	2022-Q3	2022-09	Post-pandemic recovery	Cytology (Pap)	60-64	64	Not performed	Satisfactory	NILM	NILM	Negative (NILM)	
P09315	2022	2022-Q3	2022-09	Post-pandemic recovery	Cytology (Pap)	40-44	40	Not performed	Satisfactory	NILM	NILM	Negative (NILM)	
P09316	2022	2022-Q3	2022-09	Post-pandemic recovery	Cytology (Pap)	40-44	41	Not performed	Satisfactory	Positive	LSIL	Low-grade / equivocal squamous	
P09317	2022	2022-Q3	2022-09	Post-pandemic recovery	Cytology (Pap)	60-64	64	Not performed	Satisfactory	NILM	NILM	Negative (NILM)	
P09318	2022	2022-Q3	2022-09	Post-pandemic recovery	Cytology (Pap)	30-34	31	Not performed	Satisfactory	NILM	NILM	Negative (NILM)	
P09319	2022	2022-Q3	2022-09	Post-pandemic recovery	Cytology (Pap)	45-49	48	Not performed	Satisfactory	Positive	ASC-US	Low-grade / equivocal squamous	
P09320	2022	2022-Q3	2022-09	Post-pandemic recovery	Cytology (Pap)	45-49	48	Not performed	Satisfactory	NILM	NILM	Negative (NILM)	
P09321	2022	2022-Q3	2022-09	Post-pandemic recovery	Cytology (Pap)	30-34	32	Not performed	Satisfactory	NILM	NILM	Negative (NILM)	
P09322	2022	2022-Q3	2022-09	Post-pandemic recovery	Cytology (Pap)	45-49	46	Not performed	Satisfactory	NILM	NILM	Negative (NILM)	
P09323	2022	2022-Q3	2022-09	Post-pandemic recovery	Cytology (Pap)	55-59	58	Not performed	Satisfactory	NILM	NILM	Negative (NILM)	
P09324	2022	2022-Q3	2022-09	Post-pandemic recovery	Cytology (Pap)	45-49	45	Not performed	Satisfactory	NILM	NILM	Negative (NILM)	
P09325	2022	2022-Q3	2022-09	Post-pandemic recovery	Cytology (Pap)	50-54	52	Not performed	Satisfactory	NILM	NILM	Negative (NILM)	
P09326	2022	2022-Q3	2022-09	Post-pandemic recovery	Cytology (Pap)	60-64	61	Not performed	Satisfactory	NILM	NILM	Negative (NILM)	Candida
P09327	2022	2022-Q3	2022-09	Post-pandemic recovery	Cytology (Pap)	55-59	58	Not performed	Satisfactory	NILM	NILM	Negative (NILM)	
P09328	2022	2022-Q4	2022-10	Post-pandemic recovery	Cytology (Pap)	35-39	38	Not performed	Satisfactory	Positive	ASC-US	Low-grade / equivocal squamous	
P09329	2022	2022-Q4	2022-10	Post-pandemic recovery	Cytology (Pap)	40-44	41	Not performed	Satisfactory	NILM	NILM	Negative (NILM)	
P09330	2022	2022-Q4	2022-10	Post-pandemic recovery	Cytology (Pap)	35-39	36	Not performed	Satisfactory	NILM	NILM	Negative (NILM)	
P09331	2022	2022-Q4	2022-10	Post-pandemic recovery	Cytology (Pap)	40-44	43	Not performed	Satisfactory	NILM	NILM	Negative (NILM)	
P09332	2022	2022-Q4	2022-10	Post-pandemic recovery	Cytology (Pap)	50-54	52	Not performed	Satisfactory	NILM	NILM	Negative (NILM)	
P09333	2022	2022-Q4	2022-10	Post-pandemic recovery	Cytology (Pap)	30-34	30	Not performed	Satisfactory	Positive	LSIL	Low-grade / equivocal squamous	
P09334	2022	2022-Q4	2022-10	Post-pandemic recovery	Cytology (Pap)	45-49	48	Not performed	Satisfactory	Positive	HSIL	High-grade squamous	Candida
P09335	2022	2022-Q4	2022-10	Post-pandemic recovery	Cytology (Pap)	35-39	35	Not performed	Satisfactory	NILM	NILM	Negative (NILM)	
P09336	2022	2022-Q4	2022-10	Post-pandemic recovery	Cytology (Pap)	50-54	52	Not performed	Satisfactory	NILM	NILM	Negative (NILM)	
P09337	2022	2022-Q4	2022-10	Post-pandemic recovery	Cytology (Pap)	60-64	60	Not performed	Satisfactory	NILM	NILM	Negative (NILM)	
P09338	2022	2022-Q4	2022-10	Post-pandemic recovery	Cytology (Pap)	60-64	63	Not performed	Satisfactory	Positive	LSIL	Low-grade / equivocal squamous	
P09339	2022	2022-Q4	2022-10	Post-pandemic recovery	Cytology (Pap)	30-34	32	Not performed	Satisfactory	NILM	NILM	Negative (NILM)	Candida
P09340	2022	2022-Q4	2022-10	Post-pandemic recovery	Cytology (Pap)	35-39	37	Not performed	Unsatisfactory	Unsatisfactory	Unsatisfactory	Unsatisfactory	
P09341	2022	2022-Q4	2022-10	Post-pandemic recovery	Cytology (Pap)	50-54	50	Not performed	Satisfactory	NILM	NILM	Negative (NILM)	
P09342	2022	2022-Q4	2022-10	Post-pandemic recovery	Cytology (Pap)	35-39	38	Not performed	Satisfactory	NILM	NILM	Negative (NILM)	
P09343	2022	2022-Q4	2022-10	Post-pandemic recovery	Cytology (Pap)	50-54	53	Not performed	Satisfactory	NILM	NILM	Negative (NILM)	
P09344	2022	2022-Q4	2022-10	Post-pandemic recovery	Cytology (Pap)	35-39	35	Not performed	Satisfactory	NILM	NILM	Negative (NILM)	
P09345	2022	2022-Q4	2022-10	Post-pandemic recovery	Cytology (Pap)	30-34	31	Not performed	Satisfactory	NILM	NILM	Negative (NILM)	
P09346	2022	2022-Q4	2022-10	Post-pandemic recovery	Cytology (Pap)	40-44	42	Not performed	Satisfactory	NILM	NILM	Negative (NILM)	
P09347	2022	2022-Q4	2022-10	Post-pandemic recovery	Cytology (Pap)	55-59	56	Not performed	Satisfactory	NILM	NILM	Negative (NILM)	
P09348	2022	2022-Q4	2022-10	Post-pandemic recovery	Cytology (Pap)	45-49	49	Not performed	Satisfactory	NILM	NILM	Negative (NILM)	
P09349	2022	2022-Q4	2022-10	Post-pandemic recovery	Cytology (Pap)	50-54	52	Not performed	Satisfactory	NILM	NILM	Negative (NILM)	
P09350	2022	2022-Q4	2022-10	Post-pandemic recovery	Cytology (Pap)	30-34	30	Not performed	Satisfactory	NILM	NILM	Negative (NILM)	
P09351	2022	2022-Q4	2022-10	Post-pandemic recovery	Cytology (Pap)	40-44	42	Not performed	Satisfactory	NILM	NILM	Negative (NILM)	
P09352	2022	2022-Q4	2022-10	Post-pandemic recovery	Cytology (Pap)	50-54	50	Not performed	Satisfactory	NILM	NILM	Negative (NILM)	
P09353	2022	2022-Q4	2022-10	Post-pandemic recovery	Cytology (Pap)	40-44	41	Not performed	Satisfactory	NILM	NILM	Negative (NILM)	
P09354	2022	2022-Q4	2022-10	Post-pandemic recovery	Cytology (Pap)	40-44	43	Not performed	Satisfactory	NILM	NILM	Negative (NILM)	
P09355	2022	2022-Q4	2022-10	Post-pandemic recovery	Cytology (Pap)	30-34	33	Not performed	Satisfactory	NILM	NILM	Negative (NILM)	
P09356	2022	2022-Q4	2022-10	Post-pandemic recovery	Cytology (Pap)	25-29	29	Not performed	Satisfactory	NILM	NILM	Negative (NILM)	
P09357	2022	2022-Q4	2022-10	Post-pandemic recovery	Cytology (Pap)	55-59	55	Not performed	Satisfactory	NILM	NILM	Negative (NILM)	
P09358	2022	2022-Q4	2022-10	Post-pandemic recovery	Cytology (Pap)	30-34	31	Not performed	Satisfactory	NILM	NILM	Negative (NILM)	

patient_id	year	quarter	month	period	primary_test	age_group	age_years	hpv_result	adequacy	result	bethesda	grade	infection
P09359	2022	2022-Q4	2022-10	Post-pandemic recovery	Cytology (Pap)	55-59	57	Not performed	Satisfactory	NILM	NILM	Negative (NILM)	
P09360	2022	2022-Q4	2022-10	Post-pandemic recovery	Cytology (Pap)	60-64	63	Not performed	Satisfactory	NILM	NILM	Negative (NILM)	
P09361	2022	2022-Q4	2022-10	Post-pandemic recovery	Cytology (Pap)	35-39	35	Not performed	Satisfactory	NILM	NILM	Negative (NILM)	
P09362	2022	2022-Q4	2022-10	Post-pandemic recovery	Cytology (Pap)	55-59	56	Not performed	Satisfactory	NILM	NILM	Negative (NILM)	
P09363	2022	2022-Q4	2022-10	Post-pandemic recovery	Cytology (Pap)	30-34	30	Not performed	Satisfactory	NILM	NILM	Negative (NILM)	
P09364	2022	2022-Q4	2022-10	Post-pandemic recovery	Cytology (Pap)	35-39	36	Not performed	Satisfactory	NILM	NILM	Negative (NILM)	
P09365	2022	2022-Q4	2022-10	Post-pandemic recovery	Cytology (Pap)	30-34	34	Not performed	Satisfactory	NILM	NILM	Negative (NILM)	
P09366	2022	2022-Q4	2022-10	Post-pandemic recovery	Cytology (Pap)	45-49	47	Not performed	Satisfactory	NILM	NILM	Negative (NILM)	
P09367	2022	2022-Q4	2022-10	Post-pandemic recovery	Cytology (Pap)	45-49	45	Not performed	Satisfactory	NILM	NILM	Negative (NILM)	
P09368	2022	2022-Q4	2022-10	Post-pandemic recovery	Cytology (Pap)	35-39	36	Not performed	Satisfactory	NILM	NILM	Negative (NILM)	
P09369	2022	2022-Q4	2022-10	Post-pandemic recovery	Cytology (Pap)	45-49	45	Not performed	Satisfactory	NILM	NILM	Negative (NILM)	
P09370	2022	2022-Q4	2022-10	Post-pandemic recovery	Cytology (Pap)	40-44	43	Not performed	Satisfactory	NILM	NILM	Negative (NILM)	
P09371	2022	2022-Q4	2022-10	Post-pandemic recovery	Cytology (Pap)	40-44	44	Not performed	Satisfactory	Positive	LSIL	Low-grade / equivocal squamous	
P09372	2022	2022-Q4	2022-10	Post-pandemic recovery	Cytology (Pap)	40-44	44	Not performed	Satisfactory	Positive	ASC-US	Low-grade / equivocal squamous	
P09373	2022	2022-Q4	2022-10	Post-pandemic recovery	Cytology (Pap)	40-44	41	Not performed	Satisfactory	NILM	NILM	Negative (NILM)	
P09374	2022	2022-Q4	2022-10	Post-pandemic recovery	Cytology (Pap)	45-49	45	Not performed	Satisfactory	Positive	HSIL	High-grade squamous	
P09375	2022	2022-Q4	2022-10	Post-pandemic recovery	Cytology (Pap)	25-29	26	Not performed	Satisfactory	NILM	NILM	Negative (NILM)	
P09376	2022	2022-Q4	2022-10	Post-pandemic recovery	Cytology (Pap)	45-49	45	Not performed	Satisfactory	NILM	NILM	Negative (NILM)	
P09377	2022	2022-Q4	2022-10	Post-pandemic recovery	Cytology (Pap)	35-39	38	Not performed	Satisfactory	Positive	LSIL	Low-grade / equivocal squamous	
P09378	2022	2022-Q4	2022-10	Post-pandemic recovery	Cytology (Pap)	40-44	41	Not performed	Satisfactory	NILM	NILM	Negative (NILM)	
P09379	2022	2022-Q4	2022-10	Post-pandemic recovery	Cytology (Pap)	25-29	25	Not performed	Satisfactory	NILM	NILM	Negative (NILM)	
P09380	2022	2022-Q4	2022-10	Post-pandemic recovery	Cytology (Pap)	45-49	45	Not performed	Satisfactory	NILM	NILM	Negative (NILM)	
P09381	2022	2022-Q4	2022-10	Post-pandemic recovery	Cytology (Pap)	30-34	32	Not performed	Satisfactory	NILM	NILM	Negative (NILM)	
P09382	2022	2022-Q4	2022-10	Post-pandemic recovery	Cytology (Pap)	30-34	31	Not performed	Satisfactory	NILM	NILM	Negative (NILM)	
P09383	2022	2022-Q4	2022-10	Post-pandemic recovery	Cytology (Pap)	30-34	34	Not performed	Satisfactory	Positive	ASC-US	Low-grade / equivocal squamous	
P09384	2022	2022-Q4	2022-10	Post-pandemic recovery	Cytology (Pap)	50-54	51	Not performed	Satisfactory	Positive	ASC-US	Low-grade / equivocal squamous	
P09385	2022	2022-Q4	2022-10	Post-pandemic recovery	Cytology (Pap)	30-34	32	Not performed	Satisfactory	NILM	NILM	Negative (NILM)	
P09386	2022	2022-Q4	2022-10	Post-pandemic recovery	Cytology (Pap)	30-34	33	Not performed	Satisfactory	NILM	NILM	Negative (NILM)	
P09387	2022	2022-Q4	2022-10	Post-pandemic recovery	Cytology (Pap)	25-29	25	Not performed	Satisfactory	NILM	NILM	Negative (NILM)	
P09388	2022	2022-Q4	2022-10	Post-pandemic recovery	Cytology (Pap)	35-39	37	Not performed	Satisfactory	NILM	NILM	Negative (NILM)	
P09389	2022	2022-Q4	2022-10	Post-pandemic recovery	Cytology (Pap)	50-54	53	Not performed	Unsatisfactory	Unsatisfactory	Unsatisfactory	Unsatisfactory	
P09390	2022	2022-Q4	2022-10	Post-pandemic recovery	Cytology (Pap)	45-49	47	Not performed	Satisfactory	NILM	NILM	Negative (NILM)	
P09391	2022	2022-Q4	2022-10	Post-pandemic recovery	Cytology (Pap)	35-39	38	Not performed	Satisfactory	Positive	ASC-US	Low-grade / equivocal squamous	
P09392	2022	2022-Q4	2022-10	Post-pandemic recovery	Cytology (Pap)	45-49	47	Not performed	Satisfactory	NILM	NILM	Negative (NILM)	Candida
P09393	2022	2022-Q4	2022-10	Post-pandemic recovery	Cytology (Pap)	50-54	50	Not performed	Satisfactory	NILM	NILM	Negative (NILM)	
P09394	2022	2022-Q4	2022-10	Post-pandemic recovery	Cytology (Pap)	40-44	44	Not performed	Satisfactory	NILM	NILM	Negative (NILM)	
P09395	2022	2022-Q4	2022-10	Post-pandemic recovery	Cytology (Pap)	30-34	32	Not performed	Satisfactory	NILM	NILM	Negative (NILM)	
P09396	2022	2022-Q4	2022-10	Post-pandemic recovery	Cytology (Pap)	55-59	55	Not performed	Satisfactory	Positive	ASC-H	High-grade squamous	
P09397	2022	2022-Q4	2022-10	Post-pandemic recovery	Cytology (Pap)	50-54	52	Not performed	Satisfactory	Positive	ASC-US	Low-grade / equivocal squamous	
P09398	2022	2022-Q4	2022-10	Post-pandemic recovery	Cytology (Pap)	45-49	48	Not performed	Satisfactory	Positive	ASC-H	High-grade squamous	
P09399	2022	2022-Q4	2022-10	Post-pandemic recovery	Cytology (Pap)	40-44	42	Not performed	Satisfactory	NILM	NILM	Negative (NILM)	
P09400	2022	2022-Q4	2022-10	Post-pandemic recovery	Cytology (Pap)	35-39	39	Not performed	Satisfactory	Positive	AGC	Glandular	
P09401	2022	2022-Q4	2022-10	Post-pandemic recovery	Cytology (Pap)	40-44	41	Not performed	Satisfactory	NILM	NILM	Negative (NILM)	
P09402	2022	2022-Q4	2022-10	Post-pandemic recovery	Cytology (Pap)	40-44	41	Not performed	Satisfactory	NILM	NILM	Negative (NILM)	
P09403	2022	2022-Q4	2022-10	Post-pandemic recovery	Cytology (Pap)	35-39	37	Not performed	Satisfactory	NILM	NILM	Negative (NILM)	
P09404	2022	2022-Q4	2022-10	Post-pandemic recovery	Cytology (Pap)	35-39	35	Not performed	Satisfactory	NILM	NILM	Negative (NILM)	
P09405	2022	2022-Q4	2022-10	Post-pandemic recovery	Cytology (Pap)	55-59	57	Not performed	Satisfactory	NILM	NILM	Negative (NILM)	
P09406	2022	2022-Q4	2022-10	Post-pandemic recovery	Cytology (Pap)	55-59	58	Not performed	Satisfactory	NILM	NILM	Negative (NILM)	
P09407	2022	2022-Q4	2022-10	Post-pandemic recovery	Cytology (Pap)	50-54	52	Not performed	Satisfactory	NILM	NILM	Negative (NILM)	
P09408	2022	2022-Q4	2022-10	Post-pandemic recovery	Cytology (Pap)	55-59	55	Not performed	Satisfactory	Positive	HSIL	High-grade squamous	
P09409	2022	2022-Q4	2022-10	Post-pandemic recovery	Cytology (Pap)	55-59	58	Not performed	Satisfactory	NILM	NILM	Negative (NILM)	
P09410	2022	2022-Q4	2022-10	Post-pandemic recovery	Cytology (Pap)	55-59	56	Not performed	Satisfactory	NILM	NILM	Negative (NILM)	
P09411	2022	2022-Q4	2022-10	Post-pandemic recovery	Cytology (Pap)	35-39	36	Not performed	Satisfactory	Positive	ASC-H	High-grade squamous	
P09412	2022	2022-Q4	2022-10	Post-pandemic recovery	Cytology (Pap)	30-34	33	Not performed	Satisfactory	NILM	NILM	Negative (NILM)	Candida
P09413	2022	2022-Q4	2022-10	Post-pandemic recovery	Cytology (Pap)	45-49	47	Not performed	Satisfactory	NILM	NILM	Negative (NILM)	
P09414	2022	2022-Q4	2022-10	Post-pandemic recovery	Cytology (Pap)	40-44	40	Not performed	Satisfactory	NILM	NILM	Negative (NILM)	
P09415	2022	2022-Q4	2022-10	Post-pandemic recovery	Cytology (Pap)	40-44	44	Not performed	Satisfactory	NILM	NILM	Negative (NILM)	
P09416	2022	2022-Q4	2022-10	Post-pandemic recovery	Cytology (Pap)	35-39	38	Not performed	Satisfactory	Positive	HSIL	High-grade squamous	
P09417	2022	2022-Q4	2022-10	Post-pandemic recovery	Cytology (Pap)	60-64	61	Not performed	Satisfactory	NILM	NILM	Negative (NILM)	
P09418	2022	2022-Q4	2022-10	Post-pandemic recovery	Cytology (Pap)	25-29	28	Not performed	Satisfactory	NILM	NILM	Negative (NILM)	
P09419	2022	2022-Q4	2022-10	Post-pandemic recovery	Cytology (Pap)	30-34	33	Not performed	Satisfactory	NILM	NILM	Negative (NILM)	Candida
P09420	2022	2022-Q4	2022-10	Post-pandemic recovery	Cytology (Pap)	55-59	59	Not performed	Satisfactory	NILM	NILM	Negative (NILM)	
P09421	2022	2022-Q4	2022-10	Post-pandemic recovery	Cytology (Pap)	60-64	62	Not performed	Satisfactory	NILM	NILM	Negative (NILM)	Candida
P09422	2022	2022-Q4	2022-10	Post-pandemic recovery	Cytology (Pap)	55-59	58	Not performed	Satisfactory	NILM	NILM	Negative (NILM)	
P09423	2022	2022-Q4	2022-10	Post-pandemic recovery	Cytology (Pap)	35-39	37	Not performed	Satisfactory	NILM	NILM	Negative (NILM)	
P09424	2022	2022-Q4	2022-10	Post-pandemic recovery	Cytology (Pap)	55-59	56	Not performed	Satisfactory	NILM	NILM	Negative (NILM)	
P09425	2022	2022-Q4	2022-10	Post-pandemic recovery	Cytology (Pap)	35-39	37	Not performed	Satisfactory	NILM	NILM	Negative (NILM)	
P09426	2022	2022-Q4	2022-10	Post-pandemic recovery	Cytology (Pap)	35-39	36	Not performed	Satisfactory	Positive	ASC-US	Low-grade / equivocal squamous	
P09427	2022	2022-Q4	2022-10	Post-pandemic recovery	Cytology (Pap)	60-64	63	Not performed	Satisfactory	NILM	NILM	Negative (NILM)	
P09428	2022	2022-Q4	2022-10	Post-pandemic recovery	Cytology (Pap)	55-59	55	Not performed	Satisfactory	NILM	NILM	Negative (NILM)	
P09429	2022	2022-Q4	2022-10	Post-pandemic recovery	Cytology (Pap)	50-54	52	Not performed	Satisfactory	NILM	NILM	Negative (NILM)	
P09430	2022	2022-Q4	2022-10	Post-pandemic recovery	Cytology (Pap)	35-39	36	Not performed	Satisfactory	NILM	NILM	Negative (NILM)	
P09431	2022	2022-Q4	2022-10	Post-pandemic recovery	Cytology (Pap)	40-44	41	Not performed	Satisfactory	NILM	NILM	Negative (NILM)	
P09432	2022	2022-Q4	2022-10	Post-pandemic recovery	Cytology (Pap)	60-64	61	Not performed	Satisfactory	NILM	NILM	Negative (NILM)	
P09433	2022	2022-Q4	2022-10	Post-pandemic recovery	Cytology (Pap)	55-59	56	Not performed	Satisfactory	NILM	NILM	Negative (NILM)	
P09434	2022	2022-Q4	2022-10	Post-pandemic recovery	Cytology (Pap)	50-54	52	Not performed	Satisfactory	NILM	NILM	Negative (NILM)	
P09435	2022	2022-Q4	2022-10	Post-pandemic recovery	Cytology (Pap)	60-64	61	Not performed	Satisfactory	Positive	ASC-H	High-grade squamous	
P09436	2022	2022-Q4	2022-10	Post-pandemic recovery	Cytology (Pap)	40-44	44	Not performed	Satisfactory	NILM	NILM	Negative (NILM)	
P09437	2022	2022-Q4	2022-10	Post-pandemic recovery	Cytology (Pap)	50-54	51	Not performed	Satisfactory	NILM	NILM	Negative (NILM)	
P09438	2022	2022-Q4	2022-10	Post-pandemic recovery	Cytology (Pap)	35-39	37	Not performed	Satisfactory	NILM	NILM	Negative (NILM)	

patient_id	year	quarter	month	period	primary_test	age_group	age_years	hpv_result	adequacy	result	bethesda	grade	infection
P09439	2022	2022-Q4	2022-10	Post-pandemic recovery	Cytology (Pap)	40-44	41	Not performed	Satisfactory	NILM	NILM	Negative (NILM)	
P09440	2022	2022-Q4	2022-10	Post-pandemic recovery	Cytology (Pap)	40-44	40	Not performed	Satisfactory	NILM	NILM	Negative (NILM)	
P09441	2022	2022-Q4	2022-10	Post-pandemic recovery	Cytology (Pap)	25-29	27	Not performed	Satisfactory	NILM	NILM	Negative (NILM)	
P09442	2022	2022-Q4	2022-10	Post-pandemic recovery	Cytology (Pap)	30-34	32	Not performed	Satisfactory	NILM	NILM	Negative (NILM)	
P09443	2022	2022-Q4	2022-10	Post-pandemic recovery	Cytology (Pap)	45-49	49	Not performed	Satisfactory	NILM	NILM	Negative (NILM)	
P09444	2022	2022-Q4	2022-10	Post-pandemic recovery	Cytology (Pap)	35-39	36	Not performed	Satisfactory	NILM	NILM	Negative (NILM)	
P09445	2022	2022-Q4	2022-10	Post-pandemic recovery	Cytology (Pap)	50-54	50	Not performed	Satisfactory	NILM	NILM	Negative (NILM)	
P09446	2022	2022-Q4	2022-10	Post-pandemic recovery	Cytology (Pap)	25-29	27	Not performed	Satisfactory	NILM	NILM	Negative (NILM)	
P09447	2022	2022-Q4	2022-10	Post-pandemic recovery	Cytology (Pap)	35-39	38	Not performed	Satisfactory	Positive	LSIL	Low-grade / equivocal squamous	
P09448	2022	2022-Q4	2022-10	Post-pandemic recovery	Cytology (Pap)	40-44	44	Not performed	Satisfactory	NILM	NILM	Negative (NILM)	
P09449	2022	2022-Q4	2022-10	Post-pandemic recovery	Cytology (Pap)	50-54	51	Not performed	Satisfactory	NILM	NILM	Negative (NILM)	
P09450	2022	2022-Q4	2022-10	Post-pandemic recovery	Cytology (Pap)	35-39	38	Not performed	Satisfactory	NILM	NILM	Negative (NILM)	
P09451	2022	2022-Q4	2022-10	Post-pandemic recovery	Cytology (Pap)	25-29	29	Not performed	Satisfactory	NILM	NILM	Negative (NILM)	
P09452	2022	2022-Q4	2022-10	Post-pandemic recovery	Cytology (Pap)	60-64	63	Not performed	Satisfactory	Positive	ASC-US	Low-grade / equivocal squamous	
P09453	2022	2022-Q4	2022-10	Post-pandemic recovery	Cytology (Pap)	60-64	60	Not performed	Satisfactory	NILM	NILM	Negative (NILM)	
P09454	2022	2022-Q4	2022-10	Post-pandemic recovery	Cytology (Pap)	40-44	41	Not performed	Satisfactory	NILM	NILM	Negative (NILM)	
P09455	2022	2022-Q4	2022-10	Post-pandemic recovery	Cytology (Pap)	35-39	35	Not performed	Satisfactory	NILM	NILM	Negative (NILM)	
P09456	2022	2022-Q4	2022-10	Post-pandemic recovery	Cytology (Pap)	35-39	36	Not performed	Satisfactory	NILM	NILM	Negative (NILM)	
P09457	2022	2022-Q4	2022-10	Post-pandemic recovery	Cytology (Pap)	40-44	43	Not performed	Satisfactory	NILM	NILM	Negative (NILM)	
P09458	2022	2022-Q4	2022-10	Post-pandemic recovery	Cytology (Pap)	50-54	53	Not performed	Satisfactory	Positive	ASC-US	Low-grade / equivocal squamous	
P09459	2022	2022-Q4	2022-10	Post-pandemic recovery	Cytology (Pap)	50-54	54	Not performed	Satisfactory	Positive	LSIL	Low-grade / equivocal squamous	
P09460	2022	2022-Q4	2022-10	Post-pandemic recovery	Cytology (Pap)	25-29	29	Not performed	Satisfactory	NILM	NILM	Negative (NILM)	
P09461	2022	2022-Q4	2022-10	Post-pandemic recovery	Cytology (Pap)	35-39	39	Not performed	Satisfactory	NILM	NILM	Negative (NILM)	
P09462	2022	2022-Q4	2022-10	Post-pandemic recovery	Cytology (Pap)	60-64	62	Not performed	Satisfactory	NILM	NILM	Negative (NILM)	
P09463	2022	2022-Q4	2022-10	Post-pandemic recovery	Cytology (Pap)	30-34	30	Not performed	Satisfactory	NILM	NILM	Negative (NILM)	
P09464	2022	2022-Q4	2022-10	Post-pandemic recovery	Cytology (Pap)	60-64	60	Not performed	Satisfactory	NILM	NILM	Negative (NILM)	
P09465	2022	2022-Q4	2022-10	Post-pandemic recovery	Cytology (Pap)	30-34	30	Not performed	Satisfactory	NILM	NILM	Negative (NILM)	
P09466	2022	2022-Q4	2022-10	Post-pandemic recovery	Cytology (Pap)	35-39	36	Not performed	Satisfactory	NILM	NILM	Negative (NILM)	
P09467	2022	2022-Q4	2022-10	Post-pandemic recovery	Cytology (Pap)	40-44	42	Not performed	Satisfactory	NILM	NILM	Negative (NILM)	
P09468	2022	2022-Q4	2022-10	Post-pandemic recovery	Cytology (Pap)	40-44	43	Not performed	Satisfactory	NILM	NILM	Negative (NILM)	
P09469	2022	2022-Q4	2022-10	Post-pandemic recovery	Cytology (Pap)	40-44	43	Not performed	Satisfactory	NILM	NILM	Negative (NILM)	
P09470	2022	2022-Q4	2022-10	Post-pandemic recovery	Cytology (Pap)	40-44	41	Not performed	Satisfactory	Positive	ASC-US	Low-grade / equivocal squamous	
P09471	2022	2022-Q4	2022-10	Post-pandemic recovery	Cytology (Pap)	45-49	48	Not performed	Satisfactory	NILM	NILM	Negative (NILM)	
P09472	2022	2022-Q4	2022-10	Post-pandemic recovery	Cytology (Pap)	30-34	30	Not performed	Satisfactory	Positive	LSIL	Low-grade / equivocal squamous	
P09473	2022	2022-Q4	2022-10	Post-pandemic recovery	Cytology (Pap)	30-34	34	Not performed	Unsatisfactory	Unsatisfactory	Unsatisfactory	Unsatisfactory	
P09474	2022	2022-Q4	2022-10	Post-pandemic recovery	Cytology (Pap)	35-39	36	Not performed	Satisfactory	Positive	ASC-US	Low-grade / equivocal squamous	
P09475	2022	2022-Q4	2022-10	Post-pandemic recovery	Cytology (Pap)	40-44	42	Not performed	Satisfactory	NILM	NILM	Negative (NILM)	
P09476	2022	2022-Q4	2022-10	Post-pandemic recovery	Cytology (Pap)	50-54	53	Not performed	Satisfactory	NILM	NILM	Negative (NILM)	
P09477	2022	2022-Q4	2022-10	Post-pandemic recovery	Cytology (Pap)	60-64	62	Not performed	Unsatisfactory	Unsatisfactory	Unsatisfactory	Unsatisfactory	
P09478	2022	2022-Q4	2022-10	Post-pandemic recovery	Cytology (Pap)	40-44	41	Not performed	Satisfactory	NILM	NILM	Negative (NILM)	
P09479	2022	2022-Q4	2022-10	Post-pandemic recovery	Cytology (Pap)	60-64	60	Not performed	Satisfactory	Positive	HSIL	High-grade squamous	
P09480	2022	2022-Q4	2022-10	Post-pandemic recovery	Cytology (Pap)	30-34	31	Not performed	Satisfactory	NILM	NILM	Negative (NILM)	
P09481	2022	2022-Q4	2022-10	Post-pandemic recovery	Cytology (Pap)	25-29	29	Not performed	Satisfactory	NILM	NILM	Negative (NILM)	
P09482	2022	2022-Q4	2022-10	Post-pandemic recovery	Cytology (Pap)	40-44	41	Not performed	Satisfactory	NILM	NILM	Negative (NILM)	
P09483	2022	2022-Q4	2022-10	Post-pandemic recovery	Cytology (Pap)	45-49	46	Not performed	Unsatisfactory	Unsatisfactory	Unsatisfactory	Unsatisfactory	
P09484	2022	2022-Q4	2022-10	Post-pandemic recovery	Cytology (Pap)	40-44	42	Not performed	Satisfactory	NILM	NILM	Negative (NILM)	
P09485	2022	2022-Q4	2022-10	Post-pandemic recovery	Cytology (Pap)	35-39	39	Not performed	Unsatisfactory	Unsatisfactory	Unsatisfactory	Unsatisfactory	
P09486	2022	2022-Q4	2022-10	Post-pandemic recovery	Cytology (Pap)	55-59	58	Not performed	Satisfactory	NILM	NILM	Negative (NILM)	
P09487	2022	2022-Q4	2022-10	Post-pandemic recovery	Cytology (Pap)	40-44	40	Not performed	Satisfactory	NILM	NILM	Negative (NILM)	
P09488	2022	2022-Q4	2022-10	Post-pandemic recovery	Cytology (Pap)	35-39	37	Not performed	Unsatisfactory	Unsatisfactory	Unsatisfactory	Unsatisfactory	
P09489	2022	2022-Q4	2022-10	Post-pandemic recovery	Cytology (Pap)	45-49	47	Not performed	Satisfactory	NILM	NILM	Negative (NILM)	
P09490	2022	2022-Q4	2022-10	Post-pandemic recovery	Cytology (Pap)	45-49	45	Not performed	Satisfactory	NILM	NILM	Negative (NILM)	
P09491	2022	2022-Q4	2022-10	Post-pandemic recovery	Cytology (Pap)	30-34	32	Not performed	Satisfactory	NILM	NILM	Negative (NILM)	
P09492	2022	2022-Q4	2022-10	Post-pandemic recovery	Cytology (Pap)	60-64	61	Not performed	Satisfactory	NILM	NILM	Negative (NILM)	
P09493	2022	2022-Q4	2022-10	Post-pandemic recovery	Cytology (Pap)	25-29	29	Not performed	Satisfactory	NILM	NILM	Negative (NILM)	
P09494	2022	2022-Q4	2022-10	Post-pandemic recovery	Cytology (Pap)	30-34	34	Not performed	Satisfactory	Positive	ASC-H	High-grade squamous	
P09495	2022	2022-Q4	2022-10	Post-pandemic recovery	Cytology (Pap)	60-64	61	Not performed	Satisfactory	NILM	NILM	Negative (NILM)	
P09496	2022	2022-Q4	2022-10	Post-pandemic recovery	Cytology (Pap)	30-34	34	Not performed	Satisfactory	NILM	NILM	Negative (NILM)	
P09497	2022	2022-Q4	2022-10	Post-pandemic recovery	Cytology (Pap)	55-59	55	Not performed	Satisfactory	NILM	NILM	Negative (NILM)	
P09498	2022	2022-Q4	2022-10	Post-pandemic recovery	Cytology (Pap)	40-44	42	Not performed	Satisfactory	Positive	ASC-US	Low-grade / equivocal squamous	
P09499	2022	2022-Q4	2022-10	Post-pandemic recovery	Cytology (Pap)	35-39	38	Not performed	Satisfactory	Positive	ASC-US	Low-grade / equivocal squamous	
P09500	2022	2022-Q4	2022-10	Post-pandemic recovery	Cytology (Pap)	40-44	42	Not performed	Satisfactory	NILM	NILM	Negative (NILM)	
P09501	2022	2022-Q4	2022-10	Post-pandemic recovery	Cytology (Pap)	25-29	28	Not performed	Satisfactory	NILM	NILM	Negative (NILM)	
P09502	2022	2022-Q4	2022-10	Post-pandemic recovery	Cytology (Pap)	60-64	61	Not performed	Satisfactory	NILM	NILM	Negative (NILM)	
P09503	2022	2022-Q4	2022-10	Post-pandemic recovery	Cytology (Pap)	30-34	31	Not performed	Satisfactory	NILM	NILM	Negative (NILM)	
P09504	2022	2022-Q4	2022-10	Post-pandemic recovery	Cytology (Pap)	45-49	48	Not performed	Satisfactory	NILM	NILM	Negative (NILM)	
P09505	2022	2022-Q4	2022-11	Post-pandemic recovery	Cytology (Pap)	50-54	51	Not performed	Satisfactory	NILM	NILM	Negative (NILM)	
P09506	2022	2022-Q4	2022-11	Post-pandemic recovery	Cytology (Pap)	40-44	40	Not performed	Satisfactory	NILM	NILM	Negative (NILM)	
P09507	2022	2022-Q4	2022-11	Post-pandemic recovery	Cytology (Pap)	35-39	38	Not performed	Satisfactory	NILM	NILM	Negative (NILM)	
P09508	2022	2022-Q4	2022-11	Post-pandemic recovery	Cytology (Pap)	40-44	41	Not performed	Satisfactory	NILM	NILM	Negative (NILM)	
P09509	2022	2022-Q4	2022-11	Post-pandemic recovery	Cytology (Pap)	50-54	54	Not performed	Satisfactory	NILM	NILM	Negative (NILM)	
P09510	2022	2022-Q4	2022-11	Post-pandemic recovery	Cytology (Pap)	55-59	55	Not performed	Satisfactory	NILM	NILM	Negative (NILM)	
P09511	2022	2022-Q4	2022-11	Post-pandemic recovery	Cytology (Pap)	35-39	39	Not performed	Satisfactory	NILM	NILM	Negative (NILM)	
P09512	2022	2022-Q4	2022-11	Post-pandemic recovery	Cytology (Pap)	55-59	56	Not performed	Satisfactory	NILM	NILM	Negative (NILM)	
P09513	2022	2022-Q4	2022-11	Post-pandemic recovery	Cytology (Pap)	25-29	27	Not performed	Satisfactory	NILM	NILM	Negative (NILM)	
P09514	2022	2022-Q4	2022-11	Post-pandemic recovery	Cytology (Pap)	40-44	42	Not performed	Satisfactory	NILM	NILM	Negative (NILM)	
P09515	2022	2022-Q4	2022-11	Post-pandemic recovery	Cytology (Pap)	30-34	30	Not performed	Satisfactory	NILM	NILM	Negative (NILM)	
P09516	2022	2022-Q4	2022-11	Post-pandemic recovery	Cytology (Pap)	40-44	43	Not performed	Satisfactory	NILM	NILM	Negative (NILM)	
P09517	2022	2022-Q4	2022-11	Post-pandemic recovery	Cytology (Pap)	55-59	59	Not performed	Unsatisfactory	Unsatisfactory	Unsatisfactory	Unsatisfactory	
P09518	2022	2022-Q4	2022-11	Post-pandemic recovery	Cytology (Pap)	30-34	32	Not performed	Satisfactory	Positive	LSIL	Low-grade / equivocal squamous	

patient_id	year	quarter	month	period	primary_test	age_group	age_years	hpv_result	adequacy	result	bethesda	grade	infection
P09519	2022	2022-Q4	2022-11	Post-pandemic recovery	Cytology (Pap)	35-39	37	Not performed	Satisfactory	NILM	NILM	Negative (NILM)	
P09520	2022	2022-Q4	2022-11	Post-pandemic recovery	Cytology (Pap)	45-49	49	Not performed	Satisfactory	NILM	NILM	Negative (NILM)	Candida
P09521	2022	2022-Q4	2022-11	Post-pandemic recovery	Cytology (Pap)	45-49	46	Not performed	Satisfactory	NILM	NILM	Negative (NILM)	
P09522	2022	2022-Q4	2022-11	Post-pandemic recovery	Cytology (Pap)	40-44	44	Not performed	Satisfactory	NILM	NILM	Negative (NILM)	
P09523	2022	2022-Q4	2022-11	Post-pandemic recovery	Cytology (Pap)	50-54	50	Not performed	Satisfactory	Positive	LSIL	Low-grade / equivocal squamous	
P09524	2022	2022-Q4	2022-11	Post-pandemic recovery	Cytology (Pap)	55-59	55	Not performed	Satisfactory	NILM	NILM	Negative (NILM)	
P09525	2022	2022-Q4	2022-11	Post-pandemic recovery	Cytology (Pap)	40-44	44	Not performed	Unsatisfactory	Unsatisfactory	Unsatisfactory	Unsatisfactory	
P09526	2022	2022-Q4	2022-11	Post-pandemic recovery	Cytology (Pap)	50-54	52	Not performed	Satisfactory	NILM	NILM	Negative (NILM)	
P09527	2022	2022-Q4	2022-11	Post-pandemic recovery	Cytology (Pap)	30-34	31	Not performed	Satisfactory	NILM	NILM	Negative (NILM)	
P09528	2022	2022-Q4	2022-11	Post-pandemic recovery	Cytology (Pap)	40-44	42	Not performed	Satisfactory	NILM	NILM	Negative (NILM)	
P09529	2022	2022-Q4	2022-11	Post-pandemic recovery	Cytology (Pap)	35-39	37	Not performed	Satisfactory	NILM	NILM	Negative (NILM)	Trichomonas
P09530	2022	2022-Q4	2022-11	Post-pandemic recovery	Cytology (Pap)	45-49	47	Not performed	Satisfactory	NILM	NILM	Negative (NILM)	
P09531	2022	2022-Q4	2022-11	Post-pandemic recovery	Cytology (Pap)	60-64	62	Not performed	Satisfactory	NILM	NILM	Negative (NILM)	
P09532	2022	2022-Q4	2022-11	Post-pandemic recovery	Cytology (Pap)	35-39	37	Not performed	Satisfactory	NILM	NILM	Negative (NILM)	
P09533	2022	2022-Q4	2022-11	Post-pandemic recovery	Cytology (Pap)	55-59	57	Not performed	Satisfactory	NILM	NILM	Negative (NILM)	
P09534	2022	2022-Q4	2022-11	Post-pandemic recovery	Cytology (Pap)	25-29	28	Not performed	Satisfactory	NILM	NILM	Negative (NILM)	
P09535	2022	2022-Q4	2022-11	Post-pandemic recovery	Cytology (Pap)	50-54	50	Not performed	Satisfactory	NILM	NILM	Negative (NILM)	
P09536	2022	2022-Q4	2022-11	Post-pandemic recovery	Cytology (Pap)	40-44	41	Not performed	Satisfactory	NILM	NILM	Negative (NILM)	
P09537	2022	2022-Q4	2022-11	Post-pandemic recovery	Cytology (Pap)	35-39	36	Not performed	Satisfactory	NILM	NILM	Negative (NILM)	Other
P09538	2022	2022-Q4	2022-11	Post-pandemic recovery	Cytology (Pap)	45-49	45	Not performed	Satisfactory	NILM	NILM	Negative (NILM)	
P09539	2022	2022-Q4	2022-11	Post-pandemic recovery	Cytology (Pap)	50-54	52	Not performed	Satisfactory	NILM	NILM	Negative (NILM)	
P09540	2022	2022-Q4	2022-11	Post-pandemic recovery	Cytology (Pap)	50-54	54	Not performed	Satisfactory	NILM	NILM	Negative (NILM)	
P09541	2022	2022-Q4	2022-11	Post-pandemic recovery	Cytology (Pap)	40-44	43	Not performed	Satisfactory	NILM	NILM	Negative (NILM)	
P09542	2022	2022-Q4	2022-11	Post-pandemic recovery	Cytology (Pap)	40-44	43	Not performed	Satisfactory	Positive	ASC-US	Low-grade / equivocal squamous	
P09543	2022	2022-Q4	2022-11	Post-pandemic recovery	Cytology (Pap)	25-29	29	Not performed	Satisfactory	NILM	NILM	Negative (NILM)	
P09544	2022	2022-Q4	2022-11	Post-pandemic recovery	Cytology (Pap)	25-29	27	Not performed	Satisfactory	NILM	NILM	Negative (NILM)	
P09545	2022	2022-Q4	2022-11	Post-pandemic recovery	Cytology (Pap)	40-44	41	Not performed	Unsatisfactory	Unsatisfactory	Unsatisfactory	Unsatisfactory	
P09546	2022	2022-Q4	2022-11	Post-pandemic recovery	Cytology (Pap)	50-54	50	Not performed	Satisfactory	NILM	NILM	Negative (NILM)	
P09547	2022	2022-Q4	2022-11	Post-pandemic recovery	Cytology (Pap)	45-49	47	Not performed	Satisfactory	NILM	NILM	Negative (NILM)	
P09548	2022	2022-Q4	2022-11	Post-pandemic recovery	Cytology (Pap)	40-44	43	Not performed	Satisfactory	NILM	NILM	Negative (NILM)	Candida
P09549	2022	2022-Q4	2022-11	Post-pandemic recovery	Cytology (Pap)	45-49	45	Not performed	Satisfactory	NILM	NILM	Negative (NILM)	
P09550	2022	2022-Q4	2022-11	Post-pandemic recovery	Cytology (Pap)	30-34	34	Not performed	Satisfactory	NILM	NILM	Negative (NILM)	
P09551	2022	2022-Q4	2022-11	Post-pandemic recovery	Cytology (Pap)	35-39	37	Not performed	Satisfactory	Positive	LSIL	Low-grade / equivocal squamous	Trichomonas
P09552	2022	2022-Q4	2022-11	Post-pandemic recovery	Cytology (Pap)	30-34	32	Not performed	Satisfactory	NILM	NILM	Negative (NILM)	
P09553	2022	2022-Q4	2022-11	Post-pandemic recovery	Cytology (Pap)	30-34	30	Not performed	Satisfactory	Positive	LSIL	Low-grade / equivocal squamous	
P09554	2022	2022-Q4	2022-11	Post-pandemic recovery	Cytology (Pap)	60-64	64	Not performed	Satisfactory	NILM	NILM	Negative (NILM)	
P09555	2022	2022-Q4	2022-11	Post-pandemic recovery	Cytology (Pap)	35-39	39	Not performed	Satisfactory	Positive	ASC-US	Low-grade / equivocal squamous	
P09556	2022	2022-Q4	2022-11	Post-pandemic recovery	Cytology (Pap)	40-44	43	Not performed	Satisfactory	NILM	NILM	Negative (NILM)	
P09557	2022	2022-Q4	2022-11	Post-pandemic recovery	Cytology (Pap)	35-39	35	Not performed	Satisfactory	Positive	LSIL	Low-grade / equivocal squamous	
P09558	2022	2022-Q4	2022-11	Post-pandemic recovery	Cytology (Pap)	35-39	38	Not performed	Satisfactory	NILM	NILM	Negative (NILM)	
P09559	2022	2022-Q4	2022-11	Post-pandemic recovery	Cytology (Pap)	35-39	39	Not performed	Satisfactory	NILM	NILM	Negative (NILM)	Candida
P09560	2022	2022-Q4	2022-11	Post-pandemic recovery	Cytology (Pap)	25-29	27	Not performed	Satisfactory	Positive	HSIL	High-grade squamous	
P09561	2022	2022-Q4	2022-11	Post-pandemic recovery	Cytology (Pap)	40-44	41	Not performed	Satisfactory	NILM	NILM	Negative (NILM)	
P09562	2022	2022-Q4	2022-11	Post-pandemic recovery	Cytology (Pap)	45-49	46	Not performed	Satisfactory	NILM	NILM	Negative (NILM)	
P09563	2022	2022-Q4	2022-11	Post-pandemic recovery	Cytology (Pap)	45-49	49	Not performed	Satisfactory	Positive	ASC-US	Low-grade / equivocal squamous	Candida
P09564	2022	2022-Q4	2022-11	Post-pandemic recovery	Cytology (Pap)	50-54	50	Not performed	Satisfactory	NILM	NILM	Negative (NILM)	
P09565	2022	2022-Q4	2022-11	Post-pandemic recovery	Cytology (Pap)	45-49	46	Not performed	Satisfactory	Positive	HSIL	High-grade squamous	
P09566	2022	2022-Q4	2022-11	Post-pandemic recovery	Cytology (Pap)	30-34	33	Not performed	Satisfactory	NILM	NILM	Negative (NILM)	
P09567	2022	2022-Q4	2022-11	Post-pandemic recovery	Cytology (Pap)	60-64	62	Not performed	Satisfactory	NILM	NILM	Negative (NILM)	
P09568	2022	2022-Q4	2022-11	Post-pandemic recovery	Cytology (Pap)	30-34	32	Not performed	Satisfactory	Positive	ASC-US	Low-grade / equivocal squamous	
P09569	2022	2022-Q4	2022-11	Post-pandemic recovery	Cytology (Pap)	30-34	34	Not performed	Satisfactory	NILM	NILM	Negative (NILM)	Trichomonas
P09570	2022	2022-Q4	2022-11	Post-pandemic recovery	Cytology (Pap)	40-44	40	Not performed	Satisfactory	Positive	ASC-H	High-grade squamous	
P09571	2022	2022-Q4	2022-11	Post-pandemic recovery	Cytology (Pap)	30-34	33	Not performed	Satisfactory	Positive	LSIL	Low-grade / equivocal squamous	
P09572	2022	2022-Q4	2022-11	Post-pandemic recovery	Cytology (Pap)	40-44	40	Not performed	Satisfactory	NILM	NILM	Negative (NILM)	Candida
P09573	2022	2022-Q4	2022-11	Post-pandemic recovery	Cytology (Pap)	35-39	38	Not performed	Satisfactory	NILM	NILM	Negative (NILM)	
P09574	2022	2022-Q4	2022-11	Post-pandemic recovery	Cytology (Pap)	35-39	38	Not performed	Satisfactory	Positive	LSIL	Low-grade / equivocal squamous	
P09575	2022	2022-Q4	2022-11	Post-pandemic recovery	Cytology (Pap)	35-39	39	Not performed	Satisfactory	NILM	NILM	Negative (NILM)	
P09576	2022	2022-Q4	2022-11	Post-pandemic recovery	Cytology (Pap)	35-39	38	Not performed	Satisfactory	NILM	NILM	Negative (NILM)	
P09577	2022	2022-Q4	2022-11	Post-pandemic recovery	Cytology (Pap)	25-29	27	Not performed	Satisfactory	NILM	NILM	Negative (NILM)	
P09578	2022	2022-Q4	2022-11	Post-pandemic recovery	Cytology (Pap)	40-44	42	Not performed	Satisfactory	NILM	NILM	Negative (NILM)	
P09579	2022	2022-Q4	2022-11	Post-pandemic recovery	Cytology (Pap)	35-39	35	Not performed	Unsatisfactory	Unsatisfactory	Unsatisfactory	Unsatisfactory	
P09580	2022	2022-Q4	2022-11	Post-pandemic recovery	Cytology (Pap)	25-29	27	Not performed	Satisfactory	NILM	NILM	Negative (NILM)	
P09581	2022	2022-Q4	2022-11	Post-pandemic recovery	Cytology (Pap)	30-34	34	Not performed	Satisfactory	Positive	LSIL	Low-grade / equivocal squamous	
P09582	2022	2022-Q4	2022-11	Post-pandemic recovery	Cytology (Pap)	40-44	41	Not performed	Satisfactory	NILM	NILM	Negative (NILM)	
P09583	2022	2022-Q4	2022-11	Post-pandemic recovery	Cytology (Pap)	25-29	29	Not performed	Satisfactory	NILM	NILM	Negative (NILM)	
P09584	2022	2022-Q4	2022-11	Post-pandemic recovery	Cytology (Pap)	55-59	57	Not performed	Satisfactory	NILM	NILM	Negative (NILM)	
P09585	2022	2022-Q4	2022-11	Post-pandemic recovery	Cytology (Pap)	40-44	40	Not performed	Satisfactory	Positive	ASC-US	Low-grade / equivocal squamous	
P09586	2022	2022-Q4	2022-11	Post-pandemic recovery	Cytology (Pap)	25-29	29	Not performed	Satisfactory	NILM	NILM	Negative (NILM)	
P09587	2022	2022-Q4	2022-11	Post-pandemic recovery	Cytology (Pap)	40-44	42	Not performed	Satisfactory	NILM	NILM	Negative (NILM)	
P09588	2022	2022-Q4	2022-11	Post-pandemic recovery	Cytology (Pap)	40-44	41	Not performed	Satisfactory	NILM	NILM	Negative (NILM)	
P09589	2022	2022-Q4	2022-11	Post-pandemic recovery	Cytology (Pap)	40-44	41	Not performed	Satisfactory	NILM	NILM	Negative (NILM)	
P09590	2022	2022-Q4	2022-11	Post-pandemic recovery	Cytology (Pap)	25-29	27	Not performed	Satisfactory	NILM	NILM	Negative (NILM)	
P09591	2022	2022-Q4	2022-11	Post-pandemic recovery	Cytology (Pap)	30-34	32	Not performed	Satisfactory	NILM	NILM	Negative (NILM)	
P09592	2022	2022-Q4	2022-11	Post-pandemic recovery	Cytology (Pap)	40-44	42	Not performed	Satisfactory	NILM	NILM	Negative (NILM)	Candida
P09593	2022	2022-Q4	2022-11	Post-pandemic recovery	Cytology (Pap)	30-34	30	Not performed	Satisfactory	NILM	NILM	Negative (NILM)	
P09594	2022	2022-Q4	2022-11	Post-pandemic recovery	Cytology (Pap)	45-49	48	Not performed	Satisfactory	NILM	NILM	Negative (NILM)	
P09595	2022	2022-Q4	2022-11	Post-pandemic recovery	Cytology (Pap)	40-44	43	Not performed	Satisfactory	NILM	NILM	Negative (NILM)	
P09596	2022	2022-Q4	2022-11	Post-pandemic recovery	Cytology (Pap)	30-34	32	Not performed	Satisfactory	NILM	NILM	Negative (NILM)	
P09597	2022	2022-Q4	2022-11	Post-pandemic recovery	Cytology (Pap)	40-44	41	Not performed	Satisfactory	Positive	HSIL	High-grade squamous	Gardnerella
P09598	2022	2022-Q4	2022-11	Post-pandemic recovery	Cytology (Pap)	50-54	50	Not performed	Satisfactory	NILM	NILM	Negative (NILM)	

patient_id	year	quarter	month	period	primary_test	age_group	age_years	hpv_result	adequacy	result	bethesda	grade	infection
P09599	2022	2022-Q4	2022-11	Post-pandemic recovery	Cytology (Pap)	30-34	30	Not performed	Satisfactory	NILM	NILM	Negative (NILM)	
P09600	2022	2022-Q4	2022-11	Post-pandemic recovery	Cytology (Pap)	60-64	60	Not performed	Unsatisfactory	Unsatisfactory	Unsatisfactory	Unsatisfactory	
P09601	2022	2022-Q4	2022-11	Post-pandemic recovery	Cytology (Pap)	35-39	37	Not performed	Satisfactory	Positive	HSIL	High-grade squamous	
P09602	2022	2022-Q4	2022-11	Post-pandemic recovery	Cytology (Pap)	30-34	31	Not performed	Satisfactory	NILM	NILM	Negative (NILM)	
P09603	2022	2022-Q4	2022-11	Post-pandemic recovery	Cytology (Pap)	60-64	60	Not performed	Satisfactory	NILM	NILM	Negative (NILM)	
P09604	2022	2022-Q4	2022-11	Post-pandemic recovery	Cytology (Pap)	25-29	29	Not performed	Satisfactory	NILM	NILM	Negative (NILM)	
P09605	2022	2022-Q4	2022-11	Post-pandemic recovery	Cytology (Pap)	25-29	25	Not performed	Satisfactory	Positive	ASC-H	High-grade squamous	
P09606	2022	2022-Q4	2022-11	Post-pandemic recovery	Cytology (Pap)	30-34	31	Not performed	Satisfactory	NILM	NILM	Negative (NILM)	
P09607	2022	2022-Q4	2022-11	Post-pandemic recovery	Cytology (Pap)	30-34	33	Not performed	Satisfactory	NILM	NILM	Negative (NILM)	
P09608	2022	2022-Q4	2022-11	Post-pandemic recovery	Cytology (Pap)	40-44	44	Not performed	Satisfactory	NILM	NILM	Negative (NILM)	
P09609	2022	2022-Q4	2022-11	Post-pandemic recovery	Cytology (Pap)	30-34	33	Not performed	Satisfactory	NILM	NILM	Negative (NILM)	
P09610	2022	2022-Q4	2022-11	Post-pandemic recovery	Cytology (Pap)	35-39	36	Not performed	Unsatisfactory	Unsatisfactory	Unsatisfactory	Unsatisfactory	
P09611	2022	2022-Q4	2022-11	Post-pandemic recovery	Cytology (Pap)	60-64	62	Not performed	Satisfactory	Positive	ASC-H	High-grade squamous	
P09612	2022	2022-Q4	2022-11	Post-pandemic recovery	Cytology (Pap)	40-44	43	Not performed	Satisfactory	NILM	NILM	Negative (NILM)	
P09613	2022	2022-Q4	2022-11	Post-pandemic recovery	Cytology (Pap)	25-29	29	Not performed	Satisfactory	NILM	NILM	Negative (NILM)	
P09614	2022	2022-Q4	2022-11	Post-pandemic recovery	Cytology (Pap)	35-39	38	Not performed	Satisfactory	Positive	ASC-US	Low-grade / equivocal squamous	
P09615	2022	2022-Q4	2022-11	Post-pandemic recovery	Cytology (Pap)	35-39	35	Not performed	Satisfactory	NILM	NILM	Negative (NILM)	
P09616	2022	2022-Q4	2022-11	Post-pandemic recovery	Cytology (Pap)	35-39	37	Not performed	Satisfactory	NILM	NILM	Negative (NILM)	
P09617	2022	2022-Q4	2022-11	Post-pandemic recovery	Cytology (Pap)	60-64	60	Not performed	Satisfactory	NILM	NILM	Negative (NILM)	
P09618	2022	2022-Q4	2022-11	Post-pandemic recovery	Cytology (Pap)	40-44	44	Not performed	Satisfactory	NILM	NILM	Negative (NILM)	
P09619	2022	2022-Q4	2022-11	Post-pandemic recovery	Cytology (Pap)	45-49	47	Not performed	Satisfactory	NILM	NILM	Negative (NILM)	
P09620	2022	2022-Q4	2022-11	Post-pandemic recovery	Cytology (Pap)	50-54	52	Not performed	Satisfactory	NILM	NILM	Negative (NILM)	
P09621	2022	2022-Q4	2022-11	Post-pandemic recovery	Cytology (Pap)	40-44	43	Not performed	Satisfactory	NILM	NILM	Negative (NILM)	
P09622	2022	2022-Q4	2022-11	Post-pandemic recovery	Cytology (Pap)	35-39	39	Not performed	Satisfactory	NILM	NILM	Negative (NILM)	
P09623	2022	2022-Q4	2022-11	Post-pandemic recovery	Cytology (Pap)	35-39	36	Not performed	Satisfactory	Positive	LSIL	Low-grade / equivocal squamous	
P09624	2022	2022-Q4	2022-11	Post-pandemic recovery	Cytology (Pap)	55-59	57	Not performed	Satisfactory	NILM	NILM	Negative (NILM)	
P09625	2022	2022-Q4	2022-11	Post-pandemic recovery	Cytology (Pap)	40-44	43	Not performed	Satisfactory	NILM	NILM	Negative (NILM)	
P09626	2022	2022-Q4	2022-11	Post-pandemic recovery	Cytology (Pap)	55-59	55	Not performed	Satisfactory	NILM	NILM	Negative (NILM)	
P09627	2022	2022-Q4	2022-11	Post-pandemic recovery	Cytology (Pap)	45-49	45	Not performed	Satisfactory	NILM	NILM	Negative (NILM)	
P09628	2022	2022-Q4	2022-11	Post-pandemic recovery	Cytology (Pap)	60-64	61	Not performed	Satisfactory	NILM	NILM	Negative (NILM)	
P09629	2022	2022-Q4	2022-11	Post-pandemic recovery	Cytology (Pap)	25-29	29	Not performed	Satisfactory	NILM	NILM	Negative (NILM)	
P09630	2022	2022-Q4	2022-11	Post-pandemic recovery	Cytology (Pap)	55-59	59	Not performed	Satisfactory	NILM	NILM	Negative (NILM)	
P09631	2022	2022-Q4	2022-11	Post-pandemic recovery	Cytology (Pap)	35-39	37	Not performed	Satisfactory	NILM	NILM	Negative (NILM)	
P09632	2022	2022-Q4	2022-11	Post-pandemic recovery	Cytology (Pap)	30-34	30	Not performed	Satisfactory	NILM	NILM	Negative (NILM)	
P09633	2022	2022-Q4	2022-11	Post-pandemic recovery	Cytology (Pap)	35-39	37	Not performed	Satisfactory	NILM	NILM	Negative (NILM)	Gardnerella
P09634	2022	2022-Q4	2022-11	Post-pandemic recovery	Cytology (Pap)	35-39	39	Not performed	Unsatisfactory	Unsatisfactory	Unsatisfactory	Unsatisfactory	
P09635	2022	2022-Q4	2022-11	Post-pandemic recovery	Cytology (Pap)	40-44	43	Not performed	Satisfactory	Positive	LSIL	Low-grade / equivocal squamous	
P09636	2022	2022-Q4	2022-11	Post-pandemic recovery	Cytology (Pap)	35-39	37	Not performed	Satisfactory	NILM	NILM	Negative (NILM)	
P09637	2022	2022-Q4	2022-11	Post-pandemic recovery	Cytology (Pap)	25-29	26	Not performed	Satisfactory	NILM	NILM	Negative (NILM)	
P09638	2022	2022-Q4	2022-11	Post-pandemic recovery	Cytology (Pap)	50-54	54	Not performed	Satisfactory	NILM	NILM	Negative (NILM)	
P09639	2022	2022-Q4	2022-11	Post-pandemic recovery	Cytology (Pap)	60-64	60	Not performed	Satisfactory	NILM	NILM	Negative (NILM)	
P09640	2022	2022-Q4	2022-11	Post-pandemic recovery	Cytology (Pap)	25-29	28	Not performed	Satisfactory	NILM	NILM	Negative (NILM)	
P09641	2022	2022-Q4	2022-11	Post-pandemic recovery	Cytology (Pap)	60-64	60	Not performed	Satisfactory	NILM	NILM	Negative (NILM)	
P09642	2022	2022-Q4	2022-11	Post-pandemic recovery	Cytology (Pap)	60-64	61	Not performed	Satisfactory	NILM	NILM	Negative (NILM)	
P09643	2022	2022-Q4	2022-11	Post-pandemic recovery	Cytology (Pap)	30-34	32	Not performed	Satisfactory	NILM	NILM	Negative (NILM)	
P09644	2022	2022-Q4	2022-11	Post-pandemic recovery	Cytology (Pap)	40-44	41	Not performed	Satisfactory	NILM	NILM	Negative (NILM)	
P09645	2022	2022-Q4	2022-11	Post-pandemic recovery	Cytology (Pap)	45-49	45	Not performed	Satisfactory	NILM	NILM	Negative (NILM)	
P09646	2022	2022-Q4	2022-11	Post-pandemic recovery	Cytology (Pap)	55-59	56	Not performed	Satisfactory	NILM	NILM	Negative (NILM)	
P09647	2022	2022-Q4	2022-11	Post-pandemic recovery	Cytology (Pap)	45-49	46	Not performed	Satisfactory	NILM	NILM	Negative (NILM)	
P09648	2022	2022-Q4	2022-11	Post-pandemic recovery	Cytology (Pap)	40-44	40	Not performed	Satisfactory	NILM	NILM	Negative (NILM)	
P09649	2022	2022-Q4	2022-11	Post-pandemic recovery	Cytology (Pap)	35-39	37	Not performed	Satisfactory	NILM	NILM	Negative (NILM)	
P09650	2022	2022-Q4	2022-11	Post-pandemic recovery	Cytology (Pap)	30-34	33	Not performed	Satisfactory	NILM	NILM	Negative (NILM)	
P09651	2022	2022-Q4	2022-11	Post-pandemic recovery	Cytology (Pap)	30-34	31	Not performed	Satisfactory	NILM	NILM	Negative (NILM)	
P09652	2022	2022-Q4	2022-11	Post-pandemic recovery	Cytology (Pap)	45-49	46	Not performed	Satisfactory	NILM	NILM	Negative (NILM)	
P09653	2022	2022-Q4	2022-11	Post-pandemic recovery	Cytology (Pap)	30-34	31	Not performed	Satisfactory	NILM	NILM	Negative (NILM)	
P09654	2022	2022-Q4	2022-11	Post-pandemic recovery	Cytology (Pap)	60-64	62	Not performed	Satisfactory	NILM	NILM	Negative (NILM)	
P09655	2022	2022-Q4	2022-11	Post-pandemic recovery	Cytology (Pap)	40-44	41	Not performed	Satisfactory	NILM	NILM	Negative (NILM)	
P09656	2022	2022-Q4	2022-11	Post-pandemic recovery	Cytology (Pap)	55-59	59	Not performed	Satisfactory	NILM	NILM	Negative (NILM)	
P09657	2022	2022-Q4	2022-11	Post-pandemic recovery	Cytology (Pap)	55-59	59	Not performed	Satisfactory	NILM	NILM	Negative (NILM)	
P09658	2022	2022-Q4	2022-11	Post-pandemic recovery	Cytology (Pap)	45-49	47	Not performed	Satisfactory	NILM	NILM	Negative (NILM)	
P09659	2022	2022-Q4	2022-11	Post-pandemic recovery	Cytology (Pap)	45-49	46	Not performed	Satisfactory	NILM	NILM	Negative (NILM)	
P09660	2022	2022-Q4	2022-11	Post-pandemic recovery	Cytology (Pap)	40-44	41	Not performed	Satisfactory	Positive	LSIL	Low-grade / equivocal squamous	
P09661	2022	2022-Q4	2022-11	Post-pandemic recovery	Cytology (Pap)	50-54	54	Not performed	Satisfactory	NILM	NILM	Negative (NILM)	
P09662	2022	2022-Q4	2022-11	Post-pandemic recovery	Cytology (Pap)	40-44	40	Not performed	Satisfactory	NILM	NILM	Negative (NILM)	
P09663	2022	2022-Q4	2022-11	Post-pandemic recovery	Cytology (Pap)	45-49	45	Not performed	Unsatisfactory	Unsatisfactory	Unsatisfactory	Unsatisfactory	
P09664	2022	2022-Q4	2022-11	Post-pandemic recovery	Cytology (Pap)	35-39	37	Not performed	Satisfactory	NILM	NILM	Negative (NILM)	
P09665	2022	2022-Q4	2022-11	Post-pandemic recovery	Cytology (Pap)	35-39	39	Not performed	Satisfactory	NILM	NILM	Negative (NILM)	
P09666	2022	2022-Q4	2022-11	Post-pandemic recovery	Cytology (Pap)	50-54	51	Not performed	Satisfactory	NILM	NILM	Negative (NILM)	
P09667	2022	2022-Q4	2022-11	Post-pandemic recovery	Cytology (Pap)	25-29	26	Not performed	Satisfactory	NILM	NILM	Negative (NILM)	
P09668	2022	2022-Q4	2022-11	Post-pandemic recovery	Cytology (Pap)	40-44	42	Not performed	Satisfactory	NILM	NILM	Negative (NILM)	
P09669	2022	2022-Q4	2022-11	Post-pandemic recovery	Cytology (Pap)	40-44	40	Not performed	Satisfactory	NILM	NILM	Negative (NILM)	Trichomonas
P09670	2022	2022-Q4	2022-11	Post-pandemic recovery	Cytology (Pap)	35-39	35	Not performed	Satisfactory	NILM	NILM	Negative (NILM)	
P09671	2022	2022-Q4	2022-11	Post-pandemic recovery	Cytology (Pap)	55-59	57	Not performed	Satisfactory	NILM	NILM	Negative (NILM)	
P09672	2022	2022-Q4	2022-11	Post-pandemic recovery	Cytology (Pap)	45-49	48	Not performed	Unsatisfactory	Unsatisfactory	Unsatisfactory	Unsatisfactory	Candida
P09673	2022	2022-Q4	2022-11	Post-pandemic recovery	Cytology (Pap)	45-49	46	Not performed	Satisfactory	NILM	NILM	Negative (NILM)	
P09674	2022	2022-Q4	2022-11	Post-pandemic recovery	Cytology (Pap)	35-39	37	Not performed	Satisfactory	NILM	NILM	Negative (NILM)	
P09675	2022	2022-Q4	2022-11	Post-pandemic recovery	Cytology (Pap)	30-34	30	Not performed	Satisfactory	Positive	ASC-US	Low-grade / equivocal squamous	
P09676	2022	2022-Q4	2022-11	Post-pandemic recovery	Cytology (Pap)	30-34	32	Not performed	Satisfactory	NILM	NILM	Negative (NILM)	
P09677	2022	2022-Q4	2022-11	Post-pandemic recovery	Cytology (Pap)	35-39	38	Not performed	Satisfactory	NILM	NILM	Negative (NILM)	
P09678	2022	2022-Q4	2022-11	Post-pandemic recovery	Cytology (Pap)	45-49	45	Not performed	Satisfactory	NILM	NILM	Negative (NILM)	

patient_id	year	quarter	month	period	primary_test	age_group	age_years	hpv_result	adequacy	result	bethesda	grade	infection
P09679	2022	2022-Q4	2022-11	Post-pandemic recovery	Cytology (Pap)	35-39	35	Not performed	Satisfactory	NILM	NILM	Negative (NILM)	
P09680	2022	2022-Q4	2022-11	Post-pandemic recovery	Cytology (Pap)	45-49	46	Not performed	Satisfactory	NILM	NILM	Negative (NILM)	
P09681	2022	2022-Q4	2022-11	Post-pandemic recovery	Cytology (Pap)	40-44	40	Not performed	Satisfactory	NILM	NILM	Negative (NILM)	
P09682	2022	2022-Q4	2022-12	Post-pandemic recovery	Cytology (Pap)	25-29	29	Not performed	Satisfactory	NILM	NILM	Negative (NILM)	
P09683	2022	2022-Q4	2022-12	Post-pandemic recovery	Cytology (Pap)	30-34	32	Not performed	Satisfactory	NILM	NILM	Negative (NILM)	
P09684	2022	2022-Q4	2022-12	Post-pandemic recovery	Cytology (Pap)	35-39	36	Not performed	Satisfactory	NILM	NILM	Negative (NILM)	
P09685	2022	2022-Q4	2022-12	Post-pandemic recovery	Cytology (Pap)	30-34	33	Not performed	Satisfactory	NILM	NILM	Negative (NILM)	
P09686	2022	2022-Q4	2022-12	Post-pandemic recovery	Cytology (Pap)	40-44	41	Not performed	Satisfactory	NILM	NILM	Negative (NILM)	
P09687	2022	2022-Q4	2022-12	Post-pandemic recovery	Cytology (Pap)	45-49	45	Not performed	Satisfactory	NILM	NILM	Negative (NILM)	
P09688	2022	2022-Q4	2022-12	Post-pandemic recovery	Cytology (Pap)	40-44	40	Not performed	Satisfactory	NILM	NILM	Negative (NILM)	
P09689	2022	2022-Q4	2022-12	Post-pandemic recovery	Cytology (Pap)	35-39	39	Not performed	Satisfactory	Positive	LSIL	Low-grade / equivocal squamous	
P09690	2022	2022-Q4	2022-12	Post-pandemic recovery	Cytology (Pap)	35-39	38	Not performed	Satisfactory	NILM	NILM	Negative (NILM)	
P09691	2022	2022-Q4	2022-12	Post-pandemic recovery	Cytology (Pap)	25-29	25	Not performed	Satisfactory	Positive	ASC-US	Low-grade / equivocal squamous	
P09692	2022	2022-Q4	2022-12	Post-pandemic recovery	Cytology (Pap)	45-49	47	Not performed	Unsatisfactory	Unsatisfactory	Unsatisfactory	Unsatisfactory	Candida
P09693	2022	2022-Q4	2022-12	Post-pandemic recovery	Cytology (Pap)	30-34	34	Not performed	Satisfactory	Positive	ASC-US	Low-grade / equivocal squamous	Candida
P09694	2022	2022-Q4	2022-12	Post-pandemic recovery	Cytology (Pap)	35-39	39	Not performed	Unsatisfactory	Unsatisfactory	Unsatisfactory	Unsatisfactory	
P09695	2022	2022-Q4	2022-12	Post-pandemic recovery	Cytology (Pap)	35-39	36	Not performed	Satisfactory	NILM	NILM	Negative (NILM)	
P09696	2022	2022-Q4	2022-12	Post-pandemic recovery	Cytology (Pap)	55-59	56	Not performed	Satisfactory	NILM	NILM	Negative (NILM)	
P09697	2022	2022-Q4	2022-12	Post-pandemic recovery	Cytology (Pap)	30-34	33	Not performed	Satisfactory	NILM	NILM	Negative (NILM)	
P09698	2022	2022-Q4	2022-12	Post-pandemic recovery	Cytology (Pap)	30-34	32	Not performed	Satisfactory	Positive	ASC-US	Low-grade / equivocal squamous	
P09699	2022	2022-Q4	2022-12	Post-pandemic recovery	Cytology (Pap)	45-49	49	Not performed	Satisfactory	NILM	NILM	Negative (NILM)	
P09700	2022	2022-Q4	2022-12	Post-pandemic recovery	Cytology (Pap)	35-39	39	Not performed	Satisfactory	NILM	NILM	Negative (NILM)	
P09701	2022	2022-Q4	2022-12	Post-pandemic recovery	Cytology (Pap)	30-34	32	Not performed	Satisfactory	NILM	NILM	Negative (NILM)	
P09702	2022	2022-Q4	2022-12	Post-pandemic recovery	Cytology (Pap)	40-44	42	Not performed	Satisfactory	NILM	NILM	Negative (NILM)	
P09703	2022	2022-Q4	2022-12	Post-pandemic recovery	Cytology (Pap)	40-44	44	Not performed	Satisfactory	NILM	NILM	Negative (NILM)	
P09704	2022	2022-Q4	2022-12	Post-pandemic recovery	Cytology (Pap)	55-59	56	Not performed	Satisfactory	NILM	NILM	Negative (NILM)	
P09705	2022	2022-Q4	2022-12	Post-pandemic recovery	Cytology (Pap)	50-54	52	Not performed	Satisfactory	NILM	NILM	Negative (NILM)	
P09706	2022	2022-Q4	2022-12	Post-pandemic recovery	Cytology (Pap)	30-34	33	Not performed	Satisfactory	NILM	NILM	Negative (NILM)	
P09707	2022	2022-Q4	2022-12	Post-pandemic recovery	Cytology (Pap)	50-54	54	Not performed	Satisfactory	Positive	AGC	Glandular	
P09708	2022	2022-Q4	2022-12	Post-pandemic recovery	Cytology (Pap)	60-64	61	Not performed	Satisfactory	NILM	NILM	Negative (NILM)	
P09709	2022	2022-Q4	2022-12	Post-pandemic recovery	Cytology (Pap)	30-34	34	Not performed	Satisfactory	NILM	NILM	Negative (NILM)	
P09710	2022	2022-Q4	2022-12	Post-pandemic recovery	Cytology (Pap)	55-59	57	Not performed	Satisfactory	Positive	ASC-US	Low-grade / equivocal squamous	
P09711	2022	2022-Q4	2022-12	Post-pandemic recovery	Cytology (Pap)	30-34	33	Not performed	Satisfactory	NILM	NILM	Negative (NILM)	
P09712	2022	2022-Q4	2022-12	Post-pandemic recovery	Cytology (Pap)	25-29	29	Not performed	Satisfactory	NILM	NILM	Negative (NILM)	
P09713	2022	2022-Q4	2022-12	Post-pandemic recovery	Cytology (Pap)	35-39	39	Not performed	Satisfactory	NILM	NILM	Negative (NILM)	
P09714	2022	2022-Q4	2022-12	Post-pandemic recovery	Cytology (Pap)	50-54	54	Not performed	Satisfactory	Positive	LSIL	Low-grade / equivocal squamous	Trichomonas
P09715	2022	2022-Q4	2022-12	Post-pandemic recovery	Cytology (Pap)	30-34	30	Not performed	Satisfactory	NILM	NILM	Negative (NILM)	
P09716	2022	2022-Q4	2022-12	Post-pandemic recovery	Cytology (Pap)	45-49	46	Not performed	Satisfactory	NILM	NILM	Negative (NILM)	
P09717	2022	2022-Q4	2022-12	Post-pandemic recovery	Cytology (Pap)	50-54	52	Not performed	Satisfactory	NILM	NILM	Negative (NILM)	
P09718	2022	2022-Q4	2022-12	Post-pandemic recovery	Cytology (Pap)	60-64	64	Not performed	Unsatisfactory	Unsatisfactory	Unsatisfactory	Unsatisfactory	
P09719	2022	2022-Q4	2022-12	Post-pandemic recovery	Cytology (Pap)	50-54	54	Not performed	Satisfactory	Positive	HSIL	High-grade squamous	
P09720	2022	2022-Q4	2022-12	Post-pandemic recovery	Cytology (Pap)	35-39	36	Not performed	Satisfactory	NILM	NILM	Negative (NILM)	
P09721	2022	2022-Q4	2022-12	Post-pandemic recovery	Cytology (Pap)	25-29	27	Not performed	Satisfactory	NILM	NILM	Negative (NILM)	
P09722	2022	2022-Q4	2022-12	Post-pandemic recovery	Cytology (Pap)	35-39	35	Not performed	Satisfactory	NILM	NILM	Negative (NILM)	
P09723	2022	2022-Q4	2022-12	Post-pandemic recovery	Cytology (Pap)	35-39	39	Not performed	Satisfactory	NILM	NILM	Negative (NILM)	
P09724	2022	2022-Q4	2022-12	Post-pandemic recovery	Cytology (Pap)	40-44	43	Not performed	Satisfactory	Positive	ASC-US	Low-grade / equivocal squamous	
P09725	2022	2022-Q4	2022-12	Post-pandemic recovery	Cytology (Pap)	25-29	29	Not performed	Satisfactory	NILM	NILM	Negative (NILM)	Candida
P09726	2022	2022-Q4	2022-12	Post-pandemic recovery	Cytology (Pap)	45-49	45	Not performed	Satisfactory	Positive	ASC-US	Low-grade / equivocal squamous	
P09727	2022	2022-Q4	2022-12	Post-pandemic recovery	Cytology (Pap)	40-44	42	Not performed	Unsatisfactory	Unsatisfactory	Unsatisfactory	Unsatisfactory	
P09728	2022	2022-Q4	2022-12	Post-pandemic recovery	Cytology (Pap)	30-34	30	Not performed	Satisfactory	NILM	NILM	Negative (NILM)	
P09729	2022	2022-Q4	2022-12	Post-pandemic recovery	Cytology (Pap)	40-44	41	Not performed	Satisfactory	NILM	NILM	Negative (NILM)	
P09730	2022	2022-Q4	2022-12	Post-pandemic recovery	Cytology (Pap)	40-44	44	Not performed	Satisfactory	NILM	NILM	Negative (NILM)	
P09731	2022	2022-Q4	2022-12	Post-pandemic recovery	Cytology (Pap)	35-39	36	Not performed	Unsatisfactory	Unsatisfactory	Unsatisfactory	Unsatisfactory	
P09732	2022	2022-Q4	2022-12	Post-pandemic recovery	Cytology (Pap)	30-34	34	Not performed	Satisfactory	NILM	NILM	Negative (NILM)	Candida
P09733	2022	2022-Q4	2022-12	Post-pandemic recovery	Cytology (Pap)	45-49	49	Not performed	Unsatisfactory	Unsatisfactory	Unsatisfactory	Unsatisfactory	
P09734	2022	2022-Q4	2022-12	Post-pandemic recovery	Cytology (Pap)	30-34	34	Not performed	Satisfactory	NILM	NILM	Negative (NILM)	
P09735	2022	2022-Q4	2022-12	Post-pandemic recovery	Cytology (Pap)	40-44	44	Not performed	Satisfactory	NILM	NILM	Negative (NILM)	
P09736	2022	2022-Q4	2022-12	Post-pandemic recovery	Cytology (Pap)	50-54	53	Not performed	Satisfactory	NILM	NILM	Negative (NILM)	
P09737	2022	2022-Q4	2022-12	Post-pandemic recovery	Cytology (Pap)	30-34	33	Not performed	Satisfactory	NILM	NILM	Negative (NILM)	
P09738	2022	2022-Q4	2022-12	Post-pandemic recovery	Cytology (Pap)	25-29	26	Not performed	Satisfactory	NILM	NILM	Negative (NILM)	
P09739	2022	2022-Q4	2022-12	Post-pandemic recovery	Cytology (Pap)	55-59	59	Not performed	Satisfactory	NILM	NILM	Negative (NILM)	
P09740	2022	2022-Q4	2022-12	Post-pandemic recovery	Cytology (Pap)	50-54	50	Not performed	Satisfactory	NILM	NILM	Negative (NILM)	
P09741	2022	2022-Q4	2022-12	Post-pandemic recovery	Cytology (Pap)	30-34	32	Not performed	Satisfactory	NILM	NILM	Negative (NILM)	
P09742	2022	2022-Q4	2022-12	Post-pandemic recovery	Cytology (Pap)	50-54	50	Not performed	Satisfactory	Positive	ASC-US	Low-grade / equivocal squamous	
P09743	2022	2022-Q4	2022-12	Post-pandemic recovery	Cytology (Pap)	45-49	46	Not performed	Satisfactory	NILM	NILM	Negative (NILM)	
P09744	2022	2022-Q4	2022-12	Post-pandemic recovery	Cytology (Pap)	50-54	54	Not performed	Satisfactory	NILM	NILM	Negative (NILM)	
P09745	2022	2022-Q4	2022-12	Post-pandemic recovery	Cytology (Pap)	30-34	33	Not performed	Satisfactory	NILM	NILM	Negative (NILM)	
P09746	2022	2022-Q4	2022-12	Post-pandemic recovery	Cytology (Pap)	45-49	45	Not performed	Satisfactory	NILM	NILM	Negative (NILM)	
P09747	2022	2022-Q4	2022-12	Post-pandemic recovery	Cytology (Pap)	30-34	33	Not performed	Satisfactory	NILM	NILM	Negative (NILM)	
P09748	2022	2022-Q4	2022-12	Post-pandemic recovery	Cytology (Pap)	30-34	34	Not performed	Satisfactory	NILM	NILM	Negative (NILM)	
P09749	2022	2022-Q4	2022-12	Post-pandemic recovery	Cytology (Pap)	35-39	35	Not performed	Satisfactory	Positive	ASC-US	Low-grade / equivocal squamous	
P09750	2022	2022-Q4	2022-12	Post-pandemic recovery	Cytology (Pap)	30-34	33	Not performed	Satisfactory	NILM	NILM	Negative (NILM)	
P09751	2022	2022-Q4	2022-12	Post-pandemic recovery	Cytology (Pap)	40-44	41	Not performed	Satisfactory	NILM	NILM	Negative (NILM)	
P09752	2022	2022-Q4	2022-12	Post-pandemic recovery	Cytology (Pap)	30-34	32	Not performed	Satisfactory	NILM	NILM	Negative (NILM)	
P09753	2022	2022-Q4	2022-12	Post-pandemic recovery	Cytology (Pap)	35-39	36	Not performed	Satisfactory	NILM	NILM	Negative (NILM)	
P09754	2022	2022-Q4	2022-12	Post-pandemic recovery	Cytology (Pap)	45-49	46	Not performed	Satisfactory	NILM	NILM	Negative (NILM)	
P09755	2022	2022-Q4	2022-12	Post-pandemic recovery	Cytology (Pap)	50-54	53	Not performed	Satisfactory	NILM	NILM	Negative (NILM)	
P09756	2022	2022-Q4	2022-12	Post-pandemic recovery	Cytology (Pap)	25-29	25	Not performed	Satisfactory	NILM	NILM	Negative (NILM)	Gardnerella
P09757	2022	2022-Q4	2022-12	Post-pandemic recovery	Cytology (Pap)	60-64	60	Not performed	Satisfactory	NILM	NILM	Negative (NILM)	
P09758	2022	2022-Q4	2022-12	Post-pandemic recovery	Cytology (Pap)	40-44	43	Not performed	Satisfactory	NILM	NILM	Negative (NILM)	Candida

patient_id	year	quarter	month	period	primary_test	age_group	age_years	hpv_result	adequacy	result	bethesda	grade	infection
P09759	2022	2022-Q4	2022-12	Post-pandemic recovery	Cytology (Pap)	40-44	41	Not performed	Unsatisfactory	Unsatisfactory	Unsatisfactory	Unsatisfactory	
P09760	2022	2022-Q4	2022-12	Post-pandemic recovery	Cytology (Pap)	40-44	42	Not performed	Satisfactory	NILM	NILM	Negative (NILM)	
P09761	2022	2022-Q4	2022-12	Post-pandemic recovery	Cytology (Pap)	40-44	43	Not performed	Satisfactory	NILM	NILM	Negative (NILM)	
P09762	2022	2022-Q4	2022-12	Post-pandemic recovery	Cytology (Pap)	40-44	40	Not performed	Satisfactory	NILM	NILM	Negative (NILM)	
P09763	2022	2022-Q4	2022-12	Post-pandemic recovery	Cytology (Pap)	30-34	31	Not performed	Satisfactory	NILM	NILM	Negative (NILM)	
P09764	2022	2022-Q4	2022-12	Post-pandemic recovery	Cytology (Pap)	35-39	39	Not performed	Satisfactory	NILM	NILM	Negative (NILM)	
P09765	2022	2022-Q4	2022-12	Post-pandemic recovery	Cytology (Pap)	60-64	60	Not performed	Satisfactory	NILM	NILM	Negative (NILM)	
P09766	2022	2022-Q4	2022-12	Post-pandemic recovery	Cytology (Pap)	50-54	53	Not performed	Unsatisfactory	Unsatisfactory	Unsatisfactory	Unsatisfactory	
P09767	2022	2022-Q4	2022-12	Post-pandemic recovery	Cytology (Pap)	35-39	39	Not performed	Satisfactory	Positive	ASC-US	Low-grade / equivocal squamous	
P09768	2022	2022-Q4	2022-12	Post-pandemic recovery	Cytology (Pap)	30-34	30	Not performed	Satisfactory	NILM	NILM	Negative (NILM)	
P09769	2022	2022-Q4	2022-12	Post-pandemic recovery	Cytology (Pap)	50-54	51	Not performed	Satisfactory	NILM	NILM	Negative (NILM)	
P09770	2022	2022-Q4	2022-12	Post-pandemic recovery	Cytology (Pap)	40-44	44	Not performed	Satisfactory	NILM	NILM	Negative (NILM)	
P09771	2022	2022-Q4	2022-12	Post-pandemic recovery	Cytology (Pap)	40-44	44	Not performed	Satisfactory	NILM	NILM	Negative (NILM)	Candida
P09772	2022	2022-Q4	2022-12	Post-pandemic recovery	Cytology (Pap)	40-44	44	Not performed	Satisfactory	NILM	NILM	Negative (NILM)	
P09773	2022	2022-Q4	2022-12	Post-pandemic recovery	Cytology (Pap)	40-44	42	Not performed	Satisfactory	Positive	ASC-US	Low-grade / equivocal squamous	
P09774	2022	2022-Q4	2022-12	Post-pandemic recovery	Cytology (Pap)	25-29	28	Not performed	Satisfactory	NILM	NILM	Negative (NILM)	
P09775	2022	2022-Q4	2022-12	Post-pandemic recovery	Cytology (Pap)	40-44	42	Not performed	Satisfactory	Positive	ASC-US	Low-grade / equivocal squamous	
P09776	2022	2022-Q4	2022-12	Post-pandemic recovery	Cytology (Pap)	60-64	64	Not performed	Satisfactory	NILM	NILM	Negative (NILM)	
P09777	2022	2022-Q4	2022-12	Post-pandemic recovery	Cytology (Pap)	30-34	33	Not performed	Satisfactory	NILM	NILM	Negative (NILM)	
P09778	2022	2022-Q4	2022-12	Post-pandemic recovery	Cytology (Pap)	60-64	64	Not performed	Satisfactory	NILM	NILM	Negative (NILM)	Candida
P09779	2022	2022-Q4	2022-12	Post-pandemic recovery	Cytology (Pap)	35-39	39	Not performed	Satisfactory	NILM	NILM	Negative (NILM)	
P09780	2022	2022-Q4	2022-12	Post-pandemic recovery	Cytology (Pap)	50-54	50	Not performed	Satisfactory	NILM	NILM	Negative (NILM)	
P09781	2022	2022-Q4	2022-12	Post-pandemic recovery	Cytology (Pap)	55-59	55	Not performed	Satisfactory	NILM	NILM	Negative (NILM)	
P09782	2022	2022-Q4	2022-12	Post-pandemic recovery	Cytology (Pap)	50-54	54	Not performed	Satisfactory	NILM	NILM	Negative (NILM)	Trichomonas
P09783	2022	2022-Q4	2022-12	Post-pandemic recovery	Cytology (Pap)	25-29	27	Not performed	Satisfactory	NILM	NILM	Negative (NILM)	
P09784	2022	2022-Q4	2022-12	Post-pandemic recovery	Cytology (Pap)	35-39	37	Not performed	Satisfactory	NILM	NILM	Negative (NILM)	
P09785	2022	2022-Q4	2022-12	Post-pandemic recovery	Cytology (Pap)	25-29	25	Not performed	Satisfactory	NILM	NILM	Negative (NILM)	
P09786	2022	2022-Q4	2022-12	Post-pandemic recovery	Cytology (Pap)	30-34	34	Not performed	Satisfactory	NILM	NILM	Negative (NILM)	
P09787	2022	2022-Q4	2022-12	Post-pandemic recovery	Cytology (Pap)	50-54	52	Not performed	Satisfactory	NILM	NILM	Negative (NILM)	
P09788	2022	2022-Q4	2022-12	Post-pandemic recovery	Cytology (Pap)	25-29	29	Not performed	Satisfactory	NILM	NILM	Negative (NILM)	
P09789	2022	2022-Q4	2022-12	Post-pandemic recovery	Cytology (Pap)	50-54	51	Not performed	Satisfactory	NILM	NILM	Negative (NILM)	
P09790	2022	2022-Q4	2022-12	Post-pandemic recovery	Cytology (Pap)	55-59	59	Not performed	Satisfactory	NILM	NILM	Negative (NILM)	Candida
P09791	2022	2022-Q4	2022-12	Post-pandemic recovery	Cytology (Pap)	45-49	49	Not performed	Satisfactory	NILM	NILM	Negative (NILM)	
P09792	2022	2022-Q4	2022-12	Post-pandemic recovery	Cytology (Pap)	50-54	52	Not performed	Satisfactory	NILM	NILM	Negative (NILM)	
P09793	2022	2022-Q4	2022-12	Post-pandemic recovery	Cytology (Pap)	30-34	30	Not performed	Satisfactory	NILM	NILM	Negative (NILM)	
P09794	2022	2022-Q4	2022-12	Post-pandemic recovery	Cytology (Pap)	45-49	45	Not performed	Satisfactory	NILM	NILM	Negative (NILM)	
P09795	2022	2022-Q4	2022-12	Post-pandemic recovery	Cytology (Pap)	40-44	43	Not performed	Unsatisfactory	Unsatisfactory	Unsatisfactory	Unsatisfactory	
P09796	2022	2022-Q4	2022-12	Post-pandemic recovery	Cytology (Pap)	45-49	49	Not performed	Satisfactory	NILM	NILM	Negative (NILM)	
P09797	2022	2022-Q4	2022-12	Post-pandemic recovery	Cytology (Pap)	50-54	52	Not performed	Satisfactory	NILM	NILM	Negative (NILM)	
P09798	2022	2022-Q4	2022-12	Post-pandemic recovery	Cytology (Pap)	35-39	39	Not performed	Satisfactory	Positive	ASC-US	Low-grade / equivocal squamous	
P09799	2022	2022-Q4	2022-12	Post-pandemic recovery	Cytology (Pap)	50-54	52	Not performed	Satisfactory	NILM	NILM	Negative (NILM)	
P09800	2022	2022-Q4	2022-12	Post-pandemic recovery	Cytology (Pap)	35-39	35	Not performed	Satisfactory	NILM	NILM	Negative (NILM)	
P09801	2022	2022-Q4	2022-12	Post-pandemic recovery	Cytology (Pap)	35-39	38	Not performed	Satisfactory	NILM	NILM	Negative (NILM)	
P09802	2022	2022-Q4	2022-12	Post-pandemic recovery	Cytology (Pap)	30-34	31	Not performed	Satisfactory	NILM	NILM	Negative (NILM)	
P09803	2022	2022-Q4	2022-12	Post-pandemic recovery	Cytology (Pap)	35-39	35	Not performed	Satisfactory	NILM	NILM	Negative (NILM)	
P09804	2022	2022-Q4	2022-12	Post-pandemic recovery	Cytology (Pap)	35-39	37	Not performed	Satisfactory	NILM	NILM	Negative (NILM)	
P09805	2022	2022-Q4	2022-12	Post-pandemic recovery	Cytology (Pap)	35-39	38	Not performed	Satisfactory	NILM	NILM	Negative (NILM)	
P09806	2022	2022-Q4	2022-12	Post-pandemic recovery	Cytology (Pap)	45-49	48	Not performed	Satisfactory	NILM	NILM	Negative (NILM)	
P09807	2022	2022-Q4	2022-12	Post-pandemic recovery	Cytology (Pap)	45-49	45	Not performed	Satisfactory	NILM	NILM	Negative (NILM)	
P09808	2022	2022-Q4	2022-12	Post-pandemic recovery	Cytology (Pap)	40-44	44	Not performed	Satisfactory	NILM	NILM	Negative (NILM)	
P09809	2022	2022-Q4	2022-12	Post-pandemic recovery	Cytology (Pap)	35-39	35	Not performed	Satisfactory	Positive	AGC	Glandular	
P09810	2022	2022-Q4	2022-12	Post-pandemic recovery	Cytology (Pap)	55-59	58	Not performed	Satisfactory	NILM	NILM	Negative (NILM)	
P09811	2022	2022-Q4	2022-12	Post-pandemic recovery	Cytology (Pap)	60-64	60	Not performed	Satisfactory	NILM	NILM	Negative (NILM)	
P09812	2022	2022-Q4	2022-12	Post-pandemic recovery	Cytology (Pap)	35-39	35	Not performed	Satisfactory	NILM	NILM	Negative (NILM)	
P09813	2022	2022-Q4	2022-12	Post-pandemic recovery	Cytology (Pap)	25-29	26	Not performed	Satisfactory	Positive	LSIL	Low-grade / equivocal squamous	
P09814	2022	2022-Q4	2022-12	Post-pandemic recovery	Cytology (Pap)	40-44	42	Not performed	Satisfactory	NILM	NILM	Negative (NILM)	
P09815	2022	2022-Q4	2022-12	Post-pandemic recovery	Cytology (Pap)	50-54	51	Not performed	Satisfactory	NILM	NILM	Negative (NILM)	Other
P09816	2022	2022-Q4	2022-12	Post-pandemic recovery	Cytology (Pap)	45-49	45	Not performed	Satisfactory	NILM	NILM	Negative (NILM)	
P09817	2022	2022-Q4	2022-12	Post-pandemic recovery	Cytology (Pap)	40-44	43	Not performed	Satisfactory	NILM	NILM	Negative (NILM)	
P09818	2022	2022-Q4	2022-12	Post-pandemic recovery	Cytology (Pap)	55-59	56	Not performed	Satisfactory	NILM	NILM	Negative (NILM)	
P09819	2022	2022-Q4	2022-12	Post-pandemic recovery	Cytology (Pap)	35-39	37	Not performed	Satisfactory	NILM	NILM	Negative (NILM)	
P09820	2022	2022-Q4	2022-12	Post-pandemic recovery	Cytology (Pap)	50-54	53	Not performed	Satisfactory	NILM	NILM	Negative (NILM)	
P09821	2022	2022-Q4	2022-12	Post-pandemic recovery	Cytology (Pap)	35-39	37	Not performed	Satisfactory	NILM	NILM	Negative (NILM)	Gardnerella
P09822	2022	2022-Q4	2022-12	Post-pandemic recovery	Cytology (Pap)	45-49	45	Not performed	Satisfactory	NILM	NILM	Negative (NILM)	
P09823	2022	2022-Q4	2022-12	Post-pandemic recovery	Cytology (Pap)	40-44	41	Not performed	Satisfactory	NILM	NILM	Negative (NILM)	
P09824	2022	2022-Q4	2022-12	Post-pandemic recovery	Cytology (Pap)	35-39	38	Not performed	Satisfactory	Positive	LSIL	Low-grade / equivocal squamous	
P09825	2022	2022-Q4	2022-12	Post-pandemic recovery	Cytology (Pap)	45-49	48	Not performed	Satisfactory	NILM	NILM	Negative (NILM)	
P09826	2022	2022-Q4	2022-12	Post-pandemic recovery	Cytology (Pap)	45-49	48	Not performed	Satisfactory	NILM	NILM	Negative (NILM)	
P09827	2022	2022-Q4	2022-12	Post-pandemic recovery	Cytology (Pap)	35-39	35	Not performed	Satisfactory	NILM	NILM	Negative (NILM)	
P09828	2022	2022-Q4	2022-12	Post-pandemic recovery	Cytology (Pap)	35-39	35	Not performed	Satisfactory	NILM	NILM	Negative (NILM)	
P09829	2022	2022-Q4	2022-12	Post-pandemic recovery	Cytology (Pap)	50-54	51	Not performed	Satisfactory	NILM	NILM	Negative (NILM)	
P09830	2022	2022-Q4	2022-12	Post-pandemic recovery	Cytology (Pap)	35-39	36	Not performed	Unsatisfactory	Unsatisfactory	Unsatisfactory	Unsatisfactory	
P09831	2022	2022-Q4	2022-12	Post-pandemic recovery	Cytology (Pap)	50-54	53	Not performed	Satisfactory	NILM	NILM	Negative (NILM)	
P09832	2022	2022-Q4	2022-12	Post-pandemic recovery	Cytology (Pap)	40-44	44	Not performed	Satisfactory	NILM	NILM	Negative (NILM)	
P09833	2022	2022-Q4	2022-12	Post-pandemic recovery	Cytology (Pap)	45-49	47	Not performed	Satisfactory	NILM	NILM	Negative (NILM)	
P09834	2022	2022-Q4	2022-12	Post-pandemic recovery	Cytology (Pap)	30-34	31	Not performed	Satisfactory	NILM	NILM	Negative (NILM)	
P09835	2022	2022-Q4	2022-12	Post-pandemic recovery	Cytology (Pap)	30-34	30	Not performed	Unsatisfactory	Unsatisfactory	Unsatisfactory	Unsatisfactory	
P09836	2022	2022-Q4	2022-12	Post-pandemic recovery	Cytology (Pap)	60-64	62	Not performed	Satisfactory	NILM	NILM	Negative (NILM)	
P09837	2022	2022-Q4	2022-12	Post-pandemic recovery	Cytology (Pap)	45-49	48	Not performed	Satisfactory	NILM	NILM	Negative (NILM)	
P09838	2022	2022-Q4	2022-12	Post-pandemic recovery	Cytology (Pap)	35-39	37	Not performed	Satisfactory	NILM	NILM	Negative (NILM)	

patient_id	year	quarter	month	period	primary_test	age_group	age_years	hpv_result	adequacy	result	bethesda	grade	infection
P09839	2022	2022-Q4	2022-12	Post-pandemic recovery	Cytology (Pap)	45-49	45	Not performed	Satisfactory	NILM	NILM	Negative (NILM)	Trichomonas
P09840	2022	2022-Q4	2022-12	Post-pandemic recovery	Cytology (Pap)	40-44	43	Not performed	Satisfactory	NILM	NILM	Negative (NILM)	
P09841	2022	2022-Q4	2022-12	Post-pandemic recovery	Cytology (Pap)	35-39	39	Not performed	Satisfactory	NILM	NILM	Negative (NILM)	
P09842	2022	2022-Q4	2022-12	Post-pandemic recovery	Cytology (Pap)	60-64	60	Not performed	Satisfactory	NILM	NILM	Negative (NILM)	
P09843	2022	2022-Q4	2022-12	Post-pandemic recovery	Cytology (Pap)	25-29	27	Not performed	Satisfactory	NILM	NILM	Negative (NILM)	
P09844	2022	2022-Q4	2022-12	Post-pandemic recovery	Cytology (Pap)	30-34	30	Not performed	Satisfactory	NILM	NILM	Negative (NILM)	
P09845	2022	2022-Q4	2022-12	Post-pandemic recovery	Cytology (Pap)	45-49	46	Not performed	Unsatisfactory	Unsatisfactory	Unsatisfactory	Unsatisfactory	
P09846	2022	2022-Q4	2022-12	Post-pandemic recovery	Cytology (Pap)	30-34	31	Not performed	Satisfactory	NILM	NILM	Negative (NILM)	
P09847	2022	2022-Q4	2022-12	Post-pandemic recovery	Cytology (Pap)	25-29	26	Not performed	Satisfactory	NILM	NILM	Negative (NILM)	
P09848	2022	2022-Q4	2022-12	Post-pandemic recovery	Cytology (Pap)	40-44	43	Not performed	Satisfactory	NILM	NILM	Negative (NILM)	
P09849	2022	2022-Q4	2022-12	Post-pandemic recovery	Cytology (Pap)	25-29	29	Not performed	Unsatisfactory	Unsatisfactory	Unsatisfactory	Unsatisfactory	
P09850	2022	2022-Q4	2022-12	Post-pandemic recovery	Cytology (Pap)	55-59	57	Not performed	Satisfactory	NILM	NILM	Negative (NILM)	
P09851	2022	2022-Q4	2022-12	Post-pandemic recovery	Cytology (Pap)	50-54	50	Not performed	Satisfactory	NILM	NILM	Negative (NILM)	
P09852	2022	2022-Q4	2022-12	Post-pandemic recovery	Cytology (Pap)	60-64	60	Not performed	Satisfactory	NILM	NILM	Negative (NILM)	
P09853	2022	2022-Q4	2022-12	Post-pandemic recovery	Cytology (Pap)	25-29	28	Not performed	Satisfactory	NILM	NILM	Negative (NILM)	
P09854	2022	2022-Q4	2022-12	Post-pandemic recovery	Cytology (Pap)	25-29	29	Not performed	Satisfactory	NILM	NILM	Negative (NILM)	
P09855	2022	2022-Q4	2022-12	Post-pandemic recovery	Cytology (Pap)	30-34	33	Not performed	Satisfactory	NILM	NILM	Negative (NILM)	
P09856	2022	2022-Q4	2022-12	Post-pandemic recovery	Cytology (Pap)	25-29	26	Not performed	Satisfactory	NILM	NILM	Negative (NILM)	
P09857	2022	2022-Q4	2022-12	Post-pandemic recovery	Cytology (Pap)	30-34	30	Not performed	Satisfactory	NILM	NILM	Negative (NILM)	
P09858	2023	2023-Q1	2023-01	Post-pandemic recovery	Cytology (Pap)	50-54	54	Not performed	Satisfactory	NILM	NILM	Negative (NILM)	
P09859	2023	2023-Q1	2023-01	Post-pandemic recovery	Cytology (Pap)	40-44	42	Not performed	Satisfactory	NILM	NILM	Negative (NILM)	
P09860	2023	2023-Q1	2023-01	Post-pandemic recovery	Cytology (Pap)	40-44	43	Not performed	Satisfactory	NILM	NILM	Negative (NILM)	
P09861	2023	2023-Q1	2023-01	Post-pandemic recovery	Cytology (Pap)	35-39	39	Not performed	Satisfactory	Positive	LSIL	Low-grade / equivocal squamous	
P09862	2023	2023-Q1	2023-01	Post-pandemic recovery	Cytology (Pap)	55-59	59	Not performed	Satisfactory	NILM	NILM	Negative (NILM)	
P09863	2023	2023-Q1	2023-01	Post-pandemic recovery	Cytology (Pap)	35-39	37	Not performed	Satisfactory	NILM	NILM	Negative (NILM)	
P09864	2023	2023-Q1	2023-01	Post-pandemic recovery	Cytology (Pap)	30-34	34	Not performed	Satisfactory	NILM	NILM	Negative (NILM)	
P09865	2023	2023-Q1	2023-01	Post-pandemic recovery	Cytology (Pap)	55-59	59	Not performed	Satisfactory	NILM	NILM	Negative (NILM)	
P09866	2023	2023-Q1	2023-01	Post-pandemic recovery	Cytology (Pap)	55-59	58	Not performed	Satisfactory	NILM	NILM	Negative (NILM)	
P09867	2023	2023-Q1	2023-01	Post-pandemic recovery	Cytology (Pap)	30-34	33	Not performed	Satisfactory	NILM	NILM	Negative (NILM)	
P09868	2023	2023-Q1	2023-01	Post-pandemic recovery	Cytology (Pap)	40-44	41	Not performed	Satisfactory	NILM	NILM	Negative (NILM)	
P09869	2023	2023-Q1	2023-01	Post-pandemic recovery	Cytology (Pap)	45-49	48	Not performed	Satisfactory	NILM	NILM	Negative (NILM)	
P09870	2023	2023-Q1	2023-01	Post-pandemic recovery	Cytology (Pap)	30-34	30	Not performed	Satisfactory	NILM	NILM	Negative (NILM)	
P09871	2023	2023-Q1	2023-01	Post-pandemic recovery	Cytology (Pap)	55-59	59	Not performed	Satisfactory	NILM	NILM	Negative (NILM)	Other
P09872	2023	2023-Q1	2023-01	Post-pandemic recovery	Cytology (Pap)	50-54	54	Not performed	Satisfactory	Positive	LSIL	Low-grade / equivocal squamous	Candida
P09873	2023	2023-Q1	2023-01	Post-pandemic recovery	Cytology (Pap)	45-49	46	Not performed	Satisfactory	NILM	NILM	Negative (NILM)	
P09874	2023	2023-Q1	2023-01	Post-pandemic recovery	Cytology (Pap)	60-64	60	Not performed	Satisfactory	NILM	NILM	Negative (NILM)	
P09875	2023	2023-Q1	2023-01	Post-pandemic recovery	Cytology (Pap)	45-49	45	Not performed	Satisfactory	NILM	NILM	Negative (NILM)	
P09876	2023	2023-Q1	2023-01	Post-pandemic recovery	Cytology (Pap)	60-64	62	Not performed	Satisfactory	NILM	NILM	Negative (NILM)	Trichomonas
P09877	2023	2023-Q1	2023-01	Post-pandemic recovery	Cytology (Pap)	55-59	57	Not performed	Satisfactory	NILM	NILM	Negative (NILM)	Candida
P09878	2023	2023-Q1	2023-01	Post-pandemic recovery	Cytology (Pap)	55-59	58	Not performed	Satisfactory	NILM	NILM	Negative (NILM)	
P09879	2023	2023-Q1	2023-01	Post-pandemic recovery	Cytology (Pap)	35-39	36	Not performed	Satisfactory	NILM	NILM	Negative (NILM)	
P09880	2023	2023-Q1	2023-01	Post-pandemic recovery	Cytology (Pap)	55-59	56	Not performed	Satisfactory	NILM	NILM	Negative (NILM)	
P09881	2023	2023-Q1	2023-01	Post-pandemic recovery	Cytology (Pap)	50-54	52	Not performed	Satisfactory	NILM	NILM	Negative (NILM)	
P09882	2023	2023-Q1	2023-01	Post-pandemic recovery	Cytology (Pap)	30-34	33	Not performed	Satisfactory	NILM	NILM	Negative (NILM)	
P09883	2023	2023-Q1	2023-01	Post-pandemic recovery	Cytology (Pap)	40-44	41	Not performed	Satisfactory	NILM	NILM	Negative (NILM)	
P09884	2023	2023-Q1	2023-01	Post-pandemic recovery	Cytology (Pap)	45-49	48	Not performed	Unsatisfactory	Unsatisfactory	Unsatisfactory	Unsatisfactory	
P09885	2023	2023-Q1	2023-01	Post-pandemic recovery	Cytology (Pap)	30-34	32	Not performed	Satisfactory	NILM	NILM	Negative (NILM)	
P09886	2023	2023-Q1	2023-01	Post-pandemic recovery	Cytology (Pap)	50-54	54	Not performed	Satisfactory	NILM	NILM	Negative (NILM)	
P09887	2023	2023-Q1	2023-01	Post-pandemic recovery	Cytology (Pap)	50-54	53	Not performed	Satisfactory	Positive	HSIL	High-grade squamous	Candida
P09888	2023	2023-Q1	2023-01	Post-pandemic recovery	Cytology (Pap)	30-34	32	Not performed	Satisfactory	NILM	NILM	Negative (NILM)	
P09889	2023	2023-Q1	2023-01	Post-pandemic recovery	Cytology (Pap)	50-54	50	Not performed	Satisfactory	NILM	NILM	Negative (NILM)	
P09890	2023	2023-Q1	2023-01	Post-pandemic recovery	Cytology (Pap)	40-44	44	Not performed	Satisfactory	NILM	NILM	Negative (NILM)	
P09891	2023	2023-Q1	2023-01	Post-pandemic recovery	Cytology (Pap)	30-34	30	Not performed	Satisfactory	NILM	NILM	Negative (NILM)	Candida
P09892	2023	2023-Q1	2023-01	Post-pandemic recovery	Cytology (Pap)	50-54	52	Not performed	Satisfactory	NILM	NILM	Negative (NILM)	
P09893	2023	2023-Q1	2023-01	Post-pandemic recovery	Cytology (Pap)	55-59	59	Not performed	Satisfactory	NILM	NILM	Negative (NILM)	
P09894	2023	2023-Q1	2023-01	Post-pandemic recovery	Cytology (Pap)	45-49	49	Not performed	Satisfactory	NILM	NILM	Negative (NILM)	
P09895	2023	2023-Q1	2023-01	Post-pandemic recovery	Cytology (Pap)	35-39	36	Not performed	Satisfactory	NILM	NILM	Negative (NILM)	
P09896	2023	2023-Q1	2023-01	Post-pandemic recovery	Cytology (Pap)	45-49	49	Not performed	Unsatisfactory	Unsatisfactory	Unsatisfactory	Unsatisfactory	
P09897	2023	2023-Q1	2023-01	Post-pandemic recovery	Cytology (Pap)	30-34	30	Not performed	Satisfactory	NILM	NILM	Negative (NILM)	
P09898	2023	2023-Q1	2023-01	Post-pandemic recovery	Cytology (Pap)	60-64	62	Not performed	Satisfactory	NILM	NILM	Negative (NILM)	
P09899	2023	2023-Q1	2023-01	Post-pandemic recovery	Cytology (Pap)	40-44	44	Not performed	Satisfactory	NILM	NILM	Negative (NILM)	
P09900	2023	2023-Q1	2023-01	Post-pandemic recovery	Cytology (Pap)	50-54	52	Not performed	Satisfactory	NILM	NILM	Negative (NILM)	
P09901	2023	2023-Q1	2023-01	Post-pandemic recovery	Cytology (Pap)	55-59	55	Not performed	Satisfactory	NILM	NILM	Negative (NILM)	
P09902	2023	2023-Q1	2023-01	Post-pandemic recovery	Cytology (Pap)	60-64	64	Not performed	Satisfactory	NILM	NILM	Negative (NILM)	
P09903	2023	2023-Q1	2023-01	Post-pandemic recovery	Cytology (Pap)	40-44	41	Not performed	Satisfactory	Positive	ASC-US	Low-grade / equivocal squamous	Candida
P09904	2023	2023-Q1	2023-01	Post-pandemic recovery	Cytology (Pap)	55-59	55	Not performed	Satisfactory	Positive	AGC	Glandular	Candida
P09905	2023	2023-Q1	2023-01	Post-pandemic recovery	Cytology (Pap)	40-44	42	Not performed	Satisfactory	NILM	NILM	Negative (NILM)	
P09906	2023	2023-Q1	2023-01	Post-pandemic recovery	Cytology (Pap)	40-44	43	Not performed	Satisfactory	NILM	NILM	Negative (NILM)	
P09907	2023	2023-Q1	2023-01	Post-pandemic recovery	Cytology (Pap)	45-49	45	Not performed	Satisfactory	NILM	NILM	Negative (NILM)	Gardnerella
P09908	2023	2023-Q1	2023-01	Post-pandemic recovery	Cytology (Pap)	60-64	64	Not performed	Satisfactory	NILM	NILM	Negative (NILM)	
P09909	2023	2023-Q1	2023-01	Post-pandemic recovery	Cytology (Pap)	25-29	29	Not performed	Satisfactory	NILM	NILM	Negative (NILM)	
P09910	2023	2023-Q1	2023-01	Post-pandemic recovery	Cytology (Pap)	30-34	33	Not performed	Satisfactory	NILM	NILM	Negative (NILM)	
P09911	2023	2023-Q1	2023-01	Post-pandemic recovery	Cytology (Pap)	40-44	40	Not performed	Satisfactory	NILM	NILM	Negative (NILM)	
P09912	2023	2023-Q1	2023-01	Post-pandemic recovery	Cytology (Pap)	40-44	44	Not performed	Satisfactory	NILM	NILM	Negative (NILM)	Candida
P09913	2023	2023-Q1	2023-01	Post-pandemic recovery	Cytology (Pap)	55-59	59	Not performed	Satisfactory	NILM	NILM	Negative (NILM)	
P09914	2023	2023-Q1	2023-01	Post-pandemic recovery	Cytology (Pap)	40-44	43	Not performed	Satisfactory	NILM	NILM	Negative (NILM)	
P09915	2023	2023-Q1	2023-01	Post-pandemic recovery	Cytology (Pap)	50-54	54	Not performed	Satisfactory	NILM	NILM	Negative (NILM)	
P09916	2023	2023-Q1	2023-01	Post-pandemic recovery	Cytology (Pap)	30-34	32	Not performed	Satisfactory	Positive	LSIL	Low-grade / equivocal squamous	
P09917	2023	2023-Q1	2023-01	Post-pandemic recovery	Cytology (Pap)	45-49	49	Not performed	Satisfactory	NILM	NILM	Negative (NILM)	
P09918	2023	2023-Q1	2023-01	Post-pandemic recovery	Cytology (Pap)	40-44	41	Not performed	Satisfactory	NILM	NILM	Negative (NILM)	

patient_id	year	quarter	month	period	primary_test	age_group	age_years	hpv_result	adequacy	result	bethesda	grade	infection
P09919	2023	2023-Q1	2023-01	Post-pandemic recovery	Cytology (Pap)	30-34	33	Not performed	Satisfactory	Positive	LSIL	Low-grade / equivocal squamous	
P09920	2023	2023-Q1	2023-01	Post-pandemic recovery	Cytology (Pap)	60-64	61	Not performed	Satisfactory	NILM	NILM	Negative (NILM)	
P09921	2023	2023-Q1	2023-01	Post-pandemic recovery	Cytology (Pap)	35-39	39	Not performed	Satisfactory	NILM	NILM	Negative (NILM)	
P09922	2023	2023-Q1	2023-01	Post-pandemic recovery	Cytology (Pap)	35-39	38	Not performed	Satisfactory	NILM	NILM	Negative (NILM)	
P09923	2023	2023-Q1	2023-01	Post-pandemic recovery	Cytology (Pap)	45-49	49	Not performed	Satisfactory	Positive	HSIL	High-grade squamous	
P09924	2023	2023-Q1	2023-01	Post-pandemic recovery	Cytology (Pap)	30-34	30	Not performed	Satisfactory	NILM	NILM	Negative (NILM)	
P09925	2023	2023-Q1	2023-01	Post-pandemic recovery	Cytology (Pap)	35-39	38	Not performed	Satisfactory	NILM	NILM	Negative (NILM)	
P09926	2023	2023-Q1	2023-01	Post-pandemic recovery	Cytology (Pap)	60-64	64	Not performed	Satisfactory	NILM	NILM	Negative (NILM)	
P09927	2023	2023-Q1	2023-01	Post-pandemic recovery	Cytology (Pap)	35-39	37	Not performed	Satisfactory	NILM	NILM	Negative (NILM)	
P09928	2023	2023-Q1	2023-01	Post-pandemic recovery	Cytology (Pap)	35-39	39	Not performed	Satisfactory	NILM	NILM	Negative (NILM)	
P09929	2023	2023-Q1	2023-01	Post-pandemic recovery	Cytology (Pap)	45-49	48	Not performed	Satisfactory	NILM	NILM	Negative (NILM)	
P09930	2023	2023-Q1	2023-01	Post-pandemic recovery	Cytology (Pap)	45-49	47	Not performed	Satisfactory	NILM	NILM	Negative (NILM)	
P09931	2023	2023-Q1	2023-01	Post-pandemic recovery	Cytology (Pap)	40-44	42	Not performed	Satisfactory	NILM	NILM	Negative (NILM)	
P09932	2023	2023-Q1	2023-01	Post-pandemic recovery	Cytology (Pap)	50-54	51	Not performed	Satisfactory	NILM	NILM	Negative (NILM)	
P09933	2023	2023-Q1	2023-01	Post-pandemic recovery	Cytology (Pap)	60-64	63	Not performed	Satisfactory	NILM	NILM	Negative (NILM)	
P09934	2023	2023-Q1	2023-01	Post-pandemic recovery	Cytology (Pap)	35-39	36	Not performed	Satisfactory	NILM	NILM	Negative (NILM)	
P09935	2023	2023-Q1	2023-01	Post-pandemic recovery	Cytology (Pap)	45-49	47	Not performed	Satisfactory	NILM	NILM	Negative (NILM)	
P09936	2023	2023-Q1	2023-01	Post-pandemic recovery	Cytology (Pap)	45-49	46	Not performed	Satisfactory	NILM	NILM	Negative (NILM)	
P09937	2023	2023-Q1	2023-01	Post-pandemic recovery	Cytology (Pap)	30-34	30	Not performed	Satisfactory	NILM	NILM	Negative (NILM)	
P09938	2023	2023-Q1	2023-01	Post-pandemic recovery	Cytology (Pap)	50-54	51	Not performed	Satisfactory	NILM	NILM	Negative (NILM)	
P09939	2023	2023-Q1	2023-01	Post-pandemic recovery	Cytology (Pap)	40-44	44	Not performed	Satisfactory	NILM	NILM	Negative (NILM)	
P09940	2023	2023-Q1	2023-01	Post-pandemic recovery	Cytology (Pap)	55-59	55	Not performed	Satisfactory	NILM	NILM	Negative (NILM)	
P09941	2023	2023-Q1	2023-01	Post-pandemic recovery	Cytology (Pap)	40-44	41	Not performed	Satisfactory	NILM	NILM	Negative (NILM)	
P09942	2023	2023-Q1	2023-01	Post-pandemic recovery	Cytology (Pap)	55-59	59	Not performed	Satisfactory	NILM	NILM	Negative (NILM)	
P09943	2023	2023-Q1	2023-01	Post-pandemic recovery	Cytology (Pap)	35-39	35	Not performed	Satisfactory	NILM	NILM	Negative (NILM)	
P09944	2023	2023-Q1	2023-01	Post-pandemic recovery	Cytology (Pap)	55-59	57	Not performed	Satisfactory	NILM	NILM	Negative (NILM)	
P09945	2023	2023-Q1	2023-01	Post-pandemic recovery	Cytology (Pap)	25-29	28	Not performed	Satisfactory	NILM	NILM	Negative (NILM)	
P09946	2023	2023-Q1	2023-01	Post-pandemic recovery	Cytology (Pap)	55-59	57	Not performed	Satisfactory	Positive	LSIL	Low-grade / equivocal squamous	
P09947	2023	2023-Q1	2023-01	Post-pandemic recovery	Cytology (Pap)	35-39	35	Not performed	Satisfactory	NILM	NILM	Negative (NILM)	
P09948	2023	2023-Q1	2023-01	Post-pandemic recovery	Cytology (Pap)	35-39	36	Not performed	Satisfactory	NILM	NILM	Negative (NILM)	
P09949	2023	2023-Q1	2023-01	Post-pandemic recovery	Cytology (Pap)	25-29	25	Not performed	Satisfactory	NILM	NILM	Negative (NILM)	
P09950	2023	2023-Q1	2023-01	Post-pandemic recovery	Cytology (Pap)	30-34	32	Not performed	Satisfactory	NILM	NILM	Negative (NILM)	
P09951	2023	2023-Q1	2023-01	Post-pandemic recovery	Cytology (Pap)	25-29	28	Not performed	Satisfactory	NILM	NILM	Negative (NILM)	
P09952	2023	2023-Q1	2023-01	Post-pandemic recovery	Cytology (Pap)	35-39	39	Not performed	Satisfactory	NILM	NILM	Negative (NILM)	
P09953	2023	2023-Q1	2023-01	Post-pandemic recovery	Cytology (Pap)	30-34	34	Not performed	Satisfactory	NILM	NILM	Negative (NILM)	
P09954	2023	2023-Q1	2023-01	Post-pandemic recovery	Cytology (Pap)	40-44	40	Not performed	Satisfactory	NILM	NILM	Negative (NILM)	
P09955	2023	2023-Q1	2023-01	Post-pandemic recovery	Cytology (Pap)	25-29	29	Not performed	Satisfactory	NILM	NILM	Negative (NILM)	
P09956	2023	2023-Q1	2023-01	Post-pandemic recovery	Cytology (Pap)	40-44	43	Not performed	Satisfactory	NILM	NILM	Negative (NILM)	
P09957	2023	2023-Q1	2023-01	Post-pandemic recovery	Cytology (Pap)	35-39	35	Not performed	Satisfactory	NILM	NILM	Negative (NILM)	
P09958	2023	2023-Q1	2023-01	Post-pandemic recovery	Cytology (Pap)	30-34	34	Not performed	Satisfactory	NILM	NILM	Negative (NILM)	
P09959	2023	2023-Q1	2023-01	Post-pandemic recovery	Cytology (Pap)	30-34	34	Not performed	Satisfactory	NILM	NILM	Negative (NILM)	
P09960	2023	2023-Q1	2023-01	Post-pandemic recovery	Cytology (Pap)	40-44	44	Not performed	Satisfactory	Positive	LSIL	Low-grade / equivocal squamous	
P09961	2023	2023-Q1	2023-01	Post-pandemic recovery	Cytology (Pap)	35-39	37	Not performed	Satisfactory	NILM	NILM	Negative (NILM)	
P09962	2023	2023-Q1	2023-01	Post-pandemic recovery	Cytology (Pap)	30-34	31	Not performed	Satisfactory	Positive	ASC-US	Low-grade / equivocal squamous	
P09963	2023	2023-Q1	2023-01	Post-pandemic recovery	Cytology (Pap)	35-39	36	Not performed	Satisfactory	NILM	NILM	Negative (NILM)	
P09964	2023	2023-Q1	2023-01	Post-pandemic recovery	Cytology (Pap)	30-34	34	Not performed	Satisfactory	NILM	NILM	Negative (NILM)	
P09965	2023	2023-Q1	2023-01	Post-pandemic recovery	Cytology (Pap)	30-34	34	Not performed	Satisfactory	NILM	NILM	Negative (NILM)	Other
P09966	2023	2023-Q1	2023-01	Post-pandemic recovery	Cytology (Pap)	45-49	47	Not performed	Satisfactory	NILM	NILM	Negative (NILM)	
P09967	2023	2023-Q1	2023-01	Post-pandemic recovery	Cytology (Pap)	25-29	27	Not performed	Satisfactory	Positive	ASC-US	Low-grade / equivocal squamous	
P09968	2023	2023-Q1	2023-01	Post-pandemic recovery	Cytology (Pap)	40-44	44	Not performed	Satisfactory	NILM	NILM	Negative (NILM)	
P09969	2023	2023-Q1	2023-01	Post-pandemic recovery	Cytology (Pap)	50-54	50	Not performed	Satisfactory	Positive	LSIL	Low-grade / equivocal squamous	
P09970	2023	2023-Q1	2023-01	Post-pandemic recovery	Cytology (Pap)	60-64	61	Not performed	Satisfactory	NILM	NILM	Negative (NILM)	
P09971	2023	2023-Q1	2023-01	Post-pandemic recovery	Cytology (Pap)	40-44	41	Not performed	Satisfactory	NILM	NILM	Negative (NILM)	
P09972	2023	2023-Q1	2023-01	Post-pandemic recovery	Cytology (Pap)	30-34	33	Not performed	Satisfactory	NILM	NILM	Negative (NILM)	
P09973	2023	2023-Q1	2023-01	Post-pandemic recovery	Cytology (Pap)	35-39	36	Not performed	Satisfactory	NILM	NILM	Negative (NILM)	
P09974	2023	2023-Q1	2023-01	Post-pandemic recovery	Cytology (Pap)	35-39	37	Not performed	Satisfactory	NILM	NILM	Negative (NILM)	
P09975	2023	2023-Q1	2023-01	Post-pandemic recovery	Cytology (Pap)	60-64	64	Not performed	Satisfactory	NILM	NILM	Negative (NILM)	
P09976	2023	2023-Q1	2023-01	Post-pandemic recovery	Cytology (Pap)	50-54	54	Not performed	Satisfactory	NILM	NILM	Negative (NILM)	
P09977	2023	2023-Q1	2023-01	Post-pandemic recovery	Cytology (Pap)	30-34	32	Not performed	Satisfactory	Positive	HSIL	High-grade squamous	
P09978	2023	2023-Q1	2023-01	Post-pandemic recovery	Cytology (Pap)	45-49	49	Not performed	Satisfactory	NILM	NILM	Negative (NILM)	
P09979	2023	2023-Q1	2023-01	Post-pandemic recovery	Cytology (Pap)	25-29	28	Not performed	Satisfactory	NILM	NILM	Negative (NILM)	
P09980	2023	2023-Q1	2023-01	Post-pandemic recovery	Cytology (Pap)	40-44	40	Not performed	Satisfactory	NILM	NILM	Negative (NILM)	
P09981	2023	2023-Q1	2023-01	Post-pandemic recovery	Cytology (Pap)	40-44	40	Not performed	Satisfactory	Positive	LSIL	Low-grade / equivocal squamous	
P09982	2023	2023-Q1	2023-01	Post-pandemic recovery	Cytology (Pap)	30-34	32	Not performed	Satisfactory	NILM	NILM	Negative (NILM)	
P09983	2023	2023-Q1	2023-01	Post-pandemic recovery	Cytology (Pap)	25-29	27	Not performed	Satisfactory	Positive	LSIL	Low-grade / equivocal squamous	
P09984	2023	2023-Q1	2023-01	Post-pandemic recovery	Cytology (Pap)	60-64	61	Not performed	Satisfactory	NILM	NILM	Negative (NILM)	Other
P09985	2023	2023-Q1	2023-01	Post-pandemic recovery	Cytology (Pap)	50-54	52	Not performed	Satisfactory	NILM	NILM	Negative (NILM)	
P09986	2023	2023-Q1	2023-01	Post-pandemic recovery	Cytology (Pap)	25-29	29	Not performed	Satisfactory	NILM	NILM	Negative (NILM)	
P09987	2023	2023-Q1	2023-01	Post-pandemic recovery	Cytology (Pap)	55-59	57	Not performed	Satisfactory	Positive	AGC	Glandular	
P09988	2023	2023-Q1	2023-01	Post-pandemic recovery	Cytology (Pap)	30-34	31	Not performed	Satisfactory	NILM	NILM	Negative (NILM)	Candida
P09989	2023	2023-Q1	2023-01	Post-pandemic recovery	Cytology (Pap)	55-59	56	Not performed	Satisfactory	NILM	NILM	Negative (NILM)	
P09990	2023	2023-Q1	2023-01	Post-pandemic recovery	Cytology (Pap)	35-39	39	Not performed	Satisfactory	NILM	NILM	Negative (NILM)	
P09991	2023	2023-Q1	2023-01	Post-pandemic recovery	Cytology (Pap)	50-54	51	Not performed	Satisfactory	NILM	NILM	Negative (NILM)	
P09992	2023	2023-Q1	2023-01	Post-pandemic recovery	Cytology (Pap)	30-34	34	Not performed	Satisfactory	NILM	NILM	Negative (NILM)	
P09993	2023	2023-Q1	2023-01	Post-pandemic recovery	Cytology (Pap)	30-34	31	Not performed	Satisfactory	NILM	NILM	Negative (NILM)	
P09994	2023	2023-Q1	2023-01	Post-pandemic recovery	Cytology (Pap)	30-34	32	Not performed	Satisfactory	NILM	NILM	Negative (NILM)	
P09995	2023	2023-Q1	2023-01	Post-pandemic recovery	Cytology (Pap)	60-64	61	Not performed	Satisfactory	NILM	NILM	Negative (NILM)	
P09996	2023	2023-Q1	2023-01	Post-pandemic recovery	Cytology (Pap)	55-59	59	Not performed	Satisfactory	NILM	NILM	Negative (NILM)	
P09997	2023	2023-Q1	2023-01	Post-pandemic recovery	Cytology (Pap)	35-39	39	Not performed	Satisfactory	NILM	NILM	Negative (NILM)	
P09998	2023	2023-Q1	2023-01	Post-pandemic recovery	Cytology (Pap)	45-49	45	Not performed	Satisfactory	NILM	NILM	Negative (NILM)	

patient_id	year	quarter	month	period	primary_test	age_group	age_years	hpv_result	adequacy	result	bethesda	grade	infection
P09999	2023	2023-Q1	2023-01	Post-pandemic recovery	Cytology (Pap)	60-64	62	Not performed	Satisfactory	NILM	NILM	Negative (NILM)	
P10000	2023	2023-Q1	2023-01	Post-pandemic recovery	Cytology (Pap)	50-54	50	Not performed	Satisfactory	NILM	NILM	Negative (NILM)	
P10001	2023	2023-Q1	2023-01	Post-pandemic recovery	Cytology (Pap)	45-49	49	Not performed	Satisfactory	NILM	NILM	Negative (NILM)	
P10002	2023	2023-Q1	2023-01	Post-pandemic recovery	Cytology (Pap)	30-34	31	Not performed	Satisfactory	NILM	NILM	Negative (NILM)	
P10003	2023	2023-Q1	2023-01	Post-pandemic recovery	Cytology (Pap)	50-54	54	Not performed	Satisfactory	NILM	NILM	Negative (NILM)	
P10004	2023	2023-Q1	2023-01	Post-pandemic recovery	Cytology (Pap)	40-44	44	Not performed	Satisfactory	NILM	NILM	Negative (NILM)	
P10005	2023	2023-Q1	2023-01	Post-pandemic recovery	Cytology (Pap)	30-34	31	Not performed	Satisfactory	NILM	NILM	Negative (NILM)	
P10006	2023	2023-Q1	2023-01	Post-pandemic recovery	Cytology (Pap)	45-49	48	Not performed	Satisfactory	NILM	NILM	Negative (NILM)	
P10007	2023	2023-Q1	2023-01	Post-pandemic recovery	Cytology (Pap)	35-39	39	Not performed	Satisfactory	NILM	NILM	Negative (NILM)	
P10008	2023	2023-Q1	2023-01	Post-pandemic recovery	Cytology (Pap)	40-44	42	Not performed	Satisfactory	NILM	NILM	Negative (NILM)	
P10009	2023	2023-Q1	2023-01	Post-pandemic recovery	Cytology (Pap)	55-59	57	Not performed	Satisfactory	NILM	NILM	Negative (NILM)	
P10010	2023	2023-Q1	2023-01	Post-pandemic recovery	Cytology (Pap)	60-64	62	Not performed	Satisfactory	NILM	NILM	Negative (NILM)	
P10011	2023	2023-Q1	2023-01	Post-pandemic recovery	Cytology (Pap)	35-39	37	Not performed	Satisfactory	Positive	HSIL	High-grade squamous	
P10012	2023	2023-Q1	2023-01	Post-pandemic recovery	Cytology (Pap)	30-34	30	Not performed	Satisfactory	NILM	NILM	Negative (NILM)	
P10013	2023	2023-Q1	2023-01	Post-pandemic recovery	Cytology (Pap)	35-39	37	Not performed	Satisfactory	NILM	NILM	Negative (NILM)	
P10014	2023	2023-Q1	2023-01	Post-pandemic recovery	Cytology (Pap)	25-29	29	Not performed	Satisfactory	NILM	NILM	Negative (NILM)	Gardnerella
P10015	2023	2023-Q1	2023-01	Post-pandemic recovery	Cytology (Pap)	50-54	50	Not performed	Satisfactory	NILM	NILM	Negative (NILM)	
P10016	2023	2023-Q1	2023-01	Post-pandemic recovery	Cytology (Pap)	45-49	47	Not performed	Satisfactory	NILM	NILM	Negative (NILM)	
P10017	2023	2023-Q1	2023-01	Post-pandemic recovery	Cytology (Pap)	35-39	37	Not performed	Satisfactory	Positive	LSIL	Low-grade / equivocal squamous	
P10018	2023	2023-Q1	2023-02	Post-pandemic recovery	Cytology (Pap)	45-49	49	Not performed	Satisfactory	NILM	NILM	Negative (NILM)	
P10019	2023	2023-Q1	2023-02	Post-pandemic recovery	Cytology (Pap)	35-39	35	Not performed	Satisfactory	NILM	NILM	Negative (NILM)	
P10020	2023	2023-Q1	2023-02	Post-pandemic recovery	Cytology (Pap)	55-59	58	Not performed	Satisfactory	NILM	NILM	Negative (NILM)	
P10021	2023	2023-Q1	2023-02	Post-pandemic recovery	Cytology (Pap)	50-54	54	Not performed	Satisfactory	NILM	NILM	Negative (NILM)	
P10022	2023	2023-Q1	2023-02	Post-pandemic recovery	Cytology (Pap)	45-49	46	Not performed	Satisfactory	NILM	NILM	Negative (NILM)	Other
P10023	2023	2023-Q1	2023-02	Post-pandemic recovery	Cytology (Pap)	60-64	64	Not performed	Satisfactory	NILM	NILM	Negative (NILM)	
P10024	2023	2023-Q1	2023-02	Post-pandemic recovery	Cytology (Pap)	25-29	27	Not performed	Satisfactory	NILM	NILM	Negative (NILM)	
P10025	2023	2023-Q1	2023-02	Post-pandemic recovery	Cytology (Pap)	40-44	40	Not performed	Satisfactory	NILM	NILM	Negative (NILM)	
P10026	2023	2023-Q1	2023-02	Post-pandemic recovery	Cytology (Pap)	30-34	32	Not performed	Satisfactory	Positive	ASC-US	Low-grade / equivocal squamous	
P10027	2023	2023-Q1	2023-02	Post-pandemic recovery	Cytology (Pap)	35-39	36	Not performed	Satisfactory	NILM	NILM	Negative (NILM)	
P10028	2023	2023-Q1	2023-02	Post-pandemic recovery	Cytology (Pap)	35-39	37	Not performed	Satisfactory	NILM	NILM	Negative (NILM)	
P10029	2023	2023-Q1	2023-02	Post-pandemic recovery	Cytology (Pap)	25-29	25	Not performed	Satisfactory	NILM	NILM	Negative (NILM)	
P10030	2023	2023-Q1	2023-02	Post-pandemic recovery	Cytology (Pap)	40-44	43	Not performed	Satisfactory	NILM	NILM	Negative (NILM)	Gardnerella
P10031	2023	2023-Q1	2023-02	Post-pandemic recovery	Cytology (Pap)	25-29	25	Not performed	Satisfactory	Positive	ASC-US	Low-grade / equivocal squamous	
P10032	2023	2023-Q1	2023-02	Post-pandemic recovery	Cytology (Pap)	25-29	26	Not performed	Satisfactory	NILM	NILM	Negative (NILM)	
P10033	2023	2023-Q1	2023-02	Post-pandemic recovery	Cytology (Pap)	40-44	41	Not performed	Satisfactory	NILM	NILM	Negative (NILM)	
P10034	2023	2023-Q1	2023-02	Post-pandemic recovery	Cytology (Pap)	30-34	33	Not performed	Satisfactory	NILM	NILM	Negative (NILM)	
P10035	2023	2023-Q1	2023-02	Post-pandemic recovery	Cytology (Pap)	50-54	54	Not performed	Satisfactory	NILM	NILM	Negative (NILM)	
P10036	2023	2023-Q1	2023-02	Post-pandemic recovery	Cytology (Pap)	40-44	40	Not performed	Satisfactory	NILM	NILM	Negative (NILM)	
P10037	2023	2023-Q1	2023-02	Post-pandemic recovery	Cytology (Pap)	50-54	53	Not performed	Satisfactory	NILM	NILM	Negative (NILM)	
P10038	2023	2023-Q1	2023-02	Post-pandemic recovery	Cytology (Pap)	45-49	47	Not performed	Satisfactory	NILM	NILM	Negative (NILM)	
P10039	2023	2023-Q1	2023-02	Post-pandemic recovery	Cytology (Pap)	50-54	53	Not performed	Satisfactory	NILM	NILM	Negative (NILM)	
P10040	2023	2023-Q1	2023-02	Post-pandemic recovery	Cytology (Pap)	30-34	31	Not performed	Satisfactory	NILM	NILM	Negative (NILM)	
P10041	2023	2023-Q1	2023-02	Post-pandemic recovery	Cytology (Pap)	45-49	49	Not performed	Satisfactory	NILM	NILM	Negative (NILM)	
P10042	2023	2023-Q1	2023-02	Post-pandemic recovery	Cytology (Pap)	50-54	51	Not performed	Satisfactory	NILM	NILM	Negative (NILM)	
P10043	2023	2023-Q1	2023-02	Post-pandemic recovery	Cytology (Pap)	45-49	48	Not performed	Satisfactory	NILM	NILM	Negative (NILM)	
P10044	2023	2023-Q1	2023-02	Post-pandemic recovery	Cytology (Pap)	30-34	33	Not performed	Satisfactory	NILM	NILM	Negative (NILM)	
P10045	2023	2023-Q1	2023-02	Post-pandemic recovery	Cytology (Pap)	50-54	52	Not performed	Satisfactory	NILM	NILM	Negative (NILM)	
P10046	2023	2023-Q1	2023-02	Post-pandemic recovery	Cytology (Pap)	35-39	36	Not performed	Satisfactory	NILM	NILM	Negative (NILM)	
P10047	2023	2023-Q1	2023-02	Post-pandemic recovery	Cytology (Pap)	40-44	44	Not performed	Satisfactory	NILM	NILM	Negative (NILM)	
P10048	2023	2023-Q1	2023-02	Post-pandemic recovery	Cytology (Pap)	40-44	42	Not performed	Satisfactory	NILM	NILM	Negative (NILM)	
P10049	2023	2023-Q1	2023-02	Post-pandemic recovery	Cytology (Pap)	40-44	42	Not performed	Satisfactory	NILM	NILM	Negative (NILM)	
P10050	2023	2023-Q1	2023-02	Post-pandemic recovery	Cytology (Pap)	50-54	52	Not performed	Satisfactory	NILM	NILM	Negative (NILM)	
P10051	2023	2023-Q1	2023-02	Post-pandemic recovery	Cytology (Pap)	30-34	34	Not performed	Satisfactory	NILM	NILM	Negative (NILM)	
P10052	2023	2023-Q1	2023-02	Post-pandemic recovery	Cytology (Pap)	25-29	27	Not performed	Satisfactory	NILM	NILM	Negative (NILM)	
P10053	2023	2023-Q1	2023-02	Post-pandemic recovery	Cytology (Pap)	40-44	44	Not performed	Satisfactory	NILM	NILM	Negative (NILM)	
P10054	2023	2023-Q1	2023-02	Post-pandemic recovery	Cytology (Pap)	25-29	28	Not performed	Satisfactory	Positive	ASC-US	Low-grade / equivocal squamous	
P10055	2023	2023-Q1	2023-02	Post-pandemic recovery	Cytology (Pap)	45-49	49	Not performed	Satisfactory	Positive	LSIL	Low-grade / equivocal squamous	
P10056	2023	2023-Q1	2023-02	Post-pandemic recovery	Cytology (Pap)	50-54	54	Not performed	Satisfactory	NILM	NILM	Negative (NILM)	
P10057	2023	2023-Q1	2023-02	Post-pandemic recovery	Cytology (Pap)	30-34	34	Not performed	Satisfactory	NILM	NILM	Negative (NILM)	
P10058	2023	2023-Q1	2023-02	Post-pandemic recovery	Cytology (Pap)	25-29	27	Not performed	Satisfactory	NILM	NILM	Negative (NILM)	
P10059	2023	2023-Q1	2023-02	Post-pandemic recovery	Cytology (Pap)	25-29	28	Not performed	Satisfactory	NILM	NILM	Negative (NILM)	
P10060	2023	2023-Q1	2023-02	Post-pandemic recovery	Cytology (Pap)	35-39	36	Not performed	Satisfactory	NILM	NILM	Negative (NILM)	
P10061	2023	2023-Q1	2023-02	Post-pandemic recovery	Cytology (Pap)	35-39	38	Not performed	Satisfactory	NILM	NILM	Negative (NILM)	
P10062	2023	2023-Q1	2023-02	Post-pandemic recovery	Cytology (Pap)	40-44	40	Not performed	Satisfactory	Positive	ASC-H	High-grade squamous	
P10063	2023	2023-Q1	2023-02	Post-pandemic recovery	Cytology (Pap)	35-39	37	Not performed	Satisfactory	NILM	NILM	Negative (NILM)	
P10064	2023	2023-Q1	2023-02	Post-pandemic recovery	Cytology (Pap)	35-39	37	Not performed	Satisfactory	NILM	NILM	Negative (NILM)	
P10065	2023	2023-Q1	2023-02	Post-pandemic recovery	Cytology (Pap)	45-49	45	Not performed	Satisfactory	NILM	NILM	Negative (NILM)	
P10066	2023	2023-Q1	2023-02	Post-pandemic recovery	Cytology (Pap)	35-39	36	Not performed	Satisfactory	NILM	NILM	Negative (NILM)	
P10067	2023	2023-Q1	2023-02	Post-pandemic recovery	Cytology (Pap)	30-34	33	Not performed	Satisfactory	NILM	NILM	Negative (NILM)	
P10068	2023	2023-Q1	2023-02	Post-pandemic recovery	Cytology (Pap)	30-34	33	Not performed	Satisfactory	NILM	NILM	Negative (NILM)	
P10069	2023	2023-Q1	2023-02	Post-pandemic recovery	Cytology (Pap)	40-44	40	Not performed	Satisfactory	NILM	NILM	Negative (NILM)	
P10070	2023	2023-Q1	2023-02	Post-pandemic recovery	Cytology (Pap)	25-29	29	Not performed	Satisfactory	NILM	NILM	Negative (NILM)	Candida
P10071	2023	2023-Q1	2023-02	Post-pandemic recovery	Cytology (Pap)	40-44	42	Not performed	Satisfactory	NILM	NILM	Negative (NILM)	
P10072	2023	2023-Q1	2023-02	Post-pandemic recovery	Cytology (Pap)	35-39	36	Not performed	Satisfactory	NILM	NILM	Negative (NILM)	Candida
P10073	2023	2023-Q1	2023-02	Post-pandemic recovery	Cytology (Pap)	45-49	46	Not performed	Satisfactory	NILM	NILM	Negative (NILM)	
P10074	2023	2023-Q1	2023-02	Post-pandemic recovery	Cytology (Pap)	30-34	34	Not performed	Satisfactory	Positive	ASC-US	Low-grade / equivocal squamous	
P10075	2023	2023-Q1	2023-02	Post-pandemic recovery	Cytology (Pap)	35-39	38	Not performed	Satisfactory	NILM	NILM	Negative (NILM)	
P10076	2023	2023-Q1	2023-02	Post-pandemic recovery	Cytology (Pap)	25-29	25	Not performed	Satisfactory	NILM	NILM	Negative (NILM)	
P10077	2023	2023-Q1	2023-02	Post-pandemic recovery	Cytology (Pap)	55-59	58	Not performed	Satisfactory	NILM	NILM	Negative (NILM)	
P10078	2023	2023-Q1	2023-02	Post-pandemic recovery	Cytology (Pap)	50-54	54	Not performed	Satisfactory	NILM	NILM	Negative (NILM)	

patient_id	year	quarter	month	period	primary_test	age_group	age_years	hpv_result	adequacy	result	bethesda	grade	infection
P10079	2023	2023-Q1	2023-02	Post-pandemic recovery	Cytology (Pap)	45-49	46	Not performed	Satisfactory	Positive	HSIL	High-grade squamous	
P10080	2023	2023-Q1	2023-02	Post-pandemic recovery	Cytology (Pap)	55-59	59	Not performed	Satisfactory	Positive	ASC-US	Low-grade / equivocal squamous	
P10081	2023	2023-Q1	2023-02	Post-pandemic recovery	Cytology (Pap)	45-49	45	Not performed	Satisfactory	Positive	LSIL	Low-grade / equivocal squamous	
P10082	2023	2023-Q1	2023-02	Post-pandemic recovery	Cytology (Pap)	45-49	45	Not performed	Satisfactory	NILM	NILM	Negative (NILM)	
P10083	2023	2023-Q1	2023-02	Post-pandemic recovery	Cytology (Pap)	25-29	27	Not performed	Satisfactory	NILM	NILM	Negative (NILM)	
P10084	2023	2023-Q1	2023-02	Post-pandemic recovery	Cytology (Pap)	40-44	42	Not performed	Satisfactory	NILM	NILM	Negative (NILM)	
P10085	2023	2023-Q1	2023-02	Post-pandemic recovery	Cytology (Pap)	35-39	37	Not performed	Satisfactory	NILM	NILM	Negative (NILM)	
P10086	2023	2023-Q1	2023-02	Post-pandemic recovery	Cytology (Pap)	55-59	59	Not performed	Satisfactory	Positive	AGC	Glandular	
P10087	2023	2023-Q1	2023-02	Post-pandemic recovery	Cytology (Pap)	35-39	39	Not performed	Satisfactory	NILM	NILM	Negative (NILM)	
P10088	2023	2023-Q1	2023-02	Post-pandemic recovery	Cytology (Pap)	60-64	61	Not performed	Satisfactory	Positive	AGC	Glandular	Other
P10089	2023	2023-Q1	2023-02	Post-pandemic recovery	Cytology (Pap)	55-59	58	Not performed	Satisfactory	NILM	NILM	Negative (NILM)	
P10090	2023	2023-Q1	2023-02	Post-pandemic recovery	Cytology (Pap)	35-39	36	Not performed	Satisfactory	NILM	NILM	Negative (NILM)	
P10091	2023	2023-Q1	2023-02	Post-pandemic recovery	Cytology (Pap)	30-34	33	Not performed	Satisfactory	Positive	ASC-US	Low-grade / equivocal squamous	
P10092	2023	2023-Q1	2023-02	Post-pandemic recovery	Cytology (Pap)	45-49	47	Not performed	Satisfactory	NILM	NILM	Negative (NILM)	
P10093	2023	2023-Q1	2023-02	Post-pandemic recovery	Cytology (Pap)	30-34	31	Not performed	Satisfactory	NILM	NILM	Negative (NILM)	
P10094	2023	2023-Q1	2023-02	Post-pandemic recovery	Cytology (Pap)	60-64	62	Not performed	Satisfactory	NILM	NILM	Negative (NILM)	
P10095	2023	2023-Q1	2023-02	Post-pandemic recovery	Cytology (Pap)	45-49	49	Not performed	Satisfactory	NILM	NILM	Negative (NILM)	
P10096	2023	2023-Q1	2023-02	Post-pandemic recovery	Cytology (Pap)	35-39	35	Not performed	Satisfactory	NILM	NILM	Negative (NILM)	
P10097	2023	2023-Q1	2023-02	Post-pandemic recovery	Cytology (Pap)	45-49	45	Not performed	Satisfactory	NILM	NILM	Negative (NILM)	
P10098	2023	2023-Q1	2023-02	Post-pandemic recovery	Cytology (Pap)	45-49	46	Not performed	Satisfactory	NILM	NILM	Negative (NILM)	
P10099	2023	2023-Q1	2023-02	Post-pandemic recovery	Cytology (Pap)	55-59	58	Not performed	Satisfactory	Positive	ASC-US	Low-grade / equivocal squamous	
P10100	2023	2023-Q1	2023-02	Post-pandemic recovery	Cytology (Pap)	40-44	44	Not performed	Satisfactory	Positive	ASC-H	High-grade squamous	Candida
P10101	2023	2023-Q1	2023-02	Post-pandemic recovery	Cytology (Pap)	35-39	38	Not performed	Satisfactory	NILM	NILM	Negative (NILM)	
P10102	2023	2023-Q1	2023-02	Post-pandemic recovery	Cytology (Pap)	55-59	59	Not performed	Satisfactory	NILM	NILM	Negative (NILM)	
P10103	2023	2023-Q1	2023-02	Post-pandemic recovery	Cytology (Pap)	55-59	59	Not performed	Satisfactory	NILM	NILM	Negative (NILM)	
P10104	2023	2023-Q1	2023-02	Post-pandemic recovery	Cytology (Pap)	55-59	58	Not performed	Satisfactory	NILM	NILM	Negative (NILM)	
P10105	2023	2023-Q1	2023-02	Post-pandemic recovery	Cytology (Pap)	45-49	48	Not performed	Satisfactory	NILM	NILM	Negative (NILM)	
P10106	2023	2023-Q1	2023-02	Post-pandemic recovery	Cytology (Pap)	25-29	29	Not performed	Satisfactory	NILM	NILM	Negative (NILM)	
P10107	2023	2023-Q1	2023-02	Post-pandemic recovery	Cytology (Pap)	50-54	53	Not performed	Satisfactory	NILM	NILM	Negative (NILM)	
P10108	2023	2023-Q1	2023-02	Post-pandemic recovery	Cytology (Pap)	50-54	53	Not performed	Satisfactory	NILM	NILM	Negative (NILM)	
P10109	2023	2023-Q1	2023-02	Post-pandemic recovery	Cytology (Pap)	60-64	64	Not performed	Satisfactory	NILM	NILM	Negative (NILM)	
P10110	2023	2023-Q1	2023-02	Post-pandemic recovery	Cytology (Pap)	40-44	44	Not performed	Satisfactory	NILM	NILM	Negative (NILM)	
P10111	2023	2023-Q1	2023-02	Post-pandemic recovery	Cytology (Pap)	30-34	31	Not performed	Satisfactory	NILM	NILM	Negative (NILM)	
P10112	2023	2023-Q1	2023-02	Post-pandemic recovery	Cytology (Pap)	30-34	34	Not performed	Satisfactory	NILM	NILM	Negative (NILM)	
P10113	2023	2023-Q1	2023-02	Post-pandemic recovery	Cytology (Pap)	30-34	31	Not performed	Satisfactory	NILM	NILM	Negative (NILM)	
P10114	2023	2023-Q1	2023-02	Post-pandemic recovery	Cytology (Pap)	25-29	26	Not performed	Satisfactory	NILM	NILM	Negative (NILM)	
P10115	2023	2023-Q1	2023-02	Post-pandemic recovery	Cytology (Pap)	60-64	63	Not performed	Satisfactory	NILM	NILM	Negative (NILM)	
P10116	2023	2023-Q1	2023-02	Post-pandemic recovery	Cytology (Pap)	40-44	44	Not performed	Unsatisfactory	Unsatisfactory	Unsatisfactory	Unsatisfactory	
P10117	2023	2023-Q1	2023-02	Post-pandemic recovery	Cytology (Pap)	45-49	47	Not performed	Satisfactory	NILM	NILM	Negative (NILM)	
P10118	2023	2023-Q1	2023-02	Post-pandemic recovery	Cytology (Pap)	25-29	28	Not performed	Unsatisfactory	Unsatisfactory	Unsatisfactory	Unsatisfactory	
P10119	2023	2023-Q1	2023-02	Post-pandemic recovery	Cytology (Pap)	55-59	59	Not performed	Satisfactory	NILM	NILM	Negative (NILM)	
P10120	2023	2023-Q1	2023-02	Post-pandemic recovery	Cytology (Pap)	25-29	25	Not performed	Satisfactory	NILM	NILM	Negative (NILM)	
P10121	2023	2023-Q1	2023-02	Post-pandemic recovery	Cytology (Pap)	35-39	35	Not performed	Satisfactory	NILM	NILM	Negative (NILM)	
P10122	2023	2023-Q1	2023-02	Post-pandemic recovery	Cytology (Pap)	50-54	54	Not performed	Satisfactory	NILM	NILM	Negative (NILM)	
P10123	2023	2023-Q1	2023-02	Post-pandemic recovery	Cytology (Pap)	40-44	41	Not performed	Satisfactory	NILM	NILM	Negative (NILM)	
P10124	2023	2023-Q1	2023-02	Post-pandemic recovery	Cytology (Pap)	45-49	45	Not performed	Satisfactory	NILM	NILM	Negative (NILM)	
P10125	2023	2023-Q1	2023-02	Post-pandemic recovery	Cytology (Pap)	60-64	61	Not performed	Satisfactory	NILM	NILM	Negative (NILM)	
P10126	2023	2023-Q1	2023-02	Post-pandemic recovery	Cytology (Pap)	25-29	29	Not performed	Satisfactory	NILM	NILM	Negative (NILM)	
P10127	2023	2023-Q1	2023-02	Post-pandemic recovery	Cytology (Pap)	25-29	25	Not performed	Satisfactory	Positive	LSIL	Low-grade / equivocal squamous	
P10128	2023	2023-Q1	2023-02	Post-pandemic recovery	Cytology (Pap)	40-44	41	Not performed	Satisfactory	NILM	NILM	Negative (NILM)	
P10129	2023	2023-Q1	2023-02	Post-pandemic recovery	Cytology (Pap)	30-34	31	Not performed	Satisfactory	NILM	NILM	Negative (NILM)	
P10130	2023	2023-Q1	2023-02	Post-pandemic recovery	Cytology (Pap)	35-39	37	Not performed	Satisfactory	NILM	NILM	Negative (NILM)	
P10131	2023	2023-Q1	2023-02	Post-pandemic recovery	Cytology (Pap)	40-44	44	Not performed	Satisfactory	NILM	NILM	Negative (NILM)	
P10132	2023	2023-Q1	2023-02	Post-pandemic recovery	Cytology (Pap)	40-44	41	Not performed	Satisfactory	NILM	NILM	Negative (NILM)	Gardnerella
P10133	2023	2023-Q1	2023-02	Post-pandemic recovery	Cytology (Pap)	60-64	64	Not performed	Satisfactory	NILM	NILM	Negative (NILM)	
P10134	2023	2023-Q1	2023-02	Post-pandemic recovery	Cytology (Pap)	45-49	46	Not performed	Satisfactory	NILM	NILM	Negative (NILM)	
P10135	2023	2023-Q1	2023-02	Post-pandemic recovery	Cytology (Pap)	45-49	46	Not performed	Satisfactory	NILM	NILM	Negative (NILM)	
P10136	2023	2023-Q1	2023-02	Post-pandemic recovery	Cytology (Pap)	30-34	32	Not performed	Satisfactory	NILM	NILM	Negative (NILM)	
P10137	2023	2023-Q1	2023-02	Post-pandemic recovery	Cytology (Pap)	25-29	29	Not performed	Satisfactory	NILM	NILM	Negative (NILM)	
P10138	2023	2023-Q1	2023-02	Post-pandemic recovery	Cytology (Pap)	40-44	41	Not performed	Satisfactory	NILM	NILM	Negative (NILM)	
P10139	2023	2023-Q1	2023-02	Post-pandemic recovery	Cytology (Pap)	30-34	32	Not performed	Satisfactory	NILM	NILM	Negative (NILM)	
P10140	2023	2023-Q1	2023-02	Post-pandemic recovery	Cytology (Pap)	40-44	44	Not performed	Satisfactory	Positive	ASC-US	Low-grade / equivocal squamous	
P10141	2023	2023-Q1	2023-02	Post-pandemic recovery	Cytology (Pap)	35-39	38	Not performed	Satisfactory	NILM	NILM	Negative (NILM)	
P10142	2023	2023-Q1	2023-02	Post-pandemic recovery	Cytology (Pap)	50-54	53	Not performed	Satisfactory	Positive	ASC-US	Low-grade / equivocal squamous	
P10143	2023	2023-Q1	2023-02	Post-pandemic recovery	Cytology (Pap)	60-64	62	Not performed	Satisfactory	NILM	NILM	Negative (NILM)	
P10144	2023	2023-Q1	2023-02	Post-pandemic recovery	Cytology (Pap)	35-39	39	Not performed	Satisfactory	NILM	NILM	Negative (NILM)	
P10145	2023	2023-Q1	2023-02	Post-pandemic recovery	Cytology (Pap)	25-29	28	Not performed	Satisfactory	NILM	NILM	Negative (NILM)	
P10146	2023	2023-Q1	2023-02	Post-pandemic recovery	Cytology (Pap)	35-39	37	Not performed	Satisfactory	NILM	NILM	Negative (NILM)	
P10147	2023	2023-Q1	2023-02	Post-pandemic recovery	Cytology (Pap)	30-34	33	Not performed	Satisfactory	NILM	NILM	Negative (NILM)	
P10148	2023	2023-Q1	2023-02	Post-pandemic recovery	Cytology (Pap)	30-34	33	Not performed	Satisfactory	NILM	NILM	Negative (NILM)	
P10149	2023	2023-Q1	2023-02	Post-pandemic recovery	Cytology (Pap)	30-34	30	Not performed	Satisfactory	NILM	NILM	Negative (NILM)	
P10150	2023	2023-Q1	2023-02	Post-pandemic recovery	Cytology (Pap)	25-29	28	Not performed	Satisfactory	NILM	NILM	Negative (NILM)	
P10151	2023	2023-Q1	2023-02	Post-pandemic recovery	Cytology (Pap)	40-44	40	Not performed	Satisfactory	NILM	NILM	Negative (NILM)	
P10152	2023	2023-Q1	2023-02	Post-pandemic recovery	Cytology (Pap)	25-29	26	Not performed	Satisfactory	NILM	NILM	Negative (NILM)	
P10153	2023	2023-Q1	2023-02	Post-pandemic recovery	Cytology (Pap)	40-44	44	Not performed	Satisfactory	NILM	NILM	Negative (NILM)	
P10154	2023	2023-Q1	2023-02	Post-pandemic recovery	Cytology (Pap)	35-39	37	Not performed	Satisfactory	NILM	NILM	Negative (NILM)	
P10155	2023	2023-Q1	2023-02	Post-pandemic recovery	Cytology (Pap)	25-29	27	Not performed	Satisfactory	NILM	NILM	Negative (NILM)	
P10156	2023	2023-Q1	2023-02	Post-pandemic recovery	Cytology (Pap)	40-44	41	Not performed	Satisfactory	Positive	LSIL	Low-grade / equivocal squamous	Candida
P10157	2023	2023-Q1	2023-02	Post-pandemic recovery	Cytology (Pap)	45-49	46	Not performed	Satisfactory	NILM	NILM	Negative (NILM)	
P10158	2023	2023-Q1	2023-02	Post-pandemic recovery	Cytology (Pap)	40-44	40	Not performed	Satisfactory	NILM	NILM	Negative (NILM)	

patient_id	year	quarter	month	period	primary_test	age_group	age_years	hpv_result	adequacy	result	bethesda	grade	infection
P10159	2023	2023-Q1	2023-02	Post-pandemic recovery	Cytology (Pap)	30-34	32	Not performed	Satisfactory	NILM	NILM	Negative (NILM)	
P10160	2023	2023-Q1	2023-02	Post-pandemic recovery	Cytology (Pap)	50-54	53	Not performed	Satisfactory	Positive	ASC-US	Low-grade / equivocal squamous	
P10161	2023	2023-Q1	2023-02	Post-pandemic recovery	Cytology (Pap)	25-29	26	Not performed	Satisfactory	NILM	NILM	Negative (NILM)	
P10162	2023	2023-Q1	2023-02	Post-pandemic recovery	Cytology (Pap)	50-54	53	Not performed	Satisfactory	NILM	NILM	Negative (NILM)	
P10163	2023	2023-Q1	2023-02	Post-pandemic recovery	Cytology (Pap)	60-64	60	Not performed	Unsatisfactory	Unsatisfactory	Unsatisfactory	Unsatisfactory	Other
P10164	2023	2023-Q1	2023-02	Post-pandemic recovery	Cytology (Pap)	25-29	28	Not performed	Satisfactory	NILM	NILM	Negative (NILM)	
P10165	2023	2023-Q1	2023-02	Post-pandemic recovery	Cytology (Pap)	25-29	29	Not performed	Satisfactory	NILM	NILM	Negative (NILM)	
P10166	2023	2023-Q1	2023-02	Post-pandemic recovery	Cytology (Pap)	35-39	38	Not performed	Satisfactory	NILM	NILM	Negative (NILM)	Trichomonas
P10167	2023	2023-Q1	2023-02	Post-pandemic recovery	Cytology (Pap)	35-39	36	Not performed	Satisfactory	NILM	NILM	Negative (NILM)	
P10168	2023	2023-Q1	2023-02	Post-pandemic recovery	Cytology (Pap)	30-34	33	Not performed	Satisfactory	NILM	NILM	Negative (NILM)	
P10169	2023	2023-Q1	2023-02	Post-pandemic recovery	Cytology (Pap)	35-39	36	Not performed	Unsatisfactory	Unsatisfactory	Unsatisfactory	Unsatisfactory	
P10170	2023	2023-Q1	2023-02	Post-pandemic recovery	Cytology (Pap)	40-44	43	Not performed	Satisfactory	NILM	NILM	Negative (NILM)	
P10171	2023	2023-Q1	2023-02	Post-pandemic recovery	Cytology (Pap)	30-34	34	Not performed	Satisfactory	NILM	NILM	Negative (NILM)	
P10172	2023	2023-Q1	2023-02	Post-pandemic recovery	Cytology (Pap)	60-64	62	Not performed	Satisfactory	Positive	ASC-US	Low-grade / equivocal squamous	
P10173	2023	2023-Q1	2023-02	Post-pandemic recovery	Cytology (Pap)	35-39	39	Not performed	Unsatisfactory	Unsatisfactory	Unsatisfactory	Unsatisfactory	
P10174	2023	2023-Q1	2023-02	Post-pandemic recovery	Cytology (Pap)	35-39	37	Not performed	Satisfactory	NILM	NILM	Negative (NILM)	
P10175	2023	2023-Q1	2023-02	Post-pandemic recovery	Cytology (Pap)	50-54	53	Not performed	Satisfactory	NILM	NILM	Negative (NILM)	
P10176	2023	2023-Q1	2023-02	Post-pandemic recovery	Cytology (Pap)	55-59	56	Not performed	Satisfactory	NILM	NILM	Negative (NILM)	
P10177	2023	2023-Q1	2023-02	Post-pandemic recovery	Cytology (Pap)	25-29	27	Not performed	Unsatisfactory	Unsatisfactory	Unsatisfactory	Unsatisfactory	
P10178	2023	2023-Q1	2023-03	Post-pandemic recovery	Cytology (Pap)	35-39	38	Not performed	Satisfactory	NILM	NILM	Negative (NILM)	
P10179	2023	2023-Q1	2023-03	Post-pandemic recovery	Cytology (Pap)	45-49	48	Not performed	Satisfactory	NILM	NILM	Negative (NILM)	
P10180	2023	2023-Q1	2023-03	Post-pandemic recovery	Cytology (Pap)	35-39	39	Not performed	Satisfactory	NILM	NILM	Negative (NILM)	
P10181	2023	2023-Q1	2023-03	Post-pandemic recovery	Cytology (Pap)	40-44	41	Not performed	Unsatisfactory	Unsatisfactory	Unsatisfactory	Unsatisfactory	
P10182	2023	2023-Q1	2023-03	Post-pandemic recovery	Cytology (Pap)	40-44	41	Not performed	Satisfactory	NILM	NILM	Negative (NILM)	
P10183	2023	2023-Q1	2023-03	Post-pandemic recovery	Cytology (Pap)	60-64	63	Not performed	Satisfactory	NILM	NILM	Negative (NILM)	
P10184	2023	2023-Q1	2023-03	Post-pandemic recovery	Cytology (Pap)	30-34	31	Not performed	Satisfactory	NILM	NILM	Negative (NILM)	
P10185	2023	2023-Q1	2023-03	Post-pandemic recovery	Cytology (Pap)	40-44	43	Not performed	Satisfactory	NILM	NILM	Negative (NILM)	
P10186	2023	2023-Q1	2023-03	Post-pandemic recovery	Cytology (Pap)	60-64	64	Not performed	Satisfactory	NILM	NILM	Negative (NILM)	
P10187	2023	2023-Q1	2023-03	Post-pandemic recovery	Cytology (Pap)	25-29	26	Not performed	Satisfactory	NILM	NILM	Negative (NILM)	
P10188	2023	2023-Q1	2023-03	Post-pandemic recovery	Cytology (Pap)	30-34	30	Not performed	Satisfactory	NILM	NILM	Negative (NILM)	
P10189	2023	2023-Q1	2023-03	Post-pandemic recovery	Cytology (Pap)	40-44	40	Not performed	Satisfactory	NILM	NILM	Negative (NILM)	
P10190	2023	2023-Q1	2023-03	Post-pandemic recovery	Cytology (Pap)	35-39	39	Not performed	Satisfactory	NILM	NILM	Negative (NILM)	
P10191	2023	2023-Q1	2023-03	Post-pandemic recovery	Cytology (Pap)	55-59	55	Not performed	Satisfactory	NILM	NILM	Negative (NILM)	Candida
P10192	2023	2023-Q1	2023-03	Post-pandemic recovery	Cytology (Pap)	35-39	35	Not performed	Satisfactory	NILM	NILM	Negative (NILM)	
P10193	2023	2023-Q1	2023-03	Post-pandemic recovery	Cytology (Pap)	35-39	37	Not performed	Satisfactory	NILM	NILM	Negative (NILM)	
P10194	2023	2023-Q1	2023-03	Post-pandemic recovery	Cytology (Pap)	45-49	47	Not performed	Satisfactory	NILM	NILM	Negative (NILM)	
P10195	2023	2023-Q1	2023-03	Post-pandemic recovery	Cytology (Pap)	35-39	35	Not performed	Satisfactory	Positive	ASC-US	Low-grade / equivocal squamous	
P10196	2023	2023-Q1	2023-03	Post-pandemic recovery	Cytology (Pap)	55-59	57	Not performed	Satisfactory	NILM	NILM	Negative (NILM)	
P10197	2023	2023-Q1	2023-03	Post-pandemic recovery	Cytology (Pap)	40-44	44	Not performed	Satisfactory	NILM	NILM	Negative (NILM)	
P10198	2023	2023-Q1	2023-03	Post-pandemic recovery	Cytology (Pap)	45-49	49	Not performed	Satisfactory	NILM	NILM	Negative (NILM)	Trichomonas
P10199	2023	2023-Q1	2023-03	Post-pandemic recovery	Cytology (Pap)	30-34	34	Not performed	Satisfactory	NILM	NILM	Negative (NILM)	
P10200	2023	2023-Q1	2023-03	Post-pandemic recovery	Cytology (Pap)	40-44	42	Not performed	Satisfactory	NILM	NILM	Negative (NILM)	
P10201	2023	2023-Q1	2023-03	Post-pandemic recovery	Cytology (Pap)	50-54	53	Not performed	Satisfactory	Positive	LSIL	Low-grade / equivocal squamous	
P10202	2023	2023-Q1	2023-03	Post-pandemic recovery	Cytology (Pap)	45-49	47	Not performed	Satisfactory	Positive	ASC-US	Low-grade / equivocal squamous	
P10203	2023	2023-Q1	2023-03	Post-pandemic recovery	Cytology (Pap)	35-39	39	Not performed	Satisfactory	NILM	NILM	Negative (NILM)	
P10204	2023	2023-Q1	2023-03	Post-pandemic recovery	Cytology (Pap)	60-64	64	Not performed	Satisfactory	Positive	LSIL	Low-grade / equivocal squamous	Trichomonas
P10205	2023	2023-Q1	2023-03	Post-pandemic recovery	Cytology (Pap)	45-49	45	Not performed	Satisfactory	NILM	NILM	Negative (NILM)	
P10206	2023	2023-Q1	2023-03	Post-pandemic recovery	Cytology (Pap)	25-29	26	Not performed	Satisfactory	NILM	NILM	Negative (NILM)	
P10207	2023	2023-Q1	2023-03	Post-pandemic recovery	Cytology (Pap)	50-54	51	Not performed	Satisfactory	NILM	NILM	Negative (NILM)	
P10208	2023	2023-Q1	2023-03	Post-pandemic recovery	Cytology (Pap)	25-29	28	Not performed	Satisfactory	Positive	LSIL	Low-grade / equivocal squamous	
P10209	2023	2023-Q1	2023-03	Post-pandemic recovery	Cytology (Pap)	45-49	46	Not performed	Satisfactory	NILM	NILM	Negative (NILM)	
P10210	2023	2023-Q1	2023-03	Post-pandemic recovery	Cytology (Pap)	50-54	53	Not performed	Satisfactory	NILM	NILM	Negative (NILM)	
P10211	2023	2023-Q1	2023-03	Post-pandemic recovery	Cytology (Pap)	25-29	28	Not performed	Satisfactory	NILM	NILM	Negative (NILM)	
P10212	2023	2023-Q1	2023-03	Post-pandemic recovery	Cytology (Pap)	35-39	35	Not performed	Satisfactory	NILM	NILM	Negative (NILM)	
P10213	2023	2023-Q1	2023-03	Post-pandemic recovery	Cytology (Pap)	45-49	46	Not performed	Satisfactory	NILM	NILM	Negative (NILM)	
P10214	2023	2023-Q1	2023-03	Post-pandemic recovery	Cytology (Pap)	45-49	45	Not performed	Satisfactory	NILM	NILM	Negative (NILM)	
P10215	2023	2023-Q1	2023-03	Post-pandemic recovery	Cytology (Pap)	55-59	58	Not performed	Satisfactory	NILM	NILM	Negative (NILM)	
P10216	2023	2023-Q1	2023-03	Post-pandemic recovery	Cytology (Pap)	30-34	31	Not performed	Satisfactory	NILM	NILM	Negative (NILM)	
P10217	2023	2023-Q1	2023-03	Post-pandemic recovery	Cytology (Pap)	60-64	62	Not performed	Satisfactory	NILM	NILM	Negative (NILM)	
P10218	2023	2023-Q1	2023-03	Post-pandemic recovery	Cytology (Pap)	60-64	60	Not performed	Satisfactory	NILM	NILM	Negative (NILM)	
P10219	2023	2023-Q1	2023-03	Post-pandemic recovery	Cytology (Pap)	30-34	33	Not performed	Satisfactory	NILM	NILM	Negative (NILM)	
P10220	2023	2023-Q1	2023-03	Post-pandemic recovery	Cytology (Pap)	35-39	36	Not performed	Satisfactory	Positive	LSIL	Low-grade / equivocal squamous	Candida
P10221	2023	2023-Q1	2023-03	Post-pandemic recovery	Cytology (Pap)	40-44	43	Not performed	Satisfactory	NILM	NILM	Negative (NILM)	
P10222	2023	2023-Q1	2023-03	Post-pandemic recovery	Cytology (Pap)	40-44	42	Not performed	Satisfactory	NILM	NILM	Negative (NILM)	
P10223	2023	2023-Q1	2023-03	Post-pandemic recovery	Cytology (Pap)	45-49	47	Not performed	Satisfactory	NILM	NILM	Negative (NILM)	Gardnerella
P10224	2023	2023-Q1	2023-03	Post-pandemic recovery	Cytology (Pap)	45-49	46	Not performed	Satisfactory	NILM	NILM	Negative (NILM)	
P10225	2023	2023-Q1	2023-03	Post-pandemic recovery	Cytology (Pap)	55-59	57	Not performed	Satisfactory	NILM	NILM	Negative (NILM)	
P10226	2023	2023-Q1	2023-03	Post-pandemic recovery	Cytology (Pap)	25-29	27	Not performed	Satisfactory	NILM	NILM	Negative (NILM)	
P10227	2023	2023-Q1	2023-03	Post-pandemic recovery	Cytology (Pap)	55-59	59	Not performed	Satisfactory	Positive	HSIL	High-grade squamous	
P10228	2023	2023-Q1	2023-03	Post-pandemic recovery	Cytology (Pap)	45-49	46	Not performed	Satisfactory	NILM	NILM	Negative (NILM)	
P10229	2023	2023-Q1	2023-03	Post-pandemic recovery	Cytology (Pap)	40-44	43	Not performed	Satisfactory	NILM	NILM	Negative (NILM)	
P10230	2023	2023-Q1	2023-03	Post-pandemic recovery	Cytology (Pap)	30-34	30	Not performed	Satisfactory	NILM	NILM	Negative (NILM)	
P10231	2023	2023-Q1	2023-03	Post-pandemic recovery	Cytology (Pap)	45-49	47	Not performed	Satisfactory	NILM	NILM	Negative (NILM)	
P10232	2023	2023-Q1	2023-03	Post-pandemic recovery	Cytology (Pap)	45-49	47	Not performed	Satisfactory	NILM	NILM	Negative (NILM)	
P10233	2023	2023-Q1	2023-03	Post-pandemic recovery	Cytology (Pap)	50-54	52	Not performed	Satisfactory	Positive	LSIL	Low-grade / equivocal squamous	
P10234	2023	2023-Q1	2023-03	Post-pandemic recovery	Cytology (Pap)	45-49	46	Not performed	Satisfactory	NILM	NILM	Negative (NILM)	
P10235	2023	2023-Q1	2023-03	Post-pandemic recovery	Cytology (Pap)	35-39	36	Not performed	Satisfactory	Positive	ASC-H	High-grade squamous	
P10236	2023	2023-Q1	2023-03	Post-pandemic recovery	Cytology (Pap)	40-44	41	Not performed	Unsatisfactory	Unsatisfactory	Unsatisfactory	Unsatisfactory	
P10237	2023	2023-Q1	2023-03	Post-pandemic recovery	Cytology (Pap)	45-49	48	Not performed	Satisfactory	NILM	NILM	Negative (NILM)	
P10238	2023	2023-Q1	2023-03	Post-pandemic recovery	Cytology (Pap)	25-29	26	Not performed	Satisfactory	NILM	NILM	Negative (NILM)	Candida

patient_id	year	quarter	month	period	primary_test	age_group	age_years	hpv_result	adequacy	result	bethesda	grade	infection
P10239	2023	2023-Q1	2023-03	Post-pandemic recovery	Cytology (Pap)	50-54	51	Not performed	Satisfactory	NILM	NILM	Negative (NILM)	
P10240	2023	2023-Q1	2023-03	Post-pandemic recovery	Cytology (Pap)	50-54	54	Not performed	Satisfactory	NILM	NILM	Negative (NILM)	
P10241	2023	2023-Q1	2023-03	Post-pandemic recovery	Cytology (Pap)	35-39	37	Not performed	Satisfactory	NILM	NILM	Negative (NILM)	
P10242	2023	2023-Q1	2023-03	Post-pandemic recovery	Cytology (Pap)	55-59	57	Not performed	Satisfactory	NILM	NILM	Negative (NILM)	
P10243	2023	2023-Q1	2023-03	Post-pandemic recovery	Cytology (Pap)	25-29	25	Not performed	Satisfactory	NILM	NILM	Negative (NILM)	
P10244	2023	2023-Q1	2023-03	Post-pandemic recovery	Cytology (Pap)	35-39	35	Not performed	Satisfactory	Positive	LSIL	Low-grade / equivocal squamous	
P10245	2023	2023-Q1	2023-03	Post-pandemic recovery	Cytology (Pap)	30-34	30	Not performed	Satisfactory	NILM	NILM	Negative (NILM)	
P10246	2023	2023-Q1	2023-03	Post-pandemic recovery	Cytology (Pap)	25-29	29	Not performed	Satisfactory	NILM	NILM	Negative (NILM)	
P10247	2023	2023-Q1	2023-03	Post-pandemic recovery	Cytology (Pap)	45-49	47	Not performed	Satisfactory	NILM	NILM	Negative (NILM)	
P10248	2023	2023-Q1	2023-03	Post-pandemic recovery	Cytology (Pap)	55-59	58	Not performed	Satisfactory	NILM	NILM	Negative (NILM)	
P10249	2023	2023-Q1	2023-03	Post-pandemic recovery	Cytology (Pap)	45-49	49	Not performed	Satisfactory	NILM	NILM	Negative (NILM)	
P10250	2023	2023-Q1	2023-03	Post-pandemic recovery	Cytology (Pap)	35-39	38	Not performed	Satisfactory	NILM	NILM	Negative (NILM)	
P10251	2023	2023-Q1	2023-03	Post-pandemic recovery	Cytology (Pap)	60-64	62	Not performed	Satisfactory	NILM	NILM	Negative (NILM)	
P10252	2023	2023-Q1	2023-03	Post-pandemic recovery	Cytology (Pap)	55-59	58	Not performed	Satisfactory	NILM	NILM	Negative (NILM)	
P10253	2023	2023-Q1	2023-03	Post-pandemic recovery	Cytology (Pap)	50-54	51	Not performed	Unsatisfactory	Unsatisfactory	Unsatisfactory	Unsatisfactory	
P10254	2023	2023-Q1	2023-03	Post-pandemic recovery	Cytology (Pap)	30-34	31	Not performed	Satisfactory	NILM	NILM	Negative (NILM)	
P10255	2023	2023-Q1	2023-03	Post-pandemic recovery	Cytology (Pap)	35-39	38	Not performed	Satisfactory	NILM	NILM	Negative (NILM)	
P10256	2023	2023-Q1	2023-03	Post-pandemic recovery	Cytology (Pap)	40-44	44	Not performed	Satisfactory	NILM	NILM	Negative (NILM)	
P10257	2023	2023-Q1	2023-03	Post-pandemic recovery	Cytology (Pap)	25-29	27	Not performed	Satisfactory	NILM	NILM	Negative (NILM)	
P10258	2023	2023-Q1	2023-03	Post-pandemic recovery	Cytology (Pap)	30-34	34	Not performed	Satisfactory	NILM	NILM	Negative (NILM)	
P10259	2023	2023-Q1	2023-03	Post-pandemic recovery	Cytology (Pap)	35-39	36	Not performed	Satisfactory	NILM	NILM	Negative (NILM)	
P10260	2023	2023-Q1	2023-03	Post-pandemic recovery	Cytology (Pap)	35-39	36	Not performed	Unsatisfactory	Unsatisfactory	Unsatisfactory	Unsatisfactory	
P10261	2023	2023-Q1	2023-03	Post-pandemic recovery	Cytology (Pap)	45-49	49	Not performed	Satisfactory	NILM	NILM	Negative (NILM)	
P10262	2023	2023-Q1	2023-03	Post-pandemic recovery	Cytology (Pap)	35-39	37	Not performed	Satisfactory	NILM	NILM	Negative (NILM)	
P10263	2023	2023-Q1	2023-03	Post-pandemic recovery	Cytology (Pap)	50-54	53	Not performed	Satisfactory	NILM	NILM	Negative (NILM)	
P10264	2023	2023-Q1	2023-03	Post-pandemic recovery	Cytology (Pap)	50-54	52	Not performed	Satisfactory	NILM	NILM	Negative (NILM)	
P10265	2023	2023-Q1	2023-03	Post-pandemic recovery	Cytology (Pap)	50-54	52	Not performed	Satisfactory	NILM	NILM	Negative (NILM)	
P10266	2023	2023-Q1	2023-03	Post-pandemic recovery	Cytology (Pap)	60-64	64	Not performed	Satisfactory	Positive	ASC-US	Low-grade / equivocal squamous	
P10267	2023	2023-Q1	2023-03	Post-pandemic recovery	Cytology (Pap)	35-39	36	Not performed	Satisfactory	Positive	LSIL	Low-grade / equivocal squamous	
P10268	2023	2023-Q1	2023-03	Post-pandemic recovery	Cytology (Pap)	40-44	43	Not performed	Satisfactory	NILM	NILM	Negative (NILM)	
P10269	2023	2023-Q1	2023-03	Post-pandemic recovery	Cytology (Pap)	35-39	38	Not performed	Satisfactory	Positive	HSIL	High-grade squamous	
P10270	2023	2023-Q1	2023-03	Post-pandemic recovery	Cytology (Pap)	30-34	33	Not performed	Satisfactory	NILM	NILM	Negative (NILM)	
P10271	2023	2023-Q1	2023-03	Post-pandemic recovery	Cytology (Pap)	40-44	41	Not performed	Satisfactory	NILM	NILM	Negative (NILM)	
P10272	2023	2023-Q1	2023-03	Post-pandemic recovery	Cytology (Pap)	40-44	40	Not performed	Satisfactory	NILM	NILM	Negative (NILM)	Candida
P10273	2023	2023-Q1	2023-03	Post-pandemic recovery	Cytology (Pap)	35-39	35	Not performed	Satisfactory	NILM	NILM	Negative (NILM)	
P10274	2023	2023-Q1	2023-03	Post-pandemic recovery	Cytology (Pap)	25-29	25	Not performed	Satisfactory	NILM	NILM	Negative (NILM)	
P10275	2023	2023-Q1	2023-03	Post-pandemic recovery	Cytology (Pap)	50-54	53	Not performed	Satisfactory	Positive	ASC-US	Low-grade / equivocal squamous	
P10276	2023	2023-Q1	2023-03	Post-pandemic recovery	Cytology (Pap)	25-29	29	Not performed	Satisfactory	NILM	NILM	Negative (NILM)	
P10277	2023	2023-Q1	2023-03	Post-pandemic recovery	Cytology (Pap)	45-49	47	Not performed	Satisfactory	NILM	NILM	Negative (NILM)	
P10278	2023	2023-Q1	2023-03	Post-pandemic recovery	Cytology (Pap)	40-44	43	Not performed	Satisfactory	NILM	NILM	Negative (NILM)	
P10279	2023	2023-Q1	2023-03	Post-pandemic recovery	Cytology (Pap)	40-44	41	Not performed	Satisfactory	NILM	NILM	Negative (NILM)	
P10280	2023	2023-Q1	2023-03	Post-pandemic recovery	Cytology (Pap)	50-54	53	Not performed	Satisfactory	NILM	NILM	Negative (NILM)	
P10281	2023	2023-Q1	2023-03	Post-pandemic recovery	Cytology (Pap)	35-39	36	Not performed	Satisfactory	NILM	NILM	Negative (NILM)	
P10282	2023	2023-Q1	2023-03	Post-pandemic recovery	Cytology (Pap)	45-49	48	Not performed	Satisfactory	NILM	NILM	Negative (NILM)	Trichomonas
P10283	2023	2023-Q1	2023-03	Post-pandemic recovery	Cytology (Pap)	40-44	41	Not performed	Satisfactory	NILM	NILM	Negative (NILM)	
P10284	2023	2023-Q1	2023-03	Post-pandemic recovery	Cytology (Pap)	40-44	42	Not performed	Satisfactory	Positive	HSIL	High-grade squamous	
P10285	2023	2023-Q1	2023-03	Post-pandemic recovery	Cytology (Pap)	55-59	56	Not performed	Satisfactory	Positive	ASC-US	Low-grade / equivocal squamous	Gardnerella
P10286	2023	2023-Q1	2023-03	Post-pandemic recovery	Cytology (Pap)	60-64	62	Not performed	Satisfactory	NILM	NILM	Negative (NILM)	
P10287	2023	2023-Q1	2023-03	Post-pandemic recovery	Cytology (Pap)	25-29	29	Not performed	Satisfactory	NILM	NILM	Negative (NILM)	
P10288	2023	2023-Q1	2023-03	Post-pandemic recovery	Cytology (Pap)	45-49	49	Not performed	Satisfactory	NILM	NILM	Negative (NILM)	
P10289	2023	2023-Q1	2023-03	Post-pandemic recovery	Cytology (Pap)	60-64	60	Not performed	Satisfactory	NILM	NILM	Negative (NILM)	
P10290	2023	2023-Q1	2023-03	Post-pandemic recovery	Cytology (Pap)	40-44	41	Not performed	Satisfactory	Positive	AGC	Glandular	
P10291	2023	2023-Q1	2023-03	Post-pandemic recovery	Cytology (Pap)	35-39	35	Not performed	Satisfactory	NILM	NILM	Negative (NILM)	
P10292	2023	2023-Q1	2023-03	Post-pandemic recovery	Cytology (Pap)	35-39	39	Not performed	Satisfactory	Positive	HSIL	High-grade squamous	
P10293	2023	2023-Q1	2023-03	Post-pandemic recovery	Cytology (Pap)	25-29	25	Not performed	Satisfactory	NILM	NILM	Negative (NILM)	
P10294	2023	2023-Q1	2023-03	Post-pandemic recovery	Cytology (Pap)	45-49	48	Not performed	Satisfactory	NILM	NILM	Negative (NILM)	Gardnerella
P10295	2023	2023-Q1	2023-03	Post-pandemic recovery	Cytology (Pap)	40-44	40	Not performed	Unsatisfactory	Unsatisfactory	Unsatisfactory	Unsatisfactory	
P10296	2023	2023-Q1	2023-03	Post-pandemic recovery	Cytology (Pap)	40-44	44	Not performed	Satisfactory	NILM	NILM	Negative (NILM)	
P10297	2023	2023-Q1	2023-03	Post-pandemic recovery	Cytology (Pap)	40-44	40	Not performed	Satisfactory	NILM	NILM	Negative (NILM)	
P10298	2023	2023-Q1	2023-03	Post-pandemic recovery	Cytology (Pap)	30-34	30	Not performed	Satisfactory	NILM	NILM	Negative (NILM)	
P10299	2023	2023-Q1	2023-03	Post-pandemic recovery	Cytology (Pap)	45-49	49	Not performed	Satisfactory	NILM	NILM	Negative (NILM)	Candida
P10300	2023	2023-Q1	2023-03	Post-pandemic recovery	Cytology (Pap)	45-49	46	Not performed	Satisfactory	NILM	NILM	Negative (NILM)	
P10301	2023	2023-Q1	2023-03	Post-pandemic recovery	Cytology (Pap)	45-49	46	Not performed	Satisfactory	NILM	NILM	Negative (NILM)	
P10302	2023	2023-Q1	2023-03	Post-pandemic recovery	Cytology (Pap)	35-39	38	Not performed	Satisfactory	Positive	ASC-US	Low-grade / equivocal squamous	
P10303	2023	2023-Q1	2023-03	Post-pandemic recovery	Cytology (Pap)	55-59	57	Not performed	Satisfactory	NILM	NILM	Negative (NILM)	
P10304	2023	2023-Q1	2023-03	Post-pandemic recovery	Cytology (Pap)	40-44	42	Not performed	Unsatisfactory	Unsatisfactory	Unsatisfactory	Unsatisfactory	
P10305	2023	2023-Q1	2023-03	Post-pandemic recovery	Cytology (Pap)	30-34	30	Not performed	Satisfactory	Positive	ASC-US	Low-grade / equivocal squamous	Candida
P10306	2023	2023-Q1	2023-03	Post-pandemic recovery	Cytology (Pap)	35-39	36	Not performed	Satisfactory	NILM	NILM	Negative (NILM)	
P10307	2023	2023-Q1	2023-03	Post-pandemic recovery	Cytology (Pap)	55-59	55	Not performed	Satisfactory	NILM	NILM	Negative (NILM)	
P10308	2023	2023-Q1	2023-03	Post-pandemic recovery	Cytology (Pap)	30-34	32	Not performed	Satisfactory	NILM	NILM	Negative (NILM)	
P10309	2023	2023-Q1	2023-03	Post-pandemic recovery	Cytology (Pap)	25-29	28	Not performed	Satisfactory	NILM	NILM	Negative (NILM)	
P10310	2023	2023-Q1	2023-03	Post-pandemic recovery	Cytology (Pap)	25-29	29	Not performed	Satisfactory	NILM	NILM	Negative (NILM)	
P10311	2023	2023-Q1	2023-03	Post-pandemic recovery	Cytology (Pap)	30-34	30	Not performed	Unsatisfactory	Unsatisfactory	Unsatisfactory	Unsatisfactory	Trichomonas
P10312	2023	2023-Q1	2023-03	Post-pandemic recovery	Cytology (Pap)	40-44	43	Not performed	Satisfactory	NILM	NILM	Negative (NILM)	
P10313	2023	2023-Q1	2023-03	Post-pandemic recovery	Cytology (Pap)	35-39	35	Not performed	Satisfactory	NILM	NILM	Negative (NILM)	
P10314	2023	2023-Q1	2023-03	Post-pandemic recovery	Cytology (Pap)	25-29	28	Not performed	Satisfactory	NILM	NILM	Negative (NILM)	
P10315	2023	2023-Q1	2023-03	Post-pandemic recovery	Cytology (Pap)	30-34	30	Not performed	Satisfactory	NILM	NILM	Negative (NILM)	
P10316	2023	2023-Q1	2023-03	Post-pandemic recovery	Cytology (Pap)	55-59	59	Not performed	Satisfactory	NILM	NILM	Negative (NILM)	
P10317	2023	2023-Q1	2023-03	Post-pandemic recovery	Cytology (Pap)	60-64	62	Not performed	Satisfactory	NILM	NILM	Negative (NILM)	
P10318	2023	2023-Q1	2023-03	Post-pandemic recovery	Cytology (Pap)	40-44	42	Not performed	Satisfactory	NILM	NILM	Negative (NILM)	

patient_id	year	quarter	month	period	primary_test	age_group	age_years	hpv_result	adequacy	result	bethesda	grade	infection
P10319	2023	2023-Q1	2023-03	Post-pandemic recovery	Cytology (Pap)	30-34	33	Not performed	Satisfactory	NILM	NILM	Negative (NILM)	
P10320	2023	2023-Q1	2023-03	Post-pandemic recovery	Cytology (Pap)	55-59	57	Not performed	Satisfactory	Positive	HSIL	High-grade squamous	
P10321	2023	2023-Q1	2023-03	Post-pandemic recovery	Cytology (Pap)	35-39	35	Not performed	Satisfactory	NILM	NILM	Negative (NILM)	
P10322	2023	2023-Q1	2023-03	Post-pandemic recovery	Cytology (Pap)	35-39	37	Not performed	Satisfactory	NILM	NILM	Negative (NILM)	
P10323	2023	2023-Q1	2023-03	Post-pandemic recovery	Cytology (Pap)	30-34	30	Not performed	Satisfactory	NILM	NILM	Negative (NILM)	
P10324	2023	2023-Q1	2023-03	Post-pandemic recovery	Cytology (Pap)	35-39	38	Not performed	Satisfactory	NILM	NILM	Negative (NILM)	
P10325	2023	2023-Q1	2023-03	Post-pandemic recovery	Cytology (Pap)	35-39	39	Not performed	Satisfactory	NILM	NILM	Negative (NILM)	
P10326	2023	2023-Q1	2023-03	Post-pandemic recovery	Cytology (Pap)	25-29	28	Not performed	Satisfactory	NILM	NILM	Negative (NILM)	Candida
P10327	2023	2023-Q1	2023-03	Post-pandemic recovery	Cytology (Pap)	45-49	47	Not performed	Satisfactory	NILM	NILM	Negative (NILM)	
P10328	2023	2023-Q1	2023-03	Post-pandemic recovery	Cytology (Pap)	40-44	42	Not performed	Satisfactory	Positive	ASC-US	Low-grade / equivocal squamous	
P10329	2023	2023-Q1	2023-03	Post-pandemic recovery	Cytology (Pap)	40-44	40	Not performed	Satisfactory	Positive	ASC-H	High-grade squamous	
P10330	2023	2023-Q1	2023-03	Post-pandemic recovery	Cytology (Pap)	30-34	32	Not performed	Satisfactory	NILM	NILM	Negative (NILM)	
P10331	2023	2023-Q1	2023-03	Post-pandemic recovery	Cytology (Pap)	60-64	61	Not performed	Satisfactory	NILM	NILM	Negative (NILM)	
P10332	2023	2023-Q1	2023-03	Post-pandemic recovery	Cytology (Pap)	45-49	49	Not performed	Satisfactory	NILM	NILM	Negative (NILM)	
P10333	2023	2023-Q1	2023-03	Post-pandemic recovery	Cytology (Pap)	45-49	49	Not performed	Satisfactory	NILM	NILM	Negative (NILM)	
P10334	2023	2023-Q1	2023-03	Post-pandemic recovery	Cytology (Pap)	30-34	31	Not performed	Satisfactory	NILM	NILM	Negative (NILM)	
P10335	2023	2023-Q1	2023-03	Post-pandemic recovery	Cytology (Pap)	40-44	43	Not performed	Satisfactory	NILM	NILM	Negative (NILM)	
P10336	2023	2023-Q1	2023-03	Post-pandemic recovery	Cytology (Pap)	40-44	43	Not performed	Satisfactory	NILM	NILM	Negative (NILM)	
P10337	2023	2023-Q1	2023-03	Post-pandemic recovery	Cytology (Pap)	30-34	30	Not performed	Satisfactory	NILM	NILM	Negative (NILM)	
P10338	2023	2023-Q2	2023-04	Post-pandemic recovery	Cytology (Pap)	30-34	31	Not performed	Satisfactory	NILM	NILM	Negative (NILM)	
P10339	2023	2023-Q2	2023-04	Post-pandemic recovery	Cytology (Pap)	35-39	38	Not performed	Satisfactory	NILM	NILM	Negative (NILM)	
P10340	2023	2023-Q2	2023-04	Post-pandemic recovery	Cytology (Pap)	30-34	33	Not performed	Satisfactory	NILM	NILM	Negative (NILM)	
P10341	2023	2023-Q2	2023-04	Post-pandemic recovery	Cytology (Pap)	30-34	30	Not performed	Satisfactory	NILM	NILM	Negative (NILM)	
P10342	2023	2023-Q2	2023-04	Post-pandemic recovery	Cytology (Pap)	35-39	39	Not performed	Unsatisfactory	Unsatisfactory	Unsatisfactory	Unsatisfactory	
P10343	2023	2023-Q2	2023-04	Post-pandemic recovery	Cytology (Pap)	40-44	41	Not performed	Satisfactory	NILM	NILM	Negative (NILM)	
P10344	2023	2023-Q2	2023-04	Post-pandemic recovery	Cytology (Pap)	50-54	54	Not performed	Satisfactory	NILM	NILM	Negative (NILM)	Gardnerella
P10345	2023	2023-Q2	2023-04	Post-pandemic recovery	Cytology (Pap)	55-59	58	Not performed	Satisfactory	NILM	NILM	Negative (NILM)	
P10346	2023	2023-Q2	2023-04	Post-pandemic recovery	Cytology (Pap)	50-54	53	Not performed	Satisfactory	NILM	NILM	Negative (NILM)	
P10347	2023	2023-Q2	2023-04	Post-pandemic recovery	Cytology (Pap)	35-39	38	Not performed	Satisfactory	NILM	NILM	Negative (NILM)	Gardnerella
P10348	2023	2023-Q2	2023-04	Post-pandemic recovery	Cytology (Pap)	45-49	49	Not performed	Satisfactory	NILM	NILM	Negative (NILM)	
P10349	2023	2023-Q2	2023-04	Post-pandemic recovery	Cytology (Pap)	60-64	63	Not performed	Satisfactory	NILM	NILM	Negative (NILM)	
P10350	2023	2023-Q2	2023-04	Post-pandemic recovery	Cytology (Pap)	35-39	38	Not performed	Satisfactory	NILM	NILM	Negative (NILM)	
P10351	2023	2023-Q2	2023-04	Post-pandemic recovery	Cytology (Pap)	25-29	25	Not performed	Satisfactory	NILM	NILM	Negative (NILM)	
P10352	2023	2023-Q2	2023-04	Post-pandemic recovery	Cytology (Pap)	30-34	34	Not performed	Satisfactory	NILM	NILM	Negative (NILM)	
P10353	2023	2023-Q2	2023-04	Post-pandemic recovery	Cytology (Pap)	55-59	56	Not performed	Satisfactory	Positive	ASC-US	Low-grade / equivocal squamous	
P10354	2023	2023-Q2	2023-04	Post-pandemic recovery	Cytology (Pap)	40-44	42	Not performed	Satisfactory	Positive	ASC-US	Low-grade / equivocal squamous	
P10355	2023	2023-Q2	2023-04	Post-pandemic recovery	Cytology (Pap)	55-59	59	Not performed	Satisfactory	NILM	NILM	Negative (NILM)	Candida
P10356	2023	2023-Q2	2023-04	Post-pandemic recovery	Cytology (Pap)	35-39	37	Not performed	Satisfactory	NILM	NILM	Negative (NILM)	
P10357	2023	2023-Q2	2023-04	Post-pandemic recovery	Cytology (Pap)	55-59	55	Not performed	Satisfactory	NILM	NILM	Negative (NILM)	
P10358	2023	2023-Q2	2023-04	Post-pandemic recovery	Cytology (Pap)	30-34	30	Not performed	Satisfactory	NILM	NILM	Negative (NILM)	
P10359	2023	2023-Q2	2023-04	Post-pandemic recovery	Cytology (Pap)	55-59	59	Not performed	Satisfactory	NILM	NILM	Negative (NILM)	
P10360	2023	2023-Q2	2023-04	Post-pandemic recovery	Cytology (Pap)	45-49	48	Not performed	Satisfactory	Positive	ASC-US	Low-grade / equivocal squamous	
P10361	2023	2023-Q2	2023-04	Post-pandemic recovery	Cytology (Pap)	35-39	36	Not performed	Satisfactory	NILM	NILM	Negative (NILM)	
P10362	2023	2023-Q2	2023-04	Post-pandemic recovery	Cytology (Pap)	30-34	34	Not performed	Satisfactory	NILM	NILM	Negative (NILM)	
P10363	2023	2023-Q2	2023-04	Post-pandemic recovery	Cytology (Pap)	30-34	31	Not performed	Satisfactory	NILM	NILM	Negative (NILM)	
P10364	2023	2023-Q2	2023-04	Post-pandemic recovery	Cytology (Pap)	40-44	44	Not performed	Satisfactory	NILM	NILM	Negative (NILM)	
P10365	2023	2023-Q2	2023-04	Post-pandemic recovery	Cytology (Pap)	40-44	43	Not performed	Satisfactory	NILM	NILM	Negative (NILM)	
P10366	2023	2023-Q2	2023-04	Post-pandemic recovery	Cytology (Pap)	40-44	41	Not performed	Satisfactory	NILM	NILM	Negative (NILM)	
P10367	2023	2023-Q2	2023-04	Post-pandemic recovery	Cytology (Pap)	45-49	45	Not performed	Satisfactory	NILM	NILM	Negative (NILM)	
P10368	2023	2023-Q2	2023-04	Post-pandemic recovery	Cytology (Pap)	35-39	36	Not performed	Satisfactory	NILM	NILM	Negative (NILM)	
P10369	2023	2023-Q2	2023-04	Post-pandemic recovery	Cytology (Pap)	40-44	42	Not performed	Satisfactory	NILM	NILM	Negative (NILM)	
P10370	2023	2023-Q2	2023-04	Post-pandemic recovery	Cytology (Pap)	45-49	47	Not performed	Satisfactory	Positive	LSIL	Low-grade / equivocal squamous	Candida
P10371	2023	2023-Q2	2023-04	Post-pandemic recovery	Cytology (Pap)	40-44	42	Not performed	Satisfactory	NILM	NILM	Negative (NILM)	
P10372	2023	2023-Q2	2023-04	Post-pandemic recovery	Cytology (Pap)	50-54	51	Not performed	Satisfactory	Positive	ASC-US	Low-grade / equivocal squamous	
P10373	2023	2023-Q2	2023-04	Post-pandemic recovery	Cytology (Pap)	40-44	43	Not performed	Satisfactory	NILM	NILM	Negative (NILM)	
P10374	2023	2023-Q2	2023-04	Post-pandemic recovery	Cytology (Pap)	35-39	35	Not performed	Satisfactory	NILM	NILM	Negative (NILM)	
P10375	2023	2023-Q2	2023-04	Post-pandemic recovery	Cytology (Pap)	60-64	62	Not performed	Satisfactory	NILM	NILM	Negative (NILM)	
P10376	2023	2023-Q2	2023-04	Post-pandemic recovery	Cytology (Pap)	50-54	53	Not performed	Satisfactory	NILM	NILM	Negative (NILM)	
P10377	2023	2023-Q2	2023-04	Post-pandemic recovery	Cytology (Pap)	30-34	33	Not performed	Satisfactory	NILM	NILM	Negative (NILM)	
P10378	2023	2023-Q2	2023-04	Post-pandemic recovery	Cytology (Pap)	50-54	54	Not performed	Unsatisfactory	Unsatisfactory	Unsatisfactory	Unsatisfactory	
P10379	2023	2023-Q2	2023-04	Post-pandemic recovery	Cytology (Pap)	35-39	35	Not performed	Satisfactory	NILM	NILM	Negative (NILM)	
P10380	2023	2023-Q2	2023-04	Post-pandemic recovery	Cytology (Pap)	25-29	28	Not performed	Satisfactory	NILM	NILM	Negative (NILM)	
P10381	2023	2023-Q2	2023-04	Post-pandemic recovery	Cytology (Pap)	35-39	38	Not performed	Unsatisfactory	Unsatisfactory	Unsatisfactory	Unsatisfactory	
P10382	2023	2023-Q2	2023-04	Post-pandemic recovery	Cytology (Pap)	55-59	58	Not performed	Satisfactory	NILM	NILM	Negative (NILM)	
P10383	2023	2023-Q2	2023-04	Post-pandemic recovery	Cytology (Pap)	60-64	64	Not performed	Satisfactory	NILM	NILM	Negative (NILM)	
P10384	2023	2023-Q2	2023-04	Post-pandemic recovery	Cytology (Pap)	60-64	64	Not performed	Satisfactory	NILM	NILM	Negative (NILM)	
P10385	2023	2023-Q2	2023-04	Post-pandemic recovery	Cytology (Pap)	45-49	47	Not performed	Satisfactory	NILM	NILM	Negative (NILM)	
P10386	2023	2023-Q2	2023-04	Post-pandemic recovery	Cytology (Pap)	25-29	25	Not performed	Satisfactory	NILM	NILM	Negative (NILM)	
P10387	2023	2023-Q2	2023-04	Post-pandemic recovery	Cytology (Pap)	30-34	33	Not performed	Satisfactory	NILM	NILM	Negative (NILM)	
P10388	2023	2023-Q2	2023-04	Post-pandemic recovery	Cytology (Pap)	40-44	44	Not performed	Satisfactory	NILM	NILM	Negative (NILM)	
P10389	2023	2023-Q2	2023-04	Post-pandemic recovery	Cytology (Pap)	35-39	39	Not performed	Satisfactory	NILM	NILM	Negative (NILM)	
P10390	2023	2023-Q2	2023-04	Post-pandemic recovery	Cytology (Pap)	60-64	61	Not performed	Satisfactory	NILM	NILM	Negative (NILM)	
P10391	2023	2023-Q2	2023-04	Post-pandemic recovery	Cytology (Pap)	40-44	42	Not performed	Satisfactory	NILM	NILM	Negative (NILM)	
P10392	2023	2023-Q2	2023-04	Post-pandemic recovery	Cytology (Pap)	55-59	59	Not performed	Satisfactory	NILM	NILM	Negative (NILM)	
P10393	2023	2023-Q2	2023-04	Post-pandemic recovery	Cytology (Pap)	40-44	43	Not performed	Unsatisfactory	Unsatisfactory	Unsatisfactory	Unsatisfactory	Candida
P10394	2023	2023-Q2	2023-04	Post-pandemic recovery	Cytology (Pap)	40-44	41	Not performed	Satisfactory	NILM	NILM	Negative (NILM)	
P10395	2023	2023-Q2	2023-04	Post-pandemic recovery	Cytology (Pap)	40-44	42	Not performed	Satisfactory	NILM	NILM	Negative (NILM)	
P10396	2023	2023-Q2	2023-04	Post-pandemic recovery	Cytology (Pap)	30-34	30	Not performed	Satisfactory	NILM	NILM	Negative (NILM)	
P10397	2023	2023-Q2	2023-04	Post-pandemic recovery	Cytology (Pap)	40-44	44	Not performed	Satisfactory	NILM	NILM	Negative (NILM)	
P10398	2023	2023-Q2	2023-04	Post-pandemic recovery	Cytology (Pap)	50-54	50	Not performed	Satisfactory	NILM	NILM	Negative (NILM)	

patient_id	year	quarter	month	period	primary_test	age_group	age_years	hpv_result	adequacy	result	bethesda	grade	infection
P10399	2023	2023-Q2	2023-04	Post-pandemic recovery	Cytology (Pap)	35-39	37	Not performed	Satisfactory	Positive	AGC	Glandular	Trichomonas
P10400	2023	2023-Q2	2023-04	Post-pandemic recovery	Cytology (Pap)	50-54	50	Not performed	Satisfactory	NILM	NILM	Negative (NILM)	
P10401	2023	2023-Q2	2023-04	Post-pandemic recovery	Cytology (Pap)	55-59	56	Not performed	Satisfactory	NILM	NILM	Negative (NILM)	
P10402	2023	2023-Q2	2023-04	Post-pandemic recovery	Cytology (Pap)	45-49	49	Not performed	Satisfactory	NILM	NILM	Negative (NILM)	
P10403	2023	2023-Q2	2023-04	Post-pandemic recovery	Cytology (Pap)	55-59	59	Not performed	Satisfactory	NILM	NILM	Negative (NILM)	
P10404	2023	2023-Q2	2023-04	Post-pandemic recovery	Cytology (Pap)	30-34	30	Not performed	Satisfactory	NILM	NILM	Negative (NILM)	
P10405	2023	2023-Q2	2023-04	Post-pandemic recovery	Cytology (Pap)	30-34	33	Not performed	Satisfactory	NILM	NILM	Negative (NILM)	
P10406	2023	2023-Q2	2023-04	Post-pandemic recovery	Cytology (Pap)	45-49	45	Not performed	Satisfactory	Positive	HSIL	High-grade squamous	
P10407	2023	2023-Q2	2023-04	Post-pandemic recovery	Cytology (Pap)	35-39	37	Not performed	Satisfactory	NILM	NILM	Negative (NILM)	
P10408	2023	2023-Q2	2023-04	Post-pandemic recovery	Cytology (Pap)	30-34	31	Not performed	Satisfactory	NILM	NILM	Negative (NILM)	
P10409	2023	2023-Q2	2023-04	Post-pandemic recovery	Cytology (Pap)	45-49	47	Not performed	Satisfactory	NILM	NILM	Negative (NILM)	
P10410	2023	2023-Q2	2023-04	Post-pandemic recovery	Cytology (Pap)	35-39	35	Not performed	Satisfactory	NILM	NILM	Negative (NILM)	
P10411	2023	2023-Q2	2023-04	Post-pandemic recovery	Cytology (Pap)	45-49	49	Not performed	Satisfactory	NILM	NILM	Negative (NILM)	
P10412	2023	2023-Q2	2023-04	Post-pandemic recovery	Cytology (Pap)	55-59	56	Not performed	Satisfactory	Positive	AGC	Glandular	
P10413	2023	2023-Q2	2023-04	Post-pandemic recovery	Cytology (Pap)	55-59	59	Not performed	Satisfactory	NILM	NILM	Negative (NILM)	
P10414	2023	2023-Q2	2023-04	Post-pandemic recovery	Cytology (Pap)	50-54	52	Not performed	Satisfactory	NILM	NILM	Negative (NILM)	
P10415	2023	2023-Q2	2023-04	Post-pandemic recovery	Cytology (Pap)	40-44	44	Not performed	Satisfactory	NILM	NILM	Negative (NILM)	
P10416	2023	2023-Q2	2023-04	Post-pandemic recovery	Cytology (Pap)	25-29	27	Not performed	Satisfactory	NILM	NILM	Negative (NILM)	
P10417	2023	2023-Q2	2023-04	Post-pandemic recovery	Cytology (Pap)	50-54	51	Not performed	Satisfactory	NILM	NILM	Negative (NILM)	
P10418	2023	2023-Q2	2023-04	Post-pandemic recovery	Cytology (Pap)	50-54	53	Not performed	Satisfactory	NILM	NILM	Negative (NILM)	
P10419	2023	2023-Q2	2023-04	Post-pandemic recovery	Cytology (Pap)	55-59	58	Not performed	Satisfactory	NILM	NILM	Negative (NILM)	
P10420	2023	2023-Q2	2023-04	Post-pandemic recovery	Cytology (Pap)	45-49	46	Not performed	Satisfactory	NILM	NILM	Negative (NILM)	
P10421	2023	2023-Q2	2023-04	Post-pandemic recovery	Cytology (Pap)	45-49	45	Not performed	Satisfactory	NILM	NILM	Negative (NILM)	
P10422	2023	2023-Q2	2023-04	Post-pandemic recovery	Cytology (Pap)	40-44	42	Not performed	Satisfactory	NILM	NILM	Negative (NILM)	
P10423	2023	2023-Q2	2023-04	Post-pandemic recovery	Cytology (Pap)	25-29	29	Not performed	Satisfactory	NILM	NILM	Negative (NILM)	
P10424	2023	2023-Q2	2023-04	Post-pandemic recovery	Cytology (Pap)	35-39	37	Not performed	Satisfactory	NILM	NILM	Negative (NILM)	
P10425	2023	2023-Q2	2023-04	Post-pandemic recovery	Cytology (Pap)	35-39	38	Not performed	Satisfactory	NILM	NILM	Negative (NILM)	
P10426	2023	2023-Q2	2023-04	Post-pandemic recovery	Cytology (Pap)	50-54	50	Not performed	Satisfactory	NILM	NILM	Negative (NILM)	
P10427	2023	2023-Q2	2023-04	Post-pandemic recovery	Cytology (Pap)	35-39	35	Not performed	Satisfactory	Positive	ASC-US	Low-grade / equivocal squamous	
P10428	2023	2023-Q2	2023-04	Post-pandemic recovery	Cytology (Pap)	35-39	39	Not performed	Satisfactory	NILM	NILM	Negative (NILM)	
P10429	2023	2023-Q2	2023-04	Post-pandemic recovery	Cytology (Pap)	35-39	38	Not performed	Satisfactory	NILM	NILM	Negative (NILM)	
P10430	2023	2023-Q2	2023-04	Post-pandemic recovery	Cytology (Pap)	45-49	46	Not performed	Satisfactory	NILM	NILM	Negative (NILM)	
P10431	2023	2023-Q2	2023-04	Post-pandemic recovery	Cytology (Pap)	40-44	40	Not performed	Unsatisfactory	Unsatisfactory	Unsatisfactory	Unsatisfactory	
P10432	2023	2023-Q2	2023-04	Post-pandemic recovery	Cytology (Pap)	35-39	37	Not performed	Satisfactory	NILM	NILM	Negative (NILM)	
P10433	2023	2023-Q2	2023-04	Post-pandemic recovery	Cytology (Pap)	35-39	35	Not performed	Satisfactory	NILM	NILM	Negative (NILM)	
P10434	2023	2023-Q2	2023-04	Post-pandemic recovery	Cytology (Pap)	35-39	38	Not performed	Satisfactory	Positive	AGC	Glandular	
P10435	2023	2023-Q2	2023-04	Post-pandemic recovery	Cytology (Pap)	25-29	26	Not performed	Satisfactory	NILM	NILM	Negative (NILM)	
P10436	2023	2023-Q2	2023-04	Post-pandemic recovery	Cytology (Pap)	55-59	57	Not performed	Satisfactory	NILM	NILM	Negative (NILM)	
P10437	2023	2023-Q2	2023-04	Post-pandemic recovery	Cytology (Pap)	35-39	36	Not performed	Satisfactory	Positive	LSIL	Low-grade / equivocal squamous	
P10438	2023	2023-Q2	2023-04	Post-pandemic recovery	Cytology (Pap)	45-49	46	Not performed	Satisfactory	NILM	NILM	Negative (NILM)	
P10439	2023	2023-Q2	2023-04	Post-pandemic recovery	Cytology (Pap)	55-59	58	Not performed	Satisfactory	NILM	NILM	Negative (NILM)	
P10440	2023	2023-Q2	2023-04	Post-pandemic recovery	Cytology (Pap)	35-39	37	Not performed	Satisfactory	NILM	NILM	Negative (NILM)	
P10441	2023	2023-Q2	2023-04	Post-pandemic recovery	Cytology (Pap)	45-49	47	Not performed	Satisfactory	NILM	NILM	Negative (NILM)	
P10442	2023	2023-Q2	2023-04	Post-pandemic recovery	Cytology (Pap)	35-39	35	Not performed	Satisfactory	NILM	NILM	Negative (NILM)	
P10443	2023	2023-Q2	2023-04	Post-pandemic recovery	Cytology (Pap)	30-34	33	Not performed	Satisfactory	NILM	NILM	Negative (NILM)	
P10444	2023	2023-Q2	2023-04	Post-pandemic recovery	Cytology (Pap)	40-44	44	Not performed	Satisfactory	NILM	NILM	Negative (NILM)	
P10445	2023	2023-Q2	2023-04	Post-pandemic recovery	Cytology (Pap)	50-54	52	Not performed	Satisfactory	NILM	NILM	Negative (NILM)	
P10446	2023	2023-Q2	2023-04	Post-pandemic recovery	Cytology (Pap)	30-34	34	Not performed	Satisfactory	NILM	NILM	Negative (NILM)	
P10447	2023	2023-Q2	2023-04	Post-pandemic recovery	Cytology (Pap)	45-49	47	Not performed	Satisfactory	NILM	NILM	Negative (NILM)	
P10448	2023	2023-Q2	2023-04	Post-pandemic recovery	Cytology (Pap)	35-39	39	Not performed	Satisfactory	NILM	NILM	Negative (NILM)	
P10449	2023	2023-Q2	2023-04	Post-pandemic recovery	Cytology (Pap)	40-44	41	Not performed	Satisfactory	NILM	NILM	Negative (NILM)	Gardnerella
P10450	2023	2023-Q2	2023-04	Post-pandemic recovery	Cytology (Pap)	45-49	45	Not performed	Satisfactory	Positive	HSIL	High-grade squamous	
P10451	2023	2023-Q2	2023-04	Post-pandemic recovery	Cytology (Pap)	40-44	43	Not performed	Satisfactory	NILM	NILM	Negative (NILM)	
P10452	2023	2023-Q2	2023-04	Post-pandemic recovery	Cytology (Pap)	50-54	52	Not performed	Satisfactory	NILM	NILM	Negative (NILM)	
P10453	2023	2023-Q2	2023-04	Post-pandemic recovery	Cytology (Pap)	50-54	53	Not performed	Satisfactory	NILM	NILM	Negative (NILM)	
P10454	2023	2023-Q2	2023-04	Post-pandemic recovery	Cytology (Pap)	40-44	40	Not performed	Satisfactory	NILM	NILM	Negative (NILM)	Gardnerella
P10455	2023	2023-Q2	2023-04	Post-pandemic recovery	Cytology (Pap)	30-34	31	Not performed	Satisfactory	NILM	NILM	Negative (NILM)	Other
P10456	2023	2023-Q2	2023-04	Post-pandemic recovery	Cytology (Pap)	40-44	42	Not performed	Satisfactory	NILM	NILM	Negative (NILM)	
P10457	2023	2023-Q2	2023-04	Post-pandemic recovery	Cytology (Pap)	35-39	38	Not performed	Satisfactory	NILM	NILM	Negative (NILM)	
P10458	2023	2023-Q2	2023-04	Post-pandemic recovery	Cytology (Pap)	35-39	38	Not performed	Satisfactory	NILM	NILM	Negative (NILM)	
P10459	2023	2023-Q2	2023-04	Post-pandemic recovery	Cytology (Pap)	40-44	40	Not performed	Satisfactory	NILM	NILM	Negative (NILM)	
P10460	2023	2023-Q2	2023-04	Post-pandemic recovery	Cytology (Pap)	50-54	51	Not performed	Satisfactory	NILM	NILM	Negative (NILM)	
P10461	2023	2023-Q2	2023-04	Post-pandemic recovery	Cytology (Pap)	35-39	37	Not performed	Satisfactory	NILM	NILM	Negative (NILM)	
P10462	2023	2023-Q2	2023-04	Post-pandemic recovery	Cytology (Pap)	40-44	42	Not performed	Satisfactory	Positive	LSIL	Low-grade / equivocal squamous	
P10463	2023	2023-Q2	2023-04	Post-pandemic recovery	Cytology (Pap)	30-34	30	Not performed	Satisfactory	NILM	NILM	Negative (NILM)	
P10464	2023	2023-Q2	2023-04	Post-pandemic recovery	Cytology (Pap)	30-34	31	Not performed	Satisfactory	NILM	NILM	Negative (NILM)	
P10465	2023	2023-Q2	2023-04	Post-pandemic recovery	Cytology (Pap)	45-49	47	Not performed	Satisfactory	NILM	NILM	Negative (NILM)	
P10466	2023	2023-Q2	2023-04	Post-pandemic recovery	Cytology (Pap)	55-59	58	Not performed	Satisfactory	NILM	NILM	Negative (NILM)	Candida
P10467	2023	2023-Q2	2023-04	Post-pandemic recovery	Cytology (Pap)	30-34	30	Not performed	Satisfactory	NILM	NILM	Negative (NILM)	
P10468	2023	2023-Q2	2023-04	Post-pandemic recovery	Cytology (Pap)	45-49	47	Not performed	Satisfactory	NILM	NILM	Negative (NILM)	
P10469	2023	2023-Q2	2023-04	Post-pandemic recovery	Cytology (Pap)	55-59	59	Not performed	Satisfactory	NILM	NILM	Negative (NILM)	
P10470	2023	2023-Q2	2023-04	Post-pandemic recovery	Cytology (Pap)	35-39	37	Not performed	Satisfactory	Positive	LSIL	Low-grade / equivocal squamous	
P10471	2023	2023-Q2	2023-04	Post-pandemic recovery	Cytology (Pap)	30-34	31	Not performed	Satisfactory	NILM	NILM	Negative (NILM)	
P10472	2023	2023-Q2	2023-04	Post-pandemic recovery	Cytology (Pap)	35-39	37	Not performed	Satisfactory	NILM	NILM	Negative (NILM)	
P10473	2023	2023-Q2	2023-04	Post-pandemic recovery	Cytology (Pap)	35-39	39	Not performed	Satisfactory	NILM	NILM	Negative (NILM)	
P10474	2023	2023-Q2	2023-04	Post-pandemic recovery	Cytology (Pap)	30-34	30	Not performed	Satisfactory	NILM	NILM	Negative (NILM)	
P10475	2023	2023-Q2	2023-04	Post-pandemic recovery	Cytology (Pap)	45-49	47	Not performed	Satisfactory	NILM	NILM	Negative (NILM)	
P10476	2023	2023-Q2	2023-04	Post-pandemic recovery	Cytology (Pap)	50-54	50	Not performed	Satisfactory	NILM	NILM	Negative (NILM)	
P10477	2023	2023-Q2	2023-04	Post-pandemic recovery	Cytology (Pap)	55-59	57	Not performed	Satisfactory	NILM	NILM	Negative (NILM)	
P10478	2023	2023-Q2	2023-04	Post-pandemic recovery	Cytology (Pap)	55-59	58	Not performed	Satisfactory	NILM	NILM	Negative (NILM)	

patient_id	year	quarter	month	period	primary_test	age_group	age_years	hpv_result	adequacy	result	bethesda	grade	infection
P10479	2023	2023-Q2	2023-04	Post-pandemic recovery	Cytology (Pap)	35-39	39	Not performed	Satisfactory	NILM	NILM	Negative (NILM)	
P10480	2023	2023-Q2	2023-04	Post-pandemic recovery	Cytology (Pap)	50-54	50	Not performed	Satisfactory	NILM	NILM	Negative (NILM)	
P10481	2023	2023-Q2	2023-04	Post-pandemic recovery	Cytology (Pap)	35-39	38	Not performed	Satisfactory	NILM	NILM	Negative (NILM)	
P10482	2023	2023-Q2	2023-04	Post-pandemic recovery	Cytology (Pap)	35-39	37	Not performed	Satisfactory	NILM	NILM	Negative (NILM)	
P10483	2023	2023-Q2	2023-04	Post-pandemic recovery	Cytology (Pap)	40-44	43	Not performed	Satisfactory	Positive	LSIL	Low-grade / equivocal squamous	
P10484	2023	2023-Q2	2023-04	Post-pandemic recovery	Cytology (Pap)	55-59	55	Not performed	Satisfactory	NILM	NILM	Negative (NILM)	
P10485	2023	2023-Q2	2023-04	Post-pandemic recovery	Cytology (Pap)	35-39	39	Not performed	Satisfactory	NILM	NILM	Negative (NILM)	
P10486	2023	2023-Q2	2023-04	Post-pandemic recovery	Cytology (Pap)	50-54	53	Not performed	Satisfactory	NILM	NILM	Negative (NILM)	
P10487	2023	2023-Q2	2023-04	Post-pandemic recovery	Cytology (Pap)	40-44	42	Not performed	Satisfactory	NILM	NILM	Negative (NILM)	
P10488	2023	2023-Q2	2023-04	Post-pandemic recovery	Cytology (Pap)	30-34	30	Not performed	Satisfactory	NILM	NILM	Negative (NILM)	
P10489	2023	2023-Q2	2023-04	Post-pandemic recovery	Cytology (Pap)	40-44	44	Not performed	Satisfactory	Positive	ASC-US	Low-grade / equivocal squamous	
P10490	2023	2023-Q2	2023-04	Post-pandemic recovery	Cytology (Pap)	30-34	32	Not performed	Satisfactory	NILM	NILM	Negative (NILM)	
P10491	2023	2023-Q2	2023-04	Post-pandemic recovery	Cytology (Pap)	25-29	29	Not performed	Satisfactory	NILM	NILM	Negative (NILM)	
P10492	2023	2023-Q2	2023-04	Post-pandemic recovery	Cytology (Pap)	30-34	33	Not performed	Satisfactory	Positive	LSIL	Low-grade / equivocal squamous	
P10493	2023	2023-Q2	2023-04	Post-pandemic recovery	Cytology (Pap)	60-64	60	Not performed	Satisfactory	NILM	NILM	Negative (NILM)	
P10494	2023	2023-Q2	2023-04	Post-pandemic recovery	Cytology (Pap)	35-39	39	Not performed	Satisfactory	NILM	NILM	Negative (NILM)	
P10495	2023	2023-Q2	2023-04	Post-pandemic recovery	Cytology (Pap)	30-34	34	Not performed	Satisfactory	NILM	NILM	Negative (NILM)	
P10496	2023	2023-Q2	2023-04	Post-pandemic recovery	Cytology (Pap)	35-39	38	Not performed	Satisfactory	Positive	ASC-US	Low-grade / equivocal squamous	Candida
P10497	2023	2023-Q2	2023-04	Post-pandemic recovery	Cytology (Pap)	35-39	39	Not performed	Satisfactory	NILM	NILM	Negative (NILM)	
P10498	2023	2023-Q2	2023-04	Post-pandemic recovery	Cytology (Pap)	25-29	29	Not performed	Satisfactory	Positive	ASC-US	Low-grade / equivocal squamous	Trichomonas
P10499	2023	2023-Q2	2023-04	Post-pandemic recovery	Cytology (Pap)	45-49	47	Not performed	Satisfactory	Positive	HSIL	High-grade squamous	
P10500	2023	2023-Q2	2023-04	Post-pandemic recovery	Cytology (Pap)	40-44	42	Not performed	Satisfactory	NILM	NILM	Negative (NILM)	
P10501	2023	2023-Q2	2023-04	Post-pandemic recovery	Cytology (Pap)	30-34	34	Not performed	Satisfactory	NILM	NILM	Negative (NILM)	
P10502	2023	2023-Q2	2023-04	Post-pandemic recovery	Cytology (Pap)	40-44	40	Not performed	Satisfactory	NILM	NILM	Negative (NILM)	
P10503	2023	2023-Q2	2023-04	Post-pandemic recovery	Cytology (Pap)	40-44	41	Not performed	Satisfactory	NILM	NILM	Negative (NILM)	Candida
P10504	2023	2023-Q2	2023-04	Post-pandemic recovery	Cytology (Pap)	40-44	40	Not performed	Satisfactory	NILM	NILM	Negative (NILM)	
P10505	2023	2023-Q2	2023-04	Post-pandemic recovery	Cytology (Pap)	40-44	42	Not performed	Satisfactory	NILM	NILM	Negative (NILM)	
P10506	2023	2023-Q2	2023-04	Post-pandemic recovery	Cytology (Pap)	55-59	59	Not performed	Satisfactory	Positive	AGC	Glandular	
P10507	2023	2023-Q2	2023-04	Post-pandemic recovery	Cytology (Pap)	30-34	34	Not performed	Satisfactory	NILM	NILM	Negative (NILM)	
P10508	2023	2023-Q2	2023-04	Post-pandemic recovery	Cytology (Pap)	50-54	53	Not performed	Satisfactory	NILM	NILM	Negative (NILM)	Candida
P10509	2023	2023-Q2	2023-04	Post-pandemic recovery	Cytology (Pap)	50-54	52	Not performed	Satisfactory	NILM	NILM	Negative (NILM)	
P10510	2023	2023-Q2	2023-04	Post-pandemic recovery	Cytology (Pap)	40-44	41	Not performed	Satisfactory	NILM	NILM	Negative (NILM)	
P10511	2023	2023-Q2	2023-04	Post-pandemic recovery	Cytology (Pap)	35-39	35	Not performed	Satisfactory	NILM	NILM	Negative (NILM)	
P10512	2023	2023-Q2	2023-04	Post-pandemic recovery	Cytology (Pap)	25-29	29	Not performed	Satisfactory	NILM	NILM	Negative (NILM)	
P10513	2023	2023-Q2	2023-04	Post-pandemic recovery	Cytology (Pap)	45-49	45	Not performed	Satisfactory	NILM	NILM	Negative (NILM)	
P10514	2023	2023-Q2	2023-04	Post-pandemic recovery	Cytology (Pap)	35-39	35	Not performed	Satisfactory	NILM	NILM	Negative (NILM)	
P10515	2023	2023-Q2	2023-04	Post-pandemic recovery	Cytology (Pap)	50-54	53	Not performed	Satisfactory	NILM	NILM	Negative (NILM)	
P10516	2023	2023-Q2	2023-04	Post-pandemic recovery	Cytology (Pap)	60-64	60	Not performed	Satisfactory	NILM	NILM	Negative (NILM)	
P10517	2023	2023-Q2	2023-04	Post-pandemic recovery	Cytology (Pap)	30-34	31	Not performed	Unsatisfactory	Unsatisfactory	Unsatisfactory	Unsatisfactory	
P10518	2023	2023-Q2	2023-05	Post-pandemic recovery	Cytology (Pap)	30-34	33	Not performed	Satisfactory	NILM	NILM	Negative (NILM)	
P10519	2023	2023-Q2	2023-05	Post-pandemic recovery	Cytology (Pap)	40-44	42	Not performed	Satisfactory	NILM	NILM	Negative (NILM)	
P10520	2023	2023-Q2	2023-05	Post-pandemic recovery	Cytology (Pap)	40-44	43	Not performed	Satisfactory	Positive	LSIL	Low-grade / equivocal squamous	
P10521	2023	2023-Q2	2023-05	Post-pandemic recovery	Cytology (Pap)	55-59	55	Not performed	Satisfactory	NILM	NILM	Negative (NILM)	
P10522	2023	2023-Q2	2023-05	Post-pandemic recovery	Cytology (Pap)	45-49	46	Not performed	Satisfactory	NILM	NILM	Negative (NILM)	
P10523	2023	2023-Q2	2023-05	Post-pandemic recovery	Cytology (Pap)	40-44	40	Not performed	Satisfactory	NILM	NILM	Negative (NILM)	
P10524	2023	2023-Q2	2023-05	Post-pandemic recovery	Cytology (Pap)	35-39	35	Not performed	Satisfactory	NILM	NILM	Negative (NILM)	
P10525	2023	2023-Q2	2023-05	Post-pandemic recovery	Cytology (Pap)	45-49	47	Not performed	Satisfactory	NILM	NILM	Negative (NILM)	
P10526	2023	2023-Q2	2023-05	Post-pandemic recovery	Cytology (Pap)	40-44	41	Not performed	Satisfactory	Positive	ASC-US	Low-grade / equivocal squamous	
P10527	2023	2023-Q2	2023-05	Post-pandemic recovery	Cytology (Pap)	40-44	40	Not performed	Satisfactory	NILM	NILM	Negative (NILM)	
P10528	2023	2023-Q2	2023-05	Post-pandemic recovery	Cytology (Pap)	40-44	42	Not performed	Satisfactory	NILM	NILM	Negative (NILM)	
P10529	2023	2023-Q2	2023-05	Post-pandemic recovery	Cytology (Pap)	30-34	33	Not performed	Satisfactory	NILM	NILM	Negative (NILM)	
P10530	2023	2023-Q2	2023-05	Post-pandemic recovery	Cytology (Pap)	25-29	27	Not performed	Satisfactory	NILM	NILM	Negative (NILM)	
P10531	2023	2023-Q2	2023-05	Post-pandemic recovery	Cytology (Pap)	30-34	33	Not performed	Satisfactory	Positive	ASC-US	Low-grade / equivocal squamous	
P10532	2023	2023-Q2	2023-05	Post-pandemic recovery	Cytology (Pap)	45-49	47	Not performed	Satisfactory	NILM	NILM	Negative (NILM)	
P10533	2023	2023-Q2	2023-05	Post-pandemic recovery	Cytology (Pap)	40-44	42	Not performed	Satisfactory	NILM	NILM	Negative (NILM)	
P10534	2023	2023-Q2	2023-05	Post-pandemic recovery	Cytology (Pap)	40-44	44	Not performed	Satisfactory	Positive	HSIL	High-grade squamous	
P10535	2023	2023-Q2	2023-05	Post-pandemic recovery	Cytology (Pap)	55-59	56	Not performed	Satisfactory	NILM	NILM	Negative (NILM)	
P10536	2023	2023-Q2	2023-05	Post-pandemic recovery	Cytology (Pap)	35-39	39	Not performed	Satisfactory	Positive	ASC-US	Low-grade / equivocal squamous	
P10537	2023	2023-Q2	2023-05	Post-pandemic recovery	Cytology (Pap)	50-54	51	Not performed	Satisfactory	NILM	NILM	Negative (NILM)	
P10538	2023	2023-Q2	2023-05	Post-pandemic recovery	Cytology (Pap)	45-49	49	Not performed	Satisfactory	NILM	NILM	Negative (NILM)	
P10539	2023	2023-Q2	2023-05	Post-pandemic recovery	Cytology (Pap)	60-64	61	Not performed	Satisfactory	NILM	NILM	Negative (NILM)	
P10540	2023	2023-Q2	2023-05	Post-pandemic recovery	Cytology (Pap)	50-54	52	Not performed	Satisfactory	NILM	NILM	Negative (NILM)	
P10541	2023	2023-Q2	2023-05	Post-pandemic recovery	Cytology (Pap)	55-59	58	Not performed	Satisfactory	NILM	NILM	Negative (NILM)	
P10542	2023	2023-Q2	2023-05	Post-pandemic recovery	Cytology (Pap)	30-34	34	Not performed	Satisfactory	NILM	NILM	Negative (NILM)	
P10543	2023	2023-Q2	2023-05	Post-pandemic recovery	Cytology (Pap)	35-39	38	Not performed	Satisfactory	Positive	AGC	Glandular	
P10544	2023	2023-Q2	2023-05	Post-pandemic recovery	Cytology (Pap)	40-44	41	Not performed	Satisfactory	Positive	ASC-US	Low-grade / equivocal squamous	
P10545	2023	2023-Q2	2023-05	Post-pandemic recovery	Cytology (Pap)	45-49	48	Not performed	Satisfactory	NILM	NILM	Negative (NILM)	
P10546	2023	2023-Q2	2023-05	Post-pandemic recovery	Cytology (Pap)	35-39	36	Not performed	Satisfactory	NILM	NILM	Negative (NILM)	
P10547	2023	2023-Q2	2023-05	Post-pandemic recovery	Cytology (Pap)	55-59	56	Not performed	Satisfactory	NILM	NILM	Negative (NILM)	Candida
P10548	2023	2023-Q2	2023-05	Post-pandemic recovery	Cytology (Pap)	25-29	25	Not performed	Satisfactory	NILM	NILM	Negative (NILM)	
P10549	2023	2023-Q2	2023-05	Post-pandemic recovery	Cytology (Pap)	55-59	58	Not performed	Satisfactory	NILM	NILM	Negative (NILM)	
P10550	2023	2023-Q2	2023-05	Post-pandemic recovery	Cytology (Pap)	25-29	27	Not performed	Satisfactory	NILM	NILM	Negative (NILM)	
P10551	2023	2023-Q2	2023-05	Post-pandemic recovery	Cytology (Pap)	60-64	64	Not performed	Satisfactory	NILM	NILM	Negative (NILM)	
P10552	2023	2023-Q2	2023-05	Post-pandemic recovery	Cytology (Pap)	35-39	35	Not performed	Satisfactory	Positive	ASC-US	Low-grade / equivocal squamous	
P10553	2023	2023-Q2	2023-05	Post-pandemic recovery	Cytology (Pap)	35-39	38	Not performed	Satisfactory	NILM	NILM	Negative (NILM)	
P10554	2023	2023-Q2	2023-05	Post-pandemic recovery	Cytology (Pap)	30-34	34	Not performed	Satisfactory	NILM	NILM	Negative (NILM)	
P10555	2023	2023-Q2	2023-05	Post-pandemic recovery	Cytology (Pap)	60-64	63	Not performed	Satisfactory	NILM	NILM	Negative (NILM)	
P10556	2023	2023-Q2	2023-05	Post-pandemic recovery	Cytology (Pap)	45-49	45	Not performed	Satisfactory	NILM	NILM	Negative (NILM)	
P10557	2023	2023-Q2	2023-05	Post-pandemic recovery	Cytology (Pap)	40-44	44	Not performed	Satisfactory	NILM	NILM	Negative (NILM)	
P10558	2023	2023-Q2	2023-05	Post-pandemic recovery	Cytology (Pap)	35-39	37	Not performed	Satisfactory	NILM	NILM	Negative (NILM)	

patient_id	year	quarter	month	period	primary_test	age_group	age_years	hpv_result	adequacy	result	bethesda	grade	infection
P10559	2023	2023-Q2	2023-05	Post-pandemic recovery	Cytology (Pap)	50-54	54	Not performed	Satisfactory	Positive	ASC-US	Low-grade / equivocal squamous	
P10560	2023	2023-Q2	2023-05	Post-pandemic recovery	Cytology (Pap)	40-44	43	Not performed	Satisfactory	Positive	LSIL	Low-grade / equivocal squamous	
P10561	2023	2023-Q2	2023-05	Post-pandemic recovery	Cytology (Pap)	35-39	39	Not performed	Satisfactory	NILM	NILM	Negative (NILM)	
P10562	2023	2023-Q2	2023-05	Post-pandemic recovery	Cytology (Pap)	30-34	30	Not performed	Satisfactory	NILM	NILM	Negative (NILM)	
P10563	2023	2023-Q2	2023-05	Post-pandemic recovery	Cytology (Pap)	35-39	35	Not performed	Satisfactory	NILM	NILM	Negative (NILM)	
P10564	2023	2023-Q2	2023-05	Post-pandemic recovery	Cytology (Pap)	45-49	48	Not performed	Unsatisfactory	Unsatisfactory	Unsatisfactory	Unsatisfactory	
P10565	2023	2023-Q2	2023-05	Post-pandemic recovery	Cytology (Pap)	45-49	47	Not performed	Satisfactory	NILM	NILM	Negative (NILM)	
P10566	2023	2023-Q2	2023-05	Post-pandemic recovery	Cytology (Pap)	35-39	39	Not performed	Satisfactory	NILM	NILM	Negative (NILM)	
P10567	2023	2023-Q2	2023-05	Post-pandemic recovery	Cytology (Pap)	45-49	45	Not performed	Satisfactory	NILM	NILM	Negative (NILM)	
P10568	2023	2023-Q2	2023-05	Post-pandemic recovery	Cytology (Pap)	40-44	44	Not performed	Satisfactory	NILM	NILM	Negative (NILM)	
P10569	2023	2023-Q2	2023-05	Post-pandemic recovery	Cytology (Pap)	30-34	33	Not performed	Satisfactory	NILM	NILM	Negative (NILM)	
P10570	2023	2023-Q2	2023-05	Post-pandemic recovery	Cytology (Pap)	50-54	50	Not performed	Satisfactory	NILM	NILM	Negative (NILM)	
P10571	2023	2023-Q2	2023-05	Post-pandemic recovery	Cytology (Pap)	55-59	59	Not performed	Satisfactory	NILM	NILM	Negative (NILM)	
P10572	2023	2023-Q2	2023-05	Post-pandemic recovery	Cytology (Pap)	45-49	45	Not performed	Satisfactory	Positive	LSIL	Low-grade / equivocal squamous	
P10573	2023	2023-Q2	2023-05	Post-pandemic recovery	Cytology (Pap)	35-39	37	Not performed	Satisfactory	NILM	NILM	Negative (NILM)	
P10574	2023	2023-Q2	2023-05	Post-pandemic recovery	Cytology (Pap)	35-39	39	Not performed	Satisfactory	NILM	NILM	Negative (NILM)	
P10575	2023	2023-Q2	2023-05	Post-pandemic recovery	Cytology (Pap)	35-39	35	Not performed	Satisfactory	NILM	NILM	Negative (NILM)	Trichomonas
P10576	2023	2023-Q2	2023-05	Post-pandemic recovery	Cytology (Pap)	30-34	33	Not performed	Satisfactory	Positive	ASC-US	Low-grade / equivocal squamous	Candida
P10577	2023	2023-Q2	2023-05	Post-pandemic recovery	Cytology (Pap)	40-44	42	Not performed	Unsatisfactory	Unsatisfactory	Unsatisfactory	Unsatisfactory	
P10578	2023	2023-Q2	2023-05	Post-pandemic recovery	Cytology (Pap)	25-29	29	Not performed	Satisfactory	NILM	NILM	Negative (NILM)	
P10579	2023	2023-Q2	2023-05	Post-pandemic recovery	Cytology (Pap)	25-29	25	Not performed	Satisfactory	Positive	ASC-US	Low-grade / equivocal squamous	
P10580	2023	2023-Q2	2023-05	Post-pandemic recovery	Cytology (Pap)	60-64	63	Not performed	Satisfactory	NILM	NILM	Negative (NILM)	
P10581	2023	2023-Q2	2023-05	Post-pandemic recovery	Cytology (Pap)	35-39	39	Not performed	Satisfactory	NILM	NILM	Negative (NILM)	
P10582	2023	2023-Q2	2023-05	Post-pandemic recovery	Cytology (Pap)	40-44	42	Not performed	Satisfactory	NILM	NILM	Negative (NILM)	
P10583	2023	2023-Q2	2023-05	Post-pandemic recovery	Cytology (Pap)	40-44	43	Not performed	Satisfactory	NILM	NILM	Negative (NILM)	
P10584	2023	2023-Q2	2023-05	Post-pandemic recovery	Cytology (Pap)	35-39	35	Not performed	Satisfactory	NILM	NILM	Negative (NILM)	
P10585	2023	2023-Q2	2023-05	Post-pandemic recovery	Cytology (Pap)	35-39	35	Not performed	Satisfactory	NILM	NILM	Negative (NILM)	
P10586	2023	2023-Q2	2023-05	Post-pandemic recovery	Cytology (Pap)	30-34	32	Not performed	Satisfactory	NILM	NILM	Negative (NILM)	
P10587	2023	2023-Q2	2023-05	Post-pandemic recovery	Cytology (Pap)	55-59	57	Not performed	Satisfactory	NILM	NILM	Negative (NILM)	
P10588	2023	2023-Q2	2023-05	Post-pandemic recovery	Cytology (Pap)	25-29	25	Not performed	Satisfactory	NILM	NILM	Negative (NILM)	
P10589	2023	2023-Q2	2023-05	Post-pandemic recovery	Cytology (Pap)	50-54	54	Not performed	Satisfactory	NILM	NILM	Negative (NILM)	
P10590	2023	2023-Q2	2023-05	Post-pandemic recovery	Cytology (Pap)	40-44	41	Not performed	Satisfactory	NILM	NILM	Negative (NILM)	
P10591	2023	2023-Q2	2023-05	Post-pandemic recovery	Cytology (Pap)	30-34	32	Not performed	Satisfactory	NILM	NILM	Negative (NILM)	
P10592	2023	2023-Q2	2023-05	Post-pandemic recovery	Cytology (Pap)	40-44	41	Not performed	Satisfactory	NILM	NILM	Negative (NILM)	
P10593	2023	2023-Q2	2023-05	Post-pandemic recovery	Cytology (Pap)	30-34	34	Not performed	Satisfactory	NILM	NILM	Negative (NILM)	
P10594	2023	2023-Q2	2023-05	Post-pandemic recovery	Cytology (Pap)	55-59	59	Not performed	Satisfactory	NILM	NILM	Negative (NILM)	
P10595	2023	2023-Q2	2023-05	Post-pandemic recovery	Cytology (Pap)	30-34	33	Not performed	Satisfactory	NILM	NILM	Negative (NILM)	
P10596	2023	2023-Q2	2023-05	Post-pandemic recovery	Cytology (Pap)	30-34	30	Not performed	Satisfactory	NILM	NILM	Negative (NILM)	
P10597	2023	2023-Q2	2023-05	Post-pandemic recovery	Cytology (Pap)	50-54	51	Not performed	Satisfactory	NILM	NILM	Negative (NILM)	
P10598	2023	2023-Q2	2023-05	Post-pandemic recovery	Cytology (Pap)	25-29	28	Not performed	Satisfactory	NILM	NILM	Negative (NILM)	Gardnerella
P10599	2023	2023-Q2	2023-05	Post-pandemic recovery	Cytology (Pap)	25-29	26	Not performed	Satisfactory	NILM	NILM	Negative (NILM)	
P10600	2023	2023-Q2	2023-05	Post-pandemic recovery	Cytology (Pap)	25-29	27	Not performed	Satisfactory	Positive	LSIL	Low-grade / equivocal squamous	
P10601	2023	2023-Q2	2023-05	Post-pandemic recovery	Cytology (Pap)	45-49	47	Not performed	Satisfactory	NILM	NILM	Negative (NILM)	
P10602	2023	2023-Q2	2023-05	Post-pandemic recovery	Cytology (Pap)	60-64	61	Not performed	Satisfactory	Positive	LSIL	Low-grade / equivocal squamous	Candida
P10603	2023	2023-Q2	2023-05	Post-pandemic recovery	Cytology (Pap)	55-59	56	Not performed	Satisfactory	NILM	NILM	Negative (NILM)	
P10604	2023	2023-Q2	2023-05	Post-pandemic recovery	Cytology (Pap)	50-54	53	Not performed	Satisfactory	NILM	NILM	Negative (NILM)	
P10605	2023	2023-Q2	2023-05	Post-pandemic recovery	Cytology (Pap)	30-34	34	Not performed	Satisfactory	NILM	NILM	Negative (NILM)	
P10606	2023	2023-Q2	2023-05	Post-pandemic recovery	Cytology (Pap)	55-59	57	Not performed	Satisfactory	NILM	NILM	Negative (NILM)	
P10607	2023	2023-Q2	2023-05	Post-pandemic recovery	Cytology (Pap)	50-54	50	Not performed	Satisfactory	NILM	NILM	Negative (NILM)	
P10608	2023	2023-Q2	2023-05	Post-pandemic recovery	Cytology (Pap)	35-39	39	Not performed	Satisfactory	NILM	NILM	Negative (NILM)	
P10609	2023	2023-Q2	2023-05	Post-pandemic recovery	Cytology (Pap)	35-39	37	Not performed	Satisfactory	NILM	NILM	Negative (NILM)	
P10610	2023	2023-Q2	2023-05	Post-pandemic recovery	Cytology (Pap)	35-39	36	Not performed	Satisfactory	NILM	NILM	Negative (NILM)	
P10611	2023	2023-Q2	2023-05	Post-pandemic recovery	Cytology (Pap)	25-29	26	Not performed	Satisfactory	NILM	NILM	Negative (NILM)	
P10612	2023	2023-Q2	2023-05	Post-pandemic recovery	Cytology (Pap)	40-44	43	Not performed	Satisfactory	NILM	NILM	Negative (NILM)	
P10613	2023	2023-Q2	2023-05	Post-pandemic recovery	Cytology (Pap)	60-64	61	Not performed	Satisfactory	NILM	NILM	Negative (NILM)	
P10614	2023	2023-Q2	2023-05	Post-pandemic recovery	Cytology (Pap)	30-34	31	Not performed	Satisfactory	NILM	NILM	Negative (NILM)	
P10615	2023	2023-Q2	2023-05	Post-pandemic recovery	Cytology (Pap)	40-44	44	Not performed	Satisfactory	NILM	NILM	Negative (NILM)	
P10616	2023	2023-Q2	2023-05	Post-pandemic recovery	Cytology (Pap)	45-49	45	Not performed	Satisfactory	NILM	NILM	Negative (NILM)	
P10617	2023	2023-Q2	2023-05	Post-pandemic recovery	Cytology (Pap)	40-44	41	Not performed	Satisfactory	Positive	ASC-US	Low-grade / equivocal squamous	
P10618	2023	2023-Q2	2023-05	Post-pandemic recovery	Cytology (Pap)	25-29	26	Not performed	Satisfactory	NILM	NILM	Negative (NILM)	
P10619	2023	2023-Q2	2023-05	Post-pandemic recovery	Cytology (Pap)	30-34	33	Not performed	Satisfactory	Positive	LSIL	Low-grade / equivocal squamous	
P10620	2023	2023-Q2	2023-05	Post-pandemic recovery	Cytology (Pap)	40-44	41	Not performed	Satisfactory	NILM	NILM	Negative (NILM)	
P10621	2023	2023-Q2	2023-05	Post-pandemic recovery	Cytology (Pap)	35-39	39	Not performed	Satisfactory	NILM	NILM	Negative (NILM)	
P10622	2023	2023-Q2	2023-05	Post-pandemic recovery	Cytology (Pap)	50-54	52	Not performed	Satisfactory	NILM	NILM	Negative (NILM)	
P10623	2023	2023-Q2	2023-05	Post-pandemic recovery	Cytology (Pap)	40-44	40	Not performed	Satisfactory	NILM	NILM	Negative (NILM)	
P10624	2023	2023-Q2	2023-05	Post-pandemic recovery	Cytology (Pap)	40-44	43	Not performed	Satisfactory	NILM	NILM	Negative (NILM)	
P10625	2023	2023-Q2	2023-05	Post-pandemic recovery	Cytology (Pap)	40-44	43	Not performed	Satisfactory	NILM	NILM	Negative (NILM)	
P10626	2023	2023-Q2	2023-05	Post-pandemic recovery	Cytology (Pap)	25-29	25	Not performed	Satisfactory	Positive	LSIL	Low-grade / equivocal squamous	
P10627	2023	2023-Q2	2023-05	Post-pandemic recovery	Cytology (Pap)	30-34	34	Not performed	Satisfactory	NILM	NILM	Negative (NILM)	
P10628	2023	2023-Q2	2023-05	Post-pandemic recovery	Cytology (Pap)	35-39	35	Not performed	Satisfactory	NILM	NILM	Negative (NILM)	
P10629	2023	2023-Q2	2023-05	Post-pandemic recovery	Cytology (Pap)	40-44	42	Not performed	Satisfactory	NILM	NILM	Negative (NILM)	
P10630	2023	2023-Q2	2023-05	Post-pandemic recovery	Cytology (Pap)	60-64	61	Not performed	Satisfactory	NILM	NILM	Negative (NILM)	
P10631	2023	2023-Q2	2023-05	Post-pandemic recovery	Cytology (Pap)	35-39	36	Not performed	Satisfactory	NILM	NILM	Negative (NILM)	
P10632	2023	2023-Q2	2023-05	Post-pandemic recovery	Cytology (Pap)	40-44	42	Not performed	Satisfactory	NILM	NILM	Negative (NILM)	
P10633	2023	2023-Q2	2023-05	Post-pandemic recovery	Cytology (Pap)	45-49	48	Not performed	Unsatisfactory	Unsatisfactory	Unsatisfactory	Unsatisfactory	
P10634	2023	2023-Q2	2023-05	Post-pandemic recovery	Cytology (Pap)	50-54	50	Not performed	Satisfactory	NILM	NILM	Negative (NILM)	
P10635	2023	2023-Q2	2023-05	Post-pandemic recovery	Cytology (Pap)	50-54	53	Not performed	Satisfactory	NILM	NILM	Negative (NILM)	
P10636	2023	2023-Q2	2023-05	Post-pandemic recovery	Cytology (Pap)	40-44	40	Not performed	Satisfactory	NILM	NILM	Negative (NILM)	
P10637	2023	2023-Q2	2023-05	Post-pandemic recovery	Cytology (Pap)	50-54	53	Not performed	Satisfactory	NILM	NILM	Negative (NILM)	
P10638	2023	2023-Q2	2023-05	Post-pandemic recovery	Cytology (Pap)	60-64	62	Not performed	Satisfactory	NILM	NILM	Negative (NILM)	

patient_id	year	quarter	month	period	primary_test	age_group	age_years	hpv_result	adequacy	result	bethesda	grade	infection
P10639	2023	2023-Q2	2023-05	Post-pandemic recovery	Cytology (Pap)	35-39	39	Not performed	Satisfactory	NILM	NILM	Negative (NILM)	
P10640	2023	2023-Q2	2023-05	Post-pandemic recovery	Cytology (Pap)	50-54	52	Not performed	Satisfactory	NILM	NILM	Negative (NILM)	
P10641	2023	2023-Q2	2023-05	Post-pandemic recovery	Cytology (Pap)	40-44	44	Not performed	Unsatisfactory	Unsatisfactory	Unsatisfactory	Unsatisfactory	
P10642	2023	2023-Q2	2023-05	Post-pandemic recovery	Cytology (Pap)	35-39	36	Not performed	Satisfactory	NILM	NILM	Negative (NILM)	Gardnerella
P10643	2023	2023-Q2	2023-05	Post-pandemic recovery	Cytology (Pap)	55-59	56	Not performed	Satisfactory	NILM	NILM	Negative (NILM)	
P10644	2023	2023-Q2	2023-05	Post-pandemic recovery	Cytology (Pap)	55-59	57	Not performed	Satisfactory	NILM	NILM	Negative (NILM)	
P10645	2023	2023-Q2	2023-05	Post-pandemic recovery	Cytology (Pap)	35-39	35	Not performed	Unsatisfactory	Unsatisfactory	Unsatisfactory	Unsatisfactory	
P10646	2023	2023-Q2	2023-05	Post-pandemic recovery	Cytology (Pap)	55-59	55	Not performed	Satisfactory	NILM	NILM	Negative (NILM)	
P10647	2023	2023-Q2	2023-05	Post-pandemic recovery	Cytology (Pap)	50-54	52	Not performed	Satisfactory	NILM	NILM	Negative (NILM)	
P10648	2023	2023-Q2	2023-05	Post-pandemic recovery	Cytology (Pap)	40-44	40	Not performed	Satisfactory	Positive	LSIL	Low-grade / equivocal squamous	
P10649	2023	2023-Q2	2023-05	Post-pandemic recovery	Cytology (Pap)	30-34	32	Not performed	Satisfactory	NILM	NILM	Negative (NILM)	Gardnerella
P10650	2023	2023-Q2	2023-05	Post-pandemic recovery	Cytology (Pap)	45-49	46	Not performed	Satisfactory	NILM	NILM	Negative (NILM)	
P10651	2023	2023-Q2	2023-05	Post-pandemic recovery	Cytology (Pap)	60-64	61	Not performed	Satisfactory	NILM	NILM	Negative (NILM)	
P10652	2023	2023-Q2	2023-05	Post-pandemic recovery	Cytology (Pap)	60-64	62	Not performed	Satisfactory	NILM	NILM	Negative (NILM)	
P10653	2023	2023-Q2	2023-05	Post-pandemic recovery	Cytology (Pap)	55-59	58	Not performed	Satisfactory	NILM	NILM	Negative (NILM)	
P10654	2023	2023-Q2	2023-05	Post-pandemic recovery	Cytology (Pap)	55-59	56	Not performed	Satisfactory	NILM	NILM	Negative (NILM)	
P10655	2023	2023-Q2	2023-05	Post-pandemic recovery	Cytology (Pap)	40-44	44	Not performed	Satisfactory	NILM	NILM	Negative (NILM)	
P10656	2023	2023-Q2	2023-05	Post-pandemic recovery	Cytology (Pap)	40-44	44	Not performed	Satisfactory	NILM	NILM	Negative (NILM)	
P10657	2023	2023-Q2	2023-05	Post-pandemic recovery	Cytology (Pap)	35-39	35	Not performed	Satisfactory	NILM	NILM	Negative (NILM)	
P10658	2023	2023-Q2	2023-05	Post-pandemic recovery	Cytology (Pap)	35-39	39	Not performed	Satisfactory	NILM	NILM	Negative (NILM)	
P10659	2023	2023-Q2	2023-05	Post-pandemic recovery	Cytology (Pap)	50-54	51	Not performed	Satisfactory	NILM	NILM	Negative (NILM)	
P10660	2023	2023-Q2	2023-05	Post-pandemic recovery	Cytology (Pap)	35-39	37	Not performed	Satisfactory	NILM	NILM	Negative (NILM)	Trichomonas
P10661	2023	2023-Q2	2023-05	Post-pandemic recovery	Cytology (Pap)	35-39	38	Not performed	Satisfactory	NILM	NILM	Negative (NILM)	
P10662	2023	2023-Q2	2023-05	Post-pandemic recovery	Cytology (Pap)	30-34	33	Not performed	Satisfactory	NILM	NILM	Negative (NILM)	
P10663	2023	2023-Q2	2023-05	Post-pandemic recovery	Cytology (Pap)	35-39	35	Not performed	Satisfactory	NILM	NILM	Negative (NILM)	
P10664	2023	2023-Q2	2023-05	Post-pandemic recovery	Cytology (Pap)	25-29	29	Not performed	Satisfactory	NILM	NILM	Negative (NILM)	
P10665	2023	2023-Q2	2023-05	Post-pandemic recovery	Cytology (Pap)	40-44	41	Not performed	Satisfactory	NILM	NILM	Negative (NILM)	
P10666	2023	2023-Q2	2023-05	Post-pandemic recovery	Cytology (Pap)	45-49	47	Not performed	Satisfactory	Positive	ASC-H	High-grade squamous	
P10667	2023	2023-Q2	2023-05	Post-pandemic recovery	Cytology (Pap)	30-34	30	Not performed	Satisfactory	NILM	NILM	Negative (NILM)	
P10668	2023	2023-Q2	2023-05	Post-pandemic recovery	Cytology (Pap)	40-44	41	Not performed	Satisfactory	Positive	ASC-US	Low-grade / equivocal squamous	
P10669	2023	2023-Q2	2023-05	Post-pandemic recovery	Cytology (Pap)	55-59	55	Not performed	Satisfactory	NILM	NILM	Negative (NILM)	
P10670	2023	2023-Q2	2023-05	Post-pandemic recovery	Cytology (Pap)	40-44	40	Not performed	Satisfactory	NILM	NILM	Negative (NILM)	
P10671	2023	2023-Q2	2023-05	Post-pandemic recovery	Cytology (Pap)	35-39	37	Not performed	Satisfactory	NILM	NILM	Negative (NILM)	
P10672	2023	2023-Q2	2023-05	Post-pandemic recovery	Cytology (Pap)	40-44	41	Not performed	Satisfactory	NILM	NILM	Negative (NILM)	
P10673	2023	2023-Q2	2023-05	Post-pandemic recovery	Cytology (Pap)	30-34	33	Not performed	Satisfactory	Positive	LSIL	Low-grade / equivocal squamous	Gardnerella
P10674	2023	2023-Q2	2023-05	Post-pandemic recovery	Cytology (Pap)	35-39	37	Not performed	Satisfactory	Positive	ASC-US	Low-grade / equivocal squamous	
P10675	2023	2023-Q2	2023-05	Post-pandemic recovery	Cytology (Pap)	30-34	32	Not performed	Satisfactory	NILM	NILM	Negative (NILM)	
P10676	2023	2023-Q2	2023-05	Post-pandemic recovery	Cytology (Pap)	30-34	32	Not performed	Satisfactory	NILM	NILM	Negative (NILM)	
P10677	2023	2023-Q2	2023-05	Post-pandemic recovery	Cytology (Pap)	55-59	58	Not performed	Satisfactory	NILM	NILM	Negative (NILM)	
P10678	2023	2023-Q2	2023-05	Post-pandemic recovery	Cytology (Pap)	30-34	33	Not performed	Satisfactory	NILM	NILM	Negative (NILM)	
P10679	2023	2023-Q2	2023-05	Post-pandemic recovery	Cytology (Pap)	25-29	26	Not performed	Satisfactory	NILM	NILM	Negative (NILM)	
P10680	2023	2023-Q2	2023-05	Post-pandemic recovery	Cytology (Pap)	55-59	56	Not performed	Satisfactory	NILM	NILM	Negative (NILM)	
P10681	2023	2023-Q2	2023-05	Post-pandemic recovery	Cytology (Pap)	60-64	62	Not performed	Satisfactory	NILM	NILM	Negative (NILM)	
P10682	2023	2023-Q2	2023-05	Post-pandemic recovery	Cytology (Pap)	45-49	47	Not performed	Satisfactory	NILM	NILM	Negative (NILM)	Candida
P10683	2023	2023-Q2	2023-05	Post-pandemic recovery	Cytology (Pap)	40-44	42	Not performed	Satisfactory	NILM	NILM	Negative (NILM)	
P10684	2023	2023-Q2	2023-05	Post-pandemic recovery	Cytology (Pap)	30-34	34	Not performed	Satisfactory	NILM	NILM	Negative (NILM)	Candida
P10685	2023	2023-Q2	2023-05	Post-pandemic recovery	Cytology (Pap)	40-44	41	Not performed	Satisfactory	NILM	NILM	Negative (NILM)	
P10686	2023	2023-Q2	2023-05	Post-pandemic recovery	Cytology (Pap)	30-34	33	Not performed	Satisfactory	NILM	NILM	Negative (NILM)	
P10687	2023	2023-Q2	2023-05	Post-pandemic recovery	Cytology (Pap)	50-54	54	Not performed	Satisfactory	NILM	NILM	Negative (NILM)	
P10688	2023	2023-Q2	2023-05	Post-pandemic recovery	Cytology (Pap)	30-34	30	Not performed	Satisfactory	NILM	NILM	Negative (NILM)	
P10689	2023	2023-Q2	2023-05	Post-pandemic recovery	Cytology (Pap)	40-44	43	Not performed	Satisfactory	NILM	NILM	Negative (NILM)	
P10690	2023	2023-Q2	2023-05	Post-pandemic recovery	Cytology (Pap)	30-34	33	Not performed	Satisfactory	Positive	ASC-US	Low-grade / equivocal squamous	
P10691	2023	2023-Q2	2023-05	Post-pandemic recovery	Cytology (Pap)	35-39	35	Not performed	Satisfactory	NILM	NILM	Negative (NILM)	
P10692	2023	2023-Q2	2023-05	Post-pandemic recovery	Cytology (Pap)	30-34	31	Not performed	Satisfactory	Positive	LSIL	Low-grade / equivocal squamous	Trichomonas
P10693	2023	2023-Q2	2023-05	Post-pandemic recovery	Cytology (Pap)	40-44	41	Not performed	Satisfactory	NILM	NILM	Negative (NILM)	
P10694	2023	2023-Q2	2023-05	Post-pandemic recovery	Cytology (Pap)	55-59	59	Not performed	Satisfactory	NILM	NILM	Negative (NILM)	
P10695	2023	2023-Q2	2023-05	Post-pandemic recovery	Cytology (Pap)	40-44	40	Not performed	Satisfactory	Positive	HSIL	High-grade squamous	
P10696	2023	2023-Q2	2023-05	Post-pandemic recovery	Cytology (Pap)	45-49	47	Not performed	Satisfactory	NILM	NILM	Negative (NILM)	
P10697	2023	2023-Q2	2023-05	Post-pandemic recovery	Cytology (Pap)	40-44	41	Not performed	Satisfactory	Positive	ASC-US	Low-grade / equivocal squamous	
P10698	2023	2023-Q2	2023-06	Post-pandemic recovery	Cytology (Pap)	40-44	41	Not performed	Satisfactory	NILM	NILM	Negative (NILM)	
P10699	2023	2023-Q2	2023-06	Post-pandemic recovery	Cytology (Pap)	25-29	28	Not performed	Satisfactory	Positive	LSIL	Low-grade / equivocal squamous	
P10700	2023	2023-Q2	2023-06	Post-pandemic recovery	Cytology (Pap)	40-44	44	Not performed	Satisfactory	NILM	NILM	Negative (NILM)	
P10701	2023	2023-Q2	2023-06	Post-pandemic recovery	Cytology (Pap)	30-34	33	Not performed	Satisfactory	NILM	NILM	Negative (NILM)	
P10702	2023	2023-Q2	2023-06	Post-pandemic recovery	Cytology (Pap)	35-39	37	Not performed	Satisfactory	NILM	NILM	Negative (NILM)	
P10703	2023	2023-Q2	2023-06	Post-pandemic recovery	Cytology (Pap)	50-54	51	Not performed	Satisfactory	NILM	NILM	Negative (NILM)	
P10704	2023	2023-Q2	2023-06	Post-pandemic recovery	Cytology (Pap)	40-44	40	Not performed	Satisfactory	NILM	NILM	Negative (NILM)	
P10705	2023	2023-Q2	2023-06	Post-pandemic recovery	Cytology (Pap)	45-49	48	Not performed	Satisfactory	NILM	NILM	Negative (NILM)	
P10706	2023	2023-Q2	2023-06	Post-pandemic recovery	Cytology (Pap)	60-64	64	Not performed	Satisfactory	NILM	NILM	Negative (NILM)	
P10707	2023	2023-Q2	2023-06	Post-pandemic recovery	Cytology (Pap)	35-39	39	Not performed	Satisfactory	NILM	NILM	Negative (NILM)	
P10708	2023	2023-Q2	2023-06	Post-pandemic recovery	Cytology (Pap)	45-49	45	Not performed	Satisfactory	NILM	NILM	Negative (NILM)	
P10709	2023	2023-Q2	2023-06	Post-pandemic recovery	Cytology (Pap)	25-29	27	Not performed	Satisfactory	Positive	LSIL	Low-grade / equivocal squamous	
P10710	2023	2023-Q2	2023-06	Post-pandemic recovery	Cytology (Pap)	45-49	45	Not performed	Satisfactory	NILM	NILM	Negative (NILM)	
P10711	2023	2023-Q2	2023-06	Post-pandemic recovery	Cytology (Pap)	40-44	41	Not performed	Satisfactory	NILM	NILM	Negative (NILM)	
P10712	2023	2023-Q2	2023-06	Post-pandemic recovery	Cytology (Pap)	30-34	34	Not performed	Satisfactory	NILM	NILM	Negative (NILM)	
P10713	2023	2023-Q2	2023-06	Post-pandemic recovery	Cytology (Pap)	50-54	50	Not performed	Satisfactory	NILM	NILM	Negative (NILM)	
P10714	2023	2023-Q2	2023-06	Post-pandemic recovery	Cytology (Pap)	40-44	41	Not performed	Satisfactory	Positive	LSIL	Low-grade / equivocal squamous	Gardnerella
P10715	2023	2023-Q2	2023-06	Post-pandemic recovery	Cytology (Pap)	45-49	47	Not performed	Satisfactory	Positive	ASC-H	High-grade squamous	
P10716	2023	2023-Q2	2023-06	Post-pandemic recovery	Cytology (Pap)	35-39	39	Not performed	Satisfactory	NILM	NILM	Negative (NILM)	
P10717	2023	2023-Q2	2023-06	Post-pandemic recovery	Cytology (Pap)	35-39	35	Not performed	Satisfactory	NILM	NILM	Negative (NILM)	
P10718	2023	2023-Q2	2023-06	Post-pandemic recovery	Cytology (Pap)	45-49	47	Not performed	Satisfactory	NILM	NILM	Negative (NILM)	

patient_id	year	quarter	month	period	primary_test	age_group	age_years	hpv_result	adequacy	result	bethesda	grade	infection
P10719	2023	2023-Q2	2023-06	Post-pandemic recovery	Cytology (Pap)	40-44	44	Not performed	Satisfactory	NILM	NILM	Negative (NILM)	
P10720	2023	2023-Q2	2023-06	Post-pandemic recovery	Cytology (Pap)	40-44	41	Not performed	Satisfactory	NILM	NILM	Negative (NILM)	
P10721	2023	2023-Q2	2023-06	Post-pandemic recovery	Cytology (Pap)	35-39	39	Not performed	Satisfactory	NILM	NILM	Negative (NILM)	
P10722	2023	2023-Q2	2023-06	Post-pandemic recovery	Cytology (Pap)	30-34	34	Not performed	Satisfactory	NILM	NILM	Negative (NILM)	
P10723	2023	2023-Q2	2023-06	Post-pandemic recovery	Cytology (Pap)	55-59	59	Not performed	Unsatisfactory	Unsatisfactory	Unsatisfactory	Unsatisfactory	
P10724	2023	2023-Q2	2023-06	Post-pandemic recovery	Cytology (Pap)	55-59	58	Not performed	Satisfactory	Positive	HSIL	High-grade squamous	
P10725	2023	2023-Q2	2023-06	Post-pandemic recovery	Cytology (Pap)	40-44	42	Not performed	Satisfactory	NILM	NILM	Negative (NILM)	
P10726	2023	2023-Q2	2023-06	Post-pandemic recovery	Cytology (Pap)	60-64	61	Not performed	Satisfactory	Positive	ASC-US	Low-grade / equivocal squamous	
P10727	2023	2023-Q2	2023-06	Post-pandemic recovery	Cytology (Pap)	35-39	35	Not performed	Satisfactory	NILM	NILM	Negative (NILM)	
P10728	2023	2023-Q2	2023-06	Post-pandemic recovery	Cytology (Pap)	45-49	47	Not performed	Satisfactory	NILM	NILM	Negative (NILM)	
P10729	2023	2023-Q2	2023-06	Post-pandemic recovery	Cytology (Pap)	40-44	44	Not performed	Satisfactory	NILM	NILM	Negative (NILM)	
P10730	2023	2023-Q2	2023-06	Post-pandemic recovery	Cytology (Pap)	35-39	38	Not performed	Satisfactory	NILM	NILM	Negative (NILM)	Other
P10731	2023	2023-Q2	2023-06	Post-pandemic recovery	Cytology (Pap)	30-34	30	Not performed	Satisfactory	NILM	NILM	Negative (NILM)	Trichomonas
P10732	2023	2023-Q2	2023-06	Post-pandemic recovery	Cytology (Pap)	35-39	35	Not performed	Satisfactory	NILM	NILM	Negative (NILM)	
P10733	2023	2023-Q2	2023-06	Post-pandemic recovery	Cytology (Pap)	55-59	58	Not performed	Satisfactory	NILM	NILM	Negative (NILM)	
P10734	2023	2023-Q2	2023-06	Post-pandemic recovery	Cytology (Pap)	55-59	57	Not performed	Satisfactory	NILM	NILM	Negative (NILM)	
P10735	2023	2023-Q2	2023-06	Post-pandemic recovery	Cytology (Pap)	50-54	52	Not performed	Satisfactory	Positive	LSIL	Low-grade / equivocal squamous	Candida
P10736	2023	2023-Q2	2023-06	Post-pandemic recovery	Cytology (Pap)	45-49	46	Not performed	Satisfactory	NILM	NILM	Negative (NILM)	
P10737	2023	2023-Q2	2023-06	Post-pandemic recovery	Cytology (Pap)	30-34	31	Not performed	Satisfactory	NILM	NILM	Negative (NILM)	
P10738	2023	2023-Q2	2023-06	Post-pandemic recovery	Cytology (Pap)	25-29	25	Not performed	Satisfactory	NILM	NILM	Negative (NILM)	
P10739	2023	2023-Q2	2023-06	Post-pandemic recovery	Cytology (Pap)	55-59	59	Not performed	Satisfactory	NILM	NILM	Negative (NILM)	
P10740	2023	2023-Q2	2023-06	Post-pandemic recovery	Cytology (Pap)	45-49	47	Not performed	Satisfactory	NILM	NILM	Negative (NILM)	
P10741	2023	2023-Q2	2023-06	Post-pandemic recovery	Cytology (Pap)	40-44	42	Not performed	Satisfactory	NILM	NILM	Negative (NILM)	
P10742	2023	2023-Q2	2023-06	Post-pandemic recovery	Cytology (Pap)	35-39	37	Not performed	Satisfactory	NILM	NILM	Negative (NILM)	Trichomonas
P10743	2023	2023-Q2	2023-06	Post-pandemic recovery	Cytology (Pap)	55-59	58	Not performed	Satisfactory	NILM	NILM	Negative (NILM)	
P10744	2023	2023-Q2	2023-06	Post-pandemic recovery	Cytology (Pap)	40-44	44	Not performed	Satisfactory	NILM	NILM	Negative (NILM)	
P10745	2023	2023-Q2	2023-06	Post-pandemic recovery	Cytology (Pap)	30-34	30	Not performed	Satisfactory	NILM	NILM	Negative (NILM)	Candida
P10746	2023	2023-Q2	2023-06	Post-pandemic recovery	Cytology (Pap)	45-49	47	Not performed	Satisfactory	NILM	NILM	Negative (NILM)	
P10747	2023	2023-Q2	2023-06	Post-pandemic recovery	Cytology (Pap)	35-39	37	Not performed	Satisfactory	NILM	NILM	Negative (NILM)	
P10748	2023	2023-Q2	2023-06	Post-pandemic recovery	Cytology (Pap)	45-49	49	Not performed	Satisfactory	NILM	NILM	Negative (NILM)	
P10749	2023	2023-Q2	2023-06	Post-pandemic recovery	Cytology (Pap)	25-29	26	Not performed	Satisfactory	NILM	NILM	Negative (NILM)	
P10750	2023	2023-Q2	2023-06	Post-pandemic recovery	Cytology (Pap)	35-39	35	Not performed	Satisfactory	NILM	NILM	Negative (NILM)	
P10751	2023	2023-Q2	2023-06	Post-pandemic recovery	Cytology (Pap)	60-64	64	Not performed	Satisfactory	NILM	NILM	Negative (NILM)	
P10752	2023	2023-Q2	2023-06	Post-pandemic recovery	Cytology (Pap)	40-44	42	Not performed	Satisfactory	NILM	NILM	Negative (NILM)	
P10753	2023	2023-Q2	2023-06	Post-pandemic recovery	Cytology (Pap)	40-44	42	Not performed	Satisfactory	NILM	NILM	Negative (NILM)	
P10754	2023	2023-Q2	2023-06	Post-pandemic recovery	Cytology (Pap)	35-39	35	Not performed	Unsatisfactory	Unsatisfactory	Unsatisfactory	Unsatisfactory	
P10755	2023	2023-Q2	2023-06	Post-pandemic recovery	Cytology (Pap)	30-34	31	Not performed	Satisfactory	NILM	NILM	Negative (NILM)	
P10756	2023	2023-Q2	2023-06	Post-pandemic recovery	Cytology (Pap)	60-64	64	Not performed	Satisfactory	Positive	LSIL	Low-grade / equivocal squamous	
P10757	2023	2023-Q2	2023-06	Post-pandemic recovery	Cytology (Pap)	25-29	28	Not performed	Satisfactory	NILM	NILM	Negative (NILM)	
P10758	2023	2023-Q2	2023-06	Post-pandemic recovery	Cytology (Pap)	45-49	47	Not performed	Satisfactory	NILM	NILM	Negative (NILM)	
P10759	2023	2023-Q2	2023-06	Post-pandemic recovery	Cytology (Pap)	55-59	57	Not performed	Satisfactory	Positive	AGC	Glandular	Trichomonas
P10760	2023	2023-Q2	2023-06	Post-pandemic recovery	Cytology (Pap)	45-49	49	Not performed	Satisfactory	NILM	NILM	Negative (NILM)	Trichomonas
P10761	2023	2023-Q2	2023-06	Post-pandemic recovery	Cytology (Pap)	30-34	34	Not performed	Satisfactory	NILM	NILM	Negative (NILM)	Candida
P10762	2023	2023-Q2	2023-06	Post-pandemic recovery	Cytology (Pap)	35-39	36	Not performed	Satisfactory	NILM	NILM	Negative (NILM)	
P10763	2023	2023-Q2	2023-06	Post-pandemic recovery	Cytology (Pap)	35-39	38	Not performed	Satisfactory	NILM	NILM	Negative (NILM)	
P10764	2023	2023-Q2	2023-06	Post-pandemic recovery	Cytology (Pap)	30-34	30	Not performed	Satisfactory	NILM	NILM	Negative (NILM)	
P10765	2023	2023-Q2	2023-06	Post-pandemic recovery	Cytology (Pap)	40-44	40	Not performed	Satisfactory	NILM	NILM	Negative (NILM)	
P10766	2023	2023-Q2	2023-06	Post-pandemic recovery	Cytology (Pap)	40-44	44	Not performed	Satisfactory	NILM	NILM	Negative (NILM)	Candida
P10767	2023	2023-Q2	2023-06	Post-pandemic recovery	Cytology (Pap)	40-44	43	Not performed	Satisfactory	NILM	NILM	Negative (NILM)	
P10768	2023	2023-Q2	2023-06	Post-pandemic recovery	Cytology (Pap)	40-44	43	Not performed	Satisfactory	NILM	NILM	Negative (NILM)	
P10769	2023	2023-Q2	2023-06	Post-pandemic recovery	Cytology (Pap)	35-39	35	Not performed	Satisfactory	NILM	NILM	Negative (NILM)	
P10770	2023	2023-Q2	2023-06	Post-pandemic recovery	Cytology (Pap)	40-44	40	Not performed	Satisfactory	NILM	NILM	Negative (NILM)	
P10771	2023	2023-Q2	2023-06	Post-pandemic recovery	Cytology (Pap)	30-34	33	Not performed	Satisfactory	Positive	LSIL	Low-grade / equivocal squamous	
P10772	2023	2023-Q2	2023-06	Post-pandemic recovery	Cytology (Pap)	35-39	35	Not performed	Satisfactory	NILM	NILM	Negative (NILM)	
P10773	2023	2023-Q2	2023-06	Post-pandemic recovery	Cytology (Pap)	40-44	42	Not performed	Satisfactory	NILM	NILM	Negative (NILM)	
P10774	2023	2023-Q2	2023-06	Post-pandemic recovery	Cytology (Pap)	35-39	35	Not performed	Satisfactory	NILM	NILM	Negative (NILM)	
P10775	2023	2023-Q2	2023-06	Post-pandemic recovery	Cytology (Pap)	35-39	38	Not performed	Unsatisfactory	Unsatisfactory	Unsatisfactory	Unsatisfactory	
P10776	2023	2023-Q2	2023-06	Post-pandemic recovery	Cytology (Pap)	40-44	44	Not performed	Satisfactory	NILM	NILM	Negative (NILM)	
P10777	2023	2023-Q2	2023-06	Post-pandemic recovery	Cytology (Pap)	30-34	33	Not performed	Satisfactory	NILM	NILM	Negative (NILM)	
P10778	2023	2023-Q2	2023-06	Post-pandemic recovery	Cytology (Pap)	40-44	42	Not performed	Satisfactory	NILM	NILM	Negative (NILM)	
P10779	2023	2023-Q2	2023-06	Post-pandemic recovery	Cytology (Pap)	60-64	63	Not performed	Satisfactory	Positive	HSIL	High-grade squamous	
P10780	2023	2023-Q2	2023-06	Post-pandemic recovery	Cytology (Pap)	35-39	38	Not performed	Satisfactory	NILM	NILM	Negative (NILM)	Trichomonas
P10781	2023	2023-Q2	2023-06	Post-pandemic recovery	Cytology (Pap)	55-59	59	Not performed	Satisfactory	NILM	NILM	Negative (NILM)	
P10782	2023	2023-Q2	2023-06	Post-pandemic recovery	Cytology (Pap)	55-59	55	Not performed	Satisfactory	NILM	NILM	Negative (NILM)	
P10783	2023	2023-Q2	2023-06	Post-pandemic recovery	Cytology (Pap)	25-29	25	Not performed	Satisfactory	NILM	NILM	Negative (NILM)	
P10784	2023	2023-Q2	2023-06	Post-pandemic recovery	Cytology (Pap)	55-59	57	Not performed	Satisfactory	NILM	NILM	Negative (NILM)	
P10785	2023	2023-Q2	2023-06	Post-pandemic recovery	Cytology (Pap)	60-64	64	Not performed	Satisfactory	NILM	NILM	Negative (NILM)	
P10786	2023	2023-Q2	2023-06	Post-pandemic recovery	Cytology (Pap)	60-64	62	Not performed	Satisfactory	NILM	NILM	Negative (NILM)	
P10787	2023	2023-Q2	2023-06	Post-pandemic recovery	Cytology (Pap)	35-39	37	Not performed	Satisfactory	NILM	NILM	Negative (NILM)	
P10788	2023	2023-Q2	2023-06	Post-pandemic recovery	Cytology (Pap)	25-29	29	Not performed	Satisfactory	NILM	NILM	Negative (NILM)	
P10789	2023	2023-Q2	2023-06	Post-pandemic recovery	Cytology (Pap)	40-44	43	Not performed	Satisfactory	NILM	NILM	Negative (NILM)	
P10790	2023	2023-Q2	2023-06	Post-pandemic recovery	Cytology (Pap)	25-29	26	Not performed	Satisfactory	Positive	HSIL	High-grade squamous	
P10791	2023	2023-Q2	2023-06	Post-pandemic recovery	Cytology (Pap)	30-34	30	Not performed	Satisfactory	NILM	NILM	Negative (NILM)	
P10792	2023	2023-Q2	2023-06	Post-pandemic recovery	Cytology (Pap)	25-29	28	Not performed	Satisfactory	NILM	NILM	Negative (NILM)	
P10793	2023	2023-Q2	2023-06	Post-pandemic recovery	Cytology (Pap)	35-39	38	Not performed	Satisfactory	NILM	NILM	Negative (NILM)	
P10794	2023	2023-Q2	2023-06	Post-pandemic recovery	Cytology (Pap)	25-29	27	Not performed	Satisfactory	Positive	HSIL	High-grade squamous	
P10795	2023	2023-Q2	2023-06	Post-pandemic recovery	Cytology (Pap)	30-34	32	Not performed	Satisfactory	NILM	NILM	Negative (NILM)	
P10796	2023	2023-Q2	2023-06	Post-pandemic recovery	Cytology (Pap)	30-34	30	Not performed	Satisfactory	NILM	NILM	Negative (NILM)	
P10797	2023	2023-Q2	2023-06	Post-pandemic recovery	Cytology (Pap)	30-34	32	Not performed	Satisfactory	NILM	NILM	Negative (NILM)	
P10798	2023	2023-Q2	2023-06	Post-pandemic recovery	Cytology (Pap)	55-59	55	Not performed	Satisfactory	Positive	HSIL	High-grade squamous	

patient_id	year	quarter	month	period	primary_test	age_group	age_years	hpv_result	adequacy	result	bethesda	grade	infection
P10799	2023	2023-Q2	2023-06	Post-pandemic recovery	Cytology (Pap)	45-49	49	Not performed	Satisfactory	NILM	NILM	Negative (NILM)	
P10800	2023	2023-Q2	2023-06	Post-pandemic recovery	Cytology (Pap)	30-34	32	Not performed	Unsatisfactory	Unsatisfactory	Unsatisfactory	Unsatisfactory	
P10801	2023	2023-Q2	2023-06	Post-pandemic recovery	Cytology (Pap)	45-49	46	Not performed	Satisfactory	NILM	NILM	Negative (NILM)	
P10802	2023	2023-Q2	2023-06	Post-pandemic recovery	Cytology (Pap)	50-54	51	Not performed	Satisfactory	NILM	NILM	Negative (NILM)	
P10803	2023	2023-Q2	2023-06	Post-pandemic recovery	Cytology (Pap)	30-34	33	Not performed	Satisfactory	NILM	NILM	Negative (NILM)	
P10804	2023	2023-Q2	2023-06	Post-pandemic recovery	Cytology (Pap)	25-29	26	Not performed	Satisfactory	NILM	NILM	Negative (NILM)	
P10805	2023	2023-Q2	2023-06	Post-pandemic recovery	Cytology (Pap)	30-34	32	Not performed	Satisfactory	NILM	NILM	Negative (NILM)	
P10806	2023	2023-Q2	2023-06	Post-pandemic recovery	Cytology (Pap)	25-29	27	Not performed	Satisfactory	NILM	NILM	Negative (NILM)	
P10807	2023	2023-Q2	2023-06	Post-pandemic recovery	Cytology (Pap)	25-29	26	Not performed	Satisfactory	NILM	NILM	Negative (NILM)	
P10808	2023	2023-Q2	2023-06	Post-pandemic recovery	Cytology (Pap)	40-44	41	Not performed	Satisfactory	NILM	NILM	Negative (NILM)	
P10809	2023	2023-Q2	2023-06	Post-pandemic recovery	Cytology (Pap)	45-49	47	Not performed	Satisfactory	Positive	ASC-US	Low-grade / equivocal squamous	
P10810	2023	2023-Q2	2023-06	Post-pandemic recovery	Cytology (Pap)	35-39	37	Not performed	Satisfactory	NILM	NILM	Negative (NILM)	
P10811	2023	2023-Q2	2023-06	Post-pandemic recovery	Cytology (Pap)	50-54	54	Not performed	Satisfactory	NILM	NILM	Negative (NILM)	
P10812	2023	2023-Q2	2023-06	Post-pandemic recovery	Cytology (Pap)	30-34	32	Not performed	Satisfactory	NILM	NILM	Negative (NILM)	
P10813	2023	2023-Q2	2023-06	Post-pandemic recovery	Cytology (Pap)	35-39	35	Not performed	Satisfactory	NILM	NILM	Negative (NILM)	
P10814	2023	2023-Q2	2023-06	Post-pandemic recovery	Cytology (Pap)	40-44	44	Not performed	Satisfactory	NILM	NILM	Negative (NILM)	Candida
P10815	2023	2023-Q2	2023-06	Post-pandemic recovery	Cytology (Pap)	30-34	32	Not performed	Satisfactory	NILM	NILM	Negative (NILM)	
P10816	2023	2023-Q2	2023-06	Post-pandemic recovery	Cytology (Pap)	35-39	39	Not performed	Satisfactory	NILM	NILM	Negative (NILM)	
P10817	2023	2023-Q2	2023-06	Post-pandemic recovery	Cytology (Pap)	25-29	25	Not performed	Satisfactory	NILM	NILM	Negative (NILM)	
P10818	2023	2023-Q2	2023-06	Post-pandemic recovery	Cytology (Pap)	30-34	34	Not performed	Satisfactory	NILM	NILM	Negative (NILM)	
P10819	2023	2023-Q2	2023-06	Post-pandemic recovery	Cytology (Pap)	25-29	29	Not performed	Satisfactory	NILM	NILM	Negative (NILM)	
P10820	2023	2023-Q2	2023-06	Post-pandemic recovery	Cytology (Pap)	30-34	31	Not performed	Satisfactory	NILM	NILM	Negative (NILM)	
P10821	2023	2023-Q2	2023-06	Post-pandemic recovery	Cytology (Pap)	25-29	29	Not performed	Unsatisfactory	Unsatisfactory	Unsatisfactory	Unsatisfactory	
P10822	2023	2023-Q2	2023-06	Post-pandemic recovery	Cytology (Pap)	30-34	31	Not performed	Satisfactory	NILM	NILM	Negative (NILM)	Candida
P10823	2023	2023-Q2	2023-06	Post-pandemic recovery	Cytology (Pap)	25-29	25	Not performed	Satisfactory	Positive	ASC-US	Low-grade / equivocal squamous	
P10824	2023	2023-Q2	2023-06	Post-pandemic recovery	Cytology (Pap)	25-29	28	Not performed	Satisfactory	NILM	NILM	Negative (NILM)	
P10825	2023	2023-Q2	2023-06	Post-pandemic recovery	Cytology (Pap)	35-39	37	Not performed	Satisfactory	NILM	NILM	Negative (NILM)	
P10826	2023	2023-Q2	2023-06	Post-pandemic recovery	Cytology (Pap)	30-34	30	Not performed	Satisfactory	NILM	NILM	Negative (NILM)	
P10827	2023	2023-Q2	2023-06	Post-pandemic recovery	Cytology (Pap)	45-49	47	Not performed	Satisfactory	NILM	NILM	Negative (NILM)	
P10828	2023	2023-Q2	2023-06	Post-pandemic recovery	Cytology (Pap)	35-39	37	Not performed	Satisfactory	NILM	NILM	Negative (NILM)	
P10829	2023	2023-Q2	2023-06	Post-pandemic recovery	Cytology (Pap)	45-49	47	Not performed	Satisfactory	NILM	NILM	Negative (NILM)	
P10830	2023	2023-Q2	2023-06	Post-pandemic recovery	Cytology (Pap)	45-49	48	Not performed	Satisfactory	NILM	NILM	Negative (NILM)	
P10831	2023	2023-Q2	2023-06	Post-pandemic recovery	Cytology (Pap)	30-34	32	Not performed	Satisfactory	NILM	NILM	Negative (NILM)	
P10832	2023	2023-Q2	2023-06	Post-pandemic recovery	Cytology (Pap)	40-44	40	Not performed	Satisfactory	NILM	NILM	Negative (NILM)	
P10833	2023	2023-Q2	2023-06	Post-pandemic recovery	Cytology (Pap)	55-59	58	Not performed	Satisfactory	NILM	NILM	Negative (NILM)	Candida
P10834	2023	2023-Q2	2023-06	Post-pandemic recovery	Cytology (Pap)	50-54	51	Not performed	Satisfactory	NILM	NILM	Negative (NILM)	
P10835	2023	2023-Q2	2023-06	Post-pandemic recovery	Cytology (Pap)	30-34	31	Not performed	Satisfactory	NILM	NILM	Negative (NILM)	
P10836	2023	2023-Q2	2023-06	Post-pandemic recovery	Cytology (Pap)	50-54	50	Not performed	Satisfactory	NILM	NILM	Negative (NILM)	
P10837	2023	2023-Q2	2023-06	Post-pandemic recovery	Cytology (Pap)	60-64	61	Not performed	Satisfactory	Positive	LSIL	Low-grade / equivocal squamous	
P10838	2023	2023-Q2	2023-06	Post-pandemic recovery	Cytology (Pap)	35-39	39	Not performed	Satisfactory	NILM	NILM	Negative (NILM)	
P10839	2023	2023-Q2	2023-06	Post-pandemic recovery	Cytology (Pap)	40-44	41	Not performed	Satisfactory	NILM	NILM	Negative (NILM)	
P10840	2023	2023-Q2	2023-06	Post-pandemic recovery	Cytology (Pap)	50-54	53	Not performed	Satisfactory	NILM	NILM	Negative (NILM)	
P10841	2023	2023-Q2	2023-06	Post-pandemic recovery	Cytology (Pap)	40-44	42	Not performed	Satisfactory	NILM	NILM	Negative (NILM)	
P10842	2023	2023-Q2	2023-06	Post-pandemic recovery	Cytology (Pap)	50-54	53	Not performed	Satisfactory	NILM	NILM	Negative (NILM)	
P10843	2023	2023-Q2	2023-06	Post-pandemic recovery	Cytology (Pap)	40-44	41	Not performed	Satisfactory	NILM	NILM	Negative (NILM)	
P10844	2023	2023-Q2	2023-06	Post-pandemic recovery	Cytology (Pap)	25-29	29	Not performed	Satisfactory	NILM	NILM	Negative (NILM)	
P10845	2023	2023-Q2	2023-06	Post-pandemic recovery	Cytology (Pap)	35-39	38	Not performed	Satisfactory	NILM	NILM	Negative (NILM)	
P10846	2023	2023-Q2	2023-06	Post-pandemic recovery	Cytology (Pap)	55-59	58	Not performed	Satisfactory	NILM	NILM	Negative (NILM)	
P10847	2023	2023-Q2	2023-06	Post-pandemic recovery	Cytology (Pap)	40-44	42	Not performed	Satisfactory	NILM	NILM	Negative (NILM)	
P10848	2023	2023-Q2	2023-06	Post-pandemic recovery	Cytology (Pap)	35-39	37	Not performed	Satisfactory	Positive	ASC-US	Low-grade / equivocal squamous	
P10849	2023	2023-Q2	2023-06	Post-pandemic recovery	Cytology (Pap)	40-44	40	Not performed	Satisfactory	Positive	LSIL	Low-grade / equivocal squamous	
P10850	2023	2023-Q2	2023-06	Post-pandemic recovery	Cytology (Pap)	35-39	37	Not performed	Satisfactory	NILM	NILM	Negative (NILM)	Candida
P10851	2023	2023-Q2	2023-06	Post-pandemic recovery	Cytology (Pap)	60-64	60	Not performed	Satisfactory	NILM	NILM	Negative (NILM)	
P10852	2023	2023-Q2	2023-06	Post-pandemic recovery	Cytology (Pap)	55-59	59	Not performed	Satisfactory	NILM	NILM	Negative (NILM)	
P10853	2023	2023-Q2	2023-06	Post-pandemic recovery	Cytology (Pap)	45-49	45	Not performed	Satisfactory	NILM	NILM	Negative (NILM)	
P10854	2023	2023-Q2	2023-06	Post-pandemic recovery	Cytology (Pap)	50-54	50	Not performed	Unsatisfactory	Unsatisfactory	Unsatisfactory	Unsatisfactory	
P10855	2023	2023-Q2	2023-06	Post-pandemic recovery	Cytology (Pap)	60-64	63	Not performed	Satisfactory	NILM	NILM	Negative (NILM)	
P10856	2023	2023-Q2	2023-06	Post-pandemic recovery	Cytology (Pap)	30-34	34	Not performed	Satisfactory	NILM	NILM	Negative (NILM)	Other
P10857	2023	2023-Q2	2023-06	Post-pandemic recovery	Cytology (Pap)	35-39	39	Not performed	Satisfactory	NILM	NILM	Negative (NILM)	
P10858	2023	2023-Q2	2023-06	Post-pandemic recovery	Cytology (Pap)	60-64	60	Not performed	Satisfactory	Positive	ASC-US	Low-grade / equivocal squamous	
P10859	2023	2023-Q2	2023-06	Post-pandemic recovery	Cytology (Pap)	50-54	53	Not performed	Satisfactory	NILM	NILM	Negative (NILM)	
P10860	2023	2023-Q2	2023-06	Post-pandemic recovery	Cytology (Pap)	30-34	30	Not performed	Satisfactory	NILM	NILM	Negative (NILM)	
P10861	2023	2023-Q2	2023-06	Post-pandemic recovery	Cytology (Pap)	40-44	43	Not performed	Satisfactory	NILM	NILM	Negative (NILM)	Trichomonas
P10862	2023	2023-Q2	2023-06	Post-pandemic recovery	Cytology (Pap)	40-44	42	Not performed	Satisfactory	NILM	NILM	Negative (NILM)	
P10863	2023	2023-Q2	2023-06	Post-pandemic recovery	Cytology (Pap)	25-29	26	Not performed	Satisfactory	NILM	NILM	Negative (NILM)	
P10864	2023	2023-Q2	2023-06	Post-pandemic recovery	Cytology (Pap)	25-29	29	Not performed	Satisfactory	NILM	NILM	Negative (NILM)	
P10865	2023	2023-Q2	2023-06	Post-pandemic recovery	Cytology (Pap)	30-34	32	Not performed	Satisfactory	NILM	NILM	Negative (NILM)	
P10866	2023	2023-Q2	2023-06	Post-pandemic recovery	Cytology (Pap)	40-44	41	Not performed	Satisfactory	NILM	NILM	Negative (NILM)	
P10867	2023	2023-Q2	2023-06	Post-pandemic recovery	Cytology (Pap)	35-39	37	Not performed	Satisfactory	Positive	ASC-US	Low-grade / equivocal squamous	
P10868	2023	2023-Q2	2023-06	Post-pandemic recovery	Cytology (Pap)	55-59	56	Not performed	Satisfactory	NILM	NILM	Negative (NILM)	
P10869	2023	2023-Q2	2023-06	Post-pandemic recovery	Cytology (Pap)	55-59	58	Not performed	Satisfactory	Positive	LSIL	Low-grade / equivocal squamous	
P10870	2023	2023-Q2	2023-06	Post-pandemic recovery	Cytology (Pap)	50-54	54	Not performed	Satisfactory	NILM	NILM	Negative (NILM)	Gardnerella
P10871	2023	2023-Q2	2023-06	Post-pandemic recovery	Cytology (Pap)	40-44	40	Not performed	Satisfactory	Positive	ASC-US	Low-grade / equivocal squamous	Candida
P10872	2023	2023-Q2	2023-06	Post-pandemic recovery	Cytology (Pap)	25-29	28	Not performed	Satisfactory	NILM	NILM	Negative (NILM)	
P10873	2023	2023-Q2	2023-06	Post-pandemic recovery	Cytology (Pap)	30-34	32	Not performed	Satisfactory	NILM	NILM	Negative (NILM)	
P10874	2023	2023-Q2	2023-06	Post-pandemic recovery	Cytology (Pap)	25-29	28	Not performed	Satisfactory	Positive	ASC-US	Low-grade / equivocal squamous	
P10875	2023	2023-Q2	2023-06	Post-pandemic recovery	Cytology (Pap)	30-34	34	Not performed	Satisfactory	NILM	NILM	Negative (NILM)	
P10876	2023	2023-Q2	2023-06	Post-pandemic recovery	Cytology (Pap)	40-44	42	Not performed	Satisfactory	NILM	NILM	Negative (NILM)	
P10877	2023	2023-Q2	2023-06	Post-pandemic recovery	Cytology (Pap)	50-54	52	Not performed	Satisfactory	NILM	NILM	Negative (NILM)	
P10878	2023	2023-Q3	2023-07	Post-pandemic recovery	Cytology (Pap)	50-54	52	Not performed	Unsatisfactory	Unsatisfactory	Unsatisfactory	Unsatisfactory	

patient_id	year	quarter	month	period	primary_test	age_group	age_years	hpv_result	adequacy	result	bethesda	grade	infection
P10879	2023	2023-Q3	2023-07	Post-pandemic recovery	Cytology (Pap)	25-29	27	Not performed	Satisfactory	NILM	NILM	Negative (NILM)	
P10880	2023	2023-Q3	2023-07	Post-pandemic recovery	Cytology (Pap)	25-29	28	Not performed	Satisfactory	Positive	LSIL	Low-grade / equivocal squamous	
P10881	2023	2023-Q3	2023-07	Post-pandemic recovery	Cytology (Pap)	35-39	39	Not performed	Satisfactory	NILM	NILM	Negative (NILM)	
P10882	2023	2023-Q3	2023-07	Post-pandemic recovery	Cytology (Pap)	25-29	28	Not performed	Satisfactory	NILM	NILM	Negative (NILM)	
P10883	2023	2023-Q3	2023-07	Post-pandemic recovery	Cytology (Pap)	40-44	44	Not performed	Satisfactory	NILM	NILM	Negative (NILM)	
P10884	2023	2023-Q3	2023-07	Post-pandemic recovery	Cytology (Pap)	40-44	44	Not performed	Satisfactory	NILM	NILM	Negative (NILM)	
P10885	2023	2023-Q3	2023-07	Post-pandemic recovery	Cytology (Pap)	45-49	49	Not performed	Satisfactory	NILM	NILM	Negative (NILM)	
P10886	2023	2023-Q3	2023-07	Post-pandemic recovery	Cytology (Pap)	40-44	44	Not performed	Unsatisfactory	Unsatisfactory	Unsatisfactory	Unsatisfactory	
P10887	2023	2023-Q3	2023-07	Post-pandemic recovery	Cytology (Pap)	60-64	64	Not performed	Satisfactory	NILM	NILM	Negative (NILM)	
P10888	2023	2023-Q3	2023-07	Post-pandemic recovery	Cytology (Pap)	40-44	43	Not performed	Satisfactory	NILM	NILM	Negative (NILM)	
P10889	2023	2023-Q3	2023-07	Post-pandemic recovery	Cytology (Pap)	50-54	51	Not performed	Satisfactory	NILM	NILM	Negative (NILM)	
P10890	2023	2023-Q3	2023-07	Post-pandemic recovery	Cytology (Pap)	30-34	31	Not performed	Satisfactory	NILM	NILM	Negative (NILM)	
P10891	2023	2023-Q3	2023-07	Post-pandemic recovery	Cytology (Pap)	50-54	52	Not performed	Satisfactory	NILM	NILM	Negative (NILM)	
P10892	2023	2023-Q3	2023-07	Post-pandemic recovery	Cytology (Pap)	40-44	40	Not performed	Satisfactory	NILM	NILM	Negative (NILM)	
P10893	2023	2023-Q3	2023-07	Post-pandemic recovery	Cytology (Pap)	30-34	33	Not performed	Satisfactory	NILM	NILM	Negative (NILM)	
P10894	2023	2023-Q3	2023-07	Post-pandemic recovery	Cytology (Pap)	40-44	44	Not performed	Satisfactory	NILM	NILM	Negative (NILM)	
P10895	2023	2023-Q3	2023-07	Post-pandemic recovery	Cytology (Pap)	40-44	42	Not performed	Satisfactory	Positive	ASC-US	Low-grade / equivocal squamous	
P10896	2023	2023-Q3	2023-07	Post-pandemic recovery	Cytology (Pap)	55-59	58	Not performed	Satisfactory	NILM	NILM	Negative (NILM)	Trichomonas
P10897	2023	2023-Q3	2023-07	Post-pandemic recovery	Cytology (Pap)	45-49	46	Not performed	Satisfactory	NILM	NILM	Negative (NILM)	
P10898	2023	2023-Q3	2023-07	Post-pandemic recovery	Cytology (Pap)	35-39	35	Not performed	Satisfactory	Positive	ASC-H	High-grade squamous	
P10899	2023	2023-Q3	2023-07	Post-pandemic recovery	Cytology (Pap)	45-49	46	Not performed	Satisfactory	NILM	NILM	Negative (NILM)	Candida
P10900	2023	2023-Q3	2023-07	Post-pandemic recovery	Cytology (Pap)	40-44	41	Not performed	Satisfactory	NILM	NILM	Negative (NILM)	
P10901	2023	2023-Q3	2023-07	Post-pandemic recovery	Cytology (Pap)	30-34	33	Not performed	Satisfactory	Positive	ASC-US	Low-grade / equivocal squamous	
P10902	2023	2023-Q3	2023-07	Post-pandemic recovery	Cytology (Pap)	50-54	50	Not performed	Unsatisfactory	Unsatisfactory	Unsatisfactory	Unsatisfactory	
P10903	2023	2023-Q3	2023-07	Post-pandemic recovery	Cytology (Pap)	30-34	31	Not performed	Satisfactory	Positive	LSIL	Low-grade / equivocal squamous	
P10904	2023	2023-Q3	2023-07	Post-pandemic recovery	Cytology (Pap)	30-34	31	Not performed	Satisfactory	NILM	NILM	Negative (NILM)	
P10905	2023	2023-Q3	2023-07	Post-pandemic recovery	Cytology (Pap)	50-54	53	Not performed	Satisfactory	NILM	NILM	Negative (NILM)	
P10906	2023	2023-Q3	2023-07	Post-pandemic recovery	Cytology (Pap)	45-49	48	Not performed	Satisfactory	NILM	NILM	Negative (NILM)	
P10907	2023	2023-Q3	2023-07	Post-pandemic recovery	Cytology (Pap)	50-54	54	Not performed	Satisfactory	NILM	NILM	Negative (NILM)	
P10908	2023	2023-Q3	2023-07	Post-pandemic recovery	Cytology (Pap)	35-39	36	Not performed	Satisfactory	NILM	NILM	Negative (NILM)	
P10909	2023	2023-Q3	2023-07	Post-pandemic recovery	Cytology (Pap)	35-39	38	Not performed	Satisfactory	Positive	HSIL	High-grade squamous	
P10910	2023	2023-Q3	2023-07	Post-pandemic recovery	Cytology (Pap)	45-49	45	Not performed	Satisfactory	Positive	HSIL	High-grade squamous	
P10911	2023	2023-Q3	2023-07	Post-pandemic recovery	Cytology (Pap)	45-49	48	Not performed	Satisfactory	NILM	NILM	Negative (NILM)	
P10912	2023	2023-Q3	2023-07	Post-pandemic recovery	Cytology (Pap)	40-44	40	Not performed	Satisfactory	NILM	NILM	Negative (NILM)	
P10913	2023	2023-Q3	2023-07	Post-pandemic recovery	Cytology (Pap)	40-44	40	Not performed	Satisfactory	Positive	ASC-US	Low-grade / equivocal squamous	
P10914	2023	2023-Q3	2023-07	Post-pandemic recovery	Cytology (Pap)	40-44	43	Not performed	Satisfactory	NILM	NILM	Negative (NILM)	
P10915	2023	2023-Q3	2023-07	Post-pandemic recovery	Cytology (Pap)	50-54	50	Not performed	Satisfactory	NILM	NILM	Negative (NILM)	
P10916	2023	2023-Q3	2023-07	Post-pandemic recovery	Cytology (Pap)	35-39	35	Not performed	Satisfactory	NILM	NILM	Negative (NILM)	
P10917	2023	2023-Q3	2023-07	Post-pandemic recovery	Cytology (Pap)	30-34	30	Not performed	Satisfactory	NILM	NILM	Negative (NILM)	
P10918	2023	2023-Q3	2023-07	Post-pandemic recovery	Cytology (Pap)	25-29	27	Not performed	Satisfactory	Positive	ASC-H	High-grade squamous	
P10919	2023	2023-Q3	2023-07	Post-pandemic recovery	Cytology (Pap)	40-44	43	Not performed	Satisfactory	NILM	NILM	Negative (NILM)	
P10920	2023	2023-Q3	2023-07	Post-pandemic recovery	Cytology (Pap)	50-54	52	Not performed	Satisfactory	NILM	NILM	Negative (NILM)	
P10921	2023	2023-Q3	2023-07	Post-pandemic recovery	Cytology (Pap)	25-29	25	Not performed	Satisfactory	NILM	NILM	Negative (NILM)	
P10922	2023	2023-Q3	2023-07	Post-pandemic recovery	Cytology (Pap)	30-34	32	Not performed	Satisfactory	NILM	NILM	Negative (NILM)	
P10923	2023	2023-Q3	2023-07	Post-pandemic recovery	Cytology (Pap)	45-49	49	Not performed	Satisfactory	NILM	NILM	Negative (NILM)	
P10924	2023	2023-Q3	2023-07	Post-pandemic recovery	Cytology (Pap)	45-49	47	Not performed	Satisfactory	NILM	NILM	Negative (NILM)	
P10925	2023	2023-Q3	2023-07	Post-pandemic recovery	Cytology (Pap)	45-49	49	Not performed	Satisfactory	NILM	NILM	Negative (NILM)	
P10926	2023	2023-Q3	2023-07	Post-pandemic recovery	Cytology (Pap)	55-59	55	Not performed	Satisfactory	Positive	ASC-US	Low-grade / equivocal squamous	
P10927	2023	2023-Q3	2023-07	Post-pandemic recovery	Cytology (Pap)	25-29	27	Not performed	Satisfactory	NILM	NILM	Negative (NILM)	
P10928	2023	2023-Q3	2023-07	Post-pandemic recovery	Cytology (Pap)	40-44	44	Not performed	Satisfactory	NILM	NILM	Negative (NILM)	
P10929	2023	2023-Q3	2023-07	Post-pandemic recovery	Cytology (Pap)	40-44	40	Not performed	Satisfactory	NILM	NILM	Negative (NILM)	
P10930	2023	2023-Q3	2023-07	Post-pandemic recovery	Cytology (Pap)	40-44	41	Not performed	Satisfactory	NILM	NILM	Negative (NILM)	
P10931	2023	2023-Q3	2023-07	Post-pandemic recovery	Cytology (Pap)	50-54	53	Not performed	Satisfactory	NILM	NILM	Negative (NILM)	
P10932	2023	2023-Q3	2023-07	Post-pandemic recovery	Cytology (Pap)	40-44	41	Not performed	Satisfactory	NILM	NILM	Negative (NILM)	
P10933	2023	2023-Q3	2023-07	Post-pandemic recovery	Cytology (Pap)	55-59	59	Not performed	Satisfactory	NILM	NILM	Negative (NILM)	
P10934	2023	2023-Q3	2023-07	Post-pandemic recovery	Cytology (Pap)	30-34	30	Not performed	Satisfactory	NILM	NILM	Negative (NILM)	
P10935	2023	2023-Q3	2023-07	Post-pandemic recovery	Cytology (Pap)	40-44	41	Not performed	Satisfactory	NILM	NILM	Negative (NILM)	
P10936	2023	2023-Q3	2023-07	Post-pandemic recovery	Cytology (Pap)	40-44	40	Not performed	Satisfactory	NILM	NILM	Negative (NILM)	
P10937	2023	2023-Q3	2023-07	Post-pandemic recovery	Cytology (Pap)	25-29	28	Not performed	Satisfactory	NILM	NILM	Negative (NILM)	
P10938	2023	2023-Q3	2023-07	Post-pandemic recovery	Cytology (Pap)	50-54	54	Not performed	Satisfactory	NILM	NILM	Negative (NILM)	
P10939	2023	2023-Q3	2023-07	Post-pandemic recovery	Cytology (Pap)	60-64	61	Not performed	Unsatisfactory	Unsatisfactory	Unsatisfactory	Unsatisfactory	
P10940	2023	2023-Q3	2023-07	Post-pandemic recovery	Cytology (Pap)	35-39	37	Not performed	Satisfactory	NILM	NILM	Negative (NILM)	
P10941	2023	2023-Q3	2023-07	Post-pandemic recovery	Cytology (Pap)	50-54	54	Not performed	Satisfactory	NILM	NILM	Negative (NILM)	Candida
P10942	2023	2023-Q3	2023-07	Post-pandemic recovery	Cytology (Pap)	40-44	40	Not performed	Satisfactory	NILM	NILM	Negative (NILM)	
P10943	2023	2023-Q3	2023-07	Post-pandemic recovery	Cytology (Pap)	45-49	48	Not performed	Satisfactory	NILM	NILM	Negative (NILM)	
P10944	2023	2023-Q3	2023-07	Post-pandemic recovery	Cytology (Pap)	40-44	43	Not performed	Satisfactory	NILM	NILM	Negative (NILM)	
P10945	2023	2023-Q3	2023-07	Post-pandemic recovery	Cytology (Pap)	25-29	25	Not performed	Satisfactory	Positive	ASC-US	Low-grade / equivocal squamous	
P10946	2023	2023-Q3	2023-07	Post-pandemic recovery	Cytology (Pap)	60-64	63	Not performed	Satisfactory	NILM	NILM	Negative (NILM)	Trichomonas
P10947	2023	2023-Q3	2023-07	Post-pandemic recovery	Cytology (Pap)	35-39	36	Not performed	Satisfactory	NILM	NILM	Negative (NILM)	
P10948	2023	2023-Q3	2023-07	Post-pandemic recovery	Cytology (Pap)	30-34	30	Not performed	Satisfactory	Positive	ASC-US	Low-grade / equivocal squamous	
P10949	2023	2023-Q3	2023-07	Post-pandemic recovery	Cytology (Pap)	30-34	34	Not performed	Satisfactory	NILM	NILM	Negative (NILM)	
P10950	2023	2023-Q3	2023-07	Post-pandemic recovery	Cytology (Pap)	60-64	62	Not performed	Satisfactory	NILM	NILM	Negative (NILM)	
P10951	2023	2023-Q3	2023-07	Post-pandemic recovery	Cytology (Pap)	40-44	40	Not performed	Satisfactory	NILM	NILM	Negative (NILM)	
P10952	2023	2023-Q3	2023-07	Post-pandemic recovery	Cytology (Pap)	40-44	41	Not performed	Satisfactory	NILM	NILM	Negative (NILM)	
P10953	2023	2023-Q3	2023-07	Post-pandemic recovery	Cytology (Pap)	35-39	38	Not performed	Satisfactory	Positive	LSIL	Low-grade / equivocal squamous	
P10954	2023	2023-Q3	2023-07	Post-pandemic recovery	Cytology (Pap)	45-49	47	Not performed	Satisfactory	NILM	NILM	Negative (NILM)	
P10955	2023	2023-Q3	2023-07	Post-pandemic recovery	Cytology (Pap)	50-54	50	Not performed	Satisfactory	NILM	NILM	Negative (NILM)	
P10956	2023	2023-Q3	2023-07	Post-pandemic recovery	Cytology (Pap)	30-34	31	Not performed	Satisfactory	Positive	ASC-US	Low-grade / equivocal squamous	
P10957	2023	2023-Q3	2023-07	Post-pandemic recovery	Cytology (Pap)	40-44	42	Not performed	Satisfactory	NILM	NILM	Negative (NILM)	
P10958	2023	2023-Q3	2023-07	Post-pandemic recovery	Cytology (Pap)	35-39	38	Not performed	Satisfactory	NILM	NILM	Negative (NILM)	

patient_id	year	quarter	month	period	primary_test	age_group	age_years	hpv_result	adequacy	result	bethesda	grade	infection
P10959	2023	2023-Q3	2023-07	Post-pandemic recovery	Cytology (Pap)	40-44	41	Not performed	Satisfactory	NILM	NILM	Negative (NILM)	
P10960	2023	2023-Q3	2023-07	Post-pandemic recovery	Cytology (Pap)	25-29	25	Not performed	Satisfactory	Positive	LSIL	Low-grade / equivocal squamous	
P10961	2023	2023-Q3	2023-07	Post-pandemic recovery	Cytology (Pap)	55-59	58	Not performed	Satisfactory	NILM	NILM	Negative (NILM)	
P10962	2023	2023-Q3	2023-07	Post-pandemic recovery	Cytology (Pap)	35-39	38	Not performed	Satisfactory	NILM	NILM	Negative (NILM)	
P10963	2023	2023-Q3	2023-07	Post-pandemic recovery	Cytology (Pap)	55-59	56	Not performed	Satisfactory	NILM	NILM	Negative (NILM)	
P10964	2023	2023-Q3	2023-07	Post-pandemic recovery	Cytology (Pap)	40-44	41	Not performed	Satisfactory	NILM	NILM	Negative (NILM)	
P10965	2023	2023-Q3	2023-07	Post-pandemic recovery	Cytology (Pap)	55-59	57	Not performed	Satisfactory	NILM	NILM	Negative (NILM)	
P10966	2023	2023-Q3	2023-07	Post-pandemic recovery	Cytology (Pap)	55-59	57	Not performed	Satisfactory	NILM	NILM	Negative (NILM)	
P10967	2023	2023-Q3	2023-07	Post-pandemic recovery	Cytology (Pap)	35-39	38	Not performed	Satisfactory	NILM	NILM	Negative (NILM)	
P10968	2023	2023-Q3	2023-07	Post-pandemic recovery	Cytology (Pap)	25-29	26	Not performed	Satisfactory	NILM	NILM	Negative (NILM)	
P10969	2023	2023-Q3	2023-07	Post-pandemic recovery	Cytology (Pap)	25-29	28	Not performed	Unsatisfactory	Unsatisfactory	Unsatisfactory	Unsatisfactory	
P10970	2023	2023-Q3	2023-07	Post-pandemic recovery	Cytology (Pap)	35-39	37	Not performed	Satisfactory	NILM	NILM	Negative (NILM)	
P10971	2023	2023-Q3	2023-07	Post-pandemic recovery	Cytology (Pap)	30-34	30	Not performed	Satisfactory	NILM	NILM	Negative (NILM)	
P10972	2023	2023-Q3	2023-07	Post-pandemic recovery	Cytology (Pap)	35-39	39	Not performed	Satisfactory	Positive	LSIL	Low-grade / equivocal squamous	Candida
P10973	2023	2023-Q3	2023-07	Post-pandemic recovery	Cytology (Pap)	25-29	28	Not performed	Satisfactory	NILM	NILM	Negative (NILM)	
P10974	2023	2023-Q3	2023-07	Post-pandemic recovery	Cytology (Pap)	25-29	29	Not performed	Satisfactory	NILM	NILM	Negative (NILM)	
P10975	2023	2023-Q3	2023-07	Post-pandemic recovery	Cytology (Pap)	40-44	44	Not performed	Satisfactory	NILM	NILM	Negative (NILM)	
P10976	2023	2023-Q3	2023-07	Post-pandemic recovery	Cytology (Pap)	40-44	41	Not performed	Satisfactory	NILM	NILM	Negative (NILM)	
P10977	2023	2023-Q3	2023-07	Post-pandemic recovery	Cytology (Pap)	30-34	33	Not performed	Satisfactory	NILM	NILM	Negative (NILM)	
P10978	2023	2023-Q3	2023-07	Post-pandemic recovery	Cytology (Pap)	50-54	54	Not performed	Satisfactory	NILM	NILM	Negative (NILM)	
P10979	2023	2023-Q3	2023-07	Post-pandemic recovery	Cytology (Pap)	55-59	58	Not performed	Satisfactory	NILM	NILM	Negative (NILM)	
P10980	2023	2023-Q3	2023-07	Post-pandemic recovery	Cytology (Pap)	40-44	41	Not performed	Satisfactory	NILM	NILM	Negative (NILM)	
P10981	2023	2023-Q3	2023-07	Post-pandemic recovery	Cytology (Pap)	60-64	64	Not performed	Satisfactory	NILM	NILM	Negative (NILM)	
P10982	2023	2023-Q3	2023-07	Post-pandemic recovery	Cytology (Pap)	40-44	42	Not performed	Satisfactory	NILM	NILM	Negative (NILM)	
P10983	2023	2023-Q3	2023-07	Post-pandemic recovery	Cytology (Pap)	45-49	47	Not performed	Satisfactory	NILM	NILM	Negative (NILM)	
P10984	2023	2023-Q3	2023-07	Post-pandemic recovery	Cytology (Pap)	40-44	43	Not performed	Satisfactory	NILM	NILM	Negative (NILM)	
P10985	2023	2023-Q3	2023-07	Post-pandemic recovery	Cytology (Pap)	30-34	34	Not performed	Satisfactory	NILM	NILM	Negative (NILM)	
P10986	2023	2023-Q3	2023-07	Post-pandemic recovery	Cytology (Pap)	40-44	40	Not performed	Satisfactory	NILM	NILM	Negative (NILM)	
P10987	2023	2023-Q3	2023-07	Post-pandemic recovery	Cytology (Pap)	40-44	44	Not performed	Satisfactory	NILM	NILM	Negative (NILM)	
P10988	2023	2023-Q3	2023-07	Post-pandemic recovery	Cytology (Pap)	60-64	61	Not performed	Satisfactory	Positive	ASC-US	Low-grade / equivocal squamous	
P10989	2023	2023-Q3	2023-07	Post-pandemic recovery	Cytology (Pap)	35-39	36	Not performed	Satisfactory	NILM	NILM	Negative (NILM)	
P10990	2023	2023-Q3	2023-07	Post-pandemic recovery	Cytology (Pap)	45-49	45	Not performed	Satisfactory	NILM	NILM	Negative (NILM)	
P10991	2023	2023-Q3	2023-07	Post-pandemic recovery	Cytology (Pap)	30-34	30	Not performed	Satisfactory	NILM	NILM	Negative (NILM)	
P10992	2023	2023-Q3	2023-07	Post-pandemic recovery	Cytology (Pap)	30-34	31	Not performed	Satisfactory	NILM	NILM	Negative (NILM)	
P10993	2023	2023-Q3	2023-07	Post-pandemic recovery	Cytology (Pap)	25-29	27	Not performed	Satisfactory	NILM	NILM	Negative (NILM)	
P10994	2023	2023-Q3	2023-07	Post-pandemic recovery	Cytology (Pap)	35-39	38	Not performed	Satisfactory	NILM	NILM	Negative (NILM)	
P10995	2023	2023-Q3	2023-07	Post-pandemic recovery	Cytology (Pap)	25-29	29	Not performed	Satisfactory	NILM	NILM	Negative (NILM)	
P10996	2023	2023-Q3	2023-07	Post-pandemic recovery	Cytology (Pap)	45-49	49	Not performed	Satisfactory	NILM	NILM	Negative (NILM)	
P10997	2023	2023-Q3	2023-07	Post-pandemic recovery	Cytology (Pap)	60-64	62	Not performed	Satisfactory	NILM	NILM	Negative (NILM)	
P10998	2023	2023-Q3	2023-07	Post-pandemic recovery	Cytology (Pap)	30-34	34	Not performed	Satisfactory	NILM	NILM	Negative (NILM)	
P10999	2023	2023-Q3	2023-07	Post-pandemic recovery	Cytology (Pap)	30-34	31	Not performed	Satisfactory	NILM	NILM	Negative (NILM)	
P11000	2023	2023-Q3	2023-07	Post-pandemic recovery	Cytology (Pap)	45-49	45	Not performed	Satisfactory	Positive	LSIL	Low-grade / equivocal squamous	
P11001	2023	2023-Q3	2023-07	Post-pandemic recovery	Cytology (Pap)	40-44	41	Not performed	Satisfactory	Positive	HSIL	High-grade squamous	
P11002	2023	2023-Q3	2023-07	Post-pandemic recovery	Cytology (Pap)	50-54	54	Not performed	Satisfactory	NILM	NILM	Negative (NILM)	
P11003	2023	2023-Q3	2023-07	Post-pandemic recovery	Cytology (Pap)	40-44	43	Not performed	Satisfactory	Positive	ASC-H	High-grade squamous	Trichomonas
P11004	2023	2023-Q3	2023-07	Post-pandemic recovery	Cytology (Pap)	55-59	55	Not performed	Satisfactory	NILM	NILM	Negative (NILM)	
P11005	2023	2023-Q3	2023-07	Post-pandemic recovery	Cytology (Pap)	55-59	59	Not performed	Satisfactory	NILM	NILM	Negative (NILM)	
P11006	2023	2023-Q3	2023-07	Post-pandemic recovery	Cytology (Pap)	40-44	44	Not performed	Satisfactory	NILM	NILM	Negative (NILM)	
P11007	2023	2023-Q3	2023-07	Post-pandemic recovery	Cytology (Pap)	40-44	44	Not performed	Satisfactory	NILM	NILM	Negative (NILM)	
P11008	2023	2023-Q3	2023-07	Post-pandemic recovery	Cytology (Pap)	40-44	42	Not performed	Satisfactory	NILM	NILM	Negative (NILM)	
P11009	2023	2023-Q3	2023-07	Post-pandemic recovery	Cytology (Pap)	55-59	59	Not performed	Satisfactory	Positive	HSIL	High-grade squamous	
P11010	2023	2023-Q3	2023-07	Post-pandemic recovery	Cytology (Pap)	40-44	40	Not performed	Satisfactory	NILM	NILM	Negative (NILM)	
P11011	2023	2023-Q3	2023-07	Post-pandemic recovery	Cytology (Pap)	50-54	53	Not performed	Satisfactory	NILM	NILM	Negative (NILM)	
P11012	2023	2023-Q3	2023-07	Post-pandemic recovery	Cytology (Pap)	35-39	37	Not performed	Satisfactory	Positive	ASC-US	Low-grade / equivocal squamous	
P11013	2023	2023-Q3	2023-07	Post-pandemic recovery	Cytology (Pap)	45-49	47	Not performed	Satisfactory	NILM	NILM	Negative (NILM)	
P11014	2023	2023-Q3	2023-07	Post-pandemic recovery	Cytology (Pap)	45-49	49	Not performed	Satisfactory	NILM	NILM	Negative (NILM)	
P11015	2023	2023-Q3	2023-07	Post-pandemic recovery	Cytology (Pap)	40-44	40	Not performed	Satisfactory	NILM	NILM	Negative (NILM)	Candida
P11016	2023	2023-Q3	2023-07	Post-pandemic recovery	Cytology (Pap)	25-29	28	Not performed	Satisfactory	Positive	LSIL	Low-grade / equivocal squamous	
P11017	2023	2023-Q3	2023-07	Post-pandemic recovery	Cytology (Pap)	40-44	41	Not performed	Satisfactory	NILM	NILM	Negative (NILM)	
P11018	2023	2023-Q3	2023-07	Post-pandemic recovery	Cytology (Pap)	30-34	34	Not performed	Satisfactory	NILM	NILM	Negative (NILM)	
P11019	2023	2023-Q3	2023-07	Post-pandemic recovery	Cytology (Pap)	50-54	50	Not performed	Satisfactory	NILM	NILM	Negative (NILM)	
P11020	2023	2023-Q3	2023-07	Post-pandemic recovery	Cytology (Pap)	30-34	33	Not performed	Satisfactory	NILM	NILM	Negative (NILM)	Trichomonas
P11021	2023	2023-Q3	2023-07	Post-pandemic recovery	Cytology (Pap)	30-34	33	Not performed	Satisfactory	NILM	NILM	Negative (NILM)	
P11022	2023	2023-Q3	2023-07	Post-pandemic recovery	Cytology (Pap)	30-34	31	Not performed	Satisfactory	NILM	NILM	Negative (NILM)	
P11023	2023	2023-Q3	2023-07	Post-pandemic recovery	Cytology (Pap)	25-29	26	Not performed	Satisfactory	NILM	NILM	Negative (NILM)	
P11024	2023	2023-Q3	2023-07	Post-pandemic recovery	Cytology (Pap)	60-64	60	Not performed	Satisfactory	NILM	NILM	Negative (NILM)	
P11025	2023	2023-Q3	2023-07	Post-pandemic recovery	Cytology (Pap)	35-39	35	Not performed	Satisfactory	Positive	ASC-US	Low-grade / equivocal squamous	
P11026	2023	2023-Q3	2023-07	Post-pandemic recovery	Cytology (Pap)	35-39	36	Not performed	Satisfactory	NILM	NILM	Negative (NILM)	
P11027	2023	2023-Q3	2023-07	Post-pandemic recovery	Cytology (Pap)	30-34	32	Not performed	Satisfactory	NILM	NILM	Negative (NILM)	
P11028	2023	2023-Q3	2023-07	Post-pandemic recovery	Cytology (Pap)	35-39	39	Not performed	Satisfactory	NILM	NILM	Negative (NILM)	
P11029	2023	2023-Q3	2023-07	Post-pandemic recovery	Cytology (Pap)	30-34	33	Not performed	Satisfactory	NILM	NILM	Negative (NILM)	
P11030	2023	2023-Q3	2023-07	Post-pandemic recovery	Cytology (Pap)	30-34	33	Not performed	Satisfactory	NILM	NILM	Negative (NILM)	
P11031	2023	2023-Q3	2023-07	Post-pandemic recovery	Cytology (Pap)	55-59	58	Not performed	Satisfactory	NILM	NILM	Negative (NILM)	
P11032	2023	2023-Q3	2023-07	Post-pandemic recovery	Cytology (Pap)	35-39	36	Not performed	Satisfactory	NILM	NILM	Negative (NILM)	
P11033	2023	2023-Q3	2023-07	Post-pandemic recovery	Cytology (Pap)	30-34	33	Not performed	Satisfactory	NILM	NILM	Negative (NILM)	
P11034	2023	2023-Q3	2023-07	Post-pandemic recovery	Cytology (Pap)	55-59	58	Not performed	Satisfactory	NILM	NILM	Negative (NILM)	
P11035	2023	2023-Q3	2023-07	Post-pandemic recovery	Cytology (Pap)	40-44	41	Not performed	Satisfactory	NILM	NILM	Negative (NILM)	
P11036	2023	2023-Q3	2023-07	Post-pandemic recovery	Cytology (Pap)	40-44	44	Not performed	Satisfactory	Positive	HSIL	High-grade squamous	Candida
P11037	2023	2023-Q3	2023-07	Post-pandemic recovery	Cytology (Pap)	45-49	48	Not performed	Satisfactory	NILM	NILM	Negative (NILM)	
P11038	2023	2023-Q3	2023-07	Post-pandemic recovery	Cytology (Pap)	35-39	39	Not performed	Satisfactory	NILM	NILM	Negative (NILM)	

patient_id	year	quarter	month	period	primary_test	age_group	age_years	hpv_result	adequacy	result	bethesda	grade	infection
P11039	2023	2023-Q3	2023-07	Post-pandemic recovery	Cytology (Pap)	30-34	33	Not performed	Satisfactory	NILM	NILM	Negative (NILM)	Trichomonas
P11040	2023	2023-Q3	2023-07	Post-pandemic recovery	Cytology (Pap)	45-49	45	Not performed	Satisfactory	NILM	NILM	Negative (NILM)	
P11041	2023	2023-Q3	2023-07	Post-pandemic recovery	Cytology (Pap)	40-44	43	Not performed	Satisfactory	NILM	NILM	Negative (NILM)	
P11042	2023	2023-Q3	2023-07	Post-pandemic recovery	Cytology (Pap)	40-44	42	Not performed	Satisfactory	NILM	NILM	Negative (NILM)	
P11043	2023	2023-Q3	2023-07	Post-pandemic recovery	Cytology (Pap)	30-34	31	Not performed	Satisfactory	Positive	ASC-US	Low-grade / equivocal squamous	
P11044	2023	2023-Q3	2023-07	Post-pandemic recovery	Cytology (Pap)	50-54	50	Not performed	Satisfactory	NILM	NILM	Negative (NILM)	
P11045	2023	2023-Q3	2023-07	Post-pandemic recovery	Cytology (Pap)	40-44	41	Not performed	Satisfactory	NILM	NILM	Negative (NILM)	
P11046	2023	2023-Q3	2023-07	Post-pandemic recovery	Cytology (Pap)	45-49	46	Not performed	Satisfactory	NILM	NILM	Negative (NILM)	
P11047	2023	2023-Q3	2023-07	Post-pandemic recovery	Cytology (Pap)	40-44	44	Not performed	Satisfactory	NILM	NILM	Negative (NILM)	
P11048	2023	2023-Q3	2023-07	Post-pandemic recovery	Cytology (Pap)	60-64	64	Not performed	Satisfactory	NILM	NILM	Negative (NILM)	Candida
P11049	2023	2023-Q3	2023-07	Post-pandemic recovery	Cytology (Pap)	40-44	42	Not performed	Satisfactory	NILM	NILM	Negative (NILM)	
P11050	2023	2023-Q3	2023-07	Post-pandemic recovery	Cytology (Pap)	45-49	49	Not performed	Satisfactory	Positive	HSIL	High-grade squamous	
P11051	2023	2023-Q3	2023-07	Post-pandemic recovery	Cytology (Pap)	45-49	47	Not performed	Satisfactory	NILM	NILM	Negative (NILM)	
P11052	2023	2023-Q3	2023-07	Post-pandemic recovery	Cytology (Pap)	35-39	39	Not performed	Satisfactory	NILM	NILM	Negative (NILM)	
P11053	2023	2023-Q3	2023-07	Post-pandemic recovery	Cytology (Pap)	25-29	25	Not performed	Satisfactory	NILM	NILM	Negative (NILM)	
P11054	2023	2023-Q3	2023-07	Post-pandemic recovery	Cytology (Pap)	45-49	49	Not performed	Satisfactory	NILM	NILM	Negative (NILM)	
P11055	2023	2023-Q3	2023-07	Post-pandemic recovery	Cytology (Pap)	30-34	30	Not performed	Satisfactory	Positive	ASC-H	High-grade squamous	
P11056	2023	2023-Q3	2023-07	Post-pandemic recovery	Cytology (Pap)	25-29	28	Not performed	Satisfactory	Positive	AGC	Glandular	
P11057	2023	2023-Q3	2023-07	Post-pandemic recovery	Cytology (Pap)	45-49	45	Not performed	Satisfactory	Positive	ASC-US	Low-grade / equivocal squamous	
P11058	2023	2023-Q3	2023-07	Post-pandemic recovery	Cytology (Pap)	50-54	54	Not performed	Satisfactory	NILM	NILM	Negative (NILM)	
P11059	2023	2023-Q3	2023-07	Post-pandemic recovery	Cytology (Pap)	55-59	55	Not performed	Satisfactory	NILM	NILM	Negative (NILM)	
P11060	2023	2023-Q3	2023-07	Post-pandemic recovery	Cytology (Pap)	55-59	58	Not performed	Satisfactory	NILM	NILM	Negative (NILM)	
P11061	2023	2023-Q3	2023-07	Post-pandemic recovery	Cytology (Pap)	35-39	37	Not performed	Satisfactory	NILM	NILM	Negative (NILM)	Trichomonas
P11062	2023	2023-Q3	2023-07	Post-pandemic recovery	Cytology (Pap)	35-39	38	Not performed	Satisfactory	NILM	NILM	Negative (NILM)	
P11063	2023	2023-Q3	2023-07	Post-pandemic recovery	Cytology (Pap)	35-39	37	Not performed	Satisfactory	NILM	NILM	Negative (NILM)	Candida
P11064	2023	2023-Q3	2023-07	Post-pandemic recovery	Cytology (Pap)	25-29	27	Not performed	Satisfactory	NILM	NILM	Negative (NILM)	
P11065	2023	2023-Q3	2023-07	Post-pandemic recovery	Cytology (Pap)	25-29	26	Not performed	Satisfactory	NILM	NILM	Negative (NILM)	
P11066	2023	2023-Q3	2023-07	Post-pandemic recovery	Cytology (Pap)	30-34	33	Not performed	Satisfactory	NILM	NILM	Negative (NILM)	Gardnerella
P11067	2023	2023-Q3	2023-07	Post-pandemic recovery	Cytology (Pap)	45-49	46	Not performed	Satisfactory	NILM	NILM	Negative (NILM)	
P11068	2023	2023-Q3	2023-07	Post-pandemic recovery	Cytology (Pap)	35-39	36	Not performed	Satisfactory	Positive	HSIL	High-grade squamous	
P11069	2023	2023-Q3	2023-07	Post-pandemic recovery	Cytology (Pap)	35-39	36	Not performed	Satisfactory	Positive	ASC-US	Low-grade / equivocal squamous	
P11070	2023	2023-Q3	2023-07	Post-pandemic recovery	Cytology (Pap)	40-44	43	Not performed	Satisfactory	NILM	NILM	Negative (NILM)	Candida
P11071	2023	2023-Q3	2023-07	Post-pandemic recovery	Cytology (Pap)	25-29	28	Not performed	Satisfactory	NILM	NILM	Negative (NILM)	
P11072	2023	2023-Q3	2023-07	Post-pandemic recovery	Cytology (Pap)	60-64	60	Not performed	Satisfactory	Positive	ASC-US	Low-grade / equivocal squamous	
P11073	2023	2023-Q3	2023-07	Post-pandemic recovery	Cytology (Pap)	50-54	51	Not performed	Satisfactory	NILM	NILM	Negative (NILM)	
P11074	2023	2023-Q3	2023-07	Post-pandemic recovery	Cytology (Pap)	40-44	44	Not performed	Satisfactory	NILM	NILM	Negative (NILM)	Candida
P11075	2023	2023-Q3	2023-08	Post-pandemic recovery	Cytology (Pap)	45-49	49	Not performed	Satisfactory	NILM	NILM	Negative (NILM)	
P11076	2023	2023-Q3	2023-08	Post-pandemic recovery	Cytology (Pap)	40-44	42	Not performed	Satisfactory	NILM	NILM	Negative (NILM)	
P11077	2023	2023-Q3	2023-08	Post-pandemic recovery	Cytology (Pap)	55-59	58	Not performed	Satisfactory	NILM	NILM	Negative (NILM)	
P11078	2023	2023-Q3	2023-08	Post-pandemic recovery	Cytology (Pap)	35-39	36	Not performed	Satisfactory	NILM	NILM	Negative (NILM)	
P11079	2023	2023-Q3	2023-08	Post-pandemic recovery	Cytology (Pap)	30-34	34	Not performed	Satisfactory	NILM	NILM	Negative (NILM)	
P11080	2023	2023-Q3	2023-08	Post-pandemic recovery	Cytology (Pap)	35-39	35	Not performed	Satisfactory	NILM	NILM	Negative (NILM)	
P11081	2023	2023-Q3	2023-08	Post-pandemic recovery	Cytology (Pap)	60-64	63	Not performed	Satisfactory	NILM	NILM	Negative (NILM)	
P11082	2023	2023-Q3	2023-08	Post-pandemic recovery	Cytology (Pap)	25-29	29	Not performed	Satisfactory	NILM	NILM	Negative (NILM)	
P11083	2023	2023-Q3	2023-08	Post-pandemic recovery	Cytology (Pap)	40-44	44	Not performed	Satisfactory	NILM	NILM	Negative (NILM)	
P11084	2023	2023-Q3	2023-08	Post-pandemic recovery	Cytology (Pap)	30-34	32	Not performed	Satisfactory	NILM	NILM	Negative (NILM)	
P11085	2023	2023-Q3	2023-08	Post-pandemic recovery	Cytology (Pap)	25-29	29	Not performed	Satisfactory	NILM	NILM	Negative (NILM)	
P11086	2023	2023-Q3	2023-08	Post-pandemic recovery	Cytology (Pap)	45-49	49	Not performed	Satisfactory	Positive	LSIL	Low-grade / equivocal squamous	Candida
P11087	2023	2023-Q3	2023-08	Post-pandemic recovery	Cytology (Pap)	30-34	30	Not performed	Satisfactory	NILM	NILM	Negative (NILM)	
P11088	2023	2023-Q3	2023-08	Post-pandemic recovery	Cytology (Pap)	50-54	54	Not performed	Satisfactory	NILM	NILM	Negative (NILM)	
P11089	2023	2023-Q3	2023-08	Post-pandemic recovery	Cytology (Pap)	35-39	36	Not performed	Satisfactory	NILM	NILM	Negative (NILM)	
P11090	2023	2023-Q3	2023-08	Post-pandemic recovery	Cytology (Pap)	25-29	26	Not performed	Satisfactory	NILM	NILM	Negative (NILM)	Gardnerella
P11091	2023	2023-Q3	2023-08	Post-pandemic recovery	Cytology (Pap)	60-64	63	Not performed	Satisfactory	NILM	NILM	Negative (NILM)	
P11092	2023	2023-Q3	2023-08	Post-pandemic recovery	Cytology (Pap)	40-44	43	Not performed	Satisfactory	NILM	NILM	Negative (NILM)	
P11093	2023	2023-Q3	2023-08	Post-pandemic recovery	Cytology (Pap)	50-54	50	Not performed	Satisfactory	NILM	NILM	Negative (NILM)	
P11094	2023	2023-Q3	2023-08	Post-pandemic recovery	Cytology (Pap)	40-44	40	Not performed	Satisfactory	NILM	NILM	Negative (NILM)	
P11095	2023	2023-Q3	2023-08	Post-pandemic recovery	Cytology (Pap)	30-34	31	Not performed	Satisfactory	NILM	NILM	Negative (NILM)	
P11096	2023	2023-Q3	2023-08	Post-pandemic recovery	Cytology (Pap)	40-44	40	Not performed	Unsatisfactory	Unsatisfactory	Unsatisfactory	Unsatisfactory	
P11097	2023	2023-Q3	2023-08	Post-pandemic recovery	Cytology (Pap)	25-29	27	Not performed	Satisfactory	NILM	NILM	Negative (NILM)	Candida
P11098	2023	2023-Q3	2023-08	Post-pandemic recovery	Cytology (Pap)	60-64	64	Not performed	Satisfactory	NILM	NILM	Negative (NILM)	
P11099	2023	2023-Q3	2023-08	Post-pandemic recovery	Cytology (Pap)	25-29	29	Not performed	Satisfactory	NILM	NILM	Negative (NILM)	
P11100	2023	2023-Q3	2023-08	Post-pandemic recovery	Cytology (Pap)	35-39	36	Not performed	Satisfactory	NILM	NILM	Negative (NILM)	
P11101	2023	2023-Q3	2023-08	Post-pandemic recovery	Cytology (Pap)	30-34	32	Not performed	Satisfactory	NILM	NILM	Negative (NILM)	
P11102	2023	2023-Q3	2023-08	Post-pandemic recovery	Cytology (Pap)	30-34	32	Not performed	Satisfactory	Positive	ASC-US	Low-grade / equivocal squamous	
P11103	2023	2023-Q3	2023-08	Post-pandemic recovery	Cytology (Pap)	45-49	49	Not performed	Satisfactory	NILM	NILM	Negative (NILM)	
P11104	2023	2023-Q3	2023-08	Post-pandemic recovery	Cytology (Pap)	30-34	33	Not performed	Satisfactory	NILM	NILM	Negative (NILM)	
P11105	2023	2023-Q3	2023-08	Post-pandemic recovery	Cytology (Pap)	50-54	54	Not performed	Satisfactory	Positive	ASC-US	Low-grade / equivocal squamous	
P11106	2023	2023-Q3	2023-08	Post-pandemic recovery	Cytology (Pap)	35-39	39	Not performed	Satisfactory	NILM	NILM	Negative (NILM)	
P11107	2023	2023-Q3	2023-08	Post-pandemic recovery	Cytology (Pap)	35-39	36	Not performed	Satisfactory	NILM	NILM	Negative (NILM)	Gardnerella
P11108	2023	2023-Q3	2023-08	Post-pandemic recovery	Cytology (Pap)	55-59	58	Not performed	Satisfactory	NILM	NILM	Negative (NILM)	
P11109	2023	2023-Q3	2023-08	Post-pandemic recovery	Cytology (Pap)	50-54	52	Not performed	Satisfactory	NILM	NILM	Negative (NILM)	
P11110	2023	2023-Q3	2023-08	Post-pandemic recovery	Cytology (Pap)	45-49	45	Not performed	Satisfactory	NILM	NILM	Negative (NILM)	
P11111	2023	2023-Q3	2023-08	Post-pandemic recovery	Cytology (Pap)	50-54	50	Not performed	Satisfactory	NILM	NILM	Negative (NILM)	
P11112	2023	2023-Q3	2023-08	Post-pandemic recovery	Cytology (Pap)	40-44	44	Not performed	Unsatisfactory	Unsatisfactory	Unsatisfactory	Unsatisfactory	
P11113	2023	2023-Q3	2023-08	Post-pandemic recovery	Cytology (Pap)	60-64	63	Not performed	Satisfactory	NILM	NILM	Negative (NILM)	
P11114	2023	2023-Q3	2023-08	Post-pandemic recovery	Cytology (Pap)	45-49	48	Not performed	Satisfactory	NILM	NILM	Negative (NILM)	
P11115	2023	2023-Q3	2023-08	Post-pandemic recovery	Cytology (Pap)	30-34	34	Not performed	Satisfactory	NILM	NILM	Negative (NILM)	
P11116	2023	2023-Q3	2023-08	Post-pandemic recovery	Cytology (Pap)	45-49	46	Not performed	Satisfactory	NILM	NILM	Negative (NILM)	
P11117	2023	2023-Q3	2023-08	Post-pandemic recovery	Cytology (Pap)	35-39	36	Not performed	Satisfactory	NILM	NILM	Negative (NILM)	
P11118	2023	2023-Q3	2023-08	Post-pandemic recovery	Cytology (Pap)	40-44	41	Not performed	Unsatisfactory	Unsatisfactory	Unsatisfactory	Unsatisfactory	

patient_id	year	quarter	month	period	primary_test	age_group	age_years	hpv_result	adequacy	result	bethesda	grade	infection
P11119	2023	2023-Q3	2023-08	Post-pandemic recovery	Cytology (Pap)	35-39	39	Not performed	Satisfactory	NILM	NILM	Negative (NILM)	Candida
P11120	2023	2023-Q3	2023-08	Post-pandemic recovery	Cytology (Pap)	35-39	37	Not performed	Satisfactory	Positive	HSIL	High-grade squamous	
P11121	2023	2023-Q3	2023-08	Post-pandemic recovery	Cytology (Pap)	40-44	40	Not performed	Satisfactory	Positive	LSIL	Low-grade / equivocal squamous	
P11122	2023	2023-Q3	2023-08	Post-pandemic recovery	Cytology (Pap)	30-34	34	Not performed	Satisfactory	NILM	NILM	Negative (NILM)	
P11123	2023	2023-Q3	2023-08	Post-pandemic recovery	Cytology (Pap)	50-54	54	Not performed	Satisfactory	NILM	NILM	Negative (NILM)	
P11124	2023	2023-Q3	2023-08	Post-pandemic recovery	Cytology (Pap)	55-59	57	Not performed	Satisfactory	NILM	NILM	Negative (NILM)	Candida
P11125	2023	2023-Q3	2023-08	Post-pandemic recovery	Cytology (Pap)	45-49	46	Not performed	Satisfactory	NILM	NILM	Negative (NILM)	Gardnerella
P11126	2023	2023-Q3	2023-08	Post-pandemic recovery	Cytology (Pap)	55-59	58	Not performed	Satisfactory	NILM	NILM	Negative (NILM)	
P11127	2023	2023-Q3	2023-08	Post-pandemic recovery	Cytology (Pap)	45-49	45	Not performed	Satisfactory	NILM	NILM	Negative (NILM)	Gardnerella
P11128	2023	2023-Q3	2023-08	Post-pandemic recovery	Cytology (Pap)	50-54	50	Not performed	Satisfactory	NILM	NILM	Negative (NILM)	
P11129	2023	2023-Q3	2023-08	Post-pandemic recovery	Cytology (Pap)	60-64	63	Not performed	Satisfactory	NILM	NILM	Negative (NILM)	Candida
P11130	2023	2023-Q3	2023-08	Post-pandemic recovery	Cytology (Pap)	30-34	32	Not performed	Satisfactory	NILM	NILM	Negative (NILM)	
P11131	2023	2023-Q3	2023-08	Post-pandemic recovery	Cytology (Pap)	40-44	41	Not performed	Satisfactory	NILM	NILM	Negative (NILM)	
P11132	2023	2023-Q3	2023-08	Post-pandemic recovery	Cytology (Pap)	25-29	29	Not performed	Satisfactory	NILM	NILM	Negative (NILM)	
P11133	2023	2023-Q3	2023-08	Post-pandemic recovery	Cytology (Pap)	40-44	43	Not performed	Satisfactory	NILM	NILM	Negative (NILM)	
P11134	2023	2023-Q3	2023-08	Post-pandemic recovery	Cytology (Pap)	55-59	55	Not performed	Satisfactory	NILM	NILM	Negative (NILM)	
P11135	2023	2023-Q3	2023-08	Post-pandemic recovery	Cytology (Pap)	30-34	33	Not performed	Satisfactory	NILM	NILM	Negative (NILM)	
P11136	2023	2023-Q3	2023-08	Post-pandemic recovery	Cytology (Pap)	25-29	28	Not performed	Satisfactory	NILM	NILM	Negative (NILM)	
P11137	2023	2023-Q3	2023-08	Post-pandemic recovery	Cytology (Pap)	40-44	43	Not performed	Satisfactory	NILM	NILM	Negative (NILM)	
P11138	2023	2023-Q3	2023-08	Post-pandemic recovery	Cytology (Pap)	25-29	29	Not performed	Satisfactory	NILM	NILM	Negative (NILM)	
P11139	2023	2023-Q3	2023-08	Post-pandemic recovery	Cytology (Pap)	60-64	64	Not performed	Satisfactory	NILM	NILM	Negative (NILM)	
P11140	2023	2023-Q3	2023-08	Post-pandemic recovery	Cytology (Pap)	30-34	30	Not performed	Satisfactory	Positive	LSIL	Low-grade / equivocal squamous	
P11141	2023	2023-Q3	2023-08	Post-pandemic recovery	Cytology (Pap)	60-64	60	Not performed	Satisfactory	Positive	HSIL	High-grade squamous	
P11142	2023	2023-Q3	2023-08	Post-pandemic recovery	Cytology (Pap)	40-44	44	Not performed	Satisfactory	NILM	NILM	Negative (NILM)	
P11143	2023	2023-Q3	2023-08	Post-pandemic recovery	Cytology (Pap)	45-49	49	Not performed	Satisfactory	NILM	NILM	Negative (NILM)	
P11144	2023	2023-Q3	2023-08	Post-pandemic recovery	Cytology (Pap)	25-29	25	Not performed	Satisfactory	NILM	NILM	Negative (NILM)	
P11145	2023	2023-Q3	2023-08	Post-pandemic recovery	Cytology (Pap)	30-34	30	Not performed	Satisfactory	NILM	NILM	Negative (NILM)	
P11146	2023	2023-Q3	2023-08	Post-pandemic recovery	Cytology (Pap)	25-29	25	Not performed	Satisfactory	NILM	NILM	Negative (NILM)	
P11147	2023	2023-Q3	2023-08	Post-pandemic recovery	Cytology (Pap)	50-54	51	Not performed	Satisfactory	NILM	NILM	Negative (NILM)	
P11148	2023	2023-Q3	2023-08	Post-pandemic recovery	Cytology (Pap)	35-39	36	Not performed	Satisfactory	NILM	NILM	Negative (NILM)	
P11149	2023	2023-Q3	2023-08	Post-pandemic recovery	Cytology (Pap)	40-44	44	Not performed	Satisfactory	NILM	NILM	Negative (NILM)	
P11150	2023	2023-Q3	2023-08	Post-pandemic recovery	Cytology (Pap)	40-44	42	Not performed	Satisfactory	NILM	NILM	Negative (NILM)	
P11151	2023	2023-Q3	2023-08	Post-pandemic recovery	Cytology (Pap)	30-34	31	Not performed	Satisfactory	NILM	NILM	Negative (NILM)	
P11152	2023	2023-Q3	2023-08	Post-pandemic recovery	Cytology (Pap)	35-39	35	Not performed	Satisfactory	NILM	NILM	Negative (NILM)	
P11153	2023	2023-Q3	2023-08	Post-pandemic recovery	Cytology (Pap)	55-59	58	Not performed	Satisfactory	NILM	NILM	Negative (NILM)	Gardnerella
P11154	2023	2023-Q3	2023-08	Post-pandemic recovery	Cytology (Pap)	35-39	35	Not performed	Satisfactory	NILM	NILM	Negative (NILM)	
P11155	2023	2023-Q3	2023-08	Post-pandemic recovery	Cytology (Pap)	45-49	49	Not performed	Satisfactory	NILM	NILM	Negative (NILM)	
P11156	2023	2023-Q3	2023-08	Post-pandemic recovery	Cytology (Pap)	35-39	36	Not performed	Satisfactory	NILM	NILM	Negative (NILM)	
P11157	2023	2023-Q3	2023-08	Post-pandemic recovery	Cytology (Pap)	30-34	30	Not performed	Satisfactory	NILM	NILM	Negative (NILM)	
P11158	2023	2023-Q3	2023-08	Post-pandemic recovery	Cytology (Pap)	25-29	26	Not performed	Satisfactory	NILM	NILM	Negative (NILM)	
P11159	2023	2023-Q3	2023-08	Post-pandemic recovery	Cytology (Pap)	40-44	44	Not performed	Satisfactory	NILM	NILM	Negative (NILM)	
P11160	2023	2023-Q3	2023-08	Post-pandemic recovery	Cytology (Pap)	35-39	39	Not performed	Satisfactory	Positive	ASC-US	Low-grade / equivocal squamous	
P11161	2023	2023-Q3	2023-08	Post-pandemic recovery	Cytology (Pap)	35-39	39	Not performed	Satisfactory	NILM	NILM	Negative (NILM)	
P11162	2023	2023-Q3	2023-08	Post-pandemic recovery	Cytology (Pap)	40-44	43	Not performed	Satisfactory	NILM	NILM	Negative (NILM)	
P11163	2023	2023-Q3	2023-08	Post-pandemic recovery	Cytology (Pap)	55-59	57	Not performed	Satisfactory	NILM	NILM	Negative (NILM)	
P11164	2023	2023-Q3	2023-08	Post-pandemic recovery	Cytology (Pap)	50-54	51	Not performed	Satisfactory	NILM	NILM	Negative (NILM)	
P11165	2023	2023-Q3	2023-08	Post-pandemic recovery	Cytology (Pap)	30-34	30	Not performed	Satisfactory	NILM	NILM	Negative (NILM)	
P11166	2023	2023-Q3	2023-08	Post-pandemic recovery	Cytology (Pap)	40-44	44	Not performed	Satisfactory	NILM	NILM	Negative (NILM)	
P11167	2023	2023-Q3	2023-08	Post-pandemic recovery	Cytology (Pap)	40-44	43	Not performed	Satisfactory	Positive	LSIL	Low-grade / equivocal squamous	
P11168	2023	2023-Q3	2023-08	Post-pandemic recovery	Cytology (Pap)	60-64	63	Not performed	Satisfactory	NILM	NILM	Negative (NILM)	Trichomonas
P11169	2023	2023-Q3	2023-08	Post-pandemic recovery	Cytology (Pap)	50-54	51	Not performed	Satisfactory	NILM	NILM	Negative (NILM)	
P11170	2023	2023-Q3	2023-08	Post-pandemic recovery	Cytology (Pap)	25-29	27	Not performed	Satisfactory	NILM	NILM	Negative (NILM)	
P11171	2023	2023-Q3	2023-08	Post-pandemic recovery	Cytology (Pap)	40-44	44	Not performed	Satisfactory	NILM	NILM	Negative (NILM)	
P11172	2023	2023-Q3	2023-08	Post-pandemic recovery	Cytology (Pap)	30-34	31	Not performed	Satisfactory	NILM	NILM	Negative (NILM)	
P11173	2023	2023-Q3	2023-08	Post-pandemic recovery	Cytology (Pap)	35-39	35	Not performed	Satisfactory	NILM	NILM	Negative (NILM)	Candida
P11174	2023	2023-Q3	2023-08	Post-pandemic recovery	Cytology (Pap)	45-49	49	Not performed	Satisfactory	NILM	NILM	Negative (NILM)	
P11175	2023	2023-Q3	2023-08	Post-pandemic recovery	Cytology (Pap)	55-59	57	Not performed	Satisfactory	NILM	NILM	Negative (NILM)	
P11176	2023	2023-Q3	2023-08	Post-pandemic recovery	Cytology (Pap)	25-29	28	Not performed	Satisfactory	Positive	ASC-US	Low-grade / equivocal squamous	Trichomonas
P11177	2023	2023-Q3	2023-08	Post-pandemic recovery	Cytology (Pap)	30-34	33	Not performed	Satisfactory	NILM	NILM	Negative (NILM)	Candida
P11178	2023	2023-Q3	2023-08	Post-pandemic recovery	Cytology (Pap)	25-29	28	Not performed	Satisfactory	NILM	NILM	Negative (NILM)	
P11179	2023	2023-Q3	2023-08	Post-pandemic recovery	Cytology (Pap)	50-54	50	Not performed	Satisfactory	NILM	NILM	Negative (NILM)	
P11180	2023	2023-Q3	2023-08	Post-pandemic recovery	Cytology (Pap)	25-29	29	Not performed	Satisfactory	NILM	NILM	Negative (NILM)	
P11181	2023	2023-Q3	2023-08	Post-pandemic recovery	Cytology (Pap)	35-39	39	Not performed	Satisfactory	NILM	NILM	Negative (NILM)	
P11182	2023	2023-Q3	2023-08	Post-pandemic recovery	Cytology (Pap)	35-39	36	Not performed	Satisfactory	NILM	NILM	Negative (NILM)	
P11183	2023	2023-Q3	2023-08	Post-pandemic recovery	Cytology (Pap)	25-29	27	Not performed	Satisfactory	NILM	NILM	Negative (NILM)	
P11184	2023	2023-Q3	2023-08	Post-pandemic recovery	Cytology (Pap)	35-39	37	Not performed	Satisfactory	NILM	NILM	Negative (NILM)	
P11185	2023	2023-Q3	2023-08	Post-pandemic recovery	Cytology (Pap)	45-49	47	Not performed	Satisfactory	NILM	NILM	Negative (NILM)	
P11186	2023	2023-Q3	2023-08	Post-pandemic recovery	Cytology (Pap)	40-44	43	Not performed	Satisfactory	NILM	NILM	Negative (NILM)	
P11187	2023	2023-Q3	2023-08	Post-pandemic recovery	Cytology (Pap)	55-59	57	Not performed	Unsatisfactory	Unsatisfactory	Unsatisfactory	Unsatisfactory	
P11188	2023	2023-Q3	2023-08	Post-pandemic recovery	Cytology (Pap)	60-64	63	Not performed	Satisfactory	NILM	NILM	Negative (NILM)	
P11189	2023	2023-Q3	2023-08	Post-pandemic recovery	Cytology (Pap)	45-49	45	Not performed	Satisfactory	NILM	NILM	Negative (NILM)	
P11190	2023	2023-Q3	2023-08	Post-pandemic recovery	Cytology (Pap)	30-34	32	Not performed	Satisfactory	Positive	HSIL	High-grade squamous	
P11191	2023	2023-Q3	2023-08	Post-pandemic recovery	Cytology (Pap)	35-39	36	Not performed	Satisfactory	NILM	NILM	Negative (NILM)	
P11192	2023	2023-Q3	2023-08	Post-pandemic recovery	Cytology (Pap)	40-44	44	Not performed	Satisfactory	Positive	ASC-US	Low-grade / equivocal squamous	
P11193	2023	2023-Q3	2023-08	Post-pandemic recovery	Cytology (Pap)	45-49	47	Not performed	Satisfactory	NILM	NILM	Negative (NILM)	
P11194	2023	2023-Q3	2023-08	Post-pandemic recovery	Cytology (Pap)	25-29	26	Not performed	Satisfactory	Positive	LSIL	Low-grade / equivocal squamous	
P11195	2023	2023-Q3	2023-08	Post-pandemic recovery	Cytology (Pap)	35-39	39	Not performed	Satisfactory	NILM	NILM	Negative (NILM)	
P11196	2023	2023-Q3	2023-08	Post-pandemic recovery	Cytology (Pap)	25-29	29	Not performed	Satisfactory	NILM	NILM	Negative (NILM)	
P11197	2023	2023-Q3	2023-08	Post-pandemic recovery	Cytology (Pap)	55-59	55	Not performed	Satisfactory	NILM	NILM	Negative (NILM)	
P11198	2023	2023-Q3	2023-08	Post-pandemic recovery	Cytology (Pap)	40-44	43	Not performed	Satisfactory	NILM	NILM	Negative (NILM)	

patient_id	year	quarter	month	period	primary_test	age_group	age_years	hpv_result	adequacy	result	bethesda	grade	infection
P11199	2023	2023-Q3	2023-08	Post-pandemic recovery	Cytology (Pap)	50-54	53	Not performed	Satisfactory	NILM	NILM	Negative (NILM)	
P11200	2023	2023-Q3	2023-08	Post-pandemic recovery	Cytology (Pap)	40-44	43	Not performed	Satisfactory	NILM	NILM	Negative (NILM)	
P11201	2023	2023-Q3	2023-08	Post-pandemic recovery	Cytology (Pap)	45-49	46	Not performed	Unsatisfactory	Unsatisfactory	Unsatisfactory	Unsatisfactory	
P11202	2023	2023-Q3	2023-08	Post-pandemic recovery	Cytology (Pap)	40-44	41	Not performed	Satisfactory	NILM	NILM	Negative (NILM)	
P11203	2023	2023-Q3	2023-08	Post-pandemic recovery	Cytology (Pap)	60-64	61	Not performed	Unsatisfactory	Unsatisfactory	Unsatisfactory	Unsatisfactory	
P11204	2023	2023-Q3	2023-08	Post-pandemic recovery	Cytology (Pap)	50-54	50	Not performed	Satisfactory	NILM	NILM	Negative (NILM)	
P11205	2023	2023-Q3	2023-08	Post-pandemic recovery	Cytology (Pap)	30-34	33	Not performed	Satisfactory	NILM	NILM	Negative (NILM)	
P11206	2023	2023-Q3	2023-08	Post-pandemic recovery	Cytology (Pap)	40-44	43	Not performed	Satisfactory	NILM	NILM	Negative (NILM)	
P11207	2023	2023-Q3	2023-08	Post-pandemic recovery	Cytology (Pap)	25-29	29	Not performed	Satisfactory	NILM	NILM	Negative (NILM)	
P11208	2023	2023-Q3	2023-08	Post-pandemic recovery	Cytology (Pap)	35-39	36	Not performed	Satisfactory	NILM	NILM	Negative (NILM)	
P11209	2023	2023-Q3	2023-08	Post-pandemic recovery	Cytology (Pap)	60-64	63	Not performed	Satisfactory	NILM	NILM	Negative (NILM)	
P11210	2023	2023-Q3	2023-08	Post-pandemic recovery	Cytology (Pap)	25-29	27	Not performed	Unsatisfactory	Unsatisfactory	Unsatisfactory	Unsatisfactory	
P11211	2023	2023-Q3	2023-08	Post-pandemic recovery	Cytology (Pap)	45-49	49	Not performed	Satisfactory	Positive	AGC	Glandular	
P11212	2023	2023-Q3	2023-08	Post-pandemic recovery	Cytology (Pap)	35-39	39	Not performed	Satisfactory	NILM	NILM	Negative (NILM)	
P11213	2023	2023-Q3	2023-08	Post-pandemic recovery	Cytology (Pap)	45-49	48	Not performed	Satisfactory	NILM	NILM	Negative (NILM)	
P11214	2023	2023-Q3	2023-08	Post-pandemic recovery	Cytology (Pap)	55-59	59	Not performed	Satisfactory	Positive	LSIL	Low-grade / equivocal squamous	
P11215	2023	2023-Q3	2023-08	Post-pandemic recovery	Cytology (Pap)	25-29	27	Not performed	Satisfactory	NILM	NILM	Negative (NILM)	
P11216	2023	2023-Q3	2023-08	Post-pandemic recovery	Cytology (Pap)	45-49	48	Not performed	Unsatisfactory	Unsatisfactory	Unsatisfactory	Unsatisfactory	
P11217	2023	2023-Q3	2023-08	Post-pandemic recovery	Cytology (Pap)	30-34	31	Not performed	Satisfactory	NILM	NILM	Negative (NILM)	
P11218	2023	2023-Q3	2023-08	Post-pandemic recovery	Cytology (Pap)	40-44	43	Not performed	Satisfactory	NILM	NILM	Negative (NILM)	
P11219	2023	2023-Q3	2023-08	Post-pandemic recovery	Cytology (Pap)	45-49	45	Not performed	Satisfactory	NILM	NILM	Negative (NILM)	
P11220	2023	2023-Q3	2023-08	Post-pandemic recovery	Cytology (Pap)	30-34	30	Not performed	Unsatisfactory	Unsatisfactory	Unsatisfactory	Unsatisfactory	
P11221	2023	2023-Q3	2023-08	Post-pandemic recovery	Cytology (Pap)	55-59	55	Not performed	Satisfactory	Positive	ASC-US	Low-grade / equivocal squamous	
P11222	2023	2023-Q3	2023-08	Post-pandemic recovery	Cytology (Pap)	40-44	42	Not performed	Satisfactory	NILM	NILM	Negative (NILM)	
P11223	2023	2023-Q3	2023-08	Post-pandemic recovery	Cytology (Pap)	30-34	30	Not performed	Satisfactory	NILM	NILM	Negative (NILM)	
P11224	2023	2023-Q3	2023-08	Post-pandemic recovery	Cytology (Pap)	25-29	26	Not performed	Satisfactory	Positive	ASC-H	High-grade squamous	
P11225	2023	2023-Q3	2023-08	Post-pandemic recovery	Cytology (Pap)	35-39	39	Not performed	Satisfactory	NILM	NILM	Negative (NILM)	
P11226	2023	2023-Q3	2023-08	Post-pandemic recovery	Cytology (Pap)	35-39	35	Not performed	Satisfactory	NILM	NILM	Negative (NILM)	
P11227	2023	2023-Q3	2023-08	Post-pandemic recovery	Cytology (Pap)	40-44	42	Not performed	Satisfactory	NILM	NILM	Negative (NILM)	Candida
P11228	2023	2023-Q3	2023-08	Post-pandemic recovery	Cytology (Pap)	25-29	27	Not performed	Satisfactory	NILM	NILM	Negative (NILM)	
P11229	2023	2023-Q3	2023-08	Post-pandemic recovery	Cytology (Pap)	35-39	36	Not performed	Satisfactory	NILM	NILM	Negative (NILM)	
P11230	2023	2023-Q3	2023-08	Post-pandemic recovery	Cytology (Pap)	35-39	35	Not performed	Satisfactory	NILM	NILM	Negative (NILM)	
P11231	2023	2023-Q3	2023-08	Post-pandemic recovery	Cytology (Pap)	40-44	42	Not performed	Satisfactory	Positive	ASC-US	Low-grade / equivocal squamous	
P11232	2023	2023-Q3	2023-08	Post-pandemic recovery	Cytology (Pap)	35-39	36	Not performed	Unsatisfactory	Unsatisfactory	Unsatisfactory	Unsatisfactory	
P11233	2023	2023-Q3	2023-08	Post-pandemic recovery	Cytology (Pap)	35-39	36	Not performed	Satisfactory	NILM	NILM	Negative (NILM)	
P11234	2023	2023-Q3	2023-08	Post-pandemic recovery	Cytology (Pap)	45-49	48	Not performed	Satisfactory	NILM	NILM	Negative (NILM)	
P11235	2023	2023-Q3	2023-08	Post-pandemic recovery	Cytology (Pap)	40-44	41	Not performed	Satisfactory	NILM	NILM	Negative (NILM)	
P11236	2023	2023-Q3	2023-08	Post-pandemic recovery	Cytology (Pap)	30-34	34	Not performed	Unsatisfactory	Unsatisfactory	Unsatisfactory	Unsatisfactory	
P11237	2023	2023-Q3	2023-08	Post-pandemic recovery	Cytology (Pap)	30-34	31	Not performed	Satisfactory	NILM	NILM	Negative (NILM)	
P11238	2023	2023-Q3	2023-08	Post-pandemic recovery	Cytology (Pap)	25-29	25	Not performed	Satisfactory	NILM	NILM	Negative (NILM)	
P11239	2023	2023-Q3	2023-08	Post-pandemic recovery	Cytology (Pap)	55-59	56	Not performed	Satisfactory	NILM	NILM	Negative (NILM)	
P11240	2023	2023-Q3	2023-08	Post-pandemic recovery	Cytology (Pap)	60-64	61	Not performed	Satisfactory	NILM	NILM	Negative (NILM)	
P11241	2023	2023-Q3	2023-08	Post-pandemic recovery	Cytology (Pap)	40-44	43	Not performed	Satisfactory	NILM	NILM	Negative (NILM)	
P11242	2023	2023-Q3	2023-08	Post-pandemic recovery	Cytology (Pap)	40-44	43	Not performed	Satisfactory	NILM	NILM	Negative (NILM)	
P11243	2023	2023-Q3	2023-08	Post-pandemic recovery	Cytology (Pap)	35-39	36	Not performed	Satisfactory	NILM	NILM	Negative (NILM)	
P11244	2023	2023-Q3	2023-08	Post-pandemic recovery	Cytology (Pap)	45-49	47	Not performed	Satisfactory	NILM	NILM	Negative (NILM)	
P11245	2023	2023-Q3	2023-08	Post-pandemic recovery	Cytology (Pap)	40-44	43	Not performed	Satisfactory	NILM	NILM	Negative (NILM)	
P11246	2023	2023-Q3	2023-08	Post-pandemic recovery	Cytology (Pap)	30-34	34	Not performed	Satisfactory	NILM	NILM	Negative (NILM)	
P11247	2023	2023-Q3	2023-08	Post-pandemic recovery	Cytology (Pap)	40-44	41	Not performed	Satisfactory	NILM	NILM	Negative (NILM)	
P11248	2023	2023-Q3	2023-08	Post-pandemic recovery	Cytology (Pap)	40-44	43	Not performed	Satisfactory	NILM	NILM	Negative (NILM)	
P11249	2023	2023-Q3	2023-08	Post-pandemic recovery	Cytology (Pap)	60-64	64	Not performed	Satisfactory	NILM	NILM	Negative (NILM)	
P11250	2023	2023-Q3	2023-08	Post-pandemic recovery	Cytology (Pap)	30-34	32	Not performed	Satisfactory	NILM	NILM	Negative (NILM)	Gardnerella
P11251	2023	2023-Q3	2023-08	Post-pandemic recovery	Cytology (Pap)	40-44	42	Not performed	Satisfactory	Positive	ASC-US	Low-grade / equivocal squamous	Candida
P11252	2023	2023-Q3	2023-08	Post-pandemic recovery	Cytology (Pap)	30-34	30	Not performed	Satisfactory	NILM	NILM	Negative (NILM)	
P11253	2023	2023-Q3	2023-08	Post-pandemic recovery	Cytology (Pap)	40-44	44	Not performed	Satisfactory	NILM	NILM	Negative (NILM)	
P11254	2023	2023-Q3	2023-08	Post-pandemic recovery	Cytology (Pap)	40-44	43	Not performed	Satisfactory	NILM	NILM	Negative (NILM)	
P11255	2023	2023-Q3	2023-08	Post-pandemic recovery	Cytology (Pap)	50-54	54	Not performed	Satisfactory	Positive	AGC	Glandular	
P11256	2023	2023-Q3	2023-08	Post-pandemic recovery	Cytology (Pap)	30-34	34	Not performed	Satisfactory	NILM	NILM	Negative (NILM)	
P11257	2023	2023-Q3	2023-08	Post-pandemic recovery	Cytology (Pap)	50-54	52	Not performed	Satisfactory	NILM	NILM	Negative (NILM)	
P11258	2023	2023-Q3	2023-08	Post-pandemic recovery	Cytology (Pap)	40-44	44	Not performed	Satisfactory	NILM	NILM	Negative (NILM)	
P11259	2023	2023-Q3	2023-08	Post-pandemic recovery	Cytology (Pap)	50-54	53	Not performed	Satisfactory	NILM	NILM	Negative (NILM)	
P11260	2023	2023-Q3	2023-08	Post-pandemic recovery	Cytology (Pap)	55-59	59	Not performed	Satisfactory	NILM	NILM	Negative (NILM)	
P11261	2023	2023-Q3	2023-08	Post-pandemic recovery	Cytology (Pap)	30-34	30	Not performed	Satisfactory	NILM	NILM	Negative (NILM)	
P11262	2023	2023-Q3	2023-08	Post-pandemic recovery	Cytology (Pap)	50-54	54	Not performed	Satisfactory	NILM	NILM	Negative (NILM)	
P11263	2023	2023-Q3	2023-08	Post-pandemic recovery	Cytology (Pap)	50-54	52	Not performed	Satisfactory	NILM	NILM	Negative (NILM)	
P11264	2023	2023-Q3	2023-08	Post-pandemic recovery	Cytology (Pap)	30-34	34	Not performed	Satisfactory	NILM	NILM	Negative (NILM)	
P11265	2023	2023-Q3	2023-08	Post-pandemic recovery	Cytology (Pap)	40-44	43	Not performed	Satisfactory	NILM	NILM	Negative (NILM)	
P11266	2023	2023-Q3	2023-08	Post-pandemic recovery	Cytology (Pap)	30-34	32	Not performed	Satisfactory	NILM	NILM	Negative (NILM)	
P11267	2023	2023-Q3	2023-08	Post-pandemic recovery	Cytology (Pap)	40-44	42	Not performed	Satisfactory	NILM	NILM	Negative (NILM)	
P11268	2023	2023-Q3	2023-08	Post-pandemic recovery	Cytology (Pap)	40-44	43	Not performed	Satisfactory	Positive	ASC-US	Low-grade / equivocal squamous	
P11269	2023	2023-Q3	2023-08	Post-pandemic recovery	Cytology (Pap)	30-34	31	Not performed	Unsatisfactory	Unsatisfactory	Unsatisfactory	Unsatisfactory	
P11270	2023	2023-Q3	2023-08	Post-pandemic recovery	Cytology (Pap)	40-44	42	Not performed	Satisfactory	NILM	NILM	Negative (NILM)	
P11271	2023	2023-Q3	2023-08	Post-pandemic recovery	Cytology (Pap)	40-44	44	Not performed	Satisfactory	Positive	HSIL	High-grade squamous	
P11272	2023	2023-Q3	2023-09	Post-pandemic recovery	Cytology (Pap)	55-59	58	Not performed	Satisfactory	NILM	NILM	Negative (NILM)	Candida
P11273	2023	2023-Q3	2023-09	Post-pandemic recovery	Cytology (Pap)	50-54	53	Not performed	Satisfactory	NILM	NILM	Negative (NILM)	
P11274	2023	2023-Q3	2023-09	Post-pandemic recovery	Cytology (Pap)	50-54	51	Not performed	Satisfactory	NILM	NILM	Negative (NILM)	
P11275	2023	2023-Q3	2023-09	Post-pandemic recovery	Cytology (Pap)	45-49	47	Not performed	Satisfactory	NILM	NILM	Negative (NILM)	
P11276	2023	2023-Q3	2023-09	Post-pandemic recovery	Cytology (Pap)	45-49	49	Not performed	Satisfactory	NILM	NILM	Negative (NILM)	
P11277	2023	2023-Q3	2023-09	Post-pandemic recovery	Cytology (Pap)	30-34	31	Not performed	Satisfactory	NILM	NILM	Negative (NILM)	
P11278	2023	2023-Q3	2023-09	Post-pandemic recovery	Cytology (Pap)	25-29	26	Not performed	Satisfactory	Positive	LSIL	Low-grade / equivocal squamous	

patient_id	year	quarter	month	period	primary_test	age_group	age_years	hpv_result	adequacy	result	bethesda	grade	infection
P11279	2023	2023-Q3	2023-09	Post-pandemic recovery	Cytology (Pap)	25-29	25	Not performed	Satisfactory	Positive	ASC-US	Low-grade / equivocal squamous	
P11280	2023	2023-Q3	2023-09	Post-pandemic recovery	Cytology (Pap)	60-64	62	Not performed	Satisfactory	NILM	NILM	Negative (NILM)	Trichomonas
P11281	2023	2023-Q3	2023-09	Post-pandemic recovery	Cytology (Pap)	45-49	45	Not performed	Satisfactory	NILM	NILM	Negative (NILM)	
P11282	2023	2023-Q3	2023-09	Post-pandemic recovery	Cytology (Pap)	35-39	39	Not performed	Satisfactory	NILM	NILM	Negative (NILM)	
P11283	2023	2023-Q3	2023-09	Post-pandemic recovery	Cytology (Pap)	40-44	44	Not performed	Satisfactory	NILM	NILM	Negative (NILM)	
P11284	2023	2023-Q3	2023-09	Post-pandemic recovery	Cytology (Pap)	45-49	46	Not performed	Satisfactory	Positive	ASC-US	Low-grade / equivocal squamous	
P11285	2023	2023-Q3	2023-09	Post-pandemic recovery	Cytology (Pap)	55-59	56	Not performed	Satisfactory	Positive	HSIL	High-grade squamous	
P11286	2023	2023-Q3	2023-09	Post-pandemic recovery	Cytology (Pap)	25-29	28	Not performed	Satisfactory	NILM	NILM	Negative (NILM)	
P11287	2023	2023-Q3	2023-09	Post-pandemic recovery	Cytology (Pap)	30-34	30	Not performed	Satisfactory	NILM	NILM	Negative (NILM)	
P11288	2023	2023-Q3	2023-09	Post-pandemic recovery	Cytology (Pap)	40-44	42	Not performed	Satisfactory	NILM	NILM	Negative (NILM)	
P11289	2023	2023-Q3	2023-09	Post-pandemic recovery	Cytology (Pap)	30-34	33	Not performed	Satisfactory	NILM	NILM	Negative (NILM)	
P11290	2023	2023-Q3	2023-09	Post-pandemic recovery	Cytology (Pap)	25-29	25	Not performed	Satisfactory	NILM	NILM	Negative (NILM)	
P11291	2023	2023-Q3	2023-09	Post-pandemic recovery	Cytology (Pap)	60-64	61	Not performed	Satisfactory	NILM	NILM	Negative (NILM)	
P11292	2023	2023-Q3	2023-09	Post-pandemic recovery	Cytology (Pap)	40-44	44	Not performed	Satisfactory	NILM	NILM	Negative (NILM)	
P11293	2023	2023-Q3	2023-09	Post-pandemic recovery	Cytology (Pap)	45-49	49	Not performed	Satisfactory	NILM	NILM	Negative (NILM)	
P11294	2023	2023-Q3	2023-09	Post-pandemic recovery	Cytology (Pap)	50-54	54	Not performed	Satisfactory	NILM	NILM	Negative (NILM)	
P11295	2023	2023-Q3	2023-09	Post-pandemic recovery	Cytology (Pap)	45-49	46	Not performed	Satisfactory	NILM	NILM	Negative (NILM)	
P11296	2023	2023-Q3	2023-09	Post-pandemic recovery	Cytology (Pap)	30-34	30	Not performed	Satisfactory	Positive	HSIL	High-grade squamous	
P11297	2023	2023-Q3	2023-09	Post-pandemic recovery	Cytology (Pap)	25-29	28	Not performed	Satisfactory	NILM	NILM	Negative (NILM)	Gardnerella
P11298	2023	2023-Q3	2023-09	Post-pandemic recovery	Cytology (Pap)	30-34	30	Not performed	Satisfactory	NILM	NILM	Negative (NILM)	Candida
P11299	2023	2023-Q3	2023-09	Post-pandemic recovery	Cytology (Pap)	35-39	35	Not performed	Satisfactory	NILM	NILM	Negative (NILM)	
P11300	2023	2023-Q3	2023-09	Post-pandemic recovery	Cytology (Pap)	35-39	38	Not performed	Satisfactory	NILM	NILM	Negative (NILM)	
P11301	2023	2023-Q3	2023-09	Post-pandemic recovery	Cytology (Pap)	50-54	54	Not performed	Satisfactory	NILM	NILM	Negative (NILM)	
P11302	2023	2023-Q3	2023-09	Post-pandemic recovery	Cytology (Pap)	25-29	29	Not performed	Satisfactory	NILM	NILM	Negative (NILM)	
P11303	2023	2023-Q3	2023-09	Post-pandemic recovery	Cytology (Pap)	45-49	48	Not performed	Satisfactory	NILM	NILM	Negative (NILM)	
P11304	2023	2023-Q3	2023-09	Post-pandemic recovery	Cytology (Pap)	30-34	32	Not performed	Satisfactory	NILM	NILM	Negative (NILM)	
P11305	2023	2023-Q3	2023-09	Post-pandemic recovery	Cytology (Pap)	45-49	46	Not performed	Satisfactory	NILM	NILM	Negative (NILM)	Candida
P11306	2023	2023-Q3	2023-09	Post-pandemic recovery	Cytology (Pap)	30-34	33	Not performed	Satisfactory	NILM	NILM	Negative (NILM)	
P11307	2023	2023-Q3	2023-09	Post-pandemic recovery	Cytology (Pap)	40-44	41	Not performed	Satisfactory	NILM	NILM	Negative (NILM)	
P11308	2023	2023-Q3	2023-09	Post-pandemic recovery	Cytology (Pap)	40-44	41	Not performed	Satisfactory	NILM	NILM	Negative (NILM)	
P11309	2023	2023-Q3	2023-09	Post-pandemic recovery	Cytology (Pap)	25-29	29	Not performed	Satisfactory	NILM	NILM	Negative (NILM)	
P11310	2023	2023-Q3	2023-09	Post-pandemic recovery	Cytology (Pap)	40-44	42	Not performed	Satisfactory	NILM	NILM	Negative (NILM)	
P11311	2023	2023-Q3	2023-09	Post-pandemic recovery	Cytology (Pap)	35-39	35	Not performed	Satisfactory	NILM	NILM	Negative (NILM)	
P11312	2023	2023-Q3	2023-09	Post-pandemic recovery	Cytology (Pap)	50-54	50	Not performed	Satisfactory	NILM	NILM	Negative (NILM)	
P11313	2023	2023-Q3	2023-09	Post-pandemic recovery	Cytology (Pap)	35-39	38	Not performed	Satisfactory	NILM	NILM	Negative (NILM)	
P11314	2023	2023-Q3	2023-09	Post-pandemic recovery	Cytology (Pap)	40-44	42	Not performed	Satisfactory	NILM	NILM	Negative (NILM)	Other
P11315	2023	2023-Q3	2023-09	Post-pandemic recovery	Cytology (Pap)	30-34	32	Not performed	Satisfactory	NILM	NILM	Negative (NILM)	
P11316	2023	2023-Q3	2023-09	Post-pandemic recovery	Cytology (Pap)	40-44	44	Not performed	Unsatisfactory	Unsatisfactory	Unsatisfactory	Unsatisfactory	
P11317	2023	2023-Q3	2023-09	Post-pandemic recovery	Cytology (Pap)	35-39	36	Not performed	Satisfactory	NILM	NILM	Negative (NILM)	
P11318	2023	2023-Q3	2023-09	Post-pandemic recovery	Cytology (Pap)	40-44	41	Not performed	Satisfactory	NILM	NILM	Negative (NILM)	
P11319	2023	2023-Q3	2023-09	Post-pandemic recovery	Cytology (Pap)	40-44	44	Not performed	Satisfactory	NILM	NILM	Negative (NILM)	
P11320	2023	2023-Q3	2023-09	Post-pandemic recovery	Cytology (Pap)	40-44	40	Not performed	Satisfactory	Positive	ASC-H	High-grade squamous	
P11321	2023	2023-Q3	2023-09	Post-pandemic recovery	Cytology (Pap)	45-49	49	Not performed	Satisfactory	NILM	NILM	Negative (NILM)	
P11322	2023	2023-Q3	2023-09	Post-pandemic recovery	Cytology (Pap)	55-59	57	Not performed	Satisfactory	NILM	NILM	Negative (NILM)	
P11323	2023	2023-Q3	2023-09	Post-pandemic recovery	Cytology (Pap)	30-34	34	Not performed	Satisfactory	NILM	NILM	Negative (NILM)	
P11324	2023	2023-Q3	2023-09	Post-pandemic recovery	Cytology (Pap)	45-49	46	Not performed	Satisfactory	NILM	NILM	Negative (NILM)	Trichomonas
P11325	2023	2023-Q3	2023-09	Post-pandemic recovery	Cytology (Pap)	35-39	39	Not performed	Satisfactory	NILM	NILM	Negative (NILM)	
P11326	2023	2023-Q3	2023-09	Post-pandemic recovery	Cytology (Pap)	40-44	40	Not performed	Satisfactory	NILM	NILM	Negative (NILM)	
P11327	2023	2023-Q3	2023-09	Post-pandemic recovery	Cytology (Pap)	35-39	35	Not performed	Satisfactory	NILM	NILM	Negative (NILM)	
P11328	2023	2023-Q3	2023-09	Post-pandemic recovery	Cytology (Pap)	35-39	37	Not performed	Satisfactory	NILM	NILM	Negative (NILM)	
P11329	2023	2023-Q3	2023-09	Post-pandemic recovery	Cytology (Pap)	45-49	45	Not performed	Satisfactory	NILM	NILM	Negative (NILM)	Trichomonas
P11330	2023	2023-Q3	2023-09	Post-pandemic recovery	Cytology (Pap)	50-54	50	Not performed	Satisfactory	NILM	NILM	Negative (NILM)	
P11331	2023	2023-Q3	2023-09	Post-pandemic recovery	Cytology (Pap)	30-34	31	Not performed	Satisfactory	NILM	NILM	Negative (NILM)	
P11332	2023	2023-Q3	2023-09	Post-pandemic recovery	Cytology (Pap)	40-44	40	Not performed	Satisfactory	NILM	NILM	Negative (NILM)	
P11333	2023	2023-Q3	2023-09	Post-pandemic recovery	Cytology (Pap)	45-49	46	Not performed	Satisfactory	NILM	NILM	Negative (NILM)	
P11334	2023	2023-Q3	2023-09	Post-pandemic recovery	Cytology (Pap)	45-49	46	Not performed	Satisfactory	NILM	NILM	Negative (NILM)	
P11335	2023	2023-Q3	2023-09	Post-pandemic recovery	Cytology (Pap)	40-44	42	Not performed	Unsatisfactory	Unsatisfactory	Unsatisfactory	Unsatisfactory	
P11336	2023	2023-Q3	2023-09	Post-pandemic recovery	Cytology (Pap)	35-39	38	Not performed	Satisfactory	NILM	NILM	Negative (NILM)	
P11337	2023	2023-Q3	2023-09	Post-pandemic recovery	Cytology (Pap)	40-44	40	Not performed	Unsatisfactory	Unsatisfactory	Unsatisfactory	Unsatisfactory	
P11338	2023	2023-Q3	2023-09	Post-pandemic recovery	Cytology (Pap)	45-49	45	Not performed	Satisfactory	NILM	NILM	Negative (NILM)	
P11339	2023	2023-Q3	2023-09	Post-pandemic recovery	Cytology (Pap)	35-39	37	Not performed	Satisfactory	NILM	NILM	Negative (NILM)	Candida
P11340	2023	2023-Q3	2023-09	Post-pandemic recovery	Cytology (Pap)	55-59	56	Not performed	Satisfactory	Positive	HSIL	High-grade squamous	
P11341	2023	2023-Q3	2023-09	Post-pandemic recovery	Cytology (Pap)	45-49	47	Not performed	Satisfactory	NILM	NILM	Negative (NILM)	
P11342	2023	2023-Q3	2023-09	Post-pandemic recovery	Cytology (Pap)	25-29	29	Not performed	Satisfactory	NILM	NILM	Negative (NILM)	
P11343	2023	2023-Q3	2023-09	Post-pandemic recovery	Cytology (Pap)	35-39	36	Not performed	Satisfactory	NILM	NILM	Negative (NILM)	
P11344	2023	2023-Q3	2023-09	Post-pandemic recovery	Cytology (Pap)	50-54	54	Not performed	Satisfactory	NILM	NILM	Negative (NILM)	
P11345	2023	2023-Q3	2023-09	Post-pandemic recovery	Cytology (Pap)	60-64	60	Not performed	Satisfactory	NILM	NILM	Negative (NILM)	
P11346	2023	2023-Q3	2023-09	Post-pandemic recovery	Cytology (Pap)	50-54	54	Not performed	Satisfactory	NILM	NILM	Negative (NILM)	Gardnerella
P11347	2023	2023-Q3	2023-09	Post-pandemic recovery	Cytology (Pap)	35-39	36	Not performed	Satisfactory	NILM	NILM	Negative (NILM)	
P11348	2023	2023-Q3	2023-09	Post-pandemic recovery	Cytology (Pap)	40-44	40	Not performed	Satisfactory	NILM	NILM	Negative (NILM)	
P11349	2023	2023-Q3	2023-09	Post-pandemic recovery	Cytology (Pap)	30-34	34	Not performed	Satisfactory	NILM	NILM	Negative (NILM)	
P11350	2023	2023-Q3	2023-09	Post-pandemic recovery	Cytology (Pap)	40-44	43	Not performed	Satisfactory	NILM	NILM	Negative (NILM)	
P11351	2023	2023-Q3	2023-09	Post-pandemic recovery	Cytology (Pap)	40-44	44	Not performed	Satisfactory	NILM	NILM	Negative (NILM)	
P11352	2023	2023-Q3	2023-09	Post-pandemic recovery	Cytology (Pap)	50-54	54	Not performed	Satisfactory	NILM	NILM	Negative (NILM)	
P11353	2023	2023-Q3	2023-09	Post-pandemic recovery	Cytology (Pap)	40-44	40	Not performed	Satisfactory	NILM	NILM	Negative (NILM)	
P11354	2023	2023-Q3	2023-09	Post-pandemic recovery	Cytology (Pap)	30-34	34	Not performed	Satisfactory	NILM	NILM	Negative (NILM)	
P11355	2023	2023-Q3	2023-09	Post-pandemic recovery	Cytology (Pap)	60-64	63	Not performed	Satisfactory	NILM	NILM	Negative (NILM)	
P11356	2023	2023-Q3	2023-09	Post-pandemic recovery	Cytology (Pap)	25-29	29	Not performed	Satisfactory	NILM	NILM	Negative (NILM)	
P11357	2023	2023-Q3	2023-09	Post-pandemic recovery	Cytology (Pap)	50-54	52	Not performed	Satisfactory	NILM	NILM	Negative (NILM)	
P11358	2023	2023-Q3	2023-09	Post-pandemic recovery	Cytology (Pap)	55-59	59	Not performed	Satisfactory	NILM	NILM	Negative (NILM)	

patient_id	year	quarter	month	period	primary_test	age_group	age_years	hpv_result	adequacy	result	bethesda	grade	infection
P11359	2023	2023-Q3	2023-09	Post-pandemic recovery	Cytology (Pap)	45-49	45	Not performed	Satisfactory	NILM	NILM	Negative (NILM)	
P11360	2023	2023-Q3	2023-09	Post-pandemic recovery	Cytology (Pap)	40-44	44	Not performed	Satisfactory	NILM	NILM	Negative (NILM)	
P11361	2023	2023-Q3	2023-09	Post-pandemic recovery	Cytology (Pap)	25-29	25	Not performed	Satisfactory	NILM	NILM	Negative (NILM)	
P11362	2023	2023-Q3	2023-09	Post-pandemic recovery	Cytology (Pap)	55-59	57	Not performed	Satisfactory	NILM	NILM	Negative (NILM)	
P11363	2023	2023-Q3	2023-09	Post-pandemic recovery	Cytology (Pap)	40-44	40	Not performed	Satisfactory	NILM	NILM	Negative (NILM)	
P11364	2023	2023-Q3	2023-09	Post-pandemic recovery	Cytology (Pap)	40-44	42	Not performed	Satisfactory	NILM	NILM	Negative (NILM)	
P11365	2023	2023-Q3	2023-09	Post-pandemic recovery	Cytology (Pap)	50-54	50	Not performed	Satisfactory	Positive	HSIL	High-grade squamous	
P11366	2023	2023-Q3	2023-09	Post-pandemic recovery	Cytology (Pap)	55-59	59	Not performed	Satisfactory	NILM	NILM	Negative (NILM)	
P11367	2023	2023-Q3	2023-09	Post-pandemic recovery	Cytology (Pap)	45-49	45	Not performed	Unsatisfactory	Unsatisfactory	Unsatisfactory	Unsatisfactory	
P11368	2023	2023-Q3	2023-09	Post-pandemic recovery	Cytology (Pap)	35-39	35	Not performed	Satisfactory	NILM	NILM	Negative (NILM)	
P11369	2023	2023-Q3	2023-09	Post-pandemic recovery	Cytology (Pap)	50-54	54	Not performed	Satisfactory	Positive	ASC-US	Low-grade / equivocal squamous	
P11370	2023	2023-Q3	2023-09	Post-pandemic recovery	Cytology (Pap)	50-54	51	Not performed	Satisfactory	Positive	ASC-US	Low-grade / equivocal squamous	
P11371	2023	2023-Q3	2023-09	Post-pandemic recovery	Cytology (Pap)	50-54	54	Not performed	Satisfactory	NILM	NILM	Negative (NILM)	
P11372	2023	2023-Q3	2023-09	Post-pandemic recovery	Cytology (Pap)	55-59	56	Not performed	Satisfactory	NILM	NILM	Negative (NILM)	
P11373	2023	2023-Q3	2023-09	Post-pandemic recovery	Cytology (Pap)	40-44	41	Not performed	Satisfactory	Positive	LSIL	Low-grade / equivocal squamous	
P11374	2023	2023-Q3	2023-09	Post-pandemic recovery	Cytology (Pap)	60-64	61	Not performed	Satisfactory	NILM	NILM	Negative (NILM)	Candida
P11375	2023	2023-Q3	2023-09	Post-pandemic recovery	Cytology (Pap)	35-39	35	Not performed	Satisfactory	Positive	HSIL	High-grade squamous	
P11376	2023	2023-Q3	2023-09	Post-pandemic recovery	Cytology (Pap)	50-54	54	Not performed	Satisfactory	NILM	NILM	Negative (NILM)	
P11377	2023	2023-Q3	2023-09	Post-pandemic recovery	Cytology (Pap)	35-39	38	Not performed	Satisfactory	NILM	NILM	Negative (NILM)	
P11378	2023	2023-Q3	2023-09	Post-pandemic recovery	Cytology (Pap)	55-59	56	Not performed	Satisfactory	NILM	NILM	Negative (NILM)	
P11379	2023	2023-Q3	2023-09	Post-pandemic recovery	Cytology (Pap)	35-39	38	Not performed	Satisfactory	NILM	NILM	Negative (NILM)	
P11380	2023	2023-Q3	2023-09	Post-pandemic recovery	Cytology (Pap)	40-44	43	Not performed	Satisfactory	NILM	NILM	Negative (NILM)	
P11381	2023	2023-Q3	2023-09	Post-pandemic recovery	Cytology (Pap)	40-44	43	Not performed	Satisfactory	NILM	NILM	Negative (NILM)	
P11382	2023	2023-Q3	2023-09	Post-pandemic recovery	Cytology (Pap)	45-49	45	Not performed	Satisfactory	NILM	NILM	Negative (NILM)	
P11383	2023	2023-Q3	2023-09	Post-pandemic recovery	Cytology (Pap)	50-54	53	Not performed	Satisfactory	NILM	NILM	Negative (NILM)	
P11384	2023	2023-Q3	2023-09	Post-pandemic recovery	Cytology (Pap)	45-49	49	Not performed	Satisfactory	NILM	NILM	Negative (NILM)	
P11385	2023	2023-Q3	2023-09	Post-pandemic recovery	Cytology (Pap)	40-44	44	Not performed	Satisfactory	NILM	NILM	Negative (NILM)	
P11386	2023	2023-Q3	2023-09	Post-pandemic recovery	Cytology (Pap)	55-59	56	Not performed	Satisfactory	Positive	LSIL	Low-grade / equivocal squamous	
P11387	2023	2023-Q3	2023-09	Post-pandemic recovery	Cytology (Pap)	45-49	46	Not performed	Satisfactory	NILM	NILM	Negative (NILM)	
P11388	2023	2023-Q3	2023-09	Post-pandemic recovery	Cytology (Pap)	25-29	26	Not performed	Satisfactory	Positive	LSIL	Low-grade / equivocal squamous	
P11389	2023	2023-Q3	2023-09	Post-pandemic recovery	Cytology (Pap)	25-29	28	Not performed	Satisfactory	NILM	NILM	Negative (NILM)	
P11390	2023	2023-Q3	2023-09	Post-pandemic recovery	Cytology (Pap)	60-64	60	Not performed	Satisfactory	NILM	NILM	Negative (NILM)	
P11391	2023	2023-Q3	2023-09	Post-pandemic recovery	Cytology (Pap)	45-49	45	Not performed	Satisfactory	NILM	NILM	Negative (NILM)	
P11392	2023	2023-Q3	2023-09	Post-pandemic recovery	Cytology (Pap)	50-54	53	Not performed	Satisfactory	NILM	NILM	Negative (NILM)	
P11393	2023	2023-Q3	2023-09	Post-pandemic recovery	Cytology (Pap)	45-49	47	Not performed	Unsatisfactory	Unsatisfactory	Unsatisfactory	Unsatisfactory	
P11394	2023	2023-Q3	2023-09	Post-pandemic recovery	Cytology (Pap)	50-54	50	Not performed	Satisfactory	NILM	NILM	Negative (NILM)	
P11395	2023	2023-Q3	2023-09	Post-pandemic recovery	Cytology (Pap)	50-54	51	Not performed	Satisfactory	Positive	AGC	Glandular	
P11396	2023	2023-Q3	2023-09	Post-pandemic recovery	Cytology (Pap)	55-59	58	Not performed	Satisfactory	NILM	NILM	Negative (NILM)	
P11397	2023	2023-Q3	2023-09	Post-pandemic recovery	Cytology (Pap)	40-44	43	Not performed	Satisfactory	NILM	NILM	Negative (NILM)	
P11398	2023	2023-Q3	2023-09	Post-pandemic recovery	Cytology (Pap)	30-34	30	Not performed	Satisfactory	NILM	NILM	Negative (NILM)	Trichomonas
P11399	2023	2023-Q3	2023-09	Post-pandemic recovery	Cytology (Pap)	55-59	56	Not performed	Satisfactory	NILM	NILM	Negative (NILM)	
P11400	2023	2023-Q3	2023-09	Post-pandemic recovery	Cytology (Pap)	40-44	40	Not performed	Satisfactory	NILM	NILM	Negative (NILM)	
P11401	2023	2023-Q3	2023-09	Post-pandemic recovery	Cytology (Pap)	30-34	31	Not performed	Satisfactory	NILM	NILM	Negative (NILM)	Gardnerella
P11402	2023	2023-Q3	2023-09	Post-pandemic recovery	Cytology (Pap)	30-34	32	Not performed	Unsatisfactory	Unsatisfactory	Unsatisfactory	Unsatisfactory	
P11403	2023	2023-Q3	2023-09	Post-pandemic recovery	Cytology (Pap)	35-39	35	Not performed	Satisfactory	NILM	NILM	Negative (NILM)	
P11404	2023	2023-Q3	2023-09	Post-pandemic recovery	Cytology (Pap)	60-64	63	Not performed	Satisfactory	NILM	NILM	Negative (NILM)	
P11405	2023	2023-Q3	2023-09	Post-pandemic recovery	Cytology (Pap)	40-44	43	Not performed	Satisfactory	NILM	NILM	Negative (NILM)	
P11406	2023	2023-Q3	2023-09	Post-pandemic recovery	Cytology (Pap)	30-34	34	Not performed	Satisfactory	NILM	NILM	Negative (NILM)	
P11407	2023	2023-Q3	2023-09	Post-pandemic recovery	Cytology (Pap)	35-39	38	Not performed	Satisfactory	NILM	NILM	Negative (NILM)	
P11408	2023	2023-Q3	2023-09	Post-pandemic recovery	Cytology (Pap)	60-64	61	Not performed	Satisfactory	NILM	NILM	Negative (NILM)	
P11409	2023	2023-Q3	2023-09	Post-pandemic recovery	Cytology (Pap)	40-44	42	Not performed	Satisfactory	Positive	ASC-US	Low-grade / equivocal squamous	Gardnerella
P11410	2023	2023-Q3	2023-09	Post-pandemic recovery	Cytology (Pap)	35-39	38	Not performed	Unsatisfactory	Unsatisfactory	Unsatisfactory	Unsatisfactory	
P11411	2023	2023-Q3	2023-09	Post-pandemic recovery	Cytology (Pap)	60-64	64	Not performed	Satisfactory	NILM	NILM	Negative (NILM)	
P11412	2023	2023-Q3	2023-09	Post-pandemic recovery	Cytology (Pap)	50-54	52	Not performed	Satisfactory	NILM	NILM	Negative (NILM)	
P11413	2023	2023-Q3	2023-09	Post-pandemic recovery	Cytology (Pap)	40-44	41	Not performed	Satisfactory	NILM	NILM	Negative (NILM)	
P11414	2023	2023-Q3	2023-09	Post-pandemic recovery	Cytology (Pap)	35-39	35	Not performed	Unsatisfactory	Unsatisfactory	Unsatisfactory	Unsatisfactory	
P11415	2023	2023-Q3	2023-09	Post-pandemic recovery	Cytology (Pap)	40-44	40	Not performed	Satisfactory	NILM	NILM	Negative (NILM)	
P11416	2023	2023-Q3	2023-09	Post-pandemic recovery	Cytology (Pap)	40-44	43	Not performed	Satisfactory	NILM	NILM	Negative (NILM)	
P11417	2023	2023-Q3	2023-09	Post-pandemic recovery	Cytology (Pap)	35-39	35	Not performed	Satisfactory	NILM	NILM	Negative (NILM)	
P11418	2023	2023-Q3	2023-09	Post-pandemic recovery	Cytology (Pap)	35-39	36	Not performed	Satisfactory	NILM	NILM	Negative (NILM)	
P11419	2023	2023-Q3	2023-09	Post-pandemic recovery	Cytology (Pap)	45-49	46	Not performed	Satisfactory	NILM	NILM	Negative (NILM)	
P11420	2023	2023-Q3	2023-09	Post-pandemic recovery	Cytology (Pap)	50-54	52	Not performed	Satisfactory	Positive	AGC	Glandular	
P11421	2023	2023-Q3	2023-09	Post-pandemic recovery	Cytology (Pap)	30-34	33	Not performed	Satisfactory	NILM	NILM	Negative (NILM)	
P11422	2023	2023-Q3	2023-09	Post-pandemic recovery	Cytology (Pap)	45-49	46	Not performed	Unsatisfactory	Unsatisfactory	Unsatisfactory	Unsatisfactory	
P11423	2023	2023-Q3	2023-09	Post-pandemic recovery	Cytology (Pap)	30-34	32	Not performed	Satisfactory	NILM	NILM	Negative (NILM)	
P11424	2023	2023-Q3	2023-09	Post-pandemic recovery	Cytology (Pap)	50-54	50	Not performed	Satisfactory	Positive	ASC-H	High-grade squamous	
P11425	2023	2023-Q3	2023-09	Post-pandemic recovery	Cytology (Pap)	40-44	40	Not performed	Satisfactory	NILM	NILM	Negative (NILM)	
P11426	2023	2023-Q3	2023-09	Post-pandemic recovery	Cytology (Pap)	35-39	37	Not performed	Satisfactory	NILM	NILM	Negative (NILM)	
P11427	2023	2023-Q3	2023-09	Post-pandemic recovery	Cytology (Pap)	35-39	35	Not performed	Satisfactory	NILM	NILM	Negative (NILM)	
P11428	2023	2023-Q3	2023-09	Post-pandemic recovery	Cytology (Pap)	45-49	47	Not performed	Satisfactory	NILM	NILM	Negative (NILM)	
P11429	2023	2023-Q3	2023-09	Post-pandemic recovery	Cytology (Pap)	30-34	34	Not performed	Satisfactory	NILM	NILM	Negative (NILM)	
P11430	2023	2023-Q3	2023-09	Post-pandemic recovery	Cytology (Pap)	60-64	64	Not performed	Satisfactory	NILM	NILM	Negative (NILM)	
P11431	2023	2023-Q3	2023-09	Post-pandemic recovery	Cytology (Pap)	30-34	33	Not performed	Satisfactory	NILM	NILM	Negative (NILM)	
P11432	2023	2023-Q3	2023-09	Post-pandemic recovery	Cytology (Pap)	30-34	32	Not performed	Unsatisfactory	Unsatisfactory	Unsatisfactory	Unsatisfactory	
P11433	2023	2023-Q3	2023-09	Post-pandemic recovery	Cytology (Pap)	40-44	40	Not performed	Satisfactory	NILM	NILM	Negative (NILM)	
P11434	2023	2023-Q3	2023-09	Post-pandemic recovery	Cytology (Pap)	40-44	41	Not performed	Satisfactory	Positive	HSIL	High-grade squamous	
P11435	2023	2023-Q3	2023-09	Post-pandemic recovery	Cytology (Pap)	35-39	38	Not performed	Satisfactory	NILM	NILM	Negative (NILM)	
P11436	2023	2023-Q3	2023-09	Post-pandemic recovery	Cytology (Pap)	50-54	51	Not performed	Satisfactory	NILM	NILM	Negative (NILM)	
P11437	2023	2023-Q3	2023-09	Post-pandemic recovery	Cytology (Pap)	50-54	54	Not performed	Satisfactory	NILM	NILM	Negative (NILM)	
P11438	2023	2023-Q3	2023-09	Post-pandemic recovery	Cytology (Pap)	35-39	35	Not performed	Satisfactory	NILM	NILM	Negative (NILM)	

patient_id	year	quarter	month	period	primary_test	age_group	age_years	hpv_result	adequacy	result	bethesda	grade	infection
P11439	2023	2023-Q3	2023-09	Post-pandemic recovery	Cytology (Pap)	40-44	43	Not performed	Unsatisfactory	Unsatisfactory	Unsatisfactory	Unsatisfactory	
P11440	2023	2023-Q3	2023-09	Post-pandemic recovery	Cytology (Pap)	55-59	56	Not performed	Satisfactory	Positive	AGC	Glandular	
P11441	2023	2023-Q3	2023-09	Post-pandemic recovery	Cytology (Pap)	50-54	54	Not performed	Satisfactory	Positive	ASC-US	Low-grade / equivocal squamous	
P11442	2023	2023-Q3	2023-09	Post-pandemic recovery	Cytology (Pap)	35-39	37	Not performed	Satisfactory	NILM	NILM	Negative (NILM)	
P11443	2023	2023-Q3	2023-09	Post-pandemic recovery	Cytology (Pap)	40-44	44	Not performed	Satisfactory	NILM	NILM	Negative (NILM)	
P11444	2023	2023-Q3	2023-09	Post-pandemic recovery	Cytology (Pap)	50-54	54	Not performed	Satisfactory	NILM	NILM	Negative (NILM)	
P11445	2023	2023-Q3	2023-09	Post-pandemic recovery	Cytology (Pap)	35-39	35	Not performed	Satisfactory	NILM	NILM	Negative (NILM)	
P11446	2023	2023-Q3	2023-09	Post-pandemic recovery	Cytology (Pap)	50-54	54	Not performed	Satisfactory	NILM	NILM	Negative (NILM)	
P11447	2023	2023-Q3	2023-09	Post-pandemic recovery	Cytology (Pap)	50-54	52	Not performed	Satisfactory	Positive	LSIL	Low-grade / equivocal squamous	
P11448	2023	2023-Q3	2023-09	Post-pandemic recovery	Cytology (Pap)	55-59	56	Not performed	Satisfactory	NILM	NILM	Negative (NILM)	
P11449	2023	2023-Q3	2023-09	Post-pandemic recovery	Cytology (Pap)	40-44	44	Not performed	Satisfactory	NILM	NILM	Negative (NILM)	
P11450	2023	2023-Q3	2023-09	Post-pandemic recovery	Cytology (Pap)	45-49	48	Not performed	Satisfactory	NILM	NILM	Negative (NILM)	
P11451	2023	2023-Q3	2023-09	Post-pandemic recovery	Cytology (Pap)	45-49	45	Not performed	Satisfactory	NILM	NILM	Negative (NILM)	
P11452	2023	2023-Q3	2023-09	Post-pandemic recovery	Cytology (Pap)	30-34	33	Not performed	Satisfactory	NILM	NILM	Negative (NILM)	
P11453	2023	2023-Q3	2023-09	Post-pandemic recovery	Cytology (Pap)	30-34	33	Not performed	Satisfactory	NILM	NILM	Negative (NILM)	
P11454	2023	2023-Q3	2023-09	Post-pandemic recovery	Cytology (Pap)	30-34	31	Not performed	Satisfactory	NILM	NILM	Negative (NILM)	
P11455	2023	2023-Q3	2023-09	Post-pandemic recovery	Cytology (Pap)	40-44	40	Not performed	Satisfactory	Positive	HSIL	High-grade squamous	
P11456	2023	2023-Q3	2023-09	Post-pandemic recovery	Cytology (Pap)	35-39	37	Not performed	Satisfactory	NILM	NILM	Negative (NILM)	
P11457	2023	2023-Q3	2023-09	Post-pandemic recovery	Cytology (Pap)	30-34	30	Not performed	Satisfactory	Positive	ASC-H	High-grade squamous	
P11458	2023	2023-Q3	2023-09	Post-pandemic recovery	Cytology (Pap)	45-49	47	Not performed	Satisfactory	Positive	ASC-US	Low-grade / equivocal squamous	
P11459	2023	2023-Q3	2023-09	Post-pandemic recovery	Cytology (Pap)	40-44	42	Not performed	Satisfactory	NILM	NILM	Negative (NILM)	
P11460	2023	2023-Q3	2023-09	Post-pandemic recovery	Cytology (Pap)	30-34	32	Not performed	Satisfactory	NILM	NILM	Negative (NILM)	
P11461	2023	2023-Q3	2023-09	Post-pandemic recovery	Cytology (Pap)	30-34	30	Not performed	Satisfactory	Positive	ASC-US	Low-grade / equivocal squamous	
P11462	2023	2023-Q3	2023-09	Post-pandemic recovery	Cytology (Pap)	40-44	43	Not performed	Satisfactory	NILM	NILM	Negative (NILM)	
P11463	2023	2023-Q3	2023-09	Post-pandemic recovery	Cytology (Pap)	30-34	31	Not performed	Satisfactory	NILM	NILM	Negative (NILM)	
P11464	2023	2023-Q3	2023-09	Post-pandemic recovery	Cytology (Pap)	55-59	57	Not performed	Satisfactory	NILM	NILM	Negative (NILM)	Candida
P11465	2023	2023-Q3	2023-09	Post-pandemic recovery	Cytology (Pap)	40-44	40	Not performed	Satisfactory	Positive	ASC-US	Low-grade / equivocal squamous	
P11466	2023	2023-Q3	2023-09	Post-pandemic recovery	Cytology (Pap)	25-29	29	Not performed	Unsatisfactory	Unsatisfactory	Unsatisfactory	Unsatisfactory	
P11467	2023	2023-Q3	2023-09	Post-pandemic recovery	Cytology (Pap)	40-44	40	Not performed	Satisfactory	NILM	NILM	Negative (NILM)	
P11468	2023	2023-Q4	2023-10	Post-pandemic recovery	Cytology (Pap)	35-39	35	Not performed	Satisfactory	NILM	NILM	Negative (NILM)	
P11469	2023	2023-Q4	2023-10	Post-pandemic recovery	Cytology (Pap)	30-34	32	Not performed	Satisfactory	NILM	NILM	Negative (NILM)	
P11470	2023	2023-Q4	2023-10	Post-pandemic recovery	Cytology (Pap)	55-59	57	Not performed	Satisfactory	NILM	NILM	Negative (NILM)	
P11471	2023	2023-Q4	2023-10	Post-pandemic recovery	Cytology (Pap)	40-44	44	Not performed	Satisfactory	NILM	NILM	Negative (NILM)	
P11472	2023	2023-Q4	2023-10	Post-pandemic recovery	Cytology (Pap)	55-59	58	Not performed	Satisfactory	NILM	NILM	Negative (NILM)	
P11473	2023	2023-Q4	2023-10	Post-pandemic recovery	Cytology (Pap)	60-64	60	Not performed	Satisfactory	NILM	NILM	Negative (NILM)	
P11474	2023	2023-Q4	2023-10	Post-pandemic recovery	Cytology (Pap)	30-34	32	Not performed	Unsatisfactory	Unsatisfactory	Unsatisfactory	Unsatisfactory	
P11475	2023	2023-Q4	2023-10	Post-pandemic recovery	Cytology (Pap)	45-49	49	Not performed	Satisfactory	NILM	NILM	Negative (NILM)	
P11476	2023	2023-Q4	2023-10	Post-pandemic recovery	Cytology (Pap)	35-39	37	Not performed	Satisfactory	NILM	NILM	Negative (NILM)	Candida
P11477	2023	2023-Q4	2023-10	Post-pandemic recovery	Cytology (Pap)	25-29	29	Not performed	Satisfactory	NILM	NILM	Negative (NILM)	
P11478	2023	2023-Q4	2023-10	Post-pandemic recovery	Cytology (Pap)	30-34	31	Not performed	Satisfactory	NILM	NILM	Negative (NILM)	
P11479	2023	2023-Q4	2023-10	Post-pandemic recovery	Cytology (Pap)	60-64	62	Not performed	Satisfactory	NILM	NILM	Negative (NILM)	
P11480	2023	2023-Q4	2023-10	Post-pandemic recovery	Cytology (Pap)	40-44	44	Not performed	Satisfactory	NILM	NILM	Negative (NILM)	
P11481	2023	2023-Q4	2023-10	Post-pandemic recovery	Cytology (Pap)	35-39	39	Not performed	Satisfactory	NILM	NILM	Negative (NILM)	Other
P11482	2023	2023-Q4	2023-10	Post-pandemic recovery	Cytology (Pap)	30-34	31	Not performed	Satisfactory	NILM	NILM	Negative (NILM)	
P11483	2023	2023-Q4	2023-10	Post-pandemic recovery	Cytology (Pap)	55-59	55	Not performed	Satisfactory	NILM	NILM	Negative (NILM)	
P11484	2023	2023-Q4	2023-10	Post-pandemic recovery	Cytology (Pap)	35-39	39	Not performed	Satisfactory	NILM	NILM	Negative (NILM)	
P11485	2023	2023-Q4	2023-10	Post-pandemic recovery	Cytology (Pap)	50-54	50	Not performed	Satisfactory	NILM	NILM	Negative (NILM)	
P11486	2023	2023-Q4	2023-10	Post-pandemic recovery	Cytology (Pap)	35-39	37	Not performed	Satisfactory	NILM	NILM	Negative (NILM)	
P11487	2023	2023-Q4	2023-10	Post-pandemic recovery	Cytology (Pap)	30-34	34	Not performed	Satisfactory	NILM	NILM	Negative (NILM)	
P11488	2023	2023-Q4	2023-10	Post-pandemic recovery	Cytology (Pap)	45-49	45	Not performed	Satisfactory	NILM	NILM	Negative (NILM)	
P11489	2023	2023-Q4	2023-10	Post-pandemic recovery	Cytology (Pap)	55-59	57	Not performed	Satisfactory	NILM	NILM	Negative (NILM)	
P11490	2023	2023-Q4	2023-10	Post-pandemic recovery	Cytology (Pap)	30-34	31	Not performed	Satisfactory	NILM	NILM	Negative (NILM)	
P11491	2023	2023-Q4	2023-10	Post-pandemic recovery	Cytology (Pap)	50-54	50	Not performed	Satisfactory	NILM	NILM	Negative (NILM)	
P11492	2023	2023-Q4	2023-10	Post-pandemic recovery	Cytology (Pap)	45-49	45	Not performed	Satisfactory	Positive	LSIL	Low-grade / equivocal squamous	
P11493	2023	2023-Q4	2023-10	Post-pandemic recovery	Cytology (Pap)	45-49	45	Not performed	Satisfactory	Positive	LSIL	Low-grade / equivocal squamous	
P11494	2023	2023-Q4	2023-10	Post-pandemic recovery	Cytology (Pap)	45-49	49	Not performed	Satisfactory	NILM	NILM	Negative (NILM)	
P11495	2023	2023-Q4	2023-10	Post-pandemic recovery	Cytology (Pap)	35-39	37	Not performed	Satisfactory	Positive	ASC-US	Low-grade / equivocal squamous	
P11496	2023	2023-Q4	2023-10	Post-pandemic recovery	Cytology (Pap)	40-44	40	Not performed	Satisfactory	NILM	NILM	Negative (NILM)	
P11497	2023	2023-Q4	2023-10	Post-pandemic recovery	Cytology (Pap)	35-39	38	Not performed	Satisfactory	NILM	NILM	Negative (NILM)	Trichomonas
P11498	2023	2023-Q4	2023-10	Post-pandemic recovery	Cytology (Pap)	40-44	44	Not performed	Satisfactory	NILM	NILM	Negative (NILM)	
P11499	2023	2023-Q4	2023-10	Post-pandemic recovery	Cytology (Pap)	25-29	27	Not performed	Satisfactory	NILM	NILM	Negative (NILM)	
P11500	2023	2023-Q4	2023-10	Post-pandemic recovery	Cytology (Pap)	25-29	28	Not performed	Unsatisfactory	Unsatisfactory	Unsatisfactory	Unsatisfactory	
P11501	2023	2023-Q4	2023-10	Post-pandemic recovery	Cytology (Pap)	35-39	37	Not performed	Satisfactory	Positive	ASC-US	Low-grade / equivocal squamous	
P11502	2023	2023-Q4	2023-10	Post-pandemic recovery	Cytology (Pap)	35-39	38	Not performed	Satisfactory	NILM	NILM	Negative (NILM)	
P11503	2023	2023-Q4	2023-10	Post-pandemic recovery	Cytology (Pap)	55-59	57	Not performed	Satisfactory	NILM	NILM	Negative (NILM)	Gardnerella
P11504	2023	2023-Q4	2023-10	Post-pandemic recovery	Cytology (Pap)	60-64	61	Not performed	Satisfactory	Positive	HSIL	High-grade squamous	Candida
P11505	2023	2023-Q4	2023-10	Post-pandemic recovery	Cytology (Pap)	50-54	53	Not performed	Satisfactory	Positive	AGC	Glandular	
P11506	2023	2023-Q4	2023-10	Post-pandemic recovery	Cytology (Pap)	35-39	37	Not performed	Satisfactory	NILM	NILM	Negative (NILM)	
P11507	2023	2023-Q4	2023-10	Post-pandemic recovery	Cytology (Pap)	40-44	44	Not performed	Satisfactory	NILM	NILM	Negative (NILM)	
P11508	2023	2023-Q4	2023-10	Post-pandemic recovery	Cytology (Pap)	35-39	36	Not performed	Satisfactory	NILM	NILM	Negative (NILM)	
P11509	2023	2023-Q4	2023-10	Post-pandemic recovery	Cytology (Pap)	50-54	54	Not performed	Satisfactory	NILM	NILM	Negative (NILM)	
P11510	2023	2023-Q4	2023-10	Post-pandemic recovery	Cytology (Pap)	35-39	39	Not performed	Satisfactory	Positive	ASC-US	Low-grade / equivocal squamous	
P11511	2023	2023-Q4	2023-10	Post-pandemic recovery	Cytology (Pap)	60-64	61	Not performed	Satisfactory	NILM	NILM	Negative (NILM)	
P11512	2023	2023-Q4	2023-10	Post-pandemic recovery	Cytology (Pap)	30-34	34	Not performed	Satisfactory	Positive	ASC-US	Low-grade / equivocal squamous	
P11513	2023	2023-Q4	2023-10	Post-pandemic recovery	Cytology (Pap)	35-39	36	Not performed	Satisfactory	Positive	ASC-US	Low-grade / equivocal squamous	
P11514	2023	2023-Q4	2023-10	Post-pandemic recovery	Cytology (Pap)	60-64	60	Not performed	Satisfactory	NILM	NILM	Negative (NILM)	
P11515	2023	2023-Q4	2023-10	Post-pandemic recovery	Cytology (Pap)	55-59	56	Not performed	Satisfactory	NILM	NILM	Negative (NILM)	
P11516	2023	2023-Q4	2023-10	Post-pandemic recovery	Cytology (Pap)	25-29	29	Not performed	Satisfactory	NILM	NILM	Negative (NILM)	
P11517	2023	2023-Q4	2023-10	Post-pandemic recovery	Cytology (Pap)	35-39	39	Not performed	Satisfactory	NILM	NILM	Negative (NILM)	
P11518	2023	2023-Q4	2023-10	Post-pandemic recovery	Cytology (Pap)	50-54	53	Not performed	Satisfactory	NILM	NILM	Negative (NILM)	

patient_id	year	quarter	month	period	primary_test	age_group	age_years	hpv_result	adequacy	result	bethesda	grade	infection
P11519	2023	2023-Q4	2023-10	Post-pandemic recovery	Cytology (Pap)	40-44	40	Not performed	Satisfactory	NILM	NILM	Negative (NILM)	
P11520	2023	2023-Q4	2023-10	Post-pandemic recovery	Cytology (Pap)	40-44	44	Not performed	Satisfactory	Positive	ASC-US	Low-grade / equivocal squamous	
P11521	2023	2023-Q4	2023-10	Post-pandemic recovery	Cytology (Pap)	35-39	37	Not performed	Satisfactory	NILM	NILM	Negative (NILM)	
P11522	2023	2023-Q4	2023-10	Post-pandemic recovery	Cytology (Pap)	50-54	51	Not performed	Satisfactory	NILM	NILM	Negative (NILM)	
P11523	2023	2023-Q4	2023-10	Post-pandemic recovery	Cytology (Pap)	50-54	52	Not performed	Satisfactory	NILM	NILM	Negative (NILM)	
P11524	2023	2023-Q4	2023-10	Post-pandemic recovery	Cytology (Pap)	25-29	26	Not performed	Satisfactory	NILM	NILM	Negative (NILM)	Candida
P11525	2023	2023-Q4	2023-10	Post-pandemic recovery	Cytology (Pap)	55-59	59	Not performed	Satisfactory	NILM	NILM	Negative (NILM)	
P11526	2023	2023-Q4	2023-10	Post-pandemic recovery	Cytology (Pap)	55-59	56	Not performed	Satisfactory	NILM	NILM	Negative (NILM)	
P11527	2023	2023-Q4	2023-10	Post-pandemic recovery	Cytology (Pap)	35-39	37	Not performed	Satisfactory	NILM	NILM	Negative (NILM)	
P11528	2023	2023-Q4	2023-10	Post-pandemic recovery	Cytology (Pap)	30-34	30	Not performed	Satisfactory	NILM	NILM	Negative (NILM)	
P11529	2023	2023-Q4	2023-10	Post-pandemic recovery	Cytology (Pap)	45-49	45	Not performed	Satisfactory	NILM	NILM	Negative (NILM)	
P11530	2023	2023-Q4	2023-10	Post-pandemic recovery	Cytology (Pap)	45-49	46	Not performed	Satisfactory	NILM	NILM	Negative (NILM)	
P11531	2023	2023-Q4	2023-10	Post-pandemic recovery	Cytology (Pap)	25-29	27	Not performed	Satisfactory	NILM	NILM	Negative (NILM)	
P11532	2023	2023-Q4	2023-10	Post-pandemic recovery	Cytology (Pap)	60-64	61	Not performed	Satisfactory	Positive	LSIL	Low-grade / equivocal squamous	
P11533	2023	2023-Q4	2023-10	Post-pandemic recovery	Cytology (Pap)	35-39	35	Not performed	Satisfactory	NILM	NILM	Negative (NILM)	
P11534	2023	2023-Q4	2023-10	Post-pandemic recovery	Cytology (Pap)	45-49	46	Not performed	Satisfactory	Positive	ASC-US	Low-grade / equivocal squamous	
P11535	2023	2023-Q4	2023-10	Post-pandemic recovery	Cytology (Pap)	45-49	48	Not performed	Satisfactory	NILM	NILM	Negative (NILM)	
P11536	2023	2023-Q4	2023-10	Post-pandemic recovery	Cytology (Pap)	35-39	39	Not performed	Satisfactory	NILM	NILM	Negative (NILM)	
P11537	2023	2023-Q4	2023-10	Post-pandemic recovery	Cytology (Pap)	35-39	37	Not performed	Satisfactory	NILM	NILM	Negative (NILM)	
P11538	2023	2023-Q4	2023-10	Post-pandemic recovery	Cytology (Pap)	35-39	35	Not performed	Satisfactory	Positive	ASC-US	Low-grade / equivocal squamous	
P11539	2023	2023-Q4	2023-10	Post-pandemic recovery	Cytology (Pap)	25-29	27	Not performed	Satisfactory	Positive	HSIL	High-grade squamous	
P11540	2023	2023-Q4	2023-10	Post-pandemic recovery	Cytology (Pap)	45-49	46	Not performed	Satisfactory	NILM	NILM	Negative (NILM)	
P11541	2023	2023-Q4	2023-10	Post-pandemic recovery	Cytology (Pap)	35-39	38	Not performed	Satisfactory	NILM	NILM	Negative (NILM)	
P11542	2023	2023-Q4	2023-10	Post-pandemic recovery	Cytology (Pap)	40-44	40	Not performed	Satisfactory	NILM	NILM	Negative (NILM)	
P11543	2023	2023-Q4	2023-10	Post-pandemic recovery	Cytology (Pap)	45-49	47	Not performed	Satisfactory	NILM	NILM	Negative (NILM)	
P11544	2023	2023-Q4	2023-10	Post-pandemic recovery	Cytology (Pap)	40-44	42	Not performed	Satisfactory	NILM	NILM	Negative (NILM)	
P11545	2023	2023-Q4	2023-10	Post-pandemic recovery	Cytology (Pap)	25-29	25	Not performed	Satisfactory	Positive	LSIL	Low-grade / equivocal squamous	Candida
P11546	2023	2023-Q4	2023-10	Post-pandemic recovery	Cytology (Pap)	50-54	53	Not performed	Satisfactory	NILM	NILM	Negative (NILM)	
P11547	2023	2023-Q4	2023-10	Post-pandemic recovery	Cytology (Pap)	55-59	57	Not performed	Satisfactory	Positive	HSIL	High-grade squamous	
P11548	2023	2023-Q4	2023-10	Post-pandemic recovery	Cytology (Pap)	60-64	64	Not performed	Satisfactory	NILM	NILM	Negative (NILM)	
P11549	2023	2023-Q4	2023-10	Post-pandemic recovery	Cytology (Pap)	60-64	60	Not performed	Satisfactory	NILM	NILM	Negative (NILM)	
P11550	2023	2023-Q4	2023-10	Post-pandemic recovery	Cytology (Pap)	40-44	41	Not performed	Satisfactory	NILM	NILM	Negative (NILM)	
P11551	2023	2023-Q4	2023-10	Post-pandemic recovery	Cytology (Pap)	30-34	30	Not performed	Satisfactory	NILM	NILM	Negative (NILM)	
P11552	2023	2023-Q4	2023-10	Post-pandemic recovery	Cytology (Pap)	30-34	32	Not performed	Satisfactory	NILM	NILM	Negative (NILM)	
P11553	2023	2023-Q4	2023-10	Post-pandemic recovery	Cytology (Pap)	60-64	62	Not performed	Satisfactory	NILM	NILM	Negative (NILM)	
P11554	2023	2023-Q4	2023-10	Post-pandemic recovery	Cytology (Pap)	30-34	33	Not performed	Satisfactory	NILM	NILM	Negative (NILM)	
P11555	2023	2023-Q4	2023-10	Post-pandemic recovery	Cytology (Pap)	30-34	32	Not performed	Satisfactory	Positive	LSIL	Low-grade / equivocal squamous	
P11556	2023	2023-Q4	2023-10	Post-pandemic recovery	Cytology (Pap)	60-64	63	Not performed	Satisfactory	NILM	NILM	Negative (NILM)	
P11557	2023	2023-Q4	2023-10	Post-pandemic recovery	Cytology (Pap)	25-29	27	Not performed	Satisfactory	NILM	NILM	Negative (NILM)	
P11558	2023	2023-Q4	2023-10	Post-pandemic recovery	Cytology (Pap)	35-39	38	Not performed	Satisfactory	NILM	NILM	Negative (NILM)	
P11559	2023	2023-Q4	2023-10	Post-pandemic recovery	Cytology (Pap)	60-64	61	Not performed	Satisfactory	NILM	NILM	Negative (NILM)	
P11560	2023	2023-Q4	2023-10	Post-pandemic recovery	Cytology (Pap)	25-29	26	Not performed	Satisfactory	NILM	NILM	Negative (NILM)	
P11561	2023	2023-Q4	2023-10	Post-pandemic recovery	Cytology (Pap)	45-49	45	Not performed	Satisfactory	NILM	NILM	Negative (NILM)	
P11562	2023	2023-Q4	2023-10	Post-pandemic recovery	Cytology (Pap)	50-54	52	Not performed	Satisfactory	Positive	ASC-US	Low-grade / equivocal squamous	
P11563	2023	2023-Q4	2023-10	Post-pandemic recovery	Cytology (Pap)	55-59	58	Not performed	Satisfactory	Positive	HSIL	High-grade squamous	
P11564	2023	2023-Q4	2023-10	Post-pandemic recovery	Cytology (Pap)	50-54	54	Not performed	Satisfactory	NILM	NILM	Negative (NILM)	
P11565	2023	2023-Q4	2023-10	Post-pandemic recovery	Cytology (Pap)	60-64	64	Not performed	Satisfactory	NILM	NILM	Negative (NILM)	
P11566	2023	2023-Q4	2023-10	Post-pandemic recovery	Cytology (Pap)	45-49	46	Not performed	Satisfactory	NILM	NILM	Negative (NILM)	Candida
P11567	2023	2023-Q4	2023-10	Post-pandemic recovery	Cytology (Pap)	45-49	46	Not performed	Unsatisfactory	Unsatisfactory	Unsatisfactory	Unsatisfactory	Trichomonas
P11568	2023	2023-Q4	2023-10	Post-pandemic recovery	Cytology (Pap)	40-44	44	Not performed	Satisfactory	NILM	NILM	Negative (NILM)	
P11569	2023	2023-Q4	2023-10	Post-pandemic recovery	Cytology (Pap)	40-44	41	Not performed	Satisfactory	NILM	NILM	Negative (NILM)	
P11570	2023	2023-Q4	2023-10	Post-pandemic recovery	Cytology (Pap)	35-39	38	Not performed	Satisfactory	NILM	NILM	Negative (NILM)	
P11571	2023	2023-Q4	2023-10	Post-pandemic recovery	Cytology (Pap)	30-34	32	Not performed	Satisfactory	NILM	NILM	Negative (NILM)	
P11572	2023	2023-Q4	2023-10	Post-pandemic recovery	Cytology (Pap)	50-54	51	Not performed	Satisfactory	NILM	NILM	Negative (NILM)	
P11573	2023	2023-Q4	2023-10	Post-pandemic recovery	Cytology (Pap)	60-64	62	Not performed	Unsatisfactory	Unsatisfactory	Unsatisfactory	Unsatisfactory	
P11574	2023	2023-Q4	2023-10	Post-pandemic recovery	Cytology (Pap)	45-49	48	Not performed	Satisfactory	Positive	ASC-US	Low-grade / equivocal squamous	Gardnerella
P11575	2023	2023-Q4	2023-10	Post-pandemic recovery	Cytology (Pap)	30-34	33	Not performed	Satisfactory	NILM	NILM	Negative (NILM)	
P11576	2023	2023-Q4	2023-10	Post-pandemic recovery	Cytology (Pap)	40-44	42	Not performed	Satisfactory	NILM	NILM	Negative (NILM)	
P11577	2023	2023-Q4	2023-10	Post-pandemic recovery	Cytology (Pap)	55-59	55	Not performed	Satisfactory	NILM	NILM	Negative (NILM)	
P11578	2023	2023-Q4	2023-10	Post-pandemic recovery	Cytology (Pap)	40-44	43	Not performed	Satisfactory	NILM	NILM	Negative (NILM)	
P11579	2023	2023-Q4	2023-10	Post-pandemic recovery	Cytology (Pap)	50-54	51	Not performed	Satisfactory	NILM	NILM	Negative (NILM)	
P11580	2023	2023-Q4	2023-10	Post-pandemic recovery	Cytology (Pap)	30-34	34	Not performed	Satisfactory	NILM	NILM	Negative (NILM)	
P11581	2023	2023-Q4	2023-10	Post-pandemic recovery	Cytology (Pap)	35-39	36	Not performed	Satisfactory	NILM	NILM	Negative (NILM)	Candida
P11582	2023	2023-Q4	2023-10	Post-pandemic recovery	Cytology (Pap)	40-44	40	Not performed	Satisfactory	NILM	NILM	Negative (NILM)	
P11583	2023	2023-Q4	2023-10	Post-pandemic recovery	Cytology (Pap)	35-39	39	Not performed	Satisfactory	NILM	NILM	Negative (NILM)	
P11584	2023	2023-Q4	2023-10	Post-pandemic recovery	Cytology (Pap)	35-39	35	Not performed	Satisfactory	NILM	NILM	Negative (NILM)	
P11585	2023	2023-Q4	2023-10	Post-pandemic recovery	Cytology (Pap)	30-34	30	Not performed	Unsatisfactory	Unsatisfactory	Unsatisfactory	Unsatisfactory	
P11586	2023	2023-Q4	2023-10	Post-pandemic recovery	Cytology (Pap)	45-49	45	Not performed	Satisfactory	NILM	NILM	Negative (NILM)	
P11587	2023	2023-Q4	2023-10	Post-pandemic recovery	Cytology (Pap)	60-64	62	Not performed	Satisfactory	NILM	NILM	Negative (NILM)	
P11588	2023	2023-Q4	2023-10	Post-pandemic recovery	Cytology (Pap)	30-34	32	Not performed	Satisfactory	NILM	NILM	Negative (NILM)	
P11589	2023	2023-Q4	2023-10	Post-pandemic recovery	Cytology (Pap)	50-54	54	Not performed	Satisfactory	NILM	NILM	Negative (NILM)	
P11590	2023	2023-Q4	2023-10	Post-pandemic recovery	Cytology (Pap)	45-49	47	Not performed	Satisfactory	NILM	NILM	Negative (NILM)	
P11591	2023	2023-Q4	2023-10	Post-pandemic recovery	Cytology (Pap)	40-44	43	Not performed	Satisfactory	NILM	NILM	Negative (NILM)	
P11592	2023	2023-Q4	2023-10	Post-pandemic recovery	Cytology (Pap)	45-49	47	Not performed	Satisfactory	Positive	ASC-US	Low-grade / equivocal squamous	
P11593	2023	2023-Q4	2023-10	Post-pandemic recovery	Cytology (Pap)	50-54	53	Not performed	Satisfactory	NILM	NILM	Negative (NILM)	
P11594	2023	2023-Q4	2023-10	Post-pandemic recovery	Cytology (Pap)	50-54	54	Not performed	Satisfactory	NILM	NILM	Negative (NILM)	
P11595	2023	2023-Q4	2023-10	Post-pandemic recovery	Cytology (Pap)	45-49	48	Not performed	Satisfactory	NILM	NILM	Negative (NILM)	
P11596	2023	2023-Q4	2023-10	Post-pandemic recovery	Cytology (Pap)	30-34	31	Not performed	Satisfactory	NILM	NILM	Negative (NILM)	
P11597	2023	2023-Q4	2023-10	Post-pandemic recovery	Cytology (Pap)	30-34	33	Not performed	Satisfactory	NILM	NILM	Negative (NILM)	
P11598	2023	2023-Q4	2023-10	Post-pandemic recovery	Cytology (Pap)	55-59	59	Not performed	Satisfactory	NILM	NILM	Negative (NILM)	

patient_id	year	quarter	month	period	primary_test	age_group	age_years	hpv_result	adequacy	result	bethesda	grade	infection
P11599	2023	2023-Q4	2023-10	Post-pandemic recovery	Cytology (Pap)	30-34	34	Not performed	Satisfactory	Positive	ASC-US	Low-grade / equivocal squamous	
P11600	2023	2023-Q4	2023-10	Post-pandemic recovery	Cytology (Pap)	30-34	30	Not performed	Unsatisfactory	Unsatisfactory	Unsatisfactory	Unsatisfactory	
P11601	2023	2023-Q4	2023-10	Post-pandemic recovery	Cytology (Pap)	45-49	48	Not performed	Satisfactory	NILM	NILM	Negative (NILM)	
P11602	2023	2023-Q4	2023-10	Post-pandemic recovery	Cytology (Pap)	55-59	57	Not performed	Satisfactory	NILM	NILM	Negative (NILM)	Candida
P11603	2023	2023-Q4	2023-10	Post-pandemic recovery	Cytology (Pap)	35-39	36	Not performed	Satisfactory	NILM	NILM	Negative (NILM)	
P11604	2023	2023-Q4	2023-10	Post-pandemic recovery	Cytology (Pap)	40-44	42	Not performed	Satisfactory	NILM	NILM	Negative (NILM)	Candida
P11605	2023	2023-Q4	2023-10	Post-pandemic recovery	Cytology (Pap)	50-54	52	Not performed	Satisfactory	NILM	NILM	Negative (NILM)	
P11606	2023	2023-Q4	2023-10	Post-pandemic recovery	Cytology (Pap)	25-29	29	Not performed	Satisfactory	NILM	NILM	Negative (NILM)	
P11607	2023	2023-Q4	2023-10	Post-pandemic recovery	Cytology (Pap)	60-64	64	Not performed	Satisfactory	Positive	LSIL	Low-grade / equivocal squamous	
P11608	2023	2023-Q4	2023-10	Post-pandemic recovery	Cytology (Pap)	25-29	25	Not performed	Satisfactory	NILM	NILM	Negative (NILM)	Candida
P11609	2023	2023-Q4	2023-10	Post-pandemic recovery	Cytology (Pap)	40-44	44	Not performed	Satisfactory	NILM	NILM	Negative (NILM)	
P11610	2023	2023-Q4	2023-10	Post-pandemic recovery	Cytology (Pap)	45-49	49	Not performed	Satisfactory	NILM	NILM	Negative (NILM)	
P11611	2023	2023-Q4	2023-10	Post-pandemic recovery	Cytology (Pap)	25-29	25	Not performed	Satisfactory	Positive	ASC-US	Low-grade / equivocal squamous	
P11612	2023	2023-Q4	2023-10	Post-pandemic recovery	Cytology (Pap)	25-29	28	Not performed	Satisfactory	NILM	NILM	Negative (NILM)	
P11613	2023	2023-Q4	2023-10	Post-pandemic recovery	Cytology (Pap)	40-44	40	Not performed	Satisfactory	NILM	NILM	Negative (NILM)	
P11614	2023	2023-Q4	2023-10	Post-pandemic recovery	Cytology (Pap)	55-59	59	Not performed	Satisfactory	NILM	NILM	Negative (NILM)	
P11615	2023	2023-Q4	2023-10	Post-pandemic recovery	Cytology (Pap)	40-44	41	Not performed	Satisfactory	NILM	NILM	Negative (NILM)	
P11616	2023	2023-Q4	2023-10	Post-pandemic recovery	Cytology (Pap)	35-39	39	Not performed	Satisfactory	NILM	NILM	Negative (NILM)	Gardnerella
P11617	2023	2023-Q4	2023-10	Post-pandemic recovery	Cytology (Pap)	40-44	43	Not performed	Satisfactory	NILM	NILM	Negative (NILM)	
P11618	2023	2023-Q4	2023-10	Post-pandemic recovery	Cytology (Pap)	50-54	54	Not performed	Satisfactory	Positive	AGC	Glandular	
P11619	2023	2023-Q4	2023-10	Post-pandemic recovery	Cytology (Pap)	50-54	50	Not performed	Satisfactory	NILM	NILM	Negative (NILM)	
P11620	2023	2023-Q4	2023-10	Post-pandemic recovery	Cytology (Pap)	35-39	39	Not performed	Satisfactory	Positive	LSIL	Low-grade / equivocal squamous	
P11621	2023	2023-Q4	2023-10	Post-pandemic recovery	Cytology (Pap)	45-49	49	Not performed	Unsatisfactory	Unsatisfactory	Unsatisfactory	Unsatisfactory	
P11622	2023	2023-Q4	2023-10	Post-pandemic recovery	Cytology (Pap)	35-39	35	Not performed	Satisfactory	Positive	LSIL	Low-grade / equivocal squamous	
P11623	2023	2023-Q4	2023-10	Post-pandemic recovery	Cytology (Pap)	35-39	36	Not performed	Satisfactory	Positive	LSIL	Low-grade / equivocal squamous	
P11624	2023	2023-Q4	2023-10	Post-pandemic recovery	Cytology (Pap)	50-54	54	Not performed	Satisfactory	NILM	NILM	Negative (NILM)	
P11625	2023	2023-Q4	2023-10	Post-pandemic recovery	Cytology (Pap)	45-49	48	Not performed	Satisfactory	NILM	NILM	Negative (NILM)	
P11626	2023	2023-Q4	2023-10	Post-pandemic recovery	Cytology (Pap)	50-54	52	Not performed	Satisfactory	NILM	NILM	Negative (NILM)	
P11627	2023	2023-Q4	2023-10	Post-pandemic recovery	Cytology (Pap)	40-44	40	Not performed	Satisfactory	Positive	ASC-US	Low-grade / equivocal squamous	
P11628	2023	2023-Q4	2023-10	Post-pandemic recovery	Cytology (Pap)	30-34	31	Not performed	Satisfactory	NILM	NILM	Negative (NILM)	Candida
P11629	2023	2023-Q4	2023-10	Post-pandemic recovery	Cytology (Pap)	45-49	48	Not performed	Satisfactory	NILM	NILM	Negative (NILM)	
P11630	2023	2023-Q4	2023-10	Post-pandemic recovery	Cytology (Pap)	35-39	37	Not performed	Satisfactory	NILM	NILM	Negative (NILM)	
P11631	2023	2023-Q4	2023-10	Post-pandemic recovery	Cytology (Pap)	40-44	43	Not performed	Satisfactory	NILM	NILM	Negative (NILM)	
P11632	2023	2023-Q4	2023-10	Post-pandemic recovery	Cytology (Pap)	40-44	44	Not performed	Satisfactory	Positive	LSIL	Low-grade / equivocal squamous	
P11633	2023	2023-Q4	2023-10	Post-pandemic recovery	Cytology (Pap)	55-59	57	Not performed	Satisfactory	NILM	NILM	Negative (NILM)	
P11634	2023	2023-Q4	2023-10	Post-pandemic recovery	Cytology (Pap)	30-34	33	Not performed	Satisfactory	NILM	NILM	Negative (NILM)	Candida
P11635	2023	2023-Q4	2023-10	Post-pandemic recovery	Cytology (Pap)	50-54	50	Not performed	Satisfactory	NILM	NILM	Negative (NILM)	
P11636	2023	2023-Q4	2023-10	Post-pandemic recovery	Cytology (Pap)	45-49	48	Not performed	Satisfactory	NILM	NILM	Negative (NILM)	
P11637	2023	2023-Q4	2023-10	Post-pandemic recovery	Cytology (Pap)	45-49	46	Not performed	Satisfactory	NILM	NILM	Negative (NILM)	
P11638	2023	2023-Q4	2023-10	Post-pandemic recovery	Cytology (Pap)	50-54	50	Not performed	Satisfactory	NILM	NILM	Negative (NILM)	
P11639	2023	2023-Q4	2023-10	Post-pandemic recovery	Cytology (Pap)	50-54	50	Not performed	Satisfactory	NILM	NILM	Negative (NILM)	
P11640	2023	2023-Q4	2023-10	Post-pandemic recovery	Cytology (Pap)	25-29	29	Not performed	Satisfactory	NILM	NILM	Negative (NILM)	
P11641	2023	2023-Q4	2023-10	Post-pandemic recovery	Cytology (Pap)	30-34	34	Not performed	Satisfactory	NILM	NILM	Negative (NILM)	
P11642	2023	2023-Q4	2023-10	Post-pandemic recovery	Cytology (Pap)	35-39	39	Not performed	Satisfactory	NILM	NILM	Negative (NILM)	
P11643	2023	2023-Q4	2023-10	Post-pandemic recovery	Cytology (Pap)	40-44	43	Not performed	Satisfactory	Positive	ASC-US	Low-grade / equivocal squamous	
P11644	2023	2023-Q4	2023-10	Post-pandemic recovery	Cytology (Pap)	30-34	33	Not performed	Unsatisfactory	Unsatisfactory	Unsatisfactory	Unsatisfactory	
P11645	2023	2023-Q4	2023-10	Post-pandemic recovery	Cytology (Pap)	25-29	29	Not performed	Satisfactory	NILM	NILM	Negative (NILM)	
P11646	2023	2023-Q4	2023-10	Post-pandemic recovery	Cytology (Pap)	50-54	52	Not performed	Satisfactory	Positive	ASC-US	Low-grade / equivocal squamous	
P11647	2023	2023-Q4	2023-10	Post-pandemic recovery	Cytology (Pap)	25-29	25	Not performed	Satisfactory	NILM	NILM	Negative (NILM)	
P11648	2023	2023-Q4	2023-10	Post-pandemic recovery	Cytology (Pap)	40-44	41	Not performed	Satisfactory	NILM	NILM	Negative (NILM)	
P11649	2023	2023-Q4	2023-10	Post-pandemic recovery	Cytology (Pap)	55-59	57	Not performed	Satisfactory	NILM	NILM	Negative (NILM)	
P11650	2023	2023-Q4	2023-10	Post-pandemic recovery	Cytology (Pap)	40-44	42	Not performed	Satisfactory	NILM	NILM	Negative (NILM)	
P11651	2023	2023-Q4	2023-10	Post-pandemic recovery	Cytology (Pap)	45-49	46	Not performed	Satisfactory	NILM	NILM	Negative (NILM)	
P11652	2023	2023-Q4	2023-10	Post-pandemic recovery	Cytology (Pap)	45-49	49	Not performed	Satisfactory	NILM	NILM	Negative (NILM)	
P11653	2023	2023-Q4	2023-10	Post-pandemic recovery	Cytology (Pap)	40-44	43	Not performed	Satisfactory	Positive	ASC-US	Low-grade / equivocal squamous	
P11654	2023	2023-Q4	2023-10	Post-pandemic recovery	Cytology (Pap)	25-29	29	Not performed	Satisfactory	NILM	NILM	Negative (NILM)	
P11655	2023	2023-Q4	2023-10	Post-pandemic recovery	Cytology (Pap)	40-44	41	Not performed	Satisfactory	NILM	NILM	Negative (NILM)	
P11656	2023	2023-Q4	2023-10	Post-pandemic recovery	Cytology (Pap)	40-44	44	Not performed	Satisfactory	NILM	NILM	Negative (NILM)	
P11657	2023	2023-Q4	2023-10	Post-pandemic recovery	Cytology (Pap)	30-34	31	Not performed	Satisfactory	Positive	ASC-US	Low-grade / equivocal squamous	
P11658	2023	2023-Q4	2023-10	Post-pandemic recovery	Cytology (Pap)	50-54	51	Not performed	Satisfactory	NILM	NILM	Negative (NILM)	Gardnerella
P11659	2023	2023-Q4	2023-10	Post-pandemic recovery	Cytology (Pap)	35-39	36	Not performed	Satisfactory	Positive	HSIL	High-grade squamous	
P11660	2023	2023-Q4	2023-10	Post-pandemic recovery	Cytology (Pap)	25-29	27	Not performed	Satisfactory	NILM	NILM	Negative (NILM)	
P11661	2023	2023-Q4	2023-10	Post-pandemic recovery	Cytology (Pap)	55-59	55	Not performed	Satisfactory	NILM	NILM	Negative (NILM)	
P11662	2023	2023-Q4	2023-10	Post-pandemic recovery	Cytology (Pap)	45-49	49	Not performed	Satisfactory	NILM	NILM	Negative (NILM)	
P11663	2023	2023-Q4	2023-10	Post-pandemic recovery	Cytology (Pap)	30-34	31	Not performed	Satisfactory	NILM	NILM	Negative (NILM)	
P11664	2023	2023-Q4	2023-10	Post-pandemic recovery	Cytology (Pap)	25-29	27	Not performed	Satisfactory	Positive	ASC-H	High-grade squamous	
P11665	2023	2023-Q4	2023-10	Post-pandemic recovery	Cytology (Pap)	30-34	31	Not performed	Satisfactory	NILM	NILM	Negative (NILM)	
P11666	2023	2023-Q4	2023-10	Post-pandemic recovery	Cytology (Pap)	30-34	31	Not performed	Satisfactory	NILM	NILM	Negative (NILM)	
P11667	2023	2023-Q4	2023-10	Post-pandemic recovery	Cytology (Pap)	40-44	44	Not performed	Satisfactory	NILM	NILM	Negative (NILM)	Gardnerella
P11668	2023	2023-Q4	2023-10	Post-pandemic recovery	Cytology (Pap)	30-34	33	Not performed	Satisfactory	NILM	NILM	Negative (NILM)	
P11669	2023	2023-Q4	2023-10	Post-pandemic recovery	Cytology (Pap)	40-44	44	Not performed	Satisfactory	NILM	NILM	Negative (NILM)	
P11670	2023	2023-Q4	2023-10	Post-pandemic recovery	Cytology (Pap)	30-34	30	Not performed	Satisfactory	NILM	NILM	Negative (NILM)	
P11671	2023	2023-Q4	2023-10	Post-pandemic recovery	Cytology (Pap)	35-39	39	Not performed	Satisfactory	NILM	NILM	Negative (NILM)	
P11672	2023	2023-Q4	2023-10	Post-pandemic recovery	Cytology (Pap)	40-44	40	Not performed	Satisfactory	NILM	NILM	Negative (NILM)	
P11673	2023	2023-Q4	2023-10	Post-pandemic recovery	Cytology (Pap)	50-54	52	Not performed	Unsatisfactory	Unsatisfactory	Unsatisfactory	Unsatisfactory	
P11674	2023	2023-Q4	2023-10	Post-pandemic recovery	Cytology (Pap)	50-54	53	Not performed	Satisfactory	NILM	NILM	Negative (NILM)	
P11675	2023	2023-Q4	2023-10	Post-pandemic recovery	Cytology (Pap)	60-64	60	Not performed	Satisfactory	NILM	NILM	Negative (NILM)	
P11676	2023	2023-Q4	2023-10	Post-pandemic recovery	Cytology (Pap)	50-54	50	Not performed	Satisfactory	NILM	NILM	Negative (NILM)	
P11677	2023	2023-Q4	2023-10	Post-pandemic recovery	Cytology (Pap)	45-49	45	Not performed	Satisfactory	NILM	NILM	Negative (NILM)	
P11678	2023	2023-Q4	2023-10	Post-pandemic recovery	Cytology (Pap)	35-39	37	Not performed	Satisfactory	NILM	NILM	Negative (NILM)	

patient_id	year	quarter	month	period	primary_test	age_group	age_years	hpv_result	adequacy	result	bethesda	grade	infection
P11679	2023	2023-Q4	2023-10	Post-pandemic recovery	Cytology (Pap)	60-64	60	Not performed	Satisfactory	Positive	ASC-US	Low-grade / equivocal squamous	
P11680	2023	2023-Q4	2023-10	Post-pandemic recovery	Cytology (Pap)	40-44	43	Not performed	Satisfactory	NILM	NILM	Negative (NILM)	
P11681	2023	2023-Q4	2023-10	Post-pandemic recovery	Cytology (Pap)	50-54	53	Not performed	Satisfactory	NILM	NILM	Negative (NILM)	
P11682	2023	2023-Q4	2023-11	Post-pandemic recovery	Cytology (Pap)	35-39	39	Not performed	Satisfactory	NILM	NILM	Negative (NILM)	
P11683	2023	2023-Q4	2023-11	Post-pandemic recovery	Cytology (Pap)	25-29	29	Not performed	Satisfactory	NILM	NILM	Negative (NILM)	
P11684	2023	2023-Q4	2023-11	Post-pandemic recovery	Cytology (Pap)	30-34	34	Not performed	Satisfactory	NILM	NILM	Negative (NILM)	
P11685	2023	2023-Q4	2023-11	Post-pandemic recovery	Cytology (Pap)	40-44	41	Not performed	Satisfactory	Positive	LSIL	Low-grade / equivocal squamous	
P11686	2023	2023-Q4	2023-11	Post-pandemic recovery	Cytology (Pap)	35-39	37	Not performed	Satisfactory	Positive	LSIL	Low-grade / equivocal squamous	
P11687	2023	2023-Q4	2023-11	Post-pandemic recovery	Cytology (Pap)	40-44	41	Not performed	Satisfactory	NILM	NILM	Negative (NILM)	
P11688	2023	2023-Q4	2023-11	Post-pandemic recovery	Cytology (Pap)	50-54	52	Not performed	Satisfactory	NILM	NILM	Negative (NILM)	
P11689	2023	2023-Q4	2023-11	Post-pandemic recovery	Cytology (Pap)	35-39	39	Not performed	Satisfactory	Positive	ASC-US	Low-grade / equivocal squamous	
P11690	2023	2023-Q4	2023-11	Post-pandemic recovery	Cytology (Pap)	55-59	59	Not performed	Satisfactory	NILM	NILM	Negative (NILM)	
P11691	2023	2023-Q4	2023-11	Post-pandemic recovery	Cytology (Pap)	40-44	44	Not performed	Satisfactory	NILM	NILM	Negative (NILM)	
P11692	2023	2023-Q4	2023-11	Post-pandemic recovery	Cytology (Pap)	35-39	37	Not performed	Satisfactory	Positive	ASC-US	Low-grade / equivocal squamous	
P11693	2023	2023-Q4	2023-11	Post-pandemic recovery	Cytology (Pap)	45-49	49	Not performed	Satisfactory	NILM	NILM	Negative (NILM)	
P11694	2023	2023-Q4	2023-11	Post-pandemic recovery	Cytology (Pap)	35-39	39	Not performed	Satisfactory	NILM	NILM	Negative (NILM)	
P11695	2023	2023-Q4	2023-11	Post-pandemic recovery	Cytology (Pap)	45-49	49	Not performed	Satisfactory	NILM	NILM	Negative (NILM)	
P11696	2023	2023-Q4	2023-11	Post-pandemic recovery	Cytology (Pap)	40-44	44	Not performed	Satisfactory	NILM	NILM	Negative (NILM)	Candida
P11697	2023	2023-Q4	2023-11	Post-pandemic recovery	Cytology (Pap)	35-39	38	Not performed	Satisfactory	Positive	HSIL	High-grade squamous	Candida
P11698	2023	2023-Q4	2023-11	Post-pandemic recovery	Cytology (Pap)	40-44	41	Not performed	Satisfactory	NILM	NILM	Negative (NILM)	
P11699	2023	2023-Q4	2023-11	Post-pandemic recovery	Cytology (Pap)	45-49	47	Not performed	Satisfactory	NILM	NILM	Negative (NILM)	
P11700	2023	2023-Q4	2023-11	Post-pandemic recovery	Cytology (Pap)	25-29	28	Not performed	Satisfactory	Positive	ASC-US	Low-grade / equivocal squamous	
P11701	2023	2023-Q4	2023-11	Post-pandemic recovery	Cytology (Pap)	35-39	38	Not performed	Satisfactory	NILM	NILM	Negative (NILM)	
P11702	2023	2023-Q4	2023-11	Post-pandemic recovery	Cytology (Pap)	35-39	39	Not performed	Satisfactory	NILM	NILM	Negative (NILM)	
P11703	2023	2023-Q4	2023-11	Post-pandemic recovery	Cytology (Pap)	25-29	29	Not performed	Satisfactory	NILM	NILM	Negative (NILM)	
P11704	2023	2023-Q4	2023-11	Post-pandemic recovery	Cytology (Pap)	45-49	47	Not performed	Satisfactory	NILM	NILM	Negative (NILM)	
P11705	2023	2023-Q4	2023-11	Post-pandemic recovery	Cytology (Pap)	35-39	39	Not performed	Unsatisfactory	Unsatisfactory	Unsatisfactory	Unsatisfactory	
P11706	2023	2023-Q4	2023-11	Post-pandemic recovery	Cytology (Pap)	30-34	31	Not performed	Satisfactory	NILM	NILM	Negative (NILM)	
P11707	2023	2023-Q4	2023-11	Post-pandemic recovery	Cytology (Pap)	60-64	64	Not performed	Satisfactory	Positive	AGC	Glandular	
P11708	2023	2023-Q4	2023-11	Post-pandemic recovery	Cytology (Pap)	60-64	64	Not performed	Unsatisfactory	Unsatisfactory	Unsatisfactory	Unsatisfactory	
P11709	2023	2023-Q4	2023-11	Post-pandemic recovery	Cytology (Pap)	30-34	33	Not performed	Satisfactory	NILM	NILM	Negative (NILM)	
P11710	2023	2023-Q4	2023-11	Post-pandemic recovery	Cytology (Pap)	25-29	25	Not performed	Satisfactory	Positive	LSIL	Low-grade / equivocal squamous	Candida
P11711	2023	2023-Q4	2023-11	Post-pandemic recovery	Cytology (Pap)	40-44	44	Not performed	Satisfactory	NILM	NILM	Negative (NILM)	
P11712	2023	2023-Q4	2023-11	Post-pandemic recovery	Cytology (Pap)	45-49	46	Not performed	Unsatisfactory	Unsatisfactory	Unsatisfactory	Unsatisfactory	
P11713	2023	2023-Q4	2023-11	Post-pandemic recovery	Cytology (Pap)	40-44	43	Not performed	Satisfactory	NILM	NILM	Negative (NILM)	
P11714	2023	2023-Q4	2023-11	Post-pandemic recovery	Cytology (Pap)	55-59	56	Not performed	Satisfactory	NILM	NILM	Negative (NILM)	
P11715	2023	2023-Q4	2023-11	Post-pandemic recovery	Cytology (Pap)	45-49	48	Not performed	Satisfactory	NILM	NILM	Negative (NILM)	
P11716	2023	2023-Q4	2023-11	Post-pandemic recovery	Cytology (Pap)	30-34	34	Not performed	Satisfactory	NILM	NILM	Negative (NILM)	
P11717	2023	2023-Q4	2023-11	Post-pandemic recovery	Cytology (Pap)	60-64	64	Not performed	Satisfactory	Positive	HSIL	High-grade squamous	Candida
P11718	2023	2023-Q4	2023-11	Post-pandemic recovery	Cytology (Pap)	55-59	58	Not performed	Satisfactory	NILM	NILM	Negative (NILM)	
P11719	2023	2023-Q4	2023-11	Post-pandemic recovery	Cytology (Pap)	40-44	41	Not performed	Unsatisfactory	Unsatisfactory	Unsatisfactory	Unsatisfactory	
P11720	2023	2023-Q4	2023-11	Post-pandemic recovery	Cytology (Pap)	40-44	41	Not performed	Satisfactory	NILM	NILM	Negative (NILM)	
P11721	2023	2023-Q4	2023-11	Post-pandemic recovery	Cytology (Pap)	25-29	27	Not performed	Satisfactory	NILM	NILM	Negative (NILM)	
P11722	2023	2023-Q4	2023-11	Post-pandemic recovery	Cytology (Pap)	35-39	39	Not performed	Satisfactory	NILM	NILM	Negative (NILM)	
P11723	2023	2023-Q4	2023-11	Post-pandemic recovery	Cytology (Pap)	45-49	46	Not performed	Satisfactory	NILM	NILM	Negative (NILM)	
P11724	2023	2023-Q4	2023-11	Post-pandemic recovery	Cytology (Pap)	50-54	52	Not performed	Satisfactory	NILM	NILM	Negative (NILM)	
P11725	2023	2023-Q4	2023-11	Post-pandemic recovery	Cytology (Pap)	30-34	30	Not performed	Satisfactory	NILM	NILM	Negative (NILM)	
P11726	2023	2023-Q4	2023-11	Post-pandemic recovery	Cytology (Pap)	50-54	51	Not performed	Satisfactory	NILM	NILM	Negative (NILM)	
P11727	2023	2023-Q4	2023-11	Post-pandemic recovery	Cytology (Pap)	50-54	50	Not performed	Satisfactory	NILM	NILM	Negative (NILM)	
P11728	2023	2023-Q4	2023-11	Post-pandemic recovery	Cytology (Pap)	25-29	26	Not performed	Satisfactory	NILM	NILM	Negative (NILM)	
P11729	2023	2023-Q4	2023-11	Post-pandemic recovery	Cytology (Pap)	25-29	26	Not performed	Satisfactory	NILM	NILM	Negative (NILM)	
P11730	2023	2023-Q4	2023-11	Post-pandemic recovery	Cytology (Pap)	50-54	54	Not performed	Satisfactory	NILM	NILM	Negative (NILM)	
P11731	2023	2023-Q4	2023-11	Post-pandemic recovery	Cytology (Pap)	55-59	55	Not performed	Satisfactory	NILM	NILM	Negative (NILM)	
P11732	2023	2023-Q4	2023-11	Post-pandemic recovery	Cytology (Pap)	40-44	42	Not performed	Satisfactory	NILM	NILM	Negative (NILM)	
P11733	2023	2023-Q4	2023-11	Post-pandemic recovery	Cytology (Pap)	40-44	44	Not performed	Satisfactory	NILM	NILM	Negative (NILM)	
P11734	2023	2023-Q4	2023-11	Post-pandemic recovery	Cytology (Pap)	30-34	32	Not performed	Satisfactory	NILM	NILM	Negative (NILM)	
P11735	2023	2023-Q4	2023-11	Post-pandemic recovery	Cytology (Pap)	50-54	52	Not performed	Satisfactory	NILM	NILM	Negative (NILM)	
P11736	2023	2023-Q4	2023-11	Post-pandemic recovery	Cytology (Pap)	60-64	64	Not performed	Satisfactory	NILM	NILM	Negative (NILM)	
P11737	2023	2023-Q4	2023-11	Post-pandemic recovery	Cytology (Pap)	40-44	41	Not performed	Satisfactory	NILM	NILM	Negative (NILM)	
P11738	2023	2023-Q4	2023-11	Post-pandemic recovery	Cytology (Pap)	35-39	38	Not performed	Satisfactory	NILM	NILM	Negative (NILM)	
P11739	2023	2023-Q4	2023-11	Post-pandemic recovery	Cytology (Pap)	35-39	38	Not performed	Satisfactory	NILM	NILM	Negative (NILM)	Candida
P11740	2023	2023-Q4	2023-11	Post-pandemic recovery	Cytology (Pap)	55-59	57	Not performed	Satisfactory	NILM	NILM	Negative (NILM)	Trichomonas
P11741	2023	2023-Q4	2023-11	Post-pandemic recovery	Cytology (Pap)	40-44	42	Not performed	Satisfactory	NILM	NILM	Negative (NILM)	
P11742	2023	2023-Q4	2023-11	Post-pandemic recovery	Cytology (Pap)	45-49	47	Not performed	Satisfactory	Positive	ASC-H	High-grade squamous	
P11743	2023	2023-Q4	2023-11	Post-pandemic recovery	Cytology (Pap)	25-29	27	Not performed	Satisfactory	NILM	NILM	Negative (NILM)	
P11744	2023	2023-Q4	2023-11	Post-pandemic recovery	Cytology (Pap)	30-34	34	Not performed	Satisfactory	NILM	NILM	Negative (NILM)	
P11745	2023	2023-Q4	2023-11	Post-pandemic recovery	Cytology (Pap)	30-34	32	Not performed	Unsatisfactory	Unsatisfactory	Unsatisfactory	Unsatisfactory	
P11746	2023	2023-Q4	2023-11	Post-pandemic recovery	Cytology (Pap)	35-39	35	Not performed	Satisfactory	Positive	ASC-US	Low-grade / equivocal squamous	
P11747	2023	2023-Q4	2023-11	Post-pandemic recovery	Cytology (Pap)	40-44	42	Not performed	Satisfactory	NILM	NILM	Negative (NILM)	
P11748	2023	2023-Q4	2023-11	Post-pandemic recovery	Cytology (Pap)	40-44	41	Not performed	Satisfactory	NILM	NILM	Negative (NILM)	Candida
P11749	2023	2023-Q4	2023-11	Post-pandemic recovery	Cytology (Pap)	35-39	37	Not performed	Satisfactory	NILM	NILM	Negative (NILM)	
P11750	2023	2023-Q4	2023-11	Post-pandemic recovery	Cytology (Pap)	55-59	55	Not performed	Satisfactory	NILM	NILM	Negative (NILM)	
P11751	2023	2023-Q4	2023-11	Post-pandemic recovery	Cytology (Pap)	30-34	32	Not performed	Satisfactory	NILM	NILM	Negative (NILM)	Trichomonas
P11752	2023	2023-Q4	2023-11	Post-pandemic recovery	Cytology (Pap)	45-49	49	Not performed	Satisfactory	NILM	NILM	Negative (NILM)	
P11753	2023	2023-Q4	2023-11	Post-pandemic recovery	Cytology (Pap)	40-44	40	Not performed	Satisfactory	NILM	NILM	Negative (NILM)	
P11754	2023	2023-Q4	2023-11	Post-pandemic recovery	Cytology (Pap)	45-49	49	Not performed	Satisfactory	NILM	NILM	Negative (NILM)	
P11755	2023	2023-Q4	2023-11	Post-pandemic recovery	Cytology (Pap)	40-44	44	Not performed	Satisfactory	Positive	ASC-US	Low-grade / equivocal squamous	
P11756	2023	2023-Q4	2023-11	Post-pandemic recovery	Cytology (Pap)	50-54	52	Not performed	Satisfactory	NILM	NILM	Negative (NILM)	
P11757	2023	2023-Q4	2023-11	Post-pandemic recovery	Cytology (Pap)	25-29	27	Not performed	Satisfactory	NILM	NILM	Negative (NILM)	
P11758	2023	2023-Q4	2023-11	Post-pandemic recovery	Cytology (Pap)	25-29	26	Not performed	Satisfactory	NILM	NILM	Negative (NILM)	

patient_id	year	quarter	month	period	primary_test	age_group	age_years	hpv_result	adequacy	result	bethesda	grade	infection
P11759	2023	2023-Q4	2023-11	Post-pandemic recovery	Cytology (Pap)	30-34	32	Not performed	Satisfactory	NILM	NILM	Negative (NILM)	
P11760	2023	2023-Q4	2023-11	Post-pandemic recovery	Cytology (Pap)	25-29	26	Not performed	Satisfactory	NILM	NILM	Negative (NILM)	
P11761	2023	2023-Q4	2023-11	Post-pandemic recovery	Cytology (Pap)	35-39	37	Not performed	Satisfactory	NILM	NILM	Negative (NILM)	
P11762	2023	2023-Q4	2023-11	Post-pandemic recovery	Cytology (Pap)	40-44	44	Not performed	Satisfactory	NILM	NILM	Negative (NILM)	
P11763	2023	2023-Q4	2023-11	Post-pandemic recovery	Cytology (Pap)	45-49	47	Not performed	Satisfactory	NILM	NILM	Negative (NILM)	
P11764	2023	2023-Q4	2023-11	Post-pandemic recovery	Cytology (Pap)	30-34	32	Not performed	Satisfactory	NILM	NILM	Negative (NILM)	
P11765	2023	2023-Q4	2023-11	Post-pandemic recovery	Cytology (Pap)	40-44	43	Not performed	Satisfactory	NILM	NILM	Negative (NILM)	
P11766	2023	2023-Q4	2023-11	Post-pandemic recovery	Cytology (Pap)	40-44	40	Not performed	Satisfactory	NILM	NILM	Negative (NILM)	
P11767	2023	2023-Q4	2023-11	Post-pandemic recovery	Cytology (Pap)	45-49	45	Not performed	Satisfactory	NILM	NILM	Negative (NILM)	
P11768	2023	2023-Q4	2023-11	Post-pandemic recovery	Cytology (Pap)	55-59	59	Not performed	Satisfactory	NILM	NILM	Negative (NILM)	
P11769	2023	2023-Q4	2023-11	Post-pandemic recovery	Cytology (Pap)	45-49	46	Not performed	Satisfactory	NILM	NILM	Negative (NILM)	
P11770	2023	2023-Q4	2023-11	Post-pandemic recovery	Cytology (Pap)	50-54	54	Not performed	Satisfactory	NILM	NILM	Negative (NILM)	
P11771	2023	2023-Q4	2023-11	Post-pandemic recovery	Cytology (Pap)	45-49	49	Not performed	Satisfactory	NILM	NILM	Negative (NILM)	
P11772	2023	2023-Q4	2023-11	Post-pandemic recovery	Cytology (Pap)	35-39	38	Not performed	Satisfactory	NILM	NILM	Negative (NILM)	
P11773	2023	2023-Q4	2023-11	Post-pandemic recovery	Cytology (Pap)	40-44	42	Not performed	Satisfactory	NILM	NILM	Negative (NILM)	
P11774	2023	2023-Q4	2023-11	Post-pandemic recovery	Cytology (Pap)	55-59	55	Not performed	Satisfactory	NILM	NILM	Negative (NILM)	
P11775	2023	2023-Q4	2023-11	Post-pandemic recovery	Cytology (Pap)	30-34	34	Not performed	Satisfactory	NILM	NILM	Negative (NILM)	
P11776	2023	2023-Q4	2023-11	Post-pandemic recovery	Cytology (Pap)	25-29	27	Not performed	Satisfactory	Positive	AGC	Glandular	
P11777	2023	2023-Q4	2023-11	Post-pandemic recovery	Cytology (Pap)	40-44	43	Not performed	Satisfactory	NILM	NILM	Negative (NILM)	
P11778	2023	2023-Q4	2023-11	Post-pandemic recovery	Cytology (Pap)	30-34	33	Not performed	Satisfactory	NILM	NILM	Negative (NILM)	
P11779	2023	2023-Q4	2023-11	Post-pandemic recovery	Cytology (Pap)	50-54	52	Not performed	Satisfactory	NILM	NILM	Negative (NILM)	
P11780	2023	2023-Q4	2023-11	Post-pandemic recovery	Cytology (Pap)	60-64	63	Not performed	Satisfactory	NILM	NILM	Negative (NILM)	Trichomonas
P11781	2023	2023-Q4	2023-11	Post-pandemic recovery	Cytology (Pap)	30-34	32	Not performed	Satisfactory	NILM	NILM	Negative (NILM)	
P11782	2023	2023-Q4	2023-11	Post-pandemic recovery	Cytology (Pap)	35-39	38	Not performed	Satisfactory	NILM	NILM	Negative (NILM)	
P11783	2023	2023-Q4	2023-11	Post-pandemic recovery	Cytology (Pap)	35-39	35	Not performed	Satisfactory	Positive	ASC-US	Low-grade / equivocal squamous	
P11784	2023	2023-Q4	2023-11	Post-pandemic recovery	Cytology (Pap)	60-64	64	Not performed	Satisfactory	NILM	NILM	Negative (NILM)	
P11785	2023	2023-Q4	2023-11	Post-pandemic recovery	Cytology (Pap)	30-34	31	Not performed	Satisfactory	NILM	NILM	Negative (NILM)	
P11786	2023	2023-Q4	2023-11	Post-pandemic recovery	Cytology (Pap)	40-44	40	Not performed	Satisfactory	NILM	NILM	Negative (NILM)	
P11787	2023	2023-Q4	2023-11	Post-pandemic recovery	Cytology (Pap)	55-59	57	Not performed	Unsatisfactory	Unsatisfactory	Unsatisfactory	Unsatisfactory	
P11788	2023	2023-Q4	2023-11	Post-pandemic recovery	Cytology (Pap)	40-44	43	Not performed	Satisfactory	NILM	NILM	Negative (NILM)	
P11789	2023	2023-Q4	2023-11	Post-pandemic recovery	Cytology (Pap)	35-39	37	Not performed	Satisfactory	NILM	NILM	Negative (NILM)	
P11790	2023	2023-Q4	2023-11	Post-pandemic recovery	Cytology (Pap)	40-44	44	Not performed	Satisfactory	NILM	NILM	Negative (NILM)	
P11791	2023	2023-Q4	2023-11	Post-pandemic recovery	Cytology (Pap)	55-59	55	Not performed	Satisfactory	Positive	LSIL	Low-grade / equivocal squamous	
P11792	2023	2023-Q4	2023-11	Post-pandemic recovery	Cytology (Pap)	30-34	30	Not performed	Satisfactory	NILM	NILM	Negative (NILM)	
P11793	2023	2023-Q4	2023-11	Post-pandemic recovery	Cytology (Pap)	50-54	52	Not performed	Satisfactory	NILM	NILM	Negative (NILM)	
P11794	2023	2023-Q4	2023-11	Post-pandemic recovery	Cytology (Pap)	55-59	55	Not performed	Satisfactory	NILM	NILM	Negative (NILM)	
P11795	2023	2023-Q4	2023-11	Post-pandemic recovery	Cytology (Pap)	25-29	25	Not performed	Satisfactory	NILM	NILM	Negative (NILM)	Gardnerella
P11796	2023	2023-Q4	2023-11	Post-pandemic recovery	Cytology (Pap)	35-39	36	Not performed	Satisfactory	NILM	NILM	Negative (NILM)	
P11797	2023	2023-Q4	2023-11	Post-pandemic recovery	Cytology (Pap)	50-54	54	Not performed	Satisfactory	NILM	NILM	Negative (NILM)	
P11798	2023	2023-Q4	2023-11	Post-pandemic recovery	Cytology (Pap)	55-59	56	Not performed	Satisfactory	NILM	NILM	Negative (NILM)	
P11799	2023	2023-Q4	2023-11	Post-pandemic recovery	Cytology (Pap)	45-49	48	Not performed	Satisfactory	NILM	NILM	Negative (NILM)	
P11800	2023	2023-Q4	2023-11	Post-pandemic recovery	Cytology (Pap)	35-39	36	Not performed	Satisfactory	NILM	NILM	Negative (NILM)	
P11801	2023	2023-Q4	2023-11	Post-pandemic recovery	Cytology (Pap)	45-49	46	Not performed	Satisfactory	Positive	LSIL	Low-grade / equivocal squamous	
P11802	2023	2023-Q4	2023-11	Post-pandemic recovery	Cytology (Pap)	40-44	41	Not performed	Unsatisfactory	Unsatisfactory	Unsatisfactory	Unsatisfactory	
P11803	2023	2023-Q4	2023-11	Post-pandemic recovery	Cytology (Pap)	30-34	31	Not performed	Satisfactory	NILM	NILM	Negative (NILM)	
P11804	2023	2023-Q4	2023-11	Post-pandemic recovery	Cytology (Pap)	50-54	53	Not performed	Satisfactory	NILM	NILM	Negative (NILM)	
P11805	2023	2023-Q4	2023-11	Post-pandemic recovery	Cytology (Pap)	35-39	37	Not performed	Satisfactory	NILM	NILM	Negative (NILM)	
P11806	2023	2023-Q4	2023-11	Post-pandemic recovery	Cytology (Pap)	50-54	51	Not performed	Satisfactory	NILM	NILM	Negative (NILM)	
P11807	2023	2023-Q4	2023-11	Post-pandemic recovery	Cytology (Pap)	35-39	37	Not performed	Satisfactory	NILM	NILM	Negative (NILM)	
P11808	2023	2023-Q4	2023-11	Post-pandemic recovery	Cytology (Pap)	35-39	37	Not performed	Satisfactory	NILM	NILM	Negative (NILM)	
P11809	2023	2023-Q4	2023-11	Post-pandemic recovery	Cytology (Pap)	30-34	34	Not performed	Satisfactory	NILM	NILM	Negative (NILM)	
P11810	2023	2023-Q4	2023-11	Post-pandemic recovery	Cytology (Pap)	25-29	26	Not performed	Satisfactory	NILM	NILM	Negative (NILM)	
P11811	2023	2023-Q4	2023-11	Post-pandemic recovery	Cytology (Pap)	35-39	35	Not performed	Satisfactory	NILM	NILM	Negative (NILM)	
P11812	2023	2023-Q4	2023-11	Post-pandemic recovery	Cytology (Pap)	25-29	29	Not performed	Satisfactory	Positive	HSIL	High-grade squamous	
P11813	2023	2023-Q4	2023-11	Post-pandemic recovery	Cytology (Pap)	60-64	60	Not performed	Satisfactory	Positive	HSIL	High-grade squamous	
P11814	2023	2023-Q4	2023-11	Post-pandemic recovery	Cytology (Pap)	35-39	36	Not performed	Satisfactory	NILM	NILM	Negative (NILM)	
P11815	2023	2023-Q4	2023-11	Post-pandemic recovery	Cytology (Pap)	35-39	39	Not performed	Satisfactory	NILM	NILM	Negative (NILM)	
P11816	2023	2023-Q4	2023-11	Post-pandemic recovery	Cytology (Pap)	35-39	37	Not performed	Satisfactory	NILM	NILM	Negative (NILM)	
P11817	2023	2023-Q4	2023-11	Post-pandemic recovery	Cytology (Pap)	40-44	44	Not performed	Satisfactory	NILM	NILM	Negative (NILM)	
P11818	2023	2023-Q4	2023-11	Post-pandemic recovery	Cytology (Pap)	55-59	56	Not performed	Satisfactory	NILM	NILM	Negative (NILM)	
P11819	2023	2023-Q4	2023-11	Post-pandemic recovery	Cytology (Pap)	40-44	43	Not performed	Satisfactory	NILM	NILM	Negative (NILM)	
P11820	2023	2023-Q4	2023-11	Post-pandemic recovery	Cytology (Pap)	50-54	52	Not performed	Satisfactory	NILM	NILM	Negative (NILM)	
P11821	2023	2023-Q4	2023-11	Post-pandemic recovery	Cytology (Pap)	40-44	41	Not performed	Satisfactory	NILM	NILM	Negative (NILM)	
P11822	2023	2023-Q4	2023-11	Post-pandemic recovery	Cytology (Pap)	60-64	62	Not performed	Satisfactory	NILM	NILM	Negative (NILM)	
P11823	2023	2023-Q4	2023-11	Post-pandemic recovery	Cytology (Pap)	55-59	58	Not performed	Satisfactory	NILM	NILM	Negative (NILM)	
P11824	2023	2023-Q4	2023-11	Post-pandemic recovery	Cytology (Pap)	40-44	43	Not performed	Satisfactory	NILM	NILM	Negative (NILM)	
P11825	2023	2023-Q4	2023-11	Post-pandemic recovery	Cytology (Pap)	45-49	47	Not performed	Satisfactory	NILM	NILM	Negative (NILM)	
P11826	2023	2023-Q4	2023-11	Post-pandemic recovery	Cytology (Pap)	55-59	59	Not performed	Satisfactory	NILM	NILM	Negative (NILM)	
P11827	2023	2023-Q4	2023-11	Post-pandemic recovery	Cytology (Pap)	50-54	52	Not performed	Satisfactory	NILM	NILM	Negative (NILM)	
P11828	2023	2023-Q4	2023-11	Post-pandemic recovery	Cytology (Pap)	30-34	33	Not performed	Satisfactory	NILM	NILM	Negative (NILM)	
P11829	2023	2023-Q4	2023-11	Post-pandemic recovery	Cytology (Pap)	50-54	52	Not performed	Satisfactory	NILM	NILM	Negative (NILM)	
P11830	2023	2023-Q4	2023-11	Post-pandemic recovery	Cytology (Pap)	40-44	42	Not performed	Unsatisfactory	Unsatisfactory	Unsatisfactory	Unsatisfactory	
P11831	2023	2023-Q4	2023-11	Post-pandemic recovery	Cytology (Pap)	40-44	41	Not performed	Satisfactory	NILM	NILM	Negative (NILM)	
P11832	2023	2023-Q4	2023-11	Post-pandemic recovery	Cytology (Pap)	45-49	47	Not performed	Unsatisfactory	Unsatisfactory	Unsatisfactory	Unsatisfactory	
P11833	2023	2023-Q4	2023-11	Post-pandemic recovery	Cytology (Pap)	40-44	41	Not performed	Satisfactory	NILM	NILM	Negative (NILM)	Gardnerella
P11834	2023	2023-Q4	2023-11	Post-pandemic recovery	Cytology (Pap)	40-44	40	Not performed	Satisfactory	NILM	NILM	Negative (NILM)	
P11835	2023	2023-Q4	2023-11	Post-pandemic recovery	Cytology (Pap)	60-64	61	Not performed	Satisfactory	NILM	NILM	Negative (NILM)	
P11836	2023	2023-Q4	2023-11	Post-pandemic recovery	Cytology (Pap)	40-44	40	Not performed	Satisfactory	Positive	ASC-H	High-grade squamous	
P11837	2023	2023-Q4	2023-11	Post-pandemic recovery	Cytology (Pap)	45-49	48	Not performed	Satisfactory	NILM	NILM	Negative (NILM)	
P11838	2023	2023-Q4	2023-11	Post-pandemic recovery	Cytology (Pap)	55-59	57	Not performed	Satisfactory	NILM	NILM	Negative (NILM)	

patient_id	year	quarter	month	period	primary_test	age_group	age_years	hpv_result	adequacy	result	bethesda	grade	infection
P11839	2023	2023-Q4	2023-11	Post-pandemic recovery	Cytology (Pap)	30-34	31	Not performed	Satisfactory	NILM	NILM	Negative (NILM)	
P11840	2023	2023-Q4	2023-11	Post-pandemic recovery	Cytology (Pap)	55-59	56	Not performed	Satisfactory	NILM	NILM	Negative (NILM)	
P11841	2023	2023-Q4	2023-11	Post-pandemic recovery	Cytology (Pap)	25-29	25	Not performed	Satisfactory	NILM	NILM	Negative (NILM)	
P11842	2023	2023-Q4	2023-11	Post-pandemic recovery	Cytology (Pap)	60-64	60	Not performed	Satisfactory	NILM	NILM	Negative (NILM)	
P11843	2023	2023-Q4	2023-11	Post-pandemic recovery	Cytology (Pap)	55-59	55	Not performed	Satisfactory	NILM	NILM	Negative (NILM)	
P11844	2023	2023-Q4	2023-11	Post-pandemic recovery	Cytology (Pap)	35-39	36	Not performed	Satisfactory	NILM	NILM	Negative (NILM)	
P11845	2023	2023-Q4	2023-11	Post-pandemic recovery	Cytology (Pap)	45-49	49	Not performed	Satisfactory	NILM	NILM	Negative (NILM)	
P11846	2023	2023-Q4	2023-11	Post-pandemic recovery	Cytology (Pap)	25-29	25	Not performed	Satisfactory	NILM	NILM	Negative (NILM)	
P11847	2023	2023-Q4	2023-11	Post-pandemic recovery	Cytology (Pap)	40-44	40	Not performed	Satisfactory	NILM	NILM	Negative (NILM)	
P11848	2023	2023-Q4	2023-11	Post-pandemic recovery	Cytology (Pap)	30-34	31	Not performed	Satisfactory	NILM	NILM	Negative (NILM)	
P11849	2023	2023-Q4	2023-11	Post-pandemic recovery	Cytology (Pap)	45-49	47	Not performed	Satisfactory	NILM	NILM	Negative (NILM)	
P11850	2023	2023-Q4	2023-11	Post-pandemic recovery	Cytology (Pap)	35-39	37	Not performed	Satisfactory	Positive	LSIL	Low-grade / equivocal squamous	
P11851	2023	2023-Q4	2023-11	Post-pandemic recovery	Cytology (Pap)	40-44	41	Not performed	Satisfactory	NILM	NILM	Negative (NILM)	
P11852	2023	2023-Q4	2023-11	Post-pandemic recovery	Cytology (Pap)	25-29	26	Not performed	Satisfactory	Positive	LSIL	Low-grade / equivocal squamous	
P11853	2023	2023-Q4	2023-11	Post-pandemic recovery	Cytology (Pap)	55-59	57	Not performed	Satisfactory	NILM	NILM	Negative (NILM)	
P11854	2023	2023-Q4	2023-11	Post-pandemic recovery	Cytology (Pap)	45-49	49	Not performed	Satisfactory	NILM	NILM	Negative (NILM)	
P11855	2023	2023-Q4	2023-11	Post-pandemic recovery	Cytology (Pap)	45-49	47	Not performed	Satisfactory	NILM	NILM	Negative (NILM)	
P11856	2023	2023-Q4	2023-11	Post-pandemic recovery	Cytology (Pap)	30-34	31	Not performed	Satisfactory	NILM	NILM	Negative (NILM)	
P11857	2023	2023-Q4	2023-11	Post-pandemic recovery	Cytology (Pap)	45-49	46	Not performed	Satisfactory	NILM	NILM	Negative (NILM)	
P11858	2023	2023-Q4	2023-11	Post-pandemic recovery	Cytology (Pap)	25-29	27	Not performed	Satisfactory	NILM	NILM	Negative (NILM)	
P11859	2023	2023-Q4	2023-11	Post-pandemic recovery	Cytology (Pap)	35-39	35	Not performed	Satisfactory	Positive	ASC-US	Low-grade / equivocal squamous	
P11860	2023	2023-Q4	2023-11	Post-pandemic recovery	Cytology (Pap)	30-34	34	Not performed	Satisfactory	Positive	AGC	Glandular	
P11861	2023	2023-Q4	2023-11	Post-pandemic recovery	Cytology (Pap)	40-44	41	Not performed	Satisfactory	NILM	NILM	Negative (NILM)	
P11862	2023	2023-Q4	2023-11	Post-pandemic recovery	Cytology (Pap)	35-39	39	Not performed	Satisfactory	Positive	ASC-US	Low-grade / equivocal squamous	
P11863	2023	2023-Q4	2023-11	Post-pandemic recovery	Cytology (Pap)	25-29	29	Not performed	Satisfactory	NILM	NILM	Negative (NILM)	
P11864	2023	2023-Q4	2023-11	Post-pandemic recovery	Cytology (Pap)	30-34	32	Not performed	Satisfactory	NILM	NILM	Negative (NILM)	
P11865	2023	2023-Q4	2023-11	Post-pandemic recovery	Cytology (Pap)	25-29	27	Not performed	Satisfactory	NILM	NILM	Negative (NILM)	
P11866	2023	2023-Q4	2023-11	Post-pandemic recovery	Cytology (Pap)	35-39	35	Not performed	Satisfactory	NILM	NILM	Negative (NILM)	
P11867	2023	2023-Q4	2023-11	Post-pandemic recovery	Cytology (Pap)	45-49	46	Not performed	Satisfactory	NILM	NILM	Negative (NILM)	
P11868	2023	2023-Q4	2023-11	Post-pandemic recovery	Cytology (Pap)	60-64	61	Not performed	Satisfactory	NILM	NILM	Negative (NILM)	
P11869	2023	2023-Q4	2023-11	Post-pandemic recovery	Cytology (Pap)	35-39	37	Not performed	Satisfactory	NILM	NILM	Negative (NILM)	
P11870	2023	2023-Q4	2023-11	Post-pandemic recovery	Cytology (Pap)	40-44	42	Not performed	Satisfactory	NILM	NILM	Negative (NILM)	
P11871	2023	2023-Q4	2023-11	Post-pandemic recovery	Cytology (Pap)	40-44	41	Not performed	Satisfactory	NILM	NILM	Negative (NILM)	
P11872	2023	2023-Q4	2023-11	Post-pandemic recovery	Cytology (Pap)	30-34	33	Not performed	Satisfactory	Positive	LSIL	Low-grade / equivocal squamous	Trichomonas
P11873	2023	2023-Q4	2023-11	Post-pandemic recovery	Cytology (Pap)	40-44	41	Not performed	Satisfactory	NILM	NILM	Negative (NILM)	
P11874	2023	2023-Q4	2023-11	Post-pandemic recovery	Cytology (Pap)	30-34	30	Not performed	Unsatisfactory	Unsatisfactory	Unsatisfactory	Unsatisfactory	
P11875	2023	2023-Q4	2023-11	Post-pandemic recovery	Cytology (Pap)	30-34	33	Not performed	Satisfactory	NILM	NILM	Negative (NILM)	
P11876	2023	2023-Q4	2023-11	Post-pandemic recovery	Cytology (Pap)	60-64	63	Not performed	Satisfactory	NILM	NILM	Negative (NILM)	
P11877	2023	2023-Q4	2023-11	Post-pandemic recovery	Cytology (Pap)	25-29	26	Not performed	Satisfactory	NILM	NILM	Negative (NILM)	
P11878	2023	2023-Q4	2023-11	Post-pandemic recovery	Cytology (Pap)	55-59	57	Not performed	Satisfactory	NILM	NILM	Negative (NILM)	
P11879	2023	2023-Q4	2023-11	Post-pandemic recovery	Cytology (Pap)	55-59	55	Not performed	Satisfactory	Positive	AGC	Glandular	Candida
P11880	2023	2023-Q4	2023-11	Post-pandemic recovery	Cytology (Pap)	35-39	38	Not performed	Satisfactory	Positive	LSIL	Low-grade / equivocal squamous	
P11881	2023	2023-Q4	2023-11	Post-pandemic recovery	Cytology (Pap)	40-44	44	Not performed	Satisfactory	Positive	ASC-US	Low-grade / equivocal squamous	
P11882	2023	2023-Q4	2023-11	Post-pandemic recovery	Cytology (Pap)	40-44	42	Not performed	Satisfactory	NILM	NILM	Negative (NILM)	
P11883	2023	2023-Q4	2023-11	Post-pandemic recovery	Cytology (Pap)	45-49	49	Not performed	Satisfactory	NILM	NILM	Negative (NILM)	
P11884	2023	2023-Q4	2023-11	Post-pandemic recovery	Cytology (Pap)	35-39	39	Not performed	Satisfactory	NILM	NILM	Negative (NILM)	
P11885	2023	2023-Q4	2023-11	Post-pandemic recovery	Cytology (Pap)	35-39	37	Not performed	Satisfactory	NILM	NILM	Negative (NILM)	
P11886	2023	2023-Q4	2023-11	Post-pandemic recovery	Cytology (Pap)	45-49	46	Not performed	Satisfactory	NILM	NILM	Negative (NILM)	
P11887	2023	2023-Q4	2023-11	Post-pandemic recovery	Cytology (Pap)	25-29	26	Not performed	Satisfactory	NILM	NILM	Negative (NILM)	
P11888	2023	2023-Q4	2023-11	Post-pandemic recovery	Cytology (Pap)	50-54	50	Not performed	Satisfactory	Positive	ASC-US	Low-grade / equivocal squamous	
P11889	2023	2023-Q4	2023-11	Post-pandemic recovery	Cytology (Pap)	35-39	39	Not performed	Satisfactory	NILM	NILM	Negative (NILM)	Gardnerella
P11890	2023	2023-Q4	2023-11	Post-pandemic recovery	Cytology (Pap)	40-44	42	Not performed	Satisfactory	NILM	NILM	Negative (NILM)	
P11891	2023	2023-Q4	2023-11	Post-pandemic recovery	Cytology (Pap)	45-49	48	Not performed	Satisfactory	NILM	NILM	Negative (NILM)	Trichomonas
P11892	2023	2023-Q4	2023-11	Post-pandemic recovery	Cytology (Pap)	30-34	30	Not performed	Satisfactory	NILM	NILM	Negative (NILM)	
P11893	2023	2023-Q4	2023-11	Post-pandemic recovery	Cytology (Pap)	45-49	49	Not performed	Satisfactory	NILM	NILM	Negative (NILM)	
P11894	2023	2023-Q4	2023-11	Post-pandemic recovery	Cytology (Pap)	35-39	38	Not performed	Satisfactory	NILM	NILM	Negative (NILM)	
P11895	2023	2023-Q4	2023-12	Post-pandemic recovery	Cytology (Pap)	25-29	25	Not performed	Satisfactory	Positive	ASC-US	Low-grade / equivocal squamous	
P11896	2023	2023-Q4	2023-12	Post-pandemic recovery	Cytology (Pap)	45-49	47	Not performed	Satisfactory	NILM	NILM	Negative (NILM)	
P11897	2023	2023-Q4	2023-12	Post-pandemic recovery	Cytology (Pap)	25-29	29	Not performed	Satisfactory	NILM	NILM	Negative (NILM)	
P11898	2023	2023-Q4	2023-12	Post-pandemic recovery	Cytology (Pap)	50-54	51	Not performed	Satisfactory	NILM	NILM	Negative (NILM)	
P11899	2023	2023-Q4	2023-12	Post-pandemic recovery	Cytology (Pap)	40-44	40	Not performed	Satisfactory	Positive	ASC-US	Low-grade / equivocal squamous	
P11900	2023	2023-Q4	2023-12	Post-pandemic recovery	Cytology (Pap)	55-59	55	Not performed	Satisfactory	NILM	NILM	Negative (NILM)	
P11901	2023	2023-Q4	2023-12	Post-pandemic recovery	Cytology (Pap)	25-29	29	Not performed	Satisfactory	NILM	NILM	Negative (NILM)	
P11902	2023	2023-Q4	2023-12	Post-pandemic recovery	Cytology (Pap)	35-39	39	Not performed	Satisfactory	NILM	NILM	Negative (NILM)	
P11903	2023	2023-Q4	2023-12	Post-pandemic recovery	Cytology (Pap)	40-44	44	Not performed	Satisfactory	NILM	NILM	Negative (NILM)	Candida
P11904	2023	2023-Q4	2023-12	Post-pandemic recovery	Cytology (Pap)	40-44	43	Not performed	Satisfactory	Positive	LSIL	Low-grade / equivocal squamous	
P11905	2023	2023-Q4	2023-12	Post-pandemic recovery	Cytology (Pap)	50-54	53	Not performed	Satisfactory	NILM	NILM	Negative (NILM)	
P11906	2023	2023-Q4	2023-12	Post-pandemic recovery	Cytology (Pap)	25-29	28	Not performed	Satisfactory	NILM	NILM	Negative (NILM)	Candida
P11907	2023	2023-Q4	2023-12	Post-pandemic recovery	Cytology (Pap)	40-44	40	Not performed	Satisfactory	NILM	NILM	Negative (NILM)	
P11908	2023	2023-Q4	2023-12	Post-pandemic recovery	Cytology (Pap)	40-44	41	Not performed	Satisfactory	NILM	NILM	Negative (NILM)	
P11909	2023	2023-Q4	2023-12	Post-pandemic recovery	Cytology (Pap)	45-49	46	Not performed	Satisfactory	NILM	NILM	Negative (NILM)	
P11910	2023	2023-Q4	2023-12	Post-pandemic recovery	Cytology (Pap)	30-34	34	Not performed	Satisfactory	NILM	NILM	Negative (NILM)	
P11911	2023	2023-Q4	2023-12	Post-pandemic recovery	Cytology (Pap)	25-29	25	Not performed	Satisfactory	NILM	NILM	Negative (NILM)	
P11912	2023	2023-Q4	2023-12	Post-pandemic recovery	Cytology (Pap)	40-44	40	Not performed	Satisfactory	NILM	NILM	Negative (NILM)	
P11913	2023	2023-Q4	2023-12	Post-pandemic recovery	Cytology (Pap)	40-44	43	Not performed	Satisfactory	Positive	HSIL	High-grade squamous	
P11914	2023	2023-Q4	2023-12	Post-pandemic recovery	Cytology (Pap)	50-54	54	Not performed	Satisfactory	Positive	ASC-US	Low-grade / equivocal squamous	Candida
P11915	2023	2023-Q4	2023-12	Post-pandemic recovery	Cytology (Pap)	25-29	25	Not performed	Satisfactory	NILM	NILM	Negative (NILM)	
P11916	2023	2023-Q4	2023-12	Post-pandemic recovery	Cytology (Pap)	45-49	48	Not performed	Satisfactory	NILM	NILM	Negative (NILM)	
P11917	2023	2023-Q4	2023-12	Post-pandemic recovery	Cytology (Pap)	35-39	39	Not performed	Satisfactory	NILM	NILM	Negative (NILM)	
P11918	2023	2023-Q4	2023-12	Post-pandemic recovery	Cytology (Pap)	45-49	45	Not performed	Satisfactory	NILM	NILM	Negative (NILM)	

patient_id	year	quarter	month	period	primary_test	age_group	age_years	hpv_result	adequacy	result	bethesda	grade	infection
P11919	2023	2023-Q4	2023-12	Post-pandemic recovery	Cytology (Pap)	50-54	52	Not performed	Satisfactory	NILM	NILM	Negative (NILM)	
P11920	2023	2023-Q4	2023-12	Post-pandemic recovery	Cytology (Pap)	45-49	46	Not performed	Unsatisfactory	Unsatisfactory	Unsatisfactory	Unsatisfactory	
P11921	2023	2023-Q4	2023-12	Post-pandemic recovery	Cytology (Pap)	55-59	59	Not performed	Unsatisfactory	Unsatisfactory	Unsatisfactory	Unsatisfactory	
P11922	2023	2023-Q4	2023-12	Post-pandemic recovery	Cytology (Pap)	30-34	32	Not performed	Satisfactory	NILM	NILM	Negative (NILM)	
P11923	2023	2023-Q4	2023-12	Post-pandemic recovery	Cytology (Pap)	55-59	57	Not performed	Satisfactory	NILM	NILM	Negative (NILM)	
P11924	2023	2023-Q4	2023-12	Post-pandemic recovery	Cytology (Pap)	45-49	47	Not performed	Satisfactory	NILM	NILM	Negative (NILM)	
P11925	2023	2023-Q4	2023-12	Post-pandemic recovery	Cytology (Pap)	40-44	41	Not performed	Satisfactory	Positive	ASC-US	Low-grade / equivocal squamous	
P11926	2023	2023-Q4	2023-12	Post-pandemic recovery	Cytology (Pap)	60-64	61	Not performed	Satisfactory	NILM	NILM	Negative (NILM)	
P11927	2023	2023-Q4	2023-12	Post-pandemic recovery	Cytology (Pap)	35-39	37	Not performed	Satisfactory	NILM	NILM	Negative (NILM)	
P11928	2023	2023-Q4	2023-12	Post-pandemic recovery	Cytology (Pap)	45-49	48	Not performed	Satisfactory	NILM	NILM	Negative (NILM)	
P11929	2023	2023-Q4	2023-12	Post-pandemic recovery	Cytology (Pap)	60-64	63	Not performed	Satisfactory	NILM	NILM	Negative (NILM)	
P11930	2023	2023-Q4	2023-12	Post-pandemic recovery	Cytology (Pap)	45-49	49	Not performed	Satisfactory	Positive	LSIL	Low-grade / equivocal squamous	
P11931	2023	2023-Q4	2023-12	Post-pandemic recovery	Cytology (Pap)	40-44	44	Not performed	Satisfactory	Positive	HSIL	High-grade squamous	
P11932	2023	2023-Q4	2023-12	Post-pandemic recovery	Cytology (Pap)	40-44	42	Not performed	Satisfactory	NILM	NILM	Negative (NILM)	
P11933	2023	2023-Q4	2023-12	Post-pandemic recovery	Cytology (Pap)	35-39	36	Not performed	Satisfactory	NILM	NILM	Negative (NILM)	
P11934	2023	2023-Q4	2023-12	Post-pandemic recovery	Cytology (Pap)	30-34	32	Not performed	Satisfactory	NILM	NILM	Negative (NILM)	Candida
P11935	2023	2023-Q4	2023-12	Post-pandemic recovery	Cytology (Pap)	55-59	55	Not performed	Satisfactory	Positive	ASC-US	Low-grade / equivocal squamous	
P11936	2023	2023-Q4	2023-12	Post-pandemic recovery	Cytology (Pap)	25-29	28	Not performed	Satisfactory	NILM	NILM	Negative (NILM)	
P11937	2023	2023-Q4	2023-12	Post-pandemic recovery	Cytology (Pap)	60-64	60	Not performed	Satisfactory	NILM	NILM	Negative (NILM)	
P11938	2023	2023-Q4	2023-12	Post-pandemic recovery	Cytology (Pap)	45-49	46	Not performed	Satisfactory	NILM	NILM	Negative (NILM)	
P11939	2023	2023-Q4	2023-12	Post-pandemic recovery	Cytology (Pap)	50-54	54	Not performed	Satisfactory	NILM	NILM	Negative (NILM)	
P11940	2023	2023-Q4	2023-12	Post-pandemic recovery	Cytology (Pap)	45-49	45	Not performed	Satisfactory	NILM	NILM	Negative (NILM)	
P11941	2023	2023-Q4	2023-12	Post-pandemic recovery	Cytology (Pap)	50-54	52	Not performed	Satisfactory	Positive	HSIL	High-grade squamous	
P11942	2023	2023-Q4	2023-12	Post-pandemic recovery	Cytology (Pap)	25-29	25	Not performed	Satisfactory	Positive	LSIL	Low-grade / equivocal squamous	
P11943	2023	2023-Q4	2023-12	Post-pandemic recovery	Cytology (Pap)	45-49	46	Not performed	Satisfactory	NILM	NILM	Negative (NILM)	
P11944	2023	2023-Q4	2023-12	Post-pandemic recovery	Cytology (Pap)	40-44	40	Not performed	Satisfactory	NILM	NILM	Negative (NILM)	
P11945	2023	2023-Q4	2023-12	Post-pandemic recovery	Cytology (Pap)	55-59	55	Not performed	Satisfactory	Positive	HSIL	High-grade squamous	
P11946	2023	2023-Q4	2023-12	Post-pandemic recovery	Cytology (Pap)	55-59	59	Not performed	Satisfactory	NILM	NILM	Negative (NILM)	Candida
P11947	2023	2023-Q4	2023-12	Post-pandemic recovery	Cytology (Pap)	60-64	62	Not performed	Satisfactory	NILM	NILM	Negative (NILM)	
P11948	2023	2023-Q4	2023-12	Post-pandemic recovery	Cytology (Pap)	35-39	36	Not performed	Satisfactory	NILM	NILM	Negative (NILM)	Candida
P11949	2023	2023-Q4	2023-12	Post-pandemic recovery	Cytology (Pap)	60-64	62	Not performed	Satisfactory	Positive	ASC-US	Low-grade / equivocal squamous	
P11950	2023	2023-Q4	2023-12	Post-pandemic recovery	Cytology (Pap)	35-39	36	Not performed	Satisfactory	NILM	NILM	Negative (NILM)	
P11951	2023	2023-Q4	2023-12	Post-pandemic recovery	Cytology (Pap)	30-34	32	Not performed	Satisfactory	Positive	ASC-H	High-grade squamous	
P11952	2023	2023-Q4	2023-12	Post-pandemic recovery	Cytology (Pap)	35-39	37	Not performed	Satisfactory	Positive	HSIL	High-grade squamous	Candida
P11953	2023	2023-Q4	2023-12	Post-pandemic recovery	Cytology (Pap)	35-39	38	Not performed	Satisfactory	NILM	NILM	Negative (NILM)	
P11954	2023	2023-Q4	2023-12	Post-pandemic recovery	Cytology (Pap)	35-39	39	Not performed	Satisfactory	Positive	ASC-US	Low-grade / equivocal squamous	
P11955	2023	2023-Q4	2023-12	Post-pandemic recovery	Cytology (Pap)	40-44	44	Not performed	Satisfactory	NILM	NILM	Negative (NILM)	
P11956	2023	2023-Q4	2023-12	Post-pandemic recovery	Cytology (Pap)	30-34	32	Not performed	Satisfactory	NILM	NILM	Negative (NILM)	
P11957	2023	2023-Q4	2023-12	Post-pandemic recovery	Cytology (Pap)	40-44	41	Not performed	Satisfactory	NILM	NILM	Negative (NILM)	
P11958	2023	2023-Q4	2023-12	Post-pandemic recovery	Cytology (Pap)	25-29	28	Not performed	Satisfactory	NILM	NILM	Negative (NILM)	
P11959	2023	2023-Q4	2023-12	Post-pandemic recovery	Cytology (Pap)	40-44	42	Not performed	Satisfactory	NILM	NILM	Negative (NILM)	
P11960	2023	2023-Q4	2023-12	Post-pandemic recovery	Cytology (Pap)	45-49	49	Not performed	Satisfactory	NILM	NILM	Negative (NILM)	Gardnerella
P11961	2023	2023-Q4	2023-12	Post-pandemic recovery	Cytology (Pap)	40-44	43	Not performed	Satisfactory	Positive	LSIL	Low-grade / equivocal squamous	
P11962	2023	2023-Q4	2023-12	Post-pandemic recovery	Cytology (Pap)	45-49	48	Not performed	Satisfactory	NILM	NILM	Negative (NILM)	
P11963	2023	2023-Q4	2023-12	Post-pandemic recovery	Cytology (Pap)	40-44	40	Not performed	Satisfactory	NILM	NILM	Negative (NILM)	
P11964	2023	2023-Q4	2023-12	Post-pandemic recovery	Cytology (Pap)	60-64	60	Not performed	Satisfactory	NILM	NILM	Negative (NILM)	
P11965	2023	2023-Q4	2023-12	Post-pandemic recovery	Cytology (Pap)	25-29	26	Not performed	Unsatisfactory	Unsatisfactory	Unsatisfactory	Unsatisfactory	
P11966	2023	2023-Q4	2023-12	Post-pandemic recovery	Cytology (Pap)	45-49	46	Not performed	Satisfactory	Positive	LSIL	Low-grade / equivocal squamous	
P11967	2023	2023-Q4	2023-12	Post-pandemic recovery	Cytology (Pap)	50-54	51	Not performed	Satisfactory	NILM	NILM	Negative (NILM)	
P11968	2023	2023-Q4	2023-12	Post-pandemic recovery	Cytology (Pap)	35-39	38	Not performed	Satisfactory	Positive	ASC-US	Low-grade / equivocal squamous	Trichomonas
P11969	2023	2023-Q4	2023-12	Post-pandemic recovery	Cytology (Pap)	55-59	55	Not performed	Satisfactory	NILM	NILM	Negative (NILM)	
P11970	2023	2023-Q4	2023-12	Post-pandemic recovery	Cytology (Pap)	50-54	52	Not performed	Satisfactory	Positive	HSIL	High-grade squamous	
P11971	2023	2023-Q4	2023-12	Post-pandemic recovery	Cytology (Pap)	35-39	39	Not performed	Satisfactory	NILM	NILM	Negative (NILM)	
P11972	2023	2023-Q4	2023-12	Post-pandemic recovery	Cytology (Pap)	45-49	48	Not performed	Satisfactory	NILM	NILM	Negative (NILM)	
P11973	2023	2023-Q4	2023-12	Post-pandemic recovery	Cytology (Pap)	25-29	28	Not performed	Satisfactory	NILM	NILM	Negative (NILM)	
P11974	2023	2023-Q4	2023-12	Post-pandemic recovery	Cytology (Pap)	40-44	40	Not performed	Satisfactory	NILM	NILM	Negative (NILM)	
P11975	2023	2023-Q4	2023-12	Post-pandemic recovery	Cytology (Pap)	25-29	29	Not performed	Satisfactory	NILM	NILM	Negative (NILM)	
P11976	2023	2023-Q4	2023-12	Post-pandemic recovery	Cytology (Pap)	50-54	53	Not performed	Satisfactory	Positive	AGC	Glandular	
P11977	2023	2023-Q4	2023-12	Post-pandemic recovery	Cytology (Pap)	45-49	46	Not performed	Satisfactory	NILM	NILM	Negative (NILM)	
P11978	2023	2023-Q4	2023-12	Post-pandemic recovery	Cytology (Pap)	45-49	46	Not performed	Satisfactory	NILM	NILM	Negative (NILM)	
P11979	2023	2023-Q4	2023-12	Post-pandemic recovery	Cytology (Pap)	35-39	38	Not performed	Satisfactory	NILM	NILM	Negative (NILM)	Other
P11980	2023	2023-Q4	2023-12	Post-pandemic recovery	Cytology (Pap)	35-39	37	Not performed	Satisfactory	Positive	ASC-US	Low-grade / equivocal squamous	
P11981	2023	2023-Q4	2023-12	Post-pandemic recovery	Cytology (Pap)	50-54	51	Not performed	Satisfactory	NILM	NILM	Negative (NILM)	
P11982	2023	2023-Q4	2023-12	Post-pandemic recovery	Cytology (Pap)	55-59	59	Not performed	Satisfactory	NILM	NILM	Negative (NILM)	
P11983	2023	2023-Q4	2023-12	Post-pandemic recovery	Cytology (Pap)	35-39	36	Not performed	Unsatisfactory	Unsatisfactory	Unsatisfactory	Unsatisfactory	
P11984	2023	2023-Q4	2023-12	Post-pandemic recovery	Cytology (Pap)	35-39	36	Not performed	Unsatisfactory	Unsatisfactory	Unsatisfactory	Unsatisfactory	
P11985	2023	2023-Q4	2023-12	Post-pandemic recovery	Cytology (Pap)	35-39	37	Not performed	Satisfactory	NILM	NILM	Negative (NILM)	
P11986	2023	2023-Q4	2023-12	Post-pandemic recovery	Cytology (Pap)	35-39	35	Not performed	Unsatisfactory	Unsatisfactory	Unsatisfactory	Unsatisfactory	
P11987	2023	2023-Q4	2023-12	Post-pandemic recovery	Cytology (Pap)	35-39	38	Not performed	Satisfactory	NILM	NILM	Negative (NILM)	
P11988	2023	2023-Q4	2023-12	Post-pandemic recovery	Cytology (Pap)	30-34	30	Not performed	Unsatisfactory	Unsatisfactory	Unsatisfactory	Unsatisfactory	
P11989	2023	2023-Q4	2023-12	Post-pandemic recovery	Cytology (Pap)	55-59	58	Not performed	Satisfactory	NILM	NILM	Negative (NILM)	Candida
P11990	2023	2023-Q4	2023-12	Post-pandemic recovery	Cytology (Pap)	55-59	55	Not performed	Satisfactory	NILM	NILM	Negative (NILM)	
P11991	2023	2023-Q4	2023-12	Post-pandemic recovery	Cytology (Pap)	45-49	48	Not performed	Satisfactory	Positive	ASC-US	Low-grade / equivocal squamous	
P11992	2023	2023-Q4	2023-12	Post-pandemic recovery	Cytology (Pap)	40-44	43	Not performed	Satisfactory	NILM	NILM	Negative (NILM)	
P11993	2023	2023-Q4	2023-12	Post-pandemic recovery	Cytology (Pap)	35-39	35	Not performed	Satisfactory	NILM	NILM	Negative (NILM)	
P11994	2023	2023-Q4	2023-12	Post-pandemic recovery	Cytology (Pap)	40-44	43	Not performed	Satisfactory	NILM	NILM	Negative (NILM)	
P11995	2023	2023-Q4	2023-12	Post-pandemic recovery	Cytology (Pap)	30-34	30	Not performed	Satisfactory	NILM	NILM	Negative (NILM)	Trichomonas
P11996	2023	2023-Q4	2023-12	Post-pandemic recovery	Cytology (Pap)	50-54	51	Not performed	Satisfactory	NILM	NILM	Negative (NILM)	
P11997	2023	2023-Q4	2023-12	Post-pandemic recovery	Cytology (Pap)	30-34	30	Not performed	Satisfactory	NILM	NILM	Negative (NILM)	
P11998	2023	2023-Q4	2023-12	Post-pandemic recovery	Cytology (Pap)	30-34	31	Not performed	Satisfactory	NILM	NILM	Negative (NILM)	

patient_id	year	quarter	month	period	primary_test	age_group	age_years	hpv_result	adequacy	result	bethesda	grade	infection
P11999	2023	2023-Q4	2023-12	Post-pandemic recovery	Cytology (Pap)	30-34	34	Not performed	Satisfactory	NILM	NILM	Negative (NILM)	
P12000	2023	2023-Q4	2023-12	Post-pandemic recovery	Cytology (Pap)	45-49	45	Not performed	Satisfactory	NILM	NILM	Negative (NILM)	
P12001	2023	2023-Q4	2023-12	Post-pandemic recovery	Cytology (Pap)	40-44	44	Not performed	Satisfactory	NILM	NILM	Negative (NILM)	
P12002	2023	2023-Q4	2023-12	Post-pandemic recovery	Cytology (Pap)	55-59	55	Not performed	Satisfactory	NILM	NILM	Negative (NILM)	
P12003	2023	2023-Q4	2023-12	Post-pandemic recovery	Cytology (Pap)	35-39	39	Not performed	Unsatisfactory	Unsatisfactory	Unsatisfactory	Unsatisfactory	
P12004	2023	2023-Q4	2023-12	Post-pandemic recovery	Cytology (Pap)	45-49	47	Not performed	Satisfactory	NILM	NILM	Negative (NILM)	
P12005	2023	2023-Q4	2023-12	Post-pandemic recovery	Cytology (Pap)	30-34	34	Not performed	Unsatisfactory	Unsatisfactory	Unsatisfactory	Unsatisfactory	
P12006	2023	2023-Q4	2023-12	Post-pandemic recovery	Cytology (Pap)	40-44	43	Not performed	Satisfactory	NILM	NILM	Negative (NILM)	
P12007	2023	2023-Q4	2023-12	Post-pandemic recovery	Cytology (Pap)	60-64	61	Not performed	Satisfactory	NILM	NILM	Negative (NILM)	
P12008	2023	2023-Q4	2023-12	Post-pandemic recovery	Cytology (Pap)	45-49	45	Not performed	Satisfactory	NILM	NILM	Negative (NILM)	
P12009	2023	2023-Q4	2023-12	Post-pandemic recovery	Cytology (Pap)	45-49	49	Not performed	Satisfactory	NILM	NILM	Negative (NILM)	
P12010	2023	2023-Q4	2023-12	Post-pandemic recovery	Cytology (Pap)	35-39	38	Not performed	Satisfactory	NILM	NILM	Negative (NILM)	
P12011	2023	2023-Q4	2023-12	Post-pandemic recovery	Cytology (Pap)	25-29	26	Not performed	Satisfactory	NILM	NILM	Negative (NILM)	
P12012	2023	2023-Q4	2023-12	Post-pandemic recovery	Cytology (Pap)	25-29	25	Not performed	Unsatisfactory	Unsatisfactory	Unsatisfactory	Unsatisfactory	
P12013	2023	2023-Q4	2023-12	Post-pandemic recovery	Cytology (Pap)	40-44	40	Not performed	Satisfactory	NILM	NILM	Negative (NILM)	
P12014	2023	2023-Q4	2023-12	Post-pandemic recovery	Cytology (Pap)	30-34	32	Not performed	Satisfactory	NILM	NILM	Negative (NILM)	
P12015	2023	2023-Q4	2023-12	Post-pandemic recovery	Cytology (Pap)	45-49	45	Not performed	Satisfactory	NILM	NILM	Negative (NILM)	
P12016	2023	2023-Q4	2023-12	Post-pandemic recovery	Cytology (Pap)	35-39	37	Not performed	Satisfactory	NILM	NILM	Negative (NILM)	
P12017	2023	2023-Q4	2023-12	Post-pandemic recovery	Cytology (Pap)	25-29	26	Not performed	Satisfactory	NILM	NILM	Negative (NILM)	
P12018	2023	2023-Q4	2023-12	Post-pandemic recovery	Cytology (Pap)	55-59	59	Not performed	Satisfactory	NILM	NILM	Negative (NILM)	
P12019	2023	2023-Q4	2023-12	Post-pandemic recovery	Cytology (Pap)	50-54	54	Not performed	Satisfactory	NILM	NILM	Negative (NILM)	
P12020	2023	2023-Q4	2023-12	Post-pandemic recovery	Cytology (Pap)	40-44	42	Not performed	Satisfactory	Positive	ASC-US	Low-grade / equivocal squamous	
P12021	2023	2023-Q4	2023-12	Post-pandemic recovery	Cytology (Pap)	45-49	45	Not performed	Satisfactory	NILM	NILM	Negative (NILM)	
P12022	2023	2023-Q4	2023-12	Post-pandemic recovery	Cytology (Pap)	45-49	49	Not performed	Satisfactory	NILM	NILM	Negative (NILM)	
P12023	2023	2023-Q4	2023-12	Post-pandemic recovery	Cytology (Pap)	50-54	51	Not performed	Satisfactory	NILM	NILM	Negative (NILM)	
P12024	2023	2023-Q4	2023-12	Post-pandemic recovery	Cytology (Pap)	25-29	25	Not performed	Satisfactory	NILM	NILM	Negative (NILM)	
P12025	2023	2023-Q4	2023-12	Post-pandemic recovery	Cytology (Pap)	45-49	49	Not performed	Satisfactory	NILM	NILM	Negative (NILM)	
P12026	2023	2023-Q4	2023-12	Post-pandemic recovery	Cytology (Pap)	30-34	31	Not performed	Satisfactory	NILM	NILM	Negative (NILM)	
P12027	2023	2023-Q4	2023-12	Post-pandemic recovery	Cytology (Pap)	40-44	43	Not performed	Satisfactory	Positive	ASC-US	Low-grade / equivocal squamous	
P12028	2023	2023-Q4	2023-12	Post-pandemic recovery	Cytology (Pap)	40-44	44	Not performed	Satisfactory	NILM	NILM	Negative (NILM)	Trichomonas
P12029	2023	2023-Q4	2023-12	Post-pandemic recovery	Cytology (Pap)	30-34	30	Not performed	Satisfactory	NILM	NILM	Negative (NILM)	
P12030	2023	2023-Q4	2023-12	Post-pandemic recovery	Cytology (Pap)	40-44	41	Not performed	Satisfactory	NILM	NILM	Negative (NILM)	Gardnerella
P12031	2023	2023-Q4	2023-12	Post-pandemic recovery	Cytology (Pap)	35-39	35	Not performed	Satisfactory	NILM	NILM	Negative (NILM)	
P12032	2023	2023-Q4	2023-12	Post-pandemic recovery	Cytology (Pap)	40-44	41	Not performed	Unsatisfactory	Unsatisfactory	Unsatisfactory	Unsatisfactory	
P12033	2023	2023-Q4	2023-12	Post-pandemic recovery	Cytology (Pap)	30-34	30	Not performed	Satisfactory	NILM	NILM	Negative (NILM)	Gardnerella
P12034	2023	2023-Q4	2023-12	Post-pandemic recovery	Cytology (Pap)	25-29	26	Not performed	Satisfactory	NILM	NILM	Negative (NILM)	
P12035	2023	2023-Q4	2023-12	Post-pandemic recovery	Cytology (Pap)	50-54	54	Not performed	Satisfactory	Positive	LSIL	Low-grade / equivocal squamous	
P12036	2023	2023-Q4	2023-12	Post-pandemic recovery	Cytology (Pap)	30-34	31	Not performed	Satisfactory	NILM	NILM	Negative (NILM)	
P12037	2023	2023-Q4	2023-12	Post-pandemic recovery	Cytology (Pap)	35-39	36	Not performed	Satisfactory	NILM	NILM	Negative (NILM)	
P12038	2023	2023-Q4	2023-12	Post-pandemic recovery	Cytology (Pap)	30-34	33	Not performed	Satisfactory	NILM	NILM	Negative (NILM)	
P12039	2023	2023-Q4	2023-12	Post-pandemic recovery	Cytology (Pap)	55-59	58	Not performed	Satisfactory	NILM	NILM	Negative (NILM)	
P12040	2023	2023-Q4	2023-12	Post-pandemic recovery	Cytology (Pap)	40-44	41	Not performed	Satisfactory	NILM	NILM	Negative (NILM)	
P12041	2023	2023-Q4	2023-12	Post-pandemic recovery	Cytology (Pap)	55-59	59	Not performed	Satisfactory	NILM	NILM	Negative (NILM)	
P12042	2023	2023-Q4	2023-12	Post-pandemic recovery	Cytology (Pap)	30-34	32	Not performed	Satisfactory	Positive	LSIL	Low-grade / equivocal squamous	
P12043	2023	2023-Q4	2023-12	Post-pandemic recovery	Cytology (Pap)	35-39	36	Not performed	Satisfactory	NILM	NILM	Negative (NILM)	
P12044	2023	2023-Q4	2023-12	Post-pandemic recovery	Cytology (Pap)	30-34	33	Not performed	Satisfactory	NILM	NILM	Negative (NILM)	
P12045	2023	2023-Q4	2023-12	Post-pandemic recovery	Cytology (Pap)	40-44	43	Not performed	Satisfactory	NILM	NILM	Negative (NILM)	
P12046	2023	2023-Q4	2023-12	Post-pandemic recovery	Cytology (Pap)	25-29	28	Not performed	Satisfactory	NILM	NILM	Negative (NILM)	
P12047	2023	2023-Q4	2023-12	Post-pandemic recovery	Cytology (Pap)	35-39	36	Not performed	Satisfactory	NILM	NILM	Negative (NILM)	
P12048	2023	2023-Q4	2023-12	Post-pandemic recovery	Cytology (Pap)	25-29	26	Not performed	Satisfactory	Positive	LSIL	Low-grade / equivocal squamous	
P12049	2023	2023-Q4	2023-12	Post-pandemic recovery	Cytology (Pap)	40-44	41	Not performed	Satisfactory	NILM	NILM	Negative (NILM)	
P12050	2023	2023-Q4	2023-12	Post-pandemic recovery	Cytology (Pap)	40-44	41	Not performed	Satisfactory	NILM	NILM	Negative (NILM)	
P12051	2023	2023-Q4	2023-12	Post-pandemic recovery	Cytology (Pap)	30-34	30	Not performed	Satisfactory	NILM	NILM	Negative (NILM)	
P12052	2023	2023-Q4	2023-12	Post-pandemic recovery	Cytology (Pap)	35-39	35	Not performed	Satisfactory	NILM	NILM	Negative (NILM)	
P12053	2023	2023-Q4	2023-12	Post-pandemic recovery	Cytology (Pap)	55-59	55	Not performed	Satisfactory	NILM	NILM	Negative (NILM)	Other
P12054	2023	2023-Q4	2023-12	Post-pandemic recovery	Cytology (Pap)	45-49	49	Not performed	Satisfactory	NILM	NILM	Negative (NILM)	
P12055	2023	2023-Q4	2023-12	Post-pandemic recovery	Cytology (Pap)	40-44	40	Not performed	Satisfactory	NILM	NILM	Negative (NILM)	
P12056	2023	2023-Q4	2023-12	Post-pandemic recovery	Cytology (Pap)	25-29	28	Not performed	Satisfactory	NILM	NILM	Negative (NILM)	
P12057	2023	2023-Q4	2023-12	Post-pandemic recovery	Cytology (Pap)	25-29	26	Not performed	Satisfactory	NILM	NILM	Negative (NILM)	
P12058	2023	2023-Q4	2023-12	Post-pandemic recovery	Cytology (Pap)	40-44	40	Not performed	Satisfactory	NILM	NILM	Negative (NILM)	
P12059	2023	2023-Q4	2023-12	Post-pandemic recovery	Cytology (Pap)	40-44	44	Not performed	Satisfactory	NILM	NILM	Negative (NILM)	
P12060	2023	2023-Q4	2023-12	Post-pandemic recovery	Cytology (Pap)	55-59	58	Not performed	Unsatisfactory	Unsatisfactory	Unsatisfactory	Unsatisfactory	
P12061	2023	2023-Q4	2023-12	Post-pandemic recovery	Cytology (Pap)	30-34	32	Not performed	Satisfactory	NILM	NILM	Negative (NILM)	
P12062	2023	2023-Q4	2023-12	Post-pandemic recovery	Cytology (Pap)	30-34	33	Not performed	Satisfactory	NILM	NILM	Negative (NILM)	
P12063	2023	2023-Q4	2023-12	Post-pandemic recovery	Cytology (Pap)	40-44	42	Not performed	Satisfactory	NILM	NILM	Negative (NILM)	
P12064	2023	2023-Q4	2023-12	Post-pandemic recovery	Cytology (Pap)	55-59	55	Not performed	Satisfactory	NILM	NILM	Negative (NILM)	
P12065	2023	2023-Q4	2023-12	Post-pandemic recovery	Cytology (Pap)	45-49	49	Not performed	Satisfactory	NILM	NILM	Negative (NILM)	
P12066	2023	2023-Q4	2023-12	Post-pandemic recovery	Cytology (Pap)	55-59	55	Not performed	Satisfactory	NILM	NILM	Negative (NILM)	
P12067	2023	2023-Q4	2023-12	Post-pandemic recovery	Cytology (Pap)	40-44	42	Not performed	Satisfactory	NILM	NILM	Negative (NILM)	
P12068	2023	2023-Q4	2023-12	Post-pandemic recovery	Cytology (Pap)	50-54	54	Not performed	Satisfactory	NILM	NILM	Negative (NILM)	
P12069	2023	2023-Q4	2023-12	Post-pandemic recovery	Cytology (Pap)	45-49	47	Not performed	Satisfactory	NILM	NILM	Negative (NILM)	
P12070	2023	2023-Q4	2023-12	Post-pandemic recovery	Cytology (Pap)	45-49	49	Not performed	Unsatisfactory	Unsatisfactory	Unsatisfactory	Unsatisfactory	
P12071	2023	2023-Q4	2023-12	Post-pandemic recovery	Cytology (Pap)	55-59	55	Not performed	Satisfactory	NILM	NILM	Negative (NILM)	
P12072	2023	2023-Q4	2023-12	Post-pandemic recovery	Cytology (Pap)	45-49	49	Not performed	Satisfactory	Positive	ASC-H	High-grade squamous	
P12073	2023	2023-Q4	2023-12	Post-pandemic recovery	Cytology (Pap)	50-54	52	Not performed	Satisfactory	NILM	NILM	Negative (NILM)	Other
P12074	2023	2023-Q4	2023-12	Post-pandemic recovery	Cytology (Pap)	45-49	49	Not performed	Satisfactory	NILM	NILM	Negative (NILM)	
P12075	2023	2023-Q4	2023-12	Post-pandemic recovery	Cytology (Pap)	25-29	27	Not performed	Satisfactory	NILM	NILM	Negative (NILM)	
P12076	2023	2023-Q4	2023-12	Post-pandemic recovery	Cytology (Pap)	35-39	35	Not performed	Satisfactory	NILM	NILM	Negative (NILM)	
P12077	2023	2023-Q4	2023-12	Post-pandemic recovery	Cytology (Pap)	40-44	44	Not performed	Satisfactory	NILM	NILM	Negative (NILM)	
P12078	2023	2023-Q4	2023-12	Post-pandemic recovery	Cytology (Pap)	40-44	40	Not performed	Satisfactory	Positive	ASC-US	Low-grade / equivocal squamous	Candida

patient_id	year	quarter	month	period	primary_test	age_group	age_years	hpv_result	adequacy	result	bethesda	grade	infection
P12079	2023	2023-Q4	2023-12	Post-pandemic recovery	Cytology (Pap)	30-34	30	Not performed	Satisfactory	NILM	NILM	Negative (NILM)	
P12080	2023	2023-Q4	2023-12	Post-pandemic recovery	Cytology (Pap)	50-54	50	Not performed	Satisfactory	NILM	NILM	Negative (NILM)	
P12081	2023	2023-Q4	2023-12	Post-pandemic recovery	Cytology (Pap)	35-39	39	Not performed	Satisfactory	NILM	NILM	Negative (NILM)	
P12082	2023	2023-Q4	2023-12	Post-pandemic recovery	Cytology (Pap)	40-44	43	Not performed	Satisfactory	NILM	NILM	Negative (NILM)	
P12083	2023	2023-Q4	2023-12	Post-pandemic recovery	Cytology (Pap)	25-29	28	Not performed	Satisfactory	NILM	NILM	Negative (NILM)	
P12084	2023	2023-Q4	2023-12	Post-pandemic recovery	Cytology (Pap)	25-29	25	Not performed	Satisfactory	NILM	NILM	Negative (NILM)	
P12085	2023	2023-Q4	2023-12	Post-pandemic recovery	Cytology (Pap)	55-59	55	Not performed	Satisfactory	Positive	LSIL	Low-grade / equivocal squamous	
P12086	2023	2023-Q4	2023-12	Post-pandemic recovery	Cytology (Pap)	40-44	41	Not performed	Satisfactory	Positive	ASC-US	Low-grade / equivocal squamous	
P12087	2023	2023-Q4	2023-12	Post-pandemic recovery	Cytology (Pap)	45-49	47	Not performed	Satisfactory	NILM	NILM	Negative (NILM)	
P12088	2023	2023-Q4	2023-12	Post-pandemic recovery	Cytology (Pap)	60-64	62	Not performed	Satisfactory	NILM	NILM	Negative (NILM)	
P12089	2023	2023-Q4	2023-12	Post-pandemic recovery	Cytology (Pap)	55-59	57	Not performed	Satisfactory	Positive	HSIL	High-grade squamous	
P12090	2023	2023-Q4	2023-12	Post-pandemic recovery	Cytology (Pap)	40-44	44	Not performed	Satisfactory	Positive	HSIL	High-grade squamous	
P12091	2023	2023-Q4	2023-12	Post-pandemic recovery	Cytology (Pap)	30-34	30	Not performed	Satisfactory	NILM	NILM	Negative (NILM)	
P12092	2023	2023-Q4	2023-12	Post-pandemic recovery	Cytology (Pap)	35-39	35	Not performed	Satisfactory	NILM	NILM	Negative (NILM)	
P12093	2023	2023-Q4	2023-12	Post-pandemic recovery	Cytology (Pap)	55-59	56	Not performed	Satisfactory	NILM	NILM	Negative (NILM)	
P12094	2023	2023-Q4	2023-12	Post-pandemic recovery	Cytology (Pap)	40-44	40	Not performed	Satisfactory	NILM	NILM	Negative (NILM)	
P12095	2023	2023-Q4	2023-12	Post-pandemic recovery	Cytology (Pap)	40-44	44	Not performed	Satisfactory	NILM	NILM	Negative (NILM)	
P12096	2023	2023-Q4	2023-12	Post-pandemic recovery	Cytology (Pap)	35-39	35	Not performed	Satisfactory	NILM	NILM	Negative (NILM)	
P12097	2023	2023-Q4	2023-12	Post-pandemic recovery	Cytology (Pap)	50-54	54	Not performed	Satisfactory	Positive	ASC-US	Low-grade / equivocal squamous	
P12098	2023	2023-Q4	2023-12	Post-pandemic recovery	Cytology (Pap)	45-49	46	Not performed	Satisfactory	NILM	NILM	Negative (NILM)	Gardnerella
P12099	2023	2023-Q4	2023-12	Post-pandemic recovery	Cytology (Pap)	60-64	63	Not performed	Satisfactory	NILM	NILM	Negative (NILM)	
P12100	2023	2023-Q4	2023-12	Post-pandemic recovery	Cytology (Pap)	40-44	42	Not performed	Satisfactory	NILM	NILM	Negative (NILM)	
P12101	2023	2023-Q4	2023-12	Post-pandemic recovery	Cytology (Pap)	35-39	38	Not performed	Satisfactory	NILM	NILM	Negative (NILM)	
P12102	2023	2023-Q4	2023-12	Post-pandemic recovery	Cytology (Pap)	35-39	35	Not performed	Satisfactory	NILM	NILM	Negative (NILM)	
P12103	2023	2023-Q4	2023-12	Post-pandemic recovery	Cytology (Pap)	30-34	30	Not performed	Satisfactory	NILM	NILM	Negative (NILM)	
P12104	2023	2023-Q4	2023-12	Post-pandemic recovery	Cytology (Pap)	40-44	40	Not performed	Satisfactory	NILM	NILM	Negative (NILM)	
P12105	2023	2023-Q4	2023-12	Post-pandemic recovery	Cytology (Pap)	30-34	32	Not performed	Satisfactory	NILM	NILM	Negative (NILM)	Candida
P12106	2023	2023-Q4	2023-12	Post-pandemic recovery	Cytology (Pap)	40-44	42	Not performed	Satisfactory	NILM	NILM	Negative (NILM)	
P12107	2023	2023-Q4	2023-12	Post-pandemic recovery	Cytology (Pap)	30-34	31	Not performed	Satisfactory	NILM	NILM	Negative (NILM)	
P12108	2024	2024-Q1	2024-01	HPV-primary era	HR-HPV DNA	40-44	40	Positive	Satisfactory	NILM	NILM	HPV-positive / cytology normal	
P12109	2024	2024-Q1	2024-01	HPV-primary era	HR-HPV DNA	55-59	58	Positive	Satisfactory	NILM	NILM	HPV-positive / cytology normal	
P12110	2024	2024-Q1	2024-01	HPV-primary era	HR-HPV DNA	40-44	44	Negative	N/A (no reflex cytology)	No reflex cytology (HPV-negative)	Not performed	HPV-negative (routine recall)	
P12111	2024	2024-Q1	2024-01	HPV-primary era	HR-HPV DNA	45-49	47	Negative	N/A (no reflex cytology)	No reflex cytology (HPV-negative)	Not performed	HPV-negative (routine recall)	
P12112	2024	2024-Q1	2024-01	HPV-primary era	HR-HPV DNA	30-34	32	Negative	N/A (no reflex cytology)	No reflex cytology (HPV-negative)	Not performed	HPV-negative (routine recall)	
P12113	2024	2024-Q1	2024-01	HPV-primary era	HR-HPV DNA	35-39	38	Negative	N/A (no reflex cytology)	No reflex cytology (HPV-negative)	Not performed	HPV-negative (routine recall)	
P12114	2024	2024-Q1	2024-01	HPV-primary era	HR-HPV DNA	30-34	30	Negative	N/A (no reflex cytology)	No reflex cytology (HPV-negative)	Not performed	HPV-negative (routine recall)	Gardnerella
P12115	2024	2024-Q1	2024-01	HPV-primary era	HR-HPV DNA	30-34	32	Negative	N/A (no reflex cytology)	No reflex cytology (HPV-negative)	Not performed	HPV-negative (routine recall)	
P12116	2024	2024-Q1	2024-01	HPV-primary era	HR-HPV DNA	30-34	32	Negative	N/A (no reflex cytology)	No reflex cytology (HPV-negative)	Not performed	HPV-negative (routine recall)	
P12117	2024	2024-Q1	2024-01	HPV-primary era	HR-HPV DNA	25-29	25	Negative	N/A (no reflex cytology)	No reflex cytology (HPV-negative)	Not performed	HPV-negative (routine recall)	
P12118	2024	2024-Q1	2024-01	HPV-primary era	HR-HPV DNA	35-39	35	Negative	N/A (no reflex cytology)	No reflex cytology (HPV-negative)	Not performed	HPV-negative (routine recall)	
P12119	2024	2024-Q1	2024-01	HPV-primary era	HR-HPV DNA	25-29	26	Negative	N/A (no reflex cytology)	No reflex cytology (HPV-negative)	Not performed	HPV-negative (routine recall)	
P12120	2024	2024-Q1	2024-01	HPV-primary era	HR-HPV DNA	45-49	48	Positive	Satisfactory	NILM	NILM	HPV-positive / cytology normal	
P12121	2024	2024-Q1	2024-01	HPV-primary era	HR-HPV DNA	35-39	37	Negative	N/A (no reflex cytology)	No reflex cytology (HPV-negative)	Not performed	HPV-negative (routine recall)	
P12122	2024	2024-Q1	2024-01	HPV-primary era	HR-HPV DNA	35-39	37	Negative	N/A (no reflex cytology)	No reflex cytology (HPV-negative)	Not performed	HPV-negative (routine recall)	
P12123	2024	2024-Q1	2024-01	HPV-primary era	HR-HPV DNA	45-49	46	Negative	N/A (no reflex cytology)	No reflex cytology (HPV-negative)	Not performed	HPV-negative (routine recall)	
P12124	2024	2024-Q1	2024-01	HPV-primary era	HR-HPV DNA	45-49	48	Positive	Satisfactory	Positive	ASC-US	Low-grade / equivocal squamous	
P12125	2024	2024-Q1	2024-01	HPV-primary era	HR-HPV DNA	40-44	42	Negative	N/A (no reflex cytology)	No reflex cytology (HPV-negative)	Not performed	HPV-negative (routine recall)	
P12126	2024	2024-Q1	2024-01	HPV-primary era	HR-HPV DNA	25-29	27	Positive	Satisfactory	Positive	LSIL	Low-grade / equivocal squamous	
P12127	2024	2024-Q1	2024-01	HPV-primary era	HR-HPV DNA	50-54	50	Negative	N/A (no reflex cytology)	No reflex cytology (HPV-negative)	Not performed	HPV-negative (routine recall)	
P12128	2024	2024-Q1	2024-01	HPV-primary era	HR-HPV DNA	50-54	51	Positive	Satisfactory	Positive	ASC-US	Low-grade / equivocal squamous	
P12129	2024	2024-Q1	2024-01	HPV-primary era	HR-HPV DNA	30-34	32	Positive	Satisfactory	Positive	ASC-H	High-grade squamous	Candida
P12130	2024	2024-Q1	2024-01	HPV-primary era	HR-HPV DNA	35-39	38	Positive	Satisfactory	Positive	ASC-H	High-grade squamous	
P12131	2024	2024-Q1	2024-01	HPV-primary era	HR-HPV DNA	25-29	27	Negative	N/A (no reflex cytology)	No reflex cytology (HPV-negative)	Not performed	HPV-negative (routine recall)	
P12132	2024	2024-Q1	2024-01	HPV-primary era	HR-HPV DNA	35-39	36	Negative	N/A (no reflex cytology)	No reflex cytology (HPV-negative)	Not performed	HPV-negative (routine recall)	
P12133	2024	2024-Q1	2024-01	HPV-primary era	HR-HPV DNA	50-54	50	Negative	N/A (no reflex cytology)	No reflex cytology (HPV-negative)	Not performed	HPV-negative (routine recall)	
P12134	2024	2024-Q1	2024-01	HPV-primary era	HR-HPV DNA	30-34	30	Negative	N/A (no reflex cytology)	No reflex cytology (HPV-negative)	Not performed	HPV-negative (routine recall)	
P12135	2024	2024-Q1	2024-01	HPV-primary era	HR-HPV DNA	25-29	29	Negative	N/A (no reflex cytology)	No reflex cytology (HPV-negative)	Not performed	HPV-negative (routine recall)	
P12136	2024	2024-Q1	2024-01	HPV-primary era	HR-HPV DNA	40-44	44	Negative	N/A (no reflex cytology)	No reflex cytology (HPV-negative)	Not performed	HPV-negative (routine recall)	
P12137	2024	2024-Q1	2024-01	HPV-primary era	HR-HPV DNA	40-44	44	Positive	Satisfactory	Positive	ASC-US	Low-grade / equivocal squamous	
P12138	2024	2024-Q1	2024-01	HPV-primary era	HR-HPV DNA	55-59	58	Negative	N/A (no reflex cytology)	No reflex cytology (HPV-negative)	Not performed	HPV-negative (routine recall)	
P12139	2024	2024-Q1	2024-01	HPV-primary era	HR-HPV DNA	50-54	53	Positive	Satisfactory	Positive	ASC-US	Low-grade / equivocal squamous	
P12140	2024	2024-Q1	2024-01	HPV-primary era	HR-HPV DNA	25-29	28	Negative	N/A (no reflex cytology)	No reflex cytology (HPV-negative)	Not performed	HPV-negative (routine recall)	
P12141	2024	2024-Q1	2024-01	HPV-primary era	HR-HPV DNA	50-54	50	Negative	N/A (no reflex cytology)	No reflex cytology (HPV-negative)	Not performed	HPV-negative (routine recall)	
P12142	2024	2024-Q1	2024-01	HPV-primary era	HR-HPV DNA	50-54	52	Positive	Satisfactory	Positive	ASC-US	Low-grade / equivocal squamous	
P12143	2024	2024-Q1	2024-01	HPV-primary era	HR-HPV DNA	40-44	42	Negative	N/A (no reflex cytology)	No reflex cytology (HPV-negative)	Not performed	HPV-negative (routine recall)	
P12144	2024	2024-Q1	2024-01	HPV-primary era	HR-HPV DNA	30-34	31	Negative	N/A (no reflex cytology)	No reflex cytology (HPV-negative)	Not performed	HPV-negative (routine recall)	
P12145	2024	2024-Q1	2024-01	HPV-primary era	HR-HPV DNA	35-39	35	Negative	N/A (no reflex cytology)	No reflex cytology (HPV-negative)	Not performed	HPV-negative (routine recall)	
P12146	2024	2024-Q1	2024-01	HPV-primary era	HR-HPV DNA	50-54	53	Negative	N/A (no reflex cytology)	No reflex cytology (HPV-negative)	Not performed	HPV-negative (routine recall)	
P12147	2024	2024-Q1	2024-01	HPV-primary era	HR-HPV DNA	25-29	26	Negative	N/A (no reflex cytology)	No reflex cytology (HPV-negative)	Not performed	HPV-negative (routine recall)	
P12148	2024	2024-Q1	2024-01	HPV-primary era	HR-HPV DNA	25-29	26	Negative	N/A (no reflex cytology)	No reflex cytology (HPV-negative)	Not performed	HPV-negative (routine recall)	
P12149	2024	2024-Q1	2024-01	HPV-primary era	HR-HPV DNA	35-39	38	Negative	N/A (no reflex cytology)	No reflex cytology (HPV-negative)	Not performed	HPV-negative (routine recall)	
P12150	2024	2024-Q1	2024-01	HPV-primary era	HR-HPV DNA	45-49	47	Positive	Satisfactory	NILM	NILM	HPV-positive / cytology normal	
P12151	2024	2024-Q1	2024-01	HPV-primary era	HR-HPV DNA	40-44	40	Negative	N/A (no reflex cytology)	No reflex cytology (HPV-negative)	Not performed	HPV-negative (routine recall)	
P12152	2024	2024-Q1	2024-01	HPV-primary era	HR-HPV DNA	30-34	33	Positive	Satisfactory	NILM	NILM	HPV-positive / cytology normal	
P12153	2024	2024-Q1	2024-01	HPV-primary era	HR-HPV DNA	45-49	48	Negative	N/A (no reflex cytology)	No reflex cytology (HPV-negative)	Not performed	HPV-negative (routine recall)	
P12154	2024	2024-Q1	2024-01	HPV-primary era	HR-HPV DNA	40-44	40	Negative	N/A (no reflex cytology)	No reflex cytology (HPV-negative)	Not performed	HPV-negative (routine recall)	
P12155	2024	2024-Q1	2024-01	HPV-primary era	HR-HPV DNA	35-39	38	Negative	N/A (no reflex cytology)	No reflex cytology (HPV-negative)	Not performed	HPV-negative (routine recall)	
P12156	2024	2024-Q1	2024-01	HPV-primary era	HR-HPV DNA	40-44	44	Negative	N/A (no reflex cytology)	No reflex cytology (HPV-negative)	Not performed	HPV-negative (routine recall)	
P12157	2024	2024-Q1	2024-01	HPV-primary era	HR-HPV DNA	50-54	50	Positive	Satisfactory	NILM	NILM	HPV-positive / cytology normal	
P12158	2024	2024-Q1	2024-01	HPV-primary era	HR-HPV DNA	30-34	31	Negative	N/A (no reflex cytology)	No reflex cytology (HPV-negative)	Not performed	HPV-negative (routine recall)	

patient_id	year	quarter	month	period	primary_test	age_group	age_years	hpv_result	adequacy	result	bethesda	grade	infection
P12159	2024	2024-Q1	2024-01	HPV-primary era	HR-HPV DNA	50-54	52	Negative	N/A (no reflex cytology)	No reflex cytology (HPV-negative)	Not performed	HPV-negative (routine recall)	
P12160	2024	2024-Q1	2024-01	HPV-primary era	HR-HPV DNA	35-39	37	Negative	N/A (no reflex cytology)	No reflex cytology (HPV-negative)	Not performed	HPV-negative (routine recall)	
P12161	2024	2024-Q1	2024-01	HPV-primary era	HR-HPV DNA	40-44	42	Negative	N/A (no reflex cytology)	No reflex cytology (HPV-negative)	Not performed	HPV-negative (routine recall)	
P12162	2024	2024-Q1	2024-01	HPV-primary era	HR-HPV DNA	40-44	43	Positive	Satisfactory	Positive	ASC-US	Low-grade / equivocal squamous	
P12163	2024	2024-Q1	2024-01	HPV-primary era	HR-HPV DNA	35-39	37	Negative	N/A (no reflex cytology)	No reflex cytology (HPV-negative)	Not performed	HPV-negative (routine recall)	
P12164	2024	2024-Q1	2024-01	HPV-primary era	HR-HPV DNA	30-34	34	Negative	N/A (no reflex cytology)	No reflex cytology (HPV-negative)	Not performed	HPV-negative (routine recall)	
P12165	2024	2024-Q1	2024-01	HPV-primary era	HR-HPV DNA	50-54	50	Negative	N/A (no reflex cytology)	No reflex cytology (HPV-negative)	Not performed	HPV-negative (routine recall)	
P12166	2024	2024-Q1	2024-01	HPV-primary era	HR-HPV DNA	50-54	50	Negative	N/A (no reflex cytology)	No reflex cytology (HPV-negative)	Not performed	HPV-negative (routine recall)	
P12167	2024	2024-Q1	2024-01	HPV-primary era	HR-HPV DNA	45-49	45	Positive	Satisfactory	Positive	HSIL	High-grade squamous	
P12168	2024	2024-Q1	2024-01	HPV-primary era	HR-HPV DNA	50-54	54	Negative	N/A (no reflex cytology)	No reflex cytology (HPV-negative)	Not performed	HPV-negative (routine recall)	
P12169	2024	2024-Q1	2024-01	HPV-primary era	HR-HPV DNA	50-54	51	Positive	Satisfactory	NILM	NILM	HPV-positive / cytology normal	
P12170	2024	2024-Q1	2024-01	HPV-primary era	HR-HPV DNA	35-39	35	Negative	N/A (no reflex cytology)	No reflex cytology (HPV-negative)	Not performed	HPV-negative (routine recall)	
P12171	2024	2024-Q1	2024-01	HPV-primary era	HR-HPV DNA	25-29	27	Negative	N/A (no reflex cytology)	No reflex cytology (HPV-negative)	Not performed	HPV-negative (routine recall)	
P12172	2024	2024-Q1	2024-01	HPV-primary era	HR-HPV DNA	40-44	43	Negative	N/A (no reflex cytology)	No reflex cytology (HPV-negative)	Not performed	HPV-negative (routine recall)	
P12173	2024	2024-Q1	2024-01	HPV-primary era	HR-HPV DNA	30-34	30	Negative	N/A (no reflex cytology)	No reflex cytology (HPV-negative)	Not performed	HPV-negative (routine recall)	
P12174	2024	2024-Q1	2024-01	HPV-primary era	HR-HPV DNA	35-39	37	Negative	N/A (no reflex cytology)	No reflex cytology (HPV-negative)	Not performed	HPV-negative (routine recall)	
P12175	2024	2024-Q1	2024-01	HPV-primary era	HR-HPV DNA	40-44	44	Positive	Satisfactory	NILM	NILM	HPV-positive / cytology normal	
P12176	2024	2024-Q1	2024-01	HPV-primary era	HR-HPV DNA	55-59	58	Negative	N/A (no reflex cytology)	No reflex cytology (HPV-negative)	Not performed	HPV-negative (routine recall)	
P12177	2024	2024-Q1	2024-01	HPV-primary era	HR-HPV DNA	35-39	37	Positive	Satisfactory	NILM	NILM	HPV-positive / cytology normal	
P12178	2024	2024-Q1	2024-01	HPV-primary era	HR-HPV DNA	45-49	48	Positive	Satisfactory	Positive	HSIL	High-grade squamous	
P12179	2024	2024-Q1	2024-01	HPV-primary era	HR-HPV DNA	30-34	33	Negative	N/A (no reflex cytology)	No reflex cytology (HPV-negative)	Not performed	HPV-negative (routine recall)	
P12180	2024	2024-Q1	2024-01	HPV-primary era	HR-HPV DNA	60-64	63	Positive	Satisfactory	NILM	NILM	HPV-positive / cytology normal	
P12181	2024	2024-Q1	2024-01	HPV-primary era	HR-HPV DNA	35-39	39	Negative	N/A (no reflex cytology)	No reflex cytology (HPV-negative)	Not performed	HPV-negative (routine recall)	Candida
P12182	2024	2024-Q1	2024-01	HPV-primary era	HR-HPV DNA	60-64	63	Positive	Satisfactory	NILM	NILM	HPV-positive / cytology normal	
P12183	2024	2024-Q1	2024-01	HPV-primary era	HR-HPV DNA	40-44	44	Negative	N/A (no reflex cytology)	No reflex cytology (HPV-negative)	Not performed	HPV-negative (routine recall)	
P12184	2024	2024-Q1	2024-01	HPV-primary era	HR-HPV DNA	30-34	30	Negative	N/A (no reflex cytology)	No reflex cytology (HPV-negative)	Not performed	HPV-negative (routine recall)	
P12185	2024	2024-Q1	2024-01	HPV-primary era	HR-HPV DNA	40-44	40	Negative	N/A (no reflex cytology)	No reflex cytology (HPV-negative)	Not performed	HPV-negative (routine recall)	
P12186	2024	2024-Q1	2024-01	HPV-primary era	HR-HPV DNA	35-39	35	Negative	N/A (no reflex cytology)	No reflex cytology (HPV-negative)	Not performed	HPV-negative (routine recall)	
P12187	2024	2024-Q1	2024-01	HPV-primary era	HR-HPV DNA	45-49	48	Negative	N/A (no reflex cytology)	No reflex cytology (HPV-negative)	Not performed	HPV-negative (routine recall)	
P12188	2024	2024-Q1	2024-01	HPV-primary era	HR-HPV DNA	50-54	53	Positive	Satisfactory	NILM	NILM	HPV-positive / cytology normal	
P12189	2024	2024-Q1	2024-01	HPV-primary era	HR-HPV DNA	30-34	30	Negative	N/A (no reflex cytology)	No reflex cytology (HPV-negative)	Not performed	HPV-negative (routine recall)	
P12190	2024	2024-Q1	2024-01	HPV-primary era	HR-HPV DNA	45-49	45	Negative	N/A (no reflex cytology)	No reflex cytology (HPV-negative)	Not performed	HPV-negative (routine recall)	
P12191	2024	2024-Q1	2024-01	HPV-primary era	HR-HPV DNA	35-39	39	Negative	N/A (no reflex cytology)	No reflex cytology (HPV-negative)	Not performed	HPV-negative (routine recall)	
P12192	2024	2024-Q1	2024-01	HPV-primary era	HR-HPV DNA	25-29	29	Positive	Satisfactory	NILM	NILM	HPV-positive / cytology normal	
P12193	2024	2024-Q1	2024-01	HPV-primary era	HR-HPV DNA	45-49	49	Negative	N/A (no reflex cytology)	No reflex cytology (HPV-negative)	Not performed	HPV-negative (routine recall)	
P12194	2024	2024-Q1	2024-01	HPV-primary era	HR-HPV DNA	55-59	56	Negative	N/A (no reflex cytology)	No reflex cytology (HPV-negative)	Not performed	HPV-negative (routine recall)	
P12195	2024	2024-Q1	2024-01	HPV-primary era	HR-HPV DNA	25-29	29	Negative	N/A (no reflex cytology)	No reflex cytology (HPV-negative)	Not performed	HPV-negative (routine recall)	
P12196	2024	2024-Q1	2024-01	HPV-primary era	HR-HPV DNA	30-34	33	Negative	N/A (no reflex cytology)	No reflex cytology (HPV-negative)	Not performed	HPV-negative (routine recall)	
P12197	2024	2024-Q1	2024-01	HPV-primary era	HR-HPV DNA	50-54	50	Negative	N/A (no reflex cytology)	No reflex cytology (HPV-negative)	Not performed	HPV-negative (routine recall)	
P12198	2024	2024-Q1	2024-01	HPV-primary era	HR-HPV DNA	25-29	29	Negative	N/A (no reflex cytology)	No reflex cytology (HPV-negative)	Not performed	HPV-negative (routine recall)	
P12199	2024	2024-Q1	2024-01	HPV-primary era	HR-HPV DNA	30-34	31	Negative	N/A (no reflex cytology)	No reflex cytology (HPV-negative)	Not performed	HPV-negative (routine recall)	
P12200	2024	2024-Q1	2024-01	HPV-primary era	HR-HPV DNA	55-59	55	Negative	N/A (no reflex cytology)	No reflex cytology (HPV-negative)	Not performed	HPV-negative (routine recall)	
P12201	2024	2024-Q1	2024-01	HPV-primary era	HR-HPV DNA	40-44	41	Positive	Satisfactory	NILM	NILM	HPV-positive / cytology normal	
P12202	2024	2024-Q1	2024-01	HPV-primary era	HR-HPV DNA	60-64	60	Negative	N/A (no reflex cytology)	No reflex cytology (HPV-negative)	Not performed	HPV-negative (routine recall)	
P12203	2024	2024-Q1	2024-01	HPV-primary era	HR-HPV DNA	25-29	29	Negative	N/A (no reflex cytology)	No reflex cytology (HPV-negative)	Not performed	HPV-negative (routine recall)	
P12204	2024	2024-Q1	2024-01	HPV-primary era	HR-HPV DNA	30-34	34	Negative	N/A (no reflex cytology)	No reflex cytology (HPV-negative)	Not performed	HPV-negative (routine recall)	
P12205	2024	2024-Q1	2024-01	HPV-primary era	HR-HPV DNA	25-29	28	Negative	N/A (no reflex cytology)	No reflex cytology (HPV-negative)	Not performed	HPV-negative (routine recall)	
P12206	2024	2024-Q1	2024-01	HPV-primary era	HR-HPV DNA	60-64	60	Positive	Satisfactory	Positive	ASC-US	Low-grade / equivocal squamous	
P12207	2024	2024-Q1	2024-01	HPV-primary era	HR-HPV DNA	35-39	37	Negative	N/A (no reflex cytology)	No reflex cytology (HPV-negative)	Not performed	HPV-negative (routine recall)	
P12208	2024	2024-Q1	2024-01	HPV-primary era	HR-HPV DNA	45-49	46	Negative	N/A (no reflex cytology)	No reflex cytology (HPV-negative)	Not performed	HPV-negative (routine recall)	
P12209	2024	2024-Q1	2024-01	HPV-primary era	HR-HPV DNA	30-34	32	Positive	Satisfactory	NILM	NILM	HPV-positive / cytology normal	
P12210	2024	2024-Q1	2024-01	HPV-primary era	HR-HPV DNA	35-39	35	Positive	Satisfactory	NILM	NILM	HPV-positive / cytology normal	
P12211	2024	2024-Q1	2024-01	HPV-primary era	HR-HPV DNA	60-64	62	Negative	N/A (no reflex cytology)	No reflex cytology (HPV-negative)	Not performed	HPV-negative (routine recall)	
P12212	2024	2024-Q1	2024-01	HPV-primary era	HR-HPV DNA	45-49	45	Negative	N/A (no reflex cytology)	No reflex cytology (HPV-negative)	Not performed	HPV-negative (routine recall)	
P12213	2024	2024-Q1	2024-01	HPV-primary era	HR-HPV DNA	50-54	54	Negative	N/A (no reflex cytology)	No reflex cytology (HPV-negative)	Not performed	HPV-negative (routine recall)	
P12214	2024	2024-Q1	2024-01	HPV-primary era	HR-HPV DNA	45-49	46	Negative	N/A (no reflex cytology)	No reflex cytology (HPV-negative)	Not performed	HPV-negative (routine recall)	
P12215	2024	2024-Q1	2024-01	HPV-primary era	HR-HPV DNA	40-44	44	Negative	N/A (no reflex cytology)	No reflex cytology (HPV-negative)	Not performed	HPV-negative (routine recall)	
P12216	2024	2024-Q1	2024-01	HPV-primary era	HR-HPV DNA	40-44	40	Negative	N/A (no reflex cytology)	No reflex cytology (HPV-negative)	Not performed	HPV-negative (routine recall)	
P12217	2024	2024-Q1	2024-01	HPV-primary era	HR-HPV DNA	40-44	41	Negative	N/A (no reflex cytology)	No reflex cytology (HPV-negative)	Not performed	HPV-negative (routine recall)	
P12218	2024	2024-Q1	2024-01	HPV-primary era	HR-HPV DNA	35-39	39	Negative	N/A (no reflex cytology)	No reflex cytology (HPV-negative)	Not performed	HPV-negative (routine recall)	
P12219	2024	2024-Q1	2024-01	HPV-primary era	HR-HPV DNA	40-44	40	Negative	N/A (no reflex cytology)	No reflex cytology (HPV-negative)	Not performed	HPV-negative (routine recall)	
P12220	2024	2024-Q1	2024-01	HPV-primary era	HR-HPV DNA	45-49	47	Negative	N/A (no reflex cytology)	No reflex cytology (HPV-negative)	Not performed	HPV-negative (routine recall)	
P12221	2024	2024-Q1	2024-01	HPV-primary era	HR-HPV DNA	50-54	54	Negative	N/A (no reflex cytology)	No reflex cytology (HPV-negative)	Not performed	HPV-negative (routine recall)	
P12222	2024	2024-Q1	2024-01	HPV-primary era	HR-HPV DNA	55-59	59	Negative	N/A (no reflex cytology)	No reflex cytology (HPV-negative)	Not performed	HPV-negative (routine recall)	
P12223	2024	2024-Q1	2024-01	HPV-primary era	HR-HPV DNA	55-59	57	Negative	N/A (no reflex cytology)	No reflex cytology (HPV-negative)	Not performed	HPV-negative (routine recall)	
P12224	2024	2024-Q1	2024-01	HPV-primary era	HR-HPV DNA	45-49	49	Negative	N/A (no reflex cytology)	No reflex cytology (HPV-negative)	Not performed	HPV-negative (routine recall)	
P12225	2024	2024-Q1	2024-01	HPV-primary era	HR-HPV DNA	30-34	32	Negative	N/A (no reflex cytology)	No reflex cytology (HPV-negative)	Not performed	HPV-negative (routine recall)	
P12226	2024	2024-Q1	2024-01	HPV-primary era	HR-HPV DNA	60-64	64	Negative	N/A (no reflex cytology)	No reflex cytology (HPV-negative)	Not performed	HPV-negative (routine recall)	
P12227	2024	2024-Q1	2024-01	HPV-primary era	HR-HPV DNA	30-34	33	Positive	Satisfactory	NILM	NILM	HPV-positive / cytology normal	
P12228	2024	2024-Q1	2024-01	HPV-primary era	HR-HPV DNA	40-44	41	Negative	N/A (no reflex cytology)	No reflex cytology (HPV-negative)	Not performed	HPV-negative (routine recall)	
P12229	2024	2024-Q1	2024-01	HPV-primary era	HR-HPV DNA	45-49	45	Negative	N/A (no reflex cytology)	No reflex cytology (HPV-negative)	Not performed	HPV-negative (routine recall)	
P12230	2024	2024-Q1	2024-01	HPV-primary era	HR-HPV DNA	45-49	47	Negative	N/A (no reflex cytology)	No reflex cytology (HPV-negative)	Not performed	HPV-negative (routine recall)	
P12231	2024	2024-Q1	2024-01	HPV-primary era	HR-HPV DNA	45-49	46	Negative	N/A (no reflex cytology)	No reflex cytology (HPV-negative)	Not performed	HPV-negative (routine recall)	
P12232	2024	2024-Q1	2024-01	HPV-primary era	HR-HPV DNA	45-49	46	Positive	Satisfactory	NILM	NILM	HPV-positive / cytology normal	
P12233	2024	2024-Q1	2024-01	HPV-primary era	HR-HPV DNA	55-59	59	Negative	N/A (no reflex cytology)	No reflex cytology (HPV-negative)	Not performed	HPV-negative (routine recall)	
P12234	2024	2024-Q1	2024-01	HPV-primary era	HR-HPV DNA	50-54	54	Negative	N/A (no reflex cytology)	No reflex cytology (HPV-negative)	Not performed	HPV-negative (routine recall)	
P12235	2024	2024-Q1	2024-01	HPV-primary era	HR-HPV DNA	35-39	35	Negative	N/A (no reflex cytology)	No reflex cytology (HPV-negative)	Not performed	HPV-negative (routine recall)	
P12236	2024	2024-Q1	2024-01	HPV-primary era	HR-HPV DNA	50-54	51	Negative	N/A (no reflex cytology)	No reflex cytology (HPV-negative)	Not performed	HPV-negative (routine recall)	
P12237	2024	2024-Q1	2024-01	HPV-primary era	HR-HPV DNA	25-29	25	Positive	Satisfactory	NILM	NILM	HPV-positive / cytology normal	
P12238	2024	2024-Q1	2024-01	HPV-primary era	HR-HPV DNA	25-29	29	Negative	N/A (no reflex cytology)	No reflex cytology (HPV-negative)	Not performed	HPV-negative (routine recall)	

patient_id	year	quarter	month	period	primary_test	age_group	age_years	hpv_result	adequacy	result	bethesda	grade	infection
P12239	2024	2024-Q1	2024-01	HPV-primary era	HR-HPV DNA	35-39	38	Negative	N/A (no reflex cytology)	No reflex cytology (HPV-negative)	Not performed	HPV-negative (routine recall)	
P12240	2024	2024-Q1	2024-01	HPV-primary era	HR-HPV DNA	50-54	52	Negative	N/A (no reflex cytology)	No reflex cytology (HPV-negative)	Not performed	HPV-negative (routine recall)	
P12241	2024	2024-Q1	2024-01	HPV-primary era	HR-HPV DNA	45-49	48	Positive	Satisfactory	Positive	ASC-US	Low-grade / equivocal squamous	
P12242	2024	2024-Q1	2024-01	HPV-primary era	HR-HPV DNA	60-64	62	Negative	N/A (no reflex cytology)	No reflex cytology (HPV-negative)	Not performed	HPV-negative (routine recall)	
P12243	2024	2024-Q1	2024-01	HPV-primary era	HR-HPV DNA	60-64	63	Negative	N/A (no reflex cytology)	No reflex cytology (HPV-negative)	Not performed	HPV-negative (routine recall)	
P12244	2024	2024-Q1	2024-01	HPV-primary era	HR-HPV DNA	40-44	41	Positive	Satisfactory	Positive	HSIL	High-grade squamous	
P12245	2024	2024-Q1	2024-01	HPV-primary era	HR-HPV DNA	35-39	38	Negative	N/A (no reflex cytology)	No reflex cytology (HPV-negative)	Not performed	HPV-negative (routine recall)	
P12246	2024	2024-Q1	2024-01	HPV-primary era	HR-HPV DNA	40-44	40	Positive	Satisfactory	NILM	NILM	HPV-positive / cytology normal	
P12247	2024	2024-Q1	2024-01	HPV-primary era	HR-HPV DNA	25-29	27	Negative	N/A (no reflex cytology)	No reflex cytology (HPV-negative)	Not performed	HPV-negative (routine recall)	
P12248	2024	2024-Q1	2024-01	HPV-primary era	HR-HPV DNA	50-54	50	Positive	Satisfactory	Positive	LSIL	Low-grade / equivocal squamous	
P12249	2024	2024-Q1	2024-01	HPV-primary era	HR-HPV DNA	35-39	36	Positive	Satisfactory	NILM	NILM	HPV-positive / cytology normal	
P12250	2024	2024-Q1	2024-01	HPV-primary era	HR-HPV DNA	30-34	31	Negative	N/A (no reflex cytology)	No reflex cytology (HPV-negative)	Not performed	HPV-negative (routine recall)	
P12251	2024	2024-Q1	2024-01	HPV-primary era	HR-HPV DNA	50-54	50	Negative	N/A (no reflex cytology)	No reflex cytology (HPV-negative)	Not performed	HPV-negative (routine recall)	
P12252	2024	2024-Q1	2024-01	HPV-primary era	HR-HPV DNA	30-34	32	Negative	N/A (no reflex cytology)	No reflex cytology (HPV-negative)	Not performed	HPV-negative (routine recall)	
P12253	2024	2024-Q1	2024-01	HPV-primary era	HR-HPV DNA	45-49	49	Positive	Satisfactory	NILM	NILM	HPV-positive / cytology normal	
P12254	2024	2024-Q1	2024-01	HPV-primary era	HR-HPV DNA	40-44	43	Negative	N/A (no reflex cytology)	No reflex cytology (HPV-negative)	Not performed	HPV-negative (routine recall)	
P12255	2024	2024-Q1	2024-01	HPV-primary era	HR-HPV DNA	25-29	25	Negative	N/A (no reflex cytology)	No reflex cytology (HPV-negative)	Not performed	HPV-negative (routine recall)	
P12256	2024	2024-Q1	2024-01	HPV-primary era	HR-HPV DNA	40-44	44	Negative	N/A (no reflex cytology)	No reflex cytology (HPV-negative)	Not performed	HPV-negative (routine recall)	
P12257	2024	2024-Q1	2024-01	HPV-primary era	HR-HPV DNA	35-39	37	Negative	N/A (no reflex cytology)	No reflex cytology (HPV-negative)	Not performed	HPV-negative (routine recall)	
P12258	2024	2024-Q1	2024-01	HPV-primary era	HR-HPV DNA	45-49	46	Negative	N/A (no reflex cytology)	No reflex cytology (HPV-negative)	Not performed	HPV-negative (routine recall)	
P12259	2024	2024-Q1	2024-01	HPV-primary era	HR-HPV DNA	55-59	56	Positive	Satisfactory	NILM	NILM	HPV-positive / cytology normal	
P12260	2024	2024-Q1	2024-01	HPV-primary era	HR-HPV DNA	25-29	27	Positive	Satisfactory	Positive	HSIL	High-grade squamous	
P12261	2024	2024-Q1	2024-01	HPV-primary era	HR-HPV DNA	35-39	38	Negative	N/A (no reflex cytology)	No reflex cytology (HPV-negative)	Not performed	HPV-negative (routine recall)	
P12262	2024	2024-Q1	2024-01	HPV-primary era	HR-HPV DNA	40-44	40	Negative	N/A (no reflex cytology)	No reflex cytology (HPV-negative)	Not performed	HPV-negative (routine recall)	
P12263	2024	2024-Q1	2024-01	HPV-primary era	HR-HPV DNA	40-44	42	Positive	Satisfactory	NILM	NILM	HPV-positive / cytology normal	
P12264	2024	2024-Q1	2024-01	HPV-primary era	HR-HPV DNA	55-59	56	Negative	N/A (no reflex cytology)	No reflex cytology (HPV-negative)	Not performed	HPV-negative (routine recall)	
P12265	2024	2024-Q1	2024-01	HPV-primary era	HR-HPV DNA	35-39	37	Negative	N/A (no reflex cytology)	No reflex cytology (HPV-negative)	Not performed	HPV-negative (routine recall)	
P12266	2024	2024-Q1	2024-01	HPV-primary era	HR-HPV DNA	45-49	48	Negative	N/A (no reflex cytology)	No reflex cytology (HPV-negative)	Not performed	HPV-negative (routine recall)	
P12267	2024	2024-Q1	2024-01	HPV-primary era	HR-HPV DNA	40-44	42	Negative	N/A (no reflex cytology)	No reflex cytology (HPV-negative)	Not performed	HPV-negative (routine recall)	Trichomonas
P12268	2024	2024-Q1	2024-01	HPV-primary era	HR-HPV DNA	30-34	34	Positive	Satisfactory	Positive	HSIL	High-grade squamous	
P12269	2024	2024-Q1	2024-01	HPV-primary era	HR-HPV DNA	55-59	59	Negative	N/A (no reflex cytology)	No reflex cytology (HPV-negative)	Not performed	HPV-negative (routine recall)	
P12270	2024	2024-Q1	2024-01	HPV-primary era	HR-HPV DNA	55-59	58	Positive	Satisfactory	NILM	NILM	HPV-positive / cytology normal	
P12271	2024	2024-Q1	2024-01	HPV-primary era	HR-HPV DNA	50-54	53	Negative	N/A (no reflex cytology)	No reflex cytology (HPV-negative)	Not performed	HPV-negative (routine recall)	
P12272	2024	2024-Q1	2024-01	HPV-primary era	HR-HPV DNA	35-39	35	Negative	N/A (no reflex cytology)	No reflex cytology (HPV-negative)	Not performed	HPV-negative (routine recall)	
P12273	2024	2024-Q1	2024-01	HPV-primary era	HR-HPV DNA	25-29	28	Negative	N/A (no reflex cytology)	No reflex cytology (HPV-negative)	Not performed	HPV-negative (routine recall)	
P12274	2024	2024-Q1	2024-01	HPV-primary era	HR-HPV DNA	55-59	56	Negative	N/A (no reflex cytology)	No reflex cytology (HPV-negative)	Not performed	HPV-negative (routine recall)	
P12275	2024	2024-Q1	2024-01	HPV-primary era	HR-HPV DNA	25-29	28	Negative	N/A (no reflex cytology)	No reflex cytology (HPV-negative)	Not performed	HPV-negative (routine recall)	
P12276	2024	2024-Q1	2024-01	HPV-primary era	HR-HPV DNA	25-29	28	Negative	N/A (no reflex cytology)	No reflex cytology (HPV-negative)	Not performed	HPV-negative (routine recall)	
P12277	2024	2024-Q1	2024-01	HPV-primary era	HR-HPV DNA	30-34	33	Positive	Satisfactory	NILM	NILM	HPV-positive / cytology normal	
P12278	2024	2024-Q1	2024-01	HPV-primary era	HR-HPV DNA	45-49	48	Positive	Satisfactory	Positive	LSIL	Low-grade / equivocal squamous	
P12279	2024	2024-Q1	2024-01	HPV-primary era	HR-HPV DNA	40-44	43	Negative	N/A (no reflex cytology)	No reflex cytology (HPV-negative)	Not performed	HPV-negative (routine recall)	
P12280	2024	2024-Q1	2024-01	HPV-primary era	HR-HPV DNA	25-29	26	Negative	N/A (no reflex cytology)	No reflex cytology (HPV-negative)	Not performed	HPV-negative (routine recall)	
P12281	2024	2024-Q1	2024-01	HPV-primary era	HR-HPV DNA	40-44	42	Negative	N/A (no reflex cytology)	No reflex cytology (HPV-negative)	Not performed	HPV-negative (routine recall)	
P12282	2024	2024-Q1	2024-01	HPV-primary era	HR-HPV DNA	35-39	37	Negative	N/A (no reflex cytology)	No reflex cytology (HPV-negative)	Not performed	HPV-negative (routine recall)	
P12283	2024	2024-Q1	2024-01	HPV-primary era	HR-HPV DNA	40-44	44	Negative	N/A (no reflex cytology)	No reflex cytology (HPV-negative)	Not performed	HPV-negative (routine recall)	
P12284	2024	2024-Q1	2024-01	HPV-primary era	HR-HPV DNA	40-44	42	Negative	N/A (no reflex cytology)	No reflex cytology (HPV-negative)	Not performed	HPV-negative (routine recall)	
P12285	2024	2024-Q1	2024-01	HPV-primary era	HR-HPV DNA	45-49	48	Negative	N/A (no reflex cytology)	No reflex cytology (HPV-negative)	Not performed	HPV-negative (routine recall)	
P12286	2024	2024-Q1	2024-01	HPV-primary era	HR-HPV DNA	60-64	61	Negative	N/A (no reflex cytology)	No reflex cytology (HPV-negative)	Not performed	HPV-negative (routine recall)	
P12287	2024	2024-Q1	2024-01	HPV-primary era	HR-HPV DNA	50-54	54	Negative	N/A (no reflex cytology)	No reflex cytology (HPV-negative)	Not performed	HPV-negative (routine recall)	
P12288	2024	2024-Q1	2024-01	HPV-primary era	HR-HPV DNA	40-44	44	Negative	N/A (no reflex cytology)	No reflex cytology (HPV-negative)	Not performed	HPV-negative (routine recall)	
P12289	2024	2024-Q1	2024-01	HPV-primary era	HR-HPV DNA	35-39	37	Negative	N/A (no reflex cytology)	No reflex cytology (HPV-negative)	Not performed	HPV-negative (routine recall)	
P12290	2024	2024-Q1	2024-01	HPV-primary era	HR-HPV DNA	35-39	39	Negative	N/A (no reflex cytology)	No reflex cytology (HPV-negative)	Not performed	HPV-negative (routine recall)	
P12291	2024	2024-Q1	2024-01	HPV-primary era	HR-HPV DNA	30-34	34	Positive	Satisfactory	Positive	ASC-US	Low-grade / equivocal squamous	
P12292	2024	2024-Q1	2024-01	HPV-primary era	HR-HPV DNA	60-64	64	Positive	Satisfactory	Positive	LSIL	Low-grade / equivocal squamous	
P12293	2024	2024-Q1	2024-01	HPV-primary era	HR-HPV DNA	35-39	37	Negative	N/A (no reflex cytology)	No reflex cytology (HPV-negative)	Not performed	HPV-negative (routine recall)	
P12294	2024	2024-Q1	2024-01	HPV-primary era	HR-HPV DNA	45-49	48	Negative	N/A (no reflex cytology)	No reflex cytology (HPV-negative)	Not performed	HPV-negative (routine recall)	
P12295	2024	2024-Q1	2024-02	HPV-primary era	HR-HPV DNA	50-54	51	Negative	N/A (no reflex cytology)	No reflex cytology (HPV-negative)	Not performed	HPV-negative (routine recall)	
P12296	2024	2024-Q1	2024-02	HPV-primary era	HR-HPV DNA	40-44	44	Negative	N/A (no reflex cytology)	No reflex cytology (HPV-negative)	Not performed	HPV-negative (routine recall)	
P12297	2024	2024-Q1	2024-02	HPV-primary era	HR-HPV DNA	35-39	37	Negative	N/A (no reflex cytology)	No reflex cytology (HPV-negative)	Not performed	HPV-negative (routine recall)	
P12298	2024	2024-Q1	2024-02	HPV-primary era	HR-HPV DNA	25-29	25	Negative	N/A (no reflex cytology)	No reflex cytology (HPV-negative)	Not performed	HPV-negative (routine recall)	
P12299	2024	2024-Q1	2024-02	HPV-primary era	HR-HPV DNA	25-29	25	Positive	Satisfactory	Positive	AGC	Glandular	
P12300	2024	2024-Q1	2024-02	HPV-primary era	HR-HPV DNA	35-39	35	Negative	N/A (no reflex cytology)	No reflex cytology (HPV-negative)	Not performed	HPV-negative (routine recall)	
P12301	2024	2024-Q1	2024-02	HPV-primary era	HR-HPV DNA	55-59	57	Negative	N/A (no reflex cytology)	No reflex cytology (HPV-negative)	Not performed	HPV-negative (routine recall)	
P12302	2024	2024-Q1	2024-02	HPV-primary era	HR-HPV DNA	50-54	51	Negative	N/A (no reflex cytology)	No reflex cytology (HPV-negative)	Not performed	HPV-negative (routine recall)	
P12303	2024	2024-Q1	2024-02	HPV-primary era	HR-HPV DNA	25-29	25	Positive	Satisfactory	NILM	NILM	HPV-positive / cytology normal	
P12304	2024	2024-Q1	2024-02	HPV-primary era	HR-HPV DNA	45-49	49	Negative	N/A (no reflex cytology)	No reflex cytology (HPV-negative)	Not performed	HPV-negative (routine recall)	
P12305	2024	2024-Q1	2024-02	HPV-primary era	HR-HPV DNA	60-64	60	Negative	N/A (no reflex cytology)	No reflex cytology (HPV-negative)	Not performed	HPV-negative (routine recall)	
P12306	2024	2024-Q1	2024-02	HPV-primary era	HR-HPV DNA	40-44	42	Positive	Unsatisfactory	Unsatisfactory	Unsatisfactory	Unsatisfactory	
P12307	2024	2024-Q1	2024-02	HPV-primary era	HR-HPV DNA	55-59	56	Positive	Satisfactory	NILM	NILM	HPV-positive / cytology normal	
P12308	2024	2024-Q1	2024-02	HPV-primary era	HR-HPV DNA	30-34	30	Negative	N/A (no reflex cytology)	No reflex cytology (HPV-negative)	Not performed	HPV-negative (routine recall)	
P12309	2024	2024-Q1	2024-02	HPV-primary era	HR-HPV DNA	40-44	41	Negative	N/A (no reflex cytology)	No reflex cytology (HPV-negative)	Not performed	HPV-negative (routine recall)	
P12310	2024	2024-Q1	2024-02	HPV-primary era	HR-HPV DNA	50-54	52	Negative	N/A (no reflex cytology)	No reflex cytology (HPV-negative)	Not performed	HPV-negative (routine recall)	
P12311	2024	2024-Q1	2024-02	HPV-primary era	HR-HPV DNA	45-49	48	Negative	N/A (no reflex cytology)	No reflex cytology (HPV-negative)	Not performed	HPV-negative (routine recall)	Trichomonas
P12312	2024	2024-Q1	2024-02	HPV-primary era	HR-HPV DNA	45-49	46	Negative	N/A (no reflex cytology)	No reflex cytology (HPV-negative)	Not performed	HPV-negative (routine recall)	
P12313	2024	2024-Q1	2024-02	HPV-primary era	HR-HPV DNA	25-29	25	Positive	Satisfactory	Positive	ASC-US	Low-grade / equivocal squamous	
P12314	2024	2024-Q1	2024-02	HPV-primary era	HR-HPV DNA	30-34	34	Negative	N/A (no reflex cytology)	No reflex cytology (HPV-negative)	Not performed	HPV-negative (routine recall)	
P12315	2024	2024-Q1	2024-02	HPV-primary era	HR-HPV DNA	35-39	36	Negative	N/A (no reflex cytology)	No reflex cytology (HPV-negative)	Not performed	HPV-negative (routine recall)	
P12316	2024	2024-Q1	2024-02	HPV-primary era	HR-HPV DNA	50-54	50	Positive	Satisfactory	Positive	LSIL	Low-grade / equivocal squamous	
P12317	2024	2024-Q1	2024-02	HPV-primary era	HR-HPV DNA	35-39	37	Negative	N/A (no reflex cytology)	No reflex cytology (HPV-negative)	Not performed	HPV-negative (routine recall)	
P12318	2024	2024-Q1	2024-02	HPV-primary era	HR-HPV DNA	40-44	42	Positive	Satisfactory	NILM	NILM	HPV-positive / cytology normal	

patient_id	year	quarter	month	period	primary_test	age_group	age_years	hpv_result	adequacy	result	bethesda	grade	infection
P12319	2024	2024-Q1	2024-02	HPV-primary era	HR-HPV DNA	25-29	29	Positive	Satisfactory	Positive	HSIL	High-grade squamous	
P12320	2024	2024-Q1	2024-02	HPV-primary era	HR-HPV DNA	45-49	47	Negative	N/A (no reflex cytology)	No reflex cytology (HPV-negative)	Not performed	HPV-negative (routine recall)	
P12321	2024	2024-Q1	2024-02	HPV-primary era	HR-HPV DNA	25-29	26	Positive	Satisfactory	Positive	ASC-US	Low-grade / equivocal squamous	
P12322	2024	2024-Q1	2024-02	HPV-primary era	HR-HPV DNA	30-34	31	Negative	N/A (no reflex cytology)	No reflex cytology (HPV-negative)	Not performed	HPV-negative (routine recall)	
P12323	2024	2024-Q1	2024-02	HPV-primary era	HR-HPV DNA	35-39	37	Negative	N/A (no reflex cytology)	No reflex cytology (HPV-negative)	Not performed	HPV-negative (routine recall)	
P12324	2024	2024-Q1	2024-02	HPV-primary era	HR-HPV DNA	40-44	43	Negative	N/A (no reflex cytology)	No reflex cytology (HPV-negative)	Not performed	HPV-negative (routine recall)	
P12325	2024	2024-Q1	2024-02	HPV-primary era	HR-HPV DNA	35-39	36	Negative	N/A (no reflex cytology)	No reflex cytology (HPV-negative)	Not performed	HPV-negative (routine recall)	
P12326	2024	2024-Q1	2024-02	HPV-primary era	HR-HPV DNA	60-64	60	Negative	N/A (no reflex cytology)	No reflex cytology (HPV-negative)	Not performed	HPV-negative (routine recall)	
P12327	2024	2024-Q1	2024-02	HPV-primary era	HR-HPV DNA	30-34	33	Negative	N/A (no reflex cytology)	No reflex cytology (HPV-negative)	Not performed	HPV-negative (routine recall)	
P12328	2024	2024-Q1	2024-02	HPV-primary era	HR-HPV DNA	35-39	36	Negative	N/A (no reflex cytology)	No reflex cytology (HPV-negative)	Not performed	HPV-negative (routine recall)	
P12329	2024	2024-Q1	2024-02	HPV-primary era	HR-HPV DNA	45-49	49	Negative	N/A (no reflex cytology)	No reflex cytology (HPV-negative)	Not performed	HPV-negative (routine recall)	
P12330	2024	2024-Q1	2024-02	HPV-primary era	HR-HPV DNA	45-49	45	Negative	N/A (no reflex cytology)	No reflex cytology (HPV-negative)	Not performed	HPV-negative (routine recall)	
P12331	2024	2024-Q1	2024-02	HPV-primary era	HR-HPV DNA	50-54	52	Negative	N/A (no reflex cytology)	No reflex cytology (HPV-negative)	Not performed	HPV-negative (routine recall)	
P12332	2024	2024-Q1	2024-02	HPV-primary era	HR-HPV DNA	40-44	43	Positive	Satisfactory	Positive	LSIL	Low-grade / equivocal squamous	
P12333	2024	2024-Q1	2024-02	HPV-primary era	HR-HPV DNA	30-34	31	Negative	N/A (no reflex cytology)	No reflex cytology (HPV-negative)	Not performed	HPV-negative (routine recall)	
P12334	2024	2024-Q1	2024-02	HPV-primary era	HR-HPV DNA	30-34	32	Negative	N/A (no reflex cytology)	No reflex cytology (HPV-negative)	Not performed	HPV-negative (routine recall)	
P12335	2024	2024-Q1	2024-02	HPV-primary era	HR-HPV DNA	35-39	36	Negative	N/A (no reflex cytology)	No reflex cytology (HPV-negative)	Not performed	HPV-negative (routine recall)	
P12336	2024	2024-Q1	2024-02	HPV-primary era	HR-HPV DNA	40-44	44	Negative	N/A (no reflex cytology)	No reflex cytology (HPV-negative)	Not performed	HPV-negative (routine recall)	
P12337	2024	2024-Q1	2024-02	HPV-primary era	HR-HPV DNA	30-34	34	Positive	Satisfactory	Positive	HSIL	High-grade squamous	
P12338	2024	2024-Q1	2024-02	HPV-primary era	HR-HPV DNA	55-59	57	Negative	N/A (no reflex cytology)	No reflex cytology (HPV-negative)	Not performed	HPV-negative (routine recall)	
P12339	2024	2024-Q1	2024-02	HPV-primary era	HR-HPV DNA	25-29	28	Negative	N/A (no reflex cytology)	No reflex cytology (HPV-negative)	Not performed	HPV-negative (routine recall)	
P12340	2024	2024-Q1	2024-02	HPV-primary era	HR-HPV DNA	45-49	46	Negative	N/A (no reflex cytology)	No reflex cytology (HPV-negative)	Not performed	HPV-negative (routine recall)	
P12341	2024	2024-Q1	2024-02	HPV-primary era	HR-HPV DNA	25-29	25	Positive	Satisfactory	NILM	NILM	HPV-positive / cytology normal	
P12342	2024	2024-Q1	2024-02	HPV-primary era	HR-HPV DNA	35-39	35	Negative	N/A (no reflex cytology)	No reflex cytology (HPV-negative)	Not performed	HPV-negative (routine recall)	
P12343	2024	2024-Q1	2024-02	HPV-primary era	HR-HPV DNA	40-44	40	Negative	N/A (no reflex cytology)	No reflex cytology (HPV-negative)	Not performed	HPV-negative (routine recall)	
P12344	2024	2024-Q1	2024-02	HPV-primary era	HR-HPV DNA	35-39	37	Negative	N/A (no reflex cytology)	No reflex cytology (HPV-negative)	Not performed	HPV-negative (routine recall)	
P12345	2024	2024-Q1	2024-02	HPV-primary era	HR-HPV DNA	55-59	57	Negative	N/A (no reflex cytology)	No reflex cytology (HPV-negative)	Not performed	HPV-negative (routine recall)	
P12346	2024	2024-Q1	2024-02	HPV-primary era	HR-HPV DNA	50-54	52	Negative	N/A (no reflex cytology)	No reflex cytology (HPV-negative)	Not performed	HPV-negative (routine recall)	
P12347	2024	2024-Q1	2024-02	HPV-primary era	HR-HPV DNA	35-39	39	Positive	Satisfactory	NILM	NILM	HPV-positive / cytology normal	
P12348	2024	2024-Q1	2024-02	HPV-primary era	HR-HPV DNA	40-44	44	Negative	N/A (no reflex cytology)	No reflex cytology (HPV-negative)	Not performed	HPV-negative (routine recall)	
P12349	2024	2024-Q1	2024-02	HPV-primary era	HR-HPV DNA	55-59	55	Positive	Satisfactory	Positive	LSIL	Low-grade / equivocal squamous	
P12350	2024	2024-Q1	2024-02	HPV-primary era	HR-HPV DNA	35-39	35	Negative	N/A (no reflex cytology)	No reflex cytology (HPV-negative)	Not performed	HPV-negative (routine recall)	
P12351	2024	2024-Q1	2024-02	HPV-primary era	HR-HPV DNA	30-34	32	Positive	Satisfactory	NILM	NILM	HPV-positive / cytology normal	
P12352	2024	2024-Q1	2024-02	HPV-primary era	HR-HPV DNA	25-29	25	Positive	Satisfactory	Positive	HSIL	High-grade squamous	
P12353	2024	2024-Q1	2024-02	HPV-primary era	HR-HPV DNA	40-44	44	Negative	N/A (no reflex cytology)	No reflex cytology (HPV-negative)	Not performed	HPV-negative (routine recall)	
P12354	2024	2024-Q1	2024-02	HPV-primary era	HR-HPV DNA	55-59	55	Negative	N/A (no reflex cytology)	No reflex cytology (HPV-negative)	Not performed	HPV-negative (routine recall)	
P12355	2024	2024-Q1	2024-02	HPV-primary era	HR-HPV DNA	30-34	34	Negative	N/A (no reflex cytology)	No reflex cytology (HPV-negative)	Not performed	HPV-negative (routine recall)	
P12356	2024	2024-Q1	2024-02	HPV-primary era	HR-HPV DNA	50-54	54	Positive	Satisfactory	Positive	LSIL	Low-grade / equivocal squamous	
P12357	2024	2024-Q1	2024-02	HPV-primary era	HR-HPV DNA	40-44	44	Negative	N/A (no reflex cytology)	No reflex cytology (HPV-negative)	Not performed	HPV-negative (routine recall)	
P12358	2024	2024-Q1	2024-02	HPV-primary era	HR-HPV DNA	30-34	30	Positive	Satisfactory	NILM	NILM	HPV-positive / cytology normal	
P12359	2024	2024-Q1	2024-02	HPV-primary era	HR-HPV DNA	40-44	41	Positive	Satisfactory	Positive	ASC-US	Low-grade / equivocal squamous	
P12360	2024	2024-Q1	2024-02	HPV-primary era	HR-HPV DNA	30-34	31	Negative	N/A (no reflex cytology)	No reflex cytology (HPV-negative)	Not performed	HPV-negative (routine recall)	
P12361	2024	2024-Q1	2024-02	HPV-primary era	HR-HPV DNA	35-39	35	Negative	N/A (no reflex cytology)	No reflex cytology (HPV-negative)	Not performed	HPV-negative (routine recall)	
P12362	2024	2024-Q1	2024-02	HPV-primary era	HR-HPV DNA	30-34	30	Positive	Satisfactory	Positive	LSIL	Low-grade / equivocal squamous	
P12363	2024	2024-Q1	2024-02	HPV-primary era	HR-HPV DNA	45-49	48	Negative	N/A (no reflex cytology)	No reflex cytology (HPV-negative)	Not performed	HPV-negative (routine recall)	
P12364	2024	2024-Q1	2024-02	HPV-primary era	HR-HPV DNA	35-39	38	Positive	Satisfactory	NILM	NILM	HPV-positive / cytology normal	
P12365	2024	2024-Q1	2024-02	HPV-primary era	HR-HPV DNA	45-49	49	Negative	N/A (no reflex cytology)	No reflex cytology (HPV-negative)	Not performed	HPV-negative (routine recall)	
P12366	2024	2024-Q1	2024-02	HPV-primary era	HR-HPV DNA	55-59	57	Negative	N/A (no reflex cytology)	No reflex cytology (HPV-negative)	Not performed	HPV-negative (routine recall)	
P12367	2024	2024-Q1	2024-02	HPV-primary era	HR-HPV DNA	60-64	62	Negative	N/A (no reflex cytology)	No reflex cytology (HPV-negative)	Not performed	HPV-negative (routine recall)	
P12368	2024	2024-Q1	2024-02	HPV-primary era	HR-HPV DNA	30-34	32	Negative	N/A (no reflex cytology)	No reflex cytology (HPV-negative)	Not performed	HPV-negative (routine recall)	
P12369	2024	2024-Q1	2024-02	HPV-primary era	HR-HPV DNA	40-44	40	Negative	N/A (no reflex cytology)	No reflex cytology (HPV-negative)	Not performed	HPV-negative (routine recall)	
P12370	2024	2024-Q1	2024-02	HPV-primary era	HR-HPV DNA	55-59	55	Negative	N/A (no reflex cytology)	No reflex cytology (HPV-negative)	Not performed	HPV-negative (routine recall)	
P12371	2024	2024-Q1	2024-02	HPV-primary era	HR-HPV DNA	35-39	36	Negative	N/A (no reflex cytology)	No reflex cytology (HPV-negative)	Not performed	HPV-negative (routine recall)	
P12372	2024	2024-Q1	2024-02	HPV-primary era	HR-HPV DNA	35-39	36	Negative	N/A (no reflex cytology)	No reflex cytology (HPV-negative)	Not performed	HPV-negative (routine recall)	
P12373	2024	2024-Q1	2024-02	HPV-primary era	HR-HPV DNA	45-49	49	Positive	Satisfactory	NILM	NILM	HPV-positive / cytology normal	
P12374	2024	2024-Q1	2024-02	HPV-primary era	HR-HPV DNA	50-54	54	Negative	N/A (no reflex cytology)	No reflex cytology (HPV-negative)	Not performed	HPV-negative (routine recall)	
P12375	2024	2024-Q1	2024-02	HPV-primary era	HR-HPV DNA	35-39	37	Negative	N/A (no reflex cytology)	No reflex cytology (HPV-negative)	Not performed	HPV-negative (routine recall)	
P12376	2024	2024-Q1	2024-02	HPV-primary era	HR-HPV DNA	50-54	52	Negative	N/A (no reflex cytology)	No reflex cytology (HPV-negative)	Not performed	HPV-negative (routine recall)	
P12377	2024	2024-Q1	2024-02	HPV-primary era	HR-HPV DNA	30-34	33	Positive	Satisfactory	NILM	NILM	HPV-positive / cytology normal	
P12378	2024	2024-Q1	2024-02	HPV-primary era	HR-HPV DNA	55-59	57	Negative	N/A (no reflex cytology)	No reflex cytology (HPV-negative)	Not performed	HPV-negative (routine recall)	
P12379	2024	2024-Q1	2024-02	HPV-primary era	HR-HPV DNA	50-54	50	Negative	N/A (no reflex cytology)	No reflex cytology (HPV-negative)	Not performed	HPV-negative (routine recall)	
P12380	2024	2024-Q1	2024-02	HPV-primary era	HR-HPV DNA	45-49	46	Negative	N/A (no reflex cytology)	No reflex cytology (HPV-negative)	Not performed	HPV-negative (routine recall)	
P12381	2024	2024-Q1	2024-02	HPV-primary era	HR-HPV DNA	40-44	40	Positive	Satisfactory	Positive	AGC	Glandular	
P12382	2024	2024-Q1	2024-02	HPV-primary era	HR-HPV DNA	35-39	39	Negative	N/A (no reflex cytology)	No reflex cytology (HPV-negative)	Not performed	HPV-negative (routine recall)	
P12383	2024	2024-Q1	2024-02	HPV-primary era	HR-HPV DNA	60-64	62	Negative	N/A (no reflex cytology)	No reflex cytology (HPV-negative)	Not performed	HPV-negative (routine recall)	
P12384	2024	2024-Q1	2024-02	HPV-primary era	HR-HPV DNA	35-39	35	Negative	N/A (no reflex cytology)	No reflex cytology (HPV-negative)	Not performed	HPV-negative (routine recall)	
P12385	2024	2024-Q1	2024-02	HPV-primary era	HR-HPV DNA	45-49	45	Negative	N/A (no reflex cytology)	No reflex cytology (HPV-negative)	Not performed	HPV-negative (routine recall)	
P12386	2024	2024-Q1	2024-02	HPV-primary era	HR-HPV DNA	50-54	51	Negative	N/A (no reflex cytology)	No reflex cytology (HPV-negative)	Not performed	HPV-negative (routine recall)	
P12387	2024	2024-Q1	2024-02	HPV-primary era	HR-HPV DNA	60-64	63	Positive	Satisfactory	NILM	NILM	HPV-positive / cytology normal	
P12388	2024	2024-Q1	2024-02	HPV-primary era	HR-HPV DNA	40-44	40	Negative	N/A (no reflex cytology)	No reflex cytology (HPV-negative)	Not performed	HPV-negative (routine recall)	
P12389	2024	2024-Q1	2024-02	HPV-primary era	HR-HPV DNA	50-54	52	Negative	N/A (no reflex cytology)	No reflex cytology (HPV-negative)	Not performed	HPV-negative (routine recall)	
P12390	2024	2024-Q1	2024-02	HPV-primary era	HR-HPV DNA	40-44	42	Negative	N/A (no reflex cytology)	No reflex cytology (HPV-negative)	Not performed	HPV-negative (routine recall)	
P12391	2024	2024-Q1	2024-02	HPV-primary era	HR-HPV DNA	30-34	32	Negative	N/A (no reflex cytology)	No reflex cytology (HPV-negative)	Not performed	HPV-negative (routine recall)	
P12392	2024	2024-Q1	2024-02	HPV-primary era	HR-HPV DNA	45-49	47	Negative	N/A (no reflex cytology)	No reflex cytology (HPV-negative)	Not performed	HPV-negative (routine recall)	
P12393	2024	2024-Q1	2024-02	HPV-primary era	HR-HPV DNA	50-54	51	Positive	Satisfactory	Positive	HSIL	High-grade squamous	
P12394	2024	2024-Q1	2024-02	HPV-primary era	HR-HPV DNA	40-44	41	Positive	Satisfactory	NILM	NILM	HPV-positive / cytology normal	
P12395	2024	2024-Q1	2024-02	HPV-primary era	HR-HPV DNA	50-54	52	Negative	N/A (no reflex cytology)	No reflex cytology (HPV-negative)	Not performed	HPV-negative (routine recall)	
P12396	2024	2024-Q1	2024-02	HPV-primary era	HR-HPV DNA	40-44	43	Negative	N/A (no reflex cytology)	No reflex cytology (HPV-negative)	Not performed	HPV-negative (routine recall)	
P12397	2024	2024-Q1	2024-02	HPV-primary era	HR-HPV DNA	35-39	39	Negative	N/A (no reflex cytology)	No reflex cytology (HPV-negative)	Not performed	HPV-negative (routine recall)	
P12398	2024	2024-Q1	2024-02	HPV-primary era	HR-HPV DNA	30-34	30	Negative	N/A (no reflex cytology)	No reflex cytology (HPV-negative)	Not performed	HPV-negative (routine recall)	

patient_id	year	quarter	month	period	primary_test	age_group	age_years	hpv_result	adequacy	result	bethesda	grade	infection
P12399	2024	2024-Q1	2024-02	HPV-primary era	HR-HPV DNA	40-44	42	Negative	N/A (no reflex cytology)	No reflex cytology (HPV-negative)	Not performed	HPV-negative (routine recall)	
P12400	2024	2024-Q1	2024-02	HPV-primary era	HR-HPV DNA	55-59	55	Negative	N/A (no reflex cytology)	No reflex cytology (HPV-negative)	Not performed	HPV-negative (routine recall)	
P12401	2024	2024-Q1	2024-02	HPV-primary era	HR-HPV DNA	45-49	49	Negative	N/A (no reflex cytology)	No reflex cytology (HPV-negative)	Not performed	HPV-negative (routine recall)	
P12402	2024	2024-Q1	2024-02	HPV-primary era	HR-HPV DNA	35-39	37	Negative	N/A (no reflex cytology)	No reflex cytology (HPV-negative)	Not performed	HPV-negative (routine recall)	
P12403	2024	2024-Q1	2024-02	HPV-primary era	HR-HPV DNA	35-39	38	Positive	Satisfactory	NILM	NILM	HPV-positive / cytology normal	
P12404	2024	2024-Q1	2024-02	HPV-primary era	HR-HPV DNA	50-54	50	Negative	N/A (no reflex cytology)	No reflex cytology (HPV-negative)	Not performed	HPV-negative (routine recall)	
P12405	2024	2024-Q1	2024-02	HPV-primary era	HR-HPV DNA	25-29	29	Negative	N/A (no reflex cytology)	No reflex cytology (HPV-negative)	Not performed	HPV-negative (routine recall)	
P12406	2024	2024-Q1	2024-02	HPV-primary era	HR-HPV DNA	25-29	26	Negative	N/A (no reflex cytology)	No reflex cytology (HPV-negative)	Not performed	HPV-negative (routine recall)	
P12407	2024	2024-Q1	2024-02	HPV-primary era	HR-HPV DNA	25-29	28	Negative	N/A (no reflex cytology)	No reflex cytology (HPV-negative)	Not performed	HPV-negative (routine recall)	
P12408	2024	2024-Q1	2024-02	HPV-primary era	HR-HPV DNA	25-29	27	Positive	Satisfactory	NILM	NILM	HPV-positive / cytology normal	
P12409	2024	2024-Q1	2024-02	HPV-primary era	HR-HPV DNA	25-29	27	Negative	N/A (no reflex cytology)	No reflex cytology (HPV-negative)	Not performed	HPV-negative (routine recall)	
P12410	2024	2024-Q1	2024-02	HPV-primary era	HR-HPV DNA	35-39	38	Negative	N/A (no reflex cytology)	No reflex cytology (HPV-negative)	Not performed	HPV-negative (routine recall)	
P12411	2024	2024-Q1	2024-02	HPV-primary era	HR-HPV DNA	55-59	55	Negative	N/A (no reflex cytology)	No reflex cytology (HPV-negative)	Not performed	HPV-negative (routine recall)	
P12412	2024	2024-Q1	2024-02	HPV-primary era	HR-HPV DNA	35-39	39	Positive	Satisfactory	NILM	NILM	HPV-positive / cytology normal	
P12413	2024	2024-Q1	2024-02	HPV-primary era	HR-HPV DNA	45-49	48	Negative	N/A (no reflex cytology)	No reflex cytology (HPV-negative)	Not performed	HPV-negative (routine recall)	
P12414	2024	2024-Q1	2024-02	HPV-primary era	HR-HPV DNA	30-34	30	Negative	N/A (no reflex cytology)	No reflex cytology (HPV-negative)	Not performed	HPV-negative (routine recall)	
P12415	2024	2024-Q1	2024-02	HPV-primary era	HR-HPV DNA	35-39	38	Positive	Satisfactory	NILM	NILM	HPV-positive / cytology normal	
P12416	2024	2024-Q1	2024-02	HPV-primary era	HR-HPV DNA	35-39	38	Negative	N/A (no reflex cytology)	No reflex cytology (HPV-negative)	Not performed	HPV-negative (routine recall)	
P12417	2024	2024-Q1	2024-02	HPV-primary era	HR-HPV DNA	40-44	41	Negative	N/A (no reflex cytology)	No reflex cytology (HPV-negative)	Not performed	HPV-negative (routine recall)	
P12418	2024	2024-Q1	2024-02	HPV-primary era	HR-HPV DNA	45-49	48	Positive	Satisfactory	Positive	ASC-US	Low-grade / equivocal squamous	
P12419	2024	2024-Q1	2024-02	HPV-primary era	HR-HPV DNA	35-39	38	Negative	N/A (no reflex cytology)	No reflex cytology (HPV-negative)	Not performed	HPV-negative (routine recall)	
P12420	2024	2024-Q1	2024-02	HPV-primary era	HR-HPV DNA	35-39	39	Positive	Satisfactory	Positive	ASC-US	Low-grade / equivocal squamous	
P12421	2024	2024-Q1	2024-02	HPV-primary era	HR-HPV DNA	25-29	25	Positive	Satisfactory	NILM	NILM	HPV-positive / cytology normal	
P12422	2024	2024-Q1	2024-02	HPV-primary era	HR-HPV DNA	40-44	44	Positive	Satisfactory	Positive	HSIL	High-grade squamous	
P12423	2024	2024-Q1	2024-02	HPV-primary era	HR-HPV DNA	40-44	41	Negative	N/A (no reflex cytology)	No reflex cytology (HPV-negative)	Not performed	HPV-negative (routine recall)	
P12424	2024	2024-Q1	2024-02	HPV-primary era	HR-HPV DNA	40-44	40	Negative	N/A (no reflex cytology)	No reflex cytology (HPV-negative)	Not performed	HPV-negative (routine recall)	
P12425	2024	2024-Q1	2024-02	HPV-primary era	HR-HPV DNA	45-49	45	Negative	N/A (no reflex cytology)	No reflex cytology (HPV-negative)	Not performed	HPV-negative (routine recall)	
P12426	2024	2024-Q1	2024-02	HPV-primary era	HR-HPV DNA	55-59	56	Negative	N/A (no reflex cytology)	No reflex cytology (HPV-negative)	Not performed	HPV-negative (routine recall)	
P12427	2024	2024-Q1	2024-02	HPV-primary era	HR-HPV DNA	40-44	41	Negative	N/A (no reflex cytology)	No reflex cytology (HPV-negative)	Not performed	HPV-negative (routine recall)	
P12428	2024	2024-Q1	2024-02	HPV-primary era	HR-HPV DNA	45-49	46	Negative	N/A (no reflex cytology)	No reflex cytology (HPV-negative)	Not performed	HPV-negative (routine recall)	
P12429	2024	2024-Q1	2024-02	HPV-primary era	HR-HPV DNA	50-54	54	Positive	Satisfactory	Positive	LSIL	Low-grade / equivocal squamous	
P12430	2024	2024-Q1	2024-02	HPV-primary era	HR-HPV DNA	35-39	39	Negative	N/A (no reflex cytology)	No reflex cytology (HPV-negative)	Not performed	HPV-negative (routine recall)	
P12431	2024	2024-Q1	2024-02	HPV-primary era	HR-HPV DNA	40-44	41	Positive	Satisfactory	NILM	NILM	HPV-positive / cytology normal	
P12432	2024	2024-Q1	2024-02	HPV-primary era	HR-HPV DNA	35-39	39	Negative	N/A (no reflex cytology)	No reflex cytology (HPV-negative)	Not performed	HPV-negative (routine recall)	
P12433	2024	2024-Q1	2024-02	HPV-primary era	HR-HPV DNA	50-54	53	Negative	N/A (no reflex cytology)	No reflex cytology (HPV-negative)	Not performed	HPV-negative (routine recall)	
P12434	2024	2024-Q1	2024-02	HPV-primary era	HR-HPV DNA	30-34	33	Negative	N/A (no reflex cytology)	No reflex cytology (HPV-negative)	Not performed	HPV-negative (routine recall)	
P12435	2024	2024-Q1	2024-02	HPV-primary era	HR-HPV DNA	30-34	32	Negative	N/A (no reflex cytology)	No reflex cytology (HPV-negative)	Not performed	HPV-negative (routine recall)	
P12436	2024	2024-Q1	2024-02	HPV-primary era	HR-HPV DNA	35-39	35	Negative	N/A (no reflex cytology)	No reflex cytology (HPV-negative)	Not performed	HPV-negative (routine recall)	
P12437	2024	2024-Q1	2024-02	HPV-primary era	HR-HPV DNA	40-44	44	Negative	N/A (no reflex cytology)	No reflex cytology (HPV-negative)	Not performed	HPV-negative (routine recall)	
P12438	2024	2024-Q1	2024-02	HPV-primary era	HR-HPV DNA	40-44	41	Negative	N/A (no reflex cytology)	No reflex cytology (HPV-negative)	Not performed	HPV-negative (routine recall)	
P12439	2024	2024-Q1	2024-02	HPV-primary era	HR-HPV DNA	40-44	41	Negative	N/A (no reflex cytology)	No reflex cytology (HPV-negative)	Not performed	HPV-negative (routine recall)	
P12440	2024	2024-Q1	2024-02	HPV-primary era	HR-HPV DNA	50-54	53	Negative	N/A (no reflex cytology)	No reflex cytology (HPV-negative)	Not performed	HPV-negative (routine recall)	
P12441	2024	2024-Q1	2024-02	HPV-primary era	HR-HPV DNA	30-34	34	Negative	N/A (no reflex cytology)	No reflex cytology (HPV-negative)	Not performed	HPV-negative (routine recall)	
P12442	2024	2024-Q1	2024-02	HPV-primary era	HR-HPV DNA	55-59	58	Negative	N/A (no reflex cytology)	No reflex cytology (HPV-negative)	Not performed	HPV-negative (routine recall)	
P12443	2024	2024-Q1	2024-02	HPV-primary era	HR-HPV DNA	60-64	60	Negative	N/A (no reflex cytology)	No reflex cytology (HPV-negative)	Not performed	HPV-negative (routine recall)	
P12444	2024	2024-Q1	2024-02	HPV-primary era	HR-HPV DNA	60-64	63	Negative	N/A (no reflex cytology)	No reflex cytology (HPV-negative)	Not performed	HPV-negative (routine recall)	
P12445	2024	2024-Q1	2024-02	HPV-primary era	HR-HPV DNA	55-59	58	Positive	Satisfactory	NILM	NILM	HPV-positive / cytology normal	
P12446	2024	2024-Q1	2024-02	HPV-primary era	HR-HPV DNA	45-49	45	Negative	N/A (no reflex cytology)	No reflex cytology (HPV-negative)	Not performed	HPV-negative (routine recall)	
P12447	2024	2024-Q1	2024-02	HPV-primary era	HR-HPV DNA	35-39	37	Negative	N/A (no reflex cytology)	No reflex cytology (HPV-negative)	Not performed	HPV-negative (routine recall)	
P12448	2024	2024-Q1	2024-02	HPV-primary era	HR-HPV DNA	55-59	55	Negative	N/A (no reflex cytology)	No reflex cytology (HPV-negative)	Not performed	HPV-negative (routine recall)	
P12449	2024	2024-Q1	2024-02	HPV-primary era	HR-HPV DNA	35-39	37	Negative	N/A (no reflex cytology)	No reflex cytology (HPV-negative)	Not performed	HPV-negative (routine recall)	
P12450	2024	2024-Q1	2024-02	HPV-primary era	HR-HPV DNA	50-54	54	Negative	N/A (no reflex cytology)	No reflex cytology (HPV-negative)	Not performed	HPV-negative (routine recall)	
P12451	2024	2024-Q1	2024-02	HPV-primary era	HR-HPV DNA	30-34	34	Positive	Satisfactory	NILM	NILM	HPV-positive / cytology normal	
P12452	2024	2024-Q1	2024-02	HPV-primary era	HR-HPV DNA	40-44	41	Positive	Satisfactory	NILM	NILM	HPV-positive / cytology normal	
P12453	2024	2024-Q1	2024-02	HPV-primary era	HR-HPV DNA	35-39	39	Negative	N/A (no reflex cytology)	No reflex cytology (HPV-negative)	Not performed	HPV-negative (routine recall)	
P12454	2024	2024-Q1	2024-02	HPV-primary era	HR-HPV DNA	30-34	32	Positive	Satisfactory	NILM	NILM	HPV-positive / cytology normal	
P12455	2024	2024-Q1	2024-02	HPV-primary era	HR-HPV DNA	55-59	56	Negative	N/A (no reflex cytology)	No reflex cytology (HPV-negative)	Not performed	HPV-negative (routine recall)	
P12456	2024	2024-Q1	2024-02	HPV-primary era	HR-HPV DNA	60-64	63	Negative	N/A (no reflex cytology)	No reflex cytology (HPV-negative)	Not performed	HPV-negative (routine recall)	
P12457	2024	2024-Q1	2024-02	HPV-primary era	HR-HPV DNA	25-29	26	Negative	N/A (no reflex cytology)	No reflex cytology (HPV-negative)	Not performed	HPV-negative (routine recall)	
P12458	2024	2024-Q1	2024-02	HPV-primary era	HR-HPV DNA	40-44	43	Negative	N/A (no reflex cytology)	No reflex cytology (HPV-negative)	Not performed	HPV-negative (routine recall)	
P12459	2024	2024-Q1	2024-02	HPV-primary era	HR-HPV DNA	40-44	41	Negative	N/A (no reflex cytology)	No reflex cytology (HPV-negative)	Not performed	HPV-negative (routine recall)	
P12460	2024	2024-Q1	2024-02	HPV-primary era	HR-HPV DNA	30-34	31	Negative	N/A (no reflex cytology)	No reflex cytology (HPV-negative)	Not performed	HPV-negative (routine recall)	
P12461	2024	2024-Q1	2024-02	HPV-primary era	HR-HPV DNA	35-39	37	Negative	N/A (no reflex cytology)	No reflex cytology (HPV-negative)	Not performed	HPV-negative (routine recall)	
P12462	2024	2024-Q1	2024-02	HPV-primary era	HR-HPV DNA	55-59	58	Negative	N/A (no reflex cytology)	No reflex cytology (HPV-negative)	Not performed	HPV-negative (routine recall)	
P12463	2024	2024-Q1	2024-02	HPV-primary era	HR-HPV DNA	50-54	53	Negative	N/A (no reflex cytology)	No reflex cytology (HPV-negative)	Not performed	HPV-negative (routine recall)	
P12464	2024	2024-Q1	2024-02	HPV-primary era	HR-HPV DNA	40-44	43	Negative	N/A (no reflex cytology)	No reflex cytology (HPV-negative)	Not performed	HPV-negative (routine recall)	
P12465	2024	2024-Q1	2024-02	HPV-primary era	HR-HPV DNA	60-64	61	Positive	Satisfactory	Positive	ASC-US	Low-grade / equivocal squamous	
P12466	2024	2024-Q1	2024-02	HPV-primary era	HR-HPV DNA	25-29	27	Negative	N/A (no reflex cytology)	No reflex cytology (HPV-negative)	Not performed	HPV-negative (routine recall)	
P12467	2024	2024-Q1	2024-02	HPV-primary era	HR-HPV DNA	35-39	35	Negative	N/A (no reflex cytology)	No reflex cytology (HPV-negative)	Not performed	HPV-negative (routine recall)	
P12468	2024	2024-Q1	2024-02	HPV-primary era	HR-HPV DNA	45-49	45	Negative	N/A (no reflex cytology)	No reflex cytology (HPV-negative)	Not performed	HPV-negative (routine recall)	
P12469	2024	2024-Q1	2024-02	HPV-primary era	HR-HPV DNA	35-39	38	Negative	N/A (no reflex cytology)	No reflex cytology (HPV-negative)	Not performed	HPV-negative (routine recall)	
P12470	2024	2024-Q1	2024-02	HPV-primary era	HR-HPV DNA	45-49	48	Negative	N/A (no reflex cytology)	No reflex cytology (HPV-negative)	Not performed	HPV-negative (routine recall)	
P12471	2024	2024-Q1	2024-02	HPV-primary era	HR-HPV DNA	30-34	34	Negative	N/A (no reflex cytology)	No reflex cytology (HPV-negative)	Not performed	HPV-negative (routine recall)	
P12472	2024	2024-Q1	2024-02	HPV-primary era	HR-HPV DNA	35-39	35	Positive	Satisfactory	NILM	NILM	HPV-positive / cytology normal	
P12473	2024	2024-Q1	2024-02	HPV-primary era	HR-HPV DNA	35-39	36	Negative	N/A (no reflex cytology)	No reflex cytology (HPV-negative)	Not performed	HPV-negative (routine recall)	
P12474	2024	2024-Q1	2024-02	HPV-primary era	HR-HPV DNA	40-44	41	Negative	N/A (no reflex cytology)	No reflex cytology (HPV-negative)	Not performed	HPV-negative (routine recall)	
P12475	2024	2024-Q1	2024-02	HPV-primary era	HR-HPV DNA	35-39	37	Negative	N/A (no reflex cytology)	No reflex cytology (HPV-negative)	Not performed	HPV-negative (routine recall)	
P12476	2024	2024-Q1	2024-02	HPV-primary era	HR-HPV DNA	40-44	41	Positive	Satisfactory	NILM	NILM	HPV-positive / cytology normal	
P12477	2024	2024-Q1	2024-02	HPV-primary era	HR-HPV DNA	40-44	42	Positive	Satisfactory	NILM	NILM	HPV-positive / cytology normal	
P12478	2024	2024-Q1	2024-02	HPV-primary era	HR-HPV DNA	25-29	27	Positive	Satisfactory	Positive	ASC-US	Low-grade / equivocal squamous	

patient_id	year	quarter	month	period	primary_test	age_group	age_years	hpv_result	adequacy	result	bethesda	grade	infection
P12479	2024	2024-Q1	2024-02	HPV-primary era	HR-HPV DNA	25-29	25	Negative	N/A (no reflex cytology)	No reflex cytology (HPV-negative)	Not performed	HPV-negative (routine recall)	
P12480	2024	2024-Q1	2024-02	HPV-primary era	HR-HPV DNA	35-39	39	Negative	N/A (no reflex cytology)	No reflex cytology (HPV-negative)	Not performed	HPV-negative (routine recall)	
P12481	2024	2024-Q1	2024-02	HPV-primary era	HR-HPV DNA	30-34	30	Negative	N/A (no reflex cytology)	No reflex cytology (HPV-negative)	Not performed	HPV-negative (routine recall)	
P12482	2024	2024-Q1	2024-03	HPV-primary era	HR-HPV DNA	60-64	62	Negative	N/A (no reflex cytology)	No reflex cytology (HPV-negative)	Not performed	HPV-negative (routine recall)	
P12483	2024	2024-Q1	2024-03	HPV-primary era	HR-HPV DNA	40-44	40	Negative	N/A (no reflex cytology)	No reflex cytology (HPV-negative)	Not performed	HPV-negative (routine recall)	Trichomonas
P12484	2024	2024-Q1	2024-03	HPV-primary era	HR-HPV DNA	35-39	37	Negative	N/A (no reflex cytology)	No reflex cytology (HPV-negative)	Not performed	HPV-negative (routine recall)	
P12485	2024	2024-Q1	2024-03	HPV-primary era	HR-HPV DNA	55-59	55	Positive	Satisfactory	Positive	ASC-H	High-grade squamous	
P12486	2024	2024-Q1	2024-03	HPV-primary era	HR-HPV DNA	30-34	33	Negative	N/A (no reflex cytology)	No reflex cytology (HPV-negative)	Not performed	HPV-negative (routine recall)	
P12487	2024	2024-Q1	2024-03	HPV-primary era	HR-HPV DNA	50-54	54	Negative	N/A (no reflex cytology)	No reflex cytology (HPV-negative)	Not performed	HPV-negative (routine recall)	
P12488	2024	2024-Q1	2024-03	HPV-primary era	HR-HPV DNA	30-34	33	Negative	N/A (no reflex cytology)	No reflex cytology (HPV-negative)	Not performed	HPV-negative (routine recall)	
P12489	2024	2024-Q1	2024-03	HPV-primary era	HR-HPV DNA	50-54	51	Negative	N/A (no reflex cytology)	No reflex cytology (HPV-negative)	Not performed	HPV-negative (routine recall)	
P12490	2024	2024-Q1	2024-03	HPV-primary era	HR-HPV DNA	35-39	38	Negative	N/A (no reflex cytology)	No reflex cytology (HPV-negative)	Not performed	HPV-negative (routine recall)	
P12491	2024	2024-Q1	2024-03	HPV-primary era	HR-HPV DNA	60-64	61	Negative	N/A (no reflex cytology)	No reflex cytology (HPV-negative)	Not performed	HPV-negative (routine recall)	
P12492	2024	2024-Q1	2024-03	HPV-primary era	HR-HPV DNA	35-39	38	Negative	N/A (no reflex cytology)	No reflex cytology (HPV-negative)	Not performed	HPV-negative (routine recall)	
P12493	2024	2024-Q1	2024-03	HPV-primary era	HR-HPV DNA	55-59	59	Positive	Satisfactory	NILM	NILM	HPV-positive / cytology normal	
P12494	2024	2024-Q1	2024-03	HPV-primary era	HR-HPV DNA	55-59	56	Negative	N/A (no reflex cytology)	No reflex cytology (HPV-negative)	Not performed	HPV-negative (routine recall)	
P12495	2024	2024-Q1	2024-03	HPV-primary era	HR-HPV DNA	55-59	57	Negative	N/A (no reflex cytology)	No reflex cytology (HPV-negative)	Not performed	HPV-negative (routine recall)	
P12496	2024	2024-Q1	2024-03	HPV-primary era	HR-HPV DNA	40-44	41	Positive	Satisfactory	NILM	NILM	HPV-positive / cytology normal	
P12497	2024	2024-Q1	2024-03	HPV-primary era	HR-HPV DNA	35-39	38	Negative	N/A (no reflex cytology)	No reflex cytology (HPV-negative)	Not performed	HPV-negative (routine recall)	
P12498	2024	2024-Q1	2024-03	HPV-primary era	HR-HPV DNA	40-44	41	Negative	N/A (no reflex cytology)	No reflex cytology (HPV-negative)	Not performed	HPV-negative (routine recall)	
P12499	2024	2024-Q1	2024-03	HPV-primary era	HR-HPV DNA	35-39	35	Negative	N/A (no reflex cytology)	No reflex cytology (HPV-negative)	Not performed	HPV-negative (routine recall)	
P12500	2024	2024-Q1	2024-03	HPV-primary era	HR-HPV DNA	25-29	26	Negative	N/A (no reflex cytology)	No reflex cytology (HPV-negative)	Not performed	HPV-negative (routine recall)	
P12501	2024	2024-Q1	2024-03	HPV-primary era	HR-HPV DNA	55-59	58	Negative	N/A (no reflex cytology)	No reflex cytology (HPV-negative)	Not performed	HPV-negative (routine recall)	
P12502	2024	2024-Q1	2024-03	HPV-primary era	HR-HPV DNA	35-39	39	Negative	N/A (no reflex cytology)	No reflex cytology (HPV-negative)	Not performed	HPV-negative (routine recall)	
P12503	2024	2024-Q1	2024-03	HPV-primary era	HR-HPV DNA	40-44	43	Positive	Satisfactory	NILM	NILM	HPV-positive / cytology normal	
P12504	2024	2024-Q1	2024-03	HPV-primary era	HR-HPV DNA	45-49	49	Positive	Satisfactory	Positive	ASC-US	Low-grade / equivocal squamous	
P12505	2024	2024-Q1	2024-03	HPV-primary era	HR-HPV DNA	45-49	49	Negative	N/A (no reflex cytology)	No reflex cytology (HPV-negative)	Not performed	HPV-negative (routine recall)	
P12506	2024	2024-Q1	2024-03	HPV-primary era	HR-HPV DNA	30-34	33	Negative	N/A (no reflex cytology)	No reflex cytology (HPV-negative)	Not performed	HPV-negative (routine recall)	
P12507	2024	2024-Q1	2024-03	HPV-primary era	HR-HPV DNA	30-34	31	Negative	N/A (no reflex cytology)	No reflex cytology (HPV-negative)	Not performed	HPV-negative (routine recall)	
P12508	2024	2024-Q1	2024-03	HPV-primary era	HR-HPV DNA	55-59	55	Negative	N/A (no reflex cytology)	No reflex cytology (HPV-negative)	Not performed	HPV-negative (routine recall)	
P12509	2024	2024-Q1	2024-03	HPV-primary era	HR-HPV DNA	45-49	46	Negative	N/A (no reflex cytology)	No reflex cytology (HPV-negative)	Not performed	HPV-negative (routine recall)	
P12510	2024	2024-Q1	2024-03	HPV-primary era	HR-HPV DNA	45-49	46	Negative	N/A (no reflex cytology)	No reflex cytology (HPV-negative)	Not performed	HPV-negative (routine recall)	
P12511	2024	2024-Q1	2024-03	HPV-primary era	HR-HPV DNA	40-44	44	Positive	Satisfactory	NILM	NILM	HPV-positive / cytology normal	
P12512	2024	2024-Q1	2024-03	HPV-primary era	HR-HPV DNA	30-34	31	Negative	N/A (no reflex cytology)	No reflex cytology (HPV-negative)	Not performed	HPV-negative (routine recall)	
P12513	2024	2024-Q1	2024-03	HPV-primary era	HR-HPV DNA	35-39	38	Negative	N/A (no reflex cytology)	No reflex cytology (HPV-negative)	Not performed	HPV-negative (routine recall)	
P12514	2024	2024-Q1	2024-03	HPV-primary era	HR-HPV DNA	30-34	31	Negative	N/A (no reflex cytology)	No reflex cytology (HPV-negative)	Not performed	HPV-negative (routine recall)	
P12515	2024	2024-Q1	2024-03	HPV-primary era	HR-HPV DNA	40-44	41	Negative	N/A (no reflex cytology)	No reflex cytology (HPV-negative)	Not performed	HPV-negative (routine recall)	
P12516	2024	2024-Q1	2024-03	HPV-primary era	HR-HPV DNA	40-44	43	Positive	Satisfactory	Positive	HSIL	High-grade squamous	
P12517	2024	2024-Q1	2024-03	HPV-primary era	HR-HPV DNA	35-39	39	Negative	N/A (no reflex cytology)	No reflex cytology (HPV-negative)	Not performed	HPV-negative (routine recall)	
P12518	2024	2024-Q1	2024-03	HPV-primary era	HR-HPV DNA	60-64	63	Negative	N/A (no reflex cytology)	No reflex cytology (HPV-negative)	Not performed	HPV-negative (routine recall)	
P12519	2024	2024-Q1	2024-03	HPV-primary era	HR-HPV DNA	30-34	32	Negative	N/A (no reflex cytology)	No reflex cytology (HPV-negative)	Not performed	HPV-negative (routine recall)	
P12520	2024	2024-Q1	2024-03	HPV-primary era	HR-HPV DNA	50-54	52	Negative	N/A (no reflex cytology)	No reflex cytology (HPV-negative)	Not performed	HPV-negative (routine recall)	
P12521	2024	2024-Q1	2024-03	HPV-primary era	HR-HPV DNA	25-29	29	Negative	N/A (no reflex cytology)	No reflex cytology (HPV-negative)	Not performed	HPV-negative (routine recall)	
P12522	2024	2024-Q1	2024-03	HPV-primary era	HR-HPV DNA	45-49	49	Negative	N/A (no reflex cytology)	No reflex cytology (HPV-negative)	Not performed	HPV-negative (routine recall)	
P12523	2024	2024-Q1	2024-03	HPV-primary era	HR-HPV DNA	25-29	25	Negative	N/A (no reflex cytology)	No reflex cytology (HPV-negative)	Not performed	HPV-negative (routine recall)	
P12524	2024	2024-Q1	2024-03	HPV-primary era	HR-HPV DNA	50-54	53	Positive	Satisfactory	NILM	NILM	HPV-positive / cytology normal	
P12525	2024	2024-Q1	2024-03	HPV-primary era	HR-HPV DNA	30-34	34	Negative	N/A (no reflex cytology)	No reflex cytology (HPV-negative)	Not performed	HPV-negative (routine recall)	
P12526	2024	2024-Q1	2024-03	HPV-primary era	HR-HPV DNA	60-64	60	Negative	N/A (no reflex cytology)	No reflex cytology (HPV-negative)	Not performed	HPV-negative (routine recall)	
P12527	2024	2024-Q1	2024-03	HPV-primary era	HR-HPV DNA	25-29	29	Negative	N/A (no reflex cytology)	No reflex cytology (HPV-negative)	Not performed	HPV-negative (routine recall)	
P12528	2024	2024-Q1	2024-03	HPV-primary era	HR-HPV DNA	30-34	34	Positive	Satisfactory	NILM	NILM	HPV-positive / cytology normal	
P12529	2024	2024-Q1	2024-03	HPV-primary era	HR-HPV DNA	25-29	28	Negative	N/A (no reflex cytology)	No reflex cytology (HPV-negative)	Not performed	HPV-negative (routine recall)	
P12530	2024	2024-Q1	2024-03	HPV-primary era	HR-HPV DNA	40-44	43	Negative	N/A (no reflex cytology)	No reflex cytology (HPV-negative)	Not performed	HPV-negative (routine recall)	
P12531	2024	2024-Q1	2024-03	HPV-primary era	HR-HPV DNA	25-29	27	Negative	N/A (no reflex cytology)	No reflex cytology (HPV-negative)	Not performed	HPV-negative (routine recall)	
P12532	2024	2024-Q1	2024-03	HPV-primary era	HR-HPV DNA	40-44	44	Negative	N/A (no reflex cytology)	No reflex cytology (HPV-negative)	Not performed	HPV-negative (routine recall)	
P12533	2024	2024-Q1	2024-03	HPV-primary era	HR-HPV DNA	60-64	64	Negative	N/A (no reflex cytology)	No reflex cytology (HPV-negative)	Not performed	HPV-negative (routine recall)	
P12534	2024	2024-Q1	2024-03	HPV-primary era	HR-HPV DNA	50-54	54	Negative	N/A (no reflex cytology)	No reflex cytology (HPV-negative)	Not performed	HPV-negative (routine recall)	
P12535	2024	2024-Q1	2024-03	HPV-primary era	HR-HPV DNA	40-44	43	Negative	N/A (no reflex cytology)	No reflex cytology (HPV-negative)	Not performed	HPV-negative (routine recall)	
P12536	2024	2024-Q1	2024-03	HPV-primary era	HR-HPV DNA	45-49	47	Negative	N/A (no reflex cytology)	No reflex cytology (HPV-negative)	Not performed	HPV-negative (routine recall)	
P12537	2024	2024-Q1	2024-03	HPV-primary era	HR-HPV DNA	60-64	62	Negative	N/A (no reflex cytology)	No reflex cytology (HPV-negative)	Not performed	HPV-negative (routine recall)	
P12538	2024	2024-Q1	2024-03	HPV-primary era	HR-HPV DNA	55-59	57	Negative	N/A (no reflex cytology)	No reflex cytology (HPV-negative)	Not performed	HPV-negative (routine recall)	
P12539	2024	2024-Q1	2024-03	HPV-primary era	HR-HPV DNA	45-49	46	Negative	N/A (no reflex cytology)	No reflex cytology (HPV-negative)	Not performed	HPV-negative (routine recall)	
P12540	2024	2024-Q1	2024-03	HPV-primary era	HR-HPV DNA	50-54	51	Negative	N/A (no reflex cytology)	No reflex cytology (HPV-negative)	Not performed	HPV-negative (routine recall)	
P12541	2024	2024-Q1	2024-03	HPV-primary era	HR-HPV DNA	50-54	51	Negative	N/A (no reflex cytology)	No reflex cytology (HPV-negative)	Not performed	HPV-negative (routine recall)	
P12542	2024	2024-Q1	2024-03	HPV-primary era	HR-HPV DNA	55-59	55	Positive	Satisfactory	Positive	ASC-US	Low-grade / equivocal squamous	
P12543	2024	2024-Q1	2024-03	HPV-primary era	HR-HPV DNA	40-44	40	Negative	N/A (no reflex cytology)	No reflex cytology (HPV-negative)	Not performed	HPV-negative (routine recall)	
P12544	2024	2024-Q1	2024-03	HPV-primary era	HR-HPV DNA	35-39	35	Negative	N/A (no reflex cytology)	No reflex cytology (HPV-negative)	Not performed	HPV-negative (routine recall)	
P12545	2024	2024-Q1	2024-03	HPV-primary era	HR-HPV DNA	40-44	44	Negative	N/A (no reflex cytology)	No reflex cytology (HPV-negative)	Not performed	HPV-negative (routine recall)	
P12546	2024	2024-Q1	2024-03	HPV-primary era	HR-HPV DNA	25-29	27	Negative	N/A (no reflex cytology)	No reflex cytology (HPV-negative)	Not performed	HPV-negative (routine recall)	
P12547	2024	2024-Q1	2024-03	HPV-primary era	HR-HPV DNA	45-49	46	Negative	N/A (no reflex cytology)	No reflex cytology (HPV-negative)	Not performed	HPV-negative (routine recall)	
P12548	2024	2024-Q1	2024-03	HPV-primary era	HR-HPV DNA	35-39	38	Negative	N/A (no reflex cytology)	No reflex cytology (HPV-negative)	Not performed	HPV-negative (routine recall)	
P12549	2024	2024-Q1	2024-03	HPV-primary era	HR-HPV DNA	35-39	38	Positive	Satisfactory	Positive	ASC-US	Low-grade / equivocal squamous	
P12550	2024	2024-Q1	2024-03	HPV-primary era	HR-HPV DNA	30-34	32	Negative	N/A (no reflex cytology)	No reflex cytology (HPV-negative)	Not performed	HPV-negative (routine recall)	
P12551	2024	2024-Q1	2024-03	HPV-primary era	HR-HPV DNA	35-39	39	Positive	Satisfactory	Positive	ASC-H	High-grade squamous	
P12552	2024	2024-Q1	2024-03	HPV-primary era	HR-HPV DNA	40-44	44	Negative	N/A (no reflex cytology)	No reflex cytology (HPV-negative)	Not performed	HPV-negative (routine recall)	
P12553	2024	2024-Q1	2024-03	HPV-primary era	HR-HPV DNA	40-44	41	Negative	N/A (no reflex cytology)	No reflex cytology (HPV-negative)	Not performed	HPV-negative (routine recall)	
P12554	2024	2024-Q1	2024-03	HPV-primary era	HR-HPV DNA	45-49	48	Negative	N/A (no reflex cytology)	No reflex cytology (HPV-negative)	Not performed	HPV-negative (routine recall)	
P12555	2024	2024-Q1	2024-03	HPV-primary era	HR-HPV DNA	45-49	48	Positive	Satisfactory	Positive	ASC-US	Low-grade / equivocal squamous	
P12556	2024	2024-Q1	2024-03	HPV-primary era	HR-HPV DNA	30-34	33	Negative	N/A (no reflex cytology)	No reflex cytology (HPV-negative)	Not performed	HPV-negative (routine recall)	
P12557	2024	2024-Q1	2024-03	HPV-primary era	HR-HPV DNA	30-34	34	Positive	Satisfactory	Positive	LSIL	Low-grade / equivocal squamous	
P12558	2024	2024-Q1	2024-03	HPV-primary era	HR-HPV DNA	35-39	35	Positive	Satisfactory	NILM	NILM	HPV-positive / cytology normal	

patient_id	year	quarter	month	period	primary_test	age_group	age_years	hpv_result	adequacy	result	bethesda	grade	infection
P12559	2024	2024-Q1	2024-03	HPV-primary era	HR-HPV DNA	45-49	47	Negative	N/A (no reflex cytology)	No reflex cytology (HPV-negative)	Not performed	HPV-negative (routine recall)	
P12560	2024	2024-Q1	2024-03	HPV-primary era	HR-HPV DNA	45-49	48	Negative	N/A (no reflex cytology)	No reflex cytology (HPV-negative)	Not performed	HPV-negative (routine recall)	
P12561	2024	2024-Q1	2024-03	HPV-primary era	HR-HPV DNA	30-34	33	Positive	Satisfactory	Positive	LSIL	Low-grade / equivocal squamous	
P12562	2024	2024-Q1	2024-03	HPV-primary era	HR-HPV DNA	30-34	30	Negative	N/A (no reflex cytology)	No reflex cytology (HPV-negative)	Not performed	HPV-negative (routine recall)	Trichomonas
P12563	2024	2024-Q1	2024-03	HPV-primary era	HR-HPV DNA	40-44	43	Negative	N/A (no reflex cytology)	No reflex cytology (HPV-negative)	Not performed	HPV-negative (routine recall)	
P12564	2024	2024-Q1	2024-03	HPV-primary era	HR-HPV DNA	45-49	49	Positive	Satisfactory	NILM	NILM	HPV-positive / cytology normal	
P12565	2024	2024-Q1	2024-03	HPV-primary era	HR-HPV DNA	40-44	44	Negative	N/A (no reflex cytology)	No reflex cytology (HPV-negative)	Not performed	HPV-negative (routine recall)	
P12566	2024	2024-Q1	2024-03	HPV-primary era	HR-HPV DNA	25-29	26	Negative	N/A (no reflex cytology)	No reflex cytology (HPV-negative)	Not performed	HPV-negative (routine recall)	
P12567	2024	2024-Q1	2024-03	HPV-primary era	HR-HPV DNA	40-44	42	Negative	N/A (no reflex cytology)	No reflex cytology (HPV-negative)	Not performed	HPV-negative (routine recall)	
P12568	2024	2024-Q1	2024-03	HPV-primary era	HR-HPV DNA	35-39	38	Negative	N/A (no reflex cytology)	No reflex cytology (HPV-negative)	Not performed	HPV-negative (routine recall)	
P12569	2024	2024-Q1	2024-03	HPV-primary era	HR-HPV DNA	60-64	63	Negative	N/A (no reflex cytology)	No reflex cytology (HPV-negative)	Not performed	HPV-negative (routine recall)	
P12570	2024	2024-Q1	2024-03	HPV-primary era	HR-HPV DNA	35-39	39	Negative	N/A (no reflex cytology)	No reflex cytology (HPV-negative)	Not performed	HPV-negative (routine recall)	
P12571	2024	2024-Q1	2024-03	HPV-primary era	HR-HPV DNA	40-44	40	Negative	N/A (no reflex cytology)	No reflex cytology (HPV-negative)	Not performed	HPV-negative (routine recall)	
P12572	2024	2024-Q1	2024-03	HPV-primary era	HR-HPV DNA	55-59	57	Negative	N/A (no reflex cytology)	No reflex cytology (HPV-negative)	Not performed	HPV-negative (routine recall)	
P12573	2024	2024-Q1	2024-03	HPV-primary era	HR-HPV DNA	50-54	50	Positive	Satisfactory	Positive	ASC-US	Low-grade / equivocal squamous	
P12574	2024	2024-Q1	2024-03	HPV-primary era	HR-HPV DNA	55-59	58	Negative	N/A (no reflex cytology)	No reflex cytology (HPV-negative)	Not performed	HPV-negative (routine recall)	
P12575	2024	2024-Q1	2024-03	HPV-primary era	HR-HPV DNA	60-64	62	Negative	N/A (no reflex cytology)	No reflex cytology (HPV-negative)	Not performed	HPV-negative (routine recall)	
P12576	2024	2024-Q1	2024-03	HPV-primary era	HR-HPV DNA	55-59	58	Negative	N/A (no reflex cytology)	No reflex cytology (HPV-negative)	Not performed	HPV-negative (routine recall)	
P12577	2024	2024-Q1	2024-03	HPV-primary era	HR-HPV DNA	25-29	26	Negative	N/A (no reflex cytology)	No reflex cytology (HPV-negative)	Not performed	HPV-negative (routine recall)	
P12578	2024	2024-Q1	2024-03	HPV-primary era	HR-HPV DNA	25-29	29	Positive	Satisfactory	Positive	HSIL	High-grade squamous	
P12579	2024	2024-Q1	2024-03	HPV-primary era	HR-HPV DNA	25-29	29	Positive	Satisfactory	Positive	ASC-US	Low-grade / equivocal squamous	
P12580	2024	2024-Q1	2024-03	HPV-primary era	HR-HPV DNA	45-49	48	Negative	N/A (no reflex cytology)	No reflex cytology (HPV-negative)	Not performed	HPV-negative (routine recall)	
P12581	2024	2024-Q1	2024-03	HPV-primary era	HR-HPV DNA	30-34	31	Negative	N/A (no reflex cytology)	No reflex cytology (HPV-negative)	Not performed	HPV-negative (routine recall)	
P12582	2024	2024-Q1	2024-03	HPV-primary era	HR-HPV DNA	45-49	46	Negative	N/A (no reflex cytology)	No reflex cytology (HPV-negative)	Not performed	HPV-negative (routine recall)	
P12583	2024	2024-Q1	2024-03	HPV-primary era	HR-HPV DNA	40-44	44	Negative	N/A (no reflex cytology)	No reflex cytology (HPV-negative)	Not performed	HPV-negative (routine recall)	
P12584	2024	2024-Q1	2024-03	HPV-primary era	HR-HPV DNA	60-64	64	Negative	N/A (no reflex cytology)	No reflex cytology (HPV-negative)	Not performed	HPV-negative (routine recall)	
P12585	2024	2024-Q1	2024-03	HPV-primary era	HR-HPV DNA	40-44	40	Positive	Satisfactory	Positive	ASC-US	Low-grade / equivocal squamous	
P12586	2024	2024-Q1	2024-03	HPV-primary era	HR-HPV DNA	25-29	28	Negative	N/A (no reflex cytology)	No reflex cytology (HPV-negative)	Not performed	HPV-negative (routine recall)	
P12587	2024	2024-Q1	2024-03	HPV-primary era	HR-HPV DNA	35-39	39	Negative	N/A (no reflex cytology)	No reflex cytology (HPV-negative)	Not performed	HPV-negative (routine recall)	
P12588	2024	2024-Q1	2024-03	HPV-primary era	HR-HPV DNA	40-44	44	Negative	N/A (no reflex cytology)	No reflex cytology (HPV-negative)	Not performed	HPV-negative (routine recall)	
P12589	2024	2024-Q1	2024-03	HPV-primary era	HR-HPV DNA	50-54	51	Negative	N/A (no reflex cytology)	No reflex cytology (HPV-negative)	Not performed	HPV-negative (routine recall)	
P12590	2024	2024-Q1	2024-03	HPV-primary era	HR-HPV DNA	55-59	58	Negative	N/A (no reflex cytology)	No reflex cytology (HPV-negative)	Not performed	HPV-negative (routine recall)	
P12591	2024	2024-Q1	2024-03	HPV-primary era	HR-HPV DNA	40-44	42	Negative	N/A (no reflex cytology)	No reflex cytology (HPV-negative)	Not performed	HPV-negative (routine recall)	
P12592	2024	2024-Q1	2024-03	HPV-primary era	HR-HPV DNA	30-34	30	Negative	N/A (no reflex cytology)	No reflex cytology (HPV-negative)	Not performed	HPV-negative (routine recall)	
P12593	2024	2024-Q1	2024-03	HPV-primary era	HR-HPV DNA	35-39	37	Negative	N/A (no reflex cytology)	No reflex cytology (HPV-negative)	Not performed	HPV-negative (routine recall)	
P12594	2024	2024-Q1	2024-03	HPV-primary era	HR-HPV DNA	40-44	41	Positive	Satisfactory	NILM	NILM	HPV-positive / cytology normal	
P12595	2024	2024-Q1	2024-03	HPV-primary era	HR-HPV DNA	50-54	53	Positive	Satisfactory	Positive	HSIL	High-grade squamous	
P12596	2024	2024-Q1	2024-03	HPV-primary era	HR-HPV DNA	40-44	43	Negative	N/A (no reflex cytology)	No reflex cytology (HPV-negative)	Not performed	HPV-negative (routine recall)	
P12597	2024	2024-Q1	2024-03	HPV-primary era	HR-HPV DNA	30-34	34	Negative	N/A (no reflex cytology)	No reflex cytology (HPV-negative)	Not performed	HPV-negative (routine recall)	
P12598	2024	2024-Q1	2024-03	HPV-primary era	HR-HPV DNA	40-44	42	Positive	Satisfactory	NILM	NILM	HPV-positive / cytology normal	
P12599	2024	2024-Q1	2024-03	HPV-primary era	HR-HPV DNA	30-34	30	Negative	N/A (no reflex cytology)	No reflex cytology (HPV-negative)	Not performed	HPV-negative (routine recall)	
P12600	2024	2024-Q1	2024-03	HPV-primary era	HR-HPV DNA	50-54	51	Positive	Satisfactory	Positive	HSIL	High-grade squamous	
P12601	2024	2024-Q1	2024-03	HPV-primary era	HR-HPV DNA	40-44	43	Negative	N/A (no reflex cytology)	No reflex cytology (HPV-negative)	Not performed	HPV-negative (routine recall)	
P12602	2024	2024-Q1	2024-03	HPV-primary era	HR-HPV DNA	30-34	34	Negative	N/A (no reflex cytology)	No reflex cytology (HPV-negative)	Not performed	HPV-negative (routine recall)	
P12603	2024	2024-Q1	2024-03	HPV-primary era	HR-HPV DNA	40-44	43	Negative	N/A (no reflex cytology)	No reflex cytology (HPV-negative)	Not performed	HPV-negative (routine recall)	
P12604	2024	2024-Q1	2024-03	HPV-primary era	HR-HPV DNA	50-54	52	Negative	N/A (no reflex cytology)	No reflex cytology (HPV-negative)	Not performed	HPV-negative (routine recall)	
P12605	2024	2024-Q1	2024-03	HPV-primary era	HR-HPV DNA	55-59	55	Negative	N/A (no reflex cytology)	No reflex cytology (HPV-negative)	Not performed	HPV-negative (routine recall)	
P12606	2024	2024-Q1	2024-03	HPV-primary era	HR-HPV DNA	35-39	36	Negative	N/A (no reflex cytology)	No reflex cytology (HPV-negative)	Not performed	HPV-negative (routine recall)	
P12607	2024	2024-Q1	2024-03	HPV-primary era	HR-HPV DNA	45-49	49	Negative	N/A (no reflex cytology)	No reflex cytology (HPV-negative)	Not performed	HPV-negative (routine recall)	
P12608	2024	2024-Q1	2024-03	HPV-primary era	HR-HPV DNA	25-29	27	Negative	N/A (no reflex cytology)	No reflex cytology (HPV-negative)	Not performed	HPV-negative (routine recall)	
P12609	2024	2024-Q1	2024-03	HPV-primary era	HR-HPV DNA	35-39	35	Negative	N/A (no reflex cytology)	No reflex cytology (HPV-negative)	Not performed	HPV-negative (routine recall)	
P12610	2024	2024-Q1	2024-03	HPV-primary era	HR-HPV DNA	25-29	29	Negative	N/A (no reflex cytology)	No reflex cytology (HPV-negative)	Not performed	HPV-negative (routine recall)	
P12611	2024	2024-Q1	2024-03	HPV-primary era	HR-HPV DNA	40-44	42	Negative	N/A (no reflex cytology)	No reflex cytology (HPV-negative)	Not performed	HPV-negative (routine recall)	
P12612	2024	2024-Q1	2024-03	HPV-primary era	HR-HPV DNA	60-64	60	Negative	N/A (no reflex cytology)	No reflex cytology (HPV-negative)	Not performed	HPV-negative (routine recall)	
P12613	2024	2024-Q1	2024-03	HPV-primary era	HR-HPV DNA	55-59	57	Negative	N/A (no reflex cytology)	No reflex cytology (HPV-negative)	Not performed	HPV-negative (routine recall)	
P12614	2024	2024-Q1	2024-03	HPV-primary era	HR-HPV DNA	35-39	38	Negative	N/A (no reflex cytology)	No reflex cytology (HPV-negative)	Not performed	HPV-negative (routine recall)	
P12615	2024	2024-Q1	2024-03	HPV-primary era	HR-HPV DNA	45-49	48	Positive	Satisfactory	Positive	HSIL	High-grade squamous	
P12616	2024	2024-Q1	2024-03	HPV-primary era	HR-HPV DNA	35-39	37	Negative	N/A (no reflex cytology)	No reflex cytology (HPV-negative)	Not performed	HPV-negative (routine recall)	
P12617	2024	2024-Q1	2024-03	HPV-primary era	HR-HPV DNA	55-59	57	Negative	N/A (no reflex cytology)	No reflex cytology (HPV-negative)	Not performed	HPV-negative (routine recall)	
P12618	2024	2024-Q1	2024-03	HPV-primary era	HR-HPV DNA	30-34	33	Negative	N/A (no reflex cytology)	No reflex cytology (HPV-negative)	Not performed	HPV-negative (routine recall)	
P12619	2024	2024-Q1	2024-03	HPV-primary era	HR-HPV DNA	55-59	57	Negative	N/A (no reflex cytology)	No reflex cytology (HPV-negative)	Not performed	HPV-negative (routine recall)	
P12620	2024	2024-Q1	2024-03	HPV-primary era	HR-HPV DNA	25-29	28	Negative	N/A (no reflex cytology)	No reflex cytology (HPV-negative)	Not performed	HPV-negative (routine recall)	
[truncated: 1,025,802 more chars]
